# Detection and phylogenetic analysis of highly pathogenic A/H5N1 avian influenza clade 2.3.4.4b virus in Chile, 2022

**DOI:** 10.1080/22221751.2023.2220569

**Published:** 2023-06-20

**Authors:** Pedro Jimenez-Bluhm, Jurre Y. Siegers, Shaoyuan Tan, Bridgett Sharp, Pamela Freiden, Magdalena Johow, Katherinne Orozco, Soledad Ruiz, Cecilia Baumberger, Pablo Galdames, Maria Antonieta Gonzalez, Camila Rojas, Erik A. Karlsson, Christopher Hamilton-West, Stacey Schultz-Cherry

**Affiliations:** a Escuela de Medicina Veterinaria, Facultad de Ciencias Biológicas, Facultad de Medicina y Facultad de Agronomía e Ingeniería Forestal, Pontificia Universidad Católica de Chile, Santiago, Chile; b Virology Unit, WHO H5 Regional Reference Laboratory, Institut Pasteur du Cambodge, Phnom Penh, Cambodia; c Department of Infectious Diseases, St. Jude Children's Research Hospital, Memphis, TN, USA; d Servicio Agrícola y Ganadero (SAG), Santiago, Chile; e Unidad de Epidemiologia, Departamento de Medicina Preventiva Animal, Facultad de Ciencias Veterinarias y Pecuarias, Universidad de Chile, Santiago, Chile

**Keywords:** Avian influenza, Chile, South America, HPAI, wild birds, phylogenetic analysis

## Abstract

Highly pathogenic avian influenza (HPAI) A/H5N1 viruses continue to pose a significant threat to animal and human health worldwide. In late 2022, the first confirmed case of HPAI A/H5N1 infection in wild birds in Chile near the Chilean-Peruvian border was reported. Active surveillance by our group in the adyacent Lluta river estuary revealed an increase in A/H5N1 prevalence coinciding with the arrival of migratory birds from the Northern Hemisphere. Genomic analysis of A/H5N1-positive samples demonstrated a close genetic relationship to strains detected in Peru during the same period, which originated from A/H5N1 viruses causing outbreaks in North America. Notably, we identified genetic mutations that did not correlate with known enhanced transmission or binding traits to mammalian receptors. In summary, this study provides valuable genomic insights into the A/H5N1 Clade 2.3.4.4b viruses in wild birds in Chile, emphasizing the need for enhanced surveillance and response strategies to mitigate the threat posed by these highly pathogenic avian influenza viruses in South America.

## Main text

Highly pathogenic avian influenza (HPAI) viruses remain a major threat to both animal and human health. Since the emergence of the HPAI A/H5N1 goose/Guangdong (Gs/GD) lineage in 1996, these viruses have spread globally. This includes the recent introduction and reassortment of lineage 2.3.4.4b into the Americas in 2021, impacting both wild and domestic bird populations with high mortality [1]. Since October 2022, reports of mortality in wild birds in numerous South American countries along the Pacific Migratory Flyway have been attributed to HPAI A/H5Nx viruses [2]. These same countries have also reported infections in domestic bird populations to the World Organization for Animal Health (WOAH). However, genomic data for the viruses entering South American remains sparse. Here we report an initial genomic characterization of A/H5N1 Clade 2.3.4.4b viruses detected in wild birds in Chile.

On 7th December 2022, the Agricultural and Livestock Service of Chile (SAG) reported the first confirmed case of HPAI A/H5N1 infection in Peruvian pelicans (*Pelecanus thagus)* at the Lluta River wetland located 10 km south of the Chilean-Peruvian border. We have been conducting active, longitudinal avian influenza (AIV) surveillance in Chile since 2015, with a focus on wild birds and high-risk interfaces with domestic poultry and humans. In anticipation of 2.3.4.4 encroachment into South America, we intensified our ongoing AIV surveillance in the Lluta river estuary (S 18° 24’ 59.306”; W 70° 19’ 20.668) to biweekly sampling in August 2022. AIV prevalence (as measured by M gene specific qRT-PCR) remained below 1% in August and September, 2022. However, prevalence jumped to 2.6% and 11.7% in November and December, respectively, coinciding with the arrival of migratory birds from the Northern Hemisphere. Of the 69 AIV positive environmental fecal samples from November and December (total samples: 2023), 7 (10.1%) were identified as hemagglutinin (HA) subtype A/H5: 1 obtained in late November, and 6 in December. As per regulations, original samples and RNA were submitted to SAG (submission #230014). Cytochrome Oxidase I speciation [3] shows the A/H5 positive samples come from several species: Peruvian pelican (*Pelecanus thagus*) (n = 1), Franklin’s gull (*Larus pipixcan*) (n = 1), Grey gull (*Leucophaeus modestus*) (n = 1), Elegant tern (*Thalasseus elegans*) (n = 2) and Black skimmer (*Rynchops niger*) (n = 2).

**Figure 1.**
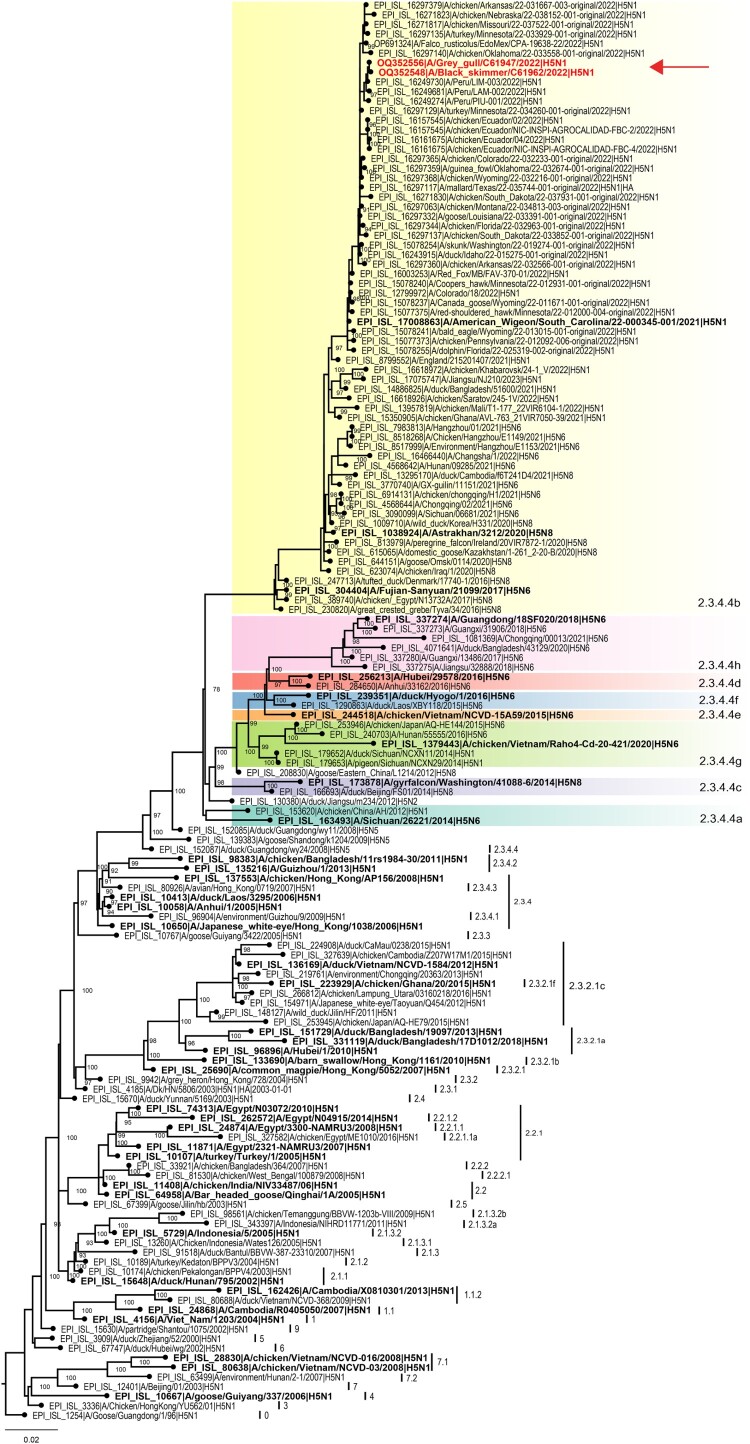
**Maximum likelihood phylogeny of the HA genes of H5N1 viruses detected in Chile, 2022.** A/H5Nx phylogeny of all H5 clades and 2.3.4.4b clade viruses containing Chilean A/H5N1 viruses and closest relatives. Chilean viruses are highlighted in bold red indicated by a red arrow. H5 clade 2.3.4.4a-g are indicated by coloured shading. Candidate vaccine viruses and their associated H5 clade numbering is shown in bold.

Full genome sequencing of 3 of the samples yielded 2 complete genomes (A/Grey gull/Chile/61947/2022 and A/Black skimmer/Chile/61962/2022) and one partial genome (A/Peruvian pelican/Chile/61740/2022) missing segments 2 and 4 due to low viral load, GenBank accession numbers OQ352540 to OQ352552. The A/H5 HA genes were genetically closest related to Peruvian pelican and chicken samples detected in Peru around the same period, and both Peruvian and Chilean strains derive from A/H5N1 viruses causing widespread outbreaks in poultry and wild birds across North America [2] (Figure 1). Chilean A/H5N1 HA sequences have 99.9%/99.5% identity in nucleotide/amino acid similarity to each other, respectively, and 98.1%/98.9% identity in nucleotide/amino acid similarity relative to the A/H5N1 clade 2.3.4.4b candidate vaccine strain, A/Astrakhan/3212/2020 (Supplemental Table 1). HA mutations 101M, 112Q, 200A and 486 V (H3 numbering) were shared across both Chilean A/H5N1 HA segments (Supplemental Table 1). These mutations do not correlate with any previously reported phenotypic traits associated with increased risk, like enhanced contact transmission in ferrets or increased binding to mammalian receptors, among others [4]. Consistent with 2.3.4.4b HA genes, Chilean A/H5N1 viruses contain the HPAI polybasic cleavage site motif, REKRRKR|GLF. Likewise, N1 neuraminidase (NA) gene segments were closest to Peruvian pelican strains (Supplemental Figure 1). The NA sequences do not contain any stalk deletions or markers of antiviral drug resistance. Further analysis of the internal gene cassette of the Chilean H5 viruses using FluSurver (https://flusurver.bii.a-star.edu.sg/) revealed the presence of several mutation compared to their respective reference strains in the HA, PA and NS gene that could potentially affect host specificity, antigenic escape and/or virulence (Supplemental Table 3.). Analysis of the internal genes using BLASTn revealed all genes are most similar (>99% nucleotide identity) to A/H5N1 viruses circulating in North and South America (Supplemental Table 4.).

Emergence of A/H5 2.3.4.4b in South America poses major risk, as exemplified by the catastrophic impacts to the domestic poultry and wildlife sectors in Europe and North America since 2021 [5]. There is limited experience in South America on management and containment of AIV, possibly due to minimal HPAI in the region (Chile, 2002) [6]. For the moment, regional A/H5 outbreaks/detections are limited to shorebirds, backyard poultry, and a few commercial farms. However, prevalence is likely grossly underestimated, constrained by the sheer size of the affected territory, economic factors, a dearth of exhaustive AIV surveillance, diagnostics, and adequately trained veterinary personnel to identify the disease. There is also a lack of incentive to report due to insufficient economic compensation mechanisms for culling of exposed poultry throughout the region. Considering the large, socio-economically vulnerable population in South America, mass culling of exposed poultry could also greatly reduce access to critical animal protein sources and promote food insecurity, further complicating eradication plans. Aside from domestic poultry, A/H5 2.3.4.4b in South America will also likely affect entire populations of already vulnerable wild bird species. Over 22,000 wild birds were lost in Peru to A/H5 infection between late November and early December 2022, many already considered endangered [2].

Enhanced surveillance in both domestic and wild birds is critical in Chile and across South America. HPAI A/H5Nx viruses in South America have only been detected in shorebirds and Charadriiformes hosts so far, likely due to passive detection systems. Active, longitudinal surveillance, especially in known AIV hotspots [7,8] should also focus on Anseriformes, especially given year long AIV circulation and genetic diversity in these species in Chile [8]. Further sequencing of A/H5Nx and non-A/H5 positive samples is also critical to identify possible reassortment events with locally circulating AIV strains.

## Supplementary Material

Supplemental Material
