# Supplementary material for: Detection and phylogenetic analysis of highly pathogenic A/H5N1 avian influenza clade 2.3.4.4b virus in Chile, 2022
Source: Emerg Microbes Infect. 2023 Jun 20;12(2):2220569. doi: 10.1080/22221751.2023.2220569 (PMC10283444; doi:10.1080/22221751.2023.2220569)
Supplement: Supplemental Material [file TEMI_A_2220569_SM7933.zip › Chilean_H5_Material_and_Method_FINAL.docx]

**Material and Method**

**Sample Collection and RNA extraction**

Oropharyngeal and cloacal samples were collected from domestic birds (ducks and chickens) as described previously (PMID: 31815962). Briefly, viral RNA was extracted using the QIAamp Viral RNA Mini Kit (Qiagen, Maryland, USA) according to the manufacturer’s protocol. All samples were screened by real-time reverse transcription PCR with M gene as previously described (PMID: 34586866), and samples with a cycle threshold (Ct) of less than 38 were deemed positive. All positive original samples and isolates with Ct<32 were selected for sequencing regardless of their subtype.

**Sequencing**

Whole genomes were amplified using custom Uni12/Inf-1 and Uni13/Inf-1 barcoded primers (PMID: 19605485) kindly provided by Peter Thielen with SSIII One-step RT-PCR with Platinum Taq High Fidelity kit (Thermo Fisher, Massachusetts, USA). Sequencing libraries were prepared using ligation sequencing kit SQK-LSK109 (Oxford Nanopore Technologies, Oxford, UK). Samples were sequenced using the GridION platform (Oxford Nanopore Technologies, Oxford, UK). Sequencing reads were then de-multiplexed, quality trimmed, and filtered using Porechop software (https://github.com/rrwick/Porechop). Gene segment sequences obtained from influenza A viruses in this study were deposited to the GenBank under accession numbers OQ352540 to OQ352552. To generate the consensus sequence, IRMA was run with default settings. Consensus sequences were manually inspected for errors such as INDELs, and mixed bases and corrected if required. A minimum 100bp cutoff for depth coverage for any gene segments was used.

**Phylogenetic Analysis**

All H5Nx sequences were obtained from the GenBank and GISAID databases (assessed on 2023-01-22. Additionally, for the HA and NA genes, we added the top 100 BLAST matches that were closest to the Chilean H5N1 sequences and a select number of non-2.3.4.4b viruses and candidate virus vaccine (CVV) strains. Duplicates (based on strain name), laboratory derived, mixed subtype, and low coverage (<90% of full length) sequences were excluded from downstream analysis when possible. Sequences were aligned with MAFFT v.7.490 (PMID: 23329690), trimmed using trimAL (PMID: 19505945) and phylogenetic trees were constructed using FastTree using the generalized time-reversible (GTR) models of nucleotide evolution (PMID: 20224823) and IQ-TREE v.2.0.3 (PMID: 32011700). with the best-fit nucleotide substitution model (TPM2u+F+I chosen according to Bayesian Information Criterion (BIC)). Trees were visualized and annotated using FigTree v.1.4.4 (<http://tree.bio.ed.ac.uk/software/figtree/>). Clade nomenclature was adopted from the WHO antigenic and genetic characteristics of zoonotic influenza A viruses and development of candidate vaccine viruses for pandemic preparedness (<https://apps.who.int/iris/handle/10665/363866>).
