# Supplementary material for: Detection and phylogenetic analysis of highly pathogenic A/H5N1 avian influenza clade 2.3.4.4b virus in Chile, 2022
Source: Emerg Microbes Infect. 2023 Jun 20;12(2):2220569. doi: 10.1080/22221751.2023.2220569 (PMC10283444; doi:10.1080/22221751.2023.2220569)
Supplement: Supplemental Material [file TEMI_A_2220569_SM7933.zip › Supplemental Data 1. NCBI and GISAD acknowledgement.pdf]

Supplemental Data 1. NCBI and GISAD acknowledgement

NCBI:

accession	length	host	segment	serotype	country	region	date	name	mutations	age	gender	lineage	vac_strain	fulllength_plus
AB932556	1742	Avian	4 (HA)	H5N8	Japan	N	2014/04/13	Influenza A virus (A/chicken/kumamoto/1-7/2014(H5N8))						c
AB979455	1742	Avian	4 (HA)	H5N1	Viet Nam	T	2014/04/15	Influenza A virus (A/muscovy duck/Vietnam/LBM631/2014(H5N1))						c
AB979463	1742	Avian	4 (HA)	H5N1	Viet Nam	T	2014/04/15	Influenza A virus (A/duck/Vietnam/LBM632/2014(H5N1))						c
AB979471	1742	Avian	4 (HA)	H5N1	Viet Nam	T	2014/04/15	Influenza A virus (A/duck/Vietnam/LBM633/2014(H5N1))						c
AB979479	1742	Avian	4 (HA)	H5N1	Viet Nam	T	2014/04/15	Influenza A virus (A/muscovy duck/Vietnam/LBM634/2014(H5N1))						c
AB979487	1734	Avian	4 (HA)	H5N1	Viet Nam	T	2014/04/15	Influenza A virus (A/muscovy duck/Vietnam/LBM635/2014(H5N1))						c
AB979495	1741	Avian	4 (HA)	H5N1	Viet Nam	T	2014/04/15	Influenza A virus (A/muscovy duck/Vietnam/LBM636/2014(H5N1))						c
AB979503	1740	Avian	4 (HA)	H5N1	Viet Nam	T	2014/04/15	Influenza A virus (A/duck/Vietnam/LBM638/2014(H5N1))						c
AB979511	1742	Avian	4 (HA)	H5N1	Viet Nam	T	2014/04/15	Influenza A virus (A/duck/Vietnam/LBM639/2014(H5N1))						c
KJ508916	1742	Avian	4 (HA)	H5N8	South Korea	N	2014/01/19	Influenza A virus (A/broiler duck/Korea/H32/2014(H5N8))						c
KP090439	1776	Avian	4 (HA)	H5N6	China	N	2014/06/29	Influenza A virus (A/chicken/Jiangxi/NCDZT1123/2014(H5N6))						c
KP090447	1776	Avian	4 (HA)	H5N6	China	N	2014/06/29	Influenza A virus (A/chicken/Jiangxi/NCDZT1126/2014(H5N6))						c
KP714479	1776	Avian	4 (HA)	H5N8	Taiwan	N	2015/01/09	Influenza A virus (A/goose/Taiwan/01003/2015(H5N8))						c
KP714480	1776	Avian	4 (HA)	H5N2	Taiwan	N	2015/01/09	Influenza A virus (A/goose/Taiwan/01004/2015(H5N2))						c
KP714481	1776	Avian	4 (HA)	H5N3	Taiwan	N	2015/01/12	Influenza A virus (A/goose/Taiwan/01042/2015(H5N3))						c
KP732644	1704	Avian	4 (HA)	H5N6	China	N	2014/03/22	Influenza A virus (A/duck/Eastern China/S0322/2014(H5N6))						c
KP851843	1743	Avian	4 (HA)	H5N8	South Korea	N	2014/02/15	Influenza A virus (A/baikal teal/Korea/K14-E016/2014(H5N8))						c
KT280932	1744	Avian	4 (HA)	H5N2	USA	N	2015/04/27	Influenza A virus (A/chicken/Iowa/21981/2015(H5N2))						c
KT327395	1776	Avian	4 (HA)	H5N2	Taiwan	N	2015/01/19	Influenza A virus (A/night heron/Taiwan/A289/2015(H5N2))						c
KT327403	1776	Avian	4 (HA)	H5N2	Taiwan	N	2015/01/17	Influenza A virus (A/thrush/Taiwan/A234/2015(H5N2))						c
KT327411	1776	Avian	4 (HA)	H5N3	Taiwan	N	2015/01/15	Influenza A virus (A/Bulbul/Taiwan/01156/2015(H5N3))						c
KT370058	1704	Environment	4 (HA)	H5N6	China	N	2014/05/06	Influenza A virus (A/environment/Guangdong/QY208/2014(H5N6))						c
KT370059	1704	Environment	4 (HA)	H5N6	China	N	2013/04/10	Influenza A virus (A/environment/Guangdong/QY025/2013(H5N6))						c
KT370060	1704	Environment	4 (HA)	H5N6	China	N	2014/05/06	Influenza A virus (A/environment/Guangdong/QY197/2014(H5N6))						c
KT370061	1704	Environment	4 (HA)	H5N6	China	N	2014/07/17	Influenza A virus (A/environment/Guangdong/ZS356/2014(H5N6))						c
KT370062	1704	Environment	4 (HA)	H5N6	China	N	2014/12/20	Influenza A virus (A/environment/Guangdong/PY955/2014(H5N6))						c
KT370063	1704	Environment	4 (HA)	H5N6	China	N	2015/02/23	Influenza A virus (A/environment/Guangdong/HY243/2015(H5N6))						c
KT370064	1704	Environment	4 (HA)	H5N6	China	N	2014/03/02	Influenza A virus (A/environment/Guangdong/JY137/2014(H5N6))						c
KT936689	1619	Avian	4 (HA)	H5N8	China	N	2014/12/15	Influenza A virus (A/Eurasian curlew/Shanghai/DT1215-1/2014(H5N8))						p
KT936690	1587	Avian	4 (HA)	H5N8	China	N	2014/12/31	Influenza A virus (A/Eurasian curlew/Shanghai/DT1231-1/2014(H5N8))						p
KT936691	1585	Avian	4 (HA)	H5N8	China	N	2014/12/31	Influenza A virus (A/Eurasian curlew/Shanghai/DT1231-2/2014(H5N8))						p
KT936692	1598	Avian	4 (HA)	H5N8	China	N	2014/12/31	Influenza A virus (A/Eurasian curlew/Shanghai/DT1231-3/2014(H5N8))						p
KT936693	1586	Avian	4 (HA)	H5N8	China	N	2014/12/31	Influenza A virus (A/Eurasian curlew/Shanghai/DT1231-4/2014(H5N8))						p
KT936694	1598	Avian	4 (HA)	H5N8	China	N	2014/12/31	Influenza A virus (A/Eurasian curlew/Shanghai/DT1231-5/2014(H5N8))						p
KT936695	1590	Avian	4 (HA)	H5N8	China	N	2014/12/31	Influenza A virus (A/Eurasian curlew/Shanghai/DT1231-6/2014(H5N8))						p
KX121198	1704	Avian	4 (HA)	H5N6	China	N	2014/11/12	Influenza A virus (A/goose/Hunan/118/2014(H5N6))						c
KX121206	1704	Avian	4 (HA)	H5N6	China	N	2014/11/13	Influenza A virus (A/duck/Hunan/144/2014(H5N6))						c
KX297862	1704	Environment	4 (HA)	H5N8	South Korea	N	2014/02/06	Influenza A virus (A/environment/Korea/W454/2014(H5N8))						c
KX297863	1704	Avian	4 (HA)	H5N8	South Korea	N	2014/02/14	Influenza A virus (A/mallard duck/Korea/W456/2014(H5N8))						c
KX297864	1704	Avian	4 (HA)	H5N8	South Korea	N	2014/02/14	Influenza A virus (A/mallard duck/Korea/W457/2014(H5N8))						c
KX297865	1704	Environment	4 (HA)	H5N8	South Korea	N	2014/02/18	Influenza A virus (A/environment/Korea/W458/2014(H5N8))						c
KX297866	1704	Environment	4 (HA)	H5N8	South Korea	N	2014/12/21	Influenza A virus (A/environment/Korea/W464/2014(H5N8))						c
KX297867	1704	Environment	4 (HA)	H5N8	South Korea	N	2014/12/21	Influenza A virus (A/environment/Korea/W465/2014(H5N8))						c
KX297868	1704	Environment	4 (HA)	H5N8	South Korea	N	2014/12/21	Influenza A virus (A/environment/Korea/W466/2014(H5N8))						c
KX297869	1704	Environment	4 (HA)	H5N8	South Korea	N	2014/12/21	Influenza A virus (A/environment/Korea/W467/2014(H5N8))						c
KX297870	1704	Environment	4 (HA)	H5N8	South Korea	N	2014/12/21	Influenza A virus (A/environment/Korea/W468/2014(H5N8))						c
KX297871	1704	Environment	4 (HA)	H5N8	South Korea	N	2014/12/21	Influenza A virus (A/environment/Korea/W469/2014(H5N8))						c
KX297872	1704	Environment	4 (HA)	H5N8	South Korea	N	2014/12/21	Influenza A virus (A/environment/Korea/W470/2014(H5N8))						c
KX297873	1704	Environment	4 (HA)	H5N8	South Korea	N	2014/12/21	Influenza A virus (A/environment/Korea/W471/2014(H5N8))						c
KX297874	1704	Environment	4 (HA)	H5N8	South Korea	N	2014/12/21	Influenza A virus (A/environment/Korea/W472/2014(H5N8))						c
KX297875	1704	Environment	4 (HA)	H5N8	South Korea	N	2014/12/21	Influenza A virus (A/environment/Korea/W473/2014(H5N8))						c
KX297876	1704	Environment	4 (HA)	H5N8	South Korea	N	2014/12/21	Influenza A virus (A/environment/Korea/W474/2014(H5N8))						c
KX297877	1704	Environment	4 (HA)	H5N8	South Korea	N	2014/12/21	Influenza A virus (A/environment/Korea/W475/2014(H5N8))						c
KX297878	1704	Environment	4 (HA)	H5N8	South Korea	N	2014/12/21	Influenza A virus (A/environment/Korea/W476/2014(H5N8))						c
KX297879	1704	Environment	4 (HA)	H5N8	South Korea	N	2014/12/21	Influenza A virus (A/environment/Korea/W477/2014(H5N8))						c
KX297880	1704	Environment	4 (HA)	H5N8	South Korea	N	2015/01/14	Influenza A virus (A/environment/Korea/W482/2015(H5N8))						c
KX297881	1704	Environment	4 (HA)	H5N8	South Korea	N	2015/01/14	Influenza A virus (A/environment/Korea/W483/2015(H5N8))						c
KX297882	1704	Environment	4 (HA)	H5N8	South Korea	N	2015/01/21	Influenza A virus (A/environment/Korea/W486/2015(H5N8))						c
KX297883	1704	Environment	4 (HA)	H5N8	South Korea	N	2015/01/21	Influenza A virus (A/environment/Korea/W487/2015(H5N8))						c
KX297884	1704	Environment	4 (HA)	H5N8	South Korea	N	2015/01/21	Influenza A virus (A/environment/Korea/W488/2015(H5N8))						c
KX297885	1704	Environment	4 (HA)	H5N8	South Korea	N	2015/01/21	Influenza A virus (A/environment/Korea/W490/2015(H5N8))						c
KX297886	1704	Environment	4 (HA)	H5N8	South Korea	N	2015/02/27	Influenza A virus (A/environment/Korea/W492/2015(H5N8))						c
KX960164	1701	Avian	4 (HA)	H5N6	China	N	2015/01/07	Influenza A virus (A/wild bird/Jiangxi/P560/2015(H5N6))						c
KX960166	1704	Avian	4 (HA)	H5N1	China	N	2015/01/07	Influenza A virus (A/wild bird/Jiangxi/P410/2015(H5N1))						c
KX960168	1701	Avian	4 (HA)	H5N6	China	N	2015/01/07	Influenza A virus (A/wild bird/Jiangxi/P38/2015(H5N6))						c
KX960170	1701	Avian	4 (HA)	H5N6	China	N	2015/01/07	Influenza A virus (A/wild bird/Jiangxi/P31/2015(H5N6))						c
KX960172	1701	Avian	4 (HA)	H5N6	China	N	2015/01/07	Influenza A virus (A/wild bird/Jiangxi/P21/2015(H5N6))						c
KX960174	1701	Avian	4 (HA)	H5N6	China	N	2015/01/07	Influenza A virus (A/wild bird/Jiangxi/P6/2015(H5N6))						c
KX960176	1701	Avian	4 (HA)	H5N6	China	N	2015/01/07	Influenza A virus (A/wild bird/Jiangxi/P5/2015(H5N6))						c
KX960178	1701	Avian	4 (HA)	H5N6	China	N	2015/01/07	Influenza A virus (A/wild bird/Jiangxi/P562/2015(H5N6))						c
KX960180	1701	Avian	4 (HA)	H5N6	China	N	2015/01/07	Influenza A virus (A/wild bird/Jiangxi/P469/2015(H5N6))						c
KX960182	1701	Avian	4 (HA)	H5N6	China	N	2015/01/07	Influenza A virus (A/wild bird/Jiangxi/P237/2015(H5N6))						c
KX960184	1704	Avian	4 (HA)	H5N1	China	N	2015/01/07	Influenza A virus (A/wild bird/Jiangxi/P126/2015(H5N1))						c
KY316512	1701	Avian	4 (HA)	H5N6	South Korea	N	2016/11/25	Influenza A virus (A/waterfowl/Korea/S57/2016(H5N6))						c
LC041308	1734	Avian	4 (HA)	H5N6	Viet Nam	T	2014/11/18	Influenza A virus (A/duck/Vietnam/HU1-1152/2014(H5N6))						c
LC041313	1710	Avian	4 (HA)	H5N6	Viet Nam	T	2014/09/03	Influenza A virus (A/duck/Vietnam/HU1-1151/2014(H5N6))						p

LC041317	1734	Avian	4 (HA)	H5N6	Viet Nam	T	2014/11/18	Influenza A virus (A/duck/Vietnam/HU1-1507/2014(H5N6))	c
LC041319	1738	Avian	4 (HA)	H5N6	Viet Nam	T	2014/11/18	Influenza A virus (A/duck/Vietnam/HU1-1511/2014(H5N6))	c
LC041321	1736	Avian	4 (HA)	H5N6	Viet Nam	T	2014/11/18	Influenza A virus (A/duck/Vietnam/HU1-1434/2014(H5N6))	c
LC041323	1736	Avian	4 (HA)	H5N6	Viet Nam	T	2014/11/18	Influenza A virus (A/duck/Vietnam/HU1-1144/2014(H5N6))	c
LC275037	1739	Avian	4 (HA)	H5N6	Japan	N	2017/01/15	Influenza A virus (A/bean goose/Ishikawa/1701A012/2017(H5N6))	c
LC275045	1739	Avian	4 (HA)	H5N6	Japan	N	2017/01/29	Influenza A virus (A/bean goose/Ishikawa/1701A014/2017(H5N6))	c
LC279816	1742	Avian	4 (HA)	H5N6	Viet Nam	T	2015/12/08	Influenza A virus (A/duck/Vietnam/LBM837/2015(H5N6))	c
LC279824	1734	Avian	4 (HA)	H5N6	Viet Nam	T	2015/11/14	Influenza A virus (A/chicken/Nha Trang/122/2015(H5N6))	c
LC279832	1721	Avian	4 (HA)	H5N6	Viet Nam	T	2015/11/14	Influenza A virus (A/chicken/Nha Trang/124/2015(H5N6))	c
LC279840	1742	Avian	4 (HA)	H5N6	Viet Nam	T	2015/11/14	Influenza A virus (A/chicken/Nha Trang/127/2015(H5N6))	c
LC279848	1727	Avian	4 (HA)	H5N6	Viet Nam	T	2015/11/14	Influenza A virus (A/chicken/Nha Trang/128/2015(H5N6))	c
LC279856	1728	Avian	4 (HA)	H5N6	Viet Nam	T	2015/11/14	Influenza A virus (A/chicken/Nha Trang/129/2015(H5N6))	c
LC279864	1742	Avian	4 (HA)	H5N6	Viet Nam	T	2016/03/04	Influenza A virus (A/muscovy duck/Quang Ninh/254/2016(H5N6))	c
LC318856	1760	Avian	4 (HA)	H5N6	Japan	N	2017/01/16	Influenza A virus (A/whooper swan/Iwate/17/2017(H5N6))	c
LC348834	1739	Environment	4 (HA)	H5N6	Japan	N	2016/12/17	Influenza A virus (A/water/Aichi/C3/2016(H5N6))	c
LC348842	1739	Environment	4 (HA)	H5N6	Japan	N	2016/12/17	Influenza A virus (A/water/Aichi/W4/2016(H5N6))	c
LC420034	1742	Environment	4 (HA)	H5N8	Japan	N	2014/12/29	Influenza A virus (A/environment/Yamaguchi/8/2014)	c
LC420042	1742	Environment	4 (HA)	H5N8	Japan	N	2014/12/14	Influenza A virus (A/environment/Miyazaki/11/2014)	c
LC536298	1742	Avian	4 (HA)	H5N6	Viet Nam	T	2017/12/27	Influenza A virus (A/duck/Vietnam/LBM1042/2017)	c
LC536322	1742	Avian	4 (HA)	H5N6	Viet Nam	T	2017/10/27	Influenza A virus (A/chicken/Nha Trang/247/2017)	c
LC718210	1773	Avian	4 (HA)	H5N1	Japan	N	2022/01/27	Influenza A virus (A/common buzzard/Kyoto/2601B013/2022)	c
LC718218	1766	Avian	4 (HA)	H5N1	Japan	N	2022/02/08	Influenza A virus (A/whooper swan/Iwate/0302I001T/2022)	c
LC718226	1772	Avian	4 (HA)	H5N1	Japan	N	2022/02/12	Influenza A virus (A/white-fronted goose/Iwate/TU16-74/2022)	c
LC718234	1771	Avian	4 (HA)	H5N1	Japan	N	2022/03/04	Influenza A virus (A/jungle crow/Iwate/0303I003/2022)	c
LC718242	1768	Avian	4 (HA)	H5N1	Japan	N	2022/03/18	Influenza A virus (A/whooper swan/Iwate/0303B006/2022)	c
LC718250	1770	Avian	4 (HA)	H5N1	Japan	N	2022/04/06	Influenza A virus (A/jungle crow/Iwate/0304I001/2022)	c
LC718258	1767	Avian	4 (HA)	H5N1	Japan	N	2022/04/09	Influenza A virus (A/jungle crow/Hokkaido/0104B085/2022)	c
LC718266	1776	Avian	4 (HA)	H5N1	Japan	N	2022/04/19	Influenza A virus (A/jungle crow/Akita/0504F001/2022)	c
LC718274	1767	Avian	4 (HA)	H5N1	Japan	N	2022/04/09	Influenza A virus (A/jungle crow/Hokkaido/0104B087/2022)	c
LC718346	1771	Avian	4 (HA)	H5N1	Japan	N	2022/04/18	Influenza A virus (A/chicken/Akita/TU22-31/2022)	c
LC718362	1765	Avian	4 (HA)	H5N1	Japan	N	2022/05/14	Influenza A virus (A/chicken/Hokkaido/TU25-3/2022)	c
LC723835	1766	Avian	4 (HA)	H5N8	Japan	N	2020	Influenza A virus (A/Unidentified_ducks/Kagoshima/NIES229/2020)	p
MF399540	1704	Avian	4 (HA)	H5N6	China	N	2015/11/11	Influenza A virus (A/Ferruginous Pochard/Ningxia/473-8/2015(H5N6))	c
MF399556	1704	Avian	4 (HA)	H5N6	China	N	2015/11/11	Influenza A virus (A/Northern Shoveler/Ningxia/475-11/2015(H5N6))	c
MF399564	1704	Avian	4 (HA)	H5N6	China	N	2015/11/11	Influenza A virus A/Eurasian Wigeon/Ningxia/476-12/2015(H5N6)	c
MF399588	1704	Avian	4 (HA)	H5N6	China	N	2015/11/11	Influenza A virus (A/Ferruginous Pochard/Ningxia/479-16/2015(H5N6))	c
MF399596	1704	Avian	4 (HA)	H5N6	China	N	2015/11/11	Influenza A virus (A/Ferruginous Pochard/Ningxia/480-17/2015(H5N6))	c
MF399604	1704	Avian	4 (HA)	H5N6	China	N	2015/11/11	Influenza A virus (A/Northern Shoveler/Ningxia/481-21/2015(H5N6))	c
MF399620	1704	Avian	4 (HA)	H5N6	China	N	2015/11/11	Influenza A virus (A/Northern Shoveler/Ningxia/483-28/2015(H5N6))	c
MF399668	1704	Avian	4 (HA)	H5N6	China	N	2015/11/16	Influenza A virus (A/Northern Shoveler/Ningxia/488-53/2015(H5N6))	c
MF926453	1742	Avian	4 (HA)	H5N8	Russia	N	2017/02/28	Influenza A virus (A/chicken/Moscow/94/2017(H5N8))	c
MF926461	1742	Avian	4 (HA)	H5N8	Russia	N	2017/01/08	Influenza A virus (A/chicken/Chechnya/58/2017(H5N8))	c
MF926469	1742	Avian	4 (HA)	H5N8	Russia	N	2017/01/05	Influenza A virus (A/swan/Krasnodar/44/2017(H5N8))	c
MF926477	1742	Avian	4 (HA)	H5N8	Russia	N	2017/01/03	Influenza A virus (A/swan/Voronezh/2/2017(H5N8))	c
MF926485	1742	Avian	4 (HA)	H5N8	Russia	N	2016/11/07	Influenza A virus (A/goose/Kalmykia/813/2016(H5N8))	c
MG029170	1701	Avian	4 (HA)	H5N6	China	N	2016/09	Influenza A virus (A/poultry/China/XY165.4/2016(H5N6))	c
MG029172	1704	Avian	4 (HA)	H5N6	China	N	2016/09	Influenza A virus (A/poultry/China/XY01.4/2016(H5N6))	c
MG220416	1751	Environment	4 (HA)	H5N6	China	N	2017/03/17	Influenza A virus (A/environment/Hunankaifu/303/2017(H5N6))	c
MH022716	1766	Avian	4 (HA)	H5N6	China	N	2016/11/29	Influenza A virus (A/greenwing duck/Shanghai/SH1/2016)	c
MH156490	1748	Environment	4 (HA)	H5N6	China	N	2014/06/17	Influenza A virus (A/environment/Chang Sha/308/2014)	c
MH156491	1748	Environment	4 (HA)	H5N6	China	N	2014/09/18	Influenza A virus (A/environment/Chang Sha/399/2014)	c
MH156492	1748	Environment	4 (HA)	H5N6	China	N	2014/10/27	Influenza A virus (A/environment/Chang Sha/441/2014)	c
MH156493	1748	Environment	4 (HA)	H5N6	China	N	2014/10/27	Influenza A virus (A/environment/Chang Sha/443/2014)	c
MH156494	1748	Environment	4 (HA)	H5N6	China	N	2014/10/27	Influenza A virus (A/environment/Chang Sha/445/2014)	c
MH156495	1748	Environment	4 (HA)	H5N6	China	N	2014/10/28	Influenza A virus (A/environment/Chang Sha/448/2014)	c
MH156496	1748	Environment	4 (HA)	H5N6	China	N	2014/10/31	Influenza A virus (A/environment/Chang Sha/455/2014)	c
MH156497	1748	Environment	4 (HA)	H5N6	China	N	2014/10/31	Influenza A virus (A/environment/Chang Sha/458/2014)	c
MH156498	1748	Environment	4 (HA)	H5N6	China	N	2014/11/25	Influenza A virus (A/environment/Chang Sha/488/2014)	c
MH156499	1748	Environment	4 (HA)	H5N6	China	N	2014/11/26	Influenza A virus (A/environment/Chang Sha/499/2014)	c
MH283029	1704	Avian	4 (HA)	H5N6	China	N	2017/10/21	Influenza A virus (A/common mallard/Ningxia/YG71/2017)	c
MH283037	1704	Avian	4 (HA)	H5N6	China	N	2017/10/21	Influenza A virus (A/spot-billed duck/Ningxia/YG83/2017)	c
MH283045	1704	Avian	4 (HA)	H5N6	China	N	2017/10/21	Influenza A virus (A/spot-billed duck/Ningxia/YG87/2017)	c
MH283053	1704	Avian	4 (HA)	H5N6	China	N	2017/10/21	Influenza A virus (A/spot-billed duck/Ningxia/YG93/2017)	c
MH569637	1704	Avian	4 (HA)	H5N8	Uganda	T	2017/01	Influenza A virus (A/white_winged_black_tern/Uganda/17RS115-3/2017)	c
MH988777	1735	Environment	4 (HA)	H5N8	Ukraine	N	2017/01/27	Influenza A virus (A/Environmental fecal/AN/2/2017)	c
MK418658	1701	Mink	4 (HA)	H5N6	China	N	2018/01/30	Influenza A virus (A/mink/Northern China/F0130m/2018)	c
MK631789	1762	Avian	4 (HA)	H5N8	Democratic R	T	2017/05/13	Influenza A virus (A/Muscovy duck/Democratic Republic of the Congo/KAF1/2017)	c
MK636726	1761	Avian	4 (HA)	H5N8	Democratic R	T	2017/05/14	Influenza A virus (A/Muscovy duck/Democratic Republic of the Congo/KAF4_HA/201	c
MK636734	1762	Avian	4 (HA)	H5N8	Democratic R	T	2017/05/14	Influenza A virus (A/Muscovy duck/Democratic Republic of the Congo/NYA4_HA/20	c
MK636742	1762	Avian	4 (HA)	H5N8	Democratic R	T	2017/05/13	Influenza A virus (A/Muscovy duck/Democratic Republic of the Congo/NYA14_HA/2	c
MK636750	1762	Avian	4 (HA)	H5N8	Democratic R	T	2017/05/13	Influenza A virus (A/Muscovy duck/Democratic Republic of the Congo/NYA15_HA/2	c
MK636758	1762	Avian	4 (HA)	H5N8	Democratic R	T	2017/05/13	Influenza A virus (A/Muscovy duck/Democratic Republic of the Congo/TCH4_HA/201	c
MK636766	1762	Avian	4 (HA)	H5N8	Democratic R	T	2017/05/13	Influenza A virus (A/Muscovy duck/Democratic Republic of the Congo/TCH5_HA/201	c
MK636774	1760	Avian	4 (HA)	H5N8	Democratic R	T	2017/05/15	Influenza A virus (A/Muscovy duck/Democratic Republic of the Congo/TCH6_HA/201	c
MK734294	1704	Avian	4 (HA)	H5N8	South Africa	S	2018/01/29	Influenza A virus (A/Sandwich tern/South Africa/18010369/2018)	c
MK734295	1704	Penguin	4 (HA)	H5N8	South Africa	S	2018/03/01	Influenza A virus (A/African penguin/South Africa/476266/2018)	c
MK734296	1704	Avian	4 (HA)	H5N8	South Africa	S	2018/01/29	Influenza A virus (A/Common tern/South Africa/18010371/2018)	c
MK734297	1704	Avian	4 (HA)	H5N8	South Africa	S	2018/01/03	Influenza A virus (A/Swift tern/South Africa/18010027/2018)	c
MK734298	1704	Avian	4 (HA)	H5N8	South Africa	S	2018/01/03	Influenza A virus (A/Swift tern/South Africa/18010028/2018)	c
MK734299	1704	Avian	4 (HA)	H5N8	South Africa	S	2018/01/29	Influenza A virus (A/Swift tern/South Africa/18010370/2018)	c
MK734300	1704	Avian	4 (HA)	H5N8	South Africa	S	2018/01/31	Influenza A virus (A/tern/South Africa/18010417/2018)	c
MK779131	1772	Avian	4 (HA)	H5N6	China	N	2018/08/11	Influenza A virus (A/white-naped crane/China/ya3/2018)	c
MK942972	1749	Avian	4 (HA)	H5N6	Viet Nam	T	2015/10/25	Influenza A virus (A/muscovy duck/Viet Nam/HN-2508/2015)	c

MK943406	1749	Avian	4 (HA)	H5N6	Viet Nam	T	2015/10/25	Influenza A virus (A/muscovy duck/Viet Nam/HN-2501/2015)	c
MK943414	1749	Avian	4 (HA)	H5N6	Viet Nam	T	2015/10/25	Influenza A virus (A/muscovy duck/Viet Nam/HN-2504/2015)	c
MK943422	1749	Avian	4 (HA)	H5N6	Viet Nam	T	2015/10/25	Influenza A virus (A/muscovy duck/Viet Nam/HN-2506/2015)	c
MK943478	1749	Avian	4 (HA)	H5N6	Viet Nam	T	2016/01/05	Influenza A virus (A/muscovy duck/Viet Nam/QN-2611/2016)	c
MK943486	1749	Avian	4 (HA)	H5N6	Viet Nam	T	2016/01/05	Influenza A virus (A/muscovy duck/Viet Nam/QN-2612/2016)	c
MK943502	1749	Avian	4 (HA)	H5N6	Viet Nam	T	2016/01/05	Influenza A virus (A/muscovy duck/Viet Nam/QN-2616/2016)	c
MK943522	1749	Avian	4 (HA)	H5N6	Viet Nam	T	2016/04/16	Influenza A virus (A/muscovy duck/Viet Nam/QN-2706/2016)	c
MK963921	1749	Avian	4 (HA)	H5	Viet Nam	T	2014/08/04	Influenza A virus (A/muscovy duck/Viet Nam/HN-1697/2014)	c
MK964973	1749	Avian	4 (HA)	H5N6	Viet Nam	T	2014/08/04	Influenza A virus (A/muscovy duck/Viet Nam/HN-1699/2014)	c
MK975995	1532	Avian	4 (HA)	H5N8	Egypt	N	2018	Influenza A virus (A/chicken/Egypt/AB2/2018)	p
MN025431	1766	Avian	4 (HA)	H5N8	Denmark	N	2016	Influenza A virus (A/tufted duck/Denmark/11740/2016)	c
MN037415	1776	Avian	4 (HA)	H5N2	USA	N	2015/01/09	Influenza A virus (A/cooper's hawk/Washington/2551/2015)	c
MN124544	1624	Avian	4 (HA)	H5N8	South Africa	S	2018/01/04	Influenza A virus (A/Tern/South Africa/18010043/2018)	p
MN124552	1624	Avian	4 (HA)	H5N8	South Africa	S	2018/01/11	Influenza A virus (A/Jackal buzzard/South Africa/18010106/2018)	p
MN124560	1624	Avian	4 (HA)	H5N8	South Africa	S	2018/01/08	Influenza A virus (A/Tern/South Africa/18010107/2018)	p
MN124568	1624	Avian	4 (HA)	H5N8	South Africa	S	2018/01/18	Influenza A virus (A/Common tern/South Africa/18010259/2018)	p
MN124576	1624	Penguin	4 (HA)	H5N8	South Africa	S	2018/01/31	Influenza A virus (A/African penguin/South Africa/18010422/2018)	p
MN124584	1624	Penguin	4 (HA)	H5N8	South Africa	S	2018/01/31	Influenza A virus (A/African penguin/South Africa/18010423/2018)	p
MN124592	1624	Avian	4 (HA)	H5N8	South Africa	S	2018/02/15	Influenza A virus (A/Swift tern/South Africa/18020273/2018)	p
MN124600	1624	Avian	4 (HA)	H5N8	South Africa	S	2018/02/15	Influenza A virus (A/Sandwich tern/South Africa/18020302/2018)	p
MN124608	1624	Avian	4 (HA)	H5N8	South Africa	S	2018/02/15	Influenza A virus (A/Cape cormorant/South Africa/18020303/2018)	p
MN124616	1624	Penguin	4 (HA)	H5N8	South Africa	S	2018/02/15	Influenza A virus (A/African penguin/South Africa/18020304/2018)	p
MN124624	1624	Penguin	4 (HA)	H5N8	South Africa	S	2018/02/22	Influenza A virus (A/African penguin/South Africa/18020408/2018)	p
MN124632	1624	Avian	4 (HA)	H5N8	South Africa	S	2018/03/09	Influenza A virus (A/Crowned cormorant/South Africa/18030213/2018)	p
MN124640	1624	Avian	4 (HA)	H5N8	South Africa	S	2018/03/09	Influenza A virus (A/African oystercatcher/South Africa/18030214/2018)	p
MN124648	1624	Avian	4 (HA)	H5N8	South Africa	S	2018/03/27	Influenza A virus (A/Swift tern/South Africa/18030478/2018)	p
MN124656	1624	Avian	4 (HA)	H5N8	South Africa	S	2018/04/12	Influenza A virus (A/Hartlaubs gull/South Africa/18040224/2018)	p
MN124664	1624	Avian	4 (HA)	H5N8	South Africa	S	2018/04/17	Influenza A virus (A/Swift tern/South Africa/18040275/2018)	p
MN124672	1624	Avian	4 (HA)	H5N8	South Africa	S	2018/04/20	Influenza A virus (A/Hartlaubs gull/South Africa/18040367/2018)	p
MN124680	1624	Penguin	4 (HA)	H5N8	South Africa	S	2018/05/12	Influenza A virus (A/African penguin/South Africa/18050256/2018)	p
MN128638	1704	Avian	4 (HA)	H5N6	China	N	2015/12/16	Influenza A virus (A/goose/Guangdong/A-Goose-Guangdong-GS017-2015)	c
MN128651	1704	Avian	4 (HA)	H5N6	China	N	2015/12/16	Influenza A virus (A/goose/Guangdong/A-Goose-Guangdong-GS114-2015)	c
MN128659	1704	Avian	4 (HA)	H5N6	China	N	2015/12/16	Influenza A virus (A/goose/Guangdong/A-Goose-Guangdong-GS116-2015)	c
MN128667	1704	Avian	4 (HA)	H5N6	China	N	2015/12/16	Influenza A virus (A/goose/Guangdong/A-Goose-Guangdong-GS120-2015)	c
MN128675	1704	Avian	4 (HA)	H5N6	China	N	2015/12/16	Influenza A virus (A/goose/Guangdong/A-Goose-Guangdong-GS119-2015)	c
MN128680	1704	Avian	4 (HA)	H5N6	China	N	2015/12/16	Influenza A virus (A/goose/Guangdong/A-Goose-Guangdong-GS144-2015)	c
MN128689	1704	Avian	4 (HA)	H5N6	China	N	2016/01/08	Influenza A virus (A/goose/Guangdong/A-Goose-Guangdong-GS148-2016)	c
MN128870	1704	Avian	4 (HA)	H5N6	China	N	2015/12/16	Influenza A virus (A/goose/Guangdong/A-Goose-Guangdong-GS018-2015)	c
MN556609	1704	Avian	4 (HA)	H5N6	China	N	2013/11/26	Influenza A virus (A/duck/China/13087-4.seq/2013)	c
MN556681	1704	Avian	4 (HA)	H5N6	China	N	2014/01/06	Influenza A virus (A/goose/China/14016HA-4.seq/2014)	c
MN556689	1704	Avian	4 (HA)	H5N6	China	N	2014/01/06	Influenza A virus (A/duck/China/14017HA-4.seq/2014)	c
MN556725	1704	Avian	4 (HA)	H5N6	China	N	2014/03/15	Influenza A virus (A/duck/China/14085HA-4.seq/2014)	c
MN559704	1695	Avian	4 (HA)	H5N8	Egypt	N	2017/01/22	Influenza A virus (A/goose/Egypt/A_Geese_Egypt_MG3_2017/2017)	c
MN559705	1567	Avian	4 (HA)	H5N8	Egypt	N	2019/04/01	Influenza A virus (A/goose/Egypt/A_Geese_Egypt_MG4_2019/2019)	p
MN565986	1701	Avian	4 (HA)	H5N6	South Korea	N	2017/12/12	Influenza A virus (A/Anas platyrhynchos/South Korea/1702/2017)	c
MN566011	1701	Avian	4 (HA)	H5N6	South Korea	N	2017/12	Influenza A virus (A/Anas platyrhynchos/South Korea/1703/2017)	c
MN566019	1701	Avian	4 (HA)	H5N6	South Korea	N	2017/12	Influenza A virus (A/Wild bird(Anas platyrhynchos)/South Korea/1705/2017)	c
MN566028	1701	Avian	4 (HA)	H5N6	South Korea	N	2017/12	Influenza A virus (A/Anas platyrhynchos/South Korea/1709/2017)	c
MN566036	1701	Avian	4 (HA)	H5N6	South Korea	N	2017/12	Influenza A virus (A/wild bird(Anas platyrhynchos)/South Korea/1710/2017)	c
MN566053	1704	Avian	4 (HA)	H5N6	South Korea	N	2018/11	Influenza A virus (A/wild duck/South Korea/1801/2018)	c
MN577280	1701	Avian	4 (HA)	H5N6	South Korea	N	2019/08	Influenza A virus (A/wild duck/South Korea/1908/2019)	c
MN577314	1701	Avian	4 (HA)	H5N6	South Korea	N	2019/08	Influenza A virus (A/wild duck/South Korea/1914/2019)	c
MN577323	1701	Avian	4 (HA)	H5N6	South Korea	N	2019/08	Influenza A virus (A/wild duck/South Korea/1915/2019)	c
MN577334	1701	Avian	4 (HA)	H5N6	South Korea	N	2019/08	Influenza A virus (A/wild duck/South Korea/1920/2019)	c
MN577345	1701	Avian	4 (HA)	H5N6	South Korea	N	2019/08	Influenza A virus (A/wild duck/South Korea/1922/2019)	c
MN708201	1776	Avian	4 (HA)	H5N8	Denmark	N	2016/11/07	Influenza A virus (A/Tufted duck/Denmark/11740-LWPL/2016)	c
MN759479	1704	Avian	4 (HA)	H5N8	Nigeria	T	2017/09	Influenza A virus (A/duck/Nigeria/KNSB-11-13T_18RS1971-38/2017)	c
MN759480	1704	Avian	4 (HA)	H5N8	Nigeria	T	2017/09	Influenza A virus (A/duck/Nigeria/KNSB-8-10T_18RS1971-37/2017)	c
MN759481	1704	Avian	4 (HA)	H5N8	Nigeria	T	2017/09	Influenza A virus (A/duck/Nigeria/KNSB-20-23T_18RS1971-39/2017)	c
MN759482	1704	Avian	4 (HA)	H5N8	Nigeria	T	2017/09	Influenza A virus (A/duck/Nigeria/KDIG-164-165T_18RS1971-40/2017)	c
MN759483	1704	Avian	4 (HA)	H5N8	Nigeria	T	2017/09	Influenza A virus (A/duck/Nigeria/KDIG-171-172C_18RS1971-41/2017)	c
MN759484	1704	Avian	4 (HA)	H5N8	Nigeria	T	2017/09	Influenza A virus (A/duck/Nigeria/KDIG-173-174_18RS1971-42/2017)	c
MN759485	1704	Avian	4 (HA)	H5N8	Nigeria	T	2017/02	Influenza A virus (A/mallard_duck/Nigeria/KN-AR91-3T_18RS1971-45/2017)	c
MN759486	1704	Avian	4 (HA)	H5N8	Nigeria	T	2017/02	Influenza A virus (A/muscovy_duck/Nigeria/KN-P31-33T_18RS1971-46/2017)	c
MN759487	1704	Avian	4 (HA)	H5N8	Nigeria	T	2017/02	Influenza A virus (A/muscovy_duck/Nigeria/KN-P52-53T_18RS1971-47/2017)	c
MN759488	1704	Avian	4 (HA)	H5N8	Nigeria	T	2017/02	Influenza A virus (A/muscovy_duck/Nigeria/KN-AR71-3T_18RS1971-49/2017)	c
MN759489	1704	Avian	4 (HA)	H5N8	Nigeria	T	2017/02	Influenza A virus (A/duck/Nigeria/KN-K71-4C_18RS1971-50/2017)	c
MN759490	1704	Avian	4 (HA)	H5N8	Nigeria	T	2016/11	Influenza A virus (A/chicken/Nigeria/VRD16-KN623_17RS737-43/2016)	c
MN809352	1704	Avian	4 (HA)	H5N6	South Korea	N	2018/12	Influenza A virus (A/Wild duck/South Korea/1804/2018)	c
MN875108	1656	Avian	4 (HA)	H5N8	France	N	2016/12/28	Influenza A virus (A/chicken/France/161585/2016)	p
MN875109	1656	Avian	4 (HA)	H5N8	France	N	2016/12/18	Influenza A virus (A/eurasian wigeon/France/161323/2016)	p
MT027086	1704	Avian	4 (HA)	H5N8	Slovakia	N	2020/01/03	Influenza A virus (A/layer hen/Slovakia/A-chicken-Slovakia-Pah_14-2020_H5N8_HA/	c
MT200035	1762	Avian	4 (HA)	H5N6	Viet Nam	T	2019/12/30	Influenza A virus (A/Domestic duck/Viet Nam/A_duck_Vietnam_HU12-1473_2019/2	c
MT256068	1704	Avian	4 (HA)	H5N8	Egypt	N	2019/03/16	Influenza A virus (A/Broiler chicken/Giza/VRLCU/2019)	c
MT256069	1704	Avian	4 (HA)	H5N8	Egypt	N	2019/05/10	Influenza A virus (A/cattle egret/Monofiya/VRLCU/2019)	c
MT547570	1758	Avian	4 (HA)	H5N1	Viet Nam	T	2019/08	Influenza A virus (A/chicken/Viet Nam/HU12-1328/2019)	c
MT781470	1704	Avian	4 (HA)	H5N6	Denmark	N	2018/09/03	Influenza A virus (A/eider/Denmark/12292-1/2018)	c
MT781474	1702	Avian	4 (HA)	H5N6	Denmark	N	2018/09/03	Influenza A virus (A/mute swan/Denmark/12293-1/2018)	nc
MT781490	1704	Avian	4 (HA)	H5N6	Denmark	N	2018/04/18	Influenza A virus (A/common buzzard/Denmark/4907-1/2018)	c
MT781495	1704	Avian	4 (HA)	H5N6	Denmark	N	2019/01/04	Influenza A virus (A/common buzzard/Denmark/613-1/2019)	c
MT781503	1704	Avian	4 (HA)	H5N6	Denmark	N	2018/07/11	Influenza A virus (A/eider/Denmark/9885-1/2018)	c
MT781511	1704	Avian	4 (HA)	H5N6	Denmark	N	2018/08/12	Influenza A virus (A/mute swan/Denmark/10771-1S/2018)	c
MT781519	1704	Avian	4 (HA)	H5N6	Denmark	N	2018/03/27	Influenza A virus (A/mute swan/Denmark/4905-1/2018)	c

MT781535	1704	Avian	4 (HA)	H5N6	Denmark	N	2018/12/22	Influenza A virus (A/white-tailed eagle/Denmark/0429-1/2018)	c
MT781546	1704	Avian	4 (HA)	H5N6	Denmark	N	2018/02/13	Influenza A virus (A/white-tailed eagle/Denmark/3073-1/2018)	c
MT781554	1704	Avian	4 (HA)	H5N6	Denmark	N	2018/02/09	Influenza A virus (A/white-tailed eagle/Denmark/4227-1/2018)	c
MT781562	1704	Avian	4 (HA)	H5N6	Denmark	N	2018/03/16	Influenza A virus (A/white-tailed eagle/Denmark/4240-1/2018)	c
MT781570	1704	Avian	4 (HA)	H5N6	Denmark	N	2018/03/14	Influenza A virus (A/white-tailed eagle/Denmark/4241-1/2018)	c
MT781578	1704	Avian	4 (HA)	H5N6	Denmark	N	2018/04/03	Influenza A virus (A/white-tailed eagle/Denmark/4896-1/2018)	c
MT781586	1704	Avian	4 (HA)	H5N6	Denmark	N	2018/04/02	Influenza A virus (A/white-tailed eagle/Denmark/4897-1/2018)	c
MT781591	1704	Avian	4 (HA)	H5N6	Denmark	N	2019/03/27	Influenza A virus (A/white-tailed eagle/Denmark/4903-1/2019)	c
MT781602	1704	Avian	4 (HA)	H5N6	Denmark	N	2018/02/12	Influenza A virus (A/white-tailed eagle/Denmark/5921-1/2018)	c
MT912721	1701	Avian	4 (HA)	H5N6	China	N	2017/12	Influenza A virus (A/egret/Zhejiang/W18/2017)	c
MT912731	1704	Avian	4 (HA)	H5N6	China	N	2017/12	Influenza A virus (A/egret/Zhejiang/W15/2017)	c
MW026072	1737	Avian	4 (HA)	H5N8	Denmark	N	2017/03/23	Influenza A virus (A/common buzzard/Denmark/4079-1p1/2017)	c
MW026080	1737	Avian	4 (HA)	H5N8	Denmark	N	2017/03/03	Influenza A virus (A/common buzzard/Denmark/3794-1p1/2017)	c
MW026088	1737	Avian	4 (HA)	H5N8	Denmark	N	2016/11/16	Influenza A virus (A/common gull/Denmark/18577-1/2016)	c
MW026112	1737	Avian	4 (HA)	H5N8	Denmark	N	2016/11/16	Influenza A virus (A/great black-backed gull/Denmark/19069-1/2016)	c
MW026120	1737	Avian	4 (HA)	H5N8	Denmark	N	2016/11/19	Influenza A virus (A/mute swan/Denmark/19192-1/2016)	c
MW026128	1737	Avian	4 (HA)	H5N8	Denmark	N	2016/11/07	Influenza A virus (A/tufted duck/Denmark/17740-1p1/2016)	c
MW097446	1704	Environment	4 (HA)	H5N6	China	N	2017/02/15	Influenza A virus (A/environment/Yunnan/2.15_DQXC083-E/2017)	c
MW097447	1704	Environment	4 (HA)	H5N6	China	N	2017/03/02	Influenza A virus (A/environment/Yunnan/3.20_DQSPZ024-E/2017)	c
MW097754	1745	Avian	4 (HA)	H5N6	China	N	2018/03/15	Influenza A virus (A/goose/Fujian/3.15_FZHX0004-O/2018)	c
MW097755	1755	Avian	4 (HA)	H5N6	China	N	2018/03/15	Influenza A virus (A/goose/Fujian/3.15_FZHX0006-O/2018)	c
MW097756	1757	Avian	4 (HA)	H5N6	China	N	2018/03/15	Influenza A virus (A/goose/Fujian/3.15_FZHX0009-O/2018)	c
MW097768	1769	Avian	4 (HA)	H5N6	China	N	2018/12/26	Influenza A virus (A/chicken/Shandong/12.26_TAWL023-O/2018)	c
MW097770	1770	Environment	4 (HA)	H5N6	China	N	2018/12/26	Influenza A virus (A/environment/Shandong/12.26_TAWL002-E/2018)	c
MW097774	1769	Avian	4 (HA)	H5N6	China	N	2018/11/03	Influenza A virus (A/chicken/Shanxi/11.30_TGRL018-O/2018)	c
MW097777	1773	Avian	4 (HA)	H5N6	China	N	2018/11/28	Influenza A virus (A/chicken/Shandong/11.28_TAWL019-O/2018)	c
MW097778	1769	Avian	4 (HA)	H5N6	China	N	2018/11/28	Influenza A virus (A/chicken/Shandong/11.28_TAWL021-O/2018)	c
MW097779	1769	Avian	4 (HA)	H5N6	China	N	2018/11/28	Influenza A virus (A/chicken/Shandong/11.28_TAWL022-O/2018)	c
MW097780	1769	Avian	4 (HA)	H5N6	China	N	2018/11/28	Influenza A virus (A/chicken/Shandong/11.28_TAWL024-O/2018)	c
MW097781	1769	Environment	4 (HA)	H5N6	China	N	2018/11/28	Influenza A virus (A/environment/Shandong/11.28_TAWL002-E/2018)	c
MW097782	1769	Avian	4 (HA)	H5N6	China	N	2018/11/01	Influenza A virus (A/chicken/Henan/11.01_XXHM013-C/2018)	c
MW097784	1773	Avian	4 (HA)	H5N6	China	N	2018/11/29	Influenza A virus (A/chicken/Anhui/11.29_YHZGS007-O/2018)	c
MW097785	1773	Avian	4 (HA)	H5N6	China	N	2018/11/29	Influenza A virus (A/chicken/Anhui/11.29_YHZGS009-O/2018)	c
MW097786	1769	Avian	4 (HA)	H5N6	China	N	2018/11/29	Influenza A virus (A/chicken/Anhui/11.29_YHZGS010-O/2018)	c
MW097787	1769	Avian	4 (HA)	H5N6	China	N	2018/11/29	Influenza A virus (A/chicken/Anhui/11.29_YHZGS011-O/2018)	c
MW097788	1769	Avian	4 (HA)	H5N6	China	N	2018/11/29	Influenza A virus (A/chicken/Anhui/11.29_YHZGS012-O/2018)	c
MW097789	1772	Avian	4 (HA)	H5N6	China	N	2018/11/29	Influenza A virus (A/chicken/Anhui/11.29_YHZGS013-O/2018)	c
MW097790	1769	Avian	4 (HA)	H5N6	China	N	2018/11/29	Influenza A virus (A/chicken/Anhui/11.29_YHZGS014-O/2018)	c
MW097791	1771	Avian	4 (HA)	H5N6	China	N	2018/11/29	Influenza A virus (A/chicken/Anhui/11.29_YHZGS015-O/2018)	c
MW097792	1769	Avian	4 (HA)	H5N6	China	N	2018/11/29	Influenza A virus (A/chicken/Anhui/11.29_YHZGS016-O/2018)	c
MW097793	1769	Avian	4 (HA)	H5N6	China	N	2018/11/29	Influenza A virus (A/chicken/Anhui/11.29_YHZGS017-O/2018)	c
MW097794	1769	Avian	4 (HA)	H5N6	China	N	2018/11/29	Influenza A virus (A/chicken/Anhui/11.29_YHZGS019-O/2018)	c
MW097795	1772	Avian	4 (HA)	H5N6	China	N	2018/11/29	Influenza A virus (A/chicken/Anhui/11.29_YHZGS020-O/2018)	c
MW097796	1769	Avian	4 (HA)	H5N6	China	N	2018/11/29	Influenza A virus (A/chicken/Anhui/11.29_YHZGS022-O/2018)	c
MW097797	1769	Avian	4 (HA)	H5N6	China	N	2018/11/29	Influenza A virus (A/chicken/Anhui/11.29_YHZGS023-O/2018)	c
MW097798	1769	Avian	4 (HA)	H5N6	China	N	2018/11/29	Influenza A virus (A/chicken/Anhui/11.29_YHZGS024-O/2018)	c
MW097799	1769	Avian	4 (HA)	H5N6	China	N	2018/11/29	Influenza A virus (A/chicken/Anhui/11.29_YHZGS025-O/2018)	c
MW097800	1772	Avian	4 (HA)	H5N6	China	N	2018/11/29	Influenza A virus (A/chicken/Anhui/11.29_YHZGS001-C/2018)	c
MW097801	1771	Avian	4 (HA)	H5N6	China	N	2018/11/29	Influenza A virus (A/chicken/Anhui/11.29_YHZGS004-C/2018)	c
MW097802	1769	Avian	4 (HA)	H5N6	China	N	2018/11/29	Influenza A virus (A/chicken/Anhui/11.29_YHZGS006-C/2018)	c
MW097803	1773	Avian	4 (HA)	H5N6	China	N	2018/11/29	Influenza A virus (A/chicken/Anhui/11.29_YHZGS009-C/2018)	c
MW097804	1773	Avian	4 (HA)	H5N6	China	N	2018/11/29	Influenza A virus (A/chicken/Anhui/11.29_YHZGS019-C/2018)	c
MW097805	1773	Avian	4 (HA)	H5N6	China	N	2018/11/29	Influenza A virus (A/chicken/Anhui/11.29_YHZGS021-C/2018)	c
MW097807	1773	Environment	4 (HA)	H5N6	China	N	2018/11/29	Influenza A virus (A/environment/Anhui/11.29_YHZGS001-E/2018)	c
MW097810	1771	Avian	4 (HA)	H5N6	China	N	2018/10/28	Influenza A virus (A/chicken/Chongqing/10.28_RCTYH003-O/2018)	c
MW097811	1771	Avian	4 (HA)	H5N6	China	N	2018/10/28	Influenza A virus (A/chicken/Chongqing/10.28_RCTYH020-O/2018)	c
MW097812	1773	Avian	4 (HA)	H5N6	China	N	2018/11/29	Influenza A virus (A/chicken/Henan/11.29_XXHM015-O/2018)	c
MW097813	1769	Avian	4 (HA)	H5N6	China	N	2018/11/29	Influenza A virus (A/chicken/Henan/11.29_XXHM017-O/2018)	c
MW097815	1769	Environment	4 (HA)	H5N6	China	N	2018/11/29	Influenza A virus (A/environment/Henan/11.29_XXHM001-E/2018)	c
MW097816	1773	Environment	4 (HA)	H5N6	China	N	2018/11/29	Influenza A virus (A/environment/Henan/11.29_XXHM003-E/2018)	c
MW097817	1773	Environment	4 (HA)	H5N6	China	N	2018/11/29	Influenza A virus (A/environment/Henan/11.29_XXHM004-E/2018)	c
MW097818	1769	Avian	4 (HA)	H5N6	China	N	2018/11/03	Influenza A virus (A/chicken/Shanxi/11.30_TGRL002-O/2018)	c
MW097819	1770	Avian	4 (HA)	H5N6	China	N	2018/12/01	Influenza A virus (A/chicken/Shanxi/12.01_TGRL005-O/2018)	c
MW097820	1770	Avian	4 (HA)	H5N6	China	N	2018/11/03	Influenza A virus (A/chicken/Shanxi/11.30_TGRL007-O/2018)	c
MW097823	1773	Avian	4 (HA)	H5N2	China	N	2018/10/28	Influenza A virus (A/chicken/Chongqing/10.28_RCTYH007-O/2018)	c
MW097824	1574	Avian	4 (HA)	H5N6	China	N	2018/08/28	Influenza A virus (A/chicken/Anhui/8.28_YHZGS012-O/2018)	p
MW097827	1753	Avian	4 (HA)	H5N6	China	N	2017/05/26	Influenza A virus (A/chicken/Jiangxi/5.26_NCNP13Q2-OC/2017)	c
MW097845	1755	Environment	4 (HA)	H5N2	China	N	2017/01/11	Influenza A virus (A/environment/Jiangxi/1.11_NCNP65E2-E/2017)	c
MW097850	1754	Environment	4 (HA)	H5N6	China	N	2017/01/11	Influenza A virus (A/environment/Jiangxi/1.11_NCDZT99F2-E/2017)	c
MW097861	1751	Avian	4 (HA)	H5N6	China	N	2017/10/03	Influenza A virus (A/chicken/Jiangxi/10.30_NCDZT17B3-OC/2017)	c
MW097866	1747	Avian	4 (HA)	H5N6	China	N	2018/03/07	Influenza A virus (A/duck/Hunan/03.07_YYGK31L3-OC/2018)	c
MW097868	1750	Avian	4 (HA)	H5N6	China	N	2018/03/07	Influenza A virus (A/chicken/Hunan/03.07_YYGK40L3-OC/2018)	c
MW097948	1769	Avian	4 (HA)	H5N6	China	N	2018/08/28	Influenza A virus (A/chicken/Henan/8.28_XXHM003-O/2018)	c
MW097949	1769	Avian	4 (HA)	H5N6	China	N	2018/08/28	Influenza A virus (A/chicken/Henan/8.28_XXHM007-O/2018)	c
MW097950	1771	Avian	4 (HA)	H5N6	China	N	2018/08/28	Influenza A virus (A/chicken/Henan/8.28_XXHM016-O/2018)	c
MW097951	1773	Avian	4 (HA)	H5N6	China	N	2018/08/28	Influenza A virus (A/chicken/Henan/8.28_XXHM004-C/2018)	c
MW097952	1770	Avian	4 (HA)	H5N6	China	N	2018/08/28	Influenza A virus (A/chicken/Shanxi/8.28_TGRL001-O/2018)	c
MW097953	1771	Avian	4 (HA)	H5N6	China	N	2018/08/28	Influenza A virus (A/chicken/Shanxi/8.28_TGRL002-O/2018)	c
MW097954	1771	Avian	4 (HA)	H5N6	China	N	2018/08/28	Influenza A virus (A/chicken/Shanxi/8.28_TGRL004-O/2018)	c
MW097955	1772	Avian	4 (HA)	H5N6	China	N	2018/08/28	Influenza A virus (A/chicken/Shanxi/8.28_TGRL009-O/2018)	c
MW097956	1769	Avian	4 (HA)	H5N6	China	N	2018/08/28	Influenza A virus (A/chicken/Shanxi/8.28_TGRL011-O/2018)	c
MW097957	1772	Avian	4 (HA)	H5N6	China	N	2018/08/28	Influenza A virus (A/chicken/Shanxi/8.28_TGRL016-O/2018)	c
MW097958	1773	Avian	4 (HA)	H5N6	China	N	2018/08/28	Influenza A virus (A/chicken/Shanxi/8.28_TGRL020-O/2018)	c

MW097959	1773	Avian	4 (HA)	H5N6	China	N	2018/08/28	Influenza A virus (A/chicken/Shanxi/8.28_TGRL023-O/2018)	c
MW097960	1772	Avian	4 (HA)	H5N6	China	N	2018/08/28	Influenza A virus (A/chicken/Shanxi/8.28_TGRL018-C/2018)	c
MW098006	1773	Environment	4 (HA)	H5N6	China	N	2019/01/23	Influenza A virus (A/environment/Shanxi/01.23_TGRL004-E/2019)	c
MW098013	1773	Avian	4 (HA)	H5N6	China	N	2019/01/26	Influenza A virus (A/chicken/Shandong/01.26_TAWL003-C/2019)	c
MW098017	1748	Avian	4 (HA)	H5N6	China	N	2019/01/26	Influenza A virus (A/chicken/Shandong/01.26_TAWL017-C/2019)	c
MW098019	1769	Environment	4 (HA)	H5	China	N	2018/12/26	Influenza A virus (A/environment/Shandong/12.26_TAWL001-E/2018)	c
MW098064	1749	Avian	4 (HA)	H5	China	N	2018/10/26	Influenza A virus (A/duck/Zhejiang/10.26_HZBX001-C/2018)	c
MW098069	1767	Avian	4 (HA)	H5	China	N	2018/10/26	Influenza A virus (A/duck/Zhejiang/10.26_HZBX004-C/2018)	c
MW098073	1742	Avian	4 (HA)	H5	China	N	2018/10/26	Influenza A virus (A/duck/Zhejiang/10.26_HZBX012-C/2018)	c
MW098112	1745	Environment	4 (HA)	H5	China	N	2018/12/02	Influenza A virus (A/environment/Inner_mongolia/12.02_EEDSWSQ013-E/2018)	c
MW098121	1769	Avian	4 (HA)	H5	China	N	2018/03/21	Influenza A virus (A/chicken/Yunnan/3.21_DQXBL001-C/2018)	c
MW098127	1773	Avian	4 (HA)	H5	China	N	2017/03/08	Influenza A virus (A/chicken/Jilin/3.08_CCNQM002-O/2017)	c
MW098134	1769	Avian	4 (HA)	H5	China	N	2018/12/24	Influenza A virus (A/chicken/Xinjiang/12.24_WLMQXL023-O/2018)	c
MW098143	1748	Avian	4 (HA)	H5	China	N	2018/10/13	Influenza A virus (A/chicken/Xinjiang/10.13_WLMQXL014-O/2018)	c
MW098144	1699	Avian	4 (HA)	H5	China	N	2018/10/13	Influenza A virus (A/chicken/Xinjiang/10.13_WLMQXL018-O/2018)	p
MW098145	1747	Avian	4 (HA)	H5	China	N	2018/10/13	Influenza A virus (A/duck/Xinjiang/10.13_WLMQXL005-O/2018)	nc
MW098147	1712	Avian	4 (HA)	H5	China	N	2018/09/19	Influenza A virus (A/chicken/Xinjiang/09.19_WLMQXL002-O/2018)	p
MW098148	1728	Avian	4 (HA)	H5	China	N	2018/09/19	Influenza A virus (A/chicken/Xinjiang/09.19_WLMQXL008-O/2018)	p
MW098156	1773	Avian	4 (HA)	H5N6	China	N	2018/08/26	Influenza A virus (A/chicken/Fujian/8.26_FZHX0065-C/2018)	c
MW098164	1773	Avian	4 (HA)	H5	China	N	2018/01/25	Influenza A virus (A/duck/Fujian/1.25_FZHX0043-O/2018)	c
MW098165	1773	Avian	4 (HA)	H5	China	N	2018/09/24	Influenza A virus (A/chicken/Fujian/9.24_FZHX0087-C/2018)	c
MW098167	1770	Avian	4 (HA)	H5	China	N	2018/10/23	Influenza A virus (A/chicken/Fujian/10.23_FZHX0106-O/2018)	c
MW098169	1772	Avian	4 (HA)	H5N6	China	N	2018/10/23	Influenza A virus (A/duck/Fujian/10.23_FZHX0142-C/2018)	c
MW098170	1773	Avian	4 (HA)	H5N6	China	N	2018/11/26	Influenza A virus (A/duck/Fujian/11.26_FZHX0143-O/2018)	c
MW098178	1773	Avian	4 (HA)	H5	China	N	2018/11/26	Influenza A virus (A/duck/Fujian/11.26_FZHX0193-C/2018)	c
MW098250	1770	Avian	4 (HA)	H5N6	China	N	2018/03/23	Influenza A virus (A/chicken/Jiangxi/3.23_NCDZT15M3-OC/2018)	c
MW098251	1769	Avian	4 (HA)	H5N6	China	N	2018/03/23	Influenza A virus (A/chicken/Jiangxi/3.23_NCDZT57M3-OC/2018)	c
MW098252	1773	Avian	4 (HA)	H5N6	China	N	2018/03/23	Influenza A virus (A/chicken/Jiangxi/3.23_NCDZT63M3-OC/2018)	c
MW098253	1770	Avian	4 (HA)	H5N6	China	N	2018/03/23	Influenza A virus (A/duck/Jiangxi/3.23_NCNP87M3-OC/2018)	c
MW098254	1769	Avian	4 (HA)	H5N6	China	N	2018/04/25	Influenza A virus (A/chicken/Jiangxi/4.25_NCNP6Q3-OC/2018)	c
MW098257	1773	Avian	4 (HA)	H5N6	China	N	2018/04/25	Influenza A virus (A/chicken/Jiangxi/4.25_NCDZT70Q3-OC/2018)	c
MW098267	1773	Avian	4 (HA)	H5N6	China	N	2018/04/26	Influenza A virus (A/chicken/Hunan/04.26_YYGK71R3-OC/2018)	c
MW098273	1769	Avian	4 (HA)	H5N6	China	N	2018/08/27	Influenza A virus (A/chicken/Hunan/8.27_YYGK70W3-OC/2018)	c
MW098274	1769	Avian	4 (HA)	H5N6	China	N	2018/10/27	Influenza A virus (A/chicken/Hunan/10.27_YYGK83B4-OC/2018)	c
MW098276	1773	Avian	4 (HA)	H5N6	China	N	2018/10/27	Influenza A virus (A/chicken/Hunan/10.27_YYGK55B4-OC/2018)	c
MW098277	1769	Avian	4 (HA)	H5N6	China	N	2018/12/24	Influenza A virus (A/chicken/Hunan/12.24_YYGK63E4-O/2018)	c
MW098278	1773	Avian	4 (HA)	H5N6	China	N	2018/12/24	Influenza A virus (A/chicken/Hunan/12.24_YYGK35E4-O/2018)	c
MW098281	1769	Avian	4 (HA)	H5	China	N	2018/03/23	Influenza A virus (A/duck/Jiangxi/3.23_NCNP99M3-OC/2018)	c
MW098299	1773	Avian	4 (HA)	H5	China	N	2018/05/28	Influenza A virus (A/duck/Jiangxi/5.28_NCNP15N3-OC/2018)	c
MW098303	1772	Avian	4 (HA)	H5	China	N	2018/06/21	Influenza A virus (A/duck/Jiangxi/6.21_NCDZT2S3-OC/2018)	c
MW098310	1772	Avian	4 (HA)	H5	China	N	2018/07/25	Influenza A virus (A/chicken/Jiangxi/7.25_NCNP64U3-OC/2018)	c
MW098313	1771	Avian	4 (HA)	H5	China	N	2018/08/28	Influenza A virus (A/duck/Jiangxi/8.28_NCNP19X3-OC/2018)	c
MW098322	1769	Avian	4 (HA)	H5	China	N	2018/09/22	Influenza A virus (A/duck/Jiangxi/9.22_NCDZT93Z3-OC/2018)	c
MW098334	1769	Avian	4 (HA)	H5	China	N	2018/11/27	Influenza A virus (A/duck/Jiangxi/11.27_NCNP93C4-OC/2018)	c
MW098337	1769	Avian	4 (HA)	H5	China	N	2018/11/27	Influenza A virus (A/chicken/Jiangxi/11.27_NCDZT28C4-OC/2018)	c
MW098339	1772	Avian	4 (HA)	H5	China	N	2018/12/17	Influenza A virus (A/chicken/Jiangxi/12.17_NCDZT57D4-O/2018)	c
MW098360	1769	Avian	4 (HA)	H5	China	N	2018/04/26	Influenza A virus (A/duck/Hunan/04.26_YYGK46R3-OC/2018)	c
MW098362	1769	Avian	4 (HA)	H5	China	N	2018/05/29	Influenza A virus (A/chicken/Hunan/5.29_YYGK1P3-OC/2018)	c
MW098364	1771	Avian	4 (HA)	H5	China	N	2018/05/29	Influenza A virus (A/chicken/Hunan/5.29_YYGK23P3-OC/2018)	c
MW098370	1769	Avian	4 (HA)	H5	China	N	2018/05/29	Influenza A virus (A/chicken/Hunan/5.29_YYGK48P3-OC/2018)	c
MW098374	1773	Avian	4 (HA)	H5	China	N	2018/05/29	Influenza A virus (A/duck/Hunan/5.29_YYGK100P3-OC/2018)	c
MW098426	1773	Avian	4 (HA)	H5N6	China	N	2019/01/26	Influenza A virus (A/chicken/Shandong/1.26_TAWL020-C/2019)	c
MW098427	1773	Avian	4 (HA)	H5N6	China	N	2019/01/26	Influenza A virus (A/chicken/Shandong/1.26_TAWL023-C/2019)	c
MW098430	1771	Environment	4 (HA)	H5N6	China	N	2019/01/26	Influenza A virus (A/environment/Shandong/1.26_TAWL002-E/2019)	c
MW098431	1773	Avian	4 (HA)	H5N6	China	N	2018/12/27	Influenza A virus (A/chicken/Shanxi/12.27_TGRL005-C/2018)	c
MW098433	1770	Avian	4 (HA)	H5N6	China	N	2019/02/22	Influenza A virus (A/chicken/Anhui/2.22_YHZGS004-O/2019)	c
MW098435	1769	Avian	4 (HA)	H5N6	China	N	2019/02/22	Influenza A virus (A/chicken/Anhui/2.22_YHZGS008-O/2019)	c
MW098436	1769	Avian	4 (HA)	H5N6	China	N	2019/02/22	Influenza A virus (A/chicken/Anhui/2.22_YHZGS013-O/2019)	c
MW098437	1772	Avian	4 (HA)	H5N6	China	N	2019/02/22	Influenza A virus (A/chicken/Anhui/2.22_YHZGS015-C/2019)	c
MW098439	1773	Avian	4 (HA)	H5N6	China	N	2019/02/22	Influenza A virus (A/chicken/Anhui/2.22_YHZGS022-C/2019)	c
MW098445	1769	Environment	4 (HA)	H5N6	China	N	2019/02/27	Influenza A virus (A/environment/Henan/2.27_XXHM003-E/2019)	c
MW098446	1769	Environment	4 (HA)	H5N6	China	N	2019/02/27	Influenza A virus (A/environment/Henan/2.27_XXHM004-E/2019)	c
MW098447	1769	Environment	4 (HA)	H5N6	China	N	2019/02/27	Influenza A virus (A/environment/Henan/2.27_XXHM005-E/2019)	c
MW098448	1769	Avian	4 (HA)	H5N6	China	N	2019/02/22	Influenza A virus (A/chicken/Shanxi/2.22_TGRL001-O/2019)	c
MW098449	1769	Environment	4 (HA)	H5N6	China	N	2019/02/22	Influenza A virus (A/environment/Shanxi/2.22_TGRL003-E/2019)	c
MW098450	1772	Environment	4 (HA)	H5N6	China	N	2019/02/22	Influenza A virus (A/environment/Shanxi/2.22_TGRL004-E/2019)	c
MW098451	1769	Environment	4 (HA)	H5N6	China	N	2019/02/22	Influenza A virus (A/environment/Shanxi/2.22_TGRL005-E/2019)	c
MW098452	1769	Avian	4 (HA)	H5N6	China	N	2019/02/25	Influenza A virus (A/chicken/Shandong/2.25_TAWL001-O/2019)	c
MW098453	1769	Avian	4 (HA)	H5N6	China	N	2019/02/25	Influenza A virus (A/chicken/Shandong/2.25_TAWL003-O/2019)	c
MW098454	1745	Environment	4 (HA)	H5	China	N	2019/01/26	Influenza A virus (A/environment/Shandong/1.26_TAWL005-E/2019)	nc
MW109328	1701	Environment	4 (HA)	H5N6	China	N	2017/10/26	Influenza A virus (A/environment/Fujian/10.26_FZHX0002-E/2017)	c
MW109336	1766	Environment	4 (HA)	H5N6	China	N	2016/12/12	Influenza A virus (A/environment/Henan/12.12_HNSQ001-E/2016)	c
MW109416	1704	Environment	4 (HA)	H5N8	China	N	2017/01/06	Influenza A virus (A/environment/Inner_mongolia/1.06_EEDSWSQ001-E/2017)	c
MW109424	1704	Environment	4 (HA)	H5N8	China	N	2016/10/12	Influenza A virus (A/environment/Inner_mongolia/10.12_EEDSWSQ001-E/2016)	c
MW109832	1704	Environment	4 (HA)	H5N8	China	N	2017/01/02	Influenza A virus (A/environment/Shanxi/1.02_YC001-E/2017)	c
MW109840	1704	Environment	4 (HA)	H5N8	China	N	2017/01/01	Influenza A virus (A/environment/XIZang/1.10_LS001-E/2017)	c
MW109848	1704	Environment	4 (HA)	H5N8	China	N	2016/10/12	Influenza A virus (A/environment/Xinjiang/10.12_WLMQXL001-E/2016)	c
MW109856	1704	Environment	4 (HA)	H5N8	China	N	2016/12/18	Influenza A virus (A/environment/Xinjiang/12.18_WLMQXL001-E/2016)	c
MW269590	1697	Avian	4 (HA)	H5N8	China	N	2020/10/16	Influenza A virus (A/common teal/Shanghai/JDS20103116/2020)	p
MW275973	1698	Avian	4 (HA)	H5N8	China	N	2020/11/16	Influenza A virus (A/tundra swan/Shanghai/CM20111601/2020)	p
MW314795	1701	Avian	4 (HA)	H5N6	China	N	2019/11	Influenza A virus (A/chicken/China/1101/2019)	c
MW334838	1776	Avian	4 (HA)	H5N2	Taiwan	N	2017/01/11	Influenza A virus (A/heron/Yilan/17DB0001/2017)	c
MW334846	1776	Avian	4 (HA)	H5N2	Taiwan	N	2015/12/14	Influenza A virus (A/thrush/Taipei City/15120021/2015)	c

MW689547	1729	Avian	4 (HA)	H5N6	Mongolia	N	2020/04	Influenza A virus (A/Swan goose/Mongolia/02/2020)	c
MW926584	1761	Avian	4 (HA)	H5N6	Viet Nam	T	2014/10/06	Influenza A virus (A/Moscovy duck/Viet Nam/1914/2014)	c
MW934713	1773	Avian	4 (HA)	H5N6	Viet Nam	T	2018/12/05	Influenza A virus (A/Muscovy duck/Vietnam/HN5135/2018)	c
MW935434	1773	Avian	4 (HA)	H5N6	Viet Nam	T	2018/12/05	Influenza A virus (A/Muscovy duck/Vietnam/HN5137/2018)	c
MW935531	1776	Avian	4 (HA)	H5N6	Viet Nam	T	2018/12/05	Influenza A virus (A/duck/Vietnam/HN5149/2018)	c
MW935546	1773	Avian	4 (HA)	H5N6	Viet Nam	T	2018/12/05	Influenza A virus (A/Muscovy duck/Vietnam/HN5132/2018)	c
MW935607	1776	Avian	4 (HA)	H5N6	Viet Nam	T	2018/12/05	Influenza A virus (A/duck/Vietnam/HN5142/2018)	c
MW935842	1776	Avian	4 (HA)	H5N6	Viet Nam	T	2018/12/05	Influenza A virus (A/duck/Vietnam/HN5143/2018)	c
MW935851	1773	Avian	4 (HA)	H5N6	Viet Nam	T	2018/12/05	Influenza A virus (A/Muscovy duck/Vietnam/HN5138/2018)	c
MW935867	1776	Avian	4 (HA)	H5N6	Viet Nam	T	2018/12/05	Influenza A virus (A/duck/Vietnam/HN5147/2018)	c
MW935877	1776	Avian	4 (HA)	H5N6	Viet Nam	T	2018/12/05	Influenza A virus (A/duck/Vietnam/HN5145/2018)	c
MW935923	1773	Avian	4 (HA)	H5N6	Viet Nam	T	2018/10/29	Influenza A virus (A/Muscovy duck/Vietnam/HN5049/2018)	c
MW935969	1773	Avian	4 (HA)	H5N6	Viet Nam	T	2018/10/29	Influenza A virus (A/Muscovy duck/Vietnam/HN5047/2018)	c
MW935980	1776	Avian	4 (HA)	H5N6	Viet Nam	T	2018/12/05	Influenza A virus (A/duck/Vietnam/HN5141/2018)	c
MW936050	1773	Avian	4 (HA)	H5N6	Viet Nam	T	2018/10/29	Influenza A virus (A/Muscovy duck/Vietnam/HN5048/2018)	c
MW936065	1773	Avian	4 (HA)	H5N6	Viet Nam	T	2018/08/10	Influenza A virus (A/Muscovy duck/Vietnam/HN4857/2018)	c
MW936079	1773	Avian	4 (HA)	H5N6	Viet Nam	T	2018/08/10	Influenza A virus (A/Muscovy duck/Vietnam/HN4858/2018)	c
MW936093	1773	Avian	4 (HA)	H5N6	Viet Nam	T	2018/10/29	Influenza A virus (A/Muscovy duck/Vietnam/HN5046/2018)	c
MW936120	1773	Avian	4 (HA)	H5N6	Viet Nam	T	2018/08/10	Influenza A virus (A/Muscovy duck/Vietnam/HN4856/2018)	c
MW936123	1773	Avian	4 (HA)	H5N6	Viet Nam	T	2018/12/05	Influenza A virus (A/Muscovy duck/Vietnam/HN5139/2018)	c
MW936140	1773	Avian	4 (HA)	H5N6	Viet Nam	T	2018/11/05	Influenza A virus (A/duck/Vietnam/HN5080/2018)	c
MW936233	1773	Avian	4 (HA)	H5N6	Viet Nam	T	2018/06/09	Influenza A virus (A/Muscovy duck/Vietnam/HN4711/2018)	c
MW936246	1773	Avian	4 (HA)	H5N6	Viet Nam	T	2018/06/09	Influenza A virus (A/duck/Vietnam/HN4721/2018)	c
MW936273	1773	Avian	4 (HA)	H5N6	Viet Nam	T	2018/06/09	Influenza A virus (A/Muscovy duck/Vietnam/HN4719/2018)	c
MW936283	1773	Avian	4 (HA)	H5N6	Viet Nam	T	2018/06/09	Influenza A virus (A/Muscovy duck/Vietnam/HN4714/2018)	c
MW936304	1773	Avian	4 (HA)	H5N6	Viet Nam	T	2018/06/09	Influenza A virus (A/Muscovy duck/Vietnam/HN4716/2018)	c
MW936310	1773	Avian	4 (HA)	H5N6	Viet Nam	T	2018/06/09	Influenza A virus (A/Muscovy duck/Vietnam/HN4720/2018)	c
MW936332	1773	Avian	4 (HA)	H5N6	Viet Nam	T	2018/06/09	Influenza A virus (A/Muscovy duck/Vietnam/HN4715/2018)	c
MW936340	1773	Avian	4 (HA)	H5N6	Viet Nam	T	2018/06/09	Influenza A virus (A/Muscovy duck/Vietnam/HN4718/2018)	c
MW936357	1773	Avian	4 (HA)	H5N6	Viet Nam	T	2018/06/09	Influenza A virus (A/Muscovy duck/Vietnam/HN4713/2018)	c
MW960368	1730	Avian	4 (HA)	H5N8	China	N	2021/01	Influenza A virus (A/Mute Swan/China/Shandong1/2021)	c
MZ166239	1704	Avian	4 (HA)	H5N8	France	N	2020/12/15	Influenza A virus (A/Mule duck/France/20437/2020)	c
MZ166240	1704	Avian	4 (HA)	H5N8	France	N	2021/01/28	Influenza A virus (A/Mule duck/France/21061/2021)	c
MZ166252	1704	Avian	4 (HA)	H5N8	France	N	2020/12/06	Influenza A virus (A/Mule duck/France/20323/2020)	c
MZ166260	1704	Avian	4 (HA)	H5N8	France	N	2020/12/10	Influenza A virus (A/Mule duck/France/20335/2020)	c
MZ166268	1704	Avian	4 (HA)	H5N8	France	N	2020/12/13	Influenza A virus (A/Mule duck/France/20338/2020)	c
MZ166276	1704	Avian	4 (HA)	H5N8	France	N	2020/12/13	Influenza A virus (A/Mule duck/France/20339/2020)	c
MZ166284	1704	Avian	4 (HA)	H5N8	France	N	2020/12/21	Influenza A virus (A/Mule duck/France/20349/2020)	c
MZ166292	1704	Avian	4 (HA)	H5N8	France	N	2020/12/26	Influenza A virus (A/Mule duck/France/20352/2020)	c
MZ166300	1704	Avian	4 (HA)	H5N8	France	N	2020/12/27	Influenza A virus (A/Mule duck/France/20353/2020)	c
MZ166308	1704	Avian	4 (HA)	H5N8	France	N	2021/01/30	Influenza A virus (A/Mule duck/France/21064/2021)	c
MZ166316	1704	Avian	4 (HA)	H5N8	France	N	2021/02/20	Influenza A virus (A/Mule duck/France/21084/2021)	c
MZ191540	1704	Avian	4 (HA)	H5N6	Viet Nam	T	2015/08/21	Influenza A virus (A/Moscovy duck/Viet Nam/4915 HA/2015)	c
MZ235334	1704	Avian	4 (HA)	H5N8	Japan	N	2021/02/03	Influenza A virus (A/white-tailed eagle/Hokkaido/20210127001/2021)	c
MZ620718	1704	Avian	4 (HA)	H5N8	Japan	N	2020/12/22	Influenza A virus (A/Mandarin duck/Kagoshima/d57/2020)	c
OK583834	1701	Avian	4 (HA)	H5N6	China	N	2018/10/26	Influenza A virus (A/duck/Jiangsu/JS80/2018)	c
OK583835	1701	Avian	4 (HA)	H5N6	China	N	2018/10/26	Influenza A virus (A/goose/Jiangsu/JS03/2018)	c
OK583836	1701	Avian	4 (HA)	H5N6	China	N	2019/01/26	Influenza A virus (A/goose/Jiangsu/JS06/2019)	c
OK583837	1701	Avian	4 (HA)	H5N6	China	N	2018/03	Influenza A virus (A/chicken/Shandong/SD25/2018)	c
OK583838	1701	Avian	4 (HA)	H5N6	China	N	2018/01	Influenza A virus (A/chicken/Shandong/SD13/2018)	c
OK583839	1701	Avian	4 (HA)	H5N6	China	N	2018/03	Influenza A virus (A/chicken/Shandong/SD78/2018)	c
OL354495	1777	Avian	4 (HA)	H5N8	Egypt	N	2018/02/05	Influenza A virus (A/chicken/Egypt/N15177A/2018)	c
OL354772	1777	Avian	4 (HA)	H5N8	Egypt	N	2018/02/05	Influenza A virus (A/chicken/Egypt/N15177D/2018)	c
OL354818	1777	Avian	4 (HA)	H5N8	Egypt	N	2018/03/20	Influenza A virus (A/chicken/Egypt/F15366B/2018)	c
OL354826	1777	Avian	4 (HA)	H5N8	Egypt	N	2018/02/05	Influenza A virus (A/chicken/Egypt/N15175A/2018)	c
OL354834	1777	Avian	4 (HA)	H5N8	Egypt	N	2018/02/05	Influenza A virus (A/chicken/Egypt/N15174B/2018)	c
OL354842	1777	Avian	4 (HA)	H5N8	Egypt	N	2018/02/05	Influenza A virus (A/chicken/Egypt/N15175B/2018)	c
OL354865	1777	Avian	4 (HA)	H5N8	Egypt	N	2018/02/05	Influenza A virus (A/chicken/Egypt/N15175D/2018)	c
OL354888	1777	Avian	4 (HA)	H5N8	Egypt	N	2018/01/14	Influenza A virus (A/duck/Egypt/F15089/2018)	c
OL354899	1744	Avian	4 (HA)	H5N8	Egypt	N	2021/02/10	Influenza A virus (A/chicken/Egypt/A19673/2021)	c
OL354917	1777	Avian	4 (HA)	H5N8	Egypt	N	2018/01/14	Influenza A virus (A/duck/Egypt/F15092/2018)	c
OL354925	1777	Avian	4 (HA)	H5N8	Egypt	N	2018/11/24	Influenza A virus (A/duck/Egypt/A16368/2018)	c
OL354942	1777	Avian	4 (HA)	H5N8	Egypt	N	2018/01/15	Influenza A virus (A/chicken/Egypt/A15044/2018)	c
OL354965	1777	Avian	4 (HA)	H5N8	Egypt	N	2018/11/24	Influenza A virus (A/duck/Egypt/A16372/2018)	c
OL354974	1777	Avian	4 (HA)	H5N8	Egypt	N	2018/01/15	Influenza A virus (A/chicken/Egypt/A15037/2018)	c
OL354982	1777	Avian	4 (HA)	H5N8	Egypt	N	2021/01/26	Influenza A virus (A/Duck/Egypt/A19643/2021)	c
OL366043	1744	Avian	4 (HA)	H5N8	Egypt	N	2021/02/10	Influenza A virus (A/chicken/Egypt/A19671/2021)	c
OL467327	1704	Avian	4 (HA)	H5N8	Egypt	N	2018	Influenza A virus (A/Duck/Egypt/BA3C/2018)	p
OL467340	1748	Avian	4 (HA)	H5N8	Egypt	N	2018	Influenza A virus (A/goose/Egypt/BA71C/2018)	c
OL519551	1704	Human	4 (HA)	H5N6	China	N	2021/09/19	Influenza A virus (A/Guangdong/lgf/2021)	c
OL638145	1742	Avian	4 (HA)	H5N1	Czech Republ	N	2021/09/27	Influenza A virus (A/goose/Czech Republic/18520-2/2021)	c
OM060672	1751	Avian	4 (HA)	H5N8	South Korea	N	2014/11/15	Influenza A virus (A/Duck/South Korea/D14-19/2014)	c
OM333435	1704	Avian	4 (HA)	H5N8	Egypt	N	2017/02/14	Influenza A virus (A/chicken/Egypt/N13731C/2017)	c
OM373230	1704	Avian	4 (HA)	H5N8	China	N	2020/12/31	Influenza A virus (A/eurasian coot/Shandong/W5611/2020)	c
OM373238	1704	Avian	4 (HA)	H5N8	China	N	2020/12/31	Influenza A virus (A/eurasian coot/Shandong/W6137/2020)	c
OM373246	1704	Avian	4 (HA)	H5N8	China	N	2020/12/31	Influenza A virus (A/eurasian coot/Shandong/W6143/2020)	c
OM373254	1704	Avian	4 (HA)	H5N8	China	N	2020/12/31	Influenza A virus (A/eurasian coot/Shandong/W6150/2020)	c
OM373310	1704	Avian	4 (HA)	H5N8	China	N	2020/11/13	Influenza A virus (A/wild duck/Shandong/W3580/2020)	c
OM403993	1695	Reassortant	4 (HA)	H5N8			1934	Influenza A virus (A/reassortant/IDCDC-RG71A(Astrakhan/3212/2020 X Puerto Rico/	c
ON024679	1588	Avian	4 (HA)	H5N8	Egypt	N	2020/01	Influenza A virus (A/chicken/Egypt/AF12/2020)	p
ON024680	1588	Avian	4 (HA)	H5N8	Egypt	N	2020/02	Influenza A virus (A/chicken/Egypt/AF14/2020)	p
ON024681	1588	Avian	4 (HA)	H5N8	Egypt	N	2020/02	Influenza A virus (A/duck/Egypt/AI2/2020)	p
ON024682	1588	Avian	4 (HA)	H5N8	Egypt	N	2019/12	Influenza A virus (A/chicken/Egypt/AI9/2019)	p

ON024683	1588	Avian	4 (HA)	H5N8	Egypt	N	2020/01	Influenza A virus (A/duck/Egypt/FAOS1/2020)	p
ON024684	1588	Avian	4 (HA)	H5N8	Egypt	N	2020/02	Influenza A virus (A/duck/Egypt/FAOS13/2020)	p
ON024685	1588	Avian	4 (HA)	H5N8	Egypt	N	2020/03	Influenza A virus (A/chicken/Egypt/FAOS18/2020)	p
ON024686	1588	Avian	4 (HA)	H5N8	Egypt	N	2020/03	Influenza A virus (A/chicken/Egypt/FAOS20/2020)	p
ON024687	1588	Avian	4 (HA)	H5N8	Egypt	N	2020/03	Influenza A virus (A/turkey/Egypt/FAOS24/2020)	p
ON024688	1588	Avian	4 (HA)	H5N8	Egypt	N	2020/01	Influenza A virus (A/turkey/Egypt/FAOS5/2020)	p
ON024689	1588	Avian	4 (HA)	H5N8	Egypt	N	2020/01	Influenza A virus (A/duck/Egypt/FAOSG3/2020)	p
ON024690	1588	Avian	4 (HA)	H5N8	Egypt	N	2020/01	Influenza A virus (A/turkey/Egypt/FAOSG5/2020)	p
ON024691	1588	Avian	4 (HA)	H5N8	Egypt	N	2020/11	Influenza A virus (A/turkey/Egypt/FAOSG6/2020)	p
ON024692	1588	Avian	4 (HA)	H5N8	Egypt	N	2020/02	Influenza A virus (A/chicken/Egypt/FAOSG11/2020)	p
ON024693	1588	Avian	4 (HA)	H5N8	Egypt	N	2020/02	Influenza A virus (A/duck/Egypt/FAOSG14/2020)	p
ON024694	1588	Avian	4 (HA)	H5N8	Egypt	N	2020/01	Influenza A virus (A/chicken/Egypt/VG37/2020)	p
ON024695	1588	Avian	4 (HA)	H5N8	Egypt	N	2019/10	Influenza A virus (A/chicken/Egypt/AL7/2019)	p
ON024696	1588	Avian	4 (HA)	H5N8	Egypt	N	2019/02	Influenza A virus (A/chicken/Egypt/AL2/2019)	p
ON024697	1588	Avian	4 (HA)	H5N8	Egypt	N	2019/10	Influenza A virus (A/chicken/Egypt/AL6/2019)	p
ON024698	1588	Avian	4 (HA)	H5N8	Egypt	N	2019/12	Influenza A virus (A/chicken/Egypt/F565/2019)	p
ON024699	1588	Avian	4 (HA)	H5N8	Egypt	N	2019/06	Influenza A virus (A/duck/Egypt/FAO-S6/2019)	p
ON024700	1588	Avian	4 (HA)	H5N8	Egypt	N	2019/05	Influenza A virus (A/turkey/Egypt/FAO-S10/2019)	p
ON024701	1588	Avian	4 (HA)	H5N8	Egypt	N	2019/06	Influenza A virus (A/duck/Egypt/FAO-S17/2019)	p
ON024702	1588	Avian	4 (HA)	H5N8	Egypt	N	2019/06	Influenza A virus (A/turkey/Egypt/FAO-S18/2019)	p
ON024703	1588	Avian	4 (HA)	H5N8	Egypt	N	2019/08	Influenza A virus (A/duck/Egypt/FAO-S24/2019)	p
ON024704	1588	Avian	4 (HA)	H5N8	Egypt	N	2019/05	Influenza A virus (A/duck/Egypt/FAO-S32/2019)	p
ON024705	1588	Avian	4 (HA)	H5N8	Egypt	N	2019/05	Influenza A virus (A/duck/Egypt/FAO-S33/2019)	p
ON024706	1588	Avian	4 (HA)	H5N8	Egypt	N	2019/07	Influenza A virus (A/duck/Egypt/FAO-S37/2019)	p
ON024707	1588	Avian	4 (HA)	H5N8	Egypt	N	2019/09	Influenza A virus (A/turkey/Egypt/FAO-S42/2019)	p
ON024708	1588	Avian	4 (HA)	H5N8	Egypt	N	2019/11	Influenza A virus (A/duck/Egypt/FAO-S76/2019)	p
ON024709	1588	Avian	4 (HA)	H5N8	Egypt	N	2019/09	Influenza A virus (A/chicken/Egypt/FAO-SG21/2019)	p
ON024710	1588	Avian	4 (HA)	H5N8	Egypt	N	2019/07	Influenza A virus (A/chicken/Egypt/FAO-SG10/2019)	p
ON024711	1588	Avian	4 (HA)	H5N8	Egypt	N	2019/07	Influenza A virus (A/duck/Egypt/FAOSG11/2019)	p
ON024712	1588	Avian	4 (HA)	H5N8	Egypt	N	2019/07	Influenza A virus (A/turkey/Egypt/FAOSG22/2019)	p
ON024713	1588	Avian	4 (HA)	H5N8	Egypt	N	2019/03	Influenza A virus (A/duck/Egypt/SD36/2019)	p
ON024714	1588	Avian	4 (HA)	H5N8	Egypt	N	2019/02	Influenza A virus (A/duck/Egypt/SM6G/2019)	p
ON024715	1588	Avian	4 (HA)	H5N8	Egypt	N	2019/02	Influenza A virus (A/duck/Egypt/SMG7/2019)	p
ON024716	1588	Avian	4 (HA)	H5N8	Egypt	N	2019/01	Influenza A virus (A/chicken/Egypt/V1345/2019)	p
ON024717	1588	Avian	4 (HA)	H5N8	Egypt	N	2021/04	Influenza A virus (A/turkey/Egypt/A2/2021)	p
ON024718	1588	Avian	4 (HA)	H5N8	Egypt	N	2021/04	Influenza A virus (A/chicken/Egypt/A4/2021)	p
ON024719	1588	Avian	4 (HA)	H5N8	Egypt	N	2021/02	Influenza A virus (A/duck/Egypt/F91/2021)	p
ON024720	1588	Avian	4 (HA)	H5N8	Egypt	N	2021/03	Influenza A virus (A/chicken/Egypt/F304/2021)	p
ON024721	1588	Avian	4 (HA)	H5N8	Egypt	N	2021/04	Influenza A virus (A/turkey/Egypt/FAOS18/2021)	p
ON024723	1588	Avian	4 (HA)	H5N8	Egypt	N	2021/01	Influenza A virus (A/turkey/Egypt/FAOSL3/2021)	p
ON024724	1588	Avian	4 (HA)	H5N8	Egypt	N	2021/01	Influenza A virus (A/duck/Egypt/FAOSL4/2021)	p
ON024725	1588	Avian	4 (HA)	H5N8	Egypt	N	2021/04	Influenza A virus (A/duck/Egypt/FAOSI11/2021)	p
ON260842	1692	Reassortant	4 (HA)	H5N6			1934	Influenza A virus (A/reassortant/IDCDC-RG65A(Guangdong/18SF020/2018 X Puerto I	c
ON716427	1704	Avian	4 (HA)	H5N8	Cambodia	T	2021/09/16	Influenza A virus (A/Duck/Cambodia/f1PPOreu241D3_C/2021)	c
ON716428	1704	Avian	4 (HA)	H5N8	Cambodia	T	2021/09/16	Influenza A virus (A/Duck/Cambodia/f1PPChba241D6/2021)	c
ON716429	1704	Avian	4 (HA)	H5N8	Cambodia	T	2021/10/15	Influenza A virus (A/Duck/Cambodia/f4K241D3_C/2021)	c
ON716430	1704	Avian	4 (HA)	H5N8	Cambodia	T	2021/10/15	Influenza A virus (A/Duck/Cambodia/f4K241D4/2021)	c
ON716431	1704	Avian	4 (HA)	H5N8	Cambodia	T	2021/11/16	Influenza A virus (A/Duck/Cambodia/f6T241D4/2021)	c
ON716432	1701	Avian	4 (HA)	H5N6	Cambodia	T	2019/01/29	Influenza A virus (A/chicken/Cambodia/c9T241C17T/2019)	c
ON716433	1701	Avian	4 (HA)	H5N6	Cambodia	T	2018/12/13	Influenza A virus (A/Duck/Cambodia/c18MKAP189/2018)	c
ON716434	1701	Avian	4 (HA)	H5N6	Cambodia	T	2018/12/13	Influenza A virus (A/Duck/Cambodia/c18MKAP211/2018)	c
ON716435	1701	Avian	4 (HA)	H5N6	Cambodia	T	2018/12/13	Influenza A virus (A/Duck/Cambodia/c18MKAP214/2018)	c
ON716436	1701	Avian	4 (HA)	H5N6	Cambodia	T	2020/09/12	Influenza A virus (A/Duck/Cambodia/e10T241C18/2020)	c
ON716437	1704	Avian	4 (HA)	H5N6	Cambodia	T	2020/06/13	Influenza A virus (A/Duck/Cambodia/e5PPOreu241D3/2020)	c
ON716438	1704	Avian	4 (HA)	H5N6	Cambodia	T	2020/06/13	Influenza A virus (A/Duck/Cambodia/e5PPOreu241D4/2020)	c
ON716439	1704	Avian	4 (HA)	H5N6	Cambodia	T	2020/06/13	Influenza A virus (A/Duck/Cambodia/e5PPOreu241D8/2020)	c
ON716440	1704	Avian	4 (HA)	H5N6	Cambodia	T	2020/08/21	Influenza A virus (A/Duck/Cambodia/e8T241D10/2020)	c
ON716441	1704	Avian	4 (HA)	H5N6	Cambodia	T	2020/08/21	Influenza A virus (A/Duck/Cambodia/e8T241D11/2020)	c
ON716442	1704	Avian	4 (HA)	H5N6	Cambodia	T	2020/08/21	Influenza A virus (A/Duck/Cambodia/e8T241D15/2020)	c
ON716443	1704	Avian	4 (HA)	H5N6	Cambodia	T	2020/08/21	Influenza A virus (A/Duck/Cambodia/e8T241D16/2020)	c
ON716444	1704	Avian	4 (HA)	H5N6	Cambodia	T	2020/08/21	Influenza A virus (A/Duck/Cambodia/e8T241D5/2020)	c
ON716445	1704	Avian	4 (HA)	H5N6	Cambodia	T	2020/08/21	Influenza A virus (A/Duck/Cambodia/e8T241D6/2020)	c
ON716446	1704	Avian	4 (HA)	H5N6	Cambodia	T	2020/08/21	Influenza A virus (A/Duck/Cambodia/e8T241D9/2020)	c
OP209769	1704	Human	4 (HA)	H5N6	China	N	2022/02/26	Influenza A virus (A/Yangzhou/125/2022)	c
OP221343	1751	Avian	4 (HA)	H5N1	USA	N	2022/03/10	Influenza A virus (A/American pelican/Kansas/W22-200/2022)	c
OP377338	1751	Avian	4 (HA)	H5N1	USA	N	2022/06/30	Influenza A virus (A/black vulture/Virginia/W22-499C/2022)	c
OP377388	1751	Avian	4 (HA)	H5N1	USA	N	2022/06/30	Influenza A virus (A/black vulture/Virginia/W22-499A/2022)	c
OP377412	1751	Avian	4 (HA)	H5N1	USA	N	2022/03/17	Influenza A virus (A/royal tern/Florida/W22-245B/2022)	c
OP377453	1751	Avian	4 (HA)	H5N1	USA	N	2022/03/07	Influenza A virus (A/black vulture/Florida/W22-168/2022)	c
OP377486	1751	Avian	4 (HA)	H5N1	USA	N	2022/02/25	Influenza A virus (A/black vulture/Florida/W22-161/2022)	c
OP377494	1751	Avian	4 (HA)	H5N1	USA	N	2022/06/30	Influenza A virus (A/black vulture/Virginia/W22-499B/2022)	c
OP377510	1751	Avian	4 (HA)	H5N1	USA	N	2022/02/24	Influenza A virus (A/great horned owl/Flordia/W22-163A/2022)	c
OP377542	1751	Avian	4 (HA)	H5N1	USA	N	2022/02/27	Influenza A virus (A/great blue heron/Florida/W22-160/2022)	c
OP377558	1751	Avian	4 (HA)	H5N1	USA	N	2022/05/10	Influenza A virus (A/black vulture/North Carolina/W22-367C/2022)	c
OP377574	1751	Avian	4 (HA)	H5N1	USA	N	2022/03/04	Influenza A virus (A/black vulture/Florida/W22-167/2022)	c
OP377606	1751	Avian	4 (HA)	H5N1	USA	N	2022/02/20	Influenza A virus (A/brown pelican/North Carolina/W22-164/2022)	c
OP377614	1751	Avian	4 (HA)	H5N1	USA	N	2022/03/12	Influenza A virus (A/royal tern/Florida/W22-245A/2022)	c
OP377638	1751	Avian	4 (HA)	H5N1	USA	N	2022/02/23	Influenza A virus (A/great horned owl/Flordia/W22-163C/2022)	c
OP413016	1777	Avian	4 (HA)	H5N8	Egypt	N	2021/03/03	Influenza A virus (A/chicken/Egypt/Menoufia/2021)	c
OP413044	1701	Avian	4 (HA)	H5N1	Egypt	N	2021/10/07	Influenza A virus (A/migratory wild bird/Egypt/25/2021)	nc
OP413045	1701	Avian	4 (HA)	H5N1	Egypt	N	2021/11/22	Influenza A virus (A/migratory wild bird/Egypt/26/2021)	nc
OP413046	1701	Avian	4 (HA)	H5N1	Egypt	N	2022/03/25	Influenza A virus (A/migratory wild bird/Egypt/30/2022)	nc
OP413047	1701	Avian	4 (HA)	H5N1	Egypt	N	2022/03/25	Influenza A virus (A/migratory wild bird/Egypt/31/2022)	nc

OP413048	1701	Avian	4 (HA)	H5N1	Egypt	N	2022/03/25	Influenza A virus (A/migratory wild bird/Egypt/32/2022)	nc
OP470716	1751	Avian	4 (HA)	H5N1	USA	N	2022/02/12	Influenza A virus (A/lesser scaup/Georgia/W22-145C/2022)	c
OP470733	1751	Avian	4 (HA)	H5N1	USA	N	2022/02/12	Influenza A virus (A/bald eagle/Florida/W22-134-CL/2022)	c
OP470749	1751	Avian	4 (HA)	H5N1	USA	N	2022/02/12	Influenza A virus (A/lesser scaup/Georgia/W22-145B/2022)	c
OP470757	1751	Avian	4 (HA)	H5N1	USA	N	2022/02/12	Influenza A virus (A/lesser scaup/Georgia/W22-145D/2022)	c
OP470765	1751	Avian	4 (HA)	H5N1	USA	N	2022/02/12	Influenza A virus (A/lesser scaup/Georgia/W22-143/2022)	c
OP470773	1751	Avian	4 (HA)	H5N1	USA	N	2022/02/12	Influenza A virus (A/lesser scaup/Georgia/W22-145A/2022)	c
OP470781	1751	Avian	4 (HA)	H5N1	USA	N	2022/02/12	Influenza A virus (A/lesser scaup/Georgia/W22-145E/2022)	c
OP470789	1751	Avian	4 (HA)	H5N1	USA	N	2022/02/08	Influenza A virus (A/snow goose/Kentucky/W22-092/2022)	c
OP470797	1751	Avian	4 (HA)	H5N1	USA	N	2022/02/12	Influenza A virus (A/bald eagle/Florida/W22-142/2022)	c
OP470804	1751	Avian	4 (HA)	H5N1	USA	N	2022/02/17	Influenza A virus (A/bald eagle/FL/W22-114/2022)	c
OP470812	1751	Avian	4 (HA)	H5N1	USA	N	2022/02/12	Influenza A virus (A/lesser scaup/Florida/W22-129A/2022)	c
OP484862	1777	Avian	4 (HA)	H5N5	Egypt	N	2016/12/21	Influenza A virus (A/purple heron/Egypt/MB 933C/2016)	c
OP597554	1735	Avian	4 (HA)	H5N5	Russia	N	2021/04/06	Influenza A virus (A/dalmatian pelican/Astrakhan/417-1/2021)	c
OP597562	1735	Avian	4 (HA)	H5N5	Russia	N	2021/04/06	Influenza A virus (A/dalmatian pelican/Astrakhan/417-2/2021)	c
OP597570	1735	Avian	4 (HA)	H5N5	Russia	N	2021/04/01	Influenza A virus (A/pelican/Dagestan/397-1/2021)	c
OP597578	1735	Avian	4 (HA)	H5N5	Russia	N	2021/04/01	Influenza A virus (A/gull/Dagestan/397-2/2021)	c
OP597586	1735	Avian	4 (HA)	H5N5	Russia	N	2020/12/29	Influenza A virus (A/swan/Rostov/2299-2/2020)	c
OP597594	1735	Avian	4 (HA)	H5N5	Russia	N	2021/11/08	Influenza A virus (A/shelduck/Kalmykia/1814-1/2021)	c
OP597602	1735	Avian	4 (HA)	H5N5	Russia	N	2021/09/28	Influenza A virus (A/waterfowl/Russia/1526-4/2021)	c
OP748410	1704	Avian	4 (HA)	H5N8	China	N	2020/12	Influenza A virus (A/chicken/China/JM01/2020)	c
OQ195215	1701	Environment	4 (HA)	H5	China	N	2020/06/22	Influenza A virus (A/Environment/China/H5N6/2020)	c
OQ195223	1701	Avian	4 (HA)	H5	China	N	2020/08/19	Influenza A virus (A/duck/China/H5N6/2020)	c
OQ195231	1701	Avian	4 (HA)	H5	China	N	2020/06/22	Influenza A virus (A/Duck/China/H5N6/2020)	c

GISAID

Isolate_Id	Isolate_Name	Subtype	Location	Isolate_Submitter
EPI_ISL_1005680	A/common teal/Chany Lake/213/2020	A / H5N2	Europe / Russian Federation / Novosibirsk	Takehiko Saito (National Institute of Animal Health)
EPI_ISL_1009679	A/duck/Korea/H007/2020	A / H5N8	Asia / Korea, Republic of / CN	Yu-Na Lee (Animal and Plant Quarantine Agency (S-2158) / Avian Influenza Research & Diagnostic Division)
EPI_ISL_1009680	A/White peacock/Korea/H533/2020	A / H5N8	Asia / Korea, Republic of / GG	Yu-Na Lee (Animal and Plant Quarantine Agency (S-2158) / Avian Influenza Research & Diagnostic Division)
EPI_ISL_1009681	A/chicken/Korea/H541/2020	A / H5N8	Asia / Korea, Republic of / CN	Yu-Na Lee (Animal and Plant Quarantine Agency (S-2158) / Avian Influenza Research & Diagnostic Division)
EPI_ISL_1009682	A/chicken/Korea/H544/2020	A / H5N8	Asia / Korea, Republic of / GG	Yu-Na Lee (Animal and Plant Quarantine Agency (S-2158) / Avian Influenza Research & Diagnostic Division)
EPI_ISL_1009683	A/duck/Korea/H542/2020	A / H5N8	Asia / Korea, Republic of / JN	Yu-Na Lee (Animal and Plant Quarantine Agency (S-2158) / Avian Influenza Research & Diagnostic Division)
EPI_ISL_1009684	A/duck/Korea/H548/2020	A / H5N8	Asia / Korea, Republic of / JB	Yu-Na Lee (Animal and Plant Quarantine Agency (S-2158) / Avian Influenza Research & Diagnostic Division)
EPI_ISL_1009685	A/duck/Korea/H549/2020	A / H5N8	Asia / Korea, Republic of / JB	Yu-Na Lee (Animal and Plant Quarantine Agency (S-2158) / Avian Influenza Research & Diagnostic Division)
EPI_ISL_1009686	A/chicken/Korea/H550/2020	A / H5N8	Asia / Korea, Republic of / GG	Yu-Na Lee (Animal and Plant Quarantine Agency (S-2158) / Avian Influenza Research & Diagnostic Division)
EPI_ISL_1009687	A/quail/Korea/H551/2020	A / H5N8	Asia / Korea, Republic of / GB	Yu-Na Lee (Animal and Plant Quarantine Agency (S-2158) / Avian Influenza Research & Diagnostic Division)
EPI_ISL_1009688	A/chicken/Korea/H001/2021	A / H5N8	Asia / Korea, Republic of / JN	Yu-Na Lee (Animal and Plant Quarantine Agency (S-2158) / Avian Influenza Research & Diagnostic Division)
EPI_ISL_1009689	A/chicken/Korea/H002/2021	A / H5N8	Asia / Korea, Republic of / CN	Yu-Na Lee (Animal and Plant Quarantine Agency (S-2158) / Avian Influenza Research & Diagnostic Division)
EPI_ISL_1009690	A/chicken/Korea/H008/2021	A / H5N8	Asia / Korea, Republic of / CN	Yu-Na Lee (Animal and Plant Quarantine Agency (S-2158) / Avian Influenza Research & Diagnostic Division)
EPI_ISL_1009691	A/duck/Korea/H009/2021	A / H5N8	Asia / Korea, Republic of / JB	Yu-Na Lee (Animal and Plant Quarantine Agency (S-2158) / Avian Influenza Research & Diagnostic Division)
EPI_ISL_1009692	A/duck/Korea/H010/2021	A / H5N8	Asia / Korea, Republic of / CB	Yu-Na Lee (Animal and Plant Quarantine Agency (S-2158) / Avian Influenza Research & Diagnostic Division)
EPI_ISL_1009693	A/chicken/Korea/H022/2021	A / H5N8	Asia / Korea, Republic of / GG	Yu-Na Lee (Animal and Plant Quarantine Agency (S-2158) / Avian Influenza Research & Diagnostic Division)
EPI_ISL_1009694	A/duck/Korea/H025/2021	A / H5N8	Asia / Korea, Republic of / GN	Yu-Na Lee (Animal and Plant Quarantine Agency (S-2158) / Avian Influenza Research & Diagnostic Division)
EPI_ISL_1009695	A/spot-billed duck/Korea/WA612/2020	A / H5N8	Asia / Korea, Republic of / CN	Yu-Na Lee (Animal and Plant Quarantine Agency (S-2158) / Avian Influenza Research & Diagnostic Division)
EPI_ISL_1009696	A/mandarin duck/Korea/WB80/2020	A / H5N8	Asia / Korea, Republic of / GG	Yu-Na Lee (Animal and Plant Quarantine Agency (S-2158) / Avian Influenza Research & Diagnostic Division)
EPI_ISL_1009697	A/wild bird/Korea/H357/2020	A / H5N8	Asia / Korea, Republic of / JB	Yu-Na Lee (Animal and Plant Quarantine Agency (S-2158) / Avian Influenza Research & Diagnostic Division)
EPI_ISL_1009698	A/mallard/Korea/WA820/2020	A / H5N8	Asia / Korea, Republic of / GG	Yu-Na Lee (Animal and Plant Quarantine Agency (S-2158) / Avian Influenza Research & Diagnostic Division)
EPI_ISL_1009699	A/mandarin duck/Korea/WA831/2020	A / H5N8	Asia / Korea, Republic of / JB	Yu-Na Lee (Animal and Plant Quarantine Agency (S-2158) / Avian Influenza Research & Diagnostic Division)
EPI_ISL_1009700	A/spot-billed duck/Korea/WA854/2020	A / H5N8	Asia / Korea, Republic of / GG	Yu-Na Lee (Animal and Plant Quarantine Agency (S-2158) / Avian Influenza Research & Diagnostic Division)
EPI_ISL_1009701	A/mandarin duck/Korea/WA857/2020	A / H5N8	Asia / Korea, Republic of / JB	Yu-Na Lee (Animal and Plant Quarantine Agency (S-2158) / Avian Influenza Research & Diagnostic Division)
EPI_ISL_1009702	A/mandarin duck/Korea/WA877/2020	A / H5N8	Asia / Korea, Republic of / JN	Yu-Na Lee (Animal and Plant Quarantine Agency (S-2158) / Avian Influenza Research & Diagnostic Division)
EPI_ISL_1009703	A/spot-billed duck/Korea/WA889/2020	A / H5N8	Asia / Korea, Republic of / GG	Yu-Na Lee (Animal and Plant Quarantine Agency (S-2158) / Avian Influenza Research & Diagnostic Division)
EPI_ISL_1009704	A/mandarin duck/Korea/WA899/2020	A / H5N8	Asia / Korea, Republic of / CN	Yu-Na Lee (Animal and Plant Quarantine Agency (S-2158) / Avian Influenza Research & Diagnostic Division)
EPI_ISL_1009705	A/mandarin duck/Korea/WA913/2020	A / H5N8	Asia / Korea, Republic of / CN	Yu-Na Lee (Animal and Plant Quarantine Agency (S-2158) / Avian Influenza Research & Diagnostic Division)
EPI_ISL_1009706	A/wild bird/Korea/H379/2020	A / H5N8	Asia / Korea, Republic of / GB	Yu-Na Lee (Animal and Plant Quarantine Agency (S-2158) / Avian Influenza Research & Diagnostic Division)
EPI_ISL_1009707	A/wild bird/Korea/H467/2020	A / H5N8	Asia / Korea, Republic of / CB	Yu-Na Lee (Animal and Plant Quarantine Agency (S-2158) / Avian Influenza Research & Diagnostic Division)
EPI_ISL_1009708	A/spot-billed duck/Korea/WA1000/2020	A / H5N8	Asia / Korea, Republic of / GG	Yu-Na Lee (Animal and Plant Quarantine Agency (S-2158) / Avian Influenza Research & Diagnostic Division)
EPI_ISL_1009709	A/wild bird/Korea/H496-3/2020	A / H5N8	Asia / Korea, Republic of / JJ	Yu-Na Lee (Animal and Plant Quarantine Agency (S-2158) / Avian Influenza Research & Diagnostic Division)
EPI_ISL_1009710	A/wild duck/Korea/H331/2020	A / H5N8	Asia / Korea, Republic of / JJ	Yu-Na Lee (Animal and Plant Quarantine Agency (S-2158) / Avian Influenza Research & Diagnostic Division)
EPI_ISL_1009711	A/duck/Korea/H016/2021	A / H5N8	Asia / Korea, Republic of / JN	Yu-Na Lee (Animal and Plant Quarantine Agency (S-2158) / Avian Influenza Research & Diagnostic Division)
EPI_ISL_10220427	A/red fox/Netherlands/22002636-005/2022	A / H5N1	Europe / Netherlands / Provincie Gelderland	Rene Heutink (Wageningen Bioveterinary Research)
EPI_ISL_10220428	A/red fox/Netherlands/22002722-004/2022	A / H5N1	Europe / Netherlands / Provincie Gelderland	Rene Heutink (Wageningen Bioveterinary Research)
EPI_ISL_10220429	A/polecat/Netherlands/22002792-005/2022	A / H5N1	Europe / Netherlands / Provincie Gelderland	Rene Heutink (Wageningen Bioveterinary Research)
EPI_ISL_10220432	A/badger/Netherlands/22003078-004/2022	A / H5N1	Europe / Netherlands / Provincie Gelderland	Rene Heutink (Wageningen Bioveterinary Research)
EPI_ISL_10255580	A/wild bird/Netherlands/22002833-002/2022	A / H5N1	Europe / Netherlands / Provincie Gelderland	Rene Heutink (Wageningen Bioveterinary Research)
EPI_ISL_10255581	A/red fox/Netherlands/22002992-003/2022	A / H5N1	Europe / Netherlands / Provincie Gelderland	Rene Heutink (Wageningen Bioveterinary Research)
EPI_ISL_10255587	A/red fox/Netherlands/22002993-003 /2022	A / H5N1	Europe / Netherlands / Provincie Noord-Holland	Rene Heutink (Wageningen Bioveterinary Research)
EPI_ISL_10261376	A/pigeon/Germany-NW/AI00951/2022	A / H5N1	Europe / Germany / North Rhine-Westphalia / Regierungsbezirk Arnsberg / Dortmund	Jacqueline King (Friedrich-Loeffler-Institut)
EPI_ISL_1033028	A/chicken/Czech Republic/2395/2021	A / H5N8	Europe / Czech Republic / Stredocesky Kraj / Okres Benesov / Jankov	Alexander Nagy (State Veterinary Institute Prague)
EPI_ISL_1033029	A/chicken/Czech Republic/2502-1/2021	A / H5N8	Europe / Czech Republic / Pardubicky kraj / Okres Chrudim / Ronov nad Doubravou; GPS 49°53'2	Alexander Nagy (State Veterinary Institute Prague)
EPI_ISL_1033030	A/chicken/Czech Republic/2502-2/2021	A / H5N8	Europe / Czech Republic / Pardubicky kraj / Okres Chrudim / Ronov nad Doubravou; GPS 49°53'2	Alexander Nagy (State Veterinary Institute Prague)
EPI_ISL_1033124	A/mute swan/Czech Republic/2600/2021	A / H5N8	Europe / Czech Republic / Jihocesky Kraj / Okres Strakonice / Strakonice	Alexander Nagy (State Veterinary Institute Prague)
EPI_ISL_10347218	A/Barnacle Goose/Netherlands/6/2021	A / H5N1	Europe / Netherlands / Provincie Friesland / Kollumerland	Pascal Lexmond (Erasmus Medical Center / Viroscience)
EPI_ISL_10347219	A/Barnacle Goose/Netherlands/8/2022	A / H5N1	Europe / Netherlands / Provincie Friesland / Gemeente Terschelling / Oosterend	Pascal Lexmond (Erasmus Medical Center / Viroscience)
EPI_ISL_10347220	A/Barnacle Goose/Netherlands/7/2021	A / H5N1	Europe / Netherlands / Provincie Friesland / Kollumerland	Pascal Lexmond (Erasmus Medical Center / Viroscience)
EPI_ISL_10347326	A/long-eared owl/Germany-NI/AI09037/2021	A / H5N1	Europe / Germany / Lower Saxony / Friesland	Jacqueline King (Friedrich-Loeffler-Institut)
EPI_ISL_10364607	A/mute swan/Czech Republic/4099/2021	A / H5N5	Europe / Czech Republic / Zlinsky Kraj / Okres Kromeriz / 49°14'23.619'', 17°28'34.391''E	Alexander Nagy (State Veterinary Institute Prague)
EPI_ISL_1038924	A/Astrakhan/3212/2020	A / H5N8	Europe / Russian Federation / Astrakhan Oblast	Ivan Susloparov (State Research Center of Virology and Biotechnology (VECTOR) / Emerging Zoonotic Diseases and Influenza)
EPI_ISL_1039231	A/chicken/Astrakhan/321-01/2020	A / H5N8	Europe / Russian Federation / Astrakhan Oblast	Natalia Goncharova (State Research Center of Virology and Biotechnology (VECTOR) / Emerging Zoonotic Diseases and Influenza)
EPI_ISL_1039234	A/chicken/Astrakhan/321-05/2020	A / H5N8	Europe / Russian Federation / Astrakhan Oblast	Natalia Goncharova (State Research Center of Virology and Biotechnology (VECTOR) / Emerging Zoonotic Diseases and Influenza)
EPI_ISL_1039236	A/chicken/Astrakhan/321-06/2020	A / H5N8	Europe / Russian Federation / Astrakhan Oblast	Natalia Goncharova (State Research Center of Virology and Biotechnology (VECTOR) / Emerging Zoonotic Diseases and Influenza)
EPI_ISL_1039238	A/chicken/Astrakhan/321-09/2020	A / H5N8	Europe / Russian Federation / Astrakhan Oblast	Natalia Goncharova (State Research Center of Virology and Biotechnology (VECTOR) / Emerging Zoonotic Diseases and Influenza)
EPI_ISL_1039239	A/chicken/Astrakhan/321-10/2020	A / H5N8	Europe / Russian Federation / Astrakhan Oblast	Natalia Goncharova (State Research Center of Virology and Biotechnology (VECTOR) / Emerging Zoonotic Diseases and Influenza)
EPI_ISL_10406912	rg A/chicken/Egypt/Q16684C/2019	A / H5N8	Africa / Egypt / Qalyubia	Yassmin Moatasim (National Research Center (Hospital) / Water Pollution)
EPI_ISL_1041108	A/duck/Chiba/D2A-8C/2021	A / H5N8	Asia / Japan / Chiba	Takehiko Saito (National Institute of Animal Health)
EPI_ISL_1041120	A/duck/Chiba/D2B-7C/2021	A / H5N8	Asia / Japan / Chiba	Takehiko Saito (National Institute of Animal Health)
EPI_ISL_1041121	A/duck/Chiba/D2B-9C/2021	A / H5N8	Asia / Japan / Chiba	Takehiko Saito (National Institute of Animal Health)
EPI_ISL_1041124	A/chicken/Chiba/I1C/2021	A / H5N8	Asia / Japan / Chiba	Takehiko Saito (National Institute of Animal Health)
EPI_ISL_1041125	A/chicken/Chiba/I1T/2021	A / H5N8	Asia / Japan / Chiba	Takehiko Saito (National Institute of Animal Health)
EPI_ISL_1041126	A/chicken/Chiba/I2T/2021	A / H5N8	Asia / Japan / Chiba	Takehiko Saito (National Institute of Animal Health)
EPI_ISL_1041127	A/chicken/Chiba/I3T/2021	A / H5N8	Asia / Japan / Chiba	Takehiko Saito (National Institute of Animal Health)
EPI_ISL_1041128	A/chicken/Chiba/J1C/2021	A / H5N8	Asia / Japan / Chiba	Takehiko Saito (National Institute of Animal Health)
EPI_ISL_1041129	A/chicken/Chiba/J1T/2021	A / H5N8	Asia / Japan / Chiba	Takehiko Saito (National Institute of Animal Health)

EPI_ISL_1041130	A/chicken/Chiba/J6C/2021	A / H5N8	Asia / Japan / Chiba	Takehiko Saito (National Institute of Animal Health)
EPI_ISL_1041131	A/chicken/Chiba/J6T/2021	A / H5N8	Asia / Japan / Chiba	Takehiko Saito (National Institute of Animal Health)
EPI_ISL_1041132	A/chicken/Tokushima/B1T/2020	A / H5N8	Asia / Japan / Tokushima	Takehiko Saito (National Institute of Animal Health)
EPI_ISL_1041133	A/chicken/Tokushima/B2T/2020	A / H5N8	Asia / Japan / Tokushima	Takehiko Saito (National Institute of Animal Health)
EPI_ISL_1041134	A/chicken/Tokushima/B3T/2020	A / H5N8	Asia / Japan / Tokushima	Takehiko Saito (National Institute of Animal Health)
EPI_ISL_1041135	A/chicken/Tokushima/B4T/2020	A / H5N8	Asia / Japan / Tokushima	Takehiko Saito (National Institute of Animal Health)
EPI_ISL_1041136	A/duck/Chiba/D1A-10T/2021	A / H5N8	Asia / Japan / Chiba	Takehiko Saito (National Institute of Animal Health)
EPI_ISL_1041137	A/duck/Chiba/D1A-1T/2021	A / H5N8	Asia / Japan / Chiba	Takehiko Saito (National Institute of Animal Health)
EPI_ISL_1041138	A/duck/Chiba/D1A-2T/2021	A / H5N8	Asia / Japan / Chiba	Takehiko Saito (National Institute of Animal Health)
EPI_ISL_1041139	A/duck/Chiba/D1A-3T/2021	A / H5N8	Asia / Japan / Chiba	Takehiko Saito (National Institute of Animal Health)
EPI_ISL_1041140	A/duck/Chiba/D1A-4T/2021	A / H5N8	Asia / Japan / Chiba	Takehiko Saito (National Institute of Animal Health)
EPI_ISL_1041141	A/duck/Chiba/D1A-5T/2021	A / H5N8	Asia / Japan / Chiba	Takehiko Saito (National Institute of Animal Health)
EPI_ISL_1041142	A/duck/Chiba/D1A-6T/2021	A / H5N8	Asia / Japan / Chiba	Takehiko Saito (National Institute of Animal Health)
EPI_ISL_1041143	A/duck/Chiba/D1A-7T/2021	A / H5N8	Asia / Japan / Chiba	Takehiko Saito (National Institute of Animal Health)
EPI_ISL_1041144	A/duck/Chiba/D1A-8T/2021	A / H5N8	Asia / Japan / Chiba	Takehiko Saito (National Institute of Animal Health)
EPI_ISL_1041145	A/duck/Chiba/D1A-9T/2021	A / H5N8	Asia / Japan / Chiba	Takehiko Saito (National Institute of Animal Health)
EPI_ISL_1041146	A/duck/Chiba/D1B-10T/2021	A / H5N8	Asia / Japan / Chiba	Takehiko Saito (National Institute of Animal Health)
EPI_ISL_1041147	A/duck/Chiba/D1B-1T/2021	A / H5N8	Asia / Japan / Chiba	Takehiko Saito (National Institute of Animal Health)
EPI_ISL_1041148	A/duck/Chiba/D1B-2T/2021	A / H5N8	Asia / Japan / Chiba	Takehiko Saito (National Institute of Animal Health)
EPI_ISL_1041149	A/duck/Chiba/D1B-3T/2021	A / H5N8	Asia / Japan / Chiba	Takehiko Saito (National Institute of Animal Health)
EPI_ISL_1041150	A/duck/Chiba/D1B-4T/2021	A / H5N8	Asia / Japan / Chiba	Takehiko Saito (National Institute of Animal Health)
EPI_ISL_1041151	A/duck/Chiba/D1B-5T/2021	A / H5N8	Asia / Japan / Chiba	Takehiko Saito (National Institute of Animal Health)
EPI_ISL_1041152	A/duck/Chiba/D1B-6T/2021	A / H5N8	Asia / Japan / Chiba	Takehiko Saito (National Institute of Animal Health)
EPI_ISL_1041153	A/duck/Chiba/D1B-7T/2021	A / H5N8	Asia / Japan / Chiba	Takehiko Saito (National Institute of Animal Health)
EPI_ISL_1041154	A/duck/Chiba/D1B-8T/2021	A / H5N8	Asia / Japan / Chiba	Takehiko Saito (National Institute of Animal Health)
EPI_ISL_1041155	A/duck/Chiba/D1B-9T/2021	A / H5N8	Asia / Japan / Chiba	Takehiko Saito (National Institute of Animal Health)
EPI_ISL_1041158	A/chicken/Chiba/K11C/2021	A / H5N8	Asia / Japan / Chiba	Takehiko Saito (National Institute of Animal Health)
EPI_ISL_1041159	A/chicken/Chiba/K11T/2021	A / H5N8	Asia / Japan / Chiba	Takehiko Saito (National Institute of Animal Health)
EPI_ISL_1041160	A/chicken/Chiba/K5C/2021	A / H5N8	Asia / Japan / Chiba	Takehiko Saito (National Institute of Animal Health)
EPI_ISL_1041161	A/chicken/Chiba/K5T/2021	A / H5N8	Asia / Japan / Chiba	Takehiko Saito (National Institute of Animal Health)
EPI_ISL_1041165	A/duck/Chiba/D2A-10T/2021	A / H5N8	Asia / Japan / Chiba	Takehiko Saito (National Institute of Animal Health)
EPI_ISL_1041166	A/duck/Chiba/D2A-1T/2021	A / H5N8	Asia / Japan / Chiba	Takehiko Saito (National Institute of Animal Health)
EPI_ISL_1041167	A/duck/Chiba/D2A-2T/2021	A / H5N8	Asia / Japan / Chiba	Takehiko Saito (National Institute of Animal Health)
EPI_ISL_1041168	A/duck/Chiba/D2A-3T/2021	A / H5N8	Asia / Japan / Chiba	Takehiko Saito (National Institute of Animal Health)
EPI_ISL_1041169	A/duck/Chiba/D2A-5T/2021	A / H5N8	Asia / Japan / Chiba	Takehiko Saito (National Institute of Animal Health)
EPI_ISL_1041170	A/duck/Chiba/D2A-7T/2021	A / H5N8	Asia / Japan / Chiba	Takehiko Saito (National Institute of Animal Health)
EPI_ISL_1041171	A/duck/Chiba/D2A-9T/2021	A / H5N8	Asia / Japan / Chiba	Takehiko Saito (National Institute of Animal Health)
EPI_ISL_1041172	A/eastern buzzard/Toyama/160213C/2021	A / H5N8	Asia / Japan / Toyama	Takehiko Saito (National Institute of Animal Health)
EPI_ISL_1041173	A/eastern buzzard/Toyama/160213T/2021	A / H5N8	Asia / Japan / Toyama	Takehiko Saito (National Institute of Animal Health)
EPI_ISL_1041176	A/owl/Tochigi/090204C/2021	A / H5N8	Asia / Japan / Tochigi	Takehiko Saito (National Institute of Animal Health)
EPI_ISL_1041177	A/peregrine falcon/Tochigi/090205C/2021	A / H5N8	Asia / Japan / Tochigi	Takehiko Saito (National Institute of Animal Health)
EPI_ISL_1041178	A/peregrine falcon/Tochigi/090205T/2021	A / H5N8	Asia / Japan / Tochigi	Takehiko Saito (National Institute of Animal Health)
EPI_ISL_1041180	A/whooper swan/Niigata/150212T/2021	A / H5N8	Asia / Japan / Niigata	Takehiko Saito (National Institute of Animal Health)
EPI_ISL_10434072	A/swan/Germany-BW/AI00996/2022	A / H5N2	Europe / Germany / Baden-Wuerttemberg / Regierungsbezirk Karlsruhe / Karlsruhe	Jacqueline King (Friedrich-Loeffler-Institut)
EPI_ISL_10434073	A/swan/Germany-BW/AI00997/2022	A / H5N2	Europe / Germany / Baden-Wuerttemberg / Regierungsbezirk Karlsruhe / Karlsruhe	Jacqueline King (Friedrich-Loeffler-Institut)
EPI_ISL_10454874	A/barnacle goose/Netherlands/22002507-002/2022	A / H5N1	Europe / Netherlands / Provincie Gelderland	Rene Heutink (Wageningen Bioveterinary Research)
EPI_ISL_10454886	A/barnacle goose/Netherlands/22002511-001/2022	A / H5N1	Europe / Netherlands / Provincie Utrecht	Rene Heutink (Wageningen Bioveterinary Research)
EPI_ISL_10454887	A/red fox/Netherlands/22003495-003/2022	A / H5N1	Europe / Netherlands / Provincie Utrecht	Rene Heutink (Wageningen Bioveterinary Research)
EPI_ISL_1046889	A/chicken/Netherlands/21023815-001005/2021	A / H5N8	Europe / Netherlands / North Brabant / Gemeente Sint-Oedenrode	Rene Heutink (Wageningen Bioveterinary Research)
EPI_ISL_1048238	A/common kestrel/Netherlands/20020264-002/2020	A / H5N8	Europe / Netherlands / Provincie Noord-Holland / Gemeente Blaricum	Rene Heutink (Wageningen Bioveterinary Research)
EPI_ISL_1048239	A/common buzzard/Netherlands/21021023-002/2021	A / H5N3	Europe / Netherlands / Provincie Groningen / Gemeente Delfzijl	Rene Heutink (Wageningen Bioveterinary Research)
EPI_ISL_1048240	A/common buzzard/Netherlands/21021396-002/2021	A / H5N1	Europe / Netherlands / Provincie Gelderland / Gemeente Zaltbommel	Rene Heutink (Wageningen Bioveterinary Research)
EPI_ISL_1048241	A/barnacle goose/Netherlands/21021591-001/2021	A / H5N1	Europe / Netherlands / Provincie Noord-Holland / Gemeente Naarden	Rene Heutink (Wageningen Bioveterinary Research)
EPI_ISL_10497305	A/red knot/Germany-SH/AI01010/2022	A / H5N1	Europe / Germany / Schleswig-Holstein / Nordfriesland	Jacqueline King (Friedrich-Loeffler-Institut)
EPI_ISL_10497306	A/buzzard/Germany-BB/AI01212/2022	A / H5N1	Europe / Germany / Brandenburg / Ostprignitz-Ruppin	Jacqueline King (Friedrich-Loeffler-Institut)
EPI_ISL_1057106	A/mute swan/Czech Republic/2669-1/2021	A / H5N8	Europe / Czech Republic / Jihocesky Kraj / Okres Tabor / Sobeslav	Alexander Nagy (State Veterinary Institute Prague)
EPI_ISL_10576444	A/Anser_anser/Belgium/1809_0002/2022	A / H5N1	Europe / Belgium / Province de Liege / Ougrée	Steven Van Borm (Sciensano, Department of Animal Infectious Diseases / Animal Infectious Diseases)
EPI_ISL_1058019	A/mute swan/Czech Republic/2669-2/2021	A / H5N8	Europe / Czech Republic / Jihocesky Kraj / Okres Tabor / Sobeslav	Alexander Nagy (State Veterinary Institute Prague)
EPI_ISL_1058020	A/chicken/Czech Republic/29392021	A / H5N8	Europe / Czech Republic / Jihocesky Kraj / Okres Strakonice / Katovice	Alexander Nagy (State Veterinary Institute Prague)
EPI_ISL_1058021	A/chicken/Czech Republic/3099-1/2021	A / H5N8	Europe / Czech Republic / Plzensky Kraj / Okres Plzen-Mesto / Bzi	Alexander Nagy (State Veterinary Institute Prague)
EPI_ISL_1058022	A/chicken/Czech Republic/3099-2/2021	A / H5N8	Europe / Czech Republic / Plzensky Kraj / Okres Plzen-Mesto / Bzi	Alexander Nagy (State Veterinary Institute Prague)
EPI_ISL_1063533	A/Mandarin duck/Kagoshima/KU-d57/2020	A / H5N8	Asia / Japan / Kagoshima	Ahmed Magdy Ahmed Khalil (Faculty of Veterinary Medicine - Zagazig University / Zoonoses)
EPI_ISL_1063993	A/common kestrel/Denmark/16023-01/2021-01-01(H5N3)	A / H5N3	Europe / Denmark / Region Syddanmark / Kolding Kommune	Charlotte Kristiane Hjulsager (Statens Serum Institute / Microbiological Diagnostic and Virology)
EPI_ISL_10724257	A/sanderling/Netherlands/22002272-001/2021	A / H5N1	Europe / Netherlands / Provincie Friesland	Rene Heutink (Wageningen Bioveterinary Research)
EPI_ISL_10724259	A/seagull/Netherlands/22002274-002/2022	A / H5N1	Europe / Netherlands / Provincie Gelderland	Rene Heutink (Wageningen Bioveterinary Research)
EPI_ISL_1080480	A/mute swan/Czech Republic/3160-1/2021	A / H5N8	Europe / Czech Republic / Liberecky Kraj / Okres Liberec / Prisovice, pond Velky pisecak, GPS: 50	Alexander Nagy (State Veterinary Institute Prague)
EPI_ISL_1080579	A/common coot/Czech Republic/3160-2/2021	A / H5N8	Europe / Czech Republic / Liberecky Kraj / Okres Liberec / Prisovice, pond Velky pisecak, GPS: 50	Alexander Nagy (State Veterinary Institute Prague)
EPI_ISL_1081353	A/whooper swan/Tochigi/090203C/2021	A / H5N8	Asia / Japan / Tochigi	Takehiko Saito (National Institute of Animal Health)
EPI_ISL_1081369	A/Chongqing/00013/2021	A / H5N6	Asia / China / Chongqinq Municipality	Lei Yang (WHO Chinese National Influenza Center / Virology Institute, Chinese CDC)
EPI_ISL_1081370	A/Anhui/2021-00011/2020	A / H5N6	Asia / China / Anhui Province	Lei Yang (WHO Chinese National Influenza Center / Virology Institute, Chinese CDC)
EPI_ISL_10915976	A/red fox/Netherlands/22004172-003/2022	A / H5N1	Europe / Netherlands / Provincie Gelderland	Rene Heutink (Wageningen Bioveterinary Research)
EPI_ISL_1093422	A/Turkey/Sweden/SVA210214SZ0002/KN035667-IP5/2021	A / H5N8	Europe / Sweden / Skane Lan / Simrishamns Kommun	Siamak Zohari (National Veterinary Institute)

EPI_ISL_1093423	A/Chicken/Sweden/SVA210217SZ0001/KN001190-IP6/2021	A / H5N8	Europe / Sweden / Vastra Gotalands Lan / Ale Kommun	Siamak Zohari (National Veterinary Institute)
EPI_ISL_1093424	A/Common goldeneye/SVA210202SZ0464/KN000277/2021	A / H5N5	Europe / Sweden / Kalmar Lan / Vasterviks Kommun	Siamak Zohari (National Veterinary Institute)
EPI_ISL_1093425	A/Taiga bean goose/SVA210217SZ0303/KN000463/2021	A / H5N8	Europe / Sweden / Skane Lan / Ystads Kommun	Siamak Zohari (National Veterinary Institute)
EPI_ISL_1093426	A/black-headed gull/Sweden/SVA210216SZ0398/KN000437/20	A / H5N8	Europe / Sweden / Skane Lan / Malmo Kommun	Siamak Zohari (National Veterinary Institute)
EPI_ISL_10943657	A/northern goshawk/Sweden/SVA220210SZ0305/FP000317/O	A / H5N1	Europe / Sweden / Vastra Gotalands Lan / Goteborgs Kommun	Siamak Zohari (National Veterinary Institute)
EPI_ISL_1095363	A/Greylag goose/Sweden/SVA210209SZ0422/KN000346/2021	A / H5N8	Europe / Sweden / Blekinge Lan / Solvesborgs Kommun	Siamak Zohari (National Veterinary Institute)
EPI_ISL_1095365	A/Canada goose/Sweden/SVA210209SZ0420/KN000345/2021	A / H5N8	Europe / Sweden / Blekinge Lan / Solvesborgs Kommun	Siamak Zohari (National Veterinary Institute)
EPI_ISL_1095450	A/barnacle goose/Sweden/SVA210202SZ0453/KN000276/202	A / H5N8	Europe / Sweden / Skane Lan / Lunds Kommun	Siamak Zohari (National Veterinary Institute)
EPI_ISL_1095451	A/barnacle goose/Sweden/SVA210126SZ0462/KN000202/202	A / H5N8	Europe / Sweden / Skane Lan / Malmo Kommun	Siamak Zohari (National Veterinary Institute)
EPI_ISL_1095453	A/Turkey/Sweden/SVA201118SZ0002/KN305633-IP1/2020	A / H5N8	Europe / Sweden / Skane Lan / Tomelilla Kommun	Siamak Zohari (National Veterinary Institute)
EPI_ISL_1095454	A/Common buzzard/Sweden/SVA210212SZ0284/KN000390/20	A / H5N8	Europe / Sweden / Skane Lan / Svedala Kommun	Siamak Zohari (National Veterinary Institute)
EPI_ISL_1098830	A/crane/Kagoshima/KU-93/2021	A / H5N8	Asia / Japan / Kagoshima	Ahmed Magdy Ahmed Khalil (Faculty of Veterinary Medicine - Zagazig University / Zoonoses)
EPI_ISL_10992813	A/Mallard/Netherlands/1/2022	A / H5N1	Europe / Netherlands / Oud Alblas	Mark Pronk (Erasmus Medical Center / Department of Virology)
EPI_ISL_10993074	A/Mallard/Netherlands/2/2022	A / H5N1	Europe / Netherlands / Oud Alblas	Mark Pronk (Erasmus Medical Center / Department of Virology)
EPI_ISL_10993075	A/Mallard/Netherlands/4/2021	A / H5N1	Europe / Netherlands / Zwaagdijk, Eendenkooi Zwaagdijk	Mark Pronk (Erasmus Medical Center / Department of Virology)
EPI_ISL_10993076	A/Mallard/Netherlands/5/2021	A / H5N1	Europe / Netherlands / Zwaagdijk, Eendenkooi Zwaagdijk	Mark Pronk (Erasmus Medical Center / Department of Virology)
EPI_ISL_10993105	A/Mallard/Netherlands/6/2021	A / H5N1	Europe / Netherlands / Zwaagdijk, Eendenkooi Zwaagdijk	Mark Pronk (Erasmus Medical Center / Department of Virology)
EPI_ISL_10993134	A/Mallard/Netherlands/7/2021	A / H5N1	Europe / Netherlands / Zwaagdijk, Eendenkooi Zwaagdijk	Mark Pronk (Erasmus Medical Center / Department of Virology)
EPI_ISL_10993166	A/Mallard/Netherlands/8/2021	A / H5N1	Europe / Netherlands / Zwaagdijk, Eendenkooi Zwaagdijk	Mark Pronk (Erasmus Medical Center / Department of Virology)
EPI_ISL_10993196	A/Sanderling/Netherlands/1/2022	A / H5N1	Europe / Netherlands / Lauwersmeer	Mark Pronk (Erasmus Medical Center / Department of Virology)
EPI_ISL_11007527	A/laying_hen/Moldova/68-1_22VIR638-1/2022	A / H5N1	Europe / Moldova, Republic of	Giacomo Barbierato (Istituto Zooprofilattico Sperimentale delle Venezie / Ricerca e innovazione)
EPI_ISL_11007528	A/rooster/Slovenia/2039_22VIR777-1/2021	A / H5N1	Europe / Slovenia	Giacomo Barbierato (Istituto Zooprofilattico Sperimentale delle Venezie / Ricerca e innovazione)
EPI_ISL_11007529	A/swan/Slovenia/2041_22VIR777-2/2021	A / H5N1	Europe / Slovenia	Giacomo Barbierato (Istituto Zooprofilattico Sperimentale delle Venezie / Ricerca e innovazione)
EPI_ISL_11007530	A/swan/Slovenia/2049_22VIR777-3/2021	A / H5N1	Europe / Slovenia	Giacomo Barbierato (Istituto Zooprofilattico Sperimentale delle Venezie / Ricerca e innovazione)
EPI_ISL_11007531	A/swan/Slovenia/2060_22VIR777-4/2021	A / H5N1	Europe / Slovenia	Giacomo Barbierato (Istituto Zooprofilattico Sperimentale delle Venezie / Ricerca e innovazione)
EPI_ISL_11007532	A/swan/Slovenia/2072_22VIR777-5/2021	A / H5N1	Europe / Slovenia	Giacomo Barbierato (Istituto Zooprofilattico Sperimentale delle Venezie / Ricerca e innovazione)
EPI_ISL_11007533	A/swan/Slovenia/2073_22VIR777-6/2021	A / H5N1	Europe / Slovenia	Giacomo Barbierato (Istituto Zooprofilattico Sperimentale delle Venezie / Ricerca e innovazione)
EPI_ISL_11007534	A/seagull/Slovenia/2075_22VIR777-7/2021	A / H5N1	Europe / Slovenia	Giacomo Barbierato (Istituto Zooprofilattico Sperimentale delle Venezie / Ricerca e innovazione)
EPI_ISL_11007535	A/swan/Slovenia/13_22VIR777-8/2022	A / H5N1	Europe / Slovenia	Giacomo Barbierato (Istituto Zooprofilattico Sperimentale delle Venezie / Ricerca e innovazione)
EPI_ISL_11007537	A/hen/Bulgaria/854-1_22VIR778-10/2021	A / H5N1	Europe / Bulgaria	Giacomo Barbierato (Istituto Zooprofilattico Sperimentale delle Venezie / Ricerca e innovazione)
EPI_ISL_11007538	A/hen/Bulgaria/722-1_22VIR778-1/2021	A / H5N1	Europe / Bulgaria	Giacomo Barbierato (Istituto Zooprofilattico Sperimentale delle Venezie / Ricerca e innovazione)
EPI_ISL_11007539	A/turkey/Bulgaria/755-1_22VIR778-4/2021	A / H5N1	Europe / Bulgaria	Giacomo Barbierato (Istituto Zooprofilattico Sperimentale delle Venezie / Ricerca e innovazione)
EPI_ISL_11007540	A/duck/Bulgaria/756-4_22VIR778-6/2021	A / H5N1	Europe / Bulgaria	Giacomo Barbierato (Istituto Zooprofilattico Sperimentale delle Venezie / Ricerca e innovazione)
EPI_ISL_11007541	A/hen/Bulgaria/757-6_22VIR778-7/2021	A / H5N1	Europe / Bulgaria	Giacomo Barbierato (Istituto Zooprofilattico Sperimentale delle Venezie / Ricerca e innovazione)
EPI_ISL_11007542	A/chicken/Niger/22VIR1409-13/2022	A / H5N1	Africa / Niger	Giacomo Barbierato (Istituto Zooprofilattico Sperimentale delle Venezie / Ricerca e innovazione)
EPI_ISL_11007543	A/chicken/Niger/22VIR1409-23/2022	A / H5N1	Africa / Niger	Giacomo Barbierato (Istituto Zooprofilattico Sperimentale delle Venezie / Ricerca e innovazione)
EPI_ISL_11007544	A/quail/Niger/22VIR1409-28/2022	A / H5N1	Africa / Niger	Giacomo Barbierato (Istituto Zooprofilattico Sperimentale delle Venezie / Ricerca e innovazione)
EPI_ISL_11007545	A/quail/Niger/22VIR1409-30/2022	A / H5N1	Africa / Niger	Giacomo Barbierato (Istituto Zooprofilattico Sperimentale delle Venezie / Ricerca e innovazione)
EPI_ISL_11007546	A/chicken/Niger/22VIR1409-5/2022	A / H5N1	Africa / Niger	Giacomo Barbierato (Istituto Zooprofilattico Sperimentale delle Venezie / Ricerca e innovazione)
EPI_ISL_11007547	A/chicken/Niger/22VIR1409-9/2022	A / H5N1	Africa / Niger	Giacomo Barbierato (Istituto Zooprofilattico Sperimentale delle Venezie / Ricerca e innovazione)
EPI_ISL_11007548	A/turkey/Italy/21VIR9520/2021	A / H5N1	Europe / Italy	Giacomo Barbierato (Istituto Zooprofilattico Sperimentale delle Venezie / Ricerca e innovazione)
EPI_ISL_11007721	A/laying_hen/Moldova/68-2_22VIR638-2/2022	A / H5N1	Europe / Moldova, Republic of	Giacomo Barbierato (Istituto Zooprofilattico Sperimentale delle Venezie / Ricerca e innovazione)
EPI_ISL_11007722	A/partridge/Bulgaria/745_22VIR778-3/2021	A / H5N1	Europe / Bulgaria	Giacomo Barbierato (Istituto Zooprofilattico Sperimentale delle Venezie / Ricerca e innovazione)
EPI_ISL_11019784	A/red fox/Netherlands/22000097-005/2022	A / H5N1	Europe / Netherlands / Provincie Noord-Holland	Rene Heutink (Wageningen Bioveterinary Research)
EPI_ISL_11019882	A/red fox/Netherlands/22002636-002/2022	A / H5N1	Europe / Netherlands / Provincie Gelderland	Rene Heutink (Wageningen Bioveterinary Research)
EPI_ISL_11112340	A/Barnacle Goose/Netherlands/10/2022	A / H5N1	Europe / Netherlands / Schellinkhout, Zuiderdijk	Mark Pronk (Erasmus Medical Center / Department of Virology)
EPI_ISL_11112342	A/Barnacle Goose/Netherlands/11/2022	A / H5N1	Europe / Netherlands / Hippolytushoef, Normerven	Mark Pronk (Erasmus Medical Center / Department of Virology)
EPI_ISL_11112344	A/Barnacle Goose/Netherlands/12/2022	A / H5N1	Europe / Netherlands / Stroe, Waddenkust	Mark Pronk (Erasmus Medical Center / Department of Virology)
EPI_ISL_11112346	A/Barnacle Goose/Netherlands/13/2022	A / H5N1	Europe / Netherlands / IJsselmeer, Enkhuizen, De Kreupel	Mark Pronk (Erasmus Medical Center / Department of Virology)
EPI_ISL_11112348	A/Barnacle Goose/Netherlands/14/2022	A / H5N1	Europe / Netherlands / IJsselmeer, Enkhuizen, De Kreupel	Mark Pronk (Erasmus Medical Center / Department of Virology)
EPI_ISL_11112350	A/Barnacle Goose/Netherlands/15/2022	A / H5N1	Europe / Netherlands / Noord Buren, Waddenkust	Mark Pronk (Erasmus Medical Center / Department of Virology)
EPI_ISL_11112352	A/Barnacle Goose/Netherlands/9/2022	A / H5N1	Europe / Netherlands / Schellinkhout, Zuiderdijk	Mark Pronk (Erasmus Medical Center / Department of Virology)
EPI_ISL_11112354	A/Common Gull/Netherlands/1/2022	A / H5N1	Europe / Netherlands / Hippolytushoef, Waddenzeekust	Mark Pronk (Erasmus Medical Center / Department of Virology)
EPI_ISL_11112356	A/Eurasian Curlew/Netherlands/1/2022	A / H5N1	Europe / Netherlands / Hippolytushoef, Normerven	Mark Pronk (Erasmus Medical Center / Department of Virology)
EPI_ISL_11112358	A/European Herring Gull/Netherlands/2/2022	A / H5N1	Europe / Netherlands / Den Oever, Voorhaven	Mark Pronk (Erasmus Medical Center / Department of Virology)
EPI_ISL_11112360	A/Great black-backed Gull/Netherlands/2/2022	A / H5N1	Europe / Netherlands / IJsselmeer, Enkhuizen, De Kreupel	Mark Pronk (Erasmus Medical Center / Department of Virology)
EPI_ISL_11112362	A/Greylag Goose/Netherlands/1/2022	A / H5N1	Europe / Netherlands / Tersluis, Zuiderdijk	Mark Pronk (Erasmus Medical Center / Department of Virology)
EPI_ISL_11112484	A/Caspian Gull/Netherlands/2/2022	A / H5N1	Europe / Netherlands / IJsselmeer, Enkhuizen, De Kreupel	Mark Pronk (Erasmus Medical Center / Department of Virology)
EPI_ISL_1114725	A/mallard/Kagoshima/KU-d89/2021	A / H5N8	Asia / Japan / Kagoshima	Ahmed Magdy Ahmed Khalil (Faculty of Veterinary Medicine - Zagazig University / Zoonoses)
EPI_ISL_1114735	A/chicken/Tyumen/302-01/2020	A / H5N8	Europe / Russian Federation / Tyumen Oblast / Tyumen	Natalia Goncharova (State Research Center of Virology and Biotechnology (VECTOR) / Emerging Zoonotic Diseases and Influenza)
EPI_ISL_1114736	A/chicken/Tyumen/302-02/2020	A / H5N8	Europe / Russian Federation / Tyumen Oblast / Tyumen	Natalia Goncharova (State Research Center of Virology and Biotechnology (VECTOR) / Emerging Zoonotic Diseases and Influenza)
EPI_ISL_1114737	A/chicken/Kostroma/304-01/2020	A / H5N8	Europe / Russian Federation / Kostroma Oblast / Kostroma	Natalia Goncharova (State Research Center of Virology and Biotechnology (VECTOR) / Emerging Zoonotic Diseases and Influenza)
EPI_ISL_1114738	A/chicken/Kostroma/304-03/2020	A / H5N8	Europe / Russian Federation / Kostroma Oblast / Kostroma	Natalia Goncharova (State Research Center of Virology and Biotechnology (VECTOR) / Emerging Zoonotic Diseases and Influenza)
EPI_ISL_1114740	A/chicken/Kostroma/304-04/2020	A / H5N8	Europe / Russian Federation / Kostroma Oblast / Kostroma	Natalia Goncharova (State Research Center of Virology and Biotechnology (VECTOR) / Emerging Zoonotic Diseases and Influenza)
EPI_ISL_1114741	A/chicken/Kostroma/304-06/2020	A / H5N8	Europe / Russian Federation / Kostroma Oblast / Kostroma	Natalia Goncharova (State Research Center of Virology and Biotechnology (VECTOR) / Emerging Zoonotic Diseases and Influenza)
EPI_ISL_1114742	A/chicken/Kostroma/304-08/2020	A / H5N8	Europe / Russian Federation / Kostroma Oblast / Kostroma	Natalia Goncharova (State Research Center of Virology and Biotechnology (VECTOR) / Emerging Zoonotic Diseases and Influenza)
EPI_ISL_1114745	A/chicken/Kostroma/304-10/2020	A / H5N8	Europe / Russian Federation / Kostroma Oblast / Kostroma	Natalia Goncharova (State Research Center of Virology and Biotechnology (VECTOR) / Emerging Zoonotic Diseases and Influenza)
EPI_ISL_1114746	A/chicken/Rostov-on-Don/308-02/2020	A / H5N8	Europe / Russian Federation / Rostov Oblast / Rostov-on-Don	Natalia Goncharova (State Research Center of Virology and Biotechnology (VECTOR) / Emerging Zoonotic Diseases and Influenza)
EPI_ISL_1114747	A/chicken/Rostov-on-Don/308-03/2020	A / H5N8	Europe / Russian Federation / Rostov Oblast / Rostov-on-Don	Natalia Goncharova (State Research Center of Virology and Biotechnology (VECTOR) / Emerging Zoonotic Diseases and Influenza)
EPI_ISL_1114748	A/chicken/Rostov-on-Don/308-04/2020	A / H5N8	Europe / Russian Federation / Rostov Oblast / Rostov-on-Don	Natalia Goncharova (State Research Center of Virology and Biotechnology (VECTOR) / Emerging Zoonotic Diseases and Influenza)
EPI_ISL_1114749	A/turkey/Stavropol/320-01/2020	A / H5N8	Europe / Russian Federation / Stavropol Krai / Stavropol	Natalia Goncharova (State Research Center of Virology and Biotechnology (VECTOR) / Emerging Zoonotic Diseases and Influenza)
EPI_ISL_1114750	A/turkey/Stavropol/320-02/2020	A / H5N8	Europe / Russian Federation / Stavropol Krai / Stavropol	Natalia Goncharova (State Research Center of Virology and Biotechnology (VECTOR) / Emerging Zoonotic Diseases and Influenza)

EPI_ISL_1114751	A/turkey/Stavropol/320-03/2020	A / H5N8	Europe / Russian Federation / Stavropol Krai / Stavropol	Natalia Goncharova (State Research Center of Virology and Biotechnology (VECTOR) / Emerging Zoonotic Diseases and Influenza)
EPI_ISL_1114752	A/mute swan/North Ossetia-Alania/325-01/2020	A / H5N8	Europe / Russian Federation / Republic of North Ossetia?Alania	Natalia Goncharova (State Research Center of Virology and Biotechnology (VECTOR) / Emerging Zoonotic Diseases and Influenza)
EPI_ISL_1114753	A/mute swan/North Ossetia-Alania/325-02/2020	A / H5N8	Europe / Russian Federation / Republic of North Ossetia?Alania	Natalia Goncharova (State Research Center of Virology and Biotechnology (VECTOR) / Emerging Zoonotic Diseases and Influenza)
EPI_ISL_1114754	A/mute swan/North Ossetia-Alania/325-03/2020	A / H5N8	Europe / Russian Federation / Republic of North Ossetia?Alania	Natalia Goncharova (State Research Center of Virology and Biotechnology (VECTOR) / Emerging Zoonotic Diseases and Influenza)
EPI_ISL_1114755	A/turkey/Rostov-on-Don/332-08/2021	A / H5N8	Europe / Russian Federation / Rostov Oblast / Rostov-on-Don	Natalia Goncharova (State Research Center of Virology and Biotechnology (VECTOR) / Emerging Zoonotic Diseases and Influenza)
EPI_ISL_1114757	A/turkey/Rostov-on-Don/332-09/2021	A / H5N8	Europe / Russian Federation / Rostov Oblast / Rostov-on-Don	Natalia Goncharova (State Research Center of Virology and Biotechnology (VECTOR) / Emerging Zoonotic Diseases and Influenza)
EPI_ISL_1114758	A/turkey/Rostov-on-Don/332-10/2021	A / H5N8	Europe / Russian Federation / Rostov Oblast / Rostov-on-Don	Natalia Goncharova (State Research Center of Virology and Biotechnology (VECTOR) / Emerging Zoonotic Diseases and Influenza)
EPI_ISL_1114759	A/turkey/Rostov-on-Don/332-12/2021	A / H5N8	Europe / Russian Federation / Rostov Oblast / Rostov-on-Don	Natalia Goncharova (State Research Center of Virology and Biotechnology (VECTOR) / Emerging Zoonotic Diseases and Influenza)
EPI_ISL_1114760	A/chicken/Krasnodar/334-01/2021	A / H5N8	Europe / Russian Federation / Krasnodar Krai / Krasnodar	Natalia Goncharova (State Research Center of Virology and Biotechnology (VECTOR) / Emerging Zoonotic Diseases and Influenza)
EPI_ISL_1114761	A/chicken/Krasnodar/334-02/2021	A / H5N8	Europe / Russian Federation / Krasnodar Krai / Krasnodar	Natalia Goncharova (State Research Center of Virology and Biotechnology (VECTOR) / Emerging Zoonotic Diseases and Influenza)
EPI_ISL_1114763	A/chicken/Krasnodar/334-03/2021	A / H5N8	Europe / Russian Federation / Krasnodar Krai / Krasnodar	Natalia Goncharova (State Research Center of Virology and Biotechnology (VECTOR) / Emerging Zoonotic Diseases and Influenza)
EPI_ISL_11208193	A/duck/Guangxi/S30428/2021(H5N6)	A / H5N6	Asia / China / Guangxi	Pengfei Cui (Harbin Veterinary Research Institute (CAAS) / Ministry of Agriculture)
EPI_ISL_11208194	A/duck/Guangxi/S31116/2021(H5N6)	A / H5N6	Asia / China / Guangxi	Pengfei Cui (Harbin Veterinary Research Institute (CAAS) / Ministry of Agriculture)
EPI_ISL_11208195	A/duck/Guizhou/S4702/2021(H5N6)	A / H5N6	Asia / China / Guizhou	Pengfei Cui (Harbin Veterinary Research Institute (CAAS) / Ministry of Agriculture)
EPI_ISL_11208196	A/duck/Hunan/S40199/2021(H5N6)	A / H5N6	Asia / China / Hunan	Pengfei Cui (Harbin Veterinary Research Institute (CAAS) / Ministry of Agriculture)
EPI_ISL_11208197	A/duck/Hunan/S40268/2021(H5N6)	A / H5N6	Asia / China / Hunan	Pengfei Cui (Harbin Veterinary Research Institute (CAAS) / Ministry of Agriculture)
EPI_ISL_11208198	A/duck/Yunnan/S4318/2021(H5N6)	A / H5N6	Asia / China / Yunnan	Pengfei Cui (Harbin Veterinary Research Institute (CAAS) / Ministry of Agriculture)
EPI_ISL_11208199	A/duck/Zhejiang/S4854/2021(H5N6)	A / H5N6	Asia / China / Zhejiang	Pengfei Cui (Harbin Veterinary Research Institute (CAAS) / Ministry of Agriculture)
EPI_ISL_11208200	A/goose/Guangdong/S4751/2021(H5N6)	A / H5N6	Asia / China / Guangdong	Pengfei Cui (Harbin Veterinary Research Institute (CAAS) / Ministry of Agriculture)
EPI_ISL_1121143	A/chicken/Ukraine/1/2016	A / H5N8	Europe / Ukraine / Kherson Oblast / Kalanchatskiy District, Novooleksandrivka	Anna Andriivna Kovalenko (Institute of Veterinary Medicine / Research Training Center for Animal Disease Diagnostics)
EPI_ISL_1121144	A/chicken/Ukraine/3/2016	A / H5N8	Europe / Ukraine / Kherson Oblast / Kalanchatskiy District, Novooleksandrivka	Anna Andriivna Kovalenko (Institute of Veterinary Medicine / Research Training Center for Animal Disease Diagnostics)
EPI_ISL_1121145	A/duck/Ukraine/4/2016	A / H5N8	Europe / Ukraine / Kherson Oblast / Kalanchatskiy District, Novooleksandrivka	Anna Andriivna Kovalenko (Institute of Veterinary Medicine / Research Training Center for Animal Disease Diagnostics)
EPI_ISL_11211731	A/duck/Bangladesh/19D1818/2021	A / H5N1	Asia / Bangladesh	Mohammad Enayet Hossain (International Centre for Diarrhoeal Disease Research (ICDDR,B) / Virology)
EPI_ISL_1123263	A/chicken/Scotland/043405/2020	A / H5N8	Europe / United Kingdom / Orkney	Alex Byrne (Animal and Plant Health Agency (APHA) / Virology Department)
EPI_ISL_1123350	A/chicken/England/043683/2020	A / H5N8	Europe / United Kingdom / Dorset / England	Alex Byrne (Animal and Plant Health Agency (APHA) / Virology Department)
EPI_ISL_1123351	A/duck/England/043628/2020	A / H5N8	Europe / United Kingdom / Norfolk	Alex Byrne (Animal and Plant Health Agency (APHA) / Virology Department)
EPI_ISL_1123352	A/chicken/England/045984/2020	A / H5N8	Europe / United Kingdom / Norfolk	Alex Byrne (Animal and Plant Health Agency (APHA) / Virology Department)
EPI_ISL_1123353	A/duck/England/046311/2020	A / H5N8	Europe / United Kingdom / Norfolk	Alex Byrne (Animal and Plant Health Agency (APHA) / Virology Department)
EPI_ISL_1123354	A/chicken/England/046491/2020	A / H5N8	Europe / United Kingdom / Devon	Alex Byrne (Animal and Plant Health Agency (APHA) / Virology Department)
EPI_ISL_1123355	A/pheasant/Wales/000252/2021	A / H5N8	Europe / United Kingdom / Isle of Anglesey	Alex Byrne (Animal and Plant Health Agency (APHA) / Virology Department)
EPI_ISL_1123357	A/brent_goose/England/233339/2020	A / H5N8	Europe / United Kingdom / Lincolnshire	Alex Byrne (Animal and Plant Health Agency (APHA) / Virology Department)
EPI_ISL_1123358	A/brent_goose/England/095684/2020	A / H5N5	Europe / United Kingdom / Northumberland	Alex Byrne (Animal and Plant Health Agency (APHA) / Virology Department)
EPI_ISL_1123359	A/mute swan/England/263814/2020	A / H5N8	Europe / United Kingdom / Dorset	Alex Byrne (Animal and Plant Health Agency (APHA) / Virology Department)
EPI_ISL_1123360	A/mute_swan/England/234135/2020	A / H5N8	Europe / United Kingdom / Norfolk	Alex Byrne (Animal and Plant Health Agency (APHA) / Virology Department)
EPI_ISL_1123361	A/pheasant/Scotland/000348/2021	A / H5N1	Europe / United Kingdom / Fife	Alex Byrne (Animal and Plant Health Agency (APHA) / Virology Department)
EPI_ISL_11259261	A/herring_gull/Ireland/033533_22VIR1325-10/2021	A / H5N1	Europe / Ireland	Giacomo Barbierato (Istituto Zooprofilattico Sperimentale delle Venezie / Ricerca e innovazione)
EPI_ISL_11259262	A/crow/Ireland/035624_22VIR1325-11/2021	A / H5N1	Europe / Ireland	Giacomo Barbierato (Istituto Zooprofilattico Sperimentale delle Venezie / Ricerca e innovazione)
EPI_ISL_11259263	A/buzzard/Ireland/000656_22VIR1325-12/2022	A / H5N1	Europe / Ireland	Giacomo Barbierato (Istituto Zooprofilattico Sperimentale delle Venezie / Ricerca e innovazione)
EPI_ISL_11259264	A/mute_swan/Ireland/033169_22VIR1325-14/2021	A / H5N1	Europe / Ireland	Giacomo Barbierato (Istituto Zooprofilattico Sperimentale delle Venezie / Ricerca e innovazione)
EPI_ISL_11259265	A/peregrine_falcon/Ireland/000191_22VIR1325-15/2022	A / H5N1	Europe / Ireland	Giacomo Barbierato (Istituto Zooprofilattico Sperimentale delle Venezie / Ricerca e innovazione)
EPI_ISL_11259266	A/mute_swan/Ireland/033945_22VIR1325-16/2021	A / H5N1	Europe / Ireland	Giacomo Barbierato (Istituto Zooprofilattico Sperimentale delle Venezie / Ricerca e innovazione)
EPI_ISL_11259267	A/turkey/Ireland/035425_22VIR1325-17/2021	A / H5N1	Europe / Ireland	Giacomo Barbierato (Istituto Zooprofilattico Sperimentale delle Venezie / Ricerca e innovazione)
EPI_ISL_11259268	A/duck/Ireland/036646_22VIR1325-18/2021	A / H5N1	Europe / Ireland	Giacomo Barbierato (Istituto Zooprofilattico Sperimentale delle Venezie / Ricerca e innovazione)
EPI_ISL_11259269	A/turkey/Ireland/033674_22VIR1325-19/2021	A / H5N1	Europe / Ireland	Giacomo Barbierato (Istituto Zooprofilattico Sperimentale delle Venezie / Ricerca e innovazione)
EPI_ISL_11259270	A/mute_swan/Ireland/032363_22VIR1325-1/2021	A / H5N1	Europe / Ireland	Giacomo Barbierato (Istituto Zooprofilattico Sperimentale delle Venezie / Ricerca e innovazione)
EPI_ISL_11259271	A/broiler/Ireland/033734_22VIR1325-20/2021	A / H5N1	Europe / Ireland	Giacomo Barbierato (Istituto Zooprofilattico Sperimentale delle Venezie / Ricerca e innovazione)
EPI_ISL_11259272	A/layer/Ireland/034424_22VIR1325-21/2021	A / H5N1	Europe / Ireland	Giacomo Barbierato (Istituto Zooprofilattico Sperimentale delle Venezie / Ricerca e innovazione)
EPI_ISL_11259273	A/duck/Ireland/036105_22VIR1325-22/2021	A / H5N1	Europe / Ireland	Giacomo Barbierato (Istituto Zooprofilattico Sperimentale delle Venezie / Ricerca e innovazione)
EPI_ISL_11259274	A/white-tailed_eagle/Ireland/032034_22VIR1325-23/2021	A / H5N1	Europe / Ireland	Giacomo Barbierato (Istituto Zooprofilattico Sperimentale delle Venezie / Ricerca e innovazione)
EPI_ISL_11259275	A/whooper_swan/Ireland/032444_22VIR1325-2/2021	A / H5N1	Europe / Ireland	Giacomo Barbierato (Istituto Zooprofilattico Sperimentale delle Venezie / Ricerca e innovazione)
EPI_ISL_11259276	A/peregrine_falcon/Ireland/032476_22VIR1325-3/2021	A / H5N1	Europe / Ireland	Giacomo Barbierato (Istituto Zooprofilattico Sperimentale delle Venezie / Ricerca e innovazione)
EPI_ISL_11259277	A/whooper_swan/Ireland/032960_22VIR1325-4/2021	A / H5N1	Europe / Ireland	Giacomo Barbierato (Istituto Zooprofilattico Sperimentale delle Venezie / Ricerca e innovazione)
EPI_ISL_11259278	A/magpie/Ireland/032958_22VIR1325-5/2021	A / H5N1	Europe / Ireland	Giacomo Barbierato (Istituto Zooprofilattico Sperimentale delle Venezie / Ricerca e innovazione)
EPI_ISL_11259279	A/greylag_goose/Ireland/032969_22VIR1325-6/2021	A / H5N1	Europe / Ireland	Giacomo Barbierato (Istituto Zooprofilattico Sperimentale delle Venezie / Ricerca e innovazione)
EPI_ISL_11259280	A/greylag_goose/Ireland/033062_22VIR1325-7/2021	A / H5N1	Europe / Ireland	Giacomo Barbierato (Istituto Zooprofilattico Sperimentale delle Venezie / Ricerca e innovazione)
EPI_ISL_11259281	A/white-fronted_goose/Ireland/033181_22VIR1325-8/2021	A / H5N1	Europe / Ireland	Giacomo Barbierato (Istituto Zooprofilattico Sperimentale delle Venezie / Ricerca e innovazione)
EPI_ISL_11259282	A/brent_goose/Ireland/033257_22VIR1325-9/2021	A / H5N1	Europe / Ireland	Giacomo Barbierato (Istituto Zooprofilattico Sperimentale delle Venezie / Ricerca e innovazione)
EPI_ISL_11259283	A/fox/Ireland/3866_22VIR2064-1/2022	A / H5N1	Europe / Ireland	Giacomo Barbierato (Istituto Zooprofilattico Sperimentale delle Venezie / Ricerca e innovazione)
EPI_ISL_11259284	A/goose/Ireland/3869_22VIR2064-2/2022	A / H5N1	Europe / Ireland	Giacomo Barbierato (Istituto Zooprofilattico Sperimentale delle Venezie / Ricerca e innovazione)
EPI_ISL_11259285	A/stork/Spain/442-8_22VIR2142-12/2022	A / H5N1	Europe / Spain	Giacomo Barbierato (Istituto Zooprofilattico Sperimentale delle Venezie / Ricerca e innovazione)
EPI_ISL_11259286	A/goose/Spain/512-2_22VIR2142-13/2022	A / H5N1	Europe / Spain	Giacomo Barbierato (Istituto Zooprofilattico Sperimentale delle Venezie / Ricerca e innovazione)
EPI_ISL_11259287	A/stork/Spain/538-2_22VIR2142-14/2022	A / H5N1	Europe / Spain	Giacomo Barbierato (Istituto Zooprofilattico Sperimentale delle Venezie / Ricerca e innovazione)
EPI_ISL_11259288	A/common_crane/Spain/597-2_22VIR2142-15/2022	A / H5N1	Europe / Spain	Giacomo Barbierato (Istituto Zooprofilattico Sperimentale delle Venezie / Ricerca e innovazione)
EPI_ISL_11259289	A/gray_heron/Spain/602-1_22VIR2142-17/2022	A / H5N1	Europe / Spain	Giacomo Barbierato (Istituto Zooprofilattico Sperimentale delle Venezie / Ricerca e innovazione)
EPI_ISL_11259290	A/stork/Spain/729-1_22VIR2142-18/2022	A / H5N1	Europe / Spain	Giacomo Barbierato (Istituto Zooprofilattico Sperimentale delle Venezie / Ricerca e innovazione)
EPI_ISL_11259291	A/turkey/Spain/140-38_22VIR2142-19/2022	A / H5N1	Europe / Spain	Giacomo Barbierato (Istituto Zooprofilattico Sperimentale delle Venezie / Ricerca e innovazione)
EPI_ISL_11259292	A/swan/Spain/4087-1_22VIR2142-1/2021	A / H5N1	Europe / Spain	Giacomo Barbierato (Istituto Zooprofilattico Sperimentale delle Venezie / Ricerca e innovazione)
EPI_ISL_11259293	A/chicken/Spain/340-37_22VIR2142-20/2022	A / H5N1	Europe / Spain	Giacomo Barbierato (Istituto Zooprofilattico Sperimentale delle Venezie / Ricerca e innovazione)
EPI_ISL_11259294	A/chicken/Spain/452-1_22VIR2142-21/2022	A / H5N1	Europe / Spain	Giacomo Barbierato (Istituto Zooprofilattico Sperimentale delle Venezie / Ricerca e innovazione)
EPI_ISL_11259295	A/chicken/Spain/452-17_22VIR2142-22/2022	A / H5N1	Europe / Spain	Giacomo Barbierato (Istituto Zooprofilattico Sperimentale delle Venezie / Ricerca e innovazione)
EPI_ISL_11259296	A/turkey/Spain/455-83_22VIR2142-23/2022	A / H5N1	Europe / Spain	Giacomo Barbierato (Istituto Zooprofilattico Sperimentale delle Venezie / Ricerca e innovazione)
EPI_ISL_11259297	A/turkey/Spain/455-96_22VIR2142-24/2022	A / H5N1	Europe / Spain	Giacomo Barbierato (Istituto Zooprofilattico Sperimentale delle Venezie / Ricerca e innovazione)

EPI_ISL_11259298	A/turkey/Spain/489-21_22VIR2142-25/2022	A / H5N1	Europe / Spain	Giacomo Barbierato (Istituto Zooprofilattico Sperimentale delle Venezie / Ricerca e innovazione)
EPI_ISL_11259299	A/turkey/Spain/490-22_22VIR2142-26/2022	A / H5N1	Europe / Spain	Giacomo Barbierato (Istituto Zooprofilattico Sperimentale delle Venezie / Ricerca e innovazione)
EPI_ISL_11259300	A/turkey/Spain/490-24_22VIR2142-27/2022	A / H5N1	Europe / Spain	Giacomo Barbierato (Istituto Zooprofilattico Sperimentale delle Venezie / Ricerca e innovazione)
EPI_ISL_11259301	A/turkey/Spain/540-26_22VIR2142-28/2022	A / H5N1	Europe / Spain	Giacomo Barbierato (Istituto Zooprofilattico Sperimentale delle Venezie / Ricerca e innovazione)
EPI_ISL_11259302	A/chicken/Spain/562-1_22VIR2142-29/2022	A / H5N1	Europe / Spain	Giacomo Barbierato (Istituto Zooprofilattico Sperimentale delle Venezie / Ricerca e innovazione)
EPI_ISL_11259303	A/goose/Spain/65-3_22VIR2142-2/2022	A / H5N1	Europe / Spain	Giacomo Barbierato (Istituto Zooprofilattico Sperimentale delle Venezie / Ricerca e innovazione)
EPI_ISL_11259304	A/chicken/Spain/564-4_22VIR2142-30/2022	A / H5N1	Europe / Spain	Giacomo Barbierato (Istituto Zooprofilattico Sperimentale delle Venezie / Ricerca e innovazione)
EPI_ISL_11259305	A/chicken/Spain/564-11_22VIR2142-31/2022	A / H5N1	Europe / Spain	Giacomo Barbierato (Istituto Zooprofilattico Sperimentale delle Venezie / Ricerca e innovazione)
EPI_ISL_11259306	A/turkey/Spain/586-4_22VIR2142-32/2022	A / H5N1	Europe / Spain	Giacomo Barbierato (Istituto Zooprofilattico Sperimentale delle Venezie / Ricerca e innovazione)
EPI_ISL_11259307	A/chicken/Spain/587-1_22VIR2142-33/2022	A / H5N1	Europe / Spain	Giacomo Barbierato (Istituto Zooprofilattico Sperimentale delle Venezie / Ricerca e innovazione)
EPI_ISL_11259308	A/chicken/Spain/622-8_22VIR2142-34/2022	A / H5N1	Europe / Spain	Giacomo Barbierato (Istituto Zooprofilattico Sperimentale delle Venezie / Ricerca e innovazione)
EPI_ISL_11259309	A/chicken/Spain/644-8_22VIR2142-35/2022	A / H5N1	Europe / Spain	Giacomo Barbierato (Istituto Zooprofilattico Sperimentale delle Venezie / Ricerca e innovazione)
EPI_ISL_11259310	A/turkey/Spain/645-1_22VIR2142-36/2022	A / H5N1	Europe / Spain	Giacomo Barbierato (Istituto Zooprofilattico Sperimentale delle Venezie / Ricerca e innovazione)
EPI_ISL_11259311	A/turkey/Spain/646-7_22VIR2142-37/2022	A / H5N1	Europe / Spain	Giacomo Barbierato (Istituto Zooprofilattico Sperimentale delle Venezie / Ricerca e innovazione)
EPI_ISL_11259312	A/chicken/Spain/649-6_22VIR2142-38/2022	A / H5N1	Europe / Spain	Giacomo Barbierato (Istituto Zooprofilattico Sperimentale delle Venezie / Ricerca e innovazione)
EPI_ISL_11259313	A/gray_heron/Spain/88-2_22VIR2142-3/2022	A / H5N1	Europe / Spain	Giacomo Barbierato (Istituto Zooprofilattico Sperimentale delle Venezie / Ricerca e innovazione)
EPI_ISL_11259314	A/duck/Spain/570-2_22VIR2142-40/2022	A / H5N1	Europe / Spain	Giacomo Barbierato (Istituto Zooprofilattico Sperimentale delle Venezie / Ricerca e innovazione)
EPI_ISL_11259315	A/goose/Spain/88-3_22VIR2142-4/2022	A / H5N1	Europe / Spain	Giacomo Barbierato (Istituto Zooprofilattico Sperimentale delle Venezie / Ricerca e innovazione)
EPI_ISL_11259316	A/goose/Spain/141-9_22VIR2142-6/2022	A / H5N1	Europe / Spain	Giacomo Barbierato (Istituto Zooprofilattico Sperimentale delle Venezie / Ricerca e innovazione)
EPI_ISL_11259317	A/stork/Spain/234-2_22VIR2142-7/2022	A / H5N1	Europe / Spain	Giacomo Barbierato (Istituto Zooprofilattico Sperimentale delle Venezie / Ricerca e innovazione)
EPI_ISL_11259318	A/goose/Spain/239-1_22VIR2142-8/2022	A / H5N1	Europe / Spain	Giacomo Barbierato (Istituto Zooprofilattico Sperimentale delle Venezie / Ricerca e innovazione)
EPI_ISL_11259319	A/goose/Spain/294-2_22VIR2142-9/2022	A / H5N1	Europe / Spain	Giacomo Barbierato (Istituto Zooprofilattico Sperimentale delle Venezie / Ricerca e innovazione)
EPI_ISL_11260218	A/mute_swan/Ireland/037311_22VIR1325-13/2021	A / H5N1	Europe / Ireland	Giacomo Barbierato (Istituto Zooprofilattico Sperimentale delle Venezie / Ricerca e innovazione)
EPI_ISL_11268632	A/mute swan/Croatia/26/2022	A / H5N1	Europe / Croatia / Vukovarsko-Srijemska Zupanija / Ivankovo (45°15'31.29 18°42'28.27)	Vladimir Savi? (Croatian Veterinary Institute / Poultry Centre)
EPI_ISL_11325034	A/turkey/Egypt/Cairo/AH/2019	A / H5N8	Africa / Egypt / Cairo	El-Sayed M. Abdelwhab (Friedrich-Loeffler-Institut)
EPI_ISL_11325035	A/turkey/Egypt/Alex-AH/2019	A / H5N8	Africa / Egypt / Alexandria	El-Sayed M. Abdelwhab (Friedrich-Loeffler-Institut)
EPI_ISL_11325036	A/turkey/Egypt/Alex-AH1/2019	A / H5N8	Africa / Egypt / Alexandria	El-Sayed M. Abdelwhab (Friedrich-Loeffler-Institut)
EPI_ISL_11325037	A/duck/Egypt/Elbehera-AH2/2019	A / H5N8	Africa / Egypt / Elbehera	El-Sayed M. Abdelwhab (Friedrich-Loeffler-Institut)
EPI_ISL_11325038	A/duck/Egypt/Behera-HB2-AH/2020	A / H5N8	Africa / Egypt / Elbehera	El-Sayed M. Abdelwhab (Friedrich-Loeffler-Institut)
EPI_ISL_11325039	A/duck/Egypt/Behera-AH1/2019	A / H5N8	Africa / Egypt / Elbehera	El-Sayed M. Abdelwhab (Friedrich-Loeffler-Institut)
EPI_ISL_11325040	A/chicken/Egypt/sohag-AH/2020	A / H5N8	Africa / Egypt / Sohag	El-Sayed M. Abdelwhab (Friedrich-Loeffler-Institut)
EPI_ISL_11325041	A/chicken/Egypt/qalyubia-layer-AH/2020	A / H5N8	Africa / Egypt / Qalyubia	El-Sayed M. Abdelwhab (Friedrich-Loeffler-Institut)
EPI_ISL_11325042	A/chicken/Egypt/kafrelsheik-AH/2019	A / H5N8	Africa / Egypt / Kafr El-Sheikh	El-Sayed M. Abdelwhab (Friedrich-Loeffler-Institut)
EPI_ISL_11325043	A/chicken/Egypt/Giza-HG4L-AH/2020	A / H5N8	Africa / Egypt / Giza	El-Sayed M. Abdelwhab (Friedrich-Loeffler-Institut)
EPI_ISL_11325044	A/chicken/Egypt/Giza-AH/2020	A / H5N8	Africa / Egypt / Giza	El-Sayed M. Abdelwhab (Friedrich-Loeffler-Institut)
EPI_ISL_11325045	A/chicken/Egypt/Elmonufia-backyard-AH/2019	A / H5N8	Africa / Egypt / El-Monoufiya	El-Sayed M. Abdelwhab (Friedrich-Loeffler-Institut)
EPI_ISL_11325046	A/chicken/Egypt/Elmonufia-AH/2020	A / H5N8	Africa / Egypt / El-Monoufiya	El-Sayed M. Abdelwhab (Friedrich-Loeffler-Institut)
EPI_ISL_11325047	A/chicken/Egypt/Cairo-HC11B-AH/2020	A / H5N8	Africa / Egypt / Cairo	El-Sayed M. Abdelwhab (Friedrich-Loeffler-Institut)
EPI_ISL_11325048	A/chicken/Egypt/behera-AH/2021	A / H5N8	Africa / Egypt / Elbehera	El-Sayed M. Abdelwhab (Friedrich-Loeffler-Institut)
EPI_ISL_11325049	A/chicken/Egypt/Assiut-AH/2019	A / H5N8	Africa / Egypt / Assiut	El-Sayed M. Abdelwhab (Friedrich-Loeffler-Institut)
EPI_ISL_11325050	A/chicken/Egypt/Alex-Breeder-AH/2021	A / H5N8	Africa / Egypt / Alexandria	El-Sayed M. Abdelwhab (Friedrich-Loeffler-Institut)
EPI_ISL_11325051	A/chicken/Egypt/Alex-AH/2020	A / H5N8	Africa / Egypt / Alexandria	El-Sayed M. Abdelwhab (Friedrich-Loeffler-Institut)
EPI_ISL_11325052	A/chicken/Egypt/Alex-AH2/2020	A / H5N8	Africa / Egypt / Alexandria	El-Sayed M. Abdelwhab (Friedrich-Loeffler-Institut)
EPI_ISL_11325053	A/chicken/Egypt/Alex-AH1/2020	A / H5N8	Africa / Egypt / Alexandria	El-Sayed M. Abdelwhab (Friedrich-Loeffler-Institut)
EPI_ISL_11327088	A/chicken/Czech Republic/61-1/2022	A / H5N1	Europe / Czech Republic / South Moravian Region / Okres Znoimo / Bojanovice, GPS: 48.969002[illegible]	Alexander Nagy (State Veterinary Institute Prague)
EPI_ISL_11327089	A/chicken/Czech Republic/61-2/2022	A / H5N1	Europe / Czech Republic / South Moravian Region / Okres Znoimo / Bojanovice, GPS: 48.969002[illegible]	Alexander Nagy (State Veterinary Institute Prague)
EPI_ISL_11327090	A/chicken/Czech Republic/63/2022	A / H5N1	Europe / Czech Republic / Ustecky Kraj / Okres Louny / Blazim GPS: 50.4061775N, 13.6296733E	Alexander Nagy (State Veterinary Institute Prague)
EPI_ISL_11330431	A/white-tailed eagle/Hokkaido/22-RU-WTE-2/2022	A / H5N1	Asia / Japan / Hokkaido / Tomamae town	Norikazu Isoda (Graduate School of Veterinary Medicine, Hokkaido University / Laboratory of Microbiology)
EPI_ISL_1139014	A/barnacle goose/Netherlands/20016511-002/2020	A / H5N8	Europe / Netherlands / Provincie Groningen / Lauwersoog	Rene Heutink (Wageningen Bioveterinary Research)
EPI_ISL_1139015	A/wild bird/Netherlands/20016515-002/2020	A / H5N8	Europe / Netherlands / Provincie Friesland / Cornwerd	Rene Heutink (Wageningen Bioveterinary Research)
EPI_ISL_1139016	A/mute swan/Netherlands/20016516-001/2020	A / H5N8	Europe / Netherlands / Provincie Friesland / Aldeboarn	Rene Heutink (Wageningen Bioveterinary Research)
EPI_ISL_1139017	A/mute swan/Netherlands/20016634-001/2020	A / H5N8	Europe / Netherlands / South Holland / Berkenwoude	Rene Heutink (Wageningen Bioveterinary Research)
EPI_ISL_1139018	A/cormorant/Netherlands/20016582-005/2020	A / H5N8	Europe / Netherlands / Provincie Noord-Holland / Edam-Volendam	Rene Heutink (Wageningen Bioveterinary Research)
EPI_ISL_1139019	A/mute swan/Netherlands/20016618-001/2020	A / H5N8	Europe / Netherlands / Provincie Utrecht / Kockengen	Rene Heutink (Wageningen Bioveterinary Research)
EPI_ISL_1139020	A/greylag goose/Netherlands/20016756-001/2020	A / H5N8	Europe / Netherlands / Provincie Friesland / Vegelinsoord	Rene Heutink (Wageningen Bioveterinary Research)
EPI_ISL_1139021	A/eurasian wigeon/Netherlands/20016758-001/2020	A / H5N8	Europe / Netherlands / Provincie Friesland / Terherne	Rene Heutink (Wageningen Bioveterinary Research)
EPI_ISL_1139022	A/barnacle goose/Netherlands/20016888-001/2020	A / H5N8	Europe / Netherlands / Provincie Friesland / Buren	Rene Heutink (Wageningen Bioveterinary Research)
EPI_ISL_1139023	A/short-eared owl/Netherlands/20016896-017/2020	A / H5N8	Europe / Netherlands / Provincie Friesland / Holwerd	Rene Heutink (Wageningen Bioveterinary Research)
EPI_ISL_1139024	A/mute swan/Netherlands/20016973-001/2020	A / H5N8	Europe / Netherlands / South Holland / Gemeente Rotterdam	Rene Heutink (Wageningen Bioveterinary Research)
EPI_ISL_1139025	A/barnacle goose/Netherlands/20016974-002/2020	A / H5N8	Europe / Netherlands / Provincie Friesland / Gemeente Leeuwarden	Rene Heutink (Wageningen Bioveterinary Research)
EPI_ISL_1139026	A/greylag goose/Netherlands/20016975-004/2020	A / H5N8	Europe / Netherlands / South Holland / Alphen a/d Rijn	Rene Heutink (Wageningen Bioveterinary Research)
EPI_ISL_1139027	A/egyptian goose/Netherlands/20017027-002/2020	A / H5N8	Europe / Netherlands / South Holland / Gemeente Leiderdorp	Rene Heutink (Wageningen Bioveterinary Research)
EPI_ISL_1139028	A/barnacle goose/Netherlands/20017051-001/2020	A / H5N8	Europe / Netherlands / Provincie Noord-Holland / Gemeente Landsmeer	Rene Heutink (Wageningen Bioveterinary Research)
EPI_ISL_1139029	A/barnacle goose/Netherlands/20017051-006/2020	A / H5N8	Europe / Netherlands / Provincie Noord-Holland / Gemeente Landsmeer	Rene Heutink (Wageningen Bioveterinary Research)
EPI_ISL_1139030	A/barnacle goose/Netherlands/20017052-001/2020	A / H5N8	Europe / Netherlands / Provincie Friesland / Gemeente Schiermonnikoog	Rene Heutink (Wageningen Bioveterinary Research)
EPI_ISL_1139031	A/mute swan/Netherlands/20017131-006/2020	A / H5N8	Europe / Netherlands / South Holland / Nieuwerkerk a/d Ijssel	Rene Heutink (Wageningen Bioveterinary Research)
EPI_ISL_1139032	A/barnacle goose/Netherlands/20017159-006/2020	A / H5N8	Europe / Netherlands / Provincie Friesland / Rijs	Rene Heutink (Wageningen Bioveterinary Research)
EPI_ISL_1139033	A/greylag goose/Netherlands/20017399-006/2020	A / H5N8	Europe / Netherlands / South Holland / Hazerswoude-Dorp	Rene Heutink (Wageningen Bioveterinary Research)
EPI_ISL_1139034	A/barnacle goose/Netherlands/20017405-002/2020	A / H5N8	Europe / Netherlands / Provincie Noord-Holland / Badhoevedorp	Rene Heutink (Wageningen Bioveterinary Research)
EPI_ISL_1139035	A/greylag goose/Netherlands/20016877-001/2020	A / H5N8	Europe / Netherlands / Provincie Noord-Holland / Gemeente Heerhugowaard	Rene Heutink (Wageningen Bioveterinary Research)
EPI_ISL_1139036	A/brant goose/Netherlands/20016948-002/2020	A / H5N8	Europe / Netherlands / Provincie Noord-Holland / Gemeente Amstelveen	Rene Heutink (Wageningen Bioveterinary Research)
EPI_ISL_1139037	A/barnacle goose/Netherlands/20016951-001/2020	A / H5N8	Europe / Netherlands / South Holland / Pijnacker	Rene Heutink (Wageningen Bioveterinary Research)

EPI_ISL_1139038	A/wild goose/Netherlands/20016959-001/2020	A / H5N8	Europe / Netherlands / Provincie Limburg / Horst	Rene Heutink (Wageningen Bioveterinary Research)
EPI_ISL_1139039	A/mute swan/Netherlands/20016960-001/2020	A / H5N8	Europe / Netherlands / South Holland / Zevenhoven	Rene Heutink (Wageningen Bioveterinary Research)
EPI_ISL_1139040	A/mute swan/Netherlands/20017061-001/2020	A / H5N8	Europe / Netherlands / Provincie Drenthe / Wapserveen	Rene Heutink (Wageningen Bioveterinary Research)
EPI_ISL_1139041	A/greylag goose/Netherlands/20017064-002/2020	A / H5N8	Europe / Netherlands / Provinvie Utrecht	Rene Heutink (Wageningen Bioveterinary Research)
EPI_ISL_1139042	A/mute swan/Netherlands/20017153-002/2020	A / H5N8	Europe / Netherlands / South Holland / Rijpwetering	Rene Heutink (Wageningen Bioveterinary Research)
EPI_ISL_1139043	A/mute swan/Netherlands/20017547-002/2020	A / H5N8	Europe / Netherlands / South Holland / Gemeente Zoeterwoude	Rene Heutink (Wageningen Bioveterinary Research)
EPI_ISL_1139044	A/eurasian oystercatcher/Netherlands/20017557-003/2020	A / H5N8	Europe / Netherlands / Provincie Friesland / West Terschelling	Rene Heutink (Wageningen Bioveterinary Research)
EPI_ISL_1139045	A/greylag goose/Netherlands/20017476-001/2020	A / H5N8	Europe / Netherlands / Provincie Noord-Holland / West-Graftdijk	Rene Heutink (Wageningen Bioveterinary Research)
EPI_ISL_1139046	A/greater canada goose/Netherlands/20017479-002/2020	A / H5N8	Europe / Netherlands / South Holland / Krimpen a/d Ijssel	Rene Heutink (Wageningen Bioveterinary Research)
EPI_ISL_1139047	A/northern lapwing/Netherlands/20017480-001/2020	A / H5N8	Europe / Netherlands / South Holland / Gemeente Spijkenisse	Rene Heutink (Wageningen Bioveterinary Research)
EPI_ISL_1139048	A/wild goose/Netherlands/20017495-002/2020	A / H5N8	Europe / Netherlands / Provincie Groningen / Gemeente Winschoten	Rene Heutink (Wageningen Bioveterinary Research)
EPI_ISL_1139049	A/gadwall/Netherlands/20017254-001/2020	A / H5N8	Europe / Netherlands / South Holland / Gemeente Delft	Rene Heutink (Wageningen Bioveterinary Research)
EPI_ISL_1139050	A/northern lapwing/Netherlands/20017255-001/2020	A / H5N8	Europe / Netherlands / South Holland / Gemeente Delft	Rene Heutink (Wageningen Bioveterinary Research)
EPI_ISL_1139051	A/greylag goose/Netherlands/20017256-002/2020	A / H5N8	Europe / Netherlands / Provincie Noord-Holland / Gemeente Zaanstad	Rene Heutink (Wageningen Bioveterinary Research)
EPI_ISL_1139076	A/common kestrel/Netherlands/20017381-001/2020	A / H5N8	Europe / Netherlands / South Holland / Roelofarendsveen	Rene Heutink (Wageningen Bioveterinary Research)
EPI_ISL_1139078	A/pink-footed goose/Netherlands/20017382-001/2020	A / H5N8	Europe / Netherlands / Provincie Limburg / Leudal	Rene Heutink (Wageningen Bioveterinary Research)
EPI_ISL_1139079	A/barnacle goose/Netherlands/20016888-002/2020	A / H5N8	Europe / Netherlands / Provincie Friesland / Buren	Rene Heutink (Wageningen Bioveterinary Research)
EPI_ISL_1139080	A/barnacle goose/Netherlands/20016888-003/2020	A / H5N8	Europe / Netherlands / Provincie Friesland / Buren	Rene Heutink (Wageningen Bioveterinary Research)
EPI_ISL_1139081	A/greylag goose/Netherlands/20016896-001/2020	A / H5N8	Europe / Netherlands / Provincie Friesland / Holwerd	Rene Heutink (Wageningen Bioveterinary Research)
EPI_ISL_1139082	A/barnacle goose/Netherlands/20016896-011/2020	A / H5N8	Europe / Netherlands / Provincie Friesland / Holwerd	Rene Heutink (Wageningen Bioveterinary Research)
EPI_ISL_1139083	A/barnacle goose/Netherlands/20016896-012/2020	A / H5N8	Europe / Netherlands / Provincie Friesland / Holwerd	Rene Heutink (Wageningen Bioveterinary Research)
EPI_ISL_1139084	A/eurasian wigeon/Netherlands/20016896-025/2020	A / H5N8	Europe / Netherlands / Provincie Friesland / Holwerd	Rene Heutink (Wageningen Bioveterinary Research)
EPI_ISL_1139085	A/barnacle goose/Netherlands/20016935-003/2020	A / H5N8	Europe / Netherlands / Provincie Friesland / Holwerd	Rene Heutink (Wageningen Bioveterinary Research)
EPI_ISL_1139086	A/greater canada goose/Netherlands/20017403-003/2020	A / H5N8	Europe / Netherlands / North Brabant / Rijswijk (NB)	Rene Heutink (Wageningen Bioveterinary Research)
EPI_ISL_1139087	A/barnacle goose/Netherlands/20017557-001/2020	A / H5N8	Europe / Netherlands / Provincie Friesland / West Terschelling	Rene Heutink (Wageningen Bioveterinary Research)
EPI_ISL_1139088	A/barnacle goose/Netherlands/20017557-002/2020	A / H5N8	Europe / Netherlands / Provincie Friesland / West Terschelling	Rene Heutink (Wageningen Bioveterinary Research)
EPI_ISL_1139089	A/wild duck/Netherlands/20017794-001/2020	A / H5N8	Europe / Netherlands / Provincie Friesland / Grou	Rene Heutink (Wageningen Bioveterinary Research)
EPI_ISL_1139090	A/common buzzard/Netherlands/20017824-001/2020	A / H5N8	Europe / Netherlands / North Brabant / Oudgastel	Rene Heutink (Wageningen Bioveterinary Research)
EPI_ISL_1139091	A/muscovy duck/Netherlands/20017611-002/2020	A / H5N8	Europe / Netherlands / Provincie Friesland / Grou	Rene Heutink (Wageningen Bioveterinary Research)
EPI_ISL_1139092	A/barnacle goose/Netherlands/20017713-002/2020	A / H5N8	Europe / Netherlands / Provincie Groningen / Den Andel	Rene Heutink (Wageningen Bioveterinary Research)
EPI_ISL_1139093	A/gadwall/Netherlands/20017716-001/2020	A / H5N8	Europe / Netherlands / Provincie Noord-Holland / Zwaanshoek	Rene Heutink (Wageningen Bioveterinary Research)
EPI_ISL_1139094	A/mute swan/Netherlands/20017717-002/2020	A / H5N8	Europe / Netherlands / Provincie Groningen / Noordlaren	Rene Heutink (Wageningen Bioveterinary Research)
EPI_ISL_1139095	A/great egret/Netherlands/20017754-002/2020	A / H5N8	Europe / Netherlands / Provincie Utrecht / Haarzuilens	Rene Heutink (Wageningen Bioveterinary Research)
EPI_ISL_1139096	A/wild goose/Netherlands/20017755-002/2020	A / H5N8	Europe / Netherlands / Provincie Utrecht / Haarzuilens	Rene Heutink (Wageningen Bioveterinary Research)
EPI_ISL_1139097	A/wild goose/Netherlands/20017761-002/2020	A / H5N8	Europe / Netherlands / Provincie Utrecht / Waverveen	Rene Heutink (Wageningen Bioveterinary Research)
EPI_ISL_1139098	A/eurasian wigeon/Netherlands/20017908-002/2020	A / H5N8	Europe / Netherlands / Provincie Groningen / Den Andel	Rene Heutink (Wageningen Bioveterinary Research)
EPI_ISL_1139099	A/barnacle goose/Netherlands/20017984-004/2020	A / H5N8	Europe / Netherlands / Provincie Zeeland / Zierikzee	Rene Heutink (Wageningen Bioveterinary Research)
EPI_ISL_1139100	A/barnacle goose/Netherlands/20017604-001/2020	A / H5N8	Europe / Netherlands / South Holland / Gemeente Oud-Beijerland	Rene Heutink (Wageningen Bioveterinary Research)
EPI_ISL_1139101	A/swan/Netherlands/20017605-002/2020	A / H5N1	Europe / Netherlands / Provincie Noord-Holland / Groet	Rene Heutink (Wageningen Bioveterinary Research)
EPI_ISL_1139102	A/swan/Netherlands/20017772-002/2020	A / H5N1	Europe / Netherlands / Provincie Noord-Holland / Schoorl	Rene Heutink (Wageningen Bioveterinary Research)
EPI_ISL_1139103	A/peregrine falcon/Netherlands/20017773-002/2020	A / H5N8	Europe / Netherlands / Provincie Friesland / Oost-Vlieland	Rene Heutink (Wageningen Bioveterinary Research)
EPI_ISL_1139104	A/wild goose/Netherlands/20017816-001/2020	A / H5N8	Europe / Netherlands / Provincie Friesland / Grou	Rene Heutink (Wageningen Bioveterinary Research)
EPI_ISL_1139105	A/greylag goose/Netherlands/20016523-001/2020	A / H5N8	Europe / Netherlands / Provincie Noord-Holland / Velserbroek	Rene Heutink (Wageningen Bioveterinary Research)
EPI_ISL_1139106	A/eurasian curlew/Netherlands/20016896-019/2020	A / H5N8	Europe / Netherlands / Provincie Friesland / Holwerd	Rene Heutink (Wageningen Bioveterinary Research)
EPI_ISL_1139107	A/muscovy duck/Netherlands/20018067-001/2020	A / H5N8	Europe / Netherlands / South Holland / Gemeente Nieuwkoop	Rene Heutink (Wageningen Bioveterinary Research)
EPI_ISL_1139108	A/mute swan/Netherlands/20018754-004/2020	A / H5N8	Europe / Netherlands / Provincie Friesland / Schraard	Rene Heutink (Wageningen Bioveterinary Research)
EPI_ISL_1139109	A/mute swan/Netherlands/20018754-006/2020	A / H5N8	Europe / Netherlands / Provincie Friesland / Schraard	Rene Heutink (Wageningen Bioveterinary Research)
EPI_ISL_1139110	A/swan/Netherlands/20018830-004/2020	A / H5N8	Europe / Netherlands / Provincie Utrecht / Mijdrecht	Rene Heutink (Wageningen Bioveterinary Research)
EPI_ISL_1139112	A/greylag goose/Netherlands/20017386-001/2020	A / H5N8	Europe / Netherlands / Provincie Friesland / Gemeente Harlingen	Rene Heutink (Wageningen Bioveterinary Research)
EPI_ISL_1139113	A/wild goose/Netherlands/20017819-001/2020	A / H5N8	Europe / Netherlands / Provincie Utrecht / Vinkeveen	Rene Heutink (Wageningen Bioveterinary Research)
EPI_ISL_1139159	A/greylag goose/Netherlands/20017058-002/2020	A / H5N8	Europe / Netherlands / Provincie Utrecht / Kockengen	Rene Heutink (Wageningen Bioveterinary Research)
EPI_ISL_1139160	A/pink-footed goose/Netherlands/20018068-001/2020	A / H5N8	Europe / Netherlands / Provincie Noord-Holland / Ankeveen	Rene Heutink (Wageningen Bioveterinary Research)
EPI_ISL_1139161	A/greylag goose/Netherlands/20018070-002/2020	A / H5N8	Europe / Netherlands / South Holland / Harddinxveld	Rene Heutink (Wageningen Bioveterinary Research)
EPI_ISL_1139162	A/black swan/Netherlands/20018185-001/2020	A / H5N8	Europe / Netherlands / South Holland / Gemeente Zoetermeer	Rene Heutink (Wageningen Bioveterinary Research)
EPI_ISL_1139163	A/common buzzard/Netherlands/20018339-002/2020	A / H5N8	Europe / Netherlands / Provincie Friesland / Grou	Rene Heutink (Wageningen Bioveterinary Research)
EPI_ISL_1139164	A/northern goshawk/Netherlands/20018560-002/2020	A / H5N8	Europe / Netherlands / South Holland / Leidschendam-Voorburg	Rene Heutink (Wageningen Bioveterinary Research)
EPI_ISL_1139165	A/common snipe/Netherlands/20018931-003/2020	A / H5N8	Europe / Netherlands / Provincie Utrecht / Ameide	Rene Heutink (Wageningen Bioveterinary Research)
EPI_ISL_1139166	A/wild goose/Netherlands/20018735-002/2020	A / H5N8	Europe / Netherlands / Provincie Overijssel / Gemeente Almelo	Rene Heutink (Wageningen Bioveterinary Research)
EPI_ISL_1139167	A/barnacle goose/Netherlands/20018737-002/2020	A / H5N8	Europe / Netherlands / South Holland / Gemeente Papendrecht	Rene Heutink (Wageningen Bioveterinary Research)
EPI_ISL_1139168	A/mute swan/Netherlands/20018738-001/2020	A / H5N8	Europe / Netherlands / South Holland / Rockanje	Rene Heutink (Wageningen Bioveterinary Research)
EPI_ISL_1139169	A/peregrine falcon/Netherlands/20018821-002/2020	A / H5N8	Europe / Netherlands / Provincie Gelderland / Garderen	Rene Heutink (Wageningen Bioveterinary Research)
EPI_ISL_1139170	A/wild goose/Netherlands/20018822-002/2020	A / H5N8	Europe / Netherlands / Provincie Utrecht / Gemeente Oudewater	Rene Heutink (Wageningen Bioveterinary Research)
EPI_ISL_1139171	A/mute swan/Netherlands/20018824-002/2020	A / H5N8	Europe / Netherlands / Provincie Utrecht / Harmelen	Rene Heutink (Wageningen Bioveterinary Research)
EPI_ISL_1139172	A/mute swan/Netherlands/20018923-001/2020	A / H5N8	Europe / Netherlands / South Holland / Stellendam	Rene Heutink (Wageningen Bioveterinary Research)
EPI_ISL_1139173	A/mute swan/Netherlands/20019137-005/2020	A / H5N8	Europe / Netherlands / South Holland / Alphen a/d Rijn	Rene Heutink (Wageningen Bioveterinary Research)
EPI_ISL_11406398	A/chicken/England/002070/2022	A / H5N1	Europe / United Kingdom / England	Alex Byrne (Animal and Plant Health Agency (APHA) / Virology Department)
EPI_ISL_11406399	A/turkey/England/004737/2022	A / H5N1	Europe / United Kingdom / England	Alex Byrne (Animal and Plant Health Agency (APHA) / Virology Department)
EPI_ISL_11406401	A/chicken/England/000187/2022	A / H5N1	Europe / United Kingdom / England	Alex Byrne (Animal and Plant Health Agency (APHA) / Virology Department)
EPI_ISL_11406402	A/domestic_duck/England/007588/2022	A / H5N1	Europe / United Kingdom / England	Alex Byrne (Animal and Plant Health Agency (APHA) / Virology Department)
EPI_ISL_11406403	A/black-headed_gull/England/306270/2022	A / H5N1	Europe / United Kingdom / England	Alex Byrne (Animal and Plant Health Agency (APHA) / Virology Department)
EPI_ISL_11449674	A/Gallus_gallus/Belgium/3194_0001/2022	A / H5N1	Europe / Belgium / Provincie Oost-Vlaanderen / Sint-Gillis-Waas	Steven Van Borm (Sciensano, Department of Animal Infectious Diseases / Animal Infectious Diseases)
EPI_ISL_11504587	A/chicken/Vietnam/HU14-LB11/2021	A / H5N8	Asia / Vietnam / Tinh Lang Son	Norikazu Isoda (Graduate School of Veterinary Medicine, Hokkaido University / Laboratory of Microbiology)

EPI_ISL_11560233	A/Brent goose/Netherlands/1/2022	A / H5N1	Europe / Netherlands / Stroe, Waddenkust	Mark Pronk (Erasmus Medical Center / Department of Virology)
EPI_ISL_11560235	A/Barnacle Goose/Netherlands/16/2022	A / H5N1	Europe / Netherlands / Ameland	Mark Pronk (Erasmus Medical Center / Department of Virology)
EPI_ISL_11560236	A/Barnacle Goose/Netherlands/17/2022	A / H5N1	Europe / Netherlands / Ameland	Mark Pronk (Erasmus Medical Center / Department of Virology)
EPI_ISL_11560323	A/Barnacle Goose/Netherlands/18/2022	A / H5N1	Europe / Netherlands / Enkhuizen, Ijsselmeer, De Kreupel	Mark Pronk (Erasmus Medical Center / Department of Virology)
EPI_ISL_11560324	A/Graylag goose/Netherlands/2/20222	A / H5N1	Europe / Netherlands / Woerden, Breeveld	Mark Pronk (Erasmus Medical Center / Department of Virology)
EPI_ISL_11560325	A/Great black-backed Gull/Netherlands/3/2022	A / H5N1	Europe / Netherlands / Wieringerwerf, Zuiderdijkweg	Mark Pronk (Erasmus Medical Center / Department of Virology)
EPI_ISL_11561589	A/chicken/England/011981/2022	A / H5N1	Europe / United Kingdom / England	Alex Byrne (Animal and Plant Health Agency (APHA) / Virology Department)
EPI_ISL_11561590	A/chicken/England/012967/2022	A / H5N1	Europe / United Kingdom / England	Alex Byrne (Animal and Plant Health Agency (APHA) / Virology Department)
EPI_ISL_11561591	A/domestic_duck/England/012973/2022	A / H5N1	Europe / United Kingdom / England	Alex Byrne (Animal and Plant Health Agency (APHA) / Virology Department)
EPI_ISL_11561592	A/turkey/England/016515/2022	A / H5N1	Europe / United Kingdom / England	Alex Byrne (Animal and Plant Health Agency (APHA) / Virology Department)
EPI_ISL_11561593	A/black-headed_gull/England/388256/2022	A / H5N1	Europe / United Kingdom / England	Alex Byrne (Animal and Plant Health Agency (APHA) / Virology Department)
EPI_ISL_11580255	A/Greylag goose/Sweden/SVA220308SZ0382/FB000559/M-20[illegible]	A / H5N1	Europe / Sweden / Skane Lan / Trelleborgs Kommun	Siamak Zohari (National Veterinary Institute)
EPI_ISL_11628212	A/chicken/Virginia/22-004415-001/2022	A / H5N1	North America / United States / Virginia / Fauquier	Mary Lea Killian (National Veterinary Services Laboratories - USDA / Diagnostic Virology Laboratory)
EPI_ISL_11628213	A/chicken/Kentucky/22-004416-001/2022	A / H5N1	North America / United States / Kentucky / Fulton	Mary Lea Killian (National Veterinary Services Laboratories - USDA / Diagnostic Virology Laboratory)
EPI_ISL_11628214	A/chicken/Kentucky/22-004416-002/2022	A / H5N1	North America / United States / Kentucky / Fulton	Mary Lea Killian (National Veterinary Services Laboratories - USDA / Diagnostic Virology Laboratory)
EPI_ISL_11628215	A/chicken/Kentucky/22-004416-003/2022	A / H5N1	North America / United States / Kentucky / Fulton	Mary Lea Killian (National Veterinary Services Laboratories - USDA / Diagnostic Virology Laboratory)
EPI_ISL_11628216	A/turkey/Kentucky/22-004546-001/2022	A / H5N1	North America / United States / Kentucky / Webster	Mary Lea Killian (National Veterinary Services Laboratories - USDA / Diagnostic Virology Laboratory)
EPI_ISL_11628217	A/turkey/Kentucky/22-004546-002/2022	A / H5N1	North America / United States / Kentucky / Webster	Mary Lea Killian (National Veterinary Services Laboratories - USDA / Diagnostic Virology Laboratory)
EPI_ISL_11628218	A/turkey/Indiana/22-004688-002/2022	A / H5N1	North America / United States / Indiana / Dubois	Mary Lea Killian (National Veterinary Services Laboratories - USDA / Diagnostic Virology Laboratory)
EPI_ISL_11628219	A/turkey/Indiana/22-004688-003/2022	A / H5N1	North America / United States / Indiana / Dubois	Mary Lea Killian (National Veterinary Services Laboratories - USDA / Diagnostic Virology Laboratory)
EPI_ISL_11628220	A/turkey/Indiana/22-004688-006/2022	A / H5N1	North America / United States / Indiana / Dubois	Mary Lea Killian (National Veterinary Services Laboratories - USDA / Diagnostic Virology Laboratory)
EPI_ISL_11628221	A/chicken/Maine/22-005158-001/2022	A / H5N1	North America / United States / Maine / Knox	Mary Lea Killian (National Veterinary Services Laboratories - USDA / Diagnostic Virology Laboratory)
EPI_ISL_11628222	A/chicken/Delaware/22-005260-001/2022	A / H5N1	North America / United States / Deleware	Mary Lea Killian (National Veterinary Services Laboratories - USDA / Diagnostic Virology Laboratory)
EPI_ISL_11628223	A/chicken/Delaware/22-005260-002/2022	A / H5N1	North America / United States / Deleware	Mary Lea Killian (National Veterinary Services Laboratories - USDA / Diagnostic Virology Laboratory)
EPI_ISL_11628224	A/chicken/Delaware/22-005260-003/2022	A / H5N1	North America / United States / Deleware	Mary Lea Killian (National Veterinary Services Laboratories - USDA / Diagnostic Virology Laboratory)
EPI_ISL_11628225	A/turkey/Indiana/22-005289-001/2022	A / H5N1	North America / United States / Indiana / Greene	Mary Lea Killian (National Veterinary Services Laboratories - USDA / Diagnostic Virology Laboratory)
EPI_ISL_11628226	A/turkey/Indiana/22-005289-002/2022	A / H5N1	North America / United States / Indiana / Greene	Mary Lea Killian (National Veterinary Services Laboratories - USDA / Diagnostic Virology Laboratory)
EPI_ISL_11628227	A/turkey/Indiana/22-005328-001/2022	A / H5N1	North America / United States / Indiana / Greene	Mary Lea Killian (National Veterinary Services Laboratories - USDA / Diagnostic Virology Laboratory)
EPI_ISL_11628228	A/turkey/Indiana/22-005328-002/2022	A / H5N1	North America / United States / Indiana / Greene	Mary Lea Killian (National Veterinary Services Laboratories - USDA / Diagnostic Virology Laboratory)
EPI_ISL_11628229	A/turkey/Michigan/22-005440-001/2022	A / H5N1	North America / United States / Michigan / Kalamazoo	Mary Lea Killian (National Veterinary Services Laboratories - USDA / Diagnostic Virology Laboratory)
EPI_ISL_11628230	A/chicken/Michigan/22-005440-002/2022	A / H5N1	North America / United States / Michigan / Kalamazoo	Mary Lea Killian (National Veterinary Services Laboratories - USDA / Diagnostic Virology Laboratory)
EPI_ISL_11628231	A/guinea fowl/Michigan/22-005440-003/2022	A / H5N1	North America / United States / Michigan / Kalamazoo	Mary Lea Killian (National Veterinary Services Laboratories - USDA / Diagnostic Virology Laboratory)
EPI_ISL_11628232	A/chicken/Maine/22-005443-001/2022	A / H5N1	North America / United States / Maine / Knox	Mary Lea Killian (National Veterinary Services Laboratories - USDA / Diagnostic Virology Laboratory)
EPI_ISL_11628233	A/pheasant/New York/22-005647-001/2022	A / H5N1	North America / United States / New York / Suffolk	Mary Lea Killian (National Veterinary Services Laboratories - USDA / Diagnostic Virology Laboratory)
EPI_ISL_11628234	A/pheasant/New York/22-005647-002/2022	A / H5N1	North America / United States / New York / Suffolk	Mary Lea Killian (National Veterinary Services Laboratories - USDA / Diagnostic Virology Laboratory)
EPI_ISL_11628235	A/turkey/Indiana/22-005696-001/2022	A / H5N1	North America / United States / Indiana / Dubois	Mary Lea Killian (National Veterinary Services Laboratories - USDA / Diagnostic Virology Laboratory)
EPI_ISL_11628236	A/turkey/Indiana/22-005696-002/2022	A / H5N1	North America / United States / Indiana / Dubois	Mary Lea Killian (National Veterinary Services Laboratories - USDA / Diagnostic Virology Laboratory)
EPI_ISL_11628237	A/turkey/Indiana/22-005696-003/2022	A / H5N1	North America / United States / Indiana / Dubois	Mary Lea Killian (National Veterinary Services Laboratories - USDA / Diagnostic Virology Laboratory)
EPI_ISL_11628238	A/silkie chicken/Iowa/22-006114-001/2022	A / H5N1	North America / United States / Iowa / Pottawattamie	Mary Lea Killian (National Veterinary Services Laboratories - USDA / Diagnostic Virology Laboratory)
EPI_ISL_11628239	A/chicken/Connecticut/22-006118-001/2022	A / H5N1	North America / United States / Conneticut	Mary Lea Killian (National Veterinary Services Laboratories - USDA / Diagnostic Virology Laboratory)
EPI_ISL_11628240	A/turkey/Indiana/22-006259-001/2022	A / H5N1	North America / United States / Indiana / Dubois	Mary Lea Killian (National Veterinary Services Laboratories - USDA / Diagnostic Virology Laboratory)
EPI_ISL_11628241	A/turkey/Indiana/22-006259-002/2022	A / H5N1	North America / United States / Indiana / Dubois	Mary Lea Killian (National Veterinary Services Laboratories - USDA / Diagnostic Virology Laboratory)
EPI_ISL_11628242	A/turkey/Indiana/22-006261-001/2022	A / H5N1	North America / United States / Indiana / Dubois	Mary Lea Killian (National Veterinary Services Laboratories - USDA / Diagnostic Virology Laboratory)
EPI_ISL_11628243	A/turkey/Indiana/22-006261-002/2022	A / H5N1	North America / United States / Indiana / Dubois	Mary Lea Killian (National Veterinary Services Laboratories - USDA / Diagnostic Virology Laboratory)
EPI_ISL_11628244	A/chicken/Missouri/22-006569-001/2022	A / H5N1	North America / United States / Missouri / Stoddard	Mary Lea Killian (National Veterinary Services Laboratories - USDA / Diagnostic Virology Laboratory)
EPI_ISL_11628245	A/chicken/Missouri/22-006569-002/2022	A / H5N1	North America / United States / Missouri / Stoddard	Mary Lea Killian (National Veterinary Services Laboratories - USDA / Diagnostic Virology Laboratory)
EPI_ISL_11628246	A/chicken/Maryland/22-006578-001/2022	A / H5N1	North America / United States / Maryland / Cecil	Mary Lea Killian (National Veterinary Services Laboratories - USDA / Diagnostic Virology Laboratory)
EPI_ISL_11628247	A/chicken/Maryland/22-006578-002/2022	A / H5N1	North America / United States / Maryland / Cecil	Mary Lea Killian (National Veterinary Services Laboratories - USDA / Diagnostic Virology Laboratory)
EPI_ISL_11628248	A/chicken/Missouri/22-006639-001/2022	A / H5N1	North America / United States / Missouri / Bates	Mary Lea Killian (National Veterinary Services Laboratories - USDA / Diagnostic Virology Laboratory)
EPI_ISL_11628249	A/turkey/South Dakota/22-006792-001/2022	A / H5N1	North America / United States / South Dakota / Charles	Mary Lea Killian (National Veterinary Services Laboratories - USDA / Diagnostic Virology Laboratory)
EPI_ISL_11628250	A/turkey/South Dakota/22-006792-002/2022	A / H5N1	North America / United States / South Dakota / Charles Mix	Mary Lea Killian (National Veterinary Services Laboratories - USDA / Diagnostic Virology Laboratory)
EPI_ISL_11628251	A/turkey/Iowa/22-006795-001/2022	A / H5N1	North America / United States / Iowa / Buena Vista	Mary Lea Killian (National Veterinary Services Laboratories - USDA / Diagnostic Virology Laboratory)
EPI_ISL_11628252	A/turkey/Iowa/22-006795-002/2022	A / H5N1	North America / United States / Iowa / Buena Vista	Mary Lea Killian (National Veterinary Services Laboratories - USDA / Diagnostic Virology Laboratory)
EPI_ISL_11633613	A/Cygnus columbianus/Hubei/117/2021	A / H5N1	Asia / China / Hubei Province	Ma Liping (Wuhan Institute of Virology, CAS)
EPI_ISL_11633614	A/Cygnus columbianus/Hubei/121/2021	A / H5N1	Asia / China / Hubei Province	Ma Liping (Wuhan Institute of Virology, CAS)
EPI_ISL_11633616	A/Cygnus columbianus/Hubei/123/2021	A / H5N1	Asia / China / Hubei Province	Ma Liping (Wuhan Institute of Virology, CAS)
EPI_ISL_11633707	A/Cygnus columbianus/Hubei/127/2021	A / H5N1	Asia / China / Hubei Province	Ma Liping (Wuhan Institute of Virology, CAS)
EPI_ISL_11633708	A/Cygnus columbianus/Hubei/126/2021	A / H5N1	Asia / China / Hubei Province	Ma Liping (Wuhan Institute of Virology, CAS)
EPI_ISL_11633709	A/Cygnus columbianus/Hubei/128/2021	A / H5N1	Asia / China / Hubei Province	Ma Liping (Wuhan Institute of Virology, CAS)
EPI_ISL_11725869	A/domestic duck/Germany-BB/AI06239/2021	A / H5N1	Europe / Germany / Brandenburg / Spree-Neiße	Jacqueline King (Friedrich-Loeffler-Institut)
EPI_ISL_11725962	A/chicken/Germany-BB/AI06219/2021	A / H5N1	Europe / Germany / Brandenburg / Spree-Neiße	Jacqueline King (Friedrich-Loeffler-Institut)
EPI_ISL_11725988	A/chicken/Germany-BB/AI06242/2021	A / H5N1	Europe / Germany / Brandenburg / Spree-Neiße	Jacqueline King (Friedrich-Loeffler-Institut)
EPI_ISL_11725991	A/barnacle goose/Germany-MV/AI06175/2021	A / H5N1	Europe / Germany / Mecklenburg-Vorpommern / Vorpommern-Rügen	Jacqueline King (Friedrich-Loeffler-Institut)
EPI_ISL_11725994	A/buzzard/Germany-SH/AI07099/2021	A / H5N1	Europe / Germany / Schleswig-Holstein / Dithmarschen	Jacqueline King (Friedrich-Loeffler-Institut)
EPI_ISL_11798566	A/barnacle/goose_Denmark/19027-1.02/2021-04-18	A / H5N1	Europe / Denmark / Region Midtjylland / Viborg Kommune	Charlotte Kristiane Hjulsager (Statens Serum Institute / Microbiological Diagnostic and Virology)
EPI_ISL_11798568	A/barnacle_goose/Denmark/24273-1.02/2021-10-26	A / H5N1	Europe / Denmark / Region Sjaland / Guldborgsund	Charlotte Kristiane Hjulsager (Statens Serum Institute / Microbiological Diagnostic and Virology)
EPI_ISL_11798569	A/barnacle_goose/Denmark/24342-1.02/2021-10-30	A / H5N1	Europe / Denmark / Region Midtjylland / Arhus Kommune	Charlotte Kristiane Hjulsager (Statens Serum Institute / Microbiological Diagnostic and Virology)
EPI_ISL_11798570	A/chicken/Denmark/24357-11/2021-11-02	A / H5N1	Europe / Denmark / Region Sjaland / Slagelse Kommune	Charlotte Kristiane Hjulsager (Statens Serum Institute / Microbiological Diagnostic and Virology)
EPI_ISL_11798571	A/common_buzzard/Denmark/24271-1.02/2021-10-25	A / H5N1	Europe / Denmark / Region Sjaland / Slagelse Kommune	Charlotte Kristiane Hjulsager (Statens Serum Institute / Microbiological Diagnostic and Virology)
EPI_ISL_11798572	A/Eurasian_wigeon/Denmark/24066-9/2021-10-13	A / H5N1	Europe / Denmark / Region Syddanmark / Tonder Kommune	Charlotte Kristiane Hjulsager (Statens Serum Institute / Microbiological Diagnostic and Virology)
EPI_ISL_11798573	A/Eurasian_teal/Denmark/24115-2/2021-10-16	A / H5N1	Europe / Denmark / Region Syddanmark / Fano Kommune	Charlotte Kristiane Hjulsager (Statens Serum Institute / Microbiological Diagnostic and Virology)
EPI_ISL_11798574	A/Eurasian_wigeon/Denmark/24279-1/2021-10-24	A / H5N1	Europe / Denmark / Region Syddanmark / Fano Kommune	Charlotte Kristiane Hjulsager (Statens Serum Institute / Microbiological Diagnostic and Virology)

EPI_ISL_11798575	A/European_herring_gull/Denmark/19968-1.02/2021-05-14	A / H5N1	Europe / Denmark / Region Midtjylland / Hostebro Kommune	Charlotte Kristiane Hjulsager (Statens Serum Institute / Microbiological Diagnostic and Virology)
EPI_ISL_11798577	A/gray_heron/Denmark/24326-1.02/2021-10-28	A / H5N1	Europe / Denmark / Region Midtjylland / Hostebro Kommune	Charlotte Kristiane Hjulsager (Statens Serum Institute / Microbiological Diagnostic and Virology)
EPI_ISL_11798578	A/greylag_goose/Denmark/24309-1.01/2021-10-27	A / H5N1	Europe / Denmark / Region Syddanmark / Faaborg-Midtfyn	Charlotte Kristiane Hjulsager (Statens Serum Institute / Microbiological Diagnostic and Virology)
EPI_ISL_11798579	A/greylag_goose/Denmark/24343-1.02/2021-11-01	A / H5N1	Europe / Denmark / Region Nordjylland / Mariagerfjord	Charlotte Kristiane Hjulsager (Statens Serum Institute / Microbiological Diagnostic and Virology)
EPI_ISL_11798580	A/turkey/Denmark/24325-25/2021-10-30	A / H5N1	Europe / Denmark / Region Sjaland / Slagelse Kommune	Charlotte Kristiane Hjulsager (Statens Serum Institute / Microbiological Diagnostic and Virology)
EPI_ISL_1180234	A/mute swan/Czech Republic/1656-1/2021	A / H5N8	Europe / Czech Republic / Jihocesky Kraj / Okres Ceske Budejovice / Haklovy Dvory, Starohaklovs	Alexander Nagy (State Veterinary Institute Prague)
EPI_ISL_1184508	A/chicken/Miyazaki/L10T/2021	A / H5N8	Asia / Japan / Miyazaki	Takehiko Saito (National Institute of Animal Health)
EPI_ISL_1184509	A/chicken/Miyazaki/L1T/2021	A / H5N8	Asia / Japan / Miyazaki	Takehiko Saito (National Institute of Animal Health)
EPI_ISL_1184510	A/chicken/Miyazaki/L4T/2021	A / H5N8	Asia / Japan / Miyazaki	Takehiko Saito (National Institute of Animal Health)
EPI_ISL_1184511	A/chicken/Miyazaki/L9T/2021	A / H5N8	Asia / Japan / Miyazaki	Takehiko Saito (National Institute of Animal Health)
EPI_ISL_1184517	A/eastern buzzard/Toyama/160208C/2021	A / H5N8	Asia / Japan / Toyama	Takehiko Saito (National Institute of Animal Health)
EPI_ISL_1184518	A/eastern buzzard/Toyama/160208T/2021	A / H5N8	Asia / Japan / Toyama	Takehiko Saito (National Institute of Animal Health)
EPI_ISL_1185026	A/chicken/Astrakhan/2171-1/2020	A / H5N8	Europe / Russian Federation / Astrakhan Oblast	Nikolay Zinyakov (Federal Centre for Animal Health (ARRIAH) / OIE Regional Reference Laboratory)
EPI_ISL_11880371	A/Gallus_gallus/Belgium/4190_0002/2022	A / H5N1	Europe / Belgium / Provincie West-Vlaanderen / Meulebeke	Steven Van Borm (Sciensano, Department of Animal Infectious Diseases / Animal Infectious Diseases)
EPI_ISL_11897507	A/chicken/Maryland/22-006578-001-original/2022	A / H5N1	North America / United States / Maryland / Cecil	Mary Lea Killian (National Veterinary Services Laboratories - USDA / Diagnostic Virology Laboratory)
EPI_ISL_11897508	A/chicken/Maryland/22-006578-002-original/2022	A / H5N1	North America / United States / Maryland / Cecil	Mary Lea Killian (National Veterinary Services Laboratories - USDA / Diagnostic Virology Laboratory)
EPI_ISL_11897667	A/chicken/Missouri/22-006639-001-original/2022	A / H5N1	North America / United States / Missouri / Bates	Mary Lea Killian (National Veterinary Services Laboratories - USDA / Diagnostic Virology Laboratory)
EPI_ISL_11897668	A/turkey/South Dakota/22-006792-001-original/2022	A / H5N1	North America / United States / South Dakota / Charles Mix	Mary Lea Killian (National Veterinary Services Laboratories - USDA / Diagnostic Virology Laboratory)
EPI_ISL_11897669	A/turkey/South Dakota/22-006792-002-original/2022	A / H5N1	North America / United States / South Dakota / Charles Mix	Mary Lea Killian (National Veterinary Services Laboratories - USDA / Diagnostic Virology Laboratory)
EPI_ISL_11897670	A/turkey/Iowa/22-006795-001-original/2022	A / H5N1	North America / United States / Iowa / Buena Vista	Mary Lea Killian (National Veterinary Services Laboratories - USDA / Diagnostic Virology Laboratory)
EPI_ISL_11897671	A/turkey/Iowa/22-006795-002-original/2022	A / H5N1	North America / United States / Iowa / Buena Vista	Mary Lea Killian (National Veterinary Services Laboratories - USDA / Diagnostic Virology Laboratory)
EPI_ISL_11897672	A/turkey/Missouri/22-006944-002-original/2022	A / H5N1	North America / United States / Missouri / Jasper	Mary Lea Killian (National Veterinary Services Laboratories - USDA / Diagnostic Virology Laboratory)
EPI_ISL_11897673	A/chicken/Delaware/22-006945-001-original/2022	A / H5N1	North America / United States / Delaware / New Castle	Mary Lea Killian (National Veterinary Services Laboratories - USDA / Diagnostic Virology Laboratory)
EPI_ISL_11897674	A/chicken/Delaware/22-006945-002-original/2022	A / H5N1	North America / United States / Delaware / New Castle	Mary Lea Killian (National Veterinary Services Laboratories - USDA / Diagnostic Virology Laboratory)
EPI_ISL_11897675	A/chicken/Maryland/22-006948-001-original/2022	A / H5N1	North America / United States / Maryland / Queen Annes	Mary Lea Killian (National Veterinary Services Laboratories - USDA / Diagnostic Virology Laboratory)
EPI_ISL_11897676	A/chicken/Maryland/22-007086-001-original/2022	A / H5N1	North America / United States / Maryland / Queen Annes	Mary Lea Killian (National Veterinary Services Laboratories - USDA / Diagnostic Virology Laboratory)
EPI_ISL_11897677	A/turkey/Missouri/22-007087-001-original/2022	A / H5N1	North America / United States / Missouri / Lawrence	Mary Lea Killian (National Veterinary Services Laboratories - USDA / Diagnostic Virology Laboratory)
EPI_ISL_11897678	A/turkey/Missouri/22-007087-002-original/2022	A / H5N1	North America / United States / Missouri / Lawrence	Mary Lea Killian (National Veterinary Services Laboratories - USDA / Diagnostic Virology Laboratory)
EPI_ISL_11897679	A/chicken/Maryland/22-007273-001-original/2022	A / H5N1	North America / United States / Maryland / Cecil	Mary Lea Killian (National Veterinary Services Laboratories - USDA / Diagnostic Virology Laboratory)
EPI_ISL_11897680	A/chicken/Maryland/22-007273-002-original/2022	A / H5N1	North America / United States / Maryland / Cecil	Mary Lea Killian (National Veterinary Services Laboratories - USDA / Diagnostic Virology Laboratory)
EPI_ISL_11897681	A/chicken/Iowa/22-007376-001-original/2022	A / H5N1	North America / United States / Iowa / Taylor	Mary Lea Killian (National Veterinary Services Laboratories - USDA / Diagnostic Virology Laboratory)
EPI_ISL_11897682	A/chicken/Iowa/22-007376-002-original/2022	A / H5N1	North America / United States / Iowa / Taylor	Mary Lea Killian (National Veterinary Services Laboratories - USDA / Diagnostic Virology Laboratory)
EPI_ISL_11897683	A/guinea fowl/Illinois/22-007382-001-original/2022	A / H5N1	North America / United States / Illinois / Maclean	Mary Lea Killian (National Veterinary Services Laboratories - USDA / Diagnostic Virology Laboratory)
EPI_ISL_11897684	A/poultry/Kansas/22-007391-001-original/2022	A / H5N1	North America / United States / Kansas / Franklin	Mary Lea Killian (National Veterinary Services Laboratories - USDA / Diagnostic Virology Laboratory)
EPI_ISL_11897685	A/poultry/Kansas/22-007391-002-original/2022	A / H5N1	North America / United States / Kansas / Franklin	Mary Lea Killian (National Veterinary Services Laboratories - USDA / Diagnostic Virology Laboratory)
EPI_ISL_11897686	A/turkey/Maine/22-007410-001-original/2022	A / H5N1	North America / United States / Maine / Lincoln	Mary Lea Killian (National Veterinary Services Laboratories - USDA / Diagnostic Virology Laboratory)
EPI_ISL_11897687	A/chicken/Maine/22-007410-002-original/2022	A / H5N1	North America / United States / Maine / Lincoln	Mary Lea Killian (National Veterinary Services Laboratories - USDA / Diagnostic Virology Laboratory)
EPI_ISL_11897688	A/turkey/South Dakota/22-007534-001-original/2022	A / H5N1	North America / United States / South Dakota / Charles Mix	Mary Lea Killian (National Veterinary Services Laboratories - USDA / Diagnostic Virology Laboratory)
EPI_ISL_11897689	A/turkey/South Dakota/22-007534-002-original/2022	A / H5N1	North America / United States / South Dakota / Charles Mix	Mary Lea Killian (National Veterinary Services Laboratories - USDA / Diagnostic Virology Laboratory)
EPI_ISL_11897690	A/chicken/Wisconsin/22-007545-001-original/2022	A / H5N1	North America / United States / Wisconsin / Jefferson	Mary Lea Killian (National Veterinary Services Laboratories - USDA / Diagnostic Virology Laboratory)
EPI_ISL_11897691	A/chicken/Wisconsin/22-007545-002-original/2022	A / H5N1	North America / United States / Wisconsin / Jefferson	Mary Lea Killian (National Veterinary Services Laboratories - USDA / Diagnostic Virology Laboratory)
EPI_ISL_11897692	A/chicken/Maine/22-007582-001-original/2022	A / H5N1	North America / United States / Maine / York	Mary Lea Killian (National Veterinary Services Laboratories - USDA / Diagnostic Virology Laboratory)
EPI_ISL_11897693	A/chicken/Missouri/22-007677-001-original/2022	A / H5N1	North America / United States / Missouri / New London	Mary Lea Killian (National Veterinary Services Laboratories - USDA / Diagnostic Virology Laboratory)
EPI_ISL_11897694	A/chicken/Missouri/22-007677-002-original/2022	A / H5N1	North America / United States / Missouri / New London	Mary Lea Killian (National Veterinary Services Laboratories - USDA / Diagnostic Virology Laboratory)
EPI_ISL_11897695	A/chicken/Nebraska/22-007805-001-original/2022	A / H5N1	North America / United States / Nebraska / Merrick	Mary Lea Killian (National Veterinary Services Laboratories - USDA / Diagnostic Virology Laboratory)
EPI_ISL_11897696	A/waterfowl/Nebraska/22-007805-002-original/2022	A / H5N1	North America / United States / Nebraska / Merrick	Mary Lea Killian (National Veterinary Services Laboratories - USDA / Diagnostic Virology Laboratory)
EPI_ISL_11897697	A/turkey/New Hampshire/22-007886-001-original/2022	A / H5N1	North America / United States / New Hampshire / Rockingham	Mary Lea Killian (National Veterinary Services Laboratories - USDA / Diagnostic Virology Laboratory)
EPI_ISL_11897698	A/chicken/Delaware/22-008054-002-original/2022	A / H5N1	North America / United States / Delaware / Kent	Mary Lea Killian (National Veterinary Services Laboratories - USDA / Diagnostic Virology Laboratory)
EPI_ISL_11897699	A/chicken/Delaware/22-008054-003-original/2022	A / H5N1	North America / United States / Delaware / Kent	Mary Lea Killian (National Veterinary Services Laboratories - USDA / Diagnostic Virology Laboratory)
EPI_ISL_11897700	A/guinea fowl/Maine/22-008064-001-original/2022	A / H5N1	North America / United States / Maine / Lincoln	Mary Lea Killian (National Veterinary Services Laboratories - USDA / Diagnostic Virology Laboratory)
EPI_ISL_11897701	A/chicken/Maine/22-008064-002-original/2022	A / H5N1	North America / United States / Maine / Lincoln	Mary Lea Killian (National Veterinary Services Laboratories - USDA / Diagnostic Virology Laboratory)
EPI_ISL_11897702	A/duck/Kansas/22-008114-001-original/2022	A / H5N1	North America / United States / Kansas / Sedgewick	Mary Lea Killian (National Veterinary Services Laboratories - USDA / Diagnostic Virology Laboratory)
EPI_ISL_11897703	A/chicken/Kansas/22-008114-002-original/2022	A / H5N1	North America / United States / Kansas / Sedgewick	Mary Lea Killian (National Veterinary Services Laboratories - USDA / Diagnostic Virology Laboratory)
EPI_ISL_11897704	A/chicken/Iowa/22-008176-003-original/2022	A / H5N1	North America / United States / Iowa / Buena Vista	Mary Lea Killian (National Veterinary Services Laboratories - USDA / Diagnostic Virology Laboratory)
EPI_ISL_1191587	A/chicken/Czech Republic/1566-2/2021	A / H5N8	Europe / Czech Republic / Jihocesky Kraj / Okres Tabor / Dlouha Lhota/Chynov; GPS 49.3561703	Alexander Nagy (State Veterinary Institute Prague)
EPI_ISL_11922807	A/chicken/Poland/H182_22VIR2515-1/2022	A / H5N2	Europe / Poland	Giacomo Barbierato (Istituto Zooprofilattico Sperimentale delle Venezie / Ricerca e innovazione)
EPI_ISL_11922808	A/duck/Poland/H188_22VIR2515-2/2022	A / H5N1	Europe / Poland	Giacomo Barbierato (Istituto Zooprofilattico Sperimentale delle Venezie / Ricerca e innovazione)
EPI_ISL_11922809	A/chicken/Poland/H157_22VIR2515-3/2022	A / H5N1	Europe / Poland	Giacomo Barbierato (Istituto Zooprofilattico Sperimentale delle Venezie / Ricerca e innovazione)
EPI_ISL_11922810	A/duck/Poland/H126_22VIR2515-4/2022	A / H5N1	Europe / Poland	Giacomo Barbierato (Istituto Zooprofilattico Sperimentale delle Venezie / Ricerca e innovazione)
EPI_ISL_11922811	A/goose/Poland/H124_22VIR2515-5/2022	A / H5N1	Europe / Poland	Giacomo Barbierato (Istituto Zooprofilattico Sperimentale delle Venezie / Ricerca e innovazione)
EPI_ISL_11922812	A/chicken/Poland/H071_22VIR2515-6/2022	A / H5N1	Europe / Poland	Giacomo Barbierato (Istituto Zooprofilattico Sperimentale delle Venezie / Ricerca e innovazione)
EPI_ISL_11922813	A/swan/Poland/MB078_22VIR2515-7/2022	A / H5N1	Europe / Poland	Giacomo Barbierato (Istituto Zooprofilattico Sperimentale delle Venezie / Ricerca e innovazione)
EPI_ISL_11922814	A/swan/Poland/MB083_22VIR2515-8/2022	A / H5N1	Europe / Poland	Giacomo Barbierato (Istituto Zooprofilattico Sperimentale delle Venezie / Ricerca e innovazione)
EPI_ISL_11922815	A/swan/Romania/16905_22VIR2749-1/2021	A / H5N1	Europe / Romania	Giacomo Barbierato (Istituto Zooprofilattico Sperimentale delle Venezie / Ricerca e innovazione)
EPI_ISL_11922816	A/swan/Romania/10324_22VIR2749-2/2022	A / H5N1	Europe / Romania	Giacomo Barbierato (Istituto Zooprofilattico Sperimentale delle Venezie / Ricerca e innovazione)
EPI_ISL_11922817	A/swan/Romania/10394_22VIR2749-3/2022	A / H5N1	Europe / Romania	Giacomo Barbierato (Istituto Zooprofilattico Sperimentale delle Venezie / Ricerca e innovazione)
EPI_ISL_11922818	A/swan/Romania/10455_22VIR2749-4/2022	A / H5N1	Europe / Romania	Giacomo Barbierato (Istituto Zooprofilattico Sperimentale delle Venezie / Ricerca e innovazione)
EPI_ISL_11922819	A/laying_hen/Romania/10470_22VIR2749-5/2022	A / H5N1	Europe / Romania	Giacomo Barbierato (Istituto Zooprofilattico Sperimentale delle Venezie / Ricerca e innovazione)
EPI_ISL_11922820	A/swan/Romania/10656_22VIR2749-6/2022	A / H5N1	Europe / Romania	Giacomo Barbierato (Istituto Zooprofilattico Sperimentale delle Venezie / Ricerca e innovazione)
EPI_ISL_11922821	A/swan/Romania/10678_22VIR2749-7/2022	A / H5N1	Europe / Romania	Giacomo Barbierato (Istituto Zooprofilattico Sperimentale delle Venezie / Ricerca e innovazione)
EPI_ISL_11922822	A/swan/Romania/10986_22VIR2749-8/2022	A / H5N1	Europe / Romania	Giacomo Barbierato (Istituto Zooprofilattico Sperimentale delle Venezie / Ricerca e innovazione)
EPI_ISL_11971464	A/turkey/South Dakota/22-008239-001-original/2022	A / H5N1	North America / United States / South Dakota / Kingsbury	Mary Lea Killian (National Veterinary Services Laboratories - USDA / Diagnostic Virology Laboratory)

EPI_ISL_11971465	A/turkey/South Dakota/22-008239-002-original/2022	A / H5N1	North America / United States / South Dakota / Kingsbury	Mary Lea Killian (National Veterinary Services Laboratories - USDA / Diagnostic Virology Laboratory)
EPI_ISL_11971466	A/turkey/South Dakota/22-008242-001-original/2022	A / H5N1	North America / United States / South Dakota / Hanson	Mary Lea Killian (National Veterinary Services Laboratories - USDA / Diagnostic Virology Laboratory)
EPI_ISL_11971467	A/turkey/South Dakota/22-008242-002-original/2022	A / H5N1	North America / United States / South Dakota / Hanson	Mary Lea Killian (National Veterinary Services Laboratories - USDA / Diagnostic Virology Laboratory)
EPI_ISL_11971468	A/chicken/Kansas/22-008259-001-original/2022	A / H5N1	North America / United States / Kansas / Dickinson	Mary Lea Killian (National Veterinary Services Laboratories - USDA / Diagnostic Virology Laboratory)
EPI_ISL_11971469	A/chicken/Kansas/22-008259-002-original/2022	A / H5N1	North America / United States / Kansas / Dickinson	Mary Lea Killian (National Veterinary Services Laboratories - USDA / Diagnostic Virology Laboratory)
EPI_ISL_11971470	A/chicken/Maine/22-008366-001-original/2022	A / H5N1	North America / United States / Maine / Knox	Mary Lea Killian (National Veterinary Services Laboratories - USDA / Diagnostic Virology Laboratory)
EPI_ISL_11971471	A/chicken/Maine/22-008367-001-original/2022	A / H5N1	North America / United States / Maine / York	Mary Lea Killian (National Veterinary Services Laboratories - USDA / Diagnostic Virology Laboratory)
EPI_ISL_11971472	A/turkey/Maine/22-008367-002-original/2022	A / H5N1	North America / United States / Maine / York	Mary Lea Killian (National Veterinary Services Laboratories - USDA / Diagnostic Virology Laboratory)
EPI_ISL_11971475	A/chicken/Iowa/22-008373-001-original/2022	A / H5N1	North America / United States / Iowa / Warren	Mary Lea Killian (National Veterinary Services Laboratories - USDA / Diagnostic Virology Laboratory)
EPI_ISL_11971476	A/chicken/Iowa/22-008373-002-original/2022	A / H5N1	North America / United States / Iowa / Warren	Mary Lea Killian (National Veterinary Services Laboratories - USDA / Diagnostic Virology Laboratory)
EPI_ISL_11971477	A/chicken/Nebraska/22-008467-001-original/2022	A / H5N1	North America / United States / Nebraska / Butler	Mary Lea Killian (National Veterinary Services Laboratories - USDA / Diagnostic Virology Laboratory)
EPI_ISL_11971478	A/turkey/South Dakota/22-008479-001-original/2022	A / H5N1	North America / United States / South Dakota / Charles Mix	Mary Lea Killian (National Veterinary Services Laboratories - USDA / Diagnostic Virology Laboratory)
EPI_ISL_11971479	A/turkey/South Dakota/22-008479-002-original/2022	A / H5N1	North America / United States / South Dakota / Charles Mix	Mary Lea Killian (National Veterinary Services Laboratories - USDA / Diagnostic Virology Laboratory)
EPI_ISL_11971480	A/turkey/South Dakota/22-008483-001-original/2022	A / H5N1	North America / United States / South Dakota / Beadle	Mary Lea Killian (National Veterinary Services Laboratories - USDA / Diagnostic Virology Laboratory)
EPI_ISL_11971481	A/turkey/South Dakota/22-008485-001-original/2022	A / H5N1	North America / United States / South Dakota / Hanson	Mary Lea Killian (National Veterinary Services Laboratories - USDA / Diagnostic Virology Laboratory)
EPI_ISL_11971482	A/turkey/South Dakota/22-008485-002-original/2022	A / H5N1	North America / United States / South Dakota / Hanson	Mary Lea Killian (National Veterinary Services Laboratories - USDA / Diagnostic Virology Laboratory)
EPI_ISL_11971483	A/chicken/Maine/22-008540-001-original/2022	A / H5N1	North America / United States / Maine / Cumberland	Mary Lea Killian (National Veterinary Services Laboratories - USDA / Diagnostic Virology Laboratory)
EPI_ISL_11971484	A/turkey/South Dakota/22-008702-001-original/2022	A / H5N1	North America / United States / South Dakota / Edmonds	Mary Lea Killian (National Veterinary Services Laboratories - USDA / Diagnostic Virology Laboratory)
EPI_ISL_11971485	A/turkey/South Dakota/22-008702-002-original/2022	A / H5N1	North America / United States / South Dakota / Edmonds	Mary Lea Killian (National Veterinary Services Laboratories - USDA / Diagnostic Virology Laboratory)
EPI_ISL_11971486	A/chicken/South Dakota/22-008704-001-original/2022	A / H5N1	North America / United States / South Dakota / Kingsbury	Mary Lea Killian (National Veterinary Services Laboratories - USDA / Diagnostic Virology Laboratory)
EPI_ISL_11971487	A/chicken/South Dakota/22-008704-002-original/2022	A / H5N1	North America / United States / South Dakota / Kingsbury	Mary Lea Killian (National Veterinary Services Laboratories - USDA / Diagnostic Virology Laboratory)
EPI_ISL_11971488	A/turkey/South Dakota/22-008705-001-original/2022	A / H5N1	North America / United States / South Dakota / Beadle	Mary Lea Killian (National Veterinary Services Laboratories - USDA / Diagnostic Virology Laboratory)
EPI_ISL_11971489	A/turkey/South Dakota/22-008705-002-original/2022	A / H5N1	North America / United States / South Dakota / Beadle	Mary Lea Killian (National Veterinary Services Laboratories - USDA / Diagnostic Virology Laboratory)
EPI_ISL_11971490	A/pheasant/New York/22-008760-008-original/2022	A / H5N1	North America / United States / New York / Suffolk	Mary Lea Killian (National Veterinary Services Laboratories - USDA / Diagnostic Virology Laboratory)
EPI_ISL_11971491	A/mallard/New York/22-008760-007-original/2022	A / H5N1	North America / United States / New York / Suffolk	Mary Lea Killian (National Veterinary Services Laboratories - USDA / Diagnostic Virology Laboratory)
EPI_ISL_11971492	A/turkey/Iowa/22-008862-002-original/2022	A / H5N1	North America / United States / Iowa / Buena Vista	Mary Lea Killian (National Veterinary Services Laboratories - USDA / Diagnostic Virology Laboratory)
EPI_ISL_11971493	A/turkey/South Dakota/22-008866-001-original/2022	A / H5N1	North America / United States / South Dakota / Jerauld	Mary Lea Killian (National Veterinary Services Laboratories - USDA / Diagnostic Virology Laboratory)
EPI_ISL_11971494	A/turkey/South Dakota/22-008866-002-original/2022	A / H5N1	North America / United States / South Dakota / Jerauld	Mary Lea Killian (National Veterinary Services Laboratories - USDA / Diagnostic Virology Laboratory)
EPI_ISL_11971495	A/domestic duck/Michigan/22-008890-001-original/2022	A / H5N1	North America / United States / Michigan / Macomb	Mary Lea Killian (National Veterinary Services Laboratories - USDA / Diagnostic Virology Laboratory)
EPI_ISL_11971496	A/domestic goose/Michigan/22-008890-004-original/2022	A / H5N1	North America / United States / Michigan / Macomb	Mary Lea Killian (National Veterinary Services Laboratories - USDA / Diagnostic Virology Laboratory)
EPI_ISL_11971497	A/chicken/Michigan/22-008890-006-original/2022	A / H5N1	North America / United States / Michigan / Macomb	Mary Lea Killian (National Veterinary Services Laboratories - USDA / Diagnostic Virology Laboratory)
EPI_ISL_11971498	A/turkey/Michigan/22-008890-007-original/2022	A / H5N1	North America / United States / Michigan / Macomb	Mary Lea Killian (National Veterinary Services Laboratories - USDA / Diagnostic Virology Laboratory)
EPI_ISL_11971499	A/chicken/Nebraska/22-009006-001-original/2022	A / H5N1	North America / United States / Nebraska / Butler	Mary Lea Killian (National Veterinary Services Laboratories - USDA / Diagnostic Virology Laboratory)
EPI_ISL_11971500	A/turkey/South Dakota/22-009023-001-original/2022	A / H5N1	North America / United States / South Dakota / Hutchison	Mary Lea Killian (National Veterinary Services Laboratories - USDA / Diagnostic Virology Laboratory)
EPI_ISL_11971501	A/turkey/South Dakota/22-009023-002-original/2022	A / H5N1	North America / United States / South Dakota / Hutchison	Mary Lea Killian (National Veterinary Services Laboratories - USDA / Diagnostic Virology Laboratory)
EPI_ISL_11971502	A/pheasant/New York/22-009066-001-original/2022	A / H5N1	North America / United States / New York / Suffolk	Mary Lea Killian (National Veterinary Services Laboratories - USDA / Diagnostic Virology Laboratory)
EPI_ISL_12028893	A/turkey/Italy/21VIR11507/2021	A / H5N1	Europe / Italy	Giacomo Barbierato (Istituto Zooprofilattico Sperimentale delle Venezie / Ricerca e innovazione)
EPI_ISL_12045319	A/chicken/Botswana/2163-B/2021	A / H5N1	Africa / Botswana	Alex Byrne (Animal and Plant Health Agency (APHA) / Virology Department)
EPI_ISL_12045320	A/chicken/Botswana/2248-A/2021	A / H5N1	Africa / Botswana	Alex Byrne (Animal and Plant Health Agency (APHA) / Virology Department)
EPI_ISL_12045918	A/Dove/Botswana/2097/2021	A / H5N1	Africa / Botswana	Alex Byrne (Animal and Plant Health Agency (APHA) / Virology Department)
EPI_ISL_12045919	A/Dove/Botswana/2334/2021	A / H5N1	Africa / Botswana	Alex Byrne (Animal and Plant Health Agency (APHA) / Virology Department)
EPI_ISL_12045920	A/fish_eagle/Botswana/1338/2021	A / H5N1	Africa / Botswana	Alex Byrne (Animal and Plant Health Agency (APHA) / Virology Department)
EPI_ISL_1205485	A/red knot/Germany-SH/AI03419/2020	A / H5N3	Europe / Germany / Schleswig-Holstein / Nordfriesland	Jacqueline King (Friedrich-Loeffler-Institut)
EPI_ISL_1205487	A/red knot/Germany-SH/AI03421/2020	A / H5N3	Europe / Germany / Schleswig-Holstein / Nordfriesland	Jacqueline King (Friedrich-Loeffler-Institut)
EPI_ISL_1205489	A/red knot/Germany-SH/AI03424/2020	A / H5N3	Europe / Germany / Schleswig-Holstein / Nordfriesland	Jacqueline King (Friedrich-Loeffler-Institut)
EPI_ISL_1206285	A/red knot/Germany-NI/AI00394/2020	A / H5N3	Europe / Germany / Lower Saxony / Aurich	Jacqueline King (Friedrich-Loeffler-Institut)
EPI_ISL_12066188	A/Fox/Netherlands/EMC1/2022	A / H5N1	Europe / Netherlands / Huissen	Oanh Vuong-Zoet (Erasmus Medical Center / Department of Virology)
EPI_ISL_12069288	A/Fox/Netherlands/EMC2/2022	A / H5N1	Europe / Netherlands / Oirschot	Oanh Vuong-Zoet (Erasmus Medical Center / Department of Virology)
EPI_ISL_12069289	A/Fox/Netherlands/EMC3/2022	A / H5N1	Europe / Netherlands / Almere	Oanh Vuong-Zoet (Erasmus Medical Center / Department of Virology)
EPI_ISL_12069978	A/greylag goose/Netherlands/22006190-002/2022	A / H5N1	Europe / Netherlands / Provincie Gelderland	Rene Heutink (Wageningen Bioveterinary Research)
EPI_ISL_12069979	A/black-backed gull/Netherlands/22006192-001/2022	A / H5N1	Europe / Netherlands / Provincie Gelderland	Rene Heutink (Wageningen Bioveterinary Research)
EPI_ISL_12140437	A/duck/Czech Republic/913/2022	A / H5N1	Europe / Czech Republic / Jihocesky Kraj / Okres Strakonice / Kliovice; ZipCode:38601	Alexander Nagy (State Veterinary Institute Prague)
EPI_ISL_12150657	A/goose/Czech Republic/913/2022	A / H5N1	Europe / Czech Republic / Jihocesky Kraj / Okres Strakonice / Kliovice; ZipCode:38601	Alexander Nagy (State Veterinary Institute Prague)
EPI_ISL_12150658	A/chicken/Czech Republic/913/2022	A / H5N1	Europe / Czech Republic / Jihocesky Kraj / Okres Strakonice / Kliovice; ZipCode:38601	Alexander Nagy (State Veterinary Institute Prague)
EPI_ISL_12150661	A/mute swan/Czech Republic/2755/2022	A / H5N1	Europe / Czech Republic / Zlinsky Kraj / Okres Zlin / Spytihnev Zip Code: 76364	Alexander Nagy (State Veterinary Institute Prague)
EPI_ISL_12150664	A/goose/Czech Republic/25322-179/2021	A / H5N1	Europe / Czech Republic / Jihocesky Kraj / Okres Ceske Budejovice / Nove Hrady, Zip Code: 3740[illegible]	Alexander Nagy (State Veterinary Institute Prague)
EPI_ISL_12150666	A/goose/Czech Republic/25322-205/2021	A / H5N1	Europe / Czech Republic / Jihocesky Kraj / Okres Ceske Budejovice / Nove Hrady, Zip Code: 3740[illegible]	Alexander Nagy (State Veterinary Institute Prague)
EPI_ISL_12174842	A/Ezo red fox/Hokkaido/1/2022	A / H5N1	Asia / Japan / Hokkaido / Sapporo city	Norikazu Isoda (Graduate School of Veterinary Medicine, Hokkaido University / Laboratory of Microbiology)
EPI_ISL_12174843	A/crow/Hokkaido/0103B065/2022	A / H5N1	Asia / Japan / Hokkaido / Sapporo city	Norikazu Isoda (Graduate School of Veterinary Medicine, Hokkaido University / Laboratory of Microbiology)
EPI_ISL_12176837	A/pelecanus_crispus/Albania/D383-22_22VIR3125-2/2022	A / H5N1	Europe / Albania	Giacomo Barbierato (Istituto Zooprofilattico Sperimentale delle Venezie / Ricerca e innovazione)
EPI_ISL_12176838	A/chicken/Kosovo/278_22VIR3124-1/2021	A / H5N8	Europe / Kosovo	Giacomo Barbierato (Istituto Zooprofilattico Sperimentale delle Venezie / Ricerca e innovazione)
EPI_ISL_12176839	A/chicken/Kosovo/279_22VIR3124-2/2021	A / H5N8	Europe / Kosovo	Giacomo Barbierato (Istituto Zooprofilattico Sperimentale delle Venezie / Ricerca e innovazione)
EPI_ISL_12176840	A/chicken/Kosovo/280_22VIR3124-3/2021	A / H5N8	Europe / Kosovo	Giacomo Barbierato (Istituto Zooprofilattico Sperimentale delle Venezie / Ricerca e innovazione)
EPI_ISL_12176841	A/chicken/Kosovo/283_22VIR3124-4/2021	A / H5N8	Europe / Kosovo	Giacomo Barbierato (Istituto Zooprofilattico Sperimentale delle Venezie / Ricerca e innovazione)
EPI_ISL_12176843	A/chicken/Kosovo/284_22VIR3124-5/2021	A / H5N8	Europe / Kosovo	Giacomo Barbierato (Istituto Zooprofilattico Sperimentale delle Venezie / Ricerca e innovazione)
EPI_ISL_12176844	A/common_quail/Kosovo/288_22VIR3124-6/2021	A / H5N8	Europe / Kosovo	Giacomo Barbierato (Istituto Zooprofilattico Sperimentale delle Venezie / Ricerca e innovazione)
EPI_ISL_12176845	A/chicken/Kosovo/290_22VIR3124-7/2021	A / H5N8	Europe / Kosovo	Giacomo Barbierato (Istituto Zooprofilattico Sperimentale delle Venezie / Ricerca e innovazione)
EPI_ISL_12176846	A/chicken/Kosovo/299_22VIR3124-8/2021	A / H5N8	Europe / Kosovo	Giacomo Barbierato (Istituto Zooprofilattico Sperimentale delle Venezie / Ricerca e innovazione)
EPI_ISL_12176847	A/chicken/Kosovo/302_22VIR3124-9/2021	A / H5N8	Europe / Kosovo	Giacomo Barbierato (Istituto Zooprofilattico Sperimentale delle Venezie / Ricerca e innovazione)
EPI_ISL_12176848	A/chicken/Kosovo/303_22VIR3124-10/2021	A / H5N8	Europe / Kosovo	Giacomo Barbierato (Istituto Zooprofilattico Sperimentale delle Venezie / Ricerca e innovazione)
EPI_ISL_12176849	A/common_quail/Kosovo/304-2_22VIR3124-12/2021	A / H5N8	Europe / Kosovo	Giacomo Barbierato (Istituto Zooprofilattico Sperimentale delle Venezie / Ricerca e innovazione)
EPI_ISL_12176850	A/chicken/Kosovo/22-2_22VIR3124-13/2022	A / H5N8	Europe / Kosovo	Giacomo Barbierato (Istituto Zooprofilattico Sperimentale delle Venezie / Ricerca e innovazione)

EPI_ISL_12176851	A/chicken/Kosovo/22-8_22VIR3124-14/2022	A / H5N8	Europe / Kosovo	Giacomo Barbierato (Istituto Zooprofilattico Sperimentale delle Venezie / Ricerca e innovazione)
EPI_ISL_12176852	A/chicken/Kosovo/22-9_22VIR3124-15/2022	A / H5N8	Europe / Kosovo	Giacomo Barbierato (Istituto Zooprofilattico Sperimentale delle Venezie / Ricerca e innovazione)
EPI_ISL_12176853	A/chicken/Kosovo/126_22VIR3124-18/2021	A / H5N8	Europe / Kosovo	Giacomo Barbierato (Istituto Zooprofilattico Sperimentale delle Venezie / Ricerca e innovazione)
EPI_ISL_12176854	A/chicken/Kosovo/22-50_22VIR3124-19/2022	A / H5N8	Europe / Kosovo	Giacomo Barbierato (Istituto Zooprofilattico Sperimentale delle Venezie / Ricerca e innovazione)
EPI_ISL_12176855	A/chicken/Kosovo/22-59_22VIR3124-20/2022	A / H5N8	Europe / Kosovo	Giacomo Barbierato (Istituto Zooprofilattico Sperimentale delle Venezie / Ricerca e innovazione)
EPI_ISL_12176931	A/turkey/Kosovo/13-2_22VIR3124-31/2022	A / H5N8	Europe / Kosovo	Giacomo Barbierato (Istituto Zooprofilattico Sperimentale delle Venezie / Ricerca e innovazione)
EPI_ISL_12176932	A/chicken/Albania/D381-22_22VIR3125-1/2022	A / H5N8	Europe / Albania	Giacomo Barbierato (Istituto Zooprofilattico Sperimentale delle Venezie / Ricerca e innovazione)
EPI_ISL_12177629	A/black-backed gull/Netherlands/22006711-001/2022	A / H5N1	Europe / Netherlands / Provincie Gelderland	Rene Heutink (Wageningen Bioveterinary Research)
EPI_ISL_1219038	A/Chicken/Sweden/SVA210226SZ0168/KN048380-IP9/2021	A / H5N5	Europe / Sweden / Skane Lan / Lunds Kommun	Siamak Zohari (National Veterinary Institute)
EPI_ISL_1220094	A/tundra_bean_goose/Poland/MB132/2020	A / H5N8	Europe / Poland / West Pomeranian Voivodeship	Edyta ?wi?to? (National Veterinary Research Institut Poland, PIWet-PIB)
EPI_ISL_12210335	A/Falco_peregrinus/Belgium/4055_0002/2022	A / H5N1	Europe / Belgium / Provincie Antwerpen / Essen	Steven Van Borm (Sciensano, Department of Animal Infectious Diseases / Animal Infectious Diseases)
EPI_ISL_12215416	A/greylag goose/Netherlands/22005844-002/2022	A / H5N1	Europe / Netherlands / South Holland	Rene Heutink (Wageningen Bioveterinary Research)
EPI_ISL_12223688	A/grey heron/Czech Republic/25338-1/2021	A / H5N1	Europe / Czech Republic / Liberecky Kraj / Okres Ceska Lipa / Zahradky, GPS: 50.6372833N, 14.5	Alexander Nagy (State Veterinary Institute Prague)
EPI_ISL_12223734	A/grey heron/Czech Republic/25338-2/2021	A / H5N1	Europe / Czech Republic / Liberecky Kraj / Okres Ceska Lipa / Zahradky, GPS: 50.6372833N, 14.5	Alexander Nagy (State Veterinary Institute Prague)
EPI_ISL_1224942	A/peacock/Netherlands/21022591-002/2021	A / H5N8	Europe / Netherlands / Provincie Noord-Holland / Gemeente Zaanstad	Rene Heutink (Wageningen Bioveterinary Research)
EPI_ISL_1224946	A/barnacle goose/Netherlands/21022611-001/2021	A / H5N1	Europe / Netherlands / Provincie Friesland / Paesens	Rene Heutink (Wageningen Bioveterinary Research)
EPI_ISL_1224949	A/mute swan/Netherlands/21022898-002/2021	A / H5N8	Europe / Netherlands / South Holland / Gemeente Hellevoetsluis	Rene Heutink (Wageningen Bioveterinary Research)
EPI_ISL_1224989	A/common buzzard/Netherlands/21022834-002/2021	A / H5N1	Europe / Netherlands / Provincie Friesland / Gemeente Weststellingwerf	Rene Heutink (Wageningen Bioveterinary Research)
EPI_ISL_1225077	A/european herring gull/Netherlands/21023937-002/2021	A / H5N4	Europe / Netherlands / South Holland / Midden-Delfland	Rene Heutink (Wageningen Bioveterinary Research)
EPI_ISL_1225079	A/eurasian curlew/Netherlands/21024069-002/2021	A / H5N4	Europe / Netherlands / Provincie Friesland / Gemeente Harlingen	Rene Heutink (Wageningen Bioveterinary Research)
EPI_ISL_12324302	A/duck/Czech Republic/3306-1/2022	A / H5N1	Europe / Czech Republic / Stredocesky Kraj / Okres Kolin / Masojey, GPS: 50.0259761N, 14.7778	Alexander Nagy (State Veterinary Institute Prague)
EPI_ISL_12325210	A/chicken/Czech Republic/3306-2/2022	A / H5N1	Europe / Czech Republic / Stredocesky Kraj / Okres Kolin / Masojey, GPS: 50.0259761N, 14.7778	Alexander Nagy (State Veterinary Institute Prague)
EPI_ISL_12325995	A/goose/Czech Republic/25322-229/2021	A / H5N1	Europe / Czech Republic / Jihocesky Kraj / Okres Ceske Budejovice / Nove Hrady, Zip Code: 3740	Alexander Nagy (State Veterinary Institute Prague)
EPI_ISL_1235689	A/Chicken/Sweden/SVA210301SZ0001/KN049780-IP10/2021	A / H5N8	Europe / Sweden / Hallands Lan / Kungsbacka Kommun	Siamak Zohari (National Veterinary Institute)
EPI_ISL_1236901	A/goose/Sweden/SVA210301SZ0005/KN049802-IP11/2021	A / H5N8	Europe / Sweden / Skane Lan / Landskrona Kommun	Siamak Zohari (National Veterinary Institute)
EPI_ISL_1238195	A/Chicken/Sweden/SVA210302SZ0564/KN052345-IP12/2021	A / H5N5	Europe / Sweden / Skane Lan / Trelleborgs Kommun	Siamak Zohari (National Veterinary Institute)
EPI_ISL_1238902	A/common pheasant/Sweden/SVA210224SZ0005/KN000542-I	A / H5N8	Europe / Sweden / Skane Lan / Trelleborgs Kommun	Siamak Zohari (National Veterinary Institute)
EPI_ISL_1239109	A/northern goshawk/Sweden/SVA210224SZ0431/KN000626/2	A / H5N8	Europe / Sweden / Skane Lan / Kavlinge Kommun	Siamak Zohari (National Veterinary Institute)
EPI_ISL_1239110	A/northern goshawk/Sweden/SVA210224SZ0479/KN000627/2	A / H5N8	Europe / Sweden / Blekinge Lan / Karlshamns Kommun	Siamak Zohari (National Veterinary Institute)
EPI_ISL_1240677	A/common buzzard /Sweden/SVA210224SZ0485/KN000628/2	A / H5N8	Europe / Sweden / Skane Lan / Kristianstads Kommun	Siamak Zohari (National Veterinary Institute)
EPI_ISL_1240717	A/northern goshawk/Sweden/SVA210225SZ0302/KN000664/2	A / H5N8	Europe / Sweden / Kalmar Lan / Kalmar Kommun	Siamak Zohari (National Veterinary Institute)
EPI_ISL_1240718	A/barnacle_goose/Sweden/SVA210225SZ0307/KN000666/202	A / H5N8	Europe / Sweden / Skane Lan / Malmo Kommun	Siamak Zohari (National Veterinary Institute)
EPI_ISL_1240997	A/eastern buzzard/Tochigi/090311C/2021	A / H5N8	Asia / Japan / Tochigi	Takehiko Saito (National Institute of Animal Health)
EPI_ISL_1240998	A/mallard/Novosibirsk region/3509k/2020	A / H5N8	Europe / Russian Federation / Novosibirsk	Takehiko Saito (National Institute of Animal Health)
EPI_ISL_1241004	A/owl/Tochigi/090204T/2021	A / H5N8	Asia / Japan / Tochigi	Takehiko Saito (National Institute of Animal Health)
EPI_ISL_12436968	A/duck/Czech Republic/5361/2021	A / H5N8	Europe / Czech Republic / Kralovehradecky Kraj / Okres Hradec Kralove / Zabedov	Alexander Nagy (State Veterinary Institute Prague)
EPI_ISL_12437699	A/duck/Czech Republic/5467/2021	A / H5N8	Europe / Czech Republic / Kralovehradecky Kraj / Okres Hradec Kralove / Chudonice, GPS: N 50°	Alexander Nagy (State Veterinary Institute Prague)
EPI_ISL_12437700	A/duck/Czech Republic/6017/2021	A / H5N8	Europe / Czech Republic / Stredocesky Kraj / Okres Nymburk / Slibovice	Alexander Nagy (State Veterinary Institute Prague)
EPI_ISL_12471655	A/pelican/Greece/41-TR-313_22VIR3126-1/2022	A / H5N1	Europe / Greece	Giacomo Barbierato (Istituto Zooprofilattico Sperimentale delle Venezie / Ricerca e innovazione)
EPI_ISL_12471656	A/pelican/Greece/69_CL_22VIR3126-10/2022	A / H5N1	Europe / Greece	Giacomo Barbierato (Istituto Zooprofilattico Sperimentale delle Venezie / Ricerca e innovazione)
EPI_ISL_12471657	A/pelican/Greece/72_CL_22VIR3126-11/2022	A / H5N1	Europe / Greece	Giacomo Barbierato (Istituto Zooprofilattico Sperimentale delle Venezie / Ricerca e innovazione)
EPI_ISL_12471658	A/pelican/Greece/41_AL1_22VIR3126-2/2022	A / H5N1	Europe / Greece	Giacomo Barbierato (Istituto Zooprofilattico Sperimentale delle Venezie / Ricerca e innovazione)
EPI_ISL_12471659	A/pelican/Greece/41_AL2_22VIR3126-3/2022	A / H5N1	Europe / Greece	Giacomo Barbierato (Istituto Zooprofilattico Sperimentale delle Venezie / Ricerca e innovazione)
EPI_ISL_12471660	A/pelican/Greece/64_KI_22VIR3126-6/2022	A / H5N1	Europe / Greece	Giacomo Barbierato (Istituto Zooprofilattico Sperimentale delle Venezie / Ricerca e innovazione)
EPI_ISL_12471661	A/pelican/Greece/64_SP_22VIR3126-7/2022	A / H5N1	Europe / Greece	Giacomo Barbierato (Istituto Zooprofilattico Sperimentale delle Venezie / Ricerca e innovazione)
EPI_ISL_12471662	A/pelican/Greece/64_LI_22VIR3126-8/2022	A / H5N1	Europe / Greece	Giacomo Barbierato (Istituto Zooprofilattico Sperimentale delle Venezie / Ricerca e innovazione)
EPI_ISL_12471663	A/pelican/Greece/64_TR_22VIR3126-9/2022	A / H5N1	Europe / Greece	Giacomo Barbierato (Istituto Zooprofilattico Sperimentale delle Venezie / Ricerca e innovazione)
EPI_ISL_12474793	A/barnacle goose/Netherlands/22007405-004/2022	A / H5N1	Europe / Netherlands / Provincie Gelderland	Rene Heutink (Wageningen Bioveterinary Research)
EPI_ISL_12474794	A/greylag goose /Netherlands/22006859-001/2022	A / H5N1	Europe / Netherlands / South Holland	Rene Heutink (Wageningen Bioveterinary Research)
EPI_ISL_12512879	A/Black-headed gull/Netherlands/2/2022	A / H5N1	Europe / Netherlands / Provincie Noord-Holland / Normerven	Sanne Thewessen (Erasmus Medical Center / Viroscience)
EPI_ISL_12514425	A/Black-headed gull/Netherlands/3/2022	A / H5N1	Europe / Netherlands / De Kreupel	Sanne Thewessen (Erasmus Medical Center / Viroscience)
EPI_ISL_12514442	A/Black-headed gull/Netherlands/4/2022	A / H5N1	Europe / Netherlands / De Kreupel	Sanne Thewessen (Erasmus Medical Center / Viroscience)
EPI_ISL_12514483	A/Caspian Gull/Netherlands/3/2022	A / H5N1	Europe / Netherlands / De Kreupel	Sanne Thewessen (Erasmus Medical Center / Viroscience)
EPI_ISL_12514532	A/Common raven/Netherlands/1/2022	A / H5N1	Europe / Netherlands / Putten	Sanne Thewessen (Erasmus Medical Center / Viroscience)
EPI_ISL_12514573	A/Song Thrush/Netherlands/1/2022	A / H5N1	Europe / Netherlands / Wageningen	Sanne Thewessen (Erasmus Medical Center / Viroscience)
EPI_ISL_12514622	A/Common raven/Netherlands/2/2022	A / H5N1	Europe / Netherlands / Putten	Sanne Thewessen (Erasmus Medical Center / Viroscience)
EPI_ISL_12514681	A/European Herring Gull/Netherlands/3/2022	A / H5N1	Europe / Netherlands / De Kreupel	Sanne Thewessen (Erasmus Medical Center / Viroscience)
EPI_ISL_1254	A/Goose/Guangdong/1/96	A / H5N1	Asia / China / Guangdong Province	
EPI_ISL_1255051	A/tufted_duck/Poland/MB061/2021(H5N5)	A / H5N5	Europe / Poland / West Pomeranian Voivodeship	Edyta ?wi?to? (National Veterinary Research Institut Poland, PIWet-PIB)
EPI_ISL_12567390	A/chicken/Maryland/22-008243-001-original/2022	A / H5N1	North America / United States / Maryland / Cecil	Mary Lea Killian (National Veterinary Services Laboratories - USDA / Diagnostic Virology Laboratory)
EPI_ISL_12567391	A/chicken/Iowa/22-009180-001-original/2022	A / H5N1	North America / United States / Iowa / Franklin	Mary Lea Killian (National Veterinary Services Laboratories - USDA / Diagnostic Virology Laboratory)
EPI_ISL_12567392	A/chicken/Iowa/22-009180-002-original/2022	A / H5N1	North America / United States / Iowa / Franklin	Mary Lea Killian (National Veterinary Services Laboratories - USDA / Diagnostic Virology Laboratory)
EPI_ISL_12567393	A/chicken/Minnesota/22-009181-001-original/2022	A / H5N1	North America / United States / Minnesota / Mower	Mary Lea Killian (National Veterinary Services Laboratories - USDA / Diagnostic Virology Laboratory)
EPI_ISL_12567529	A/Embden goose/Minnesota/22-009181-003-original/2022	A / H5N1	North America / United States / Minnesota / Mower	Mary Lea Killian (National Veterinary Services Laboratories - USDA / Diagnostic Virology Laboratory)
EPI_ISL_12567530	A/turkey/Minnesota/22-009182-001-original/2022	A / H5N1	North America / United States / Minnesota / Meeker	Mary Lea Killian (National Veterinary Services Laboratories - USDA / Diagnostic Virology Laboratory)
EPI_ISL_12567531	A/turkey/Minnesota/22-009182-003-original/2022	A / H5N1	North America / United States / Minnesota / Meeker	Mary Lea Killian (National Veterinary Services Laboratories - USDA / Diagnostic Virology Laboratory)
EPI_ISL_12567532	A/domestic duck/Nebraska/22-009190-001-original/2022	A / H5N1	North America / United States / Nebraska / Holt	Mary Lea Killian (National Veterinary Services Laboratories - USDA / Diagnostic Virology Laboratory)
EPI_ISL_12567533	A/chicken/Nebraska/22-009190-002-original/2022	A / H5N1	North America / United States / Nebraska / Holt	Mary Lea Killian (National Veterinary Services Laboratories - USDA / Diagnostic Virology Laboratory)
EPI_ISL_12572652	A/chicken/Anhui/S1740/2022(H5N1)	A / H5N1	Asia / China / Anhui	Pengfei Cui (Harbin Veterinary Research Institute (CAAS) / Ministry of Agriculture)
EPI_ISL_12572653	A/chicken/Jiangxi/S40653/2021(H5N1)	A / H5N1	Asia / China / Jiangxi	Pengfei Cui (Harbin Veterinary Research Institute (CAAS) / Ministry of Agriculture)
EPI_ISL_12572654	A/duck/Guangdong/S4518/2021(H5N1)	A / H5N1	Asia / China / Guangdong	Pengfei Cui (Harbin Veterinary Research Institute (CAAS) / Ministry of Agriculture)
EPI_ISL_12572655	A/duck/Guangdong/S4525/2021(H5N1)	A / H5N1	Asia / China / Guangdong	Pengfei Cui (Harbin Veterinary Research Institute (CAAS) / Ministry of Agriculture)

EPI_ISL_12572656	A/duck/Guizhou/S1321/2022(H5N1)	A / H5N1	Asia / China / Guizhou	Pengfei Cui (Harbin Veterinary Research Institute (CAAS) / Ministry of Agriculture)
EPI_ISL_12572657	A/duck/Hubei/S4465/2021(H5N1)	A / H5N1	Asia / China / Hubei	Pengfei Cui (Harbin Veterinary Research Institute (CAAS) / Ministry of Agriculture)
EPI_ISL_12572658	A/duck/Hubei/SE128/2022(H5N1)	A / H5N1	Asia / China / Hubei	Pengfei Cui (Harbin Veterinary Research Institute (CAAS) / Ministry of Agriculture)
EPI_ISL_12572659	A/duck/Hubei/SE220/2022(H5N1)	A / H5N1	Asia / China / Hubei	Pengfei Cui (Harbin Veterinary Research Institute (CAAS) / Ministry of Agriculture)
EPI_ISL_12572660	A/duck/Jiangxi/S40833/2021(H5N1)	A / H5N1	Asia / China / Jiangxi	Pengfei Cui (Harbin Veterinary Research Institute (CAAS) / Ministry of Agriculture)
EPI_ISL_12572661	A/goose/Guizhou/S1541/2022(H5N1)	A / H5N1	Asia / China / Guizhou	Pengfei Cui (Harbin Veterinary Research Institute (CAAS) / Ministry of Agriculture)
EPI_ISL_12572662	A/goose/Hunan/SE284/2022(H5N1)	A / H5N1	Asia / China / Hunan	Pengfei Cui (Harbin Veterinary Research Institute (CAAS) / Ministry of Agriculture)
EPI_ISL_12572663	A/pigeon/Jiangxi/S40784/2021(H5N1)	A / H5N1	Asia / China / Jiangxi	Pengfei Cui (Harbin Veterinary Research Institute (CAAS) / Ministry of Agriculture)
EPI_ISL_12572664	A/wild duck/Hebei/SD012/2021(H5N1)	A / H5N1	Asia / China / Hebei	Pengfei Cui (Harbin Veterinary Research Institute (CAAS) / Ministry of Agriculture)
EPI_ISL_1259397	A/curlew/France/21P003648/2021	A / H5N3	Europe / France / Basse-Normandie / Departement de la Manche	Francois-Xavier Briand (ANSES Agence Nationale De Securite Sanitaire De L'alimentation / Laboratoire de Ploufragan-Plouzané)
EPI_ISL_1259399	A/red knot/France/21P003249/2021	A / H5N3	Europe / France / Basse-Normandie / Departement de la Manche	Francois-Xavier Briand (ANSES Agence Nationale De Securite Sanitaire De L'alimentation / Laboratoire de Ploufragan-Plouzané)
EPI_ISL_12690318	A/chicken/Missouri/22-009192-002-original/2022	A / H5N1	North America / United States / Missouri / Gentry	Mary Lea Killian (National Veterinary Services Laboratories - USDA / Diagnostic Virology Laboratory)
EPI_ISL_12690319	A/duck/Missouri/22-009192-003-original/2022	A / H5N1	North America / United States / Missouri / Gentry	Mary Lea Killian (National Veterinary Services Laboratories - USDA / Diagnostic Virology Laboratory)
EPI_ISL_12690320	A/chicken/South Dakota/22-009194-002-original/2022	A / H5N1	North America / United States / South Dakota / Bon Homme	Mary Lea Killian (National Veterinary Services Laboratories - USDA / Diagnostic Virology Laboratory)
EPI_ISL_12690321	A/guinea fowl/South Dakota/22-009194-003-original/2022	A / H5N1	North America / United States / South Dakota / Bon Homme	Mary Lea Killian (National Veterinary Services Laboratories - USDA / Diagnostic Virology Laboratory)
EPI_ISL_12690322	A/turkey/Minnesota/22-009195-002-original/2022	A / H5N1	North America / United States / Minnesota / Stearns	Mary Lea Killian (National Veterinary Services Laboratories - USDA / Diagnostic Virology Laboratory)
EPI_ISL_12690323	A/turkey/Minnesota/22-009195-003-original/2022	A / H5N1	North America / United States / Minnesota / Stearns	Mary Lea Killian (National Veterinary Services Laboratories - USDA / Diagnostic Virology Laboratory)
EPI_ISL_12690324	A/turkey/South Dakota/22-009196-001-original/2022	A / H5N1	North America / United States / South Dakota / Hutchison	Mary Lea Killian (National Veterinary Services Laboratories - USDA / Diagnostic Virology Laboratory)
EPI_ISL_12690325	A/turkey/South Dakota/22-009196-002-original/2022	A / H5N1	North America / United States / South Dakota / Hutchison	Mary Lea Killian (National Veterinary Services Laboratories - USDA / Diagnostic Virology Laboratory)
EPI_ISL_12690568	A/turkey/South Dakota/22-009198-001-original/2022	A / H5N1	North America / United States / South Dakota / Clark	Mary Lea Killian (National Veterinary Services Laboratories - USDA / Diagnostic Virology Laboratory)
EPI_ISL_12690569	A/turkey/Iowa/22-009227-001-original/2022	A / H5N1	North America / United States / Iowa / Buena Vista	Mary Lea Killian (National Veterinary Services Laboratories - USDA / Diagnostic Virology Laboratory)
EPI_ISL_12690570	A/turkey/Iowa/22-009286-001-original/2022	A / H5N1	North America / United States / Iowa / Hamilton	Mary Lea Killian (National Veterinary Services Laboratories - USDA / Diagnostic Virology Laboratory)
EPI_ISL_12690571	A/turkey/Iowa/22-009286-002-original/2022	A / H5N1	North America / United States / Iowa / Hamilton	Mary Lea Killian (National Veterinary Services Laboratories - USDA / Diagnostic Virology Laboratory)
EPI_ISL_12690589	A/chicken/Iowa/22-009287-001-original/2022	A / H5N1	North America / United States / Iowa / Guthrie	Mary Lea Killian (National Veterinary Services Laboratories - USDA / Diagnostic Virology Laboratory)
EPI_ISL_12690590	A/chicken/Iowa/22-009287-002-original/2022	A / H5N1	North America / United States / Iowa / Guthrie	Mary Lea Killian (National Veterinary Services Laboratories - USDA / Diagnostic Virology Laboratory)
EPI_ISL_12690591	A/turkey/Minnesota/22-009313-001-original/2022	A / H5N1	North America / United States / Minnesota / Kandiyohi	Mary Lea Killian (National Veterinary Services Laboratories - USDA / Diagnostic Virology Laboratory)
EPI_ISL_12690903	A/turkey/Minnesota/22-009314-001-original/2022	A / H5N1	North America / United States / Minnesota / Lac Qui Parle	Mary Lea Killian (National Veterinary Services Laboratories - USDA / Diagnostic Virology Laboratory)
EPI_ISL_12690904	A/turkey/Minnesota/22-009314-002-original/2022	A / H5N1	North America / United States / Minnesota / Lac Qui Parle	Mary Lea Killian (National Veterinary Services Laboratories - USDA / Diagnostic Virology Laboratory)
EPI_ISL_12690905	A/chicken/New York/22-009324-002-original/2022	A / H5N1	North America / United States / New York / Monroe	Mary Lea Killian (National Veterinary Services Laboratories - USDA / Diagnostic Virology Laboratory)
EPI_ISL_12690906	A/domestic duck/New York/22-009324-004-original/2022	A / H5N1	North America / United States / New York / Monroe	Mary Lea Killian (National Veterinary Services Laboratories - USDA / Diagnostic Virology Laboratory)
EPI_ISL_12690907	A/American Buff goose/New York/22-009324-006-original/202	A / H5N1	North America / United States / New York / Monroe	Mary Lea Killian (National Veterinary Services Laboratories - USDA / Diagnostic Virology Laboratory)
EPI_ISL_12690908	A/quail/New York/22-009324-009-original/2022	A / H5N1	North America / United States / New York / Monroe	Mary Lea Killian (National Veterinary Services Laboratories - USDA / Diagnostic Virology Laboratory)
EPI_ISL_12690909	A/chicken/Wyoming/22-009326-002-original/2022	A / H5N1	North America / United States / Wyoming / Johnson	Mary Lea Killian (National Veterinary Services Laboratories - USDA / Diagnostic Virology Laboratory)
EPI_ISL_12690910	A/turkey/South Dakota/22-009327-001-original/2022	A / H5N1	North America / United States / South Dakota / Spink	Mary Lea Killian (National Veterinary Services Laboratories - USDA / Diagnostic Virology Laboratory)
EPI_ISL_12690911	A/turkey/South Dakota/22-009327-002-original/2022	A / H5N1	North America / United States / South Dakota / Spink	Mary Lea Killian (National Veterinary Services Laboratories - USDA / Diagnostic Virology Laboratory)
EPI_ISL_12690912	A/turkey/Minnesota/22-009313-002-original/2022	A / H5N1	North America / United States / Minnesota / Kandiyohi	Mary Lea Killian (National Veterinary Services Laboratories - USDA / Diagnostic Virology Laboratory)
EPI_ISL_12691028	A/turkey/South Dakota/22-009329-001-original/2022	A / H5N1	North America / United States / South Dakota / Bon Homme	Mary Lea Killian (National Veterinary Services Laboratories - USDA / Diagnostic Virology Laboratory)
EPI_ISL_12691029	A/turkey/South Dakota/22-009329-002-original/2022	A / H5N1	North America / United States / South Dakota / Bon Homme	Mary Lea Killian (National Veterinary Services Laboratories - USDA / Diagnostic Virology Laboratory)
EPI_ISL_12691030	A/turkey/South Dakota/22-009330-001-original/2022	A / H5N1	North America / United States / South Dakota / Edmunds	Mary Lea Killian (National Veterinary Services Laboratories - USDA / Diagnostic Virology Laboratory)
EPI_ISL_12691031	A/turkey/South Dakota/22-009330-002-original/2022	A / H5N1	North America / United States / South Dakota / Edmunds	Mary Lea Killian (National Veterinary Services Laboratories - USDA / Diagnostic Virology Laboratory)
EPI_ISL_12691032	A/turkey/North Carolina/22-009332-001-original/2022	A / H5N1	North America / United States / North Carolina / Johnston	Mary Lea Killian (National Veterinary Services Laboratories - USDA / Diagnostic Virology Laboratory)
EPI_ISL_12691033	A/turkey/North Carolina/22-009332-002-original/2022	A / H5N1	North America / United States / North Carolina / Johnston	Mary Lea Killian (National Veterinary Services Laboratories - USDA / Diagnostic Virology Laboratory)
EPI_ISL_12691034	A/domestic goose/North Dakota/22-009337-001-original/2022	A / H5N1	North America / United States / North Dakota / Kidder	Mary Lea Killian (National Veterinary Services Laboratories - USDA / Diagnostic Virology Laboratory)
EPI_ISL_12691035	A/chicken/North Dakota/22-009337-002-original/2022	A / H5N1	North America / United States / North Dakota / Kidder	Mary Lea Killian (National Veterinary Services Laboratories - USDA / Diagnostic Virology Laboratory)
EPI_ISL_12693855	A/chicken/Iowa/22-009819-001-original/2022	A / H5N1	North America / United States / Iowa / Osceola	Mary Lea Killian (National Veterinary Services Laboratories - USDA / Diagnostic Virology Laboratory)
EPI_ISL_1273409	A/chicken/Chiba/F3T/2021	A / H5N8	Asia / Japan / Chiba	Takehiko Saito (National Institute of Animal Health)
EPI_ISL_1273410	A/chicken/Fukuoka/C1/2020	A / H5N8	Asia / Japan / Fukuoka	Takehiko Saito (National Institute of Animal Health)
EPI_ISL_1273411	A/chicken/Fukuoka/C2/2020	A / H5N8	Asia / Japan / Fukuoka	Takehiko Saito (National Institute of Animal Health)
EPI_ISL_1273412	A/chicken/Hyogo/3T/2020	A / H5N8	Asia / Japan / Hyogo	Takehiko Saito (National Institute of Animal Health)
EPI_ISL_1273413	A/chicken/Hyogo/4T/2020	A / H5N8	Asia / Japan / Hyogo	Takehiko Saito (National Institute of Animal Health)
EPI_ISL_1273414	A/chicken/Ibaraki/1T/2021	A / H5N8	Asia / Japan / Ibaraki	Takehiko Saito (National Institute of Animal Health)
EPI_ISL_1273415	A/chicken/Ibaraki/2T/2021	A / H5N8	Asia / Japan / Ibaraki	Takehiko Saito (National Institute of Animal Health)
EPI_ISL_1273416	A/chicken/Kagawa/10T/2020	A / H5N8	Asia / Japan / Kagawa	Takehiko Saito (National Institute of Animal Health)
EPI_ISL_1273417	A/chicken/Kagawa/2T/2020	A / H5N8	Asia / Japan / Kagawa	Takehiko Saito (National Institute of Animal Health)
EPI_ISL_1273418	A/chicken/Kagawa/6T/2020	A / H5N8	Asia / Japan / Kagawa	Takehiko Saito (National Institute of Animal Health)
EPI_ISL_1273419	A/chicken/Kagawa/7T/2020	A / H5N8	Asia / Japan / Kagawa	Takehiko Saito (National Institute of Animal Health)
EPI_ISL_1273420	A/chicken/Kagawa/B7T/2020	A / H5N8	Asia / Japan / Kagawa	Takehiko Saito (National Institute of Animal Health)
EPI_ISL_1273421	A/chicken/Kagawa/B9T/2020	A / H5N8	Asia / Japan / Kagawa	Takehiko Saito (National Institute of Animal Health)
EPI_ISL_1273422	A/chicken/Kagawa/C1T/2020	A / H5N8	Asia / Japan / Kagawa	Takehiko Saito (National Institute of Animal Health)
EPI_ISL_1273423	A/chicken/Kagawa/C2T/2020	A / H5N8	Asia / Japan / Kagawa	Takehiko Saito (National Institute of Animal Health)
EPI_ISL_1273424	A/chicken/Kagawa/D1T/2020	A / H5N8	Asia / Japan / Kagawa	Takehiko Saito (National Institute of Animal Health)
EPI_ISL_1273425	A/chicken/Kagawa/D2T/2020	A / H5N8	Asia / Japan / Kagawa	Takehiko Saito (National Institute of Animal Health)
EPI_ISL_1273426	A/chicken/Kagawa/D3T/2020	A / H5N8	Asia / Japan / Kagawa	Takehiko Saito (National Institute of Animal Health)
EPI_ISL_1273427	A/chicken/Kagawa/E4T/2020	A / H5N8	Asia / Japan / Kagawa	Takehiko Saito (National Institute of Animal Health)
EPI_ISL_1273428	A/chicken/Kagawa/E5T/2020	A / H5N8	Asia / Japan / Kagawa	Takehiko Saito (National Institute of Animal Health)
EPI_ISL_1273429	A/chicken/Kagawa/F3T/2020	A / H5N8	Asia / Japan / Kagawa	Takehiko Saito (National Institute of Animal Health)
EPI_ISL_1273430	A/chicken/Kagawa/F4T/2020	A / H5N8	Asia / Japan / Kagawa	Takehiko Saito (National Institute of Animal Health)
EPI_ISL_1273431	A/chicken/Kagawa/F5T/2020	A / H5N8	Asia / Japan / Kagawa	Takehiko Saito (National Institute of Animal Health)
EPI_ISL_1273432	A/chicken/Kagawa/G10T/2020	A / H5N8	Asia / Japan / Kagawa	Takehiko Saito (National Institute of Animal Health)
EPI_ISL_1273433	A/chicken/Kagawa/G12T/2020	A / H5N8	Asia / Japan / Kagawa	Takehiko Saito (National Institute of Animal Health)
EPI_ISL_1273434	A/chicken/Kagawa/H1T/2020	A / H5N8	Asia / Japan / Kagawa	Takehiko Saito (National Institute of Animal Health)
EPI_ISL_1273435	A/chicken/Kagawa/H4T/2020	A / H5N8	Asia / Japan / Kagawa	Takehiko Saito (National Institute of Animal Health)

EPI_ISL_1273436	A/chicken/Kagawa/H8T/2020	A / H5N8	Asia / Japan / Kagawa	Takehiko Saito (National Institute of Animal Health)
EPI_ISL_1273437	A/chicken/Tochigi/2T/2021	A / H5N8	Asia / Japan / Tochigi	Takehiko Saito (National Institute of Animal Health)
EPI_ISL_1273438	A/chicken/Tochigi/3T/2021	A / H5N8	Asia / Japan / Tochigi	Takehiko Saito (National Institute of Animal Health)
EPI_ISL_1273439	A/chicken/Tochigi/4T/2021	A / H5N8	Asia / Japan / Tochigi	Takehiko Saito (National Institute of Animal Health)
EPI_ISL_1273440	A/chicken/Tochigi/5T/2021	A / H5N8	Asia / Japan / Tochigi	Takehiko Saito (National Institute of Animal Health)
EPI_ISL_1273441	A/eastern buzzard/Tochigi/090311T/2021	A / H5N8	Asia / Japan / Tochigi	Takehiko Saito (National Institute of Animal Health)
EPI_ISL_12749687	A/turkey/Israel/537/2021	A / H5N1	Asia / Israel / Northern District / Nahalal	Irina Shkoda (Kimron Veterinary Institute)
EPI_ISL_12754531	A/laying_hen/Romania/11343_22VIR4106-1/2022	A / H5N1	Europe / Romania / Judetul Giurgiu / Branistea	Bianca Zecchin (Istituto Zooprofilattico Sperimentale Delle Venezie)
EPI_ISL_12754532	A/pelican/Romania/11335_22VIR4106-2/2022	A / H5N1	Europe / Romania / Judetul Tulcea / Sinoe Lake	Bianca Zecchin (Istituto Zooprofilattico Sperimentale Delle Venezie)
EPI_ISL_12754533	A/pelican/Romania/11334_22VIR4106-3/2022	A / H5N1	Europe / Romania / Judetul Tulcea / Sinoe Lake	Bianca Zecchin (Istituto Zooprofilattico Sperimentale Delle Venezie)
EPI_ISL_12754534	A/laying_hen/Romania/11562_22VIR4106-4/2022	A / H5N1	Europe / Romania / Judetul Giurgiu / Branistea	Bianca Zecchin (Istituto Zooprofilattico Sperimentale Delle Venezie)
EPI_ISL_12754535	A/sea_eagle/Norway/2022-07-196_22VIR3866-1/2022	A / H5N5	Europe / Norway / Tromso	Bianca Zecchin (Istituto Zooprofilattico Sperimentale Delle Venezie)
EPI_ISL_12754536	A/sea_eagle/Norway/2022-07-198_22VIR3866-2/2022	A / H5N5	Europe / Norway / Hitra	Bianca Zecchin (Istituto Zooprofilattico Sperimentale Delle Venezie)
EPI_ISL_1279262	A/mute swan/Croatia/14/2021	A / H5N8	Europe / Croatia / Vukovarsko-Srijemska Zupanija / River Bosut, Podgra?e	Vladimir Savi? (Croatian Veterinary Institute / Poultry Centre)
EPI_ISL_1279936	A/mute swan/Croatia/19/2021	A / H5N8	Europe / Croatia / Vukovarsko-Srijemska Zupanija / River Bosut, Podgra?e	Vladimir Savi? (Croatian Veterinary Institute / Poultry Centre)
EPI_ISL_12799972	A/Colorado/18/2022	A / H5N1	North America / United States / Colorado	Juliana DaSilva (Centers for Disease Control and Prevention / WHO Collaborating Center for Surveillance, Epidemiology and Control of I
EPI_ISL_12866390	A/chicken/Egypt/Army/1201/2022	A / H5N8	Africa / Egypt / Giza	Mohamed Gomaa Seadawy (Egyptian Armed Forces (EAF) / Biological Prevention Department)
EPI_ISL_1290863	A/duck/Laos/XBY118/2015	A / H5N6	Asia / Lao, People's Democratic Republic	Yunho Jang (Centers for Disease Control and Prevention / WHO Collaborating Center for Surveillance, Epidemiology and Control of Influ
EPI_ISL_1290892	A/duck/Laos/2310/2019	A / H5N6	Asia / Lao, People's Democratic Republic	Yunho Jang (Centers for Disease Control and Prevention / WHO Collaborating Center for Surveillance, Epidemiology and Control of Influ
EPI_ISL_12917757	A/turkey/Israel/564/2021	A / H5N1	Asia / Israel / Northern District / Ma'ayan Tzvi	Irina Shkoda (Kimron Veterinary Institute)
EPI_ISL_1295638	A/mute_swan/Norway/FU48/2021	A / H5N8	Europe / Norway	Alex Byrne (Animal and Plant Health Agency (APHA) / Virology Department)
EPI_ISL_1295639	A/turkey/Norway/FU496/2020	A / H5N8	Europe / Norway	Alex Byrne (Animal and Plant Health Agency (APHA) / Virology Department)
EPI_ISL_1296295	A/Chicken/Sweden/SVA210308SZ0214/KN057720-IP13/2021	A / H5N8	Europe / Sweden / Ostergotlands Lan / Mjolby Kommun	Siamak Zohari (National Veterinary Institute)
EPI_ISL_12968592	A/Fancy chicken/NL/FAV-0035/2021	A / H5N1	North America / Canada / Newfoundland and Labrador	Tamiko Hisanaga (Canadian Food Inspection Agency)
EPI_ISL_12968593	A/domestic goose/NL/FAV-0035-17/2021	A / H5N1	North America / Canada / Manitoba	Tamiko Hisanaga (Canadian Food Inspection Agency)
EPI_ISL_12968817	A/emu/NL/FAV-0035-12/2021	A / H5N1	North America / Canada / Newfoundland and Labrador	Tamiko Hisanaga (Canadian Food Inspection Agency)
EPI_ISL_12968823	A/chicken/NL/FAV-0033/2021	A / H5N1	North America / Canada / Newfoundland and Labrador	Tamiko Hisanaga (Canadian Food Inspection Agency)
EPI_ISL_12980521	A/Chicken/Sweden/SVA210201SZ0080/FB000693-IP4/2021	A / H5N5	Europe / Sweden / Kalmar Lan	Siamak Zohari (National Veterinary Institute)
EPI_ISL_129982	A/duck/Eastern China/1111/2011	A / H5N2	Asia / China	
EPI_ISL_129983	A/goose/Eastern China/1112/2011	A / H5N2	Asia / China	
EPI_ISL_13009618	A/Sebastopol goose/Massachusetts/22-009371-001-original/2(	A / H5N1	North America / United States / Massachusetts / Berkshire County	Mary Lea Killian (National Veterinary Services Laboratories - USDA / Diagnostic Virology Laboratory)
EPI_ISL_13009620	A/chicken/Maine/22-009412-001-original/2022	A / H5N1	North America / United States / Maine / Knox County	Mary Lea Killian (National Veterinary Services Laboratories - USDA / Diagnostic Virology Laboratory)
EPI_ISL_13009634	A/guinea fowl/Maine/22-009412-002-original/2022	A / H5N1	North America / United States / Maine / Knox County	Mary Lea Killian (National Veterinary Services Laboratories - USDA / Diagnostic Virology Laboratory)
EPI_ISL_13009636	A/chicken/Ohio/22-009419-001-original/2022	A / H5N1	North America / United States / Ohio / Franklin County	Mary Lea Killian (National Veterinary Services Laboratories - USDA / Diagnostic Virology Laboratory)
EPI_ISL_13009652	A/turkey/South Dakota/22-009534-001-original/2022	A / H5N1	North America / United States / South Dakota / Brule County	Mary Lea Killian (National Veterinary Services Laboratories - USDA / Diagnostic Virology Laboratory)
EPI_ISL_13009658	A/turkey/Iowa/22-009550-001-original/2022	A / H5N1	North America / United States / Iowa / Buena Vista County	Mary Lea Killian (National Veterinary Services Laboratories - USDA / Diagnostic Virology Laboratory)
EPI_ISL_13009673	A/turkey/North Carolina/22-009583-001-original/2022	A / H5N1	North America / United States / North Carolina / Johnston County	Mary Lea Killian (National Veterinary Services Laboratories - USDA / Diagnostic Virology Laboratory)
EPI_ISL_13009677	A/turkey/North Carolina/22-009583-002-original/2022	A / H5N1	North America / United States / North Carolina / Johnston County	Mary Lea Killian (National Veterinary Services Laboratories - USDA / Diagnostic Virology Laboratory)
EPI_ISL_13009689	A/chicken/Wyoming/22-009599-001-original/2022	A / H5N1	North America / United States / Wyoming / Park County	Mary Lea Killian (National Veterinary Services Laboratories - USDA / Diagnostic Virology Laboratory)
EPI_ISL_13009694	A/chicken/Wyoming/22-009599-002-original/2022	A / H5N1	North America / United States / Wyoming / Park County	Mary Lea Killian (National Veterinary Services Laboratories - USDA / Diagnostic Virology Laboratory)
EPI_ISL_13026099	A/Greylag goose/Luxembourg/21217773/2021	A / H5N1	Europe / Luxembourg / District de Grevenmacher	Chantal J. Snoeck (Luxembourg Institute of Health / Department of Infection and Immunity)
EPI_ISL_13026104	A/Canada goose/Luxembourg/21217776/2021	A / H5N1	Europe / Luxembourg / District de Grevenmacher / Wintrange	Chantal J. Snoeck (Luxembourg Institute of Health / Department of Infection and Immunity)
EPI_ISL_13026105	A/Canada goose/Luxembourg/21237199/2021	A / H5N1	Europe / Luxembourg / District de Grevenmacher / Remerschen	Chantal J. Snoeck (Luxembourg Institute of Health / Department of Infection and Immunity)
EPI_ISL_13026106	A/Canada goose/Luxembourg/21239614/2021	A / H5N1	Europe / Luxembourg / District de Grevenmacher / Remerschen	Chantal J. Snoeck (Luxembourg Institute of Health / Department of Infection and Immunity)
EPI_ISL_13026236	A/Grey heron/Luxembourg/21243177/2021	A / H5N1	Europe / Luxembourg / District de Grevenmacher / Remerschen	Chantal J. Snoeck (Luxembourg Institute of Health / Department of Infection and Immunity)
EPI_ISL_13026237	A/Great cormorant/Luxembourg/21243185/2021	A / H5N1	Europe / Luxembourg / District de Grevenmacher / Remerschen	Chantal J. Snoeck (Luxembourg Institute of Health / Department of Infection and Immunity)
EPI_ISL_13026245	A/Canada goose/Luxembourg/22012198/2022	A / H5N1	Europe / Luxembourg / District de Grevenmacher / Wintrange	Chantal J. Snoeck (Luxembourg Institute of Health / Department of Infection and Immunity)
EPI_ISL_13026246	A/Barnacle goose/Luxembourg/22033922/2022	A / H5N1	Europe / Luxembourg / District de Diekirch / Alscheid	Chantal J. Snoeck (Luxembourg Institute of Health / Department of Infection and Immunity)
EPI_ISL_1303550	A/Chichen/Sweden/SVA210313SZ0001/KN066654-IP15/2021	A / H5N8	Europe / Sweden / Ostergotlands Lan / Linkopings Kommun	Siamak Zohari (National Veterinary Institute)
EPI_ISL_1303776	A/Chicken/Sweden/SVA210313SZ0003/KN066666-IP16/2021	A / H5N8	Europe / Sweden / Skane Lan / Sjobo Kommun	Siamak Zohari (National Veterinary Institute)
EPI_ISL_130380	A/duck/Jiangsu/m234/2012	A / H5N2	Asia / China	
EPI_ISL_13048381	A/avian/Burkina_Faso/21VIR11911-1/2021	A / H5N1	Africa / Burkina Faso / Bonyollo	Bianca Zecchin (Istituto Zooprofilattico Sperimentale Delle Venezie)
EPI_ISL_13048382	A/avian/Burkina_Faso/21VIR11911-3/2021	A / H5N1	Africa / Burkina Faso / Gomboussougou	Bianca Zecchin (Istituto Zooprofilattico Sperimentale Delle Venezie)
EPI_ISL_13048383	A/chicken/Burkina_Faso/21VIR11911-5/2021	A / H5N1	Africa / Burkina Faso / Koubri	Bianca Zecchin (Istituto Zooprofilattico Sperimentale Delle Venezie)
EPI_ISL_13052412	A/turkey/Maine/22-009605-001-original/2022	A / H5N1	North America / United States / Maine / Lincoln County	Mary Lea Killian (National Veterinary Services Laboratories - USDA / Diagnostic Virology Laboratory)
EPI_ISL_13052413	A/chicken/Maine/22-009605-002-original/2022	A / H5N1	North America / United States / Maine / Lincoln County	Mary Lea Killian (National Veterinary Services Laboratories - USDA / Diagnostic Virology Laboratory)
EPI_ISL_13052414	A/chicken/North Dakota/22-009677-001-original/2022	A / H5N1	North America / United States / North Dakota / Dickey County	Mary Lea Killian (National Veterinary Services Laboratories - USDA / Diagnostic Virology Laboratory)
EPI_ISL_13052415	A/turkey/South Dakota/22-009688-001-original/2022	A / H5N1	North America / United States / South Dakota / McPherson County	Mary Lea Killian (National Veterinary Services Laboratories - USDA / Diagnostic Virology Laboratory)
EPI_ISL_13052416	A/chicken/Illinois/22-009720-001-original/2022	A / H5N1	North America / United States / Illinois / Carroll County	Mary Lea Killian (National Veterinary Services Laboratories - USDA / Diagnostic Virology Laboratory)
EPI_ISL_13052417	A/goose/Illinois/22-009720-003-original/2022	A / H5N1	North America / United States / Illinois / Carroll County	Mary Lea Killian (National Veterinary Services Laboratories - USDA / Diagnostic Virology Laboratory)
EPI_ISL_13052418	A/chicken/Minnesota/22-009735-001-original/2022	A / H5N1	North America / United States / Minnesota / Stearns County	Mary Lea Killian (National Veterinary Services Laboratories - USDA / Diagnostic Virology Laboratory)
EPI_ISL_13052419	A/turkey/Minnesota/22-009820-001-original/2022	A / H5N1	North America / United States / Minnesota / Morrison County	Mary Lea Killian (National Veterinary Services Laboratories - USDA / Diagnostic Virology Laboratory)
EPI_ISL_13052420	A/turkey/Iowa/22-009825-005-original/2022	A / H5N1	North America / United States / Iowa / Cherokee County	Mary Lea Killian (National Veterinary Services Laboratories - USDA / Diagnostic Virology Laboratory)
EPI_ISL_13052422	A/turkey/South Dakota/22-009839-001-original/2022	A / H5N1	North America / United States / South Dakota / Edmunds County	Mary Lea Killian (National Veterinary Services Laboratories - USDA / Diagnostic Virology Laboratory)
EPI_ISL_13052423	A/turkey/North Dakota/22-009840-001-original/2022	A / H5N1	North America / United States / North Dakota / Dickey County	Mary Lea Killian (National Veterinary Services Laboratories - USDA / Diagnostic Virology Laboratory)
EPI_ISL_13052424	A/turkey/South Dakota/22-009841-001-original/2022	A / H5N1	North America / United States / South Dakota / Charles Mix County	Mary Lea Killian (National Veterinary Services Laboratories - USDA / Diagnostic Virology Laboratory)
EPI_ISL_13052425	A/turkey/Missouri/22-009845-004-original/2022	A / H5N1	North America / United States / Missouri / Jasper County	Mary Lea Killian (National Veterinary Services Laboratories - USDA / Diagnostic Virology Laboratory)
EPI_ISL_13052426	A/chicken/Wyoming/22-009849-001-original/2022	A / H5N1	North America / United States / Wyoming / Fremont County	Mary Lea Killian (National Veterinary Services Laboratories - USDA / Diagnostic Virology Laboratory)
EPI_ISL_13052427	A/silkie chicken/Wisconsin/22-009934-002-original/2022	A / H5N1	North America / United States / Wisconsin / Rock County	Mary Lea Killian (National Veterinary Services Laboratories - USDA / Diagnostic Virology Laboratory)
EPI_ISL_13052429	A/turkey/Minnesota/22-009976-003-original/2022	A / H5N1	North America / United States / Minnesota / Stearns County	Mary Lea Killian (National Veterinary Services Laboratories - USDA / Diagnostic Virology Laboratory)
EPI_ISL_13052430	A/turkey/North Dakota/22-009978-001-original/2022	A / H5N1	North America / United States / North Dakota / LaMoure County	Mary Lea Killian (National Veterinary Services Laboratories - USDA / Diagnostic Virology Laboratory)
EPI_ISL_13052431	A/turkey/North Dakota/22-009978-002-original/2022	A / H5N1	North America / United States / North Dakota / LaMoure County	Mary Lea Killian (National Veterinary Services Laboratories - USDA / Diagnostic Virology Laboratory)

EPI_ISL_13052432	A/turkey/South Dakota/22-009979-001-original/2022	A / H5N1	North America / United States / South Dakota / Spink County	Mary Lea Killian (National Veterinary Services Laboratories - USDA / Diagnostic Virology Laboratory)
EPI_ISL_13052433	A/turkey/South Dakota/22-009979-002-original/2022	A / H5N1	North America / United States / South Dakota / Spink County	Mary Lea Killian (National Veterinary Services Laboratories - USDA / Diagnostic Virology Laboratory)
EPI_ISL_13052717	A/fox/Wisconsin/22-013774-002-original/2022	A / H5N1	North America / United States / Wisconsin / Jefferson County	Mary Lea Killian (National Veterinary Services Laboratories - USDA / Diagnostic Virology Laboratory)
EPI_ISL_13052718	A/fox/Wisconsin/22-013774-009-original/2022	A / H5N1	North America / United States / Wisconsin / Waushara County	Mary Lea Killian (National Veterinary Services Laboratories - USDA / Diagnostic Virology Laboratory)
EPI_ISL_13052719	A/fox/Wisconsin/22-013774-014-original/2022	A / H5N1	North America / United States / Wisconsin / Rock County	Mary Lea Killian (National Veterinary Services Laboratories - USDA / Diagnostic Virology Laboratory)
EPI_ISL_13052720	A/fox/Minnesota/22-014182-001-original/2022	A / H5N1	North America / United States / Minnesota / Anoka County	Mary Lea Killian (National Veterinary Services Laboratories - USDA / Diagnostic Virology Laboratory)
EPI_ISL_13052721	A/fox/Iowa/22-014421-001-original/2022	A / H5N1	North America / United States / Iowa / Hancock County	Mary Lea Killian (National Veterinary Services Laboratories - USDA / Diagnostic Virology Laboratory)
EPI_ISL_13052722	A/fox/Minnesota/22-014660-001-original/2022	A / H5N1	North America / United States / Minnesota / Dakota County	Mary Lea Killian (National Veterinary Services Laboratories - USDA / Diagnostic Virology Laboratory)
EPI_ISL_13052723	A/fox/Minnesota/22-014660-002-original/2022	A / H5N1	North America / United States / Minnesota / Dakota County	Mary Lea Killian (National Veterinary Services Laboratories - USDA / Diagnostic Virology Laboratory)
EPI_ISL_13052724	A/fox/Minnesota/22-014661-001-original/2022	A / H5N1	North America / United States / Minnesota / Washington County	Mary Lea Killian (National Veterinary Services Laboratories - USDA / Diagnostic Virology Laboratory)
EPI_ISL_13052725	A/fox/Wisconsin/22-014746-010-original/2022	A / H5N1	North America / United States / Wisconsin / Adam County	Mary Lea Killian (National Veterinary Services Laboratories - USDA / Diagnostic Virology Laboratory)
EPI_ISL_13052726	A/fox/Wisconsin/22-014746-016-original/2022	A / H5N1	North America / United States / Wisconsin / Adam County	Mary Lea Killian (National Veterinary Services Laboratories - USDA / Diagnostic Virology Laboratory)
EPI_ISL_13052727	A/fox/Wisconsin/22-014746-023-original/2022	A / H5N1	North America / United States / Wisconsin / Grant County	Mary Lea Killian (National Veterinary Services Laboratories - USDA / Diagnostic Virology Laboratory)
EPI_ISL_13052728	A/fox/Wisconsin/22-014746-029-original/2022	A / H5N1	North America / United States / Wisconsin / Grant County	Mary Lea Killian (National Veterinary Services Laboratories - USDA / Diagnostic Virology Laboratory)
EPI_ISL_1305775	A/Chicken/Sweden/SVA210314SZ0001/KN066672-IP17/2021	A / H5N8	Europe / Sweden / Ostergotlands Lan / Mjolby Kommun	Siamak Zohari (National Veterinary Institute)
EPI_ISL_1305776	A/Chicken/Sweden/SVA210314SZ0002/KN066679-IP18/2021	A / H5N8	Europe / Sweden / Ostergotlands Lan / Mjolby Kommun	Siamak Zohari (National Veterinary Institute)
EPI_ISL_1305777	A/Common peacock/Sweden/SVA210311SZ0002/KN001568-IP	A / H5N5	Europe / Sweden / Skane Lan / Skurups Kommun	Siamak Zohari (National Veterinary Institute)
EPI_ISL_1305778	A/Goose/Sweden/SVA210311SZ0003/KN0001570-IP14/2021	A / H5N5	Europe / Sweden / Skane Lan / Skurups Kommun	Siamak Zohari (National Veterinary Institute)
EPI_ISL_1305779	A/Duck/Sweden/SVA210311SZ0004/KN0001570-IP14/2021	A / H5N5	Europe / Sweden / Skane Lan / Skurups Kommun	Siamak Zohari (National Veterinary Institute)
EPI_ISL_1305780	A/Canada_goose/Sweden/SVA210302SZ0455/KN000714/SKsir	A / H5N8	Europe / Sweden / Skane Lan / Simrishamns Kommun	Siamak Zohari (National Veterinary Institute)
EPI_ISL_1305781	A/Mute Swan/Sweden/SVA210302Z0465/KN000716/SOTR/20:	A / H5N5	Europe / Sweden / Sodermanlands Lan / Trosa Kommun	Siamak Zohari (National Veterinary Institute)
EPI_ISL_13068727	A/common crane/Israel/670/2021	A / H5N1	Asia / Israel / Northern District / Agamon Hula	Irina Shkoda (Kimron Veterinary Institute)
EPI_ISL_1307695	A/Mute Swan/Sweden/SVA210304SZ0311/KN000739/VG/202	A / H5N8	Europe / Sweden / Vastra Gotalands Lan / Ockero Kommun	Siamak Zohari (National Veterinary Institute)
EPI_ISL_1307703	A/Canade goose/Sweden/SVA210304SZ0320/KN000779/SK/2C	A / H5N8	Europe / Sweden / Skane Lan / Klippans Kommun	Siamak Zohari (National Veterinary Institute)
EPI_ISL_1307709	A/Canada goose/Sweden/SVA210305SZ0255/KN000797/SK/2C	A / H5N8	Europe / Sweden / Skane Lan / Tomelilla Kommun	Siamak Zohari (National Veterinary Institute)
EPI_ISL_1307716	A/Mute Swan/Sweden/SVA210303SZ0380/KN000800/St/2021	A / H5N8	Europe / Sweden / Stockholms Lan / Sodertalje Kommun	Siamak Zohari (National Veterinary Institute)
EPI_ISL_1307720	A/northern goshawk/Sweden/SVA210303SZ0381/KN000801/J	A / H5N8	Europe / Sweden / Jonkopings Lan / Jonkopings Kommun	Siamak Zohari (National Veterinary Institute)
EPI_ISL_1308010	A/Mute Swan/Sweden/SVA210303SZ0392/KN000806/Kal/202	A / H5N5	Europe / Sweden / Kalmar Lan / Monsteras Kommun	Siamak Zohari (National Veterinary Institute)
EPI_ISL_13114176	A/Broiler chicken/BC/FAV-0228-OS/2022	A / H5N1	North America / Canada / British Columbia	Yohannes Berhane (Canadian Food Inspection Agency / Avian Unit)
EPI_ISL_13116131	A/Chicken/BC/FAV-0346-OS/2022	A / H5N1	North America / Canada / British Columbia	Yohannes Berhane (Canadian Food Inspection Agency / Avian Unit)
EPI_ISL_13117168	A/Chicken/BC/FAV-0348-OS/2022	A / H5N1	North America / Canada / British Columbia	Yohannes Berhane (Canadian Food Inspection Agency / Avian Unit)
EPI_ISL_13117288	A/Chicken/BC/FAV-0402-OS/2022	A / H5N1	North America / Canada / British Columbia	Yohannes Berhane (Canadian Food Inspection Agency / Avian Unit)
EPI_ISL_13117289	A/Chicken/BC/FAV-0369-OS/2022	A / H5N1	North America / Canada / British Columbia	Yohannes Berhane (Canadian Food Inspection Agency / Avian Unit)
EPI_ISL_13117290	A/Chicken/BC/FAV-0460-OS/2022	A / H5N1	North America / Canada / British Columbia	Yohannes Berhane (Canadian Food Inspection Agency / Avian Unit)
EPI_ISL_13117299	A/Chicken/BC/FAV-0488-OS/2022	A / H5N1	North America / Canada / British Columbia	Yohannes Berhane (Canadian Food Inspection Agency / Avian Unit)
EPI_ISL_13175606	A/Turkey/Sweden/SVA210315SZ0260/FB067516-IP19/2021	A / H5N8	Europe / Sweden / Skane Lan / Tomelilla Kommun	Siamak Zohari (National Veterinary Institute)
EPI_ISL_13175608	A/Chicken/Sweden/SVA210223SZ0647/FB046588-IP7/2021	A / H5N8	Europe / Sweden / Ostergotlands Lan / Linkopings Kommun	Siamak Zohari (National Veterinary Institute)
EPI_ISL_13175667	A/Common pheasant/Sweden/SVA210224SZ0005/KN000542-I	A / H5N8	Europe / Sweden / Skane Lan / Trelleborgs Kommun	Siamak Zohari (National Veterinary Institute)
EPI_ISL_13201050	A/Lesser Black-backed Gull/Netherlands/1/2022	A / H5N1	Europe / Netherlands / Provincie Noord-Holland / De Kreupel	Sanne Thewessen (Erasmus Medical Center / Viroscience)
EPI_ISL_13201074	A/European polecat/Netherlands/1/2022	A / H5N1	Europe / Netherlands / Zuid-Holland	Sanne Thewessen (Erasmus Medical Center / Viroscience)
EPI_ISL_13243350	A/duck/Bangladesh/19D1874/2022	A / H5N1	Asia / Bangladesh	Mohammad Enayet Hossain (International Centre for Diarrhoeal Disease Research (ICDDR,B) / Virology)
EPI_ISL_13243698	A/duck/Bangladesh/19D1819/2021	A / H5N1	Asia / Bangladesh	Mohammad Enayet Hossain (International Centre for Diarrhoeal Disease Research (ICDDR,B) / Virology)
EPI_ISL_13245602	A/white-tailed eagle/Iceland/2022AI02104/2021	A / H5N1	Europe / Iceland / Westfjords / Kerlingarfjörður	Jacqueline King (Friedrich-Loeffler-Institut)
EPI_ISL_13246267	A/chicken/Iceland/2022AI02564/2022	A / H5N1	Europe / Iceland / South	Jacqueline King (Friedrich-Loeffler-Institut)
EPI_ISL_13246657	A/chicken/Iceland/2022AI02565/2022	A / H5N1	Europe / Iceland / South	Jacqueline King (Friedrich-Loeffler-Institut)
EPI_ISL_13285237	A/turkey/South Dakota/22-009980-001-original/2022	A / H5N1	North America / United States / South Dakota / Lake County	Mary Lea Killian (National Veterinary Services Laboratories - USDA / Diagnostic Virology Laboratory)
EPI_ISL_13285238	A/turkey/South Dakota/22-009980-002-original/2022	A / H5N1	North America / United States / South Dakota / Lake County	Mary Lea Killian (National Veterinary Services Laboratories - USDA / Diagnostic Virology Laboratory)
EPI_ISL_13285239	A/turkey/North Carolina/22-009981-001-original/2022	A / H5N1	North America / United States / North Carolina / Johnston County	Mary Lea Killian (National Veterinary Services Laboratories - USDA / Diagnostic Virology Laboratory)
EPI_ISL_13285240	A/turkey/North Carolina/22-009981-004-original/2022	A / H5N1	North America / United States / North Carolina / Johnston County	Mary Lea Killian (National Veterinary Services Laboratories - USDA / Diagnostic Virology Laboratory)
EPI_ISL_13285241	A/turkey/North Carolina/22-009982-001-original/2022	A / H5N1	North America / United States / North Carolina / Johnston County	Mary Lea Killian (National Veterinary Services Laboratories - USDA / Diagnostic Virology Laboratory)
EPI_ISL_13285242	A/turkey/North Carolina/22-009982-002-original/2022	A / H5N1	North America / United States / North Carolina / Johnston County	Mary Lea Killian (National Veterinary Services Laboratories - USDA / Diagnostic Virology Laboratory)
EPI_ISL_13285243	A/turkey/North Carolina/22-009983-007-original/2022	A / H5N1	North America / United States / North Carolina / Wayne County	Mary Lea Killian (National Veterinary Services Laboratories - USDA / Diagnostic Virology Laboratory)
EPI_ISL_13285244	A/turkey/North Carolina/22-009983-008-original/2022	A / H5N1	North America / United States / North Carolina / Wayne County	Mary Lea Killian (National Veterinary Services Laboratories - USDA / Diagnostic Virology Laboratory)
EPI_ISL_13285245	A/pheasant/Texas/22-009984-001-original/2022	A / H5N1	North America / United States / Texas / Erath County	Mary Lea Killian (National Veterinary Services Laboratories - USDA / Diagnostic Virology Laboratory)
EPI_ISL_13285246	A/chicken/Iowa/22-009985-001-original/2022	A / H5N1	North America / United States / Iowa / Humboldt County	Mary Lea Killian (National Veterinary Services Laboratories - USDA / Diagnostic Virology Laboratory)
EPI_ISL_13285536	A/turkey/Iowa/22-009986-001-original/2022	A / H5N1	North America / United States / Iowa / Sac County	Mary Lea Killian (National Veterinary Services Laboratories - USDA / Diagnostic Virology Laboratory)
EPI_ISL_13285537	A/turkey/Iowa/22-009986-002-original/2022	A / H5N1	North America / United States / Iowa / Sac County	Mary Lea Killian (National Veterinary Services Laboratories - USDA / Diagnostic Virology Laboratory)
EPI_ISL_13285538	A/turkey/Minnesota/22-009996-001-original/2022	A / H5N1	North America / United States / Minnesota / Le Sueur County	Mary Lea Killian (National Veterinary Services Laboratories - USDA / Diagnostic Virology Laboratory)
EPI_ISL_13285539	A/turkey/Minnesota/22-009997-001-original/2022	A / H5N1	North America / United States / Minnesota / Dodge County	Mary Lea Killian (National Veterinary Services Laboratories - USDA / Diagnostic Virology Laboratory)
EPI_ISL_13285540	A/turkey/Minnesota/22-009997-002-original/2022	A / H5N1	North America / United States / Minnesota / Dodge County	Mary Lea Killian (National Veterinary Services Laboratories - USDA / Diagnostic Virology Laboratory)
EPI_ISL_13285541	A/turkey/Minnesota/22-009998-001-original/2022	A / H5N1	North America / United States / Minnesota / Kandiyohi County	Mary Lea Killian (National Veterinary Services Laboratories - USDA / Diagnostic Virology Laboratory)
EPI_ISL_13285542	A/turkey/Minnesota/22-009999-001-original/2022	A / H5N1	North America / United States / Minnesota / Becker County	Mary Lea Killian (National Veterinary Services Laboratories - USDA / Diagnostic Virology Laboratory)
EPI_ISL_13285543	A/turkey/Minnesota/22-010000-001-original/2022	A / H5N1	North America / United States / Minnesota / Kandiyohi County	Mary Lea Killian (National Veterinary Services Laboratories - USDA / Diagnostic Virology Laboratory)
EPI_ISL_13285548	A/turkey/Minnesota/22-009999-002-original/2022	A / H5N1	North America / United States / Minnesota / Becker County	Mary Lea Killian (National Veterinary Services Laboratories - USDA / Diagnostic Virology Laboratory)
EPI_ISL_13285549	A/pheasant/Texas/22-010008-001-original/2022	A / H5N1	North America / United States / Texas / Erath County	Mary Lea Killian (National Veterinary Services Laboratories - USDA / Diagnostic Virology Laboratory)
EPI_ISL_13285550	A/pheasant/Texas/22-010008-002-original/2022	A / H5N1	North America / United States / Texas / Erath County	Mary Lea Killian (National Veterinary Services Laboratories - USDA / Diagnostic Virology Laboratory)
EPI_ISL_13285551	A/turkey/South Dakota/22-010013-001-original/2022	A / H5N1	North America / United States / South Dakota / McPherson County	Mary Lea Killian (National Veterinary Services Laboratories - USDA / Diagnostic Virology Laboratory)
EPI_ISL_13285552	A/turkey/South Dakota/22-010013-002-original/2022	A / H5N1	North America / United States / South Dakota / McPherson County	Mary Lea Killian (National Veterinary Services Laboratories - USDA / Diagnostic Virology Laboratory)
EPI_ISL_13285553	A/turkey/North Dakota/22-010015-001-original/2022	A / H5N1	North America / United States / North Dakota / LaMoure County	Mary Lea Killian (National Veterinary Services Laboratories - USDA / Diagnostic Virology Laboratory)
EPI_ISL_13285554	A/turkey/North Dakota/22-010015-002-original/2022	A / H5N1	North America / United States / North Dakota / LaMoure County	Mary Lea Killian (National Veterinary Services Laboratories - USDA / Diagnostic Virology Laboratory)
EPI_ISL_13285555	A/turkey/Minnesota/22-010085-001-original/2022	A / H5N1	North America / United States / Minnesota / Morrison County	Mary Lea Killian (National Veterinary Services Laboratories - USDA / Diagnostic Virology Laboratory)
EPI_ISL_13285556	A/turkey/Minnesota/22-010085-002-original/2022	A / H5N1	North America / United States / Minnesota / Morrison County	Mary Lea Killian (National Veterinary Services Laboratories - USDA / Diagnostic Virology Laboratory)

EPI_ISL_13285557	A/turkey/Minnesota/22-010092-002-original/2022	A / H5N1	North America / United States / Minnesota / Kandiyohi County	Mary Lea Killian (National Veterinary Services Laboratories - USDA / Diagnostic Virology Laboratory)
EPI_ISL_13285558	A/turkey/Minnesota/22-010092-003-original/2022	A / H5N1	North America / United States / Minnesota / Kandiyohi County	Mary Lea Killian (National Veterinary Services Laboratories - USDA / Diagnostic Virology Laboratory)
EPI_ISL_13285559	A/turkey/Iowa/22-010094-001-original/2022	A / H5N1	North America / United States / Iowa / Hamilton County	Mary Lea Killian (National Veterinary Services Laboratories - USDA / Diagnostic Virology Laboratory)
EPI_ISL_13285560	A/turkey/Iowa/22-010094-002-original/2022	A / H5N1	North America / United States / Iowa / Hamilton County	Mary Lea Killian (National Veterinary Services Laboratories - USDA / Diagnostic Virology Laboratory)
EPI_ISL_13286627	A/turkey/Minnesota/22-009996-002-original/2022	A / H5N1	North America / United States / Minnesota / Le Sueur County	Mary Lea Killian (National Veterinary Services Laboratories - USDA / Diagnostic Virology Laboratory)
EPI_ISL_13300319	A/brant goose/Netherlands/22009881-002/2022	A / H5N1	Europe / Netherlands / Provincie Friesland	Rene Heutink (Wageningen Bioveterinary Research)
EPI_ISL_13300324	A/white stork/Netherlands/22009973-002/2022	A / H5N1	Europe / Netherlands / Provincie Gelderland	Rene Heutink (Wageningen Bioveterinary Research)
EPI_ISL_13338081	A/Northern gannet/Sweden/SVA220525SZ0402/FB001671/O-2	A / H5N1	Europe / Sweden / Vastra Gotalands Lan / Molndals Kommun	Siamak Zohari (National Veterinary Institute)
EPI_ISL_13370915	A/domestic_duck/England/032919/2022	A / H5N1	Europe / United Kingdom / England	Alex Byrne (Animal and Plant Health Agency (APHA) / Virology Department)
EPI_ISL_13370918	A/domestic_duck/England/041295/2022	A / H5N1	Europe / United Kingdom / England	Alex Byrne (Animal and Plant Health Agency (APHA) / Virology Department)
EPI_ISL_13370924	A/chicken/England/053826/2022	A / H5N1	Europe / United Kingdom / England	Alex Byrne (Animal and Plant Health Agency (APHA) / Virology Department)
EPI_ISL_13370925	A/chicken/England/063896/2022	A / H5N1	Europe / United Kingdom / England	Alex Byrne (Animal and Plant Health Agency (APHA) / Virology Department)
EPI_ISL_13418526	A/Little egret/Israel/172/2022	A / H5N1	Asia / Israel / Northern District / Kinneret Beach	Irina Shkoda (Kimron Veterinary Institute)
EPI_ISL_13429290	A/Caspian Gull/Netherlands/4/2022	A / H5N1	Europe / Netherlands / De Kreupel	Sanne Thewessen (Erasmus Medical Center / Viroscience)
EPI_ISL_13429291	A/Sandwich Tern/Netherlands/8/2022	A / H5N1	Europe / Netherlands / Stellendam, Scheelhoekeiland	Sanne Thewessen (Erasmus Medical Center / Viroscience)
EPI_ISL_13429292	A/Sandwich Tern/Netherlands/5/2022	A / H5N1	Europe / Netherlands / Stellendam, Scheelhoekeiland	Sanne Thewessen (Erasmus Medical Center / Viroscience)
EPI_ISL_13429293	A/Black-headed gull/Netherlands/5/2022	A / H5N1	Europe / Netherlands / De Kreupel	Sanne Thewessen (Erasmus Medical Center / Viroscience)
EPI_ISL_13429294	A/Black-headed gull/Netherlands/6/2022	A / H5N1	Europe / Netherlands / De Kreupel	Sanne Thewessen (Erasmus Medical Center / Viroscience)
EPI_ISL_13429295	A/Sandwich Tern/Netherlands/1/2022	A / H5N1	Europe / Netherlands / Den Oever, Waddenhaven	Sanne Thewessen (Erasmus Medical Center / Viroscience)
EPI_ISL_13429296	A/Sandwich Tern/Netherlands/2/2022	A / H5N1	Europe / Netherlands / Haringvliet, Slijkplaat	Sanne Thewessen (Erasmus Medical Center / Viroscience)
EPI_ISL_13429297	A/Sandwich Tern/Netherlands/3/2022	A / H5N1	Europe / Netherlands / Haringvliet, Slijkplaat	Sanne Thewessen (Erasmus Medical Center / Viroscience)
EPI_ISL_13429298	A/Common Tern/Netherlands/1/2022	A / H5N1	Europe / Netherlands / Haringvliet, Bliek	Sanne Thewessen (Erasmus Medical Center / Viroscience)
EPI_ISL_13429299	A/Sandwich Tern/Netherlands/6/2022	A / H5N1	Europe / Netherlands / Stellendam, Scheelhoekeiland	Sanne Thewessen (Erasmus Medical Center / Viroscience)
EPI_ISL_13429300	A/Sandwich Tern/Netherlands/7/2022	A / H5N1	Europe / Netherlands / Stellendam, Scheelhoekeiland	Sanne Thewessen (Erasmus Medical Center / Viroscience)
EPI_ISL_13431305	A/turkey/Minnesota/22-010122-001-original/2022	A / H5N1	North America / United States / Minnesota / Stearns County	Mary Lea Killian (National Veterinary Services Laboratories - USDA / Diagnostic Virology Laboratory)
EPI_ISL_13431306	A/turkey/Minnesota/22-010122-002-original/2022	A / H5N1	North America / United States / Minnesota / Stearns County	Mary Lea Killian (National Veterinary Services Laboratories - USDA / Diagnostic Virology Laboratory)
EPI_ISL_13431307	A/turkey/Minnesota/22-010123-001-original/2022	A / H5N1	North America / United States / Minnesota / Waseca County	Mary Lea Killian (National Veterinary Services Laboratories - USDA / Diagnostic Virology Laboratory)
EPI_ISL_13431308	A/turkey/Minnesota/22-010123-004-original/2022	A / H5N1	North America / United States / Minnesota / Waseca County	Mary Lea Killian (National Veterinary Services Laboratories - USDA / Diagnostic Virology Laboratory)
EPI_ISL_13431309	A/turkey/Minnesota/22-010124-001-original/2022	A / H5N1	North America / United States / Minnesota / Meeker County	Mary Lea Killian (National Veterinary Services Laboratories - USDA / Diagnostic Virology Laboratory)
EPI_ISL_13431310	A/turkey/Minnesota/22-010124-002-original/2022	A / H5N1	North America / United States / Minnesota / Meeker County	Mary Lea Killian (National Veterinary Services Laboratories - USDA / Diagnostic Virology Laboratory)
EPI_ISL_13431311	A/turkey/Minnesota/22-010125-001-original/2022	A / H5N1	North America / United States / Minnesota / Morrison County	Mary Lea Killian (National Veterinary Services Laboratories - USDA / Diagnostic Virology Laboratory)
EPI_ISL_13431312	A/turkey/Minnesota/22-010125-002-original/2022	A / H5N1	North America / United States / Minnesota / Morrison County	Mary Lea Killian (National Veterinary Services Laboratories - USDA / Diagnostic Virology Laboratory)
EPI_ISL_13431313	A/turkey/Minnesota/22-010126-001-original/2022	A / H5N1	North America / United States / Minnesota / Big Stone County	Mary Lea Killian (National Veterinary Services Laboratories - USDA / Diagnostic Virology Laboratory)
EPI_ISL_13431314	A/turkey/Minnesota/22-010126-002-original/2022	A / H5N1	North America / United States / Minnesota / Big Stone County	Mary Lea Killian (National Veterinary Services Laboratories - USDA / Diagnostic Virology Laboratory)
EPI_ISL_13431688	A/turkey/Minnesota/22-010127-001-original/2022	A / H5N1	North America / United States / Minnesota / Morrison County	Mary Lea Killian (National Veterinary Services Laboratories - USDA / Diagnostic Virology Laboratory)
EPI_ISL_13431689	A/turkey/Minnesota/22-010127-002-original/2022	A / H5N1	North America / United States / Minnesota / Morrison County	Mary Lea Killian (National Veterinary Services Laboratories - USDA / Diagnostic Virology Laboratory)
EPI_ISL_13431690	A/turkey/Iowa/22-010132-001-original/2022	A / H5N1	North America / United States / Iowa / Hardin County	Mary Lea Killian (National Veterinary Services Laboratories - USDA / Diagnostic Virology Laboratory)
EPI_ISL_13431691	A/turkey/Iowa/22-010132-002-original/2022	A / H5N1	North America / United States / Iowa / Hardin County	Mary Lea Killian (National Veterinary Services Laboratories - USDA / Diagnostic Virology Laboratory)
EPI_ISL_13431692	A/turkey/South Dakota/22-010135-001-original/2022	A / H5N1	North America / United States / South Dakota / Faulk County	Mary Lea Killian (National Veterinary Services Laboratories - USDA / Diagnostic Virology Laboratory)
EPI_ISL_13431693	A/turkey/South Dakota/22-010135-002-original/2022	A / H5N1	North America / United States / South Dakota / Faulk County	Mary Lea Killian (National Veterinary Services Laboratories - USDA / Diagnostic Virology Laboratory)
EPI_ISL_13431694	A/turkey/South Dakota/22-010137-001-original/2022	A / H5N1	North America / United States / South Dakota / Clark County	Mary Lea Killian (National Veterinary Services Laboratories - USDA / Diagnostic Virology Laboratory)
EPI_ISL_13431695	A/turkey/South Dakota/22-010137-002-original/2022	A / H5N1	North America / United States / South Dakota / Clark County	Mary Lea Killian (National Veterinary Services Laboratories - USDA / Diagnostic Virology Laboratory)
EPI_ISL_13431696	A/turkey/South Dakota/22-010138-001-original/2022	A / H5N1	North America / United States / South Dakota / Spink County	Mary Lea Killian (National Veterinary Services Laboratories - USDA / Diagnostic Virology Laboratory)
EPI_ISL_13431697	A/turkey/South Dakota/22-010138-002-original/2022	A / H5N1	North America / United States / South Dakota / Spink County	Mary Lea Killian (National Veterinary Services Laboratories - USDA / Diagnostic Virology Laboratory)
EPI_ISL_13432603	A/turkey/South Dakota/22-010139-001-original/2022	A / H5N1	North America / United States / South Dakota / Beadle County	Mary Lea Killian (National Veterinary Services Laboratories - USDA / Diagnostic Virology Laboratory)
EPI_ISL_13432604	A/turkey/South Dakota/22-010139-002-original/2022	A / H5N1	North America / United States / South Dakota / Beadle County	Mary Lea Killian (National Veterinary Services Laboratories - USDA / Diagnostic Virology Laboratory)
EPI_ISL_13432605	A/chicken/North Dakota/22-010141-001-original/2022	A / H5N1	North America / United States / North Dakota / Cass County	Mary Lea Killian (National Veterinary Services Laboratories - USDA / Diagnostic Virology Laboratory)
EPI_ISL_13432606	A/turkey/Missouri/22-010142-001-original/2022	A / H5N1	North America / United States / Missouri / Lawrence County	Mary Lea Killian (National Veterinary Services Laboratories - USDA / Diagnostic Virology Laboratory)
EPI_ISL_13432607	A/turkey/North Carolina/22-010144-001-original/2022	A / H5N1	North America / United States / North Carolina / Wayne County	Mary Lea Killian (National Veterinary Services Laboratories - USDA / Diagnostic Virology Laboratory)
EPI_ISL_13432608	A/turkey/North Carolina/22-010144-002-original/2022	A / H5N1	North America / United States / North Carolina / Wayne County	Mary Lea Killian (National Veterinary Services Laboratories - USDA / Diagnostic Virology Laboratory)
EPI_ISL_13432609	A/turkey/North Carolina/22-010145-001-original/2022	A / H5N1	North America / United States / North Carolina / Wayne County	Mary Lea Killian (National Veterinary Services Laboratories - USDA / Diagnostic Virology Laboratory)
EPI_ISL_13432610	A/turkey/North Carolina/22-010145-002-original/2022	A / H5N1	North America / United States / North Carolina / Wayne County	Mary Lea Killian (National Veterinary Services Laboratories - USDA / Diagnostic Virology Laboratory)
EPI_ISL_13432611	A/turkey/North Carolina/22-010146-001-original/2022	A / H5N1	North America / United States / North Carolina / Wayne County	Mary Lea Killian (National Veterinary Services Laboratories - USDA / Diagnostic Virology Laboratory)
EPI_ISL_13432612	A/turkey/North Carolina/22-010146-002-original/2022	A / H5N1	North America / United States / North Carolina / Wayne County	Mary Lea Killian (National Veterinary Services Laboratories - USDA / Diagnostic Virology Laboratory)
EPI_ISL_13432752	A/chicken/Nebraska/22-010155-001-original/2022	A / H5N1	North America / United States / Nebraska / Scotts Bluff County	Mary Lea Killian (National Veterinary Services Laboratories - USDA / Diagnostic Virology Laboratory)
EPI_ISL_13432764	A/chicken/Wyoming/22-010157-001-original/2022	A / H5N1	North America / United States / Wyoming / Sheridan County	Mary Lea Killian (National Veterinary Services Laboratories - USDA / Diagnostic Virology Laboratory)
EPI_ISL_13432776	A/chicken/Wyoming/22-010157-002-original/2022	A / H5N1	North America / United States / Wyoming / Sheridan County	Mary Lea Killian (National Veterinary Services Laboratories - USDA / Diagnostic Virology Laboratory)
EPI_ISL_13432788	A/guinea fowl/Maine/22-010181-001-original/2022	A / H5N1	North America / United States / Maine / Waldo County	Mary Lea Killian (National Veterinary Services Laboratories - USDA / Diagnostic Virology Laboratory)
EPI_ISL_13432800	A/goose/Maine/22-010181-002-original/2022	A / H5N1	North America / United States / Maine / Waldo County	Mary Lea Killian (National Veterinary Services Laboratories - USDA / Diagnostic Virology Laboratory)
EPI_ISL_13432814	A/chicken/New York/22-010200-002-original/2022	A / H5N1	North America / United States / New York / Orleans County	Mary Lea Killian (National Veterinary Services Laboratories - USDA / Diagnostic Virology Laboratory)
EPI_ISL_13432826	A/guinea fowl/New York/22-010200-003-original/2022	A / H5N1	North America / United States / New York / Orleans County	Mary Lea Killian (National Veterinary Services Laboratories - USDA / Diagnostic Virology Laboratory)
EPI_ISL_13432838	A/turkey/Missouri/22-010296-001-original/2022	A / H5N1	North America / United States / Missouri / Dade County	Mary Lea Killian (National Veterinary Services Laboratories - USDA / Diagnostic Virology Laboratory)
EPI_ISL_13432849	A/chicken/North Carolina/22-010298-001-original/2022	A / H5N1	North America / United States / North Carolina / Wayne County	Mary Lea Killian (National Veterinary Services Laboratories - USDA / Diagnostic Virology Laboratory)
EPI_ISL_13432861	A/chicken/North Carolina/22-010298-002-original/2022	A / H5N1	North America / United States / North Carolina / Wayne County	Mary Lea Killian (National Veterinary Services Laboratories - USDA / Diagnostic Virology Laboratory)
EPI_ISL_13433340	A/chicken/North Carolina/22-010299-002-original/2022	A / H5N1	North America / United States / North Carolina / Wayne County	Mary Lea Killian (National Veterinary Services Laboratories - USDA / Diagnostic Virology Laboratory)
EPI_ISL_13433352	A/turkey/Minnesota/22-010312-001-original/2022	A / H5N1	North America / United States / Minnesota / Waseca County	Mary Lea Killian (National Veterinary Services Laboratories - USDA / Diagnostic Virology Laboratory)
EPI_ISL_13433365	A/turkey/Minnesota/22-010312-004-original/2022	A / H5N1	North America / United States / Minnesota / Waseca County	Mary Lea Killian (National Veterinary Services Laboratories - USDA / Diagnostic Virology Laboratory)
EPI_ISL_13433376	A/chicken/New York/22-010321-001-original/2022	A / H5N1	North America / United States / New York / Fulton County	Mary Lea Killian (National Veterinary Services Laboratories - USDA / Diagnostic Virology Laboratory)
EPI_ISL_13433388	A/chicken/New York/22-010321-002-original/2022	A / H5N1	North America / United States / New York / Fulton County	Mary Lea Killian (National Veterinary Services Laboratories - USDA / Diagnostic Virology Laboratory)
EPI_ISL_13433400	A/domestic duck/New York/22-010321-003-original/2022	A / H5N1	North America / United States / New York / Fulton County	Mary Lea Killian (National Veterinary Services Laboratories - USDA / Diagnostic Virology Laboratory)
EPI_ISL_13433412	A/chicken/Wisconsin/22-010325-001-original/2022	A / H5N1	North America / United States / Wisconsin / Racine County	Mary Lea Killian (National Veterinary Services Laboratories - USDA / Diagnostic Virology Laboratory)
EPI_ISL_13433423	A/turkey/Wisconsin/22-010325-003-original/2022	A / H5N1	North America / United States / Wisconsin / Racine County	Mary Lea Killian (National Veterinary Services Laboratories - USDA / Diagnostic Virology Laboratory)

EPI_ISL_13433435	A/turkey/South Dakota/22-010346-001-original/2022	A / H5N1	North America / United States / South Dakota / McPherson County	Mary Lea Killian (National Veterinary Services Laboratories - USDA / Diagnostic Virology Laboratory)
EPI_ISL_13433447	A/turkey/South Dakota/22-010346-002-original/2022	A / H5N1	North America / United States / South Dakota / McPherson County	Mary Lea Killian (National Veterinary Services Laboratories - USDA / Diagnostic Virology Laboratory)
EPI_ISL_13434393	A/chicken/Wyoming/22-010378-001-original/2022	A / H5N1	North America / United States / Wyoming / Park County	Mary Lea Killian (National Veterinary Services Laboratories - USDA / Diagnostic Virology Laboratory)
EPI_ISL_13434407	A/chicken/Montana/22-010445-001-original/2022	A / H5N1	North America / United States / Montana / Judith Basin County	Mary Lea Killian (National Veterinary Services Laboratories - USDA / Diagnostic Virology Laboratory)
EPI_ISL_13434418	A/chicken/Montana/22-010445-003-original/2022	A / H5N1	North America / United States / Montana / Judith Basin County	Mary Lea Killian (National Veterinary Services Laboratories - USDA / Diagnostic Virology Laboratory)
EPI_ISL_13434430	A/turkey/North Carolina/22-010448-001-original/2022	A / H5N1	North America / United States / North Carolina / Wayne County	Mary Lea Killian (National Veterinary Services Laboratories - USDA / Diagnostic Virology Laboratory)
EPI_ISL_13434442	A/turkey/North Carolina/22-010448-003-original/2022	A / H5N1	North America / United States / North Carolina / Wayne County	Mary Lea Killian (National Veterinary Services Laboratories - USDA / Diagnostic Virology Laboratory)
EPI_ISL_13434454	A/Cascade duck/Montana/22-010454-001-original/2022	A / H5N1	North America / United States / Montana / Cascade County	Mary Lea Killian (National Veterinary Services Laboratories - USDA / Diagnostic Virology Laboratory)
EPI_ISL_13434466	A/Cascade duck/Montana/22-010454-003-original/2022	A / H5N1	North America / United States / Montana / Cascade County	Mary Lea Killian (National Veterinary Services Laboratories - USDA / Diagnostic Virology Laboratory)
EPI_ISL_13434477	A/turkey/Minnesota/22-010463-002-original/2022	A / H5N1	North America / United States / Minnesota / Kandiyohi County	Mary Lea Killian (National Veterinary Services Laboratories - USDA / Diagnostic Virology Laboratory)
EPI_ISL_13434489	A/turkey/Minnesota/22-010464-001-original/2022	A / H5N1	North America / United States / Minnesota / Renville County	Mary Lea Killian (National Veterinary Services Laboratories - USDA / Diagnostic Virology Laboratory)
EPI_ISL_13434551	A/turkey/Minnesota/22-010464-002-original/2022	A / H5N1	North America / United States / Minnesota / Renville County	Mary Lea Killian (National Veterinary Services Laboratories - USDA / Diagnostic Virology Laboratory)
EPI_ISL_13453559	A/domestic_duck/England/073865/2022	A / H5N1	Europe / United Kingdom / England	Alex Byrne (Animal and Plant Health Agency (APHA) / Virology Department)
EPI_ISL_13453560	A/chicken/Scotland/078873/2022	A / H5N1	Europe / United Kingdom / Scotland	Alex Byrne (Animal and Plant Health Agency (APHA) / Virology Department)
EPI_ISL_13453565	A/buzzard/Scotland/043118/2022	A / H5N1	Europe / United Kingdom / Scotland	Alex Byrne (Animal and Plant Health Agency (APHA) / Virology Department)
EPI_ISL_13453567	A/turkey/England/081395/2022	A / H5N1	Europe / United Kingdom / England	Alex Byrne (Animal and Plant Health Agency (APHA) / Virology Department)
EPI_ISL_13453568	A/turkey/England/081490/2022	A / H5N1	Europe / United Kingdom / England	Alex Byrne (Animal and Plant Health Agency (APHA) / Virology Department)
EPI_ISL_13465456	A/Great white pelican/Israel/619/2021	A / H5N1	Asia / Israel / Central District / Mishmar HaSharon	Irina Shkoda (Kimron Veterinary Institute)
EPI_ISL_13477015	A/chicken/Israel/88/2022	A / H5N1	Asia / Israel / Haifa District / Binyamina	Irina Shkoda (Kimron Veterinary Institute)
EPI_ISL_13486807	A/Common_buzzard/Scotland/020817/2022	A / H5N1	Europe / United Kingdom / Scotland	Alex Byrne (Animal and Plant Health Agency (APHA) / Virology Department)
EPI_ISL_13486869	A/Greylag_goose/Scotland/024915/2022	A / H5N1	Europe / United Kingdom / Scotland	Alex Byrne (Animal and Plant Health Agency (APHA) / Virology Department)
EPI_ISL_13514370	A/Thalasseus sandvicensis/Belgium/7473_0009/2022	A / H5N1	Europe / Belgium / Provincie West-Vlaanderen / Oostende	Steven Van Borm (Sciensano, Department of Animal Infectious Diseases / Animal Infectious Diseases)
EPI_ISL_13514375	A/Gallus gallus/Belgium/8092_0002/2022	A / H5N1	Europe / Belgium / Provincie Vlaams-Brabant / Zoutleeuw	Steven Van Borm (Sciensano, Department of Animal Infectious Diseases / Animal Infectious Diseases)
EPI_ISL_13519451	A/gull/France/22P015977/2022	A / H5N1	Europe / France / Picardie / Departement de la Somme	Francois-Xavier Briand (ANSES Agence Nationale De Securite Sanitaire De L'alimentation / Laboratoire de Ploufragan-Plouzané)
EPI_ISL_13609610	A/Great white pelican/Israel/123/2022	A / H5N1	Asia / Israel / Haifa District / Ma'ayan Tzvi	Irina Shkoda (Kimron Veterinary Institute)
EPI_ISL_13613219	A/Greylag Goose/Netherlands/4/2022	A / H5N1	Europe / Netherlands / Obdam	Sanne Thewessen (Erasmus Medical Center / Viroscience)
EPI_ISL_13613220	A/Greylag Goose/Netherlands/5/2022	A / H5N1	Europe / Netherlands / Obdam	Sanne Thewessen (Erasmus Medical Center / Viroscience)
EPI_ISL_13613221	A/Greylag Goose/Netherlands/6/2022	A / H5N1	Europe / Netherlands / Obdam	Sanne Thewessen (Erasmus Medical Center / Viroscience)
EPI_ISL_13613222	A/Greylag Goose/Netherlands/7/2022	A / H5N1	Europe / Netherlands / Obdam	Sanne Thewessen (Erasmus Medical Center / Viroscience)
EPI_ISL_13613223	A/Greylag Goose/Netherlands/8/2022	A / H5N1	Europe / Netherlands / Obdam	Sanne Thewessen (Erasmus Medical Center / Viroscience)
EPI_ISL_13613224	A/Common Tern/Netherlands/2/2022	A / H5N1	Europe / Netherlands / De Kreupel, Enkhuizen	Sanne Thewessen (Erasmus Medical Center / Viroscience)
EPI_ISL_13613225	A/Black-headed gull/Netherlands/7/2022	A / H5N1	Europe / Netherlands / De Kreupel, Enkhuizen	Sanne Thewessen (Erasmus Medical Center / Viroscience)
EPI_ISL_13613226	A/Black-headed gull/Netherlands/8/2022	A / H5N1	Europe / Netherlands / De Kreupel, Enkhuizen	Sanne Thewessen (Erasmus Medical Center / Viroscience)
EPI_ISL_13613227	A/Black-headed gull/Netherlands/9/2022	A / H5N1	Europe / Netherlands / De Kreupel, Enkhuizen	Sanne Thewessen (Erasmus Medical Center / Viroscience)
EPI_ISL_13613228	A/Sandwich Tern/Netherlands/9/2022	A / H5N1	Europe / Netherlands / Den Oever, Waddenhaven	Sanne Thewessen (Erasmus Medical Center / Viroscience)
EPI_ISL_13613229	A/Sandwich Tern/Netherlands/10/2022	A / H5N1	Europe / Netherlands / Den Oever, Waddenhaven	Sanne Thewessen (Erasmus Medical Center / Viroscience)
EPI_ISL_13613230	A/Common Tern/Netherlands/3/2022	A / H5N1	Europe / Netherlands / Wieringerwerf, Noorderdijkweg	Sanne Thewessen (Erasmus Medical Center / Viroscience)
EPI_ISL_13618384	A/duck/Israel/589/2021	A / H5N1	Asia / Israel / Northern District / Kfar Baruch	Irina Shkoda (Kimron Veterinary Institute)
EPI_ISL_13632084	A/chicken/Kazakhstan/23/2020	A / H5N8	Asia / Kazakhstan / Almaty Province	Kairat Tabynov (Kazakh National Agrarian University / International Center for Vaccinology)
EPI_ISL_13658359	A/common kestel/Israel/49-2/2022	A / H5N1	Asia / Israel / Northern District / Agamon Hula	Irina Shkoda (Kimron Veterinary Institute)
EPI_ISL_13692620	A/turkey/Stavropol/211-1V/2022	A / H5N1	Europe / Russian Federation / Stavropol Krai	Natalia Goncharova (State Research Center of Virology and Biotechnology (VECTOR) / Emerging Zoonotic Diseases and Influenza)
EPI_ISL_13692621	A/turkey/Stavropol/211-4V/2022	A / H5N1	Europe / Russian Federation / Stavropol Krai	Natalia Goncharova (State Research Center of Virology and Biotechnology (VECTOR) / Emerging Zoonotic Diseases and Influenza)
EPI_ISL_13692622	A/turkey/Stavropol/211-7V/2022	A / H5N1	Europe / Russian Federation / Stavropol Krai	Natalia Goncharova (State Research Center of Virology and Biotechnology (VECTOR) / Emerging Zoonotic Diseases and Influenza)
EPI_ISL_13692623	A/turkey/Stavropol/211-12V/2022	A / H5N1	Europe / Russian Federation / Stavropol Krai	Natalia Goncharova (State Research Center of Virology and Biotechnology (VECTOR) / Emerging Zoonotic Diseases and Influenza)
EPI_ISL_13692625	A/turkey/Stavropol/211-15V/2022	A / H5N1	Europe / Russian Federation / Stavropol Krai	Natalia Goncharova (State Research Center of Virology and Biotechnology (VECTOR) / Emerging Zoonotic Diseases and Influenza)
EPI_ISL_13692626	A/turkey/Stavropol/211-18V/2022	A / H5N1	Europe / Russian Federation / Stavropol Krai	Natalia Goncharova (State Research Center of Virology and Biotechnology (VECTOR) / Emerging Zoonotic Diseases and Influenza)
EPI_ISL_13692628	A/turkey/Stavropol/211-5V/2022	A / H5N1	Europe / Russian Federation / Stavropol Krai	Natalia Goncharova (State Research Center of Virology and Biotechnology (VECTOR) / Emerging Zoonotic Diseases and Influenza)
EPI_ISL_13692629	A/turkey/Stavropol/211-9V/2022	A / H5N1	Europe / Russian Federation / Stavropol Krai	Natalia Goncharova (State Research Center of Virology and Biotechnology (VECTOR) / Emerging Zoonotic Diseases and Influenza)
EPI_ISL_13692630	A/crow/Khabarovsk/216-11V/2022	A / H5N1	Europe / Russian Federation / Khabarovsk Krai	Natalia Goncharova (State Research Center of Virology and Biotechnology (VECTOR) / Emerging Zoonotic Diseases and Influenza)
EPI_ISL_13692632	A/crow/Khabarovsk/216-12V/2022	A / H5N1	Europe / Russian Federation / Khabarovsk Krai	Natalia Goncharova (State Research Center of Virology and Biotechnology (VECTOR) / Emerging Zoonotic Diseases and Influenza)
EPI_ISL_13692634	A/crow/Khabarovsk/216-13V/2022	A / H5N1	Europe / Russian Federation / Khabarovsk Krai	Natalia Goncharova (State Research Center of Virology and Biotechnology (VECTOR) / Emerging Zoonotic Diseases and Influenza)
EPI_ISL_13692637	A/dalmatian pelican/Astrakhan/213-1V/2022	A / H5N1	Europe / Russian Federation / Astrakhan Oblast	Natalia Goncharova (State Research Center of Virology and Biotechnology (VECTOR) / Emerging Zoonotic Diseases and Influenza)
EPI_ISL_13692638	A/dalmatian pelican/Astrakhan/213-2V/2022	A / H5N1	Europe / Russian Federation / Astrakhan Oblast	Natalia Goncharova (State Research Center of Virology and Biotechnology (VECTOR) / Emerging Zoonotic Diseases and Influenza)
EPI_ISL_137254	A/duck/Hebei/2/2011	A / H5N2	Asia / China / Hebei Province	Di Liu (Institute of Microbiology, Chinese Academy of Sciences)
EPI_ISL_137255	A/duck/Hebei/3/2011	A / H5N2	Asia / China / Hebei Province	Di Liu (Institute of Microbiology, Chinese Academy of Sciences)
EPI_ISL_13778462	A/Lesser Black-backed Gull/Netherlands/2/2022	A / H5N1	Europe / Netherlands / Enkhuizen, Ijsselmeer, De Kreupel	Sanne Thewessen (Erasmus Medical Center / Viroscience)
EPI_ISL_13778464	A/European Herring Gull/Netherlands/4/2022	A / H5N1	Europe / Netherlands / Den Oever, Waddenhaven	Sanne Thewessen (Erasmus Medical Center / Viroscience)
EPI_ISL_13778466	A/Oystercatcher/Netherlands/2/2022	A / H5N1	Europe / Netherlands / Den Oever, Waddenhaven	Sanne Thewessen (Erasmus Medical Center / Viroscience)
EPI_ISL_13778467	A/Sandwich Tern/Netherlands/11/2022	A / H5N1	Europe / Netherlands / Den Oever, Waddenhaven	Sanne Thewessen (Erasmus Medical Center / Viroscience)
EPI_ISL_13778468	A/European Herring Gull/Netherlands/5/2022	A / H5N1	Europe / Netherlands / Walcheren, 't Vroon Westkapelle	Sanne Thewessen (Erasmus Medical Center / Viroscience)
EPI_ISL_13778469	A/European Herring Gull/Netherlands/6/2022	A / H5N1	Europe / Netherlands / Walcheren, 't Vroon Westkapelle	Sanne Thewessen (Erasmus Medical Center / Viroscience)
EPI_ISL_13778474	A/European Herring Gull/Netherlands/7/2022	A / H5N1	Europe / Netherlands / Walcheren, 't Vroon Westkapelle	Sanne Thewessen (Erasmus Medical Center / Viroscience)
EPI_ISL_13778475	A/Sandwich Tern/Netherlands/12/2022	A / H5N1	Europe / Netherlands / Walcheren, 't Vroon Westkapelle	Sanne Thewessen (Erasmus Medical Center / Viroscience)
EPI_ISL_13778476	A/Sandwich Tern/Netherlands/13/2022	A / H5N1	Europe / Netherlands / Walcheren, 't Vroon Westkapelle	Sanne Thewessen (Erasmus Medical Center / Viroscience)
EPI_ISL_13778477	A/Common Tern/Netherlands/8/2022	A / H5N1	Europe / Netherlands / Haringvliet, Ventjagersplaten, Den Bommel	Sanne Thewessen (Erasmus Medical Center / Viroscience)
EPI_ISL_13778478	A/Sandwich Tern/Netherlands/14/2022	A / H5N1	Europe / Netherlands / Walcheren, 't Vroon Westkapelle	Sanne Thewessen (Erasmus Medical Center / Viroscience)
EPI_ISL_13778479	A/Common Tern/Netherlands/9/2022	A / H5N1	Europe / Netherlands / Walcheren, 't Vroon Westkapelle	Sanne Thewessen (Erasmus Medical Center / Viroscience)
EPI_ISL_13778522	A/Sandwich Tern/Netherlands/15/2022	A / H5N1	Europe / Netherlands / Walcheren, 't Vroon Westkapelle	Sanne Thewessen (Erasmus Medical Center / Viroscience)
EPI_ISL_13778523	A/Common Tern/Netherlands/4/2022	A / H5N1	Europe / Netherlands / Wieringerwerf, Noorderdijkweg	Sanne Thewessen (Erasmus Medical Center / Viroscience)
EPI_ISL_13778524	A/Common Tern/Netherlands/5/2022	A / H5N1	Europe / Netherlands / Wieringerwerf, Noorderdijkweg	Sanne Thewessen (Erasmus Medical Center / Viroscience)
EPI_ISL_13778525	A/Common Tern/Netherlands/6/2022	A / H5N1	Europe / Netherlands / Wieringerwerf, Noorderdijkweg	Sanne Thewessen (Erasmus Medical Center / Viroscience)
EPI_ISL_13778526	A/Common Tern/Netherlands/7/2022	A / H5N1	Europe / Netherlands / Wieringerwerf, Noorderdijkweg	Sanne Thewessen (Erasmus Medical Center / Viroscience)

EPI_ISL_1379443	A/chicken/Vietnam/Raho4-Cd-20-421/2020	A / H5N6	Asia / Vietnam	Yunho Jang (Centers for Disease Control and Prevention / WHO Collaborating Center for Surveillance, Epidemiology and Control of Infl
EPI_ISL_1385792	A/Chicken/Sweden/SVA210323SZ001/KN001982-IP21/2021	A / H5N5	Europe / Sweden / Stockholms Lan / Ekero Kommun	Siamak Zohari (National Veterinary Institute)
EPI_ISL_1386128	A/Chiken/Sweden/SVA210321SZ0001/KN073551-IP20/2021	A / H5N8	Europe / Sweden / Skane Lan / Hassleholms Kommun	Siamak Zohari (National Veterinary Institute)
EPI_ISL_1386249	A/western marsh harrier/Sweden/SVA210316SZ0486/KN0010:	A / H5N8	Europe / Sweden / Ostergotlands Lan / Linkopings Kommun	Siamak Zohari (National Veterinary Institute)
EPI_ISL_13876272	A/chicken/Ryazan/224-1V/2022	A / H5N1	Europe / Russian Federation / Ryazan Oblast	Natalia Goncharova (State Research Center of Virology and Biotechnology (VECTOR) / Emerging Zoonotic Diseases and Influenza)
EPI_ISL_1389398	A/northern goshawk/Sweden/SVA210316SZ0489/KN001044/N	A / H5N8	Europe / Sweden / Skane Lan / Kavlinge Kommun	Siamak Zohari (National Veterinary Institute)
EPI_ISL_13902858	A/Eurasian Curlew/Netherlands/2/2022	A / H5N1	Europe / Netherlands / Den Oever, Waddenhaven	Sanne Thewessen (Erasmus Medical Center / Viroscience)
EPI_ISL_1390546	A/northern goshawk/Sweden/SVA210316SZ0489/KN001045/N	A / H5N8	Europe / Sweden / Skane Lan / Lomma Kommun	Siamak Zohari (National Veterinary Institute)
EPI_ISL_1391019	A/common golden eye/Sweden/SVA210316SZ0489/KN001046	A / H5N5	Europe / Sweden / Skane Lan / Lomma Kommun	Siamak Zohari (National Veterinary Institute)
EPI_ISL_1391276	A/northern goshawk/Sweden/SVA210318SZ0322/KN001092/N	A / H5N5	Europe / Sweden / Skane Lan / Simrishamns Kommun	Siamak Zohari (National Veterinary Institute)
EPI_ISL_139383	A/goose/Shandong/k1204/2009	A / H5N5	Asia / China	
EPI_ISL_139384	A/goose/Guangdong/k0103/2010	A / H5N5	Asia / China	
EPI_ISL_139385	A/duck/Jiangsu/k1203/2010	A / H5N8	Asia / China	
EPI_ISL_139386	A/quail/Jiangsu/k0104/2010	A / H5N5	Asia / China	
EPI_ISL_13955171	A/chicken/Czech Republic/2968/2022	A / H5N1	Europe / Czech Republic / Plzensky Kraj / Okres Plzen-Jih / Lišice, Dolní Lukavice, ZipCode: 629 71	Alexander Nagy (State Veterinary Institute Prague)
EPI_ISL_13955204	A/chicken/Czech Republic/8028-1/2022	A / H5N1	Europe / Czech Republic / Plzensky Kraj / Okres Klatovy / Plánice	Alexander Nagy (State Veterinary Institute Prague)
EPI_ISL_13957819	A/chicken/Mali/T1-177_22VIR6104-1/2022	A / H5N1	Africa / Mali / Kati	Bianca Zecchin (Istituto Zooprofilattico Sperimentale Delle Venezie)
EPI_ISL_13957821	A/chicken/Mali/T2-178_22VIR6104-3/2022	A / H5N1	Africa / Mali / Kati	Bianca Zecchin (Istituto Zooprofilattico Sperimentale Delle Venezie)
EPI_ISL_13957822	A/chicken/Mali/S3-179_22VIR6104-5/2022	A / H5N1	Africa / Mali / Kati	Bianca Zecchin (Istituto Zooprofilattico Sperimentale Delle Venezie)
EPI_ISL_13957823	A/chicken/Mali/T4_180_22VIR6104-7/2022	A / H5N1	Africa / Mali / Kati	Bianca Zecchin (Istituto Zooprofilattico Sperimentale Delle Venezie)
EPI_ISL_13967740	A/chicken/Czech Republic/8028-2/2022	A / H5N1	Europe / Czech Republic / Plzensky Kraj / Okres Klatovy / Plánice	Alexander Nagy (State Veterinary Institute Prague)
EPI_ISL_13969422	A/Goose/Scotland/036879/2022	A / H5N1	Europe / United Kingdom / Scotland	Alex Byrne (Animal and Plant Health Agency (APHA) / Virology Department)
EPI_ISL_13969423	A/Gull/Scotland/060376/2022	A / H5N1	Europe / United Kingdom / Scotland	Alex Byrne (Animal and Plant Health Agency (APHA) / Virology Department)
EPI_ISL_13969424	A/Gannet/Scotland/084490/2022	A / H5N1	Europe / United Kingdom / Scotland	Alex Byrne (Animal and Plant Health Agency (APHA) / Virology Department)
EPI_ISL_13969426	A/Gannet/Scotland/090910/2022	A / H5N1	Europe / United Kingdom / Scotland	Alex Byrne (Animal and Plant Health Agency (APHA) / Virology Department)
EPI_ISL_13969428	A/Greylag_goose/England/247696/2022	A / H5N1	Europe / United Kingdom / England	Alex Byrne (Animal and Plant Health Agency (APHA) / Virology Department)
EPI_ISL_13969430	A/Mute_swan/Wales/058560/2022	A / H5N1	Europe / United Kingdom / Wales	Alex Byrne (Animal and Plant Health Agency (APHA) / Virology Department)
EPI_ISL_13990713	A/Anser_anser/Spain/1277-10_22VIR6312-10/2022	A / H5N1	Europe / Spain	Giacomo Barbierato (Istituto Zooprofilattico Sperimentale delle Venezie / Ricerca e innovazione)
EPI_ISL_13990714	A/wild_bird/Spain/1302-1_22VIR6312-11/2022	A / H5N1	Europe / Spain	Giacomo Barbierato (Istituto Zooprofilattico Sperimentale delle Venezie / Ricerca e innovazione)
EPI_ISL_13990715	A/Cygnus_olor/Spain/1675-1_22VIR6312-14/2022	A / H5N1	Europe / Spain	Giacomo Barbierato (Istituto Zooprofilattico Sperimentale delle Venezie / Ricerca e innovazione)
EPI_ISL_13990716	A/Rhea/Spain/1950-6_22VIR6312-17/2022	A / H5N1	Europe / Spain	Giacomo Barbierato (Istituto Zooprofilattico Sperimentale delle Venezie / Ricerca e innovazione)
EPI_ISL_13990717	A/Gypaetus_barbatus/Spain/1878-9_22VIR6312-18/2022	A / H5N1	Europe / Spain	Giacomo Barbierato (Istituto Zooprofilattico Sperimentale delle Venezie / Ricerca e innovazione)
EPI_ISL_13990718	A/Gypaetus_barbatus/Spain/1956-25_22VIR6312-19/2022	A / H5N1	Europe / Spain	Giacomo Barbierato (Istituto Zooprofilattico Sperimentale delle Venezie / Ricerca e innovazione)
EPI_ISL_13990719	A/Falco_peregrinus/Spain/2010-11_22VIR6312-20/2022	A / H5N1	Europe / Spain	Giacomo Barbierato (Istituto Zooprofilattico Sperimentale delle Venezie / Ricerca e innovazione)
EPI_ISL_13990720	A/Anser_anser/Spain/2010-3_22VIR6312-21/2022	A / H5N1	Europe / Spain	Giacomo Barbierato (Istituto Zooprofilattico Sperimentale delle Venezie / Ricerca e innovazione)
EPI_ISL_13990721	A/turkey/Spain/711-5_22VIR6312-23/2022	A / H5N1	Europe / Spain	Giacomo Barbierato (Istituto Zooprofilattico Sperimentale delle Venezie / Ricerca e innovazione)
EPI_ISL_13990722	A/turkey/Spain/712-7_22VIR6312-24/2022	A / H5N1	Europe / Spain	Giacomo Barbierato (Istituto Zooprofilattico Sperimentale delle Venezie / Ricerca e innovazione)
EPI_ISL_13990723	A/turkey/Spain/801-6_22VIR6312-25/2022	A / H5N1	Europe / Spain	Giacomo Barbierato (Istituto Zooprofilattico Sperimentale delle Venezie / Ricerca e innovazione)
EPI_ISL_13990724	A/turkey/Spain/802-7_22VIR6312-26/2022	A / H5N1	Europe / Spain	Giacomo Barbierato (Istituto Zooprofilattico Sperimentale delle Venezie / Ricerca e innovazione)
EPI_ISL_13990725	A/turkey/Spain/803-5_22VIR6312-27/2022	A / H5N1	Europe / Spain	Giacomo Barbierato (Istituto Zooprofilattico Sperimentale delle Venezie / Ricerca e innovazione)
EPI_ISL_13990726	A/turkey/Spain/805-8_22VIR6312-28/2022	A / H5N1	Europe / Spain	Giacomo Barbierato (Istituto Zooprofilattico Sperimentale delle Venezie / Ricerca e innovazione)
EPI_ISL_13990727	A/turkey/Spain/830-5_22VIR6312-29/2022	A / H5N1	Europe / Spain	Giacomo Barbierato (Istituto Zooprofilattico Sperimentale delle Venezie / Ricerca e innovazione)
EPI_ISL_13990728	A/Anser_anser/Spain/750-4_22VIR6312-2/2022	A / H5N1	Europe / Spain	Giacomo Barbierato (Istituto Zooprofilattico Sperimentale delle Venezie / Ricerca e innovazione)
EPI_ISL_13990729	A/turkey/Spain/859-8_22VIR6312-30/2022	A / H5N1	Europe / Spain	Giacomo Barbierato (Istituto Zooprofilattico Sperimentale delle Venezie / Ricerca e innovazione)
EPI_ISL_13990730	A/chicken/Spain/897-7_22VIR6312-31/2022	A / H5N1	Europe / Spain	Giacomo Barbierato (Istituto Zooprofilattico Sperimentale delle Venezie / Ricerca e innovazione)
EPI_ISL_13990731	A/chicken/Spain/899-7_22VIR6312-32/2022	A / H5N1	Europe / Spain	Giacomo Barbierato (Istituto Zooprofilattico Sperimentale delle Venezie / Ricerca e innovazione)
EPI_ISL_13990732	A/chicken/Spain/924-3_22VIR6312-33/2022	A / H5N1	Europe / Spain	Giacomo Barbierato (Istituto Zooprofilattico Sperimentale delle Venezie / Ricerca e innovazione)
EPI_ISL_13990733	A/chicken/Spain/942-8_22VIR6312-34/2022	A / H5N1	Europe / Spain	Giacomo Barbierato (Istituto Zooprofilattico Sperimentale delle Venezie / Ricerca e innovazione)
EPI_ISL_13990734	A/chicken/Spain/1096-8_22VIR6312-35/2022	A / H5N1	Europe / Spain	Giacomo Barbierato (Istituto Zooprofilattico Sperimentale delle Venezie / Ricerca e innovazione)
EPI_ISL_13990735	A/turkey/Spain/1097-7_22VIR6312-36/2022	A / H5N1	Europe / Spain	Giacomo Barbierato (Istituto Zooprofilattico Sperimentale delle Venezie / Ricerca e innovazione)
EPI_ISL_13990736	A/chicken/Spain/340-37_22VIR6312-37/2022	A / H5N1	Europe / Spain	Giacomo Barbierato (Istituto Zooprofilattico Sperimentale delle Venezie / Ricerca e innovazione)
EPI_ISL_13990737	A/Anser_anser/Spain/638-6_22VIR6312-39/2022	A / H5N1	Europe / Spain	Giacomo Barbierato (Istituto Zooprofilattico Sperimentale delle Venezie / Ricerca e innovazione)
EPI_ISL_13990738	A/chicken/Spain/806-7_22VIR6312-41/2022	A / H5N1	Europe / Spain	Giacomo Barbierato (Istituto Zooprofilattico Sperimentale delle Venezie / Ricerca e innovazione)
EPI_ISL_13990739	A/Ciconia_ciconia/Spain/853-14_22VIR6312-42/2022	A / H5N1	Europe / Spain	Giacomo Barbierato (Istituto Zooprofilattico Sperimentale delle Venezie / Ricerca e innovazione)
EPI_ISL_13990740	A/Falco_peregrinus/Spain/1932-1_22VIR6312-45/2022	A / H5N1	Europe / Spain	Giacomo Barbierato (Istituto Zooprofilattico Sperimentale delle Venezie / Ricerca e innovazione)
EPI_ISL_13990741	A/Cygnus_olor/Spain/1950-10_22VIR6312-46/2022	A / H5N1	Europe / Spain	Giacomo Barbierato (Istituto Zooprofilattico Sperimentale delle Venezie / Ricerca e innovazione)
EPI_ISL_13990743	A/Ardea_cinerea/Spain/863-1_22VIR6312-5/2022	A / H5N1	Europe / Spain	Giacomo Barbierato (Istituto Zooprofilattico Sperimentale delle Venezie / Ricerca e innovazione)
EPI_ISL_13990744	A/Anser_anser/Spain/863-2_22VIR6312-6/2022	A / H5N1	Europe / Spain	Giacomo Barbierato (Istituto Zooprofilattico Sperimentale delle Venezie / Ricerca e innovazione)
EPI_ISL_13990745	A/Ciconia_ciconia/Spain/971-6_22VIR6312-7/2022	A / H5N1	Europe / Spain	Giacomo Barbierato (Istituto Zooprofilattico Sperimentale delle Venezie / Ricerca e innovazione)
EPI_ISL_13990746	A/Anser_anser/Spain/1035-5_22VIR6312-8/2022	A / H5N1	Europe / Spain	Giacomo Barbierato (Istituto Zooprofilattico Sperimentale delle Venezie / Ricerca e innovazione)
EPI_ISL_13990747	A/Anser_anser/Spain/1124-65_22VIR6312-9/2022	A / H5N1	Europe / Spain	Giacomo Barbierato (Istituto Zooprofilattico Sperimentale delle Venezie / Ricerca e innovazione)
EPI_ISL_1399231	A/chicken/Czech Republic/3531-1/2021	A / H5N8	Europe / Czech Republic / Vysocina Kraj / Okres Pelhrimov / Horni Cerekev; GPS: N 49°19.49288'	Alexander Nagy (State Veterinary Institute Prague)
EPI_ISL_1399232	A/chicken/Czech Republic/3893/2021	A / H5N8	Europe / Czech Republic / Jihocesky Kraj / Okres Strakonice / Rojice	Alexander Nagy (State Veterinary Institute Prague)
EPI_ISL_1399233	A/chicken/Czech Republic/4092-1/2021	A / H5N8	Europe / Czech Republic / Plzensky Kraj / Okres Klatovy / Hnevnice; GPS: N 49°44.08495', E 13°6.	Alexander Nagy (State Veterinary Institute Prague)
EPI_ISL_1399234	A/chicken/Czech Republic/4092-2/2021	A / H5N8	Europe / Czech Republic / Plzensky Kraj / Okres Klatovy / Hnevnice; GPS: N 49°44.08495', E 13°6.	Alexander Nagy (State Veterinary Institute Prague)
EPI_ISL_1399235	A/chicken/Czech Republic/4527-1/2021	A / H5N8	Europe / Czech Republic / Stredocesky Kraj / Okres Mlada Boleslav / Brezno	Alexander Nagy (State Veterinary Institute Prague)
EPI_ISL_1399236	A/chicken/Czech Republic/4756/2021	A / H5N8	Europe / Czech Republic / Stredocesky Kraj / Okres Pribram / Pocaply, Breznice	Alexander Nagy (State Veterinary Institute Prague)
EPI_ISL_1399237	A/mute swan/Czech Republic/3549/2021	A / H5N8	Europe / Czech Republic / Stredocesky Kraj / Okres Nymburk / hydroelectric power Nymburk; GF	Alexander Nagy (State Veterinary Institute Prague)
EPI_ISL_1399238	A/mute swan/Czech Republic/3777/2021	A / H5N8	Europe / Czech Republic / Hlavni mesto Praha / Okres Praha / Kvetnice, Mlynsky rybnik; GPS: N 5	Alexander Nagy (State Veterinary Institute Prague)
EPI_ISL_1399239	A/mute swan/Czech Republic/4100/2021	A / H5N8	Europe / Czech Republic / Olomoucky Kraj / Okres Jesenik / Javornik; GPS: 50°24'35.8''N, 17°2'44	Alexander Nagy (State Veterinary Institute Prague)
EPI_ISL_1399240	A/mute swan/Czech Republic/4270/2021	A / H5N8	Europe / Czech Republic / Stredocesky Kraj / Okres Mlada Boleslav / rybnik Ohrada, Bosen	Alexander Nagy (State Veterinary Institute Prague)
EPI_ISL_1399241	A/chicken/Czech Republic/4526/2021	A / H5N8	Europe / Czech Republic / Morovskoslezsky Kraj / Okres Opava / Vyhon, Velke Hostice; GPS: N 49	Alexander Nagy (State Veterinary Institute Prague)

EPI_ISL_1399242	A/mute swan/Czech Republic/4606/2021	A / H5N8	Europe / Czech Republic / Zlinsky Kraj / Okres Uherske Hradiste / Ostrozska jezera, Ostrozska No	Alexander Nagy (State Veterinary Institute Prague)
EPI_ISL_1399243	A/mute swan/Czech Republic/4607-1/2021	A / H5N8	Europe / Czech Republic / Olomoucky Kraj / Okres Olomouc / Chomoutov	Alexander Nagy (State Veterinary Institute Prague)
EPI_ISL_1399244	A/mute swan/Czech Republic/4607-2/2021	A / H5N8	Europe / Czech Republic / Olomoucky Kraj / Okres Olomouc / Chomoutov	Alexander Nagy (State Veterinary Institute Prague)
EPI_ISL_1399245	A/mute swan/Czech Republic/4799/2021	A / H5N8	Europe / Czech Republic / Morovskoslezsky Kraj / Okres Opava / Katerinky u Opavy, Stribrne jeze	Alexander Nagy (State Veterinary Institute Prague)
EPI_ISL_14028104	A/Ciconia_ciconia/Spain/485-2_22VIR6312-1/2022	A / H5N1	Europe / Spain	Giacomo Barbierato (Istituto Zooprofilattico Sperimentale delle Venezie / Ricerca e innovazione)
EPI_ISL_14036187	A/chicken/Egypt/HS.1/2022	A / H5N8	Africa / Egypt / Monufia / 35 days chicken	Hazem Salah Khalil (Animal Health Research Institute (Egypt) / Poultry Diseases)
EPI_ISL_14036311	A/Duck/Egypt/HS.2/2022	A / H5N8	Africa / Egypt / Monufia	Hazem Salah Khalil (Animal Health Research Institute (Egypt) / Poultry Diseases)
EPI_ISL_14036428	A/Quail/Egypt/HS.3/2022	A / H5N8	Africa / Egypt / Monufia	Hazem Salah Khalil (Animal Health Research Institute (Egypt) / Poultry Diseases)
EPI_ISL_14037832	A/chicken/Egypt/HS.4/2022	A / H5N8	Africa / Egypt	Hazem Salah Khalil (Animal Health Research Institute (Egypt) / Poultry Diseases)
EPI_ISL_14037833	A/Duck/Egypt/HS.5/2022	A / H5N8	Africa / Egypt	Hazem Salah Khalil (Animal Health Research Institute (Egypt) / Poultry Diseases)
EPI_ISL_14037834	A/Quail/Egypt/HS.6/2022	A / H5N8	Africa / Egypt	Hazem Salah Khalil (Animal Health Research Institute (Egypt) / Poultry Diseases)
EPI_ISL_14064695	A/crow/Hokkaido/0101Q045/2022	A / H5N1	Asia / Japan / Hokkaido / Nemuro	Norikazu Isoda (Graduate School of Veterinary Medicine, Hokkaido University / Laboratory of Microbiology)
EPI_ISL_14064697	A/crow/Hokkaido/0101Q044/2022 (H5N1)	A / H5N1	Asia / Japan / Hokkaido / Nemuro	Norikazu Isoda (Graduate School of Veterinary Medicine, Hokkaido University / Laboratory of Microbiology)
EPI_ISL_14064698	A/crow/Hokkaido/0101Q054/2022	A / H5N1	Asia / Japan / Hokkaido / Nemuro	Norikazu Isoda (Graduate School of Veterinary Medicine, Hokkaido University / Laboratory of Microbiology)
EPI_ISL_14064699	A/crow/Hokkaido/0102F046/2022	A / H5N1	Asia / Japan / Hokkaido / Erimo	Norikazu Isoda (Graduate School of Veterinary Medicine, Hokkaido University / Laboratory of Microbiology)
EPI_ISL_14064768	A/crow/Hokkaido/0102F048/2022	A / H5N1	Asia / Japan / Hokkaido / Erimo	Norikazu Isoda (Graduate School of Veterinary Medicine, Hokkaido University / Laboratory of Microbiology)
EPI_ISL_14064803	A/crow/Hokkaido/0102F043/2022	A / H5N1	Asia / Japan / Hokkaido / Erimo	Norikazu Isoda (Graduate School of Veterinary Medicine, Hokkaido University / Laboratory of Microbiology)
EPI_ISL_14064890	A/crow/Hokkaido/0103B073/2022 (H5N1)	A / H5N1	Asia / Japan / Hokkaido / Sapporo	Norikazu Isoda (Graduate School of Veterinary Medicine, Hokkaido University / Laboratory of Microbiology)
EPI_ISL_14064983	A/chicken/Scotland/093091/2022	A / H5N1	Europe / United Kingdom / Scotland	Alex Byrne (Animal and Plant Health Agency (APHA) / Virology Department)
EPI_ISL_14064984	A/chicken/England/093459/2022	A / H5N1	Europe / United Kingdom / England	Alex Byrne (Animal and Plant Health Agency (APHA) / Virology Department)
EPI_ISL_14064985	A/Domestic_goose/England/093469/2022	A / H5N1	Europe / United Kingdom / England	Alex Byrne (Animal and Plant Health Agency (APHA) / Virology Department)
EPI_ISL_14064986	A/crow/Hokkaido/0102L010/2022	A / H5N1	Asia / Japan / Hokkaido / Rishirifuji	Norikazu Isoda (Graduate School of Veterinary Medicine, Hokkaido University / Laboratory of Microbiology)
EPI_ISL_14064988	A/crow/Hokkaido/0102M086/2022	A / H5N1	Asia / Japan / Hokkaido / Syari	Norikazu Isoda (Graduate School of Veterinary Medicine, Hokkaido University / Laboratory of Microbiology)
EPI_ISL_14065167	A/white-tailed eagle/Hokkaido/20220210001/2022	A / H5N1	Asia / Japan / Hokkaido / Rausu	Norikazu Isoda (Graduate School of Veterinary Medicine, Hokkaido University / Laboratory of Microbiology)
EPI_ISL_14070455	A/tanuki/Hokkaido/1/2022	A / H5N1	Asia / Japan / Hokkaido / Sapporo	Norikazu Isoda (Graduate School of Veterinary Medicine, Hokkaido University / Laboratory of Microbiology)
EPI_ISL_14098915	A/harbor seal/Maine/22-020455-001-original/2022	A / H5N1	North America / United States / Maine / Cumberland County	Mary Lea Killian (National Veterinary Services Laboratories - USDA / Diagnostic Virology Laboratory)
EPI_ISL_14098916	A/harbor seal/Maine/22-020455-002-original/2022	A / H5N1	North America / United States / Maine / Cumberland County	Mary Lea Killian (National Veterinary Services Laboratories - USDA / Diagnostic Virology Laboratory)
EPI_ISL_14098917	A/harbor seal/Maine/22-020455-003-original/2022	A / H5N1	North America / United States / Maine / York County	Mary Lea Killian (National Veterinary Services Laboratories - USDA / Diagnostic Virology Laboratory)
EPI_ISL_14098918	A/harbor seal/Maine/22-020455-004-original/2022	A / H5N1	North America / United States / Maine / Sagadahoc County	Mary Lea Killian (National Veterinary Services Laboratories - USDA / Diagnostic Virology Laboratory)
EPI_ISL_14098919	A/harbor seal/Maine/22-020455-005-original/2022	A / H5N1	North America / United States / Maine / Lincoln County	Mary Lea Killian (National Veterinary Services Laboratories - USDA / Diagnostic Virology Laboratory)
EPI_ISL_14098920	A/harbor seal/Maine/22-020983-001-original/2022	A / H5N1	North America / United States / Maine / Cumberland County	Mary Lea Killian (National Veterinary Services Laboratories - USDA / Diagnostic Virology Laboratory)
EPI_ISL_14098921	A/harbor seal/Maine/22-020983-002-original/2022	A / H5N1	North America / United States / Maine / Cumberland County	Mary Lea Killian (National Veterinary Services Laboratories - USDA / Diagnostic Virology Laboratory)
EPI_ISL_14098922	A/grey seal/Maine/22-020983-003-original/2022	A / H5N1	North America / United States / Maine / Sagadahoc County	Mary Lea Killian (National Veterinary Services Laboratories - USDA / Diagnostic Virology Laboratory)
EPI_ISL_14098923	A/harbor seal/Maine/22-020983-006-original/2022	A / H5N1	North America / United States / Maine / Cumberland County	Mary Lea Killian (National Veterinary Services Laboratories - USDA / Diagnostic Virology Laboratory)
EPI_ISL_14098924	A/harbor seal/Maine/22-020983-007-original/2022	A / H5N1	North America / United States / Maine / Penobscot County / Plymouth County	Mary Lea Killian (National Veterinary Services Laboratories - USDA / Diagnostic Virology Laboratory)
EPI_ISL_14163711	A/Common Tern/Netherlands/10/2022	A / H5N1	Europe / Netherlands / Noordburen, Waddenkust	Sanne Thewessen (Erasmus Medical Center / Viroscience)
EPI_ISL_14163712	A/European Herring Gull/Netherlands/8/2022	A / H5N1	Europe / Netherlands / Noordburen, Waddenkust	Sanne Thewessen (Erasmus Medical Center / Viroscience)
EPI_ISL_14163713	A/Caspian Gull/Netherlands/5/2022	A / H5N1	Europe / Netherlands / Enkhuizen, Ijsselmeer, De Kreupel	Sanne Thewessen (Erasmus Medical Center / Viroscience)
EPI_ISL_14163714	A/European Herring Gull/Netherlands/9/2022	A / H5N1	Europe / Netherlands / Ijmuiden, Forteiland	Sanne Thewessen (Erasmus Medical Center / Viroscience)
EPI_ISL_14163715	A/Greylag Goose/Netherlands/9/2022	A / H5N1	Europe / Netherlands / Inlaag, Neeltje Jans	Sanne Thewessen (Erasmus Medical Center / Viroscience)
EPI_ISL_14171729	A/Eurasian Spoonbill/Netherlands/1A/2022	A / H5N1	Europe / Netherlands / Schiermonnikoog	Sanne Thewessen (Erasmus Medical Center / Viroscience)
EPI_ISL_14171741	A/Eurasian Spoonbill/Netherlands/1B/2022	A / H5N1	Europe / Netherlands / Schiermonnikoog	Sanne Thewessen (Erasmus Medical Center / Viroscience)
EPI_ISL_14174187	A/chicken/China/007/2019	A / H5N6	Asia / China	
EPI_ISL_14174306	A/goose/China/0701/2018	A / H5N6	Asia / China	
EPI_ISL_14174436	A/mink/China/181/2019	A / H5N6	Asia / China	
EPI_ISL_14174437	A/mink/China/183/2019	A / H5N6	Asia / China	
EPI_ISL_14174476	A/mink/China/191/2019	A / H5N6	Asia / China	
EPI_ISL_14174522	A/mink/China/456/2018	A / H5N6	Asia / China	
EPI_ISL_14174523	A/swine/China/RZ/2018	A / H5N6	Asia / China	
EPI_ISL_1420637	A/wigeon/Latvia/23903/2021	A / H5N8	Europe / Latvia / Jurmala	Juris Kibilds (Institute of Food Safety, Animal Health and Environment BIOR / Laboratory of Microbiology and Pathology)
EPI_ISL_14224170	A/chicken-Cobb/Egypt/55/2019	A / H5N8	Africa / Egypt	
EPI_ISL_14224174	A/chicken/Egypt/Dakahlia segment 4 HA gene/2021	A / H5N8	Africa / Egypt	
EPI_ISL_14233919	A/Eurasian Spoonbill/Netherlands/3/2022	A / H5N1	Europe / Netherlands / Schiermonnikoog, Oosterkwelder	Sanne Thewessen (Erasmus Medical Center / Viroscience)
EPI_ISL_14233920	A/Common Tern/Netherlands/17/2022	A / H5N1	Europe / Netherlands / Enkhuizen, Ijsselmeer, De Kreupel	Sanne Thewessen (Erasmus Medical Center / Viroscience)
EPI_ISL_14233921	A/Common Tern/Netherlands/18/2022	A / H5N1	Europe / Netherlands / Enkhuizen, Ijsselmeer, De Kreupel	Sanne Thewessen (Erasmus Medical Center / Viroscience)
EPI_ISL_14233922	A/European Herring Gull/Netherlands/11/2022	A / H5N1	Europe / Netherlands / Den Oever, Het Schor	Sanne Thewessen (Erasmus Medical Center / Viroscience)
EPI_ISL_14233923	A/Caspian Gull/Netherlands/7/2022	A / H5N1	Europe / Netherlands / Enkhuizen, Ijsselmeer, De Kreupel	Sanne Thewessen (Erasmus Medical Center / Viroscience)
EPI_ISL_14233924	A/Common Tern/Netherlands/19/2022	A / H5N1	Europe / Netherlands / Enkhuizen, Ijsselmeer, De Kreupel	Sanne Thewessen (Erasmus Medical Center / Viroscience)
EPI_ISL_14233925	A/Common Tern/Netherlands/20/2022	A / H5N1	Europe / Netherlands / Enkhuizen, Ijsselmeer, De Kreupel	Sanne Thewessen (Erasmus Medical Center / Viroscience)
EPI_ISL_14233926	A/European Herring Gull/Netherlands/10/2022	A / H5N1	Europe / Netherlands / Den Oever, Waddenhaven	Sanne Thewessen (Erasmus Medical Center / Viroscience)
EPI_ISL_14233927	A/Common Tern/Netherlands/11/2022	A / H5N1	Europe / Netherlands / Wieringerwerf, Noorderdijkweg	Sanne Thewessen (Erasmus Medical Center / Viroscience)
EPI_ISL_14233928	A/Common Tern/Netherlands/13/2022	A / H5N1	Europe / Netherlands / Wieringerwerf, Noorderdijkweg	Sanne Thewessen (Erasmus Medical Center / Viroscience)
EPI_ISL_14233929	A/Common Tern/Netherlands/12/2022	A / H5N1	Europe / Netherlands / Wieringerwerf, Noorderdijkweg	Sanne Thewessen (Erasmus Medical Center / Viroscience)
EPI_ISL_14233930	A/Common Tern/Netherlands/14/2022	A / H5N1	Europe / Netherlands / Wieringerwerf, Noorderdijkweg	Sanne Thewessen (Erasmus Medical Center / Viroscience)
EPI_ISL_14233931	A/Common Tern/Netherlands/15/2022	A / H5N1	Europe / Netherlands / Wieringerwerf, Noorderdijkweg	Sanne Thewessen (Erasmus Medical Center / Viroscience)
EPI_ISL_14233932	A/Common Tern/Netherlands/16/2022	A / H5N1	Europe / Netherlands / Wieringerwerf, Noorderdijkweg	Sanne Thewessen (Erasmus Medical Center / Viroscience)
EPI_ISL_14233944	A/Caspian Gull/Netherlands/6/2022	A / H5N1	Europe / Netherlands / Enkhuizen, Ijsselmeer, De Kreupel	Sanne Thewessen (Erasmus Medical Center / Viroscience)
EPI_ISL_14388346	A/Tyto_alba/Belgium/334_0012/2021	A / H5N1	Europe / Belgium / Provincie West-Vlaanderen / Waarschoot	Steven Van Borm (Sciensano, Department of Animal Infectious Diseases / Animal Infectious Diseases)
EPI_ISL_14388434	A/Buteo_buteo/belgium/334_0013/2021	A / H5N1	Europe / Belgium / Provincie West-Vlaanderen / Desselgem	Steven Van Borm (Sciensano, Department of Animal Infectious Diseases / Animal Infectious Diseases)
EPI_ISL_14389133	A/Larus_argentatus/Belgium/595_0008/2022	A / H5N1	Europe / Belgium / Provincie Antwerpen / Kapellen	Steven Van Borm (Sciensano, Department of Animal Infectious Diseases / Animal Infectious Diseases)
EPI_ISL_14389148	A/Tachybaptus_ruficollis/Belgium/1234_0008/2022	A / H5N1	Europe / Belgium / Provincie Vlaams-Brabant / Oud-Heverlee	Steven Van Borm (Sciensano, Department of Animal Infectious Diseases / Animal Infectious Diseases)
EPI_ISL_14389524	A/Anser_anser_domesticus/Belgium/1668_0016/2022	A / H5N1	Europe / Belgium / Provincie Vlaams-Brabant / Oud-Heverlee	Steven Van Borm (Sciensano, Department of Animal Infectious Diseases / Animal Infectious Diseases)

EPI_ISL_14390100	A/Larus_canus/Belgium/1668_0019/2022	A / H5N1	Europe / Belgium / Provincie Antwerpen / Berchem	Steven Van Borm (Sciensano, Department of Animal Infectious Diseases / Animal Infectious Diseases)
EPI_ISL_14390385	A/Phalacrocorax_carbo/Belgium/1734_0002/2022	A / H5N1	Europe / Belgium / Provincie Oost-Vlaanderen / Evergem	Steven Van Borm (Sciensano, Department of Animal Infectious Diseases / Animal Infectious Diseases)
EPI_ISL_14391865	A/Buteo_buteo/Belgium/2606_0006/2022	A / H5N1	Europe / Belgium / Provincie Antwerpen / Kontich	Steven Van Borm (Sciensano, Department of Animal Infectious Diseases / Animal Infectious Diseases)
EPI_ISL_14392127	A/Branta_leucopsis/Belgium/2606_0009/2022	A / H5N1	Europe / Belgium / Provincie West-Vlaanderen / Blankenberge	Steven Van Borm (Sciensano, Department of Animal Infectious Diseases / Animal Infectious Diseases)
EPI_ISL_14393097	A/Fox/Netherlands/EMC4/2022	A / H5N1	Europe / Netherlands / Den Haag	Sanne Thewessen (Erasmus Medical Center / Viroscience)
EPI_ISL_14393115	A/Fox/Netherlands/EMC5/2022	A / H5N1	Europe / Netherlands / Oosterbeek	Sanne Thewessen (Erasmus Medical Center / Viroscience)
EPI_ISL_14393126	A/Fox/Netherlands/EMC6/2022	A / H5N1	Europe / Netherlands / Leusden	Sanne Thewessen (Erasmus Medical Center / Viroscience)
EPI_ISL_14393465	A/Branta_canadensis/Belgium/4821_0001/2022	A / H5N1	Europe / Belgium / Province de Liege / Visé	Steven Van Borm (Sciensano, Department of Animal Infectious Diseases / Animal Infectious Diseases)
EPI_ISL_14393671	A/Branta_canadensis/Belgium/5177_0003/2022	A / H5N1	Europe / Belgium / Province de Liege / Visé	Steven Van Borm (Sciensano, Department of Animal Infectious Diseases / Animal Infectious Diseases)
EPI_ISL_14469689	A/Common Tern/Netherlands/21/2022	A / H5N1	Europe / Netherlands / Enkhuizen, Ijsselmeer, De Kreupel	Sanne Thewessen (Erasmus Medical Center / Viroscience)
EPI_ISL_14469690	A/Common Tern/Netherlands/22/2022	A / H5N1	Europe / Netherlands / Enkhuizen, Ijsselmeer, De Kreupel	Sanne Thewessen (Erasmus Medical Center / Viroscience)
EPI_ISL_14469691	A/Common Tern/Netherlands/23/2022	A / H5N1	Europe / Netherlands / Enkhuizen, Ijsselmeer, De Kreupel	Sanne Thewessen (Erasmus Medical Center / Viroscience)
EPI_ISL_14469692	A/Common Tern/Netherlands/24/2022	A / H5N1	Europe / Netherlands / Enkhuizen, Ijsselmeer, De Kreupel	Sanne Thewessen (Erasmus Medical Center / Viroscience)
EPI_ISL_14469693	A/Common Tern/Netherlands/25/2022	A / H5N1	Europe / Netherlands / Marker Wadden	Sanne Thewessen (Erasmus Medical Center / Viroscience)
EPI_ISL_14493899	A/Vulpes_vulpes/Belgium/8660_0016/2022	A / H5N1	Europe / Belgium / Provincie Vlaams-Brabant / Kortenberg	Steven Van Borm (Sciensano, Department of Animal Infectious Diseases / Animal Infectious Diseases)
EPI_ISL_14494351	A/Larus_argentatus/Belgium/9013_0001/2022	A / H5N1	Europe / Belgium / Provincie Oost-Vlaanderen / Gent	Steven Van Borm (Sciensano, Department of Animal Infectious Diseases / Animal Infectious Diseases)
EPI_ISL_14494796	A/Vulpes_vulpes/Belgium/9031_0008/2022	A / H5N1	Europe / Belgium / Provincie West-Vlaanderen / Knokke-Heist	Steven Van Borm (Sciensano, Department of Animal Infectious Diseases / Animal Infectious Diseases)
EPI_ISL_14494933	A/Gallus_gallus/Belgium/9548_0001/2022	A / H5N1	Europe / Belgium / Provincie West-Vlaanderen / Roeselare	Steven Van Borm (Sciensano, Department of Animal Infectious Diseases / Animal Infectious Diseases)
EPI_ISL_14497321	A/Eurasian Spoonbill/Netherlands/4/2022	A / H5N1	Europe / Netherlands / Werkeiland, Neeltje Jans	Sanne Thewessen (Erasmus Medical Center / Viroscience)
EPI_ISL_14497837	A/domestic_duck/England/100990/2022	A / H5N1	Europe / United Kingdom / England	Alex Byrne (Animal and Plant Health Agency (APHA) / Virology Department)
EPI_ISL_14497855	A/domestic_duck/England/104859/2022	A / H5N1	Europe / United Kingdom / England	Alex Byrne (Animal and Plant Health Agency (APHA) / Virology Department)
EPI_ISL_14497870	A/domestic_duck/England/105414/2022	A / H5N1	Europe / United Kingdom / England	Alex Byrne (Animal and Plant Health Agency (APHA) / Virology Department)
EPI_ISL_14497885	A/domestic_goose/England/105863/2022	A / H5N1	Europe / United Kingdom / England	Alex Byrne (Animal and Plant Health Agency (APHA) / Virology Department)
EPI_ISL_14533193	A/fox/New_York/074441/2022	A / H5N1	North America / United States / New York / Cayuga County	Brittany D Cronk (Cornell University / Population Medicine and Diagnostics)
EPI_ISL_14551957	A/fox/New_York/088592/2022	A / H5N1	North America / United States / New York / Saint Lawrence County	Brittany D Cronk (Cornell University / Population Medicine and Diagnostics)
EPI_ISL_14552806	A/fox/New_York/095525/2022	A / H5N1	North America / United States / New York / Saint Lawrence County	Brittany D Cronk (Cornell University / Population Medicine and Diagnostics)
EPI_ISL_14552975	A/fox/New_York/099451/2022	A / H5N1	North America / United States / New York / Erie County	Brittany D Cronk (Cornell University / Population Medicine and Diagnostics)
EPI_ISL_14553810	A/fox/New_York/099488/2022	A / H5N1	North America / United States / New York / Cayuga County	Brittany D Cronk (Cornell University / Population Medicine and Diagnostics)
EPI_ISL_14553811	A/fox/New_York/103994/2022	A / H5N1	North America / United States / New York / Niagara County	Brittany D Cronk (Cornell University / Population Medicine and Diagnostics)
EPI_ISL_14553813	A/fox/New_York/107242/2022	A / H5N1	North America / United States / New York / Allegany County	Brittany D Cronk (Cornell University / Population Medicine and Diagnostics)
EPI_ISL_14553997	A/fox/New_York/115912/2022	A / H5N1	North America / United States / New York / Allegany County	Brittany D Cronk (Cornell University / Population Medicine and Diagnostics)
EPI_ISL_14604371	A/crow/Hokkaido/0101Q061/2022	A / H5N1	Asia / Japan / Hokkaido / Rausu	Norikazu Isoda (Graduate School of Veterinary Medicine, Hokkaido University / Laboratory of Microbiology)
EPI_ISL_14615026	A/crow/Hokkaido/0102L015/2022	A / H5N1	Asia / Japan / Hokkaido / Rebun	Norikazu Isoda (Graduate School of Veterinary Medicine, Hokkaido University / Laboratory of Microbiology)
EPI_ISL_14615027	A/crow/Hokkaido/0103L018/2022	A / H5N1	Asia / Japan / Hokkaido / Hamatonbetsu	Norikazu Isoda (Graduate School of Veterinary Medicine, Hokkaido University / Laboratory of Microbiology)
EPI_ISL_14615036	A/crow/Hokkaido/0101Q056/2022	A / H5N1	Asia / Japan / Hokkaido / Shibetsu	Norikazu Isoda (Graduate School of Veterinary Medicine, Hokkaido University / Laboratory of Microbiology)
EPI_ISL_14702899	A/nothern_gannet/France/22P019331/2022	A / H5N1	Europe / France / Brittany / Departement des Cotes-d'Armor	Francois-Xavier Briand (ANSES Agence Nationale De Securite Sanitaire De L'alimentation / Laboratoire de Ploufragan-Plouzané)
EPI_ISL_14722960	A/vulture/France/22P018210/2022	A / H5N1	Europe / France / Rhone-Alpes / Departement de la Drome	Francois-Xavier Briand (ANSES Agence Nationale De Securite Sanitaire De L'alimentation / Laboratoire de Ploufragan-Plouzané)
EPI_ISL_14749595	A/duck/China/DK09-HA.seq/2016	A / H5N6	Asia / China	
EPI_ISL_14749603	A/goose/China/GS38-HA.seq/2016	A / H5N6	Asia / China	
EPI_ISL_14760542	A/turkey/Italy/21VIR8728-1/2021	A / H5N1	Europe / Italy	Giacomo Barbierato (Istituto Zooprofilattico Sperimentale delle Venezie / Ricerca e innovazione)
EPI_ISL_14760543	A/turkey/Italy/21VIR8816-1/2021	A / H5N1	Europe / Italy	Giacomo Barbierato (Istituto Zooprofilattico Sperimentale delle Venezie / Ricerca e innovazione)
EPI_ISL_14760544	A/turkey/Italy/21VIR8925-6/2021	A / H5N1	Europe / Italy	Giacomo Barbierato (Istituto Zooprofilattico Sperimentale delle Venezie / Ricerca e innovazione)
EPI_ISL_14760545	A/turkey/Italy/21VIR8926-1/2021	A / H5N1	Europe / Italy	Giacomo Barbierato (Istituto Zooprofilattico Sperimentale delle Venezie / Ricerca e innovazione)
EPI_ISL_14760546	A/turkey/Italy/21VIR8926-2/2021	A / H5N1	Europe / Italy	Giacomo Barbierato (Istituto Zooprofilattico Sperimentale delle Venezie / Ricerca e innovazione)
EPI_ISL_14760547	A/turkey/Italy/21VIR9073-3/2021	A / H5N1	Europe / Italy	Giacomo Barbierato (Istituto Zooprofilattico Sperimentale delle Venezie / Ricerca e innovazione)
EPI_ISL_14760548	A/chicken/Italy/21VIR9074-10/2021	A / H5N1	Europe / Italy	Giacomo Barbierato (Istituto Zooprofilattico Sperimentale delle Venezie / Ricerca e innovazione)
EPI_ISL_14760549	A/turkey/Italy/21VIR9144-2/2021	A / H5N1	Europe / Italy	Giacomo Barbierato (Istituto Zooprofilattico Sperimentale delle Venezie / Ricerca e innovazione)
EPI_ISL_14760550	A/chicken/Italy/21VIR9133-21/2021	A / H5N1	Europe / Italy	Giacomo Barbierato (Istituto Zooprofilattico Sperimentale delle Venezie / Ricerca e innovazione)
EPI_ISL_14760551	A/turkey/Italy/21VIR9209-1/2021	A / H5N1	Europe / Italy	Giacomo Barbierato (Istituto Zooprofilattico Sperimentale delle Venezie / Ricerca e innovazione)
EPI_ISL_14760552	A/chicken/Italy/21VIR9212-1/2021	A / H5N1	Europe / Italy	Giacomo Barbierato (Istituto Zooprofilattico Sperimentale delle Venezie / Ricerca e innovazione)
EPI_ISL_14760553	A/turkey/Italy/21VIR9211-1/2021	A / H5N1	Europe / Italy	Giacomo Barbierato (Istituto Zooprofilattico Sperimentale delle Venezie / Ricerca e innovazione)
EPI_ISL_14760554	A/turkey/Italy/21VIR9217-1/2021	A / H5N1	Europe / Italy	Giacomo Barbierato (Istituto Zooprofilattico Sperimentale delle Venezie / Ricerca e innovazione)
EPI_ISL_14760555	A/turkey/Italy/21VIR9213-1/2021	A / H5N1	Europe / Italy	Giacomo Barbierato (Istituto Zooprofilattico Sperimentale delle Venezie / Ricerca e innovazione)
EPI_ISL_14760556	A/turkey/Italy/21VIR9215-1/2021	A / H5N1	Europe / Italy	Giacomo Barbierato (Istituto Zooprofilattico Sperimentale delle Venezie / Ricerca e innovazione)
EPI_ISL_14760557	A/chicken/Italy/21VIR9219-6/2021	A / H5N1	Europe / Italy	Giacomo Barbierato (Istituto Zooprofilattico Sperimentale delle Venezie / Ricerca e innovazione)
EPI_ISL_14760558	A/chicken/Italy/21VIR9371-1/2021	A / H5N1	Europe / Italy	Giacomo Barbierato (Istituto Zooprofilattico Sperimentale delle Venezie / Ricerca e innovazione)
EPI_ISL_14760559	A/avian/Italy/21VIR9425-2/2021	A / H5N1	Europe / Italy	Giacomo Barbierato (Istituto Zooprofilattico Sperimentale delle Venezie / Ricerca e innovazione)
EPI_ISL_14760560	A/turkey/Italy/21VIR9372-1/2021	A / H5N1	Europe / Italy	Giacomo Barbierato (Istituto Zooprofilattico Sperimentale delle Venezie / Ricerca e innovazione)
EPI_ISL_14760561	A/quail/Italy/21VIR9474-1/2021	A / H5N1	Europe / Italy	Giacomo Barbierato (Istituto Zooprofilattico Sperimentale delle Venezie / Ricerca e innovazione)
EPI_ISL_14760562	A/duck/Italy/21VIR9373-2/2021	A / H5N1	Europe / Italy	Giacomo Barbierato (Istituto Zooprofilattico Sperimentale delle Venezie / Ricerca e innovazione)
EPI_ISL_14760563	A/turkey/Italy/21VIR9426-3/2021	A / H5N1	Europe / Italy	Giacomo Barbierato (Istituto Zooprofilattico Sperimentale delle Venezie / Ricerca e innovazione)
EPI_ISL_14760564	A/turkey/Italy/21VIR9476-1/2021	A / H5N1	Europe / Italy	Giacomo Barbierato (Istituto Zooprofilattico Sperimentale delle Venezie / Ricerca e innovazione)
EPI_ISL_14760565	A/turkey/Italy/21VIR9475-1/2021	A / H5N1	Europe / Italy	Giacomo Barbierato (Istituto Zooprofilattico Sperimentale delle Venezie / Ricerca e innovazione)
EPI_ISL_14760566	A/chicken/Italy/21VIR9509-1/2021	A / H5N1	Europe / Italy	Giacomo Barbierato (Istituto Zooprofilattico Sperimentale delle Venezie / Ricerca e innovazione)
EPI_ISL_14760567	A/chicken/Italy/21VIR9507-7/2021	A / H5N1	Europe / Italy	Giacomo Barbierato (Istituto Zooprofilattico Sperimentale delle Venezie / Ricerca e innovazione)
EPI_ISL_14760568	A/chicken/Italy/21VIR9508-3/2021	A / H5N1	Europe / Italy	Giacomo Barbierato (Istituto Zooprofilattico Sperimentale delle Venezie / Ricerca e innovazione)
EPI_ISL_14760569	A/turkey/Italy/21VIR9607-2/2021	A / H5N1	Europe / Italy	Giacomo Barbierato (Istituto Zooprofilattico Sperimentale delle Venezie / Ricerca e innovazione)
EPI_ISL_14760570	A/turkey/Italy/21VIR9606-2/2021	A / H5N1	Europe / Italy	Giacomo Barbierato (Istituto Zooprofilattico Sperimentale delle Venezie / Ricerca e innovazione)
EPI_ISL_14760571	A/turkey/Italy/21VIR9604-3/2021	A / H5N1	Europe / Italy	Giacomo Barbierato (Istituto Zooprofilattico Sperimentale delle Venezie / Ricerca e innovazione)
EPI_ISL_14760572	A/turkey/Italy/21VIR9609-2/2021	A / H5N1	Europe / Italy	Giacomo Barbierato (Istituto Zooprofilattico Sperimentale delle Venezie / Ricerca e innovazione)
EPI_ISL_14760573	A/turkey/Italy/21VIR9605-4/2021	A / H5N1	Europe / Italy	Giacomo Barbierato (Istituto Zooprofilattico Sperimentale delle Venezie / Ricerca e innovazione)
EPI_ISL_14760574	A/turkey/Italy/21VIR9608-2/2021	A / H5N1	Europe / Italy	Giacomo Barbierato (Istituto Zooprofilattico Sperimentale delle Venezie / Ricerca e innovazione)

EPI_ISL_14760575	A/quail/Italy/21VIR9515-1/2021	A / H5N1	Europe / Italy	Giacomo Barbierato (Istituto Zooprofilattico Sperimentale delle Venezie / Ricerca e innovazione)
EPI_ISL_14760576	A/turkey/Italy/21VIR9518-1/2021	A / H5N1	Europe / Italy	Giacomo Barbierato (Istituto Zooprofilattico Sperimentale delle Venezie / Ricerca e innovazione)
EPI_ISL_14760577	A/turkey/Italy/21VIR9519-1/2021	A / H5N1	Europe / Italy	Giacomo Barbierato (Istituto Zooprofilattico Sperimentale delle Venezie / Ricerca e innovazione)
EPI_ISL_14760578	A/turkey/Italy/21VIR9520-1/2021	A / H5N1	Europe / Italy	Giacomo Barbierato (Istituto Zooprofilattico Sperimentale delle Venezie / Ricerca e innovazione)
EPI_ISL_14760579	A/turkey/Italy/21VIR9643-2/2021	A / H5N1	Europe / Italy	Giacomo Barbierato (Istituto Zooprofilattico Sperimentale delle Venezie / Ricerca e innovazione)
EPI_ISL_14760580	A/turkey/Italy/21VIR9644-2/2021	A / H5N1	Europe / Italy	Giacomo Barbierato (Istituto Zooprofilattico Sperimentale delle Venezie / Ricerca e innovazione)
EPI_ISL_14760581	A/turkey/Italy/21VIR9645-2/2021	A / H5N1	Europe / Italy	Giacomo Barbierato (Istituto Zooprofilattico Sperimentale delle Venezie / Ricerca e innovazione)
EPI_ISL_14760582	A/chicken/Italy/21VIR9580-23/2021	A / H5N1	Europe / Italy	Giacomo Barbierato (Istituto Zooprofilattico Sperimentale delle Venezie / Ricerca e innovazione)
EPI_ISL_14760583	A/turkey/Italy/21VIR9579-10/2021	A / H5N1	Europe / Italy	Giacomo Barbierato (Istituto Zooprofilattico Sperimentale delle Venezie / Ricerca e innovazione)
EPI_ISL_14760584	A/turkey/Italy/21VIR9648-2/2021	A / H5N1	Europe / Italy	Giacomo Barbierato (Istituto Zooprofilattico Sperimentale delle Venezie / Ricerca e innovazione)
EPI_ISL_14760585	A/turkey/Italy/21VIR9651-2/2021	A / H5N1	Europe / Italy	Giacomo Barbierato (Istituto Zooprofilattico Sperimentale delle Venezie / Ricerca e innovazione)
EPI_ISL_14760586	A/turkey/Italy/21VIR9650-2/2021	A / H5N1	Europe / Italy	Giacomo Barbierato (Istituto Zooprofilattico Sperimentale delle Venezie / Ricerca e innovazione)
EPI_ISL_14760587	A/turkey/Italy/21VIR9646-2/2021	A / H5N1	Europe / Italy	Giacomo Barbierato (Istituto Zooprofilattico Sperimentale delle Venezie / Ricerca e innovazione)
EPI_ISL_14760588	A/turkey/Italy/21VIR9647-2/2021	A / H5N1	Europe / Italy	Giacomo Barbierato (Istituto Zooprofilattico Sperimentale delle Venezie / Ricerca e innovazione)
EPI_ISL_14760589	A/turkey/Italy/21VIR9694-2/2021	A / H5N1	Europe / Italy	Giacomo Barbierato (Istituto Zooprofilattico Sperimentale delle Venezie / Ricerca e innovazione)
EPI_ISL_14760590	A/turkey/Italy/21VIR9692-16/2021	A / H5N1	Europe / Italy	Giacomo Barbierato (Istituto Zooprofilattico Sperimentale delle Venezie / Ricerca e innovazione)
EPI_ISL_14760591	A/turkey/Italy/21VIR9695-4/2021	A / H5N1	Europe / Italy	Giacomo Barbierato (Istituto Zooprofilattico Sperimentale delle Venezie / Ricerca e innovazione)
EPI_ISL_14760592	A/turkey/Italy/21VIR9693-3/2021	A / H5N1	Europe / Italy	Giacomo Barbierato (Istituto Zooprofilattico Sperimentale delle Venezie / Ricerca e innovazione)
EPI_ISL_14760593	A/turkey/Italy/21VIR9696-2/2021	A / H5N1	Europe / Italy	Giacomo Barbierato (Istituto Zooprofilattico Sperimentale delle Venezie / Ricerca e innovazione)
EPI_ISL_14760594	A/turkey/Italy/21VIR9697-3_/2022	A / H5N1	Europe / Italy	Giacomo Barbierato (Istituto Zooprofilattico Sperimentale delle Venezie / Ricerca e innovazione)
EPI_ISL_14760595	A/turkey/Italy/21VIR9769-4/2021	A / H5N1	Europe / Italy	Giacomo Barbierato (Istituto Zooprofilattico Sperimentale delle Venezie / Ricerca e innovazione)
EPI_ISL_14760596	A/turkey/Italy/21VIR9766-3/2021	A / H5N1	Europe / Italy	Giacomo Barbierato (Istituto Zooprofilattico Sperimentale delle Venezie / Ricerca e innovazione)
EPI_ISL_14760597	A/chicken/Italy/21VIR9868-8/2021	A / H5N1	Europe / Italy	Giacomo Barbierato (Istituto Zooprofilattico Sperimentale delle Venezie / Ricerca e innovazione)
EPI_ISL_14760598	A/chicken/Italy/21VIR9902-2/2021	A / H5N1	Europe / Italy	Giacomo Barbierato (Istituto Zooprofilattico Sperimentale delle Venezie / Ricerca e innovazione)
EPI_ISL_14760599	A/turkey/Italy/21VIR9870-3/2021	A / H5N1	Europe / Italy	Giacomo Barbierato (Istituto Zooprofilattico Sperimentale delle Venezie / Ricerca e innovazione)
EPI_ISL_14760600	A/turkey/Italy/21VIR9869-4/2021	A / H5N1	Europe / Italy	Giacomo Barbierato (Istituto Zooprofilattico Sperimentale delle Venezie / Ricerca e innovazione)
EPI_ISL_14760601	A/turkey/Italy/21VIR9903-2/2021	A / H5N1	Europe / Italy	Giacomo Barbierato (Istituto Zooprofilattico Sperimentale delle Venezie / Ricerca e innovazione)
EPI_ISL_14760602	A/chicken/Italy/21VIR9830-27/2021	A / H5N1	Europe / Italy	Giacomo Barbierato (Istituto Zooprofilattico Sperimentale delle Venezie / Ricerca e innovazione)
EPI_ISL_14760603	A/chicken/Italy/21VIR9904-16/2021	A / H5N1	Europe / Italy	Giacomo Barbierato (Istituto Zooprofilattico Sperimentale delle Venezie / Ricerca e innovazione)
EPI_ISL_14760604	A/chicken/Italy/21VIR9904-17/2021	A / H5N1	Europe / Italy	Giacomo Barbierato (Istituto Zooprofilattico Sperimentale delle Venezie / Ricerca e innovazione)
EPI_ISL_14760605	A/chicken/Italy/21VIR9904-18/2021	A / H5N1	Europe / Italy	Giacomo Barbierato (Istituto Zooprofilattico Sperimentale delle Venezie / Ricerca e innovazione)
EPI_ISL_14760606	A/turkey/Italy/21VIR9906-2/2021	A / H5N1	Europe / Italy	Giacomo Barbierato (Istituto Zooprofilattico Sperimentale delle Venezie / Ricerca e innovazione)
EPI_ISL_14760607	A/turkey/Italy/21VIR9905-2/2021	A / H5N1	Europe / Italy	Giacomo Barbierato (Istituto Zooprofilattico Sperimentale delle Venezie / Ricerca e innovazione)
EPI_ISL_14760608	A/duck/Italy/21VIR9836-15/2021	A / H5N1	Europe / Italy	Giacomo Barbierato (Istituto Zooprofilattico Sperimentale delle Venezie / Ricerca e innovazione)
EPI_ISL_14760609	A/turkey/Italy/21VIR9839-1/2021	A / H5N1	Europe / Italy	Giacomo Barbierato (Istituto Zooprofilattico Sperimentale delle Venezie / Ricerca e innovazione)
EPI_ISL_14760610	A/chicken/Italy/21VIR9837-3/2021	A / H5N1	Europe / Italy	Giacomo Barbierato (Istituto Zooprofilattico Sperimentale delle Venezie / Ricerca e innovazione)
EPI_ISL_14760611	A/goose/Italy/IZSLT_21VIR10273/2021	A / H5N1	Europe / Italy	Giacomo Barbierato (Istituto Zooprofilattico Sperimentale delle Venezie / Ricerca e innovazione)
EPI_ISL_14760612	A/chicken/Italy/21VIR9858-3/2021	A / H5N1	Europe / Italy	Giacomo Barbierato (Istituto Zooprofilattico Sperimentale delle Venezie / Ricerca e innovazione)
EPI_ISL_14760613	A/turkey/Italy/21VIR9889-1/2021	A / H5N1	Europe / Italy	Giacomo Barbierato (Istituto Zooprofilattico Sperimentale delle Venezie / Ricerca e innovazione)
EPI_ISL_14760614	A/turkey/Italy/21VIR9891-21/2021	A / H5N1	Europe / Italy	Giacomo Barbierato (Istituto Zooprofilattico Sperimentale delle Venezie / Ricerca e innovazione)
EPI_ISL_14760615	A/turkey/Italy/21VIR9996/2021	A / H5N1	Europe / Italy	Giacomo Barbierato (Istituto Zooprofilattico Sperimentale delle Venezie / Ricerca e innovazione)
EPI_ISL_14760616	A/chicken/Italy/21VIR10000/2021	A / H5N1	Europe / Italy	Giacomo Barbierato (Istituto Zooprofilattico Sperimentale delle Venezie / Ricerca e innovazione)
EPI_ISL_14760617	A/turkey/Italy/21VIR9999/2021	A / H5N1	Europe / Italy	Giacomo Barbierato (Istituto Zooprofilattico Sperimentale delle Venezie / Ricerca e innovazione)
EPI_ISL_14760618	A/turkey/Italy/21VIR9997/2021	A / H5N1	Europe / Italy	Giacomo Barbierato (Istituto Zooprofilattico Sperimentale delle Venezie / Ricerca e innovazione)
EPI_ISL_14760619	A/turkey/Italy/21VIR10001/2021	A / H5N1	Europe / Italy	Giacomo Barbierato (Istituto Zooprofilattico Sperimentale delle Venezie / Ricerca e innovazione)
EPI_ISL_14760620	A/chicken/Italy/21VIR9998/2021	A / H5N1	Europe / Italy	Giacomo Barbierato (Istituto Zooprofilattico Sperimentale delle Venezie / Ricerca e innovazione)
EPI_ISL_14760621	A/turkey/Italy/21VIR9894-3/2021	A / H5N1	Europe / Italy	Giacomo Barbierato (Istituto Zooprofilattico Sperimentale delle Venezie / Ricerca e innovazione)
EPI_ISL_14760622	A/chicken/Italy/21VIR9944-2/2021	A / H5N1	Europe / Italy	Giacomo Barbierato (Istituto Zooprofilattico Sperimentale delle Venezie / Ricerca e innovazione)
EPI_ISL_14760623	A/turkey/Italy/21VIR9949-9/2021	A / H5N1	Europe / Italy	Giacomo Barbierato (Istituto Zooprofilattico Sperimentale delle Venezie / Ricerca e innovazione)
EPI_ISL_14760624	A/chicken/Italy/21VIR9940-5/2021	A / H5N1	Europe / Italy	Giacomo Barbierato (Istituto Zooprofilattico Sperimentale delle Venezie / Ricerca e innovazione)
EPI_ISL_14760625	A/chicken/Italy/21VIR10096/2021	A / H5N1	Europe / Italy	Giacomo Barbierato (Istituto Zooprofilattico Sperimentale delle Venezie / Ricerca e innovazione)
EPI_ISL_14760626	A/chicken/Italy/21VIR10093/2021	A / H5N1	Europe / Italy	Giacomo Barbierato (Istituto Zooprofilattico Sperimentale delle Venezie / Ricerca e innovazione)
EPI_ISL_14760627	A/turkey/Italy/21VIR10097/2021	A / H5N1	Europe / Italy	Giacomo Barbierato (Istituto Zooprofilattico Sperimentale delle Venezie / Ricerca e innovazione)
EPI_ISL_14760628	A/turkey/Italy/21VIR10095/2021	A / H5N1	Europe / Italy	Giacomo Barbierato (Istituto Zooprofilattico Sperimentale delle Venezie / Ricerca e innovazione)
EPI_ISL_14760629	A/chicken/Italy/21VIR10094/2021	A / H5N1	Europe / Italy	Giacomo Barbierato (Istituto Zooprofilattico Sperimentale delle Venezie / Ricerca e innovazione)
EPI_ISL_14760630	A/turkey/Italy/21VIR10092/2021	A / H5N1	Europe / Italy	Giacomo Barbierato (Istituto Zooprofilattico Sperimentale delle Venezie / Ricerca e innovazione)
EPI_ISL_14760631	A/turkey/Italy/21VIR9901-3/2021	A / H5N1	Europe / Italy	Giacomo Barbierato (Istituto Zooprofilattico Sperimentale delle Venezie / Ricerca e innovazione)
EPI_ISL_14760632	A/Guinea_fowl/Italy/21VIR10216/2021	A / H5N1	Europe / Italy	Giacomo Barbierato (Istituto Zooprofilattico Sperimentale delle Venezie / Ricerca e innovazione)
EPI_ISL_14760633	A/Guinea_fowl/Italy/21VIR10231/2021	A / H5N1	Europe / Italy	Giacomo Barbierato (Istituto Zooprofilattico Sperimentale delle Venezie / Ricerca e innovazione)
EPI_ISL_14760634	A/turkey/Italy/21VIR10103/2021	A / H5N1	Europe / Italy	Giacomo Barbierato (Istituto Zooprofilattico Sperimentale delle Venezie / Ricerca e innovazione)
EPI_ISL_14760635	A/chicken/Italy/21VIR10252/2021	A / H5N1	Europe / Italy	Giacomo Barbierato (Istituto Zooprofilattico Sperimentale delle Venezie / Ricerca e innovazione)
EPI_ISL_14760636	A/chicken/Italy/21VIR10146/2021	A / H5N1	Europe / Italy	Giacomo Barbierato (Istituto Zooprofilattico Sperimentale delle Venezie / Ricerca e innovazione)
EPI_ISL_14760637	A/pheasant/Italy/21VIR10359/2021	A / H5N1	Europe / Italy	Giacomo Barbierato (Istituto Zooprofilattico Sperimentale delle Venezie / Ricerca e innovazione)
EPI_ISL_14760638	A/guinea_fowl/Italy/21VIR10351/2021	A / H5N1	Europe / Italy	Giacomo Barbierato (Istituto Zooprofilattico Sperimentale delle Venezie / Ricerca e innovazione)
EPI_ISL_14760639	A/quail/Italy/21VIR10356/2021	A / H5N1	Europe / Italy	Giacomo Barbierato (Istituto Zooprofilattico Sperimentale delle Venezie / Ricerca e innovazione)
EPI_ISL_14760640	A/chicken/Italy/21VIR10358/2021	A / H5N1	Europe / Italy	Giacomo Barbierato (Istituto Zooprofilattico Sperimentale delle Venezie / Ricerca e innovazione)
EPI_ISL_14760641	A/chicken/Italy/21VIR10357/2021	A / H5N1	Europe / Italy	Giacomo Barbierato (Istituto Zooprofilattico Sperimentale delle Venezie / Ricerca e innovazione)
EPI_ISL_14760642	A/chicken/Italy/21VIR10262/2021	A / H5N1	Europe / Italy	Giacomo Barbierato (Istituto Zooprofilattico Sperimentale delle Venezie / Ricerca e innovazione)
EPI_ISL_14760643	A/chicken/Italy/21VIR10263/2021	A / H5N1	Europe / Italy	Giacomo Barbierato (Istituto Zooprofilattico Sperimentale delle Venezie / Ricerca e innovazione)
EPI_ISL_14760644	A/chicken/Italy/21VIR10271/2021	A / H5N1	Europe / Italy	Giacomo Barbierato (Istituto Zooprofilattico Sperimentale delle Venezie / Ricerca e innovazione)
EPI_ISL_14760645	A/chicken/Italy/21VIR10264/2021	A / H5N1	Europe / Italy	Giacomo Barbierato (Istituto Zooprofilattico Sperimentale delle Venezie / Ricerca e innovazione)
EPI_ISL_14760646	A/chicken/Italy/21VIR10265/2021	A / H5N1	Europe / Italy	Giacomo Barbierato (Istituto Zooprofilattico Sperimentale delle Venezie / Ricerca e innovazione)

EPI_ISL_14760647	A/turkey/Italy/21VIR10338/2021	A / H5N1	Europe / Italy	Giacomo Barbierato (Istituto Zooprofilattico Sperimentale delle Venezie / Ricerca e innovazione)
EPI_ISL_14760648	A/chicken/Italy/21VIR10382/2021	A / H5N1	Europe / Italy	Giacomo Barbierato (Istituto Zooprofilattico Sperimentale delle Venezie / Ricerca e innovazione)
EPI_ISL_14760649	A/turkey/Italy/21VIR10396/2021	A / H5N1	Europe / Italy	Giacomo Barbierato (Istituto Zooprofilattico Sperimentale delle Venezie / Ricerca e innovazione)
EPI_ISL_14760650	A/turkey/Italy/21VIR10395/2021	A / H5N1	Europe / Italy	Giacomo Barbierato (Istituto Zooprofilattico Sperimentale delle Venezie / Ricerca e innovazione)
EPI_ISL_14760651	A/chicken/Italy/21VIR10394/2021	A / H5N1	Europe / Italy	Giacomo Barbierato (Istituto Zooprofilattico Sperimentale delle Venezie / Ricerca e innovazione)
EPI_ISL_14760652	A/turkey/Italy/21VIR10397/2021	A / H5N1	Europe / Italy	Giacomo Barbierato (Istituto Zooprofilattico Sperimentale delle Venezie / Ricerca e innovazione)
EPI_ISL_14760653	A/turkey/Italy/21VIR10398/2021	A / H5N1	Europe / Italy	Giacomo Barbierato (Istituto Zooprofilattico Sperimentale delle Venezie / Ricerca e innovazione)
EPI_ISL_14760654	A/chicken/Italy/21VIR10470/2021	A / H5N1	Europe / Italy	Giacomo Barbierato (Istituto Zooprofilattico Sperimentale delle Venezie / Ricerca e innovazione)
EPI_ISL_14760655	A/chicken/Italy/21VIR10387/2021	A / H5N1	Europe / Italy	Giacomo Barbierato (Istituto Zooprofilattico Sperimentale delle Venezie / Ricerca e innovazione)
EPI_ISL_14760656	A/goose/Italy/Italy/21VIR10458/2021	A / H5N1	Europe / Italy	Giacomo Barbierato (Istituto Zooprofilattico Sperimentale delle Venezie / Ricerca e innovazione)
EPI_ISL_14760658	A/chicken/Italy/21VIR10385/2021	A / H5N1	Europe / Italy	Giacomo Barbierato (Istituto Zooprofilattico Sperimentale delle Venezie / Ricerca e innovazione)
EPI_ISL_14760659	A/chicken/Italy/21VIR10469/2021	A / H5N1	Europe / Italy	Giacomo Barbierato (Istituto Zooprofilattico Sperimentale delle Venezie / Ricerca e innovazione)
EPI_ISL_14760660	A/turkey/Italy/21VIR10471/2021	A / H5N1	Europe / Italy	Giacomo Barbierato (Istituto Zooprofilattico Sperimentale delle Venezie / Ricerca e innovazione)
EPI_ISL_14760661	A/chicken/Italy/21VIR10386/2021	A / H5N1	Europe / Italy	Giacomo Barbierato (Istituto Zooprofilattico Sperimentale delle Venezie / Ricerca e innovazione)
EPI_ISL_14760662	A/duck/Italy/21VIR10523-1/2021	A / H5N1	Europe / Italy	Giacomo Barbierato (Istituto Zooprofilattico Sperimentale delle Venezie / Ricerca e innovazione)
EPI_ISL_14760663	A/turkey/Italy/21VIR10524-1/2021	A / H5N1	Europe / Italy	Giacomo Barbierato (Istituto Zooprofilattico Sperimentale delle Venezie / Ricerca e innovazione)
EPI_ISL_14760664	A/turkey/Italy/21VIR10459-1/2021	A / H5N1	Europe / Italy	Giacomo Barbierato (Istituto Zooprofilattico Sperimentale delle Venezie / Ricerca e innovazione)
EPI_ISL_14760665	A/chicken/Italy/21VIR10463-1/2021	A / H5N1	Europe / Italy	Giacomo Barbierato (Istituto Zooprofilattico Sperimentale delle Venezie / Ricerca e innovazione)
EPI_ISL_14760666	A/chicken/Italy/21VIR10522-13/2021	A / H5N1	Europe / Italy	Giacomo Barbierato (Istituto Zooprofilattico Sperimentale delle Venezie / Ricerca e innovazione)
EPI_ISL_14760667	A/turkey/Italy/21VIR10622-1/2021	A / H5N1	Europe / Italy	Giacomo Barbierato (Istituto Zooprofilattico Sperimentale delle Venezie / Ricerca e innovazione)
EPI_ISL_14760668	A/turkey/Italy/21VIR10617-2/2021	A / H5N1	Europe / Italy	Giacomo Barbierato (Istituto Zooprofilattico Sperimentale delle Venezie / Ricerca e innovazione)
EPI_ISL_14760669	A/turkey/Italy/21VIR10621-1/2021	A / H5N1	Europe / Italy	Giacomo Barbierato (Istituto Zooprofilattico Sperimentale delle Venezie / Ricerca e innovazione)
EPI_ISL_14760670	A/chicken/Italy/21VIR10616-1/2021	A / H5N1	Europe / Italy	Giacomo Barbierato (Istituto Zooprofilattico Sperimentale delle Venezie / Ricerca e innovazione)
EPI_ISL_14760671	A/chicken/Italy/21VIR10618-19/2021	A / H5N1	Europe / Italy	Giacomo Barbierato (Istituto Zooprofilattico Sperimentale delle Venezie / Ricerca e innovazione)
EPI_ISL_14760672	A/chicken/Italy/21VIR10619-1/2021	A / H5N1	Europe / Italy	Giacomo Barbierato (Istituto Zooprofilattico Sperimentale delle Venezie / Ricerca e innovazione)
EPI_ISL_14760673	A/chicken/Italy/21VIR10620-1/2021	A / H5N1	Europe / Italy	Giacomo Barbierato (Istituto Zooprofilattico Sperimentale delle Venezie / Ricerca e innovazione)
EPI_ISL_14760674	A/chicken/Italy/21VIR10724-13/2021	A / H5N1	Europe / Italy	Giacomo Barbierato (Istituto Zooprofilattico Sperimentale delle Venezie / Ricerca e innovazione)
EPI_ISL_14760675	A/chicken/Italy/21VIR10723-1/2021	A / H5N1	Europe / Italy	Giacomo Barbierato (Istituto Zooprofilattico Sperimentale delle Venezie / Ricerca e innovazione)
EPI_ISL_14760676	A/chicken/Italy/21VIR10634-1/2021	A / H5N1	Europe / Italy	Giacomo Barbierato (Istituto Zooprofilattico Sperimentale delle Venezie / Ricerca e innovazione)
EPI_ISL_14760677	A/chicken/Italy/21VIR10635-1/2021	A / H5N1	Europe / Italy	Giacomo Barbierato (Istituto Zooprofilattico Sperimentale delle Venezie / Ricerca e innovazione)
EPI_ISL_14760678	A/chicken/Italy/21VIR10637-2/2021	A / H5N1	Europe / Italy	Giacomo Barbierato (Istituto Zooprofilattico Sperimentale delle Venezie / Ricerca e innovazione)
EPI_ISL_14760679	A/turkey/Italy/21VIR10636-17/2021	A / H5N1	Europe / Italy	Giacomo Barbierato (Istituto Zooprofilattico Sperimentale delle Venezie / Ricerca e innovazione)
EPI_ISL_14760680	A/turkey/Italy/21VIR10645-15/2021	A / H5N1	Europe / Italy	Giacomo Barbierato (Istituto Zooprofilattico Sperimentale delle Venezie / Ricerca e innovazione)
EPI_ISL_14760681	A/chicken/Italy/21VIR10853/2021	A / H5N1	Europe / Italy	Giacomo Barbierato (Istituto Zooprofilattico Sperimentale delle Venezie / Ricerca e innovazione)
EPI_ISL_14760682	A/turkey/Italy/21VIR10699-1/2021	A / H5N1	Europe / Italy	Giacomo Barbierato (Istituto Zooprofilattico Sperimentale delle Venezie / Ricerca e innovazione)
EPI_ISL_14760683	A/turkey/Italy/21VIR10856/2021	A / H5N1	Europe / Italy	Giacomo Barbierato (Istituto Zooprofilattico Sperimentale delle Venezie / Ricerca e innovazione)
EPI_ISL_14760684	A/turkey/Italy/21VIR10852/2021	A / H5N1	Europe / Italy	Giacomo Barbierato (Istituto Zooprofilattico Sperimentale delle Venezie / Ricerca e innovazione)
EPI_ISL_14760685	A/turkey/Italy/21VIR10857/2021	A / H5N1	Europe / Italy	Giacomo Barbierato (Istituto Zooprofilattico Sperimentale delle Venezie / Ricerca e innovazione)
EPI_ISL_14760686	A/turkey/Italy/21VIR10854/2021	A / H5N1	Europe / Italy	Giacomo Barbierato (Istituto Zooprofilattico Sperimentale delle Venezie / Ricerca e innovazione)
EPI_ISL_14760687	A/turkey/Italy/21VIR10855/2021	A / H5N1	Europe / Italy	Giacomo Barbierato (Istituto Zooprofilattico Sperimentale delle Venezie / Ricerca e innovazione)
EPI_ISL_14760688	A/chicken/Italy/21VIR10696-7/2021	A / H5N1	Europe / Italy	Giacomo Barbierato (Istituto Zooprofilattico Sperimentale delle Venezie / Ricerca e innovazione)
EPI_ISL_14760689	A/turkey/Italy/21VIR10697-1/2021	A / H5N1	Europe / Italy	Giacomo Barbierato (Istituto Zooprofilattico Sperimentale delle Venezie / Ricerca e innovazione)
EPI_ISL_14760690	A/turkey/Italy/21VIR10737-1/2021	A / H5N1	Europe / Italy	Giacomo Barbierato (Istituto Zooprofilattico Sperimentale delle Venezie / Ricerca e innovazione)
EPI_ISL_14760691	A/chicken/Italy/21VIR10814-1/2021	A / H5N1	Europe / Italy	Giacomo Barbierato (Istituto Zooprofilattico Sperimentale delle Venezie / Ricerca e innovazione)
EPI_ISL_14760692	A/chicken/Italy/21VIR10808-1/2021	A / H5N1	Europe / Italy	Giacomo Barbierato (Istituto Zooprofilattico Sperimentale delle Venezie / Ricerca e innovazione)
EPI_ISL_14760693	A/duck/Italy/21VIR10809-1/2021	A / H5N1	Europe / Italy	Giacomo Barbierato (Istituto Zooprofilattico Sperimentale delle Venezie / Ricerca e innovazione)
EPI_ISL_14760694	A/turkey/Italy/21VIR10810-1/2021	A / H5N1	Europe / Italy	Giacomo Barbierato (Istituto Zooprofilattico Sperimentale delle Venezie / Ricerca e innovazione)
EPI_ISL_14760695	A/chicken/Italy/21VIR10811-28/2021	A / H5N1	Europe / Italy	Giacomo Barbierato (Istituto Zooprofilattico Sperimentale delle Venezie / Ricerca e innovazione)
EPI_ISL_14760696	A/chicken/Italy/21VIR10812-11/2021	A / H5N1	Europe / Italy	Giacomo Barbierato (Istituto Zooprofilattico Sperimentale delle Venezie / Ricerca e innovazione)
EPI_ISL_14760697	A/turkey/Italy/21VIR10929/2021	A / H5N1	Europe / Italy	Giacomo Barbierato (Istituto Zooprofilattico Sperimentale delle Venezie / Ricerca e innovazione)
EPI_ISL_14760698	A/chicken/Italy/21VIR10928/2021	A / H5N1	Europe / Italy	Giacomo Barbierato (Istituto Zooprofilattico Sperimentale delle Venezie / Ricerca e innovazione)
EPI_ISL_14760699	A/turkey/Italy/21VIR10930/2021	A / H5N1	Europe / Italy	Giacomo Barbierato (Istituto Zooprofilattico Sperimentale delle Venezie / Ricerca e innovazione)
EPI_ISL_14760700	A/turkey/Italy/21VIR10757-1/2021	A / H5N1	Europe / Italy	Giacomo Barbierato (Istituto Zooprofilattico Sperimentale delle Venezie / Ricerca e innovazione)
EPI_ISL_14760701	A/chicken/Italy/21VIR10843-1/2021	A / H5N1	Europe / Italy	Giacomo Barbierato (Istituto Zooprofilattico Sperimentale delle Venezie / Ricerca e innovazione)
EPI_ISL_14760702	A/turkey/Italy/21VIR10847-1/2021	A / H5N1	Europe / Italy	Giacomo Barbierato (Istituto Zooprofilattico Sperimentale delle Venezie / Ricerca e innovazione)
EPI_ISL_14760703	A/turkey/Italy/21VIR10970-3/2021	A / H5N1	Europe / Italy	Giacomo Barbierato (Istituto Zooprofilattico Sperimentale delle Venezie / Ricerca e innovazione)
EPI_ISL_14760704	A/chicken/Italy/21VIR10971-3/2021	A / H5N1	Europe / Italy	Giacomo Barbierato (Istituto Zooprofilattico Sperimentale delle Venezie / Ricerca e innovazione)
EPI_ISL_14760705	A/chicken/Italy/21VIR10973-4/2021	A / H5N1	Europe / Italy	Giacomo Barbierato (Istituto Zooprofilattico Sperimentale delle Venezie / Ricerca e innovazione)
EPI_ISL_14760706	A/chicken/Italy/21VIR10972-3/2021	A / H5N1	Europe / Italy	Giacomo Barbierato (Istituto Zooprofilattico Sperimentale delle Venezie / Ricerca e innovazione)
EPI_ISL_14760707	A/chicken/Italy/21VIR10969-3/2021	A / H5N1	Europe / Italy	Giacomo Barbierato (Istituto Zooprofilattico Sperimentale delle Venezie / Ricerca e innovazione)
EPI_ISL_14760708	A/turkey/Italy/21VIR10988-1/2021	A / H5N1	Europe / Italy	Giacomo Barbierato (Istituto Zooprofilattico Sperimentale delle Venezie / Ricerca e innovazione)
EPI_ISL_14760709	A/chicken/Italy/21VIR10989/2021	A / H5N1	Europe / Italy	Giacomo Barbierato (Istituto Zooprofilattico Sperimentale delle Venezie / Ricerca e innovazione)
EPI_ISL_14760710	A/chicken/Italy/21VIR11072-1/2021	A / H5N1	Europe / Italy	Giacomo Barbierato (Istituto Zooprofilattico Sperimentale delle Venezie / Ricerca e innovazione)
EPI_ISL_14760711	A/chicken/Italy/21VIR11074-1/2021	A / H5N1	Europe / Italy	Giacomo Barbierato (Istituto Zooprofilattico Sperimentale delle Venezie / Ricerca e innovazione)
EPI_ISL_14760712	A/chicken/Italy/21VIR11071-2/2021	A / H5N1	Europe / Italy	Giacomo Barbierato (Istituto Zooprofilattico Sperimentale delle Venezie / Ricerca e innovazione)
EPI_ISL_14760713	A/avian/Italy/21VIR10913-3/2021	A / H5N1	Europe / Italy	Giacomo Barbierato (Istituto Zooprofilattico Sperimentale delle Venezie / Ricerca e innovazione)
EPI_ISL_14760714	A/chicken/Italy/21VIR10987-1/2021	A / H5N1	Europe / Italy	Giacomo Barbierato (Istituto Zooprofilattico Sperimentale delle Venezie / Ricerca e innovazione)
EPI_ISL_14760715	A/chicken/Italy/21VIR11076-1/2021	A / H5N1	Europe / Italy	Giacomo Barbierato (Istituto Zooprofilattico Sperimentale delle Venezie / Ricerca e innovazione)
EPI_ISL_14760716	A/chicken/Italy/21VIR11079-11/2021	A / H5N1	Europe / Italy	Giacomo Barbierato (Istituto Zooprofilattico Sperimentale delle Venezie / Ricerca e innovazione)
EPI_ISL_14760717	A/chicken/Italy/21VIR11078-10/2021	A / H5N1	Europe / Italy	Giacomo Barbierato (Istituto Zooprofilattico Sperimentale delle Venezie / Ricerca e innovazione)
EPI_ISL_14760718	A/chicken/Italy/21VIR11073-1/2021	A / H5N1	Europe / Italy	Giacomo Barbierato (Istituto Zooprofilattico Sperimentale delle Venezie / Ricerca e innovazione)
EPI_ISL_14760719	A/chicken/Italy/21VIR11075-2/2021	A / H5N1	Europe / Italy	Giacomo Barbierato (Istituto Zooprofilattico Sperimentale delle Venezie / Ricerca e innovazione)

EPI_ISL_14760720	A/turkey/Italy/21VIR11077-1/2021	A / H5N1	Europe / Italy	Giacomo Barbierato (Istituto Zooprofilattico Sperimentale delle Venezie / Ricerca e innovazione)
EPI_ISL_14760721	A/chicken/Italy/21VIR10995-1/2021	A / H5N1	Europe / Italy	Giacomo Barbierato (Istituto Zooprofilattico Sperimentale delle Venezie / Ricerca e innovazione)
EPI_ISL_14760722	A/chicken/Italy/21VIR10999-1/2021	A / H5N1	Europe / Italy	Giacomo Barbierato (Istituto Zooprofilattico Sperimentale delle Venezie / Ricerca e innovazione)
EPI_ISL_14760723	A/chicken/Italy/21VIR10997-1/2021	A / H5N1	Europe / Italy	Giacomo Barbierato (Istituto Zooprofilattico Sperimentale delle Venezie / Ricerca e innovazione)
EPI_ISL_14760724	A/turkey/Italy/21VIR11006-1/2021	A / H5N1	Europe / Italy	Giacomo Barbierato (Istituto Zooprofilattico Sperimentale delle Venezie / Ricerca e innovazione)
EPI_ISL_14760725	A/turkey/Italy/21VIR11007-1/2021	A / H5N1	Europe / Italy	Giacomo Barbierato (Istituto Zooprofilattico Sperimentale delle Venezie / Ricerca e innovazione)
EPI_ISL_14760726	A/turkey/Italy/21VIR11166/2021	A / H5N1	Europe / Italy	Giacomo Barbierato (Istituto Zooprofilattico Sperimentale delle Venezie / Ricerca e innovazione)
EPI_ISL_14760727	A/chicken/Italy/21VIR11055-1/2021	A / H5N1	Europe / Italy	Giacomo Barbierato (Istituto Zooprofilattico Sperimentale delle Venezie / Ricerca e innovazione)
EPI_ISL_14760728	A/pheasant/Italy/21VIR11162/2021	A / H5N1	Europe / Italy	Giacomo Barbierato (Istituto Zooprofilattico Sperimentale delle Venezie / Ricerca e innovazione)
EPI_ISL_14760729	A/turkey/Italy/21VIR11164/2021	A / H5N1	Europe / Italy	Giacomo Barbierato (Istituto Zooprofilattico Sperimentale delle Venezie / Ricerca e innovazione)
EPI_ISL_14760730	A/pheasant/Italy/21VIR11165/2021	A / H5N1	Europe / Italy	Giacomo Barbierato (Istituto Zooprofilattico Sperimentale delle Venezie / Ricerca e innovazione)
EPI_ISL_14760731	A/laying_hen/Italy/21VIR11167/2021	A / H5N1	Europe / Italy	Giacomo Barbierato (Istituto Zooprofilattico Sperimentale delle Venezie / Ricerca e innovazione)
EPI_ISL_14760732	A/laying_hen/Italy/21VIR11168/2021	A / H5N1	Europe / Italy	Giacomo Barbierato (Istituto Zooprofilattico Sperimentale delle Venezie / Ricerca e innovazione)
EPI_ISL_14760733	A/laying_hen/Italy/21VIR11169/2021	A / H5N1	Europe / Italy	Giacomo Barbierato (Istituto Zooprofilattico Sperimentale delle Venezie / Ricerca e innovazione)
EPI_ISL_14760734	A/chicken/Italy/21VIR11234/2021	A / H5N1	Europe / Italy	Giacomo Barbierato (Istituto Zooprofilattico Sperimentale delle Venezie / Ricerca e innovazione)
EPI_ISL_14760735	A/laying_hen/Italy/21VIR11300/2021	A / H5N1	Europe / Italy	Giacomo Barbierato (Istituto Zooprofilattico Sperimentale delle Venezie / Ricerca e innovazione)
EPI_ISL_14760736	A/chicken/Italy/21VIR11054-1/2021	A / H5N1	Europe / Italy	Giacomo Barbierato (Istituto Zooprofilattico Sperimentale delle Venezie / Ricerca e innovazione)
EPI_ISL_14760737	A/chicken/Italy/21VIR11056-1/2021	A / H5N1	Europe / Italy	Giacomo Barbierato (Istituto Zooprofilattico Sperimentale delle Venezie / Ricerca e innovazione)
EPI_ISL_14760738	A/chicken/Italy/21VIR11056-6/2021	A / H5N1	Europe / Italy	Giacomo Barbierato (Istituto Zooprofilattico Sperimentale delle Venezie / Ricerca e innovazione)
EPI_ISL_14760739	A/turkey/Italy/21VIR11058/2021	A / H5N1	Europe / Italy	Giacomo Barbierato (Istituto Zooprofilattico Sperimentale delle Venezie / Ricerca e innovazione)
EPI_ISL_14760740	A/turkey/Italy/21VIR11163/2021	A / H5N1	Europe / Italy	Giacomo Barbierato (Istituto Zooprofilattico Sperimentale delle Venezie / Ricerca e innovazione)
EPI_ISL_14760741	A/chicken/Italy/21VIR11235/2021	A / H5N1	Europe / Italy	Giacomo Barbierato (Istituto Zooprofilattico Sperimentale delle Venezie / Ricerca e innovazione)
EPI_ISL_14760742	A/avian/Italy/21VIR11236/2021	A / H5N1	Europe / Italy	Giacomo Barbierato (Istituto Zooprofilattico Sperimentale delle Venezie / Ricerca e innovazione)
EPI_ISL_14760743	A/turkey/Italy/21VIR11237/2021	A / H5N1	Europe / Italy	Giacomo Barbierato (Istituto Zooprofilattico Sperimentale delle Venezie / Ricerca e innovazione)
EPI_ISL_14760744	A/chicken/Italy/21VIR11238/2021	A / H5N1	Europe / Italy	Giacomo Barbierato (Istituto Zooprofilattico Sperimentale delle Venezie / Ricerca e innovazione)
EPI_ISL_14760745	A/guinea_fowl/Italy/21VIR11298/2021	A / H5N1	Europe / Italy	Giacomo Barbierato (Istituto Zooprofilattico Sperimentale delle Venezie / Ricerca e innovazione)
EPI_ISL_14760746	A/turkey/Italy/21VIR11302/2021	A / H5N1	Europe / Italy	Giacomo Barbierato (Istituto Zooprofilattico Sperimentale delle Venezie / Ricerca e innovazione)
EPI_ISL_14760747	A/turkey/Italy/21VIR11251-1/2021	A / H5N1	Europe / Italy	Giacomo Barbierato (Istituto Zooprofilattico Sperimentale delle Venezie / Ricerca e innovazione)
EPI_ISL_14760748	A/turkey/Italy/21VIR11252-1/2021	A / H5N1	Europe / Italy	Giacomo Barbierato (Istituto Zooprofilattico Sperimentale delle Venezie / Ricerca e innovazione)
EPI_ISL_14760749	A/turkey/Italy/21VIR11254-1/2021	A / H5N1	Europe / Italy	Giacomo Barbierato (Istituto Zooprofilattico Sperimentale delle Venezie / Ricerca e innovazione)
EPI_ISL_14760750	A/laying_hen/Italy/21VIR11253-1/2021	A / H5N1	Europe / Italy	Giacomo Barbierato (Istituto Zooprofilattico Sperimentale delle Venezie / Ricerca e innovazione)
EPI_ISL_14760751	A/laying_hen/Italy/21VIR11299/2021	A / H5N1	Europe / Italy	Giacomo Barbierato (Istituto Zooprofilattico Sperimentale delle Venezie / Ricerca e innovazione)
EPI_ISL_14760752	A/avian/Italy/21VIR11301/2021	A / H5N1	Europe / Italy	Giacomo Barbierato (Istituto Zooprofilattico Sperimentale delle Venezie / Ricerca e innovazione)
EPI_ISL_14760753	A/turkey/Italy/21VIR11384-1/2021	A / H5N1	Europe / Italy	Giacomo Barbierato (Istituto Zooprofilattico Sperimentale delle Venezie / Ricerca e innovazione)
EPI_ISL_14760754	A/turkey/Italy/21VIR11387-1/2021	A / H5N1	Europe / Italy	Giacomo Barbierato (Istituto Zooprofilattico Sperimentale delle Venezie / Ricerca e innovazione)
EPI_ISL_14760755	A/laying_hen/Italy/21VIR11390-2/2021	A / H5N1	Europe / Italy	Giacomo Barbierato (Istituto Zooprofilattico Sperimentale delle Venezie / Ricerca e innovazione)
EPI_ISL_14760756	A/turkey/Italy/21VIR11391-3/2021	A / H5N1	Europe / Italy	Giacomo Barbierato (Istituto Zooprofilattico Sperimentale delle Venezie / Ricerca e innovazione)
EPI_ISL_14760757	A/turkey/Italy/21VIR11392-2/2021	A / H5N1	Europe / Italy	Giacomo Barbierato (Istituto Zooprofilattico Sperimentale delle Venezie / Ricerca e innovazione)
EPI_ISL_14760758	A/turkey/Italy/21VIR11393-3/2021	A / H5N1	Europe / Italy	Giacomo Barbierato (Istituto Zooprofilattico Sperimentale delle Venezie / Ricerca e innovazione)
EPI_ISL_14760762	A/guinea_fowl/Italy/21VIR11383-1/2021	A / H5N1	Europe / Italy	Giacomo Barbierato (Istituto Zooprofilattico Sperimentale delle Venezie / Ricerca e innovazione)
EPI_ISL_14760774	A/chicken/Italy/21VIR11385-1/2021	A / H5N1	Europe / Italy	Giacomo Barbierato (Istituto Zooprofilattico Sperimentale delle Venezie / Ricerca e innovazione)
EPI_ISL_14760784	A/turkey/Italy/21VIR11386-1/2021	A / H5N1	Europe / Italy	Giacomo Barbierato (Istituto Zooprofilattico Sperimentale delle Venezie / Ricerca e innovazione)
EPI_ISL_14760793	A/laying_hen/Italy/21VIR11394-1/2021	A / H5N1	Europe / Italy	Giacomo Barbierato (Istituto Zooprofilattico Sperimentale delle Venezie / Ricerca e innovazione)
EPI_ISL_14760803	A/laying_hen/Italy/21VIR11395-1/2021	A / H5N1	Europe / Italy	Giacomo Barbierato (Istituto Zooprofilattico Sperimentale delle Venezie / Ricerca e innovazione)
EPI_ISL_14760814	A/turkey/Italy/21VIR11396-1/2021	A / H5N1	Europe / Italy	Giacomo Barbierato (Istituto Zooprofilattico Sperimentale delle Venezie / Ricerca e innovazione)
EPI_ISL_14760826	A/laying_hen/Italy/21VIR11486/2021	A / H5N1	Europe / Italy	Giacomo Barbierato (Istituto Zooprofilattico Sperimentale delle Venezie / Ricerca e innovazione)
EPI_ISL_14760837	A/chicken/Italy/21VIR11485/2021	A / H5N1	Europe / Italy	Giacomo Barbierato (Istituto Zooprofilattico Sperimentale delle Venezie / Ricerca e innovazione)
EPI_ISL_14760849	A/turkey/Italy/21VIR11509/2021	A / H5N1	Europe / Italy	Giacomo Barbierato (Istituto Zooprofilattico Sperimentale delle Venezie / Ricerca e innovazione)
EPI_ISL_14760859	A/turkey/Italy/21VIR11510/2021	A / H5N1	Europe / Italy	Giacomo Barbierato (Istituto Zooprofilattico Sperimentale delle Venezie / Ricerca e innovazione)
EPI_ISL_14760870	A/turkey/Italy/21VIR11440-2/2021	A / H5N1	Europe / Italy	Giacomo Barbierato (Istituto Zooprofilattico Sperimentale delle Venezie / Ricerca e innovazione)
EPI_ISL_14760880	A/chicken/Italy/21VIR11508/2021	A / H5N1	Europe / Italy	Giacomo Barbierato (Istituto Zooprofilattico Sperimentale delle Venezie / Ricerca e innovazione)
EPI_ISL_14760891	A/turkey/Italy/21VIR11511/2021	A / H5N1	Europe / Italy	Giacomo Barbierato (Istituto Zooprofilattico Sperimentale delle Venezie / Ricerca e innovazione)
EPI_ISL_14760902	A/turkey/Italy/21VIR11506/2022	A / H5N1	Europe / Italy	Giacomo Barbierato (Istituto Zooprofilattico Sperimentale delle Venezie / Ricerca e innovazione)
EPI_ISL_14760913	A/turkey/Italy/21VIR11499-5/2021	A / H5N1	Europe / Italy	Giacomo Barbierato (Istituto Zooprofilattico Sperimentale delle Venezie / Ricerca e innovazione)
EPI_ISL_14760923	A/turkey/Italy/21VIR11503-1/2021	A / H5N1	Europe / Italy	Giacomo Barbierato (Istituto Zooprofilattico Sperimentale delle Venezie / Ricerca e innovazione)
EPI_ISL_14760936	A/laying_hen/Italy/21VIR11505-2/2021	A / H5N1	Europe / Italy	Giacomo Barbierato (Istituto Zooprofilattico Sperimentale delle Venezie / Ricerca e innovazione)
EPI_ISL_14760946	A/laying_hen/Italy/21VIR11498-1/2021	A / H5N1	Europe / Italy	Giacomo Barbierato (Istituto Zooprofilattico Sperimentale delle Venezie / Ricerca e innovazione)
EPI_ISL_14760956	A/turkey/Italy/21VIR11492-4/2021	A / H5N1	Europe / Italy	Giacomo Barbierato (Istituto Zooprofilattico Sperimentale delle Venezie / Ricerca e innovazione)
EPI_ISL_14760967	A/turkey/Italy/21VIR11495-5/2021	A / H5N1	Europe / Italy	Giacomo Barbierato (Istituto Zooprofilattico Sperimentale delle Venezie / Ricerca e innovazione)
EPI_ISL_14760977	A/turkey/Italy/21VIR11500-3/2021	A / H5N1	Europe / Italy	Giacomo Barbierato (Istituto Zooprofilattico Sperimentale delle Venezie / Ricerca e innovazione)
EPI_ISL_14760988	A/duck/Italy/21VIR11501/2021	A / H5N1	Europe / Italy	Giacomo Barbierato (Istituto Zooprofilattico Sperimentale delle Venezie / Ricerca e innovazione)
EPI_ISL_14760999	A/turkey/Italy/21VIR11504/2021	A / H5N1	Europe / Italy	Giacomo Barbierato (Istituto Zooprofilattico Sperimentale delle Venezie / Ricerca e innovazione)
EPI_ISL_14761009	A/chicken/Italy/21VIR11571/2021	A / H5N1	Europe / Italy	Giacomo Barbierato (Istituto Zooprofilattico Sperimentale delle Venezie / Ricerca e innovazione)
EPI_ISL_14761020	A/chicken/Italy/21VIR11572/2021	A / H5N1	Europe / Italy	Giacomo Barbierato (Istituto Zooprofilattico Sperimentale delle Venezie / Ricerca e innovazione)
EPI_ISL_14761031	A/turkey/Italy/21VIR11596-3/2021	A / H5N1	Europe / Italy	Giacomo Barbierato (Istituto Zooprofilattico Sperimentale delle Venezie / Ricerca e innovazione)
EPI_ISL_14761041	A/broiler/Italy/21VIR11583-1/2021	A / H5N1	Europe / Italy	Giacomo Barbierato (Istituto Zooprofilattico Sperimentale delle Venezie / Ricerca e innovazione)
EPI_ISL_14761052	A/laying_hen/Italy/21VIR11595-5/2021	A / H5N1	Europe / Italy	Giacomo Barbierato (Istituto Zooprofilattico Sperimentale delle Venezie / Ricerca e innovazione)
EPI_ISL_14761065	A/laying_hen/Italy/21VIR11597-1/2021	A / H5N1	Europe / Italy	Giacomo Barbierato (Istituto Zooprofilattico Sperimentale delle Venezie / Ricerca e innovazione)
EPI_ISL_14761075	A/laying_hen/Italy/21VIR11592-1/2021	A / H5N1	Europe / Italy	Giacomo Barbierato (Istituto Zooprofilattico Sperimentale delle Venezie / Ricerca e innovazione)
EPI_ISL_14761086	A/turkey/Italy/21VIR11586-1/2021	A / H5N1	Europe / Italy	Giacomo Barbierato (Istituto Zooprofilattico Sperimentale delle Venezie / Ricerca e innovazione)
EPI_ISL_14761097	A/turkey/Italy/21VIR11587-1/2021	A / H5N1	Europe / Italy	Giacomo Barbierato (Istituto Zooprofilattico Sperimentale delle Venezie / Ricerca e innovazione)
EPI_ISL_14761107	A/turkey/Italy/21VIR11587-2/2021	A / H5N1	Europe / Italy	Giacomo Barbierato (Istituto Zooprofilattico Sperimentale delle Venezie / Ricerca e innovazione)

EPI_ISL_14761119	A/turkey/Italy/21VIR11691/2021	A / H5N1	Europe / Italy	Giacomo Barbierato (Istituto Zooprofilattico Sperimentale delle Venezie / Ricerca e innovazione)
EPI_ISL_14761129	A/turkey/Italy/21VIR11692/2021	A / H5N1	Europe / Italy	Giacomo Barbierato (Istituto Zooprofilattico Sperimentale delle Venezie / Ricerca e innovazione)
EPI_ISL_14761141	A/turkey/Italy/21VIR11690/2021	A / H5N1	Europe / Italy	Giacomo Barbierato (Istituto Zooprofilattico Sperimentale delle Venezie / Ricerca e innovazione)
EPI_ISL_14761152	A/turkey/Italy/21VIR11661-7/2021	A / H5N1	Europe / Italy	Giacomo Barbierato (Istituto Zooprofilattico Sperimentale delle Venezie / Ricerca e innovazione)
EPI_ISL_14761165	A/broiler/Italy/21VIR11800/2021	A / H5N1	Europe / Italy	Giacomo Barbierato (Istituto Zooprofilattico Sperimentale delle Venezie / Ricerca e innovazione)
EPI_ISL_14761176	A/laying_hen/Italy/21VIR11801-7/2021	A / H5N1	Europe / Italy	Giacomo Barbierato (Istituto Zooprofilattico Sperimentale delle Venezie / Ricerca e innovazione)
EPI_ISL_14761188	A/broiler/Italy/21VIR11802-1/2021	A / H5N1	Europe / Italy	Giacomo Barbierato (Istituto Zooprofilattico Sperimentale delle Venezie / Ricerca e innovazione)
EPI_ISL_14761199	A/turkey/Italy/21VIR11772-7/2021	A / H5N1	Europe / Italy	Giacomo Barbierato (Istituto Zooprofilattico Sperimentale delle Venezie / Ricerca e innovazione)
EPI_ISL_14761208	A/avian/Italy/21VIR11773-4/2021	A / H5N1	Europe / Italy	Giacomo Barbierato (Istituto Zooprofilattico Sperimentale delle Venezie / Ricerca e innovazione)
EPI_ISL_14761220	A/chicken/Italy/21VIR11774-2/2021	A / H5N1	Europe / Italy	Giacomo Barbierato (Istituto Zooprofilattico Sperimentale delle Venezie / Ricerca e innovazione)
EPI_ISL_14761232	A/turkey/Italy/21VIR11935/2021	A / H5N1	Europe / Italy	Giacomo Barbierato (Istituto Zooprofilattico Sperimentale delle Venezie / Ricerca e innovazione)
EPI_ISL_14761243	A/broiler/Italy/21VIR11847-1/2021	A / H5N1	Europe / Italy	Giacomo Barbierato (Istituto Zooprofilattico Sperimentale delle Venezie / Ricerca e innovazione)
EPI_ISL_14761249	A/broiler/Italy/21VIR11886-1/2021	A / H5N1	Europe / Italy	Giacomo Barbierato (Istituto Zooprofilattico Sperimentale delle Venezie / Ricerca e innovazione)
EPI_ISL_14761250	A/heron/Italy/22VIR1562/2022	A / H5N1	Europe / Italy	Giacomo Barbierato (Istituto Zooprofilattico Sperimentale delle Venezie / Ricerca e innovazione)
EPI_ISL_14761251	A/broiler/Italy/22VIR54-1/2022	A / H5N1	Europe / Italy	Giacomo Barbierato (Istituto Zooprofilattico Sperimentale delle Venezie / Ricerca e innovazione)
EPI_ISL_14761261	A/laying_hen/Italy/22VIR48-1/2022	A / H5N1	Europe / Italy	Giacomo Barbierato (Istituto Zooprofilattico Sperimentale delle Venezie / Ricerca e innovazione)
EPI_ISL_14761272	A/turkey/Italy/IZSLT_22VIR366-3/2022	A / H5N1	Europe / Italy	Giacomo Barbierato (Istituto Zooprofilattico Sperimentale delle Venezie / Ricerca e innovazione)
EPI_ISL_14761281	A/turkey/Italy/22VIR171-2/2022	A / H5N1	Europe / Italy	Giacomo Barbierato (Istituto Zooprofilattico Sperimentale delle Venezie / Ricerca e innovazione)
EPI_ISL_14761291	A/guinea_fowl/Italy/22VIR205-4/2022	A / H5N1	Europe / Italy	Giacomo Barbierato (Istituto Zooprofilattico Sperimentale delle Venezie / Ricerca e innovazione)
EPI_ISL_14761302	A/laying_hen/Italy/22VIR203-3/2022	A / H5N1	Europe / Italy	Giacomo Barbierato (Istituto Zooprofilattico Sperimentale delle Venezie / Ricerca e innovazione)
EPI_ISL_14761312	A/chicken/Italy/22VIR234-1/2022	A / H5N1	Europe / Italy	Giacomo Barbierato (Istituto Zooprofilattico Sperimentale delle Venezie / Ricerca e innovazione)
EPI_ISL_14761318	A/laying_hen/Italy/22VIR204-1/2022	A / H5N1	Europe / Italy	Giacomo Barbierato (Istituto Zooprofilattico Sperimentale delle Venezie / Ricerca e innovazione)
EPI_ISL_14761327	A/broiler/Italy/22VIR278-3/2022	A / H5N1	Europe / Italy	Giacomo Barbierato (Istituto Zooprofilattico Sperimentale delle Venezie / Ricerca e innovazione)
EPI_ISL_14761338	A/hawk/Italy/22VIR428-5/2022	A / H5N1	Europe / Italy	Giacomo Barbierato (Istituto Zooprofilattico Sperimentale delle Venezie / Ricerca e innovazione)
EPI_ISL_14761349	A/hawk/Italy/22VIR428-7/2022	A / H5N1	Europe / Italy	Giacomo Barbierato (Istituto Zooprofilattico Sperimentale delle Venezie / Ricerca e innovazione)
EPI_ISL_14761361	A/buzzard/Italy/22VIR767-1/2022	A / H5N1	Europe / Italy	Giacomo Barbierato (Istituto Zooprofilattico Sperimentale delle Venezie / Ricerca e innovazione)
EPI_ISL_14761373	A/buzzard/Italy/22VIR767-2/2022	A / H5N1	Europe / Italy	Giacomo Barbierato (Istituto Zooprofilattico Sperimentale delle Venezie / Ricerca e innovazione)
EPI_ISL_14761383	A/swan/Italy/22VIR1560/2022	A / H5N1	Europe / Italy	Giacomo Barbierato (Istituto Zooprofilattico Sperimentale delle Venezie / Ricerca e innovazione)
EPI_ISL_14761395	A/herring_gull/Italy/22VIR1710-3/2022	A / H5N1	Europe / Italy	Giacomo Barbierato (Istituto Zooprofilattico Sperimentale delle Venezie / Ricerca e innovazione)
EPI_ISL_14761407	A/duck/Italy/22VIR1294-1/2022	A / H5N1	Europe / Italy	Giacomo Barbierato (Istituto Zooprofilattico Sperimentale delle Venezie / Ricerca e innovazione)
EPI_ISL_14761417	A/duck/Italy/22VIR1295-1/2022	A / H5N1	Europe / Italy	Giacomo Barbierato (Istituto Zooprofilattico Sperimentale delle Venezie / Ricerca e innovazione)
EPI_ISL_14761428	A/goose/Italy/22VIR1520/2022	A / H5N1	Europe / Italy	Giacomo Barbierato (Istituto Zooprofilattico Sperimentale delle Venezie / Ricerca e innovazione)
EPI_ISL_14761439	A/laying_hen/Italy/22VIR1521-2/2022	A / H5N1	Europe / Italy	Giacomo Barbierato (Istituto Zooprofilattico Sperimentale delle Venezie / Ricerca e innovazione)
EPI_ISL_14761451	A/chicken/Italy/22VIR1694-5/2022	A / H5N1	Europe / Italy	Giacomo Barbierato (Istituto Zooprofilattico Sperimentale delle Venezie / Ricerca e innovazione)
EPI_ISL_14761462	A/pheasant/Italy/22VIR1953-1/2022	A / H5N1	Europe / Italy	Giacomo Barbierato (Istituto Zooprofilattico Sperimentale delle Venezie / Ricerca e innovazione)
EPI_ISL_14761473	A/peacock/Italy/22VIR1953-2/2022	A / H5N1	Europe / Italy	Giacomo Barbierato (Istituto Zooprofilattico Sperimentale delle Venezie / Ricerca e innovazione)
EPI_ISL_14761485	A/chicken/Italy/22VIR1953-3/2022	A / H5N1	Europe / Italy	Giacomo Barbierato (Istituto Zooprofilattico Sperimentale delle Venezie / Ricerca e innovazione)
EPI_ISL_14761496	A/chicken/Italy/22VIR1953-4/2022	A / H5N1	Europe / Italy	Giacomo Barbierato (Istituto Zooprofilattico Sperimentale delle Venezie / Ricerca e innovazione)
EPI_ISL_14761507	A/broiler/Italy/22VIR1892-3/2022	A / H5N1	Europe / Italy	Giacomo Barbierato (Istituto Zooprofilattico Sperimentale delle Venezie / Ricerca e innovazione)
EPI_ISL_14761519	A/chicken/Italy/IZSLT22VIR2562-1/2022	A / H5N1	Europe / Italy	Giacomo Barbierato (Istituto Zooprofilattico Sperimentale delle Venezie / Ricerca e innovazione)
EPI_ISL_14769966	A/chicken/Lesotho/341.10/2021	A / H5N1	Africa / Lesotho	
EPI_ISL_14769967	A/chicken/Lesotho/352.3/2021	A / H5N1	Africa / Lesotho	
EPI_ISL_14770020	A/chicken/Egypt/Q16684C/2019	A / H5N8	Africa / Egypt	
EPI_ISL_14770033	A/chicken/Egypt/N13731D/2017	A / H5N8	Africa / Egypt	
EPI_ISL_14770035	A/duck/Egypt/N13736A/2017	A / H5N8	Africa / Egypt	
EPI_ISL_14770036	A/chicken/Egypt/H13794D/2017	A / H5N8	Africa / Egypt	
EPI_ISL_14770037	A/chicken/Egypt/Q13845A/2017	A / H5N8	Africa / Egypt	
EPI_ISL_14770039	A/chicken/Egypt/A13773E/2017	A / H5N8	Africa / Egypt	
EPI_ISL_14770042	A/duck/Egypt/N14205C/2017	A / H5N8	Africa / Egypt	
EPI_ISL_14770043	A/chicken/Egypt/Q13936C/2017	A / H5N8	Africa / Egypt	
EPI_ISL_14770046	A/chicken/Egypt/N13720E/2017	A / H5N8	Africa / Egypt	
EPI_ISL_14770047	A/chicken/Egypt/N15172D/2018	A / H5N8	Africa / Egypt	
EPI_ISL_14770049	A/duck/Egypt/N14200A/2017	A / H5N8	Africa / Egypt	
EPI_ISL_14770051	A/chicken/Egypt/F14110D/2017	A / H5N8	Africa / Egypt	
EPI_ISL_14770153	A/chicken/Egypt/F13829B/2017	A / H5N8	Africa / Egypt	
EPI_ISL_14770166	A/swan/Shandong/W3762/2020	A / H5N8	Asia / China	
EPI_ISL_14775174	A/chicken/Viet Nam/QN_21_3105/2021	A / H5N8	Asia / Vietnam	
EPI_ISL_14775175	A/Duck/Viet Nam/QN_21_5289/2021	A / H5N8	Asia / Vietnam	
EPI_ISL_14775176	A/chicken/Viet Nam/QN-20-985/2020	A / H5N6	Asia / Vietnam	
EPI_ISL_14775177	A/chicken/Viet Nam/QN-20-1144/2020	A / H5N6	Asia / Vietnam	
EPI_ISL_14775178	A/chicken/Viet Nam/QN-20-3597/2020	A / H5N6	Asia / Vietnam	
EPI_ISL_14775179	A/chicken/Viet Nam/QN-21-279/2021	A / H5N6	Asia / Vietnam	
EPI_ISL_14775180	A/chicken/Viet Nam/QN-21-109/2021	A / H5N6	Asia / Vietnam	
EPI_ISL_14775181	A/chicken/Viet Nam/QN-21-187/2021	A / H5N6	Asia / Vietnam	
EPI_ISL_14775182	A/chicken/Viet Nam/QN-21-237/2021	A / H5N6	Asia / Vietnam	
EPI_ISL_14775183	A/chicken/Viet Nam/QN-21-255/2021	A / H5N6	Asia / Vietnam	
EPI_ISL_14775184	A/chicken/Viet Nam/QN-21-438/2021	A / H5N6	Asia / Vietnam	
EPI_ISL_14775185	A/chicken/Viet Nam/QN-21-956/2021	A / H5N6	Asia / Vietnam	
EPI_ISL_14775186	A/chicken/Viet Nam/QN-21-1044/2021	A / H5N6	Asia / Vietnam	
EPI_ISL_14775274	A/chicken/Viet Nam/20-161/2020	A / H5N6	Asia / Vietnam	
EPI_ISL_14775517	A/goose/Hebei/HG12/2021	A / H5N8	Asia / China / Hebei Province	
EPI_ISL_14775522	A/Duck/Ismailia/F6/2021	A / H5N8	Africa / Egypt / Ismailia	

EPI_ISL_14775523	A/Duck/Ismailia/F16/2021	A / H5N8	Africa / Egypt / Ismailia	
EPI_ISL_14775524	A/chicken/Asyut/F2/2021	A / H5N8	Africa / Egypt / Asyut	
EPI_ISL_14778363	A/Wild bird/China/Cixi02/2020	A / H5N8	Asia / China	
EPI_ISL_14810369	A/porpoise /Sweden/SVA220712SZ0367/FB002184/O-2022	A / H5N1	Europe / Sweden / Vastra Gotalands Lan / Tanums Kommun	Siamak Zohari (National Veterinary Institute)
EPI_ISL_14811896	A/northern gannet/Sweden/SVA220623SZ0232/FB001982/O-2	A / H5N1	Europe / Sweden / Vastra Gotalands Lan / Sotenas Kommun	Siamak Zohari (National Veterinary Institute)
EPI_ISL_14811929	A/northern gannet/Sweden/SVA220623SZ0232/FB001984/N-2	A / H5N1	Europe / Sweden / Hallands Lan / Falkenbergs Kommun	Siamak Zohari (National Veterinary Institute)
EPI_ISL_148126	A/wild duck/Shandong/628/2011	A / H5N1	Asia / China	
EPI_ISL_14819895	A/Mallard/Ningxia/BY246/2020	A / H5N8	Asia / China / Ningxia Hui Autonomous Region	Zeyu Yang (Chinese Academy of Forestry / Research Institute of Forest Ecology, Environment and Protection)
EPI_ISL_14819898	A/Wild Duck/Ningxia/Y95/2021	A / H5N8	Asia / China / Ningxia Hui Autonomous Region	Zeyu Yang (Chinese Academy of Forestry / Research Institute of Forest Ecology, Environment and Protection)
EPI_ISL_14819899	A/Wild Duck/Ningxia/Y99/2021	A / H5N8	Asia / China / Ningxia Hui Autonomous Region	Zeyu Yang (Chinese Academy of Forestry / Research Institute of Forest Ecology, Environment and Protection)
EPI_ISL_14819900	A/Spot-billed Duck/Ningxia/Y16/2021	A / H5N8	Asia / China / Ningxia Hui Autonomous Region	Zeyu Yang (Chinese Academy of Forestry / Research Institute of Forest Ecology, Environment and Protection)
EPI_ISL_14822356	A/Spot-billed Duck/Ningxia/Y26/2021	A / H5N8	Asia / China / Ningxia Hui Autonomous Region	Zeyu Yang (Chinese Academy of Forestry / Research Institute of Forest Ecology, Environment and Protection)
EPI_ISL_14822357	A/Wild Duck/Ningxia/Y54/2021	A / H5N8	Asia / China / Ningxia Hui Autonomous Region	Zeyu Yang (Chinese Academy of Forestry / Research Institute of Forest Ecology, Environment and Protection)
EPI_ISL_14822426	A/Bar-headed Goose/Tibet/P2320/2021	A / H5N8	Asia / China / Tibet Autonomous Region	Zeyu Yang (Chinese Academy of Forestry / Research Institute of Forest Ecology, Environment and Protection)
EPI_ISL_14822451	A/Bar-headed Goose/Tibet/P1908/2021	A / H5N8	Asia / China / Tibet Autonomous Region	Zeyu Yang (Chinese Academy of Forestry / Research Institute of Forest Ecology, Environment and Protection)
EPI_ISL_14822452	A/Bar-headed Goose/Tibet/P1910/2021	A / H5N8	Asia / China / Tibet Autonomous Region	Zeyu Yang (Chinese Academy of Forestry / Research Institute of Forest Ecology, Environment and Protection)
EPI_ISL_14822547	A/Bar-headed Goose/Tibet/P1987/2021	A / H5N8	Asia / China / Tibet Autonomous Region	Zeyu Yang (Chinese Academy of Forestry / Research Institute of Forest Ecology, Environment and Protection)
EPI_ISL_14822549	A/bar_headed_goose/Tibet/T1640/2021	A / H5N8	Asia / China / Tibet Autonomous Region	Zeyu Yang (Chinese Academy of Forestry / Research Institute of Forest Ecology, Environment and Protection)
EPI_ISL_14822756	A/bar_headed_goose/Tibet/T1707/2021	A / H5N8	Asia / China / Tibet Autonomous Region	Zeyu Yang (Chinese Academy of Forestry / Research Institute of Forest Ecology, Environment and Protection)
EPI_ISL_14822757	A/brown-headed_gull/Tibet/N38/2021	A / H5N8	Asia / China / Tibet Autonomous Region	Zeyu Yang (Chinese Academy of Forestry / Research Institute of Forest Ecology, Environment and Protection)
EPI_ISL_14823405	A/Wild geese/Hubei/H358/2021	A / H5N8	Asia / China / Hubei Province	Zeyu Yang (Chinese Academy of Forestry / Research Institute of Forest Ecology, Environment and Protection)
EPI_ISL_14823421	A/Wild geese/Hubei/H418/2021	A / H5N8	Asia / China / Hubei Province	Zeyu Yang (Chinese Academy of Forestry / Research Institute of Forest Ecology, Environment and Protection)
EPI_ISL_14823422	A/Wild geese/Hubei/H549/2021	A / H5N8	Asia / China / Hubei Province	Zeyu Yang (Chinese Academy of Forestry / Research Institute of Forest Ecology, Environment and Protection)
EPI_ISL_14823424	A/Whooper swan/Sanmenxia/Y1/2020	A / H5N8	Asia / China / Henan Province	Zeyu Yang (Chinese Academy of Forestry / Research Institute of Forest Ecology, Environment and Protection)
EPI_ISL_14823425	A/Whooper swan/Sanmenxia/G2/2020	A / H5N8	Asia / China / Henan Province	Zeyu Yang (Chinese Academy of Forestry / Research Institute of Forest Ecology, Environment and Protection)
EPI_ISL_14823426	A/Whooper swan/Sanmenxia/Y6/2020	A / H5N8	Asia / China / Henan Province	Zeyu Yang (Chinese Academy of Forestry / Research Institute of Forest Ecology, Environment and Protection)
EPI_ISL_14823549	A/Whooper swan/Sanmenxia/Y8/2020	A / H5N8	Asia / China / Henan Province	Zeyu Yang (Chinese Academy of Forestry / Research Institute of Forest Ecology, Environment and Protection)
EPI_ISL_14823646	A/Whooper swan/Sanmenxia/Y10/2020	A / H5N8	Asia / China / Henan Province	Zeyu Yang (Chinese Academy of Forestry / Research Institute of Forest Ecology, Environment and Protection)
EPI_ISL_14823672	A/Whooper swan/Sanmenxia/Y11/2020	A / H5N8	Asia / China / Henan Province	Zeyu Yang (Chinese Academy of Forestry / Research Institute of Forest Ecology, Environment and Protection)
EPI_ISL_14823811	A/Whooper swan/Sanmenxia/Y12/2020	A / H5N8	Asia / China / Henan Province	Zeyu Yang (Chinese Academy of Forestry / Research Institute of Forest Ecology, Environment and Protection)
EPI_ISL_14823812	A/Whooper swan/Sanmenxia/G13/2020	A / H5N8	Asia / China / Henan Province	Zeyu Yang (Chinese Academy of Forestry / Research Institute of Forest Ecology, Environment and Protection)
EPI_ISL_14823813	A/Whooper swan/Sanmenxia/Y14/2020	A / H5N8	Asia / China / Henan Province	Zeyu Yang (Chinese Academy of Forestry / Research Institute of Forest Ecology, Environment and Protection)
EPI_ISL_14823863	A/Whooper swan/Sanmenxia/Y51/2020	A / H5N8	Asia / China / Henan Province	Zeyu Yang (Chinese Academy of Forestry / Research Institute of Forest Ecology, Environment and Protection)
EPI_ISL_14824026	A/Whooper swan/Sanmenxia/G15/2020	A / H5N8	Asia / China / Henan Province	Zeyu Yang (Chinese Academy of Forestry / Research Institute of Forest Ecology, Environment and Protection)
EPI_ISL_14824091	A/Whooper swan/Sanmenxia/Y15/2020	A / H5N8	Asia / China / Henan Province	Zeyu Yang (Chinese Academy of Forestry / Research Institute of Forest Ecology, Environment and Protection)
EPI_ISL_14824095	A/Whooper swan/Sanmenxia/Y16/2020	A / H5N8	Asia / China / Henan Province	Zeyu Yang (Chinese Academy of Forestry / Research Institute of Forest Ecology, Environment and Protection)
EPI_ISL_14824122	A/Whooper swan/Sanmenxia/Y52/2020	A / H5N8	Asia / China / Henan Province	Zeyu Yang (Chinese Academy of Forestry / Research Institute of Forest Ecology, Environment and Protection)
EPI_ISL_14835331	A/ostrich/Egypt/Egypt-Ostrich-2021-HA/2021	A / H5N8	Africa / Egypt	
EPI_ISL_14835960	A/Whooper swan/Sanmenxia/H1/2020	A / H5N8	Asia / China / Henan Province	Zeyu Yang (Chinese Academy of Forestry / Research Institute of Forest Ecology, Environment and Protection)
EPI_ISL_14835963	A/Whooper swan/Sanmenxia/H615/2020	A / H5N8	Asia / China / Henan Province	Zeyu Yang (Chinese Academy of Forestry / Research Institute of Forest Ecology, Environment and Protection)
EPI_ISL_14835964	A/Whooper swan/Sanmenxia/H735/2020	A / H5N8	Asia / China / Henan Province	Zeyu Yang (Chinese Academy of Forestry / Research Institute of Forest Ecology, Environment and Protection)
EPI_ISL_14835965	A/Whooper swan/Sanmenxia/H810/2020	A / H5N8	Asia / China / Henan Province	Zeyu Yang (Chinese Academy of Forestry / Research Institute of Forest Ecology, Environment and Protection)
EPI_ISL_14835968	A/Whooper swan/Sanmenxia/Y24/2020	A / H5N8	Asia / China / Henan Province	Zeyu Yang (Chinese Academy of Forestry / Research Institute of Forest Ecology, Environment and Protection)
EPI_ISL_14835974	A/Whooper swan/Sanmenxia/Y18/2020	A / H5N8	Asia / China / Henan Province	Zeyu Yang (Chinese Academy of Forestry / Research Institute of Forest Ecology, Environment and Protection)
EPI_ISL_14835976	A/Whooper swan/Sanmenxia/Y56/2020	A / H5N8	Asia / China / Henan Province	Zeyu Yang (Chinese Academy of Forestry / Research Institute of Forest Ecology, Environment and Protection)
EPI_ISL_14836024	A/Whooper swan/Sanmenxia/Y19/2020	A / H5N8	Asia / China / Henan Province	Zeyu Yang (Chinese Academy of Forestry / Research Institute of Forest Ecology, Environment and Protection)
EPI_ISL_14836025	A/Whooper swan/Sanmenxia/Y20/2020	A / H5N8	Asia / China / Henan Province	Zeyu Yang (Chinese Academy of Forestry / Research Institute of Forest Ecology, Environment and Protection)
EPI_ISL_14836026	A/Whooper swan/Sanmenxia/Y21/2020	A / H5N8	Asia / China / Henan Province	Zeyu Yang (Chinese Academy of Forestry / Research Institute of Forest Ecology, Environment and Protection)
EPI_ISL_14836027	A/Whooper swan/Sanmenxia/Y23-1/2020	A / H5N8	Asia / China / Henan Province	Zeyu Yang (Chinese Academy of Forestry / Research Institute of Forest Ecology, Environment and Protection)
EPI_ISL_14836077	A/Whooper swan/Sanmenxia/Y23-2/2020	A / H5N8	Asia / China / Henan Province	Zeyu Yang (Chinese Academy of Forestry / Research Institute of Forest Ecology, Environment and Protection)
EPI_ISL_14836078	A/Whooper swan/Sanmenxia/G27/2020	A / H5N8	Asia / China / Henan Province	Zeyu Yang (Chinese Academy of Forestry / Research Institute of Forest Ecology, Environment and Protection)
EPI_ISL_14836079	A/Whooper swan/Sanmenxia/G28/2020	A / H5N8	Asia / China / Henan Province	Zeyu Yang (Chinese Academy of Forestry / Research Institute of Forest Ecology, Environment and Protection)
EPI_ISL_14836080	A/Whooper swan/Sanmenxia/G29/2020	A / H5N8	Asia / China / Henan Province	Zeyu Yang (Chinese Academy of Forestry / Research Institute of Forest Ecology, Environment and Protection)
EPI_ISL_14836081	A/Whooper swan/Sanmenxia/Y26/2020	A / H5N8	Asia / China / Henan Province	Zeyu Yang (Chinese Academy of Forestry / Research Institute of Forest Ecology, Environment and Protection)
EPI_ISL_14836082	A/Whooper swan/Sanmenxia/Y27/2020	A / H5N8	Asia / China / Henan Province	Zeyu Yang (Chinese Academy of Forestry / Research Institute of Forest Ecology, Environment and Protection)
EPI_ISL_14836083	A/Whooper swan/Sanmenxia/Y31/2020	A / H5N8	Asia / China / Henan Province	Zeyu Yang (Chinese Academy of Forestry / Research Institute of Forest Ecology, Environment and Protection)
EPI_ISL_14836086	A/Whooper swan/Sanmenxia/Y36/2020	A / H5N8	Asia / China / Henan Province	Zeyu Yang (Chinese Academy of Forestry / Research Institute of Forest Ecology, Environment and Protection)
EPI_ISL_14836088	A/Whooper swan/Sanmenxia/Y48A/2020	A / H5N8	Asia / China / Henan Province	Zeyu Yang (Chinese Academy of Forestry / Research Institute of Forest Ecology, Environment and Protection)
EPI_ISL_14836098	A/Whooper swan/Sanmenxia/Y54/2020	A / H5N8	Asia / China / Henan Province	Zeyu Yang (Chinese Academy of Forestry / Research Institute of Forest Ecology, Environment and Protection)
EPI_ISL_14836101	A/Whooper swan/Sanmenxia/Y57/2020	A / H5N8	Asia / China / Henan Province	Zeyu Yang (Chinese Academy of Forestry / Research Institute of Forest Ecology, Environment and Protection)
EPI_ISL_14836104	A/Whooper swan/Sanmenxia/G48B/2020	A / H5N8	Asia / China / Henan Province	Zeyu Yang (Chinese Academy of Forestry / Research Institute of Forest Ecology, Environment and Protection)
EPI_ISL_14836369	A/Whooper swan/Sanmenxia/Y48B/2020	A / H5N8	Asia / China / Henan Province	Zeyu Yang (Chinese Academy of Forestry / Research Institute of Forest Ecology, Environment and Protection)
EPI_ISL_14836371	A/Whooper swan/Sanmenxia/Y49/2020	A / H5N8	Asia / China / Henan Province	Zeyu Yang (Chinese Academy of Forestry / Research Institute of Forest Ecology, Environment and Protection)
EPI_ISL_14836374	A/Whooper swan/Sanmenxia/G25/2020	A / H5N8	Asia / China / Henan Province	Zeyu Yang (Chinese Academy of Forestry / Research Institute of Forest Ecology, Environment and Protection)
EPI_ISL_14836376	A/Whooper swan/Sanmenxia/Y25/2020	A / H5N8	Asia / China / Henan Province	Zeyu Yang (Chinese Academy of Forestry / Research Institute of Forest Ecology, Environment and Protection)
EPI_ISL_14836426	A/Whooper swan/Sanmenxia/B560/2020	A / H5N8	Asia / China / Henan Province	Zeyu Yang (Chinese Academy of Forestry / Research Institute of Forest Ecology, Environment and Protection)
EPI_ISL_14836593	A/Whooper swan/Sanmenxia/B312/2020	A / H5N8	Asia / China / Henan Province	Zeyu Yang (Chinese Academy of Forestry / Research Institute of Forest Ecology, Environment and Protection)
EPI_ISL_14838179	A/Whooper swan/Sanmenxia/B497/2020	A / H5N8	Asia / China / Henan Province	Zeyu Yang (Chinese Academy of Forestry / Research Institute of Forest Ecology, Environment and Protection)
EPI_ISL_14839943	A/Whooper swan/Sanmenxia/B1109/2021	A / H5N8	Asia / China / Henan Province	Zeyu Yang (Chinese Academy of Forestry / Research Institute of Forest Ecology, Environment and Protection)
EPI_ISL_14840034	A/Whooper swan/Sanmenxia/B1770/2020	A / H5N8	Asia / China / Henan Province	Zeyu Yang (Chinese Academy of Forestry / Research Institute of Forest Ecology, Environment and Protection)
EPI_ISL_14840053	A/Whooper swan/Sanmenxia/B2465/2021	A / H5N8	Asia / China / Henan Province	Zeyu Yang (Chinese Academy of Forestry / Research Institute of Forest Ecology, Environment and Protection)
EPI_ISL_14841913	A/mallard/22P019377/France/2022	A / H5N1	Europe / France / Picardie / Departement de la Somme	Francois-Xavier Briand (ANSES Agence Nationale De Securite Sanitaire De L'alimentation / Laboratoire de Ploufragan-Plouzané)

EPI_ISL_14845579	A/whooper_swan/Rongcheng/M82/2021	A / H5	Asia / China / Shandong Province	Zeyu Yang (Chinese Academy of Forestry / Research Institute of Forest Ecology, Environment and Protection)
EPI_ISL_14857053	A/chicken/Kursk/230-2V/2022	A / H5N1	Europe / Russian Federation / Kursk Oblast	Natalia Goncharova (State Research Center of Virology and Biotechnology (VECTOR) / Emerging Zoonotic Diseases and Influenza)
EPI_ISL_14857054	A/chicken/Kursk/230-4V/2022	A / H5N1	Europe / Russian Federation / Kursk Oblast	Natalia Goncharova (State Research Center of Virology and Biotechnology (VECTOR) / Emerging Zoonotic Diseases and Influenza)
EPI_ISL_14857055	A/chicken/Kursk/230-5V/2022	A / H5N1	Europe / Russian Federation / Kursk Oblast	Natalia Goncharova (State Research Center of Virology and Biotechnology (VECTOR) / Emerging Zoonotic Diseases and Influenza)
EPI_ISL_14857056	A/chicken/Kursk/230-7V/2022	A / H5N1	Europe / Russian Federation / Kursk Oblast	Natalia Goncharova (State Research Center of Virology and Biotechnology (VECTOR) / Emerging Zoonotic Diseases and Influenza)
EPI_ISL_14857057	A/chicken/Kursk/230-8V/2022	A / H5N1	Europe / Russian Federation / Kursk Oblast	Natalia Goncharova (State Research Center of Virology and Biotechnology (VECTOR) / Emerging Zoonotic Diseases and Influenza)
EPI_ISL_14857058	A/chicken/Kursk/230-10V/2022	A / H5N1	Europe / Russian Federation / Kursk Oblast	Natalia Goncharova (State Research Center of Virology and Biotechnology (VECTOR) / Emerging Zoonotic Diseases and Influenza)
EPI_ISL_14857059	A/chicken/Kursk/230-15V/2022	A / H5N1	Europe / Russian Federation / Kursk Oblast	Natalia Goncharova (State Research Center of Virology and Biotechnology (VECTOR) / Emerging Zoonotic Diseases and Influenza)
EPI_ISL_14857060	A/chicken/Kursk/234-19V/2022	A / H5N1	Europe / Russian Federation / Kursk Oblast	Natalia Goncharova (State Research Center of Virology and Biotechnology (VECTOR) / Emerging Zoonotic Diseases and Influenza)
EPI_ISL_14857061	A/quail/Kursk/234-20V/2022	A / H5N1	Europe / Russian Federation / Kursk Oblast	Natalia Goncharova (State Research Center of Virology and Biotechnology (VECTOR) / Emerging Zoonotic Diseases and Influenza)
EPI_ISL_14857062	A/chicken/Magadan/235-57V/2022	A / H5N1	Europe / Russian Federation / Magadan Oblast	Natalia Goncharova (State Research Center of Virology and Biotechnology (VECTOR) / Emerging Zoonotic Diseases and Influenza)
EPI_ISL_14857063	A/chicken/Magadan/235-58V/2022	A / H5N1	Europe / Russian Federation / Magadan Oblast	Natalia Goncharova (State Research Center of Virology and Biotechnology (VECTOR) / Emerging Zoonotic Diseases and Influenza)
EPI_ISL_14857064	A/chicken/Magadan/235-59V/2022	A / H5N1	Europe / Russian Federation / Magadan Oblast	Natalia Goncharova (State Research Center of Virology and Biotechnology (VECTOR) / Emerging Zoonotic Diseases and Influenza)
EPI_ISL_14857065	A/chicken/Magadan/235-60V/2022	A / H5N1	Europe / Russian Federation / Magadan Oblast	Natalia Goncharova (State Research Center of Virology and Biotechnology (VECTOR) / Emerging Zoonotic Diseases and Influenza)
EPI_ISL_14864637	A/goose/England/317610/2022	A / H5N1	Europe / United Kingdom / England	Alex Byrne (Animal and Plant Health Agency (APHA) / Virology Department)
EPI_ISL_14864646	A/Canada_goose/England/320660/2022	A / H5N1	Europe / United Kingdom / England	Alex Byrne (Animal and Plant Health Agency (APHA) / Virology Department)
EPI_ISL_14864650	A/turkey/England/111923/2022	A / H5N1	Europe / United Kingdom / England	Alex Byrne (Animal and Plant Health Agency (APHA) / Virology Department)
EPI_ISL_14867042	A/duck/Kagoshima/NIES229/2020	A / H5N8	Asia / Japan / Kagoshima	
EPI_ISL_14871335	A/poultry/Benin/21-A-08-035-O/2021	A / H5N1	Africa / Benin	
EPI_ISL_14871336	A/poultry/Benin/21-A-08-009-O/2021	A / H5N1	Africa / Benin	
EPI_ISL_14871337	A/poultry/Benin/21-A-08-033-O/2021	A / H5N1	Africa / Benin	
EPI_ISL_14871338	A/poultry/Benin/21-A-08-034-O/2021	A / H5N1	Africa / Benin	
EPI_ISL_14871339	A/poultry/Benin/21-A-09-031-O/2021	A / H5N1	Africa / Benin	
EPI_ISL_14886367	A/duck/Bangladesh/51602/2021	A / H5N1	Asia / Bangladesh	
EPI_ISL_14886675	A/duck/Bangladesh/51601/2021	A / H5N1	Asia / Bangladesh	
EPI_ISL_14886825	A/duck/Bangladesh/51600/2021	A / H5N1	Asia / Bangladesh	
EPI_ISL_14901700	A/duck/Egypt/Ismailia/2021	A / H5N8	Africa / Egypt	
EPI_ISL_14917968	A/common_murre/Poland/MB151/2022	A / H5N1	Europe / Poland / Pomeranian Voivodeship	Edyta ?wi?to? (National Veterinary Research Institut Poland, PIWet-PIB)
EPI_ISL_14917979	A/herring_gull/Poland/MB138/2022	A / H5N1	Europe / Poland / Pomeranian Voivodeship	Edyta ?wi?to? (National Veterinary Research Institut Poland, PIWet-PIB)
EPI_ISL_14917999	A/black-headed_gull/Poland/MB139/2022	A / H5N1	Europe / Poland / Pomeranian Voivodeship	Edyta ?wi?to? (National Veterinary Research Institut Poland, PIWet-PIB)
EPI_ISL_14933724	A/duck/France/22P020165/2022	A / H5N1	Europe / France / Rhone-Alpes / Departement de l'Ain	Francois-Xavier Briand (ANSES Agence Nationale De Securite Sanitaire De L'alimentation / Laboratoire de Ploufragan-Plouzané)
EPI_ISL_14936999	A/bald eagle/Kansas/W22-197/2022	A / H5N1	North America / United States	
EPI_ISL_14937000	A/snow goose/Kansas/W22-199D/2022	A / H5N1	North America / United States	
EPI_ISL_14937001	A/snow goose/Kansas/W22-199F/2022	A / H5N1	North America / United States	
EPI_ISL_14937002	A/bald eagle/Georgia/W22-194B/2022	A / H5N1	North America / United States	
EPI_ISL_14937069	A/bald eagle/Florida/W22-191/2022	A / H5N1	North America / United States	
EPI_ISL_14937070	A/bald eagle/Florida/W22-195/2022	A / H5N1	North America / United States	
EPI_ISL_14937071	A/bald eagle/Georgia/W22-202/2022	A / H5N1	North America / United States	
EPI_ISL_14937099	A/snow goose/Kansas/W22-199A/2022	A / H5N1	North America / United States	
EPI_ISL_14937100	A/snow goose/Kansas/W22-199B/2022	A / H5N1	North America / United States	
EPI_ISL_14937101	A/bald eagle/South Carolina/W22-205/2022	A / H5N1	North America / United States	
EPI_ISL_14937102	A/snow goose/Kansas/W22-199E/2022	A / H5N1	North America / United States	
EPI_ISL_14937103	A/bald eagle/Georgia/W22-194A/2022	A / H5N1	North America / United States	
EPI_ISL_14937104	A/snow goose/Kansas/W22-199C/2022	A / H5N1	North America / United States	
EPI_ISL_14937105	A/bald eagle/Florida/W22-189/2022	A / H5N1	North America / United States	
EPI_ISL_14937233	A/Turkey/Egypt/Giza/2021	A / H5N8	Africa / Egypt	
EPI_ISL_15004053	A/Pekin duck/Indiana/22-010611-001-original/2022	A / H5N1	North America / United States / Indiana / Elkhart County	Mary Lea Killian (National Veterinary Services Laboratories - USDA / Diagnostic Virology Laboratory)
EPI_ISL_15004054	A/Pekin duck/Indiana/22-010624-001-original/2022	A / H5N1	North America / United States / Indiana / Elkhart County	Mary Lea Killian (National Veterinary Services Laboratories - USDA / Diagnostic Virology Laboratory)
EPI_ISL_15004055	A/turkey/South Dakota/22-010639-001-original/2022	A / H5N1	North America / United States / South Dakota / Clark County	Mary Lea Killian (National Veterinary Services Laboratories - USDA / Diagnostic Virology Laboratory)
EPI_ISL_15004056	A/turkey/South Dakota/22-010639-002-original/2022	A / H5N1	North America / United States / South Dakota / Clark County	Mary Lea Killian (National Veterinary Services Laboratories - USDA / Diagnostic Virology Laboratory)
EPI_ISL_15004057	A/turkey/Minnesota/22-010652-001-original/2022	A / H5N1	North America / United States / Minnesota / Otter Tail County	Mary Lea Killian (National Veterinary Services Laboratories - USDA / Diagnostic Virology Laboratory)
EPI_ISL_15004058	A/turkey/Minnesota/22-010652-002-original/2022	A / H5N1	North America / United States / Minnesota / Otter Tail County	Mary Lea Killian (National Veterinary Services Laboratories - USDA / Diagnostic Virology Laboratory)
EPI_ISL_15004059	A/turkey/Minnesota/22-010654-001-original/2022	A / H5N1	North America / United States / Minnesota / Kandiyohi County	Mary Lea Killian (National Veterinary Services Laboratories - USDA / Diagnostic Virology Laboratory)
EPI_ISL_15004060	A/chicken/North Dakota/22-010657-001-original/2022	A / H5N1	North America / United States / North Dakota / Stutsman County	Mary Lea Killian (National Veterinary Services Laboratories - USDA / Diagnostic Virology Laboratory)
EPI_ISL_15004061	A/goose/North Dakota/22-010657-002-original/2022	A / H5N1	North America / United States / North Dakota / Stutsman County	Mary Lea Killian (National Veterinary Services Laboratories - USDA / Diagnostic Virology Laboratory)
EPI_ISL_15004062	A/chicken/Colorado/22-010668-001-original/2022	A / H5N1	North America / United States / Colorado / Pitkin County	Mary Lea Killian (National Veterinary Services Laboratories - USDA / Diagnostic Virology Laboratory)
EPI_ISL_15004063	A/turkey/South Dakota/22-010765-001-original/2022	A / H5N1	North America / United States / South Dakota / Edmunds County	Mary Lea Killian (National Veterinary Services Laboratories - USDA / Diagnostic Virology Laboratory)
EPI_ISL_15004064	A/turkey/Minnesota/22-010770-002-original/2022	A / H5N1	North America / United States / Minnesota / Yankton County	Mary Lea Killian (National Veterinary Services Laboratories - USDA / Diagnostic Virology Laboratory)
EPI_ISL_15004065	A/turkey/Minnesota/22-010770-003-original/2022	A / H5N1	North America / United States / Minnesota / Yankton County	Mary Lea Killian (National Veterinary Services Laboratories - USDA / Diagnostic Virology Laboratory)
EPI_ISL_15004066	A/turkey/Minnesota/22-010771-001-original/2022	A / H5N1	North America / United States / Minnesota / Le Sueur County	Mary Lea Killian (National Veterinary Services Laboratories - USDA / Diagnostic Virology Laboratory)
EPI_ISL_15004067	A/turkey/Minnesota/22-010771-003-original/2022	A / H5N1	North America / United States / Minnesota / Le Sueur County	Mary Lea Killian (National Veterinary Services Laboratories - USDA / Diagnostic Virology Laboratory)
EPI_ISL_15004068	A/turkey/Minnesota/22-010772-004-original/2022	A / H5N1	North America / United States / Minnesota / Stearns County	Mary Lea Killian (National Veterinary Services Laboratories - USDA / Diagnostic Virology Laboratory)
EPI_ISL_15004069	A/turkey/Minnesota/22-010773-001-original/2022	A / H5N1	North America / United States / Minnesota / Swift County	Mary Lea Killian (National Veterinary Services Laboratories - USDA / Diagnostic Virology Laboratory)
EPI_ISL_15004070	A/goose/Michigan/22-010845-001-original/2022	A / H5N1	North America / United States / Michigan / Menominee County	Mary Lea Killian (National Veterinary Services Laboratories - USDA / Diagnostic Virology Laboratory)
EPI_ISL_15004071	A/goose/Michigan/22-010845-002-original/2022	A / H5N1	North America / United States / Michigan / Menominee County	Mary Lea Killian (National Veterinary Services Laboratories - USDA / Diagnostic Virology Laboratory)
EPI_ISL_15004072	A/duck/Michigan/22-010845-003-original/2022	A / H5N1	North America / United States / Michigan / Menominee County	Mary Lea Killian (National Veterinary Services Laboratories - USDA / Diagnostic Virology Laboratory)
EPI_ISL_15004372	A/Amazon parrot/Michigan/22-010848-001-original/2022	A / H5N1	North America / United States / Michigan / Washtenaw County	Mary Lea Killian (National Veterinary Services Laboratories - USDA / Diagnostic Virology Laboratory)
EPI_ISL_15004373	A/Catalina macaw/Michigan/22-010848-002-original/2022	A / H5N1	North America / United States / Michigan / Washtenaw County	Mary Lea Killian (National Veterinary Services Laboratories - USDA / Diagnostic Virology Laboratory)
EPI_ISL_15004374	A/chicken/Nebraska/22-010905-001-original/2022	A / H5N1	North America / United States / Nebraska / Dixon County	Mary Lea Killian (National Veterinary Services Laboratories - USDA / Diagnostic Virology Laboratory)
EPI_ISL_15004375	A/chicken/Montana/22-010923-005-original/2022	A / H5N1	North America / United States / Montana / Toole County	Mary Lea Killian (National Veterinary Services Laboratories - USDA / Diagnostic Virology Laboratory)
EPI_ISL_15004376	A/chicken/Minnesota/22-010928-001-original/2022	A / H5N1	North America / United States / Minnesota / Benton County	Mary Lea Killian (National Veterinary Services Laboratories - USDA / Diagnostic Virology Laboratory)
EPI_ISL_15004377	A/turkey/Minnesota/22-010928-002-original/2022	A / H5N1	North America / United States / Minnesota / Benton County	Mary Lea Killian (National Veterinary Services Laboratories - USDA / Diagnostic Virology Laboratory)

EPI_ISL_15004378	A/guinea fowl/Minnesota/22-010928-003-original/2022	A / H5N1	North America / United States / Minnesota / Benton County	Mary Lea Killian (National Veterinary Services Laboratories - USDA / Diagnostic Virology Laboratory)
EPI_ISL_15004379	A/domestic duck/Minnesota/22-010928-004-original/2022	A / H5N1	North America / United States / Minnesota / Benton County	Mary Lea Killian (National Veterinary Services Laboratories - USDA / Diagnostic Virology Laboratory)
EPI_ISL_15004380	A/chicken/North Carolina/22-010929-001-original/2022	A / H5N1	North America / United States / North Carolina / Wayne County	Mary Lea Killian (National Veterinary Services Laboratories - USDA / Diagnostic Virology Laboratory)
EPI_ISL_15004381	A/chicken/North Carolina/22-010929-002-original/2022	A / H5N1	North America / United States / North Carolina / Wayne County	Mary Lea Killian (National Veterinary Services Laboratories - USDA / Diagnostic Virology Laboratory)
EPI_ISL_15004383	A/turkey/Wisconsin/22-010935-002-original/2022	A / H5N1	North America / United States / Wisconsin / Barron County	Mary Lea Killian (National Veterinary Services Laboratories - USDA / Diagnostic Virology Laboratory)
EPI_ISL_15004384	A/chicken/North Dakota/22-010977-001-original/2022	A / H5N1	North America / United States / North Dakota / Sheridan County	Mary Lea Killian (National Veterinary Services Laboratories - USDA / Diagnostic Virology Laboratory)
EPI_ISL_15004385	A/chicken/North Dakota/22-010977-002-original/2022	A / H5N1	North America / United States / North Dakota / Sheridan County	Mary Lea Killian (National Veterinary Services Laboratories - USDA / Diagnostic Virology Laboratory)
EPI_ISL_15004386	A/turkey/Minnesota/22-010991-001-original/2022	A / H5N1	North America / United States / Minnesota / Morrison County	Mary Lea Killian (National Veterinary Services Laboratories - USDA / Diagnostic Virology Laboratory)
EPI_ISL_15004387	A/turkey/Minnesota/22-010991-002-original/2022	A / H5N1	North America / United States / Minnesota / Morrison County	Mary Lea Killian (National Veterinary Services Laboratories - USDA / Diagnostic Virology Laboratory)
EPI_ISL_15004388	A/turkey/Minnesota/22-010992-001-original/2022	A / H5N1	North America / United States / Minnesota / Yellow Medicine County	Mary Lea Killian (National Veterinary Services Laboratories - USDA / Diagnostic Virology Laboratory)
EPI_ISL_15004389	A/turkey/Minnesota/22-010992-002-original/2022	A / H5N1	North America / United States / Minnesota / Yellow Medicine County	Mary Lea Killian (National Veterinary Services Laboratories - USDA / Diagnostic Virology Laboratory)
EPI_ISL_15004390	A/chicken/Minnesota/22-010993-001-original/2022	A / H5N1	North America / United States / Minnesota / Morrison County	Mary Lea Killian (National Veterinary Services Laboratories - USDA / Diagnostic Virology Laboratory)
EPI_ISL_15004391	A/chicken/Minnesota/22-010993-004-original/2022	A / H5N1	North America / United States / Minnesota / Morrison County	Mary Lea Killian (National Veterinary Services Laboratories - USDA / Diagnostic Virology Laboratory)
EPI_ISL_15004392	A/chicken/Minnesota/22-010994-002-original/2022	A / H5N1	North America / United States / Minnesota / Morrison County	Mary Lea Killian (National Veterinary Services Laboratories - USDA / Diagnostic Virology Laboratory)
EPI_ISL_15004393	A/turkey/Minnesota/22-010995-001-original/2022	A / H5N1	North America / United States / Minnesota / Kandiyohi County	Mary Lea Killian (National Veterinary Services Laboratories - USDA / Diagnostic Virology Laboratory)
EPI_ISL_15004394	A/turkey/Minnesota/22-010995-002-original/2022	A / H5N1	North America / United States / Minnesota / Kandiyohi County	Mary Lea Killian (National Veterinary Services Laboratories - USDA / Diagnostic Virology Laboratory)
EPI_ISL_15004395	A/pheasant/South Dakota/22-011098-001-original/2022	A / H5N1	North America / United States / South Dakota / Deuel County	Mary Lea Killian (National Veterinary Services Laboratories - USDA / Diagnostic Virology Laboratory)
EPI_ISL_15004396	A/chicken/North Dakota/22-011109-001-original/2022	A / H5N1	North America / United States / North Dakota / Barnes County	Mary Lea Killian (National Veterinary Services Laboratories - USDA / Diagnostic Virology Laboratory)
EPI_ISL_15004397	A/turkey/Kansas/22-011119-004-original/2022	A / H5N1	North America / United States / Kansas / McPherson County	Mary Lea Killian (National Veterinary Services Laboratories - USDA / Diagnostic Virology Laboratory)
EPI_ISL_15004398	A/chicken/Michigan/22-011121-001-original/2022	A / H5N1	North America / United States / Michigan / Menominee County	Mary Lea Killian (National Veterinary Services Laboratories - USDA / Diagnostic Virology Laboratory)
EPI_ISL_15004399	A/domestic duck/Michigan/22-011121-002-original/2022	A / H5N1	North America / United States / Michigan / Menominee County	Mary Lea Killian (National Veterinary Services Laboratories - USDA / Diagnostic Virology Laboratory)
EPI_ISL_15004400	A/chicken/Michigan/22-011121-003-original/2022	A / H5N1	North America / United States / Michigan / Menominee County	Mary Lea Killian (National Veterinary Services Laboratories - USDA / Diagnostic Virology Laboratory)
EPI_ISL_15004401	A/domestic goose/Michigan/22-011121-005-original/2022	A / H5N1	North America / United States / Michigan / Menominee County	Mary Lea Killian (National Veterinary Services Laboratories - USDA / Diagnostic Virology Laboratory)
EPI_ISL_15004402	A/chicken/Montana/22-010923-004-original/2022	A / H5N1	North America / United States / Montana / Toole County	Mary Lea Killian (National Veterinary Services Laboratories - USDA / Diagnostic Virology Laboratory)
EPI_ISL_15004403	A/turkey/Minnesota/22-011140-001-original/2022	A / H5N1	North America / United States / Minnesota / Blue Earth County	Mary Lea Killian (National Veterinary Services Laboratories - USDA / Diagnostic Virology Laboratory)
EPI_ISL_15004404	A/turkey/Minnesota/22-011141-001-original/2022	A / H5N1	North America / United States / Minnesota / Otter Tail County	Mary Lea Killian (National Veterinary Services Laboratories - USDA / Diagnostic Virology Laboratory)
EPI_ISL_15004405	A/turkey/Minnesota/22-011141-002-original/2022	A / H5N1	North America / United States / Minnesota / Otter Tail County	Mary Lea Killian (National Veterinary Services Laboratories - USDA / Diagnostic Virology Laboratory)
EPI_ISL_15004406	A/turkey/Minnesota/22-011142-001-original/2022	A / H5N1	North America / United States / Minnesota / Waseca County	Mary Lea Killian (National Veterinary Services Laboratories - USDA / Diagnostic Virology Laboratory)
EPI_ISL_15004407	A/turkey/Minnesota/22-011142-002-original/2022	A / H5N1	North America / United States / Minnesota / Waseca County	Mary Lea Killian (National Veterinary Services Laboratories - USDA / Diagnostic Virology Laboratory)
EPI_ISL_15004408	A/chicken/Wisconsin/22-011213-001-original/2022	A / H5N1	North America / United States / Wisconsin / Sheboygan County	Mary Lea Killian (National Veterinary Services Laboratories - USDA / Diagnostic Virology Laboratory)
EPI_ISL_15004409	A/chicken/Wisconsin/22-011213-002-original/2022	A / H5N1	North America / United States / Wisconsin / Sheboygan County	Mary Lea Killian (National Veterinary Services Laboratories - USDA / Diagnostic Virology Laboratory)
EPI_ISL_15004410	A/chicken/North Dakota/22-011286-001-original/2022	A / H5N1	North America / United States / North Dakota / LaMoure County	Mary Lea Killian (National Veterinary Services Laboratories - USDA / Diagnostic Virology Laboratory)
EPI_ISL_15004411	A/chicken/Wisconsin/22-011334-002-original/2022	A / H5N1	North America / United States / Wisconsin / Columbia County	Mary Lea Killian (National Veterinary Services Laboratories - USDA / Diagnostic Virology Laboratory)
EPI_ISL_15004412	A/chicken/Wisconsin/22-011334-003-original/2022	A / H5N1	North America / United States / Wisconsin / Columbia County	Mary Lea Killian (National Veterinary Services Laboratories - USDA / Diagnostic Virology Laboratory)
EPI_ISL_15004413	A/chicken/Minnesota/22-010994-001-original/2022	A / H5N1	North America / United States / Minnesota / Morrison County	Mary Lea Killian (National Veterinary Services Laboratories - USDA / Diagnostic Virology Laboratory)
EPI_ISL_15022831	A/swan/Lithuania/1220PG1_22VIR7255-1/2022	A / H5N1	Europe / Lithuania	Giacomo Barbierato (Istituto Zooprofilattico Sperimentale delle Venezie / Ricerca e innovazione)
EPI_ISL_15023114	A/stork/Lithuania/4371PG1_22VIR7255-3/2022	A / H5N1	Europe / Lithuania	Giacomo Barbierato (Istituto Zooprofilattico Sperimentale delle Venezie / Ricerca e innovazione)
EPI_ISL_15038822	A/chicken/England/114595/2022	A / H5N1	Europe / United Kingdom / England	Alex Byrne (Animal and Plant Health Agency (APHA) / Virology Department)
EPI_ISL_15063427	A/chicken/Ehime/TU10-2-13/2022	A / H5N1	Asia / Japan / Ehime	
EPI_ISL_15063428	A/environment/Akita/TU1-49/2021	A / H5N8	Asia / Japan / Akita	
EPI_ISL_15063618	A/snow goose/North Dakota/N22-06/2022	A / H5N1	North America / United States	
EPI_ISL_15063648	A/snow goose/North Dakota/N22-04/2022	A / H5N1	North America / United States	
EPI_ISL_15063668	A/snow goose/North Dakota/N22-05/2022	A / H5N1	North America / United States	
EPI_ISL_15063669	A/ring-billed gull/Florida/W22-169/2022	A / H5N1	North America / United States	
EPI_ISL_15063839	A/bald eagle/Virginia/W22-306/2022	A / H5N1	North America / United States	
EPI_ISL_15063841	A/muscovy duck/Florida/W22-306/2022	A / H5N1	North America / United States	
EPI_ISL_15063842	A/bald eagle/North Carolina/W22-186/2022	A / H5N1	North America / United States	
EPI_ISL_15063844	A/gull/Florida/W22-162/2022	A / H5N1	North America / United States	
EPI_ISL_15063845	A/snow goose/Kansas/W22-177B/2022	A / H5N1	North America / United States	
EPI_ISL_15063846	A/bald eagle/Florida/W22-134-OP/2022	A / H5N1	North America / United States	
EPI_ISL_15063847	A/bald eagle/North Carolina/W22-229/2022	A / H5N1	North America / United States	
EPI_ISL_15069397	A/bottlenose dolphin/Florida/UFTt2203/2022	A / H5N1	North America / United States / Florida / Dixie County	Thomas Paul Fabrizio (St. Jude Children's Research Hospital / Infectious Diseases)
EPI_ISL_15069398	A/European Herring Gull/Netherlands/12/2022	A / H5N1	Europe / Netherlands / Neeltje Jans	Sanne Thewessen (Erasmus Medical Center / Viroscience)
EPI_ISL_15069399	A/Black-headed gull/Netherlands/10/2022	A / H5N1	Europe / Netherlands / Neeltje Jans	Sanne Thewessen (Erasmus Medical Center / Viroscience)
EPI_ISL_15069400	A/Mute Swan/Netherlands/1/2022	A / H5N1	Europe / Netherlands / Weversinlaag, Schouwen	Sanne Thewessen (Erasmus Medical Center / Viroscience)
EPI_ISL_15069401	A/Common Tern/Netherlands/26/2022	A / H5N1	Europe / Netherlands / Weversinlaag	Sanne Thewessen (Erasmus Medical Center / Viroscience)
EPI_ISL_15072537	A/chicken/Hokkaido/A-1/2022	A / H5	Asia / Japan / Hokkaido / Abashiri	Norikazu Isoda (Graduate School of Veterinary Medicine, Hokkaido University / Laboratory of Microbiology)
EPI_ISL_15072538	A/chicken/Hokkaido/I-1/2022	A / H5N1	Asia / Japan / Hokkaido / Shiraoi	Norikazu Isoda (Graduate School of Veterinary Medicine, Hokkaido University / Laboratory of Microbiology)
EPI_ISL_15072539	A/emu/Hokkaido/A-2/2022	A / H5N1	Asia / Japan / Hokkaido / Abashiri	Norikazu Isoda (Graduate School of Veterinary Medicine, Hokkaido University / Laboratory of Microbiology)
EPI_ISL_15072540	A/crow/Hokkaido/HU-1/2022	A / H5N1	Asia / Japan / Hokkaido / Sapporo	Norikazu Isoda (Graduate School of Veterinary Medicine, Hokkaido University / Laboratory of Microbiology)
EPI_ISL_15077371	A/chicken/Idaho/22-011347-004-original/2022	A / H5N1	North America / United States / Idaho / Gooding County	Mary Lea Killian (National Veterinary Services Laboratories - USDA / Diagnostic Virology Laboratory)
EPI_ISL_15077373	A/chicken/Pennsylvania/22-012092-006-original/2022	A / H5N1	North America / United States / Pennsylvania / Lancaster County	Mary Lea Killian (National Veterinary Services Laboratories - USDA / Diagnostic Virology Laboratory)
EPI_ISL_15077374	A/black vulture/Florida/22-010358-001-original/2022	A / H5N1	North America / United States / Florida / Citrus County	Mary Lea Killian (National Veterinary Services Laboratories - USDA / Diagnostic Virology Laboratory)
EPI_ISL_15077375	A/red-shouldered hawk/Minnesota/22-012000-004-original/2(	A / H5N1	North America / United States / Minnesota	Mary Lea Killian (National Veterinary Services Laboratories - USDA / Diagnostic Virology Laboratory)
EPI_ISL_15077376	A/black vulture/Florida/22-012331-001-original/2022	A / H5N1	North America / United States / Florida / Brevard County	Mary Lea Killian (National Veterinary Services Laboratories - USDA / Diagnostic Virology Laboratory)
EPI_ISL_15077377	A/black vulture/Florida/22-012333-001-original/2022	A / H5N1	North America / United States / Florida / Brevard County	Mary Lea Killian (National Veterinary Services Laboratories - USDA / Diagnostic Virology Laboratory)
EPI_ISL_15078237	A/Canada goose/Wyoming/22-011671-001-original/2022	A / H5N1	North America / United States / Wyoming / Fremont County	Mary Lea Killian (National Veterinary Services Laboratories - USDA / Diagnostic Virology Laboratory)
EPI_ISL_15078238	A/turkey/Iowa/22-012098-001-original/2022	A / H5N1	North America / United States / Iowa / Bremer County	Mary Lea Killian (National Veterinary Services Laboratories - USDA / Diagnostic Virology Laboratory)
EPI_ISL_15078239	A/black vulture/Maryland/22-012407-001-original/2022	A / H5N1	North America / United States / Maryland / Cecil County	Mary Lea Killian (National Veterinary Services Laboratories - USDA / Diagnostic Virology Laboratory)
EPI_ISL_15078240	A/Cooper's hawk/Minnesota/22-012931-001-original/2022	A / H5N1	North America / United States / Minnesota / Ramsey County	Mary Lea Killian (National Veterinary Services Laboratories - USDA / Diagnostic Virology Laboratory)
EPI_ISL_15078241	A/bald eagle/Wyoming/22-013015-001-original/2022	A / H5N1	North America / United States / Wyoming / Park County	Mary Lea Killian (National Veterinary Services Laboratories - USDA / Diagnostic Virology Laboratory)
EPI_ISL_15078244	A/chicken/Michigan/22-013961-001-original/2022	A / H5N1	North America / United States / Michigan / Oakland County / Oakland Count	Mary Lea Killian (National Veterinary Services Laboratories - USDA / Diagnostic Virology Laboratory)

EPI_ISL_15078245	A/fox/Michigan/22-014536-004-original/2022	A / H5N1	North America / United States / Michigan / Lapeer County	Mary Lea Killian (National Veterinary Services Laboratories - USDA / Diagnostic Virology Laboratory)
EPI_ISL_15078246	A/fox/Wisconsin/22-014746-008-original/2022	A / H5N1	North America / United States / Wisconsin / Adam County	Mary Lea Killian (National Veterinary Services Laboratories - USDA / Diagnostic Virology Laboratory)
EPI_ISL_15078247	A/fox/Wisconsin/22-014746-030-original/2022	A / H5N1	North America / United States / Wisconsin / Grant County	Mary Lea Killian (National Veterinary Services Laboratories - USDA / Diagnostic Virology Laboratory)
EPI_ISL_15078248	A/bobcat/Wisconsin/22-016051-001-original/2022	A / H5N1	North America / United States / Wisconsin	Mary Lea Killian (National Veterinary Services Laboratories - USDA / Diagnostic Virology Laboratory)
EPI_ISL_15078249	A/fox/Minnesota/22-016487-001-original/2022	A / H5N1	North America / United States / Minnesota / Itasca County	Mary Lea Killian (National Veterinary Services Laboratories - USDA / Diagnostic Virology Laboratory)
EPI_ISL_15078250	A/Virginia opossum/Iowa/22-016780-001-original/2022	A / H5N1	North America / United States / Iowa / Buchanan County	Mary Lea Killian (National Veterinary Services Laboratories - USDA / Diagnostic Virology Laboratory)
EPI_ISL_15078251	A/red fox/North Dakota/22-017354-001-original/2022	A / H5N1	North America / United States / North Dakota / Burleigh County	Mary Lea Killian (National Veterinary Services Laboratories - USDA / Diagnostic Virology Laboratory)
EPI_ISL_15078252	A/raccoon/Washington/22-018406-002-original/2022	A / H5N1	North America / United States / Washington / Franklin County	Mary Lea Killian (National Veterinary Services Laboratories - USDA / Diagnostic Virology Laboratory)
EPI_ISL_15078253	A/red fox/Michigan/22-018712-001-original/2022	A / H5N1	North America / United States / Michigan / Muskegon County	Mary Lea Killian (National Veterinary Services Laboratories - USDA / Diagnostic Virology Laboratory)
EPI_ISL_15078254	A/skunk/Washington/22-019274-001-original/2022	A / H5N1	North America / United States / Washington / Whitman County	Mary Lea Killian (National Veterinary Services Laboratories - USDA / Diagnostic Virology Laboratory)
EPI_ISL_15078255	A/dolphin/Florida/22-025319-002-original/2022	A / H5N1	North America / United States / Florida / Dixie County	Mary Lea Killian (National Veterinary Services Laboratories - USDA / Diagnostic Virology Laboratory)
EPI_ISL_15078261	A/chicken/Pennsylvania/22-012092-010-original/2022	A / H5N1	North America / United States / Pennsylvania / Lancaster County	Mary Lea Killian (National Veterinary Services Laboratories - USDA / Diagnostic Virology Laboratory)
EPI_ISL_15081424	A/wigeon/Sakhalin/37M/2021	A / H5N1	Europe / Russian Federation	Ivan Sobolev (Research Institute of Experimental and Clinical Medicine)
EPI_ISL_15088295	A/Common Buzzard/Netherlands/1/2022	A / H5N1	Europe / Netherlands / Hippolytushoef, Mulders Eendenkooi	Sanne Thewessen (Erasmus Medical Center / Viroscience)
EPI_ISL_15088296	A/European Herring Gull/Netherlands/13/2022	A / H5N1	Europe / Netherlands / Den Oever, Waddenhaven	Sanne Thewessen (Erasmus Medical Center / Viroscience)
EPI_ISL_15088297	A/European Herring Gull/Netherlands/14/2022	A / H5N1	Europe / Netherlands / Hippolytushoef, normerven	Sanne Thewessen (Erasmus Medical Center / Viroscience)
EPI_ISL_15088298	A/Northern Gannet/Netherlands/1/2022	A / H5N1	Europe / Netherlands / Hippolytushoef, normerven	Sanne Thewessen (Erasmus Medical Center / Viroscience)
EPI_ISL_15088299	A/Greylag Goose/Netherlands/10/2022	A / H5N1	Europe / Netherlands / Wieringerwerf, Noorderdijkweg	Sanne Thewessen (Erasmus Medical Center / Viroscience)
EPI_ISL_15088300	A/Greylag Goose/Netherlands/11/2022	A / H5N1	Europe / Netherlands / Wieringerwerf, Noorderdijkweg	Sanne Thewessen (Erasmus Medical Center / Viroscience)
EPI_ISL_15088301	A/Greylag Goose/Netherlands/12/2022	A / H5N1	Europe / Netherlands / Wieringerwerf, Noorderdijkweg	Sanne Thewessen (Erasmus Medical Center / Viroscience)
EPI_ISL_15088302	A/Greylag Goose/Netherlands/13/2022	A / H5N1	Europe / Netherlands / Noord Buren, Waddenkust	Sanne Thewessen (Erasmus Medical Center / Viroscience)
EPI_ISL_15088303	A/Lesser Black-backed Gull/Netherlands/3/2022	A / H5N1	Europe / Netherlands / IJmuiden, Forteiland	Sanne Thewessen (Erasmus Medical Center / Viroscience)
EPI_ISL_15088304	A/European Herring Gull/Netherlands/15/2022	A / H5N1	Europe / Netherlands / IJmuiden, Forteiland	Sanne Thewessen (Erasmus Medical Center / Viroscience)
EPI_ISL_15088305	A/Common Teal/Netherlands/1/2022	A / H5N1	Europe / Netherlands / Wieringerwerf, Noorderdijkweg	Sanne Thewessen (Erasmus Medical Center / Viroscience)
EPI_ISL_15088306	A/Mallard/Netherlands/3/2022	A / H5N1	Europe / Netherlands / Hippolytushoef, Mulders Eendenkooi	Sanne Thewessen (Erasmus Medical Center / Viroscience)
EPI_ISL_15088307	A/Mallard/Netherlands/4/2022	A / H5N1	Europe / Netherlands / Hippolytushoef, Mulders Eendenkooi	Sanne Thewessen (Erasmus Medical Center / Viroscience)
EPI_ISL_15088308	A/Mallard/Netherlands/5/2022	A / H5N1	Europe / Netherlands / Hippolytushoef, Mulders Eendenkooi	Sanne Thewessen (Erasmus Medical Center / Viroscience)
EPI_ISL_15088309	A/Mallard/Netherlands/6/2022	A / H5N1	Europe / Netherlands / Hippolytushoef, Mulders Eendenkooi	Sanne Thewessen (Erasmus Medical Center / Viroscience)
EPI_ISL_15115556	A/turkey/England/112259/2022	A / H5N1	Europe / United Kingdom / East Riding of Yorkshire	Alex Byrne (Animal and Plant Health Agency (APHA) / Virology Department)
EPI_ISL_152085	A/duck/Guangdong/wy11/2008	A / H5N5	Asia / China	
EPI_ISL_152086	A/duck/Guangdong/wy19/2008	A / H5N5	Asia / China	
EPI_ISL_152087	A/duck/Guangdong/wy24/2008	A / H5N5	Asia / China	
EPI_ISL_15214832	A/bald eagle/Florida/W22-153B/2022	A / H5N1	North America / United States	
EPI_ISL_15214835	A/common tern/Maine/W22-480A/2022	A / H5N1	North America / United States	
EPI_ISL_15214837	A/snow goose/Kansas/W22-260/2022	A / H5N1	North America / United States	
EPI_ISL_15214838	A/common eiders/Maine/W22-481B/2022	A / H5N1	North America / United States	
EPI_ISL_15214839	A/bald eagle/Florida/W22-153A/2022	A / H5N1	North America / United States	
EPI_ISL_15214841	A/common tern/Maine/W22-480B/2022	A / H5N1	North America / United States	
EPI_ISL_15214843	A/Ross's goose/North Dakota/N22-08/2022	A / H5N1	North America / United States	
EPI_ISL_15214845	A/lesser snow goose/North Dakota/ND-10/2022	A / H5N1	North America / United States	
EPI_ISL_15214846	A/snow goose/Kansas/W22-174B/2022	A / H5N1	North America / United States	
EPI_ISL_15215193	A/common eiders/Maine/W22-481A/2022	A / H5N1	North America / United States	
EPI_ISL_15215277	A/bald eagle/North Carolina/W22-140/2022	A / H5N1	North America / United States	
EPI_ISL_15215278	A/hooded merganser/Florida/W22-154/2022	A / H5N1	North America / United States	
EPI_ISL_15215280	A/bald eagle/Kansas/W22-185/2022	A / H5N1	North America / United States	
EPI_ISL_15234355	A/red-backed-hawk/Spain/2313-1_22VIR8632-10/2022	A / H5N1	Europe / Spain	Giacomo Barbierato (Istituto Zooprofilattico Sperimentale delle Venezie / Ricerca e innovazione)
EPI_ISL_15234356	A/Anser_anser/Spain/2636-3_22VIR8632-11/2022	A / H5N1	Europe / Spain	Giacomo Barbierato (Istituto Zooprofilattico Sperimentale delle Venezie / Ricerca e innovazione)
EPI_ISL_15234357	A/Anser_anser/Spain/2753-3_22VIR8632-12/2022	A / H5N1	Europe / Spain	Giacomo Barbierato (Istituto Zooprofilattico Sperimentale delle Venezie / Ricerca e innovazione)
EPI_ISL_15234358	A/Anser_anser/Spain/2825-1_22VIR8632-13/2022	A / H5N1	Europe / Spain	Giacomo Barbierato (Istituto Zooprofilattico Sperimentale delle Venezie / Ricerca e innovazione)
EPI_ISL_15234359	A/Anser_anser/Spain/810-6_22VIR8632-16/2022	A / H5N1	Europe / Spain	Giacomo Barbierato (Istituto Zooprofilattico Sperimentale delle Venezie / Ricerca e innovazione)
EPI_ISL_15234360	A/turkey/Spain/2755-5_22VIR8632-1/2022	A / H5N1	Europe / Spain	Giacomo Barbierato (Istituto Zooprofilattico Sperimentale delle Venezie / Ricerca e innovazione)
EPI_ISL_15234361	A/turkey/Spain/2996-43_22VIR8632-21/2022	A / H5N1	Europe / Spain	Giacomo Barbierato (Istituto Zooprofilattico Sperimentale delle Venezie / Ricerca e innovazione)
EPI_ISL_15234362	A/turkey/Spain/2996-42_22VIR8632-22/2022	A / H5N1	Europe / Spain	Giacomo Barbierato (Istituto Zooprofilattico Sperimentale delle Venezie / Ricerca e innovazione)
EPI_ISL_15234363	A/turkey/Spain/2755-6_22VIR8632-2/2022	A / H5N1	Europe / Spain	Giacomo Barbierato (Istituto Zooprofilattico Sperimentale delle Venezie / Ricerca e innovazione)
EPI_ISL_15234364	A/chicken/Spain/2854-5_22VIR8632-3/2022	A / H5N1	Europe / Spain	Giacomo Barbierato (Istituto Zooprofilattico Sperimentale delle Venezie / Ricerca e innovazione)
EPI_ISL_15234365	A/chicken/Spain/2854-7_22VIR8632-4/2022	A / H5N1	Europe / Spain	Giacomo Barbierato (Istituto Zooprofilattico Sperimentale delle Venezie / Ricerca e innovazione)
EPI_ISL_15234366	A/duck/Spain/2095-2_22VIR8632-5/2022	A / H5N1	Europe / Spain	Giacomo Barbierato (Istituto Zooprofilattico Sperimentale delle Venezie / Ricerca e innovazione)
EPI_ISL_15234656	A/bearded_vulture/Spain/2116-3_22VIR8632-8/2022	A / H5N1	Europe / Spain	Giacomo Barbierato (Istituto Zooprofilattico Sperimentale delle Venezie / Ricerca e innovazione)
EPI_ISL_15267012	A/Lesser Black-backed Gull/Netherlands/4/2022	A / H5N1	Europe / Netherlands / Den Oever, Waddenhaven	Sanne Thewessen (Erasmus Medical Center / Viroscience)
EPI_ISL_15267013	A/Eurasian Spoonbill/Netherlands/5/2022	A / H5N1	Europe / Netherlands / Texel, de Schorren	Sanne Thewessen (Erasmus Medical Center / Viroscience)
EPI_ISL_15267014	A/Eurasian Spoonbill/Netherlands/6/2022	A / H5N1	Europe / Netherlands / Texel, de Schorren	Sanne Thewessen (Erasmus Medical Center / Viroscience)
EPI_ISL_15267016	A/Eurasian Spoonbill/Netherlands/7/2022	A / H5N1	Europe / Netherlands / Texel, de Schorren	Sanne Thewessen (Erasmus Medical Center / Viroscience)
EPI_ISL_15267017	A/Eurasian Spoonbill/Netherlands/8/2022	A / H5N1	Europe / Netherlands / Wieringerwerf, Noorderdijkweg	Sanne Thewessen (Erasmus Medical Center / Viroscience)
EPI_ISL_15267018	A/Greylag Goose/Netherlands/14/2022	A / H5N1	Europe / Netherlands / Wieringerwerf, Noorderdijkweg	Sanne Thewessen (Erasmus Medical Center / Viroscience)
EPI_ISL_15267019	A/Greylag Goose/Netherlands/15/2022	A / H5N1	Europe / Netherlands / Enkhuizen, Ijsselmeer, De Kreupel	Sanne Thewessen (Erasmus Medical Center / Viroscience)
EPI_ISL_15267020	A/Greylag Goose/Netherlands/16/2022	A / H5N1	Europe / Netherlands / Enkhuizen, Ijsselmeer, De Kreupel	Sanne Thewessen (Erasmus Medical Center / Viroscience)
EPI_ISL_15267021	A/Common Tern/Netherlands/27/2022	A / H5N1	Europe / Netherlands / Enkhuizen, Ijsselmeer, De Kreupel	Sanne Thewessen (Erasmus Medical Center / Viroscience)
EPI_ISL_15267022	A/European Herring Gull/Netherlands/16/2022	A / H5N1	Europe / Netherlands / Hippolytushoef, Mulders Eendenkooi	Sanne Thewessen (Erasmus Medical Center / Viroscience)
EPI_ISL_15267023	A/Northern Gannet/Netherlands/2/2022	A / H5N1	Europe / Netherlands / Vlieland, Noordzee strand	Sanne Thewessen (Erasmus Medical Center / Viroscience)
EPI_ISL_15267024	A/Northern Gannet/Netherlands/3/2022	A / H5N1	Europe / Netherlands / Vlieland, Noordzee strand	Sanne Thewessen (Erasmus Medical Center / Viroscience)
EPI_ISL_15267025	A/Northern Gannet/Netherlands/4/2022	A / H5N1	Europe / Netherlands / Vlieland, Noordzee strand	Sanne Thewessen (Erasmus Medical Center / Viroscience)
EPI_ISL_15267026	A/Northern Gannet/Netherlands/5/2022	A / H5N1	Europe / Netherlands / Vlieland, Noordzee strand	Sanne Thewessen (Erasmus Medical Center / Viroscience)

EPI_ISL_15267027	A/Northern Gannet/Netherlands/6/2022	A / H5N1	Europe / Netherlands / Vlieland, Noordzee strand	Sanne Thewessen (Erasmus Medical Center / Viroscience)
EPI_ISL_15350905	A/chicken/Ghana/AVL-763_21VIR7050-39/2021	A / H5N1	Africa / Ghana	Giacomo Barbierato (Istituto Zooprofilattico Sperimentale delle Venezie / Ricerca e innovazione)
EPI_ISL_153620	A/chicken/China/AH/2012	A / H5N1	Asia / China	
EPI_ISL_15364788	A/European Herring Gull/Netherlands/17/2022	A / H5N1	Europe / Netherlands / Vlieland, Noordzee strand	Sanne Thewessen (Erasmus Medical Center / Viroscience)
EPI_ISL_15364789	A/European Herring Gull/Netherlands/18/2022	A / H5N1	Europe / Netherlands / Vlieland, eerste Kroonspolder	Sanne Thewessen (Erasmus Medical Center / Viroscience)
EPI_ISL_15364790	A/Greylag Goose/Netherlands/17/2022	A / H5N1	Europe / Netherlands / Hippolytushoef, Normerven	Sanne Thewessen (Erasmus Medical Center / Viroscience)
EPI_ISL_15364792	A/Mallard/Netherlands/11/2022	A / H5N1	Europe / Netherlands / Hippolytushoef, Mulders Eendenkooi	Sanne Thewessen (Erasmus Medical Center / Viroscience)
EPI_ISL_15364793	A/Mallard/Netherlands/12/2022	A / H5N1	Europe / Netherlands / Lithse eendenkooi	Sanne Thewessen (Erasmus Medical Center / Viroscience)
EPI_ISL_15364794	A/Mallard/Netherlands/13/2022	A / H5N1	Europe / Netherlands / Lithse eendenkooi	Sanne Thewessen (Erasmus Medical Center / Viroscience)
EPI_ISL_15364795	A/Mallard/Netherlands/8/2022	A / H5N1	Europe / Netherlands / Hippolytushoef, Mulders Eendenkooi	Sanne Thewessen (Erasmus Medical Center / Viroscience)
EPI_ISL_15364796	A/Mallard/Netherlands/9/2022	A / H5N1	Europe / Netherlands / Hippolytushoef, Mulders Eendenkooi	Sanne Thewessen (Erasmus Medical Center / Viroscience)
EPI_ISL_15364797	A/Mute Swan/Netherlands/2/2022	A / H5N1	Europe / Netherlands / Kollum	Sanne Thewessen (Erasmus Medical Center / Viroscience)
EPI_ISL_15430045	A/herring_gull/Poland/MB82B/2016	A / H5N8	Europe / Poland / West Pomeranian Voivodeship	Edyta ?wi?to? (National Veterinary Research Institut Poland, PIWet-PIB)
EPI_ISL_15430209	A/common crane/Yunnan-Huize/11/2021(H5N8)	A / H5N8	Asia / China / Yunnan Province / Huize Black-necked Crane National Nature Reserve, Yunnan Pro	Jinglin Wang (Yunnan Animal Science and Veterinary Institute / Yunnan Tropical and Subtropical Animal Viruses Disease Laborator)
EPI_ISL_15433349	A/common crane /Yunnan-Huize/22/2021(H5N8)	A / H5N8	Asia / China / Yunnan Province / Huize Black-necked Crane National Nature Reserve, Yunnan Pro	Jinglin Wang (Yunnan Animal Science and Veterinary Institute / Yunnan Tropical and Subtropical Animal Viruses Disease Laborator)
EPI_ISL_15435500	A/common crane/Yunnan-Huize/24/2021(H5N8)	A / H5N8	Asia / China / Yunnan Province / Huize Black-necked Crane National Nature Reserve, Yunnan Pro	Jinglin Wang (Yunnan Animal Science and Veterinary Institute / Yunnan Tropical and Subtropical Animal Viruses Disease Laborator)
EPI_ISL_15435511	A/common crane/Yunnan-Huize/27/2021(H5N8)	A / H5N8	Asia / China / Yunnan Province / Huize Black-necked Crane National Nature Reserve, Yunnan Pro	Jinglin Wang (Yunnan Animal Science and Veterinary Institute / Yunnan Tropical and Subtropical Animal Viruses Disease Laborator)
EPI_ISL_15535272	A/domestic_goose/Poland/H397-N/2022	A / H5N1	Europe / Poland / Lodz Voivodeship	Edyta ?wi?to? (National Veterinary Research Institut Poland, PIWet-PIB)
EPI_ISL_15542438	A/CastillaLaMancha/223739/2022	A / H5N1	Europe / Spain / Castille-La Mancha	Francisco Pozo (Instituto de Salud Carlos III)
EPI_ISL_15576616	A/white-fronted goose/Miyagi/0410D001/2022	A / H5N1	Asia / Japan / Miyagi / Kurihara city	Norikazu Isoda (Graduate School of Veterinary Medicine, Hokkaido University / Laboratory of Microbiology)
EPI_ISL_15576617	A/Eurasian wigeon/Hokkaido/Q71/2022	A / H5N1	Asia / Japan / Hokkaido / Noduke Peninsula, Bekkai town	Norikazu Isoda (Graduate School of Veterinary Medicine, Hokkaido University / Laboratory of Microbiology)
EPI_ISL_15579535	A/European Herring Gull/Netherlands/19/2022	A / H5N1	Europe / Netherlands / Hippolytushoef, Normerven	Sanne Thewessen (Erasmus Medical Center / Viroscience)
EPI_ISL_15579536	A/Greylag Goose/Netherlands/18/2022	A / H5N1	Europe / Netherlands / Stroe, Waddenkust	Sanne Thewessen (Erasmus Medical Center / Viroscience)
EPI_ISL_15579537	A/Barnacle Goose/Netherlands/19/2022	A / H5N1	Europe / Netherlands / Enkhuizen, Ijsselmeer, De Kreupel	Sanne Thewessen (Erasmus Medical Center / Viroscience)
EPI_ISL_15579538	A/Eurasian Curlew/Netherlands/4/2022	A / H5N1	Europe / Netherlands / Hippolytushoef, Normerven	Sanne Thewessen (Erasmus Medical Center / Viroscience)
EPI_ISL_15579539	A/European Herring Gull/Netherlands/20/2022	A / H5N1	Europe / Netherlands / Noord Buren, Waddenkust	Sanne Thewessen (Erasmus Medical Center / Viroscience)
EPI_ISL_15579540	A/Mallard/Netherlands/15/2022	A / H5N1	Europe / Netherlands / Eendenkooi Wieringen	Sanne Thewessen (Erasmus Medical Center / Viroscience)
EPI_ISL_15579543	A/Mallard/Netherlands/18/2022	A / H5N1	Europe / Netherlands / Eendenkooi Wieringen	Sanne Thewessen (Erasmus Medical Center / Viroscience)
EPI_ISL_15579544	A/Great Black-backed Gull/4/2022	A / H5N1	Europe / Netherlands / Kwade hoek, Stellendam	Sanne Thewessen (Erasmus Medical Center / Viroscience)
EPI_ISL_15579545	A/Sanderling/Netherlands/2/2022	A / H5N1	Europe / Netherlands / Kwade hoek, Stellendam	Sanne Thewessen (Erasmus Medical Center / Viroscience)
EPI_ISL_15581789	A/Mallard/Netherlands/19/2022	A / H5N1	Europe / Netherlands / Eendenkooi Wieringen	Sanne Thewessen (Erasmus Medical Center / Viroscience)
EPI_ISL_15581790	A/Mallard/Netherlands/20/2022	A / H5N1	Europe / Netherlands / Oud-Albas	Sanne Thewessen (Erasmus Medical Center / Viroscience)
EPI_ISL_15581791	A/Mallard/Netherlands/21/2022	A / H5N1	Europe / Netherlands / Oud-Albas	Sanne Thewessen (Erasmus Medical Center / Viroscience)
EPI_ISL_15581792	A/Mallard/Netherlands/22/2022	A / H5N1	Europe / Netherlands / Oud-Albas	Sanne Thewessen (Erasmus Medical Center / Viroscience)
EPI_ISL_15585858	A/chicken/England/119017/2022	A / H5N1	Europe / United Kingdom / England	Alex Byrne (Animal and Plant Health Agency (APHA) / Virology Department)
EPI_ISL_15585864	A/chicken/England/123975/2022	A / H5N1	Europe / United Kingdom / England	Alex Byrne (Animal and Plant Health Agency (APHA) / Virology Department)
EPI_ISL_15585890	A/chicken/Wales/116603/2022	A / H5N1	Europe / United Kingdom / Wales	Alex Byrne (Animal and Plant Health Agency (APHA) / Virology Department)
EPI_ISL_15586122	A/domestic_duck/England/117887/2022	A / H5N1	Europe / United Kingdom / England	Alex Byrne (Animal and Plant Health Agency (APHA) / Virology Department)
EPI_ISL_15586133	A/mute_swan/England/117298/2022	A / H5N1	Europe / United Kingdom / England	Alex Byrne (Animal and Plant Health Agency (APHA) / Virology Department)
EPI_ISL_15602898	A/turkey/Montana/22-028879-001-original/2022	A / H5N4	North America / United States / Montana / Teton County	Mary Lea Killian (National Veterinary Services Laboratories - USDA / Diagnostic Virology Laboratory)
EPI_ISL_15613488	A/mallard/Miyazaki/4501C607-c1/2021	A / H5N8	Asia / Japan / Miyazaki	
EPI_ISL_15613489	A/mallard/Miyazaki/4501C607-c3/2021	A / H5N8	Asia / Japan / Miyazaki	
EPI_ISL_15613490	A/mallard/Miyazaki/4501C607-c5/2021	A / H5N8	Asia / Japan / Miyazaki	
EPI_ISL_15613491	A/mallard/Miyazaki/4501C607-c6/2021	A / H5N8	Asia / Japan / Miyazaki	
EPI_ISL_15613492	A/mallard/Miyazaki/4501C607-c7/2021	A / H5N8	Asia / Japan / Miyazaki	
EPI_ISL_15613493	A/mallard/Miyazaki/4501C607-c8/2021	A / H5N8	Asia / Japan / Miyazaki	
EPI_ISL_15613494	A/teal/Miyazaki/211109-32/2021	A / H5N1	Asia / Japan / Miyazaki	
EPI_ISL_15613502	A/whooper swan/Iwate/0302I010/2022	A / H5	Asia / Japan / Iwate	
EPI_ISL_15613505	A/white-tailed eagle/Hokkaido/20220322001/2022	A / H5	Asia / Japan / Hokkaido	
EPI_ISL_15613506	A/white-tailed eagle/Hokkaido/20220324001/2022	A / H5	Asia / Japan / Hokkaido	
EPI_ISL_15613515	A/white-tailed eagle/Hokkaido/20220426001/2022	A / H5	Asia / Japan / Hokkaido	
EPI_ISL_15613516	A/black kite/Hokkaido/0104P081/2022	A / H5	Asia / Japan / Hokkaido	
EPI_ISL_15613574	A/Guangdong/1/2021	A / H5N6	Asia / China / Guangdong Province	
EPI_ISL_15614803	A/ibis/Egypt/RLQP-229S/2022	A / H5N1	Africa / Egypt	
EPI_ISL_15614805	A/Lesser scaup/MD/-LC-EESC-024/2022	A / H5N1	North America / United States	
EPI_ISL_15614807	A/red-tailed hawk/Kansas/W22-198/2022	A / H5N1	North America / United States	
EPI_ISL_15647834	A/duck/Korea/H493/2022	A / H5N1	Asia / Korea, Republic of	Se-hee An (Animal and Plant Quarantine Agency (APQA) / Avian Influenza Research and Diagsnotic Division)
EPI_ISL_15647835	A/common teal/Korea/WA537/2022	A / H5N1	Asia / Korea, Republic of	Se-hee An (Animal and Plant Quarantine Agency (APQA) / Avian Influenza Research and Diagsnotic Division)
EPI_ISL_15647836	A/Mandarin duck/Korea/WA496	A / H5N1	Asia / Korea, Republic of	Se-hee An (Animal and Plant Quarantine Agency (APQA) / Avian Influenza Research and Diagsnotic Division)
EPI_ISL_15647837	A/duck/Korea/H125/2022	A / H5N1	Asia / Korea, Republic of	Se-hee An (Animal and Plant Quarantine Agency (APQA) / Avian Influenza Research and Diagsnotic Division)
EPI_ISL_15676637	A/chicken/Croatia/55/2022	A / H5N1	Europe / Croatia / Osjecko-Baranjska Zupanija / Beli Manstir	Vladimir Savi? (Croatian Veterinary Institute / Poultry Centre)
EPI_ISL_15676638	A/mute swan/Croatia/108/2022	A / H5N1	Europe / Croatia / Zagrebacka Zupanija / Strmec Samoborski, Kipiš?e lake	Vladimir Savi? (Croatian Veterinary Institute / Poultry Centre)
EPI_ISL_156815	A/mallard duck/Shanghai/SH-9/2013	A / H5N8	Asia / China / Shanghai Municipality	Fan Sheng Tao (Institute of Laboratory Animal Sciences, Chinese Academy)
EPI_ISL_15697406	A/Pavo/Belgium/10906_0006/2022	A / H5N1	Europe / Belgium / Provincie Limburg / Bocholt	Steven Van Borm (Sciensano, Department of Animal Infectious Diseases / Animal Infectious Diseases)
EPI_ISL_15697407	A/Gallus_gallus/Belgium/10968_0005/2022	A / H5N1	Europe / Belgium / Provincie West-Vlaanderen / Sint Laureins	Steven Van Borm (Sciensano, Department of Animal Infectious Diseases / Animal Infectious Diseases)
EPI_ISL_15698209	A/Gallus_gallus/Belgium/11248_0001/2022	A / H5N1	Europe / Belgium / Provincie West-Vlaanderen / Sint Laureins	Steven Van Borm (Sciensano, Department of Animal Infectious Diseases / Animal Infectious Diseases)
EPI_ISL_15698370	A/Ardea_cinerea/Belgium/11304_0003/2022	A / H5N1	Europe / Belgium / Provincie Oost-Vlaanderen / Zelzate	Steven Van Borm (Sciensano, Department of Animal Infectious Diseases / Animal Infectious Diseases)
EPI_ISL_15699041	A/Cygnus_olor/Belgium/11304_0009/2022	A / H5N1	Europe / Belgium / Provincie West-Vlaanderen / Brugge	Steven Van Borm (Sciensano, Department of Animal Infectious Diseases / Animal Infectious Diseases)
EPI_ISL_15699790	A/Gallus_gallus/Belgium/11387_0002/2022	A / H5N1	Europe / Belgium / Provincie Oost-Vlaanderen / Sint Laureins	Steven Van Borm (Sciensano, Department of Animal Infectious Diseases / Animal Infectious Diseases)
EPI_ISL_15699799	A/Gallus_gallus/Belgium/11445_0002/2022	A / H5N1	Europe / Belgium / Provincie West-Vlaanderen / Wingene	Steven Van Borm (Sciensano, Department of Animal Infectious Diseases / Animal Infectious Diseases)
EPI_ISL_15699929	A/Gallus_gallus/Belgium/11943_0003/2022	A / H5N1	Europe / Belgium / Provincie Limburg / Tongeren	Steven Van Borm (Sciensano, Department of Animal Infectious Diseases / Animal Infectious Diseases)
EPI_ISL_15732741	A/large-billed crow/Hokkaido/B003/2022	A / H5N2	Asia / Japan / Hokkaido / Sapporo	Norikazu Isoda (Graduate School of Veterinary Medicine, Hokkaido University / Laboratory of Microbiology)

EPI_ISL_15732766	A/Eurasian wigeon/Hokkaido/M184/2022	A / H5N1	Asia / Japan / Hokkaido / the Komuke Lake	Norikazu Isoda (Graduate School of Veterinary Medicine, Hokkaido University / Laboratory of Microbiology)
EPI_ISL_1575129	A/Common Buzzard/Netherlands/4/2020	A / H5N8	Europe / Netherlands / Rotterdam	Theo Bestebroer (Erasmus Medical Center / Department of Virology)
EPI_ISL_1575130	A/Mute Swan/Netherlands/1/2020	A / H5N8	Europe / Netherlands / Rotterdam	Theo Bestebroer (Erasmus Medical Center / Department of Virology)
EPI_ISL_1575131	A/Herring Gull/Netherlands/1/2020	A / H5N8	Europe / Netherlands / Rotterdam	Theo Bestebroer (Erasmus Medical Center / Department of Virology)
EPI_ISL_1575132	A/Herring Gull/Netherlands/2/2020	A / H5N8	Europe / Netherlands / Rotterdam	Theo Bestebroer (Erasmus Medical Center / Department of Virology)
EPI_ISL_1575133	A/Herring Gull/Netherlands/3/2020	A / H5N8	Europe / Netherlands / Rotterdam	Theo Bestebroer (Erasmus Medical Center / Department of Virology)
EPI_ISL_1575134	A/Mute Swan/Netherlands/3/2020	A / H5N8	Europe / Netherlands / Rotterdam	Theo Bestebroer (Erasmus Medical Center / Department of Virology)
EPI_ISL_1575135	A/Greylag Goose/Netherlands/1/2020	A / H5N8	Europe / Netherlands / Rotterdam	Theo Bestebroer (Erasmus Medical Center / Department of Virology)
EPI_ISL_1575136	A/Mute Swan/Netherlands/5/2020	A / H5N8	Europe / Netherlands / Rotterdam	Theo Bestebroer (Erasmus Medical Center / Department of Virology)
EPI_ISL_15759949	A/Common Teal/Netherlands/3/2022	A / H5N1	Europe / Netherlands / Eendenkooi, de Sompen	Sanne Thewessen (Erasmus Medical Center / Viroscience)
EPI_ISL_15759950	A/Common Buzzard/2/2022	A / H5N1	Europe / Netherlands / Woerden	Sanne Thewessen (Erasmus Medical Center / Viroscience)
EPI_ISL_15759951	A/Mallard/Netherlands/24/2022	A / H5N1	Europe / Netherlands / Oud-Albas	Sanne Thewessen (Erasmus Medical Center / Viroscience)
EPI_ISL_15759952	A/Mallard/Netherlands/25/2022	A / H5N1	Europe / Netherlands / Oud-Albas	Sanne Thewessen (Erasmus Medical Center / Viroscience)
EPI_ISL_15759953	A/Mallard/Netherlands/26/2022	A / H5N1	Europe / Netherlands / Oud-Alblas	Sanne Thewessen (Erasmus Medical Center / Viroscience)
EPI_ISL_157609	A/breeder duck/Korea/Gochang1/2014	A / H5N8	Asia / Korea, Republic of	
EPI_ISL_157610	A/broiler duck/Korea/Buan2/2014	A / H5N8	Asia / Korea, Republic of	
EPI_ISL_157611	A/baikal teal/Korea/Donglim3/2014	A / H5N8	Asia / Korea, Republic of	
EPI_ISL_15812579	A/Anser_anser_domesticus/Belgium/10918_0002/2022	A / H5N1	Europe / Belgium / Provincie West-Vlaanderen / Beernem	Steven Van Borm (Sciensano, Department of Animal Infectious Diseases / Animal Infectious Diseases)
EPI_ISL_15812678	A/Anas_platyrhynchos_domesticus/Belgium/11599_0005/202	A / H5N1	Europe / Belgium / Provincie West-Vlaanderen / Diksmuide	Steven Van Borm (Sciensano, Department of Animal Infectious Diseases / Animal Infectious Diseases)
EPI_ISL_15813174	A/Gallus_gallus/Belgium/12311_0001/2022	A / H5N1	Europe / Belgium / Province de Liege / Aywaille	Steven Van Borm (Sciensano, Department of Animal Infectious Diseases / Animal Infectious Diseases)
EPI_ISL_15814043	A/Gallus_gallus/Belgium/12395_0002/2022	A / H5N1	Europe / Belgium / Provincie Antwerpen / Hoogstraten	Steven Van Borm (Sciensano, Department of Animal Infectious Diseases / Animal Infectious Diseases)
EPI_ISL_15843373	A/Bald Eagle/BC/OTH-33-36/2022 (H5N1)	A / H5N1	North America / Canada / British Columbia	Yohannes Berhane (Canadian Food Inspection Agency / Avian Unit)
EPI_ISL_15844573	A/Broiler Chicken/BC/FAV-0228/2022 (H5N1)	A / H5N1	North America / Canada / British Columbia	Yohannes Berhane (Canadian Food Inspection Agency / Avian Unit)
EPI_ISL_15857836	A/teal/Italy/22VIR10396-33/2022	A / H5N1	Europe / Italy	Giacomo Barbierato (Istituto Zooprofilattico Sperimentale delle Venezie / Ricerca e innovazione)
EPI_ISL_15857837	A/teal/Italy/22VIR10396-5/2022	A / H5N1	Europe / Italy	Giacomo Barbierato (Istituto Zooprofilattico Sperimentale delle Venezie / Ricerca e innovazione)
EPI_ISL_15857838	A/teal/Italy/22VIR10576-13/2022	A / H5N1	Europe / Italy	Giacomo Barbierato (Istituto Zooprofilattico Sperimentale delle Venezie / Ricerca e innovazione)
EPI_ISL_15857839	A/chicken/Italy/22VIR11246-6/2022	A / H5N1	Europe / Italy	Giacomo Barbierato (Istituto Zooprofilattico Sperimentale delle Venezie / Ricerca e innovazione)
EPI_ISL_15857840	A/turkey/Italy/22VIR11262-1/2022	A / H5N1	Europe / Italy	Giacomo Barbierato (Istituto Zooprofilattico Sperimentale delle Venezie / Ricerca e innovazione)
EPI_ISL_15857841	A/swan/Italy/22VIR10875-2/2022	A / H5N1	Europe / Italy	Giacomo Barbierato (Istituto Zooprofilattico Sperimentale delle Venezie / Ricerca e innovazione)
EPI_ISL_15857842	A/swan/Italy/22VIR10995-7/2022	A / H5N1	Europe / Italy	Giacomo Barbierato (Istituto Zooprofilattico Sperimentale delle Venezie / Ricerca e innovazione)
EPI_ISL_15857843	A/rural_laying_hen/Italy/22VIR10996-6/2022	A / H5N1	Europe / Italy	Giacomo Barbierato (Istituto Zooprofilattico Sperimentale delle Venezie / Ricerca e innovazione)
EPI_ISL_15857844	A/turkey/Italy/22VIR11056-4/2022	A / H5N1	Europe / Italy	Giacomo Barbierato (Istituto Zooprofilattico Sperimentale delle Venezie / Ricerca e innovazione)
EPI_ISL_15857845	A/turkey/Italy/22VIR11144-1/2022	A / H5N1	Europe / Italy	Giacomo Barbierato (Istituto Zooprofilattico Sperimentale delle Venezie / Ricerca e innovazione)
EPI_ISL_15857846	A/turkey/Italy/22VIR10627-4/2022	A / H5N1	Europe / Italy	Giacomo Barbierato (Istituto Zooprofilattico Sperimentale delle Venezie / Ricerca e innovazione)
EPI_ISL_15857847	A/rural_chicken/Italy/22VIR10651-4/2022	A / H5N1	Europe / Italy	Giacomo Barbierato (Istituto Zooprofilattico Sperimentale delle Venezie / Ricerca e innovazione)
EPI_ISL_15857848	A/turkey/Italy/22VIR10733-4/2022	A / H5N1	Europe / Italy	Giacomo Barbierato (Istituto Zooprofilattico Sperimentale delle Venezie / Ricerca e innovazione)
EPI_ISL_15857849	A/swan/Italy/22VIR10737-10/2022	A / H5N1	Europe / Italy	Giacomo Barbierato (Istituto Zooprofilattico Sperimentale delle Venezie / Ricerca e innovazione)
EPI_ISL_15857864	A/rural_goose/Italy/22VIR10789-2/2022	A / H5N1	Europe / Italy	Giacomo Barbierato (Istituto Zooprofilattico Sperimentale delle Venezie / Ricerca e innovazione)
EPI_ISL_15857953	A/laying_hen/Italy/22VIR10800-2/2022	A / H5N1	Europe / Italy	Giacomo Barbierato (Istituto Zooprofilattico Sperimentale delle Venezie / Ricerca e innovazione)
EPI_ISL_15857954	A/laying_hen/Italy/22VIR10267-7/2022	A / H5N1	Europe / Italy	Giacomo Barbierato (Istituto Zooprofilattico Sperimentale delle Venezie / Ricerca e innovazione)
EPI_ISL_15857955	A/rural_duck/Italy/22VIR10534-3/2022	A / H5N1	Europe / Italy	Giacomo Barbierato (Istituto Zooprofilattico Sperimentale delle Venezie / Ricerca e innovazione)
EPI_ISL_15857956	A/teal/Italy/22VIR9917-19/2022	A / H5N1	Europe / Italy	Giacomo Barbierato (Istituto Zooprofilattico Sperimentale delle Venezie / Ricerca e innovazione)
EPI_ISL_15857957	A/teal/Italy/22VIR9917-52/2022	A / H5N1	Europe / Italy	Giacomo Barbierato (Istituto Zooprofilattico Sperimentale delle Venezie / Ricerca e innovazione)
EPI_ISL_15857958	A/swan/Italy/22VIR10097-3/2022	A / H5N1	Europe / Italy	Giacomo Barbierato (Istituto Zooprofilattico Sperimentale delle Venezie / Ricerca e innovazione)
EPI_ISL_15857959	A/swan/Italy/22VIR10097-4/2022	A / H5N1	Europe / Italy	Giacomo Barbierato (Istituto Zooprofilattico Sperimentale delle Venezie / Ricerca e innovazione)
EPI_ISL_15857961	A/Goose/Italy/22VIR9673-3/2022	A / H5N1	Europe / Italy	Giacomo Barbierato (Istituto Zooprofilattico Sperimentale delle Venezie / Ricerca e innovazione)
EPI_ISL_15857990	A/Chicken/Italy/22VIR9832-3/2022	A / H5N1	Europe / Italy	Giacomo Barbierato (Istituto Zooprofilattico Sperimentale delle Venezie / Ricerca e innovazione)
EPI_ISL_15857991	A/Hawaiian_goose/Italy/22VIR9126-7/2022	A / H5N1	Europe / Italy	Giacomo Barbierato (Istituto Zooprofilattico Sperimentale delle Venezie / Ricerca e innovazione)
EPI_ISL_15857992	A/chicken/France/22P023203/2022	A / H5N1	Europe / France / Brittany / Departement des Cotes-d'Armor	Francois-Xavier Briand (ANSES Agence Nationale De Securite Sanitaire De L'alimentation / Laboratoire de Ploufragan-Plouzané)
EPI_ISL_15878539	A/Mink/Spain/3691-8_22VIR10586-10/2022	A / H5N1	Europe / Spain	Giacomo Barbierato (Istituto Zooprofilattico Sperimentale delle Venezie / Ricerca e innovazione)
EPI_ISL_15878540	A/Mink/Spain/3691-10_22VIR10586-11/2022	A / H5N1	Europe / Spain	Giacomo Barbierato (Istituto Zooprofilattico Sperimentale delle Venezie / Ricerca e innovazione)
EPI_ISL_15878541	A/Mink/Spain/3691-2_22VIR10586-8/2022	A / H5N1	Europe / Spain	Giacomo Barbierato (Istituto Zooprofilattico Sperimentale delle Venezie / Ricerca e innovazione)
EPI_ISL_15878542	A/Mink/Spain/3691-3_22VIR10586-9/2022	A / H5N1	Europe / Spain	Giacomo Barbierato (Istituto Zooprofilattico Sperimentale delle Venezie / Ricerca e innovazione)
EPI_ISL_15878543	A/laying_hen/Spain/3135-17_22VIR10586-1/2022	A / H5N1	Europe / Spain	Giacomo Barbierato (Istituto Zooprofilattico Sperimentale delle Venezie / Ricerca e innovazione)
EPI_ISL_15878544	A/laying_hen/Spain/3135-41_22VIR10586-2/2022	A / H5N1	Europe / Spain	Giacomo Barbierato (Istituto Zooprofilattico Sperimentale delle Venezie / Ricerca e innovazione)
EPI_ISL_15878545	A/laying_hen/Spain/3232-35_22VIR10586-3/2022	A / H5N1	Europe / Spain	Giacomo Barbierato (Istituto Zooprofilattico Sperimentale delle Venezie / Ricerca e innovazione)
EPI_ISL_15878546	A/laying_hen/Spain/3232-37_22VIR10586-4/2022	A / H5N1	Europe / Spain	Giacomo Barbierato (Istituto Zooprofilattico Sperimentale delle Venezie / Ricerca e innovazione)
EPI_ISL_15878548	A/Northern_gannet/Spain/3372-12_22VIR10586-6/2022	A / H5N1	Europe / Spain	Giacomo Barbierato (Istituto Zooprofilattico Sperimentale delle Venezie / Ricerca e innovazione)
EPI_ISL_15878549	A/White_stork/Spain/3438-3_22VIR10586-7/2022	A / H5N1	Europe / Spain	Giacomo Barbierato (Istituto Zooprofilattico Sperimentale delle Venezie / Ricerca e innovazione)
EPI_ISL_15895378	A/peafowl/ Croatia/123/2022	A / H5N1	Europe / Croatia / Zagrebacka Zupanija / Gornji Stupnik	Vladimir Savi? (Croatian Veterinary Institute / Poultry Centre)
EPI_ISL_15895379	A/mute swan/Croatia/125/2022	A / H5N1	Europe / Croatia / Vukovarsko-Srijemska Zupanija / Vukovar, Vuka river	Vladimir Savi? (Croatian Veterinary Institute / Poultry Centre)
EPI_ISL_15895380	A/mute swan/Croatia/126/2022	A / H5N1	Europe / Croatia / Vukovarsko-Srijemska Zupanija / Vukovar, Vuka river	Vladimir Savi? (Croatian Veterinary Institute / Poultry Centre)
EPI_ISL_15923322	A/peregrine falcon/Kanagawa/1409C001T1/2022	A / H5N1	Asia / Japan / Kanagawa	Takehiko Saito (National Institute of Animal Health)
EPI_ISL_15925875	A/Eurasian Wigeon/Netherlands/1/2022	A / H5N1	Europe / Netherlands / Eendenkooi, Wieringen	Oanh Vuong-Zoet (Erasmus Medical Center / Department of Virology)
EPI_ISL_15925876	A/Eurasian Wigeon/Netherlands/2/2022	A / H5N1	Europe / Netherlands / Eendenkooi, Wieringen	Oanh Vuong-Zoet (Erasmus Medical Center / Department of Virology)
EPI_ISL_15925878	A/Common Teal/Netherlands/5/2022	A / H5N1	Europe / Netherlands / Eendenkooi, Wieringen	Oanh Vuong-Zoet (Erasmus Medical Center / Department of Virology)
EPI_ISL_15925879	A/Common Teal/Netherlands/6/2022	A / H5N1	Europe / Netherlands / Eendenkooi, Wieringen	Oanh Vuong-Zoet (Erasmus Medical Center / Department of Virology)
EPI_ISL_15925880	A/Gadwall/Netherlands/1/2022	A / H5N1	Europe / Netherlands / Eendenkooi, Wieringen	Oanh Vuong-Zoet (Erasmus Medical Center / Department of Virology)
EPI_ISL_15925881	A/Gadwall/Netherlands/2/2022	A / H5N1	Europe / Netherlands / Eendenkooi, Wieringen	Oanh Vuong-Zoet (Erasmus Medical Center / Department of Virology)
EPI_ISL_15925882	A/Eurasian Wigeon/Netherlands/3/2022	A / H5N1	Europe / Netherlands / Krommenier, Woudpolder	Oanh Vuong-Zoet (Erasmus Medical Center / Department of Virology)
EPI_ISL_15925883	A/Eurasian Wigeon/Netherlands/4/2022	A / H5N1	Europe / Netherlands / Oud-Alblas	Oanh Vuong-Zoet (Erasmus Medical Center / Department of Virology)
EPI_ISL_15925884	A/Great Cormorant/Netherlands/1/2022	A / H5N1	Europe / Netherlands / Enkhuizen, Ijsselmeer, De Kreupel	Oanh Vuong-Zoet (Erasmus Medical Center / Department of Virology)

EPI_ISL_15925885	A/Black-headed Gull/Netherlands/11/2022	A / H5N1	Europe / Netherlands / Enkhuizen, Ijsselmeer, De Kreupel	Oanh Vuong-Zoet (Erasmus Medical Center / Department of Virology)
EPI_ISL_15942297	A/domestic_duck/England/152473/2022	A / H5N1	Europe / United Kingdom	Daniel Maskell (Animal and Plant Health Agency (APHA) / International Reference Laboratory)
EPI_ISL_15943002	A/Spot-billed_duck/Korea/K22-730-1/2022	A / H5N1	Asia / Korea, Republic of / Chungcheongnam-do	Dong-Hun Lee (Konkuk University / College of Veterinary Medicine)
EPI_ISL_15943015	A/Wild_bird/Korea/K22-742/2022	A / H5N1	Asia / Korea, Republic of / Chungcheongnam-do	Dong-Hun Lee (Konkuk University / College of Veterinary Medicine)
EPI_ISL_15944663	A/Spot-billed_duck/Korea/K22-856-2/2022	A / H5N1	Asia / Korea, Republic of	Dong-Hun Lee (Konkuk University / College of Veterinary Medicine)
EPI_ISL_15944665	A/Spot-billed_duck/Korea/K22-862-1/2022	A / H5N1	Asia / Korea, Republic of	Dong-Hun Lee (Konkuk University / College of Veterinary Medicine)
EPI_ISL_15944667	A/Spot-billed_duck/Korea/K22-920/2022	A / H5N1	Asia / Korea, Republic of	Dong-Hun Lee (Konkuk University / College of Veterinary Medicine)
EPI_ISL_159719	A/Chicken/Kumamoto/1-7/2014	A / H5N8	Asia / Japan / Kumamoto	Michiyo Yoshizawa Harada (National Agriculture and Food Research Organization / National Institute of Animal Health)
EPI_ISL_16003253	A/Red Fox/MB/FAV-370-01/2022	A / H5N1	North America / Canada / Manitoba	Yohannes Berhane (Canadian Food Inspection Agency / Avian Unit)
EPI_ISL_16003265	A/Red Fox/MB/FAV-414-004/2022	A / H5N1	North America / Canada / Manitoba	Yohannes Berhane (Canadian Food Inspection Agency / Avian Unit)
EPI_ISL_16003266	A/Red Fox/MB/FAV-414-08/2022	A / H5N1	North America / Canada / Manitoba	Yohannes Berhane (Canadian Food Inspection Agency / Avian Unit)
EPI_ISL_16003267	A/Red Fox/MB/FAV-0414-12/2022	A / H5N1	North America / Canada / Manitoba	Yohannes Berhane (Canadian Food Inspection Agency / Avian Unit)
EPI_ISL_16003268	A/Skunk/MB/FAV-470/2022	A / H5N1	North America / Canada / Manitoba	Yohannes Berhane (Canadian Food Inspection Agency / Avian Unit)
EPI_ISL_16013752	A/Pelican/Venezuela/Pel3/2022	A / H5N1	South America / Venezuela, Bolivarian Republic of / Estado Anzoategui	Flor Helene Pujol (Instituto Venezolano de investigaciones Científicas / Microbiology)
EPI_ISL_16013753	A/Pelican/Venezuela/Pel4/2022	A / H5N1	South America / Venezuela, Bolivarian Republic of / Estado Anzoategui	Flor Helene Pujol (Instituto Venezolano de investigaciones Científicas / Microbiology)
EPI_ISL_16056568	A/Eurasian Wigeon/Netherlands/5/2022	A / H5N1	Europe / Netherlands / Krommeniedijk-Woudpolder, Noord-Holland	Oanh Vuong-Zoet (Erasmus Medical Center / Department of Virology)
EPI_ISL_16056569	A/Gadwall/Netherlands/3/2022	A / H5N1	Europe / Netherlands / Eendenkooi Wieringen	Oanh Vuong-Zoet (Erasmus Medical Center / Department of Virology)
EPI_ISL_16056570	A/Mallard Duck/Netherlands/27/2022	A / H5N1	Europe / Netherlands / Oud Alblas	Oanh Vuong-Zoet (Erasmus Medical Center / Department of Virology)
EPI_ISL_16056571	A/Mallard Duck/Netherlands/28/2022	A / H5N1	Europe / Netherlands / Oud Alblas	Oanh Vuong-Zoet (Erasmus Medical Center / Department of Virology)
EPI_ISL_16056572	A/Mallard Duck/Netherlands/29/2022	A / H5N1	Europe / Netherlands / Oud Alblas	Oanh Vuong-Zoet (Erasmus Medical Center / Department of Virology)
EPI_ISL_16101521	A/chicken/Idaho/22-011336-001-original/2022	A / H5N1	North America / United States / Idaho / Caribou County	Mary Lea Killian (National Veterinary Services Laboratories - USDA / Diagnostic Virology Laboratory)
EPI_ISL_16101522	A/turkey/Minnesota/22-011337-001-original/2022	A / H5N1	North America / United States / Minnesota / Meeker County	Mary Lea Killian (National Veterinary Services Laboratories - USDA / Diagnostic Virology Laboratory)
EPI_ISL_16101523	A/turkey/Minnesota/22-011337-002-original/2022	A / H5N1	North America / United States / Minnesota / Meeker County	Mary Lea Killian (National Veterinary Services Laboratories - USDA / Diagnostic Virology Laboratory)
EPI_ISL_16101524	A/turkey/Minnesota/22-011338-001-original/2022	A / H5N1	North America / United States / Minnesota / Blue Earth County	Mary Lea Killian (National Veterinary Services Laboratories - USDA / Diagnostic Virology Laboratory)
EPI_ISL_16101525	A/turkey/Minnesota/22-011338-002-original/2022	A / H5N1	North America / United States / Minnesota / Blue Earth County	Mary Lea Killian (National Veterinary Services Laboratories - USDA / Diagnostic Virology Laboratory)
EPI_ISL_16101526	A/domestic duck/Indiana/22-011343-001-original/2022	A / H5N1	North America / United States / Indiana / Elkhart County	Mary Lea Killian (National Veterinary Services Laboratories - USDA / Diagnostic Virology Laboratory)
EPI_ISL_16101527	A/chicken/Idaho/22-011347-002-original/2022	A / H5N1	North America / United States / Idaho / Gooding County	Mary Lea Killian (National Veterinary Services Laboratories - USDA / Diagnostic Virology Laboratory)
EPI_ISL_16101528	A/chicken/Utah/22-011468-001-original/2022	A / H5N1	North America / United States / Utah / Utah County	Mary Lea Killian (National Veterinary Services Laboratories - USDA / Diagnostic Virology Laboratory)
EPI_ISL_16101529	A/chicken/Utah/22-011468-002-original/2022	A / H5N1	North America / United States / Utah / Utah County	Mary Lea Killian (National Veterinary Services Laboratories - USDA / Diagnostic Virology Laboratory)
EPI_ISL_16102154	A/turkey/Wisconsin/22-011508-001-original/2022	A / H5N1	North America / United States / Wisconsin / Polk County	Mary Lea Killian (National Veterinary Services Laboratories - USDA / Diagnostic Virology Laboratory)
EPI_ISL_16102155	A/turkey/Wisconsin/22-011508-002-original/2022	A / H5N1	North America / United States / Wisconsin / Polk County	Mary Lea Killian (National Veterinary Services Laboratories - USDA / Diagnostic Virology Laboratory)
EPI_ISL_16102156	A/domestic duck/Michigan/22-011521-001-original/2022	A / H5N1	North America / United States / Michigan / Macomb County	Mary Lea Killian (National Veterinary Services Laboratories - USDA / Diagnostic Virology Laboratory)
EPI_ISL_16102157	A/chicken/Michigan/22-011521-003-original/2022	A / H5N1	North America / United States / Michigan / Macomb County	Mary Lea Killian (National Veterinary Services Laboratories - USDA / Diagnostic Virology Laboratory)
EPI_ISL_16102158	A/chicken/Michigan/22-011521-004-original/2022	A / H5N1	North America / United States / Michigan / Macomb County	Mary Lea Killian (National Veterinary Services Laboratories - USDA / Diagnostic Virology Laboratory)
EPI_ISL_16102159	A/quail/Michigan/22-011521-005-original/2022	A / H5N1	North America / United States / Michigan / Macomb County	Mary Lea Killian (National Veterinary Services Laboratories - USDA / Diagnostic Virology Laboratory)
EPI_ISL_16102160	A/chicken/Michigan/22-011526-001-original/2022	A / H5N1	North America / United States / Michigan / Livingston County	Mary Lea Killian (National Veterinary Services Laboratories - USDA / Diagnostic Virology Laboratory)
EPI_ISL_16102161	A/domestic duck/Michigan/22-011526-002-original/2022	A / H5N1	North America / United States / Michigan / Livingston County	Mary Lea Killian (National Veterinary Services Laboratories - USDA / Diagnostic Virology Laboratory)
EPI_ISL_16102162	A/chicken/Michigan/22-011526-003-original/2022	A / H5N1	North America / United States / Michigan / Livingston County	Mary Lea Killian (National Veterinary Services Laboratories - USDA / Diagnostic Virology Laboratory)
EPI_ISL_16102163	A/chicken/Pennsylvania/22-011531-001-original/2022	A / H5N1	North America / United States / Pennsylvania / Lancaster County	Mary Lea Killian (National Veterinary Services Laboratories - USDA / Diagnostic Virology Laboratory)
EPI_ISL_16102164	A/chicken/Pennsylvania/22-011531-002-original/2022	A / H5N1	North America / United States / Pennsylvania / Lancaster County	Mary Lea Killian (National Veterinary Services Laboratories - USDA / Diagnostic Virology Laboratory)
EPI_ISL_16102165	A/chicken/Michigan/22-011627-001-original/2022	A / H5N1	North America / United States / Michigan / Menominee County	Mary Lea Killian (National Veterinary Services Laboratories - USDA / Diagnostic Virology Laboratory)
EPI_ISL_16102166	A/chicken/Pennsylvania/22-011767-001-original/2022	A / H5N1	North America / United States / Pennsylvania / Lancaster County	Mary Lea Killian (National Veterinary Services Laboratories - USDA / Diagnostic Virology Laboratory)
EPI_ISL_16102167	A/guinea fowl/Idaho/22-011775-001-original/2022	A / H5N1	North America / United States / Idaho / Gooding County	Mary Lea Killian (National Veterinary Services Laboratories - USDA / Diagnostic Virology Laboratory)
EPI_ISL_16102168	A/chicken/Idaho/22-011775-002-original/2022	A / H5N1	North America / United States / Idaho / Gooding County	Mary Lea Killian (National Veterinary Services Laboratories - USDA / Diagnostic Virology Laboratory)
EPI_ISL_16102169	A/chicken/North Dakota/22-011778-001-original/2022	A / H5N1	North America / United States / North Dakota / Stutsman County	Mary Lea Killian (National Veterinary Services Laboratories - USDA / Diagnostic Virology Laboratory)
EPI_ISL_16102170	A/turkey/Minnesota/22-011787-001-original/2022	A / H5N1	North America / United States / Minnesota / Otter Tail County	Mary Lea Killian (National Veterinary Services Laboratories - USDA / Diagnostic Virology Laboratory)
EPI_ISL_16102171	A/turkey/Minnesota/22-011787-002-original/2022	A / H5N1	North America / United States / Minnesota / Otter Tail County	Mary Lea Killian (National Veterinary Services Laboratories - USDA / Diagnostic Virology Laboratory)
EPI_ISL_16102172	A/turkey/Minnesota/22-011789-001-original/2022	A / H5N1	North America / United States / Minnesota / Stearns County	Mary Lea Killian (National Veterinary Services Laboratories - USDA / Diagnostic Virology Laboratory)
EPI_ISL_16102173	A/turkey/Minnesota/22-011789-002-original/2022	A / H5N1	North America / United States / Minnesota / Stearns County	Mary Lea Killian (National Veterinary Services Laboratories - USDA / Diagnostic Virology Laboratory)
EPI_ISL_16102174	A/turkey/Minnesota/22-011790-002-original/2022	A / H5N1	North America / United States / Minnesota / Morrison County	Mary Lea Killian (National Veterinary Services Laboratories - USDA / Diagnostic Virology Laboratory)
EPI_ISL_16102227	A/turkey/Minnesota/22-011791-003-original/2022	A / H5N1	North America / United States / Minnesota / Kandiyohi County	Mary Lea Killian (National Veterinary Services Laboratories - USDA / Diagnostic Virology Laboratory)
EPI_ISL_16102228	A/turkey/Minnesota/22-011791-004-original/2022	A / H5N1	North America / United States / Minnesota / Kandiyohi County	Mary Lea Killian (National Veterinary Services Laboratories - USDA / Diagnostic Virology Laboratory)
EPI_ISL_16102229	A/turkey/Minnesota/22-011792-001-original/2022	A / H5N1	North America / United States / Minnesota / Morrison County	Mary Lea Killian (National Veterinary Services Laboratories - USDA / Diagnostic Virology Laboratory)
EPI_ISL_16102230	A/turkey/Minnesota/22-011792-002-original/2022	A / H5N1	North America / United States / Minnesota / Morrison County	Mary Lea Killian (National Veterinary Services Laboratories - USDA / Diagnostic Virology Laboratory)
EPI_ISL_16102261	A/turkey/Minnesota/22-011793-001-original/2022	A / H5N1	North America / United States / Minnesota / Meeker County	Mary Lea Killian (National Veterinary Services Laboratories - USDA / Diagnostic Virology Laboratory)
EPI_ISL_16102262	A/turkey/Minnesota/22-011793-002-original/2022	A / H5N1	North America / United States / Minnesota / Meeker County	Mary Lea Killian (National Veterinary Services Laboratories - USDA / Diagnostic Virology Laboratory)
EPI_ISL_16102263	A/turkey/Minnesota/22-011794-001-original/2022	A / H5N1	North America / United States / Minnesota / Swift County	Mary Lea Killian (National Veterinary Services Laboratories - USDA / Diagnostic Virology Laboratory)
EPI_ISL_16102264	A/turkey/Minnesota/22-011794-002-original/2022	A / H5N1	North America / United States / Minnesota / Swift County	Mary Lea Killian (National Veterinary Services Laboratories - USDA / Diagnostic Virology Laboratory)
EPI_ISL_16102265	A/turkey/Minnesota/22-011795-001-original/2022	A / H5N1	North America / United States / Minnesota / Kandiyohi County	Mary Lea Killian (National Veterinary Services Laboratories - USDA / Diagnostic Virology Laboratory)
EPI_ISL_16102266	A/turkey/Minnesota/22-011796-001-original/2022	A / H5N1	North America / United States / Minnesota / Morrison County	Mary Lea Killian (National Veterinary Services Laboratories - USDA / Diagnostic Virology Laboratory)
EPI_ISL_16102267	A/turkey/Minnesota/22-011796-002-original/2022	A / H5N1	North America / United States / Minnesota / Morrison County	Mary Lea Killian (National Veterinary Services Laboratories - USDA / Diagnostic Virology Laboratory)
EPI_ISL_16102275	A/chicken/Colorado/22-011826-001-original/2022	A / H5N1	North America / United States / Colorado / Montrose County	Mary Lea Killian (National Veterinary Services Laboratories - USDA / Diagnostic Virology Laboratory)
EPI_ISL_16102276	A/chicken/Colorado/22-011826-002-original/2022	A / H5N1	North America / United States / Colorado / Montrose County	Mary Lea Killian (National Veterinary Services Laboratories - USDA / Diagnostic Virology Laboratory)
EPI_ISL_16102277	A/Indian Runner duck/Colorado/22-011827-001-original/2022	A / H5N1	North America / United States / Colorado / La Plata County	Mary Lea Killian (National Veterinary Services Laboratories - USDA / Diagnostic Virology Laboratory)
EPI_ISL_16102278	A/chicken/Colorado/22-011827-002-original/2022	A / H5N1	North America / United States / Colorado / La Plata County	Mary Lea Killian (National Veterinary Services Laboratories - USDA / Diagnostic Virology Laboratory)
EPI_ISL_16102279	A/chicken/Pennsylvania/22-011920-001-original/2022	A / H5N1	North America / United States / Pennsylvania / Lancaster County	Mary Lea Killian (National Veterinary Services Laboratories - USDA / Diagnostic Virology Laboratory)
EPI_ISL_16102280	A/chicken/Pennsylvania/22-011921-002-original/2022	A / H5N1	North America / United States / Pennsylvania / Lancaster County	Mary Lea Killian (National Veterinary Services Laboratories - USDA / Diagnostic Virology Laboratory)
EPI_ISL_16102281	A/duck/Indiana/22-011946-002-original/2022	A / H5N1	North America / United States / Indiana / Elkhart County	Mary Lea Killian (National Veterinary Services Laboratories - USDA / Diagnostic Virology Laboratory)
EPI_ISL_16102282	A/duck/Indiana/22-011946-004-original/2022	A / H5N1	North America / United States / Indiana / Elkhart County	Mary Lea Killian (National Veterinary Services Laboratories - USDA / Diagnostic Virology Laboratory)
EPI_ISL_16102283	A/turkey/Minnesota/22-012034-001-original/2022	A / H5N1	North America / United States / Minnesota / Todd County	Mary Lea Killian (National Veterinary Services Laboratories - USDA / Diagnostic Virology Laboratory)
EPI_ISL_16102926	A/turkey/Minnesota/22-012036-001-original/2022	A / H5N1	North America / United States / Minnesota / Stearns County	Mary Lea Killian (National Veterinary Services Laboratories - USDA / Diagnostic Virology Laboratory)
EPI_ISL_16102927	A/turkey/Minnesota/22-012036-002-original/2022	A / H5N1	North America / United States / Minnesota / Stearns County	Mary Lea Killian (National Veterinary Services Laboratories - USDA / Diagnostic Virology Laboratory)

EPI_ISL_16102928	A/chicken/Montana/22-012040-001-original/2022	A / H5N1	North America / United States / Montana / Glacier County	Mary Lea Killian (National Veterinary Services Laboratories - USDA / Diagnostic Virology Laboratory)
EPI_ISL_16102929	A/mallard/Montana/22-012040-005-original/2022	A / H5N1	North America / United States / Montana / Glacier County	Mary Lea Killian (National Veterinary Services Laboratories - USDA / Diagnostic Virology Laboratory)
EPI_ISL_16102930	A/chicken/Idaho/22-012045-001-original/2022	A / H5N1	North America / United States / Idaho / Madison County	Mary Lea Killian (National Veterinary Services Laboratories - USDA / Diagnostic Virology Laboratory)
EPI_ISL_16102931	A/chicken/Idaho/22-012045-002-original/2022	A / H5N1	North America / United States / Idaho / Madison County	Mary Lea Killian (National Veterinary Services Laboratories - USDA / Diagnostic Virology Laboratory)
EPI_ISL_16102932	A/duck/Idaho/22-012046-001-original/2022	A / H5N1	North America / United States / Idaho / Madison County	Mary Lea Killian (National Veterinary Services Laboratories - USDA / Diagnostic Virology Laboratory)
EPI_ISL_16102933	A/goose/Idaho/22-012047-001-original/2022	A / H5N1	North America / United States / Idaho / Madison County	Mary Lea Killian (National Veterinary Services Laboratories - USDA / Diagnostic Virology Laboratory)
EPI_ISL_16112044	A/Arctic_tern/Denmark/05803-1.01/2022	A / H5N1	Europe / Denmark / Region Nordjylland / Thisted Kommune	Charlotte Kristiane Hjulsager (Statens Serum Institute / Microbiological Diagnostic and Virology)
EPI_ISL_16113089	A/barnacle_goose/Denmark/03332-1.02/2022	A / H5N1	Europe / Denmark / Region Midtjylland / Ringkobing-Skjern	Charlotte Kristiane Hjulsager (Statens Serum Institute / Microbiological Diagnostic and Virology)
EPI_ISL_16113090	A/peregrine_falcon/Denmark/06880-1.02/2022	A / H5N1	Europe / Denmark / Region Sjaland / Vordingborg Kommune	Charlotte Kristiane Hjulsager (Statens Serum Institute / Microbiological Diagnostic and Virology)
EPI_ISL_16113225	A/sandwich_tern/Denmark/05712-1.02/2022	A / H5N1	Europe / Denmark / Region Sjaland / Slagelse Kommune	Charlotte Kristiane Hjulsager (Statens Serum Institute / Microbiological Diagnostic and Virology)
EPI_ISL_16113229	A/sandwich_tern/Denmark/05802-1.01_2022	A / H5N1	Europe / Denmark / Region Nordjylland / Thisted Kommune	Charlotte Kristiane Hjulsager (Statens Serum Institute / Microbiological Diagnostic and Virology)
EPI_ISL_16113230	A/sandwich_tern/Denmark/05806-1.01/2022	A / H5N1	Europe / Denmark / Region Nordjylland / Thisted Kommune	Charlotte Kristiane Hjulsager (Statens Serum Institute / Microbiological Diagnostic and Virology)
EPI_ISL_16113231	A/tawny_owl/Denmark/05372-1.01/2022	A / H5N1	Europe / Denmark / Region Syddanmark / Odense Kommune	Charlotte Kristiane Hjulsager (Statens Serum Institute / Microbiological Diagnostic and Virology)
EPI_ISL_16113242	A/tawny_owl/Denmark/05373-1.01/2022	A / H5N1	Europe / Denmark / Region Syddanmark / Odense Kommune	Charlotte Kristiane Hjulsager (Statens Serum Institute / Microbiological Diagnostic and Virology)
EPI_ISL_16113366	A/white-tailed_eagle/Denmark/05716-1.04/2022	A / H5N1	Europe / Denmark / Region Midtjylland / Horsens Kommune	Charlotte Kristiane Hjulsager (Statens Serum Institute / Microbiological Diagnostic and Virology)
EPI_ISL_16115054	A/common_buzzard/Denmark/08287-1.01/2022	A / H5N1	Europe / Denmark / Region Syddanmark / Sonderborg Kommune	Charlotte Kristiane Hjulsager (Statens Serum Institute / Microbiological Diagnostic and Virology)
EPI_ISL_16157545	A/chicken/Ecuador/NIC-INSPI-AGROCALIDAD-FBC-2/2022	A / H5N1	South America / Ecuador / Provincia de Cotopaxi	Alfredo Bruno Caicedo (Instituto Nacional de Investigacion en Salud Publica (INSPI) / Centro Nacional de Influenza (NIC))
EPI_ISL_16161673	A/chicken/Ecuador/NIC-INSPI-AGROCALIDAD-FBT-3/2022	A / H5N1	South America / Ecuador / Provincia de Cotopaxi	Alfredo Bruno Caicedo (Instituto Nacional de Investigacion en Salud Publica (INSPI) / Centro Nacional de Influenza (NIC))
EPI_ISL_16161675	A/chicken/Ecuador/NIC-INSPI-AGROCALIDAD-FBC-4/2022	A / H5N1	South America / Ecuador / Provincia de Cotopaxi	Alfredo Bruno Caicedo (Instituto Nacional de Investigacion en Salud Publica (INSPI) / Centro Nacional de Influenza (NIC))
EPI_ISL_16171247	A/chicken/Pennsylvania/22-012091-001-original/2022	A / H5N1	North America / United States / Pennsylvania / Lancaster County	Mary Lea Killian (National Veterinary Services Laboratories - USDA / Diagnostic Virology Laboratory)
EPI_ISL_16171248	A/chicken/Pennsylvania/22-012092-003-original/2022	A / H5N1	North America / United States / Pennsylvania / Lancaster County	Mary Lea Killian (National Veterinary Services Laboratories - USDA / Diagnostic Virology Laboratory)
EPI_ISL_16171249	A/chicken/Pennsylvania/22-012092-005-original/2022	A / H5N1	North America / United States / Pennsylvania / Lancaster County	Mary Lea Killian (National Veterinary Services Laboratories - USDA / Diagnostic Virology Laboratory)
EPI_ISL_16171250	A/turkey/North Dakota/22-012099-001-original/2022	A / H5N1	North America / United States / North Dakota / Richland County	Mary Lea Killian (National Veterinary Services Laboratories - USDA / Diagnostic Virology Laboratory)
EPI_ISL_16171251	A/turkey/North Dakota/22-012099-002-original/2022	A / H5N1	North America / United States / North Dakota / Richland County	Mary Lea Killian (National Veterinary Services Laboratories - USDA / Diagnostic Virology Laboratory)
EPI_ISL_16171252	A/chicken/North Dakota/22-012100-001-original/2022	A / H5N1	North America / United States / North Dakota / Renville County	Mary Lea Killian (National Veterinary Services Laboratories - USDA / Diagnostic Virology Laboratory)
EPI_ISL_16171254	A/turkey/Minnesota/22-012123-001-original/2022	A / H5N1	North America / United States / Minnesota / Todd County	Mary Lea Killian (National Veterinary Services Laboratories - USDA / Diagnostic Virology Laboratory)
EPI_ISL_16171255	A/turkey/Minnesota/22-012125-001-original/2022	A / H5N1	North America / United States / Minnesota / Morrison County	Mary Lea Killian (National Veterinary Services Laboratories - USDA / Diagnostic Virology Laboratory)
EPI_ISL_16171256	A/turkey/Minnesota/22-012125-002-original/2022	A / H5N1	North America / United States / Minnesota / Morrison County	Mary Lea Killian (National Veterinary Services Laboratories - USDA / Diagnostic Virology Laboratory)
EPI_ISL_16171322	A/turkey/Minnesota/22-012126-002-original/2022	A / H5N1	North America / United States / Minnesota / Todd County	Mary Lea Killian (National Veterinary Services Laboratories - USDA / Diagnostic Virology Laboratory)
EPI_ISL_16171323	A/turkey/Minnesota/22-012126-003-original/2022	A / H5N1	North America / United States / Minnesota / Todd County	Mary Lea Killian (National Veterinary Services Laboratories - USDA / Diagnostic Virology Laboratory)
EPI_ISL_16171324	A/chicken/Pennsylvania/22-012248-001-original/2022	A / H5N1	North America / United States / Pennsylvania / Lancaster County	Mary Lea Killian (National Veterinary Services Laboratories - USDA / Diagnostic Virology Laboratory)
EPI_ISL_16171325	A/chicken/Pennsylvania/22-012248-003-original/2022	A / H5N1	North America / United States / Pennsylvania / Lancaster County	Mary Lea Killian (National Veterinary Services Laboratories - USDA / Diagnostic Virology Laboratory)
EPI_ISL_16171326	A/turkey/Minnesota/22-012285-001-original/2022	A / H5N1	North America / United States / Minnesota / Swift County	Mary Lea Killian (National Veterinary Services Laboratories - USDA / Diagnostic Virology Laboratory)
EPI_ISL_16171327	A/turkey/Minnesota/22-012285-002-original/2022	A / H5N1	North America / United States / Minnesota / Swift County	Mary Lea Killian (National Veterinary Services Laboratories - USDA / Diagnostic Virology Laboratory)
EPI_ISL_16171328	A/turkey/Minnesota/22-012286-001-original/2022	A / H5N1	North America / United States / Minnesota / Otter Tail County	Mary Lea Killian (National Veterinary Services Laboratories - USDA / Diagnostic Virology Laboratory)
EPI_ISL_16171329	A/turkey/Minnesota/22-012286-002-original/2022	A / H5N1	North America / United States / Minnesota / Otter Tail County	Mary Lea Killian (National Veterinary Services Laboratories - USDA / Diagnostic Virology Laboratory)
EPI_ISL_16171330	A/turkey/Minnesota/22-012287-001-original/2022	A / H5N1	North America / United States / Minnesota / Yellow Medicine County	Mary Lea Killian (National Veterinary Services Laboratories - USDA / Diagnostic Virology Laboratory)
EPI_ISL_16171331	A/chicken/Colorado/22-012308-001-original/2022	A / H5N1	North America / United States / Colorado / Montrose County	Mary Lea Killian (National Veterinary Services Laboratories - USDA / Diagnostic Virology Laboratory)
EPI_ISL_16171332	A/chicken/Colorado/22-012308-002-original/2022	A / H5N1	North America / United States / Colorado / Montrose County	Mary Lea Killian (National Veterinary Services Laboratories - USDA / Diagnostic Virology Laboratory)
EPI_ISL_16171399	A/chicken/Minnesota/22-012373-001-original/2022	A / H5N1	North America / United States / Minnesota / Rice County	Mary Lea Killian (National Veterinary Services Laboratories - USDA / Diagnostic Virology Laboratory)
EPI_ISL_16171400	A/goose/Minnesota/22-012373-002-original/2022	A / H5N1	North America / United States / Minnesota / Rice County	Mary Lea Killian (National Veterinary Services Laboratories - USDA / Diagnostic Virology Laboratory)
EPI_ISL_16171401	A/turkey/Minnesota/22-012374-001-original/2022	A / H5N1	North America / United States / Minnesota / Stearns County	Mary Lea Killian (National Veterinary Services Laboratories - USDA / Diagnostic Virology Laboratory)
EPI_ISL_16171402	A/chicken/Utah/22-012375-001-original/2022	A / H5N1	North America / United States / Utah / Cache County	Mary Lea Killian (National Veterinary Services Laboratories - USDA / Diagnostic Virology Laboratory)
EPI_ISL_16171403	A/chicken/Wisconsin/22-012376-001-original/2022	A / H5N1	North America / United States / Wisconsin / Polk County	Mary Lea Killian (National Veterinary Services Laboratories - USDA / Diagnostic Virology Laboratory)
EPI_ISL_16171404	A/duck/Wisconsin/22-012376-002-original/2022	A / H5N1	North America / United States / Wisconsin / Polk County	Mary Lea Killian (National Veterinary Services Laboratories - USDA / Diagnostic Virology Laboratory)
EPI_ISL_16171405	A/chicken/Wisconsin/22-012376-003-original/2022	A / H5N1	North America / United States / Wisconsin / Polk County	Mary Lea Killian (National Veterinary Services Laboratories - USDA / Diagnostic Virology Laboratory)
EPI_ISL_16171406	A/domestic duck/Michigan/22-012454-001-original/2022	A / H5N1	North America / United States / Michigan / Menominee County	Mary Lea Killian (National Veterinary Services Laboratories - USDA / Diagnostic Virology Laboratory)
EPI_ISL_16171407	A/domestic goose/Michigan/22-012454-002-original/2022	A / H5N1	North America / United States / Michigan / Menominee County	Mary Lea Killian (National Veterinary Services Laboratories - USDA / Diagnostic Virology Laboratory)
EPI_ISL_16171408	A/chicken/Iowa/22-012510-002-original/2022	A / H5N1	North America / United States / Iowa / Kossuth County	Mary Lea Killian (National Veterinary Services Laboratories - USDA / Diagnostic Virology Laboratory)
EPI_ISL_16171409	A/chicken/North Dakota/22-012538-001-original/2022	A / H5N1	North America / United States / North Dakota / Richland County	Mary Lea Killian (National Veterinary Services Laboratories - USDA / Diagnostic Virology Laboratory)
EPI_ISL_16171411	A/chicken/North Dakota/22-012538-002-original/2022	A / H5N1	North America / United States / North Dakota / Richland County	Mary Lea Killian (National Veterinary Services Laboratories - USDA / Diagnostic Virology Laboratory)
EPI_ISL_16171412	A/chicken/Montana/22-012540-001-original/2022	A / H5N1	North America / United States / Montana / Missoula County	Mary Lea Killian (National Veterinary Services Laboratories - USDA / Diagnostic Virology Laboratory)
EPI_ISL_16171413	A/chicken/Montana/22-012540-002-original/2022	A / H5N1	North America / United States / Montana / Missoula County	Mary Lea Killian (National Veterinary Services Laboratories - USDA / Diagnostic Virology Laboratory)
EPI_ISL_16171415	A/chicken/Pennsylvania/22-012578-004-original/2022	A / H5N1	North America / United States / Pennsylvania / Lancaster County	Mary Lea Killian (National Veterinary Services Laboratories - USDA / Diagnostic Virology Laboratory)
EPI_ISL_16171416	A/chicken/Kansas/22-012741-003-original/2022	A / H5N1	North America / United States / Kansas / Republic County	Mary Lea Killian (National Veterinary Services Laboratories - USDA / Diagnostic Virology Laboratory)
EPI_ISL_16171417	A/chicken/Pennsylvania/22-012742-001-original/2022	A / H5N1	North America / United States / Pennsylvania / Lancaster County	Mary Lea Killian (National Veterinary Services Laboratories - USDA / Diagnostic Virology Laboratory)
EPI_ISL_16171418	A/chicken/Pennsylvania/22-012742-002-original/2022	A / H5N1	North America / United States / Pennsylvania / Lancaster County	Mary Lea Killian (National Veterinary Services Laboratories - USDA / Diagnostic Virology Laboratory)
EPI_ISL_16171419	A/chicken/Nebraska/22-012756-001-original/2022	A / H5N1	North America / United States / Nebraska / Knox County	Mary Lea Killian (National Veterinary Services Laboratories - USDA / Diagnostic Virology Laboratory)
EPI_ISL_16171449	A/chicken/Michigan/22-012855-001-original/2022	A / H5N1	North America / United States / Michigan / Saginaw County	Mary Lea Killian (National Veterinary Services Laboratories - USDA / Diagnostic Virology Laboratory)
EPI_ISL_16171450	A/guinea fowl/Michigan/22-012855-002-original/2022	A / H5N1	North America / United States / Michigan / Saginaw County	Mary Lea Killian (National Veterinary Services Laboratories - USDA / Diagnostic Virology Laboratory)
EPI_ISL_16171451	A/turkey/Michigan/22-012855-003-original/2022	A / H5N1	North America / United States / Michigan / Saginaw County	Mary Lea Killian (National Veterinary Services Laboratories - USDA / Diagnostic Virology Laboratory)
EPI_ISL_16171452	A/domestic goose/Michigan/22-012855-004-original/2022	A / H5N1	North America / United States / Michigan / Saginaw County	Mary Lea Killian (National Veterinary Services Laboratories - USDA / Diagnostic Virology Laboratory)
EPI_ISL_16171453	A/domestic duck/Michigan/22-012855-005-original/2022	A / H5N1	North America / United States / Michigan / Saginaw County	Mary Lea Killian (National Veterinary Services Laboratories - USDA / Diagnostic Virology Laboratory)
EPI_ISL_16171454	A/chicken/Minnesota/22-012932-001-original/2022	A / H5N1	North America / United States / Minnesota / Carver County	Mary Lea Killian (National Veterinary Services Laboratories - USDA / Diagnostic Virology Laboratory)
EPI_ISL_16171455	A/chicken/Minnesota/22-012932-002-original/2022	A / H5N1	North America / United States / Minnesota / Carver County	Mary Lea Killian (National Veterinary Services Laboratories - USDA / Diagnostic Virology Laboratory)
EPI_ISL_16171456	A/chicken/Indiana/22-012933-001-original/2022	A / H5N1	North America / United States / Indiana / Johnson County	Mary Lea Killian (National Veterinary Services Laboratories - USDA / Diagnostic Virology Laboratory)
EPI_ISL_16171457	A/chicken/Minnesota/22-012966-001-original/2022	A / H5N1	North America / United States / Minnesota / Swift County	Mary Lea Killian (National Veterinary Services Laboratories - USDA / Diagnostic Virology Laboratory)
EPI_ISL_16171458	A/turkey/Wisconsin/22-012973-001-original/2022	A / H5N1	North America / United States / Wisconsin / Barron County	Mary Lea Killian (National Veterinary Services Laboratories - USDA / Diagnostic Virology Laboratory)
EPI_ISL_16171459	A/turkey/Wisconsin/22-012974-001-original/2022	A / H5N1	North America / United States / Wisconsin / Barron County	Mary Lea Killian (National Veterinary Services Laboratories - USDA / Diagnostic Virology Laboratory)
EPI_ISL_16171460	A/turkey/Wisconsin/22-012974-002-original/2022	A / H5N1	North America / United States / Wisconsin / Barron County	Mary Lea Killian (National Veterinary Services Laboratories - USDA / Diagnostic Virology Laboratory)
EPI_ISL_16171461	A/chicken/Michigan/22-012977-001-original/2022	A / H5N1	North America / United States / Michigan / Wexford County	Mary Lea Killian (National Veterinary Services Laboratories - USDA / Diagnostic Virology Laboratory)
EPI_ISL_16171462	A/chicken/Montana/22-013053-001-original/2022	A / H5N1	North America / United States / Montana / Gallatin County	Mary Lea Killian (National Veterinary Services Laboratories - USDA / Diagnostic Virology Laboratory)

EPI_ISL_16171463	A/chicken/Montana/22-013053-002-original/2022	A / H5N1	North America / United States / Montana / Gallatin County	Mary Lea Killian (National Veterinary Services Laboratories - USDA / Diagnostic Virology Laboratory)
EPI_ISL_16171464	A/duck/Montana/22-013055-005-original/2022	A / H5N1	North America / United States / Montana / Pondera County	Mary Lea Killian (National Veterinary Services Laboratories - USDA / Diagnostic Virology Laboratory)
EPI_ISL_16171465	A/duck/Montana/22-013055-006-original/2022	A / H5N1	North America / United States / Montana / Pondera County	Mary Lea Killian (National Veterinary Services Laboratories - USDA / Diagnostic Virology Laboratory)
EPI_ISL_16171466	A/chicken/Montana/22-013056-002-original/2022	A / H5N1	North America / United States / Montana / Fergus County	Mary Lea Killian (National Veterinary Services Laboratories - USDA / Diagnostic Virology Laboratory)
EPI_ISL_16171467	A/chicken/Colorado/22-013057-001-original/2022	A / H5N1	North America / United States / Colorado / Weld County	Mary Lea Killian (National Veterinary Services Laboratories - USDA / Diagnostic Virology Laboratory)
EPI_ISL_16171468	A/guinea fowl/Wisconsin/22-013061-003-original/2022	A / H5N1	North America / United States / Wisconsin / Oconto County	Mary Lea Killian (National Veterinary Services Laboratories - USDA / Diagnostic Virology Laboratory)
EPI_ISL_16171469	A/peacock/Wisconsin/22-013061-004-original/2022	A / H5N1	North America / United States / Wisconsin / Oconto County	Mary Lea Killian (National Veterinary Services Laboratories - USDA / Diagnostic Virology Laboratory)
EPI_ISL_16171470	A/Lady Amherst's pheasant/Wisconsin/22-013061-005-origina	A / H5N1	North America / United States / Wisconsin / Oconto County	Mary Lea Killian (National Veterinary Services Laboratories - USDA / Diagnostic Virology Laboratory)
EPI_ISL_16171471	A/turkey/Minnesota/22-013084-001-original/2022	A / H5N1	North America / United States / Minnesota / Stearns County	Mary Lea Killian (National Veterinary Services Laboratories - USDA / Diagnostic Virology Laboratory)
EPI_ISL_16171472	A/turkey/Minnesota/22-013084-002-original/2022	A / H5N1	North America / United States / Minnesota / Stearns County	Mary Lea Killian (National Veterinary Services Laboratories - USDA / Diagnostic Virology Laboratory)
EPI_ISL_16171473	A/chicken/Nebraska/22-013105-001-original/2022	A / H5N1	North America / United States / Nebraska / Washington County	Mary Lea Killian (National Veterinary Services Laboratories - USDA / Diagnostic Virology Laboratory)
EPI_ISL_16171474	A/chicken/Alaska/22-013107-001-original/2022	A / H5N1	North America / United States / Alaska / Matanuska-Susitna Borough	Mary Lea Killian (National Veterinary Services Laboratories - USDA / Diagnostic Virology Laboratory)
EPI_ISL_16171475	A/chicken/Alaska/22-013107-002-original/2022	A / H5N1	North America / United States / Alaska / Matanuska-Susitna Borough	Mary Lea Killian (National Veterinary Services Laboratories - USDA / Diagnostic Virology Laboratory)
EPI_ISL_16171476	A/turkey/Wisconsin/22-013183-002-original/2022	A / H5N1	North America / United States / Wisconsin / Barron County	Mary Lea Killian (National Veterinary Services Laboratories - USDA / Diagnostic Virology Laboratory)
EPI_ISL_16171477	A/guinea fowl/Wisconsin/22-013183-003-original/2022	A / H5N1	North America / United States / Wisconsin / Barron County	Mary Lea Killian (National Veterinary Services Laboratories - USDA / Diagnostic Virology Laboratory)
EPI_ISL_16171478	A/domestic duck/Wisconsin/22-013183-004-original/2022	A / H5N1	North America / United States / Wisconsin / Barron County	Mary Lea Killian (National Veterinary Services Laboratories - USDA / Diagnostic Virology Laboratory)
EPI_ISL_16171479	A/peacock/Wisconsin/22-013183-005-original/2022	A / H5N1	North America / United States / Wisconsin / Barron County	Mary Lea Killian (National Veterinary Services Laboratories - USDA / Diagnostic Virology Laboratory)
EPI_ISL_16171480	A/Pekin duck/Pennsylvania/22-013189-003-original/2022	A / H5N1	North America / United States / Pennsylvania / Lancaster County	Mary Lea Killian (National Veterinary Services Laboratories - USDA / Diagnostic Virology Laboratory)
EPI_ISL_16171481	A/Pekin duck/Pennsylvania/22-013189-004-original/2022	A / H5N1	North America / United States / Pennsylvania / Lancaster County	Mary Lea Killian (National Veterinary Services Laboratories - USDA / Diagnostic Virology Laboratory)
EPI_ISL_16171482	A/chicken/Oklahoma/22-013220-001-original/2022	A / H5N1	North America / United States / Oklahoma / Sequoyah County	Mary Lea Killian (National Veterinary Services Laboratories - USDA / Diagnostic Virology Laboratory)
EPI_ISL_16171483	A/chicken/Oklahoma/22-013220-002-original/2022	A / H5N1	North America / United States / Oklahoma / Sequoyah County	Mary Lea Killian (National Veterinary Services Laboratories - USDA / Diagnostic Virology Laboratory)
EPI_ISL_16171485	A/goose/Minnesota/22-013221-002-original/2022	A / H5N1	North America / United States / Minnesota / Morrison County	Mary Lea Killian (National Veterinary Services Laboratories - USDA / Diagnostic Virology Laboratory)
EPI_ISL_16171486	A/duck/Minnesota/22-013221-003-original/2022	A / H5N1	North America / United States / Minnesota / Morrison County	Mary Lea Killian (National Veterinary Services Laboratories - USDA / Diagnostic Virology Laboratory)
EPI_ISL_16171487	A/duck/Minnesota/22-013221-004-original/2022	A / H5N1	North America / United States / Minnesota / Morrison County	Mary Lea Killian (National Veterinary Services Laboratories - USDA / Diagnostic Virology Laboratory)
EPI_ISL_16171488	A/chicken/Minnesota/22-013222-001-original/2022	A / H5N1	North America / United States / Minnesota / Chisago County	Mary Lea Killian (National Veterinary Services Laboratories - USDA / Diagnostic Virology Laboratory)
EPI_ISL_16171490	A/goose/Minnesota/22-013222-004-original/2022	A / H5N1	North America / United States / Minnesota / Chisago County	Mary Lea Killian (National Veterinary Services Laboratories - USDA / Diagnostic Virology Laboratory)
EPI_ISL_16171491	A/goose/Minnesota/22-013222-005-original/2022	A / H5N1	North America / United States / Minnesota / Chisago County	Mary Lea Killian (National Veterinary Services Laboratories - USDA / Diagnostic Virology Laboratory)
EPI_ISL_16171492	A/chicken/Minnesota/22-013223-001-original/2022	A / H5N1	North America / United States / Minnesota / Chisago County	Mary Lea Killian (National Veterinary Services Laboratories - USDA / Diagnostic Virology Laboratory)
EPI_ISL_16171493	A/chicken/Minnesota/22-013223-003-original/2022	A / H5N1	North America / United States / Minnesota / Chisago County	Mary Lea Killian (National Veterinary Services Laboratories - USDA / Diagnostic Virology Laboratory)
EPI_ISL_16171494	A/chicken/Michigan/22-013224-001-original/2022	A / H5N1	North America / United States / Michigan / Branch County	Mary Lea Killian (National Veterinary Services Laboratories - USDA / Diagnostic Virology Laboratory)
EPI_ISL_16171495	A/duck/Pennsylvania/22-013368-002-original/2022	A / H5N1	North America / United States / Pennsylvania / Berks County	Mary Lea Killian (National Veterinary Services Laboratories - USDA / Diagnostic Virology Laboratory)
EPI_ISL_16171496	A/duck/Wisconsin/22-013373-001-original/2022	A / H5N1	North America / United States / Wisconsin / Sauk County	Mary Lea Killian (National Veterinary Services Laboratories - USDA / Diagnostic Virology Laboratory)
EPI_ISL_16171497	A/chicken/Wisconsin/22-013373-002-original/2022	A / H5N1	North America / United States / Wisconsin / Sauk County	Mary Lea Killian (National Veterinary Services Laboratories - USDA / Diagnostic Virology Laboratory)
EPI_ISL_16171498	A/chicken/Illinois/22-013392-001-original/2022	A / H5N1	North America / United States / Illinois / Kane County	Mary Lea Killian (National Veterinary Services Laboratories - USDA / Diagnostic Virology Laboratory)
EPI_ISL_16171499	A/chicken/Illinois/22-013392-002-original/2022	A / H5N1	North America / United States / Illinois / Kane County	Mary Lea Killian (National Veterinary Services Laboratories - USDA / Diagnostic Virology Laboratory)
EPI_ISL_16171500	A/chicken/Minnesota/22-013412-001-original/2022	A / H5N1	North America / United States / Minnesota / Chisago County	Mary Lea Killian (National Veterinary Services Laboratories - USDA / Diagnostic Virology Laboratory)
EPI_ISL_16171501	A/chicken/Minnesota/22-013412-002-original/2022	A / H5N1	North America / United States / Minnesota / Chisago County	Mary Lea Killian (National Veterinary Services Laboratories - USDA / Diagnostic Virology Laboratory)
EPI_ISL_16171502	A/turkey/Minnesota/22-013413-002-original/2022	A / H5N1	North America / United States / Minnesota / Lyon County	Mary Lea Killian (National Veterinary Services Laboratories - USDA / Diagnostic Virology Laboratory)
EPI_ISL_16171503	A/turkey/Minnesota/22-013413-003-original/2022	A / H5N1	North America / United States / Minnesota / Lyon County	Mary Lea Killian (National Veterinary Services Laboratories - USDA / Diagnostic Virology Laboratory)
EPI_ISL_16171504	A/chicken/Wisconsin/22-013445-001-original/2022	A / H5N1	North America / United States / Wisconsin / Fond du Lac County	Mary Lea Killian (National Veterinary Services Laboratories - USDA / Diagnostic Virology Laboratory)
EPI_ISL_16171505	A/chicken/Wisconsin/22-013446-001-original/2022	A / H5N1	North America / United States / Wisconsin / Polk County	Mary Lea Killian (National Veterinary Services Laboratories - USDA / Diagnostic Virology Laboratory)
EPI_ISL_16171506	A/turkey/Wisconsin/22-013447-001-original/2022	A / H5N1	North America / United States / Wisconsin / Barron County	Mary Lea Killian (National Veterinary Services Laboratories - USDA / Diagnostic Virology Laboratory)
EPI_ISL_16171507	A/turkey/Wisconsin/22-013447-002-original/2022	A / H5N1	North America / United States / Wisconsin / Barron County	Mary Lea Killian (National Veterinary Services Laboratories - USDA / Diagnostic Virology Laboratory)
EPI_ISL_16171508	A/turkey/Wisconsin/22-013448-001-original/2022	A / H5N1	North America / United States / Wisconsin / Barron County	Mary Lea Killian (National Veterinary Services Laboratories - USDA / Diagnostic Virology Laboratory)
EPI_ISL_16171509	A/turkey/Wisconsin/22-013448-002-original/2022	A / H5N1	North America / United States / Wisconsin / Barron County	Mary Lea Killian (National Veterinary Services Laboratories - USDA / Diagnostic Virology Laboratory)
EPI_ISL_16171510	A/turkey/South Dakota/22-013454-001-original/2022	A / H5N1	North America / United States / South Dakota / Beadle County	Mary Lea Killian (National Veterinary Services Laboratories - USDA / Diagnostic Virology Laboratory)
EPI_ISL_16171511	A/turkey/South Dakota/22-013454-002-original/2022	A / H5N1	North America / United States / South Dakota / Beadle County	Mary Lea Killian (National Veterinary Services Laboratories - USDA / Diagnostic Virology Laboratory)
EPI_ISL_16171512	A/turkey/South Dakota/22-013547-001-original/2022	A / H5N1	North America / United States / South Dakota / Hamlin County	Mary Lea Killian (National Veterinary Services Laboratories - USDA / Diagnostic Virology Laboratory)
EPI_ISL_16171513	A/chicken/South Dakota/22-013554-001-original/2022	A / H5N1	North America / United States / South Dakota / Day County	Mary Lea Killian (National Veterinary Services Laboratories - USDA / Diagnostic Virology Laboratory)
EPI_ISL_16171514	A/duck/Minnesota/22-013605-001-original/2022	A / H5N1	North America / United States / Minnesota / Carver County	Mary Lea Killian (National Veterinary Services Laboratories - USDA / Diagnostic Virology Laboratory)
EPI_ISL_16171516	A/duck/Minnesota/22-013605-003-original/2022	A / H5N1	North America / United States / Minnesota / Carver County	Mary Lea Killian (National Veterinary Services Laboratories - USDA / Diagnostic Virology Laboratory)
EPI_ISL_16171517	A/turkey/Utah/22-013612-001-original/2022	A / H5N1	North America / United States / Utah / Cache County	Mary Lea Killian (National Veterinary Services Laboratories - USDA / Diagnostic Virology Laboratory)
EPI_ISL_16171518	A/chicken/Minnesota/22-013752-001-original/2022	A / H5N1	North America / United States / Minnesota / Anoka County	Mary Lea Killian (National Veterinary Services Laboratories - USDA / Diagnostic Virology Laboratory)
EPI_ISL_16171519	A/chicken/Washington/22-013763-002-original/2022	A / H5N1	North America / United States / Washington / Pacific County	Mary Lea Killian (National Veterinary Services Laboratories - USDA / Diagnostic Virology Laboratory)
EPI_ISL_16171520	A/chicken/Washington/22-013763-003-original/2022	A / H5N1	North America / United States / Washington / Pacific County	Mary Lea Killian (National Veterinary Services Laboratories - USDA / Diagnostic Virology Laboratory)
EPI_ISL_16171521	A/chicken/Wisconsin/22-013776-001-original/2022	A / H5N1	North America / United States / Wisconsin / Pierce County	Mary Lea Killian (National Veterinary Services Laboratories - USDA / Diagnostic Virology Laboratory)
EPI_ISL_16171522	A/goose/Oregon/22-013779-001-original/2022	A / H5N1	North America / United States / Oregon / Linn County	Mary Lea Killian (National Veterinary Services Laboratories - USDA / Diagnostic Virology Laboratory)
EPI_ISL_16171523	A/chicken/Montana/22-013942-001-original/2022	A / H5N1	North America / United States / Montana / Fergus County	Mary Lea Killian (National Veterinary Services Laboratories - USDA / Diagnostic Virology Laboratory)
EPI_ISL_16171524	A/chicken/Montana/22-013942-002-original/2022	A / H5N1	North America / United States / Montana / Fergus County	Mary Lea Killian (National Veterinary Services Laboratories - USDA / Diagnostic Virology Laboratory)
EPI_ISL_16196676	A/duck/Cyprus/750-2_22VIR12133-10/2022	A / H5N1	Europe / Cyprus	Ambra Pastori (Istituto Zooprofilattico Sperimentale Delle Venezie)
EPI_ISL_16196756	A/goose/Cyprus/750-3_22VIR12133-11/2022	A / H5N1	Europe / Cyprus	Ambra Pastori (Istituto Zooprofilattico Sperimentale Delle Venezie)
EPI_ISL_16196757	A/duck/Cyprus/752-2_22VIR12133-13/2022	A / H5N1	Europe / Cyprus	Ambra Pastori (Istituto Zooprofilattico Sperimentale Delle Venezie)
EPI_ISL_16196758	A/duck/Cyprus/745-1_22VIR12133-1/2022	A / H5N1	Europe / Cyprus	Ambra Pastori (Istituto Zooprofilattico Sperimentale Delle Venezie)
EPI_ISL_16196759	A/goose/Cyprus/746-1_22VIR12133-3/2022	A / H5N1	Europe / Cyprus	Ambra Pastori (Istituto Zooprofilattico Sperimentale Delle Venezie)
EPI_ISL_16196760	A/duck/Cyprus/749-1_22VIR12133-5/2022	A / H5N1	Europe / Cyprus	Ambra Pastori (Istituto Zooprofilattico Sperimentale Delle Venezie)
EPI_ISL_16196761	A/goose/Cyprus/749-3_22VIR12133-7/2022	A / H5N1	Europe / Cyprus	Ambra Pastori (Istituto Zooprofilattico Sperimentale Delle Venezie)
EPI_ISL_161972	A/duck/Jiangxi/95/2014	A / H5N6	Asia / China / Jiangxi Province	Guang Liu (Beijing Genomics Institute (BGI))
EPI_ISL_161973	A/environment/Shenzhen/25-24/2013	A / H5N6	Asia / China / Guangdong Province	Guang Liu (Beijing Genomics Institute (BGI))
EPI_ISL_16199040	A/chicken/Austria/2514-19_22VIR11382-1/2022	A / H5N1	Europe / Austria	Ambra Pastori (Istituto Zooprofilattico Sperimentale Delle Venezie)
EPI_ISL_16199509	A/chicken/Austria/2514-20_22VIR11382-2/2022	A / H5N1	Europe / Austria	Ambra Pastori (Istituto Zooprofilattico Sperimentale Delle Venezie)
EPI_ISL_16206845	A/goose/Luxembourg/22253283/2022	A / H5N1	Europe / Luxembourg / District de Diekirch	Chantal J. Snoeck (Luxembourg Institute of Health / Department of Infection and Immunity)
EPI_ISL_162300	A/duck/Zhejiang/W24/2013	A / H5N8	Asia / China	

EPI_ISL_162301	A/duck/Zhejiang/6D18/2013	A / H5N8	Asia / China	
EPI_ISL_16240161	A/domestic goose/Washington/22-014023-001-original/2022	A / H5N1	North America / United States / Washington / Spokane County	Mary Lea Killian (National Veterinary Services Laboratories - USDA / Diagnostic Virology Laboratory)
EPI_ISL_16240162	A/turkey/Wisconsin/22-014125-001-original/2022	A / H5N1	North America / United States / Wisconsin / Barron County	Mary Lea Killian (National Veterinary Services Laboratories - USDA / Diagnostic Virology Laboratory)
EPI_ISL_16240163	A/turkey/Wisconsin/22-014126-001-original/2022	A / H5N1	North America / United States / Wisconsin / Barron County	Mary Lea Killian (National Veterinary Services Laboratories - USDA / Diagnostic Virology Laboratory)
EPI_ISL_16240164	A/turkey/Wisconsin/22-014126-002-original/2022	A / H5N1	North America / United States / Wisconsin / Barron County	Mary Lea Killian (National Veterinary Services Laboratories - USDA / Diagnostic Virology Laboratory)
EPI_ISL_16240165	A/chicken/Nebraska/22-014140-001-original/2022	A / H5N1	North America / United States / Nebraska / Knox County	Mary Lea Killian (National Veterinary Services Laboratories - USDA / Diagnostic Virology Laboratory)
EPI_ISL_16240166	A/chicken/Nebraska/22-014140-002-original/2022	A / H5N1	North America / United States / Nebraska / Knox County	Mary Lea Killian (National Veterinary Services Laboratories - USDA / Diagnostic Virology Laboratory)
EPI_ISL_16240167	A/chicken/Nebraska/22-014141-002-original/2022	A / H5N1	North America / United States / Nebraska / Knox County	Mary Lea Killian (National Veterinary Services Laboratories - USDA / Diagnostic Virology Laboratory)
EPI_ISL_16240168	A/chicken/Nebraska/22-014141-003-original/2022	A / H5N1	North America / United States / Nebraska / Knox County	Mary Lea Killian (National Veterinary Services Laboratories - USDA / Diagnostic Virology Laboratory)
EPI_ISL_16240169	A/chicken/Idaho/22-014175-001-original/2022	A / H5N1	North America / United States / Idaho / Canyon County	Mary Lea Killian (National Veterinary Services Laboratories - USDA / Diagnostic Virology Laboratory)
EPI_ISL_16240220	A/chicken/Idaho/22-014175-003-original/2022	A / H5N1	North America / United States / Idaho / Canyon County	Mary Lea Killian (National Veterinary Services Laboratories - USDA / Diagnostic Virology Laboratory)
EPI_ISL_16240221	A/turkey/Michigan/22-014263-001-original/2022	A / H5N1	North America / United States / Michigan / Muskegon County	Mary Lea Killian (National Veterinary Services Laboratories - USDA / Diagnostic Virology Laboratory)
EPI_ISL_16240223	A/chicken/Idaho/22-014275-001-original/2022	A / H5N1	North America / United States / Idaho / Ada County	Mary Lea Killian (National Veterinary Services Laboratories - USDA / Diagnostic Virology Laboratory)
EPI_ISL_16240224	A/chicken/Idaho/22-014275-002-original/2022	A / H5N1	North America / United States / Idaho / Ada County	Mary Lea Killian (National Veterinary Services Laboratories - USDA / Diagnostic Virology Laboratory)
EPI_ISL_16240225	A/duck/Idaho/22-014275-003-original/2022	A / H5N1	North America / United States / Idaho / Ada County	Mary Lea Killian (National Veterinary Services Laboratories - USDA / Diagnostic Virology Laboratory)
EPI_ISL_16240227	A/chicken/Minnesota/22-014294-002-original/2022	A / H5N1	North America / United States / Minnesota / Crow Wing County	Mary Lea Killian (National Veterinary Services Laboratories - USDA / Diagnostic Virology Laboratory)
EPI_ISL_16240228	A/turkey/Minnesota/22-014294-003-original/2022	A / H5N1	North America / United States / Minnesota / Crow Wing County	Mary Lea Killian (National Veterinary Services Laboratories - USDA / Diagnostic Virology Laboratory)
EPI_ISL_16240229	A/turkey/Minnesota/22-014294-006-original/2022	A / H5N1	North America / United States / Minnesota / Crow Wing County	Mary Lea Killian (National Veterinary Services Laboratories - USDA / Diagnostic Virology Laboratory)
EPI_ISL_16240239	A/goose/Minnesota/22-014294-007-original/2022	A / H5N1	North America / United States / Minnesota / Crow Wing County	Mary Lea Killian (National Veterinary Services Laboratories - USDA / Diagnostic Virology Laboratory)
EPI_ISL_16240351	A/chicken/Minnesota/22-014295-001-original/2022	A / H5N1	North America / United States / Minnesota / Todd County	Mary Lea Killian (National Veterinary Services Laboratories - USDA / Diagnostic Virology Laboratory)
EPI_ISL_16240352	A/chicken/Minnesota/22-014295-002-original/2022	A / H5N1	North America / United States / Minnesota / Todd County	Mary Lea Killian (National Veterinary Services Laboratories - USDA / Diagnostic Virology Laboratory)
EPI_ISL_16240353	A/duck/Minnesota/22-014295-003-original/2022	A / H5N1	North America / United States / Minnesota / Todd County	Mary Lea Killian (National Veterinary Services Laboratories - USDA / Diagnostic Virology Laboratory)
EPI_ISL_16240361	A/turkey/Washington/22-014316-001-original/2022	A / H5N1	North America / United States / Washington / Pierce County	Mary Lea Killian (National Veterinary Services Laboratories - USDA / Diagnostic Virology Laboratory)
EPI_ISL_16240372	A/duck/Washington/22-014327-001-original/2022	A / H5N1	North America / United States / Washington / Pierce County	Mary Lea Killian (National Veterinary Services Laboratories - USDA / Diagnostic Virology Laboratory)
EPI_ISL_16240382	A/duck/Washington/22-014327-002-original/2022	A / H5N1	North America / United States / Washington / Pierce County	Mary Lea Killian (National Veterinary Services Laboratories - USDA / Diagnostic Virology Laboratory)
EPI_ISL_16240392	A/chicken/Washington/22-014327-003-original/2022	A / H5N1	North America / United States / Washington / Pierce County	Mary Lea Killian (National Veterinary Services Laboratories - USDA / Diagnostic Virology Laboratory)
EPI_ISL_16240404	A/duck/Pennsylvania/22-014337-007-original/2022	A / H5N1	North America / United States / Pennsylvania / Berks County	Mary Lea Killian (National Veterinary Services Laboratories - USDA / Diagnostic Virology Laboratory)
EPI_ISL_16240414	A/chicken/Pennsylvania/22-014338-001-original/2022	A / H5N1	North America / United States / Pennsylvania / Lancaster County	Mary Lea Killian (National Veterinary Services Laboratories - USDA / Diagnostic Virology Laboratory)
EPI_ISL_16240423	A/chicken/Pennsylvania/22-014338-002-original/2022	A / H5N1	North America / United States / Pennsylvania / Lancaster County	Mary Lea Killian (National Veterinary Services Laboratories - USDA / Diagnostic Virology Laboratory)
EPI_ISL_16240434	A/duck/Pennsylvania/22-014339-001-original/2022	A / H5N1	North America / United States / Pennsylvania / Berks County	Mary Lea Killian (National Veterinary Services Laboratories - USDA / Diagnostic Virology Laboratory)
EPI_ISL_16240445	A/duck/Pennsylvania/22-014339-002-original/2022	A / H5N1	North America / United States / Pennsylvania / Berks County	Mary Lea Killian (National Veterinary Services Laboratories - USDA / Diagnostic Virology Laboratory)
EPI_ISL_16240456	A/chicken/Idaho/22-014425-002-original/2022	A / H5N1	North America / United States / Idaho / Ada County	Mary Lea Killian (National Veterinary Services Laboratories - USDA / Diagnostic Virology Laboratory)
EPI_ISL_16240466	A/chicken/Washington/22-014449-001-original/2022	A / H5N1	North America / United States / Washington / Clallam County	Mary Lea Killian (National Veterinary Services Laboratories - USDA / Diagnostic Virology Laboratory)
EPI_ISL_16240476	A/chicken/Washington/22-014449-002-original/2022	A / H5N1	North America / United States / Washington / Clallam County	Mary Lea Killian (National Veterinary Services Laboratories - USDA / Diagnostic Virology Laboratory)
EPI_ISL_16240486	A/goose/Washington/22-014450-001-original/2022	A / H5N1	North America / United States / Washington / Clallam county	Mary Lea Killian (National Veterinary Services Laboratories - USDA / Diagnostic Virology Laboratory)
EPI_ISL_16240497	A/duck/Utah/22-014481-001-original/2022	A / H5N1	North America / United States / Utah / Cache County	Mary Lea Killian (National Veterinary Services Laboratories - USDA / Diagnostic Virology Laboratory)
EPI_ISL_16240508	A/guinea fowl/Utah/22-014481-002-original/2022	A / H5N1	North America / United States / Utah / Cache County	Mary Lea Killian (National Veterinary Services Laboratories - USDA / Diagnostic Virology Laboratory)
EPI_ISL_16240519	A/chicken/Minnesota/22-014503-001-original/2022	A / H5N1	North America / United States / Minnesota / Chisago County	Mary Lea Killian (National Veterinary Services Laboratories - USDA / Diagnostic Virology Laboratory)
EPI_ISL_16240529	A/chicken/Minnesota/22-014503-002-original/2022	A / H5N1	North America / United States / Minnesota / Chisago County	Mary Lea Killian (National Veterinary Services Laboratories - USDA / Diagnostic Virology Laboratory)
EPI_ISL_16240634	A/fox/Michigan/22-014536-001-original/2022	A / H5N1	North America / United States / Michigan / Macomb County	Mary Lea Killian (National Veterinary Services Laboratories - USDA / Diagnostic Virology Laboratory)
EPI_ISL_16240635	A/fox/Michigan/22-014536-002-original/2022	A / H5N1	North America / United States / Michigan / Macomb County	Mary Lea Killian (National Veterinary Services Laboratories - USDA / Diagnostic Virology Laboratory)
EPI_ISL_16240636	A/fox/Michigan/22-014536-007-original/2022	A / H5N1	North America / United States / Michigan / Saint Clair County	Mary Lea Killian (National Veterinary Services Laboratories - USDA / Diagnostic Virology Laboratory)
EPI_ISL_16240637	A/fox/Michigan/22-014536-008-original/2022	A / H5N1	North America / United States / Michigan / Saint Clair County	Mary Lea Killian (National Veterinary Services Laboratories - USDA / Diagnostic Virology Laboratory)
EPI_ISL_16240638	A/chicken/Minnesota/22-014663-001-original/2022	A / H5N1	North America / United States / Minnesota / Chisago County	Mary Lea Killian (National Veterinary Services Laboratories - USDA / Diagnostic Virology Laboratory)
EPI_ISL_16240639	A/chicken/Minnesota/22-014663-002-original/2022	A / H5N1	North America / United States / Minnesota / Chisago County	Mary Lea Killian (National Veterinary Services Laboratories - USDA / Diagnostic Virology Laboratory)
EPI_ISL_16240640	A/duck/Minnesota/22-014663-003-original/2022	A / H5N1	North America / United States / Minnesota / Chisago County	Mary Lea Killian (National Veterinary Services Laboratories - USDA / Diagnostic Virology Laboratory)
EPI_ISL_16240641	A/duck/Minnesota/22-014663-004-original/2022	A / H5N1	North America / United States / Minnesota / Chisago County	Mary Lea Killian (National Veterinary Services Laboratories - USDA / Diagnostic Virology Laboratory)
EPI_ISL_16240642	A/turkey/Idaho/22-014701-001-original/2022	A / H5N1	North America / United States / Idaho / Ada County	Mary Lea Killian (National Veterinary Services Laboratories - USDA / Diagnostic Virology Laboratory)
EPI_ISL_16240643	A/chicken/Idaho/22-014701-002-original/2022	A / H5N1	North America / United States / Idaho / Ada County	Mary Lea Killian (National Veterinary Services Laboratories - USDA / Diagnostic Virology Laboratory)
EPI_ISL_16240644	A/chicken/Minnesota/22-014734-001-original/2022	A / H5N1	North America / United States / Minnesota / Grant County	Mary Lea Killian (National Veterinary Services Laboratories - USDA / Diagnostic Virology Laboratory)
EPI_ISL_16240880	A/chicken/Minnesota/22-014734-002-original/2022	A / H5N1	North America / United States / Minnesota / Grant County	Mary Lea Killian (National Veterinary Services Laboratories - USDA / Diagnostic Virology Laboratory)
EPI_ISL_16240881	A/chicken/Washington/22-014739-001-original/2022	A / H5N1	North America / United States / Washington / Okanogan County	Mary Lea Killian (National Veterinary Services Laboratories - USDA / Diagnostic Virology Laboratory)
EPI_ISL_16240882	A/chicken/Washington/22-014744-001-original/2022	A / H5N1	North America / United States / Washington / Whatcom County	Mary Lea Killian (National Veterinary Services Laboratories - USDA / Diagnostic Virology Laboratory)
EPI_ISL_16240883	A/goose/Washington/22-014744-003-original/2022	A / H5N1	North America / United States / Washington / Whatcom County	Mary Lea Killian (National Veterinary Services Laboratories - USDA / Diagnostic Virology Laboratory)
EPI_ISL_16240884	A/goose/Washington/22-014744-004-original/2022	A / H5N1	North America / United States / Washington / Whatcom County	Mary Lea Killian (National Veterinary Services Laboratories - USDA / Diagnostic Virology Laboratory)
EPI_ISL_16240885	A/chicken/Wisconsin/22-014859-001-original/2022	A / H5N1	North America / United States / Wisconsin / Marinette County	Mary Lea Killian (National Veterinary Services Laboratories - USDA / Diagnostic Virology Laboratory)
EPI_ISL_16240886	A/chicken/Wyoming/22-014916-001-original/2022	A / H5N1	North America / United States / Wyoming / Sheridan County	Mary Lea Killian (National Veterinary Services Laboratories - USDA / Diagnostic Virology Laboratory)
EPI_ISL_16240887	A/chicken/Wyoming/22-014917-001-original/2022	A / H5N1	North America / United States / Wyoming / Sheridan County	Mary Lea Killian (National Veterinary Services Laboratories - USDA / Diagnostic Virology Laboratory)
EPI_ISL_16240888	A/chicken/Wyoming/22-014918-001-original/2022	A / H5N1	North America / United States / Wyoming / Lincoln County	Mary Lea Killian (National Veterinary Services Laboratories - USDA / Diagnostic Virology Laboratory)
EPI_ISL_16240889	A/chicken/Wyoming/22-014920-001-original/2022	A / H5N1	North America / United States / Wyoming / Lincoln County	Mary Lea Killian (National Veterinary Services Laboratories - USDA / Diagnostic Virology Laboratory)
EPI_ISL_16240985	A/guinea fowl/Minnesota/22-014734-003-original/2022	A / H5N1	North America / United States / Minnesota / Grant County	Mary Lea Killian (National Veterinary Services Laboratories - USDA / Diagnostic Virology Laboratory)
EPI_ISL_16243202	A/chicken/Idaho/22-014942-001-original/2022	A / H5N1	North America / United States / Idaho / Canyon County	Mary Lea Killian (National Veterinary Services Laboratories - USDA / Diagnostic Virology Laboratory)
EPI_ISL_16243210	A/goose/Idaho/22-014944-001-original/2022	A / H5N1	North America / United States / Idaho / Ada County	Mary Lea Killian (National Veterinary Services Laboratories - USDA / Diagnostic Virology Laboratory)
EPI_ISL_16243221	A/turkey/Wisconsin/22-014948-001-original/2022	A / H5N1	North America / United States / Wisconsin / Barron County	Mary Lea Killian (National Veterinary Services Laboratories - USDA / Diagnostic Virology Laboratory)
EPI_ISL_16243231	A/chicken/Pennsylvania/22-015036-001-original/2022	A / H5N1	North America / United States / Pennsylvania / Berks County	Mary Lea Killian (National Veterinary Services Laboratories - USDA / Diagnostic Virology Laboratory)
EPI_ISL_16243241	A/chicken/Pennsylvania/22-015036-002-original/2022	A / H5N1	North America / United States / Pennsylvania / Berks County	Mary Lea Killian (National Veterinary Services Laboratories - USDA / Diagnostic Virology Laboratory)
EPI_ISL_16243253	A/fox/New York/22-015063-001-original/2022	A / H5N1	North America / United States / New York / Saint Lawrence County	Mary Lea Killian (National Veterinary Services Laboratories - USDA / Diagnostic Virology Laboratory)
EPI_ISL_16243264	A/fox/New York/22-015064-001-original/2022	A / H5N1	North America / United States / New York / Erie County	Mary Lea Killian (National Veterinary Services Laboratories - USDA / Diagnostic Virology Laboratory)
EPI_ISL_16243273	A/fox/New York/22-015065-001-original/2022	A / H5N1	North America / United States / New York / Erie County	Mary Lea Killian (National Veterinary Services Laboratories - USDA / Diagnostic Virology Laboratory)
EPI_ISL_16243294	A/chicken/Wisconsin/22-015068-003-original/2022	A / H5N1	North America / United States / Wisconsin / Dunn County	Mary Lea Killian (National Veterinary Services Laboratories - USDA / Diagnostic Virology Laboratory)
EPI_ISL_16243306	A/chicken/Wisconsin/22-015068-005-original/2022	A / H5N1	North America / United States / Wisconsin / Dunn County	Mary Lea Killian (National Veterinary Services Laboratories - USDA / Diagnostic Virology Laboratory)
EPI_ISL_16243316	A/chicken/Idaho/22-015073-001-original/2022	A / H5N1	North America / United States / Idaho / Ada County	Mary Lea Killian (National Veterinary Services Laboratories - USDA / Diagnostic Virology Laboratory)

EPI_ISL_16243326	A/chicken/North Dakota/22-015199-001-original/2022	A / H5N1	North America / United States / North Dakota / Burke County	Mary Lea Killian (National Veterinary Services Laboratories - USDA / Diagnostic Virology Laboratory)
EPI_ISL_16243588	A/chicken/North Dakota/22-015199-002-original/2022	A / H5N1	North America / United States / North Dakota / Burke County	Mary Lea Killian (National Veterinary Services Laboratories - USDA / Diagnostic Virology Laboratory)
EPI_ISL_16243598	A/chicken/Pennsylvania/22-015201-001-original/2022	A / H5N1	North America / United States / Pennsylvania / Berks County	Mary Lea Killian (National Veterinary Services Laboratories - USDA / Diagnostic Virology Laboratory)
EPI_ISL_16243610	A/chicken/Pennsylvania/22-015201-002-original/202	A / H5N1	North America / United States / Pennsylvania / Berks County	Mary Lea Killian (National Veterinary Services Laboratories - USDA / Diagnostic Virology Laboratory)
EPI_ISL_16243619	A/chicken/Washington/22-015210-001-original/2022	A / H5N1	North America / United States / Washington / Thurston County	Mary Lea Killian (National Veterinary Services Laboratories - USDA / Diagnostic Virology Laboratory)
EPI_ISL_16243630	A/chicken/Oregon/22-015228-001-original/2022	A / H5N1	North America / United States / Oregon / Lane County	Mary Lea Killian (National Veterinary Services Laboratories - USDA / Diagnostic Virology Laboratory)
EPI_ISL_16243641	A/chicken/Illinois/22-015233-005-original/2022	A / H5N1	North America / United States / Illinois / Boone County	Mary Lea Killian (National Veterinary Services Laboratories - USDA / Diagnostic Virology Laboratory)
EPI_ISL_16243651	A/chicken/Illinois/22-015233-006-original/2022	A / H5N1	North America / United States / Illinois / Boone County	Mary Lea Killian (National Veterinary Services Laboratories - USDA / Diagnostic Virology Laboratory)
EPI_ISL_16243660	A/chicken/Utah/22-015245-001-original/2022	A / H5N1	North America / United States / Utah / Salt Lake County	Mary Lea Killian (National Veterinary Services Laboratories - USDA / Diagnostic Virology Laboratory)
EPI_ISL_16243667	A/goose/Utah/22-015245-002-original/2022	A / H5N1	North America / United States / Utah / Salt Lake County	Mary Lea Killian (National Veterinary Services Laboratories - USDA / Diagnostic Virology Laboratory)
EPI_ISL_16243872	A/duck/New Jersey/22-015259-001-original/2022	A / H5N1	North America / United States / New Jersey / Monmouth County	Mary Lea Killian (National Veterinary Services Laboratories - USDA / Diagnostic Virology Laboratory)
EPI_ISL_16243883	A/duck/New Jersey/22-015259-002-original/2022	A / H5N1	North America / United States / New Jersey / Monmouth County	Mary Lea Killian (National Veterinary Services Laboratories - USDA / Diagnostic Virology Laboratory)
EPI_ISL_16243894	A/chicken/Idaho/22-015271-002-original/2022	A / H5N1	North America / United States / Idaho / Ada County	Mary Lea Killian (National Veterinary Services Laboratories - USDA / Diagnostic Virology Laboratory)
EPI_ISL_16243915	A/duck/Idaho/22-015275-001-original/2022	A / H5N1	North America / United States / Idaho / Canyon County	Mary Lea Killian (National Veterinary Services Laboratories - USDA / Diagnostic Virology Laboratory)
EPI_ISL_16243926	A/duck/Idaho/22-015275-002-original/2022	A / H5N1	North America / United States / Idaho / Canyon County	Mary Lea Killian (National Veterinary Services Laboratories - USDA / Diagnostic Virology Laboratory)
EPI_ISL_16243948	A/Pekin duck/Pennsylvania/22-015337-001-original/2022	A / H5N1	North America / United States / Pennsylvania / Berks County	Mary Lea Killian (National Veterinary Services Laboratories - USDA / Diagnostic Virology Laboratory)
EPI_ISL_16243958	A/Pekin duck/Pennsylvania/22-015337-003-original/2022	A / H5N1	North America / United States / Pennsylvania / Berks County	Mary Lea Killian (National Veterinary Services Laboratories - USDA / Diagnostic Virology Laboratory)
EPI_ISL_16243970	A/fox/Iowa/22-015357-002-original/2022	A / H5N1	North America / United States / Iowa / Cedar County	Mary Lea Killian (National Veterinary Services Laboratories - USDA / Diagnostic Virology Laboratory)
EPI_ISL_16243981	A/chicken/Minnesota/22-015398-001-original/2022	A / H5N1	North America / United States / Minnesota / Clay County	Mary Lea Killian (National Veterinary Services Laboratories - USDA / Diagnostic Virology Laboratory)
EPI_ISL_16243992	A/chicken/Minnesota/22-015398-002-original/2022	A / H5N1	North America / United States / Minnesota / Clay County	Mary Lea Killian (National Veterinary Services Laboratories - USDA / Diagnostic Virology Laboratory)
EPI_ISL_16244002	A/turkey/Minnesota/22-015400-001-original/2022	A / H5N1	North America / United States / Minnesota / Kandiyohi County	Mary Lea Killian (National Veterinary Services Laboratories - USDA / Diagnostic Virology Laboratory)
EPI_ISL_16244003	A/turkey/Minnesota/22-015400-002-original/2022	A / H5N1	North America / United States / Minnesota / Kandiyohi County	Mary Lea Killian (National Veterinary Services Laboratories - USDA / Diagnostic Virology Laboratory)
EPI_ISL_16244004	A/chicken/Idaho/22-015407-001-original/2022	A / H5N1	North America / United States / Idaho / Canyon County	Mary Lea Killian (National Veterinary Services Laboratories - USDA / Diagnostic Virology Laboratory)
EPI_ISL_16244005	A/chicken/Idaho/22-015408-001-original/2022	A / H5N1	North America / United States / Idaho / Ada County	Mary Lea Killian (National Veterinary Services Laboratories - USDA / Diagnostic Virology Laboratory)
EPI_ISL_16244006	A/chicken/Idaho/22-015409-001-original/2022	A / H5N1	North America / United States / Idaho / Canyon County	Mary Lea Killian (National Veterinary Services Laboratories - USDA / Diagnostic Virology Laboratory)
EPI_ISL_16244007	A/chicken/Idaho/22-015410-001-original/2022	A / H5N1	North America / United States / Idaho / Canyon County	Mary Lea Killian (National Veterinary Services Laboratories - USDA / Diagnostic Virology Laboratory)
EPI_ISL_16244008	A/duck/Idaho/22-015410-002-original/2022	A / H5N1	North America / United States / Idaho / Canyon County	Mary Lea Killian (National Veterinary Services Laboratories - USDA / Diagnostic Virology Laboratory)
EPI_ISL_16244009	A/Pekin duck/Pennsylvania/22-015427-002-original/2022	A / H5N1	North America / United States / Pennsylvania / Berks County	Mary Lea Killian (National Veterinary Services Laboratories - USDA / Diagnostic Virology Laboratory)
EPI_ISL_16244010	A/chicken/Indiana/22-015386-002-original/2022	A / H5N1	North America / United States / Indiana / Allen County	Mary Lea Killian (National Veterinary Services Laboratories - USDA / Diagnostic Virology Laboratory)
EPI_ISL_16244011	A/duck/Colorado/22-015599-001-original/2022	A / H5N1	North America / United States / Colorado / Jefferson County	Mary Lea Killian (National Veterinary Services Laboratories - USDA / Diagnostic Virology Laboratory)
EPI_ISL_16244012	A/chicken/Idaho/22-015654-001-original/2022	A / H5N1	North America / United States / Idaho / Ada County	Mary Lea Killian (National Veterinary Services Laboratories - USDA / Diagnostic Virology Laboratory)
EPI_ISL_16244013	A/chicken/Idaho/22-015655-001-original/2022	A / H5N1	North America / United States / Idaho / Canyon County	Mary Lea Killian (National Veterinary Services Laboratories - USDA / Diagnostic Virology Laboratory)
EPI_ISL_16244014	A/chicken/Idaho/22-015656-001-original/2022	A / H5N1	North America / United States / Idaho / Canyon County	Mary Lea Killian (National Veterinary Services Laboratories - USDA / Diagnostic Virology Laboratory)
EPI_ISL_16244015	A/chicken/Idaho/22-015657-001-original/2022	A / H5N1	North America / United States / Idaho / Ada County	Mary Lea Killian (National Veterinary Services Laboratories - USDA / Diagnostic Virology Laboratory)
EPI_ISL_16244016	A/chicken/Idaho/22-015657-002-original/2022	A / H5N1	North America / United States / Idaho / Ada County	Mary Lea Killian (National Veterinary Services Laboratories - USDA / Diagnostic Virology Laboratory)
EPI_ISL_16244017	A/chicken/South Dakota/22-015737-001-original/2022	A / H5N1	North America / United States / South Dakota / Codington County	Mary Lea Killian (National Veterinary Services Laboratories - USDA / Diagnostic Virology Laboratory)
EPI_ISL_16244018	A/chicken/Idaho/22-015744-001-original/2022	A / H5N1	North America / United States / Idaho / Ada County	Mary Lea Killian (National Veterinary Services Laboratories - USDA / Diagnostic Virology Laboratory)
EPI_ISL_16244019	A/chicken/Minnesota/22-015759-001-original/2022	A / H5N1	North America / United States / Minnesota / Polk County	Mary Lea Killian (National Veterinary Services Laboratories - USDA / Diagnostic Virology Laboratory)
EPI_ISL_16244020	A/chicken/Minnesota/22-015759-002-original/2022	A / H5N1	North America / United States / Minnesota / Polk County	Mary Lea Killian (National Veterinary Services Laboratories - USDA / Diagnostic Virology Laboratory)
EPI_ISL_16244021	A/turkey/Minnesota/22-015759-003-original/2022	A / H5N1	North America / United States / Minnesota / Polk County	Mary Lea Killian (National Veterinary Services Laboratories - USDA / Diagnostic Virology Laboratory)
EPI_ISL_16244022	A/chicken/Wyoming/22-015810-001-original/2022	A / H5N1	North America / United States / Wyoming / Campbell County	Mary Lea Killian (National Veterinary Services Laboratories - USDA / Diagnostic Virology Laboratory)
EPI_ISL_16244023	A/chicken/Wyoming/22-015810-002-original/2022	A / H5N1	North America / United States / Wyoming / Campbell County	Mary Lea Killian (National Veterinary Services Laboratories - USDA / Diagnostic Virology Laboratory)
EPI_ISL_16244024	A/turkey/Minnesota/22-015885-001-original/2022	A / H5N1	North America / United States / Minnesota / Dakota County	Mary Lea Killian (National Veterinary Services Laboratories - USDA / Diagnostic Virology Laboratory)
EPI_ISL_16244025	A/turkey/Minnesota/22-015885-002-original/2022	A / H5N1	North America / United States / Minnesota / Dakota County	Mary Lea Killian (National Veterinary Services Laboratories - USDA / Diagnostic Virology Laboratory)
EPI_ISL_16244026	A/chicken/Wisconsin/22-015940-001-original/2022	A / H5N1	North America / United States / Wisconsin / Bayfield County	Mary Lea Killian (National Veterinary Services Laboratories - USDA / Diagnostic Virology Laboratory)
EPI_ISL_16244028	A/Pekin duck/Pennsylvania/22-015943-001-original/2022	A / H5N1	North America / United States / Pennsylvania / Berks County	Mary Lea Killian (National Veterinary Services Laboratories - USDA / Diagnostic Virology Laboratory)
EPI_ISL_16244029	A/duck/Washington/22-016021-001-original/2022	A / H5N1	North America / United States / Washington / King County	Mary Lea Killian (National Veterinary Services Laboratories - USDA / Diagnostic Virology Laboratory)
EPI_ISL_16244030	A/duck/Washington/22-016021-002-original/2022	A / H5N1	North America / United States / Washington / King County	Mary Lea Killian (National Veterinary Services Laboratories - USDA / Diagnostic Virology Laboratory)
EPI_ISL_162467	A/duck/Guangdong/GD01/2014	A / H5N6	Asia / China	
EPI_ISL_16249274	A/Peru/PIU-001/2022	A / H5N1	South America / Peru / Departamento de Piura	Carlos Patricio Padilla Rojas (Instituto Nacional de Salud Peru / Laboratorio de Biotecnolgia y Biologia Molecular)
EPI_ISL_16249681	A/Peru/LAM-002/2022	A / H5N1	South America / Peru / Departamento de Lambayeque	Carlos Patricio Padilla Rojas (Instituto Nacional de Salud Peru / Laboratorio de Biotecnolgia y Biologia Molecular)
EPI_ISL_16249730	A/Peru/LIM-003/2022	A / H5N1	South America / Peru / Departamento de Lima	Carlos Patricio Padilla Rojas (Instituto Nacional de Salud Peru / Laboratorio de Biotecnolgia y Biologia Molecular)
EPI_ISL_16253341	A/Mallard Duck/Netherlands/31/2022	A / H5N1	Europe / Netherlands / Eendenkooi, Wieringen (Slijkerman)	Sanne Thewessen (Erasmus Medical Center / Viroscience)
EPI_ISL_16253342	A/Eurasian Wigeon/Netherlands/6/2022	A / H5N2	Europe / Netherlands / Eendenkooi Stolwijk	Sanne Thewessen (Erasmus Medical Center / Viroscience)
EPI_ISL_16271814	A/goose/Alabama/22-038970-002-original/2022	A / H5N1	North America / United States / Alabama / Lawrence County	Mary Lea Killian (National Veterinary Services Laboratories - USDA / Diagnostic Virology Laboratory)
EPI_ISL_16271815	A/chicken/Alabama/22-038970-003-original/2022	A / H5N1	North America / United States / Alabama / Lawrence County	Mary Lea Killian (National Veterinary Services Laboratories - USDA / Diagnostic Virology Laboratory)
EPI_ISL_16271816	A/duck/Alabama/22-038970-004-original/2022	A / H5N1	North America / United States / Alabama / Lawrence County	Mary Lea Killian (National Veterinary Services Laboratories - USDA / Diagnostic Virology Laboratory)
EPI_ISL_16271817	A/chicken/Missouri/22-037522-001-original/2022	A / H5N1	North America / United States / Missouri / Webster County	Mary Lea Killian (National Veterinary Services Laboratories - USDA / Diagnostic Virology Laboratory)
EPI_ISL_16271818	A/turkey/South Dakota/22-037527-001-original/2022	A / H5N1	North America / United States / South Dakota / Beadle County	Mary Lea Killian (National Veterinary Services Laboratories - USDA / Diagnostic Virology Laboratory)
EPI_ISL_16271819	A/turkey/South Dakota/22-037357-001-original/2022	A / H5N1	North America / United States / South Dakota / Edmunds County	Mary Lea Killian (National Veterinary Services Laboratories - USDA / Diagnostic Virology Laboratory)
EPI_ISL_16271820	A/domestic goose/Kentucky/22-037359-001-original/2022	A / H5N1	North America / United States / Kentucky / Jefferson County	Mary Lea Killian (National Veterinary Services Laboratories - USDA / Diagnostic Virology Laboratory)
EPI_ISL_16271821	A/domestic goose/Maine/22-037360-001-original/2022	A / H5N1	North America / United States / Maine / Washington County	Mary Lea Killian (National Veterinary Services Laboratories - USDA / Diagnostic Virology Laboratory)
EPI_ISL_16271822	A/chicken/Maine/22-037360-002-original/2022	A / H5N1	North America / United States / Maine / Washington County	Mary Lea Killian (National Veterinary Services Laboratories - USDA / Diagnostic Virology Laboratory)
EPI_ISL_16271823	A/chicken/Nebraska/22-038152-001-original/2022	A / H5N1	North America / United States / Nebraska / Dixon County	Mary Lea Killian (National Veterinary Services Laboratories - USDA / Diagnostic Virology Laboratory)
EPI_ISL_16271824	A/chicken/Nebraska/22-038149-001-original/2022	A / H5N1	North America / United States / Nebraska / Dixon County	Mary Lea Killian (National Veterinary Services Laboratories - USDA / Diagnostic Virology Laboratory)
EPI_ISL_16271825	A/chicken/Iowa/22-035154-002-original/2022	A / H5N1	North America / United States / Iowa / Wright County	Mary Lea Killian (National Veterinary Services Laboratories - USDA / Diagnostic Virology Laboratory)
EPI_ISL_16271826	A/turkey/Missouri/22-038012-001-original/2022	A / H5N1	North America / United States / Missouri / Webster County	Mary Lea Killian (National Veterinary Services Laboratories - USDA / Diagnostic Virology Laboratory)
EPI_ISL_16271827	A/rhea/Arizona/22-037270-002-original/2022	A / H5N1	North America / United States / Arizona / Maricopa County	Mary Lea Killian (National Veterinary Services Laboratories - USDA / Diagnostic Virology Laboratory)
EPI_ISL_16271828	A/domestic duck/North Carolina/22-037942-001-original/2022	A / H5N1	North America / United States / North Carolina / Durham County	Mary Lea Killian (National Veterinary Services Laboratories - USDA / Diagnostic Virology Laboratory)
EPI_ISL_16271829	A/chicken/Alaska/22-037940-001-original/2022	A / H5N1	North America / United States / Alaska / Matanuska-Susitna Borough	Mary Lea Killian (National Veterinary Services Laboratories - USDA / Diagnostic Virology Laboratory)
EPI_ISL_16271830	A/chicken/South Dakota/22-037931-001-original/2022	A / H5N1	North America / United States / South Dakota / Turner County	Mary Lea Killian (National Veterinary Services Laboratories - USDA / Diagnostic Virology Laboratory)
EPI_ISL_16271831	A/turkey/South Dakota/22-037933-001-original/2022	A / H5N1	North America / United States / South Dakota / Beadle County	Mary Lea Killian (National Veterinary Services Laboratories - USDA / Diagnostic Virology Laboratory)

EPI_ISL_16271832	A/domestic goose/Kentucky/22-037359-002-original/2022	A / H5N1	North America / United States / Kentucky / Jefferson County	Mary Lea Killian (National Veterinary Services Laboratories - USDA / Diagnostic Virology Laboratory)
EPI_ISL_16271833	A/turkey/South Dakota/22-037934-001-original/2022	A / H5N1	North America / United States / South Dakota / Beadle County	Mary Lea Killian (National Veterinary Services Laboratories - USDA / Diagnostic Virology Laboratory)
EPI_ISL_16271834	A/turkey/South Dakota/22-037930-001-original/2022	A / H5N1	North America / United States / South Dakota / Spink County	Mary Lea Killian (National Veterinary Services Laboratories - USDA / Diagnostic Virology Laboratory)
EPI_ISL_16271835	A/striped skunk/Idaho/22-037165-002-original/2022	A / H5N1	North America / United States / Idaho / Jefferson County	Mary Lea Killian (National Veterinary Services Laboratories - USDA / Diagnostic Virology Laboratory)
EPI_ISL_16271836	A/chicken/Tennessee/22-037736-001-original/2022	A / H5N1	North America / United States / Tennessee / Davidson County	Mary Lea Killian (National Veterinary Services Laboratories - USDA / Diagnostic Virology Laboratory)
EPI_ISL_16271837	A/chicken/Florida/22-037745-001-original/2022	A / H5N1	North America / United States / Florida / Broward County	Mary Lea Killian (National Veterinary Services Laboratories - USDA / Diagnostic Virology Laboratory)
EPI_ISL_16271838	A/turkey/Minnesota/22-037734-001-original/2022	A / H5N1	North America / United States / Minnesota / Todd County	Mary Lea Killian (National Veterinary Services Laboratories - USDA / Diagnostic Virology Laboratory)
EPI_ISL_16271839	A/chicken/South Dakota/22-037701-001-original/2022	A / H5N1	North America / United States / South Dakota / Brule County	Mary Lea Killian (National Veterinary Services Laboratories - USDA / Diagnostic Virology Laboratory)
EPI_ISL_16271840	A/chicken/Oregon/22-037712-001-original/2022	A / H5N1	North America / United States / Oregon / Columbia County	Mary Lea Killian (National Veterinary Services Laboratories - USDA / Diagnostic Virology Laboratory)
EPI_ISL_16271841	A/turkey/South Dakota/22-037700-001-original/2022	A / H5N1	North America / United States / South Dakota / Faulk County	Mary Lea Killian (National Veterinary Services Laboratories - USDA / Diagnostic Virology Laboratory)
EPI_ISL_16271842	A/chicken/Ohio/22-035584-001-original/2022	A / H5N1	North America / United States / Ohio / Butler County	Mary Lea Killian (National Veterinary Services Laboratories - USDA / Diagnostic Virology Laboratory)
EPI_ISL_16271843	A/Chinese ringneck pheasant/Wisconsin/22-035394-001-origir	A / H5N1	North America / United States / Wisconsin / Jefferson County	Mary Lea Killian (National Veterinary Services Laboratories - USDA / Diagnostic Virology Laboratory)
EPI_ISL_16271844	A/Chinese ringneck pheasant/Wisconsin/22-035394-002-origir	A / H5N1	North America / United States / Wisconsin / Jefferson County	Mary Lea Killian (National Veterinary Services Laboratories - USDA / Diagnostic Virology Laboratory)
EPI_ISL_16271845	A/chicken/New York/22-035475-001-original/2022	A / H5N1	North America / United States / New York / Sullivan County	Mary Lea Killian (National Veterinary Services Laboratories - USDA / Diagnostic Virology Laboratory)
EPI_ISL_16271846	A/chicken/New York/22-035476-001-original/2022	A / H5N1	North America / United States / New York / Sullivan County	Mary Lea Killian (National Veterinary Services Laboratories - USDA / Diagnostic Virology Laboratory)
EPI_ISL_16271847	A/chicken/Wyoming/22-035578-001-original/2022	A / H5N1	North America / United States / Wyoming / Converse County	Mary Lea Killian (National Veterinary Services Laboratories - USDA / Diagnostic Virology Laboratory)
EPI_ISL_16271848	A/black bear/Alaska/22-036396-001-original/2022	A / H5N1	North America / United States / Alaska / Juneau City and Borough	Mary Lea Killian (National Veterinary Services Laboratories - USDA / Diagnostic Virology Laboratory)
EPI_ISL_16271849	A/chicken/North Carolina/22-037124-002-original/2022	A / H5N1	North America / United States / North Carolina / Union County	Mary Lea Killian (National Veterinary Services Laboratories - USDA / Diagnostic Virology Laboratory)
EPI_ISL_16271850	A/chicken/Florida/22-036965-001-original/2022	A / H5N1	North America / United States / Florida / Broward County	Mary Lea Killian (National Veterinary Services Laboratories - USDA / Diagnostic Virology Laboratory)
EPI_ISL_16271851	A/chicken/Florida/22-036767-001-original/2022	A / H5N1	North America / United States / Florida / Hillsborough County	Mary Lea Killian (National Veterinary Services Laboratories - USDA / Diagnostic Virology Laboratory)
EPI_ISL_16271852	A/turkey/Florida/22-036768-001-original/2022	A / H5N1	North America / United States / Florida / Seminole County	Mary Lea Killian (National Veterinary Services Laboratories - USDA / Diagnostic Virology Laboratory)
EPI_ISL_16271853	A/pheasant/Michigan/22-036108-001-original/2022	A / H5N1	North America / United States / Michigan / Lapeer County	Mary Lea Killian (National Veterinary Services Laboratories - USDA / Diagnostic Virology Laboratory)
EPI_ISL_16271854	A/chicken/North Dakota/22-036126-001-original/2022	A / H5N1	North America / United States / North Dakota / Mountrail County	Mary Lea Killian (National Veterinary Services Laboratories - USDA / Diagnostic Virology Laboratory)
EPI_ISL_16271855	A/domestic goose/Tennessee/22-036130-001-original/2022	A / H5N1	North America / United States / Tennessee / Lincoln County	Mary Lea Killian (National Veterinary Services Laboratories - USDA / Diagnostic Virology Laboratory)
EPI_ISL_16271856	A/Muscovy duck/New York/22-036304-003-original/2022	A / H5N1	North America / United States / New York / Queens County	Mary Lea Killian (National Veterinary Services Laboratories - USDA / Diagnostic Virology Laboratory)
EPI_ISL_16271857	A/chicken/New York/22-036304-007-original/2022	A / H5N1	North America / United States / New York / Queens County	Mary Lea Killian (National Veterinary Services Laboratories - USDA / Diagnostic Virology Laboratory)
EPI_ISL_16271858	A/chicken/Tennessee/22-036544-002-original/2022	A / H5N1	North America / United States / Tennessee / Bledsoe County	Mary Lea Killian (National Veterinary Services Laboratories - USDA / Diagnostic Virology Laboratory)
EPI_ISL_16271859	A/turkey/Minnesota/22-036547-001-original/2022	A / H5N1	North America / United States / Minnesota / Otter Tail County	Mary Lea Killian (National Veterinary Services Laboratories - USDA / Diagnostic Virology Laboratory)
EPI_ISL_16271860	A/chicken/Florida/22-036555-001-original/2022	A / H5N1	North America / United States / Florida / Duval County	Mary Lea Killian (National Veterinary Services Laboratories - USDA / Diagnostic Virology Laboratory)
EPI_ISL_16271861	A/Pekin duck/California/22-036722-001-original/2022	A / H5N1	North America / United States / California / Fresno County	Mary Lea Killian (National Veterinary Services Laboratories - USDA / Diagnostic Virology Laboratory)
EPI_ISL_16271862	A/chicken/Wyoming/22-035579-001-original/2022	A / H5N1	North America / United States / Wyoming / Converse County	Mary Lea Killian (National Veterinary Services Laboratories - USDA / Diagnostic Virology Laboratory)
EPI_ISL_16271863	A/chicken/Wyoming/22-035580-001-original/2022	A / H5N1	North America / United States / Wyoming / Converse County	Mary Lea Killian (National Veterinary Services Laboratories - USDA / Diagnostic Virology Laboratory)
EPI_ISL_16271864	A/chicken/Wyoming/22-035581-001-original/2022	A / H5N1	North America / United States / Wyoming / Converse County	Mary Lea Killian (National Veterinary Services Laboratories - USDA / Diagnostic Virology Laboratory)
EPI_ISL_16271865	A/domestic goose/Kentucky/22-037028-001-original/2022	A / H5N1	North America / United States / Kentucky / Jefferson County	Mary Lea Killian (National Veterinary Services Laboratories - USDA / Diagnostic Virology Laboratory)
EPI_ISL_16297060	A/chicken/Oregon/22-030743-001-original/2022	A / H5N1	North America / United States / Oregon / Douglas County	Mary Lea Killian (National Veterinary Services Laboratories - USDA / Diagnostic Virology Laboratory)
EPI_ISL_16297061	A/chicken/North Dakota/22-030784-001-original/2022	A / H5N1	North America / United States / North Dakota / Nelson County	Mary Lea Killian (National Veterinary Services Laboratories - USDA / Diagnostic Virology Laboratory)
EPI_ISL_16297062	A/pheasant/South Dakota/22-034797-001-original/2022	A / H5N1	North America / United States / South Dakota / Gregory County	Mary Lea Killian (National Veterinary Services Laboratories - USDA / Diagnostic Virology Laboratory)
EPI_ISL_16297063	A/chicken/Montana/22-034813-003-original/2022	A / H5N1	North America / United States / Montana / Granite County	Mary Lea Killian (National Veterinary Services Laboratories - USDA / Diagnostic Virology Laboratory)
EPI_ISL_16297064	A/chicken/Oregon/22-034833-001-original/2022	A / H5N1	North America / United States / Oregon / Deschutes County	Mary Lea Killian (National Veterinary Services Laboratories - USDA / Diagnostic Virology Laboratory)
EPI_ISL_16297065	A/turkey/Minnesota/22-034849-001-original/2022	A / H5N1	North America / United States / Minnesota / Stearns County	Mary Lea Killian (National Veterinary Services Laboratories - USDA / Diagnostic Virology Laboratory)
EPI_ISL_16297066	A/skunk/Minnesota/22-034925-001-original/2022	A / H5N1	North America / United States / Minnesota / Itasca County	Mary Lea Killian (National Veterinary Services Laboratories - USDA / Diagnostic Virology Laboratory)
EPI_ISL_16297067	A/Muscovy duck/California/22-034335-001-original/2022	A / H5N1	North America / United States / California / Fresno County	Mary Lea Killian (National Veterinary Services Laboratories - USDA / Diagnostic Virology Laboratory)
EPI_ISL_16297068	A/chicken/Pennsylvania/22-034995-001-original/2022	A / H5N1	North America / United States / Pennsylvania / Allegheny County	Mary Lea Killian (National Veterinary Services Laboratories - USDA / Diagnostic Virology Laboratory)
EPI_ISL_16297069	A/chicken/New Jersey/22-035003-009-original/2022	A / H5N1	North America / United States / New Jersey / Bergen County	Mary Lea Killian (National Veterinary Services Laboratories - USDA / Diagnostic Virology Laboratory)
EPI_ISL_16297070	A/chicken/Arizona/22-035006-002-original/2022	A / H5N1	North America / United States / Arizona / Yavapai County	Mary Lea Killian (National Veterinary Services Laboratories - USDA / Diagnostic Virology Laboratory)
EPI_ISL_16297071	A/chicken/North Dakota/22-035070-001-original/2022	A / H5N1	North America / United States / North Dakota / Bottineau County	Mary Lea Killian (National Veterinary Services Laboratories - USDA / Diagnostic Virology Laboratory)
EPI_ISL_16297072	A/turkey/North Dakota/22-035070-002-original/2022	A / H5N1	North America / United States / North Dakota / Bottineau County	Mary Lea Killian (National Veterinary Services Laboratories - USDA / Diagnostic Virology Laboratory)
EPI_ISL_16297073	A/chicken/California/22-035144-001-original/2022	A / H5N1	North America / United States / California / San Diego County	Mary Lea Killian (National Veterinary Services Laboratories - USDA / Diagnostic Virology Laboratory)
EPI_ISL_16297074	A/domestic goose/California/22-035144-003-original/2022	A / H5N1	North America / United States / California / San Diego County	Mary Lea Killian (National Veterinary Services Laboratories - USDA / Diagnostic Virology Laboratory)
EPI_ISL_16297075	A/chicken/South Carolina/22-035153-001-original/2022	A / H5N1	North America / United States / South Carolina / Beaufort County	Mary Lea Killian (National Veterinary Services Laboratories - USDA / Diagnostic Virology Laboratory)
EPI_ISL_16297076	A/guinea fowl/South Carolina/22-035153-002-original/2022	A / H5N1	North America / United States / South Carolina / Beaufort County	Mary Lea Killian (National Veterinary Services Laboratories - USDA / Diagnostic Virology Laboratory)
EPI_ISL_16297077	A/turkey/Pennsylvania/22-035178-001-original/2022	A / H5N1	North America / United States / Pennsylvania / Lehigh County	Mary Lea Killian (National Veterinary Services Laboratories - USDA / Diagnostic Virology Laboratory)
EPI_ISL_16297078	A/chicken/Oregon/22-035197-001-original/2022	A / H5N1	North America / United States / Oregon / Yamhill County	Mary Lea Killian (National Veterinary Services Laboratories - USDA / Diagnostic Virology Laboratory)
EPI_ISL_16297079	A/chicken/Massachussetts/22-035225-001-original/2022	A / H5N1	North America / United States / Massachussetts	Mary Lea Killian (National Veterinary Services Laboratories - USDA / Diagnostic Virology Laboratory)
EPI_ISL_16297080	A/chicken/Massachussetts/22-035234-001-original/2022	A / H5N1	North America / United States / Massachussetts	Mary Lea Killian (National Veterinary Services Laboratories - USDA / Diagnostic Virology Laboratory)
EPI_ISL_16297081	A/chicken/Arizona/22-035241-003-original/2022	A / H5N1	North America / United States / Arizona / Yavapai County	Mary Lea Killian (National Veterinary Services Laboratories - USDA / Diagnostic Virology Laboratory)
EPI_ISL_16297082	A/chicken/Iowa/22-035700-001-original/2022	A / H5N1	North America / United States / Iowa / Louisa County	Mary Lea Killian (National Veterinary Services Laboratories - USDA / Diagnostic Virology Laboratory)
EPI_ISL_16297083	A/domestic duck/Iowa/22-035700-002-original/2022	A / H5N1	North America / United States / Iowa / Louisa County	Mary Lea Killian (National Veterinary Services Laboratories - USDA / Diagnostic Virology Laboratory)
EPI_ISL_16297084	A/turkey/Pennsylvania/22-035743-001-original/2022	A / H5N1	North America / United States / Pennsylvania / Lehigh County	Mary Lea Killian (National Veterinary Services Laboratories - USDA / Diagnostic Virology Laboratory)
EPI_ISL_16297085	A/chicken/Wisconsin/22-034812-002-original/2022	A / H5N1	North America / United States / Wisconsin / Waukesha County	Mary Lea Killian (National Veterinary Services Laboratories - USDA / Diagnostic Virology Laboratory)
EPI_ISL_16297086	A/wild turkey/New York/22-034864-002-original/202	A / H5N1	North America / United States / New York / Dutchess County	Mary Lea Killian (National Veterinary Services Laboratories - USDA / Diagnostic Virology Laboratory)
EPI_ISL_16297117	A/mallard/Texas/22-035744-001-original/2022	A / H5N1	North America / United States / Texas / Tarrant County	Mary Lea Killian (National Veterinary Services Laboratories - USDA / Diagnostic Virology Laboratory)
EPI_ISL_16297118	A/chicken/Washington/22-035749-001-original/2022	A / H5N1	North America / United States / Washington / Snohomish County	Mary Lea Killian (National Veterinary Services Laboratories - USDA / Diagnostic Virology Laboratory)
EPI_ISL_16297119	A/turkey/Pennsylvania/22-036105-007-original/2022	A / H5N1	North America / United States / Pennsylvania / Lehigh County	Mary Lea Killian (National Veterinary Services Laboratories - USDA / Diagnostic Virology Laboratory)
EPI_ISL_16297120	A/turkey/Pennsylvania/22-036115-004-original/2022	A / H5N1	North America / United States / Pennsylvania / Lehigh County	Mary Lea Killian (National Veterinary Services Laboratories - USDA / Diagnostic Virology Laboratory)
EPI_ISL_16297121	A/chicken/Nebraska/22-037884-001-original/2022	A / H5N1	North America / United States / Nebraska / Dixon County	Mary Lea Killian (National Veterinary Services Laboratories - USDA / Diagnostic Virology Laboratory)
EPI_ISL_16297122	A/skunk/Wisconsin/22-037029-001-original/2022	A / H5N1	North America / United States / Wisconsin / Douglas County	Mary Lea Killian (National Veterinary Services Laboratories - USDA / Diagnostic Virology Laboratory)
EPI_ISL_16297123	A/chicken/Iowa/22-035569-001-original/2022	A / H5N1	North America / United States / Iowa / Wright County	Mary Lea Killian (National Veterinary Services Laboratories - USDA / Diagnostic Virology Laboratory)
EPI_ISL_16297124	A/chicken/Iowa/22-035567-003-original/2022	A / H5N1	North America / United States / Iowa / Wright County	Mary Lea Killian (National Veterinary Services Laboratories - USDA / Diagnostic Virology Laboratory)
EPI_ISL_16297125	A/turkey/Pennsylvania/22-035424-001-original/2022	A / H5N1	North America / United States / Pennsylvania / Lehigh County	Mary Lea Killian (National Veterinary Services Laboratories - USDA / Diagnostic Virology Laboratory)
EPI_ISL_16297126	A/chicken/Iowa/22-034682-001-original/2022	A / H5N1	North America / United States / Iowa / Wright County	Mary Lea Killian (National Veterinary Services Laboratories - USDA / Diagnostic Virology Laboratory)
EPI_ISL_16297127	A/turkey/Minnesota/22-034666-001-original/2022	A / H5N1	North America / United States / Minnesota / Swift County	Mary Lea Killian (National Veterinary Services Laboratories - USDA / Diagnostic Virology Laboratory)

EPI_ISL_16297128	A/chicken/Colorado/22-034082-001-original/2022	A / H5N1	North America / United States / Colorado / La Plata County	Mary Lea Killian (National Veterinary Services Laboratories - USDA / Diagnostic Virology Laboratory)
EPI_ISL_16297129	A/turkey/Minnesota/22-034260-001-original/2022	A / H5N1	North America / United States / Minnesota / Le Sueur County	Mary Lea Killian (National Veterinary Services Laboratories - USDA / Diagnostic Virology Laboratory)
EPI_ISL_16297130	A/chicken/Florida/22-034076-001-original/2022	A / H5N1	North America / United States / Florida / Pasco County	Mary Lea Killian (National Veterinary Services Laboratories - USDA / Diagnostic Virology Laboratory)
EPI_ISL_16297131	A/chicken/Florida/22-033918-001-original/2022	A / H5N1	North America / United States / Florida / Indian River County	Mary Lea Killian (National Veterinary Services Laboratories - USDA / Diagnostic Virology Laboratory)
EPI_ISL_16297132	A/domestic duck/Florida/22-033918-002-original/2022	A / H5N1	North America / United States / Florida / Indian River County	Mary Lea Killian (National Veterinary Services Laboratories - USDA / Diagnostic Virology Laboratory)
EPI_ISL_16297133	A/chicken/Florida/22-033923-001-original/2022	A / H5N1	North America / United States / Florida / Lake County	Mary Lea Killian (National Veterinary Services Laboratories - USDA / Diagnostic Virology Laboratory)
EPI_ISL_16297134	A/chicken/Florida/22-033924-001-original/2022	A / H5N1	North America / United States / Florida / Indian River County	Mary Lea Killian (National Veterinary Services Laboratories - USDA / Diagnostic Virology Laboratory)
EPI_ISL_16297135	A/turkey/Minnesota/22-033929-001-original/2022	A / H5N1	North America / United States / Minnesota / Swift County	Mary Lea Killian (National Veterinary Services Laboratories - USDA / Diagnostic Virology Laboratory)
EPI_ISL_16297136	A/turkey/Utah/22-033909-001-original/2022	A / H5N1	North America / United States / Utah / Sanpete County	Mary Lea Killian (National Veterinary Services Laboratories - USDA / Diagnostic Virology Laboratory)
EPI_ISL_16297137	A/chicken/South Dakota/22-033852-001-original/2022	A / H5N1	North America / United States / South Dakota / Grant County	Mary Lea Killian (National Veterinary Services Laboratories - USDA / Diagnostic Virology Laboratory)
EPI_ISL_16297138	A/African goose/New Jersey/22-033679-001-original/2022	A / H5N1	North America / United States / New Jersey / Ocean County	Mary Lea Killian (National Veterinary Services Laboratories - USDA / Diagnostic Virology Laboratory)
EPI_ISL_16297139	A/chicken/Virginia/22-033680-002-original/2022	A / H5N1	North America / United States / Virginia / Southampton County	Mary Lea Killian (National Veterinary Services Laboratories - USDA / Diagnostic Virology Laboratory)
EPI_ISL_16297140	A/chicken/Oklahoma/22-033558-001-original/2022	A / H5N1	North America / United States / Oklahoma / Cleveland County	Mary Lea Killian (National Veterinary Services Laboratories - USDA / Diagnostic Virology Laboratory)
EPI_ISL_16297141	A/peafowl/Florida/22-033621-001-original/2022	A / H5N1	North America / United States / Florida / Indian River County	Mary Lea Killian (National Veterinary Services Laboratories - USDA / Diagnostic Virology Laboratory)
EPI_ISL_16297142	A/chicken/North Carolina/22-033524-001-original/2022	A / H5N1	North America / United States / North Carolina / Wake County	Mary Lea Killian (National Veterinary Services Laboratories - USDA / Diagnostic Virology Laboratory)
EPI_ISL_16297326	A/chicken/North Carolina/22-033522-001-original/2022	A / H5N1	North America / United States / North Carolina / Wake County	Mary Lea Killian (National Veterinary Services Laboratories - USDA / Diagnostic Virology Laboratory)
EPI_ISL_16297327	A/chicken/Florida/22-035222-003-original/2022	A / H5N1	North America / United States / Florida / Broward County	Mary Lea Killian (National Veterinary Services Laboratories - USDA / Diagnostic Virology Laboratory)
EPI_ISL_16297328	A/chicken/Colorado/22-033394-001-original/2022	A / H5N1	North America / United States / Colorado / Jefferson County	Mary Lea Killian (National Veterinary Services Laboratories - USDA / Diagnostic Virology Laboratory)
EPI_ISL_16297329	A/chicken/Idaho/22-033409-001-original/2022	A / H5N1	North America / United States / Idaho / Valley County	Mary Lea Killian (National Veterinary Services Laboratories - USDA / Diagnostic Virology Laboratory)
EPI_ISL_16297330	A/chicken/Oklahoma/22-033387-001-original/2022	A / H5N1	North America / United States / Oklahoma / Creek County	Mary Lea Killian (National Veterinary Services Laboratories - USDA / Diagnostic Virology Laboratory)
EPI_ISL_16297331	A/chicken/Florida/22-033389-001-original/2022	A / H5N1	North America / United States / Florida / Okeechobee County	Mary Lea Killian (National Veterinary Services Laboratories - USDA / Diagnostic Virology Laboratory)
EPI_ISL_16297332	A/goose/Louisiana/22-033391-001-original/2022	A / H5N1	North America / United States / Louisiana / Ouachita Parish	Mary Lea Killian (National Veterinary Services Laboratories - USDA / Diagnostic Virology Laboratory)
EPI_ISL_16297333	A/chicken/Florida/22-033392-001-original/2022	A / H5N1	North America / United States / Florida / Indian River County	Mary Lea Killian (National Veterinary Services Laboratories - USDA / Diagnostic Virology Laboratory)
EPI_ISL_16297334	A/domestic duck/South Dakota/22-033350-004-original/2022	A / H5N1	North America / United States / South Dakota / Spink County	Mary Lea Killian (National Veterinary Services Laboratories - USDA / Diagnostic Virology Laboratory)
EPI_ISL_16297335	A/turkey/South Dakota/22-033350-005-original/2022	A / H5N1	North America / United States / South Dakota / Spink County	Mary Lea Killian (National Veterinary Services Laboratories - USDA / Diagnostic Virology Laboratory)
EPI_ISL_16297336	A/chicken/Iowa/22-033362-001-original/2022	A / H5N1	North America / United States / Iowa / Dallas County	Mary Lea Killian (National Veterinary Services Laboratories - USDA / Diagnostic Virology Laboratory)
EPI_ISL_16297337	A/chicken/South Dakota/22-033350-001-original/2022	A / H5N1	North America / United States / South Dakota / Spink County	Mary Lea Killian (National Veterinary Services Laboratories - USDA / Diagnostic Virology Laboratory)
EPI_ISL_16297338	A/chicken/Virginia/22-033345-001-original/2022	A / H5N1	North America / United States / Virginia / Virginia County	Mary Lea Killian (National Veterinary Services Laboratories - USDA / Diagnostic Virology Laboratory)
EPI_ISL_16297339	A/chicken/Rhode Island/22-033346-001-original/2022	A / H5N1	North America / United States / Rhode Island / Newport County	Mary Lea Killian (National Veterinary Services Laboratories - USDA / Diagnostic Virology Laboratory)
EPI_ISL_16297340	A/chicken/Montana/22-033348-001-original/2022	A / H5N1	North America / United States / Montana / Hill County	Mary Lea Killian (National Veterinary Services Laboratories - USDA / Diagnostic Virology Laboratory)
EPI_ISL_16297341	A/chicken/guinea fowl/Kansas/22-033226-001-original/2022	A / H5N1	North America / United States / Kansas / Shawnee County	Mary Lea Killian (National Veterinary Services Laboratories - USDA / Diagnostic Virology Laboratory)
EPI_ISL_16297342	A/chicken/Texas/22-033221-001-original/2022	A / H5N1	North America / United States / Texas / Rockwall County	Mary Lea Killian (National Veterinary Services Laboratories - USDA / Diagnostic Virology Laboratory)
EPI_ISL_16297343	A/domestic duck/Florida/22-032962-001-original/2022	A / H5N1	North America / United States / Florida / Saint Lucie County	Mary Lea Killian (National Veterinary Services Laboratories - USDA / Diagnostic Virology Laboratory)
EPI_ISL_16297344	A/chicken/Florida/22-032963-001-original/2022	A / H5N1	North America / United States / Florida / Osceola County	Mary Lea Killian (National Veterinary Services Laboratories - USDA / Diagnostic Virology Laboratory)
EPI_ISL_16297345	A/chicken/Florida/22-032965-001-original/2022	A / H5N1	North America / United States / Florida / Indian River County	Mary Lea Killian (National Veterinary Services Laboratories - USDA / Diagnostic Virology Laboratory)
EPI_ISL_16297346	A/domestic goose/South Dakota/22-033171-001-original/2022	A / H5N1	North America / United States / South Dakota / Roberts County	Mary Lea Killian (National Veterinary Services Laboratories - USDA / Diagnostic Virology Laboratory)
EPI_ISL_16297347	A/chicken/North Dakota/22-032950-001-original/2022	A / H5N1	North America / United States / North Dakota / Traill County	Mary Lea Killian (National Veterinary Services Laboratories - USDA / Diagnostic Virology Laboratory)
EPI_ISL_16297348	A/guinea fowl/North Dakota/22-032950-002-original/2022	A / H5N1	North America / United States / North Dakota / Traill County	Mary Lea Killian (National Veterinary Services Laboratories - USDA / Diagnostic Virology Laboratory)
EPI_ISL_16297349	A/turkey/North Dakota/22-032950-003-original/2022	A / H5N1	North America / United States / North Dakota / Traill County	Mary Lea Killian (National Veterinary Services Laboratories - USDA / Diagnostic Virology Laboratory)
EPI_ISL_16297350	A/turkey/Utah/22-032951-001-original/2022	A / H5N1	North America / United States / Utah / Sanpete County	Mary Lea Killian (National Veterinary Services Laboratories - USDA / Diagnostic Virology Laboratory)
EPI_ISL_16297351	A/pheasant/Nebraska/22-032910-001-original/2022	A / H5N1	North America / United States / Nebraska / York County	Mary Lea Killian (National Veterinary Services Laboratories - USDA / Diagnostic Virology Laboratory)
EPI_ISL_16297352	A/turkey/Pennsylvania/22-032809-001-original/2022	A / H5N1	North America / United States / Pennsylvania / Adams County	Mary Lea Killian (National Veterinary Services Laboratories - USDA / Diagnostic Virology Laboratory)
EPI_ISL_16297353	A/chicken/Nevada/22-032799-001-original/2022	A / H5N1	North America / United States / Nevada / Nye County	Mary Lea Killian (National Veterinary Services Laboratories - USDA / Diagnostic Virology Laboratory)
EPI_ISL_16297354	A/turkey/Alaska/22-032677-004-original/2022	A / H5N1	North America / United States / Alaska / Matanuska-Susitna Borough	Mary Lea Killian (National Veterinary Services Laboratories - USDA / Diagnostic Virology Laboratory)
EPI_ISL_16297355	A/chicken/Colorado/22-032675-001-original/2022	A / H5N1	North America / United States / Colorado / La Plata County	Mary Lea Killian (National Veterinary Services Laboratories - USDA / Diagnostic Virology Laboratory)
EPI_ISL_16297356	A/turkey/Utah/22-032657-001-original/2022	A / H5N1	North America / United States / Utah / Sanpete County	Mary Lea Killian (National Veterinary Services Laboratories - USDA / Diagnostic Virology Laboratory)
EPI_ISL_16297357	A/chicken/Florida/22-032661-001-original/2022	A / H5N1	North America / United States / Florida / Boward County	Mary Lea Killian (National Veterinary Services Laboratories - USDA / Diagnostic Virology Laboratory)
EPI_ISL_16297358	A/chicken/Colorado/22-032670-001-original/2022	A / H5N1	North America / United States / Colorado / Larimer County	Mary Lea Killian (National Veterinary Services Laboratories - USDA / Diagnostic Virology Laboratory)
EPI_ISL_16297359	A/guinea fowl/Oklahoma/22-032674-001-original/2022	A / H5N1	North America / United States / Oklahoma / Tulsa County	Mary Lea Killian (National Veterinary Services Laboratories - USDA / Diagnostic Virology Laboratory)
EPI_ISL_16297360	A/chicken/Arkansas/22-032566-001-original/2022	A / H5N1	North America / United States / Arkansas / Pope County	Mary Lea Killian (National Veterinary Services Laboratories - USDA / Diagnostic Virology Laboratory)
EPI_ISL_16297361	A/goose/Oklahoma/22-032413-001-original/2022	A / H5N1	North America / United States / Oklahoma / Payne County	Mary Lea Killian (National Veterinary Services Laboratories - USDA / Diagnostic Virology Laboratory)
EPI_ISL_16297362	A/swan/Oklahoma/22-032414-001-original/2022	A / H5N1	North America / United States / Oklahoma / Oklahoma County	Mary Lea Killian (National Veterinary Services Laboratories - USDA / Diagnostic Virology Laboratory)
EPI_ISL_16297363	A/domestic duck/New Jersey/22-032412-001-original/2022	A / H5N1	North America / United States / New Jersey / Warren County	Mary Lea Killian (National Veterinary Services Laboratories - USDA / Diagnostic Virology Laboratory)
EPI_ISL_16297364	A/turkey/Utah/22-032405-001-original/2022	A / H5N1	North America / United States / Utah / Sanpete County	Mary Lea Killian (National Veterinary Services Laboratories - USDA / Diagnostic Virology Laboratory)
EPI_ISL_16297365	A/chicken/Colorado/22-032233-001-original/2022	A / H5N1	North America / United States / Colorado / Weld County	Mary Lea Killian (National Veterinary Services Laboratories - USDA / Diagnostic Virology Laboratory)
EPI_ISL_16297366	A/rhea/Pennsylvania/22-032402-001-original/2022	A / H5N1	North America / United States / Pennsylvania / Dauphin County	Mary Lea Killian (National Veterinary Services Laboratories - USDA / Diagnostic Virology Laboratory)
EPI_ISL_16297367	A/cape teal/Texas/22-032200-001-original/2022	A / H5N1	North America / United States / Texas / El Paso County	Mary Lea Killian (National Veterinary Services Laboratories - USDA / Diagnostic Virology Laboratory)
EPI_ISL_16297368	A/chicken/Wyoming/22-032216-001-original/2022	A / H5N1	North America / United States / Wyoming / Fremont County	Mary Lea Killian (National Veterinary Services Laboratories - USDA / Diagnostic Virology Laboratory)
EPI_ISL_16297369	A/chicken/South Dakota/22-032160-001-original/2022	A / H5N1	North America / United States / South Dakota / Hamlin County	Mary Lea Killian (National Veterinary Services Laboratories - USDA / Diagnostic Virology Laboratory)
EPI_ISL_16297370	A/turkey/California/22-032159-001-original/2022	A / H5N1	North America / United States / California / Stanislaus County	Mary Lea Killian (National Veterinary Services Laboratories - USDA / Diagnostic Virology Laboratory)
EPI_ISL_16297371	A/chicken/Kentucky/22-031959-001-original/2022	A / H5N1	North America / United States / Kentucky / Logan County	Mary Lea Killian (National Veterinary Services Laboratories - USDA / Diagnostic Virology Laboratory)
EPI_ISL_16297372	A/Egyptian goose/Florida/22-031886-001-original/2022	A / H5N1	North America / United States / Florida / Martin County	Mary Lea Killian (National Veterinary Services Laboratories - USDA / Diagnostic Virology Laboratory)
EPI_ISL_16297373	A/chicken/Florida/22-031886-002-original/2022	A / H5N1	North America / United States / Florida / Martin County	Mary Lea Killian (National Veterinary Services Laboratories - USDA / Diagnostic Virology Laboratory)
EPI_ISL_16297374	A/swan/Oklahoma/22-031912-001-original/2022	A / H5N1	North America / United States / Oklahoma / Oklahoma County	Mary Lea Killian (National Veterinary Services Laboratories - USDA / Diagnostic Virology Laboratory)
EPI_ISL_16297375	A/turkey/Michigan/22-031751-001-original/2022	A / H5N1	North America / United States / Michigan / Lapeer County	Mary Lea Killian (National Veterinary Services Laboratories - USDA / Diagnostic Virology Laboratory)
EPI_ISL_16297376	A/chicken/Florida/22-031859-001-original/2022	A / H5N1	North America / United States / Florida / Broward County	Mary Lea Killian (National Veterinary Services Laboratories - USDA / Diagnostic Virology Laboratory)
EPI_ISL_16297377	A/turkey/Utah/22-031727-001-original/2022	A / H5N1	North America / United States / Utah / Sanpete County	Mary Lea Killian (National Veterinary Services Laboratories - USDA / Diagnostic Virology Laboratory)
EPI_ISL_16297378	A/chicken/Arkansas/22-031676-001-original/2022	A / H5N1	North America / United States / Arkansas / Madison County	Mary Lea Killian (National Veterinary Services Laboratories - USDA / Diagnostic Virology Laboratory)
EPI_ISL_16297379	A/chicken/Arkansas/22-031667-003-original/2022	A / H5N1	North America / United States / Arkansas / Madison County	Mary Lea Killian (National Veterinary Services Laboratories - USDA / Diagnostic Virology Laboratory)
EPI_ISL_16297380	A/turkey/Utah/22-033401-002-original/2022	A / H5N1	North America / United States / Utah / Sanpete County	Mary Lea Killian (National Veterinary Services Laboratories - USDA / Diagnostic Virology Laboratory)
EPI_ISL_16297381	A/chicken/Michigan/22-032218-001-original/2022	A / H5N1	North America / United States / Michigan / Genesee County	Mary Lea Killian (National Veterinary Services Laboratories - USDA / Diagnostic Virology Laboratory)
EPI_ISL_16297382	A/turkey/Utah/22-031721-004-original/2022	A / H5N1	North America / United States / Utah / Sanpete County	Mary Lea Killian (National Veterinary Services Laboratories - USDA / Diagnostic Virology Laboratory)

EPI_ISL_163493	A/Sichuan/26221/2014	A / H5N6	Asia / China / Sichuan Province	Lei Yang (WHO Chinese National Influenza Center / Virology Institute, Chinese CDC)
EPI_ISL_16388458	A/whooper swan/Akita/0511B002/2022	A / H5N1	Asia / Japan / Akita / Odate city	Norikazu Isoda (Graduate School of Veterinary Medicine, Hokkaido University / Laboratory of Microbiology)
EPI_ISL_16395206	A/cat/France/22P026544/2022	A / H5N1	Europe / France / Region Pays de la Loire	Francois-Xavier Briand (ANSES Agence Nationale De Securite Sanitaire De L'alimentation / Laboratoire de Ploufragan-Plouzané)
EPI_ISL_16466440	A/Changsha/1/2022	A / H5N6	Asia / China / Hunan Province / Changsha city	Zheng Huang (Changsha Disease Prevention and Control Center)
EPI_ISL_16524187	A/Common gull/Netherlands/1/2023	A / H5N1	Europe / Netherlands / Hippolytushoef, Mulders Eendenkooi	Sanne Thewessen (Erasmus Medical Center / Viroscience)
EPI_ISL_16524203	A/Black-headed gull/Netherlands/1/2023	A / H5N1	Europe / Netherlands / Groningen, Pioenpark	Sanne Thewessen (Erasmus Medical Center / Viroscience)
EPI_ISL_16524213	A/European herring gull/Netherlands/1/2023	A / H5N1	Europe / Netherlands / Den Oever, Waddenhaven	Sanne Thewessen (Erasmus Medical Center / Viroscience)
EPI_ISL_16524230	A/Black-headed gull/Netherlands/2/2023	A / H5N1	Europe / Netherlands / Den Oever, Waddenhaven	Sanne Thewessen (Erasmus Medical Center / Viroscience)
EPI_ISL_16524243	A/European herring gull/Netherlands/2/2023	A / H5N1	Europe / Netherlands / Den Oever, Waddenhaven	Sanne Thewessen (Erasmus Medical Center / Viroscience)
EPI_ISL_16524245	A/European herring gull/Netherlands/3/2023	A / H5N1	Europe / Netherlands / Den Oever, Waddenhaven	Sanne Thewessen (Erasmus Medical Center / Viroscience)
EPI_ISL_16524246	A/Caspian gull/Netherlands/1/2023	A / H5N1	Europe / Netherlands / Den Oever, Waddenhaven	Sanne Thewessen (Erasmus Medical Center / Viroscience)
EPI_ISL_16524247	A/Peregrine falcon/Netherlands/1/2023	A / H5N1	Europe / Netherlands / Wieingerwerf, Sluitgatweg	Sanne Thewessen (Erasmus Medical Center / Viroscience)
EPI_ISL_16524248	A/European herring gull/Netherlands/4/2023	A / H5N1	Europe / Netherlands / Den Oever, Waddenhaven	Sanne Thewessen (Erasmus Medical Center / Viroscience)
EPI_ISL_16547552	A/Mustela_furo/Belgium/14312-0001/2022	A / H5N1	Europe / Belgium / Provincie Limburg / Riemst	Steven Van Borm (Sciensano, Department of Animal Infectious Diseases / Animal Infectious Diseases)
EPI_ISL_16547666	A/Mustela_furo/Belgium/14312-0002/2022	A / H5N1	Europe / Belgium / Provincie Limburg / Riemst	Steven Van Borm (Sciensano, Department of Animal Infectious Diseases / Animal Infectious Diseases)
EPI_ISL_16547851	A/Gallus_gallus/Belgium/14313-001/2022	A / H5N1	Europe / Belgium / Provincie Limburg / Riemst	Steven Van Borm (Sciensano, Department of Animal Infectious Diseases / Animal Infectious Diseases)
EPI_ISL_16548347	A/Pelecanus_conspicillatus/Belgium/12905-0001/2022	A / H5N1	Europe / Belgium / Province de Hainaut / Cambron-Casteau	Steven Van Borm (Sciensano, Department of Animal Infectious Diseases / Animal Infectious Diseases)
EPI_ISL_16548356	A/Phasianus_colchicus/Belgium/13027-0001/2022	A / H5N1	Europe / Belgium / Province de Hainaut / Soignies	Steven Van Borm (Sciensano, Department of Animal Infectious Diseases / Animal Infectious Diseases)
EPI_ISL_16548429	A/Gallus_gallus/Belgium/13078-0001/2022	A / H5N1	Europe / Belgium / Provincie Antwerpen / Turnhout	Steven Van Borm (Sciensano, Department of Animal Infectious Diseases / Animal Infectious Diseases)
EPI_ISL_16548600	A/Gallus_gallus/Belgium/12485-0001/2022	A / H5N1	Europe / Belgium / Provincie West-Vlaanderen / Eke	Steven Van Borm (Sciensano, Department of Animal Infectious Diseases / Animal Infectious Diseases)
EPI_ISL_16548638	A/Phasianus_colchicus/Belgium/11704-0003/2022	A / H5N1	Europe / Belgium / Province de Liege / Clavier	Steven Van Borm (Sciensano, Department of Animal Infectious Diseases / Animal Infectious Diseases)
EPI_ISL_16549157	A/Gallus_gallus/Belgium/13357-0001/2022	A / H5N1	Europe / Belgium / Provincie Vlaams-Brabant / Lennik	Steven Van Borm (Sciensano, Department of Animal Infectious Diseases / Animal Infectious Diseases)
EPI_ISL_16549245	A/Otus_scops/Belgium/13463-0002/2022	A / H5N1	Europe / Belgium / Province de Liege / Lontzen	Steven Van Borm (Sciensano, Department of Animal Infectious Diseases / Animal Infectious Diseases)
EPI_ISL_16549349	A/Phasianus_colchicus/Belgium/10993-0003/2022	A / H5N1	Europe / Belgium / Province de Liege / Sainte-Fontaine	Steven Van Borm (Sciensano, Department of Animal Infectious Diseases / Animal Infectious Diseases)
EPI_ISL_16549379	A/Phasianus_colchicus/Belgium/12352-0002/2022	A / H5N1	Europe / Belgium / Province de Liege / Clavier	Steven Van Borm (Sciensano, Department of Animal Infectious Diseases / Animal Infectious Diseases)
EPI_ISL_16549380	A/Vulpes_vulpes/Belgium/14775-0001/2022	A / H5N1	Europe / Belgium / Provincie Vlaams-Brabant / Vilvoorde	Steven Van Borm (Sciensano, Department of Animal Infectious Diseases / Animal Infectious Diseases)
EPI_ISL_16555202	A/turkey/Minnesota/22-010654-002-original/2022	A / H5N1	North America / United States / Minnesota / Kandiyohi County	Mary Lea Killian (National Veterinary Services Laboratories - USDA / Diagnostic Virology Laboratory)
EPI_ISL_16555203	A/neotropic cormorant/Arizona/245467/2022	A / H5N1	North America / United States / Arizona / Maricopa County	Mary Lea Killian (National Veterinary Services Laboratories - USDA / Diagnostic Virology Laboratory)
EPI_ISL_16555204	A/eared grebe/North Dakota/245625/2022	A / H5N1	North America / United States / North Dakota / Stutsman County	Mary Lea Killian (National Veterinary Services Laboratories - USDA / Diagnostic Virology Laboratory)
EPI_ISL_16555205	A/Canada goose/California/246038/2022	A / H5N1	North America / United States / California / Colusa County	Mary Lea Killian (National Veterinary Services Laboratories - USDA / Diagnostic Virology Laboratory)
EPI_ISL_1665247	A/chicken/Italy/21VIR1151-2/2021	A / H5N8	Europe / Italy / Pordenone	Bianca Zecchin (Istituto Zooprofilattico Sperimentale Delle Venezie)
EPI_ISL_1665248	A/chicken/Italy/21VIR1293-9/2021	A / H5N8	Europe / Italy / Padova	Bianca Zecchin (Istituto Zooprofilattico Sperimentale Delle Venezie)
EPI_ISL_1665249	A/chicken/Romania/10101_21VIR2044-1/2021	A / H5N5	Europe / Romania / Judetul Timis	Bianca Zecchin (Istituto Zooprofilattico Sperimentale Delle Venezie)
EPI_ISL_1665250	A/chicken/Slovakia/Pah10_21VIR1086-5/2021	A / H5N5	Europe / Slovakia / Dobrohost	Bianca Zecchin (Istituto Zooprofilattico Sperimentale Delle Venezie)
EPI_ISL_1665251	A/common_teal/Italy/20VIR7439-191/2020	A / H5N8	Europe / Italy / Rovigo	Bianca Zecchin (Istituto Zooprofilattico Sperimentale Delle Venezie)
EPI_ISL_1665252	A/duck/Italy/21VIR1293-15/2021	A / H5N8	Europe / Italy / Padova	Bianca Zecchin (Istituto Zooprofilattico Sperimentale Delle Venezie)
EPI_ISL_1665254	A/duck/Romania/10206_21VIR849-4/2021	A / H5N8	Europe / Romania / Judetul Ilfov	Bianca Zecchin (Istituto Zooprofilattico Sperimentale Delle Venezie)
EPI_ISL_1665255	A/guinea_fowl/Italy/21VIR1293-20/2021	A / H5N8	Europe / Italy / Padova	Bianca Zecchin (Istituto Zooprofilattico Sperimentale Delle Venezie)
EPI_ISL_1665256	A/muscovy_duck/Slovakia/Pah1_21VIR1086-1/2021	A / H5N8	Europe / Slovakia / Kosice	Bianca Zecchin (Istituto Zooprofilattico Sperimentale Delle Venezie)
EPI_ISL_1665257	A/mute_swan/Austria/21011165_21VIR1085-2/2021	A / H5N8	Europe / Austria / Tulln	Bianca Zecchin (Istituto Zooprofilattico Sperimentale Delle Venezie)
EPI_ISL_1665258	A/mute_swan/Austria/21013162_21VIR1085-5/2021	A / H5N5	Europe / Austria / Leibnitz	Bianca Zecchin (Istituto Zooprofilattico Sperimentale Delle Venezie)
EPI_ISL_1665259	A/mute_swan/Austria/21014124_21VIR1085-8/2021	A / H5N8	Europe / Austria / Vienna	Bianca Zecchin (Istituto Zooprofilattico Sperimentale Delle Venezie)
EPI_ISL_1665260	A/mute_swan/Norway/FU5_21VIR850-3/2021	A / H5N8	Europe / Norway	Bianca Zecchin (Istituto Zooprofilattico Sperimentale Delle Venezie)
EPI_ISL_1665261	A/mute_swan/Slovakia/Pah15_21VIR1086-3/2021	A / H5N5	Europe / Slovakia / Kalinkovo	Bianca Zecchin (Istituto Zooprofilattico Sperimentale Delle Venezie)
EPI_ISL_1665262	A/mute_swan/Slovakia/Pah6_21VIR1086-2/2021	A / H5N5	Europe / Slovakia / Petrzalka	Bianca Zecchin (Istituto Zooprofilattico Sperimentale Delle Venezie)
EPI_ISL_1665263	A/mute_swan/Slovenia/1639-20_21VIR959-1/2020	A / H5N8	Europe / Slovenia	Bianca Zecchin (Istituto Zooprofilattico Sperimentale Delle Venezie)
EPI_ISL_1665264	A/mute_swan/Slovenia/1756-20_21VIR959-3/2020	A / H5N8	Europe / Slovenia	Bianca Zecchin (Istituto Zooprofilattico Sperimentale Delle Venezie)
EPI_ISL_1665265	A/mute_swan/Slovenia/1799-20_21VIR959-2/2020	A / H5N8	Europe / Slovenia	Bianca Zecchin (Istituto Zooprofilattico Sperimentale Delle Venezie)
EPI_ISL_1665266	A/mute_swan/Slovenia/1820-20_21VIR959-4/2020	A / H5N8	Europe / Slovenia	Bianca Zecchin (Istituto Zooprofilattico Sperimentale Delle Venezie)
EPI_ISL_1665267	A/mute_swan/Slovenia/1914-20_21VIR959-5/2020	A / H5N5	Europe / Slovenia	Bianca Zecchin (Istituto Zooprofilattico Sperimentale Delle Venezie)
EPI_ISL_1665268	A/whooper_swan/Romania/10123_21VIR849-1/2021	A / H5N5	Europe / Romania / Judetul Constanta	Bianca Zecchin (Istituto Zooprofilattico Sperimentale Delle Venezie)
EPI_ISL_1665269	A/whooper_swan/Romania/10311_21VIR849-2/2021	A / H5N5	Europe / Romania / Judetul Constanta	Bianca Zecchin (Istituto Zooprofilattico Sperimentale Delle Venezie)
EPI_ISL_1665272	A/common_buzzard/Italy/21VIR431-7/2020	A / H5N8	Europe / Italy / Lecce	Bianca Zecchin (Istituto Zooprofilattico Sperimentale Delle Venezie)
EPI_ISL_1665273	A/geese/Romania/10206_21VIR849-3/2021	A / H5N8	Europe / Romania / Judetul Ilfov	Bianca Zecchin (Istituto Zooprofilattico Sperimentale Delle Venezie)
EPI_ISL_1665274	A/house_sparrow/Romania/10327_21VIR849-5/2021	A / H5N8	Europe / Romania / Judetul Ilfov	Bianca Zecchin (Istituto Zooprofilattico Sperimentale Delle Venezie)
EPI_ISL_166584	A/duck/Beijing/FS01/2013	A / H5N8	Asia / China / Beijing Municipality / Fangshan District	Di Liu (Institute of Microbiology, Chinese Academy of Sciences)
EPI_ISL_166630	A/mallard/Korea/W452/2014	A / H5N8	Asia / Korea, Republic of	
EPI_ISL_166693	A/duck/Beijing/FS01/2014	A / H5N8	Asia / China / Beijing Municipality / Fangshan District	Di Liu (Institute of Microbiology, Chinese Academy of Sciences)
EPI_ISL_166694	A/duck/Beijing/CT01/2014	A / H5N8	Asia / China / Beijing Municipality / Shunyi District	Di Liu (Institute of Microbiology, Chinese Academy of Sciences)
EPI_ISL_167140	A/turkey/Germany-MV/R2472/2014	A / H5N8	Europe / Germany / Mecklenburg-Vorpommern	Elke Starick (Friedrich-Loeffler-Institut)
EPI_ISL_167178	A/duck/Shandong/Q1/2013	A / H5N8	Asia / China	
EPI_ISL_167767	A/environment/Zhenjiang/C13/2013	A / H5N6	Asia / China	
EPI_ISL_167904	A/duck/England/36254/14	A / H5N8	Europe / United Kingdom / East Riding of Yorkshire	Amanda Seekings (Animal and Plant Health Agency (APHA) / Virology Department)
EPI_ISL_167905	A/Chicken/Netherlands/14015526/2014	A / H5N8	Europe / Netherlands / Provincie Utrecht / Gemeente Oudewater	Guus Koch (Wageningen Bioveterinary Research)
EPI_ISL_168024	A/duck/Chiba/26-372-48/2014	A / H5N8	Asia / Japan / Chiba	Takehiko Saito (National Institute of Animal Health)
EPI_ISL_168025	A/duck/Chiba/26-372-61/2014	A / H5N8	Asia / Japan / Chiba	Takehiko Saito (National Institute of Animal Health)
EPI_ISL_168075	A/chicken/Netherlands/14015531/2014	A / H5N8	Europe / Netherlands / Provincie Utrecht / Gemeente Oudewater	Guus Koch (Wageningen Bioveterinary Research)
EPI_ISL_168384	A/chicken/Laos/LPQ001/2014	A / H5N6	Asia / Lao, People's Democratic Republic	
EPI_ISL_168385	A/duck/Laos/XBY004/2014	A / H5N6	Asia / Lao, People's Democratic Republic	
EPI_ISL_168663	A/duck/Laos/LPQ002/2014	A / H5N6	Asia / Lao, People's Democratic Republic	
EPI_ISL_168664	A/chicken/Laos/XBY003/2014	A / H5N6	Asia / Lao, People's Democratic Republic	
EPI_ISL_168696	A/duck/England/36038/14	A / H5N8	Europe / United Kingdom / East Riding of Yorkshire	Amanda Seekings (Animal and Plant Health Agency (APHA) / Virology Department)

EPI_ISL_168697	A/duck/England/36226/14	A / H5N8	Europe / United Kingdom / East Riding of Yorkshire	Amanda Seekings (Animal and Plant Health Agency (APHA) / Virology Department)
EPI_ISL_168746	A/eurasian wigeon/Netherlands/emc-1/2014	A / H5N8	Europe / Netherlands / Provincie Utrecht / Gemeente Woerden / Between Kamerik and Kockeng	Josanne Verhagen (Erasmus Medical Center / Department of Virology)
EPI_ISL_168747	A/eurasian wigeon/Netherlands/emc-2/2014	A / H5N8	Europe / Netherlands / Provincie Utrecht / Gemeente Woerden / Between Kamerik and Kockeng	Josanne Verhagen (Erasmus Medical Center / Department of Virology)
EPI_ISL_169273	A/turkey/Germany/AR2485-86-L00899/2014	A / H5N8	Europe / Germany / Mecklenburg-Vorpommern	Anne Pohlmann (Friedrich-Loeffler-Institut)
EPI_ISL_169282	A/chicken/Netherlands/emc-3/2014	A / H5N8	Europe / Netherlands / South Holland / Gemeente Nieuwkoop	Josanne Verhagen (Erasmus Medical Center / Department of Virology)
EPI_ISL_169350	A/turkey/Italy/14VIR7898-10/2014	A / H5N8	Europe / Italy / Veneto / Province of Rovigo	Alice Fusaro (Istituto Zooprofilattico Sperimentale Delle Venezie)
EPI_ISL_169390	A/crane/Kagoshima/KU1/2014	A / H5N8	Asia / Japan / Kagoshima	Makoto Ozawa (Kagoshima University)
EPI_ISL_169422	A/chicken/Miyazaki/3/2014	A / H5N8	Asia / Japan / Miyazaki	Takehiko Saito (National Institute of Animal Health)
EPI_ISL_169423	A/chicken/Miyazaki/4/2014	A / H5N8	Asia / Japan / Miyazaki	Takehiko Saito (National Institute of Animal Health)
EPI_ISL_169424	A/chicken/Miyazaki/7/2014	A / H5N8	Asia / Japan / Miyazaki	Takehiko Saito (National Institute of Animal Health)
EPI_ISL_169427	A/wigeon/Sakha/1/2014	A / H5N8	Europe / Russian Federation / Sakha (Yakutia) Republic	Ivan Susloparov (State Research Center of Virology and Biotechnology (VECTOR) / Emerging Zoonotic Diseases and Influenza)
EPI_ISL_169429	A/environment/Kagoshima/KU-ngr-H/2014	A / H5N8	Asia / Japan / Kagoshima	Makoto Ozawa (Kagoshima University)
EPI_ISL_169632	A/domestic duck/Germany-NI/R3468/2014	A / H5N8	Europe / Germany / Lower Saxony	Elke Starick (Friedrich-Loeffler-Institut)
EPI_ISL_1697181	A/chicken/Czech Republic/6151-1/2021	A / H5N8	Europe / Czech Republic / Kralovehradecky Kraj / Okres Hradec Kralove / Kosicky	Alexander Nagy (State Veterinary Institute Prague)
EPI_ISL_1697182	A/chicken/Czech Republic/5903/2021	A / H5N8	Europe / Czech Republic / Kralovehradecky Kraj / Okres Jicin / Vysoke Veseli	Alexander Nagy (State Veterinary Institute Prague)
EPI_ISL_1697183	A/chicken/Czech Republic/6684/2021	A / H5N8	Europe / Czech Republic / Ustecky Kraj / Okres Most / Polerady	Alexander Nagy (State Veterinary Institute Prague)
EPI_ISL_1697184	A/chicken/Czech Republic/6654/2021	A / H5N8	Europe / Czech Republic / Plzensky Kraj / Okres Tacov / Bor; GPS: 49.7115939N, 12.7751636E	Alexander Nagy (State Veterinary Institute Prague)
EPI_ISL_1697185	A/duck/Czech Republic/6653-4/2021	A / H5N8	Europe / Czech Republic / Kralovehradecky Kraj / Okres Hradec Kralove / Stary Bydzov	Alexander Nagy (State Veterinary Institute Prague)
EPI_ISL_1697186	A/duck/Czech Republic/6653-6/2021	A / H5N8	Europe / Czech Republic / Kralovehradecky Kraj / Okres Hradec Kralove / Stary Bydzov	Alexander Nagy (State Veterinary Institute Prague)
EPI_ISL_1697187	A/chicken/Czech Republic/6542-1/2021	A / H5N8	Europe / Czech Republic / Hlavni mesto Praha / Okres Praha / Trebotov; GPS: 49.6993983N, 14.2	Alexander Nagy (State Veterinary Institute Prague)
EPI_ISL_1697188	A/chicken/Czech Republic/6532-1/2021	A / H5N8	Europe / Czech Republic / Morovskoslezsky Kraj / Okres Bruntal / Zator	Alexander Nagy (State Veterinary Institute Prague)
EPI_ISL_1697189	A/chicken/Czech Republic/6532-2/2021	A / H5N8	Europe / Czech Republic / Morovskoslezsky Kraj / Okres Bruntal / Zator	Alexander Nagy (State Veterinary Institute Prague)
EPI_ISL_1697190	A/peacock/Czech Republic/6529-2/2021	A / H5N8	Europe / Czech Republic / Ustecky Kraj / Okres Teplice / Bilina, Razice; GPS: 50.5215139N, 13.82	Alexander Nagy (State Veterinary Institute Prague)
EPI_ISL_1697191	A/chicken/Czech Republic/6527/2021	A / H5N8	Europe / Czech Republic / Kralovehradecky Kraj / Okres Hradec Kralove / Dobrenice	Alexander Nagy (State Veterinary Institute Prague)
EPI_ISL_1697192	A/duck/Czech Republic/5792-13/2021	A / H5N8	Europe / Czech Republic / Kralovehradecky Kraj / Okres Hradec Kralove / Lukova	Alexander Nagy (State Veterinary Institute Prague)
EPI_ISL_1697193	A/duck/Czech Republic/5792-19/2021	A / H5N8	Europe / Czech Republic / Kralovehradecky Kraj / Okres Hradec Kralove / Lukova	Alexander Nagy (State Veterinary Institute Prague)
EPI_ISL_1697194	A/chicken/Czech Republic/4980/2021	A / H5N8	Europe / Czech Republic / Zlinsky Kraj / Okres Uherske Hradiste / Osvetimany	Alexander Nagy (State Veterinary Institute Prague)
EPI_ISL_1697195	A/duck/Czech Republic/5466/2021	A / H5N8	Europe / Czech Republic / Pardubicky kraj / Okres Pardubice / Vapno; GPS: 50.1074822N, 15.527	Alexander Nagy (State Veterinary Institute Prague)
EPI_ISL_1697196	A/duck/Czech Republic/5448/2021	A / H5N8	Europe / Czech Republic / Stredocesky Kraj / Okres Nymburk / Vinice u Mestce Kralove	Alexander Nagy (State Veterinary Institute Prague)
EPI_ISL_1697197	A/australian brushturkey/Czech Republic/5904/2021	A / H5N8	Europe / Czech Republic / Plzensky Kraj	Alexander Nagy (State Veterinary Institute Prague)
EPI_ISL_1697198	A/duck/Czech Republic/5360-1/2021	A / H5N8	Europe / Czech Republic / Kralovehradecky Kraj / Okres Hradec Kralove / Dobrenice	Alexander Nagy (State Veterinary Institute Prague)
EPI_ISL_1697199	A/duck/Czech Republic/5360-2/2021	A / H5N8	Europe / Czech Republic / Kralovehradecky Kraj / Okres Hradec Kralove / Dobrenice	Alexander Nagy (State Veterinary Institute Prague)
EPI_ISL_1697200	A/chicken/Czech Republic/6542-2/2021	A / H5N8	Europe / Czech Republic / Hlavni mesto Praha / Okres Praha / Trebotov; GPS: 49.6993983N, 14.2	Alexander Nagy (State Veterinary Institute Prague)
EPI_ISL_169851	A/stork/Germany-MV/R24/2015	A / H5N8	Europe / Germany / Mecklenburg-Vorpommern	Elke Starick (Friedrich-Loeffler-Institut)
EPI_ISL_170155	A/Environment/Xuzhou/yj010/2014	A / H5N6	Asia / China / jiangsu	Lunbiao Cui (Jiangsu Provincial Center for Disease Control & Prevention)
EPI_ISL_170184	A/chicken/Germany-MV/R153/2015	A / H5N8	Europe / Germany / Mecklenburg-Vorpommern	Elke Starick (Friedrich-Loeffler-Institut)
EPI_ISL_170230	A/Environment/Zhenjiang/zj35/2014	A / H5N8	Asia / China / jiangsu	Lunbiao Cui (Jiangsu Provincial Center for Disease Control & Prevention)
EPI_ISL_170234	A/Chicken/Changzhou/cz93/2014	A / H5N8	Asia / China / jiangsu	Lunbiao Cui (Jiangsu Provincial Center for Disease Control & Prevention)
EPI_ISL_170236	A/Environment/Huaian/ha118/2014	A / H5N8	Asia / China / jiangsu	Lunbiao Cui (Jiangsu Provincial Center for Disease Control & Prevention)
EPI_ISL_171655	A/waterfowl/Korea/S005/2014	A / H5N8	Asia / Korea, Republic of	
EPI_ISL_171656	A/broiler duck/Korea/H29/2014	A / H5N8	Asia / Korea, Republic of	
EPI_ISL_171657	A/broiler duck/Korea/H31/2014	A / H5N8	Asia / Korea, Republic of	
EPI_ISL_171674	A/Baikal teal/Korea/H41/2014	A / H5N8	Asia / Korea, Republic of	
EPI_ISL_171675	A/broiler duck/Korea/H47/2014	A / H5N8	Asia / Korea, Republic of	
EPI_ISL_171676	A/broiler duck/Korea/H48/2014	A / H5N8	Asia / Korea, Republic of	
EPI_ISL_171677	A/broiler duck/Korea/H49/2014	A / H5N8	Asia / Korea, Republic of	
EPI_ISL_171678	A/bean goose/Korea/H40/2014	A / H5N8	Asia / Korea, Republic of	
EPI_ISL_171695	A/Baikal teal/Korea/H52/2014	A / H5N8	Asia / Korea, Republic of	
EPI_ISL_171696	A/bean goose/Korea/H53/2014	A / H5N8	Asia / Korea, Republic of	
EPI_ISL_171697	A/Baikal teal/Korea/H62/2014	A / H5N8	Asia / Korea, Republic of	
EPI_ISL_171698	A/broiler duck/Korea/H65/2014	A / H5N8	Asia / Korea, Republic of	
EPI_ISL_171699	A/Baikal teal/Korea/H66/2014	A / H5N8	Asia / Korea, Republic of	
EPI_ISL_171700	A/Baikal teal/Korea/H68/2014	A / H5N8	Asia / Korea, Republic of	
EPI_ISL_171701	A/Baikal teal/Korea/H80/2014	A / H5N8	Asia / Korea, Republic of	
EPI_ISL_171702	A/Coot/Korea/H81/2014	A / H5N8	Asia / Korea, Republic of	
EPI_ISL_171703	A/Baikal teal/Korea/H84/2014	A / H5N8	Asia / Korea, Republic of	
EPI_ISL_171704	A/Baikal teal/Korea/H96/2014	A / H5N8	Asia / Korea, Republic of	
EPI_ISL_171705	A/breeder chicken/Korea/H122/2014	A / H5N8	Asia / Korea, Republic of	
EPI_ISL_171706	A/breeder duck/Korea/H128/2014	A / H5N8	Asia / Korea, Republic of	
EPI_ISL_171707	A/broiler duck/Korea/H133/2014	A / H5N8	Asia / Korea, Republic of	
EPI_ISL_171708	A/broiler duck/Korea/H145/2014	A / H5N8	Asia / Korea, Republic of	
EPI_ISL_171709	A/breeder duck/Korea/H158/2014	A / H5N8	Asia / Korea, Republic of	
EPI_ISL_171710	A/breeder duck/Korea/H200/2014	A / H5N8	Asia / Korea, Republic of	
EPI_ISL_171711	A/mallard/Korea/H207/2014	A / H5N8	Asia / Korea, Republic of	
EPI_ISL_171712	A/white-fronted goose/Korea/H231/2014	A / H5N8	Asia / Korea, Republic of	
EPI_ISL_171713	A/breeder duck/Korea/H249/2014	A / H5N8	Asia / Korea, Republic of	
EPI_ISL_171714	A/breeder chicken/Korea/H250/2014	A / H5N8	Asia / Korea, Republic of	
EPI_ISL_171715	A/Korean native chicken/Korea/H257/2014	A / H5N8	Asia / Korea, Republic of	
EPI_ISL_171716	A/mallard/Korea/H297/2014	A / H5N8	Asia / Korea, Republic of	
EPI_ISL_171717	A/bean goose/Korea/H328/2014	A / H5N8	Asia / Korea, Republic of	
EPI_ISL_171718	A/tundra swan/Korea/H411/2014	A / H5N8	Asia / Korea, Republic of	
EPI_ISL_171719	A/common teal/Korea/H455-30/2014	A / H5N8	Asia / Korea, Republic of	

EPI_ISL_171720	A/spot-billed duck/Korea/H455-42/2014	A / H5N8	Asia / Korea, Republic of	
EPI_ISL_1719909	A/swan/Lithuania/1258PG1_21VIR2606-2/2021	A / H5N8	Europe / Lithuania / Vilnius	Bianca Zecchin (Istituto Zooprofilattico Sperimentale Delle Venezie)
EPI_ISL_1719910	A/swan/Lithuania/1298PG1_21VIR2606-3/2021	A / H5N8	Europe / Lithuania / Vilnius	Bianca Zecchin (Istituto Zooprofilattico Sperimentale Delle Venezie)
EPI_ISL_1719911	A/swan/Lithuania/1842PG1_21VIR2606-5/2021	A / H5N8	Europe / Lithuania / Vilnius	Bianca Zecchin (Istituto Zooprofilattico Sperimentale Delle Venezie)
EPI_ISL_1719912	A/swan/Lithuania/2306PG1_21VIR2606-7/2021	A / H5N8	Europe / Lithuania / Vilnius	Bianca Zecchin (Istituto Zooprofilattico Sperimentale Delle Venezie)
EPI_ISL_1719913	A/chicken/Bulgaria/39_21VIR1454-3/2021	A / H5N8	Europe / Bulgaria / Slavyanovo	Bianca Zecchin (Istituto Zooprofilattico Sperimentale Delle Venezie)
EPI_ISL_1719914	A/pekin_duck/Bulgaria/48-3_21VIR1454-7/2021	A / H5N8	Europe / Bulgaria / Slavyanovo	Bianca Zecchin (Istituto Zooprofilattico Sperimentale Delle Venezie)
EPI_ISL_1719915	A/chicken/Bulgaria/50-1_21VIR1454-9/2021	A / H5N8	Europe / Bulgaria / Slavyanovo	Bianca Zecchin (Istituto Zooprofilattico Sperimentale Delle Venezie)
EPI_ISL_172815	A/ibis/Germany-MV/R44/2015	A / H5N8	Europe / Germany / Mecklenburg-Vorpommern	Elke Starick (Friedrich-Loeffler-Institut)
EPI_ISL_173478	A/duck/Jiangxi/13469/2014	A / H5N6	Asia / China	
EPI_ISL_173479	A/duck/Jiangxi/13475/2014	A / H5N6	Asia / China	
EPI_ISL_173480	A/duck/Jiangxi/13484/2014	A / H5N6	Asia / China	
EPI_ISL_173482	A/black chicken/Jiangxi/10129/2014	A / H5N6	Asia / China	
EPI_ISL_173483	A/environment/Jiangxi/10170/2014	A / H5N6	Asia / China	
EPI_ISL_173531	A/goose/Shantou/1763/2014	A / H5N6	Asia / China	
EPI_ISL_173532	A/goose/Shantou/1791/2014	A / H5N6	Asia / China	
EPI_ISL_173533	A/goose/Shantou/1806/2014	A / H5N6	Asia / China	
EPI_ISL_173568	A/green-winged teal/Korea/KU-12/2015	A / H5	Asia / Korea, Republic of / Chungcheongnam-do	Dong-Hun Lee (Konkuk University / College of Veterinary Medicine)
EPI_ISL_173664	A/chicken/Washington/61-9/2014	A / H5N2	North America / United States	
EPI_ISL_173665	A/turkey/Washington/61-22/2014	A / H5N2	North America / United States	
EPI_ISL_173666	A/chicken/Oregon/41613-2/2014	A / H5N8	North America / United States	
EPI_ISL_173667	A/domestic duck/Washington/61-16/2014	A / H5N2	North America / United States	
EPI_ISL_173668	A/guinea fowl/Oregon/41613-1/2014	A / H5N8	North America / United States	
EPI_ISL_173669	A/duck/Zhejiang/6D2/2013	A / H5N6	Asia / China	
EPI_ISL_173670	A/chicken/Zhejiang/6C2/2013	A / H5N6	Asia / China	
EPI_ISL_173877	A/Northern pintail/Washington/40964/2014	A / H5N2	North America / United States	
EPI_ISL_173878	A/gyrfalcon/Washington/41088-6/2014	A / H5N8	North America / United States	
EPI_ISL_174349	A/chicken/Netherlands/14015766/2014	A / H5N8	Europe / Netherlands / South Holland / Gemeente Nieuwkoop / Ter Aar	Guus Koch (Wageningen Bioveterinary Research)
EPI_ISL_174350	A/Chicken/Netherlands/14015824/2014	A / H5N8	Europe / Netherlands / Provincie Overijssel / Gemeente Kampen / Kamperveen	Guus Koch (Wageningen Bioveterinary Research)
EPI_ISL_174351	A/duck/Netherlands/14015989/2014	A / H5N8	Europe / Netherlands / Provincie Overijssel / Gemeente Kampen / Kamperveen	Guus Koch (Wageningen Bioveterinary Research)
EPI_ISL_174352	A/chicken/Netherlands/14016437/2014	A / H5N8	Europe / Netherlands / South Holland / Gemeente Zoeterwoude / 2381 NZ	Guus Koch (Wageningen Bioveterinary Research)
EPI_ISL_174353	A/breeder chicken/Korea/H0503/2014	A / H5N8	Asia / Korea, Republic of / Jeollabuk-do	Eun-Kyoung Lee (Animal and Plant Quarantine Agency (APQA) / Avian Influenza Research and Diagsnotic Division)
EPI_ISL_174354	A/breeder chicken/Korea/H0818/2014	A / H5N8	Asia / Korea, Republic of / Chungcheongnam-do	Eun-Kyoung Lee (Animal and Plant Quarantine Agency (APQA) / Avian Influenza Research and Diagsnotic Division)
EPI_ISL_174355	A/broiler duck/Korea/H0651/2014	A / H5N8	Asia / Korea, Republic of / Jeollanam-do	Eun-Kyoung Lee (Animal and Plant Quarantine Agency (APQA) / Avian Influenza Research and Diagsnotic Division)
EPI_ISL_174356	A/chicken/Korea/H0881/2014	A / H5N8	Asia / Korea, Republic of	Eun-Kyoung Lee (Animal and Plant Quarantine Agency (APQA) / Avian Influenza Research and Diagsnotic Division)
EPI_ISL_174357	A/broiler duck/Korea/H0959/2014	A / H5N8	Asia / Korea, Republic of / Jeollanam-do	Eun-Kyoung Lee (Animal and Plant Quarantine Agency (APQA) / Avian Influenza Research and Diagsnotic Division)
EPI_ISL_174358	A/breeder chicken/Korea/H1068/2014	A / H5N8	Asia / Korea, Republic of / Chungcheongnam-do	Eun-Kyoung Lee (Animal and Plant Quarantine Agency (APQA) / Avian Influenza Research and Diagsnotic Division)
EPI_ISL_174359	A/Korean native chicken/Korea/H1139/2014	A / H5N8	Asia / Korea, Republic of / Chungcheongnam-do	Eun-Kyoung Lee (Animal and Plant Quarantine Agency (APQA) / Avian Influenza Research and Diagsnotic Division)
EPI_ISL_174360	A/chicken/Korea/H1236/2014	A / H5N8	Asia / Korea, Republic of / Chungcheongnam-do	Eun-Kyoung Lee (Animal and Plant Quarantine Agency (APQA) / Avian Influenza Research and Diagsnotic Division)
EPI_ISL_174361	A/chicken/Korea/H1268/2014	A / H5N8	Asia / Korea, Republic of / Gyeongsangnam-do	Eun-Kyoung Lee (Animal and Plant Quarantine Agency (APQA) / Avian Influenza Research and Diagsnotic Division)
EPI_ISL_174362	A/chicken/Korea/H1292/2014	A / H5N8	Asia / Korea, Republic of / Gyeonggi-do	Eun-Kyoung Lee (Animal and Plant Quarantine Agency (APQA) / Avian Influenza Research and Diagsnotic Division)
EPI_ISL_174363	A/goose/Korea/H1296/2014	A / H5N8	Asia / Korea, Republic of / Chungcheongbuk-do	Eun-Kyoung Lee (Animal and Plant Quarantine Agency (APQA) / Avian Influenza Research and Diagsnotic Division)
EPI_ISL_174364	A/Korean native chicken/Korea/H1299/2014	A / H5N8	Asia / Korea, Republic of / Ulsan	Eun-Kyoung Lee (Animal and Plant Quarantine Agency (APQA) / Avian Influenza Research and Diagsnotic Division)
EPI_ISL_174365	A/breeder duck/Korea/H1343/2014	A / H5N8	Asia / Korea, Republic of / Jeollanam-do	Eun-Kyoung Lee (Animal and Plant Quarantine Agency (APQA) / Avian Influenza Research and Diagsnotic Division)
EPI_ISL_174366	A/chicken/Korea/H1350/2014	A / H5N8	Asia / Korea, Republic of / Chungcheongnam-do	Eun-Kyoung Lee (Animal and Plant Quarantine Agency (APQA) / Avian Influenza Research and Diagsnotic Division)
EPI_ISL_174367	A/gadwall/Korea/H1351/2014	A / H5N8	Asia / Korea, Republic of / Jeju-teukbyeoljachido	Eun-Kyoung Lee (Animal and Plant Quarantine Agency (APQA) / Avian Influenza Research and Diagsnotic Division)
EPI_ISL_174368	A/broiler duck/Korea/H1413/2014	A / H5N8	Asia / Korea, Republic of / Jeollanam-do	Eun-Kyoung Lee (Animal and Plant Quarantine Agency (APQA) / Avian Influenza Research and Diagsnotic Division)
EPI_ISL_174369	A/broiler duck/Korea/H1414/2014	A / H5N8	Asia / Korea, Republic of / Jeollanam-do	Eun-Kyoung Lee (Animal and Plant Quarantine Agency (APQA) / Avian Influenza Research and Diagsnotic Division)
EPI_ISL_174370	A/goose/Korea/H1545/2014	A / H5N8	Asia / Korea, Republic of / Gangwon-do	Eun-Kyoung Lee (Animal and Plant Quarantine Agency (APQA) / Avian Influenza Research and Diagsnotic Division)
EPI_ISL_174371	A/Korean native chicken/Korea/H1554/2014	A / H5N8	Asia / Korea, Republic of / Daegu	Eun-Kyoung Lee (Animal and Plant Quarantine Agency (APQA) / Avian Influenza Research and Diagsnotic Division)
EPI_ISL_174372	A/broiler duck/Korea/H1556/2014	A / H5N8	Asia / Korea, Republic of / Jeollanam-do	Eun-Kyoung Lee (Animal and Plant Quarantine Agency (APQA) / Avian Influenza Research and Diagsnotic Division)
EPI_ISL_174373	A/broiler duck/Korea/H1582/2014	A / H5N8	Asia / Korea, Republic of / Gyeonggi-do	Eun-Kyoung Lee (Animal and Plant Quarantine Agency (APQA) / Avian Influenza Research and Diagsnotic Division)
EPI_ISL_174374	A/breeder duck/Korea/H1596/2014	A / H5N8	Asia / Korea, Republic of / Jeollanam-do	Eun-Kyoung Lee (Animal and Plant Quarantine Agency (APQA) / Avian Influenza Research and Diagsnotic Division)
EPI_ISL_174375	A/broiler duck/Korea/H1683/2014	A / H5N8	Asia / Korea, Republic of / Jeollanam-do	Eun-Kyoung Lee (Animal and Plant Quarantine Agency (APQA) / Avian Influenza Research and Diagsnotic Division)
EPI_ISL_174376	A/broiler duck/Korea/H1685/2014	A / H5N8	Asia / Korea, Republic of / Jeollanam-do	Eun-Kyoung Lee (Animal and Plant Quarantine Agency (APQA) / Avian Influenza Research and Diagsnotic Division)
EPI_ISL_174377	A/Korean native chicken/Korea/H1687/2014	A / H5N8	Asia / Korea, Republic of / Jeollanam-do	Eun-Kyoung Lee (Animal and Plant Quarantine Agency (APQA) / Avian Influenza Research and Diagsnotic Division)
EPI_ISL_174378	A/goose/Korea/H1689/2014	A / H5N8	Asia / Korea, Republic of / Jeollanam-do	Eun-Kyoung Lee (Animal and Plant Quarantine Agency (APQA) / Avian Influenza Research and Diagsnotic Division)
EPI_ISL_174379	A/goose/Korea/H1698/2014	A / H5N8	Asia / Korea, Republic of / Jeollanam-do	Eun-Kyoung Lee (Animal and Plant Quarantine Agency (APQA) / Avian Influenza Research and Diagsnotic Division)
EPI_ISL_174380	A/broiler duck/Korea/H1731/2014	A / H5N8	Asia / Korea, Republic of / Jeollanam-do	Eun-Kyoung Lee (Animal and Plant Quarantine Agency (APQA) / Avian Influenza Research and Diagsnotic Division)
EPI_ISL_174381	A/broiler duck/Korea/H1733/2014	A / H5N8	Asia / Korea, Republic of / Jeollanam-do	Eun-Kyoung Lee (Animal and Plant Quarantine Agency (APQA) / Avian Influenza Research and Diagsnotic Division)
EPI_ISL_174382	A/broiler duck/Korea/H1734/2014	A / H5N8	Asia / Korea, Republic of / Jeollanam-do	Eun-Kyoung Lee (Animal and Plant Quarantine Agency (APQA) / Avian Influenza Research and Diagsnotic Division)
EPI_ISL_174383	A/broiler duck/Korea/H1739/2014	A / H5N8	Asia / Korea, Republic of / Jeollanam-do	Eun-Kyoung Lee (Animal and Plant Quarantine Agency (APQA) / Avian Influenza Research and Diagsnotic Division)
EPI_ISL_174384	A/broiler duck/Korea/H1745/2014	A / H5N8	Asia / Korea, Republic of / Jeollanam-do	Eun-Kyoung Lee (Animal and Plant Quarantine Agency (APQA) / Avian Influenza Research and Diagsnotic Division)
EPI_ISL_174385	A/Korean native chicken/Korea/H1747/2014	A / H5N8	Asia / Korea, Republic of / Jeollanam-do	Eun-Kyoung Lee (Animal and Plant Quarantine Agency (APQA) / Avian Influenza Research and Diagsnotic Division)
EPI_ISL_174386	A/breeder duck/Korea/H1752/2014	A / H5N8	Asia / Korea, Republic of / Jeollanam-do	Eun-Kyoung Lee (Animal and Plant Quarantine Agency (APQA) / Avian Influenza Research and Diagsnotic Division)
EPI_ISL_174387	A/broiler duck/Korea/H1755/2014	A / H5N8	Asia / Korea, Republic of / Jeollanam-do	Eun-Kyoung Lee (Animal and Plant Quarantine Agency (APQA) / Avian Influenza Research and Diagsnotic Division)
EPI_ISL_174388	A/broiler duck/Korea/H1763/2014	A / H5N8	Asia / Korea, Republic of / Jeollanam-do	Eun-Kyoung Lee (Animal and Plant Quarantine Agency (APQA) / Avian Influenza Research and Diagsnotic Division)
EPI_ISL_174389	A/broiler duck/Korea/H1803/2014	A / H5N8	Asia / Korea, Republic of / Jeollanam-do	Eun-Kyoung Lee (Animal and Plant Quarantine Agency (APQA) / Avian Influenza Research and Diagsnotic Division)
EPI_ISL_174390	A/broiler duck/Korea/H1839/2014	A / H5N8	Asia / Korea, Republic of / Jeollanam-do	Eun-Kyoung Lee (Animal and Plant Quarantine Agency (APQA) / Avian Influenza Research and Diagsnotic Division)
EPI_ISL_174391	A/broiler duck/Korea/H1840/2014	A / H5N8	Asia / Korea, Republic of / Jeollabuk-do	Eun-Kyoung Lee (Animal and Plant Quarantine Agency (APQA) / Avian Influenza Research and Diagsnotic Division)
EPI_ISL_174392	A/Korean native chicken/Korea/H1847/2014	A / H5N8	Asia / Korea, Republic of / Jeollanam-do	Eun-Kyoung Lee (Animal and Plant Quarantine Agency (APQA) / Avian Influenza Research and Diagsnotic Division)
EPI_ISL_174393	A/broiler duck/Korea/H1864/2014	A / H5N8	Asia / Korea, Republic of / Jeollabuk-do	Eun-Kyoung Lee (Animal and Plant Quarantine Agency (APQA) / Avian Influenza Research and Diagsnotic Division)

EPI_ISL_174394	A/Korean native chicken/Korea/H1903/2014	A / H5N8 Asia / Korea, Republic of / Gyeongsangbuk-do	Eun-Kyoung Lee (Animal and Plant Quarantine Agency (APQA) / Avian Influenza Research and Diagsnotic Division)
EPI_ISL_174395	A/spot-billed duck/Korea/H1981/2014	A / H5N8 Asia / Korea, Republic of / Chungcheongbuk-do	Eun-Kyoung Lee (Animal and Plant Quarantine Agency (APQA) / Avian Influenza Research and Diagsnotic Division)
EPI_ISL_174396	A/mallard/Korea/H1991/2014	A / H5N8 Asia / Korea, Republic of / Gyeonggi-do	Eun-Kyoung Lee (Animal and Plant Quarantine Agency (APQA) / Avian Influenza Research and Diagsnotic Division)
EPI_ISL_174397	A/mallard/Korea/H2003/2014	A / H5N8 Asia / Korea, Republic of / Gyeonggi-do	Eun-Kyoung Lee (Animal and Plant Quarantine Agency (APQA) / Avian Influenza Research and Diagsnotic Division)
EPI_ISL_174398	A/mallard/Korea/H1924-6/2014	A / H5N8 Asia / Korea, Republic of / Jeollanam-do	Eun-Kyoung Lee (Animal and Plant Quarantine Agency (APQA) / Avian Influenza Research and Diagsnotic Division)
EPI_ISL_174399	A/breeder duck/Korea/H0345/2014	A / H5N8 Asia / Korea, Republic of / Chungcheongbuk-do	Eun-Kyoung Lee (Animal and Plant Quarantine Agency (APQA) / Avian Influenza Research and Diagsnotic Division)
EPI_ISL_174400	A/breeder duck/Korea/H0566/2014	A / H5N8 Asia / Korea, Republic of / Chungcheongbuk-do	Eun-Kyoung Lee (Animal and Plant Quarantine Agency (APQA) / Avian Influenza Research and Diagsnotic Division)
EPI_ISL_174401	A/Common Teal/Korea/H844/2014	A / H5N8 Asia / Korea, Republic of / Chungcheongnam-do	Eun-Kyoung Lee (Animal and Plant Quarantine Agency (APQA) / Avian Influenza Research and Diagsnotic Division)
EPI_ISL_174409	A/American green-winged teal/Washington/195750/2014	A / H5N1 North America / United States	
EPI_ISL_174411	A/turkey/BC/FAV10/2014	A / H5N2 North America / Canada	
EPI_ISL_174516	A/crane/Kagoshima/KU13/2014(H5N8)	A / H5N8 Asia / Japan / Kagoshima	Makoto Ozawa (Kagoshima University)
EPI_ISL_174517	A/crane/Kagoshima/KU21/2014(H5N8)	A / H5N8 Asia / Japan / Kagoshima	Makoto Ozawa (Kagoshima University)
EPI_ISL_174518	A/crane/Kagoshima/KU41/2014(H5N8)	A / H5N8 Asia / Japan / Kagoshima	Makoto Ozawa (Kagoshima University)
EPI_ISL_174519	A/crane/Kagoshima/KU53/2015(H5N8)	A / H5N8 Asia / Japan / Kagoshima	Makoto Ozawa (Kagoshima University)
EPI_ISL_174520	A/mallard duck/Kagoshima/KU70/2015(H5N8)	A / H5N8 Asia / Japan / Kagoshima	Makoto Ozawa (Kagoshima University)
EPI_ISL_174521	A/mallard duck/Kagoshima/KU116/2015(H5N8)	A / H5N8 Asia / Japan / Kagoshima	Makoto Ozawa (Kagoshima University)
EPI_ISL_175335	A/Guangzhou/39715/2014	A / H5N6 Asia / China	
EPI_ISL_175336	A/duck/Guangzhou/41227/2014	A / H5N6 Asia / China	
EPI_ISL_175339	A/chicken/BC/FAV8/2014	A / H5N2 North America / Canada	
EPI_ISL_175340	A/chicken/BC/FAV9/2014	A / H5N2 North America / Canada	
EPI_ISL_175534	A/MuteSwan/Sweden/SVA150311KU0277/SZ502/2015	A / H5N8 Europe / Sweden / Stockholms Lan / Stockholms Kommun / Strömkajen, Stockholm	Siamak Zohari (National Veterinary Institute)
EPI_ISL_175535	A/MuteSwan/Sweden/SVA150313KU0141/SZ543/2015	A / H5N8 Europe / Sweden / Stockholms Lan / Stockholms Kommun / Ladugårdslandsviken, Stockholm	Siamak Zohari (National Veterinary Institute)
EPI_ISL_1760446	A/whooper swan/Shaanxi/SXY26/2020	A / H5N8 Asia / China / Shaanxi Province	Hongliang Chai (Northeast Forestry University / College of Wildlife Resources)
EPI_ISL_1760447	A/whooper swan/Shaanxi/SXY66/2020	A / H5N8 Asia / China / Shaanxi Province	Hongliang Chai (Northeast Forestry University / College of Wildlife Resources)
EPI_ISL_1760448	A/common teal/Shaanxi/SXY1-1/2020	A / H5N8 Asia / China / Shaanxi Province	Hongliang Chai (Northeast Forestry University / College of Wildlife Resources)
EPI_ISL_1760450	A/whooper swan/Shaanxi/SXY2-1/2020	A / H5N8 Asia / China / Shaanxi Province	Hongliang Chai (Northeast Forestry University / College of Wildlife Resources)
EPI_ISL_1760451	A/whooper swan/Shanxi/SX16/2020	A / H5N8 Asia / China / Shanxi Province	Hongliang Chai (Northeast Forestry University / College of Wildlife Resources)
EPI_ISL_1760452	A/whooper swan/Shanxi/SX31/2020	A / H5N8 Asia / China / Shanxi Province	Hongliang Chai (Northeast Forestry University / College of Wildlife Resources)
EPI_ISL_1760453	A/whooper swan/Shanxi/SX56/2020	A / H5N8 Asia / China / Shanxi Province	Hongliang Chai (Northeast Forestry University / College of Wildlife Resources)
EPI_ISL_1760454	A/whooper swan/Shanxi/SX106/2020	A / H5N8 Asia / China / Shanxi Province	Hongliang Chai (Northeast Forestry University / College of Wildlife Resources)
EPI_ISL_1760455	A/whooper swan/Shanxi/SX116/2020	A / H5N2 Asia / China / Shanxi Province	Hongliang Chai (Northeast Forestry University / College of Wildlife Resources)
EPI_ISL_176840	A/feline/Guangdong/1/2014(H5N6)	A / H5N6 Asia / China / Guangdong Province	Yuhai Bi (Institute of Microbiology, Chinese Academy of Sciences)
EPI_ISL_176841	A/feline/Guangdong/2/2014(H5N6)	A / H5N6 Asia / China / Guangdong Province	Yuhai Bi (Institute of Microbiology, Chinese Academy of Sciences)
EPI_ISL_177455	A/turkey/BC/FAV14/2014	A / H5N2 North America / Canada / British Columbia	Tamiko Hisanaga (Canadian Food Inspection Agency)
EPI_ISL_177584	A/domestic duck/Hungary/7341/2015	A / H5N8 Europe / Hungary / Bekes megye / Füzesgyarmat	Adam Dan (Danam.Vet.Molbiol)
EPI_ISL_177647	A/turkey/California/K1500169-1.2/2015	A / H5N8 North America / United States	
EPI_ISL_177648	A/pheasant/Washington/3147-2/2015	A / H5N2 North America / United States	
EPI_ISL_177649	A/eurasian wigeon/Netherlands/1/2015	A / H5N8 Europe / Netherlands / Provincie Noord-Holland / Gemeente Purmerend / Ilpendam	Josanne Verhagen (Erasmus Medical Center / Department of Virology)
EPI_ISL_177699	A/duck/Vietnam/1152/2014	A / H5N6 Asia / Vietnam	
EPI_ISL_177700	A/duck/Vietnam/1151/2014	A / H5N6 Asia / Vietnam	
EPI_ISL_177701	A/duck/Vietnam/1507/2014	A / H5N6 Asia / Vietnam	
EPI_ISL_177702	A/duck/Vietnam/1511/2014	A / H5N6 Asia / Vietnam	
EPI_ISL_177703	A/duck/Vietnam/1434/2014	A / H5N6 Asia / Vietnam	
EPI_ISL_177704	A/duck/Vietnam/1144/2014	A / H5N6 Asia / Vietnam	
EPI_ISL_177725	A/baikal teal/Korea/1437/2014	A / H5N8 Asia / Korea, Republic of	
EPI_ISL_177726	A/baikal teal/Korea/1441/2014	A / H5N8 Asia / Korea, Republic of	
EPI_ISL_177727	A/baikal teal/Korea/1445/2014	A / H5N8 Asia / Korea, Republic of	
EPI_ISL_177728	A/baikal teal/Korea/1446/2014	A / H5N8 Asia / Korea, Republic of	
EPI_ISL_177729	A/baikal teal/Korea/1447/2014	A / H5N8 Asia / Korea, Republic of	
EPI_ISL_177730	A/baikal teal/Korea/1448/2014	A / H5N8 Asia / Korea, Republic of	
EPI_ISL_177731	A/baikal teal/Korea/1449/2014	A / H5N8 Asia / Korea, Republic of	
EPI_ISL_177732	A/baikal teal/Korea/1452/2014	A / H5N8 Asia / Korea, Republic of	
EPI_ISL_177733	A/baikal teal/Korea/1454/2014	A / H5N8 Asia / Korea, Republic of	
EPI_ISL_177734	A/baikal teal/Korea/1456/2014	A / H5N8 Asia / Korea, Republic of	
EPI_ISL_177735	A/baikal teal/Korea/1457/2014	A / H5N8 Asia / Korea, Republic of	
EPI_ISL_177736	A/baikal teal/Korea/1458/2014	A / H5N8 Asia / Korea, Republic of	
EPI_ISL_177737	A/baikal teal/Korea/2399/2014	A / H5N8 Asia / Korea, Republic of	
EPI_ISL_177738	A/baikal teal/Korea/2402/2014	A / H5N8 Asia / Korea, Republic of	
EPI_ISL_177739	A/baikal teal/Korea/2403/2014	A / H5N8 Asia / Korea, Republic of	
EPI_ISL_177740	A/baikal teal/Korea/2406/2014	A / H5N8 Asia / Korea, Republic of	
EPI_ISL_177741	A/baikal teal/Korea/2414/2014	A / H5N8 Asia / Korea, Republic of	
EPI_ISL_177742	A/baikal teal/Korea/2416/2014	A / H5N8 Asia / Korea, Republic of	
EPI_ISL_177743	A/baikal teal/Korea/2417/2014	A / H5N8 Asia / Korea, Republic of	
EPI_ISL_177761	A/cat/Sichuan/SC18/2014	A / H5N6 Asia / China	
EPI_ISL_177762	A/swan goose/Jilin/JL01/2014	A / H5N6 Asia / China	
EPI_ISL_177869	A/chicken/TongHai/302/2014	A / H5N1 Asia / China	
EPI_ISL_177989	A/Spotted dove/Vietnam/WBT191/2014	A / H5N6 Asia / Vietnam	
EPI_ISL_177990	A/Black-crowned night heron/Vietnam/WBT198/2014	A / H5N6 Asia / Vietnam	
EPI_ISL_177991	A/Little egret/Vietnam/WBT210/2014	A / H5N6 Asia / Vietnam	
EPI_ISL_177992	A/Common moorhen/Vietnam/WBT226/2014	A / H5N6 Asia / Vietnam	
EPI_ISL_177993	A/Chinese pond heron/Vietnam/WBT231/2014	A / H5N6 Asia / Vietnam	
EPI_ISL_178079	A/poultry/BC/FAV15/2014	A / H5N2 North America / Canada / British Columbia	Tamiko Hisanaga (Canadian Food Inspection Agency)

EPI_ISL_178080	A/poultry/BC/FAV17/2014	A / H5N2 North America / Canada / British Columbia	Tamiko Hisanaga (Canadian Food Inspection Agency)
EPI_ISL_178081	A/poultry/BC/FAV19/2014	A / H5N2 North America / Canada / British Columbia	Tamiko Hisanaga (Canadian Food Inspection Agency)
EPI_ISL_178082	A/chicken/BC/FAV20/2014	A / H5N2 North America / Canada / British Columbia	Tamiko Hisanaga (Canadian Food Inspection Agency)
EPI_ISL_178083	A/chicken/BC/FAV21/2014	A / H5N2 North America / Canada / British Columbia	Tamiko Hisanaga (Canadian Food Inspection Agency)
EPI_ISL_178084	A/chicken/BC/FAV22/2014	A / H5N2 North America / Canada / British Columbia	Tamiko Hisanaga (Canadian Food Inspection Agency)
EPI_ISL_178085	A/chicken/BC/FAV23/2014	A / H5N2 North America / Canada / British Columbia	Tamiko Hisanaga (Canadian Food Inspection Agency)
EPI_ISL_178086	A/chicken/BC/FAV24/2014	A / H5N2 North America / Canada / British Columbia	Tamiko Hisanaga (Canadian Food Inspection Agency)
EPI_ISL_178087	A/chicken/BC/FAV25/2014	A / H5N2 North America / Canada / British Columbia	Tamiko Hisanaga (Canadian Food Inspection Agency)
EPI_ISL_178249	A/American wigeon/BC/050-31/2015	A / H5N8 North America / Canada / British Columbia	Tamiko Hisanaga (Canadian Food Inspection Agency)
EPI_ISL_178250	A/Environment/Yunnan/DQ5/2015	A / H5N6 Asia / China / Yunnan	Lei Yang (WHO Chinese National Influenza Center / Virology Institute, Chinese CDC)
EPI_ISL_178251	A/Environment/Yunnan/DQ8/2015	A / H5N6 Asia / China / Yunnan	Lei Yang (WHO Chinese National Influenza Center / Virology Institute, Chinese CDC)
EPI_ISL_178252	A/Environment/Yunnan/DQ9/2015	A / H5N6 Asia / China / Yunnan	Lei Yang (WHO Chinese National Influenza Center / Virology Institute, Chinese CDC)
EPI_ISL_178253	A/Environment/Yunnan/DQ16/2015	A / H5N6 Asia / China / Yunnan	Lei Yang (WHO Chinese National Influenza Center / Virology Institute, Chinese CDC)
EPI_ISL_178255	A/Environment/Yunnan/DQ53/2015	A / H5N6 Asia / China / Yunnan	Lei Yang (WHO Chinese National Influenza Center / Virology Institute, Chinese CDC)
EPI_ISL_178261	A/Yunnan/14563/2015	A / H5N6 Asia / China / Yunnan	Lei Yang (WHO Chinese National Influenza Center / Virology Institute, Chinese CDC)
EPI_ISL_178262	A/Yunnan/14564/2015	A / H5N6 Asia / China / Yunnan	Lei Yang (WHO Chinese National Influenza Center / Virology Institute, Chinese CDC)
EPI_ISL_178263	A/chicken/Iowa/04-20/2015	A / H5N2 North America / United States	
EPI_ISL_179023	A/goose/Taiwan/a015/2015	A / H5N8 Asia / Taiwan	Yu-Pin Liu (Animal Health Research Institute)
EPI_ISL_179024	A/duck/Taiwan/a043/2015	A / H5N2 Asia / Taiwan	Yu-Pin Liu (Animal Health Research Institute)
EPI_ISL_179025	A/chicken/Taiwan/a174/2015	A / H5N3 Asia / Taiwan	Yu-Pin Liu (Animal Health Research Institute)
EPI_ISL_179026	A/duck/Taiwan/a068/2015	A / H5N8 Asia / Taiwan	Yu-Pin Liu (Animal Health Research Institute)
EPI_ISL_179175	A/muscovy duck/Quang Ninh/4c111/2013	A / H5N6 Asia / Vietnam	
EPI_ISL_179176	A/muscovy duck/Quang Ninh/5c112/2013	A / H5N6 Asia / Vietnam	
EPI_ISL_179374	A/duck/Nha Trang/75c131/2014	A / H5N6 Asia / Vietnam	
EPI_ISL_179395	A/chicken/AnNing/1/2014	A / H5N1 Asia / China	
EPI_ISL_179396	A/chicken/AnNing/4/2014	A / H5N1 Asia / China	
EPI_ISL_179397	A/chicken/AnNing/6/2014	A / H5N1 Asia / China	
EPI_ISL_179398	A/chicken/DaLi/302/2014	A / H5N1 Asia / China	
EPI_ISL_179399	A/chicken/DaLi/502/2014	A / H5N1 Asia / China	
EPI_ISL_179400	A/chicken/Tonghai/3/2014	A / H5N1 Asia / China	
EPI_ISL_179401	A/chicken/Tonghai/5/2014	A / H5N1 Asia / China	
EPI_ISL_179402	A/chicken/Tonghai/802/2014	A / H5N1 Asia / China	
EPI_ISL_179543	A/turkey/Minnesota/9892-2/2015	A / H5N2 North America / United States	
EPI_ISL_179544	A/chicken/Washington/3490-18/2015	A / H5N2 North America / United States	
EPI_ISL_179545	A/chicken/Oregon/A01819044/2015	A / H5N2 North America / United States	
EPI_ISL_179546	A/turkey/Minnesota/7172-1/2015	A / H5N2 North America / United States	
EPI_ISL_179547	A/turkey/Missouri/7458-1/2015	A / H5N2 North America / United States	
EPI_ISL_179548	A/turkey/Arkansas/7791-1/2015	A / H5N2 North America / United States	
EPI_ISL_179549	A/chicken/Kansas/8395-3/2015	A / H5N2 North America / United States	
EPI_ISL_179550	A/turkey/Minnesota/9845-4/2015	A / H5N2 North America / United States	
EPI_ISL_179634	A/duck/Jiangxi/NCDZT1123/2014	A / H5N6 Asia / China	
EPI_ISL_179635	A/duck/Jiangxi/NCDZT1126/2014	A / H5N6 Asia / China	
EPI_ISL_179644	A/Anas_crecca/Hubei/Chenhu1623-5/2014	A / H5N6 Asia / China	
EPI_ISL_179645	A/chicken/Sichuan/NCJPL1/2014	A / H5N6 Asia / China	
EPI_ISL_179646	A/duck/Sichuan/NCJPL7/2014	A / H5N6 Asia / China	
EPI_ISL_179647	A/duck/Sichuan/NCXJ15/2014	A / H5N6 Asia / China	
EPI_ISL_179648	A/duck/Sichuan/NCXJ24/2014	A / H5N6 Asia / China	
EPI_ISL_179649	A/duck/Sichuan/NCXJ16/2014	A / H5N6 Asia / China	
EPI_ISL_179650	A/environment/Sichuan/NCLL1/2014	A / H5N6 Asia / China	
EPI_ISL_179651	A/duck/Sichuan/NCXN10/2014	A / H5N1 Asia / China	
EPI_ISL_179652	A/duck/Sichuan/NCXN11/2014	A / H5N1 Asia / China	
EPI_ISL_179653	A/pigeon/Sichuan/NCXN29/2014	A / H5N1 Asia / China	
EPI_ISL_180495	A/Environment/Jiangxi/20983/2013	A / H5N1 Asia / China / jiangxi	Lei Yang (WHO Chinese National Influenza Center / Virology Institute, Chinese CDC)
EPI_ISL_180499	A/Environment/Jiangxi/23094/2013	A / H5N6 Asia / China / jiangxi	Lei Yang (WHO Chinese National Influenza Center / Virology Institute, Chinese CDC)
EPI_ISL_180649	A/goose/Shandong/k1201/2009	A / H5N1 Asia / China	
EPI_ISL_180655	A/goose/Yangzhou/ZG62/2011	A / H5N5 Asia / China	
EPI_ISL_180656	A/goose/Yangzhou/ZG60/2009	A / H5N5 Asia / China	
EPI_ISL_181080	A/common teal/Korea/KU-12/2015	A / H5N8 Asia / Korea, Republic of / Chungcheongnam-do / Galsangyo	Jung Hoon Kwon (Konkuk University / College of Veterinary Medicine)
EPI_ISL_181081	A/mallard/Korea/KU3-2/2015	A / H5N8 Asia / Korea, Republic of / Gyeongsangnam-do / Junam reservoir	Jung Hoon Kwon (Konkuk University / College of Veterinary Medicine)
EPI_ISL_181082	A/mallard/Korea/N15-99/2015	A / H5N8 Asia / Korea, Republic of / Seoul / Jungnangcheon	Jung Hoon Kwon (Konkuk University / College of Veterinary Medicine)
EPI_ISL_181083	A/mandarin duck/Korea/K14-363-1/2014	A / H5N8 Asia / Korea, Republic of / Chungcheongnam-do / pungsecheon	Jung Hoon Kwon (Konkuk University / College of Veterinary Medicine)
EPI_ISL_181084	A/mandarin duck/Korea/K14-366-1/2014	A / H5N8 Asia / Korea, Republic of / Chungcheongnam-do / pungsecheon	Jung Hoon Kwon (Konkuk University / College of Veterinary Medicine)
EPI_ISL_181085	A/mandarin duck/Korea/K14-367-1/2014	A / H5N8 Asia / Korea, Republic of / Chungcheongnam-do / pungsecheon	Jung Hoon Kwon (Konkuk University / College of Veterinary Medicine)
EPI_ISL_181086	A/greater white-fronted goose/Korea/K14-367-4/2014	A / H5N8 Asia / Korea, Republic of / Chungcheongnam-do / pungsecheon	Jung Hoon Kwon (Konkuk University / College of Veterinary Medicine)
EPI_ISL_181087	A/greater white-fronted goose/Korea/K14-369-3/2014	A / H5N8 Asia / Korea, Republic of / Chungcheongnam-do / pungsecheon	Jung Hoon Kwon (Konkuk University / College of Veterinary Medicine)
EPI_ISL_181088	A/greater white-fronted goose/Korea/K14-371-4/2014	A / H5N8 Asia / Korea, Republic of / Chungcheongnam-do / pungsecheon	Jung Hoon Kwon (Konkuk University / College of Veterinary Medicine)
EPI_ISL_181089	A/greater white-fronted goose/Korea/K14-372-2/2014	A / H5N8 Asia / Korea, Republic of / Chungcheongnam-do / pungsecheon	Jung Hoon Kwon (Konkuk University / College of Veterinary Medicine)
EPI_ISL_181090	A/greater white-fronted goose/Korea/K14-374-1/2014	A / H5N8 Asia / Korea, Republic of / Chungcheongnam-do / pungsecheon	Jung Hoon Kwon (Konkuk University / College of Veterinary Medicine)
EPI_ISL_181093	A/eurasian wigeon/Netherlands/1/2014	A / H5N8 Europe / Netherlands	
EPI_ISL_181094	A/eurasian wigeon/Netherlands/2/2014	A / H5N8 Europe / Netherlands	
EPI_ISL_181127	A/duck/Vietnam/LBM751/2014	A / H5N6 Asia / Vietnam	
EPI_ISL_181128	A/duck/Vietnam/LBM752/2014	A / H5N6 Asia / Vietnam	

EPI_ISL_181129	A/muscovy duck/Vietnam/LBM754/2014	A / H5N6	Asia / Vietnam	
EPI_ISL_181130	A/muscovy duck/Vietnam/LBM755/2014	A / H5N6	Asia / Vietnam	
EPI_ISL_181131	A/muscovy duck/Vietnam/LBM756/2014	A / H5N6	Asia / Vietnam	
EPI_ISL_181132	A/muscovy duck/Vietnam/LBM757/2014	A / H5N6	Asia / Vietnam	
EPI_ISL_181133	A/duck/Vietnam/LBM758/2014	A / H5N6	Asia / Vietnam	
EPI_ISL_181134	A/duck/Vietnam/LBM759/2014	A / H5N6	Asia / Vietnam	
EPI_ISL_181135	A/duck/Vietnam/LBM760/2014	A / H5N6	Asia / Vietnam	
EPI_ISL_181665	A/chicken/Shenzhen/715/2013	A / H5N6	Asia / China	
EPI_ISL_181666	A/chicken/Shenzhen/1061/2013	A / H5N6	Asia / China	
EPI_ISL_181667	A/chicken/Shenzhen/2269/2013	A / H5N6	Asia / China	
EPI_ISL_181668	A/chicken/Dongguan/2690/2013	A / H5N6	Asia / China	
EPI_ISL_181669	A/chicken/Dongguan/3363/2013	A / H5N6	Asia / China	
EPI_ISL_182059	A/chicken/Shenzhen/433/2013	A / H5N6	Asia / China	
EPI_ISL_182060	A/chicken/Shenzhen/1395/2013	A / H5N6	Asia / China	
EPI_ISL_182061	A/chicken/Shenzhen/1845/2013	A / H5N6	Asia / China	
EPI_ISL_182062	A/chicken/Shenzhen/2396/2013	A / H5N6	Asia / China	
EPI_ISL_182063	A/chicken/Shenzhen/2464/2013	A / H5N6	Asia / China	
EPI_ISL_182064	A/duck/Dongguan/2685/2013	A / H5N6	Asia / China	
EPI_ISL_182065	A/silkie chicken/Dongguan/2809/2013	A / H5N6	Asia / China	
EPI_ISL_182066	A/duck/Dongguan/3069/2013	A / H5N6	Asia / China	
EPI_ISL_182067	A/chicken/Dongguan/4259/2013	A / H5N6	Asia / China	
EPI_ISL_182068	A/duck/Jiangxi/10160/2014	A / H5N6	Asia / China	
EPI_ISL_182069	A/environment/Jiangxi/10164/2014	A / H5N6	Asia / China	
EPI_ISL_182070	A/environment/Jiangxi/10171/2014	A / H5N6	Asia / China	
EPI_ISL_182086	A/chicken/Shenzhen/552/2013	A / H5N6	Asia / China	
EPI_ISL_1822592	A/whooper swan/Shanxi/SX166/2020	A / H5N8	Asia / China / Shanxi Province	Hongliang Chai (Northeast Forestry University / College of Wildlife Resources)
EPI_ISL_1822593	A/whooper swan/Shanxi/SX206/2020	A / H5N8	Asia / China / Shanxi Province	Hongliang Chai (Northeast Forestry University / College of Wildlife Resources)
EPI_ISL_1822594	A/whooper swan/Shanxi/SX216/2020	A / H5N8	Asia / China / Shanxi Province	Hongliang Chai (Northeast Forestry University / College of Wildlife Resources)
EPI_ISL_1822595	A/whooper swan/Shanxi/SX231/2020	A / H5N8	Asia / China / Shanxi Province	Hongliang Chai (Northeast Forestry University / College of Wildlife Resources)
EPI_ISL_1822596	A/whooper swan/Shanxi/SX251/2020	A / H5N8	Asia / China / Shanxi Province	Hongliang Chai (Northeast Forestry University / College of Wildlife Resources)
EPI_ISL_1822597	A/whooper swan/Shanxi/SX276/2020	A / H5N8	Asia / China / Shanxi Province	Hongliang Chai (Northeast Forestry University / College of Wildlife Resources)
EPI_ISL_1822598	A/whooper swan/Shanxi/SX291/2020	A / H5N8	Asia / China / Shanxi Province	Hongliang Chai (Northeast Forestry University / College of Wildlife Resources)
EPI_ISL_1822599	A/whooper swan/Shanxi/SX346/2020	A / H5N8	Asia / China / Shanxi Province	Hongliang Chai (Northeast Forestry University / College of Wildlife Resources)
EPI_ISL_1829205	A/whooper swan/Henan/SMQ5/2020	A / H5N8	Asia / China / Henan Province	Hongliang Chai (Northeast Forestry University / College of Wildlife Resources)
EPI_ISL_1834185	A/whooper swan/Henan/SMQ6/2020	A / H5N8	Asia / China / Henan Province	Hongliang Chai (Northeast Forestry University / College of Wildlife Resources)
EPI_ISL_190456	A/turkey/Iowa/11762-1/2015	A / H5N2	North America / United States	
EPI_ISL_190457	A/turkey/Iowa/13541-1/2015	A / H5N2	North America / United States	
EPI_ISL_190458	A/chicken/Iowa/13542-2/2015	A / H5N2	North America / United States	
EPI_ISL_190459	A/turkey/Iowa/14319-1/2015	A / H5N2	North America / United States	
EPI_ISL_190460	A/turkey/Iowa/14318-1/2015	A / H5N2	North America / United States	
EPI_ISL_190461	A/chicken/Iowa/14322-6/2015	A / H5N2	North America / United States	
EPI_ISL_190462	A/chicken/Iowa/14399-4/2015	A / H5N2	North America / United States	
EPI_ISL_190464	A/chicken/Iowa/14589-1/2015	A / H5N2	North America / United States	
EPI_ISL_191765	A/scarlet_ibis/Germany/AR44-L01279/2015	A / H5N8	Europe / Germany / Mecklenburg-Vorpommern / zoo	Anne Pohlmann (Friedrich-Loeffler-Institut)
EPI_ISL_1937883	A/whooper swan/Henan/SMQ7/2020	A / H5N8	Asia / China / Henan Province	Hongliang Chai (Northeast Forestry University / College of Wildlife Resources)
EPI_ISL_1937884	A/whooper swan/Henan/SM1/2020	A / H5N8	Asia / China / Henan Province	Hongliang Chai (Northeast Forestry University / College of Wildlife Resources)
EPI_ISL_1937889	A/whooper swan/Henan/SM16/2020	A / H5N8	Asia / China / Henan Province	Hongliang Chai (Northeast Forestry University / College of Wildlife Resources)
EPI_ISL_1937890	A/whooper swan/Henan/SM31/2020	A / H5N8	Asia / China / Henan Province	Hongliang Chai (Northeast Forestry University / College of Wildlife Resources)
EPI_ISL_1937891	A/whooper swan/Henan/SM61/2020	A / H5N8	Asia / China / Henan Province	Hongliang Chai (Northeast Forestry University / College of Wildlife Resources)
EPI_ISL_1937892	A/whooper swan/Henan/SM76/2020	A / H5N8	Asia / China / Henan Province	Hongliang Chai (Northeast Forestry University / College of Wildlife Resources)
EPI_ISL_1938307	A/whooper swan/Henan/SM86/2020	A / H5N8	Asia / China / Henan Province	Hongliang Chai (Northeast Forestry University / College of Wildlife Resources)
EPI_ISL_1938309	A/whooper swan/Henan/SM111/2020	A / H5N8	Asia / China / Henan Province	Hongliang Chai (Northeast Forestry University / College of Wildlife Resources)
EPI_ISL_1938478	A/whooper swan/Henan/SMQ9/2020	A / H5N8	Asia / China / Henan Province	Hongliang Chai (Northeast Forestry University / College of Wildlife Resources)
EPI_ISL_1939080	A/whooper swan/Henan/SMQ10/2020	A / H5N8	Asia / China / Henan Province	Hongliang Chai (Northeast Forestry University / College of Wildlife Resources)
EPI_ISL_1939617	A/eurasian eagle-owl/Henan/SMQ11/2020	A / H5N8	Asia / China / Henan Province	Hongliang Chai (Northeast Forestry University / College of Wildlife Resources)
EPI_ISL_1941351	A/duck/Czech Republic/7682-9/2021	A / H5N8	Europe / Czech Republic / Kralovehradecky Kraj / Okres Hradec Kralove / Nove Mesto	Alexander Nagy (State Veterinary Institute Prague)
EPI_ISL_1941365	A/duck/Czech Republic/7682-5/2021	A / H5N8	Europe / Czech Republic / Kralovehradecky Kraj / Okres Hradec Kralove / Nove Mesto	Alexander Nagy (State Veterinary Institute Prague)
EPI_ISL_1941375	A/duck/Czech Republic/7682-2/2021	A / H5N8	Europe / Czech Republic / Kralovehradecky Kraj / Okres Hradec Kralove / Nove Mesto	Alexander Nagy (State Veterinary Institute Prague)
EPI_ISL_1941417	A/duck/Czech Republic/7681-8/2021	A / H5N8	Europe / Czech Republic / Kralovehradecky Kraj / Okres Hradec Kralove / Klamos	Alexander Nagy (State Veterinary Institute Prague)
EPI_ISL_1941444	A/duck/Czech Republic/7681-7/2021	A / H5N8	Europe / Czech Republic / Kralovehradecky Kraj / Okres Hradec Kralove / Klamos	Alexander Nagy (State Veterinary Institute Prague)
EPI_ISL_1941480	A/duck/Czech Republic/7681-5/2021	A / H5N8	Europe / Czech Republic / Kralovehradecky Kraj / Okres Hradec Kralove / Klamos	Alexander Nagy (State Veterinary Institute Prague)
EPI_ISL_1941542	A/duck/Czech Republic/7681-3/2021	A / H5N8	Europe / Czech Republic / Kralovehradecky Kraj / Okres Hradec Kralove / Klamos	Alexander Nagy (State Veterinary Institute Prague)
EPI_ISL_1941578	A/duck/Czech Republic/7681-10/2021	A / H5N8	Europe / Czech Republic / Kralovehradecky Kraj / Okres Hradec Kralove / Klamos	Alexander Nagy (State Veterinary Institute Prague)
EPI_ISL_1941579	A/duck/Czech Republic/6653-5/2021	A / H5N8	Europe / Czech Republic / Kralovehradecky Kraj / Okres Hradec Kralove / Stary Bydzov	Alexander Nagy (State Veterinary Institute Prague)
EPI_ISL_1941580	A/duck/Czech Republic/6653-15/2021	A / H5N8	Europe / Czech Republic / Kralovehradecky Kraj / Okres Hradec Kralove / Stary Bydzov	Alexander Nagy (State Veterinary Institute Prague)
EPI_ISL_1941581	A/goose/Czech Republic/5363-10/2021	A / H5N8	Europe / Czech Republic / Plzensky Kraj / Okres Plzen-Jih / Zbuch, pond; GPS: 49.6897778N, 13.2	Alexander Nagy (State Veterinary Institute Prague)
EPI_ISL_1941582	A/chicken/Czech Republic/7100/2021	A / H5N8	Europe / Czech Republic / Jihocesky Kraj / Okres Strakonice / Vodnany	Alexander Nagy (State Veterinary Institute Prague)
EPI_ISL_195294	A/Yunnan/0127/2015	A / H5N6	Asia / China	
EPI_ISL_195308	A/chicken/Yunnan/19/2015	A / H5N6	Asia / China	
EPI_ISL_195309	A/chicken/Yunnan/22/2015	A / H5N6	Asia / China	
EPI_ISL_195310	A/chicken/Yunnan/25A/2015	A / H5N6	Asia / China	
EPI_ISL_195769	A/Environment/Guangdong/GZ55/2013(H5N8)	A / H5N8	Asia / China / Guangdong Province	Xianqiao Zeng (Guangdong Provincial Center for Disease Control and Prevention)

EPI_ISL_196100	A/swine/Guangdong/1/2014	A / H5N6	Asia / China	
EPI_ISL_196101	A/swine/Guangdong/2/2014	A / H5N6	Asia / China	
EPI_ISL_196571	A/duck/Jiangsu/WX156/2013	A / H5N6	Asia / China / Jiangsu Provice	Maijuan Ma (Beijing Institute of Microbiology and Epidemiology / State Key Laboratory of Pathogen and Biosecurity)
EPI_ISL_196572	A/environment/Jiangsu/WX175/2013	A / H5N6	Asia / China / Jiangsu Provice	Maijuan Ma (Beijing Institute of Microbiology and Epidemiology / State Key Laboratory of Pathogen and Biosecurity)
EPI_ISL_196573	A/chicken/Jiangsu/WXS7393/2014	A / H5N6	Asia / China / Jiangsu Provice	Maijuan Ma (Beijing Institute of Microbiology and Epidemiology / State Key Laboratory of Pathogen and Biosecurity)
EPI_ISL_196591	A/chicken/Jiangsu/WXBING2/2014	A / H5N6	Asia / China / Jiangsu Provice	Maijuan Ma (Beijing Institute of Microbiology and Epidemiology / State Key Laboratory of Pathogen and Biosecurity)
EPI_ISL_196592	A/goose/Jiangsu/WX202/2014	A / H5N8	Asia / China / Jiangsu Provice	Maijuan Ma (Beijing Institute of Microbiology and Epidemiology / State Key Laboratory of Pathogen and Biosecurity)
EPI_ISL_198663	A/turkey/Germany/AR3382-L00937/2014	A / H5N8	Europe / Germany / Lower Saxony	Anne Pohlmann (Friedrich-Loeffler-Institut)
EPI_ISL_198665	A/turkey/Germany/AR3390-L00939/2014	A / H5N8	Europe / Germany / Lower Saxony	Anne Pohlmann (Friedrich-Loeffler-Institut)
EPI_ISL_198754	A/duck/Hunan/12.17 YYFQH012-O/2014(H5N6)	A / H5N6	Asia / China	Yuhai Bi (Institute of Microbiology, Chinese Academy of Sciences)
EPI_ISL_198757	A/goose/Jilin/04.04 SY003-O/2015(H5N6)	A / H5N6	Asia / China	Yuhai Bi (Institute of Microbiology, Chinese Academy of Sciences)
EPI_ISL_198758	A/chicken/Jiangxi/04.01 NCJD131-1-O/2015(H5N6)	A / H5N6	Asia / China	Yuhai Bi (Institute of Microbiology, Chinese Academy of Sciences)
EPI_ISL_198761	A/chicken/Hunan/12.07 YYGK114-P/2013(H5N6)	A / H5N6	Asia / China	Yuhai Bi (Institute of Microbiology, Chinese Academy of Sciences)
EPI_ISL_198762	A/chicken/Hunan/12.28 YYGK034-P/2013(H5N6)	A / H5N6	Asia / China	Yuhai Bi (Institute of Microbiology, Chinese Academy of Sciences)
EPI_ISL_198765	A/duck/Hunan/12.07 YYGK108-P/2013(H5N6)	A / H5N6	Asia / China	Yuhai Bi (Institute of Microbiology, Chinese Academy of Sciences)
EPI_ISL_198766	A/duck/Hunan/12.07 YYGK110-P/2013(H5N6)	A / H5N6	Asia / China	Yuhai Bi (Institute of Microbiology, Chinese Academy of Sciences)
EPI_ISL_198767	A/duck/Hunan/12.07 YYGK111-P/2013(H5N6)	A / H5N6	Asia / China	Yuhai Bi (Institute of Microbiology, Chinese Academy of Sciences)
EPI_ISL_198768	A/duck/Hunan/12.07 YYGK112-P/2013(H5N6)	A / H5N6	Asia / China	Yuhai Bi (Institute of Microbiology, Chinese Academy of Sciences)
EPI_ISL_198769	A/duck/Hunan/12.07 YYGK113-P/2013(H5N6)	A / H5N6	Asia / China	Yuhai Bi (Institute of Microbiology, Chinese Academy of Sciences)
EPI_ISL_198770	A/duck/Hunan/12.07 YYGK118-P/2013(H5N6)	A / H5N6	Asia / China	Yuhai Bi (Institute of Microbiology, Chinese Academy of Sciences)
EPI_ISL_198771	A/duck/Hunan/12.07 YYGK032-P/2013(H5N6)	A / H5N6	Asia / China	Yuhai Bi (Institute of Microbiology, Chinese Academy of Sciences)
EPI_ISL_198772	A/duck/Hunan/12.07 YYGK0043-P/2013(H5N6)	A / H5N6	Asia / China	Yuhai Bi (Institute of Microbiology, Chinese Academy of Sciences)
EPI_ISL_198773	A/duck/Hunan/12.07 YYGK68-P/2013(H5N6)	A / H5N6	Asia / China	Yuhai Bi (Institute of Microbiology, Chinese Academy of Sciences)
EPI_ISL_198777	A/duck/Hunan/12.07 YGK098-P/2013(H5N6)	A / H5N6	Asia / China	Yuhai Bi (Institute of Microbiology, Chinese Academy of Sciences)
EPI_ISL_198781	A/chicken/Hunan/02.26 YYGKJ281-P/2014(H5N6)	A / H5N6	Asia / China	Yuhai Bi (Institute of Microbiology, Chinese Academy of Sciences)
EPI_ISL_198782	A/chicken/Hunan/02.26 YYGKJ295-P/2014(H5N6)	A / H5N6	Asia / China	Yuhai Bi (Institute of Microbiology, Chinese Academy of Sciences)
EPI_ISL_198783	A/duck/Hunan/01.16 YYFQH185-P/2014(H5N6)	A / H5N6	Asia / China	Yuhai Bi (Institute of Microbiology, Chinese Academy of Sciences)
EPI_ISL_198784	A/duck/Hunan/01.16 YYGK0204-P/2014(H5N6)	A / H5N6	Asia / China	Yuhai Bi (Institute of Microbiology, Chinese Academy of Sciences)
EPI_ISL_198785	A/duck/Hunan/01.16 YYGK218-P/2014(H5N6)	A / H5N6	Asia / China	Yuhai Bi (Institute of Microbiology, Chinese Academy of Sciences)
EPI_ISL_198789	A/duck/Hunan/02.26 YYGK249-P/2014(H5N6)	A / H5N6	Asia / China	Yuhai Bi (Institute of Microbiology, Chinese Academy of Sciences)
EPI_ISL_198791	A/duck/Hunan/02.26 YYGK250-P/2014(H5N6)	A / H5N6	Asia / China	Yuhai Bi (Institute of Microbiology, Chinese Academy of Sciences)
EPI_ISL_198792	A/duck/Hunan/02.26 YYGK251-P/2014(H5N6)	A / H5N6	Asia / China	Yuhai Bi (Institute of Microbiology, Chinese Academy of Sciences)
EPI_ISL_198793	A/duck/Hunan/02.26 YYGK254-P/2014(H5N6)	A / H5N6	Asia / China	Yuhai Bi (Institute of Microbiology, Chinese Academy of Sciences)
EPI_ISL_198794	A/duck/Hunan/02.26 YYGK0255-P/2014(H5N6)	A / H5N6	Asia / China	Yuhai Bi (Institute of Microbiology, Chinese Academy of Sciences)
EPI_ISL_198795	A/duck/Hunan/02.26 YYGK263-P/2014(H5N6)	A / H5N6	Asia / China	Yuhai Bi (Institute of Microbiology, Chinese Academy of Sciences)
EPI_ISL_198796	A/duck/Hunan/02.26 YYGK265-P/2014(H5N6)	A / H5N6	Asia / China	Yuhai Bi (Institute of Microbiology, Chinese Academy of Sciences)
EPI_ISL_198797	A/duck/Hunan/02.26 YYGK266-P/2014(H5N6)	A / H5N6	Asia / China	Yuhai Bi (Institute of Microbiology, Chinese Academy of Sciences)
EPI_ISL_198798	A/environment/Jiangxi/02.10 NCDZT023/2015(H5N6)	A / H5N6	Asia / China	Yuhai Bi (Institute of Microbiology, Chinese Academy of Sciences)
EPI_ISL_198799	A/Pigeon/Jiangxi/02.10 NCDZT0017-P/2015(H5N6)	A / H5N6	Asia / China	Yuhai Bi (Institute of Microbiology, Chinese Academy of Sciences)
EPI_ISL_198800	A/environment/Jiangxi/02.10 NCDZT009/2015(H5N6)	A / H5N6	Asia / China	Yuhai Bi (Institute of Microbiology, Chinese Academy of Sciences)
EPI_ISL_198801	A/duck/Hunan/02.26 YYGK267-P/2014(H5N6)	A / H5N6	Asia / China	Yuhai Bi (Institute of Microbiology, Chinese Academy of Sciences)
EPI_ISL_198802	A/duck/Hunan/02.26 YYGK271-P/2014(H5N6)	A / H5N6	Asia / China	Yuhai Bi (Institute of Microbiology, Chinese Academy of Sciences)
EPI_ISL_198803	A/duck/Hunan/02.26 YYGK275/2014(H5N6)	A / H5N6	Asia / China	Yuhai Bi (Institute of Microbiology, Chinese Academy of Sciences)
EPI_ISL_198804	A/duck/Hunan/02.26 YYGK276-P/2014(H5N6)	A / H5N6	Asia / China	Yuhai Bi (Institute of Microbiology, Chinese Academy of Sciences)
EPI_ISL_198805	A/duck/Hunan/02.16 YYGK281-P/2014(H5N6)	A / H5N6	Asia / China	Yuhai Bi (Institute of Microbiology, Chinese Academy of Sciences)
EPI_ISL_198806	A/duck/Hunan/02.26 YYGK283-P/2014(H5N6)	A / H5N6	Asia / China	Yuhai Bi (Institute of Microbiology, Chinese Academy of Sciences)
EPI_ISL_198807	A/duck/Hunan/02.26 YYGK286-P/2014(H5N6)	A / H5N6	Asia / China	Yuhai Bi (Institute of Microbiology, Chinese Academy of Sciences)
EPI_ISL_198808	A/duck/Hunan/02.26 YYGK289-P/2014(H5N6)	A / H5N6	Asia / China	Yuhai Bi (Institute of Microbiology, Chinese Academy of Sciences)
EPI_ISL_198809	A/duck/Hunan/02.26 YYFQH290-P/2014(H5N6)	A / H5N6	Asia / China	Yuhai Bi (Institute of Microbiology, Chinese Academy of Sciences)
EPI_ISL_198810	A/duck/Hunan/02.26 YYFQHY291-P/2014(H5N6)	A / H5N6	Asia / China	Yuhai Bi (Institute of Microbiology, Chinese Academy of Sciences)
EPI_ISL_198811	A/duck/Hunan/02.26 YYFQH293-P/2014(H5N6)	A / H5N6	Asia / China	Yuhai Bi (Institute of Microbiology, Chinese Academy of Sciences)
EPI_ISL_198812	A/duck/Hunan/02.26 YYFQH294-P/2014(H5N6)	A / H5N6	Asia / China	Yuhai Bi (Institute of Microbiology, Chinese Academy of Sciences)
EPI_ISL_198813	A/duck/Hunan/02.26 YYGQH295-P/2014(H5N6)	A / H5N6	Asia / China	Yuhai Bi (Institute of Microbiology, Chinese Academy of Sciences)
EPI_ISL_198814	A/duck/Hunan/02.26 YYFQH297-P/2014(H5N6)	A / H5N6	Asia / China	Yuhai Bi (Institute of Microbiology, Chinese Academy of Sciences)
EPI_ISL_198815	A/duck/Hunan/02.26 YYGKY298-P/2014(H5N6)	A / H5N6	Asia / China	Yuhai Bi (Institute of Microbiology, Chinese Academy of Sciences)
EPI_ISL_198816	A/duck/Hunan/01.16 YYFQH299-P/2014(H5N6)	A / H5N6	Asia / China	Yuhai Bi (Institute of Microbiology, Chinese Academy of Sciences)
EPI_ISL_198817	A/duck/Hunan/01.16 YYFQH300-P/2014(H5N6)	A / H5N6	Asia / China	Yuhai Bi (Institute of Microbiology, Chinese Academy of Sciences)
EPI_ISL_198818	A/duck/Hunan/01.16 YYFQH302-P/2014(H5N6)	A / H5N6	Asia / China	Yuhai Bi (Institute of Microbiology, Chinese Academy of Sciences)
EPI_ISL_198819	A/duck/Hunan/01.16 YYFQH303-P/2014(H5N6)	A / H5N6	Asia / China	Yuhai Bi (Institute of Microbiology, Chinese Academy of Sciences)
EPI_ISL_198820	A/duck/Hunan/01.16 YYFQH304-P/2014(H5N6)	A / H5N6	Asia / China	Yuhai Bi (Institute of Microbiology, Chinese Academy of Sciences)
EPI_ISL_198821	A/duck/Hunan/01.16 YYFQH305-P/2014(H5N6)	A / H5N6	Asia / China	Yuhai Bi (Institute of Microbiology, Chinese Academy of Sciences)
EPI_ISL_198822	A/duck/Hunan/01.16 YYFQH306-P/2014(H5N6)	A / H5N6	Asia / China	Yuhai Bi (Institute of Microbiology, Chinese Academy of Sciences)
EPI_ISL_198823	A/duck/Hunan/01.16 YYFQH307-P/2014(H5N6)	A / H5N6	Asia / China	Yuhai Bi (Institute of Microbiology, Chinese Academy of Sciences)
EPI_ISL_198824	A/duck/Hunan/02.26 YYFQH308-P/2014(H5N6)	A / H5N6	Asia / China	Yuhai Bi (Institute of Microbiology, Chinese Academy of Sciences)
EPI_ISL_198825	A/duck/Hunan/01.16 YYFQH309-P/2014(H5N6)	A / H5N6	Asia / China	Yuhai Bi (Institute of Microbiology, Chinese Academy of Sciences)
EPI_ISL_198826	A/duck/Hunan/01.16 YYFQH310-P/2014(H5N6)	A / H5N6	Asia / China	Yuhai Bi (Institute of Microbiology, Chinese Academy of Sciences)
EPI_ISL_198827	A/duck/Hunan/02.26 YYFQH311-P/2014(H5N6)	A / H5N6	Asia / China	Yuhai Bi (Institute of Microbiology, Chinese Academy of Sciences)
EPI_ISL_198828	A/duck/Hunan/01.16 YYFQH315-P/2014(H5N6)	A / H5N6	Asia / China	Yuhai Bi (Institute of Microbiology, Chinese Academy of Sciences)
EPI_ISL_198829	A/duck/Hunan/01.16 YYFQH317-P/2014(H5N6)	A / H5N6	Asia / China	Yuhai Bi (Institute of Microbiology, Chinese Academy of Sciences)
EPI_ISL_198830	A/duck/Hunan/01.16 YYFQH318-P/2014(H5N6)	A / H5N6	Asia / China	Yuhai Bi (Institute of Microbiology, Chinese Academy of Sciences)
EPI_ISL_198831	A/duck/Hunan/01.16 YYFQH319-P/2014(H5N6)	A / H5N6	Asia / China	Yuhai Bi (Institute of Microbiology, Chinese Academy of Sciences)
EPI_ISL_198832	A/duck/Hunan/02.26 YYFQH320-P/2014(H5N6)	A / H5N6	Asia / China	Yuhai Bi (Institute of Microbiology, Chinese Academy of Sciences)

EPI_ISL_198833	A/duck/Hunan/12.17 YYGK014/2014(H5N6)	A / H5N6	Asia / China	Yuhai Bi (Institute of Microbiology, Chinese Academy of Sciences)
EPI_ISL_198835	A/environment/Jiangxi/02.10 NCDZT0018/2015(H5N6)	A / H5N6	Asia / China	Yuhai Bi (Institute of Microbiology, Chinese Academy of Sciences)
EPI_ISL_198836	A/environment/Jiangxi/02.10 NCDZT018/2015(H5N6)	A / H5N6	Asia / China	Yuhai Bi (Institute of Microbiology, Chinese Academy of Sciences)
EPI_ISL_198838	A/environment/JiangXi/02.10 NCNP0019/2015(H5N6)	A / H5N6	Asia / China	Yuhai Bi (Institute of Microbiology, Chinese Academy of Sciences)
EPI_ISL_198849	A/pigeon/Jilin/04.25 CCHL018-O/2015(H5N6)	A / H5N6	Asia / China	Yuhai Bi (Institute of Microbiology, Chinese Academy of Sciences)
EPI_ISL_198856	A/chicken/Jiangxi/04.01 NCDZT0040-O/2015(H5N6)	A / H5N6	Asia / China	Yuhai Bi (Institute of Microbiology, Chinese Academy of Sciences)
EPI_ISL_198857	A/duck/Hunan/12.17 YYGK040/2014(H5N6)	A / H5N6	Asia / China	Yuhai Bi (Institute of Microbiology, Chinese Academy of Sciences)
EPI_ISL_198860	A/duck/Hunan/12.17 YYFQH052-P/2014(H5N6)	A / H5N6	Asia / China	Yuhai Bi (Institute of Microbiology, Chinese Academy of Sciences)
EPI_ISL_198861	A/chicken/Hunan/04.14 YYKG0533-Z-O/2015(H5N6)	A / H5N6	Asia / China	Yuhai Bi (Institute of Microbiology, Chinese Academy of Sciences)
EPI_ISL_198862	A/duck/Hunan/12.17 YYGK056/2014(H5N6)	A / H5N6	Asia / China	Yuhai Bi (Institute of Microbiology, Chinese Academy of Sciences)
EPI_ISL_198863	A/duck/Hunan/12.17 YYGK0057/2014(H5N6)	A / H5N6	Asia / China	Yuhai Bi (Institute of Microbiology, Chinese Academy of Sciences)
EPI_ISL_198866	A/duck/Sichuan/04.27 NBXJ717/2014(H5N6)	A / H5N6	Asia / China	Yuhai Bi (Institute of Microbiology, Chinese Academy of Sciences)
EPI_ISL_198867	A/duck/Sichuan/04.27 NBXJ718/2014(H5N6)	A / H5N6	Asia / China	Yuhai Bi (Institute of Microbiology, Chinese Academy of Sciences)
EPI_ISL_198868	A/duck/Sichuan/04.27 NBXJ71/2014(H5N6)	A / H5N6	Asia / China	Yuhai Bi (Institute of Microbiology, Chinese Academy of Sciences)
EPI_ISL_198869	A/duck/Sichuan/04.27 NBXJ720/2014(H5N6)	A / H5N6	Asia / China	Yuhai Bi (Institute of Microbiology, Chinese Academy of Sciences)
EPI_ISL_198870	A/duck/Sichuan/04.27 NBXJ731/2014(H5N6)	A / H5N6	Asia / China	Yuhai Bi (Institute of Microbiology, Chinese Academy of Sciences)
EPI_ISL_198871	A/duck/Sichuan/04.27 NCXJ073/2014(H5N6)	A / H5N6	Asia / China	Yuhai Bi (Institute of Microbiology, Chinese Academy of Sciences)
EPI_ISL_198872	A/duck/Sichuan/04.27 NBXJ79/2014(H5N6)	A / H5N6	Asia / China	Yuhai Bi (Institute of Microbiology, Chinese Academy of Sciences)
EPI_ISL_198873	A/duck/Hunan/12.17 YYGK079-1/2014(H5N6)	A / H5N6	Asia / China	Yuhai Bi (Institute of Microbiology, Chinese Academy of Sciences)
EPI_ISL_198874	A/duck/Sichuan/04.27 NBXJ713/2014(H5N6)	A / H5N6	Asia / China	Yuhai Bi (Institute of Microbiology, Chinese Academy of Sciences)
EPI_ISL_198875	A/environment/Jilin/04.11 CCHSL012/2015(H5N6)	A / H5N6	Asia / China	Yuhai Bi (Institute of Microbiology, Chinese Academy of Sciences)
EPI_ISL_198876	A/duck/Hunan/12.17 YYGK082/2014(H5N6)	A / H5N6	Asia / China	Yuhai Bi (Institute of Microbiology, Chinese Academy of Sciences)
EPI_ISL_198878	A/environment/Jilin/04.25 NA004/2015(H5N6)	A / H5N6	Asia / China	Yuhai Bi (Institute of Microbiology, Chinese Academy of Sciences)
EPI_ISL_198880	A/chicken/Yunnan/03.16 DQXYL0034-O/2015(H5N6)	A / H5N6	Asia / China	Yuhai Bi (Institute of Microbiology, Chinese Academy of Sciences)
EPI_ISL_198881	A/chicken/Yunnan/03.16 DQJT0048-O/2015(H5N6)	A / H5N6	Asia / China	Yuhai Bi (Institute of Microbiology, Chinese Academy of Sciences)
EPI_ISL_198882	A/duck/Yunnan/03.16 DQXYL0039-O/2015(H5N6)	A / H5N6	Asia / China	Yuhai Bi (Institute of Microbiology, Chinese Academy of Sciences)
EPI_ISL_198883	A/duck/Yunnan/03.15 DQWGH005-O/2015(H5N6)	A / H5N6	Asia / China	Yuhai Bi (Institute of Microbiology, Chinese Academy of Sciences)
EPI_ISL_198884	A/chicken/Jiangxi/12.05 NCDZT0012/2014(H5N6)	A / H5N6	Asia / China	Yuhai Bi (Institute of Microbiology, Chinese Academy of Sciences)
EPI_ISL_198885	A/chicken/Jiangxi/12.05 NCDZT0020/2014(H5N6)	A / H5N6	Asia / China	Yuhai Bi (Institute of Microbiology, Chinese Academy of Sciences)
EPI_ISL_198886	A/chicken/Jiangxi/12.05 NCDZT0021/2014(H5N6)	A / H5N6	Asia / China	Yuhai Bi (Institute of Microbiology, Chinese Academy of Sciences)
EPI_ISL_198887	A/chicken/Jiangxi/12.05 NCDZT0031/2014(H5N6)	A / H5N6	Asia / China	Yuhai Bi (Institute of Microbiology, Chinese Academy of Sciences)
EPI_ISL_198888	A/chicken/Jiangxi/12.05 NCDZT0032/2014(H5N6)	A / H5N6	Asia / China	Yuhai Bi (Institute of Microbiology, Chinese Academy of Sciences)
EPI_ISL_198889	A/chicken/Jiangxi/12.05 NCDZT0040/2014(H5N6)	A / H5N6	Asia / China	Yuhai Bi (Institute of Microbiology, Chinese Academy of Sciences)
EPI_ISL_198890	A/chicken/Jiangxi/12.05 NCDZT007/2014(H5N6)	A / H5N6	Asia / China	Yuhai Bi (Institute of Microbiology, Chinese Academy of Sciences)
EPI_ISL_198891	A/chicken/Jiangxi/12.05 NCDZT009/2014(H5N6)	A / H5N6	Asia / China	Yuhai Bi (Institute of Microbiology, Chinese Academy of Sciences)
EPI_ISL_198893	A/pigeon/Sichuan/04.27 NBXJ743/2014(H5N6)	A / H5N6	Asia / China	Yuhai Bi (Institute of Microbiology, Chinese Academy of Sciences)
EPI_ISL_198894	A/environment/Jiangxi/02.10 NCDZT16/2015(H5N6)	A / H5N6	Asia / China	Yuhai Bi (Institute of Microbiology, Chinese Academy of Sciences)
EPI_ISL_198895	A/environment/Yunnan/03.15 DQWGH012/2015(H5N6)	A / H5N6	Asia / China	Yuhai Bi (Institute of Microbiology, Chinese Academy of Sciences)
EPI_ISL_198896	A/environment/Yunnan/03.15 DQWGH010/2015(H5N6)	A / H5N6	Asia / China	Yuhai Bi (Institute of Microbiology, Chinese Academy of Sciences)
EPI_ISL_198897	A/environment/Yunnan/03.15 DQWGH011-2/2015(H5N6)	A / H5N6	Asia / China	Yuhai Bi (Institute of Microbiology, Chinese Academy of Sciences)
EPI_ISL_198898	A/environment/Yunnan/03.15 DQWGH014/2015(H5N6)	A / H5N6	Asia / China	Yuhai Bi (Institute of Microbiology, Chinese Academy of Sciences)
EPI_ISL_198899	A/environment/Yunnan/03.17 DQJT039/2015(H5N6)	A / H5N6	Asia / China	Yuhai Bi (Institute of Microbiology, Chinese Academy of Sciences)
EPI_ISL_198900	A/environment/Yunnan/03.15 DQWGH009/2015(H5N6)	A / H5N6	Asia / China	Yuhai Bi (Institute of Microbiology, Chinese Academy of Sciences)
EPI_ISL_198901	A/environment/Yunnan/03.15 DQWGH020/2015(H5N6)	A / H5N6	Asia / China	Yuhai Bi (Institute of Microbiology, Chinese Academy of Sciences)
EPI_ISL_198902	A/environment/Yunnan/03.15 DQWGH017/2015(H5N6)	A / H5N6	Asia / China	Yuhai Bi (Institute of Microbiology, Chinese Academy of Sciences)
EPI_ISL_198903	A/environment/Yunnan/03.15 DQWGH004/2015(H5N6)	A / H5N6	Asia / China	Yuhai Bi (Institute of Microbiology, Chinese Academy of Sciences)
EPI_ISL_198904	A/environment/Yunnan/03.15 DQWGH008-1/2015(H5N6)	A / H5N6	Asia / China	Yuhai Bi (Institute of Microbiology, Chinese Academy of Sciences)
EPI_ISL_198906	A/duck/Hunan/12.17 YYFQH0018-Z-P/2014(H5N6)	A / H5N6	Asia / China	Yuhai Bi (Institute of Microbiology, Chinese Academy of Sciences)
EPI_ISL_198908	A/duck/Hunan/12.17 YYGK0063-O/2014(H5N6)	A / H5N6	Asia / China	Yuhai Bi (Institute of Microbiology, Chinese Academy of Sciences)
EPI_ISL_198909	A/duck/Hunan/12.17 YYGK0056/2014(H5N6)	A / H5N6	Asia / China	Yuhai Bi (Institute of Microbiology, Chinese Academy of Sciences)
EPI_ISL_198911	A/duck/Hunan/12.17 YYGK0066-P/2014(H5N6)	A / H5N6	Asia / China	Yuhai Bi (Institute of Microbiology, Chinese Academy of Sciences)
EPI_ISL_198916	A/chicken/Jiangxi/05.06 NCDZT1174-O/2015(H5N6)	A / H5N6	Asia / China	Yuhai Bi (Institute of Microbiology, Chinese Academy of Sciences)
EPI_ISL_198917	A/chicken/Jiangxi/04.01 NCJD0119-O/2015(H5N6)	A / H5N6	Asia / China	Yuhai Bi (Institute of Microbiology, Chinese Academy of Sciences)
EPI_ISL_198919	A/chicken/Jiangxi/04.01 NCJD0010-O/2015(H5N6)	A / H5N6	Asia / China	Yuhai Bi (Institute of Microbiology, Chinese Academy of Sciences)
EPI_ISL_198920	A/chicken/Jiangxi/04.01 NCJD0137-O/2015(H5N6)	A / H5N6	Asia / China	Yuhai Bi (Institute of Microbiology, Chinese Academy of Sciences)
EPI_ISL_198921	A/chicken/Jiangxi/04.01 NCJD0146-O/2015(H5N6)	A / H5N6	Asia / China	Yuhai Bi (Institute of Microbiology, Chinese Academy of Sciences)
EPI_ISL_198923	A/chicken/Jiangxi/04.01 NCDZT0173-P/2015(H5N6)	A / H5N6	Asia / China	Yuhai Bi (Institute of Microbiology, Chinese Academy of Sciences)
EPI_ISL_198924	A/chicken/Jiangxi/04.01 NCDZT0175-P/2015(H5N6)	A / H5N6	Asia / China	Yuhai Bi (Institute of Microbiology, Chinese Academy of Sciences)
EPI_ISL_198926	A/chicken/Jiangxi/04.01 NCDZT0192-P/2015(H5N6)	A / H5N6	Asia / China	Yuhai Bi (Institute of Microbiology, Chinese Academy of Sciences)
EPI_ISL_198927	A/chicken/Jiangxi/04.01 NCDZT0203-P/2015(H5N6)	A / H5N6	Asia / China	Yuhai Bi (Institute of Microbiology, Chinese Academy of Sciences)
EPI_ISL_198929	A/chicken/Jiangxi/04.01 NCDZT0206-P/2015(H5N6)	A / H5N6	Asia / China	Yuhai Bi (Institute of Microbiology, Chinese Academy of Sciences)
EPI_ISL_198930	A/chicken/Jiangxi/04.01 NCDZT0238-P/2015(H5N6)	A / H5N6	Asia / China	Yuhai Bi (Institute of Microbiology, Chinese Academy of Sciences)
EPI_ISL_198932	A/chicken/Jiangxi/04.01 NCDZT0279-P/2015(H5N6)	A / H5N6	Asia / China	Yuhai Bi (Institute of Microbiology, Chinese Academy of Sciences)
EPI_ISL_198933	A/chicken/Jiangxi/04.01 NCDZT0285-P/2015(H5N6)	A / H5N6	Asia / China	Yuhai Bi (Institute of Microbiology, Chinese Academy of Sciences)
EPI_ISL_198934	A/chicken/Jiangxi/04.01 NCDZT0291-P/2015(H5N6)	A / H5N6	Asia / China	Yuhai Bi (Institute of Microbiology, Chinese Academy of Sciences)
EPI_ISL_198935	A/chicken/Jiangxi/04.01 NCDZT0292-P/2015(H5N6)	A / H5N6	Asia / China	Yuhai Bi (Institute of Microbiology, Chinese Academy of Sciences)
EPI_ISL_198937	A/duck/Jiangxi/04.01 NCDZT0312-O/2015(H5N6)	A / H5N6	Asia / China	Yuhai Bi (Institute of Microbiology, Chinese Academy of Sciences)
EPI_ISL_198939	A/duck/Jiangxi/04.01 NCDZT0334-P/2015(H5N6)	A / H5N6	Asia / China	Yuhai Bi (Institute of Microbiology, Chinese Academy of Sciences)
EPI_ISL_198949	A/environment/Yunnan/03.15 DQWGH001/2015(H5N6)	A / H5N6	Asia / China	Yuhai Bi (Institute of Microbiology, Chinese Academy of Sciences)
EPI_ISL_198952	A/environment/Yunnan/03.17 DQJT040/2015(H5N6)	A / H5N6	Asia / China	Yuhai Bi (Institute of Microbiology, Chinese Academy of Sciences)
EPI_ISL_198953	A/environment/Jiangxi/05.07 NCJD0010D/2015(H5N6)	A / H5N6	Asia / China	Yuhai Bi (Institute of Microbiology, Chinese Academy of Sciences)
EPI_ISL_198954	A/environment/Jiangxi/05.07 NCJD0012D/2015(H5N6)	A / H5N6	Asia / China	Yuhai Bi (Institute of Microbiology, Chinese Academy of Sciences)

EPI_ISL_198956	A/environment/Jiangxi/05.07 NCJD0017D/2015(H5N6)	A / H5N6	Asia / China	Yuhai Bi (Institute of Microbiology, Chinese Academy of Sciences)
EPI_ISL_198958	A/environment/Jiangxi/05.07 NCJD0022D/2015(H5N6)	A / H5N6	Asia / China	Yuhai Bi (Institute of Microbiology, Chinese Academy of Sciences)
EPI_ISL_198959	A/environment/Jiangxi/05.07 NCJD0023D/2015(H5N6)	A / H5N6	Asia / China	Yuhai Bi (Institute of Microbiology, Chinese Academy of Sciences)
EPI_ISL_198960	A/environment/Jiangxi/05.07 NCJD0027D/2015(H5N6)	A / H5N6	Asia / China	Yuhai Bi (Institute of Microbiology, Chinese Academy of Sciences)
EPI_ISL_198962	A/Pigeon/Jiangxi/04.01 NCDZT0347-O/2015(H5N6)	A / H5N6	Asia / China	Yuhai Bi (Institute of Microbiology, Chinese Academy of Sciences)
EPI_ISL_198964	A/Pigeon/Jiangxi/04.01 NCDZT0348-O/2015(H5N6)	A / H5N6	Asia / China	Yuhai Bi (Institute of Microbiology, Chinese Academy of Sciences)
EPI_ISL_198967	A/Pigeon/Jiangxi/04.01 NCDZT0352-O/2015(H5N6)	A / H5N6	Asia / China	Yuhai Bi (Institute of Microbiology, Chinese Academy of Sciences)
EPI_ISL_198968	A/Pigeon/Jiangxi/04.01 NCDZT0353-O/2015(H5N6)	A / H5N6	Asia / China	Yuhai Bi (Institute of Microbiology, Chinese Academy of Sciences)
EPI_ISL_198971	A/chicken/Jiangxi/05.06 NCDZT0039B-P/2015(H5N6)	A / H5N6	Asia / China	Yuhai Bi (Institute of Microbiology, Chinese Academy of Sciences)
EPI_ISL_198972	A/chicken/Jiangxi/05.06 NCDZT0003C-P/2015(H5N6)	A / H5N6	Asia / China	Yuhai Bi (Institute of Microbiology, Chinese Academy of Sciences)
EPI_ISL_198976	A/chicken/Jiangxi/04.01 NCDZT0042-O/2015(H5N6)	A / H5N6	Asia / China	Yuhai Bi (Institute of Microbiology, Chinese Academy of Sciences)
EPI_ISL_198977	A/chicken/Jiangxi/05.06 NCDZT0043B-P/2015(H5N6)	A / H5N6	Asia / China	Yuhai Bi (Institute of Microbiology, Chinese Academy of Sciences)
EPI_ISL_198981	A/environment/Jiangxi/05.07 NCJD0052C/2015(H5N6)	A / H5N6	Asia / China	Yuhai Bi (Institute of Microbiology, Chinese Academy of Sciences)
EPI_ISL_198984	A/duck/Jiangxi/05.07 NCJD0056A-P/2015(H5N6)	A / H5N6	Asia / China	Yuhai Bi (Institute of Microbiology, Chinese Academy of Sciences)
EPI_ISL_198985	A/chicken/Jiangxi/05.06 NCDZT0058B-P/2015(H5N6)	A / H5N6	Asia / China	Yuhai Bi (Institute of Microbiology, Chinese Academy of Sciences)
EPI_ISL_198987	A/duck/Jiangxi/05.07 NCJD0061A-P/2015(H5N6)	A / H5N6	Asia / China	Yuhai Bi (Institute of Microbiology, Chinese Academy of Sciences)
EPI_ISL_198990	A/chicken/Jiangxi/04.01 NCNP0065-Z-O/2015(H5N6)	A / H5N6	Asia / China	Yuhai Bi (Institute of Microbiology, Chinese Academy of Sciences)
EPI_ISL_198991	A/duck/Jiangxi/05.07 NCJD0067A-P/2015(H5N6)	A / H5N6	Asia / China	Yuhai Bi (Institute of Microbiology, Chinese Academy of Sciences)
EPI_ISL_198992	A/environment/Jiangxi/05.07 NCJD0070C/2015(H5N6)	A / H5N6	Asia / China	Yuhai Bi (Institute of Microbiology, Chinese Academy of Sciences)
EPI_ISL_198996	A/environment/Jiangxi/05.07 NCJD0078C/2015(H5N6)	A / H5N6	Asia / China	Yuhai Bi (Institute of Microbiology, Chinese Academy of Sciences)
EPI_ISL_199006	A/environment/Jiangxi/05.07 NCJD0095C/2015(H5N6)	A / H5N6	Asia / China	Yuhai Bi (Institute of Microbiology, Chinese Academy of Sciences)
EPI_ISL_199007	A/environment/Jiangxi/05.07 NCJD0098C/2015(H5N6)	A / H5N6	Asia / China	Yuhai Bi (Institute of Microbiology, Chinese Academy of Sciences)
EPI_ISL_199009	A/environment/Jiangxi/05.07 NCJD0093C/2015(H5N6)	A / H5N6	Asia / China	Yuhai Bi (Institute of Microbiology, Chinese Academy of Sciences)
EPI_ISL_199010	A/chicken/Jiangxi/12.05 NCNP006/2014(H5N6)	A / H5N6	Asia / China	Yuhai Bi (Institute of Microbiology, Chinese Academy of Sciences)
EPI_ISL_199011	A/chicken/Hubei/03.06 WHWTZ0115-O/2015(H5N6)	A / H5N6	Asia / China	Yuhai Bi (Institute of Microbiology, Chinese Academy of Sciences)
EPI_ISL_199015	A/duck/Hubei/03.06 WHWTZ0129-P/2015(H5N6)	A / H5N6	Asia / China	Yuhai Bi (Institute of Microbiology, Chinese Academy of Sciences)
EPI_ISL_199017	A/chicken/Hubei/03.06 WHWTZ0148-O/2015(H5N6)	A / H5N6	Asia / China	Yuhai Bi (Institute of Microbiology, Chinese Academy of Sciences)
EPI_ISL_199026	A/duck/Hubei/03.06 WHWTZ0123-O/2015(H5N6)	A / H5N6	Asia / China	Yuhai Bi (Institute of Microbiology, Chinese Academy of Sciences)
EPI_ISL_199028	A/duck/Hubei/03.06 WHWTZ0124-O/2015(H5N6)	A / H5N6	Asia / China	Yuhai Bi (Institute of Microbiology, Chinese Academy of Sciences)
EPI_ISL_199029	A/duck/HuBei/03.06 WHWTZ0158-P/2015(H5N6)	A / H5N6	Asia / China	Yuhai Bi (Institute of Microbiology, Chinese Academy of Sciences)
EPI_ISL_199030	A/duck/Hubei/03.06 WHWTZ0125-O/2015(H5N6)	A / H5N6	Asia / China	Yuhai Bi (Institute of Microbiology, Chinese Academy of Sciences)
EPI_ISL_199031	A/duck/Hubei/03.06 WHWTZ0130-O/2015(H5N6)	A / H5N6	Asia / China	Yuhai Bi (Institute of Microbiology, Chinese Academy of Sciences)
EPI_ISL_199032	A/duck/Hubei/03.06 WHWTZ0148-O/2015(H5N6)	A / H5N6	Asia / China	Yuhai Bi (Institute of Microbiology, Chinese Academy of Sciences)
EPI_ISL_199034	A/chicken/Yunnan/03.16 DQJT0068-O/2015(H5N6)	A / H5N6	Asia / China	Yuhai Bi (Institute of Microbiology, Chinese Academy of Sciences)
EPI_ISL_199035	A/chicken/Yunnan/03.15 DQXYL0073-O/2015(H5N6)	A / H5N6	Asia / China	Yuhai Bi (Institute of Microbiology, Chinese Academy of Sciences)
EPI_ISL_199036	A/chicken/Yunnan/03.16 DQJT0066-O/2015(H5N6)	A / H5N6	Asia / China	Yuhai Bi (Institute of Microbiology, Chinese Academy of Sciences)
EPI_ISL_199037	A/duck/Yunnan/03.16 DQXYL0013-O/2015	A / H5N6	Asia / China	Yuhai Bi (Institute of Microbiology, Chinese Academy of Sciences)
EPI_ISL_199038	A/duck/Hunan/04.22 LDDX037-P/2015(H5N6)	A / H5N6	Asia / China	Yuhai Bi (Institute of Microbiology, Chinese Academy of Sciences)
EPI_ISL_199041	A/chicken/Yunnan/03.16 DQXYL023-O/2015(H5N6)	A / H5N6	Asia / China	Yuhai Bi (Institute of Microbiology, Chinese Academy of Sciences)
EPI_ISL_199042	A/duck/Yunnan/03.16 DQXYL003-O/2015(H5N6)	A / H5N6	Asia / China	Yuhai Bi (Institute of Microbiology, Chinese Academy of Sciences)
EPI_ISL_199043	A/duck/Yunnan/03.16 DQXYL017-O/2015(H5N6)	A / H5N6	Asia / China	Yuhai Bi (Institute of Microbiology, Chinese Academy of Sciences)
EPI_ISL_199044	A/duck/Yunnan/03.16 DQXYL022-O/2015(H5N6)	A / H5N6	Asia / China	Yuhai Bi (Institute of Microbiology, Chinese Academy of Sciences)
EPI_ISL_199045	A/duck/Hunan/02.07 YYFQH010-P/2015(H5N6)	A / H5N6	Asia / China	Yuhai Bi (Institute of Microbiology, Chinese Academy of Sciences)
EPI_ISL_199046	A/duck/Hunan/01.21 YYFQH010-O/2015(H5N6)	A / H5N6	Asia / China	Yuhai Bi (Institute of Microbiology, Chinese Academy of Sciences)
EPI_ISL_199047	A/duck/Hunan/01.21 YYFQH011-O/2015(H5N6)	A / H5N6	Asia / China	Yuhai Bi (Institute of Microbiology, Chinese Academy of Sciences)
EPI_ISL_199049	A/duck/Hunan/02.07 YYFQHY12Y-O/2015(H5N6)	A / H5N6	Asia / China	Yuhai Bi (Institute of Microbiology, Chinese Academy of Sciences)
EPI_ISL_199050	A/duck/Hunan/01.21 YYFQH012-O/2015(H5N6)	A / H5N6	Asia / China	Yuhai Bi (Institute of Microbiology, Chinese Academy of Sciences)
EPI_ISL_199051	A/duck/Hunan/03.09 YYFQH0047-O/2015(H5N6)	A / H5N6	Asia / China	Yuhai Bi (Institute of Microbiology, Chinese Academy of Sciences)
EPI_ISL_199052	A/duck/Hunan/02.07 YYFQH015-P/2015(H5N6)	A / H5N6	Asia / China	Yuhai Bi (Institute of Microbiology, Chinese Academy of Sciences)
EPI_ISL_199053	A/duck/Hunan/01.21 YYFQH015-O/2015(H5N6)	A / H5N6	Asia / China	Yuhai Bi (Institute of Microbiology, Chinese Academy of Sciences)
EPI_ISL_199054	A/duck/Hunan/01.21 YYFQH016-O/2015(H5N6)	A / H5N6	Asia / China	Yuhai Bi (Institute of Microbiology, Chinese Academy of Sciences)
EPI_ISL_199055	A/duck/Hunan/01.21 YYFQH018-O/2015(H5N6)	A / H5N6	Asia / China	Yuhai Bi (Institute of Microbiology, Chinese Academy of Sciences)
EPI_ISL_199056	A/duck/Hunan/01.21 YYFQH019-P/2015(H5N6)	A / H5N6	Asia / China	Yuhai Bi (Institute of Microbiology, Chinese Academy of Sciences)
EPI_ISL_199057	A/duck/Hunan/02.07 YYFQHY19Y-O/2015(H5N6)	A / H5N6	Asia / China	Yuhai Bi (Institute of Microbiology, Chinese Academy of Sciences)
EPI_ISL_199058	A/duck/Hunan/02.07 YYFQHY20Y-O/2015(H5N6)	A / H5N6	Asia / China	Yuhai Bi (Institute of Microbiology, Chinese Academy of Sciences)
EPI_ISL_199059	A/duck/Hunan/01.21 YYFQH022-O/2015(H5N6)	A / H5N6	Asia / China	Yuhai Bi (Institute of Microbiology, Chinese Academy of Sciences)
EPI_ISL_199062	A/Phalacrocorax/Hubei/01.09 V5/2015(H5N6)	A / H5N6	Asia / China	Yuhai Bi (Institute of Microbiology, Chinese Academy of Sciences)
EPI_ISL_199063	A/Phalacrocorax/Hubei/01.09 V7/2015(H5N6)	A / H5N6	Asia / China	Yuhai Bi (Institute of Microbiology, Chinese Academy of Sciences)
EPI_ISL_199064	A/duck/Hunan/01.21 YYFQH023-O/2015(H5N6)	A / H5N6	Asia / China	Yuhai Bi (Institute of Microbiology, Chinese Academy of Sciences)
EPI_ISL_199065	A/duck/Hunan/01.21 YYFQH024-P/2015(H5N6)	A / H5N6	Asia / China	Yuhai Bi (Institute of Microbiology, Chinese Academy of Sciences)
EPI_ISL_199066	A/duck/Hunan/01.21 YYFQH025-P/2015(H5N6)	A / H5N6	Asia / China	Yuhai Bi (Institute of Microbiology, Chinese Academy of Sciences)
EPI_ISL_199067	A/duck/Hunan/02.07 YYFQH026-O/2015(H5N6)	A / H5N6	Asia / China	Yuhai Bi (Institute of Microbiology, Chinese Academy of Sciences)
EPI_ISL_199068	A/duck/Hunan/01.21 YYFQH027-O/2015(H5N6)	A / H5N6	Asia / China	Yuhai Bi (Institute of Microbiology, Chinese Academy of Sciences)
EPI_ISL_199069	A/duck/Hunan/02.07 YYFQH029-O/2015(H5N6)	A / H5N6	Asia / China	Yuhai Bi (Institute of Microbiology, Chinese Academy of Sciences)
EPI_ISL_199070	A/duck/Hunan/01.21 YYFQH030-O/2015(H5N6)	A / H5N6	Asia / China	Yuhai Bi (Institute of Microbiology, Chinese Academy of Sciences)
EPI_ISL_199071	A/duck/Hunan/01.21 YYFQH032-P/2015(H5N6)	A / H5N6	Asia / China	Yuhai Bi (Institute of Microbiology, Chinese Academy of Sciences)
EPI_ISL_199072	A/duck/Hunan/02.07 YYFQH032-O/2015(H5N6)	A / H5N6	Asia / China	Yuhai Bi (Institute of Microbiology, Chinese Academy of Sciences)
EPI_ISL_199073	A/duck/Hunan/02.07 YYFQH003-P/2015(H5N6)	A / H5N6	Asia / China	Yuhai Bi (Institute of Microbiology, Chinese Academy of Sciences)
EPI_ISL_199074	A/duck/Hunan/01.21 YYFQH003-O/2015(H5N6)	A / H5N6	Asia / China	Yuhai Bi (Institute of Microbiology, Chinese Academy of Sciences)
EPI_ISL_199075	A/duck/Hunan/01.21 YYFQH005-P/2015(H5N6)	A / H5N6	Asia / China	Yuhai Bi (Institute of Microbiology, Chinese Academy of Sciences)
EPI_ISL_199076	A/duck/Hunan/01.21 YYFQH005-O/2015(H5N6)	A / H5N6	Asia / China	Yuhai Bi (Institute of Microbiology, Chinese Academy of Sciences)
EPI_ISL_199077	A/duck/Hunan/02.07 YYFQH006-P/2015(H5N6)	A / H5N6	Asia / China	Yuhai Bi (Institute of Microbiology, Chinese Academy of Sciences)

EPI_ISL_199079	A/duck/Hunan/01.21 YYFQH006-O/2015(H5N6)	A / H5N6	Asia / China	Yuhai Bi (Institute of Microbiology, Chinese Academy of Sciences)
EPI_ISL_199080	A/duck/Hunan/01.21 YYFQH007-O/2015(H5N6)	A / H5N6	Asia / China	Yuhai Bi (Institute of Microbiology, Chinese Academy of Sciences)
EPI_ISL_199081	A/chicken/Hunan/04.14 YYXS0835-O/2015(H5N6)	A / H5N6	Asia / China	Yuhai Bi (Institute of Microbiology, Chinese Academy of Sciences)
EPI_ISL_199083	A/duck/Hunan/01.21 YYFQH009-O/2015(H5N6)	A / H5N6	Asia / China	Yuhai Bi (Institute of Microbiology, Chinese Academy of Sciences)
EPI_ISL_199084	A/environment/Yunnan/03.16 DQXYL0053-Z-O/2015(H5N6)	A / H5N6	Asia / China	Yuhai Bi (Institute of Microbiology, Chinese Academy of Sciences)
EPI_ISL_199085	A/chicken/Yunnan/03.15 DQJT0054-Z-P/2015(H5N6)	A / H5N6	Asia / China	Yuhai Bi (Institute of Microbiology, Chinese Academy of Sciences)
EPI_ISL_199086	A/chicken/Yunnan/03.16 DQXYL0063-Z-O/2015(H5N6)	A / H5N6	Asia / China	Yuhai Bi (Institute of Microbiology, Chinese Academy of Sciences)
EPI_ISL_199087	A/environment/Yunnan/03.17 DQJT0015/2015(H5N6)	A / H5N6	Asia / China	Yuhai Bi (Institute of Microbiology, Chinese Academy of Sciences)
EPI_ISL_199093	A/duck/Yunnan/03.15 DQXYL007-Z-O/2015(H5N6)	A / H5N6	Asia / China	Yuhai Bi (Institute of Microbiology, Chinese Academy of Sciences)
EPI_ISL_199094	A/environment/Yunnan/03.15 DQWGH008-Z/2015(H5N6)	A / H5N6	Asia / China	Yuhai Bi (Institute of Microbiology, Chinese Academy of Sciences)
EPI_ISL_199095	A/environment/Yunnan/03.16 DQXYL0027/2015(H5N6)	A / H5N6	Asia / China	Yuhai Bi (Institute of Microbiology, Chinese Academy of Sciences)
EPI_ISL_199099	A/environment/Yunnan/03.16 DQXYL0032/2015(H5N6)	A / H5N6	Asia / China	Yuhai Bi (Institute of Microbiology, Chinese Academy of Sciences)
EPI_ISL_199100	A/chicken/Yunnan/03.17 DQJT0041-O/2015(H5N6)	A / H5N6	Asia / China	Yuhai Bi (Institute of Microbiology, Chinese Academy of Sciences)
EPI_ISL_199101	A/chicken/Yunnan/03.16 DQXYL0044-O/2015(N6)	A / H5N6	Asia / China	Yuhai Bi (Institute of Microbiology, Chinese Academy of Sciences)
EPI_ISL_199102	A/chicken/Yunnan/03.16 DQXYL0050-O/2015(H5N6)	A / H5N6	Asia / China	Yuhai Bi (Institute of Microbiology, Chinese Academy of Sciences)
EPI_ISL_199103	A/environment/Yunnan/03.17 DQJT0051-P/2015(H5N6)	A / H5N6	Asia / China	Yuhai Bi (Institute of Microbiology, Chinese Academy of Sciences)
EPI_ISL_199104	A/chicken/Yunnan/03.16 DQXYL0052-O/2015(H5N6)	A / H5N6	Asia / China	Yuhai Bi (Institute of Microbiology, Chinese Academy of Sciences)
EPI_ISL_199106	A/chicken/Yunnan/03.16 DQXYL057-O/2015(H5N6)	A / H5N6	Asia / China	Yuhai Bi (Institute of Microbiology, Chinese Academy of Sciences)
EPI_ISL_199109	A/chicken/Yunnan/03.16 DQJT069-O/2015(H5N6)	A / H5N6	Asia / China	Yuhai Bi (Institute of Microbiology, Chinese Academy of Sciences)
EPI_ISL_199110	A/Anser fabalis/Hunan/03.09 YYDTH0007/2015(H5N6)	A / H5N6	Asia / China	Yuhai Bi (Institute of Microbiology, Chinese Academy of Sciences)
EPI_ISL_199112	A/environment/Hunan/04.14 YYGK391/2015(H5N6)	A / H5N6	Asia / China	Yuhai Bi (Institute of Microbiology, Chinese Academy of Sciences)
EPI_ISL_199113	A/environment/Hunan/04.14 YYGK0393-1/2015(H5N6)	A / H5N6	Asia / China	Yuhai Bi (Institute of Microbiology, Chinese Academy of Sciences)
EPI_ISL_199119	A/duck/Hunan/04.14 YYGK460-O/2015(H5N6)	A / H5N6	Asia / China	Yuhai Bi (Institute of Microbiology, Chinese Academy of Sciences)
EPI_ISL_199120	A/duck/Hunan/04.14 YYGK464-O/2015(H5N6)	A / H5N6	Asia / China	Yuhai Bi (Institute of Microbiology, Chinese Academy of Sciences)
EPI_ISL_199122	A/duck/Hunan/04.14 YYGK468-O/2015(H5N6)	A / H5N6	Asia / China	Yuhai Bi (Institute of Microbiology, Chinese Academy of Sciences)
EPI_ISL_199130	A/chicken/Hunan/04.14 YYGK607-P/2015(H5N6)	A / H5N6	Asia / China	Yuhai Bi (Institute of Microbiology, Chinese Academy of Sciences)
EPI_ISL_199137	A/duck/Hunan/04.14 YYXS877-P/2015(H5N6)	A / H5N6	Asia / China	Yuhai Bi (Institute of Microbiology, Chinese Academy of Sciences)
EPI_ISL_199138	A/duck/Hunan/04.14 YYGK0878-P/2015(H5N6)	A / H5N6	Asia / China	Yuhai Bi (Institute of Microbiology, Chinese Academy of Sciences)
EPI_ISL_199146	A/chicken/Miyazaki/2-4/2014	A / H5N8	Asia / Japan / Miyazaki / Miyazaki city	Takehiko Saito (National Institute of Animal Health)
EPI_ISL_199147	A/chicken/Yamaguchi/6/2014	A / H5N8	Asia / Japan / Yamaguchi	Takehiko Saito (National Institute of Animal Health)
EPI_ISL_199148	A/chicken/Okayama/1-2/2015	A / H5N8	Asia / Japan / Okayama	Takehiko Saito (National Institute of Animal Health)
EPI_ISL_199149	A/chicken/Saga/1-1/2015	A / H5N8	Asia / Japan / Saga	Takehiko Saito (National Institute of Animal Health)
EPI_ISL_199151	A/duck/Shandong/11.14 DL-DKd11/2013(H5N8)	A / H5N8	Asia / China	Yuhai Bi (Institute of Microbiology, Chinese Academy of Sciences)
EPI_ISL_199153	A/goose/Shandong/11.02 CQ-GS/2014(H5N6)	A / H5N6	Asia / China	Yuhai Bi (Institute of Microbiology, Chinese Academy of Sciences)
EPI_ISL_199155	A/goose/Shandong/12.05 YG-GS/2014(H5N6)	A / H5N6	Asia / China	Yuhai Bi (Institute of Microbiology, Chinese Academy of Sciences)
EPI_ISL_199156	A/goose/Shandong/12.08 YG-GS/2014(H5N8)	A / H5N8	Asia / China	Yuhai Bi (Institute of Microbiology, Chinese Academy of Sciences)
EPI_ISL_199158	A/goose/Shandong/05.23 DT-GS/2014(H5N8)	A / H5N8	Asia / China	Yuhai Bi (Institute of Microbiology, Chinese Academy of Sciences)
EPI_ISL_199159	A/goose/Shandong/GD-GS/2014(H5N8)	A / H5N8	Asia / China	Yuhai Bi (Institute of Microbiology, Chinese Academy of Sciences)
EPI_ISL_199160	A/goose/Shandong/01.01 JY-GS/2015(H5N2)	A / H5N2	Asia / China	Yuhai Bi (Institute of Microbiology, Chinese Academy of Sciences)
EPI_ISL_199161	A/goose/Shandong/01.07 JQ-GS/2015(H5N2)	A / H5N2	Asia / China	Yuhai Bi (Institute of Microbiology, Chinese Academy of Sciences)
EPI_ISL_199163	A/goose/Shandong/02.16 YG-GS/2015(H5N6)	A / H5N6	Asia / China	Yuhai Bi (Institute of Microbiology, Chinese Academy of Sciences)
EPI_ISL_199174	A/duck/Guangdong/03.26 DGCPLB021-O/2015(H5N6)	A / H5N6	Asia / China	Yuhai Bi (Institute of Microbiology, Chinese Academy of Sciences)
EPI_ISL_199175	A/duck/Guangdong/03.26 DGCP066-O/2015(H5N6)	A / H5N6	Asia / China	Yuhai Bi (Institute of Microbiology, Chinese Academy of Sciences)
EPI_ISL_199178	A/duck/Guangdong/03.26 DGCP074-O/2015(H5N6)	A / H5N6	Asia / China	Yuhai Bi (Institute of Microbiology, Chinese Academy of Sciences)
EPI_ISL_199179	A/duck/Guangdong/03.26 DGCP079-O/2015(H5N6)	A / H5N6	Asia / China	Yuhai Bi (Institute of Microbiology, Chinese Academy of Sciences)
EPI_ISL_199180	A/duck/Guangdong/03.26 DGCP080-O/2015(H5N6)	A / H5N6	Asia / China	Yuhai Bi (Institute of Microbiology, Chinese Academy of Sciences)
EPI_ISL_199181	A/duck/Guangdong/03.26 DGCP076-O/2015(H5N6)	A / H5N6	Asia / China	Yuhai Bi (Institute of Microbiology, Chinese Academy of Sciences)
EPI_ISL_199183	A/duck/Guangdong/03.26 DGCPLB020-P/2015(H5N6)	A / H5N6	Asia / China	Yuhai Bi (Institute of Microbiology, Chinese Academy of Sciences)
EPI_ISL_199185	A/duck/Guangdong/03.26 DGCP709-P/2015(H5N6)	A / H5N6	Asia / China	Yuhai Bi (Institute of Microbiology, Chinese Academy of Sciences)
EPI_ISL_199186	A/duck/Guangdong/03.26 DGCPLB022-P/2015(H5N6)	A / H5N6	Asia / China	Yuhai Bi (Institute of Microbiology, Chinese Academy of Sciences)
EPI_ISL_199187	A/duck/Guangdong/03.26 DGCPLB023-P/2015(H5N6)	A / H5N6	Asia / China	Yuhai Bi (Institute of Microbiology, Chinese Academy of Sciences)
EPI_ISL_199188	A/duck/Guangdong/03.26 DGCPLB019-P/2015(H5N6)	A / H5N6	Asia / China	Yuhai Bi (Institute of Microbiology, Chinese Academy of Sciences)
EPI_ISL_199189	A/duck/Guangdong/03.26 DGCP078-P/2015(H5N6)	A / H5N6	Asia / China	Yuhai Bi (Institute of Microbiology, Chinese Academy of Sciences)
EPI_ISL_199190	A/duck/Guangdong/03.26 DGCPLB009-O/2015(H5N6)	A / H5N6	Asia / China	Yuhai Bi (Institute of Microbiology, Chinese Academy of Sciences)
EPI_ISL_199192	A/duck/Guangdong/03.26 DGCPLB007-P/2015(H5N6)	A / H5N6	Asia / China	Yuhai Bi (Institute of Microbiology, Chinese Academy of Sciences)
EPI_ISL_199195	A/duck/Guangdong/03.26 DGCP080-P/2015(H5N6)	A / H5N6	Asia / China	Yuhai Bi (Institute of Microbiology, Chinese Academy of Sciences)
EPI_ISL_199196	A/goose/Shandong/04.22 GT/2015(H5N2)	A / H5N2	Asia / China	Yuhai Bi (Institute of Microbiology, Chinese Academy of Sciences)
EPI_ISL_199198	A/duck/Guangdong/03.26 DGCP081-P/2015(H5N6)	A / H5N6	Asia / China	Yuhai Bi (Institute of Microbiology, Chinese Academy of Sciences)
EPI_ISL_199199	A/duck/Guangdong/03.26 DGCP078-O/2015(H5N6)	A / H5N6	Asia / China	Yuhai Bi (Institute of Microbiology, Chinese Academy of Sciences)
EPI_ISL_199200	A/duck/Guangdong/03.26 DGCP084-P/2015(H5N6)	A / H5N6	Asia / China	Yuhai Bi (Institute of Microbiology, Chinese Academy of Sciences)
EPI_ISL_199201	A/duck/Guangdong/03.26 DGCP061-P/2015(H5N6)	A / H5N6	Asia / China	Yuhai Bi (Institute of Microbiology, Chinese Academy of Sciences)
EPI_ISL_199207	A/goose/Guangdong/04.22 DGCP117-O/2015(H5N6)	A / H5N6	Asia / China	Yuhai Bi (Institute of Microbiology, Chinese Academy of Sciences)
EPI_ISL_199208	A/duck/Guangdong/04.23 DGQTXC229-O/2015(H5N6)	A / H5N6	Asia / China	Yuhai Bi (Institute of Microbiology, Chinese Academy of Sciences)
EPI_ISL_199209	A/duck/Guangdong/04.22 DGCP068-O/2015(H5N6)	A / H5N6	Asia / China	Yuhai Bi (Institute of Microbiology, Chinese Academy of Sciences)
EPI_ISL_199211	A/duck/Guangdong/04.23 DGQTSJ150-O/2015(H5N6)	A / H5N6	Asia / China	Yuhai Bi (Institute of Microbiology, Chinese Academy of Sciences)
EPI_ISL_199214	A/duck/Guangdong/04.23 DGQTXC202-O/2015(H5N6)	A / H5N6	Asia / China	Yuhai Bi (Institute of Microbiology, Chinese Academy of Sciences)
EPI_ISL_199215	A/duck/Guangdong/04.22 DGCP069-O/2015(H5N6)	A / H5N6	Asia / China	Yuhai Bi (Institute of Microbiology, Chinese Academy of Sciences)
EPI_ISL_199222	A/duck/Guangdong/04.23 DGQTXC203-O/2015(H5N6)	A / H5N6	Asia / China	Yuhai Bi (Institute of Microbiology, Chinese Academy of Sciences)
EPI_ISL_199230	A/duck/Guangdong/04.22 DGCPLB018-O/2015(H5N6)	A / H5N6	Asia / China	Yuhai Bi (Institute of Microbiology, Chinese Academy of Sciences)
EPI_ISL_199234	A/duck/Guangdong/04.23 DGQTXC229-P/2015(H5N6)	A / H5N6	Asia / China	Yuhai Bi (Institute of Microbiology, Chinese Academy of Sciences)
EPI_ISL_199235	A/duck/Guangdong/04.23 DGQTSJ145-O/2015(H5N6)	A / H5N6	Asia / China	Yuhai Bi (Institute of Microbiology, Chinese Academy of Sciences)
EPI_ISL_199236	A/duck/Guangdong/04.23 DGQTXC231-O/2015(H5N6)	A / H5N6	Asia / China	Yuhai Bi (Institute of Microbiology, Chinese Academy of Sciences)

EPI_ISL_199241	A/duck/Guangdong/04.23 DGQTXC226-O/2015(H5N6)	A / H5N6	Asia / China	Yuhai Bi (Institute of Microbiology, Chinese Academy of Sciences)
EPI_ISL_199242	A/duck/Guangdong/04.22 DGCP061-O/2015(H5N6)	A / H5N6	Asia / China	Yuhai Bi (Institute of Microbiology, Chinese Academy of Sciences)
EPI_ISL_199243	A/environment/Guangdong/04.22 DGCP027/2015(H5N6)	A / H5N6	Asia / China	Yuhai Bi (Institute of Microbiology, Chinese Academy of Sciences)
EPI_ISL_199244	A/goose/Guangdong/04.22 DGCP120-P/2015(H5N6)	A / H5N6	Asia / China	Yuhai Bi (Institute of Microbiology, Chinese Academy of Sciences)
EPI_ISL_199250	A/duck/Guangdong/04.22 DGCP079-O/2015(H5N6)	A / H5N6	Asia / China	Yuhai Bi (Institute of Microbiology, Chinese Academy of Sciences)
EPI_ISL_199252	A/duck/Guangdong/04.22 DGCPLB020-O/2015(H5N6)	A / H5N6	Asia / China	Yuhai Bi (Institute of Microbiology, Chinese Academy of Sciences)
EPI_ISL_199255	A/duck/Guangdong/04.22 DGCP071-O/2015(H5N6)	A / H5N6	Asia / China	Yuhai Bi (Institute of Microbiology, Chinese Academy of Sciences)
EPI_ISL_199259	A/duck/Guangdong/04.22 DGCP062-O/2015(H5N6)	A / H5N6	Asia / China	Yuhai Bi (Institute of Microbiology, Chinese Academy of Sciences)
EPI_ISL_199260	A/goose/Guangdong/04.22 DGCPLB045-O/2015(H5N6)	A / H5N6	Asia / China	Yuhai Bi (Institute of Microbiology, Chinese Academy of Sciences)
EPI_ISL_199262	A/duck/Guangdong/04.23 DGQTXC228-O/2015(H5N6)	A / H5N6	Asia / China	Yuhai Bi (Institute of Microbiology, Chinese Academy of Sciences)
EPI_ISL_199268	A/goose/Guangdong/04.22 DGCP118-P/2015(H5N6)	A / H5N6	Asia / China	Yuhai Bi (Institute of Microbiology, Chinese Academy of Sciences)
EPI_ISL_199269	A/goose/Guangdong/04.23 DGQTXC216-P/2015(H5N6)	A / H5N6	Asia / China	Yuhai Bi (Institute of Microbiology, Chinese Academy of Sciences)
EPI_ISL_199271	A/duck/Guangdong/04.23 DGQTXC202-P/2015(H5N6)	A / H5N6	Asia / China	Yuhai Bi (Institute of Microbiology, Chinese Academy of Sciences)
EPI_ISL_199280	A/duck/Guangdong/04.22 DGCP067-O/2015(H5N6)	A / H5N6	Asia / China	Yuhai Bi (Institute of Microbiology, Chinese Academy of Sciences)
EPI_ISL_199281	A/duck/Guangdong/04.22 DGCP080-O/2015(H5N6)	A / H5N6	Asia / China	Yuhai Bi (Institute of Microbiology, Chinese Academy of Sciences)
EPI_ISL_199289	A/duck/Guangdong/04.23 DGQTXC213-P/2015(H5N6)	A / H5N6	Asia / China	Yuhai Bi (Institute of Microbiology, Chinese Academy of Sciences)
EPI_ISL_199290	A/duck/Guangdong/04.23 DGQTXC236-P/2015(H5N6)	A / H5N6	Asia / China	Yuhai Bi (Institute of Microbiology, Chinese Academy of Sciences)
EPI_ISL_199291	A/duck/Guangdong/04.23 DGQTXC213-O/2015(H5N6)	A / H5N6	Asia / China	Yuhai Bi (Institute of Microbiology, Chinese Academy of Sciences)
EPI_ISL_199292	A/duck/Guangdong/04.23 DGQTXC232-P/2015(H5N6)	A / H5N6	Asia / China	Yuhai Bi (Institute of Microbiology, Chinese Academy of Sciences)
EPI_ISL_199293	A/duck/Guangdong/04.23 DGQTSJ131-O/2015(H5N6)	A / H5N6	Asia / China	Yuhai Bi (Institute of Microbiology, Chinese Academy of Sciences)
EPI_ISL_199294	A/goose/Guangdong/04.23 DGQTXC236-O/2015(H5N6)	A / H5N6	Asia / China	Yuhai Bi (Institute of Microbiology, Chinese Academy of Sciences)
EPI_ISL_199295	A/duck/Guangdong/04.23 DGQTXC228-P/2015(H5N6)	A / H5N6	Asia / China	Yuhai Bi (Institute of Microbiology, Chinese Academy of Sciences)
EPI_ISL_199296	A/pigeon/Guangdong/04.23 DGQTXC197-O/2015(H5N6)	A / H5N6	Asia / China	Yuhai Bi (Institute of Microbiology, Chinese Academy of Sciences)
EPI_ISL_199297	A/duck/Guangdong/04.23 DGQTXC235-O/2015(H5N6)	A / H5N6	Asia / China	Yuhai Bi (Institute of Microbiology, Chinese Academy of Sciences)
EPI_ISL_199299	A/duck/Guangdong/04.22 DGCP081-O/2015(H5N6)	A / H5N6	Asia / China	Yuhai Bi (Institute of Microbiology, Chinese Academy of Sciences)
EPI_ISL_199301	A/goose/Guangdong/04.23 DGQTXC237-O1/2015(H5N6)	A / H5N6	Asia / China	Yuhai Bi (Institute of Microbiology, Chinese Academy of Sciences)
EPI_ISL_199302	A/duck/Guangdong/04.23 DGQTSJ130-O/2015(H5N6)	A / H5N6	Asia / China	Yuhai Bi (Institute of Microbiology, Chinese Academy of Sciences)
EPI_ISL_199303	A/duck/Guangdong/04.22 DGCP084-O/2015(H5N6)	A / H5N6	Asia / China	Yuhai Bi (Institute of Microbiology, Chinese Academy of Sciences)
EPI_ISL_199304	A/duck/Guangdong/04.22 DGCPLB041-P/2015(H5N6)	A / H5N6	Asia / China	Yuhai Bi (Institute of Microbiology, Chinese Academy of Sciences)
EPI_ISL_199305	A/duck/Guangdong/04.22 DGCPLB044-P/2015(H5N6)	A / H5N6	Asia / China	Yuhai Bi (Institute of Microbiology, Chinese Academy of Sciences)
EPI_ISL_199314	A/environment/Jiangxi/01.14 NCJD005/2015(H5N6)	A / H5N6	Asia / China	Yuhai Bi (Institute of Microbiology, Chinese Academy of Sciences)
EPI_ISL_199328	A/duck/Jiangxi/02.05 YGYXG015-P/2015(H6N6)	A / H5N6	Asia / China	Yuhai Bi (Institute of Microbiology, Chinese Academy of Sciences)
EPI_ISL_199337	A/duck/Jiangxi/01.14 NCJD054-P/2015(H5N6)	A / H5N6	Asia / China	Yuhai Bi (Institute of Microbiology, Chinese Academy of Sciences)
EPI_ISL_199341	A/environment/Jiangxi/01.14 NCJD027/2015(H5N6)	A / H5N6	Asia / China	Yuhai Bi (Institute of Microbiology, Chinese Academy of Sciences)
EPI_ISL_199342	A/chicken/Jiangxi/01.14 NCJD181-P/2015(H5N6)	A / H5N6	Asia / China	Yuhai Bi (Institute of Microbiology, Chinese Academy of Sciences)
EPI_ISL_199347	A/duck/Guangdong/04.23 DGQTXC230-O/2015(H5N6)	A / H5N6	Asia / China	Yuhai Bi (Institute of Microbiology, Chinese Academy of Sciences)
EPI_ISL_199380	A/duck/Guangdong/04.23 DGQTXC235-P/2015(H5N6)	A / H5N6	Asia / China	Yuhai Bi (Institute of Microbiology, Chinese Academy of Sciences)
EPI_ISL_199389	A/environment/Guangdong/03.27 DGQTSJ046/2015(H5N6)	A / H5N6	Asia / China	Yuhai Bi (Institute of Microbiology, Chinese Academy of Sciences)
EPI_ISL_199392	A/environment/Guangdong/03.27 DGQTXC056/2015(H5N6)	A / H5N6	Asia / China	Yuhai Bi (Institute of Microbiology, Chinese Academy of Sciences)
EPI_ISL_199397	A/chicken/Jiangxi/01.14 NCJD125-P/2015(H5N6)	A / H5N6	Asia / China	Yuhai Bi (Institute of Microbiology, Chinese Academy of Sciences)
EPI_ISL_199398	A/duck/Jiangxi/02.06 NCJD039-P/2015(H5N6)	A / H5N6	Asia / China	Yuhai Bi (Institute of Microbiology, Chinese Academy of Sciences)
EPI_ISL_199399	A/duck/Jiangxi/02.06 NCJD036-P/2015(H5N6)	A / H5N6	Asia / China	Yuhai Bi (Institute of Microbiology, Chinese Academy of Sciences)
EPI_ISL_199400	A/chicken/Jiangxi/02.05 YGYXG035-P/2015(H5N6)	A / H5N6	Asia / China	Yuhai Bi (Institute of Microbiology, Chinese Academy of Sciences)
EPI_ISL_199401	A/chicken/Jiangxi/02.05 YGYXG023-P/2015(H5N6)	A / H5N6	Asia / China	Yuhai Bi (Institute of Microbiology, Chinese Academy of Sciences)
EPI_ISL_199402	A/duck/Jiangxi/02.05 YGYXG038-P/2015(H5N6)	A / H5N6	Asia / China	Yuhai Bi (Institute of Microbiology, Chinese Academy of Sciences)
EPI_ISL_199403	A/duck/Guangdong/03.26 DGCP062-P/2015(H5N6)	A / H5N6	Asia / China	Yuhai Bi (Institute of Microbiology, Chinese Academy of Sciences)
EPI_ISL_199404	A/duck/Guangdong/03.26 DGCPLB007-O/2015(H5N6)	A / H5N6	Asia / China	Yuhai Bi (Institute of Microbiology, Chinese Academy of Sciences)
EPI_ISL_199405	A/duck/Guangdong/03.26 DGCPLB022-O/2015(H5N6)	A / H5N6	Asia / China	Yuhai Bi (Institute of Microbiology, Chinese Academy of Sciences)
EPI_ISL_199406	A/duck/Guangdong/03.26 DGCP076-P/2015(H5N6)	A / H5N6	Asia / China	Yuhai Bi (Institute of Microbiology, Chinese Academy of Sciences)
EPI_ISL_199407	A/duck/Guangdong/03.26 DGCP077-O/2015(H5N6)	A / H5N6	Asia / China	Yuhai Bi (Institute of Microbiology, Chinese Academy of Sciences)
EPI_ISL_199409	A/duck/Guangdong/03.26 DGCP082-O/2015(H5N6)	A / H5N6	Asia / China	Yuhai Bi (Institute of Microbiology, Chinese Academy of Sciences)
EPI_ISL_199410	A/goose/Shandong/03.16 JNRe-5/2014(H5N6)	A / H5N6	Asia / China	Yuhai Bi (Institute of Microbiology, Chinese Academy of Sciences)
EPI_ISL_199412	A/duck/Shandong/11.14 RD/2013(H5N8)	A / H5N8	Asia / China	Yuhai Bi (Institute of Microbiology, Chinese Academy of Sciences)
EPI_ISL_199414	A/goose/Shandong/02.26 GD/2015(H5N6)	A / H5N6	Asia / China	Yuhai Bi (Institute of Microbiology, Chinese Academy of Sciences)
EPI_ISL_199415	A/duck/Guangdong/03.26 DGCPLB023-O/2015(H5N6)	A / H5N6	Asia / China	Yuhai Bi (Institute of Microbiology, Chinese Academy of Sciences)
EPI_ISL_199416	A/duck/Guangdong/03.26 DGCPLB024-O/2015(H5N6)	A / H5N6	Asia / China	Yuhai Bi (Institute of Microbiology, Chinese Academy of Sciences)
EPI_ISL_199417	A/duck/Guangdong/03.26 DGCP062-O/2015(H5N6)	A / H5N6	Asia / China	Yuhai Bi (Institute of Microbiology, Chinese Academy of Sciences)
EPI_ISL_199419	A/duck/Guangxi/04.10 JX046/2015(H5N6)	A / H5N6	Asia / China	Yuhai Bi (Institute of Microbiology, Chinese Academy of Sciences)
EPI_ISL_199444	A/chicken/Guangxi/04.10 NM140/2015(H5N6)	A / H5N6	Asia / China	Yuhai Bi (Institute of Microbiology, Chinese Academy of Sciences)
EPI_ISL_199445	A/environment/Guangdong/04.15 SZBAXQshui2/2015(H5N6)	A / H5N6	Asia / China	Yuhai Bi (Institute of Microbiology, Chinese Academy of Sciences)
EPI_ISL_199446	A/chicken/Guangdong/04.15 SZBAXQ019/2015(H5N6)	A / H5N6	Asia / China	Yuhai Bi (Institute of Microbiology, Chinese Academy of Sciences)
EPI_ISL_199449	A/duck/Guangdong/04.15 SZBAXQ011/2015(H5N6)	A / H5N6	Asia / China	Yuhai Bi (Institute of Microbiology, Chinese Academy of Sciences)
EPI_ISL_199450	A/duck/Guangdong/04.15 SZBAXQ017/2015(H5N6)	A / H5N6	Asia / China	Yuhai Bi (Institute of Microbiology, Chinese Academy of Sciences)
EPI_ISL_199451	A/duck/Guangdong/04.15 SZBAXQ020/2015(H5N6)	A / H5N6	Asia / China	Yuhai Bi (Institute of Microbiology, Chinese Academy of Sciences)
EPI_ISL_199452	A/duck/Guangdong/04.15 SZBAXQ021/2015(H5N6)	A / H5N6	Asia / China	Yuhai Bi (Institute of Microbiology, Chinese Academy of Sciences)
EPI_ISL_199453	A/duck/Guangdong/04.15 SZBAXQ003/2015(H5N6)	A / H5N6	Asia / China	Yuhai Bi (Institute of Microbiology, Chinese Academy of Sciences)
EPI_ISL_199456	A/pigeon/Guangdong/04.15 SZBAXQ004/2015(H5N6)	A / H5N6	Asia / China	Yuhai Bi (Institute of Microbiology, Chinese Academy of Sciences)
EPI_ISL_199458	A/duck/Guangdong/04.16 SZLGDBL013/2015(H5N6)	A / H5N6	Asia / China	Yuhai Bi (Institute of Microbiology, Chinese Academy of Sciences)
EPI_ISL_199459	A/duck/Guangdong/04.16 SZLGDBL002/2015(H5N6)	A / H5N6	Asia / China	Yuhai Bi (Institute of Microbiology, Chinese Academy of Sciences)
EPI_ISL_199460	A/duck/Guangdong/04.16 SZLGDBL003/2015(H5N6)	A / H5N6	Asia / China	Yuhai Bi (Institute of Microbiology, Chinese Academy of Sciences)
EPI_ISL_199461	A/duck/Guangdong/04.16 SZLGDBL005/2015(H5N6)	A / H5N6	Asia / China	Yuhai Bi (Institute of Microbiology, Chinese Academy of Sciences)
EPI_ISL_199462	A/duck/Guangdong/04.16 SZLGDBL009/2015(H5N6)	A / H5N6	Asia / China	Yuhai Bi (Institute of Microbiology, Chinese Academy of Sciences)

EPI_ISL_199463	A/duck/Guangdong/04.16 SZLGWL002/2015(H5N6)	A / H5N6	Asia / China	Yuhai Bi (Institute of Microbiology, Chinese Academy of Sciences)
EPI_ISL_199465	A/pigeon/Guangdong/04.16 SZLGWL006/2015(H5N6)	A / H5N6	Asia / China	Yuhai Bi (Institute of Microbiology, Chinese Academy of Sciences)
EPI_ISL_199466	A/environment/Guangdong/04.16 SZLGWLShui1/2015(H5N6)	A / H5N6	Asia / China	Yuhai Bi (Institute of Microbiology, Chinese Academy of Sciences)
EPI_ISL_199468	A/environment/Guangdong/04.16 SZLGWLShui5/2015(H5N6)	A / H5N6	Asia / China	Yuhai Bi (Institute of Microbiology, Chinese Academy of Sciences)
EPI_ISL_199536	A/chicken/Montana/15-010559-1/2015	A / H5N2	North America / United States / Montana / Judith Basin County	
EPI_ISL_199546	A/turkey/North Dakota/15-011420-13/2015	A / H5N2	North America / United States / North Dakota / Dickey County	
EPI_ISL_199547	A/chicken/Nebraska/15-017897-1/2015	A / H5N2	North America / United States / Nebraska / Dixon County	
EPI_ISL_199548	A/turkey/Wisconsin/15-012012-2/2015	A / H5N2	North America / United States / Wisconsin / Barron County	
EPI_ISL_199549	A/turkey/North Dakota/15-013049-1/2015	A / H5N2	North America / United States / North Dakota / LaMoure County / commercial; 12 week old	
EPI_ISL_199550	A/chicken/Minnesota/15-013533-1/2015	A / H5N2	North America / United States / Minnesota / Stearns County	
EPI_ISL_199551	A/turkey/South Dakota/15-010371/2015	A / H5N2	North America / United States / South Dakota / Beadle County	
EPI_ISL_199552	A/chicken/Nebraska/15-017990-5/2015	A / H5N2	North America / United States / Nebraska / Dixon County	
EPI_ISL_199553	A/chicken/Wisconsin/15-011595-1/2015	A / H5N2	North America / United States / Wisconsin / Jefferson County	
EPI_ISL_199554	A/chicken/Wisconsin/15-012160-1/2015	A / H5N2	North America / United States / Wisconsin / Juneau County	
EPI_ISL_200819	A/chicken/Yunnan/02.09 DQXY008L/2015(H5N6)	A / H5N6	Asia / China	Yuhai Bi (Institute of Microbiology, Chinese Academy of Sciences)
EPI_ISL_200820	A/goose/Yunnan/07.14 DQWCK119/2015(H5N6)	A / H5N6	Asia / China	Yuhai Bi (Institute of Microbiology, Chinese Academy of Sciences)
EPI_ISL_200821	A/goose/Yunnan/07.14 DQWCK122/2015(H5N6)	A / H5N6	Asia / China	Yuhai Bi (Institute of Microbiology, Chinese Academy of Sciences)
EPI_ISL_200822	A/duck/Yunnan/07.15 DQNPH124/2015(H5N6)	A / H5N6	Asia / China	Yuhai Bi (Institute of Microbiology, Chinese Academy of Sciences)
EPI_ISL_200823	A/duck/Yunnan/07.15 DQNPH125/2015(H5N6)	A / H5N6	Asia / China	Yuhai Bi (Institute of Microbiology, Chinese Academy of Sciences)
EPI_ISL_200824	A/duck/Yunnan/07.15 DQNPH127/2015(H5N6)	A / H5N6	Asia / China	Yuhai Bi (Institute of Microbiology, Chinese Academy of Sciences)
EPI_ISL_200825	A/duck/Yunnan/07.15 DQNPH129/2015(H5N6)	A / H5N6	Asia / China	Yuhai Bi (Institute of Microbiology, Chinese Academy of Sciences)
EPI_ISL_200826	A/environment/Yunnan/07.11 DQENM015/2015(H5N6)	A / H5N6	Asia / China	Yuhai Bi (Institute of Microbiology, Chinese Academy of Sciences)
EPI_ISL_200827	A/environment/Yunnan/07.11 DQENM006/2015(H5N6)	A / H5N6	Asia / China	Yuhai Bi (Institute of Microbiology, Chinese Academy of Sciences)
EPI_ISL_200828	A/environment/Yunnan/07.11 DQENM004/2015(H5N6)	A / H5N6	Asia / China	Yuhai Bi (Institute of Microbiology, Chinese Academy of Sciences)
EPI_ISL_200829	A/chicken/Yunnan/07.11 DQENM005/2015(H5N6)	A / H5N6	Asia / China	Yuhai Bi (Institute of Microbiology, Chinese Academy of Sciences)
EPI_ISL_200831	A/environment/Yunnan/07.11 DQXY011/2015(H5N6)	A / H5N6	Asia / China	Yuhai Bi (Institute of Microbiology, Chinese Academy of Sciences)
EPI_ISL_200832	A/environment/Yunnan/07.11 DQXY20/2015(H5N6)	A / H5N6	Asia / China	Yuhai Bi (Institute of Microbiology, Chinese Academy of Sciences)
EPI_ISL_200833	A/environment/Yunnan/02.09 DQWGH013-2E/2015(H5N6)	A / H5N6	Asia / China	Yuhai Bi (Institute of Microbiology, Chinese Academy of Sciences)
EPI_ISL_200834	A/environment/Yunnan/02.09 DQXY005-1E/2015(H5N6)	A / H5N6	Asia / China	Yuhai Bi (Institute of Microbiology, Chinese Academy of Sciences)
EPI_ISL_200835	A/environment/Yunnan/02.09 DQXY009-1E/2015(H5N6)	A / H5N6	Asia / China	Yuhai Bi (Institute of Microbiology, Chinese Academy of Sciences)
EPI_ISL_200836	A/environment/Yunnan/02.10 DQXY053-1E/2015(H5N6)	A / H5N6	Asia / China	Yuhai Bi (Institute of Microbiology, Chinese Academy of Sciences)
EPI_ISL_200837	A/Yunnan/DQ001/2015(H5N6)	A / H5N6	Asia / China / Yunnan Province	Yuhai Bi (Institute of Microbiology, Chinese Academy of Sciences)
EPI_ISL_200838	A/Yunnan/DQ002/2015(H5N6)	A / H5N6	Asia / China / Yunnan Province	Yuhai Bi (Institute of Microbiology, Chinese Academy of Sciences)
EPI_ISL_201355	A/duck/Fujian/05.07 FZHMDK14-O/2015(H5N6)	A / H5N6	Asia / China	Yuhai Bi (Institute of Microbiology, Chinese Academy of Sciences)
EPI_ISL_201378	A/duck/Jiangxi/09.04 JX-14-11/2014(H5N6)	A / H5N6	Asia / China / Jiangxi	Yuhai Bi (Institute of Microbiology, Chinese Academy of Sciences)
EPI_ISL_201798	A/goose/Guangdong/s13124/2013	A / H5N8	Asia / China	
EPI_ISL_201799	A/duck/Guangdong/s14044/2014	A / H5N8	Asia / China	
EPI_ISL_202553	A/chicken/Guizhou/4/2013	A / H5N1	Asia / China	Huihui Kong (Harbin Veterinary Research Institute (CAAS) / Ministry of Agriculture)
EPI_ISL_202554	A/chicken/Sichuan/J1/2014	A / H5N6	Asia / China	Huihui Kong (Harbin Veterinary Research Institute (CAAS) / Ministry of Agriculture)
EPI_ISL_202555	A/chicken/Yunnan/3/2014	A / H5N1	Asia / China	Huihui Kong (Harbin Veterinary Research Institute (CAAS) / Ministry of Agriculture)
EPI_ISL_202556	A/duck/Anhui/S1300/2014	A / H5N6	Asia / China	Huihui Kong (Harbin Veterinary Research Institute (CAAS) / Ministry of Agriculture)
EPI_ISL_202558	A/duck/Liaoning/S1001/2014	A / H5N8	Asia / China	Huihui Kong (Harbin Veterinary Research Institute (CAAS) / Ministry of Agriculture)
EPI_ISL_202559	A/wild bird/Liaoning/S1660/2014	A / H5N2	Asia / China	Huihui Kong (Harbin Veterinary Research Institute (CAAS) / Ministry of Agriculture)
EPI_ISL_202763	A/environment/GZ/75/2014	A / H5N6	Asia / China / Guangdong	Jay Zhou (The University of Hong Kong / Department of Microbiology)
EPI_ISL_202764	A/environment/GZ/76/2014	A / H5N2	Asia / China / Guangdong	Jay Zhou (The University of Hong Kong / Department of Microbiology)
EPI_ISL_202765	A/environment/GZ/77/2014	A / H5N6	Asia / China / Guangdong	Jay Zhou (The University of Hong Kong / Department of Microbiology)
EPI_ISL_202766	A/environment/GZ/NIOSH-187/2014	A / H5N6	Asia / China / Guangdong Province / Guangdong	Jay Zhou (The University of Hong Kong / Department of Microbiology)
EPI_ISL_203646	A/Environment/Guangdong/79900/2014	A / H5N6	Asia / China / Guangdong	Lei Yang (WHO Chinese National Influenza Center / Virology Institute, Chinese CDC)
EPI_ISL_203647	A/Environment/Guangdong/79478/2014	A / H5N6	Asia / China / Guangdong	Lei Yang (WHO Chinese National Influenza Center / Virology Institute, Chinese CDC)
EPI_ISL_203648	A/Environment/Guangdong/79929/2014	A / H5N6	Asia / China / Guangdong	Lei Yang (WHO Chinese National Influenza Center / Virology Institute, Chinese CDC)
EPI_ISL_203649	A/Environment/Chongqing/28975/2014	A / H5N6	Asia / China / Chongqing	Lei Yang (WHO Chinese National Influenza Center / Virology Institute, Chinese CDC)
EPI_ISL_203650	A/Environment/Chongqing/72076/2014	A / H5N6	Asia / China / Chongqing	Lei Yang (WHO Chinese National Influenza Center / Virology Institute, Chinese CDC)
EPI_ISL_203651	A/Environment/Chongqing/28957/2014	A / H5N6	Asia / China / Chongqing	Lei Yang (WHO Chinese National Influenza Center / Virology Institute, Chinese CDC)
EPI_ISL_203652	A/Environment/Hunan/18327/2014	A / H5N6	Asia / China / Hunan	Lei Yang (WHO Chinese National Influenza Center / Virology Institute, Chinese CDC)
EPI_ISL_203653	A/Environment/Zhejiang/21574/2014	A / H5N6	Asia / China / Zhejiang	Lei Yang (WHO Chinese National Influenza Center / Virology Institute, Chinese CDC)
EPI_ISL_203654	A/Environment/Jiangxi/10733/2014	A / H5N6	Asia / China / Jiangxi	Lei Yang (WHO Chinese National Influenza Center / Virology Institute, Chinese CDC)
EPI_ISL_203655	A/Environment/Chongqing/28970/2014	A / H5N6	Asia / China / Chongqing	Lei Yang (WHO Chinese National Influenza Center / Virology Institute, Chinese CDC)
EPI_ISL_203731	A/duck/Zhejiang/77138/2014	A / H5N2	Asia / China	
EPI_ISL_203732	A/duck/Zhejiang/727041/2014	A / H5N2	Asia / China	
EPI_ISL_203733	A/goose/Zhejiang/77166/2014	A / H5N2	Asia / China	
EPI_ISL_203734	A/goose/Zhejiang/77167/2014	A / H5N2	Asia / China	
EPI_ISL_203735	A/goose/Zhejiang/77168/2014	A / H5N2	Asia / China	
EPI_ISL_203736	A/chicken/Zhejiang/727159/2014	A / H5N2	Asia / China	
EPI_ISL_203737	A/chicken/Zhejiang/727079/2014	A / H5N2	Asia / China	
EPI_ISL_203738	A/duck/Zhejiang/727158/2014	A / H5N6	Asia / China	
EPI_ISL_203739	A/goose/Zhejiang/727092/2014	A / H5N6	Asia / China	
EPI_ISL_203740	A/goose/Zhejiang/727110/2014	A / H5N6	Asia / China	
EPI_ISL_203741	A/goose/Zhejiang/925036/2014	A / H5N6	Asia / China	
EPI_ISL_203742	A/goose/Zhejiang/925105/2014	A / H5N6	Asia / China	
EPI_ISL_203743	A/goose/Zhejiang/925106/2014	A / H5N6	Asia / China	
EPI_ISL_203744	A/goose/Zhejiang/925108/2014	A / H5N6	Asia / China	
EPI_ISL_203745	A/goose/Zhejiang/112080/2014	A / H5N6	Asia / China	

EPI_ISL_203746	A/goose/Zhejiang/1120132/2014	A / H5N6	Asia / China	
EPI_ISL_203747	A/chicken/Zhejiang/727022/2014	A / H5N6	Asia / China	
EPI_ISL_203748	A/chicken/Zhejiang/727026/2014	A / H5N6	Asia / China	
EPI_ISL_203749	A/chicken/Zhejiang/727155/2014	A / H5N6	Asia / China	
EPI_ISL_203750	A/duck/Zhejiang/925019/2014	A / H5N8	Asia / China	
EPI_ISL_203751	A/duck/Zhejiang/925169/2014	A / H5N8	Asia / China	
EPI_ISL_203752	A/goose/Zhejiang/925037/2014	A / H5N8	Asia / China	
EPI_ISL_203753	A/goose/Zhejiang/925104/2014	A / H5N8	Asia / China	
EPI_ISL_205115	A/duck/Wuhan/WHYF02/2015	A / H5N6	Asia / China	
EPI_ISL_205116	A/duck/Wuhan/WHYF03/2015	A / H5N6	Asia / China	
EPI_ISL_205117	A/chicken/Wuhan/WHYJ01/2015	A / H5N6	Asia / China	
EPI_ISL_205118	A/chicken/Wuhan/WHYJ02/2015	A / H5N6	Asia / China	
EPI_ISL_205119	A/duck/Wenzhou/YHQL22/2014	A / H5N6	Asia / China	
EPI_ISL_205120	A/duck/Wuhan/JXYFB22/2015	A / H5N6	Asia / China	
EPI_ISL_205121	A/duck/Taizhou/TZYG12/2015	A / H5N6	Asia / China	
EPI_ISL_205137	A/turtledove/Wuhan/WHBJ12/2014	A / H5N6	Asia / China	
EPI_ISL_205138	A/turtledove/Wuhan/WHBJ28/2014	A / H5N6	Asia / China	
EPI_ISL_205139	A/turtledove/Wuhan/HKBJ27/2015	A / H5N6	Asia / China	
EPI_ISL_205140	A/turtledove/Wuhan/HKBJ43/2015	A / H5N6	Asia / China	
EPI_ISL_205141	A/turtledove/Wuhan/WHBJ26/2014	A / H5N6	Asia / China	
EPI_ISL_205142	A/turtledove/Wuhan/HKBJ07/2015	A / H5N6	Asia / China	
EPI_ISL_205313	A/Shenzhen/1/2015	A / H5N6	Asia / China / Guangdong Province / Guangdong	Lei Yang (WHO Chinese National Influenza Center / Virology Institute, Chinese CDC)
EPI_ISL_205314	A/Environment/Shenzhen/1/2015	A / H5N6	Asia / China / Guangdong	Lei Yang (WHO Chinese National Influenza Center / Virology Institute, Chinese CDC)
EPI_ISL_205315	A/Environment/Shenzhen/2/2015	A / H5N6	Asia / China / Guangdong	Lei Yang (WHO Chinese National Influenza Center / Virology Institute, Chinese CDC)
EPI_ISL_205316	A/Environment/Shenzhen/3/2015	A / H5N6	Asia / China / Guangdong	Lei Yang (WHO Chinese National Influenza Center / Virology Institute, Chinese CDC)
EPI_ISL_205503	A/Shenzhen/TH001/2015 (H5N6)	A / H5N6	Asia / China / Guangdong Province	Yuhai Bi (Institute of Microbiology, Chinese Academy of Sciences)
EPI_ISL_205821	A/peregrine_falcon/Hong_Kong/04955/2015	A / H5N6	Asia / Hong Kong (SAR)	Tak Wai Cheng (Agriculture, Fisheries and Conservation Department / Tai Lung Veterinary Laboratory)
EPI_ISL_205823	A/Oriental_magpie_robin/Hong_Kong/06154/2015	A / H5N6	Asia / Hong Kong (SAR)	Tak Wai Cheng (Agriculture, Fisheries and Conservation Department / Tai Lung Veterinary Laboratory)
EPI_ISL_205824	A/Oriental_magpie_robin/Hong_Kong/16759/2015	A / H5N6	Asia / Hong Kong (SAR)	Tak Wai Cheng (Agriculture, Fisheries and Conservation Department / Tai Lung Veterinary Laboratory)
EPI_ISL_205825	A/great_egret/Hong_Kong/00032/2016	A / H5N6	Asia / Hong Kong (SAR)	Tak Wai Cheng (Agriculture, Fisheries and Conservation Department / Tai Lung Veterinary Laboratory)
EPI_ISL_205856	A/chicken/Yunnan/1/2014	A / H5N1	Asia / China	
EPI_ISL_205959	A/Duck/Guangdong/SS1A1/2014?H5N6?	A / H5N6	Asia / China / Guangdong Province	Qi Wenbao (South China Agricultural University / Veterinary Medicine College)
EPI_ISL_205961	A/Goose/ Guangdong /JG125/2013?H5N6?	A / H5N6	Asia / China / Guangdong Province	Qi Wenbao (South China Agricultural University / Veterinary Medicine College)
EPI_ISL_205962	A/Duck/Guangdong/PY501/2014?H5N6?	A / H5N6	Asia / China / Guangdong Province	Qi Wenbao (South China Agricultural University / Veterinary Medicine College)
EPI_ISL_205963	A/Duck/Guangdong/PY503/2014?H5N6?	A / H5N6	Asia / China / Guangdong Province	Qi Wenbao (South China Agricultural University / Veterinary Medicine College)
EPI_ISL_205964	A/Chicken/Guangdong/FG594/2015?H5N6?	A / H5N6	Asia / China / Guangdong Province	Qi Wenbao (South China Agricultural University / Veterinary Medicine College)
EPI_ISL_205965	A/Enviroment/Guangdong/SS127/2013(H5N6)	A / H5N6	Asia / China / Guangdong Province	Qi Wenbao (South China Agricultural University / Veterinary Medicine College)
EPI_ISL_205966	A/Goose/ Guangdong /SSLBY/2015?H5N6?	A / H5N6	Asia / China / Guangdong Province	Qi Wenbao (South China Agricultural University / Veterinary Medicine College)
EPI_ISL_205967	A/Goose/ Guangdong /SSZYP/2015?H5N6?	A / H5N6	Asia / China / Guangdong Province	Qi Wenbao (South China Agricultural University / Veterinary Medicine College)
EPI_ISL_206036	A/Shenzhen/1/2016	A / H5N6	Asia / China / Guangdong	Lei Yang (WHO Chinese National Influenza Center / Virology Institute, Chinese CDC)
EPI_ISL_206394	A/snow goose/Missouri/15-011246-1/2015	A / H5N2	North America / United States	
EPI_ISL_206395	A/American wigeon/Utah/AH0007824/2015	A / H5N8	North America / United States	
EPI_ISL_206396	A/mallard/Oregon/AH0003952/2015	A / H5N2	North America / United States	
EPI_ISL_206397	A/Northern pintail/Oregon/AH0003967/2015	A / H5N2	North America / United States	
EPI_ISL_206398	A/wood duck/Oregon/AH0007244/2015	A / H5N2	North America / United States	
EPI_ISL_206399	A/wood duck/Oregon/AH0007257/2015	A / H5N2	North America / United States	
EPI_ISL_206400	A/wood duck/Oregon/AH0007263/2015	A / H5N2	North America / United States	
EPI_ISL_206401	A/Northern shoveler/Oregon/AH0007332/2015	A / H5N2	North America / United States	
EPI_ISL_206402	A/Northern shoveler/Oregon/AH0007337/2015	A / H5N2	North America / United States	
EPI_ISL_206403	A/Northern shoveler/Oregon/AH0007339/2015	A / H5N2	North America / United States	
EPI_ISL_206404	A/mallard/Idaho/AH0008597/2015	A / H5N8	North America / United States	
EPI_ISL_206405	A/mallard/Oregon/AH0008887/2015	A / H5N2	North America / United States	
EPI_ISL_206406	A/Northern pintail/Oregon/AH0003871/2015	A / H5N2	North America / United States	
EPI_ISL_206407	A/mallard/Idaho/AH0007412/2015	A / H5N2	North America / United States	
EPI_ISL_206408	A/mallard/Idaho/AH0007413/2015	A / H5N2	North America / United States	
EPI_ISL_206409	A/American green-winged teal/Idaho/AH0011899/2015	A / H5N2	North America / United States	
EPI_ISL_206410	A/American green-winged teal/Oregon/AH0012403/2015	A / H5N2	North America / United States	
EPI_ISL_206411	A/Canada goose/Oregon/AH0012452/2015	A / H5N8	North America / United States	
EPI_ISL_206412	A/American wigeon/Oregon/AH0012525/2015	A / H5N8	North America / United States	
EPI_ISL_206413	A/mallard/Nevada/AH0006855/2015	A / H5N8	North America / United States	
EPI_ISL_206415	A/chicken/California/15-004912/2015	A / H5N8	North America / United States	
EPI_ISL_206429	A/mallard/Idaho/AH0005954/2014	A / H5N8	North America / United States	
EPI_ISL_206430	A/mallard/Idaho/AH0005955/2014	A / H5N8	North America / United States	
EPI_ISL_206431	A/mallard/Oregon/AH0003821/2014	A / H5N2	North America / United States	
EPI_ISL_206432	A/bald eagle/Idaho/15-002892-2/2015	A / H5N8	North America / United States	
EPI_ISL_206433	A/Northern pintail/Washington/195365/2014	A / H5N2	North America / United States	
EPI_ISL_206434	A/mallard/Oregon/195547/2014	A / H5N2	North America / United States	
EPI_ISL_206435	A/American wigeon/Washington/195968/2014	A / H5N8	North America / United States	
EPI_ISL_206436	A/mallard/Washington/196262/2015	A / H5N2	North America / United States	
EPI_ISL_206437	A/Northern pintail/Washington/196271/2014	A / H5N8	North America / United States	
EPI_ISL_206438	A/mallard/Washington/196865/2015	A / H5N2	North America / United States	
EPI_ISL_206439	A/American wigeon/Washington/196336/2015	A / H5N1	North America / United States	

EPI_ISL_206440	A/American wigeon/Washington/196340/2015	A / H5N1	North America / United States	
EPI_ISL_206441	A/mallard/Oregon/195536/2014	A / H5N8	North America / United States	
EPI_ISL_206442	A/American wigeon/Washington/195198/2014	A / H5N8	North America / United States	
EPI_ISL_206443	A/American wigeon/Washington/195205/2014	A / H5N8	North America / United States	
EPI_ISL_206444	A/mallard/Washington/195246/2014	A / H5N2	North America / United States	
EPI_ISL_206445	A/peregrine falcon/Washington/196426/2014	A / H5N8	North America / United States	
EPI_ISL_206446	A/mallard/Washington/195810/2014	A / H5N2	North America / United States	
EPI_ISL_206447	A/Canada goose/Washington/197619/2014	A / H5N2	North America / United States	
EPI_ISL_206448	A/Cooper's hawk/Minnesota/198225/2015	A / H5N2	North America / United States	
EPI_ISL_206449	A/snowy owl/Wisconsin/198399/2015	A / H5N2	North America / United States	
EPI_ISL_206450	A/Canada goose/Kansas/197850/2015	A / H5N2	North America / United States	
EPI_ISL_206456	A/goose/Taiwan/TNC1/2015	A / H5N8	Asia / Taiwan	
EPI_ISL_206457	A/goose/Taiwan/TNC2/2015	A / H5N8	Asia / Taiwan	
EPI_ISL_206458	A/goose/Taiwan/TNC3/2015	A / H5N8	Asia / Taiwan	
EPI_ISL_206459	A/goose/Taiwan/TNC4/2015	A / H5N8	Asia / Taiwan	
EPI_ISL_206460	A/goose/Taiwan/TNC5/2015	A / H5N8	Asia / Taiwan	
EPI_ISL_206461	A/goose/Taiwan/TNC6/2015	A / H5N8	Asia / Taiwan	
EPI_ISL_206462	A/goose/Taiwan/TNC7/2015	A / H5N8	Asia / Taiwan	
EPI_ISL_206463	A/goose/Taiwan/TNC8/2015	A / H5N8	Asia / Taiwan	
EPI_ISL_206464	A/goose/Taiwan/TNC9/2015	A / H5N8	Asia / Taiwan	
EPI_ISL_206465	A/goose/Taiwan/TNC10/2015	A / H5N8	Asia / Taiwan	
EPI_ISL_206466	A/goose/Taiwan/TNC11/2015	A / H5N8	Asia / Taiwan	
EPI_ISL_206467	A/goose/Taiwan/TNC12/2015	A / H5N8	Asia / Taiwan	
EPI_ISL_206468	A/goose/Taiwan/TNC13/2015	A / H5N8	Asia / Taiwan	
EPI_ISL_206469	A/goose/Taiwan/TNC14/2015	A / H5N8	Asia / Taiwan	
EPI_ISL_206470	A/goose/Taiwan/TNO1/2015	A / H5N8	Asia / Taiwan	
EPI_ISL_206471	A/goose/Taiwan/TNO2/2015	A / H5N8	Asia / Taiwan	
EPI_ISL_206472	A/goose/Taiwan/TNO3/2015	A / H5N8	Asia / Taiwan	
EPI_ISL_206473	A/goose/Taiwan/TNO4/2015	A / H5N8	Asia / Taiwan	
EPI_ISL_206475	A/goose/Taiwan/TNO6/2015	A / H5N8	Asia / Taiwan	
EPI_ISL_206476	A/goose/Taiwan/TNO7/2015	A / H5N8	Asia / Taiwan	
EPI_ISL_206477	A/goose/Taiwan/TNO8/2015	A / H5N8	Asia / Taiwan	
EPI_ISL_206478	A/goose/Taiwan/TNO9/2015	A / H5N8	Asia / Taiwan	
EPI_ISL_206479	A/goose/Taiwan/TNO10/2015	A / H5N8	Asia / Taiwan	
EPI_ISL_206480	A/goose/Taiwan/TNO11/2015	A / H5N8	Asia / Taiwan	
EPI_ISL_206481	A/goose/Taiwan/TNO12/2015	A / H5N8	Asia / Taiwan	
EPI_ISL_206482	A/goose/Taiwan/TNO13/2015	A / H5N8	Asia / Taiwan	
EPI_ISL_206483	A/goose/Taiwan/TNO14/2015	A / H5N8	Asia / Taiwan	
EPI_ISL_206484	A/goose/Taiwan/TNO15/2015	A / H5N8	Asia / Taiwan	
EPI_ISL_206485	A/goose/Taiwan/TNO16/2015	A / H5N8	Asia / Taiwan	
EPI_ISL_206486	A/goose/Taiwan/TNO17/2015	A / H5N8	Asia / Taiwan	
EPI_ISL_206487	A/goose/Taiwan/TNO18/2015	A / H5N8	Asia / Taiwan	
EPI_ISL_206488	A/goose/Taiwan/TNO19/2015	A / H5N8	Asia / Taiwan	
EPI_ISL_206489	A/goose/Taiwan/TNO20/2015	A / H5N8	Asia / Taiwan	
EPI_ISL_206490	A/goose/Jiangsu/QD5/2014	A / H5N8	Asia / China	
EPI_ISL_206491	A/goose/Shandong/WFSG1/2014	A / H5N8	Asia / China	
EPI_ISL_206492	A/goose/Yangzhou/0420/2014	A / H5N8	Asia / China	
EPI_ISL_206568	A/ Guangdong /SZ872/2015?H5N6?	A / H5N6	Asia / China / Guangdong Province	Qi Wenbao (South China Agricultural University / Veterinary Medicine College)
EPI_ISL_206569	A/ Guangdong /ZQ874/2015?H5N6?	A / H5N6	Asia / China / Guangdong Province	Qi Wenbao (South China Agricultural University / Veterinary Medicine College)
EPI_ISL_206675	A/environment/Yunnan/YN19/2015	A / H5N6	Asia / China	
EPI_ISL_206676	A/environment/Yunnan/YN22/2015	A / H5N6	Asia / China	
EPI_ISL_206677	A/environment/Yunnan/YN25/2015	A / H5N6	Asia / China	
EPI_ISL_206773	A/gull/Germany-NI/R45/2015	A / H5N8	Europe / Germany / Lower Saxony	Elke Starick (Friedrich-Loeffler-Institut)
EPI_ISL_207048	A/Shenzhen/TH003/2016(H5N6)	A / H5N6	Asia / China / Guangdong Province	Yuhai Bi (Institute of Microbiology, Chinese Academy of Sciences)
EPI_ISL_207051	A/Shenzhen/TH002/2016(H5N6)	A / H5N6	Asia / China / Guangdong Province	Yuhai Bi (Institute of Microbiology, Chinese Academy of Sciences)
EPI_ISL_2081527	A/red_fox/England/AVP-M1-21-01/2020	A / H5N8	Europe / United Kingdom / Norfolk	Alex Byrne (Animal and Plant Health Agency (APHA) / Virology Department)
EPI_ISL_2081528	A/seal/England/AVP-031141/2020	A / H5N8	Europe / United Kingdom / Norfolk	Alex Byrne (Animal and Plant Health Agency (APHA) / Virology Department)
EPI_ISL_208822	A/duck/Eastern China/L0321/2010	A / H5N2	Asia / China	
EPI_ISL_208823	A/duck/Eastern China/L0230/2010	A / H5N2	Asia / China	
EPI_ISL_208824	A/duck/Eastern China/L0405/2010	A / H5N8	Asia / China	
EPI_ISL_208825	A/duck/Eastern China/L0423/2011	A / H5N8	Asia / China	
EPI_ISL_208826	A/duck/Eastern China/L0611/2011	A / H5N8	Asia / China	
EPI_ISL_208827	A/duck/Eastern China/L0722/2012	A / H5N8	Asia / China	
EPI_ISL_208828	A/duck/Eastern China/L1021/2012	A / H5N8	Asia / China	
EPI_ISL_208829	A/duck/Eastern China/L1120/2012	A / H5N8	Asia / China	
EPI_ISL_208830	A/goose/Eastern China/L1214/2012	A / H5N8	Asia / China	
EPI_ISL_208831	A/goose/Eastern China/L1204/2012	A / H5N8	Asia / China	
EPI_ISL_208832	A/duck/Eastern China/S1210/2013	A / H5N8	Asia / China	
EPI_ISL_208833	A/goose/Eastern China/S0513/2013	A / H5N6	Asia / China	
EPI_ISL_208834	A/duck/Eastern China/S0131/2014	A / H5N2	Asia / China	
EPI_ISL_208835	A/duck/Eastern China/S0215/2014	A / H5N8	Asia / China	
EPI_ISL_208836	A/duck/Eastern China/S0711/2014	A / H5N6	Asia / China	

EPI_ISL_208837	A/duck/Eastern China/S0808/2014	A / H5N2	Asia / China	
EPI_ISL_208838	A/duck/Eastern China/S0908/2014	A / H5N6	Asia / China	
EPI_ISL_208839	A/duck/Eastern China/S0322/2014	A / H5N6	Asia / China	
EPI_ISL_208840	A/goose/Eastern China/S0408/2014	A / H5N8	Asia / China	
EPI_ISL_208841	A/duck/Eastern China/S1109/2014	A / H5N8	Asia / China	
EPI_ISL_2111625	A/mute_swan/Poland/MB131/2021	A / H5N8	Europe / Poland / Pomeranian Voivodeship / Puck Bay (W?adys?awowo)	Kamila Dziadek (National Veterinary Research Institute / Department of Poultry Diseases)
EPI_ISL_2113167	A/mute_swan/Poland/MB189/2021	A / H5N8	Europe / Poland / Pomeranian Voivodeship / Gdynia	Kamila Dziadek (National Veterinary Research Institute / Department of Poultry Diseases)
EPI_ISL_2113493	A/mute_swan/Poland/MB268/2021	A / H5N8	Europe / Poland / Pomeranian Voivodeship / Puck Bay (Jastarnia)	Kamila Dziadek (National Veterinary Research Institute / Department of Poultry Diseases)
EPI_ISL_2114013	A/mute_swan/Poland/MB272/2021	A / H5N8	Europe / Poland / Pomeranian Voivodeship / Stegna	Kamila Dziadek (National Veterinary Research Institute / Department of Poultry Diseases)
EPI_ISL_2131573	A/Chicken/Sweden/SVA210420SZ0002/KN099429-IP22/2021	A / H5N8	Europe / Sweden / Skane Lan / Eslovs Kommun	Siamak Zohari (National Veterinary Institute)
EPI_ISL_213782	A/ostrich/Korea/H829/2014	A / H5N8	Asia / Korea, Republic of	
EPI_ISL_217025	A/Changsha/1/2014	A / H5N6	Asia / China	
EPI_ISL_2172517	A/common buzzard/Netherlands/21021278-002/2021	A / H5N8	Europe / Netherlands / Provincie Friesland / Ferwert	Rene Heutink (Wageningen Bioveterinary Research)
EPI_ISL_2172518	A/common kestrel/Netherlands/21021301-039/2021	A / H5N8	Europe / Netherlands / South Holland / Strijen	Rene Heutink (Wageningen Bioveterinary Research)
EPI_ISL_2172522	A/common buzzard/Netherlands/21021497-001/2021	A / H5N8	Europe / Netherlands / Provincie Noord-Holland / Rijssenhout	Rene Heutink (Wageningen Bioveterinary Research)
EPI_ISL_2172523	A/sanderling/Netherlands/21021794-002/2021	A / H5N8	Europe / Netherlands / South Holland / Hoogvliet	Rene Heutink (Wageningen Bioveterinary Research)
EPI_ISL_2172525	A/barnacle goose/Netherlands/21022039-002/2021	A / H5N8	Europe / Netherlands / South Holland / Oostvoorne	Rene Heutink (Wageningen Bioveterinary Research)
EPI_ISL_2172526	A/common buzzard/Netherlands/21023939-001/2021	A / H5N8	Europe / Netherlands / Provincie Gelderland / Rha (Bronckhorst)	Rene Heutink (Wageningen Bioveterinary Research)
EPI_ISL_2172528	A/barnacle goose/Netherlands/21024066-001/2021	A / H5N1	Europe / Netherlands / Provincie Friesland / Nes	Rene Heutink (Wageningen Bioveterinary Research)
EPI_ISL_2172529	A/wild goose/Netherlands/21024076-001/2021	A / H5N8	Europe / Netherlands / Provincie Gelderland / Apeldoorn	Rene Heutink (Wageningen Bioveterinary Research)
EPI_ISL_2172530	A/common buzzard/Netherlands/21024357-002/2021	A / H5N3	Europe / Netherlands / Provincie Friesland / Oostbierum	Rene Heutink (Wageningen Bioveterinary Research)
EPI_ISL_2172531	A/barnacle goose/Netherlands/21024358-001/2021	A / H5N8	Europe / Netherlands / Provincie Noord-Holland / Hoorn	Rene Heutink (Wageningen Bioveterinary Research)
EPI_ISL_2172532	A/gadwall/Netherlands/21024401-002/2021	A / H5N8	Europe / Netherlands / South Holland / Abbenbroek	Rene Heutink (Wageningen Bioveterinary Research)
EPI_ISL_2172533	A/common buzzard/Netherlands/21024712-002/2021	A / H5N8	Europe / Netherlands / Provincie Groningen / Leek	Rene Heutink (Wageningen Bioveterinary Research)
EPI_ISL_2172608	A/barnacle goose/Netherlands/21024897-001/2021	A / H5N8	Europe / Netherlands / South Holland / Rijswijk	Rene Heutink (Wageningen Bioveterinary Research)
EPI_ISL_2173364	A/peregrine falcon/Netherlands/21025108-001/2021	A / H5N4	Europe / Netherlands / South Holland / Brielle	Rene Heutink (Wageningen Bioveterinary Research)
EPI_ISL_2174084	A/common murre/Netherlands/21025491-002/2021	A / H5N1	Europe / Netherlands / Provincie Friesland / Schiermonnikoog	Rene Heutink (Wageningen Bioveterinary Research)
EPI_ISL_2174728	A/barnacle goose/Netherlands/21025769-002/2021	A / H5N1	Europe / Netherlands / Provincie Groningen / Termunten	Rene Heutink (Wageningen Bioveterinary Research)
EPI_ISL_2175628	A/common buzzard/Netherlands/21021187-001/2021	A / H5N8	Europe / Netherlands / Provincie Groningen / Niehoeve	Rene Heutink (Wageningen Bioveterinary Research)
EPI_ISL_2176321	A/barnacle goose/Netherlands/21023498-002/2021	A / H5N8	Europe / Netherlands / Provincie Noord-Holland / Oosterend	Rene Heutink (Wageningen Bioveterinary Research)
EPI_ISL_2176841	A/barnacle goose/Netherlands/21023501-002/2021	A / H5N8	Europe / Netherlands / Provincie Noord-Holland / Den Oever	Rene Heutink (Wageningen Bioveterinary Research)
EPI_ISL_2177398	A/peacock/Netherlands/21026542-001/2021	A / H5N8	Europe / Netherlands / Provincie Noord-Holland / Hoorn	Rene Heutink (Wageningen Bioveterinary Research)
EPI_ISL_217939	A/Waterfowl/Hubei/Chenhu1306/2014_H5N6	A / H5N6	Asia / China / Hubei Province	Jianjun Chen (Wuhan Institute of Virology / Chinese Academy of Sciences)
EPI_ISL_217940	A/Waterfowl/Hubei/Chenhu1347/2014_H5N6	A / H5N6	Asia / China / Hubei Province	Jianjun Chen (Wuhan Institute of Virology / Chinese Academy of Sciences)
EPI_ISL_217941	A/Anas crecca/Hubei/Chenhu1623-5/2014_H5N6	A / H5N6	Asia / China / Hubei Province	Jianjun Chen (Wuhan Institute of Virology / Chinese Academy of Sciences)
EPI_ISL_218200	A/Bar-headed_goose/Qinghai/X402/2015_H5N6	A / H5N6	Asia / China / Qinghai Province	Jianjun Chen (Wuhan Institute of Virology / Chinese Academy of Sciences)
EPI_ISL_218201	A/Bar-headed_goose/Qinghai/X417/2015_H5N6	A / H5N6	Asia / China / Qinghai Province	Jianjun Chen (Wuhan Institute of Virology / Chinese Academy of Sciences)
EPI_ISL_218202	A/Bar-headed_goose/Qinghai/X418-1/2015_H5N6	A / H5N6	Asia / China / Qinghai Province	Jianjun Chen (Wuhan Institute of Virology / Chinese Academy of Sciences)
EPI_ISL_218203	A/Bar-headed_goose/Qinghai/X418-2/2015_H5N6	A / H5N6	Asia / China / Qinghai Province	Jianjun Chen (Wuhan Institute of Virology / Chinese Academy of Sciences)
EPI_ISL_218204	A/Bar-headed_goose/Qinghai/X424/2015_H5N6	A / H5N6	Asia / China / Qinghai Province	Jianjun Chen (Wuhan Institute of Virology / Chinese Academy of Sciences)
EPI_ISL_218205	A/Bar-headed_goose/Qinghai/X428/2015_H5N6	A / H5N6	Asia / China / Qinghai Province	Jianjun Chen (Wuhan Institute of Virology / Chinese Academy of Sciences)
EPI_ISL_218206	A/Bar-headed_goose/Qinghai/X430/2015_H5N6	A / H5N6	Asia / China / Qinghai Province	Jianjun Chen (Wuhan Institute of Virology / Chinese Academy of Sciences)
EPI_ISL_218207	A/Bar-headed_goose/Qinghai/X437/2015_H5N6	A / H5N6	Asia / China / Qinghai Province	Jianjun Chen (Wuhan Institute of Virology / Chinese Academy of Sciences)
EPI_ISL_2193998	A/barnacle goose/Netherlands/21027016-002/2021	A / H5N1	Europe / Netherlands / Provincie Groningen / Pieterburen	Rene Heutink (Wageningen Bioveterinary Research)
EPI_ISL_2194014	A/white-tailed eagle/Netherlands/21027616-001/2021	A / H5N1	Europe / Netherlands / Provincie Groningen / Noordlaren	Rene Heutink (Wageningen Bioveterinary Research)
EPI_ISL_2194036	A/barnacle goose/Netherlands/21028196-002/2021	A / H5N1	Europe / Netherlands / Provincie Groningen / Onnen	Rene Heutink (Wageningen Bioveterinary Research)
EPI_ISL_2194218	A/red fox/Netherlands/21028774-002/2021	A / H5N1	Europe / Netherlands / Provincie Groningen / Bellingwolde	Rene Heutink (Wageningen Bioveterinary Research)
EPI_ISL_2194219	A/red fox/Netherlands/21028774-004/2021	A / H5N1	Europe / Netherlands / Provincie Groningen / Bellingwolde	Rene Heutink (Wageningen Bioveterinary Research)
EPI_ISL_2194543	A/chicken/Czech Republic/6151-2/2021	A / H5N8	Europe / Czech Republic / Kralovehradecky Kraj / Okres Hradec Kralove / Kosicky	Alexander Nagy (State Veterinary Institute Prague)
EPI_ISL_219760	A/Environment/Hunan/28147/2014	A / H5N1	Asia / China / Hunan	Lei Yang (WHO Chinese National Influenza Center / Virology Institute, Chinese CDC)
EPI_ISL_219770	A/Environment/Hunan/18459/2014	A / H5N1	Asia / China / Hunan	Lei Yang (WHO Chinese National Influenza Center / Virology Institute, Chinese CDC)
EPI_ISL_219771	A/Environment/Hunan/25952/2014	A / H5N1	Asia / China / Hunan	Lei Yang (WHO Chinese National Influenza Center / Virology Institute, Chinese CDC)
EPI_ISL_219776	A/Environment/Hunan/18478/2014	A / H5N1	Asia / China / Hunan	Lei Yang (WHO Chinese National Influenza Center / Virology Institute, Chinese CDC)
EPI_ISL_219777	A/Environment/Chongqing/45355/2015	A / H5N2	Asia / China / Chongqing	Lei Yang (WHO Chinese National Influenza Center / Virology Institute, Chinese CDC)
EPI_ISL_219778	A/Environment/Hubei/45047/2015	A / H5N2	Asia / China / Hubei	Lei Yang (WHO Chinese National Influenza Center / Virology Institute, Chinese CDC)
EPI_ISL_219779	A/Environment/Hubei/45046/2015	A / H5N2	Asia / China / Hubei	Lei Yang (WHO Chinese National Influenza Center / Virology Institute, Chinese CDC)
EPI_ISL_219780	A/Environment/Qinghai/42934/2015	A / H5N2	Asia / China / Qinghai	Lei Yang (WHO Chinese National Influenza Center / Virology Institute, Chinese CDC)
EPI_ISL_219781	A/Environment/Qinghai/39266/2015	A / H5N2	Asia / China / Qinghai	Lei Yang (WHO Chinese National Influenza Center / Virology Institute, Chinese CDC)
EPI_ISL_219782	A/Environment/Chongqing/47485/2015	A / H5N6	Asia / China / Chongqing	Lei Yang (WHO Chinese National Influenza Center / Virology Institute, Chinese CDC)
EPI_ISL_219783	A/Environment/Guangxi/46690/2015	A / H5N6	Asia / China / Guangxi	Lei Yang (WHO Chinese National Influenza Center / Virology Institute, Chinese CDC)
EPI_ISL_219784	A/Environment/Chongqing/45440/2014	A / H5N6	Asia / China / Chongqing	Lei Yang (WHO Chinese National Influenza Center / Virology Institute, Chinese CDC)
EPI_ISL_219785	A/Environment/Chongqing/45439/2014	A / H5N6	Asia / China / Chongqing	Lei Yang (WHO Chinese National Influenza Center / Virology Institute, Chinese CDC)
EPI_ISL_219786	A/Environment/Chongqing/45378/2015	A / H5N6	Asia / China / Chongqing	Lei Yang (WHO Chinese National Influenza Center / Virology Institute, Chinese CDC)
EPI_ISL_219787	A/Environment/Chongqing/45373/2015	A / H5N6	Asia / China / Chongqing	Lei Yang (WHO Chinese National Influenza Center / Virology Institute, Chinese CDC)
EPI_ISL_219788	A/Environment/Chongqing/45349/2015	A / H5N6	Asia / China / Chongqing	Lei Yang (WHO Chinese National Influenza Center / Virology Institute, Chinese CDC)
EPI_ISL_219789	A/Environment/Chongqing/45379/2015	A / H5N6	Asia / China / Chongqing	Lei Yang (WHO Chinese National Influenza Center / Virology Institute, Chinese CDC)
EPI_ISL_219790	A/Environment/Chongqing/45208/2015	A / H5N6	Asia / China / Chongqing	Lei Yang (WHO Chinese National Influenza Center / Virology Institute, Chinese CDC)
EPI_ISL_219791	A/Environment/Chongqing/45221/2015	A / H5N6	Asia / China / Chongqing	Lei Yang (WHO Chinese National Influenza Center / Virology Institute, Chinese CDC)
EPI_ISL_219792	A/Environment/Chongqing/45185/2015	A / H5N6	Asia / China / Chongqing	Lei Yang (WHO Chinese National Influenza Center / Virology Institute, Chinese CDC)
EPI_ISL_219793	A/Environment/Chongqing/45155/2015	A / H5N6	Asia / China / Chongqing	Lei Yang (WHO Chinese National Influenza Center / Virology Institute, Chinese CDC)
EPI_ISL_219794	A/Environment/Chongqing/45114/2015	A / H5N6	Asia / China / Chongqing	Lei Yang (WHO Chinese National Influenza Center / Virology Institute, Chinese CDC)
EPI_ISL_219795	A/Environment/Chongqing/45134/2015	A / H5N6	Asia / China / Chongqing	Lei Yang (WHO Chinese National Influenza Center / Virology Institute, Chinese CDC)

EPI_ISL_219796	A/Environment/Hubei/45035/2015	A / H5N6	Asia / China / Hubei	Lei Yang (WHO Chinese National Influenza Center / Virology Institute, Chinese CDC)
EPI_ISL_219797	A/Environment/Hubei/45034/2015	A / H5N6	Asia / China / Hubei	Lei Yang (WHO Chinese National Influenza Center / Virology Institute, Chinese CDC)
EPI_ISL_219798	A/Environment/Guangxi/44389/2015	A / H5N6	Asia / China / Guangxi	Lei Yang (WHO Chinese National Influenza Center / Virology Institute, Chinese CDC)
EPI_ISL_219799	A/Environment/Chongqing/99173/2014	A / H5N6	Asia / China / Chongqing	Lei Yang (WHO Chinese National Influenza Center / Virology Institute, Chinese CDC)
EPI_ISL_219800	A/Environment/Guangxi/42586/2015	A / H5N6	Asia / China / Guangxi	Lei Yang (WHO Chinese National Influenza Center / Virology Institute, Chinese CDC)
EPI_ISL_219801	A/Environment/Anhui/72105/2014	A / H5N6	Asia / China / Anhui	Lei Yang (WHO Chinese National Influenza Center / Virology Institute, Chinese CDC)
EPI_ISL_219802	A/Environment/Chongqing/77193/2014	A / H5N6	Asia / China / Chongqing	Lei Yang (WHO Chinese National Influenza Center / Virology Institute, Chinese CDC)
EPI_ISL_219803	A/Environment/Guangdong/77245/2014	A / H5N6	Asia / China / Guangdong	Lei Yang (WHO Chinese National Influenza Center / Virology Institute, Chinese CDC)
EPI_ISL_219804	A/Environment/Hunan/18487/2014	A / H5N6	Asia / China / Hunan	Lei Yang (WHO Chinese National Influenza Center / Virology Institute, Chinese CDC)
EPI_ISL_219805	A/Environment/Jiangxi/10649/2014	A / H5N6	Asia / China / Jiangxi	Lei Yang (WHO Chinese National Influenza Center / Virology Institute, Chinese CDC)
EPI_ISL_219806	A/Environment/Jiangxi/10717/2014	A / H5N6	Asia / China / Jiangxi	Lei Yang (WHO Chinese National Influenza Center / Virology Institute, Chinese CDC)
EPI_ISL_219807	A/Environment/Guangdong/40929/2015	A / H5N6	Asia / China / Guangdong	Lei Yang (WHO Chinese National Influenza Center / Virology Institute, Chinese CDC)
EPI_ISL_219808	A/Environment/Guangdong/40113/2015	A / H5N6	Asia / China / Guangdong	Lei Yang (WHO Chinese National Influenza Center / Virology Institute, Chinese CDC)
EPI_ISL_219809	A/Environment/Chongqing/72096/2014	A / H5N6	Asia / China / Chongqing	Lei Yang (WHO Chinese National Influenza Center / Virology Institute, Chinese CDC)
EPI_ISL_219810	A/Environment/Yunnan/39607/2015	A / H5N6	Asia / China / Yunnan	Lei Yang (WHO Chinese National Influenza Center / Virology Institute, Chinese CDC)
EPI_ISL_219811	A/Environment/Hubei/38005/2014	A / H5N6	Asia / China / Hubei	Lei Yang (WHO Chinese National Influenza Center / Virology Institute, Chinese CDC)
EPI_ISL_219812	A/Environment/Hubei/37983/2014	A / H5N6	Asia / China / Hubei	Lei Yang (WHO Chinese National Influenza Center / Virology Institute, Chinese CDC)
EPI_ISL_219813	A/Environment/Guangdong/33311/2015	A / H5N6	Asia / China / Guangdong	Lei Yang (WHO Chinese National Influenza Center / Virology Institute, Chinese CDC)
EPI_ISL_219814	A/Environment/Guangdong/21131/2015	A / H5N6	Asia / China / Guangdong	Lei Yang (WHO Chinese National Influenza Center / Virology Institute, Chinese CDC)
EPI_ISL_219815	A/Environment/Guangdong/21109/2015	A / H5N6	Asia / China / Guangdong	Lei Yang (WHO Chinese National Influenza Center / Virology Institute, Chinese CDC)
EPI_ISL_219816	A/Environment/Hunan/07767/2015	A / H5N6	Asia / China / Hunan	Lei Yang (WHO Chinese National Influenza Center / Virology Institute, Chinese CDC)
EPI_ISL_219817	A/Environment/Hunan/07681/2015	A / H5N6	Asia / China / Hunan	Lei Yang (WHO Chinese National Influenza Center / Virology Institute, Chinese CDC)
EPI_ISL_219818	A/Environment/Xinjiang/07002/2014	A / H5N6	Asia / China / Xinjiang	Lei Yang (WHO Chinese National Influenza Center / Virology Institute, Chinese CDC)
EPI_ISL_219819	A/Environment/Chongqing/99178/2014	A / H5N6	Asia / China / Chongqing	Lei Yang (WHO Chinese National Influenza Center / Virology Institute, Chinese CDC)
EPI_ISL_219820	A/Environment/Xinjiang/00320/2014	A / H5N6	Asia / China / Xinjiang	Lei Yang (WHO Chinese National Influenza Center / Virology Institute, Chinese CDC)
EPI_ISL_219821	A/Environment/Xinjiang/00318/2014	A / H5N6	Asia / China / Xinjiang	Lei Yang (WHO Chinese National Influenza Center / Virology Institute, Chinese CDC)
EPI_ISL_219822	A/Environment/Xinjiang/00300/2014	A / H5N6	Asia / China / Xinjiang	Lei Yang (WHO Chinese National Influenza Center / Virology Institute, Chinese CDC)
EPI_ISL_219823	A/Environment/Guangdong/00299/2014	A / H5N6	Asia / China / Guangdong	Lei Yang (WHO Chinese National Influenza Center / Virology Institute, Chinese CDC)
EPI_ISL_219824	A/Environment/Xinjiang/00324/2014	A / H5N6	Asia / China / Xinjiang	Lei Yang (WHO Chinese National Influenza Center / Virology Institute, Chinese CDC)
EPI_ISL_219825	A/Environment/Hunan/98885/2014	A / H5N6	Asia / China / Hunan	Lei Yang (WHO Chinese National Influenza Center / Virology Institute, Chinese CDC)
EPI_ISL_219826	A/Environment/Chongqing/99181/2014	A / H5N6	Asia / China / Chongqing	Lei Yang (WHO Chinese National Influenza Center / Virology Institute, Chinese CDC)
EPI_ISL_219827	A/Environment/Chongqing/99166/2014	A / H5N6	Asia / China / Chongqing	Lei Yang (WHO Chinese National Influenza Center / Virology Institute, Chinese CDC)
EPI_ISL_219828	A/Guangdong/99710/2014	A / H5N6	Asia / China / Guangdong	Lei Yang (WHO Chinese National Influenza Center / Virology Institute, Chinese CDC)
EPI_ISL_219829	A/Environment/Jiangsu/98335/2014	A / H5N8	Asia / China / Jiangsu	Lei Yang (WHO Chinese National Influenza Center / Virology Institute, Chinese CDC)
EPI_ISL_219844	A/Environment/Hunan/72935/2014	A / H5N2	Asia / China / Hunan	Lei Yang (WHO Chinese National Influenza Center / Virology Institute, Chinese CDC)
EPI_ISL_219872	A/goose/Taiwan/01038/2015	A / H5N3	Asia / Taiwan	
EPI_ISL_219873	A/chicken/Taiwan/01174/2015	A / H5N3	Asia / Taiwan	
EPI_ISL_219874	A/duck/Taiwan/A3400/2015	A / H5N8	Asia / Taiwan	
EPI_ISL_219875	A/goose/Taiwan/01019/2015	A / H5N8	Asia / Taiwan	
EPI_ISL_219876	A/goose/Taiwan/01026/2015	A / H5N8	Asia / Taiwan	
EPI_ISL_219877	A/goose/Taiwan/01039/2015	A / H5N8	Asia / Taiwan	
EPI_ISL_219878	A/duck/Taiwan/01006/2015	A / H5N2	Asia / Taiwan	
EPI_ISL_219879	A/goose/Taiwan/01022/2015	A / H5N2	Asia / Taiwan	
EPI_ISL_219880	A/goose/Taiwan/01023/2015	A / H5N2	Asia / Taiwan	
EPI_ISL_219881	A/goose/Taiwan/01031/2015	A / H5N2	Asia / Taiwan	
EPI_ISL_219882	A/goose/Taiwan/01040/2015	A / H5N2	Asia / Taiwan	
EPI_ISL_221389	A/environment/Guangdong/GZ693/2015	A / H5N6	Asia / China	
EPI_ISL_221390	A/environment/Guangdong/ZS558/2015	A / H5N6	Asia / China	
EPI_ISL_221391	A/environment/Guangdong/GZ670/2015	A / H5N6	Asia / China	
EPI_ISL_221705	A/duck/Guangdong/01.01 SZSGXJK001-Y /2016	A / H5N6	Asia / China / Guangdong Province	Yuhai Bi (Institute of Microbiology, Chinese Academy of Sciences)
EPI_ISL_221706	A/duck/Guangdong/01.01 SZSGXJK001-G/2016	A / H5N6	Asia / China / Guangdong Province	Yuhai Bi (Institute of Microbiology, Chinese Academy of Sciences)
EPI_ISL_221707	A/duck/Guangdong/01.01 SZSGXJK002-Y/2016	A / H5N6	Asia / China / Guangdong Province	Yuhai Bi (Institute of Microbiology, Chinese Academy of Sciences)
EPI_ISL_221708	A/duck/Guangdong/01.01 SZSGXJK002-G/2016	A / H5N6	Asia / China / Guangdong Province	Yuhai Bi (Institute of Microbiology, Chinese Academy of Sciences)
EPI_ISL_221709	A/duck/Guangdong/01.01 SZSGXJK003-G/2016	A / H5N6	Asia / China / Guangdong Province	Yuhai Bi (Institute of Microbiology, Chinese Academy of Sciences)
EPI_ISL_221710	A/duck/Guangdong/01.01 SZSGXJK004-Y/2016	A / H5N6	Asia / China / Guangdong Province	Yuhai Bi (Institute of Microbiology, Chinese Academy of Sciences)
EPI_ISL_221711	A/duck/Guangdong/01.01 SZSGXJK003-W/2016	A / H5N6	Asia / China / Guangdong Province	Yuhai Bi (Institute of Microbiology, Chinese Academy of Sciences)
EPI_ISL_221712	A/duck/Guangdong/01.01 SZSGXJK005-Y/2016	A / H5N6	Asia / China / Guangdong Province	Yuhai Bi (Institute of Microbiology, Chinese Academy of Sciences)
EPI_ISL_221713	A/duck/Guangdong/01.01 SZSGXJK005-G /2016	A / H5N6	Asia / China / Guangdong Province	Yuhai Bi (Institute of Microbiology, Chinese Academy of Sciences)
EPI_ISL_221714	A/duck/Guangdong/01.01 SZSGXJK006-Y/2016	A / H5N6	Asia / China / Guangdong Province	Yuhai Bi (Institute of Microbiology, Chinese Academy of Sciences)
EPI_ISL_221715	A/duck/Guangdong/01.01 SZSGXJK006-G/2016	A / H5N6	Asia / China / Guangdong Province	Yuhai Bi (Institute of Microbiology, Chinese Academy of Sciences)
EPI_ISL_221716	A/duck/Guangdong/01.01 SZSGXJK007-G/2016	A / H5N6	Asia / China / Guangdong Province	Yuhai Bi (Institute of Microbiology, Chinese Academy of Sciences)
EPI_ISL_221719	A/environment/Guangdong/01.01 SZSGXJK006-E/2016	A / H5N6	Asia / China / Guangdong Province	Yuhai Bi (Institute of Microbiology, Chinese Academy of Sciences)
EPI_ISL_221720	A/feline/Guangdong/1/2015	A / H5N6	Asia / China	
EPI_ISL_221721	A/feline/Guangdong/2/2015	A / H5N6	Asia / China	
EPI_ISL_221723	A/tiger/Yunnan/tig1404/2014	A / H5N1	Asia / China	
EPI_ISL_221885	A/chicken/Guangdong/10.16 SZLGKQHY-072/2015	A / H5N6	Asia / China / Guangdong Province	Yuhai Bi (Institute of Microbiology, Chinese Academy of Sciences)
EPI_ISL_222132	A/duck/Eastern China/JY/2014	A / H5N8	Asia / China	
EPI_ISL_222134	A/goose/Eastern China/CZ/2013	A / H5N8	Asia / China	
EPI_ISL_2227275	A/turkey/Netherlands/21028936-001005/2021	A / H5N8	Europe / Netherlands / Provincie Limburg / Weert	Rene Heutink (Wageningen Bioveterinary Research)
EPI_ISL_2227276	A/barnacle goose/Netherlands/21027357-002/2021	A / H5N1	Europe / Netherlands / Provincie Groningen / Termunten	Rene Heutink (Wageningen Bioveterinary Research)
EPI_ISL_2227277	A/barnacle goose/Netherlands/21028534-002/2021	A / H5N1	Europe / Netherlands / Provincie Groningen / Waterhuizen	Rene Heutink (Wageningen Bioveterinary Research)
EPI_ISL_2227278	A/goose/Netherlands/21028502-002/21028502/2021	A / H5N1	Europe / Netherlands / Provincie Groningen / Houwerzijl	Rene Heutink (Wageningen Bioveterinary Research)

EPI_ISL_2227279	A/western marsh harrier/Netherlands/21028606-002/2021	A / H5N1	Europe / Netherlands / Provincie Friesland / Ooststellingwerf	Rene Heutink (Wageningen Bioveterinary Research)
EPI_ISL_223364	A/chicken/Wuxi/SC7393/2015	A / H5N6	Asia / China / Jiangsu Province	Maijuan Ma (Beijing Institute of Microbiology and Epidemiology / State Key Laboratory of Pathogen and Biosecurity)
EPI_ISL_2234807	A/knot_wader/Ireland/472_21VIR2956-1/2021	A / H5N3	Europe / Ireland	Bianca Zecchin (Istituto Zooprofilattico Sperimentale Delle Venezie)
EPI_ISL_2234808	A/knot_wader/Ireland/473_21VIR2956-2/2021	A / H5N3	Europe / Ireland	Bianca Zecchin (Istituto Zooprofilattico Sperimentale Delle Venezie)
EPI_ISL_2234809	A/yellow-legged_gull/Switzerland/15-0039_21VIR3035/2021	A / H5N4	Europe / Switzerland	Bianca Zecchin (Istituto Zooprofilattico Sperimentale Delle Venezie)
EPI_ISL_2234810	A/seagull/Italy/21VIR2479/2021	A / H5N8	Europe / Italy	Bianca Zecchin (Istituto Zooprofilattico Sperimentale Delle Venezie)
EPI_ISL_2234811	A/anser_anser/Spain/102-8_21VIR1230-4/2021	A / H5N8	Europe / Spain	Bianca Zecchin (Istituto Zooprofilattico Sperimentale Delle Venezie)
EPI_ISL_2234812	A/anser_anser/Spain/297-1_21VIR1230-5/2021	A / H5N8	Europe / Spain	Bianca Zecchin (Istituto Zooprofilattico Sperimentale Delle Venezie)
EPI_ISL_2234813	A/ciconia_ciconia/Spain/102-1_21VIR1230-2/2021	A / H5N8	Europe / Spain	Bianca Zecchin (Istituto Zooprofilattico Sperimentale Delle Venezie)
EPI_ISL_2234814	A/peregrine_falcon/Spain/3365-1_21VIR1230-1/2020	A / H5N8	Europe / Spain	Bianca Zecchin (Istituto Zooprofilattico Sperimentale Delle Venezie)
EPI_ISL_2234815	A/duck/Romania/10202_21VIR2593-31/2021	A / H5N8	Europe / Romania	Bianca Zecchin (Istituto Zooprofilattico Sperimentale Delle Venezie)
EPI_ISL_2234816	A/duck/Romania/10205-t4_21VIR2593-3/2021	A / H5N8	Europe / Romania	Bianca Zecchin (Istituto Zooprofilattico Sperimentale Delle Venezie)
EPI_ISL_2234817	A/goose/Romania/10205-t3_21VIR2593-7/2021	A / H5N8	Europe / Romania	Bianca Zecchin (Istituto Zooprofilattico Sperimentale Delle Venezie)
EPI_ISL_2234818	A/goose/Romania/10205-t5_21VIR2593-8/2021	A / H5N8	Europe / Romania	Bianca Zecchin (Istituto Zooprofilattico Sperimentale Delle Venezie)
EPI_ISL_2234819	A/goose/Romania/10330_21VIR2593-35/2021	A / H5N8	Europe / Romania	Bianca Zecchin (Istituto Zooprofilattico Sperimentale Delle Venezie)
EPI_ISL_2234820	A/hawk/Romania/10363_21VIR2593-11/2021	A / H5N8	Europe / Romania	Bianca Zecchin (Istituto Zooprofilattico Sperimentale Delle Venezie)
EPI_ISL_2234821	A/whooper_swan/Romania/10122_21VIR2593-23/2021	A / H5N5	Europe / Romania	Bianca Zecchin (Istituto Zooprofilattico Sperimentale Delle Venezie)
EPI_ISL_2234822	A/whooper_swan/Romania/10171_21VIR2593-15/2021	A / H5N5	Europe / Romania	Bianca Zecchin (Istituto Zooprofilattico Sperimentale Delle Venezie)
EPI_ISL_2234823	A/whooper_swan/Romania/10213_21VIR2593-27/2021	A / H5N5	Europe / Romania	Bianca Zecchin (Istituto Zooprofilattico Sperimentale Delle Venezie)
EPI_ISL_2234824	A/whooper_swan/Romania/10362_21VIR2593-20/2021	A / H5N5	Europe / Romania	Bianca Zecchin (Istituto Zooprofilattico Sperimentale Delle Venezie)
EPI_ISL_2234831	A/peregrine_falcon/Northern_Ireland/21VIR1662-3/2021	A / H5N3	Europe / United Kingdom	Bianca Zecchin (Istituto Zooprofilattico Sperimentale Delle Venezie)
EPI_ISL_224580	A/great crested grebe/Uvs-Nuur Lake/341/2016	A / H5N8	Europe / Russian Federation / Tyva Republic	Ivan Sobolev (Research Institute of Experimental and Clinical Medicine)
EPI_ISL_224704	A/Bar-headed Goose/Qinghai/BTY1-B/2016	A / H5N8	Asia / China / Qinghai Province	Jianjun Chen (Wuhan Institute of Virology / Chinese Academy of Sciences)
EPI_ISL_224705	A/Bar-headed Goose/Qinghai/BTY1-LV/2016	A / H5N8	Asia / China / Qinghai Province	Jianjun Chen (Wuhan Institute of Virology / Chinese Academy of Sciences)
EPI_ISL_224707	A/Bar-headed Goose/Qinghai/BTY2-B/2016	A / H5N8	Asia / China / Qinghai Province	Jianjun Chen (Wuhan Institute of Virology / Chinese Academy of Sciences)
EPI_ISL_224708	A/Bar-headed Goose/Qinghai/BTY2-LU/2016	A / H5N8	Asia / China / Qinghai Province	Jianjun Chen (Wuhan Institute of Virology / Chinese Academy of Sciences)
EPI_ISL_224709	A/Bar-headed Goose/Qinghai/BTY3-B/2016	A / H5N8	Asia / China / Qinghai Province	Jianjun Chen (Wuhan Institute of Virology / Chinese Academy of Sciences)
EPI_ISL_224710	A/Bar-headed Goose/Qinghai/BTY3-LU/2016	A / H5N8	Asia / China / Qinghai Province	Jianjun Chen (Wuhan Institute of Virology / Chinese Academy of Sciences)
EPI_ISL_224711	A/Bar-headed Goose/Qinghai/BTY4-B/2016	A / H5N8	Asia / China / Qinghai Province	Jianjun Chen (Wuhan Institute of Virology / Chinese Academy of Sciences)
EPI_ISL_224712	A/Bar-headed Goose/Qinghai/BTY4-LU/2016	A / H5N8	Asia / China / Qinghai Province	Jianjun Chen (Wuhan Institute of Virology / Chinese Academy of Sciences)
EPI_ISL_224713	A/Bar-headed Goose/Qinghai/BTY5-B/2016	A / H5N8	Asia / China / Qinghai Province	Jianjun Chen (Wuhan Institute of Virology / Chinese Academy of Sciences)
EPI_ISL_224714	A/Bar-headed Goose/Qinghai/BTY6-B/2016	A / H5N8	Asia / China / Qinghai Province	Jianjun Chen (Wuhan Institute of Virology / Chinese Academy of Sciences)
EPI_ISL_224715	A/Bar-headed Goose/Qinghai/BTY6-LU/2016	A / H5N8	Asia / China / Qinghai Province	Jianjun Chen (Wuhan Institute of Virology / Chinese Academy of Sciences)
EPI_ISL_224716	A/Bar-headed Goose/Qinghai/BTY7-B/2016	A / H5N8	Asia / China / Qinghai Province	Jianjun Chen (Wuhan Institute of Virology / Chinese Academy of Sciences)
EPI_ISL_224717	A/Bar-headed Goose/Qinghai/BTY7-LU1/2016	A / H5N8	Asia / China / Qinghai Province	Jianjun Chen (Wuhan Institute of Virology / Chinese Academy of Sciences)
EPI_ISL_224718	A/Bar-headed Goose/Qinghai/BTY7-LU2/2016	A / H5N8	Asia / China / Qinghai Province	Jianjun Chen (Wuhan Institute of Virology / Chinese Academy of Sciences)
EPI_ISL_224719	A/Bar-headed Goose/Qinghai/BTY8-B/2016	A / H5N8	Asia / China / Qinghai Province	Jianjun Chen (Wuhan Institute of Virology / Chinese Academy of Sciences)
EPI_ISL_224720	A/Bar-headed Goose/Qinghai/BTY8-LU/2016	A / H5N8	Asia / China / Qinghai Province	Jianjun Chen (Wuhan Institute of Virology / Chinese Academy of Sciences)
EPI_ISL_224721	A/Bar-headed Goose/Qinghai/BTY9-B/2016	A / H5N8	Asia / China / Qinghai Province	Jianjun Chen (Wuhan Institute of Virology / Chinese Academy of Sciences)
EPI_ISL_224722	A/Bar-headed Goose/Qinghai/BTY9-LU/2016	A / H5N8	Asia / China / Qinghai Province	Jianjun Chen (Wuhan Institute of Virology / Chinese Academy of Sciences)
EPI_ISL_224723	A/Bar-headed Goose/Qinghai/BTY10-B/2016	A / H5N8	Asia / China / Qinghai Province	Jianjun Chen (Wuhan Institute of Virology / Chinese Academy of Sciences)
EPI_ISL_224724	A/Bar-headed Goose/Qinghai/BTY10-LU/2016	A / H5N8	Asia / China / Qinghai Province	Jianjun Chen (Wuhan Institute of Virology / Chinese Academy of Sciences)
EPI_ISL_224725	A/Bar-headed Goose/Qinghai/BTY11-B/2016	A / H5N8	Asia / China / Qinghai Province	Jianjun Chen (Wuhan Institute of Virology / Chinese Academy of Sciences)
EPI_ISL_224726	A/Bar-headed Goose/Qinghai/BTY11-LU/2016	A / H5N8	Asia / China / Qinghai Province	Jianjun Chen (Wuhan Institute of Virology / Chinese Academy of Sciences)
EPI_ISL_224727	A/Bar-headed Goose/Qinghai/BTY12-B/2016	A / H5N8	Asia / China / Qinghai Province	Jianjun Chen (Wuhan Institute of Virology / Chinese Academy of Sciences)
EPI_ISL_224728	A/Bar-headed Goose/Qinghai/BTY12-LU/2016	A / H5N8	Asia / China / Qinghai Province	Jianjun Chen (Wuhan Institute of Virology / Chinese Academy of Sciences)
EPI_ISL_224729	A/Bar-headed Goose/Qinghai/BTY13-B/2016	A / H5N8	Asia / China / Qinghai Province	Jianjun Chen (Wuhan Institute of Virology / Chinese Academy of Sciences)
EPI_ISL_224730	A/Bar-headed Goose/Qinghai/BTY13-LU/2016	A / H5N8	Asia / China / Qinghai Province	Jianjun Chen (Wuhan Institute of Virology / Chinese Academy of Sciences)
EPI_ISL_224731	A/Bar-headed Goose/Qinghai/BTY14-B/2016	A / H5N8	Asia / China / Qinghai Province	Jianjun Chen (Wuhan Institute of Virology / Chinese Academy of Sciences)
EPI_ISL_224732	A/Bar-headed Goose/Qinghai/BTY14-LU/2016	A / H5N8	Asia / China / Qinghai Province	Jianjun Chen (Wuhan Institute of Virology / Chinese Academy of Sciences)
EPI_ISL_224733	A/Bar-headed Goose/Qinghai/BTY15-B/2016	A / H5N8	Asia / China / Qinghai Province	Jianjun Chen (Wuhan Institute of Virology / Chinese Academy of Sciences)
EPI_ISL_224734	A/Bar-headed Goose/Qinghai/BTY15-LU/2016	A / H5N8	Asia / China / Qinghai Province	Jianjun Chen (Wuhan Institute of Virology / Chinese Academy of Sciences)
EPI_ISL_224735	A/Bar-headed Goose/Qinghai/BTY16-B/2016	A / H5N8	Asia / China / Qinghai Province	Jianjun Chen (Wuhan Institute of Virology / Chinese Academy of Sciences)
EPI_ISL_224736	A/Bar-headed Goose/Qinghai/BTY16-LU/2016	A / H5N8	Asia / China / Qinghai Province	Jianjun Chen (Wuhan Institute of Virology / Chinese Academy of Sciences)
EPI_ISL_224737	A/Bar-headed Goose/Qinghai/BTY17-B/2016	A / H5N8	Asia / China / Qinghai Province	Jianjun Chen (Wuhan Institute of Virology / Chinese Academy of Sciences)
EPI_ISL_224738	A/Bar-headed Goose/Qinghai/BTY17-LU/2016	A / H5N8	Asia / China / Qinghai Province	Jianjun Chen (Wuhan Institute of Virology / Chinese Academy of Sciences)
EPI_ISL_224739	A/Bar-headed Goose/Qinghai/BTY18-B/2016	A / H5N8	Asia / China / Qinghai Province	Jianjun Chen (Wuhan Institute of Virology / Chinese Academy of Sciences)
EPI_ISL_224740	A/Bar-headed Goose/Qinghai/BTY18-LU/2016	A / H5N8	Asia / China / Qinghai Province	Jianjun Chen (Wuhan Institute of Virology / Chinese Academy of Sciences)
EPI_ISL_224742	A/Brown-headed Gull/Qinghai/ZTO1-B/2016	A / H5N8	Asia / China / Qinghai Province	Jianjun Chen (Wuhan Institute of Virology / Chinese Academy of Sciences)
EPI_ISL_224743	A/Brown-headed Gull/Qinghai/ZTO1-LU/2016	A / H5N8	Asia / China / Qinghai Province	Jianjun Chen (Wuhan Institute of Virology / Chinese Academy of Sciences)
EPI_ISL_224744	A/Brown-headed Gull/Qinghai/ZTO3-B/2016	A / H5N8	Asia / China / Qinghai Province	Jianjun Chen (Wuhan Institute of Virology / Chinese Academy of Sciences)
EPI_ISL_224745	A/Brown-headed Gull/Qinghai/ZTO3-LU/2016	A / H5N8	Asia / China / Qinghai Province	Jianjun Chen (Wuhan Institute of Virology / Chinese Academy of Sciences)
EPI_ISL_224746	A/Brown-headed Gull/Qinghai/ZTO4-B/2016	A / H5N8	Asia / China / Qinghai Province	Jianjun Chen (Wuhan Institute of Virology / Chinese Academy of Sciences)
EPI_ISL_224747	A/Brown-headed Gull/Qinghai/ZTO5-B/2016	A / H5N8	Asia / China / Qinghai Province	Jianjun Chen (Wuhan Institute of Virology / Chinese Academy of Sciences)
EPI_ISL_224748	A/Brown-headed Gull/Qinghai/ZTO5-K/2016	A / H5N8	Asia / China / Qinghai Province	Jianjun Chen (Wuhan Institute of Virology / Chinese Academy of Sciences)
EPI_ISL_224749	A/Brown-headed Gull/Qinghai/ZTO6-B/2016	A / H5N8	Asia / China / Qinghai Province	Jianjun Chen (Wuhan Institute of Virology / Chinese Academy of Sciences)
EPI_ISL_224750	A/Brown-headed Gull/Qinghai/ZTO6-SP/2016	A / H5N8	Asia / China / Qinghai Province	Jianjun Chen (Wuhan Institute of Virology / Chinese Academy of Sciences)
EPI_ISL_224751	A/Brown-headed Gull/Qinghai/ZTO6-MU/2016	A / H5N8	Asia / China / Qinghai Province	Jianjun Chen (Wuhan Institute of Virology / Chinese Academy of Sciences)
EPI_ISL_224752	A/Great Black-headed Gull/Qinghai/YO1-B/2016	A / H5N8	Asia / China / Qinghai Province	Jianjun Chen (Wuhan Institute of Virology / Chinese Academy of Sciences)
EPI_ISL_224879	A/duck/Zhejiang/6DK19/2013	A / H5N2	Asia / China	
EPI_ISL_224984	A/duck/Guangzhou/018/2014	A / H5N6	Asia / China	
EPI_ISL_224985	A/duck/Guangzhou/021/2014	A / H5N6	Asia / China	

EPI_ISL_2254497	A/western jackdaw/SVA210317SZ0321/KN001057/E-2021	A / H5N8	Europe / Sweden / Ostergotlands Lan / Mjolby Kommun	Siamak Zohari (National Veterinary Institute)
EPI_ISL_225570	A/turkey/South Dakota/15-012511-2/2015	A / H5N2	North America / United States	
EPI_ISL_225571	A/turkey/Minnesota/15-012582-1/2015	A / H5N2	North America / United States	
EPI_ISL_225572	A/chicken/Iowa/15-013388-1/2015	A / H5N2	North America / United States	
EPI_ISL_2276068	A/chicken/Senegal/21VIR1084-3/2021	A / H5N1	Africa / Senegal / Region de Thies	Bianca Zecchin (Istituto Zooprofilattico Sperimentale Delle Venezie)
EPI_ISL_2276069	A/chicken/Senegal/21VIR1084-4/2021	A / H5N1	Africa / Senegal / Region de Thies	Bianca Zecchin (Istituto Zooprofilattico Sperimentale Delle Venezie)
EPI_ISL_2276070	A/chicken/Senegal/21VIR1084-5/2021	A / H5N1	Africa / Senegal / Region de Thies	Bianca Zecchin (Istituto Zooprofilattico Sperimentale Delle Venezie)
EPI_ISL_2276071	A/great-white_pelican/Senegal/21-67_21VIR1084-8/2021	A / H5N1	Africa / Senegal / Djoudj bird sanctuary	Bianca Zecchin (Istituto Zooprofilattico Sperimentale Delle Venezie)
EPI_ISL_229213	A/duck/Vietnam/LBM808/2015	A / H5N6	Asia / Vietnam	
EPI_ISL_2301187	A/common buzzard/Sweden/SVA210323SZ0426/KN001120/O-	A / H5N8	Europe / Sweden / Vastra Gotalands Lan / Lidkopings Kommun	Siamak Zohari (National Veterinary Institute)
EPI_ISL_230158	A/muscovy duck/LBM803/2015	A / H5N6	Asia / Vietnam	
EPI_ISL_230754	A/muscovy duck/LBM806/2015	A / H5N6	Asia / Vietnam	
EPI_ISL_230755	A/muscovy duck/Vietnam/LBM809/2015	A / H5N6	Asia / Vietnam	
EPI_ISL_230756	A/muscovy duck/Vietnam/LBM810/2015	A / H5N6	Asia / Vietnam	
EPI_ISL_230757	A/muscovy duck/Vietnam/LBM817/2015	A / H5N6	Asia / Vietnam	
EPI_ISL_230758	A/muscovy duck/Vietnam/LBM818/2015	A / H5N6	Asia / Vietnam	
EPI_ISL_230759	A/muscovy duck/Vietnam/LBM823/2015	A / H5N6	Asia / Vietnam	
EPI_ISL_230760	A/duck/Vietnam/LBM812/2015	A / H5N6	Asia / Vietnam	
EPI_ISL_230820	A/great crested grebe/Tyva/34/2016	A / H5N8	Europe / Russian Federation / Tyva Republic	Artem Fadeev (WHO National Influenza Centre Russian Federation)
EPI_ISL_231525	A/duck/Vietnam/LBM813/2015	A / H5N6	Asia / Vietnam	
EPI_ISL_231526	A/duck/Vietnam/LBM816/2015	A / H5N6	Asia / Vietnam	
EPI_ISL_231527	A/duck/Vietnam/LBM824/2015	A / H5N6	Asia / Vietnam	
EPI_ISL_231684	A/wild duck/Tyva/35/2016	A / H5N8	Europe / Russian Federation / Tyva Republic	Artem Fadeev (WHO National Influenza Centre Russian Federation)
EPI_ISL_231685	A/black-headed gull/Tyva/41/2016	A / H5N8	Europe / Russian Federation / Tyva Republic	Artem Fadeev (WHO National Influenza Centre Russian Federation)
EPI_ISL_231747	A/duck/Hubei/SZY250/2016	A / H5N2	Asia / China	
EPI_ISL_234057	A/grey heron /Uvs-Nuur Lake/20/2016	A / H5N8	Europe / Russian Federation / Tyva Republic	Ivan Sobolev (Research Institute of Experimental and Clinical Medicine)
EPI_ISL_234058	A/common tern /Uvs-Nuur Lake/26/2016	A / H5N8	Europe / Russian Federation / Tyva Republic	Ivan Sobolev (Research Institute of Experimental and Clinical Medicine)
EPI_ISL_234282	A/chicken/Hubei/XG18/2015	A / H5N6	Asia / China	
EPI_ISL_234283	A/duck/Hubei/XG18/2015	A / H5N6	Asia / China	
EPI_ISL_234284	A/duck/Hubei/WH18/2015	A / H5N6	Asia / China	
EPI_ISL_234329	A/breeder duck/Korea/H2086/2015	A / H5N8	Asia / Korea, Republic of / Jeollanam-do	Eun-Kyoung Lee (Animal and Plant Quarantine Agency (APQA) / Avian Influenza Research and Diagsnotic Division)
EPI_ISL_234330	A/mallard/Korea/H2102/2015	A / H5N8	Asia / Korea, Republic of / Chungcheongnam-do	Eun-Kyoung Lee (Animal and Plant Quarantine Agency (APQA) / Avian Influenza Research and Diagsnotic Division)
EPI_ISL_234331	A/broiler duck/Korea/H2116/2015	A / H5N8	Asia / Korea, Republic of / Jeollabuk-do	Eun-Kyoung Lee (Animal and Plant Quarantine Agency (APQA) / Avian Influenza Research and Diagsnotic Division)
EPI_ISL_234332	A/broiler duck/Korea/H2194/2015	A / H5N8	Asia / Korea, Republic of / Chungcheongnam-do	Eun-Kyoung Lee (Animal and Plant Quarantine Agency (APQA) / Avian Influenza Research and Diagsnotic Division)
EPI_ISL_234333	A/broiler duck/Korea/H2266/2015	A / H5N8	Asia / Korea, Republic of / Chungcheongnam-do	Eun-Kyoung Lee (Animal and Plant Quarantine Agency (APQA) / Avian Influenza Research and Diagsnotic Division)
EPI_ISL_234334	A/broiler duck/Korea/H2278/2015	A / H5N8	Asia / Korea, Republic of / Jeollanam-do	Eun-Kyoung Lee (Animal and Plant Quarantine Agency (APQA) / Avian Influenza Research and Diagsnotic Division)
EPI_ISL_234335	A/wild bird/Korea/H2290/2015	A / H5N8	Asia / Korea, Republic of / Chungcheongnam-do	Eun-Kyoung Lee (Animal and Plant Quarantine Agency (APQA) / Avian Influenza Research and Diagsnotic Division)
EPI_ISL_234336	A/wild bird/Korea/H2291/2015	A / H5N8	Asia / Korea, Republic of / Jeju-teukbyeoljachido	Eun-Kyoung Lee (Animal and Plant Quarantine Agency (APQA) / Avian Influenza Research and Diagsnotic Division)
EPI_ISL_234337	A/mallard/Korea/H2304/2015	A / H5N8	Asia / Korea, Republic of / Chungcheongnam-do	Eun-Kyoung Lee (Animal and Plant Quarantine Agency (APQA) / Avian Influenza Research and Diagsnotic Division)
EPI_ISL_234338	A/mallard/Korea/H2321/2015	A / H5N8	Asia / Korea, Republic of / Seoul	Eun-Kyoung Lee (Animal and Plant Quarantine Agency (APQA) / Avian Influenza Research and Diagsnotic Division)
EPI_ISL_234339	A/broiler duck/Korea/H2385/2015	A / H5N8	Asia / Korea, Republic of / Jeollanam-do	Eun-Kyoung Lee (Animal and Plant Quarantine Agency (APQA) / Avian Influenza Research and Diagsnotic Division)
EPI_ISL_234340	A/broiler duck/Korea/H2393/2015	A / H5N8	Asia / Korea, Republic of / Chungcheongbuk-do	Eun-Kyoung Lee (Animal and Plant Quarantine Agency (APQA) / Avian Influenza Research and Diagsnotic Division)
EPI_ISL_234341	A/broiler duck/Korea/H2400/2015	A / H5N8	Asia / Korea, Republic of / Chungcheongbuk-do	Eun-Kyoung Lee (Animal and Plant Quarantine Agency (APQA) / Avian Influenza Research and Diagsnotic Division)
EPI_ISL_234342	A/broiler duck/Korea/H2405/2015	A / H5N8	Asia / Korea, Republic of / Chungcheongbuk-do	Eun-Kyoung Lee (Animal and Plant Quarantine Agency (APQA) / Avian Influenza Research and Diagsnotic Division)
EPI_ISL_234343	A/breeder chicken/Korea/H2496/2015	A / H5N8	Asia / Korea, Republic of / Chungcheongbuk-do	Eun-Kyoung Lee (Animal and Plant Quarantine Agency (APQA) / Avian Influenza Research and Diagsnotic Division)
EPI_ISL_234344	A/broiler duck/Korea/H2517/2015	A / H5N8	Asia / Korea, Republic of / Chungcheongbuk-do	Eun-Kyoung Lee (Animal and Plant Quarantine Agency (APQA) / Avian Influenza Research and Diagsnotic Division)
EPI_ISL_234345	A/broiler duck/Korea/H2531/2015	A / H5N8	Asia / Korea, Republic of / Jeollabuk-do	Eun-Kyoung Lee (Animal and Plant Quarantine Agency (APQA) / Avian Influenza Research and Diagsnotic Division)
EPI_ISL_234346	A/chicken/Korea/H2553/2015	A / H5N8	Asia / Korea, Republic of / Jeollabuk-do	Eun-Kyoung Lee (Animal and Plant Quarantine Agency (APQA) / Avian Influenza Research and Diagsnotic Division)
EPI_ISL_234347	A/chicken/Korea/H2565/2015	A / H5N8	Asia / Korea, Republic of / Jeollabuk-do	Eun-Kyoung Lee (Animal and Plant Quarantine Agency (APQA) / Avian Influenza Research and Diagsnotic Division)
EPI_ISL_234348	A/chicken/Korea/H2590/2015	A / H5N8	Asia / Korea, Republic of / Gyeonggi-do	Eun-Kyoung Lee (Animal and Plant Quarantine Agency (APQA) / Avian Influenza Research and Diagsnotic Division)
EPI_ISL_234349	A/korean native chicken/Korea/H2598/2015	A / H5N8	Asia / Korea, Republic of / Gyeonggi-do	Eun-Kyoung Lee (Animal and Plant Quarantine Agency (APQA) / Avian Influenza Research and Diagsnotic Division)
EPI_ISL_234350	A/broiler duck/Korea/H2606/2015	A / H5N8	Asia / Korea, Republic of / Jeollabuk-do	Eun-Kyoung Lee (Animal and Plant Quarantine Agency (APQA) / Avian Influenza Research and Diagsnotic Division)
EPI_ISL_234351	A/broiler duck/Korea/H2618/2015	A / H5N8	Asia / Korea, Republic of / Jeollanam-do	Eun-Kyoung Lee (Animal and Plant Quarantine Agency (APQA) / Avian Influenza Research and Diagsnotic Division)
EPI_ISL_234352	A/duck/Korea/H2627/2015	A / H5N8	Asia / Korea, Republic of / Gyeonggi-do	Eun-Kyoung Lee (Animal and Plant Quarantine Agency (APQA) / Avian Influenza Research and Diagsnotic Division)
EPI_ISL_234353	A/duck/Korea/H2628/2015	A / H5N8	Asia / Korea, Republic of	Eun-Kyoung Lee (Animal and Plant Quarantine Agency (APQA) / Avian Influenza Research and Diagsnotic Division)
EPI_ISL_234354	A/broildr duck/Korea/H2637/2015	A / H5N8	Asia / Korea, Republic of / Jeollanam-do	Eun-Kyoung Lee (Animal and Plant Quarantine Agency (APQA) / Avian Influenza Research and Diagsnotic Division)
EPI_ISL_234355	A/broiler duck/Korea/H2641/2015	A / H5N8	Asia / Korea, Republic of / Jeollanam-do	Eun-Kyoung Lee (Animal and Plant Quarantine Agency (APQA) / Avian Influenza Research and Diagsnotic Division)
EPI_ISL_234356	A/broiler duck/Korea/H2649/2015	A / H5N8	Asia / Korea, Republic of / Jeollanam-do	Eun-Kyoung Lee (Animal and Plant Quarantine Agency (APQA) / Avian Influenza Research and Diagsnotic Division)
EPI_ISL_234357	A/breeder duck/Korea/H2675/2015	A / H5N8	Asia / Korea, Republic of / Jeollanam-do	Eun-Kyoung Lee (Animal and Plant Quarantine Agency (APQA) / Avian Influenza Research and Diagsnotic Division)
EPI_ISL_234358	A/broiler duck/Korea/H2826/2015	A / H5N8	Asia / Korea, Republic of / Jeollanam-do	Eun-Kyoung Lee (Animal and Plant Quarantine Agency (APQA) / Avian Influenza Research and Diagsnotic Division)
EPI_ISL_234359	A/broiler duck/Korea/H2827/2015	A / H5N8	Asia / Korea, Republic of / Jeollanam-do	Eun-Kyoung Lee (Animal and Plant Quarantine Agency (APQA) / Avian Influenza Research and Diagsnotic Division)
EPI_ISL_234360	A/broiler duck/Korea/LBM334/2015	A / H5N8	Asia / Korea, Republic of / Jeollanam-do	Eun-Kyoung Lee (Animal and Plant Quarantine Agency (APQA) / Avian Influenza Research and Diagsnotic Division)
EPI_ISL_234361	A/mallard/Korea/H2891/2015	A / H5N8	Asia / Korea, Republic of / Jeollanam-do	Eun-Kyoung Lee (Animal and Plant Quarantine Agency (APQA) / Avian Influenza Research and Diagsnotic Division)
EPI_ISL_234362	A/broiler duck/Korea/H2910/2015	A / H5N8	Asia / Korea, Republic of / Jeollanam-do	Eun-Kyoung Lee (Animal and Plant Quarantine Agency (APQA) / Avian Influenza Research and Diagsnotic Division)
EPI_ISL_234363	A/broiler duck/Korea/H3078/2015	A / H5N8	Asia / Korea, Republic of / Jeollanam-do	Eun-Kyoung Lee (Animal and Plant Quarantine Agency (APQA) / Avian Influenza Research and Diagsnotic Division)
EPI_ISL_234364	A/broiler duck/Korea/H3092/2015	A / H5N8	Asia / Korea, Republic of / Jeollanam-do	Eun-Kyoung Lee (Animal and Plant Quarantine Agency (APQA) / Avian Influenza Research and Diagsnotic Division)
EPI_ISL_234365	A/broiler duck/Korea/H3093/2015	A / H5N8	Asia / Korea, Republic of / Jeollanam-do	Eun-Kyoung Lee (Animal and Plant Quarantine Agency (APQA) / Avian Influenza Research and Diagsnotic Division)
EPI_ISL_234366	A/broiler duck/Korea/H3098/2015	A / H5N8	Asia / Korea, Republic of / Jeollanam-do	Eun-Kyoung Lee (Animal and Plant Quarantine Agency (APQA) / Avian Influenza Research and Diagsnotic Division)
EPI_ISL_234367	A/broiler duck/Korea/H3104/2015	A / H5N8	Asia / Korea, Republic of / Jeollanam-do	Eun-Kyoung Lee (Animal and Plant Quarantine Agency (APQA) / Avian Influenza Research and Diagsnotic Division)
EPI_ISL_234368	A/broiler duck/Korea/H3298/2015	A / H5N8	Asia / Korea, Republic of / Jeollanam-do	Eun-Kyoung Lee (Animal and Plant Quarantine Agency (APQA) / Avian Influenza Research and Diagsnotic Division)
EPI_ISL_234369	A/broiler duck/Korea/H3301/2015	A / H5N8	Asia / Korea, Republic of / Jeollanam-do	Eun-Kyoung Lee (Animal and Plant Quarantine Agency (APQA) / Avian Influenza Research and Diagsnotic Division)
EPI_ISL_234370	A/broiler duck/Korea/H3302/2015	A / H5N8	Asia / Korea, Republic of / Jeollanam-do	Eun-Kyoung Lee (Animal and Plant Quarantine Agency (APQA) / Avian Influenza Research and Diagsnotic Division)

EPI_ISL_234371	A/breeder duck/Korea/16AQ17/2016	A / H5N8	Asia / Korea, Republic of / Gyeonggi-do	Eun-Kyoung Lee (Animal and Plant Quarantine Agency (APQA) / Avian Influenza Research and Diagsnotic Division)
EPI_ISL_234372	A/duck/Korea/16A02/2016	A / H5N8	Asia / Korea, Republic of / Gyeonggi-do	Eun-Kyoung Lee (Animal and Plant Quarantine Agency (APQA) / Avian Influenza Research and Diagsnotic Division)
EPI_ISL_234376	A/migratory waterfowl/Hubei/Chenhu1306/2014	A / H5N6	Asia / China	
EPI_ISL_234377	A/migratory waterfowl/Hubei/Chenhu1347/2014	A / H5N6	Asia / China	
EPI_ISL_234670	A/Copsychus saularis/Guangdong/SW8/2014	A / H5N6	Asia / China	
EPI_ISL_234671	A/Gallinula chloropus/Guangdong/GZ174/2014	A / H5N6	Asia / China	
EPI_ISL_234679	A/Syrrhaptes paradoxus/Guangdong/ZH283/2015	A / H5N6	Asia / China	
EPI_ISL_235199	A/duck/Zhejiang/6DK19-MA/2013	A / H5N2	Asia / China / Zhejiang Province	
EPI_ISL_235740	A/mallard/Alaska/AH0088535/2016	A / H5N2	North America / United States	
EPI_ISL_235771	A/mandarin duck/Gifu/2112D001/2014	A / H5N8	Asia / Japan / Gifu	
EPI_ISL_235772	A/tundra swan/Tottori/C6nk/2014	A / H5N8	Asia / Japan / Tottori	
EPI_ISL_236802	A/chicken/BC/FAV2/2015	A / H5N1	North America / Canada	
EPI_ISL_237551	A/goose/Shandong/LQ01694/2013	A / H5N8	Asia / China / Shandong Province	Yuhai Bi (Institute of Microbiology, Chinese Academy of Sciences)
EPI_ISL_237552	A/chicken/Shandong/KD02699/2013	A / H5N8	Asia / China / Shandong Province	Yuhai Bi (Institute of Microbiology, Chinese Academy of Sciences)
EPI_ISL_237553	A/duck/India/10CA01/2016	A / H5N8	Asia / India / National Capital Territory of Delhi	Shanmuga sundaram Nagarajan (ICAR-National Institute of High Security Animal Diseases / Indian Council of Agricultural Research)
EPI_ISL_237554	A/painted stork/India/10CA03/2016	A / H5N8	Asia / India / Madhya Pradesh / Gwalior	Shanmuga sundaram Nagarajan (ICAR-National Institute of High Security Animal Diseases / Indian Council of Agricultural Research)
EPI_ISL_237730	A/mute_swan/Hungary/51049/2016	A / H5N8	Europe / Hungary / Csongrad megye	Adam Dan (Danam.Vet.Molbiol)
EPI_ISL_237731	A/domestic_turkey/Hungary/53433/2016	A / H5N8	Europe / Hungary / Bekes megye	Adam Dan (Danam.Vet.Molbiol)
EPI_ISL_237732	A/tufted_duck/Germany-SH/R8446/2016	A / H5N8	Europe / Germany / Schleswig-Holstein / Lake Plön	Anne Pohlmann (Friedrich-Loeffler-Institut)
EPI_ISL_237733	A/tufted_duck/Germany/AR8444-L01986/2016	A / H5N8	Europe / Germany / Schleswig-Holstein / Lake Plön	Anne Pohlmann (Friedrich-Loeffler-Institut)
EPI_ISL_237792	A/wild duck/Germany-BW/R8455/2016	A / H5N8	Europe / Germany / Baden-Wuerttemberg	Elke Starick (Friedrich-Loeffler-Institut)
EPI_ISL_237921	A/wild duck/Poland/82A/2016	A / H5N8	Europe / Poland / West Pomeranian Voivodeship	Edyta ?wi?to? (National Veterinary Research Institut Poland, PIWet-PIB)
EPI_ISL_237922	A/tufted duck/Denmark/17740-1/2016	A / H5N8	Europe / Denmark / Region Hovedstaden / Kobenhavns Kommune / Stadsgraven, Christiania	Charlotte Kristiane Hjulsager (Statens Serum Institute / Microbiological Diagnostic and Virology)
EPI_ISL_237944	A/tufted_duck/Germany/AR8444-L01987/2016	A / H5N8	Europe / Germany / Schleswig-Holstein / Lake Plön	Anne Pohlmann (Friedrich-Loeffler-Institut)
EPI_ISL_237945	A/tufted_duck/Germany/AR8459-L01988/2016	A / H5N8	Europe / Germany / Baden-Wuerttemberg / Lake Constance	Anne Pohlmann (Friedrich-Loeffler-Institut)
EPI_ISL_237947	A/duck/Japan/AnimalQuarantine-HE72/2015	A / H5N6	Asia / Japan	Takehiko Saito (National Institute of Animal Health)
EPI_ISL_237952	A/chicken/Japan/AnimalQuarantine-HE144/2016	A / H5N6	Asia / Japan	Takehiko Saito (National Institute of Animal Health)
EPI_ISL_237958	A/tufted duck/Germany-SH/R8444/2016	A / H5N8	Europe / Germany / Schleswig-Holstein	Elke Starick (Friedrich-Loeffler-Institut)
EPI_ISL_237964	A/Mulard_duck/Hungary/54494/2016	A / H5N8	Europe / Hungary / Bacs-Kiskun / Kiskunmajsa	Adam Dan (Danam.Vet.Molbiol)
EPI_ISL_237965	A/goose/Hungary/55128/2016	A / H5N8	Europe / Hungary / Bacs-Kiskun / Bugac	Adam Dan (Danam.Vet.Molbiol)
EPI_ISL_237966	A/duck/Hungary/55191/2016	A / H5N8	Europe / Hungary / Bacs-Kiskun / Kisszállás	Adam Dan (Danam.Vet.Molbiol)
EPI_ISL_237987	A/gyrfalcon/Washington/41088/6/2014	A / H5N8	North America / United States	Takehiko Saito (National Institute of Animal Health)
EPI_ISL_238037	A/turkey/Germany-SH/R8595/2016	A / H5N8	Europe / Germany / Schleswig-Holstein	Elke Starick (Friedrich-Loeffler-Institut)
EPI_ISL_238038	A/chicken/Germany-MV/R8790/2016	A / H5N8	Europe / Germany / Mecklenburg-Vorpommern	Elke Starick (Friedrich-Loeffler-Institut)
EPI_ISL_238039	A/chicken/Germany-SH/R8758/2016	A / H5N8	Europe / Germany / Schleswig-Holstein	Elke Starick (Friedrich-Loeffler-Institut)
EPI_ISL_238040	A/tufted duck/Germany-SN/R8795/2016	A / H5N8	Europe / Germany / Saxony	Elke Starick (Friedrich-Loeffler-Institut)
EPI_ISL_238148	A/Mandarin_duck/Korea/K16-187-3/2016	A / H5N6	Asia / Korea, Republic of / Chungcheongbuk-do / Gokgyo-cheon (36°45'12.3"N 127°07'12.7"E)	Jung Hoon Kwon (Konkuk University / College of Veterinary Medicine)
EPI_ISL_238196	A/mute swan/Croatia/70/2016	A / H5N8	Europe / Croatia / Vukovarsko-Srijemska Zupanija / Cerna, river Bid	Vladimir Savi? (Croatian Veterinary Institute / Poultry Centre)
EPI_ISL_238197	A/mute swan/Croatia/78/2016	A / H5N8	Europe / Croatia / Osjecko-Baranjska Zupanija / Bijelo Brdo	Vladimir Savi? (Croatian Veterinary Institute / Poultry Centre)
EPI_ISL_238605	A/environment/Kagoshima/KU-ngr-I/2016(H5N6)	A / H5N6	Asia / Japan / Kagoshima	Makoto Ozawa (Kagoshima University)
EPI_ISL_238893	A/Chicken/Sweden/SVA161122KU0453/SZ0209316/2016	A / H5N8	Europe / Sweden / Skane Lan / Helsingborgs Kommun / City of Mörarp, Lat. 56.053495 Long. 12	Siamak Zohari (National Veterinary Institute)
EPI_ISL_238894	A/Chicken/Sweden/SVA161122KU0453/SZ0209317/2016	A / H5N8	Europe / Sweden / Skane Lan / Helsingborgs Kommun / City of Mörarp, Lat. 56.053495 Long. 12.	Siamak Zohari (National Veterinary Institute)
EPI_ISL_238895	A/Chicken/Sweden/SVA161122KU0453/SZ0209318/2016	A / H5N8	Europe / Sweden / Skane Lan / Helsingborgs Kommun / City of Mörarp, Lat. 56.053495 Long. 12.	Siamak Zohari (National Veterinary Institute)
EPI_ISL_238896	A/Chicken/Sweden/SVA161122KU0453/SZ0209321/2016	A / H5N8	Europe / Sweden / Skane Lan / Helsingborgs Kommun / City of Mörarp, Lat. 56.053495 Long. 12.	Siamak Zohari (National Veterinary Institute)
EPI_ISL_238897	A/Common Goldeneye/Sweden/SVA161117KU0322/SZ000216	A / H5N8	Europe / Sweden / Skane Lan / Vellinge Kommun / Vellinge, Höllviken	Siamak Zohari (National Veterinary Institute)
EPI_ISL_239069	A/mute swan/Croatia/85/2016	A / H5N8	Europe / Croatia / Osjecko-Baranjska Zupanija / Bijelo Brdo	Vladimir Savi? (Croatian Veterinary Institute / Poultry Centre)
EPI_ISL_239260	A/chicken/Korea/H23/2016(H5N6)	A / H5N6	Asia / Korea, Republic of / Gyeonggi-do	Youn-Jeong Lee (Animal and Plant Quarantine Agency (APQA) / Avian Influenza Research and Diagsnotic Division)
EPI_ISL_239261	A/chicken/Korea/HN1/2016(H5N6)	A / H5N6	Asia / Korea, Republic of / Jeollanam-do	Youn-Jeong Lee (Animal and Plant Quarantine Agency (APQA) / Avian Influenza Research and Diagsnotic Division)
EPI_ISL_239262	A/duck/Korea/ES2/2016(H5N6)	A / H5N6	Asia / Korea, Republic of / Chungcheongbuk-do	Youn-Jeong Lee (Animal and Plant Quarantine Agency (APQA) / Avian Influenza Research and Diagsnotic Division)
EPI_ISL_239263	A/duck/Korea/H15/2016(H5N6)	A / H5N6	Asia / Korea, Republic of / Chungcheongbuk-do	Youn-Jeong Lee (Animal and Plant Quarantine Agency (APQA) / Avian Influenza Research and Diagsnotic Division)
EPI_ISL_239266	A/Eurasian eagle owl/Korea/960/2016(H5N6)	A / H5N6	Asia / Korea, Republic of / Gangwon-do	Youn-Jeong Lee (Animal and Plant Quarantine Agency (APQA) / Avian Influenza Research and Diagsnotic Division)
EPI_ISL_239267	A/mandarin duck/Korea/T102-1/2016(H5N6)	A / H5N6	Asia / Korea, Republic of / Chungcheongnam-do	Youn-Jeong Lee (Animal and Plant Quarantine Agency (APQA) / Avian Influenza Research and Diagsnotic Division)
EPI_ISL_239269	A/mandarin duck/Korea/WB246/2016(H5N6)	A / H5N6	Asia / Korea, Republic of / Chungcheongnam-do	Youn-Jeong Lee (Animal and Plant Quarantine Agency (APQA) / Avian Influenza Research and Diagsnotic Division)
EPI_ISL_239271	A/spot_billed_duck/Korea/WB141/2016(H5N6)	A / H5N6	Asia / Korea, Republic of / Jeollabuk-do	Youn-Jeong Lee (Animal and Plant Quarantine Agency (APQA) / Avian Influenza Research and Diagsnotic Division)
EPI_ISL_239351	A/duck/Hyogo/1/2016	A / H5N6	Asia / Japan / Hyogo	Ikuyo Takayama (National Institute of Infectious Diseases (NIID))
EPI_ISL_239419	A/chicken/Hungary/59048/2016	A / H5N8	Europe / Hungary / Jasz-Nagykun-Szolnok	Adam Dan (Danam.Vet.Molbiol)
EPI_ISL_239420	A/duck/Hungary/60441/2016	A / H5N8	Europe / Hungary / Bacs-Kiskun	Adam Dan (Danam.Vet.Molbiol)
EPI_ISL_239421	A/muscovy duck/Aomori/1-2T/2016	A / H5N6	Asia / Japan	Takehiko Saito (National Institute of Animal Health)
EPI_ISL_239422	A/muscovy duck/Aomori/2-4T/2016	A / H5N6	Asia / Japan	Takehiko Saito (National Institute of Animal Health)
EPI_ISL_239423	A/chicken/Niigata/2-7T/2016	A / H5N6	Asia / Japan	Takehiko Saito (National Institute of Animal Health)
EPI_ISL_239424	A/muscovy duck/Aomori/1-10C/2016	A / H5N6	Asia / Japan	Takehiko Saito (National Institute of Animal Health)
EPI_ISL_239425	A/chicken/Niigata/2-6T/2016	A / H5N6	Asia / Japan	Takehiko Saito (National Institute of Animal Health)
EPI_ISL_239426	A/chicken/Niigata/2-8T/2016	A / H5N6	Asia / Japan	Takehiko Saito (National Institute of Animal Health)
EPI_ISL_239427	A/chicken/Niigata/2-5C/2016	A / H5N6	Asia / Japan	Takehiko Saito (National Institute of Animal Health)
EPI_ISL_239428	A/muscovy duck/Aomori/2-2T/2016	A / H5N6	Asia / Japan	Takehiko Saito (National Institute of Animal Health)
EPI_ISL_239429	A/muscovy duck/Aomori/1-7T/2016	A / H5N6	Asia / Japan	Takehiko Saito (National Institute of Animal Health)
EPI_ISL_239430	A/muscovy duck/Aomori/2-1C/2016	A / H5N6	Asia / Japan	Takehiko Saito (National Institute of Animal Health)
EPI_ISL_239431	A/muscovy duck/Aomori/2-3T/2016	A / H5N6	Asia / Japan	Takehiko Saito (National Institute of Animal Health)
EPI_ISL_239433	A/muscovy duck/Aomori/1-3T/2016	A / H5N6	Asia / Japan	Takehiko Saito (National Institute of Animal Health)
EPI_ISL_239434	A/tufted_duck/Switzerland/V246-L02001/2016	A / H5N8	Europe / Switzerland / Neuenburgersee	Anne Pohlmann (Friedrich-Loeffler-Institut)
EPI_ISL_239435	A/tufted_duck/Switzerland/V254-L02002/2016	A / H5N8	Europe / Switzerland / Lake Constance	Anne Pohlmann (Friedrich-Loeffler-Institut)
EPI_ISL_239436	A/tufted_duck/Switzerland/V237-L02003/2016	A / H5N8	Europe / Switzerland / Bielersee	Anne Pohlmann (Friedrich-Loeffler-Institut)

EPI_ISL_239437	A/caspian_gull/Switzerland/V247-L02004/2016	A / H5N8	Europe / Switzerland / Neuenburgersee	Anne Pohlmann (Friedrich-Loeffler-Institut)
EPI_ISL_239573	A/crane/Kagoshima/KU-4/2016(H5N6)	A / H5N6	Asia / Japan / Kagoshima	Makoto Ozawa (Kagoshima University)
EPI_ISL_239801	A/turkey/England/052131/2016	A / H5N8	Europe / United Kingdom / Lincolnshire / Louth	James Seekings (Animal and Plant Health Agency (APHA) / Virology Department)
EPI_ISL_239802	A/Common-coot/Egypt/CA285/2016	A / H5N8	Africa / Egypt / Damietta	Mahmoud Mohamed Naguib (Friedrich-Loeffler-Institut)
EPI_ISL_239997	A/decoy_duck/France/161104e/2016	A / H5N8	Europe / France / Region Nord-Pas-De Calais / Departement du Pas-de-Calais	Francois-Xavier Briand (ANSES Agence Nationale De Securite Sanitaire De L'alimentation / Laboratoire de Ploufragan-Plouzané)
EPI_ISL_239999	A/black headed gull/Ibaraki/235T/2016	A / H5N6	Asia / Japan	Takehiko Saito (National Institute of Animal Health)
EPI_ISL_240000	A/black headed gull/Ibaraki/253T/2016	A / H5N6	Asia / Japan	Takehiko Saito (National Institute of Animal Health)
EPI_ISL_240001	A/mute swan/Ibaraki/239C/2016	A / H5N6	Asia / Japan	Takehiko Saito (National Institute of Animal Health)
EPI_ISL_240002	A/mute swan/Ibaraki/236T/2016	A / H5N6	Asia / Japan	Takehiko Saito (National Institute of Animal Health)
EPI_ISL_240003	A/mute swan/Ibaraki/250T/2016	A / H5N6	Asia / Japan	Takehiko Saito (National Institute of Animal Health)
EPI_ISL_240004	A/mute swan/Ibaraki/245C/2016	A / H5N6	Asia / Japan	Takehiko Saito (National Institute of Animal Health)
EPI_ISL_240005	A/mute swan/Ibaraki/232C/2016	A / H5N6	Asia / Japan	Takehiko Saito (National Institute of Animal Health)
EPI_ISL_240006	A/mute swan/Ibaraki/247C/2016	A / H5N6	Asia / Japan	Takehiko Saito (National Institute of Animal Health)
EPI_ISL_240007	A/mute swan/Ibaraki/240C/2016	A / H5N6	Asia / Japan	Takehiko Saito (National Institute of Animal Health)
EPI_ISL_240008	A/mute swan/Ibaraki/249T/2016	A / H5N6	Asia / Japan	Takehiko Saito (National Institute of Animal Health)
EPI_ISL_240009	A/mute swan/Ibaraki/242T/2016	A / H5N6	Asia / Japan	Takehiko Saito (National Institute of Animal Health)
EPI_ISL_240010	A/black headed gull/Ibaraki/235C/2016	A / H5N6	Asia / Japan	Takehiko Saito (National Institute of Animal Health)
EPI_ISL_240011	A/mute swan/Ibaraki/254C/2016	A / H5N6	Asia / Japan	Takehiko Saito (National Institute of Animal Health)
EPI_ISL_240012	A/duck/France/161108h/2016	A / H5N8	Europe / France / Midi-Pyrenees / Departement du Tarn	Francois-Xavier Briand (ANSES Agence Nationale De Securite Sanitaire De L'alimentation / Laboratoire de Ploufragan-Plouzané)
EPI_ISL_240101	A/mute swan/Croatia/102/2016	A / H5N5	Europe / Croatia / Sisacko-Moslavacka Zupanija / Crkveni Bok	Vladimir Savi? (Croatian Veterinary Institute / Poultry Centre)
EPI_ISL_240102	A/domestic goose/Poland/33/2016	A / H5N8	Europe / Poland / Lubusz Voivodeship	Edyta ?wi?to? (National Veterinary Research Institut Poland, PIWet-PIB)
EPI_ISL_240103	A/domestic goose/Poland/72/2016	A / H5N8	Europe / Poland / Lubusz Voivodeship	Edyta ?wi?to? (National Veterinary Research Institut Poland, PIWet-PIB)
EPI_ISL_240104	A/chicken/Poland/79A/2016	A / H5N8	Europe / Poland / Subcarpathian Voivodeship	Edyta ?wi?to? (National Veterinary Research Institut Poland, PIWet-PIB)
EPI_ISL_240105	A/chicken/Poland/85A/2016	A / H5N8	Europe / Poland / Lesser Poland Voivodeship	Edyta ?wi?to? (National Veterinary Research Institut Poland, PIWet-PIB)
EPI_ISL_240106	A/mute swan/Poland/108/2016	A / H5N8	Europe / Poland / Masovian Voivodeship	Edyta ?wi?to? (National Veterinary Research Institut Poland, PIWet-PIB)
EPI_ISL_240107	A/turkey/Poland/78/2016	A / H5N8	Europe / Poland / Lubusz Voivodeship	Edyta ?wi?to? (National Veterinary Research Institut Poland, PIWet-PIB)
EPI_ISL_240108	A/turkey/Poland/83/2016	A / H5N8	Europe / Poland / Lubusz Voivodeship	Edyta ?wi?to? (National Veterinary Research Institut Poland, PIWet-PIB)
EPI_ISL_240109	A/chicken/Kalmykia/2661/2016	A / H5N8	Europe / Russian Federation / Republic of Kalmykia	Ivan Susloparov (State Research Center of Virology and Biotechnology (VECTOR) / Emerging Zoonotic Diseases and Influenza)
EPI_ISL_240110	A/chicken/Astrakhan/3131/2016	A / H5N8	Europe / Russian Federation / Astrakhan Oblast	Ivan Susloparov (State Research Center of Virology and Biotechnology (VECTOR) / Emerging Zoonotic Diseases and Influenza)
EPI_ISL_240111	A/herring gull/Poland/84/2016	A / H5N8	Europe / Poland / West Pomeranian Voivodeship / Swinoujscie	Edyta ?wi?to? (National Veterinary Research Institut Poland, PIWet-PIB)
EPI_ISL_2402936	A/chicken/Czech Republic/10251-1/2021	A / H5N8	Europe / Czech Republic / Stredocesky Kraj / Okres Melnik / Chorusice	Alexander Nagy (State Veterinary Institute Prague)
EPI_ISL_2402937	A/chicken/Czech Republic/10251-2/2021	A / H5N8	Europe / Czech Republic / Stredocesky Kraj / Okres Melnik / Chorusice	Alexander Nagy (State Veterinary Institute Prague)
EPI_ISL_2402938	A/chicken/Czech Republic/10405/2021	A / H5N8	Europe / Czech Republic / Stredocesky Kraj / Okres Mlada Boleslav	Alexander Nagy (State Veterinary Institute Prague)
EPI_ISL_240527	A/chicken/Niigata/1-1T/2016	A / H5N6	Asia / Japan	Takehiko Saito (National Institute of Animal Health)
EPI_ISL_240528	A/chicken/Niigata/1-2C/2016	A / H5N6	Asia / Japan	Takehiko Saito (National Institute of Animal Health)
EPI_ISL_240529	A/muscovy duck/Aomori/1-3T/2016s	A / H5N6	Asia / Japan	Takehiko Saito (National Institute of Animal Health)
EPI_ISL_240530	A/chicken/Niigata/1-3C/2016	A / H5N6	Asia / Japan	Takehiko Saito (National Institute of Animal Health)
EPI_ISL_240531	A/chicken/Niigata/1-4C/2016	A / H5N6	Asia / Japan	Takehiko Saito (National Institute of Animal Health)
EPI_ISL_240532	A/muscovy duck/Aomori/1-1T/2016	A / H5N6	Asia / Japan	Takehiko Saito (National Institute of Animal Health)
EPI_ISL_240540	A/chicken/Croatia/103/2016	A / H5N8	Europe / Croatia / Viroviticko-Podravska Zupanija / Križnica	Vladimir Savi? (Croatian Veterinary Institute / Poultry Centre)
EPI_ISL_240599	A/chicken/Hokkaido/1-3-7T/2016	A / H5N6	Asia / Japan	Takehiko Saito (National Institute of Animal Health)
EPI_ISL_240601	A/mute swan/Kyoto/8T/2016	A / H5N6	Asia / Japan	Takehiko Saito (National Institute of Animal Health)
EPI_ISL_240604	A/mute swan/Kyoto/6T/2016	A / H5N6	Asia / Japan	Takehiko Saito (National Institute of Animal Health)
EPI_ISL_240605	A/mute swan/Kyoto/5T/2016	A / H5N6	Asia / Japan	Takehiko Saito (National Institute of Animal Health)
EPI_ISL_240606	A/chicken/Miyazaki/1-4C/2016	A / H5N6	Asia / Japan	Takehiko Saito (National Institute of Animal Health)
EPI_ISL_240607	A/mute swan/Kyoto/4T/2016	A / H5N6	Asia / Japan	Takehiko Saito (National Institute of Animal Health)
EPI_ISL_240608	A/black headed gull/Ibaraki/258T/2016	A / H5N6	Asia / Japan	Takehiko Saito (National Institute of Animal Health)
EPI_ISL_240609	A/mute swan/Kyoto/3T/2016	A / H5N6	Asia / Japan	Takehiko Saito (National Institute of Animal Health)
EPI_ISL_240611	A/mute swan/Ibaraki/269T/2016	A / H5N6	Asia / Japan	Takehiko Saito (National Institute of Animal Health)
EPI_ISL_240612	A/chicken/Hokkaido/1-1C2C/2016	A / H5N6	Asia / Japan	Takehiko Saito (National Institute of Animal Health)
EPI_ISL_240613	A/black headed gull/Ibaraki/265T/2016	A / H5N6	Asia / Japan	Takehiko Saito (National Institute of Animal Health)
EPI_ISL_240615	A/mute swan/Ibaraki/255C/2016	A / H5N6	Asia / Japan	Takehiko Saito (National Institute of Animal Health)
EPI_ISL_240617	A/mute swan/Kyoto/2T/2016	A / H5N6	Asia / Japan	Takehiko Saito (National Institute of Animal Health)
EPI_ISL_240618	A/chicken/Hokkaido/1-3-7C/2016	A / H5N6	Asia / Japan	Takehiko Saito (National Institute of Animal Health)
EPI_ISL_240619	A/black swan/Ibaraki/272C/2016	A / H5N6	Asia / Japan	Takehiko Saito (National Institute of Animal Health)
EPI_ISL_240621	A/chicken/Miyazaki/1-7C/2016	A / H5N6	Asia / Japan	Takehiko Saito (National Institute of Animal Health)
EPI_ISL_240622	A/chicken/Miyazaki/1-6T/2016	A / H5N6	Asia / Japan	Takehiko Saito (National Institute of Animal Health)
EPI_ISL_240624	A/chicken/Miyazaki/1-6C/2016	A / H5N6	Asia / Japan	Takehiko Saito (National Institute of Animal Health)
EPI_ISL_240625	A/chicken/Miyazaki/1-7T/2016	A / H5N6	Asia / Japan	Takehiko Saito (National Institute of Animal Health)
EPI_ISL_240626	A/black swan/Ibaraki/259T/2016	A / H5N6	Asia / Japan	Takehiko Saito (National Institute of Animal Health)
EPI_ISL_240627	A/mute swan/Ibaraki/211T/2016	A / H5N6	Asia / Japan	Takehiko Saito (National Institute of Animal Health)
EPI_ISL_240628	A/mute swan/Kyoto/1T/2016	A / H5N6	Asia / Japan	Takehiko Saito (National Institute of Animal Health)
EPI_ISL_240670	A/mute swan/Croatia/9/2017	A / H5N8	Europe / Croatia / Grad Zagreb	Vladimir Savi? (Croatian Veterinary Institute / Poultry Centre)
EPI_ISL_240671	A/mute swan/Croatia/104/2016	A / H5N8	Europe / Croatia / Sisacko-Moslavacka Zupanija / Mužilov?ica	Vladimir Savi? (Croatian Veterinary Institute / Poultry Centre)
EPI_ISL_240677	A/domestic duck/Siberia/103/2016	A / H5N8	Europe / Russian Federation / Novosibirsk Oblast	Ivan Sobolev (Research Institute of Experimental and Clinical Medicine)
EPI_ISL_240678	A/domestic duck/Siberia/50K/2016	A / H5N8	Europe / Russian Federation / Novosibirsk Oblast	Ivan Sobolev (Research Institute of Experimental and Clinical Medicine)
EPI_ISL_240702	A/Eurasian wigeon/Netherlands/1/2016	A / H5N8	Europe / Netherlands / Provincie Noord-Holland / Edam-Volendam	Maria Johanna Poen (Erasmus Medical Center / Department of Virology)
EPI_ISL_240703	A/Hunan/55555/2016	A / H5N6	Asia / China / Hunan Province	Lei Yang (WHO Chinese National Influenza Center / Virology Institute, Chinese CDC)
EPI_ISL_240704	A/Guangxi/55726/2016	A / H5N6	Asia / China / Guangxi Zhuang Autonomous Region	Lei Yang (WHO Chinese National Influenza Center / Virology Institute, Chinese CDC)
EPI_ISL_240892	A/turkey/Germany-NI/R10523/2016	A / H5N8	Europe / Germany / Lower Saxony	Elke Starick (Friedrich-Loeffler-Institut)
EPI_ISL_240893	A/swan/Germany-SN/R10645/2016	A / H5N5	Europe / Germany / Saxony	Elke Starick (Friedrich-Loeffler-Institut)
EPI_ISL_241249	A/domestic duck/Germany-MV/R9869/2016	A / H5N8	Europe / Germany / Mecklenburg-Vorpommern	Elke Starick (Friedrich-Loeffler-Institut)

EPI_ISL_241745	A/chicken/Kumamoto/1-7T/2016	A / H5N6	Asia / Japan	Takehiko Saito (National Institute of Animal Health)
EPI_ISL_241746	A/black headed gull/Ibaraki/282T/2016	A / H5N6	Asia / Japan	Takehiko Saito (National Institute of Animal Health)
EPI_ISL_241747	A/black-headed-gull/Ibaraki/267T/2016	A / H5N6	Asia / Japan	Takehiko Saito (National Institute of Animal Health)
EPI_ISL_241748	A/chicken/Kumamoto/1-1T/2016	A / H5N6	Asia / Japan	Takehiko Saito (National Institute of Animal Health)
EPI_ISL_241749	A/mute swan/Ibaraki/263T/2016	A / H5N6	Asia / Japan	Takehiko Saito (National Institute of Animal Health)
EPI_ISL_241750	A/mute swan/Ibaraki/262C/2016	A / H5N6	Asia / Japan	Takehiko Saito (National Institute of Animal Health)
EPI_ISL_241751	A/chicken/Kumamoto/1-4C/2016	A / H5N6	Asia / Japan	Takehiko Saito (National Institute of Animal Health)
EPI_ISL_241752	A/whooper swan/Ibaraki/301T/2016	A / H5N6	Asia / Japan	Takehiko Saito (National Institute of Animal Health)
EPI_ISL_241753	A/black swan/Ibaraki/290C/2016	A / H5N6	Asia / Japan	Takehiko Saito (National Institute of Animal Health)
EPI_ISL_241754	A/great crested grebe/Ibaraki/286T/2016	A / H5N6	Asia / Japan	Takehiko Saito (National Institute of Animal Health)
EPI_ISL_241755	A/great crested grebe/Ibaraki/287T/2016	A / H5N6	Asia / Japan	Takehiko Saito (National Institute of Animal Health)
EPI_ISL_241756	A/mute swan/Ibaraki/252T/2016	A / H5N6	Asia / Japan	Takehiko Saito (National Institute of Animal Health)
EPI_ISL_241757	A/black swan/Ibaraki/256C/2016	A / H5N6	Asia / Japan	Takehiko Saito (National Institute of Animal Health)
EPI_ISL_241758	A/chicken/Miyazaki/1-5C/2016	A / H5N6	Asia / Japan	Takehiko Saito (National Institute of Animal Health)
EPI_ISL_241759	A/black headed gull/Ibaraki/291T/2016	A / H5N6	Asia / Japan	Takehiko Saito (National Institute of Animal Health)
EPI_ISL_241760	A/chicken/Kumamoto/1-2C/2016	A / H5N6	Asia / Japan	Takehiko Saito (National Institute of Animal Health)
EPI_ISL_241761	A/chicken/Kumamoto/1-3T/2016	A / H5N6	Asia / Japan	Takehiko Saito (National Institute of Animal Health)
EPI_ISL_241762	A/black swan/Ibaraki/302C/2016	A / H5N6	Asia / Japan	Takehiko Saito (National Institute of Animal Health)
EPI_ISL_241763	A/chicken/Kumamoto/1-6T/2016	A / H5N6	Asia / Japan	Takehiko Saito (National Institute of Animal Health)
EPI_ISL_241764	A/chicken/Kumamoto/1-7C/2016	A / H5N6	Asia / Japan	Takehiko Saito (National Institute of Animal Health)
EPI_ISL_241765	A/chicken/Kumamoto/1-4T/2016	A / H5N6	Asia / Japan	Takehiko Saito (National Institute of Animal Health)
EPI_ISL_241766	A/chicken/Kumamoto/1-5C/2016	A / H5N6	Asia / Japan	Takehiko Saito (National Institute of Animal Health)
EPI_ISL_241767	A/black headed gull/Ibaraki/284T/2016	A / H5N6	Asia / Japan	Takehiko Saito (National Institute of Animal Health)
EPI_ISL_241768	A/mute swan/Ibaraki/270T/2016	A / H5N6	Asia / Japan	Takehiko Saito (National Institute of Animal Health)
EPI_ISL_241769	A/mute swan/Ibaraki/248C/2016	A / H5N6	Asia / Japan	Takehiko Saito (National Institute of Animal Health)
EPI_ISL_241770	A/chicken/Kumamoto/1-3C/2016	A / H5N6	Asia / Japan	Takehiko Saito (National Institute of Animal Health)
EPI_ISL_241771	A/black swan/Ibaraki/271C/2016	A / H5N6	Asia / Japan	Takehiko Saito (National Institute of Animal Health)
EPI_ISL_241772	A/black swan/Ibaraki/310C/2017	A / H5N6	Asia / Japan	Takehiko Saito (National Institute of Animal Health)
EPI_ISL_241773	A/pochard/Ibaraki/268T/2016	A / H5N6	Asia / Japan	Takehiko Saito (National Institute of Animal Health)
EPI_ISL_241774	A/chicken/Kumamoto/1-1C/2016	A / H5N6	Asia / Japan	Takehiko Saito (National Institute of Animal Health)
EPI_ISL_241775	A/chicken/Kumamoto/1-2T/2016	A / H5N6	Asia / Japan	Takehiko Saito (National Institute of Animal Health)
EPI_ISL_241776	A/black headed gull/Ibaraki/289T/2016	A / H5N6	Asia / Japan	Takehiko Saito (National Institute of Animal Health)
EPI_ISL_241777	A/great crested grebe/Ibaraki/298C/2016	A / H5N6	Asia / Japan	Takehiko Saito (National Institute of Animal Health)
EPI_ISL_241778	A/muteswan/Ibaraki/277T/2016	A / H5N6	Asia / Japan	Takehiko Saito (National Institute of Animal Health)
EPI_ISL_241779	A/chicken/Kumamoto/1-5T/2016	A / H5N6	Asia / Japan	Takehiko Saito (National Institute of Animal Health)
EPI_ISL_241952	A/breeder duck/Croatia/21/2017	A / H5N8	Europe / Croatia / Zagrebacka Zupanija / Sop Bukevski	Vladimir Savi? (Croatian Veterinary Institute / Poultry Centre)
EPI_ISL_241953	A/mute swan/Croatia/15/2017	A / H5N8	Europe / Croatia / Koprivnicko-Krizevacka Zupanija / Lake Šoderica, Legrad	Vladimir Savi? (Croatian Veterinary Institute / Poultry Centre)
EPI_ISL_242401	A/chicken/Gifu/1-1T/2017	A / H5N6	Asia / Japan	Takehiko Saito (National Institute of Animal Health)
EPI_ISL_242403	A/chicken/Gifu/1-1C/2017	A / H5N6	Asia / Japan	Takehiko Saito (National Institute of Animal Health)
EPI_ISL_242404	A/chicken/Gifu/1-10C/2017	A / H5N6	Asia / Japan	Takehiko Saito (National Institute of Animal Health)
EPI_ISL_242405	A/whooper swan/Ibaraki/331C/2017	A / H5N6	Asia / Japan	Takehiko Saito (National Institute of Animal Health)
EPI_ISL_242406	A/black swan/Ibaraki/341C/2017	A / H5N6	Asia / Japan	Takehiko Saito (National Institute of Animal Health)
EPI_ISL_242407	A/chicken/Gifu/1-2T/2017	A / H5N6	Asia / Japan	Takehiko Saito (National Institute of Animal Health)
EPI_ISL_242408	A/chicken/Gifu/1-10T/2017	A / H5N6	Asia / Japan	Takehiko Saito (National Institute of Animal Health)
EPI_ISL_242409	A/chicken/Gifu/1-6C/2017	A / H5N6	Asia / Japan	Takehiko Saito (National Institute of Animal Health)
EPI_ISL_242411	A/chicken/Gifu/1-6T/2017	A / H5N6	Asia / Japan	Takehiko Saito (National Institute of Animal Health)
EPI_ISL_242412	A/chicken/Gifu/1-9C/2017	A / H5N6	Asia / Japan	Takehiko Saito (National Institute of Animal Health)
EPI_ISL_242413	A/chicken/Gifu/1-8C/2017	A / H5N6	Asia / Japan	Takehiko Saito (National Institute of Animal Health)
EPI_ISL_242414	A/chicken/Gifu/1-2C/2017	A / H5N6	Asia / Japan	Takehiko Saito (National Institute of Animal Health)
EPI_ISL_242415	A/black swan/Ibaraki/341T/2017	A / H5N6	Asia / Japan	Takehiko Saito (National Institute of Animal Health)
EPI_ISL_242666	A/chicken/Kumamoto/1-6C/2016	A / H5N6	Asia / Japan	Takehiko Saito (National Institute of Animal Health)
EPI_ISL_242817	A/chicken/Czech Republic/206-17_2/2017(H5N8)	A / H5N8	Europe / Czech Republic / South Moravian Region / Okres Breclav / Brod nad Dyjí	Alexander Nagy (State Veterinary Institute Prague)
EPI_ISL_242902	A/wigeon/Italy/17VIR57-3/2017	A / H5N8	Europe / Italy / Friuli-Venezia Giulia / Province of Gorizia	Alice Fusaro (Istituto Zooprofilattico Sperimentale Delle Venezie)
EPI_ISL_242903	A/Northern Pintail/Tottori/b37/2016	A / H5N6	Asia / Japan / Tottori	
EPI_ISL_243038	A/domestic mallard/Korea/LBM176/2014	A / H5N8	Asia / Korea, Republic of	Eun-Kyoung Lee (Animal and Plant Quarantine Agency (APQA) / Avian Influenza Research and Diagsnotic Division)
EPI_ISL_243039	A/broiler duck/Korea/LBM281/2014	A / H5N8	Asia / Korea, Republic of	Eun-Kyoung Lee (Animal and Plant Quarantine Agency (APQA) / Avian Influenza Research and Diagsnotic Division)
EPI_ISL_243040	A/Korean native chicken/Korea/LBM282/2014	A / H5N8	Asia / Korea, Republic of	Eun-Kyoung Lee (Animal and Plant Quarantine Agency (APQA) / Avian Influenza Research and Diagsnotic Division)
EPI_ISL_243041	A/Korean native chicken/Korea/LBM287/2014	A / H5N8	Asia / Korea, Republic of	Eun-Kyoung Lee (Animal and Plant Quarantine Agency (APQA) / Avian Influenza Research and Diagsnotic Division)
EPI_ISL_243042	A/Korean native chicken/Korea/LBM288/2014	A / H5N8	Asia / Korea, Republic of	Eun-Kyoung Lee (Animal and Plant Quarantine Agency (APQA) / Avian Influenza Research and Diagsnotic Division)
EPI_ISL_243043	A/Korean native chicken/Korea/LBM367/2014	A / H5N8	Asia / Korea, Republic of	Eun-Kyoung Lee (Animal and Plant Quarantine Agency (APQA) / Avian Influenza Research and Diagsnotic Division)
EPI_ISL_243048	A/turkey/Czech Republic/38-17_5/2017 (H5N8)	A / H5N8	Europe / Czech Republic / South Moravian Region / Okres Brno-Venkov / Ivan?ice-N?m?ice	Alexander Nagy (State Veterinary Institute Prague)
EPI_ISL_243049	A/turkey/Germany-SH/R425/2017	A / H5N5	Europe / Germany / Schleswig-Holstein	Elke Starick (Friedrich-Loeffler-Institut)
EPI_ISL_243058	A/black swan/Akita/1/2016	A / H5N6	Asia / Japan / Akita	
EPI_ISL_243059	A/black swan/Akita/2/2016	A / H5N6	Asia / Japan / Akita	
EPI_ISL_243060	A/teal/Tottori/1/2016	A / H5N6	Asia / Japan / Tottori	
EPI_ISL_243061	A/teal/Tottori/2/2016	A / H5N6	Asia / Japan / Tottori	
EPI_ISL_243085	A/wigeon/Italy/16VIR9616-3/2016	A / H5N5	Europe / Italy / Friuli-Venezia Giulia / Province of Gorizia	Alice Fusaro (Istituto Zooprofilattico Sperimentale Delle Venezie)
EPI_ISL_243344	A/wild pigeon/Jilin/CC01/2014	A / H5N6	Asia / China	
EPI_ISL_243345	A/environment/Jilin/CC02/2014	A / H5N6	Asia / China	
EPI_ISL_243672	A/black swan/Ibaraki/365C/2017	A / H5N6	Asia / Japan	Takehiko Saito (National Institute of Animal Health)
EPI_ISL_243673	A/chicken/Miyazaki/2-2C/2017	A / H5N6	Asia / Japan	Takehiko Saito (National Institute of Animal Health)
EPI_ISL_243674	A/black swan/Ibaraki/356C/2017	A / H5N6	Asia / Japan	Takehiko Saito (National Institute of Animal Health)

EPI_ISL_243675	A/black swan/Ibaraki/348C/2017	A / H5N6	Asia / Japan	Takehiko Saito (National Institute of Animal Health)
EPI_ISL_243676	A/black swan/Ibaraki/357T/2017	A / H5N6	Asia / Japan	Takehiko Saito (National Institute of Animal Health)
EPI_ISL_243677	A/black swan/Ibaraki/345T/2017	A / H5N6	Asia / Japan	Takehiko Saito (National Institute of Animal Health)
EPI_ISL_243678	A/black swan/Ibaraki/350C/2017	A / H5N6	Asia / Japan	Takehiko Saito (National Institute of Animal Health)
EPI_ISL_243679	A/black swan/Ibaraki/345C/2017	A / H5N6	Asia / Japan	Takehiko Saito (National Institute of Animal Health)
EPI_ISL_243680	A/chicken/Miyazaki/2-1T/2017	A / H5N6	Asia / Japan	Takehiko Saito (National Institute of Animal Health)
EPI_ISL_243681	A/chicken/Miyazaki/2-4C/2017	A / H5N6	Asia / Japan	Takehiko Saito (National Institute of Animal Health)
EPI_ISL_243682	A/black swan/Ibaraki/365T/2017	A / H5N6	Asia / Japan	Takehiko Saito (National Institute of Animal Health)
EPI_ISL_243683	A/chicken/Miyazaki/2-1C/2017	A / H5N6	Asia / Japan	Takehiko Saito (National Institute of Animal Health)
EPI_ISL_243684	A/chicken/Miyazaki/2-5T/2017	A / H5N6	Asia / Japan	Takehiko Saito (National Institute of Animal Health)
EPI_ISL_243685	A/black swan/Ibaraki/357C/2017	A / H5N6	Asia / Japan	Takehiko Saito (National Institute of Animal Health)
EPI_ISL_243686	A/whooper swan/Ibaraki/351C/2017	A / H5N6	Asia / Japan	Takehiko Saito (National Institute of Animal Health)
EPI_ISL_243687	A/chicken/Miyazaki/2-3C/2017	A / H5N6	Asia / Japan	Takehiko Saito (National Institute of Animal Health)
EPI_ISL_243688	A/black swan/Ibaraki/356T/2017	A / H5N6	Asia / Japan	Takehiko Saito (National Institute of Animal Health)
EPI_ISL_243689	A/black swan/Ibaraki/350T/2017	A / H5N6	Asia / Japan	Takehiko Saito (National Institute of Animal Health)
EPI_ISL_243693	A/mute swan/Croatia/30/2017	A / H5N8	Europe / Croatia / Osjecko-Baranjska Zupanija / Bilje	Vladimir Savi? (Croatian Veterinary Institute / Poultry Centre)
EPI_ISL_243695	A/greylag goose/Croatia/33/2017	A / H5N8	Europe / Croatia / Bjelovarsko-Bilogorska Zupanija / Prekobrdo	Vladimir Savi? (Croatian Veterinary Institute / Poultry Centre)
EPI_ISL_243698	A/mute swan/Croatia/42/2017	A / H5N5	Europe / Croatia / Osjecko-Baranjska Zupanija / road between villages Kozjak and Tikves	Vladimir Savi? (Croatian Veterinary Institute / Poultry Centre)
EPI_ISL_244486	A/chicken/Vietnam/NCVD14-A324/2014	A / H5N6	Asia / Vietnam	
EPI_ISL_244487	A/chicken/Vietnam/NCVD-15A22/2015	A / H5N6	Asia / Vietnam	
EPI_ISL_244488	A/Pheasant/Vietnam/NCVD14-A367/2014	A / H5N6	Asia / Vietnam	
EPI_ISL_244489	A/duck/Vietnam/NCVD14-A392/2014	A / H5N6	Asia / Vietnam	
EPI_ISL_244510	A/duck/Vietnam/NCVD14-A415/2014	A / H5N6	Asia / Vietnam	
EPI_ISL_244511	A/duck/Vietnam/NCVD14-A418/2014	A / H5N6	Asia / Vietnam	
EPI_ISL_244512	A/duck/Vietnam/NCVD14-A503/2014	A / H5N6	Asia / Vietnam	
EPI_ISL_244513	A/chicken/Vietnam/NCVD-15A17/2015	A / H5N6	Asia / Vietnam	
EPI_ISL_244514	A/goose/Vietnam/NCVD-15A27/2015	A / H5N6	Asia / Vietnam	
EPI_ISL_244515	A/chicken/Vietnam/NCVD-15A51/2015	A / H5N6	Asia / Vietnam	
EPI_ISL_244516	A/chicken/Vietnam/NCVD-15A55/2015	A / H5N6	Asia / Vietnam	
EPI_ISL_244517	A/duck/Vietnam/NCVD-15A57/2015	A / H5N6	Asia / Vietnam	
EPI_ISL_244518	A/chicken/Vietnam/NCVD-15A59/2015	A / H5N6	Asia / Vietnam	
EPI_ISL_244519	A/environment/Korea/W541/2016	A / H5N6	Asia / Korea, Republic of	
EPI_ISL_244520	A/environment/Korea/W542/2016	A / H5N6	Asia / Korea, Republic of	
EPI_ISL_244521	A/environment/Korea/W543/2016	A / H5N6	Asia / Korea, Republic of	
EPI_ISL_244522	A/environment/Korea/W544/2016	A / H5N6	Asia / Korea, Republic of	
EPI_ISL_244523	A/whooper swan/Korea/Gangjin 48/2016	A / H5N6	Asia / Korea, Republic of	
EPI_ISL_244524	A/duck/Hubei/ZYSYF1/2015	A / H5N6	Asia / China	
EPI_ISL_244525	A/duck/Hubei/ZYSYF24/2015	A / H5N6	Asia / China	
EPI_ISL_244526	A/duck/Hubei/ZYSYG18/2015	A / H5N6	Asia / China	
EPI_ISL_244527	A/duck/Hubei/ZYSYG5/2015	A / H5N6	Asia / China	
EPI_ISL_244528	A/chicken/Hubei/ZYSJF11/2016	A / H5N6	Asia / China	
EPI_ISL_244529	A/chicken/Hubei/ZYSJF16/2016	A / H5N6	Asia / China	
EPI_ISL_244530	A/chicken/Hubei/ZYSJF22/2016	A / H5N6	Asia / China	
EPI_ISL_244531	A/chicken/Hubei/ZYSJF38/2016	A / H5N6	Asia / China	
EPI_ISL_244532	A/chicken/Taishun/TS12/2016	A / H5N6	Asia / China	
EPI_ISL_244533	A/chicken/Taishun/TS90/2016	A / H5N6	Asia / China	
EPI_ISL_244534	A/chicken/Taishun/TS2/2016	A / H5N6	Asia / China	
EPI_ISL_244535	A/chicken/Ganzhou/GZ27/2015	A / H5N6	Asia / China	
EPI_ISL_244536	A/chicken/Ganzhou/GZ21/2015	A / H5N6	Asia / China	
EPI_ISL_244537	A/chicken/Ganzhou/GZ50/2015	A / H5N6	Asia / China	
EPI_ISL_244538	A/whooper swan/Korea/Gangjin 49_2/2016	A / H5N6	Asia / Korea, Republic of	
EPI_ISL_244539	A/whooper swan/Korea/Gangjin 49_1/2016	A / H5N6	Asia / Korea, Republic of	
EPI_ISL_247371	A/mute swan/Ibaraki/219T/2016	A / H5N6	Asia / Japan / Ibaraki	Takehiko Saito (National Institute of Animal Health)
EPI_ISL_247372	A/mute swan/Ibaraki/220C/2016	A / H5N6	Asia / Japan / Ibaraki	Takehiko Saito (National Institute of Animal Health)
EPI_ISL_247373	A/black headed gull/Ibaraki/194C/2016	A / H5N6	Asia / Japan / Ibaraki	Takehiko Saito (National Institute of Animal Health)
EPI_ISL_247374	A/mute swan/Ibaraki/214C/2016	A / H5N6	Asia / Japan / Ibaraki	Takehiko Saito (National Institute of Animal Health)
EPI_ISL_247375	A/mute swan/Ibaraki/222C/2016	A / H5N6	Asia / Japan / Ibaraki	Takehiko Saito (National Institute of Animal Health)
EPI_ISL_247376	A/mute swan/Ibaraki/208T/2016	A / H5N6	Asia / Japan / Ibaraki	Takehiko Saito (National Institute of Animal Health)
EPI_ISL_247377	A/mute swan/Ibaraki/223C/2016	A / H5N6	Asia / Japan / Ibaraki	Takehiko Saito (National Institute of Animal Health)
EPI_ISL_247378	A/chicken/Saga/1-1T/2017	A / H5N6	Asia / Japan / Saga	Takehiko Saito (National Institute of Animal Health)
EPI_ISL_247379	A/chicken/Saga/1-5T/2017	A / H5N6	Asia / Japan / Saga	Takehiko Saito (National Institute of Animal Health)
EPI_ISL_247380	A/chicken/Saga/1-4C/2017	A / H5N6	Asia / Japan / Saga	Takehiko Saito (National Institute of Animal Health)
EPI_ISL_247381	A/mute swan/Ibaraki/217C/2016	A / H5N6	Asia / Japan / Ibaraki	Takehiko Saito (National Institute of Animal Health)
EPI_ISL_247382	A/chicken/Saga/1-1C/2017	A / H5N6	Asia / Japan / Saga	Takehiko Saito (National Institute of Animal Health)
EPI_ISL_247383	A/mute swan/Ibaraki/202C/2016	A / H5N6	Asia / Japan / Ibaraki	Takehiko Saito (National Institute of Animal Health)
EPI_ISL_247384	A/chicken/Saga/1-3C/2017	A / H5N6	Asia / Japan / Saga	Takehiko Saito (National Institute of Animal Health)
EPI_ISL_247385	A/chicken/Saga/1-2C/2017	A / H5N6	Asia / Japan / Saga	Takehiko Saito (National Institute of Animal Health)
EPI_ISL_247386	A/mute swan/Ibaraki/214T/2016	A / H5N6	Asia / Japan / Ibaraki	Takehiko Saito (National Institute of Animal Health)
EPI_ISL_247387	A/mute swan/Ibaraki/202T/2016	A / H5N6	Asia / Japan / Ibaraki	Takehiko Saito (National Institute of Animal Health)
EPI_ISL_247388	A/mute swan/Ibaraki/222T/2016	A / H5N6	Asia / Japan / Ibaraki	Takehiko Saito (National Institute of Animal Health)
EPI_ISL_247389	A/mute swan/Ibaraki/223T/2016	A / H5N6	Asia / Japan / Ibaraki	Takehiko Saito (National Institute of Animal Health)
EPI_ISL_247390	A/mute swan/Ibaraki/218T/2016	A / H5N6	Asia / Japan / Ibaraki	Takehiko Saito (National Institute of Animal Health)

EPI_ISL_247391	A/mute swan/Ibaraki/217T/2016	A / H5N6	Asia / Japan / Ibaraki	Takehiko Saito (National Institute of Animal Health)
EPI_ISL_247392	A/chicken/Saga/1-6T/2017	A / H5N6	Asia / Japan / Saga	Takehiko Saito (National Institute of Animal Health)
EPI_ISL_247393	A/chicken/Saga/1-5C/2017	A / H5N6	Asia / Japan / Saga	Takehiko Saito (National Institute of Animal Health)
EPI_ISL_247394	A/whooper swan/Ibaraki/188C/2016	A / H5N6	Asia / Japan / Ibaraki	Takehiko Saito (National Institute of Animal Health)
EPI_ISL_247395	A/chicken/Saga/1-6C/2017	A / H5N6	Asia / Japan / Saga	Takehiko Saito (National Institute of Animal Health)
EPI_ISL_247396	A/chicken/Saga/1-2T/2017	A / H5N6	Asia / Japan / Saga	Takehiko Saito (National Institute of Animal Health)
EPI_ISL_247397	A/chicken/Saga/1-3T/2017	A / H5N6	Asia / Japan / Saga	Takehiko Saito (National Institute of Animal Health)
EPI_ISL_247398	A/chicken/Saga/1-4T/2017	A / H5N6	Asia / Japan / Saga	Takehiko Saito (National Institute of Animal Health)
EPI_ISL_247399	A/chicken/Saga/1-7C/2017	A / H5N6	Asia / Japan / Saga	Takehiko Saito (National Institute of Animal Health)
EPI_ISL_247400	A/mute swan/Ibaraki/210T/2016	A / H5N6	Asia / Japan / Ibaraki	Takehiko Saito (National Institute of Animal Health)
EPI_ISL_247401	A/mute swan/Ibaraki/221C/2016	A / H5N6	Asia / Japan / Ibaraki	Takehiko Saito (National Institute of Animal Health)
EPI_ISL_247402	A/mute swan/Ibaraki/219C/2016	A / H5N6	Asia / Japan / Ibaraki	Takehiko Saito (National Institute of Animal Health)
EPI_ISL_247403	A/chicken/Saga/1-7T/2017	A / H5N6	Asia / Japan / Saga	Takehiko Saito (National Institute of Animal Health)
EPI_ISL_247404	A/mute swan/Ibaraki/210C/2016	A / H5N6	Asia / Japan / Ibaraki	Takehiko Saito (National Institute of Animal Health)
EPI_ISL_247405	A/mute swan/Ibaraki/220T/2016	A / H5N6	Asia / Japan / Ibaraki	Takehiko Saito (National Institute of Animal Health)
EPI_ISL_247406	A/black headed gull/Ibaraki/194T/2016	A / H5N6	Asia / Japan / Ibaraki	Takehiko Saito (National Institute of Animal Health)
EPI_ISL_247407	A/mute swan/Ibaraki/218C/2016	A / H5N6	Asia / Japan / Ibaraki	Takehiko Saito (National Institute of Animal Health)
EPI_ISL_247429	A/domestic duck/Germany-BB/R681ff/2017	A / H5N8	Europe / Germany / Brandenburg	Elke Starick (Friedrich-Loeffler-Institut)
EPI_ISL_247715	A/long-eared owl/Voronezh/15/2017	A / H5N8	Europe / Russian Federation / Voronezh Oblast	Ivan Susloparov (State Research Center of Virology and Biotechnology (VECTOR) / Emerging Zoonotic Diseases and Influenza)
EPI_ISL_247716	A/Ural owl/Voronezh/14/2017	A / H5N8	Europe / Russian Federation / Voronezh Oblast	Ivan Susloparov (State Research Center of Virology and Biotechnology (VECTOR) / Emerging Zoonotic Diseases and Influenza)
EPI_ISL_247717	A/long-eared owl/Voronezh/16/2017	A / H5N8	Europe / Russian Federation / Voronezh Oblast	Ivan Susloparov (State Research Center of Virology and Biotechnology (VECTOR) / Emerging Zoonotic Diseases and Influenza)
EPI_ISL_247718	A/chicken/Voronezh/18/2017	A / H5N8	Europe / Russian Federation / Voronezh Oblast	Ivan Susloparov (State Research Center of Virology and Biotechnology (VECTOR) / Emerging Zoonotic Diseases and Influenza)
EPI_ISL_247719	A/chicken/Voronezh/19/2017	A / H5N8	Europe / Russian Federation / Voronezh Oblast	Ivan Susloparov (State Research Center of Virology and Biotechnology (VECTOR) / Emerging Zoonotic Diseases and Influenza)
EPI_ISL_247720	A/chicken/Voronezh/20/2017	A / H5N8	Europe / Russian Federation / Voronezh Oblast	Ivan Susloparov (State Research Center of Virology and Biotechnology (VECTOR) / Emerging Zoonotic Diseases and Influenza)
EPI_ISL_247721	A/turkey/Rostov-on-Don/11/2017	A / H5N8	Europe / Russian Federation / Rostov Oblast	Ivan Susloparov (State Research Center of Virology and Biotechnology (VECTOR) / Emerging Zoonotic Diseases and Influenza)
EPI_ISL_247722	A/goose/Krasnodar/3144/2017	A / H5N8	Europe / Russian Federation / Krasnodar Krai	Ivan Susloparov (State Research Center of Virology and Biotechnology (VECTOR) / Emerging Zoonotic Diseases and Influenza)
EPI_ISL_247723	A/mute swan/Krasnodar/25/2017	A / H5N8	Europe / Russian Federation / Krasnodar Krai	Ivan Susloparov (State Research Center of Virology and Biotechnology (VECTOR) / Emerging Zoonotic Diseases and Influenza)
EPI_ISL_247724	A/wild duck/Tatarstan/3059/2016	A / H5N8	Europe / Russian Federation / Republic of Tatarstan	Ivan Susloparov (State Research Center of Virology and Biotechnology (VECTOR) / Emerging Zoonotic Diseases and Influenza)
EPI_ISL_247725	A/chicken/Kalmykia/2643/2016	A / H5N8	Europe / Russian Federation / Republic of Kalmykia	Ivan Susloparov (State Research Center of Virology and Biotechnology (VECTOR) / Emerging Zoonotic Diseases and Influenza)
EPI_ISL_248634	A/duck/Taiwan/1702004/2017	A / H5N6	Asia / Taiwan	Yu-Pin Liu (Animal Health Research Institute)
EPI_ISL_248657	A/mute swan/Czech Republic/653-17/2017 (H5N8)	A / H5N8	Europe / Czech Republic / Hlavni mesto Praha / Okres Praha / Praha 1, St?elecký ostrov	Alexander Nagy (State Veterinary Institute Prague)
EPI_ISL_248663	A/mallard/Czech Republic/722-17_2/2017 (H5N8)	A / H5N8	Europe / Czech Republic / Jihocesky Kraj / Okres Tabor / Praha 1, St?elecký ostrov	Alexander Nagy (State Veterinary Institute Prague)
EPI_ISL_248664	A/mute swan/Croatia/61/2017	A / H5N8	Europe / Croatia / Karlovacka Zupanija / Karlovac	Vladimir Savi? (Croatian Veterinary Institute / Poultry Centre)
EPI_ISL_248666	A/bronze turkey/Czech Republic/1414-17/2017 (H5N8)	A / H5N8	Europe / Czech Republic / Ustecky Kraj / Okres Chomutov / Bílenice, GPS: 50°25'18.168''N, 13°3(	Alexander Nagy (State Veterinary Institute Prague)
EPI_ISL_248879	A/chicken/Croatia/70/2017	A / H5N8	Europe / Croatia / Vukovarsko-Srijemska Zupanija / Mlaka Antinska	Vladimir Savi? (Croatian Veterinary Institute / Poultry Centre)
EPI_ISL_248934	A/mute swan/Czech Republic/722-17_1/2017 (H5N8)	A / H5N8	Europe / Czech Republic / Jihocesky Kraj / Okres Tabor / Lake Jordán, Van?urova Street	Alexander Nagy (State Veterinary Institute Prague)
EPI_ISL_249324	A/chicken/Czech Republic/585-17_1/2017 (H5N8)	A / H5N8	Europe / Czech Republic / Jihocesky Kraj / Okres Strakonice / Vod?any	Alexander Nagy (State Veterinary Institute Prague)
EPI_ISL_249681	A/turkey/Germany-BB/R234ff/2017	A / H5N8	Europe / Germany / Brandenburg / LK Ostprignitz-Ruppin	Elke Starick (Friedrich-Loeffler-Institut)
EPI_ISL_249691	A/chicken/Germany-MV/R10048/2016	A / H5N8	Europe / Germany / Mecklenburg-Vorpommern / Neukloster	Elke Starick (Friedrich-Loeffler-Institut)
EPI_ISL_249692	A/chicken/Germany-NI/R11406/2016	A / H5N8	Europe / Germany / Lower Saxony / LK Northeim	Elke Starick (Friedrich-Loeffler-Institut)
EPI_ISL_250231	A/domestic_duck/Siberia/49_feather/2016	A / H5N8	Europe / Russian Federation / Novosibirsk Oblast / Chany lake	Artem Fadeev (WHO National Influenza Centre Russian Federation)
EPI_ISL_250885	A/mute swan/Czech Republic/54-17_2/2017 (H5N8)	A / H5N8	Europe / Czech Republic / South Moravian Region / Okres Blansko / N?m?ice	Alexander Nagy (State Veterinary Institute Prague)
EPI_ISL_250886	A/Indian Runner Duck/Czech Republic/749-17/2017 (H5N8)	A / H5N8	Europe / Czech Republic / Kralovehradecky Kraj / Okres Nachod / Náchod-Babí GPS: 52°25'56.20	Alexander Nagy (State Veterinary Institute Prague)
EPI_ISL_250887	A/chicken/Czech Republic/55-17_1/2017 (H5N8)	A / H5N8	Europe / Czech Republic / South Moravian Region / Okres Znoimo / Moravský Krumlov-Rakšice	Alexander Nagy (State Veterinary Institute Prague)
EPI_ISL_250902	A/mute swan/Czech Republic/879-17/2017 (H5N8)	A / H5N8	Europe / Czech Republic / Kralovehradecky Kraj / Okres Hradec Kralove / Most u Soutoku, GPS: N	Alexander Nagy (State Veterinary Institute Prague)
EPI_ISL_250917	A/duck/Czech Republic/1467-17/2017 (H5N8)	A / H5N8	Europe / Czech Republic / Jihocesky Kraj / Okres Prachatice / Vlachovo B?ezí	Alexander Nagy (State Veterinary Institute Prague)
EPI_ISL_250918	A/chicken/Czech Republic/1863-17_1/2017 (H5N8)	A / H5N8	Europe / Czech Republic / Stredocesky Kraj / Okres Benesov / Dolní Kralovice	Alexander Nagy (State Veterinary Institute Prague)
EPI_ISL_250919	A/chicken/Czech Republic/1896-17_1/2017 (H5N8)	A / H5N8	Europe / Czech Republic / Jihocesky Kraj / Okres Jindrichuv Hradec / Nová Ves nad Lužnicí, GPS: [illegible]	Alexander Nagy (State Veterinary Institute Prague)
EPI_ISL_250920	A/mute swan/Czech Republic/2031-17/2017 (H5N5)	A / H5N5	Europe / Czech Republic / Liberecky Kraj / Okres Liberec / ZOO Liberec, GPS: 50°46'49.640''N, 15	Alexander Nagy (State Veterinary Institute Prague)
EPI_ISL_252881	A/chicken/Czech Republic/2764-17_1/2017 (H5N8)	A / H5N8	Europe / Czech Republic / Karlovarsky Kraj / Okres Karlovy Vary / Boži?any, GPS: 50°16'18.814''N	Alexander Nagy (State Veterinary Institute Prague)
EPI_ISL_252882	A/chicken/Czech Republic/2764-17_2/2017 (H5N8)	A / H5N8	Europe / Czech Republic / Karlovarsky Kraj / Okres Karlovy Vary / Boži?any, GPS: 50°16'18.814''N	Alexander Nagy (State Veterinary Institute Prague)
EPI_ISL_253029	A/chicken/Zhejiang/194/2016	A / H5N2	Asia / China	
EPI_ISL_253030	A/chicken/Zhejiang/209/2016	A / H5N2	Asia / China	
EPI_ISL_253031	A/chicken/Zhejiang/217/2016	A / H5N2	Asia / China	
EPI_ISL_253032	A/chicken/Zhejiang/232/2016	A / H5N2	Asia / China	
EPI_ISL_253036	A/turkey/England/003778/2017	A / H5N8	Europe / United Kingdom / Lincolnshire	James Seekings (Animal and Plant Health Agency (APHA) / Virology Department)
EPI_ISL_253037	A/chicken/Wales/000023/2016	A / H5N8	Europe / United Kingdom / County of Carmarthenshire	James Seekings (Animal and Plant Health Agency (APHA) / Virology Department)
EPI_ISL_253038	A/pochard_duck/England/SA12_157809/2016	A / H5N8	Europe / United Kingdom / Southport, Merseyside	James Seekings (Animal and Plant Health Agency (APHA) / Virology Department)
EPI_ISL_253946	A/chicken/Japan/AQ-HE144/2015	A / H5N6	Asia / Japan	
EPI_ISL_253949	A/duck/Japan/AQ-HE72/2015	A / H5N6	Asia / Japan	
EPI_ISL_254725	A/chicken/Korea/H903/2017	A / H5N8	Asia / Korea, Republic of / Jeollabuk-do	Youn-Jeong Lee (Animal and Plant Quarantine Agency (APQA) / Avian Influenza Research and Diagsnotic Division)
EPI_ISL_254745	A/mallard duck/Korea/WA137/2017	A / H5N8	Asia / Korea, Republic of / Chungcheongnam-do	Youn-Jeong Lee (Animal and Plant Quarantine Agency (APQA) / Avian Influenza Research and Diagsnotic Division)
EPI_ISL_254814	A/mallard/Hungary/57857/2016	A / H5N8	Europe / Hungary / Hajdu-Bihar	Adam Dan (Danam.Vet.Molbiol)
EPI_ISL_255012	A/American wigeon/California/UCD58P/2015	A / H5N8	North America / United States / California / Solano County / Grizzly Island Wildlife Refuge	Andrew Ramey (U.S. Geological Survey Alaska Science Center)
EPI_ISL_255174	A/Goose/Hungary/65817/2016	A / H5N8	Europe / Hungary / Hajdu-Bihar	Adam Dan (Danam.Vet.Molbiol)
EPI_ISL_255181	A/swan/Italy/17VIR537-2/2017	A / H5N8	Europe / Italy / Udine	Bianca Zecchin (Istituto Zooprofilattico Sperimentale Delle Venezie)
EPI_ISL_255182	A/turkey/Italy/17VIR538-1/2017	A / H5N8	Europe / Italy / Venezia	Bianca Zecchin (Istituto Zooprofilattico Sperimentale Delle Venezie)
EPI_ISL_255183	A/turkey/Italy/17VIR576-11/2017	A / H5N8	Europe / Italy / Padova	Bianca Zecchin (Istituto Zooprofilattico Sperimentale Delle Venezie)
EPI_ISL_255184	A/chicken/Italy/17VIR653-12/2017	A / H5N8	Europe / Italy / Rovigo	Bianca Zecchin (Istituto Zooprofilattico Sperimentale Delle Venezie)
EPI_ISL_255185	A/turkey/Italy/17VIR973-2/2017	A / H5N8	Europe / Italy / Parma	Bianca Zecchin (Istituto Zooprofilattico Sperimentale Delle Venezie)
EPI_ISL_255186	A/turkey/Italy/17VIR1338-3/2017	A / H5N8	Europe / Italy / Mantova	Bianca Zecchin (Istituto Zooprofilattico Sperimentale Delle Venezie)
EPI_ISL_255187	A/turkey/Italy/17VIR1452-22/2017	A / H5N8	Europe / Italy / Verona	Bianca Zecchin (Istituto Zooprofilattico Sperimentale Delle Venezie)

EPI_ISL_255189	A/gadwall/Italy/17VIR133-2/2017	A / H5N5	Europe / Italy / Gorizia	Bianca Zecchin (Istituto Zooprofilattico Sperimentale Delle Venezie)
EPI_ISL_255190	A/Mute swan/Hungary/119/2017	A / H5N8	Europe / Hungary / Somogy megye / Somogy	Adam Dan (Danam.Vet.Molbiol)
EPI_ISL_255191	A/Harris Hawk/Hungary/120/2017	A / H5N8	Europe / Hungary / Budapest fovaros / Budapest	Adam Dan (Danam.Vet.Molbiol)
EPI_ISL_255192	A/Goose/Hungary/982/2017	A / H5N8	Europe / Hungary / Jasz-Nagykun-Szolnok	Adam Dan (Danam.Vet.Molbiol)
EPI_ISL_255193	A/Duck/Hungary/984/2017	A / H5N8	Europe / Hungary / Bekes megye	Adam Dan (Danam.Vet.Molbiol)
EPI_ISL_255194	A/Goose/Hungary/1030/2017	A / H5N8	Europe / Hungary / Csongrad megye	Adam Dan (Danam.Vet.Molbiol)
EPI_ISL_255195	A/Duck/Hungary/1588/2017	A / H5N8	Europe / Hungary / Bacs-Kiskun	Adam Dan (Danam.Vet.Molbiol)
EPI_ISL_255196	A/Greylag_goose/Hungary/1941/2017	A / H5N8	Europe / Hungary / Fejer megye	Adam Dan (Danam.Vet.Molbiol)
EPI_ISL_255197	A/Mute swan/Hungary/1955/2017	A / H5N8	Europe / Hungary / Veszprem megye	Adam Dan (Danam.Vet.Molbiol)
EPI_ISL_255198	A/Turkey/Hungary/2030/2017	A / H5N8	Europe / Hungary / Veszprem megye	Adam Dan (Danam.Vet.Molbiol)
EPI_ISL_255199	A/Mute swan/Hungary/2193/2017	A / H5N8	Europe / Hungary / Borsod-Abauj-Zemplen	Adam Dan (Danam.Vet.Molbiol)
EPI_ISL_255200	A/Mute swan/Hungary/2508/2017	A / H5N8	Europe / Hungary / Somogy megye / Somogy	Adam Dan (Danam.Vet.Molbiol)
EPI_ISL_255201	A/Mute swan/Hungary/2825/2017	A / H5N8	Europe / Hungary / Tolna megye / Tolna	Adam Dan (Danam.Vet.Molbiol)
EPI_ISL_255202	A/Mute swan/Hungary/3139/2017	A / H5N8	Europe / Hungary / Zala	Adam Dan (Danam.Vet.Molbiol)
EPI_ISL_255203	A/Mute swan/Hungary/3513/2017	A / H5N8	Europe / Hungary / Vas	Adam Dan (Danam.Vet.Molbiol)
EPI_ISL_255204	A/Mute swan/Hungary/3542/2017	A / H5N8	Europe / Hungary / Budapest fovaros	Adam Dan (Danam.Vet.Molbiol)
EPI_ISL_255205	A/Chicken/Hungary/2496/2017	A / H5N8	Europe / Hungary / Jasz-Nagykun-Szolnok	Adam Dan (Danam.Vet.Molbiol)
EPI_ISL_255206	A/Peregrine_falcon/Hungary/4882/2017	A / H5N8	Europe / Hungary / Gyor-Moson-Sopron	Adam Dan (Danam.Vet.Molbiol)
EPI_ISL_255207	A/Mute swan/Hungary/5316/2017	A / H5N8	Europe / Hungary / Tolna megye / Tolna	Adam Dan (Danam.Vet.Molbiol)
EPI_ISL_255208	A/Common_buzzard/Hungary/7061/2017	A / H5N8	Europe / Hungary / Tolna megye / Tolna	Adam Dan (Danam.Vet.Molbiol)
EPI_ISL_255209	A/Common_tern/Hungary/8187/2017	A / H5N8	Europe / Hungary / Tolna megye	Adam Dan (Danam.Vet.Molbiol)
EPI_ISL_255210	A/Harris_hawk/Hungary/2762a/2017	A / H5N8	Europe / Hungary / Pest megye / Pest	Adam Dan (Danam.Vet.Molbiol)
EPI_ISL_255211	A/Harris_hawk/Hungary/2762b/2017	A / H5N8	Europe / Hungary / Pest megye / Pest	Adam Dan (Danam.Vet.Molbiol)
EPI_ISL_255212	A/Greylag_goose/Hungary/320/2017	A / H5N8	Europe / Hungary / Somogy megye / Somogy	Adam Dan (Danam.Vet.Molbiol)
EPI_ISL_255213	A/GuineaFowl/Hungary/596/2017	A / H5N8	Europe / Hungary / Hajdu-Bihar	Adam Dan (Danam.Vet.Molbiol)
EPI_ISL_255214	A/Chicken/Hungary/1751/2017	A / H5N8	Europe / Hungary / Gyor-Moson-Sopron	Adam Dan (Danam.Vet.Molbiol)
EPI_ISL_255215	A/Mute swan/Hungary/3137/2017	A / H5N8	Europe / Hungary / Zala	Adam Dan (Danam.Vet.Molbiol)
EPI_ISL_255216	A/Mallard/Hungary/5821/2017	A / H5N8	Europe / Hungary / Zala	Adam Dan (Danam.Vet.Molbiol)
EPI_ISL_255218	A/Mallard/Hungary/1574b/2017	A / H5N8	Europe / Hungary / Somogy megye / Somogy	Adam Dan (Danam.Vet.Molbiol)
EPI_ISL_255219	A/White_fronted_goose/Hungary/801/2017	A / H5N8	Europe / Hungary / Hajdu-Bihar	Adam Dan (Danam.Vet.Molbiol)
EPI_ISL_255220	A/Mallard/Hungary/1574a/2017	A / H5N8	Europe / Hungary / Somogy megye / Somogy	Adam Dan (Danam.Vet.Molbiol)
EPI_ISL_255388	A/goose/Hunan/161/2014	A / H5N6	Asia / China	Jie Cui (Wuhan Institute of Virology, CAS.)
EPI_ISL_255389	A/chicken/Anhui/AH325/2015	A / H5N6	Asia / China	Jie Cui (Wuhan Institute of Virology, CAS.)
EPI_ISL_255398	A/Goose/Hungary/59712/2016	A / H5N8	Europe / Hungary / Csongrad megye / Csongrad	Adam Dan (Danam.Vet.Molbiol)
EPI_ISL_255412	A/chicken/Hunan/HN158/2015	A / H5N6	Asia / China	Jie Cui (Wuhan Institute of Virology, CAS.)
EPI_ISL_255413	A/chicken/Hunan/HN244/2015	A / H5N6	Asia / China	Jie Cui (Wuhan Institute of Virology, CAS.)
EPI_ISL_255414	A/chicken/Hunan/HN245/2015	A / H5N6	Asia / China	Jie Cui (Wuhan Institute of Virology, CAS.)
EPI_ISL_255415	A/chicken/Hunan/HN247/2015	A / H5N6	Asia / China	Jie Cui (Wuhan Institute of Virology, CAS.)
EPI_ISL_255416	A/chicken/Hunan/HN248/2015	A / H5N6	Asia / China	Jie Cui (Wuhan Institute of Virology, CAS.)
EPI_ISL_255417	A/chicken/Hunan/HN249/2015	A / H5N6	Asia / China	Jie Cui (Wuhan Institute of Virology, CAS.)
EPI_ISL_255418	A/chicken/Hunan/HN250/2015	A / H5N6	Asia / China	Jie Cui (Wuhan Institute of Virology, CAS.)
EPI_ISL_255419	A/chicken/Hunan/HN251/2015	A / H5N6	Asia / China	Jie Cui (Wuhan Institute of Virology, CAS.)
EPI_ISL_255420	A/chicken/Hunan/HN252/2015	A / H5N6	Asia / China	Jie Cui (Wuhan Institute of Virology, CAS.)
EPI_ISL_255421	A/chicken/Hunan/HN254/2015	A / H5N6	Asia / China	Jie Cui (Wuhan Institute of Virology, CAS.)
EPI_ISL_255422	A/chicken/Hunan/HN263/2015	A / H5N6	Asia / China	Jie Cui (Wuhan Institute of Virology, CAS.)
EPI_ISL_255423	A/chicken/Hunan/HN255/2015	A / H5N6	Asia / China	Jie Cui (Wuhan Institute of Virology, CAS.)
EPI_ISL_255424	A/chicken/Hunan/HN264/2015	A / H5N6	Asia / China	Jie Cui (Wuhan Institute of Virology, CAS.)
EPI_ISL_255425	A/chicken/Hunan/HN265/2015	A / H5N6	Asia / China	Jie Cui (Wuhan Institute of Virology, CAS.)
EPI_ISL_255426	A/chicken/Hunan/HN266/2015	A / H5N6	Asia / China	Jie Cui (Wuhan Institute of Virology, CAS.)
EPI_ISL_255427	A/chicken/Hunan/HN301/2015	A / H5N6	Asia / China	Jie Cui (Wuhan Institute of Virology, CAS.)
EPI_ISL_255428	A/chicken/Hunan/HN302/2015	A / H5N6	Asia / China	Jie Cui (Wuhan Institute of Virology, CAS.)
EPI_ISL_255429	A/chicken/Hunan/HN303/2015	A / H5N6	Asia / China	Jie Cui (Wuhan Institute of Virology, CAS.)
EPI_ISL_255430	A/chicken/Hunan/HN304/2015	A / H5N6	Asia / China	Jie Cui (Wuhan Institute of Virology, CAS.)
EPI_ISL_255433	A/chicken/Hunan/HN305/2015	A / H5N6	Asia / China	Jie Cui (Wuhan Institute of Virology, CAS.)
EPI_ISL_255466	A/chicken/Hunan/HN306/2015	A / H5N6	Asia / China	Jie Cui (Wuhan Institute of Virology, CAS.)
EPI_ISL_255467	A/chicken/Hunan/HN307/2015	A / H5N6	Asia / China	Jie Cui (Wuhan Institute of Virology, CAS.)
EPI_ISL_255468	A/chicken/Hunan/HN308/2015	A / H5N6	Asia / China	Jie Cui (Wuhan Institute of Virology, CAS.)
EPI_ISL_255469	A/chicken/Hunan/HN40/2015	A / H5N6	Asia / China	Jie Cui (Wuhan Institute of Virology, CAS.)
EPI_ISL_255470	A/chicken/Hunan/HN58/2015	A / H5N6	Asia / China	Jie Cui (Wuhan Institute of Virology, CAS.)
EPI_ISL_255489	A/chicken/Hunan/HN75/2015	A / H5N6	Asia / China	Jie Cui (Wuhan Institute of Virology, CAS.)
EPI_ISL_255491	A/duck/Anhui/AH328/2015	A / H5N6	Asia / China	Jie Cui (Wuhan Institute of Virology, CAS.)
EPI_ISL_255492	A/duck/Hunan/193/2014	A / H5N6	Asia / China	Jie Cui (Wuhan Institute of Virology, CAS.)
EPI_ISL_255493	A/duck/Hunan/233/2014	A / H5N6	Asia / China	Jie Cui (Wuhan Institute of Virology, CAS.)
EPI_ISL_255509	A/duck/Hunan/HN05/2014	A / H5N6	Asia / China	Jie Cui (Wuhan Institute of Virology, CAS.)
EPI_ISL_255529	A/duck/Hunan/HN13/2015	A / H5N6	Asia / China	Jie Cui (Wuhan Institute of Virology, CAS.)
EPI_ISL_255530	A/duck/Hunan/HN16/2015	A / H5N6	Asia / China	Jie Cui (Wuhan Institute of Virology, CAS.)
EPI_ISL_255534	A/duck/Hunan/HN162/2015	A / H5N6	Asia / China	Jie Cui (Wuhan Institute of Virology, CAS.)
EPI_ISL_2555532	A/Cygnus columbianus/Hubei/116/2020	A / H5N8	Asia / China / Jiangxi Province / Longgan Lake National Nature Reserve	Ma Liping (Wuhan Institute of Virology, CAS)
EPI_ISL_2555534	A/Cygnus columbianus/Hubei/56/2020	A / H5N8	Asia / China / Jiangxi Province	Ma Liping (Wuhan Institute of Virology, CAS)
EPI_ISL_2555536	A/Chlidonias hybrida/Hubei/55/2020	A / H5N8	Asia / China / Jiangxi Province	Ma Liping (Wuhan Institute of Virology, CAS)
EPI_ISL_2555538	A/Cygnus columbianus/Hubei/53/2020	A / H5N8	Asia / China / Jiangxi Province	Ma Liping (Wuhan Institute of Virology, CAS)
EPI_ISL_2555541	A/Cygnus columbianus/Hubei/52/2020	A / H5N8	Asia / China / Jiangxi Province	Ma Liping (Wuhan Institute of Virology, CAS)

EPI_ISL_2555542	A/Cygnus columbianus/Hubei/51/2020	A / H5N8	Asia / China / Jiangxi Province	Ma Liping (Wuhan Institute of Virology, CAS)
EPI_ISL_2555543	A/Cygnus columbianus/Hubei/50/2020	A / H5N8	Asia / China / Jiangxi Province	Ma Liping (Wuhan Institute of Virology, CAS)
EPI_ISL_255559	A/duck/Hunan/HN163/2015	A / H5N6	Asia / China	Jie Cui (Wuhan Institute of Virology, CAS.)
EPI_ISL_255561	A/duck/Hunan/HN167/2015	A / H5N6	Asia / China	Jie Cui (Wuhan Institute of Virology, CAS.)
EPI_ISL_255562	A/duck/Hunan/HN171/2015	A / H5N6	Asia / China	Jie Cui (Wuhan Institute of Virology, CAS.)
EPI_ISL_255563	A/duck/Hunan/HN172/2015	A / H5N6	Asia / China	Jie Cui (Wuhan Institute of Virology, CAS.)
EPI_ISL_255564	A/duck/Hunan/HN174/2015	A / H5N6	Asia / China	Jie Cui (Wuhan Institute of Virology, CAS.)
EPI_ISL_255587	A/duck/Hunan/HN176/2015	A / H5N6	Asia / China	Jie Cui (Wuhan Institute of Virology, CAS.)
EPI_ISL_255592	A/duck/Hunan/HN177/2015	A / H5N6	Asia / China	Jie Cui (Wuhan Institute of Virology, CAS.)
EPI_ISL_255597	A/duck/Hunan/HN178/2015	A / H5N6	Asia / China	Jie Cui (Wuhan Institute of Virology, CAS.)
EPI_ISL_255599	A/duck/Hunan/HN182/2015	A / H5N6	Asia / China	Jie Cui (Wuhan Institute of Virology, CAS.)
EPI_ISL_255600	A/duck/Hunan/HN183/2015	A / H5N6	Asia / China	Jie Cui (Wuhan Institute of Virology, CAS.)
EPI_ISL_255603	A/duck/Hunan/HN186/2015	A / H5N6	Asia / China	Jie Cui (Wuhan Institute of Virology, CAS.)
EPI_ISL_255605	A/duck/Hunan/HN190/2015	A / H5N6	Asia / China	Jie Cui (Wuhan Institute of Virology, CAS.)
EPI_ISL_255607	A/duck/Hunan/HN191/2015	A / H5N6	Asia / China	Jie Cui (Wuhan Institute of Virology, CAS.)
EPI_ISL_255609	A/duck/Hunan/HN192/2015	A / H5N6	Asia / China	Jie Cui (Wuhan Institute of Virology, CAS.)
EPI_ISL_255610	A/duck/Hunan/HN194/2015	A / H5N6	Asia / China	Jie Cui (Wuhan Institute of Virology, CAS.)
EPI_ISL_2556113	A/Cygnus columbianus/Hubei/49/2020	A / H5N8	Asia / China / Jiangxi Province	Ma Liping (Wuhan Institute of Virology, CAS)
EPI_ISL_255612	A/duck/Hunan/HN195/2015	A / H5N6	Asia / China	Jie Cui (Wuhan Institute of Virology, CAS.)
EPI_ISL_255613	A/duck/Hunan/HN197/2015	A / H5N6	Asia / China	Jie Cui (Wuhan Institute of Virology, CAS.)
EPI_ISL_255614	A/duck/Hunan/HN201/2015	A / H5N6	Asia / China	Jie Cui (Wuhan Institute of Virology, CAS.)
EPI_ISL_255615	A/duck/Hunan/HN204/2015	A / H5N6	Asia / China	Jie Cui (Wuhan Institute of Virology, CAS.)
EPI_ISL_255616	A/duck/Hunan/HN205/2015	A / H5N6	Asia / China	Jie Cui (Wuhan Institute of Virology, CAS.)
EPI_ISL_255617	A/duck/Hunan/HN206/2015	A / H5N6	Asia / China	Jie Cui (Wuhan Institute of Virology, CAS.)
EPI_ISL_255618	A/duck/Hunan/HN207/2015	A / H5N6	Asia / China	Jie Cui (Wuhan Institute of Virology, CAS.)
EPI_ISL_255619	A/duck/Hunan/HN208/2015	A / H5N6	Asia / China	Jie Cui (Wuhan Institute of Virology, CAS.)
EPI_ISL_255620	A/duck/Hunan/HN209/2015	A / H5N6	Asia / China	Jie Cui (Wuhan Institute of Virology, CAS.)
EPI_ISL_255621	A/duck/Hunan/HN210/2015	A / H5N6	Asia / China	Jie Cui (Wuhan Institute of Virology, CAS.)
EPI_ISL_255624	A/duck/Hunan/HN212/2015	A / H5N6	Asia / China	Jie Cui (Wuhan Institute of Virology, CAS.)
EPI_ISL_255627	A/duck/France/161587/2016	A / H5N8	Europe / France / Aquitaine / Departement des Landes	Francois-Xavier Briand (ANSES Agence Nationale De Securite Sanitaire De L'alimentation / Laboratoire de Ploufragan-Plouzané)
EPI_ISL_255689	A/duck/Hunan/HN214/2015	A / H5N6	Asia / China	Jie Cui (Wuhan Institute of Virology, CAS.)
EPI_ISL_255691	A/duck/Hunan/HN215/2015	A / H5N6	Asia / China	Jie Cui (Wuhan Institute of Virology, CAS.)
EPI_ISL_255692	A/duck/Hunan/HN216/2015	A / H5N6	Asia / China	Jie Cui (Wuhan Institute of Virology, CAS.)
EPI_ISL_255694	A/duck/Hunan/HN217/2015	A / H5N6	Asia / China	Jie Cui (Wuhan Institute of Virology, CAS.)
EPI_ISL_255695	A/duck/Hunan/HN220/2015	A / H5N6	Asia / China	Jie Cui (Wuhan Institute of Virology, CAS.)
EPI_ISL_255698	A/duck/Hunan/HN221/2015	A / H5N6	Asia / China	Jie Cui (Wuhan Institute of Virology, CAS.)
EPI_ISL_255699	A/duck/Hunan/HN224/2015	A / H5N6	Asia / China	Jie Cui (Wuhan Institute of Virology, CAS.)
EPI_ISL_255701	A/duck/Hunan/HN227/2015	A / H5N6	Asia / China	Jie Cui (Wuhan Institute of Virology, CAS.)
EPI_ISL_255702	A/duck/Hunan/HN230/2015	A / H5N6	Asia / China	Jie Cui (Wuhan Institute of Virology, CAS.)
EPI_ISL_255738	A/duck/Hunan/HN236/2015	A / H5N6	Asia / China	Jie Cui (Wuhan Institute of Virology, CAS.)
EPI_ISL_255741	A/duck/Hunan/HN238/2015	A / H5N6	Asia / China	Jie Cui (Wuhan Institute of Virology, CAS.)
EPI_ISL_255742	A/duck/Hunan/HN239/2015	A / H5N6	Asia / China	Jie Cui (Wuhan Institute of Virology, CAS.)
EPI_ISL_255743	A/duck/Hunan/HN240/2015	A / H5N6	Asia / China	Jie Cui (Wuhan Institute of Virology, CAS.)
EPI_ISL_255744	A/duck/Hunan/HN241/2015	A / H5N6	Asia / China	Jie Cui (Wuhan Institute of Virology, CAS.)
EPI_ISL_255746	A/duck/Hunan/HN242/2015	A / H5N6	Asia / China	Jie Cui (Wuhan Institute of Virology, CAS.)
EPI_ISL_255747	A/duck/Hunan/HN271/2015	A / H5N6	Asia / China	Jie Cui (Wuhan Institute of Virology, CAS.)
EPI_ISL_255749	A/duck/Hunan/HN277/2015	A / H5N6	Asia / China	Jie Cui (Wuhan Institute of Virology, CAS.)
EPI_ISL_255751	A/duck/Hunan/HN278/2015	A / H5N6	Asia / China	Jie Cui (Wuhan Institute of Virology, CAS.)
EPI_ISL_255752	A/duck/Hunan/HN285/2015	A / H5N6	Asia / China	Jie Cui (Wuhan Institute of Virology, CAS.)
EPI_ISL_255754	A/duck/Hunan/HN288/2015	A / H5N6	Asia / China	Jie Cui (Wuhan Institute of Virology, CAS.)
EPI_ISL_255755	A/duck/Hunan/HN29/2015	A / H5N6	Asia / China	Jie Cui (Wuhan Institute of Virology, CAS.)
EPI_ISL_255756	A/duck/Hunan/HN333/2015	A / H5N6	Asia / China	Jie Cui (Wuhan Institute of Virology, CAS.)
EPI_ISL_255757	A/duck/Hunan/HN334/2015	A / H5N6	Asia / China	Jie Cui (Wuhan Institute of Virology, CAS.)
EPI_ISL_255758	A/duck/Hunan/HN335/2015	A / H5N6	Asia / China	Jie Cui (Wuhan Institute of Virology, CAS.)
EPI_ISL_255759	A/duck/Hunan/HN336/2015	A / H5N6	Asia / China	Jie Cui (Wuhan Institute of Virology, CAS.)
EPI_ISL_255760	A/duck/Hunan/HN339/2015	A / H5N6	Asia / China	Jie Cui (Wuhan Institute of Virology, CAS.)
EPI_ISL_255761	A/duck/Hunan/HN341/2015	A / H5N6	Asia / China	Jie Cui (Wuhan Institute of Virology, CAS.)
EPI_ISL_255762	A/duck/Hunan/HN342/2015	A / H5N6	Asia / China	Jie Cui (Wuhan Institute of Virology, CAS.)
EPI_ISL_255763	A/duck/Hunan/HN343/2015	A / H5N6	Asia / China	Jie Cui (Wuhan Institute of Virology, CAS.)
EPI_ISL_255764	A/duck/Hunan/HN42/2015	A / H5N6	Asia / China	Jie Cui (Wuhan Institute of Virology, CAS.)
EPI_ISL_255765	A/duck/Hunan/HN45/2015	A / H5N6	Asia / China	Jie Cui (Wuhan Institute of Virology, CAS.)
EPI_ISL_255766	A/duck/Hunan/HN53/2015	A / H5N6	Asia / China	Jie Cui (Wuhan Institute of Virology, CAS.)
EPI_ISL_255768	A/duck/Hunan/HN54/2015	A / H5N6	Asia / China	Jie Cui (Wuhan Institute of Virology, CAS.)
EPI_ISL_255769	A/duck/Hunan/HN59/2015	A / H5N6	Asia / China	Jie Cui (Wuhan Institute of Virology, CAS.)
EPI_ISL_255770	A/duck/Hunan/HN60/2015	A / H5N6	Asia / China	Jie Cui (Wuhan Institute of Virology, CAS.)
EPI_ISL_255771	A/duck/Hunan/HN61/2015	A / H5N6	Asia / China	Jie Cui (Wuhan Institute of Virology, CAS.)
EPI_ISL_255772	A/duck/Hunan/HN64/2015	A / H5N6	Asia / China	Jie Cui (Wuhan Institute of Virology, CAS.)
EPI_ISL_255773	A/duck/Hunan/HN66/2015	A / H5N6	Asia / China	Jie Cui (Wuhan Institute of Virology, CAS.)
EPI_ISL_255774	A/duck/Hunan/HN67/2015	A / H5N6	Asia / China	Jie Cui (Wuhan Institute of Virology, CAS.)
EPI_ISL_255775	A/duck/Hunan/HN71/2015	A / H5N6	Asia / China	Jie Cui (Wuhan Institute of Virology, CAS.)
EPI_ISL_255816	A/duck/Hunan/HN72/2015	A / H5N6	Asia / China	Jie Cui (Wuhan Institute of Virology, CAS.)
EPI_ISL_255817	A/duck/Hunan/HN74/2015	A / H5N6	Asia / China	Jie Cui (Wuhan Institute of Virology, CAS.)

EPI_ISL_255818	A/duck/Hunan/HN76/2015	A / H5N6	Asia / China	Jie Cui (Wuhan Institute of Virology, CAS.)
EPI_ISL_255819	A/duck/Hunan/HN78/2015	A / H5N6	Asia / China	Jie Cui (Wuhan Institute of Virology, CAS.)
EPI_ISL_255820	A/duck/Hunan/HN80/2015	A / H5N6	Asia / China	Jie Cui (Wuhan Institute of Virology, CAS.)
EPI_ISL_255821	A/environment/Hunan/HN261/2015	A / H5N6	Asia / China	Jie Cui (Wuhan Institute of Virology, CAS.)
EPI_ISL_255822	A/goose/Anhui/AH350/2015	A / H5N6	Asia / China	Jie Cui (Wuhan Institute of Virology, CAS.)
EPI_ISL_255823	A/goose/Hubei/113/2014	A / H5N6	Asia / China	Jie Cui (Wuhan Institute of Virology, CAS.)
EPI_ISL_255824	A/goose/HuBei/117/2014	A / H5N6	Asia / China	Jie Cui (Wuhan Institute of Virology, CAS.)
EPI_ISL_255825	A/goose/Hunan/107/2014	A / H5N6	Asia / China	Jie Cui (Wuhan Institute of Virology, CAS.)
EPI_ISL_255826	A/goose/Hunan/109/2014	A / H5N6	Asia / China	Jie Cui (Wuhan Institute of Virology, CAS.)
EPI_ISL_255827	A/goose/Hunan/110/2014	A / H5N6	Asia / China	Jie Cui (Wuhan Institute of Virology, CAS.)
EPI_ISL_255828	A/goose/Hunan/111/2014	A / H5N6	Asia / China	Jie Cui (Wuhan Institute of Virology, CAS.)
EPI_ISL_255829	A/goose/Hunan/115/2014	A / H5N6	Asia / China	Jie Cui (Wuhan Institute of Virology, CAS.)
EPI_ISL_255830	A/goose/Hunan/116/2014	A / H5N6	Asia / China	Jie Cui (Wuhan Institute of Virology, CAS.)
EPI_ISL_255831	A/goose/Hunan/155/2014	A / H5N6	Asia / China	Jie Cui (Wuhan Institute of Virology, CAS.)
EPI_ISL_255832	A/goose/Hunan/159/2014	A / H5N6	Asia / China	Jie Cui (Wuhan Institute of Virology, CAS.)
EPI_ISL_255833	A/goose/Hunan/160/2014	A / H5N6	Asia / China	Jie Cui (Wuhan Institute of Virology, CAS.)
EPI_ISL_255834	A/goose/Hunan/81/2014	A / H5N6	Asia / China	Jie Cui (Wuhan Institute of Virology, CAS.)
EPI_ISL_255835	A/goose/Hunan/82/2014	A / H5N6	Asia / China	Jie Cui (Wuhan Institute of Virology, CAS.)
EPI_ISL_255836	A/goose/Hunan/HN11/2015	A / H5N6	Asia / China	Jie Cui (Wuhan Institute of Virology, CAS.)
EPI_ISL_255837	A/goose/Hunan/HN22/2015	A / H5N6	Asia / China	Jie Cui (Wuhan Institute of Virology, CAS.)
EPI_ISL_255838	A/goose/Hunan/HN310/2015	A / H5N6	Asia / China	Jie Cui (Wuhan Institute of Virology, CAS.)
EPI_ISL_255839	A/goose/Hunan/HN311/2015	A / H5N6	Asia / China	Jie Cui (Wuhan Institute of Virology, CAS.)
EPI_ISL_255840	A/goose/Hunan/HN314/2015	A / H5N6	Asia / China	Jie Cui (Wuhan Institute of Virology, CAS.)
EPI_ISL_255841	A/goose/Hunan/HN315/2015	A / H5N6	Asia / China	Jie Cui (Wuhan Institute of Virology, CAS.)
EPI_ISL_255842	A/goose/Hunan/HN316/2015	A / H5N6	Asia / China	Jie Cui (Wuhan Institute of Virology, CAS.)
EPI_ISL_255843	A/goose/Hunan/HN317/2015	A / H5N6	Asia / China	Jie Cui (Wuhan Institute of Virology, CAS.)
EPI_ISL_255844	A/goose/Hunan/HN318/2015	A / H5N6	Asia / China	Jie Cui (Wuhan Institute of Virology, CAS.)
EPI_ISL_255845	A/goose/Hunan/HN319/2015	A / H5N6	Asia / China	Jie Cui (Wuhan Institute of Virology, CAS.)
EPI_ISL_255846	A/goose/Hunan/HN325/2015	A / H5N6	Asia / China	Jie Cui (Wuhan Institute of Virology, CAS.)
EPI_ISL_255847	A/goose/Hunan/HN57/2015	A / H5N6	Asia / China	Jie Cui (Wuhan Institute of Virology, CAS.)
EPI_ISL_255848	A/goose/Hunan/HN88/2015	A / H5N6	Asia / China	Jie Cui (Wuhan Institute of Virology, CAS.)
EPI_ISL_255849	A/goose/Hunan/HN90/2015	A / H5N6	Asia / China	Jie Cui (Wuhan Institute of Virology, CAS.)
EPI_ISL_255850	A/goose/SiChuan/15/2015	A / H5N6	Asia / China	Jie Cui (Wuhan Institute of Virology, CAS.)
EPI_ISL_255851	A/muscovy/duck/Hunan/101/2014	A / H5N6	Asia / China	Jie Cui (Wuhan Institute of Virology, CAS.)
EPI_ISL_255852	A/muscovy/duck/Hunan/102/2014	A / H5N6	Asia / China	Jie Cui (Wuhan Institute of Virology, CAS.)
EPI_ISL_255853	A/muscovy/duck/Hunan/103/2014	A / H5N6	Asia / China	Jie Cui (Wuhan Institute of Virology, CAS.)
EPI_ISL_255854	A/muscovy/duck/Hunan/104/2014	A / H5N6	Asia / China	Jie Cui (Wuhan Institute of Virology, CAS.)
EPI_ISL_255857	A/muscovy/duck/Hunan/125/2014	A / H5N6	Asia / China	Jie Cui (Wuhan Institute of Virology, CAS.)
EPI_ISL_255858	A/muscovy/duck/Hunan/147/2014	A / H5N6	Asia / China	Jie Cui (Wuhan Institute of Virology, CAS.)
EPI_ISL_255859	A/muscovy/duck/Hunan/148/2014	A / H5N6	Asia / China	Jie Cui (Wuhan Institute of Virology, CAS.)
EPI_ISL_255860	A/muscovy/duck/Hunan/167/2014	A / H5N6	Asia / China	Jie Cui (Wuhan Institute of Virology, CAS.)
EPI_ISL_255861	A/muscovy/duck/Hunan/183/2014	A / H5N6	Asia / China	Jie Cui (Wuhan Institute of Virology, CAS.)
EPI_ISL_255862	A/muscovy/duck/Hunan/202/2014	A / H5N6	Asia / China	Jie Cui (Wuhan Institute of Virology, CAS.)
EPI_ISL_255863	A/muscovy/duck/Hunan/216/2014	A / H5N6	Asia / China	Jie Cui (Wuhan Institute of Virology, CAS.)
EPI_ISL_255864	A/muscovy/duck/Hunan/229/2014	A / H5N6	Asia / China	Jie Cui (Wuhan Institute of Virology, CAS.)
EPI_ISL_255865	A/muscovy/duck/Hunan/232/2014	A / H5N6	Asia / China	Jie Cui (Wuhan Institute of Virology, CAS.)
EPI_ISL_255866	A/muscovy/duck/Hunan/85/2014	A / H5N6	Asia / China	Jie Cui (Wuhan Institute of Virology, CAS.)
EPI_ISL_255867	A/muscovy/duck/Hunan/88/2014	A / H5N6	Asia / China	Jie Cui (Wuhan Institute of Virology, CAS.)
EPI_ISL_255868	A/muscovy/duck/Hunan/89/2014	A / H5N6	Asia / China	Jie Cui (Wuhan Institute of Virology, CAS.)
EPI_ISL_255869	A/muscovy/duck/Hunan/90/2014	A / H5N6	Asia / China	Jie Cui (Wuhan Institute of Virology, CAS.)
EPI_ISL_255870	A/muscovy/duck/Hunan/93/2014	A / H5N6	Asia / China	Jie Cui (Wuhan Institute of Virology, CAS.)
EPI_ISL_255871	A/muscovy/duck/Hunan/94/2014	A / H5N6	Asia / China	Jie Cui (Wuhan Institute of Virology, CAS.)
EPI_ISL_255872	A/muscovy/duck/Hunan/HN346/2015	A / H5N6	Asia / China	Jie Cui (Wuhan Institute of Virology, CAS.)
EPI_ISL_255873	A/muscovy/duck/Hunan/HN347/2015	A / H5N6	Asia / China	Jie Cui (Wuhan Institute of Virology, CAS.)
EPI_ISL_255874	A/muscovy/duck/Hunan/HN348/2015	A / H5N6	Asia / China	Jie Cui (Wuhan Institute of Virology, CAS.)
EPI_ISL_255875	A/muscovy/duck/Hunan/HN349/2015	A / H5N6	Asia / China	Jie Cui (Wuhan Institute of Virology, CAS.)
EPI_ISL_255876	A/muscovy/duck/Hunan/HN350/2015	A / H5N6	Asia / China	Jie Cui (Wuhan Institute of Virology, CAS.)
EPI_ISL_255877	A/muscovy/duck/Hunan/HN351/2015	A / H5N6	Asia / China	Jie Cui (Wuhan Institute of Virology, CAS.)
EPI_ISL_255878	A/muscovy/duck/Hunan/HN352/2015	A / H5N6	Asia / China	Jie Cui (Wuhan Institute of Virology, CAS.)
EPI_ISL_255879	A/muscovy/duck/Hunan/HN353/2015	A / H5N6	Asia / China	Jie Cui (Wuhan Institute of Virology, CAS.)
EPI_ISL_255880	A/muscovy/duck/Hunan/HN354/2015	A / H5N6	Asia / China	Jie Cui (Wuhan Institute of Virology, CAS.)
EPI_ISL_255881	A/muscovy/duck/Hunan/HN355/2015	A / H5N6	Asia / China	Jie Cui (Wuhan Institute of Virology, CAS.)
EPI_ISL_255882	A/muscovy/duck/Hunan/HN356/2015	A / H5N6	Asia / China	Jie Cui (Wuhan Institute of Virology, CAS.)
EPI_ISL_255883	A/muscovy/duck/Hunan/HN358/2015	A / H5N6	Asia / China	Jie Cui (Wuhan Institute of Virology, CAS.)
EPI_ISL_255884	A/muscovy/duck/Hunan/HN359/2015	A / H5N6	Asia / China	Jie Cui (Wuhan Institute of Virology, CAS.)
EPI_ISL_255885	A/muscovy/duck/Hunan/HN360/2015	A / H5N6	Asia / China	Jie Cui (Wuhan Institute of Virology, CAS.)
EPI_ISL_255886	A/muscovy/duck/Hunan/HN364/2015	A / H5N6	Asia / China	Jie Cui (Wuhan Institute of Virology, CAS.)
EPI_ISL_255887	A/muscovy/duck/Hunan/HN365/2015	A / H5N6	Asia / China	Jie Cui (Wuhan Institute of Virology, CAS.)
EPI_ISL_255888	A/muscovy/duck/Hunan/HN366/2015	A / H5N6	Asia / China	Jie Cui (Wuhan Institute of Virology, CAS.)
EPI_ISL_255889	A/muscovy/duck/Hunan/HN368/2015	A / H5N6	Asia / China	Jie Cui (Wuhan Institute of Virology, CAS.)
EPI_ISL_255891	A/Tufted Duck/Netherlands/1/2016	A / H5N8	Europe / Netherlands / Provincie Noord-Holland / Edam-Volendam	Maria Johanna Poen (Erasmus Medical Center / Department of Virology)
EPI_ISL_255892	A/Great Black-backed Gull/Netherlands/2/2016	A / H5N8	Europe / Netherlands / Provincie Noord-Holland	Maria Johanna Poen (Erasmus Medical Center / Department of Virology)

EPI_ISL_255910	A/Mew Gull/Netherlands/1/2016	A / H5N8	Europe / Netherlands / Provincie Noord-Holland	Maria Johanna Poen (Erasmus Medical Center / Department of Virology)
EPI_ISL_255911	A/Eurasian Wigeon/Netherlands/2/2016	A / H5N8	Europe / Netherlands / Provincie Noord-Holland	Maria Johanna Poen (Erasmus Medical Center / Department of Virology)
EPI_ISL_255912	A/Eurasian Wigeon/Netherlands/4/2016	A / H5N8	Europe / Netherlands / South Holland	Maria Johanna Poen (Erasmus Medical Center / Department of Virology)
EPI_ISL_255913	A/Mallard/Netherlands/2/2017	A / H5N8	Europe / Netherlands / South Holland	Maria Johanna Poen (Erasmus Medical Center / Department of Virology)
EPI_ISL_255914	A/Eurasian Wigeon/Netherlands/9/2016	A / H5N8	Europe / Netherlands / Provincie Noord-Holland	Maria Johanna Poen (Erasmus Medical Center / Department of Virology)
EPI_ISL_255915	A/chicken/Poland/114/2016	A / H5N8	Europe / Poland / Lesser Poland Voivodeship	Edyta ?wi?to? (National Veterinary Research Institut Poland, PIWet-PIB)
EPI_ISL_255916	A/chicken/Poland/002/2017	A / H5N8	Europe / Poland / Opole Voivodeship	Edyta ?wi?to? (National Veterinary Research Institut Poland, PIWet-PIB)
EPI_ISL_255917	A/mute swan/Poland/64/2017	A / H5N5	Europe / Poland / Lower Silesian Voivodeship	Edyta ?wi?to? (National Veterinary Research Institut Poland, PIWet-PIB)
EPI_ISL_255933	A/Cormorant/Hungary/6102/2017	A / H5N8	Europe / Hungary / Komarom-Esztergom	Adam Dan (Danam.Vet.Molbiol)
EPI_ISL_255934	A/Mute swan/Hungary/6092/2017	A / H5N8	Europe / Hungary / Komarom-Esztergom	Adam Dan (Danam.Vet.Molbiol)
EPI_ISL_255935	A/Mute swan/Hungary/6276/2017	A / H5N8	Europe / Hungary / Zala	Adam Dan (Danam.Vet.Molbiol)
EPI_ISL_255936	A/Pheasant/Hungary/6553/2017	A / H5N8	Europe / Hungary / Jasz-Nagykun-Szolnok	Adam Dan (Danam.Vet.Molbiol)
EPI_ISL_255937	A/Pheasant/Hungary/7685/2017	A / H5N8	Europe / Hungary / Jasz-Nagykun-Szolnok	Adam Dan (Danam.Vet.Molbiol)
EPI_ISL_255938	A/Rook/Hungary/4975/2017	A / H5N8	Europe / Hungary / Szabolcs-Szatmar-Bereg	Adam Dan (Danam.Vet.Molbiol)
EPI_ISL_256213	A/Hubei/29578/2016	A / H5N6	Asia / China / Hubei Province	Lei Yang (WHO Chinese National Influenza Center / Virology Institute, Chinese CDC)
EPI_ISL_256290	A/environment/Changsha/156/2014	A / H5N1	Asia / China	
EPI_ISL_256298	A/gadwall/Kurgan/2442/2016	A / H5N8	Europe / Russian Federation / Kurgan Oblast	Ivan Susloparov (State Research Center of Virology and Biotechnology (VECTOR) / Emerging Zoonotic Diseases and Influenza)
EPI_ISL_256299	A/chicken/Sergiyev Posad/38/2017	A / H5N8	Europe / Russian Federation / Moscow Oblast	Ivan Susloparov (State Research Center of Virology and Biotechnology (VECTOR) / Emerging Zoonotic Diseases and Influenza)
EPI_ISL_256300	A/chicken/Sergiyev Posad/39/2017	A / H5N8	Europe / Russian Federation / Moscow Oblast	Ivan Susloparov (State Research Center of Virology and Biotechnology (VECTOR) / Emerging Zoonotic Diseases and Influenza)
EPI_ISL_256301	A/environment/Kamchatka/18/2016	A / H5N5	Europe / Russian Federation / Kamchatka Krai	Ivan Susloparov (State Research Center of Virology and Biotechnology (VECTOR) / Emerging Zoonotic Diseases and Influenza)
EPI_ISL_256302	A/mute swan/Czech Republic/499-17/2017 (H5N8)	A / H5N8	Europe / Czech Republic / Olomoucky Kraj / Okres Olomouc / Olomouc - Nové Sady	Alexander Nagy (State Veterinary Institute Prague)
EPI_ISL_256304	A/mute swan/Czech Republic/581-17/2017 (H5N8)	A / H5N8	Europe / Czech Republic / Olomoucky Kraj / Okres Olomouc / Olomouc city - U D?tského domova	Alexander Nagy (State Veterinary Institute Prague)
EPI_ISL_256305	A/shelduck/Italy/17VIR1572-24/2017	A / H5N8	Europe / Italy / Rovigo	Bianca Zecchin (Istituto Zooprofilattico Sperimentale Delle Venezie)
EPI_ISL_256306	A/turkey/Italy/17VIR1574-1/2017	A / H5N8	Europe / Italy / Mantova	Bianca Zecchin (Istituto Zooprofilattico Sperimentale Delle Venezie)
EPI_ISL_256307	A/chicken/Italy/17VIR1684-2/2017	A / H5N8	Europe / Italy / Venezia	Bianca Zecchin (Istituto Zooprofilattico Sperimentale Delle Venezie)
EPI_ISL_256308	A/chicken/Italy/17VIR1751-3/2017	A / H5N8	Europe / Italy / Venezia	Bianca Zecchin (Istituto Zooprofilattico Sperimentale Delle Venezie)
EPI_ISL_256309	A/goose/Czech Republic/821-17_2/2017 (H5N8)	A / H5N8	Europe / Czech Republic / Jihocesky Kraj / Okres Tabor / Sedle?ko u Sob?slav?	Alexander Nagy (State Veterinary Institute Prague)
EPI_ISL_256396	A/chicken/Taiwan/u7/2016	A / H5N2	Asia / Taiwan	Yu-Pin Liu (Animal Health Research Institute)
EPI_ISL_256398	A/chicken/Taiwan/x37/2016	A / H5N8	Asia / Taiwan	Yu-Pin Liu (Animal Health Research Institute)
EPI_ISL_256453	A/Duck/Hungary/54738/2016	A / H5N8	Europe / Hungary / Bacs-Kiskun	Adam Dan (Danam.Vet.Molbiol)
EPI_ISL_256457	A/Duck/Hungary/55764/2016	A / H5N8	Europe / Hungary / Bacs-Kiskun	Adam Dan (Danam.Vet.Molbiol)
EPI_ISL_256460	A/Turkey/Hungary/53136/2016	A / H5N8	Europe / Hungary / Bekes megye / Bekes	Adam Dan (Danam.Vet.Molbiol)
EPI_ISL_256462	A/Mute_swan/Hungary/5879/2017	A / H5N5	Europe / Hungary / Budapest fovaros	Adam Dan (Danam.Vet.Molbiol)
EPI_ISL_256495	A/owl/Hokkaido/X6/2016	A / H5N6	Asia / Japan / Hokkaido	Masatoshi Okamatsu (Hokkaido University / Graduate School of Veterinary Medicine)
EPI_ISL_256496	A/peregrine falcon/Hokkaido/X7/2016	A / H5N6	Asia / Japan / Hokkaido	Masatoshi Okamatsu (Hokkaido University / Graduate School of Veterinary Medicine)
EPI_ISL_256497	A/pintail/Hokkaido/X8/2016	A / H5N6	Asia / Japan / Hokkaido	Masatoshi Okamatsu (Hokkaido University / Graduate School of Veterinary Medicine)
EPI_ISL_256498	A/whooper swan/Hokkaido/X12/2017	A / H5N6	Asia / Japan / Hokkaido	Masatoshi Okamatsu (Hokkaido University / Graduate School of Veterinary Medicine)
EPI_ISL_256499	A/whooper swan/Hokkaido/X13/2017	A / H5N6	Asia / Japan / Hokkaido	Masatoshi Okamatsu (Hokkaido University / Graduate School of Veterinary Medicine)
EPI_ISL_256500	A/chicken/Hokkaido/002/2017	A / H5N6	Asia / Japan / Hokkaido	Masatoshi Okamatsu (Hokkaido University / Graduate School of Veterinary Medicine)
EPI_ISL_256501	A/mute swan/Aomori/4/2016	A / H5N6	Asia / Japan / Aomori	Masatoshi Okamatsu (Hokkaido University / Graduate School of Veterinary Medicine)
EPI_ISL_256505	A/whooper swan/Aomori/9/2016	A / H5N6	Asia / Japan	Masatoshi Okamatsu (Hokkaido University / Graduate School of Veterinary Medicine)
EPI_ISL_256506	A/grey-faced buzzard/Aomori/10/2016	A / H5N6	Asia / Japan	Masatoshi Okamatsu (Hokkaido University / Graduate School of Veterinary Medicine)
EPI_ISL_256507	A/whooper swan/Iwate/1/2016	A / H5N6	Asia / Japan	Masatoshi Okamatsu (Hokkaido University / Graduate School of Veterinary Medicine)
EPI_ISL_256508	A/whooper swan/Iwate/5/2016	A / H5N6	Asia / Japan	Masatoshi Okamatsu (Hokkaido University / Graduate School of Veterinary Medicine)
EPI_ISL_256509	A/whooper swan/Iwate/8/2016	A / H5N6	Asia / Japan	Masatoshi Okamatsu (Hokkaido University / Graduate School of Veterinary Medicine)
EPI_ISL_256510	A/tundra swan/Iwate/9/2016	A / H5N6	Asia / Japan	Masatoshi Okamatsu (Hokkaido University / Graduate School of Veterinary Medicine)
EPI_ISL_256511	A/whooper swan/Iwate/11/2016	A / H5N6	Asia / Japan	Masatoshi Okamatsu (Hokkaido University / Graduate School of Veterinary Medicine)
EPI_ISL_256512	A/coot/Iwate/13/2016	A / H5N6	Asia / Japan	Masatoshi Okamatsu (Hokkaido University / Graduate School of Veterinary Medicine)
EPI_ISL_256513	A/whooper swan/Iwate/17/2016	A / H5N6	Asia / Japan	Masatoshi Okamatsu (Hokkaido University / Graduate School of Veterinary Medicine)
EPI_ISL_256514	A/white-fronted goose/Miyagi/1/2016	A / H5N6	Asia / Japan	Masatoshi Okamatsu (Hokkaido University / Graduate School of Veterinary Medicine)
EPI_ISL_256515	A/white-fronted goose/Miyagi/2/2016	A / H5N6	Asia / Japan	Masatoshi Okamatsu (Hokkaido University / Graduate School of Veterinary Medicine)
EPI_ISL_256516	A/whooper swan/Fukushima/1/2016	A / H5N6	Asia / Japan	Masatoshi Okamatsu (Hokkaido University / Graduate School of Veterinary Medicine)
EPI_ISL_256517	A/whooper swan/Fukushima/3/2016	A / H5N6	Asia / Japan	Masatoshi Okamatsu (Hokkaido University / Graduate School of Veterinary Medicine)
EPI_ISL_256518	A/tundra swan/Niigata/1/2016	A / H5N6	Asia / Japan	Masatoshi Okamatsu (Hokkaido University / Graduate School of Veterinary Medicine)
EPI_ISL_256519	A/tundra swan/Niigata/4/2016	A / H5N6	Asia / Japan	Masatoshi Okamatsu (Hokkaido University / Graduate School of Veterinary Medicine)
EPI_ISL_256520	A/tundra swan/Niigata/8/2016	A / H5N6	Asia / Japan	Masatoshi Okamatsu (Hokkaido University / Graduate School of Veterinary Medicine)
EPI_ISL_256521	A/peregrine falcon/Niigata/12/2017	A / H5N6	Asia / Japan	Masatoshi Okamatsu (Hokkaido University / Graduate School of Veterinary Medicine)
EPI_ISL_256522	A/whooper swan/Niigata/13/2017	A / H5N6	Asia / Japan	Masatoshi Okamatsu (Hokkaido University / Graduate School of Veterinary Medicine)
EPI_ISL_256523	A/mute swan/Ibaraki/7/2016	A / H5N6	Asia / Japan	Masatoshi Okamatsu (Hokkaido University / Graduate School of Veterinary Medicine)
EPI_ISL_256524	A/whooper swan/Tochigi/1/2017	A / H5N6	Asia / Japan	Masatoshi Okamatsu (Hokkaido University / Graduate School of Veterinary Medicine)
EPI_ISL_256817	A/turkey/Germany-NI/R9807/2016	A / H5N8	Europe / Germany / Lower Saxony	Elke Starick (Friedrich-Loeffler-Institut)
EPI_ISL_256818	A/domestic duck/Germany-MV/R9764/2016	A / H5N8	Europe / Germany / Mecklenburg-Vorpommern	Elke Starick (Friedrich-Loeffler-Institut)
EPI_ISL_2574053	A/Towny owel/Sweden/SVAU210406SZ0042/KN001362/M-20	A / H5N8	Europe / Sweden / Skane Lan / Sjobo Kommun	Siamak Zohari (National Veterinary Institute)
EPI_ISL_257416	A/black_swan/Ibaraki/28-446T/2017	A / H5N6	Asia / Japan	Takehiko Saito (National Institute of Animal Health)
EPI_ISL_257417	A/black_swan/Ibaraki/28-446C/2017	A / H5N6	Asia / Japan	Takehiko Saito (National Institute of Animal Health)
EPI_ISL_257438	A/chicken/Miyagi/1-5C/2017	A / H5N6	Asia / Japan / Miyagi	Takehiko Saito (National Institute of Animal Health)
EPI_ISL_257439	A/chicken/Chiba/1-4C/2017	A / H5N6	Asia / Japan / Chiba	Takehiko Saito (National Institute of Animal Health)
EPI_ISL_257440	A/chicken/Chiba/1-5T/2017	A / H5N6	Asia / Japan / Chiba	Takehiko Saito (National Institute of Animal Health)
EPI_ISL_257441	A/chicken/Chiba/1-3C/2017	A / H5N6	Asia / Japan / Chiba	Takehiko Saito (National Institute of Animal Health)
EPI_ISL_257442	A/chicken/Chiba/1-3T/2017	A / H5N6	Asia / Japan / Chiba	Takehiko Saito (National Institute of Animal Health)
EPI_ISL_257443	A/chicken/Chiba/1-5C/2017	A / H5N6	Asia / Japan / Chiba	Takehiko Saito (National Institute of Animal Health)
EPI_ISL_257444	A/chicken/Chiba/1-2T/2017	A / H5N6	Asia / Japan / Chiba	Takehiko Saito (National Institute of Animal Health)

EPI_ISL_257445	A/chicken/Chiba/1-1T/2017	A / H5N6	Asia / Japan / Chiba	Takehiko Saito (National Institute of Animal Health)
EPI_ISL_257446	A/chicken/Chiba/1-4T/2017	A / H5N6	Asia / Japan / Chiba	Takehiko Saito (National Institute of Animal Health)
EPI_ISL_257447	A/chicken/Chiba/1-2C/2017	A / H5N6	Asia / Japan / Chiba	Takehiko Saito (National Institute of Animal Health)
EPI_ISL_257448	A/chicken/Miyagi/1-5T/2017	A / H5N6	Asia / Japan / Miyagi	Takehiko Saito (National Institute of Animal Health)
EPI_ISL_257449	A/chicken/Chiba/1-1C/2017	A / H5N6	Asia / Japan / Chiba	Takehiko Saito (National Institute of Animal Health)
EPI_ISL_257657	A/Pavo cristatus/Jiangxi/JA1/2016	A / H5N6	Asia / China	
EPI_ISL_257699	A/Tufted Duck/Switzerland/V237/2016	A / H5N8	Europe / Switzerland / Kanton Bern / Amt Biel / Lake Biel	Ronald Dijkman (Faculty of Veterinary Medicine at the University of Bern / Institute of Virology and Immunology IVI)
EPI_ISL_257700	A/mute swan/Czech Republic/1813-17/2017 (H5N8)	A / H5N8	Europe / Czech Republic / Karlovarsky Kraj / Okres Cheb / GPS: 50°4'56.294''N, 12°22'7.530E	Alexander Nagy (State Veterinary Institute Prague)
EPI_ISL_257701	A/chicken/Czech Republic/2643-17_1/2017 (H5N8)	A / H5N8	Europe / Czech Republic / Jihocesky Kraj / Okres Jindrichuv Hradec / Da?ice	Alexander Nagy (State Veterinary Institute Prague)
EPI_ISL_257702	A/chicken/Czech Republic/2644-17_1/2017 (H5N8)	A / H5N8	Europe / Czech Republic / Plzensky Kraj / Bohy (Kralovice), GPS: 49°56'15.55''N, 13°35'17.58E	Alexander Nagy (State Veterinary Institute Prague)
EPI_ISL_257703	A/mallard/Czech Republic/2705-17/2017 (H5N8)	A / H5N8	Europe / Czech Republic / Kralovehradecky Kraj / Havlí?k?v Brod, GPS: 49°36'13.705''N, 15°35'0.	Alexander Nagy (State Veterinary Institute Prague)
EPI_ISL_257731	A/turkey/Germany-BB/R377ff/2017	A / H5N8	Europe / Germany / Brandenburg	Elke Starick (Friedrich-Loeffler-Institut)
EPI_ISL_258415	A/grey heron/Germany-TH/R1125/2017	A / H5N8	Europe / Germany / Thuringia	Elke Starick (Friedrich-Loeffler-Institut)
EPI_ISL_258526	A/white stork/Germany-TH/R1149/2017	A / H5N8	Europe / Germany / Thuringia	Elke Starick (Friedrich-Loeffler-Institut)
EPI_ISL_258527	A/tawny owl/Germany-SN/R1186/2017	A / H5N8	Europe / Germany / Saxony	Elke Starick (Friedrich-Loeffler-Institut)
EPI_ISL_258661	A/mute swan/Germany-TH/R1126/2017	A / H5N8	Europe / Germany / Thuringia	Elke Starick (Friedrich-Loeffler-Institut)
EPI_ISL_259074	A/common buzzard/Germany-SN/R1117/2017	A / H5N5	Europe / Germany / Saxony	Elke Starick (Friedrich-Loeffler-Institut)
EPI_ISL_259525	A/egret/Germany-SH/R1459/2017	A / H5N5	Europe / Germany / Schleswig-Holstein	Elke Starick (Friedrich-Loeffler-Institut)
EPI_ISL_259923	A/duck/Guangdong/673/2014(H5N6)	A / H5N6	Asia / China / Guangdong Province	Qi Wenbao (South China Agricultural University / Veterinary Medicine College)
EPI_ISL_259924	A/goose/Guangdong/674/2014(H5N6)	A / H5N6	Asia / China / Guangdong Province	Qi Wenbao (South China Agricultural University / Veterinary Medicine College)
EPI_ISL_259927	A/goose/Guangdong/SH7/2013(H5N1)	A / H5N1	Asia / China / Guangdong Province	Qi Wenbao (South China Agricultural University / Veterinary Medicine College)
EPI_ISL_260058	A/grey heron/Germany-SN/R572/2017	A / H5N5	Europe / Germany / Saxony	Elke Starick (Friedrich-Loeffler-Institut)
EPI_ISL_260059	A/cormorant/Germany-SH/R896/2017	A / H5N5	Europe / Germany / Schleswig-Holstein	Elke Starick (Friedrich-Loeffler-Institut)
EPI_ISL_260773	A/whooper swan/Iwate/7/2016	A / H5N6	Asia / Japan	Masatoshi Okamatsu (Hokkaido University / Graduate School of Veterinary Medicine)
EPI_ISL_261332	A/chicken/Croatia/104/2017	A / H5N5	Europe / Croatia / Krapinsko-Zagorska Zupanija / Špi?kovina	Vladimir Savi? (Croatian Veterinary Institute / Poultry Centre)
EPI_ISL_261634	A/chicken/Republic of Macedonia/AR1167-L02131/2017	A / H5N8	Europe / Macedonia, the former Yogoslav Republic of / N 41°12’10, E20°42’01	Anne Pohlmann (Friedrich-Loeffler-Institut)
EPI_ISL_261687	A/black swan/Germany-BW/R1364/2017	A / H5N8	Europe / Germany / Baden-Wuerttemberg	Elke Starick (Friedrich-Loeffler-Institut)
EPI_ISL_262055	A/eurasian wigeon/Germany-NI/AR249-L02143/2017	A / H5N8	Europe / Germany / Lower Saxony / Wilhelmshaven	Anne Pohlmann (Friedrich-Loeffler-Institut)
EPI_ISL_262056	A/greylag goose/Germany-NI/AR11353-L02142/2016	A / H5N5	Europe / Germany / Lower Saxony / Wangerland	Anne Pohlmann (Friedrich-Loeffler-Institut)
EPI_ISL_262057	A/mute swan/Germany-NI/AR1529-L02145/2017	A / H5N8	Europe / Germany / Lower Saxony / Ammerland	Anne Pohlmann (Friedrich-Loeffler-Institut)
EPI_ISL_262058	A/greylag goose/Germany-NI/AR1395-L02144/2017	A / H5N8	Europe / Germany / Lower Saxony / Peine	Anne Pohlmann (Friedrich-Loeffler-Institut)
EPI_ISL_262059	A/greylag goose/Germany-NI/AR703-L02138/2017	A / H5N8	Europe / Germany / Lower Saxony / Hemmingen	Anne Pohlmann (Friedrich-Loeffler-Institut)
EPI_ISL_2652003	A/White-Tailed Eagle/Sweden/SVA210528SZ0223/KN002027/	A / H5N1	Europe / Sweden / Stockholms Lan / Ekero Kommun	Siamak Zohari (National Veterinary Institute)
EPI_ISL_266417	A/common teal/Korea/W547/2016	A / H5N8	Asia / Korea, Republic of	
EPI_ISL_266418	A/common teal/Korea/W549/2016	A / H5N8	Asia / Korea, Republic of	
EPI_ISL_266419	A/common teal/Korea/W548/2016	A / H5N8	Asia / Korea, Republic of	
EPI_ISL_266420	A/common teal/Korea/W550/2016	A / H5N8	Asia / Korea, Republic of	
EPI_ISL_266421	A/common teal/Korea/W555/2017	A / H5N8	Asia / Korea, Republic of	
EPI_ISL_266424	A/common teal/Korea/W558/2017	A / H5N6	Asia / Korea, Republic of	
EPI_ISL_266425	A/common teal/Korea/W559/2017	A / H5N6	Asia / Korea, Republic of	
EPI_ISL_266514	A/environment/Zhongshan/ZS01/2016	A / H5N6	Asia / China	
EPI_ISL_266536	A/chicken/Belgium/807/2017	A / H5N8	Europe / Belgium	
EPI_ISL_266537	A/peacock/Belgium/1017/2017	A / H5N8	Europe / Belgium	
EPI_ISL_266538	A/Cygnus olor/Belgium/1567/2017	A / H5N8	Europe / Belgium	
EPI_ISL_266596	A/swine/Guangdong/G3/2015	A / H5N6	Asia / China / Guangdong Province	Feng Qin (South China Agricultural University / Veterinary Medicine College)
EPI_ISL_266600	A/enviroment/Guangdong/F3/2015	A / H5N6	Asia / China / Guangdong Province	Feng Qin (South China Agricultural University / Veterinary Medicine College)
EPI_ISL_266819	A/enviroment/Guangdong/F4/2016	A / H5N6	Asia / China / Guangdong Province	Feng Qin (South China Agricultural University / Veterinary Medicine College)
EPI_ISL_266820	A/Bean goose/Hubei/CH-i122/2017_H5N8	A / H5N8	Asia / China / Hubei Province	Jianjun Chen (Wuhan Institute of Virology / Chinese Academy of Sciences)
EPI_ISL_266821	A/Bean goose/Hubei/CH-i119/2017	A / H5N8	Asia / China / Hubei Province	Jianjun Chen (Wuhan Institute of Virology / Chinese Academy of Sciences)
EPI_ISL_266822	A/Herring Gull/Hubei/CH-i149/2017_H5N8	A / H5N8	Asia / China / Hubei Province	Jianjun Chen (Wuhan Institute of Virology / Chinese Academy of Sciences)
EPI_ISL_266823	A/Bean goose/Hubei/CH-i336/2017	A / H5N6	Asia / China / Hubei Province	Jianjun Chen (Wuhan Institute of Virology / Chinese Academy of Sciences)
EPI_ISL_266824	A/Bean goose/Hubei/CH-i320/2017_H5N8	A / H5N8	Asia / China / Hubei Province	Jianjun Chen (Wuhan Institute of Virology / Chinese Academy of Sciences)
EPI_ISL_266825	A/Bean goose/Hubei/CH-I225/2017	A / H5N6	Asia / China / Hubei Province	Jianjun Chen (Wuhan Institute of Virology / Chinese Academy of Sciences)
EPI_ISL_266939	A/tundra swan/Tottori/3111S001/2016	A / H5N6	Asia / Japan / Tottori	
EPI_ISL_266940	A/tundra swan/Tottori/3111S002/2016	A / H5N6	Asia / Japan / Tottori	
EPI_ISL_266941	A/snowy owl/Akita/0051D007/2016	A / H5N6	Asia / Japan / Akita	
EPI_ISL_266942	A/snowy owl/Akita/0051D008/2016	A / H5N6	Asia / Japan / Akita	
EPI_ISL_266943	A/snowy owl/Akita/0051D010-4/2016	A / H5N6	Asia / Japan / Akita	
EPI_ISL_267135	A/green-winged teal/Egypt/871/2016	A / H5N8	Africa / Egypt	
EPI_ISL_267136	A/green-winged teal/Egypt/877/2016	A / H5N8	Africa / Egypt	
EPI_ISL_268021	A/duck/Egypt/SS19/2017	A / H5N8	Africa / Egypt / Sharqia	Abdel-Satar Mohamed Arafa (Animal Health Research Institute)
EPI_ISL_2681044	A/chicken/Romania/12448_21VIR3734-3/2021	A / H5N8	Europe / Romania / Mures, Ungheni	Bianca Zecchin (Istituto Zooprofilattico Sperimentale Delle Venezie)
EPI_ISL_2681045	A/white_stork/Poland/MB391/2021	A / H5N1	Europe / Poland / Swietokrzyskie Voivodeship	Edyta ?wi?to? (National Veterinary Research Institut Poland, PIWet-PIB)
EPI_ISL_268513	A/duck/Egypt/F446/2017	A / H5N8	Africa / Egypt / Cairo	Abdel-Satar Mohamed Arafa (Animal Health Research Institute)
EPI_ISL_2685842	A/tundra swan/Hubei/BQ2/2020	A / H5N8	Asia / China / Hubei Province	Hongliang Chai (Northeast Forestry University / College of Wildlife Resources)
EPI_ISL_2685846	A/tundra swan/Hubei/BQ3/2020	A / H5N8	Asia / China / Hubei Province	Hongliang Chai (Northeast Forestry University / College of Wildlife Resources)
EPI_ISL_2685847	A/tundra swan/Hubei/BQ4/2020	A / H5N8	Asia / China / Hubei Province	Hongliang Chai (Northeast Forestry University / College of Wildlife Resources)
EPI_ISL_2685848	A/tundra swan/Hubei/BQ6/2020	A / H5N8	Asia / China / Hubei Province	Hongliang Chai (Northeast Forestry University / College of Wildlife Resources)
EPI_ISL_2685900	A/tundra swan/Hubei/BQ7/2020	A / H5N8	Asia / China / Hubei Province	Hongliang Chai (Northeast Forestry University / College of Wildlife Resources)
EPI_ISL_2685947	A/tundra swan/Hubei/BQ8/2020	A / H5N8	Asia / China / Hubei Province	Hongliang Chai (Northeast Forestry University / College of Wildlife Resources)
EPI_ISL_268618	A/Bk_swan/NL-Den Oever/16013973-002/2016	A / H5N8	Europe / Netherlands / Provincie Noord-Holland / Den Oever	Saskia Bergervoet (Wageningen Bioveterinary Research)
EPI_ISL_268619	A/Bl_H_gull/NL-Slootdorp/16014102-002/2016	A / H5N8	Europe / Netherlands / Provincie Noord-Holland / Slootdorp	Saskia Bergervoet (Wageningen Bioveterinary Research)

EPI_ISL_268620	A/Buzzard/NL-Durgerdam/16015100-004/2016	A / H5N8	Europe / Netherlands / Provincie Noord-Holland / Durgerdam	Saskia Bergervoet (Wageningen Bioveterinary Research)
EPI_ISL_268621	A/C_Gull/NL-Slootdorp/16014102-003/2016	A / H5N8	Europe / Netherlands / Provincie Noord-Holland / Slootdorp	Saskia Bergervoet (Wageningen Bioveterinary Research)
EPI_ISL_268622	A/Ch/NL-Abbega/X16015736/2016	A / H5N8	Europe / Netherlands / Provincie Friesland / Abbega	Saskia Bergervoet (Wageningen Bioveterinary Research)
EPI_ISL_268623	A/Ch/NL-Boven Leeuwen/16016151-006-010/2016	A / H5N8	Europe / Netherlands / Provincie Gelderland / Boven Leeuwen	Saskia Bergervoet (Wageningen Bioveterinary Research)
EPI_ISL_268624	A/Ch/NL-Den Oever/16014231-001/2016	A / H5N8	Europe / Netherlands / Provincie Noord-Holland / Den Oever	Saskia Bergervoet (Wageningen Bioveterinary Research)
EPI_ISL_268625	A/Ch/NL-Hiaure/16016112-001-005/2016	A / H5N8	Europe / Netherlands / Provincie Friesland / Hiaure	Saskia Bergervoet (Wageningen Bioveterinary Research)
EPI_ISL_268626	A/Ch/NL-Rhenen/16016141-006/2016	A / H5N8	Europe / Netherlands / Provincie Utrecht / Gemeente Rhenen	Saskia Bergervoet (Wageningen Bioveterinary Research)
EPI_ISL_268627	A/Ch/NL-Zoeterwoude/16016484-021-025/2016	A / H5N8	Europe / Netherlands / South Holland / Gemeente Zoeterwoude	Saskia Bergervoet (Wageningen Bioveterinary Research)
EPI_ISL_268628	A/Crow/NL-Oostwoud/16015372-004/2016	A / H5N8	Europe / Netherlands / Provincie Noord-Holland / Oostwoud	Saskia Bergervoet (Wageningen Bioveterinary Research)
EPI_ISL_268629	A/Dk/NL-Biddinghuizen/16014829-011-015/2016	A / H5N8	Europe / Netherlands / Provincie Flevoland / Biddinghuizen	Saskia Bergervoet (Wageningen Bioveterinary Research)
EPI_ISL_268630	A/Dk/NL-Biddinghuizen/16015083-016-020/2016	A / H5N8	Europe / Netherlands / Provincie Flevoland / Biddinghuizen	Saskia Bergervoet (Wageningen Bioveterinary Research)
EPI_ISL_268631	A/Dk/NL-Biddinghuizen/16015145-021-025/2016	A / H5N8	Europe / Netherlands / Provincie Flevoland / Biddinghuizen	Saskia Bergervoet (Wageningen Bioveterinary Research)
EPI_ISL_268632	A/Dk/NL-Kamperveen/16016104-001-005/2016	A / H5N8	Europe / Netherlands / Provincie Overijssel / Kamperveen	Saskia Bergervoet (Wageningen Bioveterinary Research)
EPI_ISL_268633	A/Dk/NL-Rotterdam/16014008-001-005/2016	A / H5N8	Europe / Netherlands / South Holland / Gemeente Rotterdam	Saskia Bergervoet (Wageningen Bioveterinary Research)
EPI_ISL_268634	A/Dk/NL-Stolwijk/16016291-016-020/2016	A / H5N8	Europe / Netherlands / South Holland / Stolwijk	Saskia Bergervoet (Wageningen Bioveterinary Research)
EPI_ISL_268635	A/Eur_Wig/NL-Akkrum/16015817-003/2016	A / H5N8	Europe / Netherlands / Provincie Friesland / Akkrum	Saskia Bergervoet (Wageningen Bioveterinary Research)
EPI_ISL_268636	A/Eur_Wig/NL-De Waal (Texel)/16014891-003/2016	A / H5N8	Europe / Netherlands / Provincie Noord-Holland / De Waal (Texel)	Saskia Bergervoet (Wageningen Bioveterinary Research)
EPI_ISL_268637	A/Eur_Wig/NL-De Waal (Texel)/16014891-004/2016	A / H5N8	Europe / Netherlands / Provincie Noord-Holland / De Waal (Texel)	Saskia Bergervoet (Wageningen Bioveterinary Research)
EPI_ISL_268638	A/Eur_Wig/NL-Drieborg (Dollard)/16015513-001/2016	A / H5N8	Europe / Netherlands / Provincie Groningen / Drieborg (Dollard)	Saskia Bergervoet (Wageningen Bioveterinary Research)
EPI_ISL_268639	A/Eur_Wig/NL-Enumatil-Groningen/16015704-001/2016	A / H5N8	Europe / Netherlands / Provincie Groningen / Enumatil-Groningen	Saskia Bergervoet (Wageningen Bioveterinary Research)
EPI_ISL_268640	A/Eur_Wig/NL-Ferwert/16015273-002/2016	A / H5N8	Europe / Netherlands / Provincie Friesland / Ferwert	Saskia Bergervoet (Wageningen Bioveterinary Research)
EPI_ISL_268641	A/Eur_Wig/NL-Gouda/16015824-001/2016	A / H5N8	Europe / Netherlands / South Holland / Gemeente Gouda	Saskia Bergervoet (Wageningen Bioveterinary Research)
EPI_ISL_268642	A/Eur_Wig/NL-Greonterp/16015653-001/2016	A / H5N8	Europe / Netherlands / Provincie Friesland / Greonterp	Saskia Bergervoet (Wageningen Bioveterinary Research)
EPI_ISL_268643	A/Eur_Wig/NL-Groningen/16015376-003/2016	A / H5N8	Europe / Netherlands / Provincie Groningen / Gemeente Groningen	Saskia Bergervoet (Wageningen Bioveterinary Research)
EPI_ISL_268644	A/Eur_Wig/NL-Leeuwarden/16015699-002/2016	A / H5N8	Europe / Netherlands / Provincie Friesland / Gemeente Leeuwarden	Saskia Bergervoet (Wageningen Bioveterinary Research)
EPI_ISL_268645	A/Eur_Wig/NL-Leidschendam/16015697-007/2016	A / H5N8	Europe / Netherlands / South Holland / Leidschendam	Saskia Bergervoet (Wageningen Bioveterinary Research)
EPI_ISL_268646	A/Eur_Wig/NL-Reeuwijk/16015903-003/2016	A / H5N8	Europe / Netherlands / South Holland / Gemeente Reeuwijk	Saskia Bergervoet (Wageningen Bioveterinary Research)
EPI_ISL_268647	A/Eur_Wig/NL-Terschelling/16015692-010/2016	A / H5N8	Europe / Netherlands / Provincie Friesland / Gemeente Terschelling	Saskia Bergervoet (Wageningen Bioveterinary Research)
EPI_ISL_268648	A/Eur_Wig/NL-Vianen/16015917-006/2016	A / H5N8	Europe / Netherlands / Provincie Utrecht / Gemeente Vianen	Saskia Bergervoet (Wageningen Bioveterinary Research)
EPI_ISL_268649	A/Eur_Wig/NL-Walterswald/16015923-003/2016	A / H5N8	Europe / Netherlands / Provincie Friesland / Walterswald	Saskia Bergervoet (Wageningen Bioveterinary Research)
EPI_ISL_268650	A/Eur_Wig/NL-West Graftdijk/16015746-003/2016	A / H5N8	Europe / Netherlands / Provincie Noord-Holland / West Graftdijk	Saskia Bergervoet (Wageningen Bioveterinary Research)
EPI_ISL_268651	A/Eur_Wig/NL-Wormer/16016143-002/2016	A / H5N8	Europe / Netherlands / Provincie Noord-Holland / Wormer	Saskia Bergervoet (Wageningen Bioveterinary Research)
EPI_ISL_268652	A/Eur_Wig/NL-Zoeterwoude/16015702-010/2016	A / H5N8	Europe / Netherlands / South Holland / Gemeente Zoeterwoude	Saskia Bergervoet (Wageningen Bioveterinary Research)
EPI_ISL_268653	A/Eur_Wig/NL-Zwolle/16015820-002/2016	A / H5N8	Europe / Netherlands / Provincie Overijssel / Gemeente Zwolle	Saskia Bergervoet (Wageningen Bioveterinary Research)
EPI_ISL_268654	A/G_c_grebe/NL-Monnickendam/16013865-009-010/2016	A / H5N8	Europe / Netherlands / Provincie Noord-Holland / Monnickendam	Saskia Bergervoet (Wageningen Bioveterinary Research)
EPI_ISL_268655	A/Go/NL-Roggebotsluis/16014462-010/2016	A / H5N8	Europe / Netherlands / Provincie Flevoland / Roggebotsluis	Saskia Bergervoet (Wageningen Bioveterinary Research)
EPI_ISL_268656	A/Gr_bk_bd_gull/NL-Slootdorp/16014102-005/2016	A / H5N8	Europe / Netherlands / Provincie Noord-Holland / Slootdorp	Saskia Bergervoet (Wageningen Bioveterinary Research)
EPI_ISL_268657	A/Grey_Go/NL-Groot-Ammers/16015901-012/2016	A / H5N8	Europe / Netherlands / South Holland / Groot-Ammers	Saskia Bergervoet (Wageningen Bioveterinary Research)
EPI_ISL_268658	A/Gull/NL-Marker Wadden/16014466-020/2016	A / H5N8	Europe / Netherlands / Marker Wadden	Saskia Bergervoet (Wageningen Bioveterinary Research)
EPI_ISL_268659	A/Gull1/NL-Marker Wadden/16014466-011/2016	A / H5N8	Europe / Netherlands / Marker Wadden	Saskia Bergervoet (Wageningen Bioveterinary Research)
EPI_ISL_268660	A/Gull10/NL-Marker Wadden/16014466-014/2016	A / H5N8	Europe / Netherlands / Marker Wadden	Saskia Bergervoet (Wageningen Bioveterinary Research)
EPI_ISL_268661	A/L-bl-ba-gull/NL-Sovon/16014324-014/2016	A / H5N8	Europe / Netherlands	Saskia Bergervoet (Wageningen Bioveterinary Research)
EPI_ISL_268662	A/M_Swan/NL-Roggebotsluis/16014462-019/2016	A / H5N8	Europe / Netherlands / Provincie Flevoland / Roggebotsluis	Saskia Bergervoet (Wageningen Bioveterinary Research)
EPI_ISL_268663	A/Magpie/NL-Volendam/16014331-002/2016	A / H5N8	Europe / Netherlands / Provincie Noord-Holland / Edam-Volendam	Saskia Bergervoet (Wageningen Bioveterinary Research)
EPI_ISL_268664	A/Mal/NL-IJsselmuiden/16015448-002/2016	A / H5N8	Europe / Netherlands / Provincie Overijssel / IJsselmuiden	Saskia Bergervoet (Wageningen Bioveterinary Research)
EPI_ISL_268665	A/Mal/NL-Mastenbroek/16015378-002/2016	A / H5N8	Europe / Netherlands / Provincie Overijssel / Mastenbroek	Saskia Bergervoet (Wageningen Bioveterinary Research)
EPI_ISL_268666	A/P_falcon/NL-Vrouwenpolder (Zeeland)/16015510-001/2016	A / H5N8	Europe / Netherlands / Provincie Zeeland / Vrouwenpolder	Saskia Bergervoet (Wageningen Bioveterinary Research)
EPI_ISL_268667	A/Sea_eagle/NL-Assen/16015398-002/2016	A / H5N8	Europe / Netherlands / Provincie Drenthe / Gemeente Assen	Saskia Bergervoet (Wageningen Bioveterinary Research)
EPI_ISL_268668	A/T_Dk/NL-Almeerder Zand/16014341-003/2016	A / H5N8	Europe / Netherlands / Provincie Flevoland / Almeerder Zand	Saskia Bergervoet (Wageningen Bioveterinary Research)
EPI_ISL_268669	A/T_Dk/NL-Monnickendam/16013865-006-008/2016	A / H5N8	Europe / Netherlands / Provincie Noord-Holland / Monnickendam	Saskia Bergervoet (Wageningen Bioveterinary Research)
EPI_ISL_268670	A/T_Dk/NL-Roggebotsluis/16014462-015/2016	A / H5N8	Europe / Netherlands / Provincie Flevoland / Roggebotsluis	Saskia Bergervoet (Wageningen Bioveterinary Research)
EPI_ISL_268671	A/T_Dk/NL-Rotterdam/16014155-001/2016	A / H5N8	Europe / Netherlands / South Holland / Gemeente Rotterdam	Saskia Bergervoet (Wageningen Bioveterinary Research)
EPI_ISL_268672	A/T_Dk/NL-Werkendam/16014159-002/2016	A / H5N8	Europe / Netherlands / North Brabant / Gemeente Werkendam	Saskia Bergervoet (Wageningen Bioveterinary Research)
EPI_ISL_268673	A/T_Dk/NL-Werkendam/16014159-003/2016	A / H5N8	Europe / Netherlands / North Brabant / Gemeente Werkendam	Saskia Bergervoet (Wageningen Bioveterinary Research)
EPI_ISL_268674	A/T_Dk/NL-Zeewolde/16013976-001/2016	A / H5N8	Europe / Netherlands / Provincie Flevoland / Gemeente Zeewolde	Saskia Bergervoet (Wageningen Bioveterinary Research)
EPI_ISL_268675	A/T_Dk/NL-Zeewolde/16013976-001-003/2016	A / H5N8	Europe / Netherlands / Provincie Flevoland / Gemeente Zeewolde	Saskia Bergervoet (Wageningen Bioveterinary Research)
EPI_ISL_268676	A/T_Dk/NL-Zeewolde/16013976-004/2016	A / H5N8	Europe / Netherlands / Provincie Flevoland / Gemeente Zeewolde	Saskia Bergervoet (Wageningen Bioveterinary Research)
EPI_ISL_268677	A/T_Dk/NL-Zeewolde/16013976-004-006/2016	A / H5N8	Europe / Netherlands / Provincie Flevoland / Gemeente Zeewolde	Saskia Bergervoet (Wageningen Bioveterinary Research)
EPI_ISL_268678	A/T_Dk/NL-Zeewolde/16013976-005/2016	A / H5N8	Europe / Netherlands / Provincie Flevoland / Gemeente Zeewolde	Saskia Bergervoet (Wageningen Bioveterinary Research)
EPI_ISL_268679	A/T_Dk/NL-Zeewolde/16013976-006/2016	A / H5N8	Europe / Netherlands / Provincie Flevoland / Gemeente Zeewolde	Saskia Bergervoet (Wageningen Bioveterinary Research)
EPI_ISL_268680	A/T_Dk/NL-Zuidoost Beemster/16014148-002/2016	A / H5N8	Europe / Netherlands / Provincie Noord-Holland / Zuidoost Beemster	Saskia Bergervoet (Wageningen Bioveterinary Research)
EPI_ISL_268681	A/T_Dk/NL-Zuidoost Beemster/16014148-009/2016	A / H5N8	Europe / Netherlands / Provincie Noord-Holland / Zuidoost Beemster	Saskia Bergervoet (Wageningen Bioveterinary Research)
EPI_ISL_268682	A/Teal/NL-Ferwert/16015273-013/2016	A / H5N8	Europe / Netherlands / Provincie Friesland / Ferwert	Saskia Bergervoet (Wageningen Bioveterinary Research)
EPI_ISL_268799	A/Back-headed_Gull/Netherlands/8/2016	A / H5N8	Europe / Netherlands	Maria Johanna Poen (Erasmus Medical Center / Department of Virology)
EPI_ISL_268800	A/Black-headed_Gull/Netherlands/17/2016	A / H5N8	Europe / Netherlands	Maria Johanna Poen (Erasmus Medical Center / Department of Virology)
EPI_ISL_268866	A/Back-headed_Gull/Netherlands/9/2016	A / H5N8	Europe / Netherlands	Maria Johanna Poen (Erasmus Medical Center / Department of Virology)
EPI_ISL_268916	A/Caspian _Gull/Netherlands/1/2016	A / H5N8	Europe / Netherlands	Maria Johanna Poen (Erasmus Medical Center / Department of Virology)
EPI_ISL_268927	A/Common_Buzzard/Netherlands/1/2016	A / H5N8	Europe / Netherlands	Maria Johanna Poen (Erasmus Medical Center / Department of Virology)
EPI_ISL_268928	A/mute swan/Czech Republic/54-17_1/2017 (H5N8)	A / H5N8	Europe / Czech Republic / South Moravian Region / Mesto Brno / ??m?ice u Ivan?ic	Alexander Nagy (State Veterinary Institute Prague)
EPI_ISL_268929	A/Common_Eider/Netherlands/2/2016	A / H5N8	Europe / Netherlands	Maria Johanna Poen (Erasmus Medical Center / Department of Virology)
EPI_ISL_268930	A/goose/Czech Republic/136-17_1/2017 (H5N8)	A / H5N8	Europe / Czech Republic / South Moravian Region / Mesto Brno / Ivan?ice-N?m?ice	Alexander Nagy (State Veterinary Institute Prague)
EPI_ISL_268931	A/mallard/Czech Republic/136-17_2/2017 (H5N8)	A / H5N8	Europe / Czech Republic / South Moravian Region / Mesto Brno / Ivan?ice-N?m?ice	Alexander Nagy (State Veterinary Institute Prague)

EPI_ISL_268932	A/goose/Czech Republic/197-17/2017 (H5N8)	A / H5N8	Europe / Czech Republic / Hlavni mesto Praha / Okres Praha / Lázn? Touše?	Alexander Nagy (State Veterinary Institute Prague)
EPI_ISL_268933	A/chicken/Czech Republic/508-17_1/2017 (H5N8)	A / H5N8	Europe / Czech Republic / Jihocesky Kraj / Okres Tabor / Chýnov	Alexander Nagy (State Veterinary Institute Prague)
EPI_ISL_268934	A/mallard/Czech Republic/508-17_4/2017 (H5N8)	A / H5N8	Europe / Czech Republic / Jihocesky Kraj / Okres Tabor / Chýnov	Alexander Nagy (State Veterinary Institute Prague)
EPI_ISL_268935	A/mute swan/Czech Republic/572-17_3/2017 (H5N8)	A / H5N8	Europe / Czech Republic / Hlavni mesto Praha / Okres Praha / Prague 1, Alšovo náb?eží	Alexander Nagy (State Veterinary Institute Prague)
EPI_ISL_268936	A/mute swan/Czech Republic/964-17/2017 (H5N8)	A / H5N8	Europe / Czech Republic / Stredocesky Kraj / Okres Kolin	Alexander Nagy (State Veterinary Institute Prague)
EPI_ISL_268937	A/Eurasian_Wigeon/Netherlands/10/2016	A / H5N8	Europe / Netherlands	Maria Johanna Poen (Erasmus Medical Center / Department of Virology)
EPI_ISL_268938	A/mute swan/Czech Republic/967-17/2017 (H5N8)	A / H5N8	Europe / Czech Republic / Kralovehradecky Kraj / Okres Hradec Kralove / Tylovo náb?eží	Alexander Nagy (State Veterinary Institute Prague)
EPI_ISL_268939	A/mute swan/Czech Republic/987-17_2/2017 (H5N8)	A / H5N8	Europe / Czech Republic / Hlavni mesto Praha / Okres Praha / Prague 1, p?ístavišt? Four seasons	Alexander Nagy (State Veterinary Institute Prague)
EPI_ISL_268940	A/mute swan/Czech Republic/1058-17/2017 (H5N8)	A / H5N8	Europe / Czech Republic / Jihocesky Kraj / Okres Ceske Budejovice / ?eské Bud?jovice, 48°59'24.(	Alexander Nagy (State Veterinary Institute Prague)
EPI_ISL_268941	A/mute swan/Czech Republic/1060-17/2017 (H5N8)	A / H5N8	Europe / Czech Republic / Jihocesky Kraj / Okres Ceske Budejovice / ?eské Bud?jovice	Alexander Nagy (State Veterinary Institute Prague)
EPI_ISL_268942	A/mute swan/Czech Republic/1155-17/2017 (H5N8)	A / H5N8	Europe / Czech Republic / Jihocesky Kraj / Okres Ceske Budejovice / ?eské Bud?jovice	Alexander Nagy (State Veterinary Institute Prague)
EPI_ISL_268943	A/mute swan/Czech Republic/1156-17/2017 (H5N8)	A / H5N8	Europe / Czech Republic / Jihocesky Kraj / Okres Pisek / Písek	Alexander Nagy (State Veterinary Institute Prague)
EPI_ISL_268944	A/mute swan/Czech Republic/1170-17_2/2017 (H5N8)	A / H5N8	Europe / Czech Republic / Olomoucky Kraj / Okres Jesenik / Javorník-ves	Alexander Nagy (State Veterinary Institute Prague)
EPI_ISL_268945	A/mute swan/Czech Republic/1171-17/2017 (H5N8)	A / H5N8	Europe / Czech Republic / Morovskoslezsky Kraj / Okres Opava / Jilešovice	Alexander Nagy (State Veterinary Institute Prague)
EPI_ISL_268946	A/chicken/Czech Republic/1208-17_1/2017 (H5N8)	A / H5N8	Europe / Czech Republic / Liberecky Kraj / Okres Semily / Turnov	Alexander Nagy (State Veterinary Institute Prague)
EPI_ISL_268947	A/mallard/Czech Republic/1219-17_1/2017 (H5N8)	A / H5N8	Europe / Czech Republic / Jihocesky Kraj / Okres Strakonice / Blatná	Alexander Nagy (State Veterinary Institute Prague)
EPI_ISL_268948	A/mallard/Czech Republic/1226-17/2017 (H5N8)	A / H5N8	Europe / Czech Republic / Zlinsky Kraj / Okres Zlin / Otrokovice	Alexander Nagy (State Veterinary Institute Prague)
EPI_ISL_268949	A/mute swan/Czech Republic/1227-17/2017 (H5N8)	A / H5N8	Europe / Czech Republic / Zlinsky Kraj / Okres Uherske Hradiste / Uherské Hradišt?	Alexander Nagy (State Veterinary Institute Prague)
EPI_ISL_268950	A/mute swan/Czech Republic/1296-17_1/2017 (H5N8)	A / H5N8	Europe / Czech Republic / Olomoucky Kraj / Okres Prerov / Tova?ov Lakes	Alexander Nagy (State Veterinary Institute Prague)
EPI_ISL_268951	A/mute swan/Czech Republic/1330-17_1/2017 (H5N8)	A / H5N8	Europe / Czech Republic / Jihocesky Kraj / Okres Tabor / Roudná	Alexander Nagy (State Veterinary Institute Prague)
EPI_ISL_268952	A/mute swan/Czech Republic/1331-17_1/2017 (H5N8)	A / H5N8	Europe / Czech Republic / Jihocesky Kraj / Okres Tabor / Tu?apy	Alexander Nagy (State Veterinary Institute Prague)
EPI_ISL_268953	A/mute swan/Czech Republic/1337-17/2017 (H5N8)	A / H5N8	Europe / Czech Republic / Jihocesky Kraj / Okres Tabor / Veselí nad Lužnicí	Alexander Nagy (State Veterinary Institute Prague)
EPI_ISL_268954	A/mute swan/Czech Republic/1339-17/2017 (H5N8)	A / H5N8	Europe / Czech Republic / Morovskoslezsky Kraj / Okres Opava / Opava	Alexander Nagy (State Veterinary Institute Prague)
EPI_ISL_268955	A/chicken/Czech Republic/1344-17/2017 (H5N8)	A / H5N8	Europe / Czech Republic / Morovskoslezsky Kraj / Okres Ostrava-Mesto / Poruba	Alexander Nagy (State Veterinary Institute Prague)
EPI_ISL_268956	A/mute swan/Czech Republic/1461-17/2017 (H5N8)	A / H5N8	Europe / Czech Republic / Jihocesky Kraj / Okres Strakonice / Katovice	Alexander Nagy (State Veterinary Institute Prague)
EPI_ISL_268957	A/chicken/Czech Republic/1465-17/2017 (H5N8)	A / H5N8	Europe / Czech Republic / Jihocesky Kraj / Okres Ceske Budejovice / Ledenice	Alexander Nagy (State Veterinary Institute Prague)
EPI_ISL_268958	A/mute swan/Czech Republic/1519-17/2017 (H5N8)	A / H5N8	Europe / Czech Republic / Kralovehradecky Kraj / Okres Hradec Kralove / písník Kosi?ka	Alexander Nagy (State Veterinary Institute Prague)
EPI_ISL_268959	A/mute swan/Czech Republic/1576-17_C/2017 (H5N8)	A / H5N8	Europe / Czech Republic / Karlovarsky Kraj / Okres Cheb / Skalka pond	Alexander Nagy (State Veterinary Institute Prague)
EPI_ISL_268960	A/mallard/Czech Republic/1577-17/2017 (H5N8)	A / H5N8	Europe / Czech Republic / Jihocesky Kraj / Okres Jindrichuv Hradec / Zbuzany, near the bridge ac	Alexander Nagy (State Veterinary Institute Prague)
EPI_ISL_268961	A/mute swan/Czech Republic/1640-17/2017 (H5N8)	A / H5N8	Europe / Czech Republic / Ustecky Kraj / Okres Chomutov / Kada?, the Oh?e river, near the railw	Alexander Nagy (State Veterinary Institute Prague)
EPI_ISL_268962	A/mallard/Czech Republic/1672-17/2017 (H5N8)	A / H5N8	Europe / Czech Republic / Jihocesky Kraj / Okres Jindrichuv Hradec / Lásenice, the Nežárka river	Alexander Nagy (State Veterinary Institute Prague)
EPI_ISL_268963	A/chicken/Czech Republic/1675-17_2/2017 (H5N8)	A / H5N8	Europe / Czech Republic / Hlavni mesto Praha / Okres Praha / Kostelec nad Labem	Alexander Nagy (State Veterinary Institute Prague)
EPI_ISL_268964	A/grey heron/Czech Republic/1680-17_2/2017 (H5N8)	A / H5N8	Europe / Czech Republic / Jihocesky Kraj / Okres Jindrichuv Hradec / T?ebo?, Zlatá stoka	Alexander Nagy (State Veterinary Institute Prague)
EPI_ISL_268965	A/Indian Runner Duck/Czech Republic/1683-17_1/2017 (H5N8	A / H5N8	Europe / Czech Republic / Liberecky Kraj / Okres Liberec / Liberec	Alexander Nagy (State Veterinary Institute Prague)
EPI_ISL_268966	A/chicken/Czech Republic/1687-17_2/2017 (H5N8)	A / H5N8	Europe / Czech Republic / Morovskoslezsky Kraj / Okres Ostrava-Mesto / Orlová-Olmovec	Alexander Nagy (State Veterinary Institute Prague)
EPI_ISL_268967	A/chicken/Czech Republic/1688-17_1/2017 (H5N8)	A / H5N8	Europe / Czech Republic / Stredocesky Kraj / Okres Beroun / Hlásná T?ebá?	Alexander Nagy (State Veterinary Institute Prague)
EPI_ISL_268968	A/chicken/Czech Republic/1689-17/2017 (H5N8)	A / H5N8	Europe / Czech Republic / Pardubicky kraj / Okres Usti nad Orlici / Koldín-Hradišt?	Alexander Nagy (State Veterinary Institute Prague)
EPI_ISL_268969	A/mallard/Czech Republic/1690-17_2/2017 (H5N8)	A / H5N8	Europe / Czech Republic / Jihocesky Kraj / Okres Strakonice / Blatná	Alexander Nagy (State Veterinary Institute Prague)
EPI_ISL_268970	A/mute swan/Czech Republic/1691-17/2017 (H5N8)	A / H5N8	Europe / Czech Republic / Pardubicky kraj / Okres Pardubice / Pardubice, lake Bajkal	Alexander Nagy (State Veterinary Institute Prague)
EPI_ISL_268971	A/bronze turkey/Czech Republic/1755-17_1/2017 (H5N8)	A / H5N8	Europe / Czech Republic / Hlavni mesto Praha / Okres Praha / Touše?	Alexander Nagy (State Veterinary Institute Prague)
EPI_ISL_268972	A/turkey/Czech Republic/1767-17_2/2017 (H5N8)	A / H5N8	Europe / Czech Republic / Jihocesky Kraj / Okres Jindrichuv Hradec / Horní Lhota, Stráž nad Nežá	Alexander Nagy (State Veterinary Institute Prague)
EPI_ISL_268973	A/mute swan/Czech Republic/1848-17_1/2017 (H5N8)	A / H5N8	Europe / Czech Republic / Karlovarsky Kraj / Okres Karlovy Vary / Karlovy Vary, the Oh?e river	Alexander Nagy (State Veterinary Institute Prague)
EPI_ISL_268974	A/mute swan/Czech Republic/1848-17_2/2017 (H5N8)	A / H5N8	Europe / Czech Republic / Karlovarsky Kraj / Okres Karlovy Vary / Karlovy Vary, the Oh?e river	Alexander Nagy (State Veterinary Institute Prague)
EPI_ISL_268975	A/chicken/Czech Republic/1953-17/2017 (H5N8)	A / H5N8	Europe / Czech Republic / Liberecky Kraj / Okres Ceska Lipa / Velký Grunov	Alexander Nagy (State Veterinary Institute Prague)
EPI_ISL_268976	A/goose/Czech Republic/1954-17/2017 (H5N8)	A / H5N8	Europe / Czech Republic / Pardubicky kraj / Okres Pardubice / Chvaletice	Alexander Nagy (State Veterinary Institute Prague)
EPI_ISL_268977	A/goose/Czech Republic/1998-17_1/2017 (H5N8)	A / H5N8	Europe / Czech Republic / Kralovehradecky Kraj / Okres Hradec Kralove / Ž?ár nad Orlicí	Alexander Nagy (State Veterinary Institute Prague)
EPI_ISL_268978	A/mute swan/Czech Republic/2008-17_1/2017 (H5N8)	A / H5N8	Europe / Czech Republic / Morovskoslezsky Kraj / Okres Karvina / Závada nad Olší	Alexander Nagy (State Veterinary Institute Prague)
EPI_ISL_268979	A/quail/Czech Republic/2063-17_1/2017 (H5N8)	A / H5N8	Europe / Czech Republic / Jihocesky Kraj / Okres Strakonice / Volyn?	Alexander Nagy (State Veterinary Institute Prague)
EPI_ISL_268980	A/chicken/Czech Republic/2216-17_1/2017 (H5N8)	A / H5N8	Europe / Czech Republic / Jihocesky Kraj / Okres Strakonice / Volyn?	Alexander Nagy (State Veterinary Institute Prague)
EPI_ISL_268981	A/spot-billed pelican/Czech Republic/2270-17/2017 (H5N5)	A / H5N5	Europe / Czech Republic / Liberecky Kraj / Okres Liberec / ZOO Liberec	Alexander Nagy (State Veterinary Institute Prague)
EPI_ISL_268982	A/chicken/Czech Republic/2514-17/2017 (H5N8)	A / H5N8	Europe / Czech Republic / Morovskoslezsky Kraj / Okres Karvina / Doubrava	Alexander Nagy (State Veterinary Institute Prague)
EPI_ISL_268983	A/mallard/Czech Republic/2641-17/2017 (H5N8)	A / H5N8	Europe / Czech Republic / Vysocina Kraj / Okres Pelhrimov / Želiv	Alexander Nagy (State Veterinary Institute Prague)
EPI_ISL_268984	A/chicken/Czech Republic/2677-17_2/2017 (H5N8)	A / H5N8	Europe / Czech Republic / Kralovehradecky Kraj / Okres Hradec Kralove / Bernartice	Alexander Nagy (State Veterinary Institute Prague)
EPI_ISL_268985	A/mallard/Czech Republic/2678-17_1/2017 (H5N8)	A / H5N8	Europe / Czech Republic / Vysocina Kraj / Okres Havlickuv Brod / Havli?k?v Brod	Alexander Nagy (State Veterinary Institute Prague)
EPI_ISL_268986	A/mallard/Czech Republic/2820-17_1/2017 (H5N8)	A / H5N8	Europe / Czech Republic / Karlovarsky Kraj / Okres Cheb / Poustka	Alexander Nagy (State Veterinary Institute Prague)
EPI_ISL_268987	A/mallard/Czech Republic/2820-17_2/2017 (H5N8)	A / H5N8	Europe / Czech Republic / Karlovarsky Kraj / Okres Cheb / Poustka	Alexander Nagy (State Veterinary Institute Prague)
EPI_ISL_268988	A/mallard/Czech Republic/2821-17_1/2017 (H5N8)	A / H5N8	Europe / Czech Republic / Karlovarsky Kraj / Okres Cheb / Klest	Alexander Nagy (State Veterinary Institute Prague)
EPI_ISL_268989	A/chicken/Czech Republic/2821-17_2/2017 (H5N8)	A / H5N8	Europe / Czech Republic / Karlovarsky Kraj / Okres Cheb / Klest	Alexander Nagy (State Veterinary Institute Prague)
EPI_ISL_268990	A/mallard/Czech Republic/2822-17_1/2017 (H5N8)	A / H5N8	Europe / Czech Republic / Karlovarsky Kraj / Okres Cheb / Klest	Alexander Nagy (State Veterinary Institute Prague)
EPI_ISL_268991	A/chicken/Czech Republic/2822-17_2/2017 (H5N8)	A / H5N8	Europe / Czech Republic / Karlovarsky Kraj / Okres Cheb / Klest	Alexander Nagy (State Veterinary Institute Prague)
EPI_ISL_268992	A/chicken/Czech Republic/3507-17/2017 (H5N8)	A / H5N8	Europe / Czech Republic / Morovskoslezsky Kraj / Okres Frydek-Mistek / Dobrá	Alexander Nagy (State Veterinary Institute Prague)
EPI_ISL_268993	A/chicken/Czech Republic/4231-17_2/2017 (H5N8)	A / H5N7	Europe / Czech Republic / Karlovarsky Kraj / Okres Karlovy Vary / Otro?ín	Alexander Nagy (State Veterinary Institute Prague)
EPI_ISL_269591	A/Eurasian_Wigeon/Netherlands/6/2016	A / H5N8	Europe / Netherlands	Maria Johanna Poen (Erasmus Medical Center / Department of Virology)
EPI_ISL_269592	A/Eurasian_Wigeon/Netherlands/8/2016	A / H5N8	Europe / Netherlands	Maria Johanna Poen (Erasmus Medical Center / Department of Virology)
EPI_ISL_269593	A/Eurasian_Wigeon/Netherlands/11/2016	A / H5N8	Europe / Netherlands	Maria Johanna Poen (Erasmus Medical Center / Department of Virology)
EPI_ISL_269594	A/Eurasian_Wigeon/Netherlands/22/2016	A / H5N8	Europe / Netherlands	Maria Johanna Poen (Erasmus Medical Center / Department of Virology)
EPI_ISL_269595	A/Eurasian_Wigeon/Netherlands/12/2016	A / H5N8	Europe / Netherlands	Maria Johanna Poen (Erasmus Medical Center / Department of Virology)
EPI_ISL_269596	A/Eurasian_Wigeon/Netherlands/13/2016	A / H5N8	Europe / Netherlands	Maria Johanna Poen (Erasmus Medical Center / Department of Virology)
EPI_ISL_269597	A/Great_Black-backed_Gull/Netherlands/1/2016	A / H5N8	Europe / Netherlands	Maria Johanna Poen (Erasmus Medical Center / Department of Virology)
EPI_ISL_269598	A/Great_Black-backed_Gull/Netherlands/3/2016	A / H5N8	Europe / Netherlands	Maria Johanna Poen (Erasmus Medical Center / Department of Virology)
EPI_ISL_269599	A/Great_Black-backed_Gull/Netherlands/4/2016	A / H5N8	Europe / Netherlands	Maria Johanna Poen (Erasmus Medical Center / Department of Virology)
EPI_ISL_269600	A/Great_Crested_Grebe/Netherlands/2/2016	A / H5N8	Europe / Netherlands	Maria Johanna Poen (Erasmus Medical Center / Department of Virology)

EPI_ISL_269601	A/Eurasian_Herring_Gull/Netherlands/2/2016	A / H5N8	Europe / Netherlands	Maria Johanna Poen (Erasmus Medical Center / Department of Virology)
EPI_ISL_269602	A/Lesser_Black-backed_Gull/Netherlands/1/2016	A / H5N8	Europe / Netherlands	Maria Johanna Poen (Erasmus Medical Center / Department of Virology)
EPI_ISL_269603	A/Mallard/Netherlands/51/2016	A / H5N8	Europe / Netherlands	Maria Johanna Poen (Erasmus Medical Center / Department of Virology)
EPI_ISL_269604	A/Mallard/Netherlands/1/2017	A / H5N8	Europe / Netherlands	Maria Johanna Poen (Erasmus Medical Center / Department of Virology)
EPI_ISL_269692	A/Mallard/Netherlands/3/2017	A / H5N8	Europe / Netherlands	Maria Johanna Poen (Erasmus Medical Center / Department of Virology)
EPI_ISL_269693	A/Common_Pochard/Netherlands/1/2016	A / H5N8	Europe / Netherlands	Maria Johanna Poen (Erasmus Medical Center / Department of Virology)
EPI_ISL_269694	A/Eurasian_Wigeon/Netherlands/1/2016	A / H5N8	Europe / Netherlands	Maria Johanna Poen (Erasmus Medical Center / Department of Virology)
EPI_ISL_269695	A/Eurasian_Wigeon/Netherlands/21/2016	A / H5N8	Europe / Netherlands	Maria Johanna Poen (Erasmus Medical Center / Department of Virology)
EPI_ISL_269696	A/Eurasian_Wigeon/Netherlands/23/2016	A / H5N8	Europe / Netherlands / Provincie Noord-Holland	Maria Johanna Poen (Erasmus Medical Center / Department of Virology)
EPI_ISL_269703	A/Eurasian_Wigeon/Netherlands/25/2016	A / H5N8	Europe / Netherlands	Maria Johanna Poen (Erasmus Medical Center / Department of Virology)
EPI_ISL_270356	A/common teal/Shanghai/PD1108-3/2013	A / H5N8	Asia / China	
EPI_ISL_270357	A/common teal/Shanghai/PD1108-6/2013	A / H5N8	Asia / China	
EPI_ISL_270358	A/common teal/Shanghai/PD1108-13/2013	A / H5N8	Asia / China	
EPI_ISL_270359	A/common teal/Shanghai/PD1108-20/2013	A / H5N8	Asia / China	
EPI_ISL_270360	A/common teal/Shanghai/PD1108-24/2013	A / H5N8	Asia / China	
EPI_ISL_270361	A/common teal/Shanghai/PD1108-25/2013	A / H5N8	Asia / China	
EPI_ISL_270362	A/common teal/Shanghai/PD1108-27/2013	A / H5N8	Asia / China	
EPI_ISL_270363	A/common teal/Shanghai/PD1118-1/2013	A / H5N8	Asia / China	
EPI_ISL_270364	A/common teal/Shanghai/PD1121-10/2013	A / H5N8	Asia / China	
EPI_ISL_270365	A/common teal/Shanghai/PD1121-15/2013	A / H5N8	Asia / China	
EPI_ISL_270366	A/common teal/Shanghai/PD1121-19/2013	A / H5N8	Asia / China	
EPI_ISL_270367	A/common teal/Shanghai/PD1202-9/2013	A / H5N8	Asia / China	
EPI_ISL_270368	A/common teal/Shanghai/PD1121-18/2013	A / H5N8	Asia / China	
EPI_ISL_270369	A/mallard/Shanghai/SH-9/2013	A / H5N8	Asia / China	
EPI_ISL_270370	A/falcated duck/Shanghai/PD1121-26/2013	A / H5N8	Asia / China	
EPI_ISL_270371	A/falcated duck/Shanghai/PD1121-27/2013	A / H5N8	Asia / China	
EPI_ISL_270372	A/spot-billed duck/Shanghai/PD1202-3/2013	A / H5N8	Asia / China	
EPI_ISL_2713170	A/tundra swan/Hubei/BQ9/2020	A / H5N8	Asia / China / Hubei Province	Hongliang Chai (Northeast Forestry University / College of Wildlife Resources)
EPI_ISL_2713314	A/whiskered tern/Hubei/BQ10/2020	A / H5N8	Asia / China / Hubei Province	Hongliang Chai (Northeast Forestry University / College of Wildlife Resources)
EPI_ISL_2713372	A/bean goose/Hubei/BQ11/2020	A / H5N8	Asia / China / Hubei Province	Hongliang Chai (Northeast Forestry University / College of Wildlife Resources)
EPI_ISL_271704	A/Goose/Hungary/17261/2017	A / H5N8	Europe / Hungary / Bacs-Kiskun	Adam Dan (Danam.Vet.Molbiol)
EPI_ISL_271705	A/Goose/Hungary/15729/2017	A / H5N8	Europe / Hungary / Bacs-Kiskun	Adam Dan (Danam.Vet.Molbiol)
EPI_ISL_271706	A/Goose/Hungary/17051/2017	A / H5N8	Europe / Hungary / Bacs-Kiskun	Adam Dan (Danam.Vet.Molbiol)
EPI_ISL_271707	A/Goose/Hungary/17580/2017	A / H5N8	Europe / Hungary / Bacs-Kiskun	Adam Dan (Danam.Vet.Molbiol)
EPI_ISL_271708	A/Goose/Hungary/17985/2017	A / H5N8	Europe / Hungary / Bacs-Kiskun	Adam Dan (Danam.Vet.Molbiol)
EPI_ISL_271709	A/Goose/Hungary/59763/2016	A / H5N8	Europe / Hungary / Bacs-Kiskun	Adam Dan (Danam.Vet.Molbiol)
EPI_ISL_271710	A/Mulard_duck/Hungary/60369/2016	A / H5N8	Europe / Hungary / Bacs-Kiskun	Adam Dan (Danam.Vet.Molbiol)
EPI_ISL_271711	A/Mulard_duck/Hungary/62902/2016	A / H5N8	Europe / Hungary / Csongrad megye / Csongrad	Adam Dan (Danam.Vet.Molbiol)
EPI_ISL_271712	A/Goose/Hungary/63743/2016	A / H5N8	Europe / Hungary / Bacs-Kiskun	Adam Dan (Danam.Vet.Molbiol)
EPI_ISL_271713	A/Goose/Hungary/64909/2016	A / H5N8	Europe / Hungary / Bacs-Kiskun	Adam Dan (Danam.Vet.Molbiol)
EPI_ISL_271714	A/Mulard_duck/Hungary/59163/2016	A / H5N8	Europe / Hungary / Bacs-Kiskun	Adam Dan (Danam.Vet.Molbiol)
EPI_ISL_272740	A/goose/Guangdong/QY01/2016	A / H5N6	Asia / China	
EPI_ISL_272859	A/grey heron/W779/2017	A / H5N8	Asia / Korea, Republic of	
EPI_ISL_273359	A/duck/Vietnam/LBM835/2015	A / H5N6	Asia / Vietnam	
EPI_ISL_273360	A/duck/Vietnam/LBM806/2015	A / H5N6	Asia / Vietnam	
EPI_ISL_273846	A/chicken/Italy/17VIR3078/2017	A / H5N8	Europe / Italy / Emilia-Romagna / Province of Bologna / Mordano	Bianca Zecchin (Istituto Zooprofilattico Sperimentale Delle Venezie)
EPI_ISL_273847	A/turkey/Italy/17VIR5878-3/2017	A / H5N8	Europe / Italy / Veneto / Province of Verona / Albaredo d'Adige	Bianca Zecchin (Istituto Zooprofilattico Sperimentale Delle Venezie)
EPI_ISL_274858	A/mute swan/Kaliningrad/132/2017	A / H5N8	Europe / Russian Federation / Kaliningrad Oblast	Ivan Susloparov (State Research Center of Virology and Biotechnology (VECTOR) / Emerging Zoonotic Diseases and Influenza)
EPI_ISL_275283	A/chicken/Shchyolkovo/47/2017	A / H5N8	Europe / Russian Federation / Moscow Oblast	Ivan Susloparov (State Research Center of Virology and Biotechnology (VECTOR) / Emerging Zoonotic Diseases and Influenza)
EPI_ISL_275287	A/chicken/Rostov-on-Don/44/2017	A / H5N8	Europe / Russian Federation / Rostov Oblast	Ivan Susloparov (State Research Center of Virology and Biotechnology (VECTOR) / Emerging Zoonotic Diseases and Influenza)
EPI_ISL_275288	A/chicken/Tatarstan/88/2017	A / H5N8	Europe / Russian Federation / Republic of Tatarstan	Ivan Susloparov (State Research Center of Virology and Biotechnology (VECTOR) / Emerging Zoonotic Diseases and Influenza)
EPI_ISL_277035	A/chicken/Wuxi/6074/2015	A / H5N2	Asia / China / Jiangsu	Maijuan Ma (Beijing Institute of Microbiology and Epidemiology / State Key Laboratory of Pathogen and Biosecurity)
EPI_ISL_277036	A/duck/Wuxi/6462/2015	A / H5N2	Asia / China / Jiangsu	Maijuan Ma (Beijing Institute of Microbiology and Epidemiology / State Key Laboratory of Pathogen and Biosecurity)
EPI_ISL_277037	A/duck/Wuxi/6466/2015	A / H5N2	Asia / China / Jiangsu	Maijuan Ma (Beijing Institute of Microbiology and Epidemiology / State Key Laboratory of Pathogen and Biosecurity)
EPI_ISL_277040	A/chicken/Wuxi/7157/2015	A / H5N6	Asia / China / Jiangsu	Maijuan Ma (Beijing Institute of Microbiology and Epidemiology / State Key Laboratory of Pathogen and Biosecurity)
EPI_ISL_277041	A/duck/Wuxi/7249/2015	A / H5N6	Asia / China / Jiangsu	Maijuan Ma (Beijing Institute of Microbiology and Epidemiology / State Key Laboratory of Pathogen and Biosecurity)
EPI_ISL_277042	A/chicken/Wuxi/7765/2016	A / H5N6	Asia / China / Jiangsu	Maijuan Ma (Beijing Institute of Microbiology and Epidemiology / State Key Laboratory of Pathogen and Biosecurity)
EPI_ISL_278025	A/Duck/Zhanjiang/15577/2015	A / H5N6	Asia / China / Guangdong Province / Zhanjiang	Jia Weixin (South China Agricultural University / Veterinary Medicine College)
EPI_ISL_278026	A/Chicken/Huizhou/16274/2016(H5N6)	A / H5N6	Asia / China / Guangdong Province / Huizhou	Jia Weixin (South China Agricultural University / Veterinary Medicine College)
EPI_ISL_278027	A/Goose/Qingyuan/16875/2016	A / H5N6	Asia / China / Guangdong Province / Qingyuan	Jia Weixin (South China Agricultural University / Veterinary Medicine College)
EPI_ISL_278031	A/Quail/Zhanjiang/16887/2016	A / H5N6	Asia / China / Guangdong Province / Zhanjiang	Jia Weixin (South China Agricultural University / Veterinary Medicine College)
EPI_ISL_278040	A/peregrine falcon/HK/4955/2015	A / H5N6	Asia / Hong Kong (SAR)	
EPI_ISL_278041	A/oriental magpie robin/HK/6154/2015	A / H5N6	Asia / Hong Kong (SAR)	
EPI_ISL_278200	A/Brahma chicken/Belgium/6153/2017	A / H5N8	Europe / Belgium	
EPI_ISL_278201	A/chicken/Belgium/5990/2017	A / H5N8	Europe / Belgium	
EPI_ISL_278204	A/cat/Zhejiang/C1/2016	A / H5N6	Asia / China	
EPI_ISL_278205	A/cat/Zhejiang/C2/2016	A / H5N6	Asia / China	
EPI_ISL_279010	A/crane/Kagoshima/KU-6/2016	A / H5N6	Asia / Japan / Kagoshima	Makoto Ozawa (Kagoshima University)
EPI_ISL_279011	A/crane/Kagoshima/KU-8/2016	A / H5N6	Asia / Japan / Kagoshima	Makoto Ozawa (Kagoshima University)
EPI_ISL_279012	A/crane/Kagoshima/KU-9/2016	A / H5N6	Asia / Japan / Kagoshima	Makoto Ozawa (Kagoshima University)
EPI_ISL_279013	A/duck/Kagoshima/KU-d35/2016	A / H5N6	Asia / Japan / Kagoshima	Makoto Ozawa (Kagoshima University)
EPI_ISL_279014	A/duck/Kagoshima/KU-d42/2016	A / H5N6	Asia / Japan / Kagoshima	Makoto Ozawa (Kagoshima University)

EPI_ISL_279015	A/duck/Kagoshima/KU-d66/2016	A / H5N6	Asia / Japan / Kagoshima	Makoto Ozawa (Kagoshima University)
EPI_ISL_279016	A/duck/Kagoshima/KU-d79/2016	A / H5N6	Asia / Japan / Kagoshima	Makoto Ozawa (Kagoshima University)
EPI_ISL_279017	A/crane/Kagoshima/KU-10/2016	A / H5N6	Asia / Japan / Kagoshima	Makoto Ozawa (Kagoshima University)
EPI_ISL_279018	A/crane/Kagoshima/KU-12/2016	A / H5N6	Asia / Japan / Kagoshima	Makoto Ozawa (Kagoshima University)
EPI_ISL_279019	A/crane/Kagoshima/KU-15/2016	A / H5N6	Asia / Japan / Kagoshima	Makoto Ozawa (Kagoshima University)
EPI_ISL_279020	A/crane/Kagoshima/KU-16/2016	A / H5N6	Asia / Japan / Kagoshima	Makoto Ozawa (Kagoshima University)
EPI_ISL_279021	A/northern pintail/Kagoshima/KU-23/2016	A / H5N6	Asia / Japan / Kagoshima	Makoto Ozawa (Kagoshima University)
EPI_ISL_279022	A/crane/Kagoshima/KU-14/2016	A / H5N6	Asia / Japan / Kagoshima	Makoto Ozawa (Kagoshima University)
EPI_ISL_279023	A/eurasian wigeon/Kagoshima/KU-20/2016	A / H5N6	Asia / Japan / Kagoshima	Makoto Ozawa (Kagoshima University)
EPI_ISL_279024	A/eurasian wigeon/Kagoshima/KU-21/2016	A / H5N6	Asia / Japan / Kagoshima	Makoto Ozawa (Kagoshima University)
EPI_ISL_279025	A/crane/Kagoshima/KU-25/2016	A / H5N6	Asia / Japan / Kagoshima	Makoto Ozawa (Kagoshima University)
EPI_ISL_279026	A/crane/Kagoshima/KU-26/2016	A / H5N6	Asia / Japan / Kagoshima	Makoto Ozawa (Kagoshima University)
EPI_ISL_279027	A/crane/Kagoshima/KU-27/2016	A / H5N6	Asia / Japan / Kagoshima	Makoto Ozawa (Kagoshima University)
EPI_ISL_279028	A/crane/Kagoshima/KU-28/2016	A / H5N6	Asia / Japan / Kagoshima	Makoto Ozawa (Kagoshima University)
EPI_ISL_279029	A/eurasian wigeon/Kagoshima/KU-32/2016	A / H5N6	Asia / Japan / Kagoshima	Makoto Ozawa (Kagoshima University)
EPI_ISL_279030	A/crane/Kagoshima/KU-31/2016	A / H5N6	Asia / Japan / Kagoshima	Makoto Ozawa (Kagoshima University)
EPI_ISL_279031	A/crane/Kagoshima/KU-34/2016	A / H5N6	Asia / Japan / Kagoshima	Makoto Ozawa (Kagoshima University)
EPI_ISL_279032	A/crane/Kagoshima/KU-33/2016	A / H5N6	Asia / Japan / Kagoshima	Makoto Ozawa (Kagoshima University)
EPI_ISL_279033	A/crane/Kagoshima/KU-37/2016	A / H5N6	Asia / Japan / Kagoshima	Makoto Ozawa (Kagoshima University)
EPI_ISL_279034	A/crane/Kagoshima/KU-43/2016	A / H5N6	Asia / Japan / Kagoshima	Makoto Ozawa (Kagoshima University)
EPI_ISL_279035	A/crane/Kagoshima/KU-44/2016	A / H5N6	Asia / Japan / Kagoshima	Makoto Ozawa (Kagoshima University)
EPI_ISL_279036	A/crane/Kagoshima/KU-45/2016	A / H5N6	Asia / Japan / Kagoshima	Makoto Ozawa (Kagoshima University)
EPI_ISL_279037	A/crane/Kagoshima/KU-46/2016	A / H5N6	Asia / Japan / Kagoshima	Makoto Ozawa (Kagoshima University)
EPI_ISL_279038	A/crane/Kagoshima/KU-48/2016	A / H5N6	Asia / Japan / Kagoshima	Makoto Ozawa (Kagoshima University)
EPI_ISL_279039	A/crane/Kagoshima/KU-52/2016	A / H5N6	Asia / Japan / Kagoshima	Makoto Ozawa (Kagoshima University)
EPI_ISL_279040	A/crane/Kagoshima/KU-53/2016	A / H5N6	Asia / Japan / Kagoshima	Makoto Ozawa (Kagoshima University)
EPI_ISL_279178	A/duck/Zhejiang/1026109/2015	A / H5N2	Asia / China	
EPI_ISL_279179	A/chicken/Zhejiang/81643/2015	A / H5N2	Asia / China	
EPI_ISL_279180	A/chicken/Zhejiang/7450/2015	A / H5N2	Asia / China	
EPI_ISL_279181	A/chicken/Zhejiang/514135/2015	A / H5N2	Asia / China	
EPI_ISL_279424	A/wildfowl/Shandong/SD01/2015	A / H5N6	Asia / China	
EPI_ISL_279790	A/wildfowl/Shandong/SD04/2016	A / H5N8	Asia / China	
EPI_ISL_279791	A/wildfowl/Shandong/SD02/2015	A / H5N2	Asia / China	
EPI_ISL_280485	A/common teal/Shanghai/JDS36/2016	A / H5N6	Asia / China	
EPI_ISL_280486	A/common teal/Shanghai/JDS66/2016	A / H5N6	Asia / China	
EPI_ISL_280487	A/common teal/Shanghai/JDS76/2016	A / H5N6	Asia / China	
EPI_ISL_280488	A/common teal/Shanghai/JDS81/2016	A / H5N6	Asia / China	
EPI_ISL_280489	A/Eurasian wigeon/Shanghai/JDS101/2016	A / H5N6	Asia / China	
EPI_ISL_280490	A/mandarin duck/Shanghai/PD4/2016	A / H5N6	Asia / China	
EPI_ISL_280491	A/shoveller duck/Shanghai/JDS90/2016	A / H5N6	Asia / China	
EPI_ISL_280674	A/mute_swan/Switzerland/V0244.2-L02307/2017	A / H5N8	Europe / Switzerland / Yverdon-les-Bains	Anne Pohlmann (Friedrich-Loeffler-Institut)
EPI_ISL_2815336	A/common teal/Ningxia/105/2020	A / H5N8	Asia / China / Ningxia Hui Autonomous Region	Hongliang Chai (Northeast Forestry University / College of Wildlife Resources)
EPI_ISL_2815374	A/mallard/Ningxia/175/2020	A / H5N8	Asia / China / Ningxia Hui Autonomous Region	Hongliang Chai (Northeast Forestry University / College of Wildlife Resources)
EPI_ISL_2820250	A/mallard/Ningxia/176/2020	A / H5N8	Asia / China / Ningxia Hui Autonomous Region	Hongliang Chai (Northeast Forestry University / College of Wildlife Resources)
EPI_ISL_2820259	A/common teal/Ningxia/181/2020	A / H5N8	Asia / China / Ningxia Hui Autonomous Region	Hongliang Chai (Northeast Forestry University / College of Wildlife Resources)
EPI_ISL_2820261	A/common teal/Ningxia/189/2020	A / H5N8	Asia / China / Ningxia Hui Autonomous Region	Hongliang Chai (Northeast Forestry University / College of Wildlife Resources)
EPI_ISL_2820262	A/common teal/Ningxia/237/2020	A / H5N8	Asia / China / Ningxia Hui Autonomous Region	Hongliang Chai (Northeast Forestry University / College of Wildlife Resources)
EPI_ISL_2820288	A/mallard/Ningxia/239/2020	A / H5N8	Asia / China / Ningxia Hui Autonomous Region	Hongliang Chai (Northeast Forestry University / College of Wildlife Resources)
EPI_ISL_2820465	A/mallard/Ningxia/241/2020	A / H5N8	Asia / China / Ningxia Hui Autonomous Region	Hongliang Chai (Northeast Forestry University / College of Wildlife Resources)
EPI_ISL_2820479	A/common pochard/Ningxia/243/2020	A / H5N8	Asia / China / Ningxia Hui Autonomous Region	Hongliang Chai (Northeast Forestry University / College of Wildlife Resources)
EPI_ISL_2820480	A/common teal/Ningxia/245/2020	A / H5N8	Asia / China / Ningxia Hui Autonomous Region	Hongliang Chai (Northeast Forestry University / College of Wildlife Resources)
EPI_ISL_2820501	A/mallard/Ningxia/247/2020	A / H5N8	Asia / China / Ningxia Hui Autonomous Region	Hongliang Chai (Northeast Forestry University / College of Wildlife Resources)
EPI_ISL_2820503	A/mallard/Ningxia/249/2020	A / H5N8	Asia / China / Ningxia Hui Autonomous Region	Hongliang Chai (Northeast Forestry University / College of Wildlife Resources)
EPI_ISL_2820504	A/common teal/Ningxia/253/2020	A / H5N8	Asia / China / Ningxia Hui Autonomous Region	Hongliang Chai (Northeast Forestry University / College of Wildlife Resources)
EPI_ISL_282133	A/turkey/Czech Republic/38-17_1/2017 (H5N8)	A / H5N8	Europe / Czech Republic / South Moravian Region / Okres Brno-Venkov / Ivan?ice-N?m?ice	Alexander Nagy (State Veterinary Institute Prague)
EPI_ISL_282141	A/swan/Italy/17VIR7064-1/2017	A / H5N8	Europe / Italy / Lombardy / Province of Bergamo / Mozzanica	Bianca Zecchin (Istituto Zooprofilattico Sperimentale Delle Venezie)
EPI_ISL_282143	A/goose/Italy/17VIR6358-3/2017	A / H5N8	Europe / Italy / Lombardy / Province of Pavia / Chignolo Po	Bianca Zecchin (Istituto Zooprofilattico Sperimentale Delle Venezie)
EPI_ISL_282394	A/goose/Yangzhou/YZ587/2016	A / H5N6	Asia / China	
EPI_ISL_282395	A/chicken/Xuzhou/XZ6/2016	A / H5N6	Asia / China	
EPI_ISL_282396	A/duck/Anhui/S4/2016	A / H5N6	Asia / China	
EPI_ISL_282397	A/chicken/Guangdong/GD1602/2016	A / H5N6	Asia / China	
EPI_ISL_282408	A/poultry/China/XY918.4/2016	A / H5N6	Asia / China	
EPI_ISL_283129	A/chicken/Czech Republic/988-17/2017 (H5N8)	A / H5N8	Europe / Czech Republic / Olomoucky Kraj / Okres Prerov / Lov?šice	Alexander Nagy (State Veterinary Institute Prague)
EPI_ISL_283702	A/Duck/Yunnan/YN-2/2016 (H5N6)	A / H5N6	Asia / China / Yunnan Province	Qi Wenbao (South China Agricultural University / Veterinary Medicine College)
EPI_ISL_283703	A/Chicken/Yunnan/YN-3/2016 (H5N6)	A / H5N6	Asia / China / Yunnan Province	Qi Wenbao (South China Agricultural University / Veterinary Medicine College)
EPI_ISL_283705	A/Chicken/Yunnan/YN-7/2016 (H5N6)	A / H5N6	Asia / China / Yunnan Province	Qi Wenbao (South China Agricultural University / Veterinary Medicine College)
EPI_ISL_283965	A/Chicken/Yunnan/YN-8/2016 (H5N6)	A / H5N6	Asia / China / Yunnan Province	Qi Wenbao (South China Agricultural University / Veterinary Medicine College)
EPI_ISL_283966	A/Duck/Yunnan/YN-9/2016 (H5N6)	A / H5N6	Asia / China / Yunnan Province	Qi Wenbao (South China Agricultural University / Veterinary Medicine College)
EPI_ISL_283967	A/Chicken/Yunnan/YN-10/2015 (H5N6)	A / H5N6	Asia / China / Yunnan Province	Qi Wenbao (South China Agricultural University / Veterinary Medicine College)
EPI_ISL_283968	A/Chicken/Yunnan/YN-11/2016 (H5N6)	A / H5N6	Asia / China / Yunnan Province	Qi Wenbao (South China Agricultural University / Veterinary Medicine College)
EPI_ISL_283969	A/Chicken/Yunnan/YN-12/2016 (H5N6)	A / H5N6	Asia / China / Yunnan Province	Qi Wenbao (South China Agricultural University / Veterinary Medicine College)
EPI_ISL_283970	A/Ostrich/Guangxi/GX-1/2017 (H5N6)	A / H5N6	Asia / China / Guangxi Zhuang Autonomous Region	Qi Wenbao (South China Agricultural University / Veterinary Medicine College)

EPI_ISL_283971	A/Quail/Guangxi/GX-2/2017 (H5N6)	A / H5N6	Asia / China / Guangxi Zhuang Autonomous Region	Qi Wenbao (South China Agricultural University / Veterinary Medicine College)
EPI_ISL_284004	A/Chicken/South_Africa/S2017/08_0336_P3/2017	A / H5N8	Africa / South Africa / Province of the Western Cape	Florette Kathleen Treurnicht (National Institute for Communicable Diseases (NICD) / Center for Respiratory Diseases and Meningitis)
EPI_ISL_284196	A/Pigeon/South_Africa/S2017/08_0324_24/2017	A / H5N8	Africa / South Africa / Province of the Western Cape	Florette Kathleen Treurnicht (National Institute for Communicable Diseases (NICD) / Center for Respiratory Diseases and Meningitis)
EPI_ISL_284197	A/Ostrich/South_Africa/S2017/08_0046_P2/2017	A / H5N8	Africa / South Africa / Province of the Western Cape	Florette Kathleen Treurnicht (National Institute for Communicable Diseases (NICD) / Center for Respiratory Diseases and Meningitis)
EPI_ISL_284198	A/Ostrich/South_Africa/S2017/08_0361_P17/2017	A / H5N8	Africa / South Africa / Province of the Western Cape	Florette Kathleen Treurnicht (National Institute for Communicable Diseases (NICD) / Center for Respiratory Diseases and Meningitis)
EPI_ISL_284650	A/Anhui/33162/2016	A / H5N6	Asia / China / Anhui Province	Lei Yang (WHO Chinese National Influenza Center / Virology Institute, Chinese CDC)
EPI_ISL_284651	A/Anhui/33163/2016	A / H5N6	Asia / China / Anhui Province	Lei Yang (WHO Chinese National Influenza Center / Virology Institute, Chinese CDC)
EPI_ISL_284683	A/Ostrich/South_Africa/S2017/08_0268_P2/2017	A / H5N8	Africa / South Africa / Province of the Western Cape	Florette Kathleen Treurnicht (National Institute for Communicable Diseases (NICD) / Center for Respiratory Diseases and Meningitis)
EPI_ISL_284684	A/Ostrich/South_Africa/S2017/08_0268_P9/2017	A / H5N8	Africa / South Africa / Province of the Western Cape	Florette Kathleen Treurnicht (National Institute for Communicable Diseases (NICD) / Center for Respiratory Diseases and Meningitis)
EPI_ISL_284692	A/Geese/South_Africa/S2017/09_0055_P2/2017	A / H5N8	Africa / South Africa / Province of the Western Cape	Florette Kathleen Treurnicht (National Institute for Communicable Diseases (NICD) / Center for Respiratory Diseases and Meningitis)
EPI_ISL_285480	A/Ostrich/South_Africa/S2017/08_0362_P10/2017	A / H5N8	Africa / South Africa / Province of the Western Cape	Florette Kathleen Treurnicht (National Institute for Communicable Diseases (NICD) / Center for Respiratory Diseases and Meningitis)
EPI_ISL_285488	A/Ostrich/South_Africa/S2017/08_0047_P7/2017	A / H5N8	Africa / South Africa / Province of the Western Cape	Florette Kathleen Treurnicht (National Institute for Communicable Diseases (NICD) / Center for Respiratory Diseases and Meningitis)
EPI_ISL_285509	A/Ostrich/South_Africa/S2017/08_0362_P11/2017	A / H5N8	Africa / South Africa / Province of the Western Cape	Florette Kathleen Treurnicht (National Institute for Communicable Diseases (NICD) / Center for Respiratory Diseases and Meningitis)
EPI_ISL_285512	A/Chicken/South_Africa/S2017/08_0336_P1/2017	A / H5N8	Africa / South Africa / Province of the Western Cape	Florette Kathleen Treurnicht (National Institute for Communicable Diseases (NICD) / Center for Respiratory Diseases and Meningitis)
EPI_ISL_285601	A/Geese/South_Africa/S2017/09_0065_P1/2017	A / H5N8	Africa / South Africa / Province of the Western Cape	Florette Kathleen Treurnicht (National Institute for Communicable Diseases (NICD) / Center for Respiratory Diseases and Meningitis)
EPI_ISL_285602	A/Geese/South_Africa/S2017/09_0065_P2/2017	A / H5N8	Africa / South Africa / Province of the Western Cape	Florette Kathleen Treurnicht (National Institute for Communicable Diseases (NICD) / Center for Respiratory Diseases and Meningitis)
EPI_ISL_285604	A/Ostrich/South_Africa/S2017/08_0046_P3/2017	A / H5N8	Africa / South Africa / Province of the Western Cape	Florette Kathleen Treurnicht (National Institute for Communicable Diseases (NICD) / Center for Respiratory Diseases and Meningitis)
EPI_ISL_285606	A/Duck/South_Africa/S2017/08_0340_P1/2017	A / H5N8	Africa / South Africa / Province of the Western Cape	Florette Kathleen Treurnicht (National Institute for Communicable Diseases (NICD) / Center for Respiratory Diseases and Meningitis)
EPI_ISL_285607	A/Duck/South_Africa/S2017/08_0340_P2/2017	A / H5N8	Africa / South Africa / Province of the Western Cape	Florette Kathleen Treurnicht (National Institute for Communicable Diseases (NICD) / Center for Respiratory Diseases and Meningitis)
EPI_ISL_285608	A/Ostrich/South_Africa/S2017/08_0362_P8_33/2017	A / H5N8	Africa / South Africa / Province of the Western Cape	Florette Kathleen Treurnicht (National Institute for Communicable Diseases (NICD) / Center for Respiratory Diseases and Meningitis)
EPI_ISL_285609	A/Geese/South_Africa/S2017/08_0558_P2/2017	A / H5N8	Africa / South Africa / Province of the Western Cape	Florette Kathleen Treurnicht (National Institute for Communicable Diseases (NICD) / Center for Respiratory Diseases and Meningitis)
EPI_ISL_285610	A/Guineafowl/South_Africa/S2017/08_0274_P1/2017	A / H5N8	Africa / South Africa / Province of the Western Cape	Florette Kathleen Treurnicht (National Institute for Communicable Diseases (NICD) / Center for Respiratory Diseases and Meningitis)
EPI_ISL_285611	A/Wildbirds_Guineafowl_Makou_Egyptian Geese/South_Africa	A / H5N8	Africa / South Africa / Province of the Western Cape	Florette Kathleen Treurnicht (National Institute for Communicable Diseases (NICD) / Center for Respiratory Diseases and Meningitis)
EPI_ISL_285616	A/Ostrich/South_Africa/S2017/08_0161_P8/2017	A / H5N8	Africa / South Africa / Province of the Western Cape	Florette Kathleen Treurnicht (National Institute for Communicable Diseases (NICD) / Center for Respiratory Diseases and Meningitis)
EPI_ISL_285617	A/Guineafowl/South_Africa/S2017/08_0243_P1/2017	A / H5N8	Africa / South Africa / Province of the Western Cape	Florette Kathleen Treurnicht (National Institute for Communicable Diseases (NICD) / Center for Respiratory Diseases and Meningitis)
EPI_ISL_285618	A/Ostrich/South_Africa/S2017/08_0362_P7/2017	A / H5N8	Africa / South Africa / Province of the Western Cape	Florette Kathleen Treurnicht (National Institute for Communicable Diseases (NICD) / Center for Respiratory Diseases and Meningitis)
EPI_ISL_285619	A/chicken/Egypt/Kafr-Elshiekh-18/2017	A / H5N8	Africa / Egypt / Kafr-Elshiekh	El-Sayed M. Abdelwhab (Friedrich-Loeffler-Institut)
EPI_ISL_285620	A/chicken/Egypt/Buheira-12/2017	A / H5N8	Africa / Egypt / Buheira	El-Sayed M. Abdelwhab (Friedrich-Loeffler-Institut)
EPI_ISL_285621	A/chicken/Egypt/Gharbiya-15/2017	A / H5N8	Africa / Egypt / Gharbiya	El-Sayed M. Abdelwhab (Friedrich-Loeffler-Institut)
EPI_ISL_285623	A/Chicken/South_Africa/S2017/08_0416_38/2017	A / H5N8	Africa / South Africa / Province of the Western Cape	Florette Kathleen Treurnicht (National Institute for Communicable Diseases (NICD) / Center for Respiratory Diseases and Meningitis)
EPI_ISL_285624	A/Chicken/South_Africa/S2017/09_0050_56/2017	A / H5N8	Africa / South Africa / Province of the Western Cape	Florette Kathleen Treurnicht (National Institute for Communicable Diseases (NICD) / Center for Respiratory Diseases and Meningitis)
EPI_ISL_285625	A/Duck/South_Africa/S2017/08_0481_P2/2017	A / H5N8	Africa / South Africa / Province of the Western Cape	Florette Kathleen Treurnicht (National Institute for Communicable Diseases (NICD) / Center for Respiratory Diseases and Meningitis)
EPI_ISL_285627	A/Swan/South_Africa/S2017/08_0517_P2/2017	A / H5N8	Africa / South Africa / Province of the Western Cape	Florette Kathleen Treurnicht (National Institute for Communicable Diseases (NICD) / Center for Respiratory Diseases and Meningitis)
EPI_ISL_285647	A/Geese/South_Africa/S2017/08_0520_46/2017	A / H5N8	Africa / South Africa / Province of the Western Cape	Florette Kathleen Treurnicht (National Institute for Communicable Diseases (NICD) / Center for Respiratory Diseases and Meningitis)
EPI_ISL_285648	A/Geese/South_Africa/S2017/09_0055_P1/2017	A / H5N8	Africa / South Africa / Province of the Western Cape	Florette Kathleen Treurnicht (National Institute for Communicable Diseases (NICD) / Center for Respiratory Diseases and Meningitis)
EPI_ISL_285649	A/Chicken/South_Africa/S2017/09_0184_63/2017	A / H5N8	Africa / South Africa / Province of the Western Cape	Florette Kathleen Treurnicht (National Institute for Communicable Diseases (NICD) / Center for Respiratory Diseases and Meningitis)
EPI_ISL_285650	A/Ostrich/South_Africa/S2017/08_0046_AF/2017	A / H5N8	Africa / South Africa / Province of the Western Cape	Florette Kathleen Treurnicht (National Institute for Communicable Diseases (NICD) / Center for Respiratory Diseases and Meningitis)
EPI_ISL_285651	A/Guineafowl/South_Africa/S2017/08_0190_9/2017	A / H5N8	Africa / South Africa / Province of the Western Cape	Florette Kathleen Treurnicht (National Institute for Communicable Diseases (NICD) / Center for Respiratory Diseases and Meningitis)
EPI_ISL_285652	A/Guineafowl/South_Africa/S2017/08_0243_P2/2017	A / H5N8	Africa / South Africa / Province of the Western Cape	Florette Kathleen Treurnicht (National Institute for Communicable Diseases (NICD) / Center for Respiratory Diseases and Meningitis)
EPI_ISL_285653	A/Pigeon/South_Africa/S2017/08_0323_P1/2017	A / H5N8	Africa / South Africa / Province of the Western Cape	Florette Kathleen Treurnicht (National Institute for Communicable Diseases (NICD) / Center for Respiratory Diseases and Meningitis)
EPI_ISL_285655	A/Geese/South_Africa/S2017/08_0558_P1/2017	A / H5N8	Africa / South Africa / Province of the Western Cape	Florette Kathleen Treurnicht (National Institute for Communicable Diseases (NICD) / Center for Respiratory Diseases and Meningitis)
EPI_ISL_285657	A/Chicken/South_Africa/S2017/08_0561_P2/2017	A / H5N8	Africa / South Africa / Province of the Western Cape	Florette Kathleen Treurnicht (National Institute for Communicable Diseases (NICD) / Center for Respiratory Diseases and Meningitis)
EPI_ISL_285658	A/Chicken/South_Africa/S2017/08_0561_P1/2017	A / H5N8	Africa / South Africa / Province of the Western Cape	Florette Kathleen Treurnicht (National Institute for Communicable Diseases (NICD) / Center for Respiratory Diseases and Meningitis)
EPI_ISL_285915	A/Chicken/South_Africa/S2017/08_0581_P1/2017	A / H5N8	Africa / South Africa / Province of the Western Cape	Florette Kathleen Treurnicht (National Institute for Communicable Diseases (NICD) / Center for Respiratory Diseases and Meningitis)
EPI_ISL_285916	A/Chicken/South_Africa/S2017/08_0581_P2/2017	A / H5N8	Africa / South Africa / Province of the Western Cape	Florette Kathleen Treurnicht (National Institute for Communicable Diseases (NICD) / Center for Respiratory Diseases and Meningitis)
EPI_ISL_285917	A/Chicken/South_Africa/S2017/09_0184_62/2017	A / H5N8	Africa / South Africa / Province of the Western Cape	Florette Kathleen Treurnicht (National Institute for Communicable Diseases (NICD) / Center for Respiratory Diseases and Meningitis)
EPI_ISL_285918	A/Ostrich/South_Africa/S2017/08_0362_P8_34/2017	A / H5N8	Africa / South Africa / Province of the Western Cape	Florette Kathleen Treurnicht (National Institute for Communicable Diseases (NICD) / Center for Respiratory Diseases and Meningitis)
EPI_ISL_287564	A/T_Dk/NL-Werkendam/16014159-001/2016	A / H5N5	Europe / Netherlands / North Brabant / Gemeente Werkendam / Werkendam	Saskia Bergervoet (Wageningen Bioveterinary Research)
EPI_ISL_287565	A/M_Swan/NL-Groningen/16015826-001/2016	A / H5N5	Europe / Netherlands / Provincie Groningen / Gemeente Groningen / Groningen	Saskia Bergervoet (Wageningen Bioveterinary Research)
EPI_ISL_287800	A/spoonbill/Taiwan/DB645/2017	A / H5N6	Asia / Taiwan	Yu-Pin Liu (Animal Health Research Institute)
EPI_ISL_287906	A/Duck/Netherlands/17017236-001-005/2017	A / H5N6	Europe / Netherlands / Provincie Flevoland / Gemeente Dronten / Biddinghuizen	Saskia Bergervoet (Wageningen Bioveterinary Research)
EPI_ISL_287907	A/Duck/Netherlands/17017237-001-005/2017	A / H5N6	Europe / Netherlands / Provincie Flevoland / Gemeente Dronten / Biddinghuizen	Saskia Bergervoet (Wageningen Bioveterinary Research)
EPI_ISL_288362	A/chicken/Greece/39_2017/2017	A / H5N6	Europe / Greece	James Seekings (Animal and Plant Health Agency (APHA) / Virology Department)
EPI_ISL_288363	A/chicken/Greece/39_2017a/2017	A / H5N6	Europe / Greece	James Seekings (Animal and Plant Health Agency (APHA) / Virology Department)
EPI_ISL_288364	A/chicken/Greece/39_2017b/2017	A / H5N6	Europe / Greece	James Seekings (Animal and Plant Health Agency (APHA) / Virology Department)
EPI_ISL_288409	A/Mute Swan/Netherlands/17017367-012/2017	A / H5N6	Europe / Netherlands / Provincie Gelderland / Hulshorst	Saskia Bergervoet (Wageningen Bioveterinary Research)
EPI_ISL_288410	A/Mute Swan/Netherlands/17017377-001/2017	A / H5N6	Europe / Netherlands / Provincie Gelderland / Gemeente Elburg	Saskia Bergervoet (Wageningen Bioveterinary Research)
EPI_ISL_288411	A/Go/NL-Utrecht/17006881-001/2017	A / H5N5	Europe / Netherlands / Provincie Utrecht / Utrecht	Saskia Bergervoet (Wageningen Bioveterinary Research)
EPI_ISL_288412	A/Tufted Duck/Netherlands/17017367-007/2017	A / H5N6	Europe / Netherlands / Provincie Gelderland / Hulshorst	Saskia Bergervoet (Wageningen Bioveterinary Research)
EPI_ISL_288436	A/mallard/Korea/Jeju-H24/2017	A / H5N6	Asia / Korea, Republic of / Jeju-teukbyeoljachido	Eun-Kyoung Lee (Animal and Plant Quarantine Agency (APQA) / Avian Influenza Research and Diagsnotic Division)
EPI_ISL_288437	A/duck/Korea/HD1/2017	A / H5N6	Asia / Korea, Republic of / Jeollabuk-do	Eun-Kyoung Lee (Animal and Plant Quarantine Agency (APQA) / Avian Influenza Research and Diagsnotic Division)
EPI_ISL_288438	A/chicken/Korea/Gunsan/2017	A / H5N8	Asia / Korea, Republic of / Jeollabuk-do	Eun-Kyoung Lee (Animal and Plant Quarantine Agency (APQA) / Avian Influenza Research and Diagsnotic Division)
EPI_ISL_288439	A/chicken/Korea/Gimje2/2017	A / H5N8	Asia / Korea, Republic of / Jeollabuk-do	Eun-Kyoung Lee (Animal and Plant Quarantine Agency (APQA) / Avian Influenza Research and Diagsnotic Division)
EPI_ISL_289173	A/mute swan/Shimane/3211A001/2017	A / H5N6	Asia / Japan / Shimane	
EPI_ISL_289500	A/Great Black-backed Gull/Netherlands/1/2017	A / H5N6	Europe / Netherlands / Provincie Noord-Holland / De Kreupel: 52° 47' 58.3044'' N, 5° 13' 34.550	Maria Johanna Poen (Erasmus Medical Center / Department of Virology)
EPI_ISL_289501	A/Black-headed Gull/Netherlands/29/2017	A / H5N6	Europe / Netherlands / Provincie Noord-Holland / De Kreupel: 52° 47' 58.3044'' N, 5° 13' 34.550	Maria Johanna Poen (Erasmus Medical Center / Department of Virology)
EPI_ISL_290070	A/duck/Liaoning/LN/2011	A / H5N5	Asia / China	
EPI_ISL_290071	A/common porchard/Aichi/2301N025/2017	A / H5N6	Asia / Japan / Aichi	
EPI_ISL_290072	A/eastern buzzard/Oita/4401F005/2017	A / H5N6	Asia / Japan / Oita	
EPI_ISL_290073	A/mandarin duck/Tochigi/0902C033/2017	A / H5N6	Asia / Japan / Tochigi	
EPI_ISL_290156	A/mute swan/Ibaraki/0803_210/2016	A / H5N6	Asia / Japan / Ibaraki	
EPI_ISL_290157	A/northern goshawk/Tochigi/0912A004/2016	A / H5N6	Asia / Japan / Tochigi	

EPI_ISL_290158	A/environment/Gifu/21/2017	A / H5N6	Asia / Japan / Gifu	
EPI_ISL_290250	A/duck/Vietnam/QuangBinh/LBM0818/2016(H5N6)	A / H5N6	Asia / Vietnam	
EPI_ISL_290251	A/duck/Vietnam/QuangBinh/LBM0908/2016(H5N6)	A / H5N6	Asia / Vietnam	
EPI_ISL_290252	A/duck/Vietnam/QuangBinh/LBM0909/2016(H5N6)	A / H5N6	Asia / Vietnam	
EPI_ISL_290253	A/duck/Vietnam/QuangBinh/LBM0911/2016(H5N6)	A / H5N6	Asia / Vietnam	
EPI_ISL_290294	A/Heron/Vietnam/QuangBinh/LBM0910/2016(H5N6)	A / H5N6	Asia / Vietnam	
EPI_ISL_290295	A/mute swan/Hyogo/2801ITM002/2017	A / H5N6	Asia / Japan / Hyogo	
EPI_ISL_290296	A/mute swan/Hyogo/2801ITM003/2017	A / H5N6	Asia / Japan / Hyogo	
EPI_ISL_290297	A/mute swan/Hyogo/2801ITM004/2017	A / H5N6	Asia / Japan / Hyogo	
EPI_ISL_290298	A/mute swan/Hyogo/2801ITM005/2017	A / H5N6	Asia / Japan / Hyogo	
EPI_ISL_290299	A/mute swan/Hyogo/2801ITM006/2017	A / H5N6	Asia / Japan / Hyogo	
EPI_ISL_290300	A/mute swan/Hyogo/2801ITM007/2017	A / H5N6	Asia / Japan / Hyogo	
EPI_ISL_290301	A/mute swan/Hyogo/2801ITM008/2017	A / H5N6	Asia / Japan / Hyogo	
EPI_ISL_290302	A/mute swan/Hyogo/2801ITM009/2017	A / H5N6	Asia / Japan / Hyogo	
EPI_ISL_290305	A/mute swan/Hyogo/2801ITM010/2017	A / H5N6	Asia / Japan / Hyogo	
EPI_ISL_290306	A/mute swan/Hyogo/2801ITM011/2017	A / H5N6	Asia / Japan / Hyogo	
EPI_ISL_290307	A/mute swan/Hyogo/2801ITM012/2017	A / H5N6	Asia / Japan / Hyogo	
EPI_ISL_290308	A/mute swan/Hyogo/2801ITM013/2017	A / H5N6	Asia / Japan / Hyogo	
EPI_ISL_290309	A/mute swan/Hyogo/2801ITM014/2017	A / H5N6	Asia / Japan / Hyogo	
EPI_ISL_290310	A/peregrine falcon/Niigata/15/2016	A / H5N6	Asia / Japan / Niigata	
EPI_ISL_290311	A/whooper swan/Niigata/5112008/2016	A / H5N6	Asia / Japan / Niigata	
EPI_ISL_290312	A/greater scaup/Aichi/2301H050/2017	A / H5N6	Asia / Japan / Aichi	
EPI_ISL_290313	A/peregrine falcon/Gifu/2102A008/2017	A / H5N6	Asia / Japan / Gifu	
EPI_ISL_291109	A/common_pochard/Germany-BY/AR09-18-L02421/2017	A / H5N6	Europe / Germany / Bavaria	Anne Pohlmann (Friedrich-Loeffler-Institut)
EPI_ISL_291952	A/Bar-headed Goose/Qinghai/B44/2016	A / H5N8	Asia / China / Qinghai Province	Jianjun Chen (Wuhan Institute of Virology / Chinese Academy of Sciences)
EPI_ISL_292170	A/Bar-headed Goose/Qinghai/A22/2016	A / H5N8	Asia / China / Qinghai Province	Jianjun Chen (Wuhan Institute of Virology / Chinese Academy of Sciences)
EPI_ISL_292172	A/Bar-headed Goose/Qinghai/B7/2016	A / H5N8	Asia / China / Qinghai Province	Jianjun Chen (Wuhan Institute of Virology / Chinese Academy of Sciences)
EPI_ISL_292173	A/Bar-headed Goose/Qinghai/B54/2016	A / H5N8	Asia / China / Qinghai Province	Jianjun Chen (Wuhan Institute of Virology / Chinese Academy of Sciences)
EPI_ISL_292174	A/Bar-headed Goose/Qinghai/XX431/2016	A / H5N8	Asia / China / Qinghai Province	Jianjun Chen (Wuhan Institute of Virology / Chinese Academy of Sciences)
EPI_ISL_292175	A/Bar-headed Goose/Qinghai/XX446/2016	A / H5N8	Asia / China / Qinghai Province	Jianjun Chen (Wuhan Institute of Virology / Chinese Academy of Sciences)
EPI_ISL_292176	A/Bar-headed Goose/Qinghai/A17/2016	A / H5N8	Asia / China / Qinghai Province	Jianjun Chen (Wuhan Institute of Virology / Chinese Academy of Sciences)
EPI_ISL_292177	A/Bar-headed Goose/Qinghai/A20/2016	A / H5N8	Asia / China / Qinghai Province	Jianjun Chen (Wuhan Institute of Virology / Chinese Academy of Sciences)
EPI_ISL_292178	A/Bar-headed Goose/Qinghai/A12/2016	A / H5N8	Asia / China / Qinghai Province	Jianjun Chen (Wuhan Institute of Virology / Chinese Academy of Sciences)
EPI_ISL_292179	A/Bar-headed Goose/Qinghai/XX13/2016	A / H5N8	Asia / China / Qinghai Province	Jianjun Chen (Wuhan Institute of Virology / Chinese Academy of Sciences)
EPI_ISL_292180	A/Bar-headed Goose/Qinghai/XX22/2016	A / H5N8	Asia / China / Qinghai Province	Jianjun Chen (Wuhan Institute of Virology / Chinese Academy of Sciences)
EPI_ISL_292181	A/Bar-headed Goose/Qinghai/A19/2016	A / H5N8	Asia / China / Qinghai Province	Jianjun Chen (Wuhan Institute of Virology / Chinese Academy of Sciences)
EPI_ISL_292182	A/Bar-headed Goose/Qinghai/A16/2016	A / H5N8	Asia / China / Qinghai Province	Jianjun Chen (Wuhan Institute of Virology / Chinese Academy of Sciences)
EPI_ISL_292183	A/Bar-headed Goose/Qinghai/A13/2016	A / H5N8	Asia / China / Qinghai Province	Jianjun Chen (Wuhan Institute of Virology / Chinese Academy of Sciences)
EPI_ISL_292184	A/Bar-headed Goose/Qinghai/B51/2016	A / H5N8	Asia / China / Qinghai Province	Jianjun Chen (Wuhan Institute of Virology / Chinese Academy of Sciences)
EPI_ISL_292185	A/Great Cormorant/Qinghai/Y01/2016	A / H5N8	Asia / China / Qinghai Province	Jianjun Chen (Wuhan Institute of Virology / Chinese Academy of Sciences)
EPI_ISL_292186	A/Bar-headed Goose/Qinghai/B34/2016	A / H5N8	Asia / China / Qinghai Province	Jianjun Chen (Wuhan Institute of Virology / Chinese Academy of Sciences)
EPI_ISL_292187	A/Bar-headed Goose/Qinghai/XX782/2016	A / H5N8	Asia / China / Qinghai Province	Jianjun Chen (Wuhan Institute of Virology / Chinese Academy of Sciences)
EPI_ISL_292188	A/Great Cormorant/Qinghai/B82/2016	A / H5N8	Asia / China / Qinghai Province	Jianjun Chen (Wuhan Institute of Virology / Chinese Academy of Sciences)
EPI_ISL_292189	A/Bar-headed Goose/Qinghai/A21/2016	A / H5N8	Asia / China / Qinghai Province	Jianjun Chen (Wuhan Institute of Virology / Chinese Academy of Sciences)
EPI_ISL_292190	A/Bar-headed Goose/Qinghai/XX111/2016	A / H5N8	Asia / China / Qinghai Province	Jianjun Chen (Wuhan Institute of Virology / Chinese Academy of Sciences)
EPI_ISL_292191	A/Bar-headed Goose/Qinghai/B12/2016	A / H5N8	Asia / China / Qinghai Province	Jianjun Chen (Wuhan Institute of Virology / Chinese Academy of Sciences)
EPI_ISL_292192	A/Bar-headed Goose/Qinghai/A23/2016	A / H5N8	Asia / China / Qinghai Province	Jianjun Chen (Wuhan Institute of Virology / Chinese Academy of Sciences)
EPI_ISL_292193	A/Bar-headed Goose/Qinghai/XX76/2016	A / H5N8	Asia / China / Qinghai Province	Jianjun Chen (Wuhan Institute of Virology / Chinese Academy of Sciences)
EPI_ISL_292194	A/Bar-headed Goose/Qinghai/a88/2016	A / H5N8	Asia / China / Qinghai Province	Jianjun Chen (Wuhan Institute of Virology / Chinese Academy of Sciences)
EPI_ISL_292196	A/Bar-headed Goose/Qinghai/a32/2016	A / H5N8	Asia / China / Qinghai Province	Jianjun Chen (Wuhan Institute of Virology / Chinese Academy of Sciences)
EPI_ISL_292198	A/Bar-headed Goose/Qinghai/a115/2016	A / H5N8	Asia / China / Qinghai Province	Jianjun Chen (Wuhan Institute of Virology / Chinese Academy of Sciences)
EPI_ISL_292223	A/mute_swan/England/AVP_18_001986/2017	A / H5N6	Europe / United Kingdom / Dorset / Abbotsbury	James Seekings (Animal and Plant Health Agency (APHA) / Virology Department)
EPI_ISL_292224	A/pochard_duck/England/AVP_18_003254/2018	A / H5N6	Europe / United Kingdom / Dorset / Abbotsbury	James Seekings (Animal and Plant Health Agency (APHA) / Virology Department)
EPI_ISL_292225	A/canada_goose/England/AV58_18OPpoolEP1/2018	A / H5N6	Europe / United Kingdom / Dorset / Abbotsbury	James Seekings (Animal and Plant Health Agency (APHA) / Virology Department)
EPI_ISL_292229	A/Bar-headed Goose/Qinghai/a113/2016	A / H5N8	Asia / China / Qinghai Province	Jianjun Chen (Wuhan Institute of Virology / Chinese Academy of Sciences)
EPI_ISL_292230	A/Bar-headed Goose/Qinghai/a93/2016	A / H5N8	Asia / China / Qinghai Province	Jianjun Chen (Wuhan Institute of Virology / Chinese Academy of Sciences)
EPI_ISL_292231	A/Bar-headed Goose/Qinghai/a61/2016	A / H5N8	Asia / China / Qinghai Province	Jianjun Chen (Wuhan Institute of Virology / Chinese Academy of Sciences)
EPI_ISL_292232	A/Bar-headed Goose/Qinghai/HDT001/2016	A / H5N8	Asia / China / Qinghai Province	Jianjun Chen (Wuhan Institute of Virology / Chinese Academy of Sciences)
EPI_ISL_292233	A/Bar-headed Goose/Qinghai/a91/2016	A / H5N8	Asia / China / Qinghai Province	Jianjun Chen (Wuhan Institute of Virology / Chinese Academy of Sciences)
EPI_ISL_292234	A/Bar-headed Goose/Qinghai/a92/2016	A / H5N8	Asia / China / Qinghai Province	Jianjun Chen (Wuhan Institute of Virology / Chinese Academy of Sciences)
EPI_ISL_292235	A/Bar-headed Goose/Qinghai/a43/2016	A / H5N8	Asia / China / Qinghai Province	Jianjun Chen (Wuhan Institute of Virology / Chinese Academy of Sciences)
EPI_ISL_292236	A/Bar-headed Goose/Qinghai/a24/2016	A / H5N8	Asia / China / Qinghai Province	Jianjun Chen (Wuhan Institute of Virology / Chinese Academy of Sciences)
EPI_ISL_292237	A/Bar-headed Goose/Qinghai/a15/2016	A / H5N8	Asia / China / Qinghai Province	Jianjun Chen (Wuhan Institute of Virology / Chinese Academy of Sciences)
EPI_ISL_292238	A/Bar-headed Goose/Qinghai/p18/2016	A / H5N8	Asia / China / Qinghai Province	Jianjun Chen (Wuhan Institute of Virology / Chinese Academy of Sciences)
EPI_ISL_292239	A/Bar-headed Goose/Qinghai/p9/2016	A / H5N8	Asia / China / Qinghai Province	Jianjun Chen (Wuhan Institute of Virology / Chinese Academy of Sciences)
EPI_ISL_292240	A/Bar-headed Goose/Qinghai/p23/2016	A / H5N8	Asia / China / Qinghai Province	Jianjun Chen (Wuhan Institute of Virology / Chinese Academy of Sciences)
EPI_ISL_292326	A/Great Cormorant/Qinghai/a51/2016	A / H5N8	Asia / China / Qinghai Province	Jianjun Chen (Wuhan Institute of Virology / Chinese Academy of Sciences)
EPI_ISL_292327	A/Bar-headed Goose/Qinghai/p2/2016	A / H5N8	Asia / China / Qinghai Province	Jianjun Chen (Wuhan Institute of Virology / Chinese Academy of Sciences)
EPI_ISL_292328	A/Bar-headed Goose/Qinghai/a45/2016	A / H5N8	Asia / China / Qinghai Province	Jianjun Chen (Wuhan Institute of Virology / Chinese Academy of Sciences)
EPI_ISL_292329	A/Bar-headed Goose/Qinghai/a27/2016	A / H5N8	Asia / China / Qinghai Province	Jianjun Chen (Wuhan Institute of Virology / Chinese Academy of Sciences)
EPI_ISL_292330	A/Bar-headed Goose/Qinghai/a26/2016	A / H5N8	Asia / China / Qinghai Province	Jianjun Chen (Wuhan Institute of Virology / Chinese Academy of Sciences)
EPI_ISL_292331	A/Bar-headed Goose/Qinghai/a114/2016	A / H5N8	Asia / China / Qinghai Province	Jianjun Chen (Wuhan Institute of Virology / Chinese Academy of Sciences)

EPI_ISL_292332	A/water/Qinghai/i34-39/2016	A / H5N8	Asia / China / Qinghai Province	Jianjun Chen (Wuhan Institute of Virology / Chinese Academy of Sciences)
EPI_ISL_292333	A/Water/Qinghai/i40-44/2016	A / H5N8	Asia / China / Qinghai Province	Jianjun Chen (Wuhan Institute of Virology / Chinese Academy of Sciences)
EPI_ISL_292334	A/Bar-headed Goose/Qinghai/B655/2017	A / H5N8	Asia / China / Qinghai Province	Jianjun Chen (Wuhan Institute of Virology / Chinese Academy of Sciences)
EPI_ISL_292335	A/water/Qinghai/XXII2871/2017	A / H5N8	Asia / China / Qinghai Province	Jianjun Chen (Wuhan Institute of Virology / Chinese Academy of Sciences)
EPI_ISL_292340	A/Bar-headed Goose/Qinghai/a230/2017	A / H5N8	Asia / China / Qinghai Province	Jianjun Chen (Wuhan Institute of Virology / Chinese Academy of Sciences)
EPI_ISL_292341	A/Bar-headed Goose/Qinghai/a218/2017	A / H5N8	Asia / China / Qinghai Province	Jianjun Chen (Wuhan Institute of Virology / Chinese Academy of Sciences)
EPI_ISL_292345	A/duck/Korea/H35/2017	A / H5N6	Asia / Korea, Republic of / Jeollanam-do	Yu-Na Lee (Animal and Plant Quarantine Agency (S-2158) / Avian Influenza Research & Diagnostic Division)
EPI_ISL_292349	A/duck/Korea/H56/2017	A / H5N6	Asia / Korea, Republic of	Yu-Na Lee (Animal and Plant Quarantine Agency (S-2158) / Avian Influenza Research & Diagnostic Division)
EPI_ISL_292413	A/duck/Korea/H70/2017	A / H5N6	Asia / Korea, Republic of	Yu-Na Lee (Animal and Plant Quarantine Agency (S-2158) / Avian Influenza Research & Diagnostic Division)
EPI_ISL_292414	A/duck/Korea/H80/2017	A / H5N6	Asia / Korea, Republic of	Yu-Na Lee (Animal and Plant Quarantine Agency (S-2158) / Avian Influenza Research & Diagnostic Division)
EPI_ISL_292415	A/duck/Korea/H81/2017	A / H5N6	Asia / Korea, Republic of	Yu-Na Lee (Animal and Plant Quarantine Agency (S-2158) / Avian Influenza Research & Diagnostic Division)
EPI_ISL_292416	A/duck/Korea/H103/2017	A / H5N6	Asia / Korea, Republic of	Yu-Na Lee (Animal and Plant Quarantine Agency (S-2158) / Avian Influenza Research & Diagnostic Division)
EPI_ISL_292417	A/duck/Korea/H107/2017	A / H5N6	Asia / Korea, Republic of	Yu-Na Lee (Animal and Plant Quarantine Agency (S-2158) / Avian Influenza Research & Diagnostic Division)
EPI_ISL_292418	A/mandarin duck/Korea/H69/2017	A / H5N6	Asia / Korea, Republic of	Yu-Na Lee (Animal and Plant Quarantine Agency (S-2158) / Avian Influenza Research & Diagnostic Division)
EPI_ISL_292419	A/mandarin duck/Korea/H71/2017	A / H5N6	Asia / Korea, Republic of	Yu-Na Lee (Animal and Plant Quarantine Agency (S-2158) / Avian Influenza Research & Diagnostic Division)
EPI_ISL_292420	A/mandarin duck/Korea/H119-3/2017	A / H5N6	Asia / Korea, Republic of	Yu-Na Lee (Animal and Plant Quarantine Agency (S-2158) / Avian Influenza Research & Diagnostic Division)
EPI_ISL_292476	A/Bar-headed Goose/Qinghai/a237/2017	A / H5N8	Asia / China / Qinghai Province	Jianjun Chen (Wuhan Institute of Virology / Chinese Academy of Sciences)
EPI_ISL_292477	A/Great Crested Grebe/Qinghai/a737/2017	A / H5N8	Asia / China / Qinghai Province	Jianjun Chen (Wuhan Institute of Virology / Chinese Academy of Sciences)
EPI_ISL_292478	A/Bar-headed Goose/Qinghai/a765/2017	A / H5N8	Asia / China / Qinghai Province	Jianjun Chen (Wuhan Institute of Virology / Chinese Academy of Sciences)
EPI_ISL_292479	A/Bar-headed Goose/Qinghai/a893/2017	A / H5N8	Asia / China / Qinghai Province	Jianjun Chen (Wuhan Institute of Virology / Chinese Academy of Sciences)
EPI_ISL_292495	A/Bar-headed Goose/Qinghai/B11/2016	A / H5N8	Asia / China / Qinghai Province	Jianjun Chen (Wuhan Institute of Virology / Chinese Academy of Sciences)
EPI_ISL_292496	A/Bar-headed Goose/Qinghai/A11/2016	A / H5N8	Asia / China / Qinghai Province	Jianjun Chen (Wuhan Institute of Virology / Chinese Academy of Sciences)
EPI_ISL_292497	A/Bar-headed Goose/Qinghai/XXI122/2016	A / H5N8	Asia / China / Qinghai Province	Jianjun Chen (Wuhan Institute of Virology / Chinese Academy of Sciences)
EPI_ISL_292498	A/Bar-headed Goose/Qinghai/a210/2017	A / H5N8	Asia / China / Qinghai Province	Jianjun Chen (Wuhan Institute of Virology / Chinese Academy of Sciences)
EPI_ISL_2932600	A/goose/Kazakhstan/4-190-20-B-H5N8-1/2020	A / H5N8	Asia / Kazakhstan / Almaty Province / Karasai district, Umtyl, Merei village	Alexandr Shevtsov (National Center of Biotechnology Republic of Kazakhstan)
EPI_ISL_2932608	A/chicken/Kazakhstan/220-B-2-H5N8-4/2020	A / H5N8	Asia / Kazakhstan / Almaty Province / Karasai district, Umtyl, Merei village	Alexandr Shevtsov (National Center of Biotechnology Republic of Kazakhstan)
EPI_ISL_2932609	A/duck/Kazakhstan/12-20-B-Talg-11/2020	A / H5N8	Asia / Kazakhstan / North Kazakhstan / Timiryazevsky district, Timiryazevo village	Alexandr Shevtsov (National Center of Biotechnology Republic of Kazakhstan)
EPI_ISL_2932612	A/goose/Kazakhstan/7-20-B-Talg-12/2020	A / H5N8	Asia / Kazakhstan / North Kazakhstan / Ayyrtausky district, Arykbalyk, Agymtai batyr village	Alexandr Shevtsov (National Center of Biotechnology Republic of Kazakhstan)
EPI_ISL_2932614	A/swan/Kazakhstan/9-20-B-Talg-39/2020	A / H5N8	Asia / Kazakhstan / North Kazakhstan / Mamlyutsky district, Mamlyutsky, Mamlyutka village	Alexandr Shevtsov (National Center of Biotechnology Republic of Kazakhstan)
EPI_ISL_2932616	A/chicken/Kazakhstan/12-20-B-Talg-45/2020	A / H5N8	Asia / Kazakhstan / North Kazakhstan / Akzhar district, Talshyk, Kazanskoe village	Alexandr Shevtsov (National Center of Biotechnology Republic of Kazakhstan)
EPI_ISL_2932617	A/crow/Kazakhstan/15-20-B-Talg-4/2020	A / H5N8	Asia / Kazakhstan / North Kazakhstan / Petropavlovsk city	Alexandr Shevtsov (National Center of Biotechnology Republic of Kazakhstan)
EPI_ISL_2932619	A/swan/Kazakhstan/1-267-20-B-Talg-52/2020	A / H5N8	Asia / Kazakhstan / Akmola / Birzhan sal district, Baymyrza, Shoshkaly lake	Alexandr Shevtsov (National Center of Biotechnology Republic of Kazakhstan)
EPI_ISL_2932682	A/pigeon/Kazakhstan/15-20-B-Talg-5/2020	A / H5N8	Asia / Kazakhstan / North Kazakhstan / Petropavlovsk city	Alexandr Shevtsov (National Center of Biotechnology Republic of Kazakhstan)
EPI_ISL_2932683	A/chicken/Kazakhstan/1-20-B-Talg-67/2020	A / H5N8	Asia / Kazakhstan / North Kazakhstan / Tayinshinsky district, Roshchinsky, Dimitrovka village	Alexandr Shevtsov (National Center of Biotechnology Republic of Kazakhstan)
EPI_ISL_293482	A/chicken/Miyazaki/21/2016	A / H5N6	Asia / Japan / Miyazaki	
EPI_ISL_293483	A/chicken/Kumamoto/45/2016	A / H5N6	Asia / Japan / Kumamoto	
EPI_ISL_293484	A/environment/Aomori/4/2016	A / H5N6	Asia / Japan / Aomori	
EPI_ISL_293485	A/environment/Aomori/R1/2016	A / H5N6	Asia / Japan / Aomori	
EPI_ISL_293486	A/environment/Niigata/5/2016	A / H5N6	Asia / Japan / Niigata	
EPI_ISL_293487	A/environment/Aomori/2-1/2016	A / H5N6	Asia / Japan / Aomori	
EPI_ISL_293488	A/environment/Hokkaido/1-8/2016	A / H5N6	Asia / Japan / Hokkaido	
EPI_ISL_293489	A/environment/Saga/4/2017	A / H5N6	Asia / Japan / Saga	
EPI_ISL_293498	A/northern goshawk/Mie/2412C007/2016	A / H5N6	Asia / Japan / Mie	
EPI_ISL_293499	A/peregrine falcon/Nagasaki/4212A005/2016	A / H5N6	Asia / Japan / Nagasaki	
EPI_ISL_293500	A/tufted duck/Hyogo/2812A009/2016	A / H5N6	Asia / Japan / Hyogo	
EPI_ISL_293501	A/wigeon/Aichi/2301H025/2017	A / H5N6	Asia / Japan / Aichi	
EPI_ISL_293502	A/common porchard/Aichi/2301N017/2017	A / H5N6	Asia / Japan / Aichi	
EPI_ISL_293503	A/common porchard/Yamaguchi/3501B002/2017	A / H5N6	Asia / Japan / Yamaguchi	
EPI_ISL_293504	A/mute swan/Hyogo/2801ITM001/2017	A / H5N6	Asia / Japan / Hyogo	
EPI_ISL_293505	A/coot/Shiga/2501T010/2017	A / H5N6	Asia / Japan / Shiga	
EPI_ISL_293989	A/chicken/Vietnam/HU4-42/2015	A / H5N6	Asia / Vietnam	
EPI_ISL_294412	A/black swan/Aichi/2312T001/2016	A / H5N6	Asia / Japan / Aichi	
EPI_ISL_294413	A/black swan/Aichi/2312T002/2016	A / H5N6	Asia / Japan / Aichi	
EPI_ISL_294414	A/black swan/Aichi/2312T003/2016	A / H5N6	Asia / Japan / Aichi	
EPI_ISL_294600	A/Von Schrenck's bittern/Jiangxi/Y9/2014	A / H5N8	Asia / China	
EPI_ISL_294610	A/turkey/Minnesota/E3/2015	A / H5N2	North America / United States	
EPI_ISL_294611	A/turkey/Minnesota/E4/2015	A / H5N2	North America / United States	
EPI_ISL_294612	A/turkey/Minnesota/E6/2015	A / H5N2	North America / United States	
EPI_ISL_294613	A/environment/Minnesota/E1/2015	A / H5N2	North America / United States	
EPI_ISL_294614	A/environment/Minnesota/E2/2015	A / H5N2	North America / United States	
EPI_ISL_294615	A/environment/Minnesota/E5/2015	A / H5N2	North America / United States	
EPI_ISL_294616	A/environment/Minnesota/E7/2015	A / H5N2	North America / United States	
EPI_ISL_294617	A/environment/Minnesota/E8/2015	A / H5N2	North America / United States	
EPI_ISL_294618	A/environment/Minnesota/E9/2015	A / H5N2	North America / United States	
EPI_ISL_294619	A/environment/Minnesota/E10/2015	A / H5N2	North America / United States	
EPI_ISL_294628	A/turkey/Minnesota/E11/2015	A / H5N2	North America / United States	
EPI_ISL_294630	A/turkey/Minnesota/E12/2015	A / H5N2	North America / United States	
EPI_ISL_294631	A/turkey/Minnesota/E13/2015	A / H5N2	North America / United States	
EPI_ISL_294632	A/turkey/Minnesota/E14/2015	A / H5N2	North America / United States	
EPI_ISL_294635	A/turkey/Minnesota/E17/2015	A / H5N2	North America / United States	
EPI_ISL_294636	A/turkey/Minnesota/E18/2015	A / H5N2	North America / United States	
EPI_ISL_294683	A/environment/Minnesota/E15/2015	A / H5N2	North America / United States	

EPI_ISL_294685	A/environment/Minnesota/E16/2015	A / H5N2	North America / United States	
EPI_ISL_294742	A/environment/Minnesota/M1/2015	A / H5N2	North America / United States	
EPI_ISL_294747	A/environment/Minnesota/M2/2015	A / H5N2	North America / United States	
EPI_ISL_294748	A/environment/Minnesota/M3/2015	A / H5N2	North America / United States	
EPI_ISL_294749	A/environment/Minnesota/M4/2015	A / H5N2	North America / United States	
EPI_ISL_294750	A/environment/Minnesota/M5/2015	A / H5N2	North America / United States	
EPI_ISL_294751	A/environment/Minnesota/M6/2015	A / H5N2	North America / United States	
EPI_ISL_294752	A/environment/Minnesota/M7/2015	A / H5N2	North America / United States	
EPI_ISL_294753	A/environment/Minnesota/M8/2015	A / H5N2	North America / United States	
EPI_ISL_294754	A/turkey/Minnesota/M14/2015	A / H5N2	North America / United States	
EPI_ISL_294755	A/turkey/Minnesota/M15/2015	A / H5N2	North America / United States	
EPI_ISL_294756	A/turkey/Minnesota/M16/2015	A / H5N2	North America / United States	
EPI_ISL_294757	A/turkey/Minnesota/M17/2015	A / H5N2	North America / United States	
EPI_ISL_294759	A/environment/Minnesota/M9/2015	A / H5N2	North America / United States	
EPI_ISL_294760	A/environment/Minnesota/M10/2015	A / H5N2	North America / United States	
EPI_ISL_294762	A/environment/Minnesota/M11/2015	A / H5N2	North America / United States	
EPI_ISL_294763	A/environment/Minnesota/M12/2015	A / H5N2	North America / United States	
EPI_ISL_294764	A/environment/Minnesota/M13/2015	A / H5N2	North America / United States	
EPI_ISL_294767	A/environment/Nebraska/L3/2015	A / H5N2	North America / United States	
EPI_ISL_294768	A/environment/Nebraska/L4/2015	A / H5N2	North America / United States	
EPI_ISL_294769	A/environment/Nebraska/L5/2015	A / H5N2	North America / United States	
EPI_ISL_294771	A/environment/Nebraska/L6/2015	A / H5N2	North America / United States	
EPI_ISL_294773	A/environment/Nebraska/L8/2015	A / H5N2	North America / United States	
EPI_ISL_294774	A/environment/Nebraska/L9/2015	A / H5N2	North America / United States	
EPI_ISL_294777	A/environment/Iowa/L2/2015	A / H5N2	North America / United States	
EPI_ISL_295027	A/chicken/Kostroma/1718/2017	A / H5N2	Europe / Russian Federation / Kostroma Oblast	Ivan Susloparov (State Research Center of Virology and Biotechnology (VECTOR) / Emerging Zoonotic Diseases and Influenza)
EPI_ISL_295143	A/goose/Guangdong/YJD/2014	A / H5N6	Asia / China	
EPI_ISL_295144	A/chicken/Yangzhou/YD1/2014	A / H5N6	Asia / China	
EPI_ISL_295147	A/chicken/Anhui/QD1/2014	A / H5N1	Asia / China	
EPI_ISL_295492	A/Anser cygnoides/Hubei/FW44/2016	A / H5N8	Asia / China	
EPI_ISL_295493	A/Cygnus atratus/Hubei/2Z2-O/2016	A / H5N8	Asia / China	
EPI_ISL_295494	A/Cygnus atratus/Hubei/HF-1/2016	A / H5N8	Asia / China	
EPI_ISL_295496	A/Commom Teal/Ningxia/474-9/2015	A / H5N6	Asia / China	
EPI_ISL_295497	A/Shoveller/Ningxia/475-11/2015	A / H5N6	Asia / China	
EPI_ISL_295498	A/Wigeon/Ningxia/476-12/2015	A / H5N6	Asia / China	
EPI_ISL_295499	A/Commom Teal/Ningxia/478-15/2015	A / H5N6	Asia / China	
EPI_ISL_295500	A/Shoveller/Ningxia/481-21/2015	A / H5N6	Asia / China	
EPI_ISL_295501	A/Great Crested Grebe/Ningxia/482-26/2015	A / H5N6	Asia / China	
EPI_ISL_295502	A/Shoveller/Ningxia/483-28/2015	A / H5N6	Asia / China	
EPI_ISL_295503	A/Gadwall/Ningxia/472-7/2015	A / H5N6	Asia / China	
EPI_ISL_295504	A/Falcated Duck/Ningxia/484-29/2015	A / H5N6	Asia / China	
EPI_ISL_295505	A/Gadwall/Ningxia/485-31/2015	A / H5N6	Asia / China	
EPI_ISL_295519	A/Ruddy Shelduck/Ningxia/486-33/2015	A / H5N6	Asia / China	
EPI_ISL_295520	A/Gadwall/Ningxia/487-38/2015	A / H5N6	Asia / China	
EPI_ISL_295521	A/Shoveller/Ningxia/488-53/2015	A / H5N6	Asia / China	
EPI_ISL_295701	A/whooper swan/Ibaraki/28-309/2016	A / H5N6	Asia / Japan / Ibaraki	
EPI_ISL_295702	A/black-headed gull/Ibaraki/0803-194/2016	A / H5N6	Asia / Japan / Ibaraki	
EPI_ISL_295703	A/mute swan/Ibaraki/0803-201/2016	A / H5N6	Asia / Japan / Ibaraki	
EPI_ISL_295704	A/mute swan/Ibaraki/0803-208/2016	A / H5N6	Asia / Japan / Ibaraki	
EPI_ISL_295705	A/mute swan/Ibaraki/0803-214/2016	A / H5N6	Asia / Japan / Ibaraki	
EPI_ISL_295751	A/greater scaup/Aichi/2301N021/2017	A / H5N6	Asia / Japan / Aichi	
EPI_ISL_295752	A/black-headed gull/Hyogo/2801E009/2017	A / H5N6	Asia / Japan / Hyogo	
EPI_ISL_295754	A/cackling goose/Aichi/2312T020/2016	A / H5N6	Asia / Japan / Aichi	
EPI_ISL_295796	A/muscovy duck/Vietnam/HU2-26/2014	A / H5N6	Asia / Vietnam	
EPI_ISL_295818	A/muscovy duck/Vietnam/HU7-17/2017	A / H5N6	Asia / Vietnam	
EPI_ISL_295819	A/muscovy duck/Vietnam/HU7-20/2017	A / H5N6	Asia / Vietnam	
EPI_ISL_295820	A/muscovy duck/Vietnam/HU7-23/2017	A / H5N6	Asia / Vietnam	
EPI_ISL_297234	A/chicken/Rostov-on-Don/1321/2017	A / H5N8	Europe / Russian Federation / Rostov Oblast	Ivan Susloparov (State Research Center of Virology and Biotechnology (VECTOR) / Emerging Zoonotic Diseases and Influenza)
EPI_ISL_297235	A/chicken/Rostov-on-Don/1598/2017	A / H5N8	Europe / Russian Federation / Rostov Oblast	Ivan Susloparov (State Research Center of Virology and Biotechnology (VECTOR) / Emerging Zoonotic Diseases and Influenza)
EPI_ISL_297324	A/White-eyed Pochard/Ningxia/473-8/2015	A / H5N6	Asia / China	
EPI_ISL_297325	A/White-eyed Pochard/Ningxia/477-14/2015	A / H5N6	Asia / China	
EPI_ISL_297326	A/White-eyed Pochard/Ningxia/479-16/2015	A / H5N6	Asia / China	
EPI_ISL_297327	A/White-eyed Pochard/Ningxia/480-17/2015	A / H5N6	Asia / China	
EPI_ISL_297388	A/chicken/Republic of Macedonia/466/2017	A / H5N8	Europe / Macedonia, the former Yogoslav Republic of / Opstina Struga / N 41012’10, E20042’01	Aleksandar Dodovski (Faculty of Veterinary Medicine Skopje / Department for Avian Diseases)
EPI_ISL_297463	A/chicken/Kostroma/1717/2017	A / H5N2	Europe / Russian Federation / Kostroma Oblast	Ivan Susloparov (State Research Center of Virology and Biotechnology (VECTOR) / Emerging Zoonotic Diseases and Influenza)
EPI_ISL_297464	A/chicken/Kostroma/1719/2017	A / H5N2	Europe / Russian Federation / Kostroma Oblast	Ivan Susloparov (State Research Center of Virology and Biotechnology (VECTOR) / Emerging Zoonotic Diseases and Influenza)
EPI_ISL_297465	A/chicken/Kostroma/1720/2017	A / H5N2	Europe / Russian Federation / Kostroma Oblast	Ivan Susloparov (State Research Center of Virology and Biotechnology (VECTOR) / Emerging Zoonotic Diseases and Influenza)
EPI_ISL_297466	A/chicken/Kostroma/1721/2017	A / H5N2	Europe / Russian Federation / Kostroma Oblast	Ivan Susloparov (State Research Center of Virology and Biotechnology (VECTOR) / Emerging Zoonotic Diseases and Influenza)
EPI_ISL_297930	A/chicken/Anhui/MZ33/2016	A / H5N6	Asia / China	
EPI_ISL_297931	A/chicken/Anhui/MZ34/2016	A / H5N6	Asia / China	
EPI_ISL_297932	A/chicken/Henan/YB0597/2016	A / H5N6	Asia / China	
EPI_ISL_298640	A/turkey/Israel/1045/2016	A / H5N8	Asia / Israel	

EPI_ISL_298641	A/chicken/Israel/881/2016	A / H5N8	Asia / Israel	
EPI_ISL_298642	A/chicken/Israel/1048/2016	A / H5N8	Asia / Israel	
EPI_ISL_298647	A/cormorant/Israel/1035/2016	A / H5N8	Asia / Israel	
EPI_ISL_298648	A/great egret/Israel/1088/2016	A / H5N8	Asia / Israel	
EPI_ISL_298649	A/turkey/Israel/184/2017	A / H5N8	Asia / Israel	
EPI_ISL_298650	A/turkey/Israel/1076/2016	A / H5N8	Asia / Israel	
EPI_ISL_298651	A/great egret/Israel/1084/2016	A / H5N8	Asia / Israel	
EPI_ISL_298652	A/grey goose/Israel/986/2016	A / H5N8	Asia / Israel	
EPI_ISL_298653	A/peregrine falcon/Israel/1086/2016	A / H5N8	Asia / Israel	
EPI_ISL_299583	A/heron/Guangdong/C1/2013	A / H5N6	Asia / China	
EPI_ISL_3001273	A/common buzzard/Sweden/SVA210510SZ0206/FB001829/C-:	A / H5N8	Europe / Sweden / Uppsala Lan / Uppsala Kommun	Siamak Zohari (National Veterinary Institute)
EPI_ISL_300547	A/White-fronted Goose/AN/1-15-12/2016	A / H5N8	Europe / Ukraine	Susanne Koethe (Friedrich-Loeffler-Institut)
EPI_ISL_300548	A/Ruddy Shelduck/AN/2-14-12/2016	A / H5N8	Europe / Ukraine / Kherson Oblast	Susanne Koethe (Friedrich-Loeffler-Institut)
EPI_ISL_300562	A/Environmental (EM) /AN/2/17	A / H5N8	Europe / Ukraine / Kherson Oblast	Susanne Koethe (Friedrich-Loeffler-Institut)
EPI_ISL_3005950	A/barnacle goose/Sweden/SVA210511SZ0567/FB001840/M-2(	A / H5N1	Europe / Sweden / Skane Lan / Malmo Kommun	Siamak Zohari (National Veterinary Institute)
EPI_ISL_300642	A/turkey/South Dakota/15-011769-1/2015	A / H5N2	North America / United States	
EPI_ISL_300643	A/turkey/South Dakota/15-015102-5/2015	A / H5N2	North America / United States	
EPI_ISL_300644	A/turkey/South Dakota/15-015103-1/2015	A / H5N2	North America / United States	
EPI_ISL_300645	A/turkey/South Dakota/15-017406-1/2015	A / H5N2	North America / United States	
EPI_ISL_300646	A/turkey/Wisconsin/15-012886-1/2015	A / H5N2	North America / United States	
EPI_ISL_300647	A/turkey/Wisconsin/15-013180-1/2015	A / H5N2	North America / United States	
EPI_ISL_300648	A/turkey/Wisconsin/15-013771-1/2015	A / H5N2	North America / United States	
EPI_ISL_300649	A/turkey/Wisconsin/15-014296-1/2015	A / H5N2	North America / United States	
EPI_ISL_300650	A/turkey/Wisconsin/15-014298-1/2015	A / H5N2	North America / United States	
EPI_ISL_300651	A/turkey/Minnesota/15-017647-1/2015	A / H5N2	North America / United States	
EPI_ISL_300652	A/turkey/Minnesota/15-017649-1/2015	A / H5N2	North America / United States	
EPI_ISL_300653	A/turkey/Minnesota/15-017814-1/2015	A / H5N2	North America / United States	
EPI_ISL_300654	A/turkey/Minnesota/15-017989-1/2015	A / H5N2	North America / United States	
EPI_ISL_300655	A/turkey/Minnesota/15-018201-1/2015	A / H5N2	North America / United States	
EPI_ISL_300656	A/turkey/Missouri/15-007513-1/2015	A / H5N2	North America / United States	
EPI_ISL_300657	A/turkey/South Dakota/15-011089-3/2015	A / H5N2	North America / United States	
EPI_ISL_300658	A/turkey/South Dakota/15-011415-1/2015	A / H5N2	North America / United States	
EPI_ISL_300659	A/turkey/South Dakota/15-011417-1/2015	A / H5N2	North America / United States	
EPI_ISL_300660	A/tundra swan/Niigata/1511C003/2016	A / H5N6	Asia / Japan / Niigata	
EPI_ISL_300661	A/tundra swan/Niigata/5112004/2016	A / H5N6	Asia / Japan / Niigata	
EPI_ISL_300662	A/tundra swan/Niigata/5112006/2016	A / H5N6	Asia / Japan / Niigata	
EPI_ISL_300663	A/tundra swan/Niigata/5112007/2016	A / H5N6	Asia / Japan / Niigata	
EPI_ISL_300664	A/mute swan/Hyogo/2801ITM015/2017	A / H5N6	Asia / Japan / Hyogo	
EPI_ISL_300665	A/turkey/Minnesota/15-017046-1/2015	A / H5N2	North America / United States	
EPI_ISL_300666	A/turkey/Minnesota/15-017217-1/2015	A / H5N2	North America / United States	
EPI_ISL_300667	A/turkey/Minnesota/15-017218-1/2015	A / H5N2	North America / United States	
EPI_ISL_300668	A/turkey/Minnesota/15-017219-1/2015	A / H5N2	North America / United States	
EPI_ISL_300669	A/turkey/Minnesota/15-017409-1/2015	A / H5N2	North America / United States	
EPI_ISL_300670	A/turkey/Minnesota/15-017410-1/2015	A / H5N2	North America / United States	
EPI_ISL_300671	A/turkey/Minnesota/15-017540-1/2015	A / H5N2	North America / United States	
EPI_ISL_300672	A/turkey/Minnesota/15-014851-1/2015	A / H5N2	North America / United States	
EPI_ISL_300673	A/turkey/Minnesota/15-014972-1/2015	A / H5N2	North America / United States	
EPI_ISL_300674	A/turkey/Minnesota/15-016050-1/2015	A / H5N2	North America / United States	
EPI_ISL_300675	A/turkey/Minnesota/15-016876-1/2015	A / H5N2	North America / United States	
EPI_ISL_300676	A/turkey/Minnesota/15-016877-1/2015	A / H5N2	North America / United States	
EPI_ISL_300677	A/turkey/Minnesota/15-017039-1/2015	A / H5N2	North America / United States	
EPI_ISL_300678	A/turkey/Minnesota/15-017040-1/2015	A / H5N2	North America / United States	
EPI_ISL_300679	A/turkey/Minnesota/15-017044-1/2015	A / H5N2	North America / United States	
EPI_ISL_300680	A/turkey/Minnesota/15-014299-1/2015	A / H5N2	North America / United States	
EPI_ISL_300681	A/turkey/Minnesota/15-014301-1/2015	A / H5N2	North America / United States	
EPI_ISL_300682	A/turkey/Minnesota/15-014310-1/2015	A / H5N2	North America / United States	
EPI_ISL_300683	A/turkey/Minnesota/15-014311-1/2015	A / H5N2	North America / United States	
EPI_ISL_300684	A/turkey/Minnesota/15-014381-1/2015	A / H5N2	North America / United States	
EPI_ISL_300685	A/turkey/Minnesota/15-014850-1/2015	A / H5N2	North America / United States	
EPI_ISL_300686	A/turkey/Poland/63/2016	A / H5N8	Europe / Poland / Lubusz Voivodeship	Edyta ?wi?to? (National Veterinary Research Institut Poland, PIWet-PIB)
EPI_ISL_300687	A/chicken/Poland/34/2017	A / H5N8	Europe / Poland / Lesser Poland Voivodeship	Edyta ?wi?to? (National Veterinary Research Institut Poland, PIWet-PIB)
EPI_ISL_300688	A/turkey/Minnesota/15-013755-2/2015	A / H5N2	North America / United States	
EPI_ISL_300689	A/turkey/Minnesota/15-013895-1/2015	A / H5N2	North America / United States	
EPI_ISL_300690	A/turkey/Minnesota/15-013896-1/2015	A / H5N2	North America / United States	
EPI_ISL_300691	A/turkey/Minnesota/15-014095-1/2015	A / H5N2	North America / United States	
EPI_ISL_300692	A/turkey/Minnesota/15-014097-1/2015	A / H5N2	North America / United States	
EPI_ISL_300693	A/domestic_duck/Poland/47/2017	A / H5N8	Europe / Poland / Greater Poland Voivodeship	Edyta ?wi?to? (National Veterinary Research Institut Poland, PIWet-PIB)
EPI_ISL_300694	A/turkey/Minnesota/15-014098-1/2015	A / H5N2	North America / United States	
EPI_ISL_300695	A/turkey/Minnesota/15-014110-1/2015	A / H5N2	North America / United States	
EPI_ISL_300696	A/turkey/Minnesota/15-014111-1/2015	A / H5N2	North America / United States	
EPI_ISL_300697	A/turkey/Minnesota/15-014293-1/2015	A / H5N2	North America / United States	
EPI_ISL_300698	A/turkey/Poland/54/2017	A / H5N8	Europe / Poland / Masovian Voivodeship	Edyta ?wi?to? (National Veterinary Research Institut Poland, PIWet-PIB)

EPI_ISL_300699	A/domestic_goose/Poland/69/2017	A / H5N8	Europe / Poland / Greater Poland Voivodeship	Edyta ?wi?to? (National Veterinary Research Institut Poland, PIWet-PIB)
EPI_ISL_300700	A/turkey/Poland/72/2017	A / H5N8	Europe / Poland / Warmian-Masurian Voivedeship	Edyta ?wi?to? (National Veterinary Research Institut Poland, PIWet-PIB)
EPI_ISL_300701	A/turkey/Poland/94/2017	A / H5N8	Europe / Poland / Greater Poland Voivodeship	Edyta ?wi?to? (National Veterinary Research Institut Poland, PIWet-PIB)
EPI_ISL_300702	A/chicken/Poland/101/2017	A / H5N8	Europe / Poland / Lower Silesian Voivodeship	Edyta ?wi?to? (National Veterinary Research Institut Poland, PIWet-PIB)
EPI_ISL_300703	A/domestic_goose/Poland/124/2017	A / H5N8	Europe / Poland / Lower Silesian Voivodeship	Edyta ?wi?to? (National Veterinary Research Institut Poland, PIWet-PIB)
EPI_ISL_300704	A/turkey/Poland/192/2017	A / H5N8	Europe / Poland / Podlasie Voivodeship	Edyta ?wi?to? (National Veterinary Research Institut Poland, PIWet-PIB)
EPI_ISL_300705	A/turkey/Poland/285/2017	A / H5N8	Europe / Poland / Greater Poland Voivodeship	Edyta ?wi?to? (National Veterinary Research Institut Poland, PIWet-PIB)
EPI_ISL_300706	A/turkey/Minnesota/15-013181-1/2015	A / H5N2	North America / United States	
EPI_ISL_300707	A/turkey/Minnesota/15-013391-1/2015	A / H5N2	North America / United States	
EPI_ISL_300708	A/turkey/Minnesota/15-013392-1/2015	A / H5N2	North America / United States	
EPI_ISL_300709	A/turkey/Minnesota/15-013393-1/2015	A / H5N2	North America / United States	
EPI_ISL_300710	A/turkey/Minnesota/15-013395-1/2015	A / H5N2	North America / United States	
EPI_ISL_300711	A/turkey/Minnesota/15-013398-1/2015	A / H5N2	North America / United States	
EPI_ISL_300712	A/chicken/Poland/263/2017	A / H5N8	Europe / Poland / Lubusz Voivodeship	Edyta ?wi?to? (National Veterinary Research Institut Poland, PIWet-PIB)
EPI_ISL_300713	A/turkey/Minnesota/15-013399-1/2015	A / H5N2	North America / United States	
EPI_ISL_300714	A/turkey/Minnesota/15-013401-1/2015	A / H5N2	North America / United States	
EPI_ISL_300715	A/turkey/Minnesota/15-013534-1/2015	A / H5N2	North America / United States	
EPI_ISL_300716	A/turkey/Minnesota/15-012659-2/2015	A / H5N2	North America / United States	
EPI_ISL_300717	A/turkey/Minnesota/15-012661-1/2015	A / H5N2	North America / United States	
EPI_ISL_300718	A/turkey/Minnesota/15-012663-1/2015	A / H5N2	North America / United States	
EPI_ISL_300719	A/turkey/Minnesota/15-012666-1/2015	A / H5N2	North America / United States	
EPI_ISL_300720	A/turkey/Minnesota/15-012887-1/2015	A / H5N2	North America / United States	
EPI_ISL_300721	A/turkey/Minnesota/15-012888-1/2015	A / H5N2	North America / United States	
EPI_ISL_300722	A/turkey/Minnesota/15-012889-1/2015	A / H5N2	North America / United States	
EPI_ISL_300723	A/turkey/Minnesota/15-013050-1/2015	A / H5N2	North America / United States	
EPI_ISL_300724	A/turkey/Minnesota/15-013051-1/2015	A / H5N2	North America / United States	
EPI_ISL_300725	A/turkey/Minnesota/15-013052-1/2015	A / H5N2	North America / United States	
EPI_ISL_300726	A/turkey/Minnesota/15-012576-1/2015	A / H5N2	North America / United States	
EPI_ISL_300727	A/turkey/Minnesota/15-012577-1/2015	A / H5N2	North America / United States	
EPI_ISL_300728	A/turkey/Minnesota/15-012578-4/2015	A / H5N2	North America / United States	
EPI_ISL_300729	A/turkey/Minnesota/15-012580-4/2015	A / H5N2	North America / United States	
EPI_ISL_300730	A/turkey/Minnesota/15-012583-1/2015	A / H5N2	North America / United States	
EPI_ISL_300731	A/turkey/Minnesota/15-012584-1/2015	A / H5N2	North America / United States	
EPI_ISL_300732	A/turkey/Minnesota/15-012585-1/2015	A / H5N2	North America / United States	
EPI_ISL_300733	A/turkey/Minnesota/15-012658-1/2015	A / H5N2	North America / United States	
EPI_ISL_300734	A/turkey/Minnesota/15-011834-1/2015	A / H5N2	North America / United States	
EPI_ISL_300735	A/turkey/Minnesota/15-011849-1/2015	A / H5N2	North America / United States	
EPI_ISL_300736	A/turkey/Minnesota/15-011923-1/2015	A / H5N2	North America / United States	
EPI_ISL_300737	A/turkey/Minnesota/15-012289-2/2015	A / H5N2	North America / United States	
EPI_ISL_300738	A/turkey/Minnesota/15-012508-2/2015	A / H5N2	North America / United States	
EPI_ISL_300739	A/turkey/Minnesota/15-012509-1/2015	A / H5N2	North America / United States	
EPI_ISL_300740	A/turkey/Minnesota/15-012510-4/2015	A / H5N2	North America / United States	
EPI_ISL_300741	A/turkey/Minnesota/15-012574-1/2015	A / H5N2	North America / United States	
EPI_ISL_300742	A/turkey/Minnesota/15-012575-1/2015	A / H5N2	North America / United States	
EPI_ISL_300743	A/mallard/Poland/17/2017	A / H5N8	Europe / Poland / West Pomeranian Voivodeship	Edyta ?wi?to? (National Veterinary Research Institut Poland, PIWet-PIB)
EPI_ISL_300744	A/swan/Poland/32/2017	A / H5N8	Europe / Poland / Lesser Poland Voivodeship	Edyta ?wi?to? (National Veterinary Research Institut Poland, PIWet-PIB)
EPI_ISL_300745	A/wild duck/Poland/57/2017	A / H5N8	Europe / Poland / Lubusz Voivodeship	Edyta ?wi?to? (National Veterinary Research Institut Poland, PIWet-PIB)
EPI_ISL_300746	A/swan/Poland/81/2017	A / H5N5	Europe / Poland / Lower Silesian Voivodeship	Edyta ?wi?to? (National Veterinary Research Institut Poland, PIWet-PIB)
EPI_ISL_300747	A/mute_swan/Poland/137/2017	A / H5N8	Europe / Poland / Pomeranian Voivodeship	Edyta ?wi?to? (National Veterinary Research Institut Poland, PIWet-PIB)
EPI_ISL_300749	A/turkey/Poland/81/2016	A / H5N8	Europe / Poland / Lubusz Voivodeship	Edyta ?wi?to? (National Veterinary Research Institut Poland, PIWet-PIB)
EPI_ISL_300750	A/domestic_duck/Poland/88/2016	A / H5N8	Europe / Poland / Lubusz Voivodeship	Edyta ?wi?to? (National Veterinary Research Institut Poland, PIWet-PIB)
EPI_ISL_300783	A/turkey/Poland/89/2016	A / H5N8	Europe / Poland / Lubusz Voivodeship	Edyta ?wi?to? (National Veterinary Research Institut Poland, PIWet-PIB)
EPI_ISL_300808	A/turkey/Poland/93/2016	A / H5N8	Europe / Poland / Lubusz Voivodeship	Edyta ?wi?to? (National Veterinary Research Institut Poland, PIWet-PIB)
EPI_ISL_300841	A/turkey/Poland/100/2016	A / H5N8	Europe / Poland / Lubusz Voivodeship	Edyta ?wi?to? (National Veterinary Research Institut Poland, PIWet-PIB)
EPI_ISL_300872	A/chicken/Poland/103/2016	A / H5N8	Europe / Poland / Swietokrzyskie Voivodeship	Edyta ?wi?to? (National Veterinary Research Institut Poland, PIWet-PIB)
EPI_ISL_300898	A/turkey/Poland/107/2016	A / H5N8	Europe / Poland / Lubusz Voivodeship	Edyta ?wi?to? (National Veterinary Research Institut Poland, PIWet-PIB)
EPI_ISL_300920	A/domestic_duck/112/2016	A / H5N8	Europe / Poland / Lubusz Voivodeship	Edyta ?wi?to? (National Veterinary Research Institut Poland, PIWet-PIB)
EPI_ISL_300939	A/chicken/Poland/116/2016	A / H5N8	Europe / Poland / Subcarpathian Voivodeship	Edyta ?wi?to? (National Veterinary Research Institut Poland, PIWet-PIB)
EPI_ISL_300978	A/chicken/Poland/117/2016	A / H5N8	Europe / Poland / Swietokrzyskie Voivodeship	Edyta ?wi?to? (National Veterinary Research Institut Poland, PIWet-PIB)
EPI_ISL_300979	A/turkey/Poland/004/2017	A / H5N8	Europe / Poland / Lubusz Voivodeship	Edyta ?wi?to? (National Veterinary Research Institut Poland, PIWet-PIB)
EPI_ISL_300980	A/turkey/Poland/005/2017	A / H5N8	Europe / Poland / Lubusz Voivodeship	Edyta ?wi?to? (National Veterinary Research Institut Poland, PIWet-PIB)
EPI_ISL_300981	A/turkey/Poland/15/2017	A / H5N8	Europe / Poland / Lubusz Voivodeship	Edyta ?wi?to? (National Veterinary Research Institut Poland, PIWet-PIB)
EPI_ISL_301037	A/chicken/Poland/16/2017	A / H5N8	Europe / Poland / Opole Voivodeship	Edyta ?wi?to? (National Veterinary Research Institut Poland, PIWet-PIB)
EPI_ISL_301038	A/chicken/Poland/77/2017	A / H5N8	Europe / Poland / Lesser Poland Voivodeship	Edyta ?wi?to? (National Veterinary Research Institut Poland, PIWet-PIB)
EPI_ISL_301039	A/domestic_goose/Poland/190/2017	A / H5N8	Europe / Poland / Greater Poland Voivodeship	Edyta ?wi?to? (National Veterinary Research Institut Poland, PIWet-PIB)
EPI_ISL_301047	A/chicken/Poland/199/2017	A / H5N8	Europe / Poland / Masovian Voivodeship	Edyta ?wi?to? (National Veterinary Research Institut Poland, PIWet-PIB)
EPI_ISL_301048	A/chicken/Poland/208/2017	A / H5N8	Europe / Poland / Kuyavian-Pomeranian Voivodeship	Edyta ?wi?to? (National Veterinary Research Institut Poland, PIWet-PIB)
EPI_ISL_301049	A/domestic_goose/Poland/270/2017	A / H5N8	Europe / Poland / Greater Poland Voivodeship	Edyta ?wi?to? (National Veterinary Research Institut Poland, PIWet-PIB)
EPI_ISL_301050	A/mute_swan/Poland/12/2017	A / H5N8	Europe / Poland / Lower Silesian Voivodeship	Edyta ?wi?to? (National Veterinary Research Institut Poland, PIWet-PIB)
EPI_ISL_301051	A/swan/Poland/23/2017	A / H5N8	Europe / Poland / Lower Silesian Voivodeship	Edyta ?wi?to? (National Veterinary Research Institut Poland, PIWet-PIB)
EPI_ISL_301052	A/mute_swan/Poland/30/2017	A / H5N8	Europe / Poland / Lower Silesian Voivodeship	Edyta ?wi?to? (National Veterinary Research Institut Poland, PIWet-PIB)
EPI_ISL_301053	A/mallard/Poland/33/2017	A / H5N8	Europe / Poland / Masovian Voivodeship	Edyta ?wi?to? (National Veterinary Research Institut Poland, PIWet-PIB)

EPI_ISL_301054	A/swan/Poland/49/2017	A / H5N8	Europe / Poland / Lower Silesian Voivodeship	Edyta ?wi?to? (National Veterinary Research Institut Poland, PIWet-PIB)
EPI_ISL_301055	A/mute_swan/Poland/54/2017	A / H5N8	Europe / Poland / Lodz Voivodeship	Edyta ?wi?to? (National Veterinary Research Institut Poland, PIWet-PIB)
EPI_ISL_301056	A/swan/Poland/56/2017	A / H5N8	Europe / Poland / Lower Silesian Voivodeship	Edyta ?wi?to? (National Veterinary Research Institut Poland, PIWet-PIB)
EPI_ISL_301057	A/mute_swan/Poland/68/2017	A / H5N8	Europe / Poland / Lower Silesian Voivodeship	Edyta ?wi?to? (National Veterinary Research Institut Poland, PIWet-PIB)
EPI_ISL_301058	A/turkey/Minnesota/15-011602-1/2015	A / H5N2	North America / United States	
EPI_ISL_301059	A/turkey/Minnesota/15-011603-1/2015	A / H5N2	North America / United States	
EPI_ISL_301060	A/turkey/Minnesota/15-011656-1/2015	A / H5N2	North America / United States	
EPI_ISL_301061	A/turkey/Minnesota/15-011661-1/2015	A / H5N2	North America / United States	
EPI_ISL_301062	A/turkey/Minnesota/15-011666-1/2015	A / H5N2	North America / United States	
EPI_ISL_301063	A/mute_swan/Poland/72/2017	A / H5N8	Europe / Poland / Lubusz Voivodeship	Edyta ?wi?to? (National Veterinary Research Institut Poland, PIWet-PIB)
EPI_ISL_301064	A/turkey/Minnesota/15-011668-1/2015	A / H5N2	North America / United States	
EPI_ISL_301065	A/turkey/Minnesota/15-011669-1/2015	A / H5N2	North America / United States	
EPI_ISL_301066	A/turkey/Minnesota/15-011833-1/2015	A / H5N2	North America / United States	
EPI_ISL_301067	A/mute_swan/Poland/76/2017	A / H5N8	Europe / Poland / Masovian Voivodeship	Edyta ?wi?to? (National Veterinary Research Institut Poland, PIWet-PIB)
EPI_ISL_301068	A/wild_duck/Poland/78/2017	A / H5N8	Europe / Poland / Lower Silesian Voivodeship	Edyta ?wi?to? (National Veterinary Research Institut Poland, PIWet-PIB)
EPI_ISL_301069	A/swan/Poland/88/2017	A / H5N8	Europe / Poland / Greater Poland Voivodeship	Edyta ?wi?to? (National Veterinary Research Institut Poland, PIWet-PIB)
EPI_ISL_301070	A/swan/Poland/99/2017	A / H5N8	Europe / Poland / Greater Poland Voivodeship	Edyta ?wi?to? (National Veterinary Research Institut Poland, PIWet-PIB)
EPI_ISL_301071	A/turkey/Minnesota/15-010856-1/2015	A / H5N2	North America / United States	
EPI_ISL_301072	A/swan/Poland/107/2017	A / H5N8	Europe / Poland / Kuyavian-Pomeranian Voivodeship	Edyta ?wi?to? (National Veterinary Research Institut Poland, PIWet-PIB)
EPI_ISL_301073	A/turkey/Minnesota/15-010915-1/2015	A / H5N2	North America / United States	
EPI_ISL_301074	A/turkey/Minnesota/15-011079-1/2015	A / H5N2	North America / United States	
EPI_ISL_301075	A/turkey/Minnesota/15-011200-1/2015	A / H5N2	North America / United States	
EPI_ISL_301076	A/turkey/Minnesota/15-011202-1/2015	A / H5N2	North America / United States	
EPI_ISL_301077	A/turkey/Minnesota/15-011591-1/2015	A / H5N2	North America / United States	
EPI_ISL_301078	A/mute_swan/Poland/109/2017	A / H5N8	Europe / Poland / Kuyavian-Pomeranian Voivodeship	Edyta ?wi?to? (National Veterinary Research Institut Poland, PIWet-PIB)
EPI_ISL_301079	A/turkey/Minnesota/15-011593-1/2015	A / H5N2	North America / United States	
EPI_ISL_301080	A/turkey/Minnesota/15-011596-1/2015	A / H5N2	North America / United States	
EPI_ISL_301081	A/turkey/Iowa/15-017224-1/2015	A / H5N2	North America / United States	
EPI_ISL_301082	A/turkey/Iowa/15-017421-1/2015	A / H5N2	North America / United States	
EPI_ISL_301083	A/turkey/Iowa/15-017556-1/2015	A / H5N2	North America / United States	
EPI_ISL_301084	A/turkey/Iowa/15-017557-1/2015	A / H5N2	North America / United States	
EPI_ISL_301085	A/turkey/Iowa/15-017655-1/2015	A / H5N2	North America / United States	
EPI_ISL_301086	A/turkey/Iowa/15-017824-1/2015	A / H5N2	North America / United States	
EPI_ISL_301087	A/turkey/Minnesota/15-010375-2/2015	A / H5N2	North America / United States	
EPI_ISL_301088	A/turkey/Minnesota/15-010560-1/2015	A / H5N2	North America / United States	
EPI_ISL_301089	A/turkey/Minnesota/15-010777-1/2015	A / H5N2	North America / United States	
EPI_ISL_301090	A/turkey/Iowa/15-014868-1/2015	A / H5N2	North America / United States	
EPI_ISL_301091	A/turkey/Iowa/15-014869-1/2015	A / H5N2	North America / United States	
EPI_ISL_301092	A/turkey/Iowa/15-014954-1/2015	A / H5N2	North America / United States	
EPI_ISL_301093	A/turkey/Iowa/15-015071-3/2015	A / H5N2	North America / United States	
EPI_ISL_301094	A/turkey/Iowa/15-015116-1/2015	A / H5N2	North America / United States	
EPI_ISL_301095	A/turkey/Iowa/15-016077-1/2015	A / H5N2	North America / United States	
EPI_ISL_301096	A/turkey/Iowa/15-016082-1/2015	A / H5N2	North America / United States	
EPI_ISL_301097	A/turkey/Iowa/15-016365-1/2015	A / H5N2	North America / United States	
EPI_ISL_301098	A/turkey/Iowa/15-016743-1/2015	A / H5N2	North America / United States	
EPI_ISL_301099	A/turkey/Iowa/15-014789-1/2015	A / H5N2	North America / United States	
EPI_ISL_301100	A/turkey/Iowa/15-014862-1/2015	A / H5N2	North America / United States	
EPI_ISL_301101	A/turkey/Iowa/15-014863-1/2015	A / H5N2	North America / United States	
EPI_ISL_301102	A/turkey/Iowa/15-014864-1/2015	A / H5N2	North America / United States	
EPI_ISL_301103	A/turkey/Iowa/15-013773-1/2015	A / H5N2	North America / United States	
EPI_ISL_301104	A/turkey/Iowa/15-013926-1/2015	A / H5N2	North America / United States	
EPI_ISL_301105	A/turkey/Iowa/15-014119-1/2015	A / H5N2	North America / United States	
EPI_ISL_301106	A/turkey/Iowa/15-014120-1/2015	A / H5N2	North America / United States	
EPI_ISL_301107	A/turkey/Iowa/15-014562-2/2015	A / H5N2	North America / United States	
EPI_ISL_301108	A/turkey/Iowa/15-014570-1/2015	A / H5N2	North America / United States	
EPI_ISL_301109	A/turkey/Iowa/15-014772-1/2015	A / H5N2	North America / United States	
EPI_ISL_301110	A/turkey/Iowa/15-013179-4/2015	A / H5N2	North America / United States	
EPI_ISL_301111	A/great-horned owl/Idaho/15-001583-11/2015	A / H5N2	North America / United States	
EPI_ISL_301112	A/guinea fowl/Iowa/15-018413-1/2015	A / H5N2	North America / United States	
EPI_ISL_301113	A/peregrine falcon/Idaho/15-001362-3/2015	A / H5N2	North America / United States	
EPI_ISL_301114	A/red-tailed hawk/Washington/15-002551-2/2015	A / H5N2	North America / United States	
EPI_ISL_301115	A/ring-necked duck/Kentucky/15-012967-1/2015	A / H5N2	North America / United States	
EPI_ISL_301116	A/snow goose/Kentucky/15-012967-2/2015	A / H5N2	North America / United States	
EPI_ISL_301117	A/great-horned owl/Idaho/15-003155-1/2015	A / H5N2	North America / United States	
EPI_ISL_301545	A/chicken/Nebraska/15-016865-1/2015	A / H5N2	North America / United States	
EPI_ISL_301546	A/chicken/Nebraska/15-019197-1/2015	A / H5N2	North America / United States	
EPI_ISL_301547	A/chicken/Oregon/15-005256-1/2015	A / H5N2	North America / United States	
EPI_ISL_301548	A/chicken/South Dakota/15-015847-3/2015	A / H5N2	North America / United States	
EPI_ISL_301549	A/chicken/Wisconsin/15-013062-1/2015	A / H5N2	North America / United States	
EPI_ISL_301550	A/chicken/Wisconsin/15-014400-1/2015	A / H5N2	North America / United States	
EPI_ISL_301551	A/falcon/Missouri/15-009166-2/2015	A / H5N2	North America / United States	
EPI_ISL_301552	A/goose/Washington/15-001678-1/2015	A / H5N2	North America / United States	

EPI_ISL_301553	A/chicken/Iowa/15-019293-1/2015	A / H5N2	North America / United States	
EPI_ISL_301554	A/chicken/Minnesota/15-012694-1/2015	A / H5N2	North America / United States	
EPI_ISL_301555	A/chicken/Minnesota/15-012882-2/2015	A / H5N2	North America / United States	
EPI_ISL_301556	A/chicken/Minnesota/15-014297-1/2015	A / H5N2	North America / United States	
EPI_ISL_301557	A/chicken/Minnesota/15-015844-1/2015	A / H5N2	North America / United States	
EPI_ISL_301558	A/chicken/Minnesota/15-017817-1/2015	A / H5N2	North America / United States	
EPI_ISL_301559	A/chicken/Missouri/15-014275-1/2015	A / H5N2	North America / United States	
EPI_ISL_301560	A/chicken/Nebraska/15-015085-1/2015	A / H5N2	North America / United States	
EPI_ISL_301561	A/chicken/Nebraska/15-015543-1/2015	A / H5N2	North America / United States	
EPI_ISL_301562	A/chicken/Iowa/15-016078-1/2015	A / H5N2	North America / United States	
EPI_ISL_301563	A/chicken/Iowa/15-016158-1/2015	A / H5N2	North America / United States	
EPI_ISL_301564	A/chicken/Iowa/15-016159-1/2015	A / H5N2	North America / United States	
EPI_ISL_301565	A/chicken/Iowa/15-016160-1/2015	A / H5N2	North America / United States	
EPI_ISL_301566	A/chicken/Iowa/15-016887-1/2015	A / H5N2	North America / United States	
EPI_ISL_301567	A/chicken/Iowa/15-016888-1/2015	A / H5N2	North America / United States	
EPI_ISL_301568	A/chicken/Iowa/15-017038-1/2015	A / H5N2	North America / United States	
EPI_ISL_301569	A/chicken/Iowa/15-017419-1/2015	A / H5N2	North America / United States	
EPI_ISL_301570	A/chicken/Iowa/15-017656-1/2015	A / H5N2	North America / United States	
EPI_ISL_301571	A/chicken/Iowa/15-014774-1/2015	A / H5N2	North America / United States	
EPI_ISL_301572	A/chicken/Iowa/15-015016-1/2015	A / H5N2	North America / United States	
EPI_ISL_301573	A/chicken/Iowa/15-015018-1/2015	A / H5N2	North America / United States	
EPI_ISL_301574	A/chicken/Iowa/15-015073-1/2015	A / H5N2	North America / United States	
EPI_ISL_301575	A/chicken/Iowa/15-015117-1/2015	A / H5N2	North America / United States	
EPI_ISL_301576	A/chicken/Iowa/15-015300-1/2015	A / H5N2	North America / United States	
EPI_ISL_301577	A/chicken/Iowa/15-015544-2/2015	A / H5N2	North America / United States	
EPI_ISL_301578	A/chicken/Iowa/15-015555-1/2015	A / H5N2	North America / United States	
EPI_ISL_301579	A/chicken/Iowa/15-015556-1/2015	A / H5N2	North America / United States	
EPI_ISL_301580	A/chicken/Iowa/15-013982-1/2015	A / H5N2	North America / United States	
EPI_ISL_301581	A/chicken/Iowa/15-014283-1/2015	A / H5N2	North America / United States	
EPI_ISL_301582	A/chicken/Iowa/15-014294-1/2015	A / H5N2	North America / United States	
EPI_ISL_301583	A/chicken/Iowa/15-014572-1/2015	A / H5N2	North America / United States	
EPI_ISL_301584	A/chicken/Iowa/15-014586-1/2015	A / H5N2	North America / United States	
EPI_ISL_301585	A/chicken/Iowa/15-014591-1/2015	A / H5N2	North America / United States	
EPI_ISL_301586	A/chicken/Iowa/15-014719-1/2015	A / H5N2	North America / United States	
EPI_ISL_301587	A/chicken/Iowa/15-014769-1/2015	A / H5N2	North America / United States	
EPI_ISL_301588	A/chicken/Idaho/15-001711-7/2015	A / H5N2	North America / United States	
EPI_ISL_301589	A/chicken/Iowa/15-012564-7/2015	A / H5N2	North America / United States	
EPI_ISL_301590	A/chicken/Iowa/15-013408-3/2015	A / H5N2	North America / United States	
EPI_ISL_301591	A/chicken/Iowa/15-013430-16/2015	A / H5N2	North America / United States	
EPI_ISL_301592	A/chicken/Iowa/15-013540-6/2015	A / H5N2	North America / United States	
EPI_ISL_301593	A/chicken/Iowa/15-013784-2/2015	A / H5N2	North America / United States	
EPI_ISL_301594	A/chicken/Iowa/15-013972-1/2015	A / H5N2	North America / United States	
EPI_ISL_301595	A/chicken/Iowa/15-013976-6/2015	A / H5N2	North America / United States	
EPI_ISL_301596	A/Canada goose/Wyoming/15-009285-1/2015	A / H5N2	North America / United States	
EPI_ISL_301611	A/Canada goose/Michigan/15-018340-2/2015	A / H5N2	North America / United States	
EPI_ISL_301612	A/Canada goose/Michigan/15-019873-5/2015	A / H5N2	North America / United States	
EPI_ISL_301782	A/white-tailed_eagle/Denmark/3073-1w/2018-02-13	A / H5N6	Europe / Denmark / Region Sjaland / Slagelse Kommune	Charlotte Kristiane Hjulsager (Statens Serum Institute / Microbiological Diagnostic and Virology)
EPI_ISL_301784	A/Mallard/Korea/K17-1825/2017	A / H5N6	Asia / Korea, Republic of / Gyeonggi-do / Cheongmi-cheon (37°06'56.9"N 127°25'18.3"E)	Jung Hoon Kwon (Konkuk University / College of Veterinary Medicine)
EPI_ISL_301785	A/Mandarin_duck/Korea/K17-1815/2017	A / H5N6	Asia / Korea, Republic of / Gyeonggi-do / Cheongmi-cheon (37°06'56.9"N 127°25'18.3"E)	Jung Hoon Kwon (Konkuk University / College of Veterinary Medicine)
EPI_ISL_301786	A/Mandarin_duck/Korea/K17-1817/2017	A / H5N6	Asia / Korea, Republic of / Gyeonggi-do / Cheongmi-cheon (37°06'56.9"N 127°25'18.3"E)	Jung Hoon Kwon (Konkuk University / College of Veterinary Medicine)
EPI_ISL_301787	A/Mandarin_duck/Korea/K17-1826/2017	A / H5N6	Asia / Korea, Republic of / Gyeonggi-do / Cheongmi-cheon (37°06'56.9"N 127°25'18.3"E)	Jung Hoon Kwon (Konkuk University / College of Veterinary Medicine)
EPI_ISL_301803	A/Mandarin_duck/Korea/K17-1828/2017	A / H5N6	Asia / Korea, Republic of / Gyeonggi-do / Cheongmi-cheon (37°06'56.9"N 127°25'18.3"E)	Jung Hoon Kwon (Konkuk University / College of Veterinary Medicine)
EPI_ISL_302523	A/Anas platyrhynchos/Korea/W612/2017	A / H5N6	Asia / Korea, Republic of	
EPI_ISL_302524	A/Anas platyrhynchos/Korea/W613/2017	A / H5N6	Asia / Korea, Republic of	
EPI_ISL_302525	A/Anas platyrhynchos/Korea/W614/2017	A / H5N6	Asia / Korea, Republic of	
EPI_ISL_302526	A/Anas platyrhynchos/Korea/W615/2017	A / H5N6	Asia / Korea, Republic of	
EPI_ISL_3026043	A/European herring gull/Sweden/SVA210617SZ0354/FB00225	A / H5N8	Europe / Sweden / Gotlands Lan / Gotlands Kommun	Siamak Zohari (National Veterinary Institute)
EPI_ISL_3026699	A/common eider/Sweden/SVA210617SZ0354/FB002252/I-202	A / H5N8	Europe / Sweden / Gotlands Lan / Gotlands Kommun	Siamak Zohari (National Veterinary Institute)
EPI_ISL_302708	A/Great_Cormorant/Hubei/chenhu_V11/2015	A / H5N6	Asia / China / Hubei Province	Jianjun Chen (Wuhan Institute of Virology / Chinese Academy of Sciences)
EPI_ISL_302823	A/Great black-backed gull/Netherlands/1/2018	A / H5N6	Europe / Netherlands / Provincie Noord-Holland / De Kreupel	Maria Johanna Poen (Erasmus Medical Center / Department of Virology)
EPI_ISL_302824	A/Eurasian wigeon/Netherlands/1/2018	A / H5N6	Europe / Netherlands / Provincie Noord-Holland / Hippolytushoef	Maria Johanna Poen (Erasmus Medical Center / Department of Virology)
EPI_ISL_302825	A/Chicken/Netherlands/EMC-1/2018	A / H5N6	Europe / Netherlands / Provincie Groningen / Gemeente Grootegast	Maria Johanna Poen (Erasmus Medical Center / Department of Virology)
EPI_ISL_302826	A/Chicken/Netherlands/EMC-14/2018	A / H5N6	Europe / Netherlands / Provincie Groningen / Gemeente Grootegast	Maria Johanna Poen (Erasmus Medical Center / Department of Virology)
EPI_ISL_303520	A/Mallard/Republic of Georgia/1/2018	A / H5N6	Asia / Georgia	Maria Johanna Poen (Erasmus Medical Center / Department of Virology)
EPI_ISL_303630	A/Mandarin_duck/Korea/K17-1862/2017	A / H5N6	Asia / Korea, Republic of / Chungcheongnam-do / Gokgyo-cheon (36°45?12.3?N, 127°07?12.7?E	Jung Hoon Kwon (Konkuk University / College of Veterinary Medicine)
EPI_ISL_303631	A/Mandarin_duck/Korea/K17-1866/2017	A / H5N6	Asia / Korea, Republic of / Chungcheongnam-do / Gokgyo-cheon (36°45?12.3?N, 127°07?12.7?E	Jung Hoon Kwon (Konkuk University / College of Veterinary Medicine)
EPI_ISL_303632	A/Mandarin_duck/Korea/K17-1869/2017	A / H5N6	Asia / Korea, Republic of / Chungcheongnam-do / Gokgyo-cheon (36°45?12.3?N, 127°07?12.7?E	Jung Hoon Kwon (Konkuk University / College of Veterinary Medicine)
EPI_ISL_303633	A/Mandarin_duck/Korea/K17-1873/2017	A / H5N6	Asia / Korea, Republic of / Chungcheongnam-do / Gokgyo-cheon (36°45?12.3?N, 127°07?12.7?E	Jung Hoon Kwon (Konkuk University / College of Veterinary Medicine)
EPI_ISL_303634	A/Mandarin_duck/Korea/K17-1879/2017	A / H5N6	Asia / Korea, Republic of / Chungcheongnam-do / Gokgyo-cheon (36°45?12.3?N, 127°07?12.7?E	Jung Hoon Kwon (Konkuk University / College of Veterinary Medicine)
EPI_ISL_303635	A/Mandarin_duck/Korea/K17-1881/2017	A / H5N6	Asia / Korea, Republic of / Chungcheongnam-do / Gokgyo-cheon (36°45?12.3?N, 127°07?12.7?E	Jung Hoon Kwon (Konkuk University / College of Veterinary Medicine)
EPI_ISL_303636	A/Mandarin_duck/Korea/K17-1885/2017	A / H5N6	Asia / Korea, Republic of / Chungcheongnam-do / Gokgyo-cheon (36°45?12.3?N, 127°07?12.7?E	Jung Hoon Kwon (Konkuk University / College of Veterinary Medicine)
EPI_ISL_303637	A/Mandarin_duck/Korea/K17-1887/2017	A / H5N6	Asia / Korea, Republic of / Chungcheongnam-do / Gokgyo-cheon (36°45?12.3?N, 127°07?12.7?E	Jung Hoon Kwon (Konkuk University / College of Veterinary Medicine)

EPI_ISL_303638	A/Mandarin_duck/Korea/K17-1889/2017	A / H5N6	Asia / Korea, Republic of / Chungcheongnam-do / Gokgyo-cheon (36°45?12.3?N, 127°07?12.7?E)	Jung Hoon Kwon (Konkuk University / College of Veterinary Medicine)
EPI_ISL_303639	A/Mandarin_duck/Korea/K17-1891/2017	A / H5N6	Asia / Korea, Republic of / Chungcheongnam-do / Gokgyo-cheon (36°45?12.3?N, 127°07?12.7?E)	Jung Hoon Kwon (Konkuk University / College of Veterinary Medicine)
EPI_ISL_303640	A/Mandarin_duck/Korea/K17-1893/2017	A / H5N6	Asia / Korea, Republic of / Chungcheongnam-do / Gokgyo-cheon (36°45?12.3?N, 127°07?12.7?E)	Jung Hoon Kwon (Konkuk University / College of Veterinary Medicine)
EPI_ISL_303641	A/Mandarin_duck/Korea/K17-1894/2017	A / H5N6	Asia / Korea, Republic of / Chungcheongnam-do / Gokgyo-cheon (36°45?12.3?N, 127°07?12.7?E)	Jung Hoon Kwon (Konkuk University / College of Veterinary Medicine)
EPI_ISL_303642	A/Mandarin_duck/Korea/K17-1896/2017	A / H5N6	Asia / Korea, Republic of / Chungcheongnam-do / Gokgyo-cheon (36°45?12.3?N, 127°07?12.7?E)	Jung Hoon Kwon (Konkuk University / College of Veterinary Medicine)
EPI_ISL_303643	A/Mandarin_duck/Korea/K18-3/2018	A / H5N6	Asia / Korea, Republic of / Chungcheongnam-do / Gokgyo-cheon (36°45?12.3?N, 127°07?12.7?E)	Jung Hoon Kwon (Konkuk University / College of Veterinary Medicine)
EPI_ISL_303837	A/grey-headed gull/Uganda/MUWRP-538/2017	A / H5N8	Africa / Uganda	
EPI_ISL_304404	A/Fujian-Sanyuan/21099/2017	A / H5N6	Asia / China / Fujian Province	Lei Yang (WHO Chinese National Influenza Center / Virology Institute, Chinese CDC)
EPI_ISL_304956	A/duck/Vietnam/QuangBinh/DH130723/2017	A / H5N6	Asia / Vietnam	
EPI_ISL_304957	A/duck/Vietnam/QuangBinh/DH330718/2017	A / H5N6	Asia / Vietnam	
EPI_ISL_304958	A/chicken/Vietnam/QuangBinh/BD1113/2017	A / H5N6	Asia / Vietnam	
EPI_ISL_304959	A/chicken/Vietnam/QuangBinh/BoTrach1113/2017	A / H5N6	Asia / Vietnam	
EPI_ISL_304960	A/duck/Vietnam/QuangBinh/QN530206/2018	A / H5N6	Asia / Vietnam	
EPI_ISL_305416	A/Domestic_Duck/Netherlands/EMC-2/2018	A / H5N6	Europe / Netherlands / Provincie Overijssel / Gemeente Kampen / Commercial poultry farm	Maria Johanna Poen (Erasmus Medical Center / Department of Virology)
EPI_ISL_305417	A/Domestic_Duck/Netherlands/EMC-6/2018	A / H5N6	Europe / Netherlands / Provincie Overijssel / Gemeente Kampen / Commercial poultry farm	Maria Johanna Poen (Erasmus Medical Center / Department of Virology)
EPI_ISL_305453	A/chicken/Germany-SH/AR163-L02542/2018	A / H5N6	Europe / Germany / Schleswig-Holstein / Nordfriesland	Anne Pohlmann (Friedrich-Loeffler-Institut)
EPI_ISL_305454	A/chicken/Germany-SH/AR164-L02543/2018	A / H5N6	Europe / Germany / Schleswig-Holstein / Nordfriesland	Anne Pohlmann (Friedrich-Loeffler-Institut)
EPI_ISL_305455	A/duck/Germany-SH/AR165-L02544/2018	A / H5N6	Europe / Germany / Schleswig-Holstein / Nordfriesland	Anne Pohlmann (Friedrich-Loeffler-Institut)
EPI_ISL_306910	A/Turkey/Riyadh/AI1/2017	A / H5N8	Asia / Saudi Arabia / Riyadh	Gavin Smith (The University of Hong Kong / Department of Microbiology)
EPI_ISL_306911	A/Duck/Riyadh/AI2/2017	A / H5N8	Asia / Saudi Arabia / Riyadh	Gavin Smith (The University of Hong Kong / Department of Microbiology)
EPI_ISL_306912	A/Chicken/Riyadh/AI6/2017	A / H5N8	Asia / Saudi Arabia / Riyadh	Gavin Smith (The University of Hong Kong / Department of Microbiology)
EPI_ISL_306913	A/Chicken/Al-Ahsaa/AI7/2017	A / H5N8	Asia / Saudi Arabia / Al-Ahsaa	Gavin Smith (The University of Hong Kong / Department of Microbiology)
EPI_ISL_306914	A/Chicken/Riyadh/AI10/2017	A / H5N8	Asia / Saudi Arabia / Riyadh	Gavin Smith (The University of Hong Kong / Department of Microbiology)
EPI_ISL_306915	A/Chicken/Riyadh/A15/2018	A / H5N8	Asia / Saudi Arabia / Riyadh	Gavin Smith (The University of Hong Kong / Department of Microbiology)
EPI_ISL_306955	A/Bulbul/Riyadh/AI4/2017	A / H5N8	Asia / Saudi Arabia / Riyadh	Gavin Smith (The University of Hong Kong / Department of Microbiology)
EPI_ISL_306956	A/Falcon/Riyadh/AI5/2017	A / H5N8	Asia / Saudi Arabia / Riyadh	Gavin Smith (The University of Hong Kong / Department of Microbiology)
EPI_ISL_306957	A/Chicken/Al-Ahsaa/AI8/2017	A / H5N8	Asia / Saudi Arabia / Al-Ahsaa	Gavin Smith (The University of Hong Kong / Department of Microbiology)
EPI_ISL_306958	A/Ornamental bird/Al-Qasim/AI9/2017	A / H5N8	Asia / Saudi Arabia / Al-Qasim	Gavin Smith (The University of Hong Kong / Department of Microbiology)
EPI_ISL_306959	A/Holland pigeon/Riyadh/AI3/2017	A / H5N8	Asia / Saudi Arabia / Riyadh	Gavin Smith (The University of Hong Kong / Department of Microbiology)
EPI_ISL_306989	A/turkey/Germany-SH/AR185-L02549/2018	A / H5N6	Europe / Germany / Schleswig-Holstein / Nordfriesland	Anne Pohlmann (Friedrich-Loeffler-Institut)
EPI_ISL_308781	A/jungle crow/Hyogo/2803A002/2018	A / H5N6	Asia / Japan / Hyogo	
EPI_ISL_308782	A/jungle crow/Hyogo/2803E011/2018	A / H5N6	Asia / Japan / Hyogo	
EPI_ISL_308783	A/jungle crow/Hyogo/2803E022/2018	A / H5N6	Asia / Japan / Hyogo	
EPI_ISL_308802	A/duck/Democratic Republic of the Congo/17RS882-29/2017	A / H5N8	Africa / Congo, the Democatic Republic of	
EPI_ISL_308803	A/duck/Democratic Republic of the Congo/17RS882-33/2017	A / H5N8	Africa / Congo, the Democatic Republic of	
EPI_ISL_308804	A/duck/Democratic Republic of the Congo/17RS882-40/2017	A / H5N8	Africa / Congo, the Democatic Republic of	
EPI_ISL_308805	A/duck/Democratic Republic of the Congo/17RS882-5/2017	A / H5N8	Africa / Congo, the Democatic Republic of	
EPI_ISL_308806	A/chicken/Cameroon/17RS1661-1/2017	A / H5N8	Africa / Cameroon	
EPI_ISL_308807	A/duck/Cameroon/17RS1661-3/2017	A / H5N8	Africa / Cameroon	
EPI_ISL_308808	A/Indian peafowl/Cameroon/17RS1661-6/2017	A / H5N8	Africa / Cameroon	
EPI_ISL_308809	A/pigeon/Cameroon/17RS1661-4/2017	A / H5N8	Africa / Cameroon	
EPI_ISL_3090099	A/Sichuan/06681/2021	A / H5N6	Asia / China / Sichuan Province	Lei Yang (WHO Chinese National Influenza Center / Virology Institute, Chinese CDC)
EPI_ISL_309194	A/Anas platyrhynchos/Belgium/1899/2017	A / H5N8	Europe / Belgium	
EPI_ISL_309195	A/Cygnus olor/Belgium/2967/2017	A / H5N8	Europe / Belgium	
EPI_ISL_309196	A/Buteo buteo/Belgium/3022/2017	A / H5N8	Europe / Belgium	
EPI_ISL_309198	A/Guinea fowl/Belgium/6102/2017	A / H5N8	Europe / Belgium	
EPI_ISL_309199	A/quail/Vietnam/CVVI-01/2015	A / H5N6	Asia / Vietnam	
EPI_ISL_309200	A/quail/Vietnam/CVVI-03/2015	A / H5N6	Asia / Vietnam	
EPI_ISL_3098710	A/common eider/Sweden/SVA210617SZ0354/FB002253/I-202	A / H5N8	Europe / Sweden / Gotlands Lan / Gotlands Kommun	Siamak Zohari (National Veterinary Institute)
EPI_ISL_3098719	A/peregrine falcon/Sweden/SVA210309SZ0403/FB000851/K-2(	A / H5N4	Europe / Sweden / Blekinge Lan / Solvesborgs Kommun	Siamak Zohari (National Veterinary Institute)
EPI_ISL_3102024	A/barnacle goose/Sweden/SVA210423SZ0250/FB001648/M-2(	A / H5N8	Europe / Sweden / Skane Lan / Trelleborgs Kommun	Siamak Zohari (National Veterinary Institute)
EPI_ISL_3102029	A/barnacle goose/Sweden/SVA210423SZ0252/FB001647/M20	A / H5N8	Europe / Sweden / Skane Lan / Vellinge Kommun	Siamak Zohari (National Veterinary Institute)
EPI_ISL_3102054	A/European_herring_gull/Bulgaria/222_21VIR4270-1/2021	A / H5N5	Europe / Bulgaria / Kiten, Burgas	Bianca Zecchin (Istituto Zooprofilattico Sperimentale Delle Venezie)
EPI_ISL_3102055	A/grey_heron/Bulgaria/223_21VIR4270-2/2021	A / H5N5	Europe / Bulgaria / Kiten, Burgas	Bianca Zecchin (Istituto Zooprofilattico Sperimentale Delle Venezie)
EPI_ISL_3102056	A/chicken/Bulgaria/274-5_21VIR4270-4/2021	A / H5N8	Europe / Bulgaria / Krivo pole, Haskovo	Bianca Zecchin (Istituto Zooprofilattico Sperimentale Delle Venezie)
EPI_ISL_3102057	A/chicken/Bulgaria/275-4_21VIR4270-6/2021	A / H5N8	Europe / Bulgaria / Trilistnik, Plovdiv	Bianca Zecchin (Istituto Zooprofilattico Sperimentale Delle Venezie)
EPI_ISL_3102058	A/chicken/Bulgaria/297-2_21VIR4270-8/2021	A / H5N8	Europe / Bulgaria / Trilistnik, Plovdiv	Bianca Zecchin (Istituto Zooprofilattico Sperimentale Delle Venezie)
EPI_ISL_3102059	A/chicken/Bulgaria/298-1_21VIR4270-9/2021	A / H5N8	Europe / Bulgaria / Trilistnik, Plovdiv	Bianca Zecchin (Istituto Zooprofilattico Sperimentale Delle Venezie)
EPI_ISL_3102060	A/duck/Poland/H160_21RS1385-1/2021	A / H5N8	Europe / Poland / Wielkopolskie	Bianca Zecchin (Istituto Zooprofilattico Sperimentale Delle Venezie)
EPI_ISL_3102061	A/duck/Poland/H542_21RS1385-2/2021	A / H5N8	Europe / Poland / Wielkopolskie	Bianca Zecchin (Istituto Zooprofilattico Sperimentale Delle Venezie)
EPI_ISL_3102062	A/chicken/Poland/H1124_21RS1385-3/2021	A / H5N8	Europe / Poland / Wielkopolskie	Bianca Zecchin (Istituto Zooprofilattico Sperimentale Delle Venezie)
EPI_ISL_3102063	A/goose/Poland/H1044_21RS1385-4/2021	A / H5N8	Europe / Poland / Wielkopolskie	Bianca Zecchin (Istituto Zooprofilattico Sperimentale Delle Venezie)
EPI_ISL_3102064	A/duck/Poland/H1029_21RS1385-5/2021	A / H5N8	Europe / Poland / Wielkopolskie	Bianca Zecchin (Istituto Zooprofilattico Sperimentale Delle Venezie)
EPI_ISL_3102065	A/goose/Poland/H1036_21RS1385-6/2021	A / H5N8	Europe / Poland / Wielkopolskie	Bianca Zecchin (Istituto Zooprofilattico Sperimentale Delle Venezie)
EPI_ISL_3102066	A/chicken/Poland/H812_21RS1385-7/2021	A / H5N8	Europe / Poland / Wielkopolskie	Bianca Zecchin (Istituto Zooprofilattico Sperimentale Delle Venezie)
EPI_ISL_3102067	A/chicken/Poland/H293_21RS1385-8/2021	A / H5N8	Europe / Poland / Mazowieckie	Bianca Zecchin (Istituto Zooprofilattico Sperimentale Delle Venezie)
EPI_ISL_3102068	A/chicken/Poland/H984_21RS1385-9/2021	A / H5N8	Europe / Poland / Mazowieckie	Bianca Zecchin (Istituto Zooprofilattico Sperimentale Delle Venezie)
EPI_ISL_3102069	A/chicken/Poland/H1161_21RS1385-10/2021	A / H5N8	Europe / Poland / Mazowieckie	Bianca Zecchin (Istituto Zooprofilattico Sperimentale Delle Venezie)
EPI_ISL_3102070	A/white_stork/Poland/MB412_21RS1385-11/2021	A / H5N8	Europe / Poland / Mazowieckie	Bianca Zecchin (Istituto Zooprofilattico Sperimentale Delle Venezie)
EPI_ISL_3102071	A/chicken/Poland/H712_21RS1385-12/2021	A / H5N8	Europe / Poland / Mazowieckie	Bianca Zecchin (Istituto Zooprofilattico Sperimentale Delle Venezie)
EPI_ISL_3102072	A/chicken/Poland/H1084_21RS1385-13/2021	A / H5N8	Europe / Poland / Mazowieckie	Bianca Zecchin (Istituto Zooprofilattico Sperimentale Delle Venezie)
EPI_ISL_3102073	A/turkey/Poland/H1184_21RS1385-14/2021	A / H5N8	Europe / Poland / Mazowieckie	Bianca Zecchin (Istituto Zooprofilattico Sperimentale Delle Venezie)

EPI_ISL_3102074	A/turkey/Poland/H1168_21RS1385-15/2021	A / H5N8	Europe / Poland / Warmi?sko-mazurskie	Bianca Zecchin (Istituto Zooprofilattico Sperimentale Delle Venezie)
EPI_ISL_3102075	A/chicken/Poland/H928_21RS1385-16/2021	A / H5N8	Europe / Poland / Warmi?sko-mazurskie	Bianca Zecchin (Istituto Zooprofilattico Sperimentale Delle Venezie)
EPI_ISL_3102076	A/duck/Poland/H515_21RS1385-17/2021	A / H5N8	Europe / Poland / Lodzkie	Bianca Zecchin (Istituto Zooprofilattico Sperimentale Delle Venezie)
EPI_ISL_3102077	A/duck/Poland/H514_21RS1385-18/2021	A / H5N8	Europe / Poland / Pomorskie	Bianca Zecchin (Istituto Zooprofilattico Sperimentale Delle Venezie)
EPI_ISL_3102078	A/mute_swan/Poland/MB396_21RS1385-19/2021	A / H5N8	Europe / Poland / Zachodniopomorskie	Bianca Zecchin (Istituto Zooprofilattico Sperimentale Delle Venezie)
EPI_ISL_3102079	A/turkey/Poland/H1289_21RS1385-20/2021	A / H5N8	Europe / Poland / Mazowieckie	Bianca Zecchin (Istituto Zooprofilattico Sperimentale Delle Venezie)
EPI_ISL_3102080	A/chicken/Austria/21052483_21VIR3291-1/2021	A / H5N8	Europe / Austria / Oberneukirchen	Bianca Zecchin (Istituto Zooprofilattico Sperimentale Delle Venezie)
EPI_ISL_3102081	A/mute_swan/Romania/11981-1_21VIR3163-5/2021	A / H5N5	Europe / Romania / Tulcea Chilia Veche	Bianca Zecchin (Istituto Zooprofilattico Sperimentale Delle Venezie)
EPI_ISL_3102082	A/mute_swan/Romania/11981-2_21VIR3163-6/2021	A / H5N8	Europe / Romania / Tulcea Chilia Veche	Bianca Zecchin (Istituto Zooprofilattico Sperimentale Delle Venezie)
EPI_ISL_3102208	A/barnacle goose/Sweden/SVA210210SZ0372/FB000374/M-2(	A / H5N8	Europe / Sweden / Skane Lan / Svedala Kommun	Siamak Zohari (National Veterinary Institute)
EPI_ISL_312376	A/Armenian Gull/Republic of Georgia/4/2017	A / H5N6	Asia / Georgia / Mtkvari River, Tbilisi	Maria Johanna Poen (Erasmus Medical Center / Department of Virology)
EPI_ISL_3127850	A/whooper swan/Sweden/SVA210331SZ0441/FB0001325/W-2	A / H5N8	Europe / Sweden / Dalarnas Lan / Leksands Kommun	Siamak Zohari (National Veterinary Institute)
EPI_ISL_3128152	A/Canada goose/Sweden/SVA210331SZ/FB001326/W-2021	A / H5N8	Europe / Sweden / Dalarnas Lan / Leksands Kommun	Siamak Zohari (National Veterinary Institute)
EPI_ISL_3128522	A/Canada goose/Sweden/SVA210407SZ0510/FB001379/O-202	A / H5N8	Europe / Sweden / Vastra Gotalands Lan / Hjo Kommun	Siamak Zohari (National Veterinary Institute)
EPI_ISL_3128523	A/dalmatian pelican/Greece/47t_21VIR3735-3/2021	A / H5N8	Europe / Greece / Serres	Bianca Zecchin (Istituto Zooprofilattico Sperimentale Delle Venezie)
EPI_ISL_3128524	A/partridge/Kosovo/96_21VIR5162-14/2021	A / H5N8	Europe / Kosovo / Suhareke	Bianca Zecchin (Istituto Zooprofilattico Sperimentale Delle Venezie)
EPI_ISL_3128525	A/chicken/Kosovo/97_21VIR5162-15/2021	A / H5N8	Europe / Kosovo / Gjilan	Bianca Zecchin (Istituto Zooprofilattico Sperimentale Delle Venezie)
EPI_ISL_3128526	A/chicken/Kosovo/98_21VIR5162-16/2021	A / H5N8	Europe / Kosovo / Mitrovice	Bianca Zecchin (Istituto Zooprofilattico Sperimentale Delle Venezie)
EPI_ISL_3128527	A/duck/Kosovo/107_21VIR5162-17/2021	A / H5N8	Europe / Kosovo / Kacanik	Bianca Zecchin (Istituto Zooprofilattico Sperimentale Delle Venezie)
EPI_ISL_3128528	A/chicken/Kosovo/113_21VIR5162-19/2021	A / H5N8	Europe / Kosovo / Shtime	Bianca Zecchin (Istituto Zooprofilattico Sperimentale Delle Venezie)
EPI_ISL_3128529	A/chicken/Kosovo/82_21VIR5162-1/2021	A / H5N8	Europe / Kosovo / Mitrovica	Bianca Zecchin (Istituto Zooprofilattico Sperimentale Delle Venezie)
EPI_ISL_3128530	A/chicken/Kosovo/84_21VIR5162-2/2021	A / H5N8	Europe / Kosovo / Ferizaj	Bianca Zecchin (Istituto Zooprofilattico Sperimentale Delle Venezie)
EPI_ISL_3128531	A/chicken/Kosovo/90_21VIR5162-4/2021	A / H5N8	Europe / Kosovo / Rahovec	Bianca Zecchin (Istituto Zooprofilattico Sperimentale Delle Venezie)
EPI_ISL_3128532	A/chicken/Kosovo/104_21VIR5162-7/2021	A / H5N8	Europe / Kosovo / Malisheve	Bianca Zecchin (Istituto Zooprofilattico Sperimentale Delle Venezie)
EPI_ISL_3128533	A/chicken/Albania/2574_21VIR5387-1/2021	A / H5N8	Europe / Albania	Bianca Zecchin (Istituto Zooprofilattico Sperimentale Delle Venezie)
EPI_ISL_3128534	A/chicken/Albania/3816_21VIR5387-2/2021	A / H5N8	Europe / Albania	Bianca Zecchin (Istituto Zooprofilattico Sperimentale Delle Venezie)
EPI_ISL_313226	A/white stork/Germany-NI/AR251/2018	A / H5N6	Europe / Germany / Lower Saxony / Vechta	Jacqueline King (Friedrich-Loeffler-Institut)
EPI_ISL_313227	A/buzzard/Germany-NRW/AR279/2018	A / H5N6	Europe / Germany / North Rhine-Westphalia / Herford	Jacqueline King (Friedrich-Loeffler-Institut)
EPI_ISL_3135897	A/Turkey/Hungary/16603/2021 (H5N1)	A / H5N1	Europe / Hungary / Hajdu-Bihar	Katalin Szentpáli-Gavallér (National Food Chain Safety Office, Hungary / Virology)
EPI_ISL_3135926	A/eagle/Hungary/8569/2021 (H5N5)	A / H5N5	Europe / Hungary / Somogy megye	Katalin Szentpáli-Gavallér (National Food Chain Safety Office, Hungary / Virology)
EPI_ISL_3135931	A/swan/Hungary/9638/2021 (H5N8)	A / H5N8	Europe / Hungary / Csongrad megye	Katalin Szentpáli-Gavallér (National Food Chain Safety Office, Hungary / Virology)
EPI_ISL_313805	A/Northern Goshawk/Tokyo/1301B003T/2018	A / H5N6	Asia / Japan	Takehiko Saito (National Institute of Animal Health)
EPI_ISL_313806	A/chicken/Kagawa/1T-1/2018	A / H5N6	Asia / Japan	Takehiko Saito (National Institute of Animal Health)
EPI_ISL_3138567	A/common buzzard/Sweden/SVA210415SZ0338/FB001522/O-	A / H5N8	Europe / Sweden / Vastra Gotalands Lan / Boras Kommun	Siamak Zohari (National Veterinary Institute)
EPI_ISL_3138618	A/whooper swan/Sweden/SVA210420SZ0485/FB0001581/W-2	A / H5N8	Europe / Sweden / Dalarnas Lan / Borlange Kommun	Siamak Zohari (National Veterinary Institute)
EPI_ISL_3142375	A/mute_swan/Austria/21051907_21VIR3291-6/2021	A / H5N8	Europe / Austria / Oberneukirchen	Bianca Zecchin (Istituto Zooprofilattico Sperimentale Delle Venezie)
EPI_ISL_3142391	A/mute_swan/Austria/21051907_21VIR3291-7/2021	A / H5N8	Europe / Austria / Oberneukirchen	Bianca Zecchin (Istituto Zooprofilattico Sperimentale Delle Venezie)
EPI_ISL_3142407	A/turkey/Kosovo/90_21VIR5162-5/2021	A / H5N8	Europe / Kosovo / Rahovec	Bianca Zecchin (Istituto Zooprofilattico Sperimentale Delle Venezie)
EPI_ISL_3143953	A/common buzzard/Sweden/SVA210505SZ0374/FB0001789/N	A / H5N8	Europe / Sweden / Skane Lan / Bastads Kommun	Siamak Zohari (National Veterinary Institute)
EPI_ISL_3143954	A/whooper swan/Sweden/SVA2100329SZ0210/FB0001277/H-:	A / H5N5	Europe / Sweden / Kalmar Lan / Hongsby Kommun	Siamak Zohari (National Veterinary Institute)
EPI_ISL_314821	A/chicken/Kagawa/1C-1/2018	A / H5N6	Asia / Japan / Kagawa	Takehiko Saito (National Institute of Animal Health)
EPI_ISL_314856	A/chicken/Guangdong/G1012/2018	A / H5N6	Asia / China / Guangdong Province / live bird market	Jiming Chen (China Animal Health and Epidemiology Center (CAHEC))
EPI_ISL_314857	A/duck/Guangdong/G1378/2018	A / H5N6	Asia / China / Guangdong Province / live bird market	Jiming Chen (China Animal Health and Epidemiology Center (CAHEC))
EPI_ISL_315236	A/Enviroment/Xuzhou/4018/2018	A / H5N6	Asia / China / Jiangsu Province	Xian Qi (Jiangsu Provincial Center for Disease Control & Prevention)
EPI_ISL_315237	A/Chicken/Xuzhou/470/2017	A / H5N6	Asia / China / Jiangsu Provice	Xian Qi (Jiangsu Provincial Center for Disease Control & Prevention)
EPI_ISL_315238	A/Enviroment/Xuzhou/461/2017	A / H5N6	Asia / China / Jiangsu Provice	Xian Qi (Jiangsu Provincial Center for Disease Control & Prevention)
EPI_ISL_316513	A/chicken/Italy/17VIR2294-5/2017	A / H5N8	Europe / Italy / Treviso	Bianca Zecchin (Istituto Zooprofilattico Sperimentale Delle Venezie)
EPI_ISL_316514	A/chicken/Italy/17VIR2658-3/2017	A / H5N8	Europe / Italy / Pordenone	Bianca Zecchin (Istituto Zooprofilattico Sperimentale Delle Venezie)
EPI_ISL_316515	A/turkey/Italy/17VIR2722-29/2017	A / H5N8	Europe / Italy / Verona	Bianca Zecchin (Istituto Zooprofilattico Sperimentale Delle Venezie)
EPI_ISL_316516	A/chicken/Italy/17VIR2788/2017	A / H5N8	Europe / Italy / Torino	Bianca Zecchin (Istituto Zooprofilattico Sperimentale Delle Venezie)
EPI_ISL_316517	A/turkey/Italy/17VIR3192-1/2017	A / H5N8	Europe / Italy / Verona	Bianca Zecchin (Istituto Zooprofilattico Sperimentale Delle Venezie)
EPI_ISL_316518	A/turkey/Italy/17VIR4407-1/2017	A / H5N8	Europe / Italy / Mantova	Bianca Zecchin (Istituto Zooprofilattico Sperimentale Delle Venezie)
EPI_ISL_316519	A/turkey/Italy/17VIR5773-2/2017	A / H5N8	Europe / Italy / Mantova	Bianca Zecchin (Istituto Zooprofilattico Sperimentale Delle Venezie)
EPI_ISL_316520	A/duck/Italy/17VIR5769-5/2017	A / H5N8	Europe / Italy / Mantova	Bianca Zecchin (Istituto Zooprofilattico Sperimentale Delle Venezie)
EPI_ISL_316521	A/chicken/Italy/17VIR5794-5/2017	A / H5N8	Europe / Italy / Mantova	Bianca Zecchin (Istituto Zooprofilattico Sperimentale Delle Venezie)
EPI_ISL_316522	A/turkey/Italy/17VIR5964-8/2017	A / H5N8	Europe / Italy / Verona	Bianca Zecchin (Istituto Zooprofilattico Sperimentale Delle Venezie)
EPI_ISL_316523	A/chicken/Italy/17VIR5986-1/2017	A / H5N8	Europe / Italy / Mantova	Bianca Zecchin (Istituto Zooprofilattico Sperimentale Delle Venezie)
EPI_ISL_316524	A/turkey/Italy/17VIR6108-3/2017	A / H5N8	Europe / Italy / Verona	Bianca Zecchin (Istituto Zooprofilattico Sperimentale Delle Venezie)
EPI_ISL_316525	A/turkey/Italy/17VIR6111-16/2017	A / H5N8	Europe / Italy / Parma	Bianca Zecchin (Istituto Zooprofilattico Sperimentale Delle Venezie)
EPI_ISL_316526	A/turkey/Italy/17VIR6355-1/2017	A / H5N8	Europe / Italy / Mantova	Bianca Zecchin (Istituto Zooprofilattico Sperimentale Delle Venezie)
EPI_ISL_316527	A/turkey/Italy/17VIR6356-1/2017	A / H5N8	Europe / Italy / Mantova	Bianca Zecchin (Istituto Zooprofilattico Sperimentale Delle Venezie)
EPI_ISL_316528	A/turkey/Italy/17VIR6357-2/2017	A / H5N8	Europe / Italy / Mantova	Bianca Zecchin (Istituto Zooprofilattico Sperimentale Delle Venezie)
EPI_ISL_316529	A/turkey/Italy/17VIR6469-2/2017	A / H5N8	Europe / Italy / Mantova	Bianca Zecchin (Istituto Zooprofilattico Sperimentale Delle Venezie)
EPI_ISL_316530	A/turkey/Italy/17VIR6882-1/2017	A / H5N8	Europe / Italy / Verona	Bianca Zecchin (Istituto Zooprofilattico Sperimentale Delle Venezie)
EPI_ISL_316531	A/pheasant/Italy/17VIR7065-21/2017	A / H5N8	Europe / Italy / Lodi	Bianca Zecchin (Istituto Zooprofilattico Sperimentale Delle Venezie)
EPI_ISL_316532	A/turkey/Italy/17VIR6930-4/2017	A / H5N8	Europe / Italy / Verona	Bianca Zecchin (Istituto Zooprofilattico Sperimentale Delle Venezie)
EPI_ISL_316533	A/turkey/Italy/17VIR6928-5/2017	A / H5N8	Europe / Italy / Verona	Bianca Zecchin (Istituto Zooprofilattico Sperimentale Delle Venezie)
EPI_ISL_316534	A/chicken/Italy/17VIR7062-1/2017	A / H5N8	Europe / Italy / Pavia	Bianca Zecchin (Istituto Zooprofilattico Sperimentale Delle Venezie)
EPI_ISL_316535	A/turkey/Italy/17VIR7197-1/2017	A / H5N8	Europe / Italy / Verona	Bianca Zecchin (Istituto Zooprofilattico Sperimentale Delle Venezie)
EPI_ISL_316536	A/turkey/Italy/17VIR7337-1/2017	A / H5N8	Europe / Italy / Cremona	Bianca Zecchin (Istituto Zooprofilattico Sperimentale Delle Venezie)
EPI_ISL_316537	A/goose/Italy/17VIR7626-5/2017	A / H5N8	Europe / Italy / Padova	Bianca Zecchin (Istituto Zooprofilattico Sperimentale Delle Venezie)
EPI_ISL_316538	A/turkey/Italy/17VIR8199-1/2017	A / H5N8	Europe / Italy / Vicenza	Bianca Zecchin (Istituto Zooprofilattico Sperimentale Delle Venezie)

EPI_ISL_316539	A/turkey/Italy/17VIR8275-3/2017	A / H5N8	Europe / Italy / Verona	Bianca Zecchin (Istituto Zooprofilattico Sperimentale Delle Venezie)
EPI_ISL_316540	A/duck/Italy/17VIR8200-12/2017	A / H5N8	Europe / Italy / Vicenza	Bianca Zecchin (Istituto Zooprofilattico Sperimentale Delle Venezie)
EPI_ISL_316541	A/duck/Italy/17VIR8349/2017	A / H5N8	Europe / Italy / Cremona	Bianca Zecchin (Istituto Zooprofilattico Sperimentale Delle Venezie)
EPI_ISL_316542	A/chicken/Italy/17VIR8678/2017	A / H5N8	Europe / Italy / Ferrara	Bianca Zecchin (Istituto Zooprofilattico Sperimentale Delle Venezie)
EPI_ISL_316543	A/chicken/Italy/17VIR8783-5/2017	A / H5N8	Europe / Italy / Vicenza	Bianca Zecchin (Istituto Zooprofilattico Sperimentale Delle Venezie)
EPI_ISL_316544	A/chicken/Italy/17VIR8821/2017	A / H5N8	Europe / Italy / Bergamo	Bianca Zecchin (Istituto Zooprofilattico Sperimentale Delle Venezie)
EPI_ISL_316545	A/chicken/Italy/17VIR8744-9/2017	A / H5N8	Europe / Italy / Vicenza	Bianca Zecchin (Istituto Zooprofilattico Sperimentale Delle Venezie)
EPI_ISL_316546	A/chicken/Italy/17VIR8885/2017	A / H5N8	Europe / Italy / Bergamo	Bianca Zecchin (Istituto Zooprofilattico Sperimentale Delle Venezie)
EPI_ISL_316547	A/turkey/Italy/17VIR8886-3/2017	A / H5N8	Europe / Italy / Padova	Bianca Zecchin (Istituto Zooprofilattico Sperimentale Delle Venezie)
EPI_ISL_316548	A/turkey/Italy/17VIR8819-3/2017	A / H5N8	Europe / Italy / Brescia	Bianca Zecchin (Istituto Zooprofilattico Sperimentale Delle Venezie)
EPI_ISL_316549	A/chicken/Italy/17VIR9048-2/2017	A / H5N8	Europe / Italy / Bergamo	Bianca Zecchin (Istituto Zooprofilattico Sperimentale Delle Venezie)
EPI_ISL_316550	A/turkey/Italy/17VIR9106-2/2017	A / H5N8	Europe / Italy / Brescia	Bianca Zecchin (Istituto Zooprofilattico Sperimentale Delle Venezie)
EPI_ISL_316551	A/chicken/Italy/17VIR9107/2017	A / H5N8	Europe / Italy / Sondrio	Bianca Zecchin (Istituto Zooprofilattico Sperimentale Delle Venezie)
EPI_ISL_316552	A/turkey/Italy/17VIR9049-2/2017	A / H5N8	Europe / Italy / Mantova	Bianca Zecchin (Istituto Zooprofilattico Sperimentale Delle Venezie)
EPI_ISL_316553	A/chicken/Italy/17VIR9113/2017	A / H5N8	Europe / Italy / Padova	Bianca Zecchin (Istituto Zooprofilattico Sperimentale Delle Venezie)
EPI_ISL_316554	A/duck/Italy/17VIR8898/2017	A / H5N8	Europe / Italy / Padova	Bianca Zecchin (Istituto Zooprofilattico Sperimentale Delle Venezie)
EPI_ISL_316555	A/turkey/Italy/17VIR9245/2017	A / H5N8	Europe / Italy / Brescia	Bianca Zecchin (Istituto Zooprofilattico Sperimentale Delle Venezie)
EPI_ISL_316556	A/turkey/Italy/17VIR9246/2017	A / H5N8	Europe / Italy / Brescia	Bianca Zecchin (Istituto Zooprofilattico Sperimentale Delle Venezie)
EPI_ISL_316557	A/chicken/Italy/17VIR9456/2017	A / H5N8	Europe / Italy / Milano	Bianca Zecchin (Istituto Zooprofilattico Sperimentale Delle Venezie)
EPI_ISL_316558	A/duck/Italy/17VIR9533/2017	A / H5N8	Europe / Italy / Brescia	Bianca Zecchin (Istituto Zooprofilattico Sperimentale Delle Venezie)
EPI_ISL_316559	A/chicken/Italy/17VIR9679/2017	A / H5N8	Europe / Italy / Brescia	Bianca Zecchin (Istituto Zooprofilattico Sperimentale Delle Venezie)
EPI_ISL_316560	A/chicken/Italy/17VIR9680/2017	A / H5N8	Europe / Italy / Brescia	Bianca Zecchin (Istituto Zooprofilattico Sperimentale Delle Venezie)
EPI_ISL_316561	A/turkey/Italy/17VIR9699/2017	A / H5N8	Europe / Italy / Brescia	Bianca Zecchin (Istituto Zooprofilattico Sperimentale Delle Venezie)
EPI_ISL_316562	A/turkey/Italy/17VIR9773/2017	A / H5N8	Europe / Italy / Brescia	Bianca Zecchin (Istituto Zooprofilattico Sperimentale Delle Venezie)
EPI_ISL_316563	A/turkey/Italy/17VIR9775-1/2017	A / H5N8	Europe / Italy / Bergamo	Bianca Zecchin (Istituto Zooprofilattico Sperimentale Delle Venezie)
EPI_ISL_316564	A/turkey/Italy/17VIR9908/2017	A / H5N8	Europe / Italy / Brescia	Bianca Zecchin (Istituto Zooprofilattico Sperimentale Delle Venezie)
EPI_ISL_316565	A/turkey/Italy/17VIR9905-1/2017	A / H5N8	Europe / Italy / Brescia	Bianca Zecchin (Istituto Zooprofilattico Sperimentale Delle Venezie)
EPI_ISL_316566	A/duck/Italy/17VIR9906-1/2017	A / H5N8	Europe / Italy / Brescia	Bianca Zecchin (Istituto Zooprofilattico Sperimentale Delle Venezie)
EPI_ISL_316567	A/duck/Italy/17VIR9907-2/2017	A / H5N8	Europe / Italy / Brescia	Bianca Zecchin (Istituto Zooprofilattico Sperimentale Delle Venezie)
EPI_ISL_316568	A/turkey/Italy/17VIR9942-2/2017	A / H5N8	Europe / Italy / Brescia	Bianca Zecchin (Istituto Zooprofilattico Sperimentale Delle Venezie)
EPI_ISL_316569	A/chicken/Italy/17VIR9941-1/2017	A / H5N8	Europe / Italy / Brescia	Bianca Zecchin (Istituto Zooprofilattico Sperimentale Delle Venezie)
EPI_ISL_316570	A/chicken/Italy/17VIR9846-4/2017	A / H5N8	Europe / Italy / Asti	Bianca Zecchin (Istituto Zooprofilattico Sperimentale Delle Venezie)
EPI_ISL_316571	A/chicken/Italy/17VIR10053/2017	A / H5N8	Europe / Italy / Brescia	Bianca Zecchin (Istituto Zooprofilattico Sperimentale Delle Venezie)
EPI_ISL_316572	A/chicken/Italy/17VIR9868-1/2017	A / H5N8	Europe / Italy / Roma	Bianca Zecchin (Istituto Zooprofilattico Sperimentale Delle Venezie)
EPI_ISL_316573	A/turkey/Italy/17VIR10133-8/2017	A / H5N8	Europe / Italy / Cremona	Bianca Zecchin (Istituto Zooprofilattico Sperimentale Delle Venezie)
EPI_ISL_316574	A/chicken/Italy/17VIR10083/2017	A / H5N8	Europe / Italy / Brescia	Bianca Zecchin (Istituto Zooprofilattico Sperimentale Delle Venezie)
EPI_ISL_316575	A/chicken/Italy/17VIR10082/2017	A / H5N8	Europe / Italy / Brescia	Bianca Zecchin (Istituto Zooprofilattico Sperimentale Delle Venezie)
EPI_ISL_316576	A/chicken/Italy/17VIR10130-2/2017	A / H5N8	Europe / Italy / Brescia	Bianca Zecchin (Istituto Zooprofilattico Sperimentale Delle Venezie)
EPI_ISL_316577	A/chicken/Italy/17VIR10132-1/2017	A / H5N8	Europe / Italy / Brescia	Bianca Zecchin (Istituto Zooprofilattico Sperimentale Delle Venezie)
EPI_ISL_316578	A/chicken/Italy/17VIR10131-1/2017	A / H5N8	Europe / Italy / Brescia	Bianca Zecchin (Istituto Zooprofilattico Sperimentale Delle Venezie)
EPI_ISL_316579	A/chicken/Italy/17VIR10437-2/2017	A / H5N8	Europe / Italy / Brescia	Bianca Zecchin (Istituto Zooprofilattico Sperimentale Delle Venezie)
EPI_ISL_316580	A/chicken/Italy/17VIR10408/2017	A / H5N8	Europe / Italy / Treviso	Bianca Zecchin (Istituto Zooprofilattico Sperimentale Delle Venezie)
EPI_ISL_316581	A/duck/Italy/17VIR10767-13/2017	A / H5N8	Europe / Italy / Treviso	Bianca Zecchin (Istituto Zooprofilattico Sperimentale Delle Venezie)
EPI_ISL_316582	A/turkey/Italy/17VIR10983-22/2017	A / H5N8	Europe / Italy / Ravenna	Bianca Zecchin (Istituto Zooprofilattico Sperimentale Delle Venezie)
EPI_ISL_316583	A/swan/Italy/17VIR3594-3/2017	A / H5N8	Europe / Italy / Torino	Bianca Zecchin (Istituto Zooprofilattico Sperimentale Delle Venezie)
EPI_ISL_316584	A/swan/Italy/17VIR8513/2017	A / H5N8	Europe / Italy / Como	Bianca Zecchin (Istituto Zooprofilattico Sperimentale Delle Venezie)
EPI_ISL_316585	A/swan/Italy/17VIR9017/2017	A / H5N8	Europe / Italy / Padova	Bianca Zecchin (Istituto Zooprofilattico Sperimentale Delle Venezie)
EPI_ISL_316586	A/swan/Italy/17VIR9038-1/2017	A / H5N8	Europe / Italy / Verbano-Cusio-Ossola	Bianca Zecchin (Istituto Zooprofilattico Sperimentale Delle Venezie)
EPI_ISL_316587	A/swan/Italy/17VIR9038-2/2017	A / H5N8	Europe / Italy / Verbano-Cusio-Ossola	Bianca Zecchin (Istituto Zooprofilattico Sperimentale Delle Venezie)
EPI_ISL_316588	A/swan/Italy/17VIR9836-1/2017	A / H5N8	Europe / Italy / Verbano-Cusio-Ossola	Bianca Zecchin (Istituto Zooprofilattico Sperimentale Delle Venezie)
EPI_ISL_316589	A/quail/Italy/17VIR9455-2/2017	A / H5N8	Europe / Italy / Bergamo	Bianca Zecchin (Istituto Zooprofilattico Sperimentale Delle Venezie)
EPI_ISL_316591	A/kestrel/Italy/17VIR9840/2017	A / H5N8	Europe / Italy / Ferrara	Bianca Zecchin (Istituto Zooprofilattico Sperimentale Delle Venezie)
EPI_ISL_316592	A/mallard/Italy/17VIR6103-1/2017	A / H5N8	Europe / Italy / Pavia	Bianca Zecchin (Istituto Zooprofilattico Sperimentale Delle Venezie)
EPI_ISL_316593	A/pigeon/Italy/17VIR9842/2017	A / H5N8	Europe / Italy / Ferrara	Bianca Zecchin (Istituto Zooprofilattico Sperimentale Delle Venezie)
EPI_ISL_316594	A/wild_goose/Italy/17VIR9841/2017	A / H5N8	Europe / Italy / Ferrara	Bianca Zecchin (Istituto Zooprofilattico Sperimentale Delle Venezie)
EPI_ISL_319652	A/tufted duck/Shimane/3211TY001/2017	A / H5N6	Asia / Japan / Shimane	
EPI_ISL_320545	A/White-tailed sea eagle/Germany/AR3089/2017	A / H5	Europe / Germany / Mecklenburg-Vorpommern	Timm Harder (Friedrich-Loeffler-Institut)
EPI_ISL_320546	A/White-tailed sea eagle/Germany/AR3093/2017	A / H5N8	Europe / Germany / Mecklenburg-Vorpommern	Timm Harder (Friedrich-Loeffler-Institut)
EPI_ISL_320574	A/White-tailed sea eagle/Germany/AR10297/2016	A / H5N8	Europe / Germany / Schleswig-Holstein	Timm Harder (Friedrich-Loeffler-Institut)
EPI_ISL_320607	A/chicken/Kursk/284/2018	A / H5N8	Europe / Russian Federation / Kursk Oblast	Ivan Susloparov (State Research Center of Virology and Biotechnology (VECTOR) / Emerging Zoonotic Diseases and Influenza)
EPI_ISL_320608	A/chicken/Kursk/526/2018	A / H5N8	Europe / Russian Federation / Kursk Oblast	Ivan Susloparov (State Research Center of Virology and Biotechnology (VECTOR) / Emerging Zoonotic Diseases and Influenza)
EPI_ISL_320657	A/chicken/Samara/446/2018	A / H5N8	Europe / Russian Federation / Samara Oblast	Ivan Susloparov (State Research Center of Virology and Biotechnology (VECTOR) / Emerging Zoonotic Diseases and Influenza)
EPI_ISL_320658	A/chicken/Samara/447/2018	A / H5N8	Europe / Russian Federation / Samara Oblast	Ivan Susloparov (State Research Center of Virology and Biotechnology (VECTOR) / Emerging Zoonotic Diseases and Influenza)
EPI_ISL_320659	A/duck/Samara/452/2018	A / H5N8	Europe / Russian Federation / Samara Oblast	Ivan Susloparov (State Research Center of Virology and Biotechnology (VECTOR) / Emerging Zoonotic Diseases and Influenza)
EPI_ISL_320660	A/goose/Samara/455/2018	A / H5N8	Europe / Russian Federation / Samara Oblast	Ivan Susloparov (State Research Center of Virology and Biotechnology (VECTOR) / Emerging Zoonotic Diseases and Influenza)
EPI_ISL_320661	A/goose/Samara/459/2018	A / H5N8	Europe / Russian Federation / Samara Oblast	Ivan Susloparov (State Research Center of Virology and Biotechnology (VECTOR) / Emerging Zoonotic Diseases and Influenza)
EPI_ISL_320662	A/chicken/Penza/300/2018	A / H5N8	Europe / Russian Federation / Penza Oblast	Ivan Susloparov (State Research Center of Virology and Biotechnology (VECTOR) / Emerging Zoonotic Diseases and Influenza)
EPI_ISL_320663	A/chicken/Penza/301/2018	A / H5N8	Europe / Russian Federation / Penza Oblast	Ivan Susloparov (State Research Center of Virology and Biotechnology (VECTOR) / Emerging Zoonotic Diseases and Influenza)
EPI_ISL_320664	A/chicken/Orel/533/2018	A / H5N8	Europe / Russian Federation / Oryol Oblast	Ivan Susloparov (State Research Center of Virology and Biotechnology (VECTOR) / Emerging Zoonotic Diseases and Influenza)
EPI_ISL_320674	A/chicken/Rostov-on-Don/766/2018	A / H5N8	Europe / Russian Federation / Rostov Oblast	Ivan Susloparov (State Research Center of Virology and Biotechnology (VECTOR) / Emerging Zoonotic Diseases and Influenza)
EPI_ISL_320675	A/turkey/Rostov-on-Don/817/2018	A / H5N8	Europe / Russian Federation / Rostov Oblast	Ivan Susloparov (State Research Center of Virology and Biotechnology (VECTOR) / Emerging Zoonotic Diseases and Influenza)
EPI_ISL_320676	A/chicken/Penza/605/2018	A / H5N8	Europe / Russian Federation / Penza Oblast	Ivan Susloparov (State Research Center of Virology and Biotechnology (VECTOR) / Emerging Zoonotic Diseases and Influenza)

EPI_ISL_320677	A/chicken/Penza/607/2018	A / H5N8	Europe / Russian Federation / Penza Oblast	Ivan Susloparov (State Research Center of Virology and Biotechnology (VECTOR) / Emerging Zoonotic Diseases and Influenza)
EPI_ISL_320678	A/chicken/Cheboksary/805/2018	A / H5N8	Europe / Russian Federation / Chuvash Republic-Chuvashia	Ivan Susloparov (State Research Center of Virology and Biotechnology (VECTOR) / Emerging Zoonotic Diseases and Influenza)
EPI_ISL_320679	A/chicken/Cheboksary/806/2018	A / H5N8	Europe / Russian Federation / Chuvash Republic-Chuvashia	Ivan Susloparov (State Research Center of Virology and Biotechnology (VECTOR) / Emerging Zoonotic Diseases and Influenza)
EPI_ISL_320680	A/chicken/Kursk/757/2018	A / H5N8	Europe / Russian Federation / Kursk Oblast	Ivan Susloparov (State Research Center of Virology and Biotechnology (VECTOR) / Emerging Zoonotic Diseases and Influenza)
EPI_ISL_320681	A/chicken/Kursk/760/2018	A / H5N8	Europe / Russian Federation / Kursk Oblast	Ivan Susloparov (State Research Center of Virology and Biotechnology (VECTOR) / Emerging Zoonotic Diseases and Influenza)
EPI_ISL_320682	A/chicken/Kursk/762/2018	A / H5N8	Europe / Russian Federation / Kursk Oblast	Ivan Susloparov (State Research Center of Virology and Biotechnology (VECTOR) / Emerging Zoonotic Diseases and Influenza)
EPI_ISL_320683	A/goose/Samara/673/2018	A / H5N8	Europe / Russian Federation / Samara Oblast	Ivan Susloparov (State Research Center of Virology and Biotechnology (VECTOR) / Emerging Zoonotic Diseases and Influenza)
EPI_ISL_320684	A/goose/Samara/675/2018	A / H5N8	Europe / Russian Federation / Samara Oblast	Ivan Susloparov (State Research Center of Virology and Biotechnology (VECTOR) / Emerging Zoonotic Diseases and Influenza)
EPI_ISL_320685	A/chicken/Samara/679/2018	A / H5N8	Europe / Russian Federation / Samara Oblast	Ivan Susloparov (State Research Center of Virology and Biotechnology (VECTOR) / Emerging Zoonotic Diseases and Influenza)
EPI_ISL_320953	A/chicken/Cheboksary/849/2018	A / H5N8	Europe / Russian Federation / Chuvash Republic-Chuvashia	Ivan Susloparov (State Research Center of Virology and Biotechnology (VECTOR) / Emerging Zoonotic Diseases and Influenza)
EPI_ISL_320954	A/chicken/Cheboksary/850/2018	A / H5N8	Europe / Russian Federation / Chuvash Republic-Chuvashia	Ivan Susloparov (State Research Center of Virology and Biotechnology (VECTOR) / Emerging Zoonotic Diseases and Influenza)
EPI_ISL_320955	A/chicken/Cheboksary/851/2018	A / H5N8	Europe / Russian Federation / Chuvash Republic-Chuvashia	Ivan Susloparov (State Research Center of Virology and Biotechnology (VECTOR) / Emerging Zoonotic Diseases and Influenza)
EPI_ISL_320956	A/chicken/Cheboksary/853/2018	A / H5N8	Europe / Russian Federation / Chuvash Republic-Chuvashia	Ivan Susloparov (State Research Center of Virology and Biotechnology (VECTOR) / Emerging Zoonotic Diseases and Influenza)
EPI_ISL_320957	A/chicken/Cheboksary/854/2018	A / H5N8	Europe / Russian Federation / Chuvash Republic-Chuvashia	Ivan Susloparov (State Research Center of Virology and Biotechnology (VECTOR) / Emerging Zoonotic Diseases and Influenza)
EPI_ISL_320958	A/chicken/Tatarstan/7/2018	A / H5N8	Europe / Russian Federation / Republic of Tatarstan	Ivan Susloparov (State Research Center of Virology and Biotechnology (VECTOR) / Emerging Zoonotic Diseases and Influenza)
EPI_ISL_322179	A/domestic duck/Germany-MV/AR613-L02727/2018	A / H5N6	Europe / Germany / Mecklenburg-Vorpommern / Nordwestmecklenburg	Jacqueline King (Friedrich-Loeffler-Institut)
EPI_ISL_322984	A/Grey seal/361-10/BalticPL/16	A / H5N8	Europe / Poland	Dai-Lun Shin (Stiftung Tierärztliche Hochschule Hannover)
EPI_ISL_3290184	A/Canada Goose/Sweden/SVA210330SZ0426/FB001307/E-202	A / H5N8	Europe / Sweden / Ostergotlands Lan / Norrkopings Kommun	Siamak Zohari (National Veterinary Institute)
EPI_ISL_3294584	A/Mute Swan/Sweden/SVA210330SZ0417/FB001305/D-2021	A / H5N5	Europe / Sweden / Sodermanlands Lan / Oxelosunds Kummun	Siamak Zohari (National Veterinary Institute)
EPI_ISL_3298195	A/common buzzard/Sweden/SVA210329SZ0208/FB0001278/M	A / H5N8	Europe / Sweden / Skane Lan / Simrishamns Kommun	Siamak Zohari (National Veterinary Institute)
EPI_ISL_3298234	A/peregrine falcon/Sweden/SVA210325SZ0348/FP001210/H-2	A / H5N4	Europe / Sweden / Kalmar Lan / Vasterviks Kommun	Siamak Zohari (National Veterinary Institute)
EPI_ISL_3298300	A/Greylag goose/Sweden/SVA210324SZ0403/FB001169/N-202	A / H5N8	Europe / Sweden / Hallands Lan / Varbergs Kommun	Siamak Zohari (National Veterinary Institute)
EPI_ISL_331223	A/Duck/England/1279/2014	A / H5N8	Europe / United Kingdom / East Riding of Yorkshire	Alex Byrne (Animal and Plant Health Agency (APHA) / Virology Department)
EPI_ISL_3314946	A/common buzzard/Sweden/SVA10324SZ0359/FB001163/M-2	A / H5N8	Europe / Sweden / Skane Lan	Siamak Zohari (National Veterinary Institute)
EPI_ISL_3314973	A/common buzzard/Sweden/SVA210323SZ0426/FB001120/O-	A / H5N8	Europe / Sweden / Vastra Gotalands Lan / Lidkopings Kommun	Siamak Zohari (National Veterinary Institute)
EPI_ISL_3315298	A/whooper swan/Sweden/SVA210318SZ0304/FB0001089/M-2	A / H5N8	Europe / Sweden / Skane Lan / Simrishamns Kommun	Siamak Zohari (National Veterinary Institute)
EPI_ISL_3319316	A/common eider/Sweden/SVA210729SZ0320/FB002613/I-202	A / H5N8	Europe / Sweden / Gotlands Lan / Gotlands Kommun	Siamak Zohari (National Veterinary Institute)
EPI_ISL_3319859	A/common eider/Sweden/SVA210729SZ0323/FB002615/I-202	A / H5N8	Europe / Sweden / Gotlands Lan / Gotlands Kommun	Siamak Zohari (National Veterinary Institute)
EPI_ISL_332400	A/Jiangsu/1/2018	A / H5N6	Asia / China	Xian Qi (Jiangsu Provincial Center for Disease Control & Prevention)
EPI_ISL_332430	A/Chicken/Netherlands/18003041-001-005/2018	A / H5N6	Europe / Netherlands / Oldekerk	Saskia Bergervoet (Wageningen Bioveterinary Research)
EPI_ISL_332431	A/Duck/Netherlands/18003885-001-005/2018	A / H5N6	Europe / Netherlands / Kamperveen	Saskia Bergervoet (Wageningen Bioveterinary Research)
EPI_ISL_332432	A/Guineafowl/Netherlands/17017775-020-024/2017	A / H5N6	Europe / Netherlands / Biddinghuizen	Saskia Bergervoet (Wageningen Bioveterinary Research)
EPI_ISL_332433	A/Bird/Netherlands/17017775-035-039/2017	A / H5N6	Europe / Netherlands / Biddinghuizen	Saskia Bergervoet (Wageningen Bioveterinary Research)
EPI_ISL_332434	A/Peacock/Netherlands/17017775-064-068/2017	A / H5N6	Europe / Netherlands / Biddinghuizen	Saskia Bergervoet (Wageningen Bioveterinary Research)
EPI_ISL_332435	A/Chicken/Netherlands/18000887-005/2018	A / H5N6	Europe / Netherlands / Rhoon	Saskia Bergervoet (Wageningen Bioveterinary Research)
EPI_ISL_332436	A/Peacock/Netherlands/18000887-006/2018	A / H5N6	Europe / Netherlands / Rhoon	Saskia Bergervoet (Wageningen Bioveterinary Research)
EPI_ISL_332437	A/Turkey/Netherlands/18000887-007-010/2018	A / H5N6	Europe / Netherlands / Rhoon	Saskia Bergervoet (Wageningen Bioveterinary Research)
EPI_ISL_332438	A/Mute swan/Netherlands/17017903-002/2017	A / H5N6	Europe / Netherlands / Elburg	Saskia Bergervoet (Wageningen Bioveterinary Research)
EPI_ISL_332439	A/Perigrine falcon/Netherlands/18003274-001/2018	A / H5N6	Europe / Netherlands / Westernieland	Saskia Bergervoet (Wageningen Bioveterinary Research)
EPI_ISL_332440	A/Buzzard/Netherlands/18004242-002/2018	A / H5N6	Europe / Netherlands / Zandfoort	Saskia Bergervoet (Wageningen Bioveterinary Research)
EPI_ISL_332441	A/Mallard/Netherlands/18012508-017/2018	A / H5N6	Europe / Netherlands / Eemmeer	Saskia Bergervoet (Wageningen Bioveterinary Research)
EPI_ISL_332804	A/duck/Bangladesh/19D857/2017	A / H5N6	Asia / Bangladesh	Yunho Jang (Centers for Disease Control and Prevention / WHO Collaborating Center for Surveillance, Epidemiology and Control of Influ
EPI_ISL_332905	A/duck/Bangladesh/19D853/2017	A / H5N6	Asia / Bangladesh	Yunho Jang (Centers for Disease Control and Prevention / WHO Collaborating Center for Surveillance, Epidemiology and Control of Influ
EPI_ISL_332947	A/duck/Bangladesh/19D851/2017	A / H5N6	Asia / Bangladesh	Yunho Jang (Centers for Disease Control and Prevention / WHO Collaborating Center for Surveillance, Epidemiology and Control of Influ
EPI_ISL_332948	A/duck/Bangladesh/19D849/2017	A / H5N6	Asia / Bangladesh	Yunho Jang (Centers for Disease Control and Prevention / WHO Collaborating Center for Surveillance, Epidemiology and Control of Influ
EPI_ISL_333130	A/goose/Bangladesh/19D855/2017	A / H5N6	Asia / Bangladesh	Yunho Jang (Centers for Disease Control and Prevention / WHO Collaborating Center for Surveillance, Epidemiology and Control of Influ
EPI_ISL_333131	A/duck/Bangladesh/19D852/2017	A / H5N6	Asia / Bangladesh	Yunho Jang (Centers for Disease Control and Prevention / WHO Collaborating Center for Surveillance, Epidemiology and Control of Influ
EPI_ISL_333132	A/goose/Bangladesh/19D820/2017	A / H5N6	Asia / Bangladesh	Yunho Jang (Centers for Disease Control and Prevention / WHO Collaborating Center for Surveillance, Epidemiology and Control of Influ
EPI_ISL_333133	A/duck/Bangladesh/19D770/2017	A / H5N6	Asia / Bangladesh	Yunho Jang (Centers for Disease Control and Prevention / WHO Collaborating Center for Surveillance, Epidemiology and Control of Influ
EPI_ISL_333134	A/goose/Bangladesh/19D764/2017	A / H5N6	Asia / Bangladesh	Yunho Jang (Centers for Disease Control and Prevention / WHO Collaborating Center for Surveillance, Epidemiology and Control of Influ
EPI_ISL_333629	A/Environment/Fujiansanyuan/08/2017	A / H5N6	Asia / China / Fujian Province	Xie Jianfeng (Fujian Center for Disease Control and Prevention)
EPI_ISL_333630	A/Environment/Fujian/05326/2016	A / H5N6	Asia / China / Fujian Province	Xie Jianfeng (Fujian Center for Disease Control and Prevention)
EPI_ISL_333631	A/Environment/Fujian/05324/2016	A / H5N6	Asia / China / Fujian Province	Xie Jianfeng (Fujian Center for Disease Control and Prevention)
EPI_ISL_333632	A/Environment/Fujian/09991/2016	A / H5N6	Asia / China / Fujian Province	Xie Jianfeng (Fujian Center for Disease Control and Prevention)
EPI_ISL_333633	A/Environment/Fujian/28681/2016	A / H5N6	Asia / China / Fujian Province	Xie Jianfeng (Fujian Center for Disease Control and Prevention)
EPI_ISL_333634	A/Environment/Fujian/28686/2016	A / H5N6	Asia / China / Fujian Province	Xie Jianfeng (Fujian Center for Disease Control and Prevention)
EPI_ISL_333636	A/Environment/Fujian/52356/2016	A / H5N6	Asia / China / Fujian Province	Xie Jianfeng (Fujian Center for Disease Control and Prevention)
EPI_ISL_333975	A/chicken/Vietnam/NCVD-16A26/2016	A / H5N6	Asia / Vietnam	Yunho Jang (Centers for Disease Control and Prevention / WHO Collaborating Center for Surveillance, Epidemiology and Control of Influ
EPI_ISL_334419	A/chicken/Kagawa/13T/2018	A / H5N6	Asia / Japan	Takehiko Saito (National Institute of Animal Health)
EPI_ISL_334420	A/chicken/Kagawa/11C-2/2018	A / H5N6	Asia / Japan	Takehiko Saito (National Institute of Animal Health)
EPI_ISL_334421	A/chicken/Kagawa/12C-1/2018	A / H5N6	Asia / Japan	Takehiko Saito (National Institute of Animal Health)
EPI_ISL_334422	A/chicken/Kagawa/12T-2/2018	A / H5N6	Asia / Japan	Takehiko Saito (National Institute of Animal Health)
EPI_ISL_334423	A/chicken/Kagawa/14T-2/2018	A / H5N6	Asia / Japan	Takehiko Saito (National Institute of Animal Health)
EPI_ISL_334424	A/chicken/Kagawa/14T-1/2018	A / H5N6	Asia / Japan	Takehiko Saito (National Institute of Animal Health)
EPI_ISL_334425	A/chicken/Kagawa/12T-1/2018	A / H5N6	Asia / Japan	Takehiko Saito (National Institute of Animal Health)
EPI_ISL_334426	A/chicken/Kagawa/15T/2018	A / H5N6	Asia / Japan	Takehiko Saito (National Institute of Animal Health)
EPI_ISL_334427	A/chicken/Kagawa/11T-1/2018	A / H5N6	Asia / Japan	Takehiko Saito (National Institute of Animal Health)
EPI_ISL_334428	A/chicken/Kagawa/11C-1/2018	A / H5N6	Asia / Japan	Takehiko Saito (National Institute of Animal Health)
EPI_ISL_334430	A/chicken/Kagawa/11T-2/2018	A / H5N6	Asia / Japan	Takehiko Saito (National Institute of Animal Health)
EPI_ISL_334431	A/chicken/Kagawa/12C-2/2018	A / H5N6	Asia / Japan	Takehiko Saito (National Institute of Animal Health)
EPI_ISL_335257	A/chicken/Kagawa/2C-2/2018	A / H5N6	Asia / Japan	Takehiko Saito (National Institute of Animal Health)
EPI_ISL_335258	A/chicken/Kagawa/4T-1/2018	A / H5N6	Asia / Japan	Takehiko Saito (National Institute of Animal Health)

EPI_ISL_335259	A/chicken/Kagawa/5C-1/2018	A / H5N6	Asia / Japan	Takehiko Saito (National Institute of Animal Health)
EPI_ISL_335260	A/chicken/Kagawa/4C-1/2018	A / H5N6	Asia / Japan	Takehiko Saito (National Institute of Animal Health)
EPI_ISL_335261	A/chicken/Kagawa/5T-1/2018	A / H5N6	Asia / Japan	Takehiko Saito (National Institute of Animal Health)
EPI_ISL_335262	A/chicken/Kagawa/1T-2/2018	A / H5N6	Asia / Japan	Takehiko Saito (National Institute of Animal Health)
EPI_ISL_335263	A/chicken/Kagawa/2C-1/2018	A / H5N6	Asia / Japan	Takehiko Saito (National Institute of Animal Health)
EPI_ISL_335264	A/Jungle crow/Hyogo/2803E023C/2018	A / H5N6	Asia / Japan	Takehiko Saito (National Institute of Animal Health)
EPI_ISL_335265	A/chicken/Kagawa/2T-1/2018	A / H5N6	Asia / Japan	Takehiko Saito (National Institute of Animal Health)
EPI_ISL_335266	A/chicken/Kagawa/2T-2/2018	A / H5N6	Asia / Japan	Takehiko Saito (National Institute of Animal Health)
EPI_ISL_335268	A/chicken/Kagawa/5C-2/2018	A / H5N6	Asia / Japan	Takehiko Saito (National Institute of Animal Health)
EPI_ISL_335269	A/chicken/Kagawa/3T-2/2018	A / H5N6	Asia / Japan	Takehiko Saito (National Institute of Animal Health)
EPI_ISL_335270	A/chicken/Kagawa/3C-2/2018	A / H5N6	Asia / Japan	Takehiko Saito (National Institute of Animal Health)
EPI_ISL_335271	A/chicken/Kagawa/3T-1/2018	A / H5N6	Asia / Japan	Takehiko Saito (National Institute of Animal Health)
EPI_ISL_335272	A/chicken/Kagawa/3C-1/2018	A / H5N6	Asia / Japan	Takehiko Saito (National Institute of Animal Health)
EPI_ISL_335273	A/Jungle crow/Hyogo/2803E023T/2018	A / H5N6	Asia / Japan	Takehiko Saito (National Institute of Animal Health)
EPI_ISL_335440	A/mandarin_duck/Korea/H119-4/2017	A / H5N6	Asia / Korea, Republic of	Yu-Na Lee (Animal and Plant Quarantine Agency (S-2158) / Avian Influenza Research & Diagnostic Division)
EPI_ISL_335441	A/duck/Korea/H125-1/2018	A / H5N6	Asia / Korea, Republic of	Yu-Na Lee (Animal and Plant Quarantine Agency (S-2158) / Avian Influenza Research & Diagnostic Division)
EPI_ISL_335442	A/duck/Korea/H125-2/2018	A / H5N6	Asia / Korea, Republic of	Yu-Na Lee (Animal and Plant Quarantine Agency (S-2158) / Avian Influenza Research & Diagnostic Division)
EPI_ISL_335443	A/duck/Korea/H182/2018	A / H5N6	Asia / Korea, Republic of	Yu-Na Lee (Animal and Plant Quarantine Agency (S-2158) / Avian Influenza Research & Diagnostic Division)
EPI_ISL_335444	A/duck/Korea/H187/2018	A / H5N6	Asia / Korea, Republic of	Yu-Na Lee (Animal and Plant Quarantine Agency (S-2158) / Avian Influenza Research & Diagnostic Division)
EPI_ISL_335445	A/duck/Korea/H191/2018	A / H5N6	Asia / Korea, Republic of	Yu-Na Lee (Animal and Plant Quarantine Agency (S-2158) / Avian Influenza Research & Diagnostic Division)
EPI_ISL_335446	A/duck/Korea/H192/2018	A / H5N6	Asia / Korea, Republic of	Yu-Na Lee (Animal and Plant Quarantine Agency (S-2158) / Avian Influenza Research & Diagnostic Division)
EPI_ISL_335447	A/duck/Korea/H196/2018	A / H5N6	Asia / Korea, Republic of	Yu-Na Lee (Animal and Plant Quarantine Agency (S-2158) / Avian Influenza Research & Diagnostic Division)
EPI_ISL_335448	A/chicken/Korea/H203/2018	A / H5N6	Asia / Korea, Republic of	Yu-Na Lee (Animal and Plant Quarantine Agency (S-2158) / Avian Influenza Research & Diagnostic Division)
EPI_ISL_335449	A/chicken/Korea/H204/2018	A / H5N6	Asia / Korea, Republic of	Yu-Na Lee (Animal and Plant Quarantine Agency (S-2158) / Avian Influenza Research & Diagnostic Division)
EPI_ISL_335450	A/chicken/Korea/H214/2018	A / H5N6	Asia / Korea, Republic of	Yu-Na Lee (Animal and Plant Quarantine Agency (S-2158) / Avian Influenza Research & Diagnostic Division)
EPI_ISL_335451	A/chicken/Korea/H219/2018	A / H5N6	Asia / Korea, Republic of	Yu-Na Lee (Animal and Plant Quarantine Agency (S-2158) / Avian Influenza Research & Diagnostic Division)
EPI_ISL_335452	A/duck/Korea/H229/2018	A / H5N6	Asia / Korea, Republic of	Yu-Na Lee (Animal and Plant Quarantine Agency (S-2158) / Avian Influenza Research & Diagnostic Division)
EPI_ISL_335453	A/chicken/Korea/H238/2018	A / H5N6	Asia / Korea, Republic of	Yu-Na Lee (Animal and Plant Quarantine Agency (S-2158) / Avian Influenza Research & Diagnostic Division)
EPI_ISL_335454	A/chicken/Korea/H239/2018	A / H5N6	Asia / Korea, Republic of	Yu-Na Lee (Animal and Plant Quarantine Agency (S-2158) / Avian Influenza Research & Diagnostic Division)
EPI_ISL_335455	A/chicken/Korea/H241/2018	A / H5N6	Asia / Korea, Republic of	Yu-Na Lee (Animal and Plant Quarantine Agency (S-2158) / Avian Influenza Research & Diagnostic Division)
EPI_ISL_335456	A/spotbill_duck/Korea/WA159/2018	A / H5N6	Asia / Korea, Republic of	Yu-Na Lee (Animal and Plant Quarantine Agency (S-2158) / Avian Influenza Research & Diagnostic Division)
EPI_ISL_335457	A/mute swan/Shimane/3211A002/2017	A / H5N6	Asia / Japan	Takehiko Saito (National Institute of Animal Health)
EPI_ISL_335458	A/Jungle crow/Hyogo/2803E026T/2018	A / H5N6	Asia / Japan	Takehiko Saito (National Institute of Animal Health)
EPI_ISL_335459	A/Jungle crow/Hyogo/2803E025C/2018	A / H5N6	Asia / Japan	Takehiko Saito (National Institute of Animal Health)
EPI_ISL_335460	A/Jungle crow/Hyogo/2803E028C/2018	A / H5N6	Asia / Japan	Takehiko Saito (National Institute of Animal Health)
EPI_ISL_335461	A/Jungle crow/Hyogo/2803E026C/2018	A / H5N6	Asia / Japan	Takehiko Saito (National Institute of Animal Health)
EPI_ISL_335462	A/Jungle crow/Hyogo/2803E028T/2018	A / H5N6	Asia / Japan	Takehiko Saito (National Institute of Animal Health)
EPI_ISL_335463	A/Northern Goshawk/Tokyo/1301B003/2018	A / H5N6	Asia / Japan	Takehiko Saito (National Institute of Animal Health)
EPI_ISL_335464	A/Jungle crow/Hyogo/2803E027C/2018	A / H5N6	Asia / Japan	Takehiko Saito (National Institute of Animal Health)
EPI_ISL_335465	A/Jungle crow/Hyogo/2803E024T/2018	A / H5N6	Asia / Japan	Takehiko Saito (National Institute of Animal Health)
EPI_ISL_335466	A/Jungle crow/Hyogo/2803E025T/2018	A / H5N6	Asia / Japan	Takehiko Saito (National Institute of Animal Health)
EPI_ISL_335467	A/Jungle crow/Hyogo/2803E027T/2018	A / H5N6	Asia / Japan	Takehiko Saito (National Institute of Animal Health)
EPI_ISL_335468	A/Jungle crow/Hyogo/2803E024C/2018	A / H5N6	Asia / Japan	Takehiko Saito (National Institute of Animal Health)
EPI_ISL_336925	A/common gull/Saratov/1676/2018	A / H5N6	Europe / Russian Federation / Saratov Oblast / Engelssky District	Natalia Goncharova (State Research Center of Virology and Biotechnology (VECTOR) / Emerging Zoonotic Diseases and Influenza)
EPI_ISL_336929	A/chicken/Voronezh/1488/2018	A / H5N8	Europe / Russian Federation / Voronezh Oblast	Alexey Danilenko (State Research Center of Virology and Biotechnology (VECTOR) / Emerging Zoonotic Diseases and Influenza)
EPI_ISL_336930	A/turkey/Rostov-on-Don/1117/2018	A / H5N8	Europe / Russian Federation / Rostov Oblast	Alexey Danilenko (State Research Center of Virology and Biotechnology (VECTOR) / Emerging Zoonotic Diseases and Influenza)
EPI_ISL_336931	A/chiken/Voronezh/1491/2018	A / H5N8	Europe / Russian Federation / Voronezh Oblast	Alexey Danilenko (State Research Center of Virology and Biotechnology (VECTOR) / Emerging Zoonotic Diseases and Influenza)
EPI_ISL_336932	A/chicken/Voronezh/1504/2018	A / H5N8	Europe / Russian Federation / Voronezh Oblast	Alexey Danilenko (State Research Center of Virology and Biotechnology (VECTOR) / Emerging Zoonotic Diseases and Influenza)
EPI_ISL_336933	A/chicken/Voronezh/1513/2018	A / H5N8	Europe / Russian Federation / Voronezh Oblast	Alexey Danilenko (State Research Center of Virology and Biotechnology (VECTOR) / Emerging Zoonotic Diseases and Influenza)
EPI_ISL_337151	A/chicken/Mari El/870/2018	A / H5N8	Europe / Russian Federation / Mari El Republic	Natalia Goncharova (State Research Center of Virology and Biotechnology (VECTOR) / Emerging Zoonotic Diseases and Influenza)
EPI_ISL_337273	A/Guangxi/31906/2018	A / H5N6	Asia / China / Guangxi	Lei Yang (WHO Chinese National Influenza Center / Virology Institute, Chinese CDC)
EPI_ISL_337274	A/Guangdong/18SF020/2018	A / H5N6	Asia / China / Guangdong	Lei Yang (WHO Chinese National Influenza Center / Virology Institute, Chinese CDC)
EPI_ISL_337275	A/Jiangsu/32888/2018	A / H5N6	Asia / China / Jiangsu	Lei Yang (WHO Chinese National Influenza Center / Virology Institute, Chinese CDC)
EPI_ISL_337276	A/Guangxi/32797/2018	A / H5N6	Asia / China / Guangxi	Lei Yang (WHO Chinese National Influenza Center / Virology Institute, Chinese CDC)
EPI_ISL_337280	A/Guangxi/13486/2017	A / H5N6	Asia / China / Guangxi	Lei Yang (WHO Chinese National Influenza Center / Virology Institute, Chinese CDC)
EPI_ISL_337410	A/Turkey/Central Russia/5/2018	A / H5N8	Europe / Russian Federation	Takehiko Saito (National Institute of Animal Health)
EPI_ISL_338359	A/chicken/Central Russia/1/2017	A / H5N2	Europe / Russian Federation	Takehiko Saito (National Institute of Animal Health)
EPI_ISL_339105	A/Duck/France/RG1/2016 (H5N8)	A / H5N8	Europe / France	Thomas Figueroa (Ecole Veterinaire de Toulouse / UMR1225 IHAP Interactions hôtes-agents pathogènes)
EPI_ISL_339932	A/Muscovy duck/Long An/AI470/2018	A / H5N6	Asia / Vietnam	Takehiko Saito (National Institute of Animal Health)
EPI_ISL_340075	A/chicken/Tatarstan/112/2017	A / H5N8	Europe / Russian Federation / Republic of Tatarstan	Svetlana Svyatchenko (State Research Center of Virology and Biotechnology (VECTOR) / Emerging Zoonotic Diseases and Influenza)
EPI_ISL_340453	A/Env/Guangdong/172/2017	A / H5N6	Asia / China / Guangdong Province	Ru Bai (Guangdong Provincial Center for Disease Control and Prevention)
EPI_ISL_340786	A/Env/Guangdong/C17059172/YF/2017-03-06	A / H5N6	Asia / China	Ru Bai (Guangdong Provincial Center for Disease Control and Prevention)
EPI_ISL_340788	A/Env/Guangdong/zhanjiang/C17277346/2017-12-05	A / H5N6	Asia / China	Ru Bai (Guangdong Provincial Center for Disease Control and Prevention)
EPI_ISL_340789	A/Duck/Guangdong/PO17281256/MZH/2017-8-21	A / H5N6	Asia / China	Ru Bai (Guangdong Provincial Center for Disease Control and Prevention)
EPI_ISL_340791	A/Env/Guangdong/C172811415/MZH/2017-10-16	A / H5N6	Asia / China	Ru Bai (Guangdong Provincial Center for Disease Control and Prevention)
EPI_ISL_340792	A/Duck/Guangdong/PO17281388/MZH/2017-10-24	A / H5N6	Asia / China	Ru Bai (Guangdong Provincial Center for Disease Control and Prevention)
EPI_ISL_340793	A/Env/Guangdong/C172811414/MZH/2017-10-16	A / H5N6	Asia / China	Ru Bai (Guangdong Provincial Center for Disease Control and Prevention)
EPI_ISL_340794	A/Env/Guangdong/zhanjiang/C18277209/2018-05-21	A / H5N6	Asia / China	Ru Bai (Guangdong Provincial Center for Disease Control and Prevention)
EPI_ISL_340795	A/Env/Guangdong/Huizhou/C18280077/2018-02-22	A / H5N6	Asia / China	Ru Bai (Guangdong Provincial Center for Disease Control and Prevention)
EPI_ISL_340796	A/Guangdong/18SF020/2018-09-30	A / H5N6	Asia / China	Ru Bai (Guangdong Provincial Center for Disease Control and Prevention)
EPI_ISL_340797	A/Env/Guangdong/Huizhou/C18280031/2018-01-22	A / H5N6	Asia / China	Ru Bai (Guangdong Provincial Center for Disease Control and Prevention)
EPI_ISL_340798	A/Env/Guangdong/zhongshan/C182870234/2018-04-24	A / H5N6	Asia / China	Ru Bai (Guangdong Provincial Center for Disease Control and Prevention)

EPI_ISL_340799	A/Env/Guangdong/Huizhou/C17280804/2017-11-21	A / H5N6	Asia / China	Ru Bai (Guangdong Provincial Center for Disease Control and Prevention)
EPI_ISL_340800	A/Env/Guangdong/zhanjiang/C18277050/2018-01-29	A / H5N6	Asia / China	Ru Bai (Guangdong Provincial Center for Disease Control and Prevention)
EPI_ISL_340805	A/Env/Guangdong/Shenzhen/C18061184/2018-05-08	A / H5N6	Asia / China	Ru Bai (Guangdong Provincial Center for Disease Control and Prevention)
EPI_ISL_340806	A/Env/Guangdong/Qingyuan/C18285099/2018-02-28	A / H5N6	Asia / China	Ru Bai (Guangdong Provincial Center for Disease Control and Prevention)
EPI_ISL_340807	A/Env/Guangdong/Foshan/C182750200/2018-03-06	A / H5N6	Asia / China	Ru Bai (Guangdong Provincial Center for Disease Control and Prevention)
EPI_ISL_340808	A/Env/Guangdong/Foshan/C182753007/2018-01-02	A / H5N6	Asia / China	Ru Bai (Guangdong Provincial Center for Disease Control and Prevention)
EPI_ISL_340809	A/Env/Guangdong/Foshan/C172750608/2017-12-26	A / H5N6	Asia / China	Ru Bai (Guangdong Provincial Center for Disease Control and Prevention)
EPI_ISL_340810	A/Env/Guangdong/Foshan/C172750600/2017-12-26	A / H5N6	Asia / China	Ru Bai (Guangdong Provincial Center for Disease Control and Prevention)
EPI_ISL_340811	A/Env/Guangdong/Foshan/C172750528/2017-12-05	A / H5N6	Asia / China	Ru Bai (Guangdong Provincial Center for Disease Control and Prevention)
EPI_ISL_340812	A/Env/Guangdong/Dongguan/C172863577/2017-12-04	A / H5N6	Asia / China	Ru Bai (Guangdong Provincial Center for Disease Control and Prevention)
EPI_ISL_340813	A/Env/Guangdong/C17272382/SHG/2017-11-14	A / H5N6	Asia / China	Ru Bai (Guangdong Provincial Center for Disease Control and Prevention)
EPI_ISL_340814	A/Env/Guangdong/C172752325/FSH/2017-11-27	A / H5N6	Asia / China	Ru Bai (Guangdong Provincial Center for Disease Control and Prevention)
EPI_ISL_340815	A/Env/Guangdong/C172752326/FSH/2017-11-27	A / H5N6	Asia / China	Ru Bai (Guangdong Provincial Center for Disease Control and Prevention)
EPI_ISL_340816	A/Env/Guangdong/zhongshan/C182870028/2018-01-15	A / H5N6	Asia / China	Ru Bai (Guangdong Provincial Center for Disease Control and Prevention)
EPI_ISL_340817	A/Env/Guangdong/C172752329/FSH/2017-11-27	A / H5N6	Asia / China	Ru Bai (Guangdong Provincial Center for Disease Control and Prevention)
EPI_ISL_340818	A/Env/Guangdong/Foshan/C182750024/2018-01-09	A / H5N6	Asia / China	Ru Bai (Guangdong Provincial Center for Disease Control and Prevention)
EPI_ISL_340819	A/Env/Guangdong/Jieyang/C17289387/2017-12-05	A / H5N6	Asia / China	Ru Bai (Guangdong Provincial Center for Disease Control and Prevention)
EPI_ISL_340820	A/Env/Guangdong/Jieyang/C17289388/2017-12-05	A / H5N6	Asia / China	Ru Bai (Guangdong Provincial Center for Disease Control and Prevention)
EPI_ISL_340821	A/Env/Guangdong/Jieyang/C17289389/2017-12-05	A / H5N6	Asia / China	Ru Bai (Guangdong Provincial Center for Disease Control and Prevention)
EPI_ISL_340822	A/Env/Guangdong/Jieyang/C17289390/2017-12-05	A / H5N6	Asia / China	Ru Bai (Guangdong Provincial Center for Disease Control and Prevention)
EPI_ISL_340823	A/Env/Guangdong/zhanjiang/C18277136/2018-04-02	A / H5N6	Asia / China	Ru Bai (Guangdong Provincial Center for Disease Control and Prevention)
EPI_ISL_340824	A/Env/Guangdong/Foshan/C182750085/2018-01-30	A / H5N6	Asia / China	Ru Bai (Guangdong Provincial Center for Disease Control and Prevention)
EPI_ISL_340825	A/Env/Guangdong/Jieyang/C18289059/2018-02-06	A / H5N6	Asia / China	Ru Bai (Guangdong Provincial Center for Disease Control and Prevention)
EPI_ISL_340826	A/Env/Guangdong/C17059359/YF/2017-05-31	A / H5N6	Asia / China	Ru Bai (Guangdong Provincial Center for Disease Control and Prevention)
EPI_ISL_340827	A/Env/Guangdong/C172790853/ZHQ/2017-10-30	A / H5N6	Asia / China	Ru Bai (Guangdong Provincial Center for Disease Control and Prevention)
EPI_ISL_340829	A/Env/Guangdong/C17285757/QY/2017-11-21	A / H5N6	Asia / China	Ru Bai (Guangdong Provincial Center for Disease Control and Prevention)
EPI_ISL_340830	A/Env/Guangdong/zhanjiang/C17277335/2017-11-27	A / H5N6	Asia / China	Ru Bai (Guangdong Provincial Center for Disease Control and Prevention)
EPI_ISL_340831	A/Env/Guangdong/Shaoguan/C17272470/2018-01-16	A / H5N6	Asia / China	Ru Bai (Guangdong Provincial Center for Disease Control and Prevention)
EPI_ISL_340832	A/Env/Guangdong/zhanjiang/C18277135/2018-04-02	A / H5N6	Asia / China	Ru Bai (Guangdong Provincial Center for Disease Control and Prevention)
EPI_ISL_340833	A/Env/Guangdong/Foshan/C182750020/2018-01-02	A / H5N6	Asia / China	Ru Bai (Guangdong Provincial Center for Disease Control and Prevention)
EPI_ISL_340834	A/Duck/Guangdong/PO1707260017/GZH/2017-08-02	A / H5N6	Asia / China	Ru Bai (Guangdong Provincial Center for Disease Control and Prevention)
EPI_ISL_340835	A/Env/Guangdong/Zhaoqing/C172790995/2017-12-12	A / H5N6	Asia / China	Ru Bai (Guangdong Provincial Center for Disease Control and Prevention)
EPI_ISL_340838	A/Env/Guangdong/C17059521/YF/2017-11-14	A / H5N6	Asia / China	Ru Bai (Guangdong Provincial Center for Disease Control and Prevention)
EPI_ISL_340839	A/Env/Guangdong/EN17275405/FSH/2017-10-18	A / H5N6	Asia / China	Ru Bai (Guangdong Provincial Center for Disease Control and Prevention)
EPI_ISL_340844	A/Env/Guangdong/C17285752/QY/2017-11-21	A / H5N6	Asia / China	Ru Bai (Guangdong Provincial Center for Disease Control and Prevention)
EPI_ISL_340845	A/Env/Guangdong/C17285753/QY/2017-11-21	A / H5N6	Asia / China	Ru Bai (Guangdong Provincial Center for Disease Control and Prevention)
EPI_ISL_340846	A/Env/Guangdong/zhongshan/C172870590/2017-11-28	A / H5N6	Asia / China	Ru Bai (Guangdong Provincial Center for Disease Control and Prevention)
EPI_ISL_340847	A/Duck/Guangdong/Yangjiang/PO18284009/2018-01-09	A / H5N6	Asia / China	Ru Bai (Guangdong Provincial Center for Disease Control and Prevention)
EPI_ISL_340848	A/Env/Guangdong/C172790591/ZHQ/2017-5-8	A / H5N6	Asia / China	Ru Bai (Guangdong Provincial Center for Disease Control and Prevention)
EPI_ISL_340849	A/Env/Guangdong/C172811482/MZH/2017-10-16	A / H5N6	Asia / China	Ru Bai (Guangdong Provincial Center for Disease Control and Prevention)
EPI_ISL_340852	A/Goose/Guangdong/PO1707260055/GZH/2017-08-02	A / H5N6	Asia / China	Ru Bai (Guangdong Provincial Center for Disease Control and Prevention)
EPI_ISL_340853	A/Env/Guangdong/C17272335/SHG/2017-5-16	A / H5N6	Asia / China	Ru Bai (Guangdong Provincial Center for Disease Control and Prevention)
EPI_ISL_340854	A/Env/Guangdong/C17285091/QY/2017-1-17	A / H5N6	Asia / China	Ru Bai (Guangdong Provincial Center for Disease Control and Prevention)
EPI_ISL_340855	A/Waterfowl/Guangdong/PO17284158/YJ/2017-5-10	A / H5N6	Asia / China	Ru Bai (Guangdong Provincial Center for Disease Control and Prevention)
EPI_ISL_340856	A/Env/Guangdong/EN17284259/YJ/2017-7-12	A / H5N6	Asia / China	Ru Bai (Guangdong Provincial Center for Disease Control and Prevention)
EPI_ISL_340857	A/Duck/Guangdong/PO17284260/YJ/2017-7-12	A / H5N6	Asia / China	Ru Bai (Guangdong Provincial Center for Disease Control and Prevention)
EPI_ISL_340858	A/Env/Guangdong/C17280709/HZH/2017-5-2	A / H5N6	Asia / China	Ru Bai (Guangdong Provincial Center for Disease Control and Prevention)
EPI_ISL_341293	A/Hubei/29578/2016 x PR8 (CNIC-HB29578)	A / H5N6	Asia / China / Hubei Province	Lei Yang (WHO Chinese National Influenza Center / Virology Institute, Chinese CDC)
EPI_ISL_341294	A/Fujian-Sanyuan/21099/2017 x PR8 (CNIC-21099)	A / H5N6	Asia / China / Fujian Province	Lei Yang (WHO Chinese National Influenza Center / Virology Institute, Chinese CDC)
EPI_ISL_344526	A/Duck/Egypt/AR517/2017	A / H5N8	Africa / Egypt / Fayoum	Timm Harder (Friedrich-Loeffler-Institut)
EPI_ISL_344527	A/Duck/Egypt/AR518/2017	A / H5N8	Africa / Egypt / Giza	Timm Harder (Friedrich-Loeffler-Institut)
EPI_ISL_344528	A/Chicken/Egypt/AR520/2017	A / H5N8	Africa / Egypt / Qualiobia	Timm Harder (Friedrich-Loeffler-Institut)
EPI_ISL_344531	A/Chicken/Egypt/AR539/2018	A / H5N8	Africa / Egypt / Mansoura	Timm Harder (Friedrich-Loeffler-Institut)
EPI_ISL_344532	A/Chicken/Egypt/AR541/2018	A / H5N8	Africa / Egypt / Giza	Timm Harder (Friedrich-Loeffler-Institut)
EPI_ISL_344533	A/Duck/Egypt/AR542/2018	A / H5N8	Africa / Egypt / Fayoum	Timm Harder (Friedrich-Loeffler-Institut)
EPI_ISL_344534	A/Chicken/Egypt/AR543/2018	A / H5N8	Africa / Egypt / Fayoum	Timm Harder (Friedrich-Loeffler-Institut)
EPI_ISL_344537	A/Chicken/Egypt/AR548/2018	A / H5N8	Africa / Egypt / Qualiobia	Timm Harder (Friedrich-Loeffler-Institut)
EPI_ISL_344538	A/Chicken/Egypt/AR549/2018	A / H5N8	Africa / Egypt / Qualiobia	Timm Harder (Friedrich-Loeffler-Institut)
EPI_ISL_344539	A/Turkey/Egypt/AR550/2018	A / H5N8	Africa / Egypt / Fayoum	Timm Harder (Friedrich-Loeffler-Institut)
EPI_ISL_344540	A/Chicken/Egypt/AR552/2018	A / H5N8	Africa / Egypt / Beni Suef	Timm Harder (Friedrich-Loeffler-Institut)
EPI_ISL_344541	A/Chicken/Egypt/AR553/2018	A / H5N8	Africa / Egypt / Giza	Timm Harder (Friedrich-Loeffler-Institut)
EPI_ISL_344542	A/Chicken/Egypt/AR556/2018	A / H5N8	Africa / Egypt / Qualiobia	Timm Harder (Friedrich-Loeffler-Institut)
EPI_ISL_344543	A/Chicken/Egypt/AR557/2018	A / H5N8	Africa / Egypt / Qualiobia	Timm Harder (Friedrich-Loeffler-Institut)
EPI_ISL_344544	A/Chicken/Egypt/AR558/2018	A / H5N8	Africa / Egypt / Giza	Timm Harder (Friedrich-Loeffler-Institut)
EPI_ISL_344545	A/Chicken/Egypt/AR559/2018	A / H5N8	Africa / Egypt / Fayoum	Timm Harder (Friedrich-Loeffler-Institut)
EPI_ISL_344546	A/Duck/Egypt/AR560/2018	A / H5N8	Africa / Egypt / Giza	Timm Harder (Friedrich-Loeffler-Institut)
EPI_ISL_344547	A/Chicken/Egypt/AR562/2018	A / H5N8	Africa / Egypt / Qualiobia	Timm Harder (Friedrich-Loeffler-Institut)
EPI_ISL_344548	A/Chicken/Egypt/AR591/2018	A / H5N8	Africa / Egypt / Giza	Timm Harder (Friedrich-Loeffler-Institut)
EPI_ISL_346255	A/duck/Egypt/VG1099/2018	A / H5N2	Africa / Egypt / Dakahlia	Mahmoud Mohamed Naguib (Friedrich-Loeffler-Institut)
EPI_ISL_347752	A/Chicken/Yangzhou/f6/2018	A / H5N6	Asia / China / Jiangsu Provice	Xian Qi (Jiangsu Provincial Center for Disease Control & Prevention)
EPI_ISL_347754	A/Chicken/Yangzhou/f9/2018	A / H5N6	Asia / China / Jiangsu Provice	Xian Qi (Jiangsu Provincial Center for Disease Control & Prevention)
EPI_ISL_347757	A/Chicken/Yangzhou/f14/2018	A / H5N6	Asia / China / Jiangsu Provice	Xian Qi (Jiangsu Provincial Center for Disease Control & Prevention)

EPI_ISL_347793	A/Enviroment/Changzhou/768/2017	A / H5N6	Asia / China / Jiangsu Provice	Xian Qi (Jiangsu Provincial Center for Disease Control & Prevention)
EPI_ISL_348279	A/duck/Nigeria/17RS737-43/2016	A / H5N8	Africa / Nigeria / Kano state	Bianca Zecchin (Istituto Zooprofilattico Sperimentale Delle Venezie)
EPI_ISL_348282	A/chicken/Niger/17RS167-19/2017	A / H5N8	Africa / Niger / Niamey	Bianca Zecchin (Istituto Zooprofilattico Sperimentale Delle Venezie)
EPI_ISL_348283	A/goose/Niger/17RS167-22/2017	A / H5N8	Africa / Niger / Niamey	Bianca Zecchin (Istituto Zooprofilattico Sperimentale Delle Venezie)
EPI_ISL_348284	A/chicken/Niger/17RS167-1/2017	A / H5N1	Africa / Niger / Niamey	Bianca Zecchin (Istituto Zooprofilattico Sperimentale Delle Venezie)
EPI_ISL_348285	A/chicken/Niger/17RS167-3/2017	A / H5N1	Africa / Niger / Niamey	Bianca Zecchin (Istituto Zooprofilattico Sperimentale Delle Venezie)
EPI_ISL_351792	A/Chicken/Egypt/AI00994/2019	A / H5N2	Africa / Egypt / Beheira	Timm Harder (Friedrich-Loeffler-Institut)
EPI_ISL_353281	A/Chicken/Suzhou/j1/2019	A / H5N6	Asia / China / Jiangsu Provice	Xian Qi (Jiangsu Provincial Center for Disease Control & Prevention)
EPI_ISL_353285	A/Chicken/Suzhou/j5/2019	A / H5N6	Asia / China / Jiangsu Provice	Xian Qi (Jiangsu Provincial Center for Disease Control & Prevention)
EPI_ISL_353618	A/Chicken/Suzhou/j6/2019	A / H5N6	Asia / China / Jiangsu Provice	Xian Qi (Jiangsu Provincial Center for Disease Control & Prevention)
EPI_ISL_354550	A/Environment/Jiangxi/50653/2016	A / H5N6	Asia / China / Jiangxi	Xiaoxu Zeng (WHO Chinese National Influenza Center / Virology Institute, Chinese CDC)
EPI_ISL_354551	A/Environment/Jiangxi/50652/2016	A / H5N6	Asia / China / Jiangxi	Xiaoxu Zeng (WHO Chinese National Influenza Center / Virology Institute, Chinese CDC)
EPI_ISL_354552	A/Environment/Jiangxi/50624/2016	A / H5N6	Asia / China / Jiangxi	Xiaoxu Zeng (WHO Chinese National Influenza Center / Virology Institute, Chinese CDC)
EPI_ISL_354553	A/Duck/Jiangxi/50455/2016	A / H5N6	Asia / China / Jiangxi	Xiaoxu Zeng (WHO Chinese National Influenza Center / Virology Institute, Chinese CDC)
EPI_ISL_354554	A/Environment/Jiangxi/50452/2016	A / H5N6	Asia / China / Jiangxi	Xiaoxu Zeng (WHO Chinese National Influenza Center / Virology Institute, Chinese CDC)
EPI_ISL_354555	A/Environment/Jiangxi/50449/2016	A / H5N6	Asia / China / Jiangxi	Xiaoxu Zeng (WHO Chinese National Influenza Center / Virology Institute, Chinese CDC)
EPI_ISL_354556	A/Duck/Jiangxi/50446/2016	A / H5N6	Asia / China / Jiangxi	Xiaoxu Zeng (WHO Chinese National Influenza Center / Virology Institute, Chinese CDC)
EPI_ISL_354557	A/Environment/Jiangxi/50445/2016	A / H5N6	Asia / China / Jiangxi	Xiaoxu Zeng (WHO Chinese National Influenza Center / Virology Institute, Chinese CDC)
EPI_ISL_354559	A/Environment/Jiangxi/45191/2016	A / H5N6	Asia / China / Jiangxi	Xiaoxu Zeng (WHO Chinese National Influenza Center / Virology Institute, Chinese CDC)
EPI_ISL_354560	A/Chicken/Jiangxi/45153/2016	A / H5N6	Asia / China / Jiangxi	Xiaoxu Zeng (WHO Chinese National Influenza Center / Virology Institute, Chinese CDC)
EPI_ISL_354561	A/Environment/Jiangxi/45148/2015	A / H5N6	Asia / China / Jiangxi	Xiaoxu Zeng (WHO Chinese National Influenza Center / Virology Institute, Chinese CDC)
EPI_ISL_354562	A/Chicken/Jiangxi/45093/2016	A / H5N6	Asia / China / Jiangxi	Xiaoxu Zeng (WHO Chinese National Influenza Center / Virology Institute, Chinese CDC)
EPI_ISL_354563	A/Environment/Jiangxi/45084/2016	A / H5N6	Asia / China / Jiangxi	Xiaoxu Zeng (WHO Chinese National Influenza Center / Virology Institute, Chinese CDC)
EPI_ISL_354564	A/Environment/Jiangxi/44908/2015	A / H5N6	Asia / China / Jiangxi	Xiaoxu Zeng (WHO Chinese National Influenza Center / Virology Institute, Chinese CDC)
EPI_ISL_354565	A/Environment/Jiangxi/46456/2015	A / H5N6	Asia / China / Jiangxi	Xiaoxu Zeng (WHO Chinese National Influenza Center / Virology Institute, Chinese CDC)
EPI_ISL_354566	A/Environment/Jiangxi/46390/2015	A / H5N6	Asia / China / Jiangxi	Xiaoxu Zeng (WHO Chinese National Influenza Center / Virology Institute, Chinese CDC)
EPI_ISL_354567	A/Environment/Jiangxi/25004/2014	A / H5N6	Asia / China / Jiangxi	Xiaoxu Zeng (WHO Chinese National Influenza Center / Virology Institute, Chinese CDC)
EPI_ISL_354571	A/Environment/Jiangxi/24995/2014	A / H5N6	Asia / China / Jiangxi	Xiaoxu Zeng (WHO Chinese National Influenza Center / Virology Institute, Chinese CDC)
EPI_ISL_362127	A/Grey seal/361-13/BalticPL/16	A / H5N8	Europe / Poland / Pomeranian Voivodeship	Dai-Lun Shin (Stiftung Tierärztliche Hochschule Hannover)
EPI_ISL_365299	A/chicken/South Africa/17090108/2017	A / H5N8	Africa / South Africa	
EPI_ISL_365300	A/muscovy duck/Vietnam/LBM801c132/2015	A / H5N6	Asia / Vietnam	
EPI_ISL_366233	A/Cygnus olor/England/AS00778/2017	A / H5N8	Europe / United Kingdom	
EPI_ISL_366235	A/Cygnus olor/England/AS00868/2017	A / H5N8	Europe / United Kingdom	
EPI_ISL_366236	A/Cygnus olor/England/AS00918/2016	A / H5N8	Europe / United Kingdom	
EPI_ISL_366237	A/Cygnus olor/England/WVUK/2016	A / H5N8	Europe / United Kingdom	
EPI_ISL_366238	A/Cygnus olor/England/WVJX/2016	A / H5N8	Europe / United Kingdom	
EPI_ISL_366239	A/Cygnus olor/England/YBOV/2017	A / H5N8	Europe / United Kingdom	
EPI_ISL_366240	A/Cygnus olor/England/WVZP/2016	A / H5N8	Europe / United Kingdom	
EPI_ISL_366241	A/Cygnus olor/England/WULH/2016	A / H5N8	Europe / United Kingdom	
EPI_ISL_366244	A/Cygnus olor/England/UnringedBirdB/2017	A / H5N8	Europe / United Kingdom	
EPI_ISL_366612	A/Poultry/Iran/clade 2344/2018	A / H5N8	Asia / Iran, Islamic Republic of	
EPI_ISL_366635	A/chicken/Alkharj/910/2018	A / H5N8	Asia / Saudi Arabia	
EPI_ISL_368312	A/chicken/Nha Trang/185/2017	A / H5N6	Asia / Vietnam	
EPI_ISL_368313	A/muscovy duck/Vietnam/LBM941/2017	A / H5N6	Asia / Vietnam	
EPI_ISL_368368	A/China/Original/2018	A / H5N6	Asia / China	
EPI_ISL_368623	A/chicken/Egypt/AB1/2018	A / H5N8	Africa / Egypt	
EPI_ISL_368668	A/duck/Viet Nam/HU1-2361/2014	A / H5N6	Asia / Vietnam	
EPI_ISL_369336	A/chicken/South Africa/Villiers/2017	A / H5N8	Africa / South Africa	
EPI_ISL_369337	A/chicken/South Africa/Standerton/2017	A / H5N8	Africa / South Africa	
EPI_ISL_369338	A/chicken/South Africa/436893/2017	A / H5N8	Africa / South Africa	
EPI_ISL_369339	A/chicken/South Africa/440638A/2017	A / H5N8	Africa / South Africa	
EPI_ISL_369340	A/chicken/South Africa/440638B/2017	A / H5N8	Africa / South Africa	
EPI_ISL_369341	A/chicken/South Africa/441587/2017	A / H5N8	Africa / South Africa	
EPI_ISL_369342	A/chicken/South Africa/MC002/2017	A / H5N8	Africa / South Africa	
EPI_ISL_369343	A/chicken/South Africa/441839/2017	A / H5N8	Africa / South Africa	
EPI_ISL_369344	A/ostrich/South Africa/17080046/2017	A / H5N8	Africa / South Africa	
EPI_ISL_369345	A/chicken/South Africa/443397/2017	A / H5N8	Africa / South Africa	
EPI_ISL_369346	A/chicken/South Africa/17080336/2017	A / H5N8	Africa / South Africa	
EPI_ISL_369347	A/Pekin duck/South Africa/17080340/2017	A / H5N8	Africa / South Africa	
EPI_ISL_369348	A/Pekin duck/South Africa/17080481/2017	A / H5N8	Africa / South Africa	
EPI_ISL_369349	A/Swan/South Africa/17080517/2017	A / H5N8	Africa / South Africa	
EPI_ISL_369350	A/chicken/South Africa/17080561/2017	A / H5N8	Africa / South Africa	
EPI_ISL_369351	A/chicken/South Africa/17080581/2017	A / H5N8	Africa / South Africa	
EPI_ISL_369352	A/chicken/South Africa/17090050/2017	A / H5N8	Africa / South Africa	
EPI_ISL_369353	A/chicken/South Africa/17090100/2017	A / H5N8	Africa / South Africa	
EPI_ISL_369354	A/chicken/South Africa/17090202/2017	A / H5N8	Africa / South Africa	
EPI_ISL_369355	A/chicken/South Africa/448475/2017	A / H5N8	Africa / South Africa	
EPI_ISL_369356	A/chicken/South Africa/17090325/2017	A / H5N8	Africa / South Africa	
EPI_ISL_369357	A/chicken/South Africa/449300/2017	A / H5N8	Africa / South Africa	
EPI_ISL_369358	A/chicken/South Africa/17090348/2017	A / H5N8	Africa / South Africa	
EPI_ISL_369359	A/chicken/South Africa/449418/2017	A / H5N8	Africa / South Africa	
EPI_ISL_369360	A/chicken/South Africa/449443/2017	A / H5N8	Africa / South Africa	

EPI_ISL_369361	A/chicken/South Africa/17090335/2017	A / H5N8	Africa / South Africa	
EPI_ISL_369362	A/turkey/South Africa/450199/2017	A / H5N8	Africa / South Africa	
EPI_ISL_369363	A/chicken/South Africa/115370/2017	A / H5N8	Africa / South Africa	
EPI_ISL_369364	A/chicken/South Africa/450628/2017	A / H5N8	Africa / South Africa	
EPI_ISL_369365	A/chicken/South Africa/451457/2017	A / H5N8	Africa / South Africa	
EPI_ISL_369366	A/ostrich/South Africa/002/2017	A / H5N8	Africa / South Africa	
EPI_ISL_370414	A/Pavo cristatus/China/JS02/2018	A / H5N6	Asia / China	
EPI_ISL_370415	A/Pavo cristatus/China/JS01/2018	A / H5N6	Asia / China	
EPI_ISL_370416	A/Pavo cristatus/China/K10/2018	A / H5N6	Asia / China	
EPI_ISL_372404	A/goose/Guangdong/A-Goose-Guangdong-GS013-2015-HA/20	A / H5N6	Asia / China	
EPI_ISL_372409	A/goose/Guangdong/A-Goose-Guangdong-GS014-2015-HA/20	A / H5N6	Asia / China	
EPI_ISL_372430	A/goose/Guangdong/A-Goose-Guangdong-GS017-2015(H5N6)	A / H5N6	Asia / China	
EPI_ISL_372438	A/goose/Guangdong/A-Goose-Guangdong-GS114-2015(H5N6)	A / H5N6	Asia / China	
EPI_ISL_372446	A/goose/Guangdong/A-Goose-Guangdong-GS116-2015(H5N6)	A / H5N6	Asia / China	
EPI_ISL_372454	A/goose/Guangdong/A-Goose-Guangdong-GS120-2015(H5N6)	A / H5N6	Asia / China	
EPI_ISL_372462	A/goose/Guangdong/A-Goose-Guangdong-GS119-2015(H5N6)	A / H5N6	Asia / China	
EPI_ISL_372467	A/goose/Guangdong/A-Goose-Guangdong-GS144-2015(H5N6)	A / H5N6	Asia / China	
EPI_ISL_372475	A/goose/Guangdong/A-Goose-Guangdong-GS148-2016(H5N6)	A / H5N6	Asia / China	
EPI_ISL_372486	A/goose/Guangdong/A-Goose-Guangdong-GS018-2015(H5N6)	A / H5N6	Asia / China	
EPI_ISL_372491	A/chicken/South Korea/AIV49/2017	A / H5N6	Asia / Korea, Republic of	
EPI_ISL_372791	A/goose/China/GS25-HA.seq/2016	A / H5N6	Asia / China	
EPI_ISL_372799	A/goose/China/GS24-HA.seq/2016	A / H5N6	Asia / China	
EPI_ISL_372806	A/goose/China/GS37-4.seq/2016	A / H5N6	Asia / China	
EPI_ISL_372815	A/goose/China/GS42-4.seq/2016	A / H5N6	Asia / China	
EPI_ISL_372823	A/goose/China/GS45-4.seq/2016	A / H5N6	Asia / China	
EPI_ISL_372831	A/chicken/China/CK46-4.seq/2016	A / H5N6	Asia / China	
EPI_ISL_372840	A/goose/China/GS74-4.seq/2016	A / H5N6	Asia / China	
EPI_ISL_372962	A/mallard/Aichi/2312T010/2016	A / H5N6	Asia / Japan / Aichi	
EPI_ISL_372965	A/mallard/Aichi/2312T011/2016	A / H5N6	Asia / Japan / Aichi	
EPI_ISL_372968	A/wigeon/Aichi/2312T012/2016	A / H5N6	Asia / Japan / Aichi	
EPI_ISL_372971	A/mallard/Aichi/2312T018/2016	A / H5N6	Asia / Japan / Aichi	
EPI_ISL_376189	A/duck/China/waterfowl/2016	A / H5N2	Asia / China	
EPI_ISL_3770721	A/GX-hechi/01/2021	A / H5N6	Asia / China / Guangxi Zhuang Autonomous Region	Min Chen (Guangxi Center for Disease Prevention and Control)
EPI_ISL_3770740	A/GX-guilin/11151/2021	A / H5N6	Asia / China / Guangxi Zhuang Autonomous Region	Min Chen (Guangxi Center for Disease Prevention and Control)
EPI_ISL_378234	A/Domestic goose/Sulaimani/Sul.1/2018	A / H5N8	Asia / Iraq	
EPI_ISL_378252	A/duck/Vietnam/LBM805c113/2015	A / H5N6	Asia / Vietnam	
EPI_ISL_378253	A/duck/Nha Trang/194/2017	A / H5N6	Asia / Vietnam	
EPI_ISL_378254	A/duck/Nha Trang/221/2017	A / H5N6	Asia / Vietnam	
EPI_ISL_378256	A/Mulard Duck/Hungary/59163/2016	A / H5N8	Europe / Hungary	
EPI_ISL_378280	A/cooper's hawk/Washington/1298/2015	A / H5N2	North America / United States / Washington	
EPI_ISL_378311	A/pigeon/South Africa/17080323/2017	A / H5N8	Africa / South Africa	
EPI_ISL_378312	A/Domestic goose/South Africa/17090065/2017	A / H5N8	Africa / South Africa	
EPI_ISL_378362	A/whooper swan/Shanxi/RC01/2016	A / H5N8	Asia / China	
EPI_ISL_378363	A/cackling goose/Aichi/2312T008/2016	A / H5N6	Asia / Japan / Aichi	
EPI_ISL_378364	A/cackling goose/Aichi/2312T017/2016	A / H5N6	Asia / Japan / Aichi	
EPI_ISL_378365	A/cackling goose/Aichi/2312T019/2016	A / H5N6	Asia / Japan / Aichi	
EPI_ISL_378634	A/Whooper Swan/Sanmenxia/01/2016	A / H5N8	Asia / China	
EPI_ISL_379090	A/peafowl/Cameroon/17RS1661-6/2017	A / H5N8	Africa / Cameroon	
EPI_ISL_379227	A/chicken/Uganda/17RS115-15/2017	A / H5N8	Africa / Uganda	
EPI_ISL_379228	A/duck/Uganda/17RS115-9/2017	A / H5N8	Africa / Uganda	
EPI_ISL_379568	A/muscovy duck/Vietnam/HU4-160/2015	A / H5N6	Asia / Vietnam	
EPI_ISL_379682	A/goose/China/1106/2016	A / H5N2	Asia / China	
EPI_ISL_379683	A/duck/China/YD1516/2016	A / H5N2	Asia / China	
EPI_ISL_379744	A/chicken/Egypt/FL6/2018	A / H5N8	Africa / Egypt	
EPI_ISL_379963	A/duck/Vietnam/HU4-879/2015	A / H5N6	Asia / Vietnam	
EPI_ISL_380991	A/Crow/Aghakhan/2017	A / H5N8	Asia / Iran, Islamic Republic of	
EPI_ISL_381741	A/CHICKEN/Egypt/CA35/2017	A / H5N8	Africa / Egypt	
EPI_ISL_381742	A/Duck/Egypt/CAG26/2017	A / H5N8	Africa / Egypt	
EPI_ISL_381743	A/Duck/Egypt/SM57/2017	A / H5N8	Africa / Egypt	
EPI_ISL_381810	A/Egyptian goose/South Africa/001/2017	A / H5N8	Africa / South Africa	
EPI_ISL_381811	A/Speckled pigeon/South Africa/08-004B/2017	A / H5N8	Africa / South Africa	
EPI_ISL_381812	A/Sacred ibis/South Africa/009/2017	A / H5N8	Africa / South Africa	
EPI_ISL_381813	A/Guinea fowl/South Africa/17080243/2017	A / H5N8	Africa / South Africa	
EPI_ISL_381814	A/Guinea fowl/South Africa/17080274/2017	A / H5N8	Africa / South Africa	
EPI_ISL_381815	A/dove/South Africa/17080324/2017	A / H5N8	Africa / South Africa	
EPI_ISL_386922	A/Luscinia cyane/Jiangxi/U2/2016 ?H5N6?	A / H5N6	Asia / China / Jiangxi Province	Zhang Tao (Tsinghua University)
EPI_ISL_386923	A/Streptopelia decaocto/Jiangxi/E1/2015(H5N6)	A / H5N6	Asia / China / Jiangxi Province	Zhang Tao (Tsinghua University)
EPI_ISL_386924	A/Streptopelia decaocto/Jiangxi/J8/2015 ?H5N6?	A / H5N6	Asia / China / Jiangxi Province	Zhang Tao (Tsinghua University)
EPI_ISL_386925	A/quail/Jiangxi/B9/2015 ?H5N6?	A / H5N6	Asia / China / Jiangxi Province	Zhang Tao (Tsinghua University)
EPI_ISL_386926	A/Mallard/Jiangxi/JXH22/2014 ?H5N6?	A / H5N6	Asia / China / Jiangxi Province	Zhang Tao (Tsinghua University)
EPI_ISL_386927	A/Mallard/Jiangxi/JXH9/2014 ?H5N6?	A / H5N6	Asia / China / Jiangxi Province	Zhang Tao (Tsinghua University)
EPI_ISL_386928	A/duck/Jiangxi/E7/2014 ?H5N6?	A / H5N6	Asia / China / Jiangxi Province	Zhang Tao (Tsinghua University)

EPI_ISL_386929	A/chickenl/Jiangxi/E84/2014 ?H5N6?	A / H5N6	Asia / China / Jiangxi Province	Zhang Tao (Tsinghua University)
EPI_ISL_386930	A/duck/Jiangxi/F31/2014?H5N6?	A / H5N6	Asia / China / Jiangxi Province	Zhang Tao (Tsinghua University)
EPI_ISL_387133	A/chicken/Zimbabwe/AI4935/2017	A / H5N8	Africa / Zimbabwe	
EPI_ISL_387135	A/duck/Egypt/N13736E/2017	A / H5N8	Africa / Egypt	
EPI_ISL_387136	A/teal/Egypt/1198C/2017	A / H5N8	Africa / Egypt	
EPI_ISL_387137	A/chicken/Egypt/Q13804A/2017	A / H5N8	Africa / Egypt	
EPI_ISL_387138	A/teal/Egypt /823C/2016	A / H5N8	Africa / Egypt	
EPI_ISL_387139	A/duck/Egypt/F13666A/2017	A / H5N8	Africa / Egypt	
EPI_ISL_387140	A/teal/Egypt/1202C/2017	A / H5N8	Africa / Egypt	
EPI_ISL_387141	A/northern shoveler/Egypt/813C/2016	A / H5N8	Africa / Egypt	
EPI_ISL_387143	A/goose/Spain/IA17CR02699/2017	A / H5N8	Europe / Spain	
EPI_ISL_387351	A/duck/Viet Nam/HN-2431/2015	A / H5N1	Asia / Vietnam	
EPI_ISL_387352	A/duck/Viet Nam/HN-2512/2015	A / H5N6	Asia / Vietnam	
EPI_ISL_387353	A/duck/Viet Nam/HN-2520/2015	A / H5N6	Asia / Vietnam	
EPI_ISL_387354	A/duck/Viet Nam/HN-2522/2015	A / H5N6	Asia / Vietnam	
EPI_ISL_387362	A/duck/Viet Nam/QN-2624/2016	A / H5N6	Asia / Vietnam	
EPI_ISL_388773	A/little grebe/Egypt/1056OP/2016	A / H5N8	Africa / Egypt	
EPI_ISL_389022	A/chicken/Nghe An/01VTC/2018	A / H5N6	Asia / Vietnam / Tinh Nghe An	Natalia Goncharova (State Research Center of Virology and Biotechnology (VECTOR) / Emerging Zoonotic Diseases and Influenza)
EPI_ISL_389023	A/chicken/Nghe An/02VTC/2018	A / H5N6	Asia / Vietnam / Tinh Nghe An	Natalia Goncharova (State Research Center of Virology and Biotechnology (VECTOR) / Emerging Zoonotic Diseases and Influenza)
EPI_ISL_389082	A/chicken/Nghe An/14VTC/2015	A / H5N6	Asia / Vietnam / Tinh Nghe An	Natalia Goncharova (State Research Center of Virology and Biotechnology (VECTOR) / Emerging Zoonotic Diseases and Influenza)
EPI_ISL_389145	A/chicken/Nghe An/27VTC/2018	A / H5N6	Asia / Vietnam / Tinh Nghe An	Natalia Goncharova (State Research Center of Virology and Biotechnology (VECTOR) / Emerging Zoonotic Diseases and Influenza)
EPI_ISL_389290	A/chicken/Ha Tinh/73VTC/2017	A / H5N6	Asia / Vietnam / Tinh Ha Tinh	Natalia Goncharova (State Research Center of Virology and Biotechnology (VECTOR) / Emerging Zoonotic Diseases and Influenza)
EPI_ISL_389657	A/chicken/Vietnam/HU9-842/2018	A / H5N6	Asia / Vietnam	
EPI_ISL_389659	A/chicken/Vietnam/HU9-847/2018	A / H5N6	Asia / Vietnam	
EPI_ISL_389737	A/chicken/Egypt/H13795A/2017	A / H5N8	Africa / Egypt	
EPI_ISL_389738	A/chicken/Egypt/H13791C/2017	A / H5N8	Africa / Egypt	
EPI_ISL_389739	A/chicken/Egypt/N13726/2017	A / H5N8	Africa / Egypt	
EPI_ISL_389740	A/chicken/ Egypt/N13732A/2017	A / H5N8	Africa / Egypt	
EPI_ISL_389743	A/chicken/Egypt/H13793E/2017	A / H5N8	Africa / Egypt	
EPI_ISL_389746	A/duck/Egypt/N13733B/2017	A / H5N8	Africa / Egypt	
EPI_ISL_389747	A/chicken/Egypt/N13717E/2017	A / H5N8	Africa / Egypt	
EPI_ISL_389748	A/chicken/Egypt/A13775D/2017	A / H5N8	Africa / Egypt	
EPI_ISL_389750	A/duck/Egypt/N13734B/2017	A / H5N8	Africa / Egypt	
EPI_ISL_389751	A/chicken/Egypt/M14081D/2017	A / H5N8	Africa / Egypt	
EPI_ISL_389752	A/chicken/Egypt/M13844B/2017	A / H5N8	Africa / Egypt	
EPI_ISL_389753	A/duck/Egypt/H13797E/2017	A / H5N8	Africa / Egypt	
EPI_ISL_389754	A/duck/Egypt/N13736B/2017	A / H5N8	Africa / Egypt	
EPI_ISL_389755	A/chicken/Egypt/Q13944C/2017	A / H5N8	Africa / Egypt	
EPI_ISL_389756	A/chicken/Egypt/Q13941B/2017	A / H5N8	Africa / Egypt	
EPI_ISL_389757	A/duck/Egypt/N14200B/2017	A / H5N8	Africa / Egypt	
EPI_ISL_389759	A/chicken/Egypt/F13660A/2017	A / H5N8	Africa / Egypt	
EPI_ISL_389760	A/chicken/Egypt/F13830A/2017	A / H5N8	Africa / Egypt	
EPI_ISL_389761	A/chicken/Egypt/N13720A/2017	A / H5N8	Africa / Egypt	
EPI_ISL_389762	A/chicken/Egypt/Q13845B/2017	A / H5N8	Africa / Egypt	
EPI_ISL_389765	A/chicken/Egypt/F13664A/2017	A / H5N8	Africa / Egypt	
EPI_ISL_389766	A/chicken/Egypt/A13777B/2017	A / H5N8	Africa / Egypt	
EPI_ISL_389767	A/chicken/Egypt/H13794E/2017	A / H5N8	Africa / Egypt	
EPI_ISL_389768	A/chicken/Egypt/H13800C/2017	A / H5N8	Africa / Egypt	
EPI_ISL_389769	A/chicken/Egypt/H13796A/2017	A / H5N8	Africa / Egypt	
EPI_ISL_389770	A/chicken/Egypt/F13829A/2017	A / H5N8	Africa / Egypt	
EPI_ISL_389771	A/chicken/Egypt/A13776B/2017	A / H5N8	Africa / Egypt	
EPI_ISL_389772	A/chicken/Egypt/H13799B/2017	A / H5N8	Africa / Egypt	
EPI_ISL_389773	A/chicken/Egypt/Q13936B/2017	A / H5N8	Africa / Egypt	
EPI_ISL_389774	A/goose/Egypt/A13779B/2017	A / H5N8	Africa / Egypt	
EPI_ISL_389776	A/duck/Egypt/N13735A/2017	A / H5N8	Africa / Egypt	
EPI_ISL_389791	A/chicken/Bangladesh/1896-17-HA/2017	A / H5N6	Asia / Bangladesh	
EPI_ISL_393518	A/Avian/Iran/38AMA/2019	A / H5N3	Asia / Iran, Islamic Republic of	
EPI_ISL_3950049	A/Mallard/Sweden/SVA210827SZ0252/KN002973/D-2021	A / H5N8	Europe / Sweden / Sodermanlands Lan / Nykopings Kommun	Siamak Zohari (National Veterinary Institute)
EPI_ISL_3950066	A/Mallard/Sweden/SVA210827SZ0252/KN002974/D-2021	A / H5N8	Europe / Sweden / Sodermanlands Lan / Nykopings Kommun	Siamak Zohari (National Veterinary Institute)
EPI_ISL_397346	A/duck/Tottori/E9/2016	A / H5N6	Asia / Japan / Tottori	
EPI_ISL_397347	A/duck/Tottori/E10/2016	A / H5N6	Asia / Japan / Tottori	
EPI_ISL_397348	A/duck/Tottori/E13/2016	A / H5N6	Asia / Japan / Tottori	
EPI_ISL_398050	A/chicken-layer/Egypt/MR18/2018	A / H5	Africa / Egypt	
EPI_ISL_399051	A/duck/Egypt/F13663C/2017	A / H5N8	Africa / Egypt	
EPI_ISL_399055	A/duck/Egypt/F13667A/2017	A / H5N8	Africa / Egypt	
EPI_ISL_399989	A/Duck/Egypt/AI20251/2019	A / H5N8	Africa / Egypt	Timm Harder (Friedrich-Loeffler-Institut)
EPI_ISL_399990	A/Duck/Egypt/AI20252/2019	A / H5N8	Africa / Egypt	Timm Harder (Friedrich-Loeffler-Institut)
EPI_ISL_399991	A/Duck/Egypt/AI20259/2019	A / H5	Africa / Egypt	Timm Harder (Friedrich-Loeffler-Institut)
EPI_ISL_399993	A/Duck/Egypt/AI20263/2019	A / H5N8	Africa / Egypt	Timm Harder (Friedrich-Loeffler-Institut)
EPI_ISL_399994	A/Duck/Egypt/AI20266/2019	A / H5N8	Africa / Egypt	Timm Harder (Friedrich-Loeffler-Institut)
EPI_ISL_399995	A/Chicken/Egypt/AI20268/2019	A / H5N8	Africa / Egypt	Timm Harder (Friedrich-Loeffler-Institut)
EPI_ISL_399996	A/Chicken/Egypt/AI20284/2019	A / H5N8	Africa / Egypt	Timm Harder (Friedrich-Loeffler-Institut)

EPI_ISL_400027	A/Turkey/Egypt/AI20285/2019	A / H5N8	Africa / Egypt	Timm Harder (Friedrich-Loeffler-Institut)
EPI_ISL_400043	A/Chicken/Egypt/AI20286/2019	A / H5N8	Africa / Egypt	Timm Harder (Friedrich-Loeffler-Institut)
EPI_ISL_400485	A/Green pheasant/Hunan/10/2015	A / H5N6	Asia / China / Hunan	Yanbing Li (Harbin Veterinary Research Institute (CAAS) / Ministry of Agriculture)
EPI_ISL_400486	A/Common pheasant/Hunan/11/2015	A / H5N6	Asia / China / Hunan	Yanbing Li (Harbin Veterinary Research Institute (CAAS) / Ministry of Agriculture)
EPI_ISL_400487	A/Common pheasant/Hunan/2/2016	A / H5N6	Asia / China / Hunan	Yanbing Li (Harbin Veterinary Research Institute (CAAS) / Ministry of Agriculture)
EPI_ISL_400488	A/Silver Pheasant/Hunan/3/2016	A / H5N6	Asia / China / Hunan	Yanbing Li (Harbin Veterinary Research Institute (CAAS) / Ministry of Agriculture)
EPI_ISL_400489	A/Whooper Swan/Hunan/4/2016	A / H5N6	Asia / China / Hunan	Yanbing Li (Harbin Veterinary Research Institute (CAAS) / Ministry of Agriculture)
EPI_ISL_400490	A/Whooper swan/Shanxi/6/2016	A / H5N8	Asia / China / Shanxi	Yanbing Li (Harbin Veterinary Research Institute (CAAS) / Ministry of Agriculture)
EPI_ISL_400491	A/Whooper swan/Shanxi/7/2016	A / H5N8	Asia / China / Shanxi	Yanbing Li (Harbin Veterinary Research Institute (CAAS) / Ministry of Agriculture)
EPI_ISL_400492	A/Greylag goose/Hunan/1/2017	A / H5N6	Asia / China / Hunan	Yanbing Li (Harbin Veterinary Research Institute (CAAS) / Ministry of Agriculture)
EPI_ISL_400493	A/Black swan/Hubei/2/2017	A / H5N8	Asia / China / Hubei	Yanbing Li (Harbin Veterinary Research Institute (CAAS) / Ministry of Agriculture)
EPI_ISL_400494	A/Black swan/Hubei/3/2017	A / H5N8	Asia / China / Hubei	Yanbing Li (Harbin Veterinary Research Institute (CAAS) / Ministry of Agriculture)
EPI_ISL_400548	A/Peacock/Hunan/12/2015	A / H5N6	Asia / China / Hunan	Yanbing Li (Harbin Veterinary Research Institute (CAAS) / Ministry of Agriculture)
EPI_ISL_400549	A/Peacock/Hunan/13/2015	A / H5N6	Asia / China / Hunan	Yanbing Li (Harbin Veterinary Research Institute (CAAS) / Ministry of Agriculture)
EPI_ISL_400550	A/Peacock/Hunan/15/2015	A / H5N6	Asia / China / Hunan	Yanbing Li (Harbin Veterinary Research Institute (CAAS) / Ministry of Agriculture)
EPI_ISL_400551	A/Peacock/Hunan/14/2015	A / H5N2	Asia / China / Hunan	Yanbing Li (Harbin Veterinary Research Institute (CAAS) / Ministry of Agriculture)
EPI_ISL_400552	A/Peacock/Hunan/1/2016	A / H5N6	Asia / China / Hunan	Yanbing Li (Harbin Veterinary Research Institute (CAAS) / Ministry of Agriculture)
EPI_ISL_402134	A/turkey/Poland/23/2019	A / H5N8	Europe / Poland / Lublin Voivodeship	Edyta ?wi?to? (National Veterinary Research Institut Poland, PIWet-PIB)
EPI_ISL_4031377	A/Duck/China/B1/2018	A / H5N6	Asia / China	
EPI_ISL_4031378	A/Duck/China/B2/2018	A / H5N6	Asia / China	
EPI_ISL_4031379	A/Duck/China/B3/2019	A / H5N6	Asia / China	
EPI_ISL_4031380	A/Duck/China/B4/2019	A / H5N6	Asia / China	
EPI_ISL_4031381	A/Duck/China/B5/2019	A / H5N6	Asia / China	
EPI_ISL_4031382	A/Duck/China/B6/2019	A / H5N6	Asia / China	
EPI_ISL_4031383	A/Duck/China/B7/2019	A / H5N6	Asia / China	
EPI_ISL_4031384	A/Duck/China/B8/2019	A / H5N6	Asia / China	
EPI_ISL_4031385	A/Duck/China/B9/2019	A / H5N6	Asia / China	
EPI_ISL_4031450	A/Chicken/Viet Nam/AI-1606/2016	A / H5N6	Asia / Vietnam	
EPI_ISL_4031458	A/mallard/Denmark/12106-1/2018	A / H5N6	Europe / Denmark	
EPI_ISL_4031459	A/mallard/Denmark/12106-2/2018	A / H5N6	Europe / Denmark	
EPI_ISL_4031461	A/black-headed gull/Denmark/4663-1/2018	A / H5N6	Europe / Denmark	
EPI_ISL_4031467	A/pheasant/Denmark/12106-03/2018	A / H5N6	Europe / Denmark	
EPI_ISL_4032096	A/duck/Nantou/15A03445-11-20T/2015	A / H5N8	Asia / Taiwan	
EPI_ISL_4032097	A/chicken/Changhua/15010120-1/2015	A / H5N2	Asia / Taiwan	
EPI_ISL_4032098	A/chicken/Changhua/15010288/2015	A / H5N2	Asia / Taiwan	
EPI_ISL_4032099	A/chicken/Changhua/15010324-2/2015	A / H5N2	Asia / Taiwan	
EPI_ISL_4032104	A/chicken/Changhua/15010361-1/2015	A / H5N2	Asia / Taiwan	
EPI_ISL_4032148	A/chicken/Changhua/15010362-2/2015	A / H5N2	Asia / Taiwan	
EPI_ISL_4032190	A/chicken/Changhua/15010363-2/2015	A / H5N2	Asia / Taiwan	
EPI_ISL_4032194	A/chicken/Changhua/15040036-1/2015	A / H5N8	Asia / Taiwan	
EPI_ISL_4032195	A/chicken/Changhua/15040043-1/2015	A / H5N8	Asia / Taiwan	
EPI_ISL_4032196	A/chicken/Changhua/15040037-2/2015	A / H5N8	Asia / Taiwan	
EPI_ISL_4032197	A/chicken/Changhua/15040042/2015	A / H5N8	Asia / Taiwan	
EPI_ISL_4032198	A/chicken/Changhua/15050013-1/2015	A / H5N2	Asia / Taiwan	
EPI_ISL_4032199	A/chicken/Changhua/15050038-1/2015	A / H5N8	Asia / Taiwan	
EPI_ISL_4032200	A/chicken/Changhua/15060027-2/2015	A / H5N2	Asia / Taiwan	
EPI_ISL_4032201	A/chicken/Changhua/15060029/2015	A / H5N8	Asia / Taiwan	
EPI_ISL_4032202	A/chicken/Changhua/15060037-1/2015	A / H5N2	Asia / Taiwan	
EPI_ISL_4032203	A/chicken/Changhua/15120008/2015	A / H5N2	Asia / Taiwan	
EPI_ISL_4032204	A/chicken/Changhua/16010060-2/2016	A / H5N2	Asia / Taiwan	
EPI_ISL_4032205	A/chicken/Changhua/16020042-2/2016	A / H5N2	Asia / Taiwan	
EPI_ISL_4032206	A/chicken/Changhua/17030063-1/2017	A / H5N2	Asia / Taiwan	
EPI_ISL_4032207	A/chicken/Changhua/17060006/2017	A / H5N2	Asia / Taiwan	
EPI_ISL_4032208	A/chicken/Changhua/18010011-1/2018	A / H5N2	Asia / Taiwan	
EPI_ISL_4032212	A/chicken/Changhua/18040008-1/2018	A / H5N2	Asia / Taiwan	
EPI_ISL_4032213	A/chicken/Chiayi/15010287-2/2015	A / H5N2	Asia / Taiwan	
EPI_ISL_4032214	A/chicken/Chiayi/15010409-2/2015	A / H5N2	Asia / Taiwan	
EPI_ISL_4032215	A/chicken/Chiayi/15010454-1/2015	A / H5N8	Asia / Taiwan	
EPI_ISL_4032216	A/chicken/Chiayi/17030059-2/2017	A / H5N2	Asia / Taiwan	
EPI_ISL_4032217	A/chicken/Chiayi/17040009-2/2017	A / H5N2	Asia / Taiwan	
EPI_ISL_4032218	A/chicken/Chiayi/18120003-1/2018	A / H5N2	Asia / Taiwan	
EPI_ISL_4032219	A/chicken/Hsinchu/16080004-2/2016	A / H5N2	Asia / Taiwan	
EPI_ISL_4032220	A/chicken/Kaohsiung/16010047/2016	A / H5N8	Asia / Taiwan	
EPI_ISL_4032221	A/chicken/Kaohsiung/16020017-2/2016	A / H5N8	Asia / Taiwan	
EPI_ISL_4032222	A/chicken/Kaohsiung/16040021-1/2016	A / H5N8	Asia / Taiwan	
EPI_ISL_4032223	A/chicken/Kaohsiung/16070003/2016	A / H5N8	Asia / Taiwan	
EPI_ISL_4032224	A/chicken/Kaohsiung/16120012/2016	A / H5N8	Asia / Taiwan	
EPI_ISL_4032225	A/chicken/Kaohsiung/16120034/2016	A / H5N8	Asia / Taiwan	
EPI_ISL_4032226	A/chicken/Kaohsiung/16MB0889/2016	A / H5N8	Asia / Taiwan	
EPI_ISL_4032227	A/chicken/Kaohsiung/17030023/2017	A / H5N8	Asia / Taiwan	
EPI_ISL_4032228	A/chicken/Kaohsiung/17040006-2/2017	A / H5N2	Asia / Taiwan	
EPI_ISL_4032229	A/chicken/Kaohsiung/17040007/2017	A / H5N2	Asia / Taiwan	

EPI_ISL_4032230	A/chicken/Kaohsiung/17100002/2017	A / H5N2	Asia / Taiwan
EPI_ISL_4032235	A/chicken/Kaohsiung/18080004-1/2018	A / H5N2	Asia / Taiwan
EPI_ISL_4032236	A/chicken/Kaohsiung/18080004-2/2018	A / H5N2	Asia / Taiwan
EPI_ISL_4032237	A/chicken/Miaoli/15010421-1/2015	A / H5N8	Asia / Taiwan
EPI_ISL_4032238	A/chicken/Miaoli/18040011-1/2018	A / H5N2	Asia / Taiwan
EPI_ISL_4032239	A/chicken/Nantou/16110023-1/2016	A / H5N8	Asia / Taiwan
EPI_ISL_4032240	A/chicken/New Taipei City/16110019-1/2016	A / H5N8	Asia / Taiwan
EPI_ISL_4032241	A/chicken/New Taipei City/16110020-2/2016	A / H5N8	Asia / Taiwan
EPI_ISL_4032242	A/chicken/New Taipei City/17040002-2/2017	A / H5N2	Asia / Taiwan
EPI_ISL_4032243	A/chicken/New Taipei City/17050004-2/2017	A / H5N8	Asia / Taiwan
EPI_ISL_4032244	A/chicken/New Taipei City/17090001/2017	A / H5N8	Asia / Taiwan
EPI_ISL_4032245	A/chicken/New Taipei City/17090002/2017	A / H5N8	Asia / Taiwan
EPI_ISL_4032246	A/chicken/New Taipei City/17090003/2017	A / H5N8	Asia / Taiwan
EPI_ISL_4032247	A/chicken/New Taipei City/18010021-3/2018	A / H5N2	Asia / Taiwan
EPI_ISL_4032248	A/chicken/Pingtung/15010402/2015	A / H5N2	Asia / Taiwan
EPI_ISL_4032264	A/chicken/Pingtung/15050006-1/2015	A / H5N2	Asia / Taiwan
EPI_ISL_4032266	A/chicken/Pingtung/16020009-3/2016	A / H5N2	Asia / Taiwan
EPI_ISL_4032267	A/chicken/Pingtung/18010017-1/2018	A / H5N2	Asia / Taiwan
EPI_ISL_4032268	A/chicken/Pingtung/18010019-2/2018	A / H5N2	Asia / Taiwan
EPI_ISL_4032269	A/chicken/Pingtung/18020015-1/2018	A / H5N2	Asia / Taiwan
EPI_ISL_4032270	A/chicken/Pingtung/18050006-1/2018	A / H5N2	Asia / Taiwan
EPI_ISL_4032271	A/chicken/Pingtung/18080001-1/2018	A / H5N2	Asia / Taiwan
EPI_ISL_4032272	A/chicken/Taichung/15010399/2015	A / H5N2	Asia / Taiwan
EPI_ISL_4032273	A/chicken/Taichung/16040032-3/2016	A / H5N2	Asia / Taiwan
EPI_ISL_4032274	A/chicken/Taichung/16050044-2/2016	A / H5N2	Asia / Taiwan
EPI_ISL_4032275	A/chicken/Taichung/16110017-3/2016	A / H5N8	Asia / Taiwan
EPI_ISL_4032276	A/chicken/Tainan/15020214/2015	A / H5N8	Asia / Taiwan
EPI_ISL_4032277	A/chicken/Tainan/16040035-1/2016	A / H5N2	Asia / Taiwan
EPI_ISL_4032278	A/chicken/Tainan/17030058-1/2017	A / H5N8	Asia / Taiwan
EPI_ISL_4032279	A/chicken/Tainan/18040014-2/2018	A / H5N2	Asia / Taiwan
EPI_ISL_4032280	A/chicken/Taipei City/17090004/2017	A / H5N8	Asia / Taiwan
EPI_ISL_4032281	A/chicken/Taipei City/18010007-3/2018	A / H5N2	Asia / Taiwan
EPI_ISL_4032282	A/chicken/Taipei City/18010008-3/2018	A / H5N2	Asia / Taiwan
EPI_ISL_4032283	A/chicken/Taipei City/18050007-1/2018	A / H5N2	Asia / Taiwan
EPI_ISL_4032284	A/chicken/Taipei City/18050010-8/2018	A / H5N2	Asia / Taiwan
EPI_ISL_4032285	A/chicken/Taipei/16060022-4/2016	A / H5N8	Asia / Taiwan
EPI_ISL_4032286	A/chicken/Taipei/16060023-1/2016	A / H5N8	Asia / Taiwan
EPI_ISL_4032287	A/chicken/Taipei/16120021/2016	A / H5N8	Asia / Taiwan
EPI_ISL_4032288	A/chicken/Taipei/17050005-1/2017	A / H5N8	Asia / Taiwan
EPI_ISL_4032289	A/chicken/Taipei/17050006-2/2017	A / H5N8	Asia / Taiwan
EPI_ISL_4032290	A/chicken/Taipei/17050007-2/2017	A / H5N8	Asia / Taiwan
EPI_ISL_4032291	A/chicken/Taipei/17050008-3/2017	A / H5N8	Asia / Taiwan
EPI_ISL_4032292	A/chicken/Taipei/17050009-3/2017	A / H5N8	Asia / Taiwan
EPI_ISL_4032293	A/chicken/Yunlin/15020048-1/2015	A / H5N2	Asia / Taiwan
EPI_ISL_4032294	A/chicken/Yunlin/15040002-2/2015	A / H5N2	Asia / Taiwan
EPI_ISL_4032295	A/chicken/Yunlin/15040027/2015	A / H5N2	Asia / Taiwan
EPI_ISL_4032296	A/chicken/Yunlin/15050012/2015	A / H5N8	Asia / Taiwan
EPI_ISL_4032297	A/chicken/Yunlin/15050037-2/2015	A / H5N2	Asia / Taiwan
EPI_ISL_4032298	A/chicken/Yunlin/15050039-2/2015	A / H5N2	Asia / Taiwan
EPI_ISL_4032299	A/chicken/Yunlin/15060014-1/2015	A / H5N2	Asia / Taiwan
EPI_ISL_4032300	A/chicken/Yunlin/15080024/2015	A / H5N2	Asia / Taiwan
EPI_ISL_4032301	A/chicken/Yunlin/16010001-1/2016	A / H5N2	Asia / Taiwan
EPI_ISL_4032302	A/chicken/Yunlin/16010008-1/2016	A / H5N2	Asia / Taiwan
EPI_ISL_4032303	A/chicken/Yunlin/16020038/2016	A / H5N8	Asia / Taiwan
EPI_ISL_4032304	A/chicken/Yunlin/16050053/2016	A / H5N2	Asia / Taiwan
EPI_ISL_4032305	A/chicken/Yunlin/17010001/2017	A / H5N8	Asia / Taiwan
EPI_ISL_4032306	A/chicken/Yunlin/17010014/2017	A / H5N2	Asia / Taiwan
EPI_ISL_4032307	A/chicken/Yunlin/17020033-2/2017	A / H5N2	Asia / Taiwan
EPI_ISL_4032308	A/chicken/Yunlin/17020094-1/2017	A / H5N2	Asia / Taiwan
EPI_ISL_4032309	A/chicken/Yunlin/17020095-2/2017	A / H5N2	Asia / Taiwan
EPI_ISL_4032310	A/chicken/Yunlin/17030001-1/2017	A / H5N2	Asia / Taiwan
EPI_ISL_4032311	A/chicken/Yunlin/17030007-1/2017	A / H5N2	Asia / Taiwan
EPI_ISL_4032312	A/chicken/Yunlin/17030010-1/2017	A / H5N2	Asia / Taiwan
EPI_ISL_4032313	A/chicken/Yunlin/17030014-1/2017	A / H5N2	Asia / Taiwan
EPI_ISL_4032314	A/chicken/Yunlin/17030020-2/2017	A / H5N2	Asia / Taiwan
EPI_ISL_4032315	A/chicken/Yunlin/17030021-1/2017	A / H5N2	Asia / Taiwan
EPI_ISL_4032316	A/chicken/Yunlin/17030032-2/2017	A / H5N2	Asia / Taiwan
EPI_ISL_4032317	A/chicken/Yunlin/17030065-2/2017	A / H5N2	Asia / Taiwan
EPI_ISL_4032318	A/chicken/Yunlin/17040003-1/2017	A / H5N2	Asia / Taiwan
EPI_ISL_4032319	A/chicken/Yunlin/17040004-2/2017	A / H5N2	Asia / Taiwan
EPI_ISL_4032320	A/chicken/Yunlin/17040005-1/2017	A / H5N2	Asia / Taiwan
EPI_ISL_4032321	A/chicken/Yunlin/17040010-1/2017	A / H5N2	Asia / Taiwan

EPI_ISL_4032322	A/chicken/Yunlin/17040011-1/2017	A / H5N2	Asia / Taiwan
EPI_ISL_4032323	A/chicken/Yunlin/17040012-1/2017	A / H5N2	Asia / Taiwan
EPI_ISL_4032324	A/chicken/Yunlin/17060019/2017	A / H5N2	Asia / Taiwan
EPI_ISL_4032325	A/chicken/Yunlin/17070010/2017	A / H5N2	Asia / Taiwan
EPI_ISL_4032326	A/chicken/Yunlin/18010001-2/2018	A / H5N2	Asia / Taiwan
EPI_ISL_4032327	A/chicken/Yunlin/18010003-3/2018	A / H5N2	Asia / Taiwan
EPI_ISL_4032328	A/chicken/Yunlin/18010023-1/2018	A / H5N2	Asia / Taiwan
EPI_ISL_4032329	A/chicken/Yunlin/18010024-2/2018	A / H5N2	Asia / Taiwan
EPI_ISL_4032330	A/chicken/Yunlin/18020003-2/2018	A / H5N2	Asia / Taiwan
EPI_ISL_4032331	A/chicken/Yunlin/18020004-1/2018	A / H5N2	Asia / Taiwan
EPI_ISL_4032332	A/chicken/Yunlin/18020005-2/2018	A / H5N2	Asia / Taiwan
EPI_ISL_4032333	A/chicken/Yunlin/18020008-2/2018	A / H5N2	Asia / Taiwan
EPI_ISL_4032334	A/chicken/Yunlin/18020021-1/2018	A / H5N2	Asia / Taiwan
EPI_ISL_4032335	A/chicken/Yunlin/18030002-1/2018	A / H5N2	Asia / Taiwan
EPI_ISL_4032336	A/chicken/Yunlin/18040006-3/2018	A / H5N2	Asia / Taiwan
EPI_ISL_4032337	A/chicken/Yunlin/18050013-1/2018	A / H5N2	Asia / Taiwan
EPI_ISL_4032338	A/chicken/Yunlin/18060001-2/2018	A / H5N2	Asia / Taiwan
EPI_ISL_4032379	A/chicken/Yunlin/18070003-3/2018	A / H5N2	Asia / Taiwan
EPI_ISL_4032419	A/chicken/Yunlin/18080006-1/2018	A / H5N2	Asia / Taiwan
EPI_ISL_4032420	A/duck/Changhua/15A03431-1-20T/2015	A / H5N2	Asia / Taiwan
EPI_ISL_4032421	A/duck/Changhua/15A03473-1-20T/2015	A / H5N2	Asia / Taiwan
EPI_ISL_4032422	A/duck/Chiayi/15080023/2015	A / H5N8	Asia / Taiwan
EPI_ISL_4032423	A/duck/Hsinchu/15020216/2015	A / H5N2	Asia / Taiwan
EPI_ISL_4032424	A/duck/Kaohsiung/15010213/2015	A / H5N2	Asia / Taiwan
EPI_ISL_4032425	A/duck/Nantou/15A3437/2015	A / H5N2	Asia / Taiwan
EPI_ISL_4032426	A/duck/Nantou/17A0243/2017	A / H5N2	Asia / Taiwan
EPI_ISL_4032427	A/duck/Pingtung/15010145/2015	A / H5N2	Asia / Taiwan
EPI_ISL_4032428	A/duck/Pingtung/15010157-1/2015	A / H5N3	Asia / Taiwan
EPI_ISL_4032429	A/duck/Pingtung/15010180/2015	A / H5N3	Asia / Taiwan
EPI_ISL_4032430	A/duck/Pingtung/15010444/2015	A / H5N2	Asia / Taiwan
EPI_ISL_4032431	A/duck/Pingtung/15010461-1/2015	A / H5N2	Asia / Taiwan
EPI_ISL_4032432	A/duck/Pingtung/15120006/2015	A / H5N8	Asia / Taiwan
EPI_ISL_4032433	A/duck/Pingtung/15A03399-1-10T/2015	A / H5N2	Asia / Taiwan
EPI_ISL_4032434	A/duck/Pingtung/17A00408-1-10T/2017	A / H5N2	Asia / Taiwan
EPI_ISL_4032435	A/duck/Pingtung/17A0160/2017	A / H5N2	Asia / Taiwan
EPI_ISL_4032436	A/duck/Pingtung/18A00003-11-20T/2018	A / H5N2	Asia / Taiwan
EPI_ISL_4032437	A/duck/Pingtung/18A00076-11-20T/2018	A / H5N2	Asia / Taiwan
EPI_ISL_4032438	A/duck/Pingtung/18X00077/2018	A / H5N2	Asia / Taiwan
EPI_ISL_4032439	A/duck/Taichung/15010400-1/2015	A / H5N2	Asia / Taiwan
EPI_ISL_4032440	A/duck/Tainan/15010403/2015	A / H5N8	Asia / Taiwan
EPI_ISL_4032441	A/duck/Tainan/16010020-1/2016	A / H5N2	Asia / Taiwan
EPI_ISL_4032442	A/duck/Tainan/16010024-1/2016	A / H5N2	Asia / Taiwan
EPI_ISL_4032443	A/duck/Taoyuan/16010023-1/2016	A / H5N2	Asia / Taiwan
EPI_ISL_4032444	A/duck/Yilan/17A0092/2017	A / H5N2	Asia / Taiwan
EPI_ISL_4032445	A/duck/Yunlin/15010115-2/2015	A / H5N2	Asia / Taiwan
EPI_ISL_4032446	A/duck/Yunlin/15010244-1/2015	A / H5N2	Asia / Taiwan
EPI_ISL_4032447	A/duck/Yunlin/15010277/2015	A / H5N8	Asia / Taiwan
EPI_ISL_4032448	A/duck/Yunlin/15010310-4/2015	A / H5N2	Asia / Taiwan
EPI_ISL_4032449	A/duck/Yunlin/15010318-2/2015	A / H5N2	Asia / Taiwan
EPI_ISL_4032468	A/duck/Yunlin/15010369-1/2015	A / H5N2	Asia / Taiwan
EPI_ISL_4032522	A/duck/Yunlin/15010458-1/2015	A / H5N8	Asia / Taiwan
EPI_ISL_4032531	A/duck/Yunlin/15010538/2015	A / H5N8	Asia / Taiwan
EPI_ISL_4032532	A/duck/Yunlin/15070002-2/2015	A / H5N2	Asia / Taiwan
EPI_ISL_4032533	A/duck/Yunlin/15A03403-1-20T/2015	A / H5N2	Asia / Taiwan
EPI_ISL_4032534	A/duck/Yunlin/15A04883-1-10T/2015	A / H5N2	Asia / Taiwan
EPI_ISL_4032535	A/duck/Yunlin/15A5019/2015	A / H5N2	Asia / Taiwan
EPI_ISL_4032536	A/duck/Yunlin/17110001/2017	A / H5N2	Asia / Taiwan
EPI_ISL_4032537	A/duck/Yunlin/17A0086/2017	A / H5N2	Asia / Taiwan
EPI_ISL_4032538	A/duck/Yunlin/17A0686/2017	A / H5N2	Asia / Taiwan
EPI_ISL_4032539	A/duck/Yunlin/18010004-2/2018	A / H5N2	Asia / Taiwan
EPI_ISL_4032540	A/duck/Yunlin/18060006-1/2018	A / H5N2	Asia / Taiwan
EPI_ISL_4032541	A/duck/Yunlin/18060007-3/2018	A / H5N2	Asia / Taiwan
EPI_ISL_4032542	A/duck/Yunlin/18X00007-3/2018	A / H5N2	Asia / Taiwan
EPI_ISL_4032543	A/environment/Yunlin/18X00007/2018	A / H5N2	Asia / Taiwan
EPI_ISL_4032544	A/environment/Yunlin/18X00070/2018	A / H5N2	Asia / Taiwan
EPI_ISL_4032545	A/goose/Changhua/15040008/2015	A / H5N2	Asia / Taiwan
EPI_ISL_4032546	A/goose/Chiayi/15010008-3/2015	A / H5N2	Asia / Taiwan
EPI_ISL_4032547	A/goose/Chiayi/15010016-1/2015	A / H5N2	Asia / Taiwan
EPI_ISL_4032548	A/goose/Chiayi/15020022/2015	A / H5N8	Asia / Taiwan
EPI_ISL_4032549	A/goose/Chiayi/16010017-2/2016	A / H5N2	Asia / Taiwan
EPI_ISL_4032550	A/goose/Chiayi/16040003-1/2016	A / H5N2	Asia / Taiwan
EPI_ISL_4032551	A/goose/Chiayi/16040005/2016	A / H5N2	Asia / Taiwan

EPI_ISL_4032552	A/goose/Chiayi/16040007-1/2016	A / H5N2	Asia / Taiwan	
EPI_ISL_4032553	A/goose/Chiayi/17020027-1/2017	A / H5N2	Asia / Taiwan	
EPI_ISL_4032554	A/goose/Chiayi/17040014/2017	A / H5N2	Asia / Taiwan	
EPI_ISL_4032555	A/goose/Chiayi/18010006/2018	A / H5N2	Asia / Taiwan	
EPI_ISL_4032556	A/goose/Chiayi/18020002-2/2018	A / H5N2	Asia / Taiwan	
EPI_ISL_4032558	A/goose/Chiayi/18020014-2/2018	A / H5N2	Asia / Taiwan	
EPI_ISL_4032559	A/goose/Chiayi/18020014-3/2018	A / H5N2	Asia / Taiwan	
EPI_ISL_4032560	A/goose/Pingtung/15010007-4/2015	A / H5N2	Asia / Taiwan	
EPI_ISL_4032561	A/goose/Pingtung/15010037-1/2015	A / H5N2	Asia / Taiwan	
EPI_ISL_4032562	A/goose/Pingtung/15050014/2015	A / H5N8	Asia / Taiwan	
EPI_ISL_4032563	A/goose/Pingtung/16010061/2016	A / H5N8	Asia / Taiwan	
EPI_ISL_4032564	A/goose/Tainan/16010031-2/2016	A / H5N2	Asia / Taiwan	
EPI_ISL_4032565	A/goose/Tainan/16020041-2/2016	A / H5N2	Asia / Taiwan	
EPI_ISL_4032566	A/goose/Tainan/16040018-1/2016	A / H5N8	Asia / Taiwan	
EPI_ISL_4032567	A/goose/Tainan/16070002/2016	A / H5N8	Asia / Taiwan	
EPI_ISL_4032568	A/goose/Tainan/17030022-1/2017	A / H5N2	Asia / Taiwan	
EPI_ISL_4032569	A/goose/Tainan/17030024-1/2017	A / H5N2	Asia / Taiwan	
EPI_ISL_4032570	A/goose/Tainan/18010022-1/2018	A / H5N2	Asia / Taiwan	
EPI_ISL_4032571	A/goose/Tainan/18050014-1/2018	A / H5N2	Asia / Taiwan	
EPI_ISL_4032572	A/goose/Taoyuan/15010041/2015	A / H5N8	Asia / Taiwan	
EPI_ISL_4032573	A/goose/Taoyuan/18120002-2/2018	A / H5N2	Asia / Taiwan	
EPI_ISL_4032574	A/goose/Yunlin/15010005/2015	A / H5N2	Asia / Taiwan	
EPI_ISL_4032575	A/goose/Yunlin/15010009-1/2015	A / H5N2	Asia / Taiwan	
EPI_ISL_4032576	A/goose/Yunlin/15010010-2/2015	A / H5N2	Asia / Taiwan	
EPI_ISL_4032577	A/goose/Yunlin/15010011-1/2015	A / H5N2	Asia / Taiwan	
EPI_ISL_4032578	A/goose/Yunlin/15010029-1/2015	A / H5N2	Asia / Taiwan	
EPI_ISL_404207	A/duck/Nghe An/5382VTC/2019	A / H5N6	Asia / Vietnam / Tinh Ha Tinh	Natalia Goncharova (State Research Center of Virology and Biotechnology (VECTOR) / Emerging Zoonotic Diseases and Influenza)
EPI_ISL_404986	A/chicken/Ba Ria-Vung Tau/21078VTC/2019	A / H5N6	Asia / Vietnam / Tinh Ba Ria-Vung Tau	Natalia Goncharova (State Research Center of Virology and Biotechnology (VECTOR) / Emerging Zoonotic Diseases and Influenza)
EPI_ISL_404987	A/chicken/Dong Nai/25437VTC/2019	A / H5N6	Asia / Vietnam / Tinh Dong Nai	Natalia Goncharova (State Research Center of Virology and Biotechnology (VECTOR) / Emerging Zoonotic Diseases and Influenza)
EPI_ISL_404988	A/chicken/Thanh Hoa/13836VTC/2019	A / H5N6	Asia / Vietnam / Tinh Thanh Hoa	Natalia Goncharova (State Research Center of Virology and Biotechnology (VECTOR) / Emerging Zoonotic Diseases and Influenza)
EPI_ISL_404993	A/white-fronted goose/Germany-BB/AI00018/2020	A / H5N8	Europe / Germany / Brandenburg / Spree/Neiße	Jacqueline King (Friedrich-Loeffler-Institut)
EPI_ISL_405278	A/guinea_fowl/Nigeria/OG-GF11T_19VIR8424-7/2019_H5N8	A / H5N8	Africa / Nigeria / Ogun State	Bianca Zecchin (Istituto Zooprofilattico Sperimentale Delle Venezie)
EPI_ISL_405391	A/chicken/Czech Republic/1175-1/2020	A / H5N8	Europe / Czech Republic / Vysocina Kraj / Okres Zd'ar nad Szavou / Št?pánov nad Svratkou	Alexander Nagy (State Veterinary Institute Prague)
EPI_ISL_4055115	A/goose/Yunlin/15040040/2015	A / H5N8	Asia / Taiwan	
EPI_ISL_4055116	A/goose/Yunlin/16020008-4/2016	A / H5N2	Asia / Taiwan	
EPI_ISL_4055117	A/goose/Yunlin/16020011-1/2016	A / H5N2	Asia / Taiwan	
EPI_ISL_4055118	A/goose/Yunlin/16030042-2/2016	A / H5N2	Asia / Taiwan	
EPI_ISL_4055119	A/goose/Yunlin/16040002-2/2016	A / H5N2	Asia / Taiwan	
EPI_ISL_4055120	A/goose/Yunlin/17100005/2017	A / H5N2	Asia / Taiwan	
EPI_ISL_4055121	A/goose/Yunlin/18030020-1/2018	A / H5N2	Asia / Taiwan	
EPI_ISL_4055122	A/goose/Yunlin/18030020-2/2018	A / H5N2	Asia / Taiwan	
EPI_ISL_4055123	A/goose/Yunlin/18030020-3/2018	A / H5N2	Asia / Taiwan	
EPI_ISL_4055124	A/goose/Yunlin/18040002-3/2018	A / H5N2	Asia / Taiwan	
EPI_ISL_4055125	A/goose/Yunlin/18040018-2/2018	A / H5N2	Asia / Taiwan	
EPI_ISL_4055126	A/goose/Yunlin/18050003-2/2018	A / H5N2	Asia / Taiwan	
EPI_ISL_4055127	A/goose/Yunlin/18090002-3/2018	A / H5N2	Asia / Taiwan	
EPI_ISL_4055128	A/goose/Yunlin/18090006-1/2018	A / H5N2	Asia / Taiwan	
EPI_ISL_4055129	A/goose/Yunlin/18110002-3/2018	A / H5N2	Asia / Taiwan	
EPI_ISL_4055132	A/turkey/Changhua/16010011-1/2016	A / H5N2	Asia / Taiwan	
EPI_ISL_4055133	A/turkey/Chiayi/17020086-2/2017	A / H5N8	Asia / Taiwan	
EPI_ISL_4055135	A/turkey/Chiayi/17020092-2/2017	A / H5N8	Asia / Taiwan	
EPI_ISL_4055136	A/turkey/Chiayi/18010013-3/2018	A / H5N2	Asia / Taiwan	
EPI_ISL_4055137	A/turkey/Yunlin/15030069-1/2015	A / H5N2	Asia / Taiwan	
EPI_ISL_4055138	A/turkey/Yunlin/15060049-1/2015	A / H5N2	Asia / Taiwan	
EPI_ISL_4055139	A/turkey/Yunlin/18020020-2/2018	A / H5N2	Asia / Taiwan	
EPI_ISL_4055140	A/turkey/Yunlin/18070001-2/2018	A / H5N2	Asia / Taiwan	
EPI_ISL_4055141	A/turkey/Yunlin/18090003-3/2018	A / H5N2	Asia / Taiwan	
EPI_ISL_4055142	A/turkey/Yunlin/18110001-3/2018	A / H5N2	Asia / Taiwan	
EPI_ISL_4055143	A/turkey/Yunlin/18120005-2/2018	A / H5N2	Asia / Taiwan	
EPI_ISL_4055845	A/duck/Ugii lake/#66/2018	A / H5N6	Asia / Mongolia	
EPI_ISL_4055846	A/duck/Khunt lake/#500/2019	A / H5N6	Asia / Mongolia	
EPI_ISL_405813	A/hawk/Poland/003/2020	A / H5N8	Europe / Poland / Lublin Voivodeship	Edyta ?wi?to? (National Veterinary Research Institut Poland, PIWet-PIB)
EPI_ISL_4061481	A/chicken/Nigeria/VRD21-43_21VIR2288-4/2021	A / H5N8	Africa / Nigeria	
EPI_ISL_4061482	A/chicken/Nigeria/VRD21-035B_21VIR2288-1/2021	A / H5N8	Africa / Nigeria	
EPI_ISL_4061483	A/chicken/Nigeria/VRD21-100_21VIR2370-423/2021	A / H5N8	Africa / Nigeria	
EPI_ISL_4061484	A/chicken/Nigeria/VRD21-102_21VIR2370-424/2021	A / H5N1	Africa / Nigeria	
EPI_ISL_4061485	A/chicken/Nigeria/VRD21-109_21VIR2370-425/2021	A / H5N1	Africa / Nigeria	
EPI_ISL_4061486	A/chicken/Nigeria/VRD21-37_21VIR2288-2/2021	A / H5N1	Africa / Nigeria	
EPI_ISL_4061488	A/chicken/Nigeria/VRD21-53B_21VIR2288-5/2021	A / H5N8	Africa / Nigeria	
EPI_ISL_4061490	A/chicken/Nigeria/VRD21-88_21VIR2288-8/2021	A / H5N8	Africa / Nigeria	
EPI_ISL_4061491	A/chicken/Nigeria/VRD21-98_21VIR2288-6/2021	A / H5N1	Africa / Nigeria	
EPI_ISL_4061541	A/chicken/Netherlands/2113/2017	A / H5N8	Europe / Netherlands	

EPI_ISL_4061544	A/Quail/Viet Nam/4615/2015	A / H5N6	Asia / Vietnam
EPI_ISL_4061567	A/chicken/Viet Nam/6115/2015	A / H5N6	Asia / Vietnam
EPI_ISL_4061568	A/Duck/Viet Nam/5314/2014	A / H5N6	Asia / Vietnam
EPI_ISL_4061712	A/whooper swan/Miyagi/0402B001/2021	A / H5N8	Asia / Japan / Miyagi
EPI_ISL_4061713	A/whooper swan/Fukushima/0701B002/2021	A / H5N8	Asia / Japan / Fukushima
EPI_ISL_4062109	A/chicken/HeBei/HB1905/2019	A / H5N6	Asia / China
EPI_ISL_4062111	A/chicken/HeBei/HB1907/2019	A / H5N6	Asia / China
EPI_ISL_4062113	A/chicken/Pakistan/531/2018	A / H5N8	Asia / Pakistan
EPI_ISL_4062114	A/quail/Pakistan/998/2018	A / H5N8	Asia / Pakistan
EPI_ISL_4062115	A/chicken/Pakistan/1000/2018	A / H5N8	Asia / Pakistan
EPI_ISL_4062116	A/environment/Pakistan/927/2018	A / H5N8	Asia / Pakistan
EPI_ISL_4062127	A/chicken/HeBei/CK05/2019	A / H5N6	Asia / China
EPI_ISL_4062128	A/goose/HeBei/GD07/2019	A / H5N6	Asia / China
EPI_ISL_4062437	A/goose/China/21FU001/2020	A / H5N8	Asia / China
EPI_ISL_4062438	A/goose/China/21FU002/2020	A / H5N8	Asia / China
EPI_ISL_4062439	A/goose/China/21FU003/2020	A / H5N8	Asia / China
EPI_ISL_4062440	A/goose/China/21FU004/2020	A / H5N8	Asia / China
EPI_ISL_4062441	A/goose/China/21FU005/2020	A / H5N8	Asia / China
EPI_ISL_4062442	A/goose/China/21FU006/2020	A / H5N8	Asia / China
EPI_ISL_4062443	A/goose/China/21FU007/2020	A / H5N8	Asia / China
EPI_ISL_4062444	A/goose/China/21FU008/2020	A / H5N8	Asia / China
EPI_ISL_4063606	A/chicken/Northern China/F0130c/2018	A / H5N6	Asia / China
EPI_ISL_4064431	A/duck/France/161113/2016	A / H5N8	Europe / France
EPI_ISL_4064432	A/duck/France/161116/2016	A / H5N8	Europe / France
EPI_ISL_4064433	A/duck/France/161137/2016	A / H5N8	Europe / France
EPI_ISL_4064434	A/duck/France/161141/2016	A / H5N8	Europe / France
EPI_ISL_4064435	A/duck/France/161142/2016	A / H5N8	Europe / France
EPI_ISL_4064436	A/duck/France/161143/2016	A / H5N8	Europe / France
EPI_ISL_4064437	A/duck/France/161147/2016	A / H5N8	Europe / France
EPI_ISL_4064438	A/duck/France/161173/2016	A / H5N8	Europe / France
EPI_ISL_4064439	A/duck/France/161174/2016	A / H5N8	Europe / France
EPI_ISL_4064440	A/duck/France/161182/2016	A / H5N8	Europe / France
EPI_ISL_4064441	A/duck/France/161207/2016	A / H5N8	Europe / France
EPI_ISL_4064442	A/duck/France/161212/2016	A / H5N8	Europe / France
EPI_ISL_4064443	A/duck/France/161224/2016	A / H5N8	Europe / France
EPI_ISL_4064444	A/duck/France/161225/2016	A / H5N8	Europe / France
EPI_ISL_4064445	A/duck/France/161227/2016	A / H5N8	Europe / France
EPI_ISL_4064446	A/duck/France/161228/2016	A / H5N8	Europe / France
EPI_ISL_4064447	A/duck/France/161229/2016	A / H5N8	Europe / France
EPI_ISL_4064448	A/duck/France/161230/2016	A / H5N8	Europe / France
EPI_ISL_4064449	A/duck/France/161231/2016	A / H5N8	Europe / France
EPI_ISL_4064450	A/duck/France/161233/2016	A / H5N8	Europe / France
EPI_ISL_4064451	A/duck/France/161234/2016	A / H5N8	Europe / France
EPI_ISL_4064452	A/duck/France/161239/2016	A / H5N8	Europe / France
EPI_ISL_4064453	A/duck/France/161240/2016	A / H5N8	Europe / France
EPI_ISL_4064454	A/duck/France/161242/2016	A / H5N8	Europe / France
EPI_ISL_4064455	A/duck/France/161243/2016	A / H5N8	Europe / France
EPI_ISL_4064456	A/duck/France/161256/2016	A / H5N8	Europe / France
EPI_ISL_4064457	A/duck/France/161263/2016	A / H5N8	Europe / France
EPI_ISL_4064458	A/duck/France/161271/2016	A / H5N8	Europe / France
EPI_ISL_4064459	A/duck/France/161275/2016	A / H5N8	Europe / France
EPI_ISL_4064460	A/duck/France/161277/2016	A / H5N8	Europe / France
EPI_ISL_4064461	A/duck/France/161298/2016	A / H5N8	Europe / France
EPI_ISL_4064462	A/duck/France/161383/2016	A / H5N8	Europe / France
EPI_ISL_4064463	A/duck/France/161443/2016	A / H5N8	Europe / France
EPI_ISL_4064464	A/duck/France/161444/2016	A / H5N8	Europe / France
EPI_ISL_4064465	A/duck/France/161445/2016	A / H5N8	Europe / France
EPI_ISL_4064466	A/duck/France/161449/2016	A / H5N8	Europe / France
EPI_ISL_4064467	A/duck/France/161450/2016	A / H5N8	Europe / France
EPI_ISL_4064468	A/duck/France/161455/2016	A / H5N8	Europe / France
EPI_ISL_4064469	A/duck/France/161456/2016	A / H5N8	Europe / France
EPI_ISL_4064470	A/duck/France/161457/2016	A / H5N8	Europe / France
EPI_ISL_4064471	A/duck/France/161473/2016	A / H5N8	Europe / France
EPI_ISL_4064472	A/duck/France/161475/2016	A / H5N8	Europe / France
EPI_ISL_4064473	A/duck/France/161476/2016	A / H5N8	Europe / France
EPI_ISL_4064474	A/duck/France/161477/2016	A / H5N8	Europe / France
EPI_ISL_4064475	A/duck/France/161478/2016	A / H5N8	Europe / France
EPI_ISL_4064476	A/duck/France/161497/2016	A / H5N8	Europe / France
EPI_ISL_4064477	A/duck/France/161498/2016	A / H5N8	Europe / France
EPI_ISL_4064478	A/duck/France/161500/2016	A / H5N8	Europe / France
EPI_ISL_4064479	A/duck/France/161501/2016	A / H5N8	Europe / France
EPI_ISL_4064480	A/duck/France/161577/2016	A / H5N8	Europe / France

EPI_ISL_4064482	A/duck/France/161600/2016	A / H5N8	Europe / France
EPI_ISL_4064483	A/duck/France/161601/2016	A / H5N8	Europe / France
EPI_ISL_4064484	A/duck/France/170013/2016	A / H5N8	Europe / France
EPI_ISL_4064485	A/duck/France/170016/2016	A / H5N8	Europe / France
EPI_ISL_4064486	A/duck/France/170017/2016	A / H5N8	Europe / France
EPI_ISL_4064487	A/duck/France/170018/2017	A / H5N8	Europe / France
EPI_ISL_4064488	A/duck/France/170030/2016	A / H5N8	Europe / France
EPI_ISL_4064489	A/duck/France/170031/2017	A / H5N8	Europe / France
EPI_ISL_4064490	A/duck/France/170032/2017	A / H5N8	Europe / France
EPI_ISL_4064491	A/duck/France/170033/2016	A / H5N8	Europe / France
EPI_ISL_4064492	A/duck/France/170034/2017	A / H5N8	Europe / France
EPI_ISL_4064493	A/duck/France/170036/2017	A / H5N8	Europe / France
EPI_ISL_4064494	A/duck/France/170038/2017	A / H5N8	Europe / France
EPI_ISL_4064495	A/chicken/France/170063/2017	A / H5N8	Europe / France
EPI_ISL_4064496	A/duck/France/170064/2017	A / H5N8	Europe / France
EPI_ISL_4064497	A/duck/France/170067/2017	A / H5N8	Europe / France
EPI_ISL_4064498	A/duck/France/170098/2017	A / H5N8	Europe / France
EPI_ISL_4064499	A/duck/France/170117/2017	A / H5N8	Europe / France
EPI_ISL_4064500	A/duck/France/170118/2017	A / H5N8	Europe / France
EPI_ISL_4064501	A/chicken/France/170176/2017	A / H5N8	Europe / France
EPI_ISL_4064502	A/duck/France/170177/2017	A / H5N8	Europe / France
EPI_ISL_4064503	A/duck/France/170178/2017	A / H5N8	Europe / France
EPI_ISL_4064504	A/duck/France/170180/2017	A / H5N8	Europe / France
EPI_ISL_4064505	A/duck/France/170245/2017	A / H5N8	Europe / France
EPI_ISL_4064506	A/duck/France/170249/2017	A / H5N8	Europe / France
EPI_ISL_4064507	A/duck/France/170250/2017	A / H5N8	Europe / France
EPI_ISL_4064508	A/turkey/France/170278/2017	A / H5N8	Europe / France
EPI_ISL_4064509	A/duck/France/170280/2017	A / H5N8	Europe / France
EPI_ISL_4064510	A/duck/France/170284/2017	A / H5N8	Europe / France
EPI_ISL_4064511	A/duck/France/170308/2017	A / H5N8	Europe / France
EPI_ISL_4064512	A/duck/France/170309/2017	A / H5N8	Europe / France
EPI_ISL_4064513	A/duck/France/170325/2017	A / H5N8	Europe / France
EPI_ISL_4064514	A/duck/France/170330/2017	A / H5N8	Europe / France
EPI_ISL_4064515	A/duck/France/170331/2017	A / H5N8	Europe / France
EPI_ISL_4064516	A/duck/France/170338/2017	A / H5N8	Europe / France
EPI_ISL_4064517	A/duck/France/170339/2017	A / H5N8	Europe / France
EPI_ISL_4064518	A/chicken/France/170340/2017	A / H5N8	Europe / France
EPI_ISL_4064519	A/duck/France/170341/2017	A / H5N8	Europe / France
EPI_ISL_4064520	A/duck/France/170349/2017	A / H5N8	Europe / France
EPI_ISL_4064521	A/duck/France/170368/2017	A / H5N8	Europe / France
EPI_ISL_4064522	A/duck/France/170369/2017	A / H5N8	Europe / France
EPI_ISL_4064523	A/duck/France/170370/2017	A / H5N8	Europe / France
EPI_ISL_4064525	A/chicken/France/170407/2017	A / H5N8	Europe / France
EPI_ISL_4064526	A/chicken/France/170408/2017	A / H5N8	Europe / France
EPI_ISL_4064527	A/guineafowl/France/170409/2017	A / H5N8	Europe / France
EPI_ISL_4064535	A/duck/France/170432/2017	A / H5N8	Europe / France
EPI_ISL_4064541	A/chicken/France/170433/2017	A / H5N8	Europe / France
EPI_ISL_4064548	A/chicken/France/170436/2017	A / H5N8	Europe / France
EPI_ISL_4064555	A/duck/France/170437/2017	A / H5N8	Europe / France
EPI_ISL_4064569	A/duck/France/170518/2017	A / H5N8	Europe / France
EPI_ISL_4064575	A/duck/France/170612/2017	A / H5N8	Europe / France
EPI_ISL_4064582	A/duck/France/170683/2017	A / H5N8	Europe / France
EPI_ISL_4064589	A/duck/France/170685/2017	A / H5N8	Europe / France
EPI_ISL_4064597	A/chicken/France/170686/2017	A / H5N8	Europe / France
EPI_ISL_4064603	A/duck/France/170733/2017	A / H5N8	Europe / France
EPI_ISL_4064610	A/duck/France/170734/2017	A / H5N8	Europe / France
EPI_ISL_4064617	A/duck/France/170735/2017	A / H5N8	Europe / France
EPI_ISL_4064619	A/duck/France/170738/2017	A / H5N8	Europe / France
EPI_ISL_4064621	A/duck/France/170772/2017	A / H5N8	Europe / France
EPI_ISL_4064622	A/chicken/France/170773/2017	A / H5N8	Europe / France
EPI_ISL_4064623	A/chicken/France/170774/2017	A / H5N8	Europe / France
EPI_ISL_4064624	A/duck/France/170775/2017	A / H5N8	Europe / France
EPI_ISL_4064625	A/duck/France/170806/2017	A / H5N8	Europe / France
EPI_ISL_4064626	A/duck/France/170820/2017	A / H5N8	Europe / France
EPI_ISL_4064627	A/duck/France/170822/2017	A / H5N8	Europe / France
EPI_ISL_4064628	A/duck/France/170974/2017	A / H5N8	Europe / France
EPI_ISL_4064629	A/duck/France/170975/2017	A / H5N8	Europe / France
EPI_ISL_4064630	A/goose/France/170976/2017	A / H5N8	Europe / France
EPI_ISL_4064631	A/avian/France/170977/2017	A / H5N8	Europe / France
EPI_ISL_4064632	A/duck/France/171131/2017	A / H5N8	Europe / France
EPI_ISL_4064633	A/duck/France/171133/2017	A / H5N8	Europe / France
EPI_ISL_4064634	A/duck/France/171134/2017	A / H5N8	Europe / France

EPI_ISL_4064635	A/duck/France/171202/2017	A / H5N8	Europe / France
EPI_ISL_4064636	A/duck/France/171253/2017	A / H5N8	Europe / France
EPI_ISL_4064637	A/duck/France/171270/2017	A / H5N8	Europe / France
EPI_ISL_4064638	A/duck/France/171275/2017	A / H5N8	Europe / France
EPI_ISL_4064639	A/goose/France/171278/2017	A / H5N8	Europe / France
EPI_ISL_4064640	A/duck/France/171378/2017	A / H5N8	Europe / France
EPI_ISL_4064641	A/duck/France/171379/2017	A / H5N8	Europe / France
EPI_ISL_4064642	A/duck/France/171408/2017	A / H5N8	Europe / France
EPI_ISL_4064643	A/duck/France/171410/2017	A / H5N8	Europe / France
EPI_ISL_4064645	A/duck/France/171455/2017	A / H5N8	Europe / France
EPI_ISL_4064646	A/duck/France/171457/2017	A / H5N8	Europe / France
EPI_ISL_4064647	A/duck/France/171461/2017	A / H5N8	Europe / France
EPI_ISL_4064648	A/duck/France/171462/2017	A / H5N8	Europe / France
EPI_ISL_4064649	A/duck/France/171463/2017	A / H5N8	Europe / France
EPI_ISL_4064650	A/duck/France/171466/2017	A / H5N8	Europe / France
EPI_ISL_4064651	A/duck/France/171467/2017	A / H5N8	Europe / France
EPI_ISL_4064652	A/duck/France/171468/2017	A / H5N8	Europe / France
EPI_ISL_4064653	A/duck/France/171469/2017	A / H5N8	Europe / France
EPI_ISL_4064654	A/chicken/France/171471/2017	A / H5N8	Europe / France
EPI_ISL_4064655	A/duck/France/171472/2017	A / H5N8	Europe / France
EPI_ISL_4064656	A/duck/France/171486/2017	A / H5N8	Europe / France
EPI_ISL_4064657	A/duck/France/171514/2017	A / H5N8	Europe / France
EPI_ISL_4064658	A/chicken/France/171525/2017	A / H5N8	Europe / France
EPI_ISL_4064659	A/duck/France/171533/2017	A / H5N8	Europe / France
EPI_ISL_4064660	A/chicken/France/171534/2017	A / H5N8	Europe / France
EPI_ISL_4064661	A/duck/France/171535/2017	A / H5N8	Europe / France
EPI_ISL_4064662	A/duck/France/171536/2017	A / H5N8	Europe / France
EPI_ISL_4064663	A/chicken/France/171537/2017	A / H5N8	Europe / France
EPI_ISL_4064664	A/duck/France/171539/2017	A / H5N8	Europe / France
EPI_ISL_4064665	A/duck/France/171541/2017	A / H5N8	Europe / France
EPI_ISL_4064666	A/duck/France/171542/2017	A / H5N8	Europe / France
EPI_ISL_4064667	A/duck/France/171543/2017	A / H5N8	Europe / France
EPI_ISL_4064668	A/duck/France/171546/2017	A / H5N8	Europe / France
EPI_ISL_4064669	A/duck/France/171550/2017	A / H5N8	Europe / France
EPI_ISL_4064670	A/avian/France/171599/2017	A / H5N8	Europe / France
EPI_ISL_4064671	A/duck/France/171603/2017	A / H5N8	Europe / France
EPI_ISL_4064672	A/avian/France/171606/2017	A / H5N8	Europe / France
EPI_ISL_4064673	A/duck/France/171607/2017	A / H5N8	Europe / France
EPI_ISL_4064674	A/duck/France/171639/2017	A / H5N8	Europe / France
EPI_ISL_4064675	A/duck/France/171641/2017	A / H5N8	Europe / France
EPI_ISL_4064676	A/duck/France/171650/2017	A / H5N8	Europe / France
EPI_ISL_4064677	A/duck/France/171860/2017	A / H5N8	Europe / France
EPI_ISL_4064678	A/duck/France/171874/2017	A / H5N8	Europe / France
EPI_ISL_4064679	A/duck/France/171884/2017	A / H5N8	Europe / France
EPI_ISL_4064680	A/chicken/France/171915/2017	A / H5N8	Europe / France
EPI_ISL_4064681	A/duck/France/171928/2017	A / H5N8	Europe / France
EPI_ISL_4064682	A/duck/France/171929/2017	A / H5N8	Europe / France
EPI_ISL_4064683	A/duck/France/171932/2017	A / H5N8	Europe / France
EPI_ISL_4064684	A/chicken/France/172002/2017	A / H5N8	Europe / France
EPI_ISL_4064685	A/chicken/France/172003/2017	A / H5N8	Europe / France
EPI_ISL_4064686	A/duck/France/172004/2017	A / H5N8	Europe / France
EPI_ISL_4064687	A/duck/France/172012/2017	A / H5N8	Europe / France
EPI_ISL_4064688	A/duck/France/172038/2017	A / H5N8	Europe / France
EPI_ISL_4064689	A/duck/France/172057/2017	A / H5N8	Europe / France
EPI_ISL_4064690	A/chicken/France/172092/2017	A / H5N8	Europe / France
EPI_ISL_4064691	A/chicken/France/172093/2017	A / H5N8	Europe / France
EPI_ISL_4064692	A/duck/France/172094/2017	A / H5N8	Europe / France
EPI_ISL_4064693	A/duck/France/172096/2017	A / H5N8	Europe / France
EPI_ISL_4064694	A/chicken/France/172137/2017	A / H5N8	Europe / France
EPI_ISL_4064695	A/duck/France/172144/2017	A / H5N8	Europe / France
EPI_ISL_4064696	A/duck/France/172182/2017	A / H5N8	Europe / France
EPI_ISL_4064697	A/chicken/France/172287/2017	A / H5N8	Europe / France
EPI_ISL_4064698	A/duck/France/172289/2017	A / H5N8	Europe / France
EPI_ISL_4064699	A/duck/France/172331/2017	A / H5N8	Europe / France
EPI_ISL_4064700	A/duck/France/172362/2017	A / H5N8	Europe / France
EPI_ISL_4064701	A/chicken/France/172383/2017	A / H5N8	Europe / France
EPI_ISL_4064702	A/duck/France/172384/2017	A / H5N8	Europe / France
EPI_ISL_4064703	A/duck/France/172390/2017	A / H5N8	Europe / France
EPI_ISL_4064704	A/duck/France/172394/2017	A / H5N8	Europe / France
EPI_ISL_4064705	A/chicken/France/172610/2017	A / H5N8	Europe / France
EPI_ISL_4064707	A/swan/France/171265/2017	A / H5N8	Europe / France
EPI_ISL_4064708	A/gull/France/161176/2017	A / H5N8	Europe / France

EPI_ISL_4064709	A/swan/France/171377/2017	A / H5N8	Europe / France
EPI_ISL_4064710	A/swan/France/170496/2017	A / H5N8	Europe / France
EPI_ISL_4064711	A/duck/France/171779/2017	A / H5N8	Europe / France
EPI_ISL_4064712	A/swan/France/171960/2017	A / H5N8	Europe / France
EPI_ISL_4064713	A/swan/France/170166/2017	A / H5N8	Europe / France
EPI_ISL_4064714	A/goose/France/171953/2017	A / H5N8	Europe / France
EPI_ISL_4064715	A/swan/France/171585/2017	A / H5N8	Europe / France
EPI_ISL_4064716	A/swan/France/171376/2017	A / H5N8	Europe / France
EPI_ISL_4064717	A/swan/France/171267/2017	A / H5N8	Europe / France
EPI_ISL_4069650	A/chicken/Egypt/N16732/2019	A / H5N8	Africa / Egypt
EPI_ISL_4069651	A/duck/Egypt/N16719/2019	A / H5N8	Africa / Egypt
EPI_ISL_4069652	A/chicken/Egypt/Q16711B/2019	A / H5N8	Africa / Egypt
EPI_ISL_4069678	A/chicken/Egypt/Q16710C/2019	A / H5N8	Africa / Egypt
EPI_ISL_4069710	A/chicken/Egypt/Q16711A/2019	A / H5N8	Africa / Egypt
EPI_ISL_4069736	A/duck/Egypt/Q16716A/2019	A / H5N8	Africa / Egypt
EPI_ISL_4069750	A/duck/Egypt/N16720/2019	A / H5N8	Africa / Egypt
EPI_ISL_4069752	A/chicken/Egypt/N16730/2019	A / H5N8	Africa / Egypt
EPI_ISL_4069756	A/chicken/Egypt/Q16711C/2019	A / H5N8	Africa / Egypt
EPI_ISL_4069758	A/duck/Egypt/N16717/2019	A / H5N8	Africa / Egypt
EPI_ISL_4069759	A/chicken/Egypt/Q16712A/2019	A / H5N8	Africa / Egypt
EPI_ISL_4069762	A/duck/Egypt/N16722/2019	A / H5N8	Africa / Egypt
EPI_ISL_4069765	A/chicken/Egypt/Q16807E/2019	A / H5N8	Africa / Egypt
EPI_ISL_4069778	A/duck/Egypt/A16793/2019	A / H5N8	Africa / Egypt
EPI_ISL_4070128	A/duck/Denmark/19062-5Sp1c/2017	A / H5N8	Europe / Denmark
EPI_ISL_4070172	A/goose/Denmark/1365-1p1c/2017	A / H5N8	Europe / Denmark
EPI_ISL_4070378	A/pigeon/Egypt/A16805/2019	A / H5N8	Africa / Egypt
EPI_ISL_4070379	A/chicken/Egypt/Q16710B/2019	A / H5N8	Africa / Egypt
EPI_ISL_4070380	A/chicken/Egypt/Q16808B/2019	A / H5N8	Africa / Egypt
EPI_ISL_4070383	A/chicken/Egypt/Q16807B/2019	A / H5N8	Africa / Egypt
EPI_ISL_4070608	A/chicken/Egypt/Q16710A/2019	A / H5N8	Africa / Egypt
EPI_ISL_4070728	A/pigeon/Egypt/A16804/2019	A / H5N8	Africa / Egypt
EPI_ISL_4070816	A/chicken/Egypt/Q16807C/2019	A / H5N8	Africa / Egypt
EPI_ISL_4070847	A/chicken/Egypt/F17230B/2019	A / H5N8	Africa / Egypt
EPI_ISL_4070896	A/chicken/Egypt/F17230D/2019	A / H5N8	Africa / Egypt
EPI_ISL_4070932	A/duck/Egypt/N16721/2019	A / H5N8	Africa / Egypt
EPI_ISL_4070933	A/chicken/Egypt/F17229A/2019	A / H5N8	Africa / Egypt
EPI_ISL_4070935	A/pigeon/Egypt/A16800/2019	A / H5N8	Africa / Egypt
EPI_ISL_4070936	A/chicken/Egypt/F17229B/2019	A / H5N8	Africa / Egypt
EPI_ISL_4070940	A/chicken/Egypt/F17230C/2019	A / H5N8	Africa / Egypt
EPI_ISL_4070945	A/chicken/Egypt/F17230A/2019	A / H5N8	Africa / Egypt
EPI_ISL_4071019	A/falcated duck/Kagoshima/KU-d3/2020	A / H5N8	Asia / Japan / Kagoshima
EPI_ISL_4071447	A/environment/Bangladesh/42410/2020	A / H5N6	Asia / Bangladesh
EPI_ISL_4071515	A/duck/Bangladesh/43127/2020	A / H5N6	Asia / Bangladesh
EPI_ISL_4071518	A/environment/Bangladesh/42416/2020	A / H5N6	Asia / Bangladesh
EPI_ISL_4071595	A/duck/Bangladesh/43050/2020	A / H5N6	Asia / Bangladesh
EPI_ISL_4071615	A/duck/Bangladesh/43123/2020	A / H5N6	Asia / Bangladesh
EPI_ISL_4071616	A/Ferruginous duck/Bangladesh/42380/2020	A / H5N6	Asia / Bangladesh
EPI_ISL_4071631	A/duck/Bangladesh/43128/2020	A / H5N6	Asia / Bangladesh
EPI_ISL_4071634	A/duck/Bangladesh/43082/2020	A / H5N6	Asia / Bangladesh
EPI_ISL_4071635	A/duck/Bangladesh/43120/2020	A / H5N6	Asia / Bangladesh
EPI_ISL_4071638	A/duck/Bangladesh/43099/2020	A / H5N6	Asia / Bangladesh
EPI_ISL_4071641	A/duck/Bangladesh/43129/2020	A / H5N6	Asia / Bangladesh
EPI_ISL_4071723	A/duck/Bangladesh/43122/2020	A / H5N6	Asia / Bangladesh
EPI_ISL_4071724	A/Common pochard/Bangladesh/42386/2020	A / H5N6	Asia / Bangladesh
EPI_ISL_4071728	A/duck/Bangladesh/43119/2020	A / H5N6	Asia / Bangladesh
EPI_ISL_4071929	A/chicken/Zhejiang/6107/2016	A / H5N6	Asia / China / Zhejiang Province
EPI_ISL_4071930	A/chicken/Zhejiang/13160/2016	A / H5N6	Asia / China / Zhejiang Province
EPI_ISL_4071931	A/chicken/Zhejiang/528127/2016	A / H5N6	Asia / China / Zhejiang Province
EPI_ISL_4071932	A/Muscovy duck/China/H5N6/2020	A / H5N6	Asia / China
EPI_ISL_4072009	A/chicken/Zhejiang/102637/2016	A / H5N6	Asia / China / Zhejiang Province
EPI_ISL_4072173	A/chicken/Zhejiang/1130131/2016	A / H5N6	Asia / China / Zhejiang Province
EPI_ISL_4073121	A/duck/Bangladesh/44433/2020	A / H5N6	Asia / Bangladesh
EPI_ISL_4073122	A/duck/Bangladesh/44442/2020	A / H5N6	Asia / Bangladesh
EPI_ISL_4073123	A/duck/Bangladesh/44448/2020	A / H5N6	Asia / Bangladesh
EPI_ISL_4073127	A/duck/Bangladesh/44423/2020	A / H5N6	Asia / Bangladesh
EPI_ISL_4073128	A/duck/Bangladesh/44432/2020	A / H5N6	Asia / Bangladesh
EPI_ISL_4073129	A/duck/Bangladesh/44440/2020	A / H5N6	Asia / Bangladesh
EPI_ISL_4073130	A/duck/Bangladesh/44524/2020	A / H5N6	Asia / Bangladesh
EPI_ISL_4073131	A/duck/Bangladesh/44453/2020	A / H5N6	Asia / Bangladesh
EPI_ISL_4073132	A/duck/Bangladesh/44469/2020	A / H5N6	Asia / Bangladesh
EPI_ISL_4073133	A/duck/Bangladesh/44424/2020	A / H5N6	Asia / Bangladesh
EPI_ISL_4073156	A/duck/Bangladesh/44417/2020	A / H5N6	Asia / Bangladesh

EPI_ISL_4073171	A/duck/Bangladesh/44502/2020	A / H5N6	Asia / Bangladesh	
EPI_ISL_4073173	A/duck/Bangladesh/44484/2020	A / H5N6	Asia / Bangladesh	
EPI_ISL_4073174	A/duck/Bangladesh/44418/2020	A / H5N6	Asia / Bangladesh	
EPI_ISL_4073175	A/duck/Bangladesh/44447/2020	A / H5N6	Asia / Bangladesh	
EPI_ISL_4073176	A/duck/Bangladesh/44430/2020	A / H5N6	Asia / Bangladesh	
EPI_ISL_4073177	A/duck/Bangladesh/44456/2020	A / H5N6	Asia / Bangladesh	
EPI_ISL_4073178	A/duck/Bangladesh/44434/2020	A / H5N6	Asia / Bangladesh	
EPI_ISL_4073179	A/duck/Bangladesh/44471/2020	A / H5N6	Asia / Bangladesh	
EPI_ISL_4073181	A/duck/Bangladesh/44477/2020	A / H5N6	Asia / Bangladesh	
EPI_ISL_4073186	A/duck/Bangladesh/44523/2020	A / H5N6	Asia / Bangladesh	
EPI_ISL_4073189	A/duck/Bangladesh/44508/2020	A / H5N6	Asia / Bangladesh	
EPI_ISL_4073195	A/duck/Bangladesh/44504/2020	A / H5N6	Asia / Bangladesh	
EPI_ISL_408528	A/spot_billed_duck/Korea/H0027/2014	A / H5N8	Asia / Korea, Republic of	Yu-Na Lee (Animal and Plant Quarantine Agency (S-2158) / Avian Influenza Research & Diagnostic Division)
EPI_ISL_408530	A/duck/Korea/H0032/2014	A / H5N8	Asia / Korea, Republic of	Yu-Na Lee (Animal and Plant Quarantine Agency (S-2158) / Avian Influenza Research & Diagnostic Division)
EPI_ISL_408531	A/duck/Korea/H0035/2014	A / H5N8	Asia / Korea, Republic of	Yu-Na Lee (Animal and Plant Quarantine Agency (S-2158) / Avian Influenza Research & Diagnostic Division)
EPI_ISL_408532	A/duck/Korea/H0075/2014	A / H5N8	Asia / Korea, Republic of	Yu-Na Lee (Animal and Plant Quarantine Agency (S-2158) / Avian Influenza Research & Diagnostic Division)
EPI_ISL_408533	A/duck/Korea/H0078/2014	A / H5N8	Asia / Korea, Republic of	Yu-Na Lee (Animal and Plant Quarantine Agency (S-2158) / Avian Influenza Research & Diagnostic Division)
EPI_ISL_408534	A/duck/Korea/H0085/2014	A / H5N8	Asia / Korea, Republic of	Yu-Na Lee (Animal and Plant Quarantine Agency (S-2158) / Avian Influenza Research & Diagnostic Division)
EPI_ISL_408535	A/duck/Korea/H0089/2014	A / H5N8	Asia / Korea, Republic of	Yu-Na Lee (Animal and Plant Quarantine Agency (S-2158) / Avian Influenza Research & Diagnostic Division)
EPI_ISL_408536	A/duck/Korea/H0090/2014	A / H5N8	Asia / Korea, Republic of	Yu-Na Lee (Animal and Plant Quarantine Agency (S-2158) / Avian Influenza Research & Diagnostic Division)
EPI_ISL_408537	A/duck/Korea/H0106/2014	A / H5N8	Asia / Korea, Republic of	Yu-Na Lee (Animal and Plant Quarantine Agency (S-2158) / Avian Influenza Research & Diagnostic Division)
EPI_ISL_408538	A/duck/Korea/H0108/2014	A / H5N8	Asia / Korea, Republic of	Yu-Na Lee (Animal and Plant Quarantine Agency (S-2158) / Avian Influenza Research & Diagnostic Division)
EPI_ISL_408539	A/duck/Korea/H0109/2014	A / H5N8	Asia / Korea, Republic of	Yu-Na Lee (Animal and Plant Quarantine Agency (S-2158) / Avian Influenza Research & Diagnostic Division)
EPI_ISL_408540	A/duck/Korea/H0110/2014	A / H5N8	Asia / Korea, Republic of	Yu-Na Lee (Animal and Plant Quarantine Agency (S-2158) / Avian Influenza Research & Diagnostic Division)
EPI_ISL_408574	A/duck/Korea/H0111/2014	A / H5N8	Asia / Korea, Republic of	Yu-Na Lee (Animal and Plant Quarantine Agency (S-2158) / Avian Influenza Research & Diagnostic Division)
EPI_ISL_408575	A/duck/Korea/H0112/2014	A / H5N8	Asia / Korea, Republic of	Yu-Na Lee (Animal and Plant Quarantine Agency (S-2158) / Avian Influenza Research & Diagnostic Division)
EPI_ISL_408576	A/chicken/Korea/H0151/2014	A / H5N8	Asia / Korea, Republic of	Yu-Na Lee (Animal and Plant Quarantine Agency (S-2158) / Avian Influenza Research & Diagnostic Division)
EPI_ISL_408577	A/duck/Korea/H0193/2014	A / H5N8	Asia / Korea, Republic of	Yu-Na Lee (Animal and Plant Quarantine Agency (S-2158) / Avian Influenza Research & Diagnostic Division)
EPI_ISL_408578	A/duck/Korea/H0196/2014	A / H5N8	Asia / Korea, Republic of	Yu-Na Lee (Animal and Plant Quarantine Agency (S-2158) / Avian Influenza Research & Diagnostic Division)
EPI_ISL_408579	A/baikal_teal/Korea/H0203/2014	A / H5N8	Asia / Korea, Republic of	Yu-Na Lee (Animal and Plant Quarantine Agency (S-2158) / Avian Influenza Research & Diagnostic Division)
EPI_ISL_408580	A/duck/Korea/H0262/2014	A / H5N8	Asia / Korea, Republic of	Yu-Na Lee (Animal and Plant Quarantine Agency (S-2158) / Avian Influenza Research & Diagnostic Division)
EPI_ISL_408581	A/duck/Korea/H0266/2014	A / H5N8	Asia / Korea, Republic of	Yu-Na Lee (Animal and Plant Quarantine Agency (S-2158) / Avian Influenza Research & Diagnostic Division)
EPI_ISL_408582	A/duck/Korea/H0268/2014	A / H5N8	Asia / Korea, Republic of	Yu-Na Lee (Animal and Plant Quarantine Agency (S-2158) / Avian Influenza Research & Diagnostic Division)
EPI_ISL_408583	A/duck/Korea/H0277/2014	A / H5N8	Asia / Korea, Republic of	Yu-Na Lee (Animal and Plant Quarantine Agency (S-2158) / Avian Influenza Research & Diagnostic Division)
EPI_ISL_408584	A/Korean_native_chicken/Korea/H0300/2014	A / H5N8	Asia / Korea, Republic of	Yu-Na Lee (Animal and Plant Quarantine Agency (S-2158) / Avian Influenza Research & Diagnostic Division)
EPI_ISL_408585	A/duck/Korea/H0308/2014	A / H5N8	Asia / Korea, Republic of	Yu-Na Lee (Animal and Plant Quarantine Agency (S-2158) / Avian Influenza Research & Diagnostic Division)
EPI_ISL_408586	A/duck/Korea/H0309/2014	A / H5N8	Asia / Korea, Republic of	Yu-Na Lee (Animal and Plant Quarantine Agency (S-2158) / Avian Influenza Research & Diagnostic Division)
EPI_ISL_408587	A/duck/Korea/H0329/2014	A / H5N8	Asia / Korea, Republic of	Yu-Na Lee (Animal and Plant Quarantine Agency (S-2158) / Avian Influenza Research & Diagnostic Division)
EPI_ISL_408588	A/duck/Korea/H0330/2014	A / H5N8	Asia / Korea, Republic of	Yu-Na Lee (Animal and Plant Quarantine Agency (S-2158) / Avian Influenza Research & Diagnostic Division)
EPI_ISL_408590	A/duck/Korea/H0351/2014	A / H5N8	Asia / Korea, Republic of	Yu-Na Lee (Animal and Plant Quarantine Agency (S-2158) / Avian Influenza Research & Diagnostic Division)
EPI_ISL_408591	A/duck/Korea/H0356/2014	A / H5N8	Asia / Korea, Republic of	Yu-Na Lee (Animal and Plant Quarantine Agency (S-2158) / Avian Influenza Research & Diagnostic Division)
EPI_ISL_408592	A/duck/Korea/H0357/2014	A / H5N8	Asia / Korea, Republic of	Yu-Na Lee (Animal and Plant Quarantine Agency (S-2158) / Avian Influenza Research & Diagnostic Division)
EPI_ISL_408593	A/duck/Korea/H0361/2014	A / H5N8	Asia / Korea, Republic of	Yu-Na Lee (Animal and Plant Quarantine Agency (S-2158) / Avian Influenza Research & Diagnostic Division)
EPI_ISL_408594	A/duck/Korea/H0362/2014	A / H5N8	Asia / Korea, Republic of	Yu-Na Lee (Animal and Plant Quarantine Agency (S-2158) / Avian Influenza Research & Diagnostic Division)
EPI_ISL_408595	A/duck/Korea/H0390/2014	A / H5N8	Asia / Korea, Republic of	Yu-Na Lee (Animal and Plant Quarantine Agency (S-2158) / Avian Influenza Research & Diagnostic Division)
EPI_ISL_408596	A/duck/Korea/H0397/2014	A / H5N8	Asia / Korea, Republic of	Yu-Na Lee (Animal and Plant Quarantine Agency (S-2158) / Avian Influenza Research & Diagnostic Division)
EPI_ISL_408597	A/duck/Korea/H0398/2014	A / H5N8	Asia / Korea, Republic of	Yu-Na Lee (Animal and Plant Quarantine Agency (S-2158) / Avian Influenza Research & Diagnostic Division)
EPI_ISL_408598	A/duck/Korea/H0400/2014	A / H5N8	Asia / Korea, Republic of	Yu-Na Lee (Animal and Plant Quarantine Agency (S-2158) / Avian Influenza Research & Diagnostic Division)
EPI_ISL_408599	A/duck/Korea/H0414/2014	A / H5N8	Asia / Korea, Republic of	Yu-Na Lee (Animal and Plant Quarantine Agency (S-2158) / Avian Influenza Research & Diagnostic Division)
EPI_ISL_408600	A/chicken/Korea/H0421/2014	A / H5N8	Asia / Korea, Republic of	Yu-Na Lee (Animal and Plant Quarantine Agency (S-2158) / Avian Influenza Research & Diagnostic Division)
EPI_ISL_408601	A/chicken/Korea/H0422/2014	A / H5N8	Asia / Korea, Republic of	Yu-Na Lee (Animal and Plant Quarantine Agency (S-2158) / Avian Influenza Research & Diagnostic Division)
EPI_ISL_408602	A/duck/Korea/H0425/2014	A / H5N8	Asia / Korea, Republic of	Yu-Na Lee (Animal and Plant Quarantine Agency (S-2158) / Avian Influenza Research & Diagnostic Division)
EPI_ISL_408603	A/duck/Korea/H0426/2014	A / H5N8	Asia / Korea, Republic of	Yu-Na Lee (Animal and Plant Quarantine Agency (S-2158) / Avian Influenza Research & Diagnostic Division)
EPI_ISL_408604	A/duck/Korea/H0428/2014	A / H5N8	Asia / Korea, Republic of	Yu-Na Lee (Animal and Plant Quarantine Agency (S-2158) / Avian Influenza Research & Diagnostic Division)
EPI_ISL_408605	A/duck/Korea/H0429/2014	A / H5N8	Asia / Korea, Republic of	Yu-Na Lee (Animal and Plant Quarantine Agency (S-2158) / Avian Influenza Research & Diagnostic Division)
EPI_ISL_408606	A/duck/Korea/H0430/2014	A / H5N8	Asia / Korea, Republic of	Yu-Na Lee (Animal and Plant Quarantine Agency (S-2158) / Avian Influenza Research & Diagnostic Division)
EPI_ISL_408607	A/duck/Korea/H0431/2014	A / H5N8	Asia / Korea, Republic of	Yu-Na Lee (Animal and Plant Quarantine Agency (S-2158) / Avian Influenza Research & Diagnostic Division)
EPI_ISL_408608	A/duck/Korea/H0439/2014	A / H5N8	Asia / Korea, Republic of	Yu-Na Lee (Animal and Plant Quarantine Agency (S-2158) / Avian Influenza Research & Diagnostic Division)
EPI_ISL_408609	A/duck/Korea/H0445/2014	A / H5N8	Asia / Korea, Republic of	Yu-Na Lee (Animal and Plant Quarantine Agency (S-2158) / Avian Influenza Research & Diagnostic Division)
EPI_ISL_408610	A/environment/Korea/H0469/2014	A / H5N8	Asia / Korea, Republic of	Yu-Na Lee (Animal and Plant Quarantine Agency (S-2158) / Avian Influenza Research & Diagnostic Division)
EPI_ISL_408611	A/chicken/Korea/H0470/2014	A / H5N8	Asia / Korea, Republic of	Yu-Na Lee (Animal and Plant Quarantine Agency (S-2158) / Avian Influenza Research & Diagnostic Division)
EPI_ISL_408612	A/duck/Korea/H0488/2014	A / H5N8	Asia / Korea, Republic of	Yu-Na Lee (Animal and Plant Quarantine Agency (S-2158) / Avian Influenza Research & Diagnostic Division)
EPI_ISL_408613	A/spot_billed_duck/Korea/H0497/2014	A / H5N8	Asia / Korea, Republic of	Yu-Na Lee (Animal and Plant Quarantine Agency (S-2158) / Avian Influenza Research & Diagnostic Division)
EPI_ISL_408615	A/duck/Korea/H0512/2014	A / H5N8	Asia / Korea, Republic of	Yu-Na Lee (Animal and Plant Quarantine Agency (S-2158) / Avian Influenza Research & Diagnostic Division)
EPI_ISL_408616	A/duck/Korea/H0513/2014	A / H5N8	Asia / Korea, Republic of	Yu-Na Lee (Animal and Plant Quarantine Agency (S-2158) / Avian Influenza Research & Diagnostic Division)
EPI_ISL_408617	A/duck/Korea/H0525/2014	A / H5N8	Asia / Korea, Republic of	Yu-Na Lee (Animal and Plant Quarantine Agency (S-2158) / Avian Influenza Research & Diagnostic Division)
EPI_ISL_408618	A/chicken/Korea/H0526/2014	A / H5N8	Asia / Korea, Republic of	Yu-Na Lee (Animal and Plant Quarantine Agency (S-2158) / Avian Influenza Research & Diagnostic Division)
EPI_ISL_408619	A/duck/Korea/H0527/2014	A / H5N8	Asia / Korea, Republic of	Yu-Na Lee (Animal and Plant Quarantine Agency (S-2158) / Avian Influenza Research & Diagnostic Division)
EPI_ISL_408620	A/chicken/Korea/H0533/2014	A / H5N8	Asia / Korea, Republic of	Yu-Na Lee (Animal and Plant Quarantine Agency (S-2158) / Avian Influenza Research & Diagnostic Division)
EPI_ISL_408621	A/duck/Korea/H0534/2014	A / H5N8	Asia / Korea, Republic of	Yu-Na Lee (Animal and Plant Quarantine Agency (S-2158) / Avian Influenza Research & Diagnostic Division)
EPI_ISL_408622	A/duck/Korea/H0535/2014	A / H5N8	Asia / Korea, Republic of	Yu-Na Lee (Animal and Plant Quarantine Agency (S-2158) / Avian Influenza Research & Diagnostic Division)
EPI_ISL_408623	A/duck/Korea/H0537/2014	A / H5N8	Asia / Korea, Republic of	Yu-Na Lee (Animal and Plant Quarantine Agency (S-2158) / Avian Influenza Research & Diagnostic Division)

EPI_ISL_408624	A/duck/Korea/H0539/2014	A / H5N8	Asia / Korea, Republic of	Yu-Na Lee (Animal and Plant Quarantine Agency (S-2158) / Avian Influenza Research & Diagnostic Division)
EPI_ISL_408625	A/chicken/Korea/H0541/2014	A / H5N8	Asia / Korea, Republic of	Yu-Na Lee (Animal and Plant Quarantine Agency (S-2158) / Avian Influenza Research & Diagnostic Division)
EPI_ISL_408626	A/chicken/Korea/H0550/2014	A / H5N8	Asia / Korea, Republic of	Yu-Na Lee (Animal and Plant Quarantine Agency (S-2158) / Avian Influenza Research & Diagnostic Division)
EPI_ISL_408627	A/duck/Korea/H0551/2014	A / H5N8	Asia / Korea, Republic of	Yu-Na Lee (Animal and Plant Quarantine Agency (S-2158) / Avian Influenza Research & Diagnostic Division)
EPI_ISL_408628	A/mandarin_duck/Korea/H0555/2014	A / H5N8	Asia / Korea, Republic of	Yu-Na Lee (Animal and Plant Quarantine Agency (S-2158) / Avian Influenza Research & Diagnostic Division)
EPI_ISL_408629	A/duck/Korea/H0556/2014	A / H5N8	Asia / Korea, Republic of	Yu-Na Lee (Animal and Plant Quarantine Agency (S-2158) / Avian Influenza Research & Diagnostic Division)
EPI_ISL_408630	A/duck/Korea/H0557/2014	A / H5N8	Asia / Korea, Republic of	Yu-Na Lee (Animal and Plant Quarantine Agency (S-2158) / Avian Influenza Research & Diagnostic Division)
EPI_ISL_408631	A/duck/Korea/H0561/2014	A / H5N8	Asia / Korea, Republic of	Yu-Na Lee (Animal and Plant Quarantine Agency (S-2158) / Avian Influenza Research & Diagnostic Division)
EPI_ISL_408633	A/environment/Korea/H0576/2014	A / H5N8	Asia / Korea, Republic of	Yu-Na Lee (Animal and Plant Quarantine Agency (S-2158) / Avian Influenza Research & Diagnostic Division)
EPI_ISL_408634	A/environment/Korea/H0577/2014	A / H5N8	Asia / Korea, Republic of	Yu-Na Lee (Animal and Plant Quarantine Agency (S-2158) / Avian Influenza Research & Diagnostic Division)
EPI_ISL_408635	A/duck/Korea/H0580/2014	A / H5N8	Asia / Korea, Republic of	Yu-Na Lee (Animal and Plant Quarantine Agency (S-2158) / Avian Influenza Research & Diagnostic Division)
EPI_ISL_408636	A/duck/Korea/H0582/2014	A / H5N8	Asia / Korea, Republic of	Yu-Na Lee (Animal and Plant Quarantine Agency (S-2158) / Avian Influenza Research & Diagnostic Division)
EPI_ISL_408637	A/duck/Korea/H0583/2014	A / H5N8	Asia / Korea, Republic of	Yu-Na Lee (Animal and Plant Quarantine Agency (S-2158) / Avian Influenza Research & Diagnostic Division)
EPI_ISL_408638	A/duck/Korea/H0584/2014	A / H5N8	Asia / Korea, Republic of	Yu-Na Lee (Animal and Plant Quarantine Agency (S-2158) / Avian Influenza Research & Diagnostic Division)
EPI_ISL_408639	A/duck/Korea/H0585/2014	A / H5N8	Asia / Korea, Republic of	Yu-Na Lee (Animal and Plant Quarantine Agency (S-2158) / Avian Influenza Research & Diagnostic Division)
EPI_ISL_408640	A/duck/Korea/H0586/2014	A / H5N8	Asia / Korea, Republic of	Yu-Na Lee (Animal and Plant Quarantine Agency (S-2158) / Avian Influenza Research & Diagnostic Division)
EPI_ISL_408641	A/environment/Korea/H0591/2014	A / H5N8	Asia / Korea, Republic of	Yu-Na Lee (Animal and Plant Quarantine Agency (S-2158) / Avian Influenza Research & Diagnostic Division)
EPI_ISL_408642	A/duck/Korea/H0602/2014	A / H5N8	Asia / Korea, Republic of	Yu-Na Lee (Animal and Plant Quarantine Agency (S-2158) / Avian Influenza Research & Diagnostic Division)
EPI_ISL_408643	A/duck/Korea/H0603/2014	A / H5N8	Asia / Korea, Republic of	Yu-Na Lee (Animal and Plant Quarantine Agency (S-2158) / Avian Influenza Research & Diagnostic Division)
EPI_ISL_408644	A/duck/Korea/H0605/2014	A / H5N8	Asia / Korea, Republic of	Yu-Na Lee (Animal and Plant Quarantine Agency (S-2158) / Avian Influenza Research & Diagnostic Division)
EPI_ISL_408645	A/duck/Korea/H0606/2014	A / H5N8	Asia / Korea, Republic of	Yu-Na Lee (Animal and Plant Quarantine Agency (S-2158) / Avian Influenza Research & Diagnostic Division)
EPI_ISL_408646	A/duck/Korea/H0607/2014	A / H5N8	Asia / Korea, Republic of	Yu-Na Lee (Animal and Plant Quarantine Agency (S-2158) / Avian Influenza Research & Diagnostic Division)
EPI_ISL_408647	A/duck/Korea/H0609/2014	A / H5N8	Asia / Korea, Republic of	Yu-Na Lee (Animal and Plant Quarantine Agency (S-2158) / Avian Influenza Research & Diagnostic Division)
EPI_ISL_408648	A/duck/Korea/H0610/2014	A / H5N8	Asia / Korea, Republic of	Yu-Na Lee (Animal and Plant Quarantine Agency (S-2158) / Avian Influenza Research & Diagnostic Division)
EPI_ISL_408649	A/duck/Korea/H0611/2014	A / H5N8	Asia / Korea, Republic of	Yu-Na Lee (Animal and Plant Quarantine Agency (S-2158) / Avian Influenza Research & Diagnostic Division)
EPI_ISL_408650	A/duck/Korea/H0613/2014	A / H5N8	Asia / Korea, Republic of	Yu-Na Lee (Animal and Plant Quarantine Agency (S-2158) / Avian Influenza Research & Diagnostic Division)
EPI_ISL_408651	A/duck/Korea/H0614/2014	A / H5N8	Asia / Korea, Republic of	Yu-Na Lee (Animal and Plant Quarantine Agency (S-2158) / Avian Influenza Research & Diagnostic Division)
EPI_ISL_408652	A/duck/Korea/H0615/2014	A / H5N8	Asia / Korea, Republic of	Yu-Na Lee (Animal and Plant Quarantine Agency (S-2158) / Avian Influenza Research & Diagnostic Division)
EPI_ISL_408653	A/duck/Korea/H0616/2014	A / H5N8	Asia / Korea, Republic of	Yu-Na Lee (Animal and Plant Quarantine Agency (S-2158) / Avian Influenza Research & Diagnostic Division)
EPI_ISL_408654	A/duck/Korea/H0617/2014	A / H5N8	Asia / Korea, Republic of	Yu-Na Lee (Animal and Plant Quarantine Agency (S-2158) / Avian Influenza Research & Diagnostic Division)
EPI_ISL_408655	A/duck/Korea/H0618/2014	A / H5N8	Asia / Korea, Republic of	Yu-Na Lee (Animal and Plant Quarantine Agency (S-2158) / Avian Influenza Research & Diagnostic Division)
EPI_ISL_408656	A/duck/Korea/H0619/2014	A / H5N8	Asia / Korea, Republic of	Yu-Na Lee (Animal and Plant Quarantine Agency (S-2158) / Avian Influenza Research & Diagnostic Division)
EPI_ISL_408657	A/duck/Korea/H0620/2014	A / H5N8	Asia / Korea, Republic of	Yu-Na Lee (Animal and Plant Quarantine Agency (S-2158) / Avian Influenza Research & Diagnostic Division)
EPI_ISL_408658	A/duck/Korea/H0621/2014	A / H5N8	Asia / Korea, Republic of	Yu-Na Lee (Animal and Plant Quarantine Agency (S-2158) / Avian Influenza Research & Diagnostic Division)
EPI_ISL_408659	A/duck/Korea/H0622/2014	A / H5N8	Asia / Korea, Republic of	Yu-Na Lee (Animal and Plant Quarantine Agency (S-2158) / Avian Influenza Research & Diagnostic Division)
EPI_ISL_408660	A/duck/Korea/H0623/2014	A / H5N8	Asia / Korea, Republic of	Yu-Na Lee (Animal and Plant Quarantine Agency (S-2158) / Avian Influenza Research & Diagnostic Division)
EPI_ISL_408661	A/duck/Korea/H0624/2014	A / H5N8	Asia / Korea, Republic of	Yu-Na Lee (Animal and Plant Quarantine Agency (S-2158) / Avian Influenza Research & Diagnostic Division)
EPI_ISL_408662	A/duck/Korea/H0625/2014	A / H5N8	Asia / Korea, Republic of	Yu-Na Lee (Animal and Plant Quarantine Agency (S-2158) / Avian Influenza Research & Diagnostic Division)
EPI_ISL_408663	A/duck/Korea/H0626/2014	A / H5N8	Asia / Korea, Republic of	Yu-Na Lee (Animal and Plant Quarantine Agency (S-2158) / Avian Influenza Research & Diagnostic Division)
EPI_ISL_408664	A/duck/Korea/H0646/2014	A / H5N8	Asia / Korea, Republic of	Yu-Na Lee (Animal and Plant Quarantine Agency (S-2158) / Avian Influenza Research & Diagnostic Division)
EPI_ISL_408673	A/duck/Korea/H0652/2014	A / H5N8	Asia / Korea, Republic of	Yu-Na Lee (Animal and Plant Quarantine Agency (S-2158) / Avian Influenza Research & Diagnostic Division)
EPI_ISL_408674	A/duck/Korea/H0653/2014	A / H5N8	Asia / Korea, Republic of	Yu-Na Lee (Animal and Plant Quarantine Agency (S-2158) / Avian Influenza Research & Diagnostic Division)
EPI_ISL_408675	A/chicken/Korea/H0660/2014	A / H5N8	Asia / Korea, Republic of	Yu-Na Lee (Animal and Plant Quarantine Agency (S-2158) / Avian Influenza Research & Diagnostic Division)
EPI_ISL_408676	A/duck/Korea/H0663/2014	A / H5N8	Asia / Korea, Republic of	Yu-Na Lee (Animal and Plant Quarantine Agency (S-2158) / Avian Influenza Research & Diagnostic Division)
EPI_ISL_408677	A/duck/Korea/H0673/2014	A / H5N8	Asia / Korea, Republic of	Yu-Na Lee (Animal and Plant Quarantine Agency (S-2158) / Avian Influenza Research & Diagnostic Division)
EPI_ISL_408678	A/duck/Korea/H0674/2014	A / H5N8	Asia / Korea, Republic of	Yu-Na Lee (Animal and Plant Quarantine Agency (S-2158) / Avian Influenza Research & Diagnostic Division)
EPI_ISL_408679	A/duck/Korea/H0691/2014	A / H5N8	Asia / Korea, Republic of	Yu-Na Lee (Animal and Plant Quarantine Agency (S-2158) / Avian Influenza Research & Diagnostic Division)
EPI_ISL_408680	A/duck/Korea/H0692/2014	A / H5N8	Asia / Korea, Republic of	Yu-Na Lee (Animal and Plant Quarantine Agency (S-2158) / Avian Influenza Research & Diagnostic Division)
EPI_ISL_408681	A/duck/Korea/H0694/2014	A / H5N8	Asia / Korea, Republic of	Yu-Na Lee (Animal and Plant Quarantine Agency (S-2158) / Avian Influenza Research & Diagnostic Division)
EPI_ISL_408682	A/environment/Korea/H0701/2014	A / H5N8	Asia / Korea, Republic of	Yu-Na Lee (Animal and Plant Quarantine Agency (S-2158) / Avian Influenza Research & Diagnostic Division)
EPI_ISL_408683	A/duck/Korea/H0705/2014	A / H5N8	Asia / Korea, Republic of	Yu-Na Lee (Animal and Plant Quarantine Agency (S-2158) / Avian Influenza Research & Diagnostic Division)
EPI_ISL_408684	A/duck/Korea/H0708/2014	A / H5N8	Asia / Korea, Republic of	Yu-Na Lee (Animal and Plant Quarantine Agency (S-2158) / Avian Influenza Research & Diagnostic Division)
EPI_ISL_408685	A/chicken/Korea/H0709/2014	A / H5N8	Asia / Korea, Republic of	Yu-Na Lee (Animal and Plant Quarantine Agency (S-2158) / Avian Influenza Research & Diagnostic Division)
EPI_ISL_408686	A/duck/Korea/H0711/2014	A / H5N8	Asia / Korea, Republic of	Yu-Na Lee (Animal and Plant Quarantine Agency (S-2158) / Avian Influenza Research & Diagnostic Division)
EPI_ISL_408687	A/chicken/Korea/H0713/2014	A / H5N8	Asia / Korea, Republic of	Yu-Na Lee (Animal and Plant Quarantine Agency (S-2158) / Avian Influenza Research & Diagnostic Division)
EPI_ISL_408688	A/duck/Korea/H0716/2014	A / H5N8	Asia / Korea, Republic of	Yu-Na Lee (Animal and Plant Quarantine Agency (S-2158) / Avian Influenza Research & Diagnostic Division)
EPI_ISL_408689	A/duck/Korea/H0717/2014	A / H5N8	Asia / Korea, Republic of	Yu-Na Lee (Animal and Plant Quarantine Agency (S-2158) / Avian Influenza Research & Diagnostic Division)
EPI_ISL_408690	A/duck/Korea/H0731/2014	A / H5N8	Asia / Korea, Republic of	Yu-Na Lee (Animal and Plant Quarantine Agency (S-2158) / Avian Influenza Research & Diagnostic Division)
EPI_ISL_408691	A/backyard_poultry/Korea/H0734/2014	A / H5N8	Asia / Korea, Republic of	Yu-Na Lee (Animal and Plant Quarantine Agency (S-2158) / Avian Influenza Research & Diagnostic Division)
EPI_ISL_408692	A/duck/Korea/H0741/2014	A / H5N8	Asia / Korea, Republic of	Yu-Na Lee (Animal and Plant Quarantine Agency (S-2158) / Avian Influenza Research & Diagnostic Division)
EPI_ISL_408693	A/environment/Korea/H0745/2014	A / H5N8	Asia / Korea, Republic of	Yu-Na Lee (Animal and Plant Quarantine Agency (S-2158) / Avian Influenza Research & Diagnostic Division)
EPI_ISL_408694	A/environment/Korea/H0760/2014	A / H5N8	Asia / Korea, Republic of	Yu-Na Lee (Animal and Plant Quarantine Agency (S-2158) / Avian Influenza Research & Diagnostic Division)
EPI_ISL_408695	A/duck/Korea/H0761/2014	A / H5N8	Asia / Korea, Republic of	Yu-Na Lee (Animal and Plant Quarantine Agency (S-2158) / Avian Influenza Research & Diagnostic Division)
EPI_ISL_408696	A/duck/Korea/H0763/2014	A / H5N8	Asia / Korea, Republic of	Yu-Na Lee (Animal and Plant Quarantine Agency (S-2158) / Avian Influenza Research & Diagnostic Division)
EPI_ISL_408697	A/duck/Korea/H0768/2014	A / H5N8	Asia / Korea, Republic of	Yu-Na Lee (Animal and Plant Quarantine Agency (S-2158) / Avian Influenza Research & Diagnostic Division)
EPI_ISL_408698	A/Eurasian_coot/Korea/H0770/2014	A / H5N8	Asia / Korea, Republic of	Yu-Na Lee (Animal and Plant Quarantine Agency (S-2158) / Avian Influenza Research & Diagnostic Division)
EPI_ISL_408699	A/chicken/Korea/H0773/2014	A / H5N8	Asia / Korea, Republic of	Yu-Na Lee (Animal and Plant Quarantine Agency (S-2158) / Avian Influenza Research & Diagnostic Division)
EPI_ISL_408700	A/duck/Korea/H0787/2014	A / H5N8	Asia / Korea, Republic of	Yu-Na Lee (Animal and Plant Quarantine Agency (S-2158) / Avian Influenza Research & Diagnostic Division)
EPI_ISL_408701	A/backyard_poultry/Korea/H0790/2014	A / H5N8	Asia / Korea, Republic of	Yu-Na Lee (Animal and Plant Quarantine Agency (S-2158) / Avian Influenza Research & Diagnostic Division)
EPI_ISL_408702	A/chicken/Korea/H0795/2014	A / H5N8	Asia / Korea, Republic of	Yu-Na Lee (Animal and Plant Quarantine Agency (S-2158) / Avian Influenza Research & Diagnostic Division)
EPI_ISL_408703	A/duck/Korea/H0797/2014	A / H5N8	Asia / Korea, Republic of	Yu-Na Lee (Animal and Plant Quarantine Agency (S-2158) / Avian Influenza Research & Diagnostic Division)
EPI_ISL_408704	A/chicken/Korea/H0811/2014	A / H5N8	Asia / Korea, Republic of	Yu-Na Lee (Animal and Plant Quarantine Agency (S-2158) / Avian Influenza Research & Diagnostic Division)

EPI_ISL_408705	A/duck/Korea/H0813/2014	A / H5N8	Asia / Korea, Republic of	Yu-Na Lee (Animal and Plant Quarantine Agency (S-2158) / Avian Influenza Research & Diagnostic Division)
EPI_ISL_408707	A/duck/Korea/H0822/2014	A / H5N8	Asia / Korea, Republic of	Yu-Na Lee (Animal and Plant Quarantine Agency (S-2158) / Avian Influenza Research & Diagnostic Division)
EPI_ISL_408708	A/duck/Korea/H0824/2014	A / H5N8	Asia / Korea, Republic of	Yu-Na Lee (Animal and Plant Quarantine Agency (S-2158) / Avian Influenza Research & Diagnostic Division)
EPI_ISL_408711	A/environment/Korea/H0832/2014	A / H5N8	Asia / Korea, Republic of	Yu-Na Lee (Animal and Plant Quarantine Agency (S-2158) / Avian Influenza Research & Diagnostic Division)
EPI_ISL_408712	A/chicken/Korea/H0835/2014	A / H5N8	Asia / Korea, Republic of	Yu-Na Lee (Animal and Plant Quarantine Agency (S-2158) / Avian Influenza Research & Diagnostic Division)
EPI_ISL_408713	A/duck/Korea/H0839/2014	A / H5N8	Asia / Korea, Republic of	Yu-Na Lee (Animal and Plant Quarantine Agency (S-2158) / Avian Influenza Research & Diagnostic Division)
EPI_ISL_408714	A/duck/Korea/H0845/2014	A / H5N8	Asia / Korea, Republic of	Yu-Na Lee (Animal and Plant Quarantine Agency (S-2158) / Avian Influenza Research & Diagnostic Division)
EPI_ISL_408715	A/environment/Korea/H0853/2014	A / H5N8	Asia / Korea, Republic of	Yu-Na Lee (Animal and Plant Quarantine Agency (S-2158) / Avian Influenza Research & Diagnostic Division)
EPI_ISL_408716	A/environment/Korea/H0867_2/2014	A / H5N8	Asia / Korea, Republic of	Yu-Na Lee (Animal and Plant Quarantine Agency (S-2158) / Avian Influenza Research & Diagnostic Division)
EPI_ISL_408717	A/duck/Korea/H0879/2014	A / H5N8	Asia / Korea, Republic of	Yu-Na Lee (Animal and Plant Quarantine Agency (S-2158) / Avian Influenza Research & Diagnostic Division)
EPI_ISL_408719	A/duck/Korea/H0882/2014	A / H5N8	Asia / Korea, Republic of	Yu-Na Lee (Animal and Plant Quarantine Agency (S-2158) / Avian Influenza Research & Diagnostic Division)
EPI_ISL_408720	A/chicken/Korea/H0884/2014	A / H5N8	Asia / Korea, Republic of	Yu-Na Lee (Animal and Plant Quarantine Agency (S-2158) / Avian Influenza Research & Diagnostic Division)
EPI_ISL_408721	A/duck/Korea/H0887/2014	A / H5N8	Asia / Korea, Republic of	Yu-Na Lee (Animal and Plant Quarantine Agency (S-2158) / Avian Influenza Research & Diagnostic Division)
EPI_ISL_408722	A/duck/Korea/H0888/2014	A / H5N8	Asia / Korea, Republic of	Yu-Na Lee (Animal and Plant Quarantine Agency (S-2158) / Avian Influenza Research & Diagnostic Division)
EPI_ISL_408723	A/chicken/Korea/H0891/2014	A / H5N8	Asia / Korea, Republic of	Yu-Na Lee (Animal and Plant Quarantine Agency (S-2158) / Avian Influenza Research & Diagnostic Division)
EPI_ISL_408724	A/chicken/Korea/H0893/2014	A / H5N8	Asia / Korea, Republic of	Yu-Na Lee (Animal and Plant Quarantine Agency (S-2158) / Avian Influenza Research & Diagnostic Division)
EPI_ISL_408725	A/chicken/Korea/H0898/2014	A / H5N8	Asia / Korea, Republic of	Yu-Na Lee (Animal and Plant Quarantine Agency (S-2158) / Avian Influenza Research & Diagnostic Division)
EPI_ISL_408726	A/duck/Korea/H0901/2014	A / H5N8	Asia / Korea, Republic of	Yu-Na Lee (Animal and Plant Quarantine Agency (S-2158) / Avian Influenza Research & Diagnostic Division)
EPI_ISL_408727	A/chicken/Korea/H0905/2014	A / H5N8	Asia / Korea, Republic of	Yu-Na Lee (Animal and Plant Quarantine Agency (S-2158) / Avian Influenza Research & Diagnostic Division)
EPI_ISL_408728	A/great_egret/Korea/H0912/2014	A / H5N8	Asia / Korea, Republic of	Yu-Na Lee (Animal and Plant Quarantine Agency (S-2158) / Avian Influenza Research & Diagnostic Division)
EPI_ISL_408729	A/bean_goose/Korea/H0924/2014	A / H5N8	Asia / Korea, Republic of	Yu-Na Lee (Animal and Plant Quarantine Agency (S-2158) / Avian Influenza Research & Diagnostic Division)
EPI_ISL_408730	A/duck/Korea/H0936/2014	A / H5N8	Asia / Korea, Republic of	Yu-Na Lee (Animal and Plant Quarantine Agency (S-2158) / Avian Influenza Research & Diagnostic Division)
EPI_ISL_408731	A/chicken/Korea/H0940/2014	A / H5N8	Asia / Korea, Republic of	Yu-Na Lee (Animal and Plant Quarantine Agency (S-2158) / Avian Influenza Research & Diagnostic Division)
EPI_ISL_408732	A/chicken/Korea/H0950/2014	A / H5N8	Asia / Korea, Republic of	Yu-Na Lee (Animal and Plant Quarantine Agency (S-2158) / Avian Influenza Research & Diagnostic Division)
EPI_ISL_408733	A/chicken/Korea/H0952/2014	A / H5N8	Asia / Korea, Republic of	Yu-Na Lee (Animal and Plant Quarantine Agency (S-2158) / Avian Influenza Research & Diagnostic Division)
EPI_ISL_408734	A/chicken/Korea/H0953/2014	A / H5N8	Asia / Korea, Republic of	Yu-Na Lee (Animal and Plant Quarantine Agency (S-2158) / Avian Influenza Research & Diagnostic Division)
EPI_ISL_408735	A/duck/Korea/H0958/2014	A / H5N8	Asia / Korea, Republic of	Yu-Na Lee (Animal and Plant Quarantine Agency (S-2158) / Avian Influenza Research & Diagnostic Division)
EPI_ISL_408737	A/chicken/Korea/H1008/2014	A / H5N8	Asia / Korea, Republic of	Yu-Na Lee (Animal and Plant Quarantine Agency (S-2158) / Avian Influenza Research & Diagnostic Division)
EPI_ISL_408738	A/duck/Korea/H1027/2014	A / H5N8	Asia / Korea, Republic of	Yu-Na Lee (Animal and Plant Quarantine Agency (S-2158) / Avian Influenza Research & Diagnostic Division)
EPI_ISL_408739	A/duck/Korea/H1030/2014	A / H5N8	Asia / Korea, Republic of	Yu-Na Lee (Animal and Plant Quarantine Agency (S-2158) / Avian Influenza Research & Diagnostic Division)
EPI_ISL_408740	A/duck/Korea/H1031/2014	A / H5N8	Asia / Korea, Republic of	Yu-Na Lee (Animal and Plant Quarantine Agency (S-2158) / Avian Influenza Research & Diagnostic Division)
EPI_ISL_408741	A/duck/Korea/H1032/2014	A / H5N8	Asia / Korea, Republic of	Yu-Na Lee (Animal and Plant Quarantine Agency (S-2158) / Avian Influenza Research & Diagnostic Division)
EPI_ISL_408742	A/duck/Korea/H1033/2014	A / H5N8	Asia / Korea, Republic of	Yu-Na Lee (Animal and Plant Quarantine Agency (S-2158) / Avian Influenza Research & Diagnostic Division)
EPI_ISL_408743	A/duck/Korea/H1037/2014	A / H5N8	Asia / Korea, Republic of	Yu-Na Lee (Animal and Plant Quarantine Agency (S-2158) / Avian Influenza Research & Diagnostic Division)
EPI_ISL_408744	A/duck/Korea/H1041/2014	A / H5N8	Asia / Korea, Republic of	Yu-Na Lee (Animal and Plant Quarantine Agency (S-2158) / Avian Influenza Research & Diagnostic Division)
EPI_ISL_408745	A/duck/Korea/H1056/2014	A / H5N8	Asia / Korea, Republic of	Yu-Na Lee (Animal and Plant Quarantine Agency (S-2158) / Avian Influenza Research & Diagnostic Division)
EPI_ISL_408746	A/duck/Korea/H1062/2014	A / H5N8	Asia / Korea, Republic of	Yu-Na Lee (Animal and Plant Quarantine Agency (S-2158) / Avian Influenza Research & Diagnostic Division)
EPI_ISL_408747	A/duck/Korea/H1067/2014	A / H5N8	Asia / Korea, Republic of	Yu-Na Lee (Animal and Plant Quarantine Agency (S-2158) / Avian Influenza Research & Diagnostic Division)
EPI_ISL_408749	A/duck/Korea/H1072/2014	A / H5N8	Asia / Korea, Republic of	Yu-Na Lee (Animal and Plant Quarantine Agency (S-2158) / Avian Influenza Research & Diagnostic Division)
EPI_ISL_408750	A/chicken/Korea/H1078/2014	A / H5N8	Asia / Korea, Republic of	Yu-Na Lee (Animal and Plant Quarantine Agency (S-2158) / Avian Influenza Research & Diagnostic Division)
EPI_ISL_408751	A/duck/Korea/H1111/2014	A / H5N8	Asia / Korea, Republic of	Yu-Na Lee (Animal and Plant Quarantine Agency (S-2158) / Avian Influenza Research & Diagnostic Division)
EPI_ISL_408752	A/duck/Korea/H1112/2014	A / H5N8	Asia / Korea, Republic of	Yu-Na Lee (Animal and Plant Quarantine Agency (S-2158) / Avian Influenza Research & Diagnostic Division)
EPI_ISL_408753	A/chicken/Korea/H1121/2014	A / H5N8	Asia / Korea, Republic of	Yu-Na Lee (Animal and Plant Quarantine Agency (S-2158) / Avian Influenza Research & Diagnostic Division)
EPI_ISL_408755	A/chicken/Korea/H1152/2014	A / H5N8	Asia / Korea, Republic of	Yu-Na Lee (Animal and Plant Quarantine Agency (S-2158) / Avian Influenza Research & Diagnostic Division)
EPI_ISL_408756	A/duck/Korea/H1161/2014	A / H5N8	Asia / Korea, Republic of	Yu-Na Lee (Animal and Plant Quarantine Agency (S-2158) / Avian Influenza Research & Diagnostic Division)
EPI_ISL_408757	A/duck/Korea/H1173/2014	A / H5N8	Asia / Korea, Republic of	Yu-Na Lee (Animal and Plant Quarantine Agency (S-2158) / Avian Influenza Research & Diagnostic Division)
EPI_ISL_408758	A/duck/Korea/H1174/2014	A / H5N8	Asia / Korea, Republic of	Yu-Na Lee (Animal and Plant Quarantine Agency (S-2158) / Avian Influenza Research & Diagnostic Division)
EPI_ISL_408759	A/chicken/Korea/H1202/2014	A / H5N8	Asia / Korea, Republic of	Yu-Na Lee (Animal and Plant Quarantine Agency (S-2158) / Avian Influenza Research & Diagnostic Division)
EPI_ISL_408760	A/chicken/Korea/H1205/2014	A / H5N8	Asia / Korea, Republic of	Yu-Na Lee (Animal and Plant Quarantine Agency (S-2158) / Avian Influenza Research & Diagnostic Division)
EPI_ISL_408761	A/duck/Korea/H1219/2014	A / H5N8	Asia / Korea, Republic of	Yu-Na Lee (Animal and Plant Quarantine Agency (S-2158) / Avian Influenza Research & Diagnostic Division)
EPI_ISL_408762	A/environment/Korea/H1220/2014	A / H5N8	Asia / Korea, Republic of	Yu-Na Lee (Animal and Plant Quarantine Agency (S-2158) / Avian Influenza Research & Diagnostic Division)
EPI_ISL_408763	A/chicken/Korea/H1230/2014	A / H5N8	Asia / Korea, Republic of	Yu-Na Lee (Animal and Plant Quarantine Agency (S-2158) / Avian Influenza Research & Diagnostic Division)
EPI_ISL_408764	A/duck/Korea/H1267/2014	A / H5N8	Asia / Korea, Republic of	Yu-Na Lee (Animal and Plant Quarantine Agency (S-2158) / Avian Influenza Research & Diagnostic Division)
EPI_ISL_408765	A/duck/Korea/H1275/2014	A / H5N8	Asia / Korea, Republic of	Yu-Na Lee (Animal and Plant Quarantine Agency (S-2158) / Avian Influenza Research & Diagnostic Division)
EPI_ISL_408766	A/chicken/Korea/H1289/2014	A / H5N8	Asia / Korea, Republic of	Yu-Na Lee (Animal and Plant Quarantine Agency (S-2158) / Avian Influenza Research & Diagnostic Division)
EPI_ISL_408767	A/chicken/Korea/H1250/2014	A / H5N8	Asia / Korea, Republic of	Yu-Na Lee (Animal and Plant Quarantine Agency (S-2158) / Avian Influenza Research & Diagnostic Division)
EPI_ISL_408768	A/environment/Korea/H1247/2014	A / H5N8	Asia / Korea, Republic of	Yu-Na Lee (Animal and Plant Quarantine Agency (S-2158) / Avian Influenza Research & Diagnostic Division)
EPI_ISL_408770	A/quail/Korea/H1329/2014	A / H5N8	Asia / Korea, Republic of	Yu-Na Lee (Animal and Plant Quarantine Agency (S-2158) / Avian Influenza Research & Diagnostic Division)
EPI_ISL_408779	A/duck/Korea/H1597/2014	A / H5N8	Asia / Korea, Republic of	Yu-Na Lee (Animal and Plant Quarantine Agency (S-2158) / Avian Influenza Research & Diagnostic Division)
EPI_ISL_408780	A/duck/Korea/H1613/2014	A / H5N8	Asia / Korea, Republic of	Yu-Na Lee (Animal and Plant Quarantine Agency (S-2158) / Avian Influenza Research & Diagnostic Division)
EPI_ISL_408786	A/duck/Korea/H1736/2014	A / H5N8	Asia / Korea, Republic of	Yu-Na Lee (Animal and Plant Quarantine Agency (S-2158) / Avian Influenza Research & Diagnostic Division)
EPI_ISL_408788	A/duck/Korea/H1740/2014	A / H5N8	Asia / Korea, Republic of	Yu-Na Lee (Animal and Plant Quarantine Agency (S-2158) / Avian Influenza Research & Diagnostic Division)
EPI_ISL_408789	A/duck/Korea/H1742/2014	A / H5N8	Asia / Korea, Republic of	Yu-Na Lee (Animal and Plant Quarantine Agency (S-2158) / Avian Influenza Research & Diagnostic Division)
EPI_ISL_408794	A/duck/Korea/H1757/2014	A / H5N8	Asia / Korea, Republic of	Yu-Na Lee (Animal and Plant Quarantine Agency (S-2158) / Avian Influenza Research & Diagnostic Division)
EPI_ISL_408795	A/duck/Korea/H1759/2014	A / H5N8	Asia / Korea, Republic of	Yu-Na Lee (Animal and Plant Quarantine Agency (S-2158) / Avian Influenza Research & Diagnostic Division)
EPI_ISL_408796	A/duck/Korea/H1760/2014	A / H5N8	Asia / Korea, Republic of	Yu-Na Lee (Animal and Plant Quarantine Agency (S-2158) / Avian Influenza Research & Diagnostic Division)
EPI_ISL_408797	A/duck/Korea/H1764/2014	A / H5N8	Asia / Korea, Republic of	Yu-Na Lee (Animal and Plant Quarantine Agency (S-2158) / Avian Influenza Research & Diagnostic Division)
EPI_ISL_408798	A/duck/Korea/H1765/2014	A / H5N8	Asia / Korea, Republic of	Yu-Na Lee (Animal and Plant Quarantine Agency (S-2158) / Avian Influenza Research & Diagnostic Division)
EPI_ISL_408799	A/duck/Korea/H1780/2014	A / H5N8	Asia / Korea, Republic of	Yu-Na Lee (Animal and Plant Quarantine Agency (S-2158) / Avian Influenza Research & Diagnostic Division)
EPI_ISL_408800	A/duck/Korea/H1798/2014	A / H5N8	Asia / Korea, Republic of	Yu-Na Lee (Animal and Plant Quarantine Agency (S-2158) / Avian Influenza Research & Diagnostic Division)
EPI_ISL_408802	A/Korean_native_chicken/Korea/H1804/2014	A / H5N8	Asia / Korea, Republic of	Yu-Na Lee (Animal and Plant Quarantine Agency (S-2158) / Avian Influenza Research & Diagnostic Division)
EPI_ISL_408803	A/duck/Korea/H1806/2014	A / H5N8	Asia / Korea, Republic of	Yu-Na Lee (Animal and Plant Quarantine Agency (S-2158) / Avian Influenza Research & Diagnostic Division)

EPI_ISL_408804	A/duck/Korea/H1813/2014	A / H5N8	Asia / Korea, Republic of	Yu-Na Lee (Animal and Plant Quarantine Agency (S-2158) / Avian Influenza Research & Diagnostic Division)
EPI_ISL_408809	A/duck/Korea/H1880/2014	A / H5N8	Asia / Korea, Republic of	Yu-Na Lee (Animal and Plant Quarantine Agency (S-2158) / Avian Influenza Research & Diagnostic Division)
EPI_ISL_408811	A/Korean_native_chicken/Korea/H1964/2014	A / H5N8	Asia / Korea, Republic of	Yu-Na Lee (Animal and Plant Quarantine Agency (S-2158) / Avian Influenza Research & Diagnostic Division)
EPI_ISL_408812	A/Korean_native_chicken/Korea/H2028/2014	A / H5N8	Asia / Korea, Republic of	Yu-Na Lee (Animal and Plant Quarantine Agency (S-2158) / Avian Influenza Research & Diagnostic Division)
EPI_ISL_408813	A/Korean_native_chicken/Korea/H2031/2014	A / H5N8	Asia / Korea, Republic of	Yu-Na Lee (Animal and Plant Quarantine Agency (S-2158) / Avian Influenza Research & Diagnostic Division)
EPI_ISL_408816	A/Korean_native_chicken/Korea/H2036/2014	A / H5N8	Asia / Korea, Republic of	Yu-Na Lee (Animal and Plant Quarantine Agency (S-2158) / Avian Influenza Research & Diagnostic Division)
EPI_ISL_408817	A/Korean_native_chicken/Korea/H2037/2014	A / H5N8	Asia / Korea, Republic of	Yu-Na Lee (Animal and Plant Quarantine Agency (S-2158) / Avian Influenza Research & Diagnostic Division)
EPI_ISL_408818	A/Korean_native_chicken/Korea/H2038/2014	A / H5N8	Asia / Korea, Republic of	Yu-Na Lee (Animal and Plant Quarantine Agency (S-2158) / Avian Influenza Research & Diagnostic Division)
EPI_ISL_408819	A/Korean_native_chicken/Korea/H2039/2014	A / H5N8	Asia / Korea, Republic of	Yu-Na Lee (Animal and Plant Quarantine Agency (S-2158) / Avian Influenza Research & Diagnostic Division)
EPI_ISL_408820	A/duck/Korea/H2075/2015	A / H5N8	Asia / Korea, Republic of	Yu-Na Lee (Animal and Plant Quarantine Agency (S-2158) / Avian Influenza Research & Diagnostic Division)
EPI_ISL_408821	A/environment/Korea/H2081/2015	A / H5N8	Asia / Korea, Republic of	Yu-Na Lee (Animal and Plant Quarantine Agency (S-2158) / Avian Influenza Research & Diagnostic Division)
EPI_ISL_408823	A/duck/Korea/H2088/2015	A / H5N8	Asia / Korea, Republic of	Yu-Na Lee (Animal and Plant Quarantine Agency (S-2158) / Avian Influenza Research & Diagnostic Division)
EPI_ISL_408824	A/environment/Korea/H2093/2015	A / H5N8	Asia / Korea, Republic of	Yu-Na Lee (Animal and Plant Quarantine Agency (S-2158) / Avian Influenza Research & Diagnostic Division)
EPI_ISL_408825	A/goose/Korea/H2095/2015	A / H5N8	Asia / Korea, Republic of	Yu-Na Lee (Animal and Plant Quarantine Agency (S-2158) / Avian Influenza Research & Diagnostic Division)
EPI_ISL_408826	A/Korean_native_chicken/Korea/H2098/2015	A / H5N8	Asia / Korea, Republic of	Yu-Na Lee (Animal and Plant Quarantine Agency (S-2158) / Avian Influenza Research & Diagnostic Division)
EPI_ISL_408827	A/duck/Korea/H2100/2015	A / H5N8	Asia / Korea, Republic of	Yu-Na Lee (Animal and Plant Quarantine Agency (S-2158) / Avian Influenza Research & Diagnostic Division)
EPI_ISL_408828	A/chicken/Korea/H2103/2015	A / H5N8	Asia / Korea, Republic of	Yu-Na Lee (Animal and Plant Quarantine Agency (S-2158) / Avian Influenza Research & Diagnostic Division)
EPI_ISL_408829	A/duck/Korea/H2104/2015	A / H5N8	Asia / Korea, Republic of	Yu-Na Lee (Animal and Plant Quarantine Agency (S-2158) / Avian Influenza Research & Diagnostic Division)
EPI_ISL_408831	A/duck/Korea/H2117/2015	A / H5N8	Asia / Korea, Republic of	Yu-Na Lee (Animal and Plant Quarantine Agency (S-2158) / Avian Influenza Research & Diagnostic Division)
EPI_ISL_408832	A/duck/Korea/H2118/2015	A / H5N8	Asia / Korea, Republic of	Yu-Na Lee (Animal and Plant Quarantine Agency (S-2158) / Avian Influenza Research & Diagnostic Division)
EPI_ISL_408833	A/grey_heron/Korea/H2119/2015	A / H5N8	Asia / Korea, Republic of	Yu-Na Lee (Animal and Plant Quarantine Agency (S-2158) / Avian Influenza Research & Diagnostic Division)
EPI_ISL_408834	A/spot_billed_duck/Korea/H2126/2015	A / H5N8	Asia / Korea, Republic of	Yu-Na Lee (Animal and Plant Quarantine Agency (S-2158) / Avian Influenza Research & Diagnostic Division)
EPI_ISL_408835	A/duck/Korea/H2131/2015	A / H5N8	Asia / Korea, Republic of	Yu-Na Lee (Animal and Plant Quarantine Agency (S-2158) / Avian Influenza Research & Diagnostic Division)
EPI_ISL_408836	A/duck/Korea/H2147/2015	A / H5N8	Asia / Korea, Republic of	Yu-Na Lee (Animal and Plant Quarantine Agency (S-2158) / Avian Influenza Research & Diagnostic Division)
EPI_ISL_408837	A/duck/Korea/H2148/2015	A / H5N8	Asia / Korea, Republic of	Yu-Na Lee (Animal and Plant Quarantine Agency (S-2158) / Avian Influenza Research & Diagnostic Division)
EPI_ISL_408838	A/duck/Korea/H2161/2015	A / H5N8	Asia / Korea, Republic of	Yu-Na Lee (Animal and Plant Quarantine Agency (S-2158) / Avian Influenza Research & Diagnostic Division)
EPI_ISL_408839	A/chicken/Korea/H2181/2015	A / H5N8	Asia / Korea, Republic of	Yu-Na Lee (Animal and Plant Quarantine Agency (S-2158) / Avian Influenza Research & Diagnostic Division)
EPI_ISL_408840	A/duck/Korea/H2190/2015	A / H5N8	Asia / Korea, Republic of	Yu-Na Lee (Animal and Plant Quarantine Agency (S-2158) / Avian Influenza Research & Diagnostic Division)
EPI_ISL_408841	A/chicken/Korea/H2192/2015	A / H5N8	Asia / Korea, Republic of	Yu-Na Lee (Animal and Plant Quarantine Agency (S-2158) / Avian Influenza Research & Diagnostic Division)
EPI_ISL_408843	A/chicken/Korea/H2205/2015	A / H5N8	Asia / Korea, Republic of	Yu-Na Lee (Animal and Plant Quarantine Agency (S-2158) / Avian Influenza Research & Diagnostic Division)
EPI_ISL_408844	A/duck/Korea/H2206/2015	A / H5N8	Asia / Korea, Republic of	Yu-Na Lee (Animal and Plant Quarantine Agency (S-2158) / Avian Influenza Research & Diagnostic Division)
EPI_ISL_408845	A/gadwall/Korea/H2209/2015	A / H5N8	Asia / Korea, Republic of	Yu-Na Lee (Animal and Plant Quarantine Agency (S-2158) / Avian Influenza Research & Diagnostic Division)
EPI_ISL_408846	A/Eurasian_wigeon/Korea/H2210/2015	A / H5N8	Asia / Korea, Republic of	Yu-Na Lee (Animal and Plant Quarantine Agency (S-2158) / Avian Influenza Research & Diagnostic Division)
EPI_ISL_408847	A/bean_goose/Korea/H2211/2015	A / H5N8	Asia / Korea, Republic of	Yu-Na Lee (Animal and Plant Quarantine Agency (S-2158) / Avian Influenza Research & Diagnostic Division)
EPI_ISL_408848	A/duck/Korea/H2230/2015	A / H5N8	Asia / Korea, Republic of	Yu-Na Lee (Animal and Plant Quarantine Agency (S-2158) / Avian Influenza Research & Diagnostic Division)
EPI_ISL_408849	A/duck/Korea/H2239/2015	A / H5N8	Asia / Korea, Republic of	Yu-Na Lee (Animal and Plant Quarantine Agency (S-2158) / Avian Influenza Research & Diagnostic Division)
EPI_ISL_408850	A/duck/Korea/H2253/2015	A / H5N8	Asia / Korea, Republic of	Yu-Na Lee (Animal and Plant Quarantine Agency (S-2158) / Avian Influenza Research & Diagnostic Division)
EPI_ISL_408851	A/common_teal/Korea/H2261/2015	A / H5N8	Asia / Korea, Republic of	Yu-Na Lee (Animal and Plant Quarantine Agency (S-2158) / Avian Influenza Research & Diagnostic Division)
EPI_ISL_408852	A/duck/Korea/H2263/2015	A / H5N8	Asia / Korea, Republic of	Yu-Na Lee (Animal and Plant Quarantine Agency (S-2158) / Avian Influenza Research & Diagnostic Division)
EPI_ISL_408853	A/duck/Korea/H2264/2015	A / H5N8	Asia / Korea, Republic of	Yu-Na Lee (Animal and Plant Quarantine Agency (S-2158) / Avian Influenza Research & Diagnostic Division)
EPI_ISL_408855	A/duck/Korea/H2267/2015	A / H5N8	Asia / Korea, Republic of	Yu-Na Lee (Animal and Plant Quarantine Agency (S-2158) / Avian Influenza Research & Diagnostic Division)
EPI_ISL_408856	A/duck/Korea/H2268/2015	A / H5N8	Asia / Korea, Republic of	Yu-Na Lee (Animal and Plant Quarantine Agency (S-2158) / Avian Influenza Research & Diagnostic Division)
EPI_ISL_408857	A/duck/Korea/H2269/2015	A / H5N8	Asia / Korea, Republic of	Yu-Na Lee (Animal and Plant Quarantine Agency (S-2158) / Avian Influenza Research & Diagnostic Division)
EPI_ISL_408858	A/duck/Korea/H2273/2015	A / H5N8	Asia / Korea, Republic of	Yu-Na Lee (Animal and Plant Quarantine Agency (S-2158) / Avian Influenza Research & Diagnostic Division)
EPI_ISL_408860	A/duck/Korea/H2279/2015	A / H5N8	Asia / Korea, Republic of	Yu-Na Lee (Animal and Plant Quarantine Agency (S-2158) / Avian Influenza Research & Diagnostic Division)
EPI_ISL_408861	A/duck/Korea/H2280/2015	A / H5N8	Asia / Korea, Republic of	Yu-Na Lee (Animal and Plant Quarantine Agency (S-2158) / Avian Influenza Research & Diagnostic Division)
EPI_ISL_408863	A/duck/Korea/H2294/2015	A / H5N8	Asia / Korea, Republic of	Yu-Na Lee (Animal and Plant Quarantine Agency (S-2158) / Avian Influenza Research & Diagnostic Division)
EPI_ISL_408864	A/duck/Korea/H2300/2015	A / H5N8	Asia / Korea, Republic of	Yu-Na Lee (Animal and Plant Quarantine Agency (S-2158) / Avian Influenza Research & Diagnostic Division)
EPI_ISL_408866	A/environment/Korea/H2305/2015	A / H5N8	Asia / Korea, Republic of	Yu-Na Lee (Animal and Plant Quarantine Agency (S-2158) / Avian Influenza Research & Diagnostic Division)
EPI_ISL_408867	A/duck/Korea/H2311/2015	A / H5N8	Asia / Korea, Republic of	Yu-Na Lee (Animal and Plant Quarantine Agency (S-2158) / Avian Influenza Research & Diagnostic Division)
EPI_ISL_408868	A/duck/Korea/H2312/2015	A / H5N8	Asia / Korea, Republic of	Yu-Na Lee (Animal and Plant Quarantine Agency (S-2158) / Avian Influenza Research & Diagnostic Division)
EPI_ISL_408869	A/duck/Korea/H2319/2015	A / H5N8	Asia / Korea, Republic of	Yu-Na Lee (Animal and Plant Quarantine Agency (S-2158) / Avian Influenza Research & Diagnostic Division)
EPI_ISL_408870	A/duck/Korea/H2320/2015	A / H5N8	Asia / Korea, Republic of	Yu-Na Lee (Animal and Plant Quarantine Agency (S-2158) / Avian Influenza Research & Diagnostic Division)
EPI_ISL_408872	A/chicken/Korea/H2357/2015	A / H5N8	Asia / Korea, Republic of	Yu-Na Lee (Animal and Plant Quarantine Agency (S-2158) / Avian Influenza Research & Diagnostic Division)
EPI_ISL_408873	A/duck/Korea/H2358/2015	A / H5N8	Asia / Korea, Republic of	Yu-Na Lee (Animal and Plant Quarantine Agency (S-2158) / Avian Influenza Research & Diagnostic Division)
EPI_ISL_408874	A/duck/Korea/H2363/2015	A / H5N8	Asia / Korea, Republic of	Yu-Na Lee (Animal and Plant Quarantine Agency (S-2158) / Avian Influenza Research & Diagnostic Division)
EPI_ISL_408875	A/environment/Korea/H2369/2015	A / H5N8	Asia / Korea, Republic of	Yu-Na Lee (Animal and Plant Quarantine Agency (S-2158) / Avian Influenza Research & Diagnostic Division)
EPI_ISL_408877	A/duck/Korea/H2388/2015	A / H5N8	Asia / Korea, Republic of	Yu-Na Lee (Animal and Plant Quarantine Agency (S-2158) / Avian Influenza Research & Diagnostic Division)
EPI_ISL_408878	A/duck/Korea/H2391/2015	A / H5N8	Asia / Korea, Republic of	Yu-Na Lee (Animal and Plant Quarantine Agency (S-2158) / Avian Influenza Research & Diagnostic Division)
EPI_ISL_408879	A/duck/Korea/H2392/2015	A / H5N8	Asia / Korea, Republic of	Yu-Na Lee (Animal and Plant Quarantine Agency (S-2158) / Avian Influenza Research & Diagnostic Division)
EPI_ISL_408881	A/duck/Korea/H2394/2015	A / H5N8	Asia / Korea, Republic of	Yu-Na Lee (Animal and Plant Quarantine Agency (S-2158) / Avian Influenza Research & Diagnostic Division)
EPI_ISL_408882	A/duck/Korea/H2395/2015	A / H5N8	Asia / Korea, Republic of	Yu-Na Lee (Animal and Plant Quarantine Agency (S-2158) / Avian Influenza Research & Diagnostic Division)
EPI_ISL_408883	A/duck/Korea/H2397/2015	A / H5N8	Asia / Korea, Republic of	Yu-Na Lee (Animal and Plant Quarantine Agency (S-2158) / Avian Influenza Research & Diagnostic Division)
EPI_ISL_408884	A/duck/Korea/H2399/2015	A / H5N8	Asia / Korea, Republic of	Yu-Na Lee (Animal and Plant Quarantine Agency (S-2158) / Avian Influenza Research & Diagnostic Division)
EPI_ISL_408886	A/duck/Korea/H2401/2015	A / H5N8	Asia / Korea, Republic of	Yu-Na Lee (Animal and Plant Quarantine Agency (S-2158) / Avian Influenza Research & Diagnostic Division)
EPI_ISL_408887	A/duck/Korea/H2402/2015	A / H5N8	Asia / Korea, Republic of	Yu-Na Lee (Animal and Plant Quarantine Agency (S-2158) / Avian Influenza Research & Diagnostic Division)
EPI_ISL_408889	A/duck/Korea/H2410/2015	A / H5N8	Asia / Korea, Republic of	Yu-Na Lee (Animal and Plant Quarantine Agency (S-2158) / Avian Influenza Research & Diagnostic Division)
EPI_ISL_408890	A/duck/Korea/H2411/2015	A / H5N8	Asia / Korea, Republic of	Yu-Na Lee (Animal and Plant Quarantine Agency (S-2158) / Avian Influenza Research & Diagnostic Division)
EPI_ISL_408891	A/duck/Korea/H2412/2015	A / H5N8	Asia / Korea, Republic of	Yu-Na Lee (Animal and Plant Quarantine Agency (S-2158) / Avian Influenza Research & Diagnostic Division)
EPI_ISL_408892	A/duck/Korea/H2426/2015	A / H5N8	Asia / Korea, Republic of	Yu-Na Lee (Animal and Plant Quarantine Agency (S-2158) / Avian Influenza Research & Diagnostic Division)
EPI_ISL_408893	A/duck/Korea/H2435/2015	A / H5N8	Asia / Korea, Republic of	Yu-Na Lee (Animal and Plant Quarantine Agency (S-2158) / Avian Influenza Research & Diagnostic Division)
EPI_ISL_408894	A/duck/Korea/H2436/2015	A / H5N8	Asia / Korea, Republic of	Yu-Na Lee (Animal and Plant Quarantine Agency (S-2158) / Avian Influenza Research & Diagnostic Division)

EPI_ISL_408895	A/duck/Korea/H2441/2015	A / H5N8	Asia / Korea, Republic of	Yu-Na Lee (Animal and Plant Quarantine Agency (S-2158) / Avian Influenza Research & Diagnostic Division)
EPI_ISL_408896	A/duck/Korea/H2442/2015	A / H5N8	Asia / Korea, Republic of	Yu-Na Lee (Animal and Plant Quarantine Agency (S-2158) / Avian Influenza Research & Diagnostic Division)
EPI_ISL_408897	A/duck/Korea/H2444/2015	A / H5N8	Asia / Korea, Republic of	Yu-Na Lee (Animal and Plant Quarantine Agency (S-2158) / Avian Influenza Research & Diagnostic Division)
EPI_ISL_408898	A/duck/Korea/H2445/2015	A / H5N8	Asia / Korea, Republic of	Yu-Na Lee (Animal and Plant Quarantine Agency (S-2158) / Avian Influenza Research & Diagnostic Division)
EPI_ISL_408899	A/duck/Korea/H2446/2015	A / H5N8	Asia / Korea, Republic of	Yu-Na Lee (Animal and Plant Quarantine Agency (S-2158) / Avian Influenza Research & Diagnostic Division)
EPI_ISL_408900	A/duck/Korea/H2447/2015	A / H5N8	Asia / Korea, Republic of	Yu-Na Lee (Animal and Plant Quarantine Agency (S-2158) / Avian Influenza Research & Diagnostic Division)
EPI_ISL_408901	A/duck/Korea/H2449/2015	A / H5N8	Asia / Korea, Republic of	Yu-Na Lee (Animal and Plant Quarantine Agency (S-2158) / Avian Influenza Research & Diagnostic Division)
EPI_ISL_408902	A/duck/Korea/H2454/2015	A / H5N8	Asia / Korea, Republic of	Yu-Na Lee (Animal and Plant Quarantine Agency (S-2158) / Avian Influenza Research & Diagnostic Division)
EPI_ISL_408903	A/duck/Korea/H2455/2015	A / H5N8	Asia / Korea, Republic of	Yu-Na Lee (Animal and Plant Quarantine Agency (S-2158) / Avian Influenza Research & Diagnostic Division)
EPI_ISL_408904	A/duck/Korea/H2459/2015	A / H5N8	Asia / Korea, Republic of	Yu-Na Lee (Animal and Plant Quarantine Agency (S-2158) / Avian Influenza Research & Diagnostic Division)
EPI_ISL_408905	A/duck/Korea/H2460/2015	A / H5N8	Asia / Korea, Republic of	Yu-Na Lee (Animal and Plant Quarantine Agency (S-2158) / Avian Influenza Research & Diagnostic Division)
EPI_ISL_408906	A/duck/Korea/H2462/2015	A / H5N8	Asia / Korea, Republic of	Yu-Na Lee (Animal and Plant Quarantine Agency (S-2158) / Avian Influenza Research & Diagnostic Division)
EPI_ISL_408907	A/duck/Korea/H2465/2015	A / H5N8	Asia / Korea, Republic of	Yu-Na Lee (Animal and Plant Quarantine Agency (S-2158) / Avian Influenza Research & Diagnostic Division)
EPI_ISL_408908	A/duck/Korea/H2466/2015	A / H5N8	Asia / Korea, Republic of	Yu-Na Lee (Animal and Plant Quarantine Agency (S-2158) / Avian Influenza Research & Diagnostic Division)
EPI_ISL_408909	A/duck/Korea/H2467/2015	A / H5N8	Asia / Korea, Republic of	Yu-Na Lee (Animal and Plant Quarantine Agency (S-2158) / Avian Influenza Research & Diagnostic Division)
EPI_ISL_408910	A/duck/Korea/H2469/2015	A / H5N8	Asia / Korea, Republic of	Yu-Na Lee (Animal and Plant Quarantine Agency (S-2158) / Avian Influenza Research & Diagnostic Division)
EPI_ISL_408911	A/duck/Korea/H2474/2015	A / H5N8	Asia / Korea, Republic of	Yu-Na Lee (Animal and Plant Quarantine Agency (S-2158) / Avian Influenza Research & Diagnostic Division)
EPI_ISL_408912	A/duck/Korea/H2475/2015	A / H5N8	Asia / Korea, Republic of	Yu-Na Lee (Animal and Plant Quarantine Agency (S-2158) / Avian Influenza Research & Diagnostic Division)
EPI_ISL_408913	A/duck/Korea/H2484/2015	A / H5N8	Asia / Korea, Republic of	Yu-Na Lee (Animal and Plant Quarantine Agency (S-2158) / Avian Influenza Research & Diagnostic Division)
EPI_ISL_408914	A/duck/Korea/H2485/2015	A / H5N8	Asia / Korea, Republic of	Yu-Na Lee (Animal and Plant Quarantine Agency (S-2158) / Avian Influenza Research & Diagnostic Division)
EPI_ISL_408915	A/chicken/Korea/H2486/2015	A / H5N8	Asia / Korea, Republic of	Yu-Na Lee (Animal and Plant Quarantine Agency (S-2158) / Avian Influenza Research & Diagnostic Division)
EPI_ISL_408917	A/chicken/Korea/H2497/2015	A / H5N8	Asia / Korea, Republic of	Yu-Na Lee (Animal and Plant Quarantine Agency (S-2158) / Avian Influenza Research & Diagnostic Division)
EPI_ISL_408918	A/duck/Korea/H2504/2015	A / H5N8	Asia / Korea, Republic of	Yu-Na Lee (Animal and Plant Quarantine Agency (S-2158) / Avian Influenza Research & Diagnostic Division)
EPI_ISL_408919	A/chicken/Korea/H2513/2015	A / H5N8	Asia / Korea, Republic of	Yu-Na Lee (Animal and Plant Quarantine Agency (S-2158) / Avian Influenza Research & Diagnostic Division)
EPI_ISL_408920	A/chicken/Korea/H2514/2015	A / H5N8	Asia / Korea, Republic of	Yu-Na Lee (Animal and Plant Quarantine Agency (S-2158) / Avian Influenza Research & Diagnostic Division)
EPI_ISL_408922	A/chicken/Korea/H2519/2015	A / H5N8	Asia / Korea, Republic of	Yu-Na Lee (Animal and Plant Quarantine Agency (S-2158) / Avian Influenza Research & Diagnostic Division)
EPI_ISL_408923	A/duck/Korea/H2529/2015	A / H5N8	Asia / Korea, Republic of	Yu-Na Lee (Animal and Plant Quarantine Agency (S-2158) / Avian Influenza Research & Diagnostic Division)
EPI_ISL_408925	A/chicken/Korea/H2541/2015	A / H5N8	Asia / Korea, Republic of	Yu-Na Lee (Animal and Plant Quarantine Agency (S-2158) / Avian Influenza Research & Diagnostic Division)
EPI_ISL_408926	A/chicken/Korea/H2551/2015	A / H5N8	Asia / Korea, Republic of	Yu-Na Lee (Animal and Plant Quarantine Agency (S-2158) / Avian Influenza Research & Diagnostic Division)
EPI_ISL_408928	A/chicken/Korea/H2555/2015	A / H5N8	Asia / Korea, Republic of	Yu-Na Lee (Animal and Plant Quarantine Agency (S-2158) / Avian Influenza Research & Diagnostic Division)
EPI_ISL_408929	A/chicken/Korea/H2560/2015	A / H5N8	Asia / Korea, Republic of	Yu-Na Lee (Animal and Plant Quarantine Agency (S-2158) / Avian Influenza Research & Diagnostic Division)
EPI_ISL_408930	A/duck/Korea/H2564/2015	A / H5N8	Asia / Korea, Republic of	Yu-Na Lee (Animal and Plant Quarantine Agency (S-2158) / Avian Influenza Research & Diagnostic Division)
EPI_ISL_408932	A/chicken/Korea/H2568/2015	A / H5N8	Asia / Korea, Republic of	Yu-Na Lee (Animal and Plant Quarantine Agency (S-2158) / Avian Influenza Research & Diagnostic Division)
EPI_ISL_408933	A/chicken/Korea/H2570/2015	A / H5N8	Asia / Korea, Republic of	Yu-Na Lee (Animal and Plant Quarantine Agency (S-2158) / Avian Influenza Research & Diagnostic Division)
EPI_ISL_408934	A/chicken/Korea/H2573/2015	A / H5N8	Asia / Korea, Republic of	Yu-Na Lee (Animal and Plant Quarantine Agency (S-2158) / Avian Influenza Research & Diagnostic Division)
EPI_ISL_408935	A/chicken/Korea/H2580/2015	A / H5N8	Asia / Korea, Republic of	Yu-Na Lee (Animal and Plant Quarantine Agency (S-2158) / Avian Influenza Research & Diagnostic Division)
EPI_ISL_408936	A/chicken/Korea/H2584/2015	A / H5N8	Asia / Korea, Republic of	Yu-Na Lee (Animal and Plant Quarantine Agency (S-2158) / Avian Influenza Research & Diagnostic Division)
EPI_ISL_408938	A/chicken/Korea/H2596/2015	A / H5N8	Asia / Korea, Republic of	Yu-Na Lee (Animal and Plant Quarantine Agency (S-2158) / Avian Influenza Research & Diagnostic Division)
EPI_ISL_408940	A/chicken/Korea/H2599/2015	A / H5N8	Asia / Korea, Republic of	Yu-Na Lee (Animal and Plant Quarantine Agency (S-2158) / Avian Influenza Research & Diagnostic Division)
EPI_ISL_408941	A/chicken/Korea/H2600/2015	A / H5N8	Asia / Korea, Republic of	Yu-Na Lee (Animal and Plant Quarantine Agency (S-2158) / Avian Influenza Research & Diagnostic Division)
EPI_ISL_408943	A/duck/Korea/H2613/2015	A / H5N8	Asia / Korea, Republic of	Yu-Na Lee (Animal and Plant Quarantine Agency (S-2158) / Avian Influenza Research & Diagnostic Division)
EPI_ISL_408947	A/duck/Korea/H2629/2015	A / H5N8	Asia / Korea, Republic of	Yu-Na Lee (Animal and Plant Quarantine Agency (S-2158) / Avian Influenza Research & Diagnostic Division)
EPI_ISL_408948	A/duck/Korea/H2631/2015	A / H5N8	Asia / Korea, Republic of	Yu-Na Lee (Animal and Plant Quarantine Agency (S-2158) / Avian Influenza Research & Diagnostic Division)
EPI_ISL_408955	A/environment/Korea/H2833/2015	A / H5N8	Asia / Korea, Republic of	Yu-Na Lee (Animal and Plant Quarantine Agency (S-2158) / Avian Influenza Research & Diagnostic Division)
EPI_ISL_408962	A/duck/Korea/H3105/2015	A / H5N8	Asia / Korea, Republic of	Yu-Na Lee (Animal and Plant Quarantine Agency (S-2158) / Avian Influenza Research & Diagnostic Division)
EPI_ISL_408966	A/duck/Korea/H0088/2014	A / H5N8	Asia / Korea, Republic of	Yu-Na Lee (Animal and Plant Quarantine Agency (S-2158) / Avian Influenza Research & Diagnostic Division)
EPI_ISL_408967	A/chicken/Korea/H0453/2014	A / H5N8	Asia / Korea, Republic of	Yu-Na Lee (Animal and Plant Quarantine Agency (S-2158) / Avian Influenza Research & Diagnostic Division)
EPI_ISL_408969	A/duck/Korea/H1592/2014	A / H5N8	Asia / Korea, Republic of	Yu-Na Lee (Animal and Plant Quarantine Agency (S-2158) / Avian Influenza Research & Diagnostic Division)
EPI_ISL_408970	A/duck/Korea/H1750/2014	A / H5N8	Asia / Korea, Republic of	Yu-Na Lee (Animal and Plant Quarantine Agency (S-2158) / Avian Influenza Research & Diagnostic Division)
EPI_ISL_408971	A/duck/Korea/H1763/2014	A / H5N8	Asia / Korea, Republic of	Yu-Na Lee (Animal and Plant Quarantine Agency (S-2158) / Avian Influenza Research & Diagnostic Division)
EPI_ISL_408972	A/duck/Korea/H2240/2015	A / H5N8	Asia / Korea, Republic of	Yu-Na Lee (Animal and Plant Quarantine Agency (S-2158) / Avian Influenza Research & Diagnostic Division)
EPI_ISL_408973	A/chicken/Korea/H2295/2015	A / H5N8	Asia / Korea, Republic of	Yu-Na Lee (Animal and Plant Quarantine Agency (S-2158) / Avian Influenza Research & Diagnostic Division)
EPI_ISL_410213	A/ostrich/Korea/H0829/2014	A / H5N8	Asia / Korea, Republic of	Yu-Na Lee (Animal and Plant Quarantine Agency (S-2158) / Avian Influenza Research & Diagnostic Division)
EPI_ISL_410291	A/chicken/Germany-BW/AI00049/2020	A / H5N8	Europe / Germany / Baden-Wuerttemberg / Regierungsbezirk Karlsruhe / Hohenlohekreis	Jacqueline King (Friedrich-Loeffler-Institut)
EPI_ISL_412984	A/chicken/Iran/17RS654-01/2016	A / H5N8	Asia / Iran, Islamic Republic of	Bianca Zecchin (Istituto Zooprofilattico Sperimentale Delle Venezie)
EPI_ISL_412986	A/chicken/Iran/17RS654-03/2016	A / H5N8	Asia / Iran, Islamic Republic of	Bianca Zecchin (Istituto Zooprofilattico Sperimentale Delle Venezie)
EPI_ISL_412987	A/chicken/Iran/17RS654-08/2016	A / H5N8	Asia / Iran, Islamic Republic of	Bianca Zecchin (Istituto Zooprofilattico Sperimentale Delle Venezie)
EPI_ISL_412988	A/chicken/Iran/17RS654-15/2017	A / H5N8	Asia / Iran, Islamic Republic of	Bianca Zecchin (Istituto Zooprofilattico Sperimentale Delle Venezie)
EPI_ISL_412989	A/chicken/Iran/17RS654-26/2017	A / H5N8	Asia / Iran, Islamic Republic of	Bianca Zecchin (Istituto Zooprofilattico Sperimentale Delle Venezie)
EPI_ISL_412990	A/chicken/Iran/17RS654-28/2016	A / H5N8	Asia / Iran, Islamic Republic of	Bianca Zecchin (Istituto Zooprofilattico Sperimentale Delle Venezie)
EPI_ISL_412991	A/chicken/Iran/17RS654-29/2017	A / H5N8	Asia / Iran, Islamic Republic of	Bianca Zecchin (Istituto Zooprofilattico Sperimentale Delle Venezie)
EPI_ISL_412992	A/chicken/Iran/17RS654-34/2017	A / H5N8	Asia / Iran, Islamic Republic of	Bianca Zecchin (Istituto Zooprofilattico Sperimentale Delle Venezie)
EPI_ISL_412994	A/chicken/Iran/18VIR2027-01/2017	A / H5N8	Asia / Iran, Islamic Republic of	Bianca Zecchin (Istituto Zooprofilattico Sperimentale Delle Venezie)
EPI_ISL_412995	A/chicken/Iran/18VIR2027-02/2017	A / H5N8	Asia / Iran, Islamic Republic of	Bianca Zecchin (Istituto Zooprofilattico Sperimentale Delle Venezie)
EPI_ISL_412996	A/chicken/Iran/18VIR2027-03/2017	A / H5N8	Asia / Iran, Islamic Republic of	Bianca Zecchin (Istituto Zooprofilattico Sperimentale Delle Venezie)
EPI_ISL_412997	A/chicken/Iran/18VIR2027-04/2017	A / H5N8	Asia / Iran, Islamic Republic of	Bianca Zecchin (Istituto Zooprofilattico Sperimentale Delle Venezie)
EPI_ISL_412998	A/chicken/Iran/18VIR2027-05/2017	A / H5N8	Asia / Iran, Islamic Republic of	Bianca Zecchin (Istituto Zooprofilattico Sperimentale Delle Venezie)
EPI_ISL_412999	A/chicken/Iran/18VIR2027-06/2017	A / H5N8	Asia / Iran, Islamic Republic of	Bianca Zecchin (Istituto Zooprofilattico Sperimentale Delle Venezie)
EPI_ISL_413000	A/chicken/Iran/18VIR2027-07/2017	A / H5N8	Asia / Iran, Islamic Republic of	Bianca Zecchin (Istituto Zooprofilattico Sperimentale Delle Venezie)
EPI_ISL_413001	A/chicken/Iran/18VIR2027-08/2017	A / H5N8	Asia / Iran, Islamic Republic of	Bianca Zecchin (Istituto Zooprofilattico Sperimentale Delle Venezie)
EPI_ISL_413002	A/chicken/Iran/18VIR2027-09/2017	A / H5N8	Asia / Iran, Islamic Republic of	Bianca Zecchin (Istituto Zooprofilattico Sperimentale Delle Venezie)
EPI_ISL_413003	A/chicken/Iran/18VIR2027-10/2017	A / H5N8	Asia / Iran, Islamic Republic of	Bianca Zecchin (Istituto Zooprofilattico Sperimentale Delle Venezie)

EPI_ISL_413005	A/chicken/Iran/18VIR2027-14/2017	A / H5N8	Asia / Iran, Islamic Republic of	Bianca Zecchin (Istituto Zooprofilattico Sperimentale Delle Venezie)
EPI_ISL_413006	A/chicken/Iran/18VIR2027-15/2017	A / H5N8	Asia / Iran, Islamic Republic of	Bianca Zecchin (Istituto Zooprofilattico Sperimentale Delle Venezie)
EPI_ISL_413008	A/flamingo/Iran/17RS654-18/2016	A / H5N8	Asia / Iran, Islamic Republic of	Bianca Zecchin (Istituto Zooprofilattico Sperimentale Delle Venezie)
EPI_ISL_413009	A/gadwall/Iran/18VIR2027-20/2018	A / H5N6	Asia / Iran, Islamic Republic of	Bianca Zecchin (Istituto Zooprofilattico Sperimentale Delle Venezie)
EPI_ISL_413010	A/little_grebe/Iran/17RS654-10/2016	A / H5N8	Asia / Iran, Islamic Republic of	Bianca Zecchin (Istituto Zooprofilattico Sperimentale Delle Venezie)
EPI_ISL_413011	A/wild_bird/Iran/17RS654-24/2016	A / H5N8	Asia / Iran, Islamic Republic of	Bianca Zecchin (Istituto Zooprofilattico Sperimentale Delle Venezie)
EPI_ISL_413225	A/Mallard/Republic of Georgia/1/2015	A / H5N8	Asia / Georgia	Maria Johanna Poen (Erasmus Medical Center / Department of Virology)
EPI_ISL_413226	A/Mallard/Republic of Georgia/2/2015	A / H5N8	Asia / Georgia	Maria Johanna Poen (Erasmus Medical Center / Department of Virology)
EPI_ISL_415197	A/chicken/Germany-SN/AI00276/2020	A / H5N8	Europe / Germany / Saxony / Regierungsbezirk Leipzig / Leipzig	Jacqueline King (Friedrich-Loeffler-Institut)
EPI_ISL_417414	A/buzzard/Germany-SN/AI00285/2020	A / H5N8	Europe / Germany / Saxony	Jacqueline King (Friedrich-Loeffler-Institut)
EPI_ISL_417415	A/turkey/Germany-NI/AI00334/2020	A / H5N8	Europe / Germany / Lower Saxony / Aurich	Jacqueline King (Friedrich-Loeffler-Institut)
EPI_ISL_418169	A/Whooper swan/Xinjiang/1/2020	A / H5N6	Asia / China / Xinjiang	Yanbing Li (Harbin Veterinary Research Institute (CAAS) / Ministry of Agriculture)
EPI_ISL_418170	A/Whooper swan/Xinjiang/2/2020	A / H5N6	Asia / China / Xinjiang	Yanbing Li (Harbin Veterinary Research Institute (CAAS) / Ministry of Agriculture)
EPI_ISL_418171	A/Whooper swan/Xinjiang/3/2020	A / H5N6	Asia / China / Xinjiang	Yanbing Li (Harbin Veterinary Research Institute (CAAS) / Ministry of Agriculture)
EPI_ISL_418172	A/Mute swan/Xinjiang/4/2020	A / H5N6	Asia / China / Xinjiang	Yanbing Li (Harbin Veterinary Research Institute (CAAS) / Ministry of Agriculture)
EPI_ISL_418173	A/Mute swan/Xinjiang/5/2020	A / H5N6	Asia / China / Xinjiang	Yanbing Li (Harbin Veterinary Research Institute (CAAS) / Ministry of Agriculture)
EPI_ISL_418174	A/Whooper swan/Xinjiang/6/2020	A / H5N6	Asia / China / Xinjiang	Yanbing Li (Harbin Veterinary Research Institute (CAAS) / Ministry of Agriculture)
EPI_ISL_418175	A/Whooper swan/Xinjiang/7/2020	A / H5N6	Asia / China / Xinjiang	Yanbing Li (Harbin Veterinary Research Institute (CAAS) / Ministry of Agriculture)
EPI_ISL_418176	A/Whooper swan/Xinjiang/8/2020	A / H5N6	Asia / China / Xinjiang	Yanbing Li (Harbin Veterinary Research Institute (CAAS) / Ministry of Agriculture)
EPI_ISL_418177	A/Whooper swan/Xinjiang/9/2020	A / H5N6	Asia / China / Xinjiang	Yanbing Li (Harbin Veterinary Research Institute (CAAS) / Ministry of Agriculture)
EPI_ISL_418178	A/Whooper swan/Xinjiang/10/2020	A / H5N6	Asia / China / Xinjiang	Yanbing Li (Harbin Veterinary Research Institute (CAAS) / Ministry of Agriculture)
EPI_ISL_418179	A/Whooper swan/Xinjiang/11/2020	A / H5N6	Asia / China / Xinjiang	Yanbing Li (Harbin Veterinary Research Institute (CAAS) / Ministry of Agriculture)
EPI_ISL_418180	A/Whooper swan/Xinjiang/12/2020	A / H5N6	Asia / China / Xinjiang	Yanbing Li (Harbin Veterinary Research Institute (CAAS) / Ministry of Agriculture)
EPI_ISL_418181	A/Whooper swan/Xinjiang/13/2020	A / H5N6	Asia / China / Xinjiang	Yanbing Li (Harbin Veterinary Research Institute (CAAS) / Ministry of Agriculture)
EPI_ISL_418266	A/turkey/Czech Republic/3071/2020	A / H5N8	Europe / Czech Republic / Pardubicky kraj / Okres Pardubice / Slepotice, Moravany	Alexander Nagy (State Veterinary Institute Prague)
EPI_ISL_419212	A/chicken/Bulgaria/Dobrich/12-1/2018	A / H5N8	Europe / Bulgaria / Oblast Dobrich	Divya Venkatesh (University of Cambridge / Zoology)
EPI_ISL_419220	A/turkey/Hungary/1020_20VIR749-1/2020	A / H5N8	Europe / Hungary	Ambra Pastori (Istituto Zooprofilattico Sperimentale Delle Venezie)
EPI_ISL_419239	A/duck/Hungary/1565_20VIR749-2/2020	A / H5N8	Europe / Hungary	Ambra Pastori (Istituto Zooprofilattico Sperimentale Delle Venezie)
EPI_ISL_419312	A/steamer duck/Germany-SN/AI00346/2020	A / H5N8	Europe / Germany / Saxony / Zwickau	Jacqueline King (Friedrich-Loeffler-Institut)
EPI_ISL_419314	A/turkey/Germany-ST/AI00352/2020	A / H5N8	Europe / Germany / Saxony-Anhalt / Börde	Jacqueline King (Friedrich-Loeffler-Institut)
EPI_ISL_419344	A/chicken/Bulgaria/Dobrich/12-2/2018	A / H5N8	Europe / Bulgaria	Divya Venkatesh (University of Cambridge / Zoology)
EPI_ISL_419345	A/chicken/Bulgaria/Plovdiv/224-1/2018	A / H5N8	Europe / Bulgaria	Divya Venkatesh (University of Cambridge / Zoology)
EPI_ISL_419346	A/chicken/Bulgaria/Plovdiv/224-2/2018	A / H5N8	Europe / Bulgaria	Divya Venkatesh (University of Cambridge / Zoology)
EPI_ISL_419347	A/chicken/Bulgaria/Plovdiv/224-3/2018	A / H5N8	Europe / Bulgaria	Divya Venkatesh (University of Cambridge / Zoology)
EPI_ISL_419348	A/chicken/Bulgaria/Haskovo/411/2017	A / H5N8	Europe / Bulgaria	Divya Venkatesh (University of Cambridge / Zoology)
EPI_ISL_419349	A/chicken/Bulgaria/Sliven/432/2017	A / H5N8	Europe / Bulgaria	Divya Venkatesh (University of Cambridge / Zoology)
EPI_ISL_419350	A/duck/Bulgaria/Dobrich/407/2017	A / H5N8	Europe / Bulgaria	Divya Venkatesh (University of Cambridge / Zoology)
EPI_ISL_419351	A/duck/Bulgaria/Yambol/436/2017	A / H5N8	Europe / Bulgaria	Divya Venkatesh (University of Cambridge / Zoology)
EPI_ISL_419352	A/duck/Bulgaria/Stara-Zagora/623/2017	A / H5N8	Europe / Bulgaria	Divya Venkatesh (University of Cambridge / Zoology)
EPI_ISL_419353	A/duck/Bulgaria/Yambol/35-1/2018	A / H5N8	Europe / Bulgaria	Divya Venkatesh (University of Cambridge / Zoology)
EPI_ISL_419355	A/partridge/Bulgaria/Plovdiv/60-2/2018	A / H5N8	Europe / Bulgaria	Divya Venkatesh (University of Cambridge / Zoology)
EPI_ISL_419357	A/duck/Bulgaria/Plovdiv/76-1/2018	A / H5N8	Europe / Bulgaria	Divya Venkatesh (University of Cambridge / Zoology)
EPI_ISL_419358	A/duck/Bulgaria/Plovdiv/76-2/2018	A / H5N8	Europe / Bulgaria	Divya Venkatesh (University of Cambridge / Zoology)
EPI_ISL_419359	A/chicken/Bulgaria/Dobrich/115/2018	A / H5N8	Europe / Bulgaria	Divya Venkatesh (University of Cambridge / Zoology)
EPI_ISL_419360	A/chicken/Bulgaria/Dobrich/163-1/2018	A / H5N8	Europe / Bulgaria	Divya Venkatesh (University of Cambridge / Zoology)
EPI_ISL_419361	A/chicken/Bulgaria/Dobrich/163-2/2018	A / H5N8	Europe / Bulgaria	Divya Venkatesh (University of Cambridge / Zoology)
EPI_ISL_419362	A/chicken/Bulgaria/Haskovo/286/2018	A / H5N8	Europe / Bulgaria	Divya Venkatesh (University of Cambridge / Zoology)
EPI_ISL_419363	A/chicken/Bulgaria/Plovdiv/295/2018	A / H5N8	Europe / Bulgaria	Divya Venkatesh (University of Cambridge / Zoology)
EPI_ISL_419364	A/chicken/Bulgaria/Plovdiv/333/2018	A / H5N8	Europe / Bulgaria	Divya Venkatesh (University of Cambridge / Zoology)
EPI_ISL_419365	A/turkey/Bulgaria/Haskovo/336/2018	A / H5N8	Europe / Bulgaria	Divya Venkatesh (University of Cambridge / Zoology)
EPI_ISL_419366	A/duck/Bulgaria/Plovdiv/74-1/2018	A / H5N8	Europe / Bulgaria	Divya Venkatesh (University of Cambridge / Zoology)
EPI_ISL_419367	A/chicken/Bulgaria/Vidin-Kosovo/550/2018	A / H5N8	Europe / Bulgaria	Divya Venkatesh (University of Cambridge / Zoology)
EPI_ISL_436133	A/chicken/Germany-MV/AR9311-L02968/2016	A / H5N8	Europe / Germany / Mecklenburg-Vorpommern / Vorpommern-Rügen	Jacqueline King (Friedrich-Loeffler-Institut)
EPI_ISL_436134	A/domestic duck/Germany-MV/AR9433-L02969/2016	A / H5N8	Europe / Germany / Mecklenburg-Vorpommern / Vorpommern-Greifswald	Jacqueline King (Friedrich-Loeffler-Institut)
EPI_ISL_436135	A/chicken/Germany-MV/AR9528-L02970/2016	A / H5N8	Europe / Germany / Mecklenburg-Vorpommern / Vorpommern-Rügen	Jacqueline King (Friedrich-Loeffler-Institut)
EPI_ISL_436136	A/chicken/Germany-MV/AR9738-L02971/2016	A / H5N8	Europe / Germany / Mecklenburg-Vorpommern / Vorpommern-Greifswald	Jacqueline King (Friedrich-Loeffler-Institut)
EPI_ISL_436142	A/domestic duck/Germany-ST/AR11090-L02972/2016	A / H5N8	Europe / Germany / Saxony-Anhalt / Jerichower Land	Jacqueline King (Friedrich-Loeffler-Institut)
EPI_ISL_436143	A/turkey/Germany-NW/AR11095-L02973/2016	A / H5N8	Europe / Germany / North Rhine-Westphalia / Soest	Jacqueline King (Friedrich-Loeffler-Institut)
EPI_ISL_436144	A/domestic goose/Germany-NW/AR11222-L02974/2016	A / H5N8	Europe / Germany / North Rhine-Westphalia / Gütersloh	Jacqueline King (Friedrich-Loeffler-Institut)
EPI_ISL_436145	A/turkey/Germany-NW/AR11455-L02975/2016	A / H5N8	Europe / Germany / North Rhine-Westphalia / Kleve	Jacqueline King (Friedrich-Loeffler-Institut)
EPI_ISL_436146	A/chicken/Germany-ST/AR101-L02976/2017	A / H5N8	Europe / Germany / Saxony-Anhalt / Salzlandkreis	Jacqueline King (Friedrich-Loeffler-Institut)
EPI_ISL_436147	A/turkey/Germany-NW/AR246-L02977/2017	A / H5N8	Europe / Germany / North Rhine-Westphalia / Wesel	Jacqueline King (Friedrich-Loeffler-Institut)
EPI_ISL_436148	A/turkey/Germany-NW/AR258-L02994/2017	A / H5N8	Europe / Germany / North Rhine-Westphalia / Paderborn	Jacqueline King (Friedrich-Loeffler-Institut)
EPI_ISL_436149	A/domestic duck/Germany-NI/AR350-L02995/2017	A / H5N8	Europe / Germany / Lower Saxony / Diepholz	Jacqueline King (Friedrich-Loeffler-Institut)
EPI_ISL_436150	A/turkey/Germany-SH/AR495-L02996/2017	A / H5N5	Europe / Germany / Schleswig-Holstein / Steinburg	Jacqueline King (Friedrich-Loeffler-Institut)
EPI_ISL_436151	A/turkey/Germany-SH/AR804-L02997/2017	A / H5N5	Europe / Germany / Schleswig-Holstein / Steinburg	Jacqueline King (Friedrich-Loeffler-Institut)
EPI_ISL_436152	A/turkey/Germany-MV/AR622-L02998/2017	A / H5N8	Europe / Germany / Mecklenburg-Vorpommern / Grimmen	Jacqueline King (Friedrich-Loeffler-Institut)
EPI_ISL_436159	A/chicken/Germany-BY/AR845-L02999/2017	A / H5N8	Europe / Germany / Bavaria / Hof	Jacqueline King (Friedrich-Loeffler-Institut)
EPI_ISL_436160	A/chicken/Germany-MV/AR851-L03000/2017	A / H5N8	Europe / Germany / Mecklenburg-Vorpommern / Ludwigslust-Parchim	Jacqueline King (Friedrich-Loeffler-Institut)
EPI_ISL_436161	A/domestic duck/Germany-BB/AR877-L03001/2017	A / H5N8	Europe / Germany / Brandenburg / Märkisch-Oderland	Jacqueline King (Friedrich-Loeffler-Institut)
EPI_ISL_436162	A/turkey/Germany-SN/AR888-L03002/2017	A / H5N8	Europe / Germany / Saxony / Nordsachsen	Jacqueline King (Friedrich-Loeffler-Institut)
EPI_ISL_436163	A/domestic duck/Germany-BB/AR906-L03003/2017	A / H5N8	Europe / Germany / Brandenburg / Märkisch-Oderland	Jacqueline King (Friedrich-Loeffler-Institut)

EPI_ISL_436164	A/turkey/Germany-BB/AR1015-L03026/2017	A / H5N8	Europe / Germany / Brandenburg / Märkisch-Oderland	Jacqueline King (Friedrich-Loeffler-Institut)
EPI_ISL_436165	A/turkey/Germany-BY/AR1132-L03027/2017	A / H5N8	Europe / Germany / Bavaria / Straubing-Bogen	Jacqueline King (Friedrich-Loeffler-Institut)
EPI_ISL_436166	A/turkey/Germany-BB/AR1223-L03028/2017	A / H5N8	Europe / Germany / Brandenburg / Ostprignitz-Ruppin	Jacqueline King (Friedrich-Loeffler-Institut)
EPI_ISL_436167	A/domestic duck/Germany-BB/AR1341-L03029/2017	A / H5N8	Europe / Germany / Brandenburg / Märkisch-Oderland	Jacqueline King (Friedrich-Loeffler-Institut)
EPI_ISL_436168	A/chicken/Germany-NW/AR1384-L03030/2017	A / H5N8	Europe / Germany / North Rhine-Westphalia / Paderborn	Jacqueline King (Friedrich-Loeffler-Institut)
EPI_ISL_436169	A/chicken/Germany-MV/AR1398-L03031/2017	A / H5N8	Europe / Germany / Mecklenburg-Vorpommern / Rostock	Jacqueline King (Friedrich-Loeffler-Institut)
EPI_ISL_436170	A/domestic duck/Germany-BB/AR1464-L03032/2017	A / H5N8	Europe / Germany / Brandenburg / Märkisch-Oderland	Jacqueline King (Friedrich-Loeffler-Institut)
EPI_ISL_436171	A/chicken/Germany-TH/AR1489-L03033/2017	A / H5N8	Europe / Germany / Thuringia / Greiz	Jacqueline King (Friedrich-Loeffler-Institut)
EPI_ISL_436172	A/chicken/Germany-TH/AR1638-L03034/2017	A / H5N8	Europe / Germany / Thuringia / Sömmerda	Jacqueline King (Friedrich-Loeffler-Institut)
EPI_ISL_436173	A/chicken/Germany-RP/AR1774-L03035/2017	A / H5N8	Europe / Germany / Rhineland-Palatinate / Bad Dürkheim	Jacqueline King (Friedrich-Loeffler-Institut)
EPI_ISL_436174	A/chicken/Germany-NW/AR3197-L03036/2017	A / H5N8	Europe / Germany / North Rhine-Westphalia / Lippe	Jacqueline King (Friedrich-Loeffler-Institut)
EPI_ISL_436175	A/turkey/Germany-NI/AR1964-L02703/2017	A / H5N8	Europe / Germany / Lower Saxony / Cloppenburg	Jacqueline King (Friedrich-Loeffler-Institut)
EPI_ISL_436176	A/turkey/Germany-NI/AR2024-L02704/2017	A / H5N8	Europe / Germany / Lower Saxony / Cloppenburg	Jacqueline King (Friedrich-Loeffler-Institut)
EPI_ISL_436177	A/turkey/Germany-NI/AR2095-L02705/2017	A / H5N8	Europe / Germany / Lower Saxony / Cloppenburg	Jacqueline King (Friedrich-Loeffler-Institut)
EPI_ISL_436178	A/turkey/Germany-NI/AR2142-L02706/2017	A / H5N8	Europe / Germany / Lower Saxony / Cloppenburg	Jacqueline King (Friedrich-Loeffler-Institut)
EPI_ISL_436179	A/turkey/Germany-NI/AR2195-L02707/2017	A / H5N8	Europe / Germany / Lower Saxony / Cloppenburg	Jacqueline King (Friedrich-Loeffler-Institut)
EPI_ISL_436180	A/turkey/Germany-NI/AR2248-L02708/2017	A / H5N8	Europe / Germany / Lower Saxony / Cloppenburg	Jacqueline King (Friedrich-Loeffler-Institut)
EPI_ISL_436181	A/turkey/Germany-NI/AR2350-L02709/2017	A / H5N8	Europe / Germany / Lower Saxony / Cloppenburg	Jacqueline King (Friedrich-Loeffler-Institut)
EPI_ISL_436182	A/turkey/Germany-NI/AR2374-L02710/2017	A / H5N8	Europe / Germany / Lower Saxony / Cloppenburg	Jacqueline King (Friedrich-Loeffler-Institut)
EPI_ISL_436183	A/turkey/Germany-NI/AR2493-L02711/2017	A / H5N8	Europe / Germany / Lower Saxony / Cloppenburg	Jacqueline King (Friedrich-Loeffler-Institut)
EPI_ISL_436184	A/chicken/Germany-NI/AR2541-L02712/2017	A / H5N8	Europe / Germany / Lower Saxony / Cloppenburg	Jacqueline King (Friedrich-Loeffler-Institut)
EPI_ISL_436185	A/turkey/Germany-NI/AR2704-L02713/2017	A / H5N8	Europe / Germany / Lower Saxony / Cloppenburg	Jacqueline King (Friedrich-Loeffler-Institut)
EPI_ISL_436186	A/turkey/Germany-NI/AR2552-L02714/2017	A / H5N8	Europe / Germany / Lower Saxony / Cloppenburg	Jacqueline King (Friedrich-Loeffler-Institut)
EPI_ISL_436187	A/turkey/Germany-NI/AR2732-L02715/2017	A / H5N8	Europe / Germany / Lower Saxony / Cloppenburg	Jacqueline King (Friedrich-Loeffler-Institut)
EPI_ISL_436188	A/turkey/Germany-NI/AR2769-L02716/2017	A / H5N8	Europe / Germany / Lower Saxony / Cloppenburg	Jacqueline King (Friedrich-Loeffler-Institut)
EPI_ISL_436189	A/turkey/Germany-NI/AR2795-L02717/2017	A / H5N8	Europe / Germany / Lower Saxony / Cloppenburg	Jacqueline King (Friedrich-Loeffler-Institut)
EPI_ISL_436190	A/chicken/Germany-NI/AR2921-L02720/2017	A / H5N8	Europe / Germany / Lower Saxony / Ammerland	Jacqueline King (Friedrich-Loeffler-Institut)
EPI_ISL_436191	A/turkey/Germany-NI/AR2909-L02719/2017	A / H5N8	Europe / Germany / Lower Saxony / Cloppenburg	Jacqueline King (Friedrich-Loeffler-Institut)
EPI_ISL_436192	A/turkey/Germany-NI/AR3012-L02721/2017	A / H5N8	Europe / Germany / Lower Saxony / Cloppenburg	Jacqueline King (Friedrich-Loeffler-Institut)
EPI_ISL_436193	A/turkey/Germany-NI/AR3128-L02722/2017	A / H5N8	Europe / Germany / Lower Saxony / Cloppenburg	Jacqueline King (Friedrich-Loeffler-Institut)
EPI_ISL_436690	A/turkey/Germany-SH/AR417-L03291/2017	A / H5N5	Europe / Germany / Schleswig-Holstein / Steinburg	Jacqueline King (Friedrich-Loeffler-Institut)
EPI_ISL_436692	A/turkey/Germany-SH/AR420-L03292/2017	A / H5N5	Europe / Germany / Schleswig-Holstein / Steinburg	Jacqueline King (Friedrich-Loeffler-Institut)
EPI_ISL_436693	A/turkey/Germany-SH/AR424-L03293/2017	A / H5N5	Europe / Germany / Schleswig-Holstein / Steinburg	Jacqueline King (Friedrich-Loeffler-Institut)
EPI_ISL_436694	A/turkey/Germany-SH/AR425-L03294/2017	A / H5N5	Europe / Germany / Schleswig-Holstein / Steinburg	Jacqueline King (Friedrich-Loeffler-Institut)
EPI_ISL_436695	A/turkey/Germany-SH/AR426-L03295/2017	A / H5N5	Europe / Germany / Schleswig-Holstein / Steinburg	Jacqueline King (Friedrich-Loeffler-Institut)
EPI_ISL_436696	A/turkey/Germany-SH/AR494-L03296/2017	A / H5N5	Europe / Germany / Schleswig-Holstein / Steinburg	Jacqueline King (Friedrich-Loeffler-Institut)
EPI_ISL_436697	A/turkey/Germany-SH/AR769-L03298/2017	A / H5N5	Europe / Germany / Schleswig-Holstein / Steinburg	Jacqueline King (Friedrich-Loeffler-Institut)
EPI_ISL_436698	A/turkey/Germany-SH/AR780-L03299/2017	A / H5N5	Europe / Germany / Schleswig-Holstein / Steinburg	Jacqueline King (Friedrich-Loeffler-Institut)
EPI_ISL_436699	A/turkey/Germany-SH/AR786-L03300/2017	A / H5N5	Europe / Germany / Schleswig-Holstein / Steinburg	Jacqueline King (Friedrich-Loeffler-Institut)
EPI_ISL_436700	A/turkey/Germany-SH/AR793-L03301/2017	A / H5N5	Europe / Germany / Schleswig-Holstein / Steinburg	Jacqueline King (Friedrich-Loeffler-Institut)
EPI_ISL_436701	A/turkey/Germany-SH/AR803-L03302/2017	A / H5N5	Europe / Germany / Schleswig-Holstein / Steinburg	Jacqueline King (Friedrich-Loeffler-Institut)
EPI_ISL_436702	A/turkey/Germany-SH/AR806-L03303/2017	A / H5N5	Europe / Germany / Schleswig-Holstein / Steinburg	Jacqueline King (Friedrich-Loeffler-Institut)
EPI_ISL_436703	A/turkey/Germany-SH/AR815-L03304/2017	A / H5N5	Europe / Germany / Schleswig-Holstein / Steinburg	Jacqueline King (Friedrich-Loeffler-Institut)
EPI_ISL_436704	A/turkey/Germany-SH/AR822-L03305/2017	A / H5N5	Europe / Germany / Schleswig-Holstein / Steinburg	Jacqueline King (Friedrich-Loeffler-Institut)
EPI_ISL_436705	A/turkey/Germany-SH/AR827-L03306/2017	A / H5N5	Europe / Germany / Schleswig-Holstein / Steinburg	Jacqueline King (Friedrich-Loeffler-Institut)
EPI_ISL_436707	A/greylag goose/Germany-SH/AR1609-L03307/2017	A / H5N5	Europe / Germany / Schleswig-Holstein / Neumünster	Jacqueline King (Friedrich-Loeffler-Institut)
EPI_ISL_436708	A/mallard/Germany-SH/AR1659-L03308/2017	A / H5N5	Europe / Germany / Schleswig-Holstein / Rendsburg-Eckernförde	Jacqueline King (Friedrich-Loeffler-Institut)
EPI_ISL_436709	A/mute swan/Germany-SH/AR1802-L03309/2017	A / H5N5	Europe / Germany / Schleswig-Holstein / Rendsburg-Eckernförde	Jacqueline King (Friedrich-Loeffler-Institut)
EPI_ISL_436710	A/greylag goose/Germany-SH/AR1803-L03310/2017	A / H5N5	Europe / Germany / Schleswig-Holstein / Neumünster	Jacqueline King (Friedrich-Loeffler-Institut)
EPI_ISL_436712	A/common buzzard/Germany-SH/AR2172-L03311/2017	A / H5N5	Europe / Germany / Schleswig-Holstein / Segeberg	Jacqueline King (Friedrich-Loeffler-Institut)
EPI_ISL_4396748	A/Meleagris gallopavo/Belgium/11293_001/2021	A / H5N8	Europe / Belgium / Provincie West-Vlaanderen	Steven Van Borm (Sciensano, Department of Animal Infectious Diseases / Animal Infectious Diseases)
EPI_ISL_4558108	A/Sichuan/06689/2021	A / H5N6	Asia / China / Sichuan Province	Lei Yang (WHO Chinese National Influenza Center / Virology Institute, Chinese CDC)
EPI_ISL_4568642	A/Hunan/09285/2021	A / H5N6	Asia / China / Hunan Province / Hunan	Lei Yang (WHO Chinese National Influenza Center / Virology Institute, Chinese CDC)
EPI_ISL_4568643	A/Hunan/09911/2021	A / H5N6	Asia / China / Hunan Province / Hunan	Lei Yang (WHO Chinese National Influenza Center / Virology Institute, Chinese CDC)
EPI_ISL_4568644	A/Chongqing/02/2021	A / H5N6	Asia / China / Chongqinq Municipality / Chongqing	Lei Yang (WHO Chinese National Influenza Center / Virology Institute, Chinese CDC)
EPI_ISL_463009	A/duck/Bulgaria/Plovdiv/74-2/2018	A / H5N2	Europe / Bulgaria	Divya Venkatesh (University of Cambridge / Zoology)
EPI_ISL_4651963	A/common pheasant /Sweden/SVA210923SZ0341/KN000365/	A / H5N1	Europe / Sweden / Skane Lan / Kristianstads Kommun	Siamak Zohari (National Veterinary Institute)
EPI_ISL_4653177	A/common pheasant /Sweden/SVA210923SZ0341/KN000366/	A / H5N1	Europe / Sweden / Skane Lan / Kristianstads Kommun	Siamak Zohari (National Veterinary Institute)
EPI_ISL_4804850	A/seal/Germany-SH/AI05373/2021	A / H5N8	Europe / Germany / Schleswig-Holstein / Nordfriesland	Jacqueline King (Friedrich-Loeffler-Institut)
EPI_ISL_4805852	A/seal/Germany-SH/AI05377/2021	A / H5N8	Europe / Germany / Schleswig-Holstein / Dithmarschen	Jacqueline King (Friedrich-Loeffler-Institut)
EPI_ISL_4805936	A/seal/Germany-SH/AI05379/2021	A / H5N8	Europe / Germany / Schleswig-Holstein / Nordfriesland	Jacqueline King (Friedrich-Loeffler-Institut)
EPI_ISL_486440	recombinant A/tufted duck/Germany/AR8444/2016	A / H5N8	Europe / Germany	El-Sayed M. Abdelwhab (Friedrich-Loeffler-Institut)
EPI_ISL_486441	recombinant A/turkey/Germany-MV/AR2487/2014	A / H5N8	Europe / Germany	El-Sayed M. Abdelwhab (Friedrich-Loeffler-Institut)
EPI_ISL_499181	A/goose/China/GS39/2016	A / H5N6	Asia / China	
EPI_ISL_499182	A/chicken/China/CK44/2016	A / H5N6	Asia / China	
EPI_ISL_499183	A/duck/China/DK47/2016	A / H5N6	Asia / China	
EPI_ISL_499184	A/chicken/China/CK74/2016	A / H5N6	Asia / China	
EPI_ISL_501644	A/Duck/Viet Nam/HU12-981/2019	A / H5N6	Asia / Vietnam	
EPI_ISL_501645	A/Duck/Viet Nam/HU12-1303/2019	A / H5N6	Asia / Vietnam	
EPI_ISL_501646	A/Duck/Viet Nam/HU12-982/2019	A / H5N6	Asia / Vietnam	
EPI_ISL_501647	A/Duck/Viet Nam/HU12-1310/2019	A / H5N6	Asia / Vietnam	
EPI_ISL_501648	A/Duck/Viet Nam/HU12-1305/2019	A / H5N6	Asia / Vietnam	

EPI_ISL_501649	A/Duck/Viet Nam/HU12-1309/2019	A / H5N6	Asia / Vietnam	
EPI_ISL_501651	A/Duck/Viet Nam/HU12-1467/2019	A / H5N6	Asia / Vietnam	
EPI_ISL_501652	A/Duck/Viet Nam/HU12-1465/2019	A / H5N6	Asia / Vietnam	
EPI_ISL_501653	A/Duck/Viet Nam/HU12-1471/2019	A / H5N6	Asia / Vietnam	
EPI_ISL_501654	A/Duck/Viet Nam/HU12-1470/2019	A / H5N6	Asia / Vietnam	
EPI_ISL_501655	A/Duck/Viet Nam/HU12-1472/2019	A / H5N6	Asia / Vietnam	
EPI_ISL_502069	A/muscovy duck/Japan/AQ-HE30-77C1/2018	A / H5N6	Asia / Japan	
EPI_ISL_502070	A/muscovy duck/Japan/AQ-HE30-77C2/2018	A / H5N6	Asia / Japan	
EPI_ISL_502201	A/mink/Eastern China/006/2018	A / H5N6	Asia / China	
EPI_ISL_502202	A/mink/Eastern China/032/2018	A / H5N6	Asia / China	
EPI_ISL_502203	A/mink/Northern China/110/2018	A / H5N6	Asia / China	
EPI_ISL_502204	A/mink/Eastern China/149/2018	A / H5N6	Asia / China	
EPI_ISL_502205	A/mink/Eastern China/528/2018	A / H5N6	Asia / China	
EPI_ISL_502206	A/mink/Eastern China/0824/2018	A / H5N6	Asia / China	
EPI_ISL_502207	A/mink/Eastern China/0712/2018	A / H5N1	Asia / China	
EPI_ISL_502208	A/mink/Eastern China/571/2018	A / H5N6	Asia / China	
EPI_ISL_502279	A/chicken/Egypt/F15099/2018	A / H5N8	Africa / Egypt	
EPI_ISL_502280	A/chicken/Egypt/N15173D/2018	A / H5N8	Africa / Egypt	
EPI_ISL_502393	A/chicken/Taiwan/A3/2019	A / H5N2	Asia / Taiwan	
EPI_ISL_502394	A/chicken/Taiwan/A4/2019	A / H5N2	Asia / Taiwan	
EPI_ISL_502395	A/chicken/Taiwan/A5/2019	A / H5N2	Asia / Taiwan	
EPI_ISL_502396	A/chicken/Taiwan/D2/2019	A / H5N2	Asia / Taiwan	
EPI_ISL_502397	A/chicken/Taiwan/D9/2019	A / H5N2	Asia / Taiwan	
EPI_ISL_503010	A/muscovy duck/Vietnam/LBM1021/2017	A / H5N6	Asia / Vietnam	
EPI_ISL_503012	A/duck/Vietnam/LBM1099/2018	A / H5N6	Asia / Vietnam	
EPI_ISL_503013	A/muscovy duck/Vietnam/LBM1101/2018	A / H5N6	Asia / Vietnam	
EPI_ISL_503015	A/duck/Nha Trang/271/2018	A / H5N6	Asia / Vietnam / Tinh Khanh Hoa	
EPI_ISL_503016	A/duck/Nha Trang/301/2018	A / H5N6	Asia / Vietnam / Tinh Khanh Hoa	
EPI_ISL_503017	A/duck/Quang Ninh/371/2018	A / H5N6	Asia / Vietnam / Tinh Quang Ninh	
EPI_ISL_503018	A/chicken/Japan/AQ-HE30-35C1/2018	A / H5N2	Asia / Japan	
EPI_ISL_503024	A/duck/China/0936/2017	A / H5N6	Asia / China	
EPI_ISL_503031	A/Anser fabalis/China/P563/2015	A / H5N6	Asia / China	
EPI_ISL_503036	A/Anser cygnoides/China/P126/2015	A / H5N1	Asia / China	
EPI_ISL_503037	A/swan/China/ST/2016	A / H5N8	Asia / China	
EPI_ISL_503058	A/pigeon/Egypt/A15052/2018	A / H5N8	Africa / Egypt	
EPI_ISL_503150	A/duck/Nigeria/SK28T_19VIR8424-2/2019	A / H5N6	Africa / Nigeria	
EPI_ISL_5034659	A/turkey/Poland/100s3/2016	A / H5N8	Europe / Poland / Lubusz Voivodeship	Edyta ?wi?to? (National Veterinary Research Institut Poland, PIWet-PIB)
EPI_ISL_5034849	A/domestic_duck/Poland/106/2017(H5N8)	A / H5N8	Europe / Poland / Greater Poland	Edyta ?wi?to? (National Veterinary Research Institut Poland, PIWet-PIB)
EPI_ISL_5034850	A/turkey/Poland/109s1/2016(H5N8)	A / H5N8	Europe / Poland / Lubusz	Edyta ?wi?to? (National Veterinary Research Institut Poland, PIWet-PIB)
EPI_ISL_5034851	A/chicken/Poland/115/2016(H5N8)	A / H5N8	Europe / Poland / Lubusz	Edyta ?wi?to? (National Veterinary Research Institut Poland, PIWet-PIB)
EPI_ISL_5034852	A/turkey/Poland/15s4/2017(H5N8)	A / H5N8	Europe / Poland / Lubusz	Edyta ?wi?to? (National Veterinary Research Institut Poland, PIWet-PIB)
EPI_ISL_5034853	A/domestic_goose/Poland/204/2017(H5N8)	A / H5N8	Europe / Poland / Greater Poland	Edyta ?wi?to? (National Veterinary Research Institut Poland, PIWet-PIB)
EPI_ISL_5034854	A/domestic_duck/Poland/238/2017(H5N8)	A / H5N8	Europe / Poland / Greater Poland	Edyta ?wi?to? (National Veterinary Research Institut Poland, PIWet-PIB)
EPI_ISL_5034855	A/turkey/Poland/78s1/2016(H5N8)	A / H5N8	Europe / Poland / Lubusz	Edyta ?wi?to? (National Veterinary Research Institut Poland, PIWet-PIB)
EPI_ISL_5034856	A/domestic_duck/Poland/90/2017(H5N8)	A / H5N8	Europe / Poland / Greater Poland	Edyta ?wi?to? (National Veterinary Research Institut Poland, PIWet-PIB)
EPI_ISL_503594	A/duck/Viet Nam/HU13-60/2019	A / H5N6	Asia / Vietnam	
EPI_ISL_503599	A/duck/Viet Nam/HU12-970/2019	A / H5N6	Asia / Vietnam	
EPI_ISL_503600	A/duck/Viet Nam/HU12-971/2019	A / H5N6	Asia / Vietnam	
EPI_ISL_503601	A/duck/Viet Nam/HU12-972/2019	A / H5N6	Asia / Vietnam	
EPI_ISL_503602	A/duck/Viet Nam/HU12-977/2019	A / H5N6	Asia / Vietnam	
EPI_ISL_503603	A/duck/Viet Nam/HU12-978/2019	A / H5N6	Asia / Vietnam	
EPI_ISL_503604	A/duck/Viet Nam/HU12-979/2019	A / H5N6	Asia / Vietnam	
EPI_ISL_503605	A/duck/Viet Nam/HU12-980/2019	A / H5N6	Asia / Vietnam	
EPI_ISL_503606	A/duck/Viet Nam/HU13-163/2019	A / H5N6	Asia / Vietnam	
EPI_ISL_503607	A/duck/Viet Nam/HU13-164/2019	A / H5N6	Asia / Vietnam	
EPI_ISL_503608	A/duck/Viet Nam/HU13-64/2019	A / H5N6	Asia / Vietnam	
EPI_ISL_503609	A/duck/Viet Nam/HU13-65/2019	A / H5N6	Asia / Vietnam	
EPI_ISL_503610	A/duck/Viet Nam/HU13-71/2019	A / H5N6	Asia / Vietnam	
EPI_ISL_503791	A/Guinea fowl/Belgium/810/2017	A / H5N8	Europe / Belgium	
EPI_ISL_503819	A/duck/Viet Nam/HU12-1473/2019	A / H5N6	Asia / Vietnam	
EPI_ISL_503935	A/Teal/Dakahlia/VRLCU/2019	A / H5N8	Africa / Egypt / Dakahlia	
EPI_ISL_503968	A/chicken/Bulgaria/217_20VIR1724-1/2020	A / H5N8	Europe / Bulgaria	
EPI_ISL_503969	A/chicken/Bulgaria/77_20VIR1727/2020	A / H5N2	Europe / Bulgaria	
EPI_ISL_504004	A/chicken/Quang Tri/MT11/2016	A / H5N6	Asia / Vietnam / Tinh Quang Tri	
EPI_ISL_504005	A/duck/Ha Tinh/HT12/2014	A / H5N6	Asia / Vietnam	
EPI_ISL_504006	A/duck/Ha Tinh/HT7/2014	A / H5N6	Asia / Vietnam	
EPI_ISL_504007	A/chicken/Nha Trang/NT3/2017	A / H5N6	Asia / Vietnam	
EPI_ISL_5049590	A/chicken/Luxembourg/21168413/2021	A / H5N8	Europe / Luxembourg / District de Grevenmacher	Chantal J. Snoeck (Luxembourg Institute of Health / Department of Infection and Immunity)
EPI_ISL_504984	A/duck/Viet Nam/HU13-161/2019	A / H5N6	Asia / Vietnam	
EPI_ISL_504985	A/duck/Viet Nam/HU13-162/2019	A / H5N6	Asia / Vietnam	
EPI_ISL_505073	A/chicken/Sichuan/k141/2017	A / H5N6	Asia / China / Sichuan Province	
EPI_ISL_505396	A/Duck/Giza/1814/2018	A / H5N8	Africa / Egypt / Giza	

EPI_ISL_505397	A/Duck/Al-Shaqiya/172AS/2017	A / H5N8	Africa / Egypt	
EPI_ISL_505398	A/Duck/AL-Minia/1777FAO-S/2017	A / H5N8	Africa / Egypt	
EPI_ISL_505399	A/chicken/Al-Sharqia/1822FM/2018	A / H5N8	Africa / Egypt	
EPI_ISL_505400	A/chicken/Al-Minia/1785Fao-S/2017	A / H5N8	Africa / Egypt	
EPI_ISL_505401	A/Duck/Ismalia/171Fao-SI/2017	A / H5N8	Africa / Egypt	
EPI_ISL_505402	A/Duck/Ismalia/175Fao-SI/2017	A / H5N8	Africa / Egypt	
EPI_ISL_505403	A/Duck/Ismalia/1719Fao-SI/2017	A / H5N8	Africa / Egypt	
EPI_ISL_505404	A/chicken/Al Qaliobia/1755FAO-S/2017	A / H5N8	Africa / Egypt	
EPI_ISL_505405	A/Duck/Ismalia/176FAO-SI/2017	A / H5N8	Africa / Egypt	
EPI_ISL_505406	A/chicken/AL Suez/184/2018	A / H5N8	Africa / Egypt	
EPI_ISL_505407	A/chicken/Cairo/1794FAO-S/2017	A / H5N8	Africa / Egypt / Cairo	
EPI_ISL_505408	A/Duck/AL-Qaliobia/182Fao-s/2018	A / H5N8	Africa / Egypt	
EPI_ISL_505409	A/Duck/Cairo/189Fao-S/2018	A / H5N8	Africa / Egypt / Cairo	
EPI_ISL_505410	A/chicken/Al-Minia/183/2018	A / H5N8	Africa / Egypt	
EPI_ISL_505411	A/Turkey/Beni Sueif/18296F/2018	A / H5N8	Africa / Egypt	
EPI_ISL_505412	A/chicken/Giza/1836CAL/2017	A / H5N8	Africa / Egypt / Giza	
EPI_ISL_505413	A/chicken/Albehra/18319F/2018	A / H5N8	Africa / Egypt	
EPI_ISL_505414	A/chicken/Al- Minia/189CA/2018	A / H5N8	Africa / Egypt	
EPI_ISL_505415	A/chicken/Giza/1810CA/2018	A / H5N8	Africa / Egypt / Giza	
EPI_ISL_505416	A/Duck/Al-Monfia/1727/2017	A / H5N8	Africa / Egypt	
EPI_ISL_505417	A/Duck/EL-WadiAlgidid/185/2018	A / H5N8	Africa / Egypt	
EPI_ISL_505418	A/Duck/Cairo/187Fao-S/2018	A / H5N8	Africa / Egypt / Cairo	
EPI_ISL_505419	A/Duck/Ismalia/1721Fao-SI/2017	A / H5N8	Africa / Egypt	
EPI_ISL_505420	A/Duck/Al-Sharqiya/1733FM/2017	A / H5N8	Africa / Egypt	
EPI_ISL_505421	A/Duck/Cairo/1895CA/2017	A / H5N8	Africa / Egypt / Cairo	
EPI_ISL_505422	A/chicken/Al-Minia/188Fao-S/2018	A / H5N8	Africa / Egypt	
EPI_ISL_505423	A/Duck/Giza/1754FAO-S/2017	A / H5N8	Africa / Egypt / Giza	
EPI_ISL_505431	A/quail/South Africa/AI5930/2018	A / H5N8	Africa / South Africa	
EPI_ISL_505432	A/chicken/South Africa/499723/2018	A / H5N8	Africa / South Africa	
EPI_ISL_505528	A/duck/Saudi Arabia/KFU-HKU3616_2017/2017	A / H5N8	Asia / Saudi Arabia	
EPI_ISL_505529	A/duck/Saudi Arabia/KFU-HKU3617_2017/2017	A / H5N8	Asia / Saudi Arabia	
EPI_ISL_505530	A/duck/Saudi Arabia/KFU-HKU3618_2017/2017	A / H5N8	Asia / Saudi Arabia	
EPI_ISL_505531	A/duck/Saudi Arabia/KFU-HKU3619_2017/2017	A / H5N8	Asia / Saudi Arabia	
EPI_ISL_505532	A/ostrich/Saudi Arabia/KFU-HKU3590_2017/2017	A / H5N8	Asia / Saudi Arabia	
EPI_ISL_505533	A/ostrich/Saudi Arabia/KFU-HKU3591_2017/2017	A / H5N8	Asia / Saudi Arabia	
EPI_ISL_505534	A/ostrich/Saudi Arabia/KFU-HKU3592_2017/2017	A / H5N8	Asia / Saudi Arabia	
EPI_ISL_505535	A/ostrich/Saudi Arabia/KFU-HKU3593_2017/2017	A / H5N8	Asia / Saudi Arabia	
EPI_ISL_505536	A/ostrich/Saudi Arabia/KFU-HKU3594_2017/2017	A / H5N8	Asia / Saudi Arabia	
EPI_ISL_505537	A/pigeon/Saudi Arabia/KFU-HKU3598_2017/2017	A / H5N8	Asia / Saudi Arabia	
EPI_ISL_505538	A/pigeon/Saudi Arabia/KFU-HKU3599_2017/2017	A / H5N8	Asia / Saudi Arabia	
EPI_ISL_505539	A/pigeon/Saudi Arabia/KFU-HKU3600_2017/2017	A / H5N8	Asia / Saudi Arabia	
EPI_ISL_5057844	A/barnacle goose/Germany-SH/AI02168/2020	A / H5N8	Europe / Germany / Schleswig-Holstein / Nordfriesland	Jacqueline King (Friedrich-Loeffler-Institut)
EPI_ISL_5057856	A/peregrine falcon/Germany-SH/AI02162/2020	A / H5N8	Europe / Germany / Schleswig-Holstein / Nordfriesland	Jacqueline King (Friedrich-Loeffler-Institut)
EPI_ISL_5057874	A/barnacle goose/Germany-SH/AI02172/2020	A / H5N8	Europe / Germany / Schleswig-Holstein / Nordfriesland	Jacqueline King (Friedrich-Loeffler-Institut)
EPI_ISL_5057875	A/Eurasian wigeon/Germany-SH/AI02176/2020	A / H5N8	Europe / Germany / Schleswig-Holstein / Nordfriesland	Jacqueline King (Friedrich-Loeffler-Institut)
EPI_ISL_5057876	A/Eurasian wigeon/Germany-SH/AI02179/2020	A / H5N8	Europe / Germany / Schleswig-Holstein / Dithmarschen	Jacqueline King (Friedrich-Loeffler-Institut)
EPI_ISL_5057963	A/barnacle goose/Germany-SH/AI02180/2020	A / H5N8	Europe / Germany / Schleswig-Holstein / Dithmarschen	Jacqueline King (Friedrich-Loeffler-Institut)
EPI_ISL_5058090	A/herring gull/Germany-HH/AI02182/2020	A / H5N8	Europe / Germany / Hamburg / Hamburg	Jacqueline King (Friedrich-Loeffler-Institut)
EPI_ISL_5058262	A/wild duck/Germany-SH/AI02189/2020	A / H5N8	Europe / Germany / Schleswig-Holstein / Nordfriesland	Jacqueline King (Friedrich-Loeffler-Institut)
EPI_ISL_5058432	A/barnacle goose/Germany-SH/AI02190/2020	A / H5N8	Europe / Germany / Schleswig-Holstein / Nordfriesland	Jacqueline King (Friedrich-Loeffler-Institut)
EPI_ISL_5058630	A/greylag goose/Germany-SH/AI02191/2020	A / H5N8	Europe / Germany / Schleswig-Holstein / Nordfriesland	Jacqueline King (Friedrich-Loeffler-Institut)
EPI_ISL_5058855	A/wild goose/Germany-SH/AI02194/2020	A / H5N8	Europe / Germany / Schleswig-Holstein / Rendsburg-Eckernförde	Jacqueline King (Friedrich-Loeffler-Institut)
EPI_ISL_5060031	A/barnacle goose/Germany-SH/AI02199/2020	A / H5N8	Europe / Germany / Schleswig-Holstein / Nordfriesland	Jacqueline King (Friedrich-Loeffler-Institut)
EPI_ISL_5061390	A/greylag goose/Germany-SH/AI02207/2020	A / H5N8	Europe / Germany / Schleswig-Holstein / Nordfriesland	Jacqueline King (Friedrich-Loeffler-Institut)
EPI_ISL_5061617	A/chicken/Germany-MV/AI02431/2020	A / H5N5	Europe / Germany / Schleswig-Holstein / Vorpommern-Rügen	Jacqueline King (Friedrich-Loeffler-Institut)
EPI_ISL_5061890	A/domestic goose/Germany-SH/AI02884/2020	A / H5N8	Europe / Germany / Schleswig-Holstein / Nordfriesland	Jacqueline King (Friedrich-Loeffler-Institut)
EPI_ISL_5062078	A/Eurasian oystercatcher/Germany-SH/AI02269/2020	A / H5N8	Europe / Germany / Schleswig-Holstein / Nordfriesland	Jacqueline King (Friedrich-Loeffler-Institut)
EPI_ISL_5062851	A/herring gull/Germany-MV/AI02300/2020	A / H5N8	Europe / Germany / Mecklenburg-Vorpommern / Vorpommern-Rügen	Jacqueline King (Friedrich-Loeffler-Institut)
EPI_ISL_5063706	A/barnacle goose/Germany-SH/AI02379/2020	A / H5N8	Europe / Germany / Schleswig-Holstein / Nordfriesland	Jacqueline King (Friedrich-Loeffler-Institut)
EPI_ISL_5064270	A/turkey/Germany-NI/AI03453/2020	A / H5N8	Europe / Germany / Lower Saxony / Oldenburg	Jacqueline King (Friedrich-Loeffler-Institut)
EPI_ISL_5064509	A/turkey/Germany-NI/AI03584/2020	A / H5N8	Europe / Germany / Lower Saxony / Cloppenburg	Jacqueline King (Friedrich-Loeffler-Institut)
EPI_ISL_5064707	A/turkey/Germany-NI/AI03594/2020	A / H5N8	Europe / Germany / Lower Saxony / Cloppenburg	Jacqueline King (Friedrich-Loeffler-Institut)
EPI_ISL_5064825	A/turkey/Germany-NI/AI03599/2020	A / H5N8	Europe / Germany / Lower Saxony / Cloppenburg	Jacqueline King (Friedrich-Loeffler-Institut)
EPI_ISL_5064922	A/turkey/Germany-NI/AI03606/2020	A / H5N8	Europe / Germany / Lower Saxony / Cloppenburg	Jacqueline King (Friedrich-Loeffler-Institut)
EPI_ISL_5064971	A/turkey/Germany-NI/AI03609/2020	A / H5N8	Europe / Germany / Lower Saxony / Cloppenburg	Jacqueline King (Friedrich-Loeffler-Institut)
EPI_ISL_5065068	A/turkey/Germany-NI/AI00016/2021	A / H5N8	Europe / Germany / Lower Saxony / Cloppenburg	Jacqueline King (Friedrich-Loeffler-Institut)
EPI_ISL_5065144	A/turkey/Germany-NI/AI00038/2021	A / H5N8	Europe / Germany / Lower Saxony / Cloppenburg	Jacqueline King (Friedrich-Loeffler-Institut)
EPI_ISL_5065276	A/turkey/Germany-NI/AI00042/2021	A / H5N8	Europe / Germany / Lower Saxony / Cloppenburg	Jacqueline King (Friedrich-Loeffler-Institut)
EPI_ISL_5066981	A/turkey/Germany-NI/AI03452/2020	A / H5N8	Europe / Germany / Lower Saxony / Oldenburg	Jacqueline King (Friedrich-Loeffler-Institut)
EPI_ISL_5066982	A/turkey/Germany-NI/AI03654/2020	A / H5N8	Europe / Germany / Lower Saxony / Cloppenburg	Jacqueline King (Friedrich-Loeffler-Institut)
EPI_ISL_5066996	A/turkey/Germany-NI/AI03432/2020	A / H5N8	Europe / Germany / Lower Saxony / Cloppenburg	Jacqueline King (Friedrich-Loeffler-Institut)
EPI_ISL_5067044	A/turkey/Germany-NI/AI00063/2021	A / H5N8	Europe / Germany / Lower Saxony / Oldenburg	Jacqueline King (Friedrich-Loeffler-Institut)

EPI_ISL_5067335	A/turkey/Germany-NI/AI00072/2021	A / H5N8	Europe / Germany / Lower Saxony / Cloppenburg	Jacqueline King (Friedrich-Loeffler-Institut)
EPI_ISL_5067957	A/domestic duck/Germany-NI/AI00079/2021	A / H5N8	Europe / Germany / Lower Saxony / Cloppenburg	Jacqueline King (Friedrich-Loeffler-Institut)
EPI_ISL_5095319	A/turkey/Germany-NI/AI00402/2021	A / H5N8	Europe / Germany / Lower Saxony / Cloppenburg	Jacqueline King (Friedrich-Loeffler-Institut)
EPI_ISL_5095320	A/turkey/Germany-NI/AI00406/2021	A / H5N8	Europe / Germany / Lower Saxony / Cloppenburg	Jacqueline King (Friedrich-Loeffler-Institut)
EPI_ISL_5095321	A/turkey/Germany-NI/AI00429/2021	A / H5N8	Europe / Germany / Lower Saxony / Cloppenburg	Jacqueline King (Friedrich-Loeffler-Institut)
EPI_ISL_5095322	A/turkey/Germany-NI/AI00439/2021	A / H5N8	Europe / Germany / Lower Saxony / Cloppenburg	Jacqueline King (Friedrich-Loeffler-Institut)
EPI_ISL_5095323	A/wild goose/Germany-NI/AI03220/2020	A / H5N8	Europe / Germany / Lower Saxony / Friesland	Jacqueline King (Friedrich-Loeffler-Institut)
EPI_ISL_5095336	A/wild goose/Germany-NI/AI03473/2020	A / H5N8	Europe / Germany / Lower Saxony / Wilhelmshaven, Stadt	Jacqueline King (Friedrich-Loeffler-Institut)
EPI_ISL_5095554	A/chicken/Germany-MV/AI00539/2021	A / H5N8	Europe / Germany / Mecklenburg-Vorpommern / Landkreis Rostock	Jacqueline King (Friedrich-Loeffler-Institut)
EPI_ISL_5095555	A/chicken/Germany-NI/AI00547/2021	A / H5N8	Europe / Germany / Lower Saxony / Oldenburg	Jacqueline King (Friedrich-Loeffler-Institut)
EPI_ISL_5095556	A/turkey/Germany-NI/AI00589/2021	A / H5N8	Europe / Germany / Lower Saxony / Cuxhaven	Jacqueline King (Friedrich-Loeffler-Institut)
EPI_ISL_5095644	A/turkey/Germany-BB/AI00868/2021	A / H5N8	Europe / Germany / Brandenburg / Prignitz	Jacqueline King (Friedrich-Loeffler-Institut)
EPI_ISL_5095645	A/turkey/Germany-MV/AI00909/2021	A / H5N8	Europe / Germany / Mecklenburg-Vorpommern / Nordwestmecklenburg	Jacqueline King (Friedrich-Loeffler-Institut)
EPI_ISL_5095649	A/turkey/Germany-MV/AI01015/2021	A / H5N8	Europe / Germany / Mecklenburg-Vorpommern / Ludwigslust-Parchim	Jacqueline King (Friedrich-Loeffler-Institut)
EPI_ISL_5095650	A/turkey/Germany-BB/AI01023/2021	A / H5N8	Europe / Germany / Brandenburg / Uckermark	Jacqueline King (Friedrich-Loeffler-Institut)
EPI_ISL_5096070	A/mallard/Germany-MV/AI00639/2021	A / H5N8	Europe / Germany / Mecklenburg-Vorpommern / Vorpommern-Greifswald	Jacqueline King (Friedrich-Loeffler-Institut)
EPI_ISL_5096104	A/wild goose/Germany-NI/AI00855/2021	A / H5N8	Europe / Germany / Lower Saxony / Stade	Jacqueline King (Friedrich-Loeffler-Institut)
EPI_ISL_5096105	A/domestic duck/Germany-NI/AI00646/2021	A / H5N8	Europe / Germany / Lower Saxony / Wittmund	Jacqueline King (Friedrich-Loeffler-Institut)
EPI_ISL_5096107	A/turkey/Germany-NI/AI00647/2021	A / H5N8	Europe / Germany / Lower Saxony / Oldenburg	Jacqueline King (Friedrich-Loeffler-Institut)
EPI_ISL_5096629	A/wild goose/Germany-NI/AI00625/2021	A / H5N8	Europe / Germany / Lower Saxony / Cuxhaven	Jacqueline King (Friedrich-Loeffler-Institut)
EPI_ISL_5097280	A/turkey/Germany-NI/AI00621/2021	A / H5N8	Europe / Germany / Lower Saxony / Cuxhaven	Jacqueline King (Friedrich-Loeffler-Institut)
EPI_ISL_5098130	A/turkey/Germany-NI/AI00612/2021	A / H5N8	Europe / Germany / Lower Saxony / Cloppenburg	Jacqueline King (Friedrich-Loeffler-Institut)
EPI_ISL_5098132	A/turkey/Germany-NI/AI00616/2021	A / H5N8	Europe / Germany / Lower Saxony / Cloppenburg	Jacqueline King (Friedrich-Loeffler-Institut)
EPI_ISL_5098133	A/turkey/Germany-NI/AI00591/2021	A / H5N8	Europe / Germany / Lower Saxony / Cloppenburg	Jacqueline King (Friedrich-Loeffler-Institut)
EPI_ISL_5098134	A/wild duck/Germany-NI/AI03479/2020	A / H5N8	Europe / Germany / Lower Saxony / Lüneburg	Jacqueline King (Friedrich-Loeffler-Institut)
EPI_ISL_5098135	A/kestrel/Germany-NI/AI03672/2020	A / H5N8	Europe / Germany / Lower Saxony / Friesland	Jacqueline King (Friedrich-Loeffler-Institut)
EPI_ISL_5098138	A/wild goose/Germany-NI/AI00626/2021	A / H5N8	Europe / Germany / Lower Saxony / Harburg	Jacqueline King (Friedrich-Loeffler-Institut)
EPI_ISL_5098139	A/wild goose/Germany-NI/AI00854/2021	A / H5N8	Europe / Germany / Lower Saxony / Lüchow-Dannenberg	Jacqueline King (Friedrich-Loeffler-Institut)
EPI_ISL_5098142	A/Hawaiian goose/Germany-RP/AI00856/2021	A / H5N8	Europe / Germany / Rhineland-Palatinate / Rhein-Pfalz-Kreis	Jacqueline King (Friedrich-Loeffler-Institut)
EPI_ISL_5098143	A/chicken/Germany-NI/AI00887/2021	A / H5N8	Europe / Germany / Lower Saxony / Cloppenburg	Jacqueline King (Friedrich-Loeffler-Institut)
EPI_ISL_5098144	A/pomeranian goose/Germany-MV/AI01100/2021	A / H5N8	Europe / Germany / Mecklenburg-Vorpommern / Vorpommern-Rügen	Jacqueline King (Friedrich-Loeffler-Institut)
EPI_ISL_5098145	A/turkey/Germany-BB/AI01120/2021	A / H5N8	Europe / Germany / Brandenburg / Uckermark	Jacqueline King (Friedrich-Loeffler-Institut)
EPI_ISL_5098146	A/chicken/Germany-MV/AI01347/2021	A / H5N8	Europe / Germany / Mecklenburg-Vorpommern / Landkreis Rostock	Jacqueline King (Friedrich-Loeffler-Institut)
EPI_ISL_5098147	A/wild goose/Germany-NI/AI03471/2020	A / H5N8	Europe / Germany / Lower Saxony / Friesland	Jacqueline King (Friedrich-Loeffler-Institut)
EPI_ISL_5098153	A/wild goose/Germany-NI/AI03101/2020	A / H5N8	Europe / Germany / Lower Saxony / Hannover	Jacqueline King (Friedrich-Loeffler-Institut)
EPI_ISL_5098154	A/turkey/Germany-MV/AI01127/2021	A / H5N8	Europe / Germany / Mecklenburg-Vorpommern / Vorpommern-Rügen	Jacqueline King (Friedrich-Loeffler-Institut)
EPI_ISL_5098158	A/turkey/Germany-BB/AI01419/2021	A / H5N8	Europe / Germany / Brandenburg / Prignitz	Jacqueline King (Friedrich-Loeffler-Institut)
EPI_ISL_5098159	A/domestic duck/Germany-BB/AI01423/2021	A / H5N8	Europe / Germany / Brandenburg / Märkisch-Oderland	Jacqueline King (Friedrich-Loeffler-Institut)
EPI_ISL_5098160	A/domestic duck/Germany-BB/AI01437/2021	A / H5N8	Europe / Germany / Brandenburg / Uckermark	Jacqueline King (Friedrich-Loeffler-Institut)
EPI_ISL_5099356	A/domestic duck/Germany-BB/AI01444/2021	A / H5N8	Europe / Germany / Brandenburg / Märkisch-Oderland	Jacqueline King (Friedrich-Loeffler-Institut)
EPI_ISL_5099367	A/chicken/Germany-BY/AI01355/2021	A / H5N8	Europe / Germany / Bavaria / Weißenburg-Gunzenhausen	Jacqueline King (Friedrich-Loeffler-Institut)
EPI_ISL_5099368	A/turkey/Germany-MV/AI01477/2021	A / H5N8	Europe / Germany / Mecklenburg-Vorpommern / Vorpommern-Rügen	Jacqueline King (Friedrich-Loeffler-Institut)
EPI_ISL_5099372	A/Laridae/Germany-SH/AI01498/2021	A / H5N4	Europe / Germany / Schleswig-Holstein / Ostholstein	Jacqueline King (Friedrich-Loeffler-Institut)
EPI_ISL_5099373	A/chicken/Germany-MV/AI01587/2021	A / H5N8	Europe / Germany / Mecklenburg-Vorpommern / Landkreis Rostock	Jacqueline King (Friedrich-Loeffler-Institut)
EPI_ISL_5099451	A/chicken/Germany-NI/AI01599/2021	A / H5N1	Europe / Germany / Lower Saxony / Aurich	Jacqueline King (Friedrich-Loeffler-Institut)
EPI_ISL_5099456	A/barnacle goose/Germany-NI/AI01605/2021	A / H5N1	Europe / Germany / Lower Saxony / Aurich	Jacqueline King (Friedrich-Loeffler-Institut)
EPI_ISL_5099462	A/chicken/Germany-BY/AI01617/2021	A / H5N8	Europe / Germany / Bavaria / Würzburg	Jacqueline King (Friedrich-Loeffler-Institut)
EPI_ISL_5099463	A/chicken/Germany-MV/AI01794/2021	A / H5N8	Europe / Germany / Mecklenburg-Vorpommern / Vorpommern-Greifswald	Jacqueline King (Friedrich-Loeffler-Institut)
EPI_ISL_5099468	A/turkey/Germany-NI/AI01799/2021	A / H5N8	Europe / Germany / Lower Saxony / Cloppenburg	Jacqueline King (Friedrich-Loeffler-Institut)
EPI_ISL_5099474	A/turkey/Germany-NI/AI01805/2021	A / H5N8	Europe / Germany / Lower Saxony / Cloppenburg	Jacqueline King (Friedrich-Loeffler-Institut)
EPI_ISL_5099476	A/chicken/Germany-NI/AI01815/2021	A / H5N8	Europe / Germany / Lower Saxony / Vechta	Jacqueline King (Friedrich-Loeffler-Institut)
EPI_ISL_5099628	A/domestic duck/Germany-BB/AI01443/2021	A / H5N8	Europe / Germany / Brandenburg / Märkisch-Oderland	Jacqueline King (Friedrich-Loeffler-Institut)
EPI_ISL_5100056	A/turkey/Germany-NI/AI02013/2021	A / H5N8	Europe / Germany / Lower Saxony / Diepholz	Jacqueline King (Friedrich-Loeffler-Institut)
EPI_ISL_5100599	A/turkey/Germany-NI/AI02025/2021	A / H5N8	Europe / Germany / Lower Saxony / Cloppenburg	Jacqueline King (Friedrich-Loeffler-Institut)
EPI_ISL_5102033	A/domestic duck/Germany-NW/AI02049/2021	A / H5N8	Europe / Germany / North Rhine-Westphalia / Gütersloh	Jacqueline King (Friedrich-Loeffler-Institut)
EPI_ISL_5102130	A/chicken/Germany-NW/AI02054/2021	A / H5N8	Europe / Germany / North Rhine-Westphalia / Paderborn	Jacqueline King (Friedrich-Loeffler-Institut)
EPI_ISL_5102132	A/chicken/Germany-BY/AI02064/2021	A / H5N8	Europe / Germany / Bavaria / Würzburg	Jacqueline King (Friedrich-Loeffler-Institut)
EPI_ISL_5102136	A/chicken/Germany-BY/AI02066/2021	A / H5N8	Europe / Germany / Bavaria / Schwandorf	Jacqueline King (Friedrich-Loeffler-Institut)
EPI_ISL_5102449	A/white-tailed eagle/Germany-SH/AI02170/2020	A / H5N8	Europe / Germany / Schleswig-Holstein / Nordfriesland	Jacqueline King (Friedrich-Loeffler-Institut)
EPI_ISL_5102578	A/chicken/Germany-MV/AI01407/2021	A / H5N8	Europe / Germany / Mecklenburg-Vorpommern / Mecklenburgische Seenplatte	Jacqueline King (Friedrich-Loeffler-Institut)
EPI_ISL_5115613	A/chicken/Germany-MV/AI01965/2021	A / H5N8	Europe / Germany / Mecklenburg-Vorpommern / Mecklenburgische Seenplatte	Jacqueline King (Friedrich-Loeffler-Institut)
EPI_ISL_5115614	A/chicken/Germany-BE/AI01974/2021	A / H5N8	Europe / Germany / Berlin / Berlin,Stadt	Jacqueline King (Friedrich-Loeffler-Institut)
EPI_ISL_5115615	A/turkey/Germany-BB/AI01980/2021	A / H5N8	Europe / Germany / Brandenburg / Prignitz	Jacqueline King (Friedrich-Loeffler-Institut)
EPI_ISL_5115616	A/domestic goose/Germany-SH/AI02100/2021	A / H5N8	Europe / Germany / Schleswig-Holstein / Rendsburg-Eckernförde	Jacqueline King (Friedrich-Loeffler-Institut)
EPI_ISL_5115617	A/domestic goose/Germany-SH/AI02102/2021	A / H5N8	Europe / Germany / Schleswig-Holstein / Stormarn	Jacqueline King (Friedrich-Loeffler-Institut)
EPI_ISL_5115618	A/turkey/Germany-BB/AI02117/2021	A / H5N8	Europe / Germany / Brandenburg / Märkisch-Oderland	Jacqueline King (Friedrich-Loeffler-Institut)
EPI_ISL_5115619	A/turkey/Germany-NI/AI02122/2021	A / H5N8	Europe / Germany / Lower Saxony / Diepholz	Jacqueline King (Friedrich-Loeffler-Institut)
EPI_ISL_5115620	A/turkey/Germany-NI/AI02128/2021	A / H5N8	Europe / Germany / Lower Saxony / Cloppenburg	Jacqueline King (Friedrich-Loeffler-Institut)
EPI_ISL_5115621	A/chicken/Germany-MV/AI02141/2021	A / H5N8	Europe / Germany / Mecklenburg-Vorpommern / Vorpommern-Greifswald	Jacqueline King (Friedrich-Loeffler-Institut)
EPI_ISL_5115763	A/turkey/Germany-NW/AI02290/2021	A / H5N8	Europe / Germany / North Rhine-Westphalia / Minden-Lübbecke	Jacqueline King (Friedrich-Loeffler-Institut)
EPI_ISL_5115955	A/turkey/Germany-NI/AI02303/2021	A / H5N8	Europe / Germany / Lower Saxony / Cloppenburg	Jacqueline King (Friedrich-Loeffler-Institut)
EPI_ISL_5115956	A/turkey/Germany-NI/AI02306/2021	A / H5N8	Europe / Germany / Lower Saxony / Cloppenburg	Jacqueline King (Friedrich-Loeffler-Institut)

EPI_ISL_5115967	A/chicken/Germany-SH/AI02312/2021	A / H5N8	Europe / Germany / Schleswig-Holstein / Vechta Plön	Jacqueline King (Friedrich-Loeffler-Institut)
EPI_ISL_5115968	A/turkey/Germany-BB/AI02318/2021	A / H5N8	Europe / Germany / Brandenburg / Märkisch-Oderland	Jacqueline King (Friedrich-Loeffler-Institut)
EPI_ISL_5115969	A/domestic goose/Germany-MV/AI02319/2021	A / H5N8	Europe / Germany / Mecklenburg-Vorpommern / Vorpommern-Rügen	Jacqueline King (Friedrich-Loeffler-Institut)
EPI_ISL_5115970	A/chicken/Germany-NI/AI02328/2021	A / H5N8	Europe / Germany / Lower Saxony / Vechta	Jacqueline King (Friedrich-Loeffler-Institut)
EPI_ISL_5116063	A/swan/Germany-HE/AI02335/2021	A / H5N8	Europe / Germany / Hessen / Offenbach a. Main	Jacqueline King (Friedrich-Loeffler-Institut)
EPI_ISL_5116064	A/turkey/Germany-NI/AI02349/2021	A / H5N8	Europe / Germany / Lower Saxony / Cloppenburg	Jacqueline King (Friedrich-Loeffler-Institut)
EPI_ISL_5116065	A/greater rhea/Germany-SN/AI02351/2021	A / H5N8	Europe / Germany / Saxony / Zwickau	Jacqueline King (Friedrich-Loeffler-Institut)
EPI_ISL_5116093	A/chicken/Germany-SN/AI02353/2021	A / H5N8	Europe / Germany / Saxony / Mittelsachsen	Jacqueline King (Friedrich-Loeffler-Institut)
EPI_ISL_5116172	A/mute swan/Germany-HE/AI02373/2021	A / H5N8	Europe / Germany / Hessen / Frankfurt a. Main	Jacqueline King (Friedrich-Loeffler-Institut)
EPI_ISL_5116326	A/chicken/Germany-MV/AI02376/2021	A / H5N8	Europe / Germany / Mecklenburg-Vorpommern / Cloppenburg Vorpommern-Rügen	Jacqueline King (Friedrich-Loeffler-Institut)
EPI_ISL_5123578	A/domestic goose/Germany-NI/AI03093/2021	A / H5N8	Europe / Germany / Lower Saxony / Vechta	Jacqueline King (Friedrich-Loeffler-Institut)
EPI_ISL_5123579	A/domestic duck/Germany-NI/AI03099/2021	A / H5N8	Europe / Germany / Lower Saxony / Cloppenburg	Jacqueline King (Friedrich-Loeffler-Institut)
EPI_ISL_5123584	A/turkey/Germany-NI/AI03126/2021	A / H5N8	Europe / Germany / Lower Saxony / Vechta	Jacqueline King (Friedrich-Loeffler-Institut)
EPI_ISL_5123589	A/turkey/Germany-NI/AI02615/2021	A / H5N8	Europe / Germany / Lower Saxony / Cloppenburg	Jacqueline King (Friedrich-Loeffler-Institut)
EPI_ISL_5123590	A/domestic duck/Germany-BB/AI02599/2021	A / H5N8	Europe / Germany / Brandenburg / Oder-Spree	Jacqueline King (Friedrich-Loeffler-Institut)
EPI_ISL_5123847	A/chicken/Germany-SH/AI02562/2021	A / H5N8	Europe / Germany / Schleswig-Holstein / Plön	Jacqueline King (Friedrich-Loeffler-Institut)
EPI_ISL_5126214	A/eagle owl/Germany-ST/AI02542/2021	A / H5N8	Europe / Germany / Saxony-Anhalt / Altmarkkreis Salzwedel	Jacqueline King (Friedrich-Loeffler-Institut)
EPI_ISL_5133724	A/chicken/Germany-NI/AI02954/2021	A / H5N8	Europe / Germany / Lower Saxony / Diepholz	Jacqueline King (Friedrich-Loeffler-Institut)
EPI_ISL_5134186	A/turkey/Germany-NI/AI02950/2021	A / H5N8	Europe / Germany / Lower Saxony / Vechta	Jacqueline King (Friedrich-Loeffler-Institut)
EPI_ISL_5134658	A/turkey/Germany-NI/AI02943/2021	A / H5N8	Europe / Germany / Lower Saxony / Ammerland	Jacqueline King (Friedrich-Loeffler-Institut)
EPI_ISL_5135087	A/chicken/Germany-NI/AI02942/2021	A / H5N8	Europe / Germany / Lower Saxony / Cloppenburg	Jacqueline King (Friedrich-Loeffler-Institut)
EPI_ISL_5136192	A/turkey/Germany-NI/AI02933/2021	A / H5N8	Europe / Germany / Lower Saxony / Cloppenburg	Jacqueline King (Friedrich-Loeffler-Institut)
EPI_ISL_5136837	A/turkey/Germany-NI/AI02671/2021	A / H5N8	Europe / Germany / Lower Saxony / Cloppenburg	Jacqueline King (Friedrich-Loeffler-Institut)
EPI_ISL_5137351	A/turkey/Germany-NI/AI02678/2021	A / H5N8	Europe / Germany / Lower Saxony / Cloppenburg	Jacqueline King (Friedrich-Loeffler-Institut)
EPI_ISL_5137952	A/turkey/Germany-NI/AI02674/2021	A / H5N8	Europe / Germany / Lower Saxony / Cloppenburg	Jacqueline King (Friedrich-Loeffler-Institut)
EPI_ISL_5139441	A/turkey/Germany-NI/AI02663/2021	A / H5N8	Europe / Germany / Lower Saxony / Vechta	Jacqueline King (Friedrich-Loeffler-Institut)
EPI_ISL_5141340	A/domestic duck/Germany-NI/AI02660/2021	A / H5N8	Europe / Germany / Lower Saxony / Vechta	Jacqueline King (Friedrich-Loeffler-Institut)
EPI_ISL_5142451	A/turkey/Germany-NW/AI03104/2021	A / H5N8	Europe / Germany / North Rhine-Westphalia / Hochsauerlandkreis	Jacqueline King (Friedrich-Loeffler-Institut)
EPI_ISL_5142455	A/turkey/Germany-NI/AI02991/2021	A / H5N8	Europe / Germany / Lower Saxony / Cloppenburg	Jacqueline King (Friedrich-Loeffler-Institut)
EPI_ISL_5142488	A/chicken/Germany-ST/AI02967/2021	A / H5N8	Europe / Germany / Saxony-Anhalt / Altmarkkreis Salzwedel	Jacqueline King (Friedrich-Loeffler-Institut)
EPI_ISL_5142489	A/turkey/Germany-NI/AI02553/2021	A / H5N8	Europe / Germany / Lower Saxony / Cloppenburg	Jacqueline King (Friedrich-Loeffler-Institut)
EPI_ISL_5142894	A/chicken/Germany-NI/AI02543/2021	A / H5N8	Europe / Germany / Lower Saxony / Wesermarsch	Jacqueline King (Friedrich-Loeffler-Institut)
EPI_ISL_5142961	A/turkey/Germany-MV/AI02458/2021	A / H5N8	Europe / Germany / Mecklenburg-Vorpommern / Vorpommern-Rügen	Jacqueline King (Friedrich-Loeffler-Institut)
EPI_ISL_5143100	A/turkey/Germany-NI/AI02424/2021	A / H5N8	Europe / Germany / Lower Saxony / Cloppenburg	Jacqueline King (Friedrich-Loeffler-Institut)
EPI_ISL_5143300	A/domestic goose/Germany-MV/AI02558/2021	A / H5N8	Europe / Germany / Mecklenburg-Vorpommern / Vorpommern-Rügen	Jacqueline King (Friedrich-Loeffler-Institut)
EPI_ISL_5143417	A/turkey/Germany-BB/AI02434/2021	A / H5N8	Europe / Germany / Brandenburg / Märkisch-Oderland	Jacqueline King (Friedrich-Loeffler-Institut)
EPI_ISL_5143554	A/chicken/Germany-NI/AI02412/2021	A / H5N8	Europe / Germany / Lower Saxony / Cuxhaven	Jacqueline King (Friedrich-Loeffler-Institut)
EPI_ISL_5143583	A/chicken/Germany-TH/AI03513/2021	A / H5N8	Europe / Germany / Thuringia / Sömmerda	Jacqueline King (Friedrich-Loeffler-Institut)
EPI_ISL_5143584	A/chicken/Germany-BY/AI03544/2021	A / H5N8	Europe / Germany / Bavaria / Ebersberg	Jacqueline King (Friedrich-Loeffler-Institut)
EPI_ISL_5143585	A/turkey/Germany-MV/AI03535/2021	A / H5N8	Europe / Germany / Mecklenburg-Vorpommern / Landkreis Rostock	Jacqueline King (Friedrich-Loeffler-Institut)
EPI_ISL_5143586	A/chicken/Germany-SN/AI03496/2021	A / H5N8	Europe / Germany / Saxony / Mittelsachsen	Jacqueline King (Friedrich-Loeffler-Institut)
EPI_ISL_5143587	A/chicken/Germany-SN/AI03502/2021	A / H5N8	Europe / Germany / Saxony / Vogtlandkreis	Jacqueline King (Friedrich-Loeffler-Institut)
EPI_ISL_5143626	A/chicken/Germany-TH/AI03193/2021	A / H5N8	Europe / Germany / Thuringia / Weimar-Land	Jacqueline King (Friedrich-Loeffler-Institut)
EPI_ISL_5143627	A/turkey/Germany-NI/AI03209/2021	A / H5N8	Europe / Germany / Lower Saxony / Cloppenburg	Jacqueline King (Friedrich-Loeffler-Institut)
EPI_ISL_5143820	A/chicken/Germany-BW/AI03205/2021	A / H5N8	Europe / Germany / Baden-Wuerttemberg / Breisgau-Hochschwarzwald	Jacqueline King (Friedrich-Loeffler-Institut)
EPI_ISL_5143995	A/domestic duck/Germany-ST/AI03183/2021	A / H5N8	Europe / Germany / Saxony-Anhalt / Saalekreis	Jacqueline King (Friedrich-Loeffler-Institut)
EPI_ISL_5144149	A/turkey/Germany-NI/AI03160/2021	A / H5N8	Europe / Germany / Lower Saxony / Cloppenburg	Jacqueline King (Friedrich-Loeffler-Institut)
EPI_ISL_5144341	A/chicken/Germany-NW/AI03148/2021	A / H5N8	Europe / Germany / North Rhine-Westphalia / Warendorf	Jacqueline King (Friedrich-Loeffler-Institut)
EPI_ISL_5144368	A/domestic duck/Germany-NI/AI03088/2021	A / H5N8	Europe / Germany / Lower Saxony / Cloppenburg	Jacqueline King (Friedrich-Loeffler-Institut)
EPI_ISL_5144407	A/chicken/Germany-NW/AI03154/2021	A / H5N8	Europe / Germany / North Rhine-Westphalia / Paderborn	Jacqueline King (Friedrich-Loeffler-Institut)
EPI_ISL_5144529	A/domestic goose/Germany-NI/AI03142/2021	A / H5N8	Europe / Germany / Lower Saxony / Vechta	Jacqueline King (Friedrich-Loeffler-Institut)
EPI_ISL_5144632	A/turkey/Germany-NI/AI02393/2021	A / H5N8	Europe / Germany / Lower Saxony / Cloppenburg	Jacqueline King (Friedrich-Loeffler-Institut)
EPI_ISL_5144725	A/domestic goose/Germany-NI/AI02980/2021	A / H5N8	Europe / Germany / Lower Saxony / Vechta	Jacqueline King (Friedrich-Loeffler-Institut)
EPI_ISL_5144778	A/turkey/Germany-NI/AI01488/2021	A / H5N8	Europe / Germany / Lower Saxony / Cloppenburg	Jacqueline King (Friedrich-Loeffler-Institut)
EPI_ISL_5144920	A/turkey/Germany-NI/AI01483/2021	A / H5N8	Europe / Germany / Lower Saxony / Diepholz	Jacqueline King (Friedrich-Loeffler-Institut)
EPI_ISL_5145223	A/turkey/Germany-NI/AI04455/2021	A / H5N1	Europe / Germany / Lower Saxony / Emsland	Jacqueline King (Friedrich-Loeffler-Institut)
EPI_ISL_5145422	A/barnacle goose/Germany-NI/AI03914/2021	A / H5N1	Europe / Germany / Lower Saxony / Leer	Jacqueline King (Friedrich-Loeffler-Institut)
EPI_ISL_5145613	A/chicken/Germany-BY/AI02060/2021	A / H5N8	Europe / Germany / Bavaria / Würzburg	Jacqueline King (Friedrich-Loeffler-Institut)
EPI_ISL_5145744	A/oystercatcher/Germany-NI/AI05047/2021	A / H5N1	Europe / Germany / Lower Saxony / Aurich	Jacqueline King (Friedrich-Loeffler-Institut)
EPI_ISL_5145796	A/swan/Germany-NI/AI05045/2021	A / H5N8	Europe / Germany / Lower Saxony / Osnabrück	Jacqueline King (Friedrich-Loeffler-Institut)
EPI_ISL_5145858	A/chicken/Germany-TH/AI03512/2021	A / H5N8	Europe / Germany / Thuringia / Saale-Holzland-Kreis	Jacqueline King (Friedrich-Loeffler-Institut)
EPI_ISL_5145921	A/chicken/Germany-BW/AI03554/2021	A / H5N8	Europe / Germany / Baden-Wuerttemberg / Böblingen	Jacqueline King (Friedrich-Loeffler-Institut)
EPI_ISL_5146171	A/chicken/Germany-BW/AI03634/2021	A / H5N8	Europe / Germany / Baden-Wuerttemberg / Rottweil	Jacqueline King (Friedrich-Loeffler-Institut)
EPI_ISL_5146288	A/chicken/Germany-NW/AI03705/2021	A / H5N8	Europe / Germany / North Rhine-Westphalia / Märkischer Kreis	Jacqueline King (Friedrich-Loeffler-Institut)
EPI_ISL_5146291	A/barnacle goose/Germany-NI/AI03980/2021	A / H5N1	Europe / Germany / Lower Saxony / Leer	Jacqueline King (Friedrich-Loeffler-Institut)
EPI_ISL_5146292	A/turkey/Germany-NI/AI04373/2021	A / H5N1	Europe / Germany / Lower Saxony / Emsland	Jacqueline King (Friedrich-Loeffler-Institut)
EPI_ISL_5146481	A/turkey/Germany-NI/AI04425/2021	A / H5N1	Europe / Germany / Lower Saxony / Emsland	Jacqueline King (Friedrich-Loeffler-Institut)
EPI_ISL_5159426	A/Hunan/10117/2021	A / H5N6	Asia / China / Hunan	Lei Yang (WHO Chinese National Influenza Center / Virology Institute, Chinese CDC)
EPI_ISL_5234711	A/duck/Laos/NL-2175410/2021	A / H5N6	Asia / Lao, People's Democratic Republic	Yunho Jang (Centers for Disease Control and Prevention / WHO Collaborating Center for Surveillance, Epidemiology and Control of Infl
EPI_ISL_5234724	A/duck/Laos/NL-2175411/2021	A / H5N6	Asia / Lao, People's Democratic Republic	Yunho Jang (Centers for Disease Control and Prevention / WHO Collaborating Center for Surveillance, Epidemiology and Control of Infl
EPI_ISL_5234816	A/duck/Laos/NL-2175412/2021	A / H5N6	Asia / Lao, People's Democratic Republic	Yunho Jang (Centers for Disease Control and Prevention / WHO Collaborating Center for Surveillance, Epidemiology and Control of Infl
EPI_ISL_525439	A/laying_hen/Poland/002/2020	A / H5N8	Europe / Poland / Greater Poland Voivodeship	Edyta ?wi?to? (National Veterinary Research Institut Poland, PIWet-PIB)

EPI_ISL_525440	A/chicken/Poland/003/2020	A / H5N8	Europe / Poland / Lublin Voivodeship	Edyta ?wi?to? (National Veterinary Research Institut Poland, PIWet-PIB)
EPI_ISL_525441	A/chicken/Poland/004/2020	A / H5N8	Europe / Poland / Lublin Voivodeship	Edyta ?wi?to? (National Veterinary Research Institut Poland, PIWet-PIB)
EPI_ISL_525442	A/turkey/Poland/027/2020	A / H5N8	Europe / Poland	Edyta ?wi?to? (National Veterinary Research Institut Poland, PIWet-PIB)
EPI_ISL_525443	A/domestic_goose/Poland/028/2020	A / H5N8	Europe / Poland	Edyta ?wi?to? (National Veterinary Research Institut Poland, PIWet-PIB)
EPI_ISL_525444	A/chicken/Poland/054/2020	A / H5N8	Europe / Poland	Edyta ?wi?to? (National Veterinary Research Institut Poland, PIWet-PIB)
EPI_ISL_525445	A/turkey/Poland/079/2020	A / H5N8	Europe / Poland	Edyta ?wi?to? (National Veterinary Research Institut Poland, PIWet-PIB)
EPI_ISL_525446	A/laying_hen/Poland/095/2020	A / H5N8	Europe / Poland	Edyta ?wi?to? (National Veterinary Research Institut Poland, PIWet-PIB)
EPI_ISL_525447	A/turkey/Poland/096/2020	A / H5N8	Europe / Poland	Edyta ?wi?to? (National Veterinary Research Institut Poland, PIWet-PIB)
EPI_ISL_525448	A/turkey/Poland/182/2020	A / H5N8	Europe / Poland	Edyta ?wi?to? (National Veterinary Research Institut Poland, PIWet-PIB)
EPI_ISL_525449	A/domestic_duck/Poland/219/2020	A / H5N8	Europe / Poland	Edyta ?wi?to? (National Veterinary Research Institut Poland, PIWet-PIB)
EPI_ISL_525450	A/domestic_duck/Poland/221/2020	A / H5N8	Europe / Poland	Edyta ?wi?to? (National Veterinary Research Institut Poland, PIWet-PIB)
EPI_ISL_525451	A/domestic_duck/Poland/222/2020	A / H5N8	Europe / Poland	Edyta ?wi?to? (National Veterinary Research Institut Poland, PIWet-PIB)
EPI_ISL_525452	A/domestic_duck/Poland/223/2020	A / H5N8	Europe / Poland	Edyta ?wi?to? (National Veterinary Research Institut Poland, PIWet-PIB)
EPI_ISL_525453	A/domestic_duck/Poland/229/2020	A / H5N8	Europe / Poland	Edyta ?wi?to? (National Veterinary Research Institut Poland, PIWet-PIB)
EPI_ISL_525454	A/domestic_duck/Poland/230/2020	A / H5N8	Europe / Poland	Edyta ?wi?to? (National Veterinary Research Institut Poland, PIWet-PIB)
EPI_ISL_525455	A/domestic_duck/Poland/237/2020	A / H5N8	Europe / Poland	Edyta ?wi?to? (National Veterinary Research Institut Poland, PIWet-PIB)
EPI_ISL_525459	A/laying_hen/Poland/312/2020	A / H5N8	Europe / Poland	Edyta ?wi?to? (National Veterinary Research Institut Poland, PIWet-PIB)
EPI_ISL_525461	A/turkey/Poland/366/2020	A / H5N8	Europe / Poland	Edyta ?wi?to? (National Veterinary Research Institut Poland, PIWet-PIB)
EPI_ISL_525462	A/domestic_goose/Poland/274/2020	A / H5N8	Europe / Poland	Edyta ?wi?to? (National Veterinary Research Institut Poland, PIWet-PIB)
EPI_ISL_525463	A/domestic_duck/Poland/285/2020	A / H5N8	Europe / Poland	Edyta ?wi?to? (National Veterinary Research Institut Poland, PIWet-PIB)
EPI_ISL_525464	A/domestic_duck/Poland/263/2020	A / H5N8	Europe / Poland	Edyta ?wi?to? (National Veterinary Research Institut Poland, PIWet-PIB)
EPI_ISL_525465	A/domestic_duck/Poland/271/2020	A / H5N8	Europe / Poland	Edyta ?wi?to? (National Veterinary Research Institut Poland, PIWet-PIB)
EPI_ISL_5260416	A/black-backed gull/Shandong/SC189/2021	A / H5N8	Asia / China / Shandong	Pengfei Cui (Harbin Veterinary Research Institute (CAAS) / Ministry of Agriculture)
EPI_ISL_5260417	A/brown-headed gull/Tibet/1-1/2021	A / H5N8	Asia / China / Tibet	Pengfei Cui (Harbin Veterinary Research Institute (CAAS) / Ministry of Agriculture)
EPI_ISL_5260418	A/black swan/Beijing/1/2021	A / H5N8	Asia / China / Beijing	Pengfei Cui (Harbin Veterinary Research Institute (CAAS) / Ministry of Agriculture)
EPI_ISL_5260419	A/duck/Guangdong/S1269/2021	A / H5N8	Asia / China / Guangdong	Pengfei Cui (Harbin Veterinary Research Institute (CAAS) / Ministry of Agriculture)
EPI_ISL_5260420	A/duck/Guangxi/S10099/2021	A / H5N8	Asia / China / Guangxi	Pengfei Cui (Harbin Veterinary Research Institute (CAAS) / Ministry of Agriculture)
EPI_ISL_5260421	A/duck/Guangxi/S10263/2021	A / H5N8	Asia / China / Guangxi	Pengfei Cui (Harbin Veterinary Research Institute (CAAS) / Ministry of Agriculture)
EPI_ISL_5260422	A/duck/Guangxi/S11043/2021	A / H5N8	Asia / China / Guangxi	Pengfei Cui (Harbin Veterinary Research Institute (CAAS) / Ministry of Agriculture)
EPI_ISL_5260426	A/duck/Guangxi/S21194/2021	A / H5N8	Asia / China / Guangxi	Pengfei Cui (Harbin Veterinary Research Institute (CAAS) / Ministry of Agriculture)
EPI_ISL_5260435	A/duck/Henan/S1381/2021	A / H5N8	Asia / China / Henan	Pengfei Cui (Harbin Veterinary Research Institute (CAAS) / Ministry of Agriculture)
EPI_ISL_5260444	A/duck/Jiangxi/S10252/2021	A / H5N8	Asia / China / Jiangxi	Pengfei Cui (Harbin Veterinary Research Institute (CAAS) / Ministry of Agriculture)
EPI_ISL_5260445	A/duck/Hebei/S1070/2021	A / H5N8	Asia / China / Hebei	Pengfei Cui (Harbin Veterinary Research Institute (CAAS) / Ministry of Agriculture)
EPI_ISL_5260446	A/goose/Guangxi/S20601/2021	A / H5N8	Asia / China / Guangxi	Pengfei Cui (Harbin Veterinary Research Institute (CAAS) / Ministry of Agriculture)
EPI_ISL_5260447	A/goose/Henan/S1315/2021	A / H5N8	Asia / China / Henan	Pengfei Cui (Harbin Veterinary Research Institute (CAAS) / Ministry of Agriculture)
EPI_ISL_5260448	A/goose/Hunan/S11288/2021	A / H5N8	Asia / China / Hunan	Pengfei Cui (Harbin Veterinary Research Institute (CAAS) / Ministry of Agriculture)
EPI_ISL_5260449	A/goose/Jiangsu/S1385/2021	A / H5N8	Asia / China / Jiangsu	Pengfei Cui (Harbin Veterinary Research Institute (CAAS) / Ministry of Agriculture)
EPI_ISL_5260450	A/goose/Zhejiang/S1266/2021	A / H5N8	Asia / China / Zhejiang	Pengfei Cui (Harbin Veterinary Research Institute (CAAS) / Ministry of Agriculture)
EPI_ISL_5260451	A/goose/Liaoning/S1266/2021	A / H5N8	Asia / China / Liaoning	Pengfei Cui (Harbin Veterinary Research Institute (CAAS) / Ministry of Agriculture)
EPI_ISL_5260452	A/green-winged teal/Guangdong/SD004/2021	A / H5N8	Asia / China / Guangdong	Pengfei Cui (Harbin Veterinary Research Institute (CAAS) / Ministry of Agriculture)
EPI_ISL_5260453	A/mute swan/Shandong/1/2021	A / H5N8	Asia / China / Shandong	Pengfei Cui (Harbin Veterinary Research Institute (CAAS) / Ministry of Agriculture)
EPI_ISL_5260454	A/wild duck/Shandong/SC177/2021	A / H5N8	Asia / China / Shandong	Pengfei Cui (Harbin Veterinary Research Institute (CAAS) / Ministry of Agriculture)
EPI_ISL_5260455	A/whooper swan/Shandong/SC176/2021	A / H5N8	Asia / China / Shandong	Pengfei Cui (Harbin Veterinary Research Institute (CAAS) / Ministry of Agriculture)
EPI_ISL_5260456	A/whooper swan/Shandong/SC185/2021	A / H5N8	Asia / China / Shandong	Pengfei Cui (Harbin Veterinary Research Institute (CAAS) / Ministry of Agriculture)
EPI_ISL_5260457	A/whooper swan/Shandong/SC195/2021	A / H5N8	Asia / China / Shandong	Pengfei Cui (Harbin Veterinary Research Institute (CAAS) / Ministry of Agriculture)
EPI_ISL_5260458	A/whooper swan/Shandong/SC198/2021	A / H5N8	Asia / China / Shandong	Pengfei Cui (Harbin Veterinary Research Institute (CAAS) / Ministry of Agriculture)
EPI_ISL_5260459	A/whooper swan/Shandong/SC199/2021	A / H5N8	Asia / China / Shandong	Pengfei Cui (Harbin Veterinary Research Institute (CAAS) / Ministry of Agriculture)
EPI_ISL_5260460	A/whooper swan/Shandong/SC200/2021	A / H5N8	Asia / China / Shandong	Pengfei Cui (Harbin Veterinary Research Institute (CAAS) / Ministry of Agriculture)
EPI_ISL_5260461	A/whooper swan/Shandong/SC188/2021	A / H5N8	Asia / China / Shandong	Pengfei Cui (Harbin Veterinary Research Institute (CAAS) / Ministry of Agriculture)
EPI_ISL_5260462	A/whooper swan/Shanxi/4-1/2020	A / H5N8	Asia / China / Shanxi	Pengfei Cui (Harbin Veterinary Research Institute (CAAS) / Ministry of Agriculture)
EPI_ISL_5260463	A/whooper swan/Shanxi/4-2/2020	A / H5N8	Asia / China / Shanxi	Pengfei Cui (Harbin Veterinary Research Institute (CAAS) / Ministry of Agriculture)
EPI_ISL_5260464	A/common coot/Shandong/SC197/2021	A / H5N8	Asia / China / Shandong	Pengfei Cui (Harbin Veterinary Research Institute (CAAS) / Ministry of Agriculture)
EPI_ISL_5260465	A/egret/Jiangsu/SD021/2021	A / H5N8	Asia / China / Jiangsu	Pengfei Cui (Harbin Veterinary Research Institute (CAAS) / Ministry of Agriculture)
EPI_ISL_5260466	A/grebe/Shandong/SC184/2021	A / H5N8	Asia / China / Shandong	Pengfei Cui (Harbin Veterinary Research Institute (CAAS) / Ministry of Agriculture)
EPI_ISL_5260467	A/grebe/Shaanxi/SD001/2021	A / H5N8	Asia / China / Shaanxi	Pengfei Cui (Harbin Veterinary Research Institute (CAAS) / Ministry of Agriculture)
EPI_ISL_5260468	A/grebe/Ningxia/SD001/2021	A / H5N8	Asia / China / Ningxia	Pengfei Cui (Harbin Veterinary Research Institute (CAAS) / Ministry of Agriculture)
EPI_ISL_5260469	A/wild duck/Jiangsu/SD019/2021	A / H5N8	Asia / China / Jiangsu	Pengfei Cui (Harbin Veterinary Research Institute (CAAS) / Ministry of Agriculture)
EPI_ISL_5260470	A/wild goose/Shandong/SC196/2021	A / H5N8	Asia / China / Shandong	Pengfei Cui (Harbin Veterinary Research Institute (CAAS) / Ministry of Agriculture)
EPI_ISL_529179	A/duck/Netherlands/16014829-001005/2016	A / H5N8	Europe / Netherlands / Provincie Flevoland / Gemeente Dronten / Biddinghuizen	Rene Heutink (Wageningen Bioveterinary Research)
EPI_ISL_5323346	A/goose/Czech Republic/18520-1/2021	A / H5N1	Europe / Czech Republic / Stredocesky Kraj / Okres Pribram / Trhove Dusniky; Zip Code:26101	Alexander Nagy (State Veterinary Institute Prague)
EPI_ISL_5323347	A/duck/Czech Republic/18520-2/2021	A / H5N1	Europe / Czech Republic / Stredocesky Kraj / Okres Pribram / Trhove Dusniky; Zip Code:26101	Alexander Nagy (State Veterinary Institute Prague)
EPI_ISL_5403566	A/Eurasian wigeon/Germany-SH/AI05948/2021	A / H5N1	Europe / Germany / Schleswig-Holstein / Nordfriesland	Jacqueline King (Friedrich-Loeffler-Institut)
EPI_ISL_5449305	A/barnacle_goose/Denmark/17572-1.01/2021-03-01	A / H5N1	Europe / Denmark / Region Sjaland / Slagelse Kommune	Charlotte Kristiane Hjulsager (Statens Serum Institute / Microbiological Diagnostic and Virology)
EPI_ISL_5463793	A/chicken/Tyumen/27-31V/2021	A / H5N1	Europe / Russian Federation / Tyumen Oblast / Tyumen	Natalia Goncharova (State Research Center of Virology and Biotechnology (VECTOR) / Emerging Zoonotic Diseases and Influenza)
EPI_ISL_5463794	A/chicken/Tyumen/27-39V/2021	A / H5N1	Europe / Russian Federation / Tyumen Oblast / Tyumen	Natalia Goncharova (State Research Center of Virology and Biotechnology (VECTOR) / Emerging Zoonotic Diseases and Influenza)
EPI_ISL_5463795	A/chicken/Tyumen/27-40V/2021	A / H5N1	Europe / Russian Federation / Tyumen Oblast / Tyumen	Natalia Goncharova (State Research Center of Virology and Biotechnology (VECTOR) / Emerging Zoonotic Diseases and Influenza)
EPI_ISL_5463796	A/chicken/Tyumen/27-42V/2021	A / H5N1	Europe / Russian Federation / Tyumen Oblast / Tyumen	Natalia Goncharova (State Research Center of Virology and Biotechnology (VECTOR) / Emerging Zoonotic Diseases and Influenza)
EPI_ISL_5463797	A/duck/Saratov/29-02V/2021	A / H5N1	Europe / Russian Federation / Saratov Oblast / Saratov	Natalia Goncharova (State Research Center of Virology and Biotechnology (VECTOR) / Emerging Zoonotic Diseases and Influenza)
EPI_ISL_5463798	A/duck/Saratov/29-03V/2021	A / H5N1	Europe / Russian Federation / Saratov Oblast / Saratov	Natalia Goncharova (State Research Center of Virology and Biotechnology (VECTOR) / Emerging Zoonotic Diseases and Influenza)
EPI_ISL_5463799	A/duck/Saratov/29-04V/2021	A / H5N1	Europe / Russian Federation / Saratov Oblast / Saratov	Natalia Goncharova (State Research Center of Virology and Biotechnology (VECTOR) / Emerging Zoonotic Diseases and Influenza)
EPI_ISL_5463800	A/chicken/Saratov/29-06V/2021	A / H5N1	Europe / Russian Federation / Saratov Oblast / Saratov	Natalia Goncharova (State Research Center of Virology and Biotechnology (VECTOR) / Emerging Zoonotic Diseases and Influenza)
EPI_ISL_5463801	A/chicken/Saratov/29-07V/2021	A / H5N1	Europe / Russian Federation / Saratov Oblast / Saratov	Natalia Goncharova (State Research Center of Virology and Biotechnology (VECTOR) / Emerging Zoonotic Diseases and Influenza)

EPI_ISL_5463802	A/duck/Saratov/29-08V/2021	A / H5N1	Europe / Russian Federation / Saratov Oblast / Saratov	Natalia Goncharova (State Research Center of Virology and Biotechnology (VECTOR) / Emerging Zoonotic Diseases and Influenza)
EPI_ISL_5463803	A/duck/Saratov/29-11V/2021	A / H5N1	Europe / Russian Federation / Saratov Oblast / Saratov	Natalia Goncharova (State Research Center of Virology and Biotechnology (VECTOR) / Emerging Zoonotic Diseases and Influenza)
EPI_ISL_5463804	A/chicken/Tyumen/33-45V/2021	A / H5N1	Europe / Russian Federation / Tyumen Oblast / Tyumen	Natalia Goncharova (State Research Center of Virology and Biotechnology (VECTOR) / Emerging Zoonotic Diseases and Influenza)
EPI_ISL_5463805	A/goose/Tyumen/33-52V/2021	A / H5N1	Europe / Russian Federation / Tyumen Oblast / Tyumen	Natalia Goncharova (State Research Center of Virology and Biotechnology (VECTOR) / Emerging Zoonotic Diseases and Influenza)
EPI_ISL_5463806	A/goose/Tyumen/33-53V/2021	A / H5N1	Europe / Russian Federation / Tyumen Oblast / Tyumen	Natalia Goncharova (State Research Center of Virology and Biotechnology (VECTOR) / Emerging Zoonotic Diseases and Influenza)
EPI_ISL_5524384	A/turkey/Italy/21VIR8585-1/2021	A / H5N1	Europe / Italy / Veneto / Province of Verona / Ronco All'Adige	Bianca Zecchin (Istituto Zooprofilattico Sperimentale Delle Venezie)
EPI_ISL_5530613	A/great_skua/Scotland/041672/2021	A / H5N1	Europe / United Kingdom / Western Isles / Flannan Isles	Alex Byrne (Animal and Plant Health Agency (APHA) / Virology Department)
EPI_ISL_5588100	A/chicken/Netherlands/21037287-006010/2021	A / H5N1	Europe / Netherlands / Provincie Flevoland / Gemeente Zeewolde	Rene Heutink (Wageningen Bioveterinary Research)
EPI_ISL_5588106	A/chicken/Netherlands/21037233-001/2021	A / H5N1	Europe / Netherlands / Provincie Noord-Holland	Rene Heutink (Wageningen Bioveterinary Research)
EPI_ISL_5804698	A/chicken/Netherlands/21037708-006010/2021	A / H5N1	Europe / Netherlands / Provincie Noord-Holland	Rene Heutink (Wageningen Bioveterinary Research)
EPI_ISL_5804708	A/mute_swan/England/053054/2021	A / H5N1	Europe / United Kingdom / Worcestershire	Alex Byrne (Animal and Plant Health Agency (APHA) / Virology Department)
EPI_ISL_5804788	A/Mute swan/Netherlands/21037283-002/2021	A / H5N1	Europe / Netherlands / Provincie Overijssel	Rene Heutink (Wageningen Bioveterinary Research)
EPI_ISL_5804789	A/great_skua/Scotland/B07779/2021	A / H5N1	Europe / United Kingdom / Western Isles	Alex Byrne (Animal and Plant Health Agency (APHA) / Virology Department)
EPI_ISL_5862398	A/green-winged-teal/Georgia/DT-22810/2020	A / H5N8	Asia / Georgia	Nicola Lewis (Royal Veterinary College)
EPI_ISL_5863089	A/mallard/Georgia/DT-22620/2020	A / H5N8	Asia / Georgia	Nicola Lewis (Royal Veterinary College)
EPI_ISL_5863358	A/mallard/Georgia/DT-22360/2020	A / H5N8	Asia / Georgia	Nicola Lewis (Royal Veterinary College)
EPI_ISL_5863359	A/mallard/Georgia/DT-23072/2020	A / H5N8	Asia / Georgia	Nicola Lewis (Royal Veterinary College)
EPI_ISL_5863447	A/green-winged-teal/Georgia/DT-22332/2020	A / H5N8	Asia / Georgia	Nicola Lewis (Royal Veterinary College)
EPI_ISL_5863490	A/green-winged-teal/Georgia/DT-22792/2020	A / H5N8	Asia / Georgia	Nicola Lewis (Royal Veterinary College)
EPI_ISL_5863491	A/mallard/Georgia/DT-22456/2020	A / H5N8	Asia / Georgia	Nicola Lewis (Royal Veterinary College)
EPI_ISL_5863497	A/green-winged-teal/Georgia/DT-23070/2020	A / H5N8	Asia / Georgia	Nicola Lewis (Royal Veterinary College)
EPI_ISL_5863557	A/green-winged-teal/Georgia/DT-22978/2020	A / H5N8	Asia / Georgia	Nicola Lewis (Royal Veterinary College)
EPI_ISL_5863567	A/green-winged-teal/Georgia/DT-22392/2020	A / H5N8	Asia / Georgia	Nicola Lewis (Royal Veterinary College)
EPI_ISL_5863615	A/mallard/Georgia/DT-22862/2020	A / H5N8	Asia / Georgia	Nicola Lewis (Royal Veterinary College)
EPI_ISL_5863641	A/mallard/Georgia/DT-22495/2020	A / H5N8	Asia / Georgia	Nicola Lewis (Royal Veterinary College)
EPI_ISL_5863650	A/mallard/Georgia/DT-20222/2020	A / H5N8	Asia / Georgia	Nicola Lewis (Royal Veterinary College)
EPI_ISL_5863749	A/green-winged-teal/Georgia/DT-22894/2020	A / H5N3	Asia / Georgia	Nicola Lewis (Royal Veterinary College)
EPI_ISL_5863929	A/garganey/Georgia/DT-22572/2020	A / H5N8	Asia / Georgia	Nicola Lewis (Royal Veterinary College)
EPI_ISL_5865417	A/domestic duck/Georgia/DT-22368/2020	A / H5N8	Asia / Georgia	Nicola Lewis (Royal Veterinary College)
EPI_ISL_5865550	A/mallard/Georgia/DT-22362/2020	A / H5N8	Asia / Georgia	Nicola Lewis (Royal Veterinary College)
EPI_ISL_5865629	A/garganey/Georgia/DT-22770/2020	A / H5N8	Asia / Georgia	Nicola Lewis (Royal Veterinary College)
EPI_ISL_5865682	A/mallard/Georgia/DT-22356/2020	A / H5N8	Asia / Georgia	Nicola Lewis (Royal Veterinary College)
EPI_ISL_5865695	A/green-winged-teal/Georgia/DT-22246/2020	A / H5N8	Asia / Georgia	Nicola Lewis (Royal Veterinary College)
EPI_ISL_591075	A/mute swan/Netherlands/20015931-001/2020	A / H5N8	Europe / Netherlands / Provincie Utrecht / Kockengen	Rene Heutink (Wageningen Bioveterinary Research)
EPI_ISL_5941469	A/duck/Netherlands/21037958-001005/2021	A / H5N1	Europe / Netherlands / Provincie Flevoland	Rene Heutink (Wageningen Bioveterinary Research)
EPI_ISL_5942524	A/chicken/Netherlands/21037750-001005/2021	A / H5N1	Europe / Netherlands / Provincie Noord-Holland	Rene Heutink (Wageningen Bioveterinary Research)
EPI_ISL_5945412	A/European Herring Gull/Netherlands/21037412-002/2021	A / H5N1	Europe / Netherlands / Provincie Friesland	Rene Heutink (Wageningen Bioveterinary Research)
EPI_ISL_6025676	A/Barnacle goose/Netherlands/21037293-001/2021	A / H5N1	Europe / Netherlands / Provincie Friesland	Rene Heutink (Wageningen Bioveterinary Research)
EPI_ISL_6029360	A/great_skua/Scotland/042505/2021	A / H5N1	Europe / United Kingdom / Western Isles	Alex Byrne (Animal and Plant Health Agency (APHA) / Virology Department)
EPI_ISL_603132	A/chicken/Netherlands/20016597-026030/2020	A / H5N8	Europe / Netherlands / Provincie Gelderland / West Maas en Waal / Altforst	Rene Heutink (Wageningen Bioveterinary Research)
EPI_ISL_603133	A/Eurasian Wigeon/Netherlands/1/2020	A / H5N1	Europe / Netherlands	Theo Bestebroer (Erasmus Medical Center / Department of Virology)
EPI_ISL_603134	A/Eurasian Wigeon/Netherlands/4/2020	A / H5N1	Europe / Netherlands	Theo Bestebroer (Erasmus Medical Center / Department of Virology)
EPI_ISL_603135	A/Eurasian Wigeon/Netherlands/5/2020	A / H5N1	Europe / Netherlands	Theo Bestebroer (Erasmus Medical Center / Department of Virology)
EPI_ISL_603136	A/Eurasian Wigeon/Netherlands/7/2020	A / H5N8	Europe / Netherlands	Theo Bestebroer (Erasmus Medical Center / Department of Virology)
EPI_ISL_603226	A/chicken/Bulgaria/136_19VIR3315-1/2019	A / H5N8	Europe / Bulgaria / Oblast Plovdiv / Krumovo	Bianca Zecchin (Istituto Zooprofilattico Sperimentale Delle Venezie)
EPI_ISL_603227	A/chicken/Bulgaria/143_19VIR3315-2/2019	A / H5N8	Europe / Bulgaria / Oblast Plovdiv / Asenograd	Bianca Zecchin (Istituto Zooprofilattico Sperimentale Delle Venezie)
EPI_ISL_603228	A/chicken/Bulgaria/148-01_19VIR3315-3/2019	A / H5N8	Europe / Bulgaria / Oblast Plovdiv / Asenograd	Bianca Zecchin (Istituto Zooprofilattico Sperimentale Delle Venezie)
EPI_ISL_603229	A/chicken/Bulgaria/150-01_19VIR3315-5/2019	A / H5N8	Europe / Bulgaria / Oblast Plovdiv / Asenograd	Bianca Zecchin (Istituto Zooprofilattico Sperimentale Delle Venezie)
EPI_ISL_603230	A/duck/Bulgaria/78-4t_20VIR1416-3/2020	A / H5N8	Europe / Bulgaria / Oblast Plovdiv / Padarsko	Bianca Zecchin (Istituto Zooprofilattico Sperimentale Delle Venezie)
EPI_ISL_603231	A/chicken/Bulgaria/201_20VIR1723-1/2020	A / H5N2	Europe / Bulgaria / Oblast Plovdiv / Trilistnik	Bianca Zecchin (Istituto Zooprofilattico Sperimentale Delle Venezie)
EPI_ISL_603232	A/chicken/Bulgaria/221_20VIR1725-1/2020	A / H5N2	Europe / Bulgaria / Oblast Plovdiv / Manole	Bianca Zecchin (Istituto Zooprofilattico Sperimentale Delle Venezie)
EPI_ISL_603233	A/chicken/Bulgaria/380-1_20VIR3542-1/2020	A / H5N8	Europe / Bulgaria / Oblast Plovdiv / Asenograd	Bianca Zecchin (Istituto Zooprofilattico Sperimentale Delle Venezie)
EPI_ISL_603234	A/mule_duck/Bulgaria/50-506_20VIR1414-1/2020	A / H5N2	Europe / Bulgaria / Oblast Plovdiv / Obshtina Rakovski	Bianca Zecchin (Istituto Zooprofilattico Sperimentale Delle Venezie)
EPI_ISL_603235	A/mule_duck/Bulgaria/147_20VIR1721-1/2020	A / H5N8	Europe / Bulgaria / Oblast Plovdiv / Bolyarino	Bianca Zecchin (Istituto Zooprofilattico Sperimentale Delle Venezie)
EPI_ISL_603237	A/duck/Bulgaria/130-1_19VIR3314-1/2019	A / H5N8	Europe / Bulgaria / Oblast Lovech / Yoglav	Bianca Zecchin (Istituto Zooprofilattico Sperimentale Delle Venezie)
EPI_ISL_6101847	A/duck/Netherlands/21038151-001005/2021	A / H5N1	Europe / Netherlands / Provincie Flevoland	Rene Heutink (Wageningen Bioveterinary Research)
EPI_ISL_6101848	A/chicken/Netherlands/21038165-006010/2021	A / H5N1	Europe / Netherlands / Provincie Groningen	Rene Heutink (Wageningen Bioveterinary Research)
EPI_ISL_6101868	A/black-headed gull/Netherlands/21037589-002/2021	A / H5N1	Europe / Netherlands / Provincie Friesland	Rene Heutink (Wageningen Bioveterinary Research)
EPI_ISL_6101869	A/goose/Netherlands/21037720-001/2021	A / H5N1	Europe / Netherlands / Provincie Noord-Holland	Rene Heutink (Wageningen Bioveterinary Research)
EPI_ISL_614399	A/buzzard/Germany-MV/AI02166/2020	A / H5N5	Europe / Germany / Mecklenburg-Vorpommern	Jacqueline King (Friedrich-Loeffler-Institut)
EPI_ISL_614400	A/barnacle goose/Germany-SH/AI02167/2020	A / H5N8	Europe / Germany / Schleswig-Holstein	Jacqueline King (Friedrich-Loeffler-Institut)
EPI_ISL_614401	A/mute_swan/Kazakhstan/1-267-20-B/2020	A / H5N8	Asia / Kazakhstan / Akmola	Alex Byrne (Animal and Plant Health Agency (APHA) / Virology Department)
EPI_ISL_615065	A/domestic_goose/Kazakhstan/1-261_2-20-B/2020	A / H5N8	Asia / Kazakhstan / Kostanay	Alex Byrne (Animal and Plant Health Agency (APHA) / Virology Department)
EPI_ISL_615068	A/domestic_goose/Kazakhstan/1-248_2-20-B/2020	A / H5N8	Asia / Kazakhstan / Akmola	Alex Byrne (Animal and Plant Health Agency (APHA) / Virology Department)
EPI_ISL_615072	A/domestic_duck/Kazakhstan/1-274-20-B/2020	A / H5N8	Asia / Kazakhstan / Kostanay	Alex Byrne (Animal and Plant Health Agency (APHA) / Virology Department)
EPI_ISL_615073	A/domestic_goose/Kazakhstan/1-242_2-20-B/2020	A / H5N8	Asia / Kazakhstan / North Kazakhstan	Alex Byrne (Animal and Plant Health Agency (APHA) / Virology Department)
EPI_ISL_623074	A/chicken/Iraq/1/2020	A / H5N8	Asia / Iraq / Muhafazat Ninawa	Alex Byrne (Animal and Plant Health Agency (APHA) / Virology Department)
EPI_ISL_623075	A/chicken/Netherlands/20017138-016020/2020	A / H5N8	Europe / Netherlands / Provincie Gelderland / Gemeente Druten	Rene Heutink (Wageningen Bioveterinary Research)
EPI_ISL_625671	A/whooper swan/Inner Mongolia/w1-1/2020	A / H5N8	Asia / China / Inner Mongolia Autonomous Region	Hongliang Chai (Northeast Forestry University / College of Wildlife Resources)
EPI_ISL_625672	A/mute swan/Inner Mongolia/w2-1/2020	A / H5N8	Asia / China / Inner Mongolia Autonomous Region	Hongliang Chai (Northeast Forestry University / College of Wildlife Resources)
EPI_ISL_626647	A/chicken/Russian_Federation/Omsk/1680-10/2020	A / H5N5	Europe / Russian Federation / Omsk Oblast	Alex Byrne (Animal and Plant Health Agency (APHA) / Virology Department)
EPI_ISL_626648	A/goose/Russian_Federation/Omsk/1680-6/2020	A / H5N5	Europe / Russian Federation / Omsk Oblast	Alex Byrne (Animal and Plant Health Agency (APHA) / Virology Department)

EPI_ISL_626649	A/duck/Russian_Federation/Saratov/1578-2/2020	A / H5N8	Europe / Russian Federation / Saratov Oblast	Alex Byrne (Animal and Plant Health Agency (APHA) / Virology Department)
EPI_ISL_626650	A/duck/Russian_Federation/Omsk/1328-2/2020	A / H5N8	Europe / Russian Federation / Omsk Oblast	Alex Byrne (Animal and Plant Health Agency (APHA) / Virology Department)
EPI_ISL_626651	A/goose/Russian_Federation/Kurgan/1345-25/2020	A / H5N8	Europe / Russian Federation / Kurgan Oblast	Alex Byrne (Animal and Plant Health Agency (APHA) / Virology Department)
EPI_ISL_626652	A/chicken/England/030720/2020	A / H5N8	Europe / United Kingdom / Cheshire	Alex Byrne (Animal and Plant Health Agency (APHA) / Virology Department)
EPI_ISL_631824	A/Mandarin duck/Korea/H242/2020	A / H5N8	Asia / Korea, Republic of / Chungcheongbuk-do	Yu-Na Lee (Animal and Plant Quarantine Agency (S-2158) / Avian Influenza Research & Diagnostic Division)
EPI_ISL_632314	A/greylag goose/Netherlands/20016582-004/2020	A / H5N1	Europe / Netherlands / Provincie Noord-Holland / Gemeente Purmerend	Rene Heutink (Wageningen Bioveterinary Research)
EPI_ISL_632315	A/eurasian teal/Netherlands/20016896-013/2020	A / H5N1	Europe / Netherlands / Provincie Friesland / Holwerd	Rene Heutink (Wageningen Bioveterinary Research)
EPI_ISL_632316	A/eurasian curlew/Netherlands/20016890-001/2020	A / H5N1	Europe / Netherlands / Provincie Friesland / Hallum	Rene Heutink (Wageningen Bioveterinary Research)
EPI_ISL_632317	A/barnacle goose/Netherlands/20016935-002/2020	A / H5N8	Europe / Netherlands / Provincie Friesland / Holwerd	Rene Heutink (Wageningen Bioveterinary Research)
EPI_ISL_632318	A/greylag goose/Netherlands/20016879-001/2020	A / H5N8	Europe / Netherlands / Provincie Friesland / Earnewald	Rene Heutink (Wageningen Bioveterinary Research)
EPI_ISL_632319	A/chicken/Netherlands/20017460-011015/2020	A / H5N8	Europe / Netherlands / Provincie Groningen / Lutjegast	Rene Heutink (Wageningen Bioveterinary Research)
EPI_ISL_6328001	A/greylag goose/Netherlands/21037497-001/2021	A / H5N1	Europe / Netherlands / Provincie Noord-Holland	Rene Heutink (Wageningen Bioveterinary Research)
EPI_ISL_6328009	A/swan/Netherlands/21037791-001/2021	A / H5N1	Europe / Netherlands / Provincie Friesland	Rene Heutink (Wageningen Bioveterinary Research)
EPI_ISL_6328022	A/chicken/Netherlands/21037907-006010/2021	A / H5N1	Europe / Netherlands / Provincie Noord-Holland	Rene Heutink (Wageningen Bioveterinary Research)
EPI_ISL_6328036	A/chicken/Netherlands/21038675-001005/2021	A / H5N1	Europe / Netherlands / Provincie Friesland	Rene Heutink (Wageningen Bioveterinary Research)
EPI_ISL_6328409	A/mute swan/Czech Republic/21312/2021	A / H5N1	Europe / Czech Republic / Jihocesky Kraj / Okres Tabor / 49°14'23.870''N, 14°42''46.680E	Alexander Nagy (State Veterinary Institute Prague)
EPI_ISL_637098	A/duck/Chelyabinsk/1207-1/2020	A / H5N8	Europe / Russian Federation / Chelyabinsk Oblast	Nikolay Zinyakov (Federal Centre for Animal Health (ARRIAH) / OIE Regional Reference Laboratory)
EPI_ISL_6375134	A/waterfowl/Netherlands/21037914-006/2021	A / H5N1	Europe / Netherlands / Provincie Friesland	Rene Heutink (Wageningen Bioveterinary Research)
EPI_ISL_641377	A/chicken/Netherlands/20016978-001/2020	A / H5N8	Europe / Netherlands / Provincie Noord-Holland / Edam-Volendam	Rene Heutink (Wageningen Bioveterinary Research)
EPI_ISL_641378	A/greater white-fronted goose/Netherlands/20017403-004/20	A / H5N5	Europe / Netherlands / South Holland / Gemeente Rijswijk	Rene Heutink (Wageningen Bioveterinary Research)
EPI_ISL_641394	A/chicken/Netherlands/20017639-001/2020	A / H5N8	Europe / Netherlands / Provincie Friesland / Wons	Rene Heutink (Wageningen Bioveterinary Research)
EPI_ISL_641395	A/chicken/Netherlands/20017694-004/2020	A / H5N8	Europe / Netherlands / Provincie Noord-Holland / Gemeente Purmerend	Rene Heutink (Wageningen Bioveterinary Research)
EPI_ISL_641518	A/duck/Netherlands/20017868-016020/2020	A / H5N8	Europe / Netherlands / Provincie Gelderland / Gemeente Voorst	Rene Heutink (Wageningen Bioveterinary Research)
EPI_ISL_644121	A/turkey/Omsk/0001/2020	A / H5N8	Europe / Russian Federation / Omsk Oblast / Omsk	Natalia Goncharova (State Research Center of Virology and Biotechnology (VECTOR) / Emerging Zoonotic Diseases and Influenza)
EPI_ISL_644122	A/goose/Omsk/0002/2020	A / H5N8	Europe / Russian Federation / Omsk Oblast / Omsk region	Natalia Goncharova (State Research Center of Virology and Biotechnology (VECTOR) / Emerging Zoonotic Diseases and Influenza)
EPI_ISL_644123	A/turkey/Omsk/0003/2020	A / H5N8	Europe / Russian Federation / Omsk Oblast / Omsk	Natalia Goncharova (State Research Center of Virology and Biotechnology (VECTOR) / Emerging Zoonotic Diseases and Influenza)
EPI_ISL_644124	A/duck/Omsk/0004/2020	A / H5N8	Europe / Russian Federation / Omsk Oblast / Omsk	Natalia Goncharova (State Research Center of Virology and Biotechnology (VECTOR) / Emerging Zoonotic Diseases and Influenza)
EPI_ISL_644125	A/goose/Omsk/0111/2020	A / H5N8	Europe / Russian Federation / Omsk Oblast / Omsk	Natalia Goncharova (State Research Center of Virology and Biotechnology (VECTOR) / Emerging Zoonotic Diseases and Influenza)
EPI_ISL_644126	A/chicken/Omsk/0112/2020	A / H5N8	Europe / Russian Federation / Omsk Oblast / Omsk	Natalia Goncharova (State Research Center of Virology and Biotechnology (VECTOR) / Emerging Zoonotic Diseases and Influenza)
EPI_ISL_644127	A/goose/Omsk/0113/2020	A / H5N8	Europe / Russian Federation / Omsk Oblast / Omsk	Natalia Goncharova (State Research Center of Virology and Biotechnology (VECTOR) / Emerging Zoonotic Diseases and Influenza)
EPI_ISL_644128	A/goose/Omsk/0114/2020	A / H5N8	Europe / Russian Federation / Omsk Oblast / Omsk	Natalia Goncharova (State Research Center of Virology and Biotechnology (VECTOR) / Emerging Zoonotic Diseases and Influenza)
EPI_ISL_644129	A/goose/Omsk/0115/2020	A / H5N8	Europe / Russian Federation / Omsk Oblast / Omsk	Natalia Goncharova (State Research Center of Virology and Biotechnology (VECTOR) / Emerging Zoonotic Diseases and Influenza)
EPI_ISL_644130	A/goose/Omsk/01161/2020	A / H5N8	Europe / Russian Federation / Omsk Oblast / Omsk	Natalia Goncharova (State Research Center of Virology and Biotechnology (VECTOR) / Emerging Zoonotic Diseases and Influenza)
EPI_ISL_644132	A/goose/Omsk/01171/2020	A / H5N8	Europe / Russian Federation / Omsk Oblast / Omsk	Natalia Goncharova (State Research Center of Virology and Biotechnology (VECTOR) / Emerging Zoonotic Diseases and Influenza)
EPI_ISL_644134	A/chicken/Omsk/0118/2020	A / H5N8	Europe / Russian Federation / Omsk Oblast / Omsk	Natalia Goncharova (State Research Center of Virology and Biotechnology (VECTOR) / Emerging Zoonotic Diseases and Influenza)
EPI_ISL_644135	A/chicken/Omsk/0119/2020	A / H5N8	Europe / Russian Federation / Omsk Oblast / Omsk	Natalia Goncharova (State Research Center of Virology and Biotechnology (VECTOR) / Emerging Zoonotic Diseases and Influenza)
EPI_ISL_644136	A/goose/Omsk/011101/2020	A / H5N8	Europe / Russian Federation / Omsk Oblast / Omsk	Natalia Goncharova (State Research Center of Virology and Biotechnology (VECTOR) / Emerging Zoonotic Diseases and Influenza)
EPI_ISL_644138	A/wild duck/Omsk/01111/2020	A / H5N8	Europe / Russian Federation / Omsk Oblast / Omsk	Natalia Goncharova (State Research Center of Virology and Biotechnology (VECTOR) / Emerging Zoonotic Diseases and Influenza)
EPI_ISL_644139	A/goose/Omsk/0071/2020	A / H5N8	Europe / Russian Federation / Omsk Oblast / Omsk	Natalia Goncharova (State Research Center of Virology and Biotechnology (VECTOR) / Emerging Zoonotic Diseases and Influenza)
EPI_ISL_644140	A/chicken/Omsk/0073/2020	A / H5N8	Europe / Russian Federation / Omsk Oblast / Omsk	Natalia Goncharova (State Research Center of Virology and Biotechnology (VECTOR) / Emerging Zoonotic Diseases and Influenza)
EPI_ISL_644141	A/goose/Omsk/0074/2020	A / H5N8	Europe / Russian Federation / Omsk Oblast / Omsk	Natalia Goncharova (State Research Center of Virology and Biotechnology (VECTOR) / Emerging Zoonotic Diseases and Influenza)
EPI_ISL_644142	A/duck/Omsk/0075/2020	A / H5N8	Europe / Russian Federation / Omsk Oblast / Omsk	Natalia Goncharova (State Research Center of Virology and Biotechnology (VECTOR) / Emerging Zoonotic Diseases and Influenza)
EPI_ISL_644143	A/duck/Omsk/0076/2020	A / H5N8	Europe / Russian Federation / Omsk Oblast / Omsk	Natalia Goncharova (State Research Center of Virology and Biotechnology (VECTOR) / Emerging Zoonotic Diseases and Influenza)
EPI_ISL_644144	A/duck/Omsk/0077/2020	A / H5N8	Europe / Russian Federation / Omsk Oblast / Omsk	Natalia Goncharova (State Research Center of Virology and Biotechnology (VECTOR) / Emerging Zoonotic Diseases and Influenza)
EPI_ISL_644145	A/chicken/Chelyabinsk/401/2020	A / H5N8	Europe / Russian Federation / Chelyabinsk Oblast / Chelyabinsk	Natalia Goncharova (State Research Center of Virology and Biotechnology (VECTOR) / Emerging Zoonotic Diseases and Influenza)
EPI_ISL_644146	A/chicken/Chelyabinsk/402/2020	A / H5N8	Europe / Russian Federation / Chelyabinsk Oblast / Chelyabinsk	Natalia Goncharova (State Research Center of Virology and Biotechnology (VECTOR) / Emerging Zoonotic Diseases and Influenza)
EPI_ISL_644147	A/chicken/Chelyabinsk/403/2020	A / H5N8	Europe / Russian Federation / Chelyabinsk Oblast / Chelyabinsk	Natalia Goncharova (State Research Center of Virology and Biotechnology (VECTOR) / Emerging Zoonotic Diseases and Influenza)
EPI_ISL_644148	A/chicken/Chelyabinsk/404/2020	A / H5N8	Europe / Russian Federation / Chelyabinsk Oblast / Chelyabinsk	Natalia Goncharova (State Research Center of Virology and Biotechnology (VECTOR) / Emerging Zoonotic Diseases and Influenza)
EPI_ISL_644735	A/Cygnus_olor/Belgium/11956_001/2020 (H5N8)	A / H5N8	Europe / Belgium / Provincie West-Vlaanderen / Oudenburg	Steven Van Borm (Sciensano, Department of Animal Infectious Diseases / Animal Infectious Diseases)
EPI_ISL_644737	A/peregrine falcon/Denmark/13776-1/2020-10-30(H5N5)	A / H5N5	Europe / Denmark / Region Sjaland / Lolland	Charlotte Kristiane Hjulsager (Statens Serum Institute / Microbiological Diagnostic and Virology)
EPI_ISL_644824	A/barnacle goose/Denmark/14138-1/2020-11-04(H5N8)	A / H5N8	Europe / Denmark / Region Syddanmark / Tonder Kommune	Charlotte Kristiane Hjulsager (Statens Serum Institute / Microbiological Diagnostic and Virology)
EPI_ISL_647969	A/Turkey/Sweden/SVA201114SZ0001/20KN303106/2020	A / H5N8	Europe / Sweden / Skane Lan / Tomelilla Kommun	Siamak Zohari (National Veterinary Institute)
EPI_ISL_6507374	A/mute swan/Croatia/100/2021	A / H5N1	Europe / Croatia / Sisacko-Moslavacka Zupanija / Muzilovcica (45° 23' 25.9'' N; 16° 40' 49.3'' E)	Vladimir Savi? (Croatian Veterinary Institute / Poultry Centre)
EPI_ISL_653918	A/greylag goose/Netherlands/20016414-001/2020	A / H5N8	Europe / Netherlands / Provincie Utrecht / Gemeente Woerden	Rene Heutink (Wageningen Bioveterinary Research)
EPI_ISL_653919	A/greylag goose/Netherlands/20016494-001/2020	A / H5N8	Europe / Netherlands / Provincie Noord-Holland / Gemeente Naarden	Rene Heutink (Wageningen Bioveterinary Research)
EPI_ISL_653920	A/chicken/Netherlands/20018496-006010/2020	A / H5N8	Europe / Netherlands / Provincie Friesland / Witmarsum	Rene Heutink (Wageningen Bioveterinary Research)
EPI_ISL_653921	A/chicken/Netherlands/20018523-001005/2020	A / H5N8	Europe / Netherlands / Provincie Utrecht / Gemeente Oudewater	Rene Heutink (Wageningen Bioveterinary Research)
EPI_ISL_654821	A/chicken/Saratov/29801/2020	A / H5N8	Europe / Russian Federation / Saratov Oblast / Saratov	Natalia Goncharova (State Research Center of Virology and Biotechnology (VECTOR) / Emerging Zoonotic Diseases and Influenza)
EPI_ISL_654824	A/duck/Saratov/29804/2020	A / H5N8	Europe / Russian Federation / Saratov Oblast / Saratov	Natalia Goncharova (State Research Center of Virology and Biotechnology (VECTOR) / Emerging Zoonotic Diseases and Influenza)
EPI_ISL_654826	A/goose/Omsk/30001/2020	A / H5N8	Europe / Russian Federation / Omsk Oblast / Omsk	Natalia Goncharova (State Research Center of Virology and Biotechnology (VECTOR) / Emerging Zoonotic Diseases and Influenza)
EPI_ISL_654827	A/goose/Omsk/30003/2020	A / H5N8	Europe / Russian Federation / Omsk Oblast / Omsk	Natalia Goncharova (State Research Center of Virology and Biotechnology (VECTOR) / Emerging Zoonotic Diseases and Influenza)
EPI_ISL_654828	A/goose/Omsk/30004/2020	A / H5N8	Europe / Russian Federation / Omsk Oblast / Omsk	Natalia Goncharova (State Research Center of Virology and Biotechnology (VECTOR) / Emerging Zoonotic Diseases and Influenza)
EPI_ISL_654829	A/goose/Omsk/30006/2020	A / H5N8	Europe / Russian Federation / Omsk Oblast / Omsk	Natalia Goncharova (State Research Center of Virology and Biotechnology (VECTOR) / Emerging Zoonotic Diseases and Influenza)
EPI_ISL_654830	A/chicken/Omsk/30007/2020	A / H5N8	Europe / Russian Federation / Omsk Oblast / Omsk	Natalia Goncharova (State Research Center of Virology and Biotechnology (VECTOR) / Emerging Zoonotic Diseases and Influenza)
EPI_ISL_654831	A/goose/Omsk/30009/2020	A / H5N8	Europe / Russian Federation / Omsk Oblast / Omsk	Natalia Goncharova (State Research Center of Virology and Biotechnology (VECTOR) / Emerging Zoonotic Diseases and Influenza)
EPI_ISL_654832	A/goose/Omsk/30010/2020	A / H5N8	Europe / Russian Federation / Omsk Oblast / Omsk	Natalia Goncharova (State Research Center of Virology and Biotechnology (VECTOR) / Emerging Zoonotic Diseases and Influenza)
EPI_ISL_654833	A/chicken/Chelyabinsk/201/2020	A / H5N8	Europe / Russian Federation / Chelyabinsk region	Natalia Goncharova (State Research Center of Virology and Biotechnology (VECTOR) / Emerging Zoonotic Diseases and Influenza)
EPI_ISL_654834	A/chicken/Kurgan/1001/2020	A / H5N8	Europe / Russian Federation / Kurgan region	Natalia Goncharova (State Research Center of Virology and Biotechnology (VECTOR) / Emerging Zoonotic Diseases and Influenza)
EPI_ISL_654835	A/chicken/Kurgan/1003/2020	A / H5N8	Europe / Russian Federation / Kurgan region	Natalia Goncharova (State Research Center of Virology and Biotechnology (VECTOR) / Emerging Zoonotic Diseases and Influenza)
EPI_ISL_654836	A/chicken/Kurgan/1004/2020	A / H5N8	Europe / Russian Federation / Kurgan Oblast	Natalia Goncharova (State Research Center of Virology and Biotechnology (VECTOR) / Emerging Zoonotic Diseases and Influenza)
EPI_ISL_654837	A/chicken/Kurgan/1005/2020	A / H5N8	Europe / Russian Federation / Kurgan region	Natalia Goncharova (State Research Center of Virology and Biotechnology (VECTOR) / Emerging Zoonotic Diseases and Influenza)
EPI_ISL_654838	A/chicken/Kurgan/1010/2020	A / H5N8	Europe / Russian Federation / Kurgan region	Natalia Goncharova (State Research Center of Virology and Biotechnology (VECTOR) / Emerging Zoonotic Diseases and Influenza)

EPI_ISL_654839	A/goose/Kurgan/01/2020	A / H5N8	Europe / Russian Federation / Kurgan region	Natalia Goncharova (State Research Center of Virology and Biotechnology (VECTOR) / Emerging Zoonotic Diseases and Influenza)
EPI_ISL_654840	A/duck/Thanh Hoa/676VTC/2020	A / H5N6	Asia / Vietnam / Tinh Thanh Hoa / Tan Tho	Natalia Goncharova (State Research Center of Virology and Biotechnology (VECTOR) / Emerging Zoonotic Diseases and Influenza)
EPI_ISL_654841	A/duck/Nghe An/694VTC/2020	A / H5N6	Asia / Vietnam / Tinh Nghe An / Quynh Hung	Natalia Goncharova (State Research Center of Virology and Biotechnology (VECTOR) / Emerging Zoonotic Diseases and Influenza)
EPI_ISL_654842	A/duck/Nghe An/695VTC/2020	A / H5N6	Asia / Vietnam / Tinh Nghe An / Quynh Hau	Natalia Goncharova (State Research Center of Virology and Biotechnology (VECTOR) / Emerging Zoonotic Diseases and Influenza)
EPI_ISL_654843	A/duck/Thanh Hoa/722VTC/2020	A / H5N6	Asia / Vietnam / Tinh Thanh Hoa / Quang Hoa	Natalia Goncharova (State Research Center of Virology and Biotechnology (VECTOR) / Emerging Zoonotic Diseases and Influenza)
EPI_ISL_654844	A/duck/Thanh Hoa/740VTC/2020	A / H5N6	Asia / Vietnam / Tinh Thanh Hoa / Vinh	Natalia Goncharova (State Research Center of Virology and Biotechnology (VECTOR) / Emerging Zoonotic Diseases and Influenza)
EPI_ISL_654845	A/chicken/Thanh Hoa/VTC741/2020	A / H5N6	Asia / Vietnam / Tinh Thanh Hoa / Tan Binh	Natalia Goncharova (State Research Center of Virology and Biotechnology (VECTOR) / Emerging Zoonotic Diseases and Influenza)
EPI_ISL_654846	A/duck/Thanh Hoa/752VTC/2020	A / H5N6	Asia / Vietnam / Tinh Thanh Hoa / Thanh Hoa	Natalia Goncharova (State Research Center of Virology and Biotechnology (VECTOR) / Emerging Zoonotic Diseases and Influenza)
EPI_ISL_654847	A/duck/Thanh Hoa/808VTC/2020	A / H5N6	Asia / Vietnam / Tinh Thanh Hoa / Dong Cao	Natalia Goncharova (State Research Center of Virology and Biotechnology (VECTOR) / Emerging Zoonotic Diseases and Influenza)
EPI_ISL_654848	A/chicken/Nghe An/842VTC/2020	A / H5N6	Asia / Vietnam / Tinh Nghe An / Xom Thuan	Natalia Goncharova (State Research Center of Virology and Biotechnology (VECTOR) / Emerging Zoonotic Diseases and Influenza)
EPI_ISL_654849	A/chicken/Thanh Hoa/844VTC/2020	A / H5N6	Asia / Vietnam / Tinh Thanh Hoa / Van Son	Natalia Goncharova (State Research Center of Virology and Biotechnology (VECTOR) / Emerging Zoonotic Diseases and Influenza)
EPI_ISL_654850	A/muscovy duck/Thanh Hoa/879VTC/2020	A / H5N6	Asia / Vietnam / Tinh Thanh Hoa / Quang Hop	Natalia Goncharova (State Research Center of Virology and Biotechnology (VECTOR) / Emerging Zoonotic Diseases and Influenza)
EPI_ISL_654851	A/muscovy duck/Thanh Hoa/892VTC/2020	A / H5N6	Asia / Vietnam / Tinh Thanh Hoa / Hung Loc	Natalia Goncharova (State Research Center of Virology and Biotechnology (VECTOR) / Emerging Zoonotic Diseases and Influenza)
EPI_ISL_654852	A/duck/Thanh Hoa/893VTC/2020	A / H5N6	Asia / Vietnam / Tinh Thanh Hoa / Thieu Hoa	Natalia Goncharova (State Research Center of Virology and Biotechnology (VECTOR) / Emerging Zoonotic Diseases and Influenza)
EPI_ISL_654853	A/duck/Thanh Hoa/923VTC/2020	A / H5N6	Asia / Vietnam / Tinh Thanh Hoa / Dong Son	Natalia Goncharova (State Research Center of Virology and Biotechnology (VECTOR) / Emerging Zoonotic Diseases and Influenza)
EPI_ISL_654854	A/duck/Thanh Hoa/945VTC/2020	A / H5N6	Asia / Vietnam / Tinh Thanh Hoa / Quang Trung	Natalia Goncharova (State Research Center of Virology and Biotechnology (VECTOR) / Emerging Zoonotic Diseases and Influenza)
EPI_ISL_654855	A/chicken/Thanh Hoa/968VTC/2020	A / H5N6	Asia / Vietnam / Tinh Thanh Hoa / Phu Son	Natalia Goncharova (State Research Center of Virology and Biotechnology (VECTOR) / Emerging Zoonotic Diseases and Influenza)
EPI_ISL_654856	A/chicken/Thanh Hoa/980VTC/2020	A / H5N6	Asia / Vietnam / Tinh Thanh Hoa / Mai Lam	Natalia Goncharova (State Research Center of Virology and Biotechnology (VECTOR) / Emerging Zoonotic Diseases and Influenza)
EPI_ISL_654857	A/duck/Thanh Hoa/1107VTC/2020	A / H5N6	Asia / Vietnam / Tinh Thanh Hoa / Phu Son	Natalia Goncharova (State Research Center of Virology and Biotechnology (VECTOR) / Emerging Zoonotic Diseases and Influenza)
EPI_ISL_654858	A/duck/Thanh Hoa/1151VTC/2020	A / H5N6	Asia / Vietnam / Tinh Thanh Hoa / Quang Trung	Natalia Goncharova (State Research Center of Virology and Biotechnology (VECTOR) / Emerging Zoonotic Diseases and Influenza)
EPI_ISL_654859	A/chicken/Thanh Hoa/1152VTC/2020	A / H5N6	Asia / Vietnam / Tinh Thanh Hoa / Phu Son	Natalia Goncharova (State Research Center of Virology and Biotechnology (VECTOR) / Emerging Zoonotic Diseases and Influenza)
EPI_ISL_654860	A/duck/Thanh Hoa/1180VTC/2020	A / H5N6	Asia / Vietnam / Tinh Thanh Hoa / Phu Son	Natalia Goncharova (State Research Center of Virology and Biotechnology (VECTOR) / Emerging Zoonotic Diseases and Influenza)
EPI_ISL_654861	A/duck/Thanh Hoa/1182VTC/2020	A / H5N6	Asia / Vietnam / Tinh Thanh Hoa / Quang Trung	Natalia Goncharova (State Research Center of Virology and Biotechnology (VECTOR) / Emerging Zoonotic Diseases and Influenza)
EPI_ISL_654862	A/duck/Nghe An/1693VTC/2020	A / H5N6	Asia / Vietnam / Tinh Nghe An / Quynh Vinh	Natalia Goncharova (State Research Center of Virology and Biotechnology (VECTOR) / Emerging Zoonotic Diseases and Influenza)
EPI_ISL_654863	A/duck/Thanh Hoa/2202VTC/2020	A / H5N6	Asia / Vietnam / Tinh Thanh Hoa / Truong Lam	Natalia Goncharova (State Research Center of Virology and Biotechnology (VECTOR) / Emerging Zoonotic Diseases and Influenza)
EPI_ISL_654864	A/chicken/Nghe An/14475VTC/2020	A / H5N6	Asia / Vietnam / Tinh Nghe An / Quynh Chau	Natalia Goncharova (State Research Center of Virology and Biotechnology (VECTOR) / Emerging Zoonotic Diseases and Influenza)
EPI_ISL_654865	A/duck/Nghe An/14528VTC/2020	A / H5N6	Asia / Vietnam / Tinh Nghe An / Ngoc Son	Natalia Goncharova (State Research Center of Virology and Biotechnology (VECTOR) / Emerging Zoonotic Diseases and Influenza)
EPI_ISL_654958	A/mallard/Italy/20VIR7139-73/2020	A / H5N8	Europe / Italy / Veneto / Province of Rovigo / Porto Tolle, Ca' Zuliani	Bianca Zecchin (Istituto Zooprofilattico Sperimentale Delle Venezie)
EPI_ISL_6590766	A/swan/France/21P012384/2021	A / H5N1	Europe / France / Region Lorraine / Departement de la Meuse	Francois-Xavier Briand (ANSES Agence Nationale De Securite Sanitaire De L'alimentation / Laboratoire de Ploufragan-Plouzané)
EPI_ISL_6596216	A/barnacle goose/Sweden/SVA211102SZ0402/FB004395/M-2(	A / H5N1	Europe / Sweden / Skane Lan / Malmo Kommun	Siamak Zohari (National Veterinary Institute)
EPI_ISL_6599075	A/greylag goose /Sweden/SVA211103SZ0398/FB004410/M-20	A / H5N1	Europe / Sweden / Skane Lan / Svedala Kommun	Siamak Zohari (National Veterinary Institute)
EPI_ISL_6599895	A/common pheasant /Sweden/SVA211104SZ0320/FB004417/I	A / H5N1	Europe / Sweden / Skane Lan / Svedala Kommun	Siamak Zohari (National Veterinary Institute)
EPI_ISL_6600745	A/common pheasant /Sweden/SVA211104SZ0320/FB004418/I	A / H5N1	Europe / Sweden / Skane Lan / Svedala Kommun	Siamak Zohari (National Veterinary Institute)
EPI_ISL_6600769	A/common buzzard /Sweden/SVA211104SZ0320/FB004419/M	A / H5N1	Europe / Sweden / Skane Lan / Svedala Kommun	Siamak Zohari (National Veterinary Institute)
EPI_ISL_6600943	A/great black-backed gull/Sweden/SVA211109SZ0434/FB0044	A / H5N1	Europe / Sweden / Skane Lan / Landskrona Kommun	Siamak Zohari (National Veterinary Institute)
EPI_ISL_660264	A/Gallus_gallus/Belgium/12168_002/2020	A / H5N5	Europe / Belgium / Provincie West-Vlaanderen / Menen	Steven Van Borm (Sciensano, Department of Animal Infectious Diseases / Animal Infectious Diseases)
EPI_ISL_661177	A/chicken/Poland/448/2020	A / H5N8	Europe / Poland / Greater Poland Voivodeship	Edyta ?wi?to? (National Veterinary Research Institut Poland, PIWet-PIB)
EPI_ISL_661178	A/swan/Tumen/1479-2/2020	A / H5N8	Europe / Russian Federation / Tyumen Oblast	Nikolay Zinyakov (Federal Centre for Animal Health (ARRIAH) / OIE Regional Reference Laboratory)
EPI_ISL_661313	A/Anser_albifrons/Belgium/11956_005/2020	A / H5N8	Europe / Belgium / Provincie West-Vlaanderen / Bredene	Steven Van Borm (Sciensano, Department of Animal Infectious Diseases / Animal Infectious Diseases)
EPI_ISL_664102	A/Numenius_arquata/Belgium/11956_003/2020	A / H5N8	Europe / Belgium / Provincie West-Vlaanderen / Wilskerke	Steven Van Borm (Sciensano, Department of Animal Infectious Diseases / Animal Infectious Diseases)
EPI_ISL_666687	A/Madarin duck/Korea/K20-551-4/2020	A / H5N8	Asia / Korea, Republic of / Gyeonggi-do	Sol Jeong (Konkuk University / 302, School of Veterinary Medicine)
EPI_ISL_666890	A/turkey/Croatia/104/2020	A / H5N8	Europe / Croatia / Koprivnicko-Krizevacka Zupanija / Delovi	Vladimir Savi? (Croatian Veterinary Institute / Poultry Centre)
EPI_ISL_6675733	A/chicken/Netherlands/21039174-001005/2021	A / H5N1	Europe / Netherlands / Provincie Utrecht	Rene Heutink (Wageningen Bioveterinary Research)
EPI_ISL_6675739	A/chicken/Netherlands/21039108-001005/2021	A / H5N1	Europe / Netherlands / Provincie Utrecht	Rene Heutink (Wageningen Bioveterinary Research)
EPI_ISL_6675744	A/mute swan/Netherlands/21039008-003/2021	A / H5N1	Europe / Netherlands / Provincie Noord-Holland	Rene Heutink (Wageningen Bioveterinary Research)
EPI_ISL_6675750	A/mute swan/Netherlands/21038412-002/2021	A / H5N1	Europe / Netherlands / South Holland	Rene Heutink (Wageningen Bioveterinary Research)
EPI_ISL_6675755	A/goose/Netherlands/21038413-002/2021	A / H5N1	Europe / Netherlands / Provincie Friesland	Rene Heutink (Wageningen Bioveterinary Research)
EPI_ISL_667810	A/chicken/France/20P016448/2020	A / H5N8	Europe / France / Corsica / Departement de la Haute-Corse	Francois-Xavier Briand (ANSES Agence Nationale De Securite Sanitaire De L'alimentation / Laboratoire de Ploufragan-Plouzané)
EPI_ISL_668456	A/Peregrine falcon/Sweden/SVA201117SZ0467/20KN003345/2	A / H5N8	Europe / Sweden / Skane Lan / Lunds Kommun	Siamak Zohari (National Veterinary Institute)
EPI_ISL_668457	A/barnacle goose/Sweden/SVA201117SZ0468/KN003355/202(	A / H5N8	Europe / Sweden / Skane Lan / Lunds Kommun	Siamak Zohari (National Veterinary Institute)
EPI_ISL_6757629	A/Duck/Guangdong/21314/2021(H5N6)	A / H5N6	Asia / China / Guangdong Province	Jiahao Zhang (South China Agricultural University / College of Veterinary Medicine)
EPI_ISL_6757630	A/Goose/Sichuan/21406-5/2021(H5N6)	A / H5N6	Asia / China / Sichuan Province	Jiahao Zhang (South China Agricultural University / College of Veterinary Medicine)
EPI_ISL_6757631	A/Duck/Sichuan/21406-8/2021(H5N6)	A / H5N6	Asia / China / Sichuan Province	Jiahao Zhang (South China Agricultural University / College of Veterinary Medicine)
EPI_ISL_6758007	A/Goose/Sichuan/21418-2/2021(H5N6)	A / H5N6	Asia / China / Sichuan Province	Jiahao Zhang (South China Agricultural University / College of Veterinary Medicine)
EPI_ISL_6759148	A/Goose/Sichuan/21765-8/2021(H5N6)	A / H5N6	Asia / China / Sichuan Province	Jiahao Zhang (South China Agricultural University / College of Veterinary Medicine)
EPI_ISL_6760430	A/Duck/Sichuan/21826-1/2021(H5N6)	A / H5N6	Asia / China / Sichuan Province	Jiahao Zhang (South China Agricultural University / College of Veterinary Medicine)
EPI_ISL_6760679	A/Duck/Sichuan/21826-5/2021(H5N6)	A / H5N6	Asia / China / Sichuan Province	Jiahao Zhang (South China Agricultural University / College of Veterinary Medicine)
EPI_ISL_6760681	A/Duck/Sichuan/21840-5/2021(H5N6)	A / H5N6	Asia / China / Sichuan Province	Jiahao Zhang (South China Agricultural University / College of Veterinary Medicine)
EPI_ISL_6760682	A/Chicken/Sichuan/21859-5/2021(H5N6)	A / H5N6	Asia / China / Sichuan Province	Jiahao Zhang (South China Agricultural University / College of Veterinary Medicine)
EPI_ISL_6760683	A/Duck/Sichuan/21898/2021(H5N6)	A / H5N6	Asia / China / Sichuan Province	Jiahao Zhang (South China Agricultural University / College of Veterinary Medicine)
EPI_ISL_6760684	A/Duck/Sichuan/21921-1/2021(H5N6)	A / H5N6	Asia / China / Sichuan Province	Jiahao Zhang (South China Agricultural University / College of Veterinary Medicine)
EPI_ISL_6760685	A/Duck/Sichuan/21957-1/2021(H5N6)	A / H5N6	Asia / China / Sichuan Province	Jiahao Zhang (South China Agricultural University / College of Veterinary Medicine)
EPI_ISL_6760687	A/Goose/Guangdong/211057/2021(H5N6)	A / H5N6	Asia / China / Guangdong Province	Jiahao Zhang (South China Agricultural University / College of Veterinary Medicine)
EPI_ISL_6760999	A/black-headed gull/Netherlands/21037590-002/2021	A / H5N1	Europe / Netherlands / Provincie Friesland	Rene Heutink (Wageningen Bioveterinary Research)
EPI_ISL_6761000	A/greylag goose/Netherlands/21037809-001/2021	A / H5N1	Europe / Netherlands / South Holland	Rene Heutink (Wageningen Bioveterinary Research)
EPI_ISL_6761001	A/mute swan/Netherlands/21038142-001/2021	A / H5N1	Europe / Netherlands / Provincie Gelderland	Rene Heutink (Wageningen Bioveterinary Research)
EPI_ISL_6761002	A/mute swan/Netherlands/21038142-004/2021	A / H5N1	Europe / Netherlands / Provincie Gelderland	Rene Heutink (Wageningen Bioveterinary Research)
EPI_ISL_6761003	A/mute swan/Netherlands/21038142-007/2021	A / H5N1	Europe / Netherlands / Provincie Gelderland	Rene Heutink (Wageningen Bioveterinary Research)
EPI_ISL_6761004	A/greylag goose/Netherlands/21037883-001/2021	A / H5N1	Europe / Netherlands / Provincie Flevoland	Rene Heutink (Wageningen Bioveterinary Research)
EPI_ISL_6761005	A/chicken/Netherlands/21038211-001/2021	A / H5N1	Europe / Netherlands / Provincie Friesland	Rene Heutink (Wageningen Bioveterinary Research)
EPI_ISL_6761006	A/mute swan/Netherlands/21038362-001/2021	A / H5N1	Europe / Netherlands / Provincie Friesland	Rene Heutink (Wageningen Bioveterinary Research)
EPI_ISL_6761007	A/mute swan/Netherlands/21038362-002/2021	A / H5N1	Europe / Netherlands / Provincie Friesland	Rene Heutink (Wageningen Bioveterinary Research)

EPI_ISL_6761008	A/greylag goose/Netherlands/21038036-002/2021	A / H5N1	Europe / Netherlands / Provincie Noord-Holland	Rene Heutink (Wageningen Bioveterinary Research)
EPI_ISL_6761009	A/greylag goose/Netherlands/21038037-001/2021	A / H5N1	Europe / Netherlands / Provincie Noord-Holland	Rene Heutink (Wageningen Bioveterinary Research)
EPI_ISL_6761010	A/mute swan/Netherlands/21038038-001/2021	A / H5N1	Europe / Netherlands / Provincie Groningen	Rene Heutink (Wageningen Bioveterinary Research)
EPI_ISL_6761011	A/peregrine falcon/Netherlands/21038169-002/2021	A / H5N1	Europe / Netherlands / Provincie Noord-Holland	Rene Heutink (Wageningen Bioveterinary Research)
EPI_ISL_6761012	A/barnacle goose/Netherlands/21038248-001/2021	A / H5N1	Europe / Netherlands / South Holland	Rene Heutink (Wageningen Bioveterinary Research)
EPI_ISL_6761013	A/greylag goose/Netherlands/21038252-001/2021	A / H5N1	Europe / Netherlands / Provincie Utrecht	Rene Heutink (Wageningen Bioveterinary Research)
EPI_ISL_6761016	A/greylag goose/Netherlands/21038253-001/2021	A / H5N1	Europe / Netherlands / Provincie Utrecht	Rene Heutink (Wageningen Bioveterinary Research)
EPI_ISL_6761017	A/chicken/Netherlands/21038669-001005/2021	A / H5N1	Europe / Netherlands / Provincie Utrecht	Rene Heutink (Wageningen Bioveterinary Research)
EPI_ISL_6761018	A/mute swan/Netherlands/21038537-004/2021	A / H5N1	Europe / Netherlands / Provincie Noord-Holland	Rene Heutink (Wageningen Bioveterinary Research)
EPI_ISL_6761019	A/mute swan/Netherlands/21038706-002/2021	A / H5N1	Europe / Netherlands / South Holland	Rene Heutink (Wageningen Bioveterinary Research)
EPI_ISL_6761020	A/mute swan/Netherlands/21038479-002/2021	A / H5N1	Europe / Netherlands / Provincie Friesland	Rene Heutink (Wageningen Bioveterinary Research)
EPI_ISL_6761021	A/greylag goose/Netherlands/21038565-002/2021	A / H5N1	Europe / Netherlands / Provincie Utrecht	Rene Heutink (Wageningen Bioveterinary Research)
EPI_ISL_6761022	A/greylag goose/Netherlands/21038567-002/2021	A / H5N1	Europe / Netherlands / Provincie Utrecht	Rene Heutink (Wageningen Bioveterinary Research)
EPI_ISL_6761023	A/mallard/Netherlands/21038796-002/2021	A / H5N1	Europe / Netherlands / South Holland	Rene Heutink (Wageningen Bioveterinary Research)
EPI_ISL_6761024	A/goose/Netherlands/21038799-002/2021	A / H5N1	Europe / Netherlands / North Brabant	Rene Heutink (Wageningen Bioveterinary Research)
EPI_ISL_6761101	A/Branta_leucopsis/Belgium/14735_0001/2021	A / H5N1	Europe / Belgium / Provincie Antwerpen / Ekerne	Steven Van Borm (Sciensano, Department of Animal Infectious Diseases / Animal Infectious Diseases)
EPI_ISL_6772736	A/Chicken/Guangdong/211064-1/2021(H5N6)	A / H5N6	Asia / China / Guangdong Province	Jiahao Zhang (South China Agricultural University / College of Veterinary Medicine)
EPI_ISL_6772737	A/Chicken/Guangdong/211064-2/2021(H5N6)	A / H5N6	Asia / China / Guangdong Province	Jiahao Zhang (South China Agricultural University / College of Veterinary Medicine)
EPI_ISL_6772738	A/Chicken/Guangdong/211064-3/2021(H5N6)	A / H5N6	Asia / China / Guangdong Province	Jiahao Zhang (South China Agricultural University / College of Veterinary Medicine)
EPI_ISL_6772739	A/Chicken/Guangdong/211064-4/2021(H5N6)	A / H5N6	Asia / China / Guangdong Province	Jiahao Zhang (South China Agricultural University / College of Veterinary Medicine)
EPI_ISL_6772759	A/Chicken/Guangdong/211064-5/2021(H5N6)	A / H5N6	Asia / China / Guangdong Province	Jiahao Zhang (South China Agricultural University / College of Veterinary Medicine)
EPI_ISL_6772898	A/Goose/Guangdong/211106-2/2021(H5N6)	A / H5N6	Asia / China / Guangdong Province	Jiahao Zhang (South China Agricultural University / College of Veterinary Medicine)
EPI_ISL_6780544	A/Muscovy duck/Quang Ninh/353/2017	A / H5N6	Asia / Vietnam / Tinh Quang Ninh	
EPI_ISL_6780643	A/Duck/Luxor/51/2018	A / H5N8	Africa / Egypt	
EPI_ISL_6780654	A/Duck/Luxor/62/2018	A / H5N8	Africa / Egypt	
EPI_ISL_6780663	A/chicken/Luxor/103/2018	A / H5N8	Africa / Egypt	
EPI_ISL_6780673	A/Turkey/Luxor/240/2018	A / H5N8	Africa / Egypt	
EPI_ISL_6780682	A/Duck/Luxor/294/2019	A / H5N8	Africa / Egypt	
EPI_ISL_6780696	A/Duck/Luxor/297/2019	A / H5N8	Africa / Egypt	
EPI_ISL_6780727	A/Duck/Luxor/248/2018	A / H5N8	Africa / Egypt	
EPI_ISL_6780760	A/chicken/Luxor/313/2019	A / H5N8	Africa / Egypt	
EPI_ISL_6781195	A/Whooper swan/Mongolia/01/2020	A / H5N6	Asia / Mongolia	
EPI_ISL_6781200	A/Whooper swan/Mongolia/03/2020	A / H5N6	Asia / Mongolia	
EPI_ISL_6781352	A/goose/China/Wuhu01/2019	A / H5N6	Asia / China	
EPI_ISL_6781353	A/goose/China/Yizheng11/2016	A / H5N6	Asia / China	
EPI_ISL_6781354	A/goose/China/BaoYing09/2016	A / H5N6	Asia / China	
EPI_ISL_6781355	A/chicken/China/HJ11/2017	A / H5N6	Asia / China	
EPI_ISL_6781356	A/chicken/China/LA10/2017	A / H5N6	Asia / China	
EPI_ISL_6781405	A/Whooper Swan/Khuvsgul/#1/2020	A / H5N6	Asia / Mongolia	
EPI_ISL_6781406	A/Whooper Swan/Khuvsgul/#4/2020	A / H5N6	Asia / Mongolia	
EPI_ISL_6781491	A/Turkey/Egypt/A2/2021	A / H5N8	Africa / Egypt	
EPI_ISL_6795229	A/duck/China/FJ1807/2018	A / H5N6	Asia / China	
EPI_ISL_6795230	A/duck/China/FJ1808/2018	A / H5N6	Asia / China	
EPI_ISL_6795231	A/duck/China/FJ18105/2018	A / H5N6	Asia / China	
EPI_ISL_6795232	A/duck/China/FJ18106/2018	A / H5N6	Asia / China	
EPI_ISL_6795233	A/duck/China/FJ18107/2018	A / H5N6	Asia / China	
EPI_ISL_6795234	A/duck/China/FJ18108/2018	A / H5N6	Asia / China	
EPI_ISL_6795235	A/duck/China/FJ1813/2018	A / H5N6	Asia / China	
EPI_ISL_6795236	A/duck/China/FJ18203/2018	A / H5N6	Asia / China	
EPI_ISL_6795237	A/duck/China/FJ1543/2015	A / H5N6	Asia / China	
EPI_ISL_6795238	A/duck/China/FJ18210/2018	A / H5N6	Asia / China	
EPI_ISL_6795239	A/duck/China/FJ18212/2018	A / H5N6	Asia / China	
EPI_ISL_6795240	A/duck/China/FJ18220/2018	A / H5N6	Asia / China	
EPI_ISL_6795241	A/duck/China/FJ1519/2015	A / H5N6	Asia / China	
EPI_ISL_6795242	A/duck/China/FJ1545/2015	A / H5N6	Asia / China	
EPI_ISL_6795243	A/duck/China/FJ1563/2015	A / H5N6	Asia / China	
EPI_ISL_6795244	A/duck/China/FJ1567/2016	A / H5N6	Asia / China	
EPI_ISL_6795245	A/duck/China/FJ1602/2016	A / H5N6	Asia / China	
EPI_ISL_6795246	A/duck/China/FJ1683/2016	A / H5N6	Asia / China	
EPI_ISL_6795247	A/duck/China/FJ17139/2017	A / H5N6	Asia / China	
EPI_ISL_6795248	A/duck/China/FJ17152/2017	A / H5N6	Asia / China	
EPI_ISL_6795249	A/duck/China/FJ19179/2017	A / H5N6	Asia / China	
EPI_ISL_6795250	A/duck/China/JX1519/2015	A / H5N6	Asia / China	
EPI_ISL_6795251	A/duck/China/JX1545/2015	A / H5N6	Asia / China	
EPI_ISL_6795252	A/duck/China/ZJ1604/2016	A / H5N6	Asia / China	
EPI_ISL_6795253	A/duck/China/ZJ1504/2015	A / H5N6	Asia / China	
EPI_ISL_6795254	A/duck/China/ZJ1532/2015	A / H5N6	Asia / China	
EPI_ISL_6795255	A/duck/China/ZJ1606/2016	A / H5N6	Asia / China	
EPI_ISL_6795256	A/duck/China/FJ18231/2019	A / H5N6	Asia / China	
EPI_ISL_6795257	A/duck/China/FJ18248/2019	A / H5N6	Asia / China	
EPI_ISL_6795258	A/duck/China/FJ18252/2019	A / H5N6	Asia / China	
EPI_ISL_6795259	A/duck/China/FJ18262/2018	A / H5N6	Asia / China	

EPI_ISL_6795260	A/duck/China/FJ18271/2018	A / H5N6	Asia / China	
EPI_ISL_6795261	A/duck/China/FJ1829/2019	A / H5N6	Asia / China	
EPI_ISL_6795262	A/duck/China/FJ18299/2018	A / H5N6	Asia / China	
EPI_ISL_6795263	A/duck/China/FJ1854/2018	A / H5N6	Asia / China	
EPI_ISL_6795264	A/duck/China/FJ1904/2019	A / H5N6	Asia / China	
EPI_ISL_6795265	A/duck/China/FJ1914/2019	A / H5N6	Asia / China	
EPI_ISL_6795266	A/duck/China/FJ1915/2019	A / H5N6	Asia / China	
EPI_ISL_6795267	A/duck/China/FJ1921/2019	A / H5N6	Asia / China	
EPI_ISL_6795268	A/duck/China/FJ1922/2019	A / H5N6	Asia / China	
EPI_ISL_6795269	A/duck/China/FJ1923/2019	A / H5N6	Asia / China	
EPI_ISL_6795270	A/duck/China/FJ1931/2019	A / H5N6	Asia / China	
EPI_ISL_6795271	A/duck/China/FJ19323/2019	A / H5N6	Asia / China	
EPI_ISL_6795272	A/duck/China/FJ19332/2019	A / H5N6	Asia / China	
EPI_ISL_6795273	A/duck/China/FJ1935/2019	A / H5N6	Asia / China	
EPI_ISL_681271	A/chicken/Kagawa/F2T/2020	A / H5N8	Asia / Japan / Kagawa	Takehiko Saito (National Institute of Animal Health)
EPI_ISL_681272	A/chicken/Kagawa/F3C/2020	A / H5N8	Asia / Japan / Kagawa	Takehiko Saito (National Institute of Animal Health)
EPI_ISL_681273	A/chicken/Kagawa/G12C/2020	A / H5N8	Asia / Japan / Kagawa	Takehiko Saito (National Institute of Animal Health)
EPI_ISL_681274	A/chicken/Kagawa/G4T/2020	A / H5N8	Asia / Japan / Kagawa	Takehiko Saito (National Institute of Animal Health)
EPI_ISL_681275	A/chicken/Kagawa/H2T/2020	A / H5N8	Asia / Japan / Kagawa	Takehiko Saito (National Institute of Animal Health)
EPI_ISL_681276	A/chicken/Kagawa/H8C/2020	A / H5N8	Asia / Japan / Kagawa	Takehiko Saito (National Institute of Animal Health)
EPI_ISL_681281	A/swan/Niigata/151118/2020	A / H5N8	Asia / Japan / Niigata	Takehiko Saito (National Institute of Animal Health)
EPI_ISL_681282	A/chicken/Fukuoka/T1/2020	A / H5N8	Asia / Japan / Fukuoka	Takehiko Saito (National Institute of Animal Health)
EPI_ISL_681283	A/chicken/Fukuoka/T2/2020	A / H5N8	Asia / Japan / Fukuoka	Takehiko Saito (National Institute of Animal Health)
EPI_ISL_681284	A/chicken/Hyogo/1T/2020	A / H5N8	Asia / Japan / Hyogo	Takehiko Saito (National Institute of Animal Health)
EPI_ISL_681285	A/chicken/Hyogo/2T/2020	A / H5N8	Asia / Japan / Hyogo	Takehiko Saito (National Institute of Animal Health)
EPI_ISL_681286	A/chicken/Kagawa/11C/2020	A / H5N8	Asia / Japan / Kagawa	Takehiko Saito (National Institute of Animal Health)
EPI_ISL_681287	A/chicken/Kagawa/7C/2020	A / H5N8	Asia / Japan / Kagawa	Takehiko Saito (National Institute of Animal Health)
EPI_ISL_681288	A/chicken/Kagawa/B10T/2020	A / H5N8	Asia / Japan / Kagawa	Takehiko Saito (National Institute of Animal Health)
EPI_ISL_681289	A/chicken/Kagawa/B13C/2020	A / H5N8	Asia / Japan / Kagawa	Takehiko Saito (National Institute of Animal Health)
EPI_ISL_681290	A/chicken/Kagawa/B8T/2020	A / H5N8	Asia / Japan / Kagawa	Takehiko Saito (National Institute of Animal Health)
EPI_ISL_681291	A/chicken/Kagawa/C3C/2020	A / H5N8	Asia / Japan / Kagawa	Takehiko Saito (National Institute of Animal Health)
EPI_ISL_681292	A/chicken/Kagawa/C3T/2020	A / H5N8	Asia / Japan / Kagawa	Takehiko Saito (National Institute of Animal Health)
EPI_ISL_681293	A/chicken/Kagawa/C5T/2020	A / H5N8	Asia / Japan / Kagawa	Takehiko Saito (National Institute of Animal Health)
EPI_ISL_681294	A/chicken/Kagawa/D2C/2020	A / H5N8	Asia / Japan / Kagawa	Takehiko Saito (National Institute of Animal Health)
EPI_ISL_681295	A/chicken/Kagawa/D5T/2020	A / H5N8	Asia / Japan / Kagawa	Takehiko Saito (National Institute of Animal Health)
EPI_ISL_681296	A/chicken/Kagawa/E13T/2020	A / H5N8	Asia / Japan / Kagawa	Takehiko Saito (National Institute of Animal Health)
EPI_ISL_681297	A/chicken/Kagawa/E7T/2020	A / H5N8	Asia / Japan / Kagawa	Takehiko Saito (National Institute of Animal Health)
EPI_ISL_6816050	A/Duck/Egypt/BEH2/2020	A / H5N8	Africa / Egypt	
EPI_ISL_6816051	A/chicken/Egypt/GIZ4/2020	A / H5N8	Africa / Egypt	
EPI_ISL_6816052	A/chicken/Egypt/MEN6/2020	A / H5N8	Africa / Egypt	
EPI_ISL_6816053	A/chicken/Egypt/MEN10/2020	A / H5N8	Africa / Egypt	
EPI_ISL_6816054	A/chicken/Egypt/CAI11/2020	A / H5N8	Africa / Egypt	
EPI_ISL_6816057	A/environment sample/China/TZ001/2021	A / H5N8	Asia / China	Ahmed Magdy Ahmed Khalil (Faculty of Veterinary Medicine - Zagazig University / Zoonoses)
EPI_ISL_682297	A/environment/Kagoshima/KU-ngr-J2/2020(H5N8)	A / H5N8	Asia / Japan / Kagoshima	Makoto Ozawa (Kagoshima University)
EPI_ISL_6829497	A/hooded crane/Kagoshima/KU-5T/2021 (H5N8)	A / H5N8	Asia / Japan / Kagoshima	Takehiko Saito (National Institute of Animal Health)
EPI_ISL_6829532	A/chicken/Akita/7C/2021	A / H5N8	Asia / Japan / Akita	Takehiko Saito (National Institute of Animal Health)
EPI_ISL_6829533	A/chicken/Kagoshima/21A6T/2021	A / H5N1	Asia / Japan / Kagoshima	Takehiko Saito (National Institute of Animal Health)
EPI_ISL_6829536	A/chicken/Kagoshima/B3T/2021	A / H5N8	Asia / Japan / Kagoshima	
EPI_ISL_683592	A/Eurasian_wigeon/Italy/20VIR7301-206/2020	A / H5N1	Europe / Italy / Veneto / Province of Venice / Jesolo	Bianca Zecchin (Istituto Zooprofilattico Sperimentale Delle Venezie)
EPI_ISL_683593	A/Eurasian_wigeon/Italy/20VIR7139-121/2020	A / H5N8	Europe / Italy / Veneto / Province of Rovigo / Valle Ca' Zuliani	Bianca Zecchin (Istituto Zooprofilattico Sperimentale Delle Venezie)
EPI_ISL_683594	A/mallard/Italy/20VIR7139-124_feather/2020	A / H5N8	Europe / Italy / Veneto / Province of Rovigo / Valle Ca' Zuliani	Bianca Zecchin (Istituto Zooprofilattico Sperimentale Delle Venezie)
EPI_ISL_683751	A/Eurasian_wigeon/Italy/20VIR7301-31/2020	A / H5N8	Europe / Italy / Veneto / Province of Venice / Jesolo	Bianca Zecchin (Istituto Zooprofilattico Sperimentale Delle Venezie)
EPI_ISL_683752	A/Eurasian_wigeon/Italy/20VIR7301-34/2020	A / H5N8	Europe / Italy / Veneto / Province of Venice / Jesolo	Bianca Zecchin (Istituto Zooprofilattico Sperimentale Delle Venezie)
EPI_ISL_683997	A/mute swan/Wales/048069/2020	A / H5N5	Europe / United Kingdom	Alex Byrne (Animal and Plant Health Agency (APHA) / Virology Department)
EPI_ISL_683999	A/mute swan/Wales/048068/2020	A / H5N5	Europe / United Kingdom	Alex Byrne (Animal and Plant Health Agency (APHA) / Virology Department)
EPI_ISL_684037	A/goose/Tatarstan/1730-2/2020	A / H5N8	Europe / Russian Federation / Republic of Tatarstan	Nikolay Zinyakov (Federal Centre for Animal Health (ARRIAH) / OIE Regional Reference Laboratory)
EPI_ISL_6914131	A/chicken/chongqing/H1/2021	A / H5N6	Asia / China / Chongqinq Municipality	Wenming Jiang (China Animal Health and Epidemiology Center (CAHEC))
EPI_ISL_6929958	A/turkey/Poland/H1910-T3/2021	A / H5N1	Europe / Poland / Masovian Voivodeship	Edyta ?wi?to? (National Veterinary Research Institut Poland, PIWet-PIB)
EPI_ISL_6929970	A/turkey/Poland/H1911-N/2021	A / H5N1	Europe / Poland / Masovian Voivodeship	Edyta ?wi?to? (National Veterinary Research Institut Poland, PIWet-PIB)
EPI_ISL_6930238	A/turkey/Poland/H1913-T1/2021	A / H5N1	Europe / Poland / Masovian Voivodeship	Edyta ?wi?to? (National Veterinary Research Institut Poland, PIWet-PIB)
EPI_ISL_6930564	A/turkey/Poland/H1924-T1/2021	A / H5N1	Europe / Poland / Masovian Voivodeship	Edyta ?wi?to? (National Veterinary Research Institut Poland, PIWet-PIB)
EPI_ISL_6931008	A/domestic_goose/Poland/H1931-T1/2021	A / H5N1	Europe / Poland / Lubusz Voivodeship	Edyta ?wi?to? (National Veterinary Research Institut Poland, PIWet-PIB)
EPI_ISL_6931288	A/chicken/Poland/H1940-N/2021	A / H5N1	Europe / Poland / Silesian Voivodeship	Edyta ?wi?to? (National Veterinary Research Institut Poland, PIWet-PIB)
EPI_ISL_6934175	A/domestic_duck/Poland/H1942-N/2021	A / H5N1	Europe / Poland / Lodz Voivodeship	Edyta ?wi?to? (National Veterinary Research Institut Poland, PIWet-PIB)
EPI_ISL_693515	A/chicken/Netherlands/20019411-006010/2020	A / H5N8	Europe / Netherlands / South Holland / Midden-Delfland	Rene Heutink (Wageningen Bioveterinary Research)
EPI_ISL_6935584	A/turkey/Poland/H1944-N/2021	A / H5N1	Europe / Poland / Masovian Voivodeship	Edyta ?wi?to? (National Veterinary Research Institut Poland, PIWet-PIB)
EPI_ISL_6937114	A/mute_swan/Poland/MB490-L1/2021	A / H5N1	Europe / Poland / Greater Poland Voivodeship	Edyta ?wi?to? (National Veterinary Research Institut Poland, PIWet-PIB)
EPI_ISL_694042	A/chicken/Vietnam/NCVD-17A505/2017	A / H5N6	Asia / Vietnam	Yunho Jang (Centers for Disease Control and Prevention / WHO Collaborating Center for Surveillance, Epidemiology and Control of Influ
EPI_ISL_6959592	A/mandarin duck/Korea/WA585/2021	A / H5N1	Asia / Korea, Republic of / Chungcheongnam-do	Yu-Na Lee (Animal and Plant Quarantine Agency (S-2158) / Avian Influenza Research & Diagnostic Division)
EPI_ISL_6959593	A/quail/Korea/H526/2021	A / H5N1	Asia / Korea, Republic of / Chungcheongbuk-do	Yu-Na Lee (Animal and Plant Quarantine Agency (S-2158) / Avian Influenza Research & Diagnostic Division)
EPI_ISL_696629	A/chicken/Shanxi/04.28_TGRL001-O/2018	A / H5N6	Asia / China / Shanxi Province	
EPI_ISL_696630	A/chicken/Shanxi/04.28_TGRL024-O/2018	A / H5N6	Asia / China / Shanxi Province	

EPI_ISL_696686	A/duck/Jiangxi/11.29_NCNP22D3-OC/2017	A / H5N6	Asia / China / Jiangxi Province
EPI_ISL_696689	A/duck/Hunan/03.07_YYGK25L3-OC/2018	A / H5N6	Asia / China / Hunan Province
EPI_ISL_696703	A/duck/Jiangxi/11.29_NCNP39D3-OC/2017	A / H5N6	Asia / China / Jiangxi Province
EPI_ISL_696707	A/duck/Hunan/7.21_YYGK41Y1-C/2016	A / H5N6	Asia / China / Hunan Province
EPI_ISL_696739	A/chicken/Hunan/03.07_YYGK74L3-OC/2018	A / H5N6	Asia / China / Hunan Province
EPI_ISL_696740	A/duck/Hunan/11.30_YYGK75E3-OC/2017	A / H5N6	Asia / China / Hunan Province
EPI_ISL_696807	A/duck/Guizhou/7.27_ZYLJJ017-O/2018	A / H5N6	Asia / China / Guizhou Province
EPI_ISL_696813	A/duck/Guizhou/8.26_ZYLJJ016-O/2018	A / H5N6	Asia / China / Guizhou Province
EPI_ISL_696836	A/chicken/Guizhou/10.28_ZYLJJ008-C/2018	A / H5N6	Asia / China / Guizhou Province
EPI_ISL_696843	A/pigeon/Hainan/1.14_HKPL006-C/2018	A / H5N6	Asia / China / Hainan Province
EPI_ISL_696943	A/duck/Yunnan/9.27_DQXY003-O/2016	A / H5N6	Asia / China / Yunnan Province
EPI_ISL_696991	A/goose/Fujian/3.15_FZHX0001-O/2018	A / H5N6	Asia / China / Fujian Province
EPI_ISL_696992	A/goose/Fujian/3.15_FZHX0007-O/2018	A / H5N6	Asia / China / Fujian Province
EPI_ISL_696993	A/goose/Fujian/3.15_FZHX0010-O/2018	A / H5N6	Asia / China / Fujian Province
EPI_ISL_696994	A/goose/Fujian/3.15_FZHX0005-C/2018	A / H5N6	Asia / China / Fujian Province
EPI_ISL_696995	A/goose/Fujian/3.15_FZHX0007-C/2018	A / H5N6	Asia / China / Fujian Province
EPI_ISL_697004	A/duck/Guangdong/7.20_DGCP036-O/2017	A / H5N6	Asia / China / Guangdong Province
EPI_ISL_697077	A/chicken/Shandong/8.28_TAWM017-O/2017	A / H5N6	Asia / China / Shandong Province
EPI_ISL_697143	A/chicken/Fujian/11.23_FZHX0014-O/2017	A / H5N6	Asia / China / Fujian Province
EPI_ISL_697144	A/chicken/Fujian/11.23_FZHX0015-O/2017	A / H5N6	Asia / China / Fujian Province
EPI_ISL_697188	A/duck/Fujian/10.26_FZHX0034-C/2017	A / H5N6	Asia / China / Fujian Province
EPI_ISL_697189	A/duck/Fujian/11.09_FZHX-O/2017	A / H5N6	Asia / China / Fujian Province
EPI_ISL_697210	A/goose/Fujian/11.23_FZHX0008-O/2017	A / H5N6	Asia / China / Fujian Province
EPI_ISL_697211	A/goose/Fujian/11.23_FZHX0009-O/2017	A / H5N6	Asia / China / Fujian Province
EPI_ISL_697214	A/goose/Fujian/3.15_FZHX0005-O/2018	A / H5N6	Asia / China / Fujian Province
EPI_ISL_697216	A/goose/Fujian/3.15_FZHX0008-O/2018	A / H5N6	Asia / China / Fujian Province
EPI_ISL_697697	A/chicken/Egypt/ME-2018/2018	A / H5N8	Africa / Egypt
EPI_ISL_697771	A/northern pintail/Hokkaido/M13/2020	A / H5N8	Asia / Japan / Hokkaido
EPI_ISL_697804	A/chicken/Shandong/2.28_TAWM016-C/2017	A / H5N6	Asia / China / Shandong Province
EPI_ISL_697808	A/chicken/Fujian/11.23_FZHX0006-O/2017	A / H5N6	Asia / China / Fujian Province
EPI_ISL_697819	A/chicken/Guizhou/12.20_ZYLJJ017-O/2017	A / H5N6	Asia / China / Guizhou Province
EPI_ISL_697826	A/chicken/Fujian/3.15_FZHX0009-C/2018	A / H5N2	Asia / China / Fujian Province
EPI_ISL_697829	A/duck/Fujian/11.23_FZHX0010-C/2017	A / H5N2	Asia / China / Fujian Province
EPI_ISL_697830	A/duck/Fujian/11.23_FZHX0018-O/2017	A / H5N2	Asia / China / Fujian Province
EPI_ISL_697831	A/duck/Fujian/11.23_FZHX0020-O/2017	A / H5N2	Asia / China / Fujian Province
EPI_ISL_697833	A/chicken/Guizhou/8.26_ZYLJJ014-O/2018	A / H5N6	Asia / China / Guizhou Province
EPI_ISL_697834	A/chicken/Shandong/12.26_TAWL007-O/2018	A / H5N6	Asia / China / Shandong Province
EPI_ISL_697835	A/chicken/Shandong/12.26_TAWL015-O/2018	A / H5N6	Asia / China / Shandong Province
EPI_ISL_697837	A/duck/Henan/01.01_LYGL003-O/2017	A / H5N6	Asia / China / Henan Province
EPI_ISL_697839	A/chicken/Shanxi/11.30_TGRL017-O/2018	A / H5N6	Asia / China / Shanxi Province
EPI_ISL_697840	A/chicken/Shanxi/11.30_TGRL008-C/2018	A / H5N6	Asia / China / Shanxi Province
EPI_ISL_697841	A/chicken/Shanxi/12.01_TGRL025-C/2018	A / H5N6	Asia / China / Shanxi Province
EPI_ISL_697842	A/chicken/Henan/10.31_XXHM015-C/2018	A / H5N6	Asia / China / Henan Province
EPI_ISL_697846	A/chicken/Henan/11.29_XXHM014-C/2018	A / H5N6	Asia / China / Henan Province
EPI_ISL_697847	A/chicken/Shanxi/11.30_TGRL008-O/2018	A / H5N6	Asia / China / Shanxi Province
EPI_ISL_697848	A/chicken/Shanxi/12.01_TGRL016-O/2018	A / H5N6	Asia / China / Shanxi Province
EPI_ISL_697851	A/chicken/Hunan/12.27_YYGK19J2-C/2016	A / H5N6	Asia / China / Hunan Province
EPI_ISL_697884	A/chicken/Hunan/03.07_YYGK60L3-OC/2018	A / H5N2	Asia / China / Hunan Province
EPI_ISL_697885	A/chicken/Hunan/3.07_YYGK81L3-OC/2018	A / H5N6	Asia / China / Hunan Province
EPI_ISL_697888	A/goose/Hunan/1.11_YYGK92E3-OC/2018	A / H5N6	Asia / China / Hunan Province
EPI_ISL_697891	A/chicken/Anhui/9.29_YHZGS014-O/2018	A / H5N6	Asia / China / Anhui Province
EPI_ISL_697897	A/environment/Anhui/01.20_YHZGS003-E/2019	A / H5N6	Asia / China / Anhui Province
EPI_ISL_697898	A/environment/Anhui/01.20_YHZGS004-E/2019	A / H5N2	Asia / China / Anhui Province
EPI_ISL_697901	A/chicken/Shandong/01.26_TAWL003-O/2019	A / H5N6	Asia / China / Shandong Province
EPI_ISL_697903	A/chicken/Shandong/01.26_TAWL011-O/2019	A / H5N6	Asia / China / Shandong Province
EPI_ISL_697904	A/chicken/Shandong/01.26_TAWL017-O/2019	A / H5N6	Asia / China / Shandong Province
EPI_ISL_697905	A/chicken/Shandong/01.26_TAWL022-O/2019	A / H5N2	Asia / China / Shandong Province
EPI_ISL_697907	A/chicken/Shandong/01.26_TAWL007-C/2019	A / H5N6	Asia / China / Shandong Province
EPI_ISL_697908	A/chicken/Shandong/01.26_TAWL009-C/2019	A / H5N6	Asia / China / Shandong Province
EPI_ISL_697920	A/duck/Zhejiang/8.25_HZBX003-O/2018	A / H5	Asia / China / Zhejiang Province
EPI_ISL_697941	A/duck/Zhejiang/11.09_HZBX009-C/2018	A / H5	Asia / China / Zhejiang Province
EPI_ISL_697946	A/duck/Zhejiang/12.28_HZBX012-O/2018	A / H5	Asia / China / Zhejiang Province
EPI_ISL_697963	A/chicken/Guangxi/8.25_NNXH008-O/2018	A / H5	Asia / China / Guangxi Zhuang Autonomous Region
EPI_ISL_697983	A/chicken/Yunnan/11.22_DQWGH005-O/2018	A / H5	Asia / China / Yunnan Province
EPI_ISL_697984	A/environment/Yunnan/11.22_DQWGH002-E/2018	A / H5N6	Asia / China / Yunnan Province
EPI_ISL_697985	A/chicken/Fujian/9.24_FZHX0067-O/2018	A / H5N6	Asia / China / Fujian Province
EPI_ISL_697986	A/chicken/Fujian/9.24_FZHX0068-C/2018	A / H5N6	Asia / China / Fujian Province
EPI_ISL_697987	A/chicken/Fujian/9.24_FZHX0071-O/2018	A / H5	Asia / China / Fujian Province
EPI_ISL_697989	A/chicken/Fujian/9.24_FZHX0076-O/2018	A / H5	Asia / China / Fujian Province
EPI_ISL_697990	A/duck/Fujian/1.25_FZHX0041-O/2018	A / H5	Asia / China / Fujian Province
EPI_ISL_697991	A/chicken/Fujian/9.24_FZHX0088-C/2018	A / H5N6	Asia / China / Fujian Province
EPI_ISL_698002	A/chicken/Jiangxi/4.25_NCDZT53Q3-OC/2018	A / H5N6	Asia / China / Jiangxi Province

EPI_ISL_698004	A/duck/Jiangxi/6.21_NCNP83S3-OC/2018	A / H5N6	Asia / China / Jiangxi Province	
EPI_ISL_698008	A/duck/Jiangxi/12.17_NCNP71D4-O/2018	A / H5N6	Asia / China / Jiangxi Province	
EPI_ISL_698009	A/duck/Hunan/04.26_YYGK75R3-OC/2018	A / H5N6	Asia / China / Hunan Province	
EPI_ISL_698010	A/chicken/Hunan/04.26_YYGK19R3-OC/2018	A / H5N6	Asia / China / Hunan Province	
EPI_ISL_698011	A/duck/Hunan/06.22_YYGK90T3-OC/2018	A / H5N6	Asia / China / Hunan Province	
EPI_ISL_698012	A/chicken/Hunan/07.26_YYGK28V3-OC/2018	A / H5N6	Asia / China / Hunan Province	
EPI_ISL_698013	A/chicken/Hunan/7.26_YYGK56V3-OC/2018	A / H5N6	Asia / China / Hunan Province	
EPI_ISL_698015	A/chicken/Jiangxi/5.28_NCDZT44N3-OC/2018	A / H5	Asia / China / Jiangxi Province	
EPI_ISL_698017	A/duck/Jiangxi/5.28_NCNP34N3-OC/2018	A / H5	Asia / China / Jiangxi Province	
EPI_ISL_698020	A/duck/Jiangxi/05.28_NCNP30N3-OC/2018	A / H5	Asia / China / Jiangxi Province	
EPI_ISL_698022	A/duck/Jiangxi/05.28_NCNP17N3-OC/2018	A / H5	Asia / China / Jiangxi Province	
EPI_ISL_698025	A/duck/Jiangxi/5.28_NCNP12N3-OC/2018	A / H5	Asia / China / Jiangxi Province	
EPI_ISL_698031	A/chicken/Jiangxi/09.22_NCNP6Z3-OC/2018	A / H5	Asia / China / Jiangxi Province	
EPI_ISL_698034	A/duck/Jiangxi/9.22_NCDZT76Z3-OC/2018	A / H5	Asia / China / Jiangxi Province	
EPI_ISL_698045	A/chicken/Hunan/4.26_YYGK62R3-OC/2018	A / H5	Asia / China / Hunan Province	
EPI_ISL_698047	A/duck/Hunan/4.26_YYGK69R3-OC/2018	A / H5	Asia / China / Hunan Province	
EPI_ISL_698052	A/chicken/Hunan/04.26_YYGK37R3-OC/2018	A / H5	Asia / China / Hunan Province	
EPI_ISL_698060	A/duck/Hunan/09.22_YYGK26Y3-OC/2018	A / H5	Asia / China / Hunan Province	
EPI_ISL_698070	A/chicken/Anhui/2.22_YHZGS003-O/2019	A / H5N6	Asia / China / Anhui Province	
EPI_ISL_698071	A/chicken/Anhui/2.22_YHZGS007-O/2019	A / H5N2	Asia / China / Anhui Province	
EPI_ISL_699166	A/chicken/Anhui/8.28_YHZGS017-O/2018	A / H5N6	Asia / China / Anhui Province	
EPI_ISL_699204	A/chicken/Fujian/11.23_FZHX0007-O/2017	A / H5N6	Asia / China / Fujian Province	
EPI_ISL_699429	A/chicken/Hunan/02.06_YYGK37J3-OC/2018	A / H5N6	Asia / China / Hunan Province	
EPI_ISL_699451	A/chicken/Hunan/12.27_YYGK55J2-O/2016	A / H5N6	Asia / China / Hunan Province	
EPI_ISL_699454	A/chicken/Hunan/12.27_YYGKK148-OC/2016	A / H5N6	Asia / China / Hunan Province	
EPI_ISL_7049600	A/bean goose/Sweden/SVA211111SZ0372/FB004482/2021	A / H5N1	Europe / Sweden / Skane Lan / Simrishamns Kommun	Siamak Zohari (National Veterinary Institute)
EPI_ISL_7050532	A/western jackdaw/Sweden/SVA211111SZ0376/FB004483/202	A / H5N1	Europe / Sweden / Skane Lan / Malmo Kommun	Siamak Zohari (National Veterinary Institute)
EPI_ISL_7053000	A/common buzzard/Sweden/SVA211111SZ0376/FB004484/20	A / H5N1	Europe / Sweden / Skane Lan / Malmo Kommun	Siamak Zohari (National Veterinary Institute)
EPI_ISL_7053817	A/barnacle goose/Sweden/SVA211111SZ0376/FB004496/2021	A / H5N1	Europe / Sweden / Skane Lan / Malmo Kommun	Siamak Zohari (National Veterinary Institute)
EPI_ISL_7054529	A/greylag goose /Sweden/SVA211111SZ0376/FB004497/M-20	A / H5N1	Europe / Sweden / Skane Lan / Malmo Kommun	Siamak Zohari (National Veterinary Institute)
EPI_ISL_7054770	A/European herring gull/Sweden/SVA211116SZ0432/FB00451	A / H5N1	Europe / Sweden / Hallands Lan / Hylte Kommun	Siamak Zohari (National Veterinary Institute)
EPI_ISL_7055384	A/greylag goose /Sweden/SVA211118SZ0354/FB004497/I-202	A / H5N1	Europe / Sweden / Kalmar Lan / Kalmar Kommun	Siamak Zohari (National Veterinary Institute)
EPI_ISL_707015	A/Whooper swan/Mongolia/24/2020	A / H5N6	Asia / Mongolia	
EPI_ISL_707016	A/Whooper swan/Mongolia/25/2020	A / H5N6	Asia / Mongolia	
EPI_ISL_707363	A/chicken/Shanxi/12.01_TGYL014-C/2016	A / H5N6	Asia / China / Shanxi Province	
EPI_ISL_707384	A/duck/Fujian/1.17_FZHX0123-C/2017	A / H5N6	Asia / China / Fujian Province	
EPI_ISL_707414	A/duck/Fujian/10.26_FZHX0002-O/2017	A / H5N6	Asia / China / Fujian Province	
EPI_ISL_707415	A/duck/Fujian/10.26_FZHX0010-O/2017	A / H5N6	Asia / China / Fujian Province	
EPI_ISL_707417	A/duck/Fujian/11.23_FZHX0009-C/2017	A / H5N6	Asia / China / Fujian Province	
EPI_ISL_707418	A/duck/Fujian/11.23_FZHX0014-C/2017	A / H5N6	Asia / China / Fujian Province	
EPI_ISL_707419	A/duck/Fujian/11.23_FZHX0015-C/2017	A / H5N6	Asia / China / Fujian Province	
EPI_ISL_707421	A/duck/Fujian/11.3_FZHX1132-O/2016	A / H5N6	Asia / China / Fujian Province	
EPI_ISL_707422	A/duck/Fujian/11.3_FZHX1141-C/2016	A / H5N6	Asia / China / Fujian Province	
EPI_ISL_707425	A/duck/Fujian/12.7_FZHX1202-O/2016	A / H5N6	Asia / China / Fujian Province	
EPI_ISL_707426	A/duck/Fujian/12.7_FZHX1218-C/2016	A / H5N6	Asia / China / Fujian Province	
EPI_ISL_707427	A/duck/Fujian/12.7_FZHX1219-O/2016	A / H5N6	Asia / China / Fujian Province	
EPI_ISL_707428	A/duck/Fujian/12.7_FZHX1221-C/2016	A / H5N6	Asia / China / Fujian Province	
EPI_ISL_707453	A/duck/Guangdong/11.18_SZBJ003-C/2016	A / H5N6	Asia / China / Guangdong Province	
EPI_ISL_707454	A/duck/Guangdong/11.18_SZBJ003-O/2016	A / H5N6	Asia / China / Guangdong Province	
EPI_ISL_707455	A/duck/Guangdong/11.18_SZBJ007-O/2016	A / H5N6	Asia / China / Guangdong Province	
EPI_ISL_707465	A/duck/Guizhou/8.26_ZYLJJ017-O/2018	A / H5N6	Asia / China / Guizhou Province	
EPI_ISL_707469	A/duck/Hainan/12.29_HKPL002-C/2017	A / H5N6	Asia / China / Hainan Province	
EPI_ISL_707475	A/duck/Hunan/01.12_YYGK82H3-OC/2018	A / H5N6	Asia / China / Hunan Province	
EPI_ISL_707477	A/duck/Hunan/03.07_YYGK4L3-OC/2018	A / H5N6	Asia / China / Hunan Province	
EPI_ISL_707478	A/duck/Hunan/03.24_YYGK174M2-C/2017	A / H5N6	Asia / China / Hunan Province	
EPI_ISL_707479	A/duck/Hunan/03.24_YYGK178M2-C/2017	A / H5N6	Asia / China / Hunan Province	
EPI_ISL_707480	A/duck/Hunan/03.24_YYGK18M2-O/2017	A / H5N6	Asia / China / Hunan Province	
EPI_ISL_707481	A/duck/Hunan/03.24_YYGK21M2-O/2017	A / H5N6	Asia / China / Hunan Province	
EPI_ISL_707489	A/duck/Hunan/1.12_YYGK68H3-OC/2018	A / H5N6	Asia / China / Hunan Province	
EPI_ISL_707490	A/duck/Hunan/1.12_YYGK72H3-OC/2018	A / H5N6	Asia / China / Hunan Province	
EPI_ISL_707491	A/duck/Hunan/1.12_YYGK74H3-OC/2018	A / H5N6	Asia / China / Hunan Province	
EPI_ISL_707493	A/duck/Hunan/1.17_YYGKK80-OC/2017	A / H5N6	Asia / China / Hunan Province	
EPI_ISL_707500	A/duck/Hunan/11.30_YYGK59E3-OC/2017	A / H5N6	Asia / China / Hunan Province	
EPI_ISL_707501	A/duck/Hunan/11.30_YYGK62E3-OC/2017	A / H5N6	Asia / China / Hunan Province	
EPI_ISL_707502	A/duck/Hunan/11.30_YYGK63E3-OC/2017	A / H5N6	Asia / China / Hunan Province	
EPI_ISL_707503	A/duck/Hunan/11.30_YYGK74E3-OC/2017	A / H5N8	Asia / China / Hunan Province	
EPI_ISL_707504	A/duck/Hunan/12.27_YYGK100J2-C/2016	A / H5N6	Asia / China / Hunan Province	
EPI_ISL_707506	A/duck/Hunan/12.27_YYGK60J2-C/2016	A / H5N6	Asia / China / Hunan Province	
EPI_ISL_707507	A/duck/Hunan/12.27_YYGK71J2-O/2016	A / H5N6	Asia / China / Hunan Province	
EPI_ISL_707508	A/duck/Hunan/12.27_YYGK73J2-O/2016	A / H5N6	Asia / China / Hunan Province	
EPI_ISL_707510	A/duck/Hunan/12.27_YYGK81J2-O/2016	A / H5N6	Asia / China / Hunan Province	
EPI_ISL_707512	A/duck/Hunan/12.27_YYGK86J2-C/2016	A / H5N6	Asia / China / Hunan Province	

EPI_ISL_707515	A/duck/Hunan/12.27_YYGK89J2-O/2016	A / H5N6	Asia / China / Hunan Province	
EPI_ISL_707516	A/duck/Hunan/12.27_YYGK91J2-O/2016	A / H5N6	Asia / China / Hunan Province	
EPI_ISL_707517	A/duck/Hunan/12.27_YYGK93J2-O/2016	A / H5N6	Asia / China / Hunan Province	
EPI_ISL_707519	A/duck/Hunan/12.27_YYGK95J2-O/2016	A / H5N6	Asia / China / Hunan Province	
EPI_ISL_707520	A/duck/Hunan/12.27_YYGK96J2-O/2016	A / H5N6	Asia / China / Hunan Province	
EPI_ISL_707521	A/duck/Hunan/12.27_YYGK97J2-O/2016	A / H5N6	Asia / China / Hunan Province	
EPI_ISL_707523	A/duck/Hunan/2.06_YYGK77J3-OC/2018	A / H5N6	Asia / China / Hunan Province	
EPI_ISL_707524	A/duck/Hunan/2.06_YYGK78J3-OC/2018	A / H5N6	Asia / China / Hunan Province	
EPI_ISL_707525	A/duck/Hunan/2.06_YYGK84J3-OC/2018	A / H5N6	Asia / China / Hunan Province	
EPI_ISL_707527	A/duck/Hunan/2.06_YYGK92J3-OC/2018	A / H5N6	Asia / China / Hunan Province	
EPI_ISL_707533	A/duck/Jiangxi/01.11_NCNP101G2-OC/2017	A / H5N6	Asia / China / Jiangxi Province	
EPI_ISL_707543	A/duck/Jiangxi/11.17_NCNP30Y2-O/2016	A / H5N6	Asia / China / Jiangxi Province	
EPI_ISL_707544	A/duck/Jiangxi/11.17_NCNP48Y2-C/2016	A / H5N6	Asia / China / Jiangxi Province	
EPI_ISL_707546	A/duck/Jiangxi/11.29_NCDZT71H2-O/2016	A / H5N6	Asia / China / Jiangxi Province	
EPI_ISL_707554	A/duck/Jiangxi/2.28NCNP23K3-OC/2018	A / H5N6	Asia / China / Jiangxi Province	
EPI_ISL_707555	A/duck/Jiangxi/2.28_NCNP25K3-OC/2018	A / H5N6	Asia / China / Jiangxi Province	
EPI_ISL_707570	A/duck/Jiangxi/5.26_NCDZT80Q2-OC/2017	A / H5N6	Asia / China / Jiangxi Province	
EPI_ISL_707581	A/duck/Xinjiang/12.18_WLMQXL001-C/2016	A / H5N8	Asia / China / Xinjiang Uyghur Autonomous Region	
EPI_ISL_707641	A/goose/Fujian/10.11_FZHX1001-C/2016	A / H5N6	Asia / China / Fujian Province	
EPI_ISL_707642	A/goose/Fujian/10.11_FZHX1002-O/2016	A / H5N6	Asia / China / Fujian Province	
EPI_ISL_707643	A/goose/Fujian/10.26_FZHX0002-C/2017	A / H5N6	Asia / China / Fujian Province	
EPI_ISL_707644	A/goose/Fujian/10.26_FZHX0002-O/2017	A / H5N6	Asia / China / Fujian Province	
EPI_ISL_707645	A/goose/Fujian/10.26_FZHX0004-C/2017	A / H5N6	Asia / China / Fujian Province	
EPI_ISL_707646	A/goose/Fujian/10.26_FZHX0004-O/2017	A / H5N6	Asia / China / Fujian Province	
EPI_ISL_707647	A/goose/Fujian/10.26_FZHX0006-C/2017	A / H5N6	Asia / China / Fujian Province	
EPI_ISL_707648	A/goose/Fujian/10.26_FZHX0006-O/2017	A / H5N6	Asia / China / Fujian Province	
EPI_ISL_707649	A/goose/Fujian/10.26_FZHX0007-O/2017	A / H5N6	Asia / China / Fujian Province	
EPI_ISL_707650	A/goose/Fujian/10.26_FZHX0008-C/2017	A / H5N6	Asia / China / Fujian Province	
EPI_ISL_707651	A/goose/Fujian/10.26_FZHX0014-O/2017	A / H5N6	Asia / China / Fujian Province	
EPI_ISL_707652	A/goose/Fujian/11.3_FZHX1102-C/2016	A / H5N6	Asia / China / Fujian Province	
EPI_ISL_707653	A/goose/Fujian/3.15_FZHX0008-C/2018	A / H5N6	Asia / China / Fujian Province	
EPI_ISL_707658	A/goose/Guangdong/7.20_DGCP010-C/2017	A / H5N6	Asia / China / Guangdong Province	
EPI_ISL_707659	A/goose/Henan/01.01_LYGL001-O/2017	A / H5N6	Asia / China / Henan Province	
EPI_ISL_707660	A/goose/Xinjiang/11.29_WLMQXL001-O/2017	A / H5N6	Asia / China / Xinjiang Uyghur Autonomous Region	
EPI_ISL_707661	A/goose/Xinjiang/12.18_WLMQXL001-C/2016	A / H5N6	Asia / China / Xinjiang Uyghur Autonomous Region	
EPI_ISL_707662	A/goose/Xinjiang/12.18_WLMQXL002-O/2016	A / H5N6	Asia / China / Xinjiang Uyghur Autonomous Region	
EPI_ISL_707663	A/goose/Xinjiang/12.18_WLMQXL003-C/2016	A / H5N8	Asia / China / Xinjiang Uyghur Autonomous Region	
EPI_ISL_707664	A/goose/Xinjiang/12.18_WLMQXL003-O/2016	A / H5N8	Asia / China / Xinjiang Uyghur Autonomous Region	
EPI_ISL_707665	A/goose/Xinjiang/12.18_WLMQXL004-C/2016	A / H5N8	Asia / China / Xinjiang Uyghur Autonomous Region	
EPI_ISL_707666	A/goose/Xinjiang/12.18_WLMQXL004-O/2016	A / H5N8	Asia / China / Xinjiang Uyghur Autonomous Region	
EPI_ISL_707676	A/pigeon/Fujian/1.17_FZHX0104-O/2017	A / H5N6	Asia / China / Fujian Province	
EPI_ISL_708701	A/chicken/Kagawa/I1C/2020	A / H5N8	Asia / Japan / Kagawa	Takehiko Saito (National Institute of Animal Health)
EPI_ISL_708702	A/chicken/Kagawa/I1T/2020	A / H5N8	Asia / Japan / Kagawa	Takehiko Saito (National Institute of Animal Health)
EPI_ISL_708703	A/chicken/Kagawa/I2C/2020	A / H5N8	Asia / Japan / Kagawa	Takehiko Saito (National Institute of Animal Health)
EPI_ISL_708704	A/chicken/Kagawa/I2T/2020	A / H5N8	Asia / Japan / Kagawa	Takehiko Saito (National Institute of Animal Health)
EPI_ISL_708705	A/chicken/Kagawa/I4C/2020	A / H5N8	Asia / Japan / Kagawa	Takehiko Saito (National Institute of Animal Health)
EPI_ISL_708706	A/chicken/Kagawa/I4T/2020	A / H5N8	Asia / Japan / Kagawa	Takehiko Saito (National Institute of Animal Health)
EPI_ISL_708707	A/chicken/Kagawa/I5C/2020	A / H5N8	Asia / Japan / Kagawa	Takehiko Saito (National Institute of Animal Health)
EPI_ISL_708708	A/chicken/Kagawa/I5T/2020	A / H5N8	Asia / Japan / Kagawa	Takehiko Saito (National Institute of Animal Health)
EPI_ISL_708709	A/chicken/Kagawa/I6C/2020	A / H5N8	Asia / Japan / Kagawa	Takehiko Saito (National Institute of Animal Health)
EPI_ISL_708710	A/chicken/Kagawa/I6T/2020	A / H5N8	Asia / Japan / Kagawa	Takehiko Saito (National Institute of Animal Health)
EPI_ISL_708711	A/chicken/Kagawa/J3C/2020	A / H5N8	Asia / Japan / Kagawa	Takehiko Saito (National Institute of Animal Health)
EPI_ISL_708712	A/chicken/Kagawa/J3T/2020	A / H5N8	Asia / Japan / Kagawa	Takehiko Saito (National Institute of Animal Health)
EPI_ISL_708713	A/chicken/Kagawa/J4C/2020	A / H5N8	Asia / Japan / Kagawa	Takehiko Saito (National Institute of Animal Health)
EPI_ISL_708714	A/chicken/Kagawa/J4T/2020	A / H5N8	Asia / Japan / Kagawa	Takehiko Saito (National Institute of Animal Health)
EPI_ISL_708715	A/chicken/Kagawa/J7C/2020	A / H5N8	Asia / Japan / Kagawa	Takehiko Saito (National Institute of Animal Health)
EPI_ISL_708716	A/chicken/Kagawa/J7T/2020	A / H5N8	Asia / Japan / Kagawa	Takehiko Saito (National Institute of Animal Health)
EPI_ISL_708717	A/chicken/Kagawa/J8C/2020	A / H5N8	Asia / Japan / Kagawa	Takehiko Saito (National Institute of Animal Health)
EPI_ISL_708718	A/chicken/Kagawa/J8T/2020	A / H5N8	Asia / Japan / Kagawa	Takehiko Saito (National Institute of Animal Health)
EPI_ISL_708719	A/chicken/Kagawa/J9C/2020	A / H5N8	Asia / Japan / Kagawa	Takehiko Saito (National Institute of Animal Health)
EPI_ISL_708720	A/chicken/Kagawa/J9T/2020	A / H5N8	Asia / Japan / Kagawa	Takehiko Saito (National Institute of Animal Health)
EPI_ISL_710434	A/chicken/Hiroshima/1T/2020	A / H5N8	Asia / Japan / Hiroshima	Takehiko Saito (National Institute of Animal Health)
EPI_ISL_710435	A/chicken/Hiroshima/2T/2020	A / H5N8	Asia / Japan / Hiroshima	Takehiko Saito (National Institute of Animal Health)
EPI_ISL_710436	A/chicken/Hiroshima/3T/2020	A / H5N8	Asia / Japan / Hiroshima	Takehiko Saito (National Institute of Animal Health)
EPI_ISL_710437	A/chicken/Hiroshima/4T/2020	A / H5N8	Asia / Japan / Hiroshima	Takehiko Saito (National Institute of Animal Health)
EPI_ISL_710438	A/chicken/Miyazaki/1T/2020	A / H5N8	Asia / Japan / Miyazaki	Takehiko Saito (National Institute of Animal Health)
EPI_ISL_710439	A/chicken/Miyazaki/2T/2020	A / H5N8	Asia / Japan / Miyazaki	Takehiko Saito (National Institute of Animal Health)
EPI_ISL_710440	A/chicken/Miyazaki/4T/2020	A / H5N8	Asia / Japan / Miyazaki	Takehiko Saito (National Institute of Animal Health)
EPI_ISL_710441	A/chicken/Miyazaki/6T/2020	A / H5N8	Asia / Japan / Miyazaki	Takehiko Saito (National Institute of Animal Health)
EPI_ISL_710442	A/chicken/Miyazaki/B10T/2020	A / H5N8	Asia / Japan / Miyazaki	Takehiko Saito (National Institute of Animal Health)
EPI_ISL_710443	A/chicken/Miyazaki/B1T/2020	A / H5N8	Asia / Japan / Miyazaki	Takehiko Saito (National Institute of Animal Health)
EPI_ISL_710444	A/chicken/Miyazaki/B2T/2020	A / H5N8	Asia / Japan / Miyazaki	Takehiko Saito (National Institute of Animal Health)

EPI_ISL_710445	A/chicken/Miyazaki/B6T/2020	A / H5N8	Asia / Japan / Miyazaki	Takehiko Saito (National Institute of Animal Health)
EPI_ISL_710446	A/chicken/Miyazaki/C4T/2020	A / H5N8	Asia / Japan / Miyazaki	Takehiko Saito (National Institute of Animal Health)
EPI_ISL_710447	A/chicken/Miyazaki/C6T/2020	A / H5N8	Asia / Japan / Miyazaki	Takehiko Saito (National Institute of Animal Health)
EPI_ISL_710448	A/chicken/Miyazaki/C7T/2020	A / H5N8	Asia / Japan / Miyazaki	Takehiko Saito (National Institute of Animal Health)
EPI_ISL_710449	A/chicken/Miyazaki/C8T/2020	A / H5N8	Asia / Japan / Miyazaki	Takehiko Saito (National Institute of Animal Health)
EPI_ISL_710450	A/chicken/Nara/12T/2020	A / H5N8	Asia / Japan / Nara	Takehiko Saito (National Institute of Animal Health)
EPI_ISL_710451	A/chicken/Nara/1C/2020	A / H5N8	Asia / Japan / Nara	Takehiko Saito (National Institute of Animal Health)
EPI_ISL_710452	A/chicken/Nara/5T/2020	A / H5N8	Asia / Japan / Nara	Takehiko Saito (National Institute of Animal Health)
EPI_ISL_710453	A/chicken/Nara/9T/2020	A / H5N8	Asia / Japan / Nara	Takehiko Saito (National Institute of Animal Health)
EPI_ISL_710504	A/turkey/England/037784/2020	A / H5N8	Europe / United Kingdom / North Yorkshire	Alex Byrne (Animal and Plant Health Agency (APHA) / Virology Department)
EPI_ISL_710505	A/turkey/England/038115/2020	A / H5N8	Europe / United Kingdom / North Yorkshire	Alex Byrne (Animal and Plant Health Agency (APHA) / Virology Department)
EPI_ISL_710506	A/Canada_goose/England/032697/2020	A / H5N8	Europe / United Kingdom / Gloucestershire	Alex Byrne (Animal and Plant Health Agency (APHA) / Virology Department)
EPI_ISL_710507	A/Greylag_goose/England/032698/2020	A / H5N8	Europe / United Kingdom / Gloucestershire	Alex Byrne (Animal and Plant Health Agency (APHA) / Virology Department)
EPI_ISL_710508	A/Greylag_goose/England/033100/2020	A / H5N8	Europe / United Kingdom / Gloucestershire	Alex Byrne (Animal and Plant Health Agency (APHA) / Virology Department)
EPI_ISL_710509	A/chicken/England/033708/2020	A / H5N8	Europe / United Kingdom / Hertfordshire	Alex Byrne (Animal and Plant Health Agency (APHA) / Virology Department)
EPI_ISL_710511	A/chicken/England/037052/2020	A / H5N8	Europe / United Kingdom / Leicestershire	Alex Byrne (Animal and Plant Health Agency (APHA) / Virology Department)
EPI_ISL_710512	A/whistling_duck/England/035643/2020	A / H5N8	Europe / United Kingdom / Gloucestershire	Alex Byrne (Animal and Plant Health Agency (APHA) / Virology Department)
EPI_ISL_710533	A/chicken/Netherlands/20019237-001005/2020	A / H5N8	Europe / Netherlands / South Holland / Gemeente Brielle	Rene Heutink (Wageningen Bioveterinary Research)
EPI_ISL_710538	A/chicken/Netherlands/20019226-001/2020	A / H5N8	Europe / Netherlands / Provincie Noord-Holland / Edam-Volendam	Rene Heutink (Wageningen Bioveterinary Research)
EPI_ISL_710539	A/chicken/Netherlands/20019422-001005/2020	A / H5N8	Europe / Netherlands / Provincie Friesland / Sint Annaparochie	Rene Heutink (Wageningen Bioveterinary Research)
EPI_ISL_711055	A/chicken/Netherlands/20019879-001005/2020	A / H5N1	Europe / Netherlands / Provincie Friesland / Achtkarspelen	Rene Heutink (Wageningen Bioveterinary Research)
EPI_ISL_711056	A/mute swan/Netherlands/20019252-002/2020	A / H5N8	Europe / Netherlands / South Holland / Leidschendam-Voorburg	Rene Heutink (Wageningen Bioveterinary Research)
EPI_ISL_711058	A/mute swan/Netherlands/20019255-002/2020	A / H5N8	Europe / Netherlands / Provincie Utrecht / Gemeente Woerden	Rene Heutink (Wageningen Bioveterinary Research)
EPI_ISL_711059	A/greylag goose/Netherlands/20019685-002/2020	A / H5N1	Europe / Netherlands / Provincie Utrecht / Gemeente Woerden	Rene Heutink (Wageningen Bioveterinary Research)
EPI_ISL_718106	A/chicken/Miyazaki/D5C/2020	A / H5N8	Asia / Japan / Miyazaki	Takehiko Saito (National Institute of Animal Health)
EPI_ISL_718107	A/chicken/Miyazaki/D5T/2020	A / H5N8	Asia / Japan / Miyazaki	Takehiko Saito (National Institute of Animal Health)
EPI_ISL_718108	A/chicken/Miyazaki/E1T/2020	A / H5N8	Asia / Japan / Miyazaki	Takehiko Saito (National Institute of Animal Health)
EPI_ISL_718109	A/chicken/Miyazaki/E3T/2020	A / H5N8	Asia / Japan / Miyazaki	Takehiko Saito (National Institute of Animal Health)
EPI_ISL_718110	A/chicken/Miyazaki/E6T/2020	A / H5N8	Asia / Japan / Miyazaki	Takehiko Saito (National Institute of Animal Health)
EPI_ISL_718111	A/chicken/Miyazaki/E9T/2020	A / H5N8	Asia / Japan / Miyazaki	Takehiko Saito (National Institute of Animal Health)
EPI_ISL_718112	A/chicken/Oita/27T/2020	A / H5N8	Asia / Japan / Oita	Takehiko Saito (National Institute of Animal Health)
EPI_ISL_718113	A/chicken/Oita/2T/2020	A / H5N8	Asia / Japan / Oita	Takehiko Saito (National Institute of Animal Health)
EPI_ISL_718114	A/chicken/Oita/3T/2020	A / H5N8	Asia / Japan / Oita	Takehiko Saito (National Institute of Animal Health)
EPI_ISL_718115	A/chicken/Oita/5T/2020	A / H5N8	Asia / Japan / Oita	Takehiko Saito (National Institute of Animal Health)
EPI_ISL_718116	A/chicken/Okayama/1T/2020	A / H5N8	Asia / Japan / Okayama	Takehiko Saito (National Institute of Animal Health)
EPI_ISL_718117	A/chicken/Okayama/2T/2020	A / H5N8	Asia / Japan / Okayama	Takehiko Saito (National Institute of Animal Health)
EPI_ISL_718118	A/chicken/Okayama/6T/2020	A / H5N8	Asia / Japan / Okayama	Takehiko Saito (National Institute of Animal Health)
EPI_ISL_718119	A/chicken/Okayama/7T/2020	A / H5N8	Asia / Japan / Okayama	Takehiko Saito (National Institute of Animal Health)
EPI_ISL_718120	A/chicken/Wakayama/1T/2020	A / H5N8	Asia / Japan / Wakayama	Takehiko Saito (National Institute of Animal Health)
EPI_ISL_718121	A/chicken/Wakayama/2T/2020	A / H5N8	Asia / Japan / Wakayama	Takehiko Saito (National Institute of Animal Health)
EPI_ISL_718122	A/chicken/Wakayama/3T/2020	A / H5N8	Asia / Japan / Wakayama	Takehiko Saito (National Institute of Animal Health)
EPI_ISL_718123	A/chicken/Wakayama/4T/2020	A / H5N8	Asia / Japan / Wakayama	Takehiko Saito (National Institute of Animal Health)
EPI_ISL_718223	A/barnacle goose/Sweden/SVA201125SZ0472/KN003488/202(	A / H5N8	Europe / Sweden / Gotlands Lan / Gotlands Kommun	Siamak Zohari (National Veterinary Institute)
EPI_ISL_718225	A/Jiangsu/1/2020 ?H5N6?	A / H5N6	Asia / China / Jiangsu Provice	Xian Qi (Jiangsu Provincial Center for Disease Control & Prevention)
EPI_ISL_718266	A/Jiangsu/1/2020(H5N6)	A / H5N6	Asia / China / Jiangsu Provice / Nanjing	Min He (Nanjing Center for Disease Control and Prevention / Microbiological Testing)
EPI_ISL_7192735	A/Duck/Sichuan/21022-4/2021(H5N8)	A / H5N8	Asia / China / Sichuan Province	Jiahao Zhang (South China Agricultural University / College of Veterinary Medicine)
EPI_ISL_722176	A/barnacle goose/Sweden/SVA201201SZ0353/KN003624/202(	A / H5N5	Europe / Sweden / Skane Lan / Staffanstorps Kommun	Siamak Zohari (National Veterinary Institute)
EPI_ISL_7224437	A/chicken/Czech Republic/22224-2T/2021	A / H5N1	Europe / Czech Republic / Jihocesky Kraj / Okres Jindrichuv Hradec / Straz nad Nezarkou, Dolni Ll	Alexander Nagy (State Veterinary Institute Prague)
EPI_ISL_7224454	A/chicken/Czech Republic/22224-3K/2021	A / H5N1	Europe / Czech Republic / Jihocesky Kraj / Okres Jindrichuv Hradec / Straz nad Nezarkou, Dolni Ll	Alexander Nagy (State Veterinary Institute Prague)
EPI_ISL_7224463	A/chicken/Czech Republic/22224-3T/2021	A / H5N1	Europe / Czech Republic / Jihocesky Kraj / Okres Jindrichuv Hradec / Straz nad Nezarkou, Dolni Ll	Alexander Nagy (State Veterinary Institute Prague)
EPI_ISL_7224472	A/chicken/Czech Republic/22224-4T/2021	A / H5N1	Europe / Czech Republic / Jihocesky Kraj / Okres Jindrichuv Hradec / Straz nad Nezarkou, Dolni Ll	Alexander Nagy (State Veterinary Institute Prague)
EPI_ISL_7267243	A/greylag goose/Netherlands/21038570-002/2021	A / H5N1	Europe / Netherlands / Provincie Friesland	Rene Heutink (Wageningen Bioveterinary Research)
EPI_ISL_7267244	A/common buzzard/Netherlands/21038793-001/2021	A / H5N1	Europe / Netherlands / Provincie Friesland	Rene Heutink (Wageningen Bioveterinary Research)
EPI_ISL_7267245	A/mute swan/Netherlands/21038797-001/2021	A / H5N1	Europe / Netherlands / South Holland	Rene Heutink (Wageningen Bioveterinary Research)
EPI_ISL_7267246	A/mute swan/Netherlands/21039192-001/2021	A / H5N1	Europe / Netherlands / Provincie Friesland	Rene Heutink (Wageningen Bioveterinary Research)
EPI_ISL_7267247	A/mute swan/Netherlands/21039202-001/2021	A / H5N1	Europe / Netherlands / South Holland	Rene Heutink (Wageningen Bioveterinary Research)
EPI_ISL_7267248	A/goose/Netherlands/21038940-002/2021	A / H5N1	Europe / Netherlands / Provincie Flevoland	Rene Heutink (Wageningen Bioveterinary Research)
EPI_ISL_7267249	A/grey heron/Netherlands/21038941-001/2021	A / H5N1	Europe / Netherlands / Provincie Gelderland	Rene Heutink (Wageningen Bioveterinary Research)
EPI_ISL_7267250	A/greylag goose/Netherlands/21038942-001/2021	A / H5N1	Europe / Netherlands / Provincie Friesland	Rene Heutink (Wageningen Bioveterinary Research)
EPI_ISL_7267251	A/greater canada goose /Netherlands/21038971-001/2021	A / H5N1	Europe / Netherlands / South Holland	Rene Heutink (Wageningen Bioveterinary Research)
EPI_ISL_7267252	A/goose/Netherlands/21039029-001/2021	A / H5N1	Europe / Netherlands / Provincie Noord-Holland	Rene Heutink (Wageningen Bioveterinary Research)
EPI_ISL_7267253	A/gull/Netherlands/21039180-002/2021	A / H5N1	Europe / Netherlands / Provincie Noord-Holland	Rene Heutink (Wageningen Bioveterinary Research)
EPI_ISL_7267254	A/greylag goose/Netherlands/21039376-001/2021	A / H5N1	Europe / Netherlands / Provincie Utrecht	Rene Heutink (Wageningen Bioveterinary Research)
EPI_ISL_732927	A/chicken/Kagawa/K2T/2020	A / H5N8	Asia / Japan / Kagawa	Takehiko Saito (National Institute of Animal Health)
EPI_ISL_732928	A/chicken/Kagawa/K3T/2020	A / H5N8	Asia / Japan / Kagawa	Takehiko Saito (National Institute of Animal Health)
EPI_ISL_732929	A/chicken/Kagawa/K4T/2020	A / H5N8	Asia / Japan / Kagawa	Takehiko Saito (National Institute of Animal Health)
EPI_ISL_732930	A/chicken/Kagawa/K6T/2020	A / H5N8	Asia / Japan / Kagawa	Takehiko Saito (National Institute of Animal Health)
EPI_ISL_732931	A/chicken/Kochi/4C/2020	A / H5N8	Asia / Japan / Kochi	Takehiko Saito (National Institute of Animal Health)
EPI_ISL_732932	A/chicken/Kochi/5C/2020	A / H5N8	Asia / Japan / Kochi	Takehiko Saito (National Institute of Animal Health)
EPI_ISL_732933	A/chicken/Kochi/6T/2020	A / H5N8	Asia / Japan / Kochi	Takehiko Saito (National Institute of Animal Health)
EPI_ISL_732934	A/chicken/Kochi/7T/2020	A / H5N8	Asia / Japan / Kochi	Takehiko Saito (National Institute of Animal Health)
EPI_ISL_732935	A/chicken/Miyazaki/F1T/2020	A / H5N8	Asia / Japan / Miyazaki	Takehiko Saito (National Institute of Animal Health)

EPI_ISL_732936	A/chicken/Miyazaki/F4T/2020	A / H5N8	Asia / Japan / Miyazaki	Takehiko Saito (National Institute of Animal Health)
EPI_ISL_732937	A/chicken/Miyazaki/F5T/2020	A / H5N8	Asia / Japan / Miyazaki	Takehiko Saito (National Institute of Animal Health)
EPI_ISL_732938	A/chicken/Miyazaki/F7T/2020	A / H5N8	Asia / Japan / Miyazaki	Takehiko Saito (National Institute of Animal Health)
EPI_ISL_732939	A/chicken/Miyazaki/G1T/2020	A / H5N8	Asia / Japan / Miyazaki	Takehiko Saito (National Institute of Animal Health)
EPI_ISL_732940	A/chicken/Miyazaki/G3T/2020	A / H5N8	Asia / Japan / Miyazaki	Takehiko Saito (National Institute of Animal Health)
EPI_ISL_732941	A/chicken/Miyazaki/G5T/2020	A / H5N8	Asia / Japan / Miyazaki	Takehiko Saito (National Institute of Animal Health)
EPI_ISL_732942	A/chicken/Miyazaki/G9T/2020	A / H5N8	Asia / Japan / Miyazaki	Takehiko Saito (National Institute of Animal Health)
EPI_ISL_732943	A/chicken/Shiga/4T/2020	A / H5N8	Asia / Japan / Shiga	Takehiko Saito (National Institute of Animal Health)
EPI_ISL_732944	A/chicken/Shiga/5T/2020	A / H5N8	Asia / Japan / Shiga	Takehiko Saito (National Institute of Animal Health)
EPI_ISL_732945	A/chicken/Shiga/6T/2020	A / H5N8	Asia / Japan / Shiga	Takehiko Saito (National Institute of Animal Health)
EPI_ISL_732946	A/chicken/Shiga/7T/2020	A / H5N8	Asia / Japan / Shiga	Takehiko Saito (National Institute of Animal Health)
EPI_ISL_7349517	A/Duck/Sichuan/21022-1/2021(H5N8)	A / H5N8	Asia / China / Sichuan Province	Jiahao Zhang (South China Agricultural University / College of Veterinary Medicine)
EPI_ISL_7357590	A/mute swan/Croatia/101/2021	A / H5N1	Europe / Croatia / Sisacko-Moslavacka Zupanija / Cigoc (45° 25' 16,9" N; 16° 37' 1,2" E)	Vladimir Savi? (Croatian Veterinary Institute / Poultry Centre)
EPI_ISL_7379463	A/Goose/Henan/21028/2021(H5N8)	A / H5N8	Asia / China / Henan Province	Jiahao Zhang (South China Agricultural University / College of Veterinary Medicine)
EPI_ISL_7379464	A/Duck/Sichuan/21044-2/2021(H5N8)	A / H5N8	Asia / China / Sichuan Province	Jiahao Zhang (South China Agricultural University / College of Veterinary Medicine)
EPI_ISL_7379465	A/Goose/Henan/21056-2/2021(H5N8)	A / H5N8	Asia / China / Henan Province	Jiahao Zhang (South China Agricultural University / College of Veterinary Medicine)
EPI_ISL_7379509	A/Duck/Guangdong/21057/2021(H5N8)	A / H5N8	Asia / China / Guangdong Province	Jiahao Zhang (South China Agricultural University / College of Veterinary Medicine)
EPI_ISL_7380253	A/Goose/Jiangsu/21153-2/2021(H5N8)	A / H5N8	Asia / China / Jiangsu Provice	Jiahao Zhang (South China Agricultural University / College of Veterinary Medicine)
EPI_ISL_738049	A/chicken/Kagawa/L4T/2020	A / H5N8	Asia / Japan / Kagawa	Takehiko Saito (National Institute of Animal Health)
EPI_ISL_738050	A/chicken/Kagawa/L6T/2020	A / H5N8	Asia / Japan / Kagawa	Takehiko Saito (National Institute of Animal Health)
EPI_ISL_7380500	A/Goose/Shandong/21153-3/2021(H5N8)	A / H5N8	Asia / China / Shandong Province	Jiahao Zhang (South China Agricultural University / College of Veterinary Medicine)
EPI_ISL_738051	A/chicken/Kagawa/L7T/2020	A / H5N8	Asia / Japan / Kagawa	Takehiko Saito (National Institute of Animal Health)
EPI_ISL_738052	A/chicken/Kagawa/L9T/2020	A / H5N8	Asia / Japan / Kagawa	Takehiko Saito (National Institute of Animal Health)
EPI_ISL_738053	A/chicken/Miyazaki/H1T/2020	A / H5N8	Asia / Japan / Miyazaki	Takehiko Saito (National Institute of Animal Health)
EPI_ISL_738054	A/chicken/Miyazaki/H3T/2020	A / H5N8	Asia / Japan / Miyazaki	Takehiko Saito (National Institute of Animal Health)
EPI_ISL_738055	A/chicken/Miyazaki/H6T/2020	A / H5N8	Asia / Japan / Miyazaki	Takehiko Saito (National Institute of Animal Health)
EPI_ISL_738056	A/chicken/Miyazaki/H9T/2020	A / H5N8	Asia / Japan / Miyazaki	Takehiko Saito (National Institute of Animal Health)
EPI_ISL_738057	A/chicken/Tokushima/1T/20200	A / H5N8	Asia / Japan / Tokushima	Takehiko Saito (National Institute of Animal Health)
EPI_ISL_738058	A/chicken/Tokushima/2T/2020	A / H5N8	Asia / Japan / Tokushima	Takehiko Saito (National Institute of Animal Health)
EPI_ISL_738059	A/chicken/Tokushima/3T/2020	A / H5N8	Asia / Japan / Tokushima	Takehiko Saito (National Institute of Animal Health)
EPI_ISL_738060	A/chicken/Tokushima/4T/2020	A / H5N8	Asia / Japan / Tokushima	Takehiko Saito (National Institute of Animal Health)
EPI_ISL_7380623	A/Duck/Shandong/21232-5/2021(H5N8)	A / H5N8	Asia / China / Shandong Province	Jiahao Zhang (South China Agricultural University / College of Veterinary Medicine)
EPI_ISL_7380813	A/Duck/Guangdong/21316/2021(H5N8)	A / H5N8	Asia / China / Guangdong Province	Jiahao Zhang (South China Agricultural University / College of Veterinary Medicine)
EPI_ISL_7381026	A/Chicken/Liaoning/21346-2/2021(H5N8)	A / H5N8	Asia / China / Liaoning Province	Jiahao Zhang (South China Agricultural University / College of Veterinary Medicine)
EPI_ISL_7381065	A/Goose/Shandong/21369-4/2021(H5N8)	A / H5N8	Asia / China / Shandong Province	Jiahao Zhang (South China Agricultural University / College of Veterinary Medicine)
EPI_ISL_7381103	A/Goose/Shandong/21369-5/2021(H5N8)	A / H5N8	Asia / China / Shandong Province	Jiahao Zhang (South China Agricultural University / College of Veterinary Medicine)
EPI_ISL_7381115	A/Goose/Liaoning/21640/2021(H5N8)	A / H5N8	Asia / China / Liaoning Province	Jiahao Zhang (South China Agricultural University / College of Veterinary Medicine)
EPI_ISL_7381183	A/Duck/Shandong/21644-4/2021(H5N8)	A / H5N8	Asia / China / Shandong Province	Jiahao Zhang (South China Agricultural University / College of Veterinary Medicine)
EPI_ISL_7381397	A/Duck/Shandong/21644-7/2021(H5N8)	A / H5N8	Asia / China / Shandong Province	Jiahao Zhang (South China Agricultural University / College of Veterinary Medicine)
EPI_ISL_7381423	A/Duck/Shandong/21931-8/2021(H5N8)	A / H5N8	Asia / China / Shandong Province	Jiahao Zhang (South China Agricultural University / College of Veterinary Medicine)
EPI_ISL_7381438	A/Duck/Guangdong/21964/2021(H5N8)	A / H5N8	Asia / China / Guangdong Province	Jiahao Zhang (South China Agricultural University / College of Veterinary Medicine)
EPI_ISL_7381448	A/Chicken/Guangxi/21989-3/2021(H5N8)	A / H5N8	Asia / China / Guangxi Zhuang Autonomous Region	Jiahao Zhang (South China Agricultural University / College of Veterinary Medicine)
EPI_ISL_7381695	A/Goose/Guangdong/211030-1/2021(H5N8)	A / H5N8	Asia / China / Guangdong Province	Jiahao Zhang (South China Agricultural University / College of Veterinary Medicine)
EPI_ISL_739684	A/goose/Russia_Novosibirsk region/1-12/2020	A / H5N8	Europe / Russian Federation / Novosibirsk region	Ivan Sobolev (Research Institute of Experimental and Clinical Medicine)
EPI_ISL_739685	A/goose/Russia_Omsk region/55-1/2020	A / H5N8	Europe / Russian Federation / Omsk region	Ivan Sobolev (Research Institute of Experimental and Clinical Medicine)
EPI_ISL_739686	A/chicken/Kazakhstan/Kn-3/2020	A / H5N8	Asia / Kazakhstan / North Kazakhstan	Ivan Sobolev (Research Institute of Experimental and Clinical Medicine)
EPI_ISL_739687	A/chicken/Kazakhstan/Kn-6/2020	A / H5N8	Asia / Kazakhstan / North Kazakhstan	Ivan Sobolev (Research Institute of Experimental and Clinical Medicine)
EPI_ISL_739688	A/chicken/Russia_Novosibirsk region/1910-1/2020	A / H5N8	Europe / Russian Federation / Novosibirsk region	Ivan Sobolev (Research Institute of Experimental and Clinical Medicine)
EPI_ISL_739689	A/chicken/Russia_Novosibirsk region/1910-2/2020	A / H5N8	Europe / Russian Federation / Novosibirsk region	Ivan Sobolev (Research Institute of Experimental and Clinical Medicine)
EPI_ISL_739690	A/chicken/Russia_Novosibirsk region/3-1/2020	A / H5N8	Europe / Russian Federation / Novosibirsk region	Ivan Sobolev (Research Institute of Experimental and Clinical Medicine)
EPI_ISL_739691	A/chicken/Russia_Novosibirsk region/3-15/2020	A / H5N8	Europe / Russian Federation / Novosibirsk region	Ivan Sobolev (Research Institute of Experimental and Clinical Medicine)
EPI_ISL_739692	A/chicken/Russia_Novosibirsk region/3-29/2020	A / H5N8	Europe / Russian Federation / Novosibirsk Oblast / Novosibirsk region	Ivan Sobolev (Research Institute of Experimental and Clinical Medicine)
EPI_ISL_7452805	A/Chicken/Sweden/SVA211130SZ0427/FB290424-IP-1/M-2021	A / H5N1	Europe / Sweden / Skane Lan / Skurups Kommun	Siamak Zohari (National Veterinary Institute)
EPI_ISL_7457654	A/Chicken/Guangdong/211106-3/2021(H5N6)	A / H5N6	Asia / China / Guangdong Province	Jiahao Zhang (South China Agricultural University / College of Veterinary Medicine)
EPI_ISL_7458702	A/Duck/Shandong/21644-1/2021(H5N8)	A / H5N8	Asia / China / Shandong Province	Jiahao Zhang (South China Agricultural University / College of Veterinary Medicine)
EPI_ISL_7570462	A/mute swan/Croatia/104/2021	A / H5N1	Europe / Croatia / Medimurska Zupanija / Gornji Kursanec (46° 19' 27.3" N; 16° 22' 45" E)	Vladimir Savi? (Croatian Veterinary Institute / Poultry Centre)
EPI_ISL_7570634	A/goose/Croatia/107/2021	A / H5N1	Europe / Croatia / Sisacko-Moslavacka Zupanija / Staro Pracno	Vladimir Savi? (Croatian Veterinary Institute / Poultry Centre)
EPI_ISL_7585764	A/Aalopochen_Aegyptiaca/Belgium/2928_002/2021	A / H5N8	Europe / Belgium / Provincie Limburg / Genk	Steven Van Borm (Sciensano, Department of Animal Infectious Diseases / Animal Infectious Diseases)
EPI_ISL_7589566	A/Gallus_gallus/Belgium/5107_002/2021	A / H5N8	Europe / Belgium / Province de Hainaut / Silly	Steven Van Borm (Sciensano, Department of Animal Infectious Diseases / Animal Infectious Diseases)
EPI_ISL_7590312	A/Branta_canadensis/Belgium/500/2021	A / H5N8	Europe / Belgium / Province de Liege / Amay	Steven Van Borm (Sciensano, Department of Animal Infectious Diseases / Animal Infectious Diseases)
EPI_ISL_7591058	A/Anas_platyrhynchos domestica/Belgium/5517/2021	A / H5N8	Europe / Belgium / Provincie West-Vlaanderen / Beveren	Steven Van Borm (Sciensano, Department of Animal Infectious Diseases / Animal Infectious Diseases)
EPI_ISL_7596571	A/Pica_pica/Belgium/12100_005/2020	A / H5N8	Europe / Belgium / Provincie West-Vlaanderen / Oudenburg	Steven Van Borm (Sciensano, Department of Animal Infectious Diseases / Animal Infectious Diseases)
EPI_ISL_7597827	A/Podiceps_cristatus/Belgium/12659_0015/2020	A / H5N8	Europe / Belgium / Provincie Oost-Vlaanderen / Gent (Blaarmeersen)	Steven Van Borm (Sciensano, Department of Animal Infectious Diseases / Animal Infectious Diseases)
EPI_ISL_759850	A/chicken/Chiba/1T/2020	A / H5N8	Asia / Japan / Chiba	Takehiko Saito (National Institute of Animal Health)
EPI_ISL_759851	A/chicken/Chiba/2T/2020	A / H5N8	Asia / Japan / Chiba	Takehiko Saito (National Institute of Animal Health)
EPI_ISL_759852	A/chicken/Chiba/3T/2020	A / H5N8	Asia / Japan / Chiba	Takehiko Saito (National Institute of Animal Health)
EPI_ISL_759853	A/chicken/Chiba/4T/2020	A / H5N8	Asia / Japan / Chiba	Takehiko Saito (National Institute of Animal Health)
EPI_ISL_759854	A/chicken/Kagawa/M10T/2020	A / H5N8	Asia / Japan / Kagawa	Takehiko Saito (National Institute of Animal Health)
EPI_ISL_759855	A/chicken/Kagawa/M11T/2020	A / H5N8	Asia / Japan / Kagawa	Takehiko Saito (National Institute of Animal Health)
EPI_ISL_759856	A/chicken/Kagawa/M12T/2020	A / H5N8	Asia / Japan / Kagawa	Takehiko Saito (National Institute of Animal Health)
EPI_ISL_759857	A/chicken/Kagawa/M2T/2020	A / H5N8	Asia / Japan / Kagawa	Takehiko Saito (National Institute of Animal Health)
EPI_ISL_7598788	A/Anser_brachyrhynchus/Belgium/13275_0009/2020	A / H5N8	Europe / Belgium / Provincie West-Vlaanderen / De Panne	Steven Van Borm (Sciensano, Department of Animal Infectious Diseases / Animal Infectious Diseases)

EPI_ISL_7622619	A/Anser_Brachyrhynchus_Anser_Anser/Belgium/13846/2020	A / H5N8	Europe / Belgium / Provincie West-Vlaanderen / Brugge and Zwin	Steven Van Borm (Sciensano, Department of Animal Infectious Diseases / Animal Infectious Diseases)
EPI_ISL_7622862	A/Anser_brachyrhynchus/Belgium/151/2020	A / H5N8	Europe / Belgium / Provincie West-Vlaanderen / Zedelgem	Steven Van Borm (Sciensano, Department of Animal Infectious Diseases / Animal Infectious Diseases)
EPI_ISL_7623043	A/Larus_argentatus/Belgium/568/2021	A / H5N8	Europe / Belgium / Provincie Oost-Vlaanderen / Merelbeke	Steven Van Borm (Sciensano, Department of Animal Infectious Diseases / Animal Infectious Diseases)
EPI_ISL_7623299	A/Columba_palumbus/Belgium/2928_004/2021	A / H5N8	Europe / Belgium / Provincie Oost-Vlaanderen / Erembodegem	Steven Van Borm (Sciensano, Department of Animal Infectious Diseases / Animal Infectious Diseases)
EPI_ISL_7623303	A/Garrulus_glandarius/Belgium/2928_003/2021	A / H5N8	Europe / Belgium / Provincie Oost-Vlaanderen / Lokeren	Steven Van Borm (Sciensano, Department of Animal Infectious Diseases / Animal Infectious Diseases)
EPI_ISL_7623511	A/Gallus_gallus/Belgium/7578_001/2021	A / H5N8	Europe / Belgium / Province de Hainaut / Le Roeulx	Steven Van Borm (Sciensano, Department of Animal Infectious Diseases / Animal Infectious Diseases)
EPI_ISL_7623789	A/Gallus_gallus/Belgium/9247/2021	A / H5N8	Europe / Belgium / Provincie Oost-Vlaanderen / Moerzeke	Steven Van Borm (Sciensano, Department of Animal Infectious Diseases / Animal Infectious Diseases)
EPI_ISL_7624142	A/Gallus_gallus/Belgium/11644/2021	A / H5N8	Europe / Belgium / Province de Luxembourg / Attert	Steven Van Borm (Sciensano, Department of Animal Infectious Diseases / Animal Infectious Diseases)
EPI_ISL_7626484	A/chicken/Czech Republic/23404-2K/2021	A / H5N1	Europe / Czech Republic / South Moravian Region / Lednice; ZipCode:69103	Alexander Nagy (State Veterinary Institute Prague)
EPI_ISL_7626506	A/chicken/Czech Republic/23404-4K/2021	A / H5N1	Europe / Czech Republic / South Moravian Region / Okres Breclav / Lednice; ZipCode:69103	Alexander Nagy (State Veterinary Institute Prague)
EPI_ISL_7626512	A/chicken/Czech Republic/23404-2T/2021	A / H5N1	Europe / Czech Republic / South Moravian Region / Okres Breclav / Lednice; ZipCode:69103	Alexander Nagy (State Veterinary Institute Prague)
EPI_ISL_7626513	A/chicken/Czech Republic/23404-4T/2021	A / H5N1	Europe / Czech Republic / South Moravian Region / Okres Breclav / Lednice; ZipCode:69103	Alexander Nagy (State Veterinary Institute Prague)
EPI_ISL_7626514	A/duck/Czech Republic/23589-1T/2021	A / H5N1	Europe / Czech Republic / Plzensky Kraj / Okres Plzen-Jih / Zdirec-Myt; ZipCode:33601	Alexander Nagy (State Veterinary Institute Prague)
EPI_ISL_7626526	A/chicken/Czech Republic/23589-1T/2021	A / H5N1	Europe / Czech Republic / Plzensky Kraj / Okres Plzen-Jih / Zdirec-Myt; ZipCode:33601	Alexander Nagy (State Veterinary Institute Prague)
EPI_ISL_7626532	A/chicken/Czech Republic/23589-3/2021	A / H5N1	Europe / Czech Republic / Plzensky Kraj / Okres Plzen-Jih / Zdirec-Myt; ZipCode:33601	Alexander Nagy (State Veterinary Institute Prague)
EPI_ISL_7626533	A/chicken/Czech Republic/23589-4/2021	A / H5N1	Europe / Czech Republic / Plzensky Kraj / Okres Plzen-Jih / Zdirec-Myt; ZipCode:33601	Alexander Nagy (State Veterinary Institute Prague)
EPI_ISL_7635907	A/white-tailed eagle/Sweden/SVA211201SZ0380/FB004721/M	A / H5N1	Europe / Sweden / Skane Lan / Svedala Kommun	Siamak Zohari (National Veterinary Institute)
EPI_ISL_766052	A/turkey/England/038730/2020	A / H5N8	Europe / United Kingdom / Norfolk	Alex Byrne (Animal and Plant Health Agency (APHA) / Virology Department)
EPI_ISL_766053	A/turkey/England/039352/2020	A / H5N8	Europe / United Kingdom / Norfolk	Alex Byrne (Animal and Plant Health Agency (APHA) / Virology Department)
EPI_ISL_766054	A/turkey/England/039472/2020	A / H5N8	Europe / United Kingdom / Norfolk	Alex Byrne (Animal and Plant Health Agency (APHA) / Virology Department)
EPI_ISL_766056	A/falcon/England/041976/2020	A / H5N8	Europe / United Kingdom / Derbyshire	Alex Byrne (Animal and Plant Health Agency (APHA) / Virology Department)
EPI_ISL_766876	A/mute_swan/England/234255/2020	A / H5N1	Europe / United Kingdom / Essex	Alex Byrne (Animal and Plant Health Agency (APHA) / Virology Department)
EPI_ISL_7683077	A/mute swan/Netherlands/21039407-001/2021	A / H5N1	Europe / Netherlands / Provincie Gelderland	Rene Heutink (Wageningen Bioveterinary Research)
EPI_ISL_7683079	A/mute swan/Netherlands/21039627-002/2021	A / H5N1	Europe / Netherlands / South Holland	Rene Heutink (Wageningen Bioveterinary Research)
EPI_ISL_7683101	A/common buzzard/Netherlands/21039607-002/2021	A / H5N1	Europe / Netherlands / Provincie Noord-Holland	Rene Heutink (Wageningen Bioveterinary Research)
EPI_ISL_7683118	A/red fox/Netherlands/21040099-001/2021	A / H5N1	Europe / Netherlands / North Brabant	Rene Heutink (Wageningen Bioveterinary Research)
EPI_ISL_7683137	A/red fox/Netherlands/21040099-006/2021	A / H5N1	Europe / Netherlands / North Brabant	Rene Heutink (Wageningen Bioveterinary Research)
EPI_ISL_7683138	A/red fox/Netherlands/21040099-007/2021	A / H5N1	Europe / Netherlands / North Brabant	Rene Heutink (Wageningen Bioveterinary Research)
EPI_ISL_7733048	A/black-headed gull/Germany-NI/AI05962/2021	A / H5N1	Europe / Germany / Lower Saxony / Aurich	Jacqueline King (Friedrich-Loeffler-Institut)
EPI_ISL_7733582	A/chicken/Italy/21VIR10388/2021	A / H5N1	Europe / Italy / BS	Bianca Zecchin (Istituto Zooprofilattico Sperimentale Delle Venezie)
EPI_ISL_7733584	A/duck/Italy/21VIR10447/2021	A / H5N1	Europe / Italy / BS	Bianca Zecchin (Istituto Zooprofilattico Sperimentale Delle Venezie)
EPI_ISL_7733585	A/Eurasian_wigeon/Italy/21VIR8919-3/2021	A / H5N1	Europe / Italy / BS	Bianca Zecchin (Istituto Zooprofilattico Sperimentale Delle Venezie)
EPI_ISL_7733587	A/turkey/Italy/21VIR9618-7/2021	A / H5N1	Europe / Italy / BS	Bianca Zecchin (Istituto Zooprofilattico Sperimentale Delle Venezie)
EPI_ISL_7733591	A/mallard/Italy/21VIR8919-2/2021	A / H5N1	Europe / Italy / BS	Bianca Zecchin (Istituto Zooprofilattico Sperimentale Delle Venezie)
EPI_ISL_7733595	A/chicken/Italy/21VIR10239/2021	A / H5N1	Europe / Italy / CR	Bianca Zecchin (Istituto Zooprofilattico Sperimentale Delle Venezie)
EPI_ISL_7733597	A/turkey/Italy/21VIR10340/2021	A / H5N1	Europe / Italy / CR	Bianca Zecchin (Istituto Zooprofilattico Sperimentale Delle Venezie)
EPI_ISL_7733598	A/turkey/Italy/21VIR9816-1/2021	A / H5N1	Europe / Italy / MN	Bianca Zecchin (Istituto Zooprofilattico Sperimentale Delle Venezie)
EPI_ISL_7733599	A/chicken/Italy/21VIR10389/2021	A / H5N1	Europe / Italy / MN	Bianca Zecchin (Istituto Zooprofilattico Sperimentale Delle Venezie)
EPI_ISL_7733603	A/chicken/Italy/21VIR9951-25/2021	A / H5N1	Europe / Italy / MN	Bianca Zecchin (Istituto Zooprofilattico Sperimentale Delle Venezie)
EPI_ISL_7733607	A/chicken/Italy/21VIR10384/2021	A / H5N1	Europe / Italy / PD	Bianca Zecchin (Istituto Zooprofilattico Sperimentale Delle Venezie)
EPI_ISL_7733609	A/chicken/Italy/21VIR9580-22/2021	A / H5N1	Europe / Italy / PD	Bianca Zecchin (Istituto Zooprofilattico Sperimentale Delle Venezie)
EPI_ISL_7733610	A/chicken/Italy/21VIR10343/2021	A / H5N1	Europe / Italy / UD	Bianca Zecchin (Istituto Zooprofilattico Sperimentale Delle Venezie)
EPI_ISL_7733611	A/Wild_goose/Italy/21VIR10193/2021	A / H5N1	Europe / Italy / VE	Bianca Zecchin (Istituto Zooprofilattico Sperimentale Delle Venezie)
EPI_ISL_7733612	A/magpie/Italy/21VIR9487-2/2021	A / H5N1	Europe / Italy / UD	Bianca Zecchin (Istituto Zooprofilattico Sperimentale Delle Venezie)
EPI_ISL_7733613	A/turkey/Italy/21VIR10456/2021	A / H5N1	Europe / Italy / VI	Bianca Zecchin (Istituto Zooprofilattico Sperimentale Delle Venezie)
EPI_ISL_7733617	A/chicken/Italy/21VIR9133-20/2021	A / H5N1	Europe / Italy / VR	Bianca Zecchin (Istituto Zooprofilattico Sperimentale Delle Venezie)
EPI_ISL_7733621	A/seagull/Italy/21VIR9432-2/2021	A / H5N1	Europe / Italy / VR	Bianca Zecchin (Istituto Zooprofilattico Sperimentale Delle Venezie)
EPI_ISL_7733624	A/turkey/Italy/21VIR9767-3/2021	A / H5N1	Europe / Italy / VR	Bianca Zecchin (Istituto Zooprofilattico Sperimentale Delle Venezie)
EPI_ISL_7733628	A/turkey/Italy/21VIR10251/2021	A / H5N1	Europe / Italy / VR	Bianca Zecchin (Istituto Zooprofilattico Sperimentale Delle Venezie)
EPI_ISL_7733629	A/turkey/Italy/21VIR9652-2/2021	A / H5N1	Europe / Italy / VR	Bianca Zecchin (Istituto Zooprofilattico Sperimentale Delle Venezie)
EPI_ISL_7733630	A/chicken/Italy/21VIR9691-2/2021	A / H5N1	Europe / Italy / VR	Bianca Zecchin (Istituto Zooprofilattico Sperimentale Delle Venezie)
EPI_ISL_7733632	A/turkey/Italy/21VIR9512-1/2021	A / H5N1	Europe / Italy / VR	Bianca Zecchin (Istituto Zooprofilattico Sperimentale Delle Venezie)
EPI_ISL_7733633	A/turkey/Italy/21VIR9143-2/2021	A / H5N1	Europe / Italy / VR	Bianca Zecchin (Istituto Zooprofilattico Sperimentale Delle Venezie)
EPI_ISL_7733634	A/chicken/Italy/21VIR9765-12/2021	A / H5N1	Europe / Italy / VR	Bianca Zecchin (Istituto Zooprofilattico Sperimentale Delle Venezie)
EPI_ISL_7733635	A/turkey/Italy/21VIR9510-1/2021	A / H5N1	Europe / Italy / VR	Bianca Zecchin (Istituto Zooprofilattico Sperimentale Delle Venezie)
EPI_ISL_7733638	A/turkey/Italy/21VIR9768-8/2021	A / H5N1	Europe / Italy / VR	Bianca Zecchin (Istituto Zooprofilattico Sperimentale Delle Venezie)
EPI_ISL_7733641	A/chicken/Italy/21VIR10352/2021	A / H5N1	Europe / Italy / VR	Bianca Zecchin (Istituto Zooprofilattico Sperimentale Delle Venezie)
EPI_ISL_7733642	A/kestrel/Italy/21VIR10468/2021	A / H5N1	Europe / Italy / VR	Bianca Zecchin (Istituto Zooprofilattico Sperimentale Delle Venezie)
EPI_ISL_7733643	A/turkey/Italy/21VIR9649-2/2021	A / H5N1	Europe / Italy / VR	Bianca Zecchin (Istituto Zooprofilattico Sperimentale Delle Venezie)
EPI_ISL_7733644	A/chicken/Italy/IZSLT-122448_21VIR9218-1/2021	A / H5N1	Europe / Italy / Roma	Bianca Zecchin (Istituto Zooprofilattico Sperimentale Delle Venezie)
EPI_ISL_7733645	A/goose/Italy/IZSLT-21VIR10273/2021	A / H5N1	Europe / Italy / Roma	Bianca Zecchin (Istituto Zooprofilattico Sperimentale Delle Venezie)
EPI_ISL_7733647	A/turkey/Italy/21VIR9210-1/2021	A / H5N1	Europe / Italy / VR	Bianca Zecchin (Istituto Zooprofilattico Sperimentale Delle Venezie)
EPI_ISL_7733648	A/turkey/Italy/21VIR8817-1/2021	A / H5N1	Europe / Italy / VR	Bianca Zecchin (Istituto Zooprofilattico Sperimentale Delle Venezie)
EPI_ISL_7748001	A/Eurasian curlew/Germany-SH/AI05960/2021	A / H5N1	Europe / Germany / Schleswig-Holstein / Nordfriesland	Jacqueline King (Friedrich-Loeffler-Institut)
EPI_ISL_7748188	A/Eurasian wigeon/Germany-SH/AI05950/2021	A / H5N1	Europe / Germany / Schleswig-Holstein / Nordfriesland	Jacqueline King (Friedrich-Loeffler-Institut)
EPI_ISL_7748262	A/Eurasian wigeon/Germany-SH/AI05952/2021	A / H5N1	Europe / Germany / Schleswig-Holstein / Nordfriesland	Jacqueline King (Friedrich-Loeffler-Institut)
EPI_ISL_775248	A/muscovy duck/Netherlands/20019914-001/2020	A / H5N8	Europe / Netherlands / South Holland / Gemeente alphen aan den Rijn	Rene Heutink (Wageningen Bioveterinary Research)
EPI_ISL_775251	A/peregrine falcon/Netherlands/20020038-001/2020	A / H5N8	Europe / Netherlands / Provincie Limburg / Gemeente Nederweert	Rene Heutink (Wageningen Bioveterinary Research)
EPI_ISL_775253	A/mute swan/Netherlands/20020133-001/2020	A / H5N8	Europe / Netherlands / Provincie Groningen / Gemeente Groningen	Rene Heutink (Wageningen Bioveterinary Research)
EPI_ISL_775266	A/black-headed gull/Netherlands/20020162-002/2020	A / H5N8	Europe / Netherlands / Provincie Flevoland / Almere	Rene Heutink (Wageningen Bioveterinary Research)
EPI_ISL_775267	A/turkey/Netherlands/21020942-001005/2021	A / H5N8	Europe / Netherlands / North Brabant / Gemeente Oisterwijk	Rene Heutink (Wageningen Bioveterinary Research)
EPI_ISL_7753130	A/Eurasian wigeon/Germany-SH/AI05955/2021	A / H5N1	Europe / Germany / Schleswig-Holstein / Nordfriesland	Jacqueline King (Friedrich-Loeffler-Institut)

EPI_ISL_7753152	A/Eurasian wigeon/Germany-SH/AI05953/2021	A / H5N1	Europe / Germany / Schleswig-Holstein / Nordfriesland	Jacqueline King (Friedrich-Loeffler-Institut)
EPI_ISL_7753173	A/Eurasian wigeon/Germany-SH/AI05956/2021	A / H5N1	Europe / Germany / Schleswig-Holstein / Nordfriesland	Jacqueline King (Friedrich-Loeffler-Institut)
EPI_ISL_7753193	A/Eurasian wigeon/Germany-SH/AI05951/2021	A / H5N1	Europe / Germany / Schleswig-Holstein / Nordfriesland	Jacqueline King (Friedrich-Loeffler-Institut)
EPI_ISL_7753211	A/Eurasian wigeon/Germany-SH/AI05954/2021	A / H5N1	Europe / Germany / Schleswig-Holstein / Nordfriesland	Jacqueline King (Friedrich-Loeffler-Institut)
EPI_ISL_7753230	A/mallard/Germany-NI/AI06010/2021	A / H5N1	Europe / Germany / Lower Saxony / Harburg	Jacqueline King (Friedrich-Loeffler-Institut)
EPI_ISL_7753251	A/barnacle goose/Germany-SH/AI06005/2021	A / H5N1	Europe / Germany / Schleswig-Holstein / Nordfriesland	Jacqueline King (Friedrich-Loeffler-Institut)
EPI_ISL_7753272	A/white-tailed eagle/Germany-MV/AI05975/2021	A / H5N1	Europe / Germany / Mecklenburg-Vorpommern / Vorpommern-Greifswald	Jacqueline King (Friedrich-Loeffler-Institut)
EPI_ISL_7753290	A/turkey/Germany-MV/AI06035/2021	A / H5N1	Europe / Germany / Mecklenburg-Vorpommern / Vorpommern-Greifswald	Jacqueline King (Friedrich-Loeffler-Institut)
EPI_ISL_7753309	A/lesser white-fronted goose/Germany-MV/AI05973/2021	A / H5N1	Europe / Germany / Mecklenburg-Vorpommern / Vorpommern-Greifswald	Jacqueline King (Friedrich-Loeffler-Institut)
EPI_ISL_7753329	A/white stork/Germany-MV/AI05979/2021	A / H5N1	Europe / Germany / Mecklenburg-Vorpommern / Vorpommern-Greifswald	Jacqueline King (Friedrich-Loeffler-Institut)
EPI_ISL_7753347	A/herring gull/Germany-SH/AI06141/2021	A / H5N1	Europe / Germany / Schleswig-Holstein / Nordfriesland	Jacqueline King (Friedrich-Loeffler-Institut)
EPI_ISL_7753367	A/Eurasian teal/Germany-BY/AI05977/2021	A / H5N1	Europe / Germany / Bayern	Jacqueline King (Friedrich-Loeffler-Institut)
EPI_ISL_7753387	A/Eurasian wigeon/Germany-SH/AI06143/2021	A / H5N1	Europe / Germany / Schleswig-Holstein / Dithmarschen	Jacqueline King (Friedrich-Loeffler-Institut)
EPI_ISL_7753396	A/domestic goose/Germany-SH/AI06147/2021	A / H5N1	Europe / Germany / Schleswig-Holstein / Dithmarschen	Jacqueline King (Friedrich-Loeffler-Institut)
EPI_ISL_7753399	A/domestic goose/Germany-SH/AI06150/2021	A / H5N1	Europe / Germany / Schleswig-Holstein / Dithmarschen	Jacqueline King (Friedrich-Loeffler-Institut)
EPI_ISL_7753400	A/domestic goose/Germany-SH/AI06024/2021	A / H5N1	Europe / Germany / Schleswig-Holstein / Dithmarschen	Jacqueline King (Friedrich-Loeffler-Institut)
EPI_ISL_7753405	A/Eurasian wigeon/Germany-SH/AI06142/2021	A / H5N1	Europe / Germany / Schleswig-Holstein / Dithmarschen	Jacqueline King (Friedrich-Loeffler-Institut)
EPI_ISL_7753406	A/greylag goose/Germany-SH/AI06144/2021	A / H5N1	Europe / Germany / Schleswig-Holstein / Dithmarschen	Jacqueline King (Friedrich-Loeffler-Institut)
EPI_ISL_7753407	A/barnacle goose/Germany-SH/AI06145/2021	A / H5N1	Europe / Germany / Schleswig-Holstein / Dithmarschen	Jacqueline King (Friedrich-Loeffler-Institut)
EPI_ISL_7753425	A/greylag goose/Germany-SH/AI06205/2021	A / H5N1	Europe / Germany / Schleswig-Holstein / Nordfriesland	Jacqueline King (Friedrich-Loeffler-Institut)
EPI_ISL_7753443	A/buzzard/Germany-SH/AI06210/2021	A / H5N1	Europe / Germany / Schleswig-Holstein / Dithmarschen	Jacqueline King (Friedrich-Loeffler-Institut)
EPI_ISL_7778753	A/Eagle_owl/Estonia/TA2122239_21VIR10433-10/2021	A / H5N8	Europe / Estonia	Bianca Zecchin (Istituto Zooprofilattico Sperimentale Delle Venezie)
EPI_ISL_7778754	A/Withe-tiled_eagle/Estonia/TA2124126-1_21VIR10433-11/20	A / H5N1	Europe / Estonia	Bianca Zecchin (Istituto Zooprofilattico Sperimentale Delle Venezie)
EPI_ISL_7778755	A/white-tailed_eagle/Estonia/TA2111864-2_21VIR7512-6/202	A / H5N1	Europe / Estonia	Bianca Zecchin (Istituto Zooprofilattico Sperimentale Delle Venezie)
EPI_ISL_7778756	A/gull/Estonia/TA2113284-4_21VIR7512-8/2021	A / H5N1	Europe / Estonia	Bianca Zecchin (Istituto Zooprofilattico Sperimentale Delle Venezie)
EPI_ISL_7778757	A/mute_swan/Estonia/TA2106111-2_21VIR7512-1/2021	A / H5N8	Europe / Estonia	Bianca Zecchin (Istituto Zooprofilattico Sperimentale Delle Venezie)
EPI_ISL_7778758	A/mute_swan/Estonia/TA2106241_21VIR7512-2/2021	A / H5N8	Europe / Estonia	Bianca Zecchin (Istituto Zooprofilattico Sperimentale Delle Venezie)
EPI_ISL_7778759	A/mute_swan/Estonia/TA2106419-1_21VIR7512-3/2021	A / H5N8	Europe / Estonia	Bianca Zecchin (Istituto Zooprofilattico Sperimentale Delle Venezie)
EPI_ISL_7778760	A/mute_swan/Estonia/TA2106615_21VIR7512-4/2021	A / H5N8	Europe / Estonia	Bianca Zecchin (Istituto Zooprofilattico Sperimentale Delle Venezie)
EPI_ISL_7778761	A/mute_swan/Estonia/TA2108545-1_21VIR7512-5/2021	A / H5N8	Europe / Estonia	Bianca Zecchin (Istituto Zooprofilattico Sperimentale Delle Venezie)
EPI_ISL_7778762	A/guinea_fowl/Estonia/TA2104719_21VIR7512-9/2021	A / H5N8	Europe / Estonia	Bianca Zecchin (Istituto Zooprofilattico Sperimentale Delle Venezie)
EPI_ISL_7778763	A/white-tailed_eagle/Finland/6984_21VIR7689-10/2021	A / H5N1	Europe / Finland	Bianca Zecchin (Istituto Zooprofilattico Sperimentale Delle Venezie)
EPI_ISL_7778764	A/white-tailed_eagle/Finland/9257_21VIR7689-11/2021	A / H5N8	Europe / Finland	Bianca Zecchin (Istituto Zooprofilattico Sperimentale Delle Venezie)
EPI_ISL_7778765	A/golden_eagle/Finland/9378_21VIR7689-12/2021	A / H5N1	Europe / Finland	Bianca Zecchin (Istituto Zooprofilattico Sperimentale Delle Venezie)
EPI_ISL_7778766	A/European_herring_gull/Finland/9722_21VIR7689-13/2021	A / H5N1	Europe / Finland	Bianca Zecchin (Istituto Zooprofilattico Sperimentale Delle Venezie)
EPI_ISL_7778767	A/whooper_swan/Finland/9906_21VIR7689-14/2021	A / H5N8	Europe / Finland	Bianca Zecchin (Istituto Zooprofilattico Sperimentale Delle Venezie)
EPI_ISL_7778768	A/Eurasian_eagle-owl/Finland/10617_21VIR7689-15/2021	A / H5N1	Europe / Finland	Bianca Zecchin (Istituto Zooprofilattico Sperimentale Delle Venezie)
EPI_ISL_7778769	A/pheasant/Finland/499_21VIR7689-1/2021	A / H5N8	Europe / Finland	Bianca Zecchin (Istituto Zooprofilattico Sperimentale Delle Venezie)
EPI_ISL_7778770	A/mute_swan/Finland/1325_21VIR7689-2/2021	A / H5N8	Europe / Finland	Bianca Zecchin (Istituto Zooprofilattico Sperimentale Delle Venezie)
EPI_ISL_7778771	A/pheasant/Finland/1589_21VIR7689-3/2021	A / H5N8	Europe / Finland	Bianca Zecchin (Istituto Zooprofilattico Sperimentale Delle Venezie)
EPI_ISL_7778773	A/barnacle_goose/Finland/6247_21VIR7689-6/2021	A / H5N1	Europe / Finland	Bianca Zecchin (Istituto Zooprofilattico Sperimentale Delle Venezie)
EPI_ISL_7778774	A/barnacle_goose/Finland//6378_21VIR7689-7/2021	A / H5N1	Europe / Finland	Bianca Zecchin (Istituto Zooprofilattico Sperimentale Delle Venezie)
EPI_ISL_7778775	A/barnacle_goose/Finland/6955_21VIR7689-9/2021	A / H5N1	Europe / Finland	Bianca Zecchin (Istituto Zooprofilattico Sperimentale Delle Venezie)
EPI_ISL_7778776	A/greylag_goose/Norway/V294_21VIR7634-1/2021	A / H5N8	Europe / Norway	Bianca Zecchin (Istituto Zooprofilattico Sperimentale Delle Venezie)
EPI_ISL_7778777	A/mute_swan/Norway/FU452_21VIR7634-2/2021	A / H5N8	Europe / Norway	Bianca Zecchin (Istituto Zooprofilattico Sperimentale Delle Venezie)
EPI_ISL_7778778	A/common_eider/Norway/FU453_21VIR7634-3/2021	A / H5N8	Europe / Norway	Bianca Zecchin (Istituto Zooprofilattico Sperimentale Delle Venezie)
EPI_ISL_7778779	A/common_eider/Norway/FU458_21VIR7634-4/2021	A / H5N8	Europe / Norway	Bianca Zecchin (Istituto Zooprofilattico Sperimentale Delle Venezie)
EPI_ISL_7778780	A/common_eider/Norway/FU474_21VIR7634-5/2021	A / H5N8	Europe / Norway	Bianca Zecchin (Istituto Zooprofilattico Sperimentale Delle Venezie)
EPI_ISL_7778880	A/Red_fox/Estonia/TA2126820_21VIR10433-13/2021	A / H5N1	Europe / Estonia	Bianca Zecchin (Istituto Zooprofilattico Sperimentale Delle Venezie)
EPI_ISL_779129	A/turkey/Poland/464/2020(H5N8)	A / H5N8	Europe / Poland / Masovian Voivodeship	Edyta ?wi?to? (National Veterinary Research Institut Poland, PIWet-PIB)
EPI_ISL_780096	A/Duck/Hungary/14788/2020	A / H5N8	Europe / Hungary / Bacs-Kiskun	Katalin Szentpáli-Gavallér (National Food Chain Safety Office, Hungary / Virology)
EPI_ISL_7880689	A/Gallus_gallus/Belgium/16070_003/2021	A / H5N1	Europe / Belgium / Provincie Antwerpen / Weelde (Ravels)	Steven Van Borm (Sciensano, Department of Animal Infectious Diseases / Animal Infectious Diseases)
EPI_ISL_7880696	A/Gallus_gallus/Belgium/15977/2021	A / H5N1	Europe / Belgium / Provincie West-Vlaanderen / Alveringem	Steven Van Borm (Sciensano, Department of Animal Infectious Diseases / Animal Infectious Diseases)
EPI_ISL_7892492	A/grey heron/Croatia/132/2021	A / H5N1	Europe / Croatia / Osjecko-Baranjska Zupanija / Kopacki rit (45.606587° 18.801463°)	Vladimir Savi? (Croatian Veterinary Institute / Poultry Centre)
EPI_ISL_7952115	A/mute swan/Netherlands/21039526-002/2021	A / H5N1	Europe / Netherlands / South Holland	Rene Heutink (Wageningen Bioveterinary Research)
EPI_ISL_7952116	A/chicken/Netherlands/21039901-001-005/2021	A / H5N1	Europe / Netherlands / South Holland	Rene Heutink (Wageningen Bioveterinary Research)
EPI_ISL_7952117	A/chicken/Netherlands/21040808-001-005/2021	A / H5N1	Europe / Netherlands / Provincie Overijssel	Rene Heutink (Wageningen Bioveterinary Research)
EPI_ISL_7952118	A/chicken/Netherlands/21040811-001-005/2021	A / H5N1	Europe / Netherlands / Provincie Overijssel	Rene Heutink (Wageningen Bioveterinary Research)
EPI_ISL_7952119	A/great black-backed gull/Netherlands/21039609-001-002/20	A / H5N1	Europe / Netherlands / Provincie Friesland	Rene Heutink (Wageningen Bioveterinary Research)
EPI_ISL_7952120	A/greylag goose/Netherlands/21039525-001/2021	A / H5N1	Europe / Netherlands / South Holland	Rene Heutink (Wageningen Bioveterinary Research)
EPI_ISL_7952121	A/sanderling/Netherlands/21039528-002/2021	A / H5N1	Europe / Netherlands / Provincie Friesland	Rene Heutink (Wageningen Bioveterinary Research)
EPI_ISL_7952122	A/greylag goose/Netherlands/21039746-002/2021	A / H5N1	Europe / Netherlands / South Holland	Rene Heutink (Wageningen Bioveterinary Research)
EPI_ISL_7952123	A/barnacle goose/Netherlands/21039750-002/2021	A / H5N1	Europe / Netherlands / Provincie Flevoland	Rene Heutink (Wageningen Bioveterinary Research)
EPI_ISL_7952124	A/mute swan/Netherlands/21039841-002/2021	A / H5N1	Europe / Netherlands / Provincie Utrecht	Rene Heutink (Wageningen Bioveterinary Research)
EPI_ISL_7952125	A/mute swan/Netherlands/21039824-002/2021	A / H5N1	Europe / Netherlands / Provincie Friesland	Rene Heutink (Wageningen Bioveterinary Research)
EPI_ISL_7952126	A/greylag goose/Netherlands/21038417-001/2021	A / H5N1	Europe / Netherlands / South Holland	Rene Heutink (Wageningen Bioveterinary Research)
EPI_ISL_7952127	A/goose/Netherlands/21039364-002/2021	A / H5N1	Europe / Netherlands / Provincie Friesland	Rene Heutink (Wageningen Bioveterinary Research)
EPI_ISL_7952128	A/gadwall/Netherlands/21038566-002/2021	A / H5N1	Europe / Netherlands / South Holland	Rene Heutink (Wageningen Bioveterinary Research)
EPI_ISL_7952129	A/western jackdaw/Netherlands/21039297-002/2021	A / H5N1	Europe / Netherlands / Provincie Flevoland	Rene Heutink (Wageningen Bioveterinary Research)
EPI_ISL_796011	A/Goose/Hungary/15267/2020	A / H5N8	Europe / Hungary / Bacs-Kiskun	Katalin Szentpáli-Gavallér (National Food Chain Safety Office, Hungary / Virology)
EPI_ISL_7983813	A/Hangzhou/01/2021	A / H5N6	Asia / China / Zhejiang Province / Hangzhou	Jun Li (Hangzhou Center for Disease Control and Prevention)
EPI_ISL_7996368	A/mute swan/Netherlands/21038568-005/2021	A / H5N1	Europe / Netherlands / Provincie Friesland	Rene Heutink (Wageningen Bioveterinary Research)

EPI_ISL_7996369	A/mute swan/Netherlands/21039291-002/2021	A / H5N1	Europe / Netherlands / South Holland	Rene Heutink (Wageningen Bioveterinary Research)
EPI_ISL_7996370	A/western jackdaw/Netherlands/21039294-002/2021	A / H5N1	Europe / Netherlands / Provincie Flevoland	Rene Heutink (Wageningen Bioveterinary Research)
EPI_ISL_7996371	A/turkey/Netherlands/21040980-001005/2021	A / H5N1	Europe / Netherlands / Provincie Limburg	Rene Heutink (Wageningen Bioveterinary Research)
EPI_ISL_804043	A/Mallard_duck/Hungary/17319/2020	A / H5N8	Europe / Hungary / Bacs-Kiskun	Katalin Szentpáli-Gavallér (National Food Chain Safety Office, Hungary / Virology)
EPI_ISL_804045	A/Duck/Hungary/17806/2020	A / H5N8	Europe / Hungary / Bacs-Kiskun	Katalin Szentpáli-Gavallér (National Food Chain Safety Office, Hungary / Virology)
EPI_ISL_804047	A/Duck/Hungary/17957/2020	A / H5N8	Europe / Hungary / Csongrad megye	Katalin Szentpáli-Gavallér (National Food Chain Safety Office, Hungary / Virology)
EPI_ISL_808250	A/Duck/Hungary/18358/2020	A / H5N8	Europe / Hungary / Bacs-Kiskun	Katalin Szentpáli-Gavallér (National Food Chain Safety Office, Hungary / Virology)
EPI_ISL_809658	A/Goose/Hungary/18406/2020	A / H5N8	Europe / Hungary / Bacs-Kiskun	Katalin Szentpáli-Gavallér (National Food Chain Safety Office, Hungary / Virology)
EPI_ISL_810963	A/Duck/Hungary/18444/2020	A / H5N8	Europe / Hungary / Bacs-Kiskun	Katalin Szentpáli-Gavallér (National Food Chain Safety Office, Hungary / Virology)
EPI_ISL_810970	A/Chicken/Hungary/18466/2020	A / H5N8	Europe / Hungary / Bacs-Kiskun	Katalin Szentpáli-Gavallér (National Food Chain Safety Office, Hungary / Virology)
EPI_ISL_811128	A/Chicken/Hungary/18467/2020	A / H5N8	Europe / Hungary / Bacs-Kiskun	Katalin Szentpáli-Gavallér (National Food Chain Safety Office, Hungary / Virology)
EPI_ISL_811134	A/Chicken/Hungary/19776/2020	A / H5N8	Europe / Hungary / Csongrad megye	Katalin Szentpáli-Gavallér (National Food Chain Safety Office, Hungary / Virology)
EPI_ISL_811135	A/Chicken/Hungary/20227/2020	A / H5N8	Europe / Hungary / Csongrad megye	Katalin Szentpáli-Gavallér (National Food Chain Safety Office, Hungary / Virology)
EPI_ISL_811144	A/Goose/Hungary/21737/2020	A / H5N8	Europe / Hungary / Bekes megye	Katalin Szentpáli-Gavallér (National Food Chain Safety Office, Hungary / Virology)
EPI_ISL_811146	A/Turkey/Hungary/21753/2020	A / H5N8	Europe / Hungary / Csongrad megye	Katalin Szentpáli-Gavallér (National Food Chain Safety Office, Hungary / Virology)
EPI_ISL_813599	A/Goose/Hungary/22493/2020	A / H5N8	Europe / Hungary / Bacs-Kiskun	Katalin Szentpáli-Gavallér (National Food Chain Safety Office, Hungary / Virology)
EPI_ISL_813971	A/Turkey/Hungary/22494/2020	A / H5N8	Europe / Hungary / Bekes megye	Katalin Szentpáli-Gavallér (National Food Chain Safety Office, Hungary / Virology)
EPI_ISL_813972	A/Goose/Hungary/24021/2020	A / H5N8	Europe / Hungary / Bekes megye	Katalin Szentpáli-Gavallér (National Food Chain Safety Office, Hungary / Virology)
EPI_ISL_813979	A/peregrine_falcon/Ireland/20VIR7872-1/2020	A / H5N8	Europe / Ireland	Bianca Zecchin (Istituto Zooprofilattico Sperimentale Delle Venezie)
EPI_ISL_8215653	A/Bar-headed Goose/Tibet/XZQ5-1/2021	A / H5N8	Asia / China / Tibet Autonomous Region	Hongliang Chai (Northeast Forestry University / College of Wildlife Resources)
EPI_ISL_8215654	A/Bar-headed Goose/Tibet/XZQ7/2021	A / H5N8	Asia / China / Tibet Autonomous Region	Hongliang Chai (Northeast Forestry University / College of Wildlife Resources)
EPI_ISL_8215655	A/Bar-headed Goose/Tibet/XZQ8-1/2021	A / H5N8	Asia / China / Tibet Autonomous Region	Hongliang Chai (Northeast Forestry University / College of Wildlife Resources)
EPI_ISL_8215656	A/Bar-headed Goose/Tibet/XZQ9-1/2021	A / H5N8	Asia / China / Tibet Autonomous Region	Hongliang Chai (Northeast Forestry University / College of Wildlife Resources)
EPI_ISL_8215657	A/Bar-headed Goose/Tibet/XZQ10-1/2021	A / H5N8	Asia / China / Tibet Autonomous Region	Hongliang Chai (Northeast Forestry University / College of Wildlife Resources)
EPI_ISL_8215658	A/Bar-headed Goose/Tibet/XZ6/2021	A / H5N8	Asia / China / Tibet Autonomous Region	Hongliang Chai (Northeast Forestry University / College of Wildlife Resources)
EPI_ISL_8215659	A/Bar-headed Goose/Tibet/XZ71/2021	A / H5N8	Asia / China / Tibet Autonomous Region	Hongliang Chai (Northeast Forestry University / College of Wildlife Resources)
EPI_ISL_8215660	A/Bar-headed Goose/Tibet/XZ81/2021	A / H5N8	Asia / China / Tibet Autonomous Region	Hongliang Chai (Northeast Forestry University / College of Wildlife Resources)
EPI_ISL_8215661	A/Bar-headed Goose/Tibet/XZ181/2021	A / H5N8	Asia / China / Tibet Autonomous Region	Hongliang Chai (Northeast Forestry University / College of Wildlife Resources)
EPI_ISL_8215662	A/Bar-headed Goose/Tibet/XZQ13-1/2021	A / H5N8	Asia / China / Tibet Autonomous Region	Hongliang Chai (Northeast Forestry University / College of Wildlife Resources)
EPI_ISL_8215663	A/Brown-headed Gull/Tibet/XZQ15-2/2021	A / H5N8	Asia / China / Tibet Autonomous Region	Hongliang Chai (Northeast Forestry University / College of Wildlife Resources)
EPI_ISL_8215684	A/Brown-headed Gull/Tibet/XZQ16-2/2021	A / H5N8	Asia / China / Tibet Autonomous Region	Hongliang Chai (Northeast Forestry University / College of Wildlife Resources)
EPI_ISL_8215685	A/Bar-headed Goose/Tibet/XZQ17-1/2021	A / H5N8	Asia / China / Tibet Autonomous Region	Hongliang Chai (Northeast Forestry University / College of Wildlife Resources)
EPI_ISL_8215686	A/Bar-headed Goose/Tibet/XZQ18-1/2021	A / H5N8	Asia / China / Tibet Autonomous Region	Hongliang Chai (Northeast Forestry University / College of Wildlife Resources)
EPI_ISL_8215687	A/Brown-headed Gull/Tibet/XZ19/2021	A / H5N8	Asia / China / Tibet Autonomous Region	Hongliang Chai (Northeast Forestry University / College of Wildlife Resources)
EPI_ISL_8215688	A/Bar-headed Goose/Tibet/XZ901/2021	A / H5N1	Asia / China / Tibet Autonomous Region	Hongliang Chai (Northeast Forestry University / College of Wildlife Resources)
EPI_ISL_8215689	A/Bar-headed Goose/Tibet/XZ1131/2021	A / H5N1	Asia / China / Tibet Autonomous Region	Hongliang Chai (Northeast Forestry University / College of Wildlife Resources)
EPI_ISL_826283	A/Chicken/Sweden/SVA201221SZ0027/KN342632-IP2/2020	A / H5N8	Europe / Sweden / Skane Lan / Svedala Kommun	Siamak Zohari (National Veterinary Institute)
EPI_ISL_826285	A/barnacle goose/Sweden/SVA201215SZ0368/KN003768/202(	A / H5N8	Europe / Sweden / Skane Lan / Malmo Kommun	Siamak Zohari (National Veterinary Institute)
EPI_ISL_826296	A/barnacle goose/Sweden/SVA201221SZ0154/KN003828/202(	A / H5N8	Europe / Sweden / Skane Lan / Skurups Kommun	Siamak Zohari (National Veterinary Institute)
EPI_ISL_826457	A/Chicken/Sweden/SVA210102SZ0002/KN000002-IP1/2021	A / H5N8	Europe / Sweden / Skane Lan / Sjobo Kommun	Siamak Zohari (National Veterinary Institute)
EPI_ISL_832205	A/Chicken/Sweden/SVA210111SZ0065/KN005298-IP1/2021	A / H5N8	Europe / Sweden / Skane Lan / Sjobo Kommun	Siamak Zohari (National Veterinary Institute)
EPI_ISL_832206	A/Turkey/Sweden/SVA210114SZ0001/KN009555-IP2/2021	A / H5N8	Europe / Sweden / Skane Lan / Skurups Kommun	Siamak Zohari (National Veterinary Institute)
EPI_ISL_833248	A/Muscovy duck/China/FJFZ21/H5N6/2020	A / H5N6	Asia / China / Fujian Province	Rui Zhang (Fujian Agriculture and Forestry University)
EPI_ISL_833482	A/Goose/Hungary/18325/2020	A / H5N8	Europe / Hungary / Bacs-Kiskun	Katalin Szentpáli-Gavallér (National Food Chain Safety Office, Hungary / Virology)
EPI_ISL_833485	A/Mallard_duck/Hungary/18410/2020	A / H5N8	Europe / Hungary / Csongrad megye	Katalin Szentpáli-Gavallér (National Food Chain Safety Office, Hungary / Virology)
EPI_ISL_833487	A/Pheasant/Hungary/18731/2020	A / H5N8	Europe / Hungary / Bacs-Kiskun	Katalin Szentpáli-Gavallér (National Food Chain Safety Office, Hungary / Virology)
EPI_ISL_833488	A/Turkey/Hungary/19338/2020	A / H5N8	Europe / Hungary / Bacs-Kiskun	Katalin Szentpáli-Gavallér (National Food Chain Safety Office, Hungary / Virology)
EPI_ISL_833579	A/Duck/Hungary/18414/2020	A / H5N8	Europe / Hungary / Csongrad megye	Katalin Szentpáli-Gavallér (National Food Chain Safety Office, Hungary / Virology)
EPI_ISL_833580	A/Turkey/Hungary/19394/2020	A / H5N8	Europe / Hungary / Bacs-Kiskun	Katalin Szentpáli-Gavallér (National Food Chain Safety Office, Hungary / Virology)
EPI_ISL_833581	A/Goose/Hungary/19118/2020	A / H5N8	Europe / Hungary / Bacs-Kiskun	Katalin Szentpáli-Gavallér (National Food Chain Safety Office, Hungary / Virology)
EPI_ISL_833582	A/Goose/Hungary/19953/2020	A / H5N8	Europe / Hungary / Bacs-Kiskun	Katalin Szentpáli-Gavallér (National Food Chain Safety Office, Hungary / Virology)
EPI_ISL_833583	A/Goose/Hungary/19959/2020	A / H5N8	Europe / Hungary / Csongrad megye	Katalin Szentpáli-Gavallér (National Food Chain Safety Office, Hungary / Virology)
EPI_ISL_8338002	A/Turkey/Sweden/SVA211212SZ0001/FB301013-IP-2/M-2021	A / H5N1	Europe / Sweden / Skane Lan / Skurups Kommun	Siamak Zohari (National Veterinary Institute)
EPI_ISL_8338456	A/mute swan/Croatia/144/2021	A / H5N1	Europe / Croatia / Istarska Zupanija / the mouth of the river Mirna (45° 18´ 55?N, 13° 35´ 28?E)	Vladimir Savi? (Croatian Veterinary Institute / Poultry Centre)
EPI_ISL_8338502	A/mute swan/Croatia/145/2021	A / H5N1	Europe / Croatia / Zagrebacka Zupanija / Rugvica - Siromaja lakes (45° 45´ 54?N, 16° 10´ 11?E)	Vladimir Savi? (Croatian Veterinary Institute / Poultry Centre)
EPI_ISL_8377056	A/pelican/France/21P013720/2021	A / H5N1	Europe / France / Languedoc-Roussillon / Departement de l'Aude	Francois-Xavier Briand (ANSES Agence Nationale De Securite Sanitaire De L'alimentation / Laboratoire de Ploufragan-Plouzané)
EPI_ISL_8377254	A/egret/France/21P013418/2021	A / H5N1	Europe / France / Rhone-Alpes / Departement de l'Ain	Francois-Xavier Briand (ANSES Agence Nationale De Securite Sanitaire De L'alimentation / Laboratoire de Ploufragan-Plouzané)
EPI_ISL_8377417	A/chicken/France/21P013076/2021	A / H5N1	Europe / France / Region Nord-Pas-De Calais / Departement du Nord	Francois-Xavier Briand (ANSES Agence Nationale De Securite Sanitaire De L'alimentation / Laboratoire de Ploufragan-Plouzané)
EPI_ISL_8377418	A/goose/France/21P013228/2021	A / H5N1	Europe / France / Brittany / Departement du Morbihan	Francois-Xavier Briand (ANSES Agence Nationale De Securite Sanitaire De L'alimentation / Laboratoire de Ploufragan-Plouzané)
EPI_ISL_8416806	A/turkey/Lisbon/1/2021	A / H5N1	Europe / Portugal / Distrito de Setubal	Aryse Martins Melo (Instituto Nacional de Saúde Dr. Ricardo Jorge)
EPI_ISL_8439476	A/mute swan/Croatia/146/2021	A / H5N1	Europe / Croatia / Vukovarsko-Srijemska Zupanija / Lipovac (45.06517, 19.07613)	Vladimir Savi? (Croatian Veterinary Institute / Poultry Centre)
EPI_ISL_8440175	A/Cygnus_olor/Romania/16381_21VIR10306/2021	A / H5N1	Europe / Romania	Bianca Zecchin (Istituto Zooprofilattico Sperimentale Delle Venezie)
EPI_ISL_8440976	A/mute_swan/Romania/16790_21VIR11355/2021	A / H5N1	Europe / Romania	Bianca Zecchin (Istituto Zooprofilattico Sperimentale Delle Venezie)
EPI_ISL_846583	A/Chicken/Hungary/21379/2020	A / H5N8	Europe / Hungary / Bacs-Kiskun	Katalin Szentpáli-Gavallér (National Food Chain Safety Office, Hungary / Virology)
EPI_ISL_846584	A/Goose/Hungary/19128/2020	A / H5N8	Europe / Hungary / Bacs-Kiskun	Katalin Szentpáli-Gavallér (National Food Chain Safety Office, Hungary / Virology)
EPI_ISL_846585	A/Chicken/Hungary/24596/2020	A / H5N8	Europe / Hungary / Bacs-Kiskun	Katalin Szentpáli-Gavallér (National Food Chain Safety Office, Hungary / Virology)
EPI_ISL_846586	A/Chicken/Hungary/29723/2020	A / H5N8	Europe / Hungary / Bacs-Kiskun	Katalin Szentpáli-Gavallér (National Food Chain Safety Office, Hungary / Virology)
EPI_ISL_846601	A/chicken/Poland/474/2020(H5N8)	A / H5N8	Europe / Poland / pomorskie	Edyta ?wi?to? (National Veterinary Research Institut Poland, PIWet-PIB)
EPI_ISL_846602	A/turkey/Poland/475/2020(H5N8)	A / H5N8	Europe / Poland / wielkopolskie	Edyta ?wi?to? (National Veterinary Research Institut Poland, PIWet-PIB)
EPI_ISL_846603	A/chicken/Poland/476/2020(H5N8)	A / H5N8	Europe / Poland / wielkopolskie	Edyta ?wi?to? (National Veterinary Research Institut Poland, PIWet-PIB)
EPI_ISL_846604	A/turkey/Poland/477/2020(H5N8)	A / H5N8	Europe / Poland / Masovian Voivodeship / mazowieckie	Edyta ?wi?to? (National Veterinary Research Institut Poland, PIWet-PIB)
EPI_ISL_846614	A/turkey/Poland/542/2020(H5N8)	A / H5N8	Europe / Poland / warminsko-mazurskie	Edyta ?wi?to? (National Veterinary Research Institut Poland, PIWet-PIB)

EPI_ISL_846617	A/chicken/Poland/565/2020(H5N8)	A / H5N8	Europe / Poland / dolnoslaskie	Edyta ?wi?to? (National Veterinary Research Institut Poland, PIWet-PIB)
EPI_ISL_846623	A/swan/Poland/MB141/2020(H5N8)	A / H5N8	Europe / Poland / Pomeranian Voivodeship / pomorskie	Edyta ?wi?to? (National Veterinary Research Institut Poland, PIWet-PIB)
EPI_ISL_846624	A/wild_goose/Poland/MB142/2020(H5N8)	A / H5N8	Europe / Poland / pomorskie	Edyta ?wi?to? (National Veterinary Research Institut Poland, PIWet-PIB)
EPI_ISL_846625	A/tundra_bean_goose/Poland/MB128/2020(H5N8)	A / H5N8	Europe / Poland / wielkopolskie	Edyta ?wi?to? (National Veterinary Research Institut Poland, PIWet-PIB)
EPI_ISL_8515478	A/goose/Czech_Republic/22608-1/2021	A / H5N1	Europe / Czech Republic / Jihocesky Kraj / Okres Ceske Budejovice / Nové Hrady, By?ov, ZipCode	Alexander Nagy (State Veterinary Institute Prague)
EPI_ISL_8515479	A/goose/Czech_Republic/22608-2/2021	A / H5N1	Europe / Czech Republic / Jihocesky Kraj / Okres Ceske Budejovice / Nové Hrady, By?ov, ZipCode	Alexander Nagy (State Veterinary Institute Prague)
EPI_ISL_8515480	A/chicken/Czech_Republic/23404/2021	A / H5N1	Europe / Czech Republic / South Moravian Region / Okres Breclav / Lednice, ZipCode: 69103	Alexander Nagy (State Veterinary Institute Prague)
EPI_ISL_8515481	A/grey heron/Czech_Republic/23608/2021	A / H5N1	Europe / Czech Republic / Liberecky Kraj / Okres Ceska Lipa / He?mani?ky u Dobranova, GPS:50.[illegible]	Alexander Nagy (State Veterinary Institute Prague)
EPI_ISL_8515482	A/grey heron/Czech_Republic/23608-1K/2021	A / H5N1	Europe / Czech Republic / Liberecky Kraj / Okres Ceska Lipa / He?mani?ky u Dobranova, GPS: 50.	Alexander Nagy (State Veterinary Institute Prague)
EPI_ISL_8515483	A/great egret/Czech_Republic/23609/2021	A / H5N1	Europe / Czech Republic / Liberecky Kraj / Okres Ceska Lipa / He?mani?ky u Dobranova, GPS:50.[illegible]	Alexander Nagy (State Veterinary Institute Prague)
EPI_ISL_8517996	A/Environment/Hangzhou/E1147/2021	A / H5N6	Asia / China / Zhejiang	Jun Li (Hangzhou Center for Disease Control and Prevention)
EPI_ISL_8517997	A/Environment/Hangzhou/E1150/2021	A / H5N6	Asia / China / Zhejiang	Jun Li (Hangzhou Center for Disease Control and Prevention)
EPI_ISL_8517998	A/Environment/Hangzhou/E1151/2021	A / H5N6	Asia / China / Zhejiang	Jun Li (Hangzhou Center for Disease Control and Prevention)
EPI_ISL_8517999	A/Environment/Hangzhou/E1153/2021	A / H5N6	Asia / China / Zhejiang	Jun Li (Hangzhou Center for Disease Control and Prevention)
EPI_ISL_8518000	A/Environment/Hangzhou/E1154/2021	A / H5N6	Asia / China / Zhejiang	Jun Li (Hangzhou Center for Disease Control and Prevention)
EPI_ISL_8518268	A/Chicken/Hangzhou/E1149/2021	A / H5N6	Asia / China / Zhejiang	Jun Li (Hangzhou Center for Disease Control and Prevention)
EPI_ISL_854595	A/chicken/Chiba/B2T/2021	A / H5N8	Asia / Japan / Chiba	Takehiko Saito (National Institute of Animal Health)
EPI_ISL_854596	A/chicken/Chiba/B3T/2021	A / H5N8	Asia / Japan / Chiba	Takehiko Saito (National Institute of Animal Health)
EPI_ISL_854597	A/chicken/Chiba/B4T/2021	A / H5N8	Asia / Japan / Chiba	Takehiko Saito (National Institute of Animal Health)
EPI_ISL_854598	A/chicken/Chiba/B5T/2021	A / H5N8	Asia / Japan / Chiba	Takehiko Saito (National Institute of Animal Health)
EPI_ISL_854599	A/chicken/Gifu/10T/2021	A / H5N8	Asia / Japan / Gifu	Takehiko Saito (National Institute of Animal Health)
EPI_ISL_854600	A/chicken/Gifu/11T/2021	A / H5N8	Asia / Japan / Gifu	Takehiko Saito (National Institute of Animal Health)
EPI_ISL_854601	A/chicken/Gifu/2T/2021	A / H5N8	Asia / Japan / Gifu	Takehiko Saito (National Institute of Animal Health)
EPI_ISL_854602	A/chicken/Gifu/3T/2021	A / H5N8	Asia / Japan / Gifu	Takehiko Saito (National Institute of Animal Health)
EPI_ISL_854603	A/chicken/Gifu/6T/2021	A / H5N8	Asia / Japan / Gifu	Takehiko Saito (National Institute of Animal Health)
EPI_ISL_854604	A/chicken/Gifu/7T/2021	A / H5N8	Asia / Japan / Gifu	Takehiko Saito (National Institute of Animal Health)
EPI_ISL_854605	A/chicken/Kagoshima/1T/2021	A / H5N8	Asia / Japan / Kagoshima	Takehiko Saito (National Institute of Animal Health)
EPI_ISL_854606	A/chicken/Kagoshima/3T/2021	A / H5N8	Asia / Japan / Kagoshima	Takehiko Saito (National Institute of Animal Health)
EPI_ISL_854607	A/chicken/Kagoshima/5T/2021	A / H5N8	Asia / Japan / Kagoshima	Takehiko Saito (National Institute of Animal Health)
EPI_ISL_854608	A/chicken/Kagoshima/6T/2021	A / H5N8	Asia / Japan / Kagoshima	Takehiko Saito (National Institute of Animal Health)
EPI_ISL_854609	A/chicken/Miyazaki/I11T/2020	A / H5N8	Asia / Japan / Miyazaki	Takehiko Saito (National Institute of Animal Health)
EPI_ISL_854610	A/chicken/Miyazaki/I12T/2020	A / H5N8	Asia / Japan / Miyazaki	Takehiko Saito (National Institute of Animal Health)
EPI_ISL_854611	A/chicken/Miyazaki/I5T/2020	A / H5N8	Asia / Japan / Miyazaki	Takehiko Saito (National Institute of Animal Health)
EPI_ISL_854612	A/chicken/Miyazaki/I9T/2020	A / H5N8	Asia / Japan / Miyazaki	Takehiko Saito (National Institute of Animal Health)
EPI_ISL_8568483	A/gadwall/Croatia/108/2021	A / H5N1	Europe / Croatia / Splitsko-Dalmatinska Zupanija / Zaostrog, Podaca cove	Vladimir Savi? (Croatian Veterinary Institute / Poultry Centre)
EPI_ISL_8650947	A/red fox/Netherlands/22000097-004/2022	A / H5N1	Europe / Netherlands / Provincie Noord-Holland	Rene Heutink (Wageningen Bioveterinary Research)
EPI_ISL_8650949	A/red knot/Netherlands/21041030-008/2021	A / H5N1	Europe / Netherlands / Provincie Friesland	Rene Heutink (Wageningen Bioveterinary Research)
EPI_ISL_8694562	A/mute swan/Croatia/6/2022	A / H5N1	Europe / Croatia / Karlovacka Zupanija / Karlovac	Vladimir Savi? (Croatian Veterinary Institute / Poultry Centre)
EPI_ISL_8694715	A/chicken/Croatia/7/2022	A / H5N1	Europe / Croatia / Osjecko-Baranjska Zupanija / Branjin vrh	Vladimir Savi? (Croatian Veterinary Institute / Poultry Centre)
EPI_ISL_8768942	A/turkey/Tyumen/15-1V/2021	A / H5N1	Europe / Russian Federation / Tyumen Oblast / Tyumen	Natalia Goncharova (State Research Center of Virology and Biotechnology (VECTOR) / Emerging Zoonotic Diseases and Influenza)
EPI_ISL_8768944	A/turkey/Tyumen/15-2V/2021	A / H5N1	Europe / Russian Federation / Tyumen Oblast / Tyumen	Natalia Goncharova (State Research Center of Virology and Biotechnology (VECTOR) / Emerging Zoonotic Diseases and Influenza)
EPI_ISL_8768947	A/turkey/Tyumen/15-3V/2021	A / H5N1	Europe / Russian Federation / Tyumen Oblast / Tyumen	Natalia Goncharova (State Research Center of Virology and Biotechnology (VECTOR) / Emerging Zoonotic Diseases and Influenza)
EPI_ISL_8768948	A/turkey/Tyumen/15-4V/2021	A / H5N1	Europe / Russian Federation / Tyumen Oblast / Tyumen	Natalia Goncharova (State Research Center of Virology and Biotechnology (VECTOR) / Emerging Zoonotic Diseases and Influenza)
EPI_ISL_8768949	A/turkey/Tyumen/15-6V/2021	A / H5N1	Europe / Russian Federation / Tyumen Oblast / Tyumen	Natalia Goncharova (State Research Center of Virology and Biotechnology (VECTOR) / Emerging Zoonotic Diseases and Influenza)
EPI_ISL_8768950	A/turkey/Tyumen/15-9V/2021	A / H5N1	Europe / Russian Federation / Tyumen Oblast / Tyumen	Natalia Goncharova (State Research Center of Virology and Biotechnology (VECTOR) / Emerging Zoonotic Diseases and Influenza)
EPI_ISL_8768953	A/turkey/Tyumen/15-14V/2021	A / H5N1	Europe / Russian Federation / Tyumen Oblast / Tyumen	Natalia Goncharova (State Research Center of Virology and Biotechnology (VECTOR) / Emerging Zoonotic Diseases and Influenza)
EPI_ISL_8768954	A/duck/Omsk/19-1V/2021	A / H5N1	Europe / Russian Federation / Omsk Oblast / Omsk	Natalia Goncharova (State Research Center of Virology and Biotechnology (VECTOR) / Emerging Zoonotic Diseases and Influenza)
EPI_ISL_8768955	A/duck/Omsk/19-2V/2021	A / H5N1	Europe / Russian Federation / Omsk Oblast / Omsk	Natalia Goncharova (State Research Center of Virology and Biotechnology (VECTOR) / Emerging Zoonotic Diseases and Influenza)
EPI_ISL_8768965	A/duck/Omsk/19-3V/2021	A / H5N1	Europe / Russian Federation / Omsk Oblast / Omsk	Natalia Goncharova (State Research Center of Virology and Biotechnology (VECTOR) / Emerging Zoonotic Diseases and Influenza)
EPI_ISL_8768966	A/duck/Omsk/19-5V/2021	A / H5N1	Europe / Russian Federation / Omsk Oblast / Omsk	Natalia Goncharova (State Research Center of Virology and Biotechnology (VECTOR) / Emerging Zoonotic Diseases and Influenza)
EPI_ISL_8768986	A/goose/Rostov-on-Don/28-1V/2021	A / H5N1	Europe / Russian Federation / Rostov Oblast / Rostov-on-Don	Natalia Goncharova (State Research Center of Virology and Biotechnology (VECTOR) / Emerging Zoonotic Diseases and Influenza)
EPI_ISL_8768989	A/duck/Saratov/29-2V/2021	A / H5N1	Europe / Russian Federation / Saratov Oblast / Saratov	Natalia Goncharova (State Research Center of Virology and Biotechnology (VECTOR) / Emerging Zoonotic Diseases and Influenza)
EPI_ISL_8769004	A/chicken/Saratov/29-6V/2021	A / H5N1	Europe / Russian Federation / Saratov Oblast / Saratov	Natalia Goncharova (State Research Center of Virology and Biotechnology (VECTOR) / Emerging Zoonotic Diseases and Influenza)
EPI_ISL_8769010	A/goose/Chelyabinsk/34-1V/2021	A / H5N1	Europe / Russian Federation / Chelyabinsk Oblast / Chelyabinsk	Natalia Goncharova (State Research Center of Virology and Biotechnology (VECTOR) / Emerging Zoonotic Diseases and Influenza)
EPI_ISL_8769012	A/chicken/Orenburg/46-1V/2021	A / H5N1	Europe / Russian Federation / Orenburg Oblast / Orenburg	Natalia Goncharova (State Research Center of Virology and Biotechnology (VECTOR) / Emerging Zoonotic Diseases and Influenza)
EPI_ISL_8769014	A/chicken/Orenburg/46-2V/2021	A / H5N1	Europe / Russian Federation / Orenburg Oblast / Orenburg	Natalia Goncharova (State Research Center of Virology and Biotechnology (VECTOR) / Emerging Zoonotic Diseases and Influenza)
EPI_ISL_8769016	A/goose/Orenburg/46-3V/2021	A / H5N1	Europe / Russian Federation / Orenburg Oblast / Orenburg	Natalia Goncharova (State Research Center of Virology and Biotechnology (VECTOR) / Emerging Zoonotic Diseases and Influenza)
EPI_ISL_8769017	A/chicken/Tyumen/47-66V/2021	A / H5N1	Europe / Russian Federation / Tyumen Oblast / Tyumen	Natalia Goncharova (State Research Center of Virology and Biotechnology (VECTOR) / Emerging Zoonotic Diseases and Influenza)
EPI_ISL_8769018	A/chicken/Tyumen/47-79V/2021	A / H5N1	Europe / Russian Federation / Tyumen Oblast / Tyumen	Natalia Goncharova (State Research Center of Virology and Biotechnology (VECTOR) / Emerging Zoonotic Diseases and Influenza)
EPI_ISL_8769019	A/chicken/Tyumen/47-85V/2021	A / H5N1	Europe / Russian Federation / Tyumen Oblast / Tyumen	Natalia Goncharova (State Research Center of Virology and Biotechnology (VECTOR) / Emerging Zoonotic Diseases and Influenza)
EPI_ISL_8769021	A/chicken/Tyumen/47-88V/2021	A / H5N1	Europe / Russian Federation / Tyumen Oblast / Tyumen	Natalia Goncharova (State Research Center of Virology and Biotechnology (VECTOR) / Emerging Zoonotic Diseases and Influenza)
EPI_ISL_8769022	A/chicken/Tyumen/47-95V/2021	A / H5N1	Europe / Russian Federation / Tyumen Oblast / Tyumen	Natalia Goncharova (State Research Center of Virology and Biotechnology (VECTOR) / Emerging Zoonotic Diseases and Influenza)
EPI_ISL_8769023	A/chicken/Kirov/63-1V/2021	A / H5N1	Europe / Russian Federation / Kirov Oblast / Kirov	Natalia Goncharova (State Research Center of Virology and Biotechnology (VECTOR) / Emerging Zoonotic Diseases and Influenza)
EPI_ISL_8769024	A/chicken/Kirov/63-2V/2021	A / H5N1	Europe / Russian Federation / Kirov Oblast / Kirov	Natalia Goncharova (State Research Center of Virology and Biotechnology (VECTOR) / Emerging Zoonotic Diseases and Influenza)
EPI_ISL_8769025	A/chicken/Kirov/63-5V/2021	A / H5N1	Europe / Russian Federation / Kirov Oblast / Kirov	Natalia Goncharova (State Research Center of Virology and Biotechnology (VECTOR) / Emerging Zoonotic Diseases and Influenza)
EPI_ISL_8769026	A/chicken/Kurgan/72-1V/2021	A / H5N1	Europe / Russian Federation / Kurgan Oblast / Kurgan	Natalia Goncharova (State Research Center of Virology and Biotechnology (VECTOR) / Emerging Zoonotic Diseases and Influenza)
EPI_ISL_8769028	A/chicken/Orenburg/73-5V/2021	A / H5N1	Europe / Russian Federation / Orenburg Oblast / Orenburg	Natalia Goncharova (State Research Center of Virology and Biotechnology (VECTOR) / Emerging Zoonotic Diseases and Influenza)
EPI_ISL_8769029	A/chicken/Orenburg/73-6V/2021	A / H5N1	Europe / Russian Federation / Orenburg Oblast / Orenburg	Natalia Goncharova (State Research Center of Virology and Biotechnology (VECTOR) / Emerging Zoonotic Diseases and Influenza)
EPI_ISL_8769030	A/chicken/Orenburg/73-7V/2021	A / H5N1	Europe / Russian Federation / Orenburg Oblast / Orenburg	Natalia Goncharova (State Research Center of Virology and Biotechnology (VECTOR) / Emerging Zoonotic Diseases and Influenza)
EPI_ISL_8769031	A/black-necked grebe/Kalmykia/78-1V/2021	A / H5N5	Europe / Russian Federation / Republic of Kalmykia	Natalia Goncharova (State Research Center of Virology and Biotechnology (VECTOR) / Emerging Zoonotic Diseases and Influenza)
EPI_ISL_8769033	A/turkey/Tyumen/81-96V/2021	A / H5N1	Europe / Russian Federation / Tyumen Oblast / Tyumen	Natalia Goncharova (State Research Center of Virology and Biotechnology (VECTOR) / Emerging Zoonotic Diseases and Influenza)
EPI_ISL_8769034	A/chicken/Tyumen/81-97V/2021	A / H5N1	Europe / Russian Federation / Tyumen Oblast / Tyumen	Natalia Goncharova (State Research Center of Virology and Biotechnology (VECTOR) / Emerging Zoonotic Diseases and Influenza)

EPI_ISL_8769036	A/chicken/Saratov/102-12V/2021	A / H5N1	Europe / Russian Federation / Saratov Oblast / Saratov	Natalia Goncharova (State Research Center of Virology and Biotechnology (VECTOR) / Emerging Zoonotic Diseases and Influenza)
EPI_ISL_8769038	A/chicken/Saratov/102-15V/2021	A / H5N1	Europe / Russian Federation / Saratov Oblast / Saratov	Natalia Goncharova (State Research Center of Virology and Biotechnology (VECTOR) / Emerging Zoonotic Diseases and Influenza)
EPI_ISL_8799314	A/red knot/Netherlands/21040956-003/2021	A / H5N1	Europe / Netherlands / Provincie Groningen	Rene Heutink (Wageningen Bioveterinary Research)
EPI_ISL_8799317	A/seagull/Netherlands/21041370-001/2021	A / H5N1	Europe / Netherlands / Provincie Gelderland	Rene Heutink (Wageningen Bioveterinary Research)
EPI_ISL_8799318	A/common buzzard/Netherlands/21041449-002/2021	A / H5N1	Europe / Netherlands / Provincie Zeeland	Rene Heutink (Wageningen Bioveterinary Research)
EPI_ISL_8799320	A/polecat/Netherlands/22000516-003/2022	A / H5N1	Europe / Netherlands / South Holland	Rene Heutink (Wageningen Bioveterinary Research)
EPI_ISL_8799552	A/England/215201407/2021	A / H5N1	Europe / United Kingdom	Steven Platt (UK Health Security Agency (UKHSA))
EPI_ISL_8809153	A/Muscovy duck/England/074477/2021	A / H5N1	Europe / United Kingdom / Devon	Alex Byrne (Animal and Plant Health Agency (APHA) / Virology Department)
EPI_ISL_8814146	A/turkey/England/055251/2021	A / H5N1	Europe / United Kingdom / Warwickshire / England	Alex Byrne (Animal and Plant Health Agency (APHA) / Virology Department)
EPI_ISL_8814195	A/turkey/England/056764/2021	A / H5N1	Europe / United Kingdom / Lancashire / England	Alex Byrne (Animal and Plant Health Agency (APHA) / Virology Department)
EPI_ISL_885144	A/environment/Hong Kong/WCRB-01/2018	A / H5N6	Asia / Hong Kong (SAR)	Huyen Trang Bui (The University of Hong Kong)
EPI_ISL_885145	A/Spoonbill/HK/17-18259/2017	A / H5N6	Asia / Hong Kong (SAR) / Mudflat of HK Wetland Park	Huyen Trang Bui (The University of Hong Kong)
EPI_ISL_885147	A/Northern Pintail/HK/MP692/2016	A / H5N6	Asia / Hong Kong (SAR) / Mai Po Tower Hide	Huyen Trang Bui (The University of Hong Kong)
EPI_ISL_885148	A/Chicken/Egypt/F1366A/2017	A / H5N8	Africa / Egypt	Huyen Trang Bui (The University of Hong Kong)
EPI_ISL_885149	A/Grey-Headed Gull/Uganda/200144/2017	A / H5N8	Africa / Uganda	Huyen Trang Bui (The University of Hong Kong)
EPI_ISL_8882166	A/turkey/Italy/21VIR11586-2/2021	A / H5N1	Europe / Italy	Giacomo Barbierato (Istituto Zooprofilattico Sperimentale delle Venezie / Ricerca e innovazione)
EPI_ISL_8882167	A/turkey/Italy/21VIR11804-1/2021	A / H5N1	Europe / Italy	Giacomo Barbierato (Istituto Zooprofilattico Sperimentale delle Venezie / Ricerca e innovazione)
EPI_ISL_8882168	A/chicken/Italy/21VIR10850/2021	A / H5N1	Europe / Italy	Giacomo Barbierato (Istituto Zooprofilattico Sperimentale delle Venezie / Ricerca e innovazione)
EPI_ISL_8882169	A/chicken/Italy/21VIR10573-1/2021	A / H5N1	Europe / Italy	Giacomo Barbierato (Istituto Zooprofilattico Sperimentale delle Venezie / Ricerca e innovazione)
EPI_ISL_8882170	A/turkey/Italy/21VIR11887-3/2021	A / H5N1	Europe / Italy	Giacomo Barbierato (Istituto Zooprofilattico Sperimentale delle Venezie / Ricerca e innovazione)
EPI_ISL_8882171	A/turkey/Italy/21VIR11591-8/2021	A / H5N1	Europe / Italy	Giacomo Barbierato (Istituto Zooprofilattico Sperimentale delle Venezie / Ricerca e innovazione)
EPI_ISL_8882172	A/turkey/Italy/21VIR10851/2021	A / H5N1	Europe / Italy	Giacomo Barbierato (Istituto Zooprofilattico Sperimentale delle Venezie / Ricerca e innovazione)
EPI_ISL_8882173	A/laying_hen/Italy/21VIR11502/2021	A / H5N1	Europe / Italy	Giacomo Barbierato (Istituto Zooprofilattico Sperimentale delle Venezie / Ricerca e innovazione)
EPI_ISL_8882187	A/turkey/Italy/21VIR11053-1/2021	A / H5N1	Europe / Italy	Giacomo Barbierato (Istituto Zooprofilattico Sperimentale delle Venezie / Ricerca e innovazione)
EPI_ISL_8882201	A/turkey/Italy/21VIR11803-1/2021	A / H5N1	Europe / Italy	Giacomo Barbierato (Istituto Zooprofilattico Sperimentale delle Venezie / Ricerca e innovazione)
EPI_ISL_8882209	A/little_owl/Italy/21VIR11382-1/2021	A / H5N1	Europe / Italy	Giacomo Barbierato (Istituto Zooprofilattico Sperimentale delle Venezie / Ricerca e innovazione)
EPI_ISL_8882210	A/seagull/Italy/21VIR10481-2/2021	A / H5N1	Europe / Italy	Giacomo Barbierato (Istituto Zooprofilattico Sperimentale delle Venezie / Ricerca e innovazione)
EPI_ISL_8882211	A/magpie/Italy/21VIR11656/2021	A / H5N1	Europe / Italy	Giacomo Barbierato (Istituto Zooprofilattico Sperimentale delle Venezie / Ricerca e innovazione)
EPI_ISL_8882212	A/cignus_olor/Italy/IZSLT_21VIR10529-1/2021	A / H5N1	Europe / Italy	Giacomo Barbierato (Istituto Zooprofilattico Sperimentale delle Venezie / Ricerca e innovazione)
EPI_ISL_8882213	A/seagull/Italy/21VIR11259-10/2021	A / H5N1	Europe / Italy	Giacomo Barbierato (Istituto Zooprofilattico Sperimentale delle Venezie / Ricerca e innovazione)
EPI_ISL_8882214	A/heron/Italy/21VIR10998-1/2021	A / H5N1	Europe / Italy	Giacomo Barbierato (Istituto Zooprofilattico Sperimentale delle Venezie / Ricerca e innovazione)
EPI_ISL_8882215	A/owl/Italy/21VIR11899-1/2021	A / H5N1	Europe / Italy	Giacomo Barbierato (Istituto Zooprofilattico Sperimentale delle Venezie / Ricerca e innovazione)
EPI_ISL_8882216	A/buzzard/Italy/21VIR11899-5/2021	A / H5N1	Europe / Italy	Giacomo Barbierato (Istituto Zooprofilattico Sperimentale delle Venezie / Ricerca e innovazione)
EPI_ISL_8882217	A/owl/Italy/21VIR11057/2021	A / H5N1	Europe / Italy	Giacomo Barbierato (Istituto Zooprofilattico Sperimentale delle Venezie / Ricerca e innovazione)
EPI_ISL_8882218	A/seagull/Italy/21VIR11259-12/2021	A / H5N1	Europe / Italy	Giacomo Barbierato (Istituto Zooprofilattico Sperimentale delle Venezie / Ricerca e innovazione)
EPI_ISL_9009291	A/chicken/Kursk/132-1V/2021	A / H5N1	Europe / Russian Federation / Kursk Oblast / Kursk	Natalia Goncharova (State Research Center of Virology and Biotechnology (VECTOR) / Emerging Zoonotic Diseases and Influenza)
EPI_ISL_9009294	A/chicken/Stavropol/146-1V/2021	A / H5N1	Europe / Russian Federation / Stavropol Krai / Stavropol	Natalia Goncharova (State Research Center of Virology and Biotechnology (VECTOR) / Emerging Zoonotic Diseases and Influenza)
EPI_ISL_9009296	A/chicken/Rostov-on-Don/159-1V/2021	A / H5N1	Europe / Russian Federation / Rostov Oblast / Rostov-on-Don	Natalia Goncharova (State Research Center of Virology and Biotechnology (VECTOR) / Emerging Zoonotic Diseases and Influenza)
EPI_ISL_9009297	A/chicken/Rostov-on-Don/159-2V/2021	A / H5N1	Europe / Russian Federation / Rostov Oblast / Rostov-on-Don	Natalia Goncharova (State Research Center of Virology and Biotechnology (VECTOR) / Emerging Zoonotic Diseases and Influenza)
EPI_ISL_9009302	A/chicken/Rostov-on-Don/159-3V/2021	A / H5N1	Europe / Russian Federation / Rostov Oblast / Rostov-on-Don	Natalia Goncharova (State Research Center of Virology and Biotechnology (VECTOR) / Emerging Zoonotic Diseases and Influenza)
EPI_ISL_9009303	A/chicken/Rostov-on-Don/159-4V/2021	A / H5N1	Europe / Russian Federation / Rostov Oblast / Rostov-on-Don	Natalia Goncharova (State Research Center of Virology and Biotechnology (VECTOR) / Emerging Zoonotic Diseases and Influenza)
EPI_ISL_9009304	A/chicken/Rostov-on-Don/159-5V/2021	A / H5N1	Europe / Russian Federation / Rostov Oblast / Rostov-on-Don	Natalia Goncharova (State Research Center of Virology and Biotechnology (VECTOR) / Emerging Zoonotic Diseases and Influenza)
EPI_ISL_9009309	A/chicken/Rostov-on-Don/159-6V/2021	A / H5N1	Europe / Russian Federation / Rostov Oblast / Rostov-on-Don	Natalia Goncharova (State Research Center of Virology and Biotechnology (VECTOR) / Emerging Zoonotic Diseases and Influenza)
EPI_ISL_9009317	A/chicken/Rostov-on-Don/159-7V/2021	A / H5N1	Europe / Russian Federation / Rostov Oblast / Rostov-on-Don	Natalia Goncharova (State Research Center of Virology and Biotechnology (VECTOR) / Emerging Zoonotic Diseases and Influenza)
EPI_ISL_9009472	A/chicken/Quang Tri/V4S4VTC/2020	A / H5N6	Asia / Vietnam / Tinh Quang Tri	Natalia Goncharova (State Research Center of Virology and Biotechnology (VECTOR) / Emerging Zoonotic Diseases and Influenza)
EPI_ISL_9009479	A/chicken/Thanh Hoa/V3S3VTC/2020	A / H5N6	Asia / Vietnam / Tinh Thanh Hoa	Natalia Goncharova (State Research Center of Virology and Biotechnology (VECTOR) / Emerging Zoonotic Diseases and Influenza)
EPI_ISL_9009481	A/muscovy duck/Nghe An/6873VTC/2020	A / H5N6	Asia / Vietnam / Nhge An	Natalia Goncharova (State Research Center of Virology and Biotechnology (VECTOR) / Emerging Zoonotic Diseases and Influenza)
EPI_ISL_9009483	A/chicken/Nghe An/7007VTC/2020	A / H5N6	Asia / Vietnam / Nhge An	Natalia Goncharova (State Research Center of Virology and Biotechnology (VECTOR) / Emerging Zoonotic Diseases and Influenza)
EPI_ISL_9009488	A/muscovy duck/Nghe An/7006VTC/2020	A / H5N6	Asia / Vietnam / Nhge An	Natalia Goncharova (State Research Center of Virology and Biotechnology (VECTOR) / Emerging Zoonotic Diseases and Influenza)
EPI_ISL_9009499	A/muscovy duck/Nghe An/259VTC/2021	A / H5N6	Asia / Vietnam / Nhge An	Natalia Goncharova (State Research Center of Virology and Biotechnology (VECTOR) / Emerging Zoonotic Diseases and Influenza)
EPI_ISL_9009515	A/chicken/Thanh Hoa/1351VTC/2021	A / H5N6	Asia / Vietnam / Tinh Thanh Hoa / Tan Thanh	Natalia Goncharova (State Research Center of Virology and Biotechnology (VECTOR) / Emerging Zoonotic Diseases and Influenza)
EPI_ISL_9009531	A/chicken/Thanh Hoa/6081VTC/2020	A / H5N6	Asia / Vietnam / Tinh Thanh Hoa / Quang Thanh	Natalia Goncharova (State Research Center of Virology and Biotechnology (VECTOR) / Emerging Zoonotic Diseases and Influenza)
EPI_ISL_9009546	A/duck/Thanh Hoa/5331VTC/2020	A / H5N6	Asia / Vietnam / Tinh Thanh Hoa / Co Lung	Natalia Goncharova (State Research Center of Virology and Biotechnology (VECTOR) / Emerging Zoonotic Diseases and Influenza)
EPI_ISL_9009552	A/chicken/Ha Tinh/514VTC/2021	A / H5N6	Asia / Vietnam / Nam Ha Tinh / Thach Van	Natalia Goncharova (State Research Center of Virology and Biotechnology (VECTOR) / Emerging Zoonotic Diseases and Influenza)
EPI_ISL_9009554	A/duck/Thanh Hoa/4643VTC/2020	A / H5N6	Asia / Vietnam / Tinh Thanh Hoa / Tan Tho	Natalia Goncharova (State Research Center of Virology and Biotechnology (VECTOR) / Emerging Zoonotic Diseases and Influenza)
EPI_ISL_9012457	A/chicken/England/053052/2021	A / H5N1	Europe / United Kingdom / Worcestershire / England	Alex Byrne (Animal and Plant Health Agency (APHA) / Virology Department)
EPI_ISL_9012572	A/pheasant/Wales/385129/2021	A / H5N1	Europe / United Kingdom / County Borough of Wrexham / Wales	Alex Byrne (Animal and Plant Health Agency (APHA) / Virology Department)
EPI_ISL_9012574	A/Herring Gull/Netherlands/1/2021	A / H5N1	Europe / Netherlands	Pascal Lexmond (Erasmus Medical Center / Viroscience)
EPI_ISL_9012577	A/Mallard/Netherlands/13/2021	A / H5N1	Europe / Netherlands	Pascal Lexmond (Erasmus Medical Center / Viroscience)
EPI_ISL_9012578	A/Mallard/Netherlands/14/2021	A / H5N1	Europe / Netherlands	Pascal Lexmond (Erasmus Medical Center / Viroscience)
EPI_ISL_9012580	A/Mallard/Netherlands/15/2021	A / H5N1	Europe / Netherlands	Pascal Lexmond (Erasmus Medical Center / Viroscience)
EPI_ISL_9012618	A/chicken/Wales/053969/2021	A / H5N1	Europe / United Kingdom / County Borough of Wrexham / Wales	Alex Byrne (Animal and Plant Health Agency (APHA) / Virology Department)
EPI_ISL_9012694	A/guineafowl/Scotland/054471/2021	A / H5N1	Europe / United Kingdom / Angus / Scotland	Alex Byrne (Animal and Plant Health Agency (APHA) / Virology Department)
EPI_ISL_9012696	A/chicken/Scotland/054477/2021	A / H5N1	Europe / United Kingdom / Angus / Scotland	Alex Byrne (Animal and Plant Health Agency (APHA) / Virology Department)
EPI_ISL_9012700	A/domestic_duck/Scotland/054469/2021	A / H5N1	Europe / United Kingdom / Angus / Scotland	Alex Byrne (Animal and Plant Health Agency (APHA) / Virology Department)
EPI_ISL_9029956	A/chicken/England/057314/2021	A / H5N1	Europe / United Kingdom / Lancashire / England	Alex Byrne (Animal and Plant Health Agency (APHA) / Virology Department)
EPI_ISL_9029957	A/turkey/England/057679/2021	A / H5N1	Europe / United Kingdom / Derbyshire / England	Alex Byrne (Animal and Plant Health Agency (APHA) / Virology Department)
EPI_ISL_9029959	A/domestic_duck/England/058612/2021	A / H5N1	Europe / United Kingdom / Dorset / England	Alex Byrne (Animal and Plant Health Agency (APHA) / Virology Department)
EPI_ISL_9029960	A/mute_swan/England/385466/2021	A / H5N1	Europe / United Kingdom / Gloucestershire / England	Alex Byrne (Animal and Plant Health Agency (APHA) / Virology Department)
EPI_ISL_9029961	A/Canada_goose/England/385250/2021	A / H5N1	Europe / United Kingdom / Stratford-upon-Avon	Alex Byrne (Animal and Plant Health Agency (APHA) / Virology Department)
EPI_ISL_9029962	A/Whooper_swan/Scotland/056219/2021	A / H5N1	Europe / United Kingdom / Dumfries and Galloway / Scotland	Alex Byrne (Animal and Plant Health Agency (APHA) / Virology Department)
EPI_ISL_9029965	A/Greylag_goose/England/054503/2021	A / H5N1	Europe / United Kingdom / Lancashire / England	Alex Byrne (Animal and Plant Health Agency (APHA) / Virology Department)

EPI_ISL_9111095	A/goose/Netherlands/22000487-001/2022	A / H5N1	Europe / Netherlands / Provincie Gelderland	Rene Heutink (Wageningen Bioveterinary Research)
EPI_ISL_9111104	A/otter/Netherlands/22001014-005/2022	A / H5N1	Europe / Netherlands / Provincie Flevoland	Rene Heutink (Wageningen Bioveterinary Research)
EPI_ISL_9111105	A/chicken/Netherlands/22001401-001005/2022	A / H5N1	Europe / Netherlands / Provincie Flevoland	Rene Heutink (Wageningen Bioveterinary Research)
EPI_ISL_9117228	A/northern goshawk/Netherlands/22000305-002/2022	A / H5N1	Europe / Netherlands / Provincie Utrecht	Rene Heutink (Wageningen Bioveterinary Research)
EPI_ISL_9160206	A/Anser_albifrons/Belgium/15465_0010/2021	A / H5N1	Europe / Belgium / Provincie West-Vlaanderen / Oudenburg	Steven Van Borm (Sciensano, Department of Animal Infectious Diseases / Animal Infectious Diseases)
EPI_ISL_9161618	A/Gallus_gallus/Belgium/17100_0001/2021	A / H5N1	Europe / Belgium / Provincie West-Vlaanderen / Veurne	Steven Van Borm (Sciensano, Department of Animal Infectious Diseases / Animal Infectious Diseases)
EPI_ISL_9162331	A/Phasianus_colchicus/Belgium/294/2022	A / H5N1	Europe / Belgium / Provincie Limburg / Bocholt	Steven Van Borm (Sciensano, Department of Animal Infectious Diseases / Animal Infectious Diseases)
EPI_ISL_9221411	A/barnacle goose/Netherlands/21041450-001/2021	A / H5N1	Europe / Netherlands / South Holland	Rene Heutink (Wageningen Bioveterinary Research)
EPI_ISL_9221430	A/common buzzard/Netherlands/21041451-002/2021	A / H5N1	Europe / Netherlands / South Holland	Rene Heutink (Wageningen Bioveterinary Research)
EPI_ISL_9221469	A/lesser black-backed gull/Netherlands/21041570-001/2021	A / H5N1	Europe / Netherlands / Provincie Noord-Holland	Rene Heutink (Wageningen Bioveterinary Research)
EPI_ISL_9221519	A/barnacle goose/Netherlands/22000419-002/2022	A / H5N1	Europe / Netherlands / Provincie Flevoland	Rene Heutink (Wageningen Bioveterinary Research)
EPI_ISL_9250718	A/Gallus_gallus/Belgium/11372_0001/2021	A / H5N8	Europe / Belgium / Province de Luxembourg / Chassepierre	Steven Van Borm (Sciensano, Department of Animal Infectious Diseases / Animal Infectious Diseases)
EPI_ISL_9261740	A/Mallard/Netherlands/17/2021	A / H5N3	Europe / Netherlands / Provincie Noord-Holland / Wieringen	Pascal Lexmond (Erasmus Medical Center / Viroscience)
EPI_ISL_9261741	A/Barnacle goose/Netherlands/1/2022	A / H5N1	Europe / Netherlands / Provincie Gelderland / Stroe	Pascal Lexmond (Erasmus Medical Center / Viroscience)
EPI_ISL_9261742	A/Great black-backed gull/1/2022	A / H5N1	Europe / Netherlands / Provincie Gelderland / Stroe	Pascal Lexmond (Erasmus Medical Center / Viroscience)
EPI_ISL_9261743	A/Oystercatcher/Netherlands/1/2022	A / H5N1	Europe / Netherlands / Provincie Noord-Holland / Normerven	Pascal Lexmond (Erasmus Medical Center / Viroscience)
EPI_ISL_9261744	A/Barnacle goose/Netherlands/2/2022	A / H5N1	Europe / Netherlands / Provincie Noord-Holland / Normerven	Pascal Lexmond (Erasmus Medical Center / Viroscience)
EPI_ISL_9261745	A/Barnacle goose/Netherlands/3/2022	A / H5N1	Europe / Netherlands / Provincie Noord-Holland / Zuiderdijk	Pascal Lexmond (Erasmus Medical Center / Viroscience)
EPI_ISL_9261746	A/Barnacle goose/Netherlands/4/2022	A / H5N1	Europe / Netherlands / Provincie Noord-Holland / Zuiderdijk	Pascal Lexmond (Erasmus Medical Center / Viroscience)
EPI_ISL_9261747	A/Barnacle goose/Netherlands/5/2022	A / H5N1	Europe / Netherlands / Provincie Noord-Holland / De Kreupel	Pascal Lexmond (Erasmus Medical Center / Viroscience)
EPI_ISL_9261748	A/Grey heron/Netherlands/1/2022	A / H5N1	Europe / Netherlands / Provincie Noord-Holland / De Kreupel	Pascal Lexmond (Erasmus Medical Center / Viroscience)
EPI_ISL_9261749	A/Caspian gull/Netherlands/1/2022	A / H5N1	Europe / Netherlands / Provincie Noord-Holland / De Kreupel	Pascal Lexmond (Erasmus Medical Center / Viroscience)
EPI_ISL_9261750	A/Black-headed gull/Netherlands/1/2022	A / H5N1	Europe / Netherlands / Provincie Noord-Holland / De Kreupel	Pascal Lexmond (Erasmus Medical Center / Viroscience)
EPI_ISL_9261751	A/Barnacle goose/Netherlands/6/2022	A / H5N1	Europe / Netherlands / Provincie Noord-Holland / De Kreupel	Pascal Lexmond (Erasmus Medical Center / Viroscience)
EPI_ISL_9304992	A/barnacle goose/Netherlands/22000551-002/2022	A / H5N1	Europe / Netherlands / Provincie Friesland	Rene Heutink (Wageningen Bioveterinary Research)
EPI_ISL_9377021	A/goose/France/21P014207/2021	A / H5N1	Europe / France / Midi-Pyrenees / Departement des Hautes-Pyrenees	Francois-Xavier Briand (ANSES Agence Nationale De Securite Sanitaire De L'alimentation / Laboratoire de Ploufragan-Plouzané)
EPI_ISL_9401912	A/white-fronted Goose/Croatia/16/2022	A / H5N1	Europe / Croatia / Osjecko-Baranjska Zupanija	Vladimir Savi? (Croatian Veterinary Institute / Poultry Centre)
EPI_ISL_94329	A/duck/Eastern China/008/2008	A / H5N5	Asia / China	
EPI_ISL_94330	A/duck/Eastern China/031/2009	A / H5N5	Asia / China	
EPI_ISL_94331	A/duck/Eastern China/108/2008	A / H5N1	Asia / China	
EPI_ISL_943551	A/Chicken/Sweden/SVA210121SZ0033/KN000273-IP3/2021	A / H5N5	Europe / Sweden / Kalmar Lan / Monsteras Kommun	Siamak Zohari (National Veterinary Institute)
EPI_ISL_943553	A/Chicken/Sweden/SVA210117SZ0004/KN011326-IP3/2021	A / H5N5	Europe / Sweden / Kalmar Lan / Monsteras Kommun	Siamak Zohari (National Veterinary Institute)
EPI_ISL_9439410	A/grey heron/Netherlands/22000049-002/2021	A / H5N1	Europe / Netherlands / Provincie Noord-Holland	Rene Heutink (Wageningen Bioveterinary Research)
EPI_ISL_956368	A/mute swan/Czech Republic/1410-2/2021	A / H5N8	Europe / Czech Republic / Jihocesky Kraj / Okres Pisek / Zlivicky pond; GPS 49°21'26.626''N 14°6'	Alexander Nagy (State Veterinary Institute Prague)
EPI_ISL_956408	A/common_teal/Italy/20VIR7608-73/2020	A / H5N8	Europe / Italy / Venezia	Bianca Zecchin (Istituto Zooprofilattico Sperimentale Delle Venezie)
EPI_ISL_956409	A/common_teal/Italy/20VIR7439-190/2020	A / H5N5	Europe / Italy / Rovigo	Bianca Zecchin (Istituto Zooprofilattico Sperimentale Delle Venezie)
EPI_ISL_956411	A/greylag_goose/Italy/20VIR7660-6/2020	A / H5N8	Europe / Italy / Venezia	Bianca Zecchin (Istituto Zooprofilattico Sperimentale Delle Venezie)
EPI_ISL_956412	A/greater_white-fronted_goose/Italy/20VIR8073-4/2020	A / H5N1	Europe / Italy / Gorizia	Bianca Zecchin (Istituto Zooprofilattico Sperimentale Delle Venezie)
EPI_ISL_956414	A/Eurasian_wigeon/Italy/20VIR7301-362/2020	A / H5N8	Europe / Italy / Venezia	Bianca Zecchin (Istituto Zooprofilattico Sperimentale Delle Venezie)
EPI_ISL_9572654	A/duck/Nigeria/VRD19-068DC019T_19RS1081-12/2019	A / H5N8	Africa / Nigeria	
EPI_ISL_9572655	A/duck/Nigeria/VRD19-068DC004T_19RS1081-10/2019	A / H5N8	Africa / Nigeria	
EPI_ISL_9572656	A/chicken/Nigeria/VRD-19-023_19RS1081-1/2019	A / H5N8	Africa / Nigeria	
EPI_ISL_9572657	A/chicken/Nigeria/VRD-19-055_19RS1081-3/2019	A / H5N8	Africa / Nigeria	
EPI_ISL_9572658	A/chicken/Nigeria/VRD-19-069_19RS1081-5/2019	A / H5N8	Africa / Nigeria	
EPI_ISL_9572659	A/chicken/Nigeria/VRD-19-073_19RS1081-6/2019	A / H5N8	Africa / Nigeria	
EPI_ISL_9572672	A/chicken/Nigeria/VRD-18-NS10_18RS1971-29/2018	A / H5N8	Africa / Nigeria	
EPI_ISL_9572689	A/chicken/Egypt/FN1/2020	A / H5	Africa / Egypt	
EPI_ISL_9572690	A/chicken/Egypt/FN2/2020	A / H5	Africa / Egypt	
EPI_ISL_9572691	A/chicken/Egypt/FN3/2020	A / H5	Africa / Egypt	
EPI_ISL_9572739	A/Muscovy duck/Vietnam/HN6610/2020	A / H5N6	Asia / Vietnam	
EPI_ISL_9572741	A/duck/Vietnam/HN6611/2020	A / H5N6	Asia / Vietnam	
EPI_ISL_9572745	A/Muscovy duck/Vietnam/HN6609/2020	A / H5N6	Asia / Vietnam	
EPI_ISL_9572754	A/Muscovy duck/Vietnam/HN6606/2020	A / H5N6	Asia / Vietnam	
EPI_ISL_9572759	A/Muscovy duck/Vietnam/HN6607/2020	A / H5N6	Asia / Vietnam	
EPI_ISL_9572770	A/Muscovy duck/Vietnam/HN6608/2020	A / H5N6	Asia / Vietnam	
EPI_ISL_9572787	A/Muscovy duck/Vietnam/HN6113/2020	A / H5N6	Asia / Vietnam	
EPI_ISL_9572792	A/Muscovy duck/Vietnam/HN6120/2020	A / H5N6	Asia / Vietnam	
EPI_ISL_9572794	A/Muscovy duck/Vietnam/HN6115/2020	A / H5N6	Asia / Vietnam	
EPI_ISL_9572800	A/Muscovy duck/Vietnam/HN6119/2020	A / H5N6	Asia / Vietnam	
EPI_ISL_9572808	A/Muscovy duck/Vietnam/HN6114/2020	A / H5N6	Asia / Vietnam	
EPI_ISL_9572812	A/Muscovy duck/Vietnam/HN6111/2020	A / H5N6	Asia / Vietnam	
EPI_ISL_9572816	A/duck/Vietnam/QN6519/2020	A / H5N6	Asia / Vietnam	
EPI_ISL_9594290	A/chicken/Egypt/A19670/2021	A / H5N8	Africa / Egypt	
EPI_ISL_9594292	A/chicken/Egypt/N15174C/2018	A / H5N8	Africa / Egypt	
EPI_ISL_9594293	A/chicken/Egypt/N15175C/2018	A / H5N8	Africa / Egypt	
EPI_ISL_9594294	A/chicken/Egypt/N15173A/2018	A / H5N8	Africa / Egypt	
EPI_ISL_9594297	A/chicken/Egypt/N15178B/2018	A / H5N8	Africa / Egypt	
EPI_ISL_9594298	A/chicken/Egypt/N15176A/2018	A / H5N8	Africa / Egypt	
EPI_ISL_9594299	A/chicken/Egypt/N15168C/2018	A / H5N8	Africa / Egypt	
EPI_ISL_9594301	A/chicken/Egypt/N15174A/2018	A / H5N8	Africa / Egypt	
EPI_ISL_9594302	A/chicken/Egypt/F15100/2018	A / H5N8	Africa / Egypt	
EPI_ISL_9594303	A/chicken/Egypt/N15173C/2018	A / H5N8	Africa / Egypt	

EPI_ISL_9594305	A/chicken/Egypt/N15173B/2018	A / H5N8	Africa / Egypt	
EPI_ISL_9594306	A/chicken/Egypt/S18182C/2020	A / H5N8	Africa / Egypt	
EPI_ISL_9594307	A/chicken/Egypt/N15178D/2018	A / H5N8	Africa / Egypt	
EPI_ISL_9594308	A/chicken/Egypt/N15178C/2018	A / H5N8	Africa / Egypt	
EPI_ISL_9594309	A/chicken/Egypt/N15178A/2018	A / H5N8	Africa / Egypt	
EPI_ISL_9594310	A/chicken/Egypt/N15176B/2018	A / H5N8	Africa / Egypt	
EPI_ISL_9594311	A/chicken/Egypt/N15169A/2018	A / H5N8	Africa / Egypt	
EPI_ISL_9594312	A/chicken/Egypt/N15177C/2018	A / H5N8	Africa / Egypt	
EPI_ISL_9603767	A/goose/Czech Republic/23458-2T/2021	A / H5N1	Europe / Czech Republic / Jihocesky Kraj / Okres Ceske Budejovice / Nové Hrady, Štipto?	Alexander Nagy (State Veterinary Institute Prague)
EPI_ISL_9603769	A/goose/Czech Republic/23458-5T/2021	A / H5N1	Europe / Czech Republic / Jihocesky Kraj / Okres Ceske Budejovice / Nové Hrady, Štipto?	Alexander Nagy (State Veterinary Institute Prague)
EPI_ISL_9603775	A/goose/Czech Republic/23458-4T/2021	A / H5N1	Europe / Czech Republic / Jihocesky Kraj / Okres Ceske Budejovice / Nové Hrady, Štipto?	Alexander Nagy (State Veterinary Institute Prague)
EPI_ISL_9603780	A/goose/Czech Republic/23458-1K/2021	A / H5N1	Europe / Czech Republic / Jihocesky Kraj / Okres Ceske Budejovice / Nové Hrady, Štipto?	Alexander Nagy (State Veterinary Institute Prague)
EPI_ISL_9603795	A/goose/Czech Republic/22608-1T/2021	A / H5N1	Europe / Czech Republic / Nové Hrady, By?ov	Alexander Nagy (State Veterinary Institute Prague)
EPI_ISL_9603819	A/goose/Czech Republic/22608-2T/2021	A / H5N1	Europe / Czech Republic / Jihocesky Kraj / Okres Ceske Budejovice / Nové Hrady, By?ov	Alexander Nagy (State Veterinary Institute Prague)
EPI_ISL_9603901	A/goose/Czech Republic/22608-3T/2021	A / H5N1	Europe / Czech Republic / Jihocesky Kraj / Okres Ceske Budejovice / Nové Hrady, By?ov	Alexander Nagy (State Veterinary Institute Prague)
EPI_ISL_9603916	A/mute swan/Czech Republic/22477-1/2021	A / H5N1	Europe / Czech Republic / Zlinsky Kraj / Okres Kromeriz / Doubravicky pond, GPS: 49°17'17.233''	Alexander Nagy (State Veterinary Institute Prague)
EPI_ISL_9603918	A/mute swan/Czech Republic/22477-2/2021	A / H5N1	Europe / Czech Republic / Zlinsky Kraj / Okres Kromeriz / Doubravicky pond, GPS: 49°17'17.233''	Alexander Nagy (State Veterinary Institute Prague)
EPI_ISL_9603920	A/mute swan/Czech Republic/22380/2021	A / H5N1	Europe / Czech Republic / Kralovehradecky Kraj / Okres Jicin / Ostruzensky pond, GPS: 50°26'33,	Alexander Nagy (State Veterinary Institute Prague)
EPI_ISL_9603922	A/mute swan/Czech Republic/22684/2021	A / H5N1	Europe / Czech Republic / Olomoucky Kraj / Okres Prerov / Na hrazi pond, GPS: 49,3582233N, 17	Alexander Nagy (State Veterinary Institute Prague)
EPI_ISL_9603924	A/goose/Czech Republic/22750/2021	A / H5N1	Europe / Czech Republic / Jihocesky Kraj / Okres Ceske Budejovice / Nové Hrady, Velebil pond	Alexander Nagy (State Veterinary Institute Prague)
EPI_ISL_9603927	A/mute swan/Czech Republic/25702-2/2021	A / H5N1	Europe / Czech Republic / Olomoucky Kraj / Okres Prostejov / Víceme?ice	Alexander Nagy (State Veterinary Institute Prague)
EPI_ISL_9603929	A/chicken/Czech Republic/25690/2021	A / H5N1	Europe / Czech Republic / Ustecky Kraj / Okres Litomerice / Libotenice, GPS: 50°28'8.285''N, 14°	Alexander Nagy (State Veterinary Institute Prague)
EPI_ISL_9603931	A/pheasant/Czech Republic/25827-1/2021	A / H5N1	Europe / Czech Republic / South Moravian Region / Okres Hodonin / Jarohn?vice-Mist?ín	Alexander Nagy (State Veterinary Institute Prague)
EPI_ISL_9603937	A/pheasant/Czech Republic/25827-2/2021	A / H5N1	Europe / Czech Republic / South Moravian Region / Okres Hodonin / Jarohn?vice-Mist?ín	Alexander Nagy (State Veterinary Institute Prague)
EPI_ISL_9603939	A/pheasant/Czech Republic/25827-3/2021	A / H5N1	Europe / Czech Republic / South Moravian Region / Okres Hodonin / Jarohn?vice-Mist?ín	Alexander Nagy (State Veterinary Institute Prague)
EPI_ISL_9603943	A/mute swan/Czech Republic/785/2022	A / H5N1	Europe / Czech Republic / Stredocesky Kraj / Okres Praha-Zapad / Davle, GPS: 49.8909144N, 14.4	Alexander Nagy (State Veterinary Institute Prague)
EPI_ISL_9616210	A/gadwall/Netherlands/22000307-001/2022	A / H5N1	Europe / Netherlands / Provincie Noord-Holland	Rene Heutink (Wageningen Bioveterinary Research)
EPI_ISL_9616212	A/red knot/Netherlands/22000409-002/2022	A / H5N1	Europe / Netherlands / Provincie Noord-Holland	Rene Heutink (Wageningen Bioveterinary Research)
EPI_ISL_9616263	A/brant goose/Netherlands/22000648-002/2022	A / H5N1	Europe / Netherlands / Provincie Zeeland	Rene Heutink (Wageningen Bioveterinary Research)
EPI_ISL_9616265	A/red fox/Netherlands/22001901-005/2022	A / H5N1	Europe / Netherlands / Provincie Utrecht	Rene Heutink (Wageningen Bioveterinary Research)
EPI_ISL_9616266	A/polecat/Netherlands/22002047-001/2022	A / H5N1	Europe / Netherlands / Provincie Gelderland	Rene Heutink (Wageningen Bioveterinary Research)
EPI_ISL_9616271	A/red fox/Netherlands/22002190-003/2022	A / H5N1	Europe / Netherlands / Provincie Flevoland	Rene Heutink (Wageningen Bioveterinary Research)
EPI_ISL_9616272	A/red fox/Netherlands/22002191-003/2022	A / H5N1	Europe / Netherlands / Provincie Utrecht	Rene Heutink (Wageningen Bioveterinary Research)
EPI_ISL_9690841	A/great black-backed gull/Netherlands/22000090-002/2022	A / H5N1	Europe / Netherlands / South Holland	Rene Heutink (Wageningen Bioveterinary Research)
EPI_ISL_9690956	A/common buzzard/Netherlands/22000192-001/2022	A / H5N1	Europe / Netherlands / South Holland	Rene Heutink (Wageningen Bioveterinary Research)
EPI_ISL_977513	A/chicken/Czech Republic/1566-1/2021	A / H5N8	Europe / Czech Republic / Jihocesky Kraj / Okres Tabor / Dlouha Lhota/Chynov; GPS 49.3561703	Alexander Nagy (State Veterinary Institute Prague)
EPI_ISL_977582	A/duck/Northern China/ZGL/2020(H5N8)	A / H5N8	Asia / China / Shandong Province	Jiahao Zhang (South China Agricultural University / College of Veterinary Medicine)
EPI_ISL_977595	A/duck/Southwestern China/B1904/2020(H5N8)	A / H5N8	Asia / China / Sichuan Province	Jiahao Zhang (South China Agricultural University / College of Veterinary Medicine)
EPI_ISL_977599	A/duck/Northern China/LSP/2020(H5N8)	A / H5N8	Asia / China / Shandong Province	Jiahao Zhang (South China Agricultural University / College of Veterinary Medicine)
EPI_ISL_978890	A/chicken/Chiba/E1T/2021	A / H5N8	Asia / Japan / Chiba	Takehiko Saito (National Institute of Animal Health)
EPI_ISL_978891	A/chicken/Chiba/E2T/2021	A / H5N8	Asia / Japan / Chiba	Takehiko Saito (National Institute of Animal Health)
EPI_ISL_978892	A/chicken/Chiba/E3T/2021	A / H5N8	Asia / Japan / Chiba	Takehiko Saito (National Institute of Animal Health)
EPI_ISL_978893	A/chicken/Chiba/E4T/2021	A / H5N8	Asia / Japan / Chiba	Takehiko Saito (National Institute of Animal Health)
EPI_ISL_978894	A/chicken/Chiba/F1T/2021	A / H5N8	Asia / Japan / Chiba	Takehiko Saito (National Institute of Animal Health)
EPI_ISL_978895	A/chicken/Chiba/F2T/2021	A / H5N8	Asia / Japan / Chiba	Takehiko Saito (National Institute of Animal Health)
EPI_ISL_978896	A/chicken/Chiba/F3C/2021	A / H5N8	Asia / Japan / Chiba	Takehiko Saito (National Institute of Animal Health)
EPI_ISL_978897	A/chicken/Chiba/F4T/2021	A / H5N8	Asia / Japan / Chiba	Takehiko Saito (National Institute of Animal Health)
EPI_ISL_978898	A/chicken/Ibaraki/1C/2021	A / H5N8	Asia / Japan / Ibaraki	Takehiko Saito (National Institute of Animal Health)
EPI_ISL_978899	A/chicken/Ibaraki/3T/2021	A / H5N8	Asia / Japan / Ibaraki	Takehiko Saito (National Institute of Animal Health)
EPI_ISL_978900	A/chicken/Ibaraki/7C/2021	A / H5N8	Asia / Japan / Ibaraki	Takehiko Saito (National Institute of Animal Health)
EPI_ISL_978901	A/chicken/Ibaraki/8T/2021	A / H5N8	Asia / Japan / Ibaraki	Takehiko Saito (National Institute of Animal Health)
EPI_ISL_978902	A/chicken/Miyazaki/J12T/2021	A / H5N8	Asia / Japan / Miyazaki	Takehiko Saito (National Institute of Animal Health)
EPI_ISL_978903	A/chicken/Miyazaki/J3T/2021	A / H5N8	Asia / Japan / Miyazaki	Takehiko Saito (National Institute of Animal Health)
EPI_ISL_978904	A/chicken/Miyazaki/J4T/2021	A / H5N8	Asia / Japan / Miyazaki	Takehiko Saito (National Institute of Animal Health)
EPI_ISL_978905	A/chicken/Miyazaki/J6T/2021	A / H5N8	Asia / Japan / Miyazaki	Takehiko Saito (National Institute of Animal Health)
EPI_ISL_978906	A/chicken/Toyama/1T/2021	A / H5N8	Asia / Japan / Toyama	Takehiko Saito (National Institute of Animal Health)
EPI_ISL_978907	A/chicken/Toyama/2T/2021	A / H5N8	Asia / Japan / Toyama	Takehiko Saito (National Institute of Animal Health)
EPI_ISL_978908	A/chicken/Toyama/3T/2021	A / H5N8	Asia / Japan / Toyama	Takehiko Saito (National Institute of Animal Health)
EPI_ISL_978909	A/chicken/Toyama/4T/2021	A / H5N8	Asia / Japan / Toyama	Takehiko Saito (National Institute of Animal Health)
EPI_ISL_978910	A/duck/Chiba/C1T/2021	A / H5N8	Asia / Japan / Chiba	Takehiko Saito (National Institute of Animal Health)
EPI_ISL_978911	A/duck/Chiba/C2T/2021	A / H5N8	Asia / Japan / Chiba	Takehiko Saito (National Institute of Animal Health)
EPI_ISL_978912	A/duck/Chiba/C3T/2021	A / H5N8	Asia / Japan / Chiba	Takehiko Saito (National Institute of Animal Health)
EPI_ISL_978913	A/duck/Chiba/C5T/2021	A / H5N8	Asia / Japan / Chiba	Takehiko Saito (National Institute of Animal Health)
EPI_ISL_978914	A/duck/Chiba/D1-3T/2021	A / H5N8	Asia / Japan / Chiba	Takehiko Saito (National Institute of Animal Health)
EPI_ISL_978915	A/mute swan/Ibaraki/080203C/2021	A / H5N8	Asia / Japan / Ibaraki	Takehiko Saito (National Institute of Animal Health)
EPI_ISL_978916	A/mute swan/Ibaraki/080203T/2021	A / H5N8	Asia / Japan / Ibaraki	Takehiko Saito (National Institute of Animal Health)
EPI_ISL_980839	A/mute swan/Czech Republic/1410-1/2021	A / H5N8	Europe / Czech Republic / Jihocesky Kraj / Okres Pisek / Zlivicky pond; GPS 49°21'26.626''N 14°6'	Alexander Nagy (State Veterinary Institute Prague)
EPI_ISL_984670	A/barnacle goose/Denmark/14139-1/2020	A / H5N8	Europe / Denmark / Region Syddanmark / Tonder Kommune	Charlotte Kristiane Hjulsager (Statens Serum Institute / Microbiological Diagnostic and Virology)
EPI_ISL_984673	A/barnacle goose/Denmark/14139-2/2020	A / H5N8	Europe / Denmark / Region Syddanmark / Tonder Kommune	Charlotte Kristiane Hjulsager (Statens Serum Institute / Microbiological Diagnostic and Virology)
EPI_ISL_984675	A/barnacle goose/Denmark/14139-3/2020	A / H5N8	Europe / Denmark / Region Syddanmark / Tonder Kommune	Charlotte Kristiane Hjulsager (Statens Serum Institute / Microbiological Diagnostic and Virology)
EPI_ISL_984680	A/black-headed gull/Denmark/14139-4/2020	A / H5N8	Europe / Denmark / Region Syddanmark / Tonder Kommune	Charlotte Kristiane Hjulsager (Statens Serum Institute / Microbiological Diagnostic and Virology)
EPI_ISL_984681	A/barnacle goose/Denmark/14534-1/2020	A / H5N8	Europe / Denmark / Region Syddanmark / Tonder Kommune	Charlotte Kristiane Hjulsager (Statens Serum Institute / Microbiological Diagnostic and Virology)

EPI_ISL_984684	A/barnacle goose/Denmark/14537-1/2020	A / H5N8	Europe / Denmark / Region Syddanmark / Vejle Kommune	Charlotte Kristiane Hjulsager (Statens Serum Institute / Microbiological Diagnostic and Virology)
EPI_ISL_984686	A/barnacle goose/Denmark/14536-1/2020	A / H5N8	Europe / Denmark / Region Syddanmark / Abenra Kommune	Charlotte Kristiane Hjulsager (Statens Serum Institute / Microbiological Diagnostic and Virology)
EPI_ISL_984688	A/barnacle goose/Denmark/14538-1/2020	A / H5N8	Europe / Denmark / Region Syddanmark / Sonderborg Kommune	Charlotte Kristiane Hjulsager (Statens Serum Institute / Microbiological Diagnostic and Virology)
EPI_ISL_984690	A/peregrine falcon/Denmark/14596-1/2020	A / H5N8	Europe / Denmark / Region Syddanmark / Svendborg Kommune	Charlotte Kristiane Hjulsager (Statens Serum Institute / Microbiological Diagnostic and Virology)
EPI_ISL_984691	A/common buzzard/Denmark/14600-1/2020	A / H5N8	Europe / Denmark / Region Syddanmark / Abenra Kommune	Charlotte Kristiane Hjulsager (Statens Serum Institute / Microbiological Diagnostic and Virology)
EPI_ISL_984692	A/barnacle goose/Denmark/14600-2/2020	A / H5N8	Europe / Denmark / Region Syddanmark / Abenra Kommune	Charlotte Kristiane Hjulsager (Statens Serum Institute / Microbiological Diagnostic and Virology)
EPI_ISL_984693	A/barnacle goose/Denmark/14599-1/2020	A / H5N8	Europe / Denmark / Region Sjaland / Kalundborg Kommune	Charlotte Kristiane Hjulsager (Statens Serum Institute / Microbiological Diagnostic and Virology)
EPI_ISL_984694	A/graylag goose/Denmark/14535-1/2020	A / H5N8	Europe / Denmark / Region Nordjylland / Alborg	Charlotte Kristiane Hjulsager (Statens Serum Institute / Microbiological Diagnostic and Virology)
EPI_ISL_984695	A/chicken/Denmark/14819-6/2020	A / H5N8	Europe / Denmark / Region Midtjylland / Randers Kommune	Charlotte Kristiane Hjulsager (Statens Serum Institute / Microbiological Diagnostic and Virology)
EPI_ISL_985179	A/duck/Korea/H338/2020	A / H5N8	Asia / Korea, Republic of / JB	Yu-Na Lee (Animal and Plant Quarantine Agency (S-2158) / Avian Influenza Research & Diagnostic Division)
EPI_ISL_985180	A/chicken/Korea/H365/2020	A / H5N8	Asia / Korea, Republic of / GB	Yu-Na Lee (Animal and Plant Quarantine Agency (S-2158) / Avian Influenza Research & Diagnostic Division)
EPI_ISL_985181	A/duck/Korea/H385/2020	A / H5N8	Asia / Korea, Republic of / JN	Yu-Na Lee (Animal and Plant Quarantine Agency (S-2158) / Avian Influenza Research & Diagnostic Division)
EPI_ISL_985182	A/chicken/Korea/H390/2020	A / H5N8	Asia / Korea, Republic of / GG	Yu-Na Lee (Animal and Plant Quarantine Agency (S-2158) / Avian Influenza Research & Diagnostic Division)
EPI_ISL_985183	A/quail/Korea/H394/2020	A / H5N8	Asia / Korea, Republic of / CB	Yu-Na Lee (Animal and Plant Quarantine Agency (S-2158) / Avian Influenza Research & Diagnostic Division)
EPI_ISL_985184	A/duck/Korea/H411/2020	A / H5N8	Asia / Korea, Republic of / JN	Yu-Na Lee (Animal and Plant Quarantine Agency (S-2158) / Avian Influenza Research & Diagnostic Division)
EPI_ISL_985185	A/quail/Korea/H412/2020	A / H5N8	Asia / Korea, Republic of / GG	Yu-Na Lee (Animal and Plant Quarantine Agency (S-2158) / Avian Influenza Research & Diagnostic Division)
EPI_ISL_985186	A/duck/Korea/H419/2020	A / H5N8	Asia / Korea, Republic of / JN	Yu-Na Lee (Animal and Plant Quarantine Agency (S-2158) / Avian Influenza Research & Diagnostic Division)
EPI_ISL_985187	A/duck/Korea/H431/2020	A / H5N8	Asia / Korea, Republic of / JN	Yu-Na Lee (Animal and Plant Quarantine Agency (S-2158) / Avian Influenza Research & Diagnostic Division)
EPI_ISL_985188	A/duck/Korea/H432/2020	A / H5N8	Asia / Korea, Republic of / JN	Yu-Na Lee (Animal and Plant Quarantine Agency (S-2158) / Avian Influenza Research & Diagnostic Division)
EPI_ISL_985189	A/duck/Korea/H438/2020	A / H5N8	Asia / Korea, Republic of / JN	Yu-Na Lee (Animal and Plant Quarantine Agency (S-2158) / Avian Influenza Research & Diagnostic Division)
EPI_ISL_985190	A/duck/Korea/H439/2020	A / H5N8	Asia / Korea, Republic of / JN	Yu-Na Lee (Animal and Plant Quarantine Agency (S-2158) / Avian Influenza Research & Diagnostic Division)
EPI_ISL_985191	A/chicken/Korea/H440/2020	A / H5N8	Asia / Korea, Republic of / GG	Yu-Na Lee (Animal and Plant Quarantine Agency (S-2158) / Avian Influenza Research & Diagnostic Division)
EPI_ISL_985192	A/chicken/Korea/H441/2020	A / H5N8	Asia / Korea, Republic of / JB	Yu-Na Lee (Animal and Plant Quarantine Agency (S-2158) / Avian Influenza Research & Diagnostic Division)
EPI_ISL_985193	A/chicken/Korea/H450/2020	A / H5N8	Asia / Korea, Republic of / GB	Yu-Na Lee (Animal and Plant Quarantine Agency (S-2158) / Avian Influenza Research & Diagnostic Division)
EPI_ISL_985194	A/goose/Korea/H449/2020	A / H5N8	Asia / Korea, Republic of / CN	Yu-Na Lee (Animal and Plant Quarantine Agency (S-2158) / Avian Influenza Research & Diagnostic Division)
EPI_ISL_985195	A/chicken/Korea/H470/2020	A / H5N8	Asia / Korea, Republic of / GG	Yu-Na Lee (Animal and Plant Quarantine Agency (S-2158) / Avian Influenza Research & Diagnostic Division)
EPI_ISL_985196	A/duck/Korea/H471/2020	A / H5N8	Asia / Korea, Republic of / JN	Yu-Na Lee (Animal and Plant Quarantine Agency (S-2158) / Avian Influenza Research & Diagnostic Division)
EPI_ISL_985197	A/duck/Korea/H499/2020	A / H5N8	Asia / Korea, Republic of / GG	Yu-Na Lee (Animal and Plant Quarantine Agency (S-2158) / Avian Influenza Research & Diagnostic Division)
EPI_ISL_985198	A/chicken/Korea/H491/2020	A / H5N8	Asia / Korea, Republic of / GG	Yu-Na Lee (Animal and Plant Quarantine Agency (S-2158) / Avian Influenza Research & Diagnostic Division)
EPI_ISL_985199	A/chicken/Korea/H510/2020	A / H5N8	Asia / Korea, Republic of / GG	Yu-Na Lee (Animal and Plant Quarantine Agency (S-2158) / Avian Influenza Research & Diagnostic Division)
EPI_ISL_985200	A/duck/Korea/H509/2020	A / H5N8	Asia / Korea, Republic of / CB	Yu-Na Lee (Animal and Plant Quarantine Agency (S-2158) / Avian Influenza Research & Diagnostic Division)
EPI_ISL_985201	A/duck/Korea/H511/2020	A / H5N8	Asia / Korea, Republic of / JB	Yu-Na Lee (Animal and Plant Quarantine Agency (S-2158) / Avian Influenza Research & Diagnostic Division)
EPI_ISL_985202	A/duck/Korea/H514/2020	A / H5N8	Asia / Korea, Republic of / JB	Yu-Na Lee (Animal and Plant Quarantine Agency (S-2158) / Avian Influenza Research & Diagnostic Division)
EPI_ISL_985203	A/duck/Korea/H515/2020	A / H5N8	Asia / Korea, Republic of / JN	Yu-Na Lee (Animal and Plant Quarantine Agency (S-2158) / Avian Influenza Research & Diagnostic Division)
EPI_ISL_985204	A/duck/Korea/H516/2020	A / H5N8	Asia / Korea, Republic of / JN	Yu-Na Lee (Animal and Plant Quarantine Agency (S-2158) / Avian Influenza Research & Diagnostic Division)
EPI_ISL_985205	A/duck/Korea/H524/2020	A / H5N8	Asia / Korea, Republic of / CN	Yu-Na Lee (Animal and Plant Quarantine Agency (S-2158) / Avian Influenza Research & Diagnostic Division)
EPI_ISL_985206	A/chicken/Korea/H525/2020	A / H5N8	Asia / Korea, Republic of / CN	Yu-Na Lee (Animal and Plant Quarantine Agency (S-2158) / Avian Influenza Research & Diagnostic Division)
EPI_ISL_985207	A/chicken/Korea/H526/2020	A / H5N8	Asia / Korea, Republic of / GB	Yu-Na Lee (Animal and Plant Quarantine Agency (S-2158) / Avian Influenza Research & Diagnostic Division)
EPI_ISL_985208	A/duck/Korea/H528/2020	A / H5N8	Asia / Korea, Republic of / JB	Yu-Na Lee (Animal and Plant Quarantine Agency (S-2158) / Avian Influenza Research & Diagnostic Division)
EPI_ISL_985209	A/chicken/Korea/H531/2020	A / H5N8	Asia / Korea, Republic of / GG	Yu-Na Lee (Animal and Plant Quarantine Agency (S-2158) / Avian Influenza Research & Diagnostic Division)
EPI_ISL_985210	A/chicken/Korea/H532/2020	A / H5N8	Asia / Korea, Republic of / GG	Yu-Na Lee (Animal and Plant Quarantine Agency (S-2158) / Avian Influenza Research & Diagnostic Division)
EPI_ISL_985211	A/duck/Korea/H538/2020	A / H5N8	Asia / Korea, Republic of / JB	Yu-Na Lee (Animal and Plant Quarantine Agency (S-2158) / Avian Influenza Research & Diagnostic Division)
EPI_ISL_985220	A/chicken/Chiba/G1C/2021	A / H5N8	Asia / Japan / Chiba	Takehiko Saito (National Institute of Animal Health)
EPI_ISL_985221	A/chicken/Chiba/G1T/2021	A / H5N8	Asia / Japan / Chiba	Takehiko Saito (National Institute of Animal Health)
EPI_ISL_985222	A/chicken/Chiba/G2C/2021	A / H5N8	Asia / Japan / Chiba	Takehiko Saito (National Institute of Animal Health)
EPI_ISL_985223	A/chicken/Chiba/G2T/2021	A / H5N8	Asia / Japan / Chiba	Takehiko Saito (National Institute of Animal Health)
EPI_ISL_985224	A/chicken/Chiba/H1C/2021	A / H5N8	Asia / Japan / Chiba	Takehiko Saito (National Institute of Animal Health)
EPI_ISL_985225	A/chicken/Chiba/H1T/2021	A / H5N8	Asia / Japan / Chiba	Takehiko Saito (National Institute of Animal Health)
EPI_ISL_985226	A/chicken/Chiba/H2C/2021	A / H5N8	Asia / Japan / Chiba	Takehiko Saito (National Institute of Animal Health)
EPI_ISL_985227	A/chicken/Chiba/H2T/2021	A / H5N8	Asia / Japan / Chiba	Takehiko Saito (National Institute of Animal Health)
EPI_ISL_985228	A/chicken/Miyazaki/K1T/2021	A / H5N8	Asia / Japan / Miyazaki	Takehiko Saito (National Institute of Animal Health)
EPI_ISL_985229	A/chicken/Miyazaki/K3T/2021	A / H5N8	Asia / Japan / Miyazaki	Takehiko Saito (National Institute of Animal Health)
EPI_ISL_985230	A/chicken/Miyazaki/K5T/2021	A / H5N8	Asia / Japan / Miyazaki	Takehiko Saito (National Institute of Animal Health)
EPI_ISL_985231	A/chicken/Miyazaki/K8T/2021	A / H5N8	Asia / Japan / Miyazaki	Takehiko Saito (National Institute of Animal Health)
EPI_ISL_985232	A/mallard duck/Niigata/150209C/2021	A / H5N8	Asia / Japan / Niigata	Takehiko Saito (National Institute of Animal Health)
EPI_ISL_985233	A/mallard duck/Niigata/150209T/2021	A / H5N8	Asia / Japan / Niigata	Takehiko Saito (National Institute of Animal Health)
EPI_ISL_9869760	A/American wigeon/South Carolina/AH0195145/2021	A / H5N1	North America / United States / South Carolina / Colleton County	Mary Lea Killian (National Veterinary Services Laboratories - USDA / Diagnostic Virology Laboratory)
EPI_ISL_9876777	A/American blue-winged teal/South Carolina/AH0195150/202	A / H5N1	North America / United States / South Carolina / Colleton County	Mary Lea Killian (National Veterinary Services Laboratories - USDA / Diagnostic Virology Laboratory)
EPI_ISL_9880015	A/American wigeon/North Carolina/AH0182517/2022	A / H5N1	North America / United States / North Carolina / Hyde	Mary Lea Killian (National Veterinary Services Laboratories - USDA / Diagnostic Virology Laboratory)
EPI_ISL_9880019	A/mallard/North Carolina/AH0182886/2022	A / H5N1	North America / United States / North Carolina / Pamlico	Mary Lea Killian (National Veterinary Services Laboratories - USDA / Diagnostic Virology Laboratory)
EPI_ISL_9880021	A/northern pintail/North Carolina/AH0182892/2022	A / H5N1	North America / United States / North Carolina / Pamlico	Mary Lea Killian (National Veterinary Services Laboratories - USDA / Diagnostic Virology Laboratory)
EPI_ISL_9880151	A/gadwall/North Carolina/AH0182894/2022	A / H5N1	North America / United States / North Carolina / Pamlico	Mary Lea Killian (National Veterinary Services Laboratories - USDA / Diagnostic Virology Laboratory)
EPI_ISL_9880152	A/northern shoveler/North Carolina/AH0182913/2022	A / H5N1	North America / United States / North Carolina / Pamlico	Mary Lea Killian (National Veterinary Services Laboratories - USDA / Diagnostic Virology Laboratory)
EPI_ISL_9880153	A/American wigeon/North Carolina/AH0182954/2022	A / H5N1	North America / United States / North Carolina / Pamlico	Mary Lea Killian (National Veterinary Services Laboratories - USDA / Diagnostic Virology Laboratory)
EPI_ISL_9909371	A/turkey/Indiana/22-003707-003/2022	A / H5N1	North America / United States / Indiana / Jasper County / Dubois	Mary Lea Killian (National Veterinary Services Laboratories - USDA / Diagnostic Virology Laboratory)
EPI_ISL_995172	A/chicken/Northern_Ireland/2020-17671_21VIR113-11/2020	A / H5N8	Europe / United Kingdom / Northern Ireland	Bianca Zecchin (Istituto Zooprofilattico Sperimentale Delle Venezie)
EPI_ISL_996003	A/chicken/Northern_Ireland/2021-000067_21VIR114-19/2021	A / H5N8	Europe / United Kingdom / Northern Ireland	Bianca Zecchin (Istituto Zooprofilattico Sperimentale Delle Venezie)
