# Supplementary figures and images for: Detection and phylogenetic analysis of highly pathogenic A/H5N1 avian influenza clade 2.3.4.4b virus in Chile, 2022
Source: Emerg Microbes Infect. 2023 Jun 20;12(2):2220569. doi: 10.1080/22221751.2023.2220569 (PMC10283444; doi:10.1080/22221751.2023.2220569)

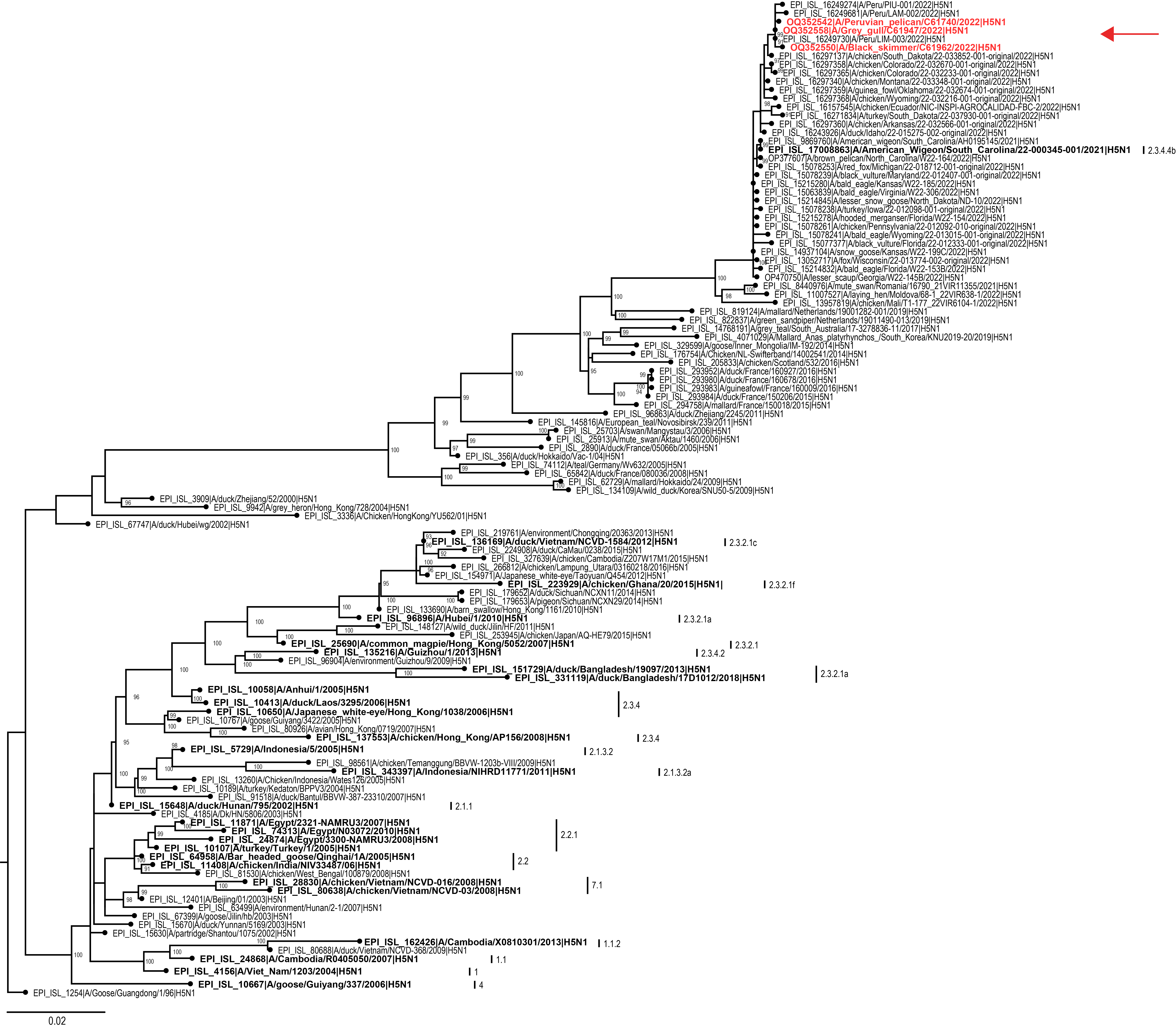

Supplement: Supplemental Material [file TEMI_A_2220569_SM7933.zip › Supplemental Figure 1. Chilean_H5_NA_FINAL.png]
